# SUMOylation of SETD8 Promotes Tumor Growth by Methylating and Stabilizing MYC in Bladder Cancer

**DOI:** 10.1002/advs.202501734

**Published:** 2025-03-16

**Authors:** Xia Zhang, Zhenxuan Chen, Xiaobo He, Jingxuan Wang, Jianliang Zhong, Yezi Zou, Xianchong Zheng, Yujie Lin, Ruhua Zhang, Tiebang Kang, Liwen Zhou, Yuanzhong Wu

**Affiliations:** ^1^ State Key Laboratory of Oncology in South China Collaborative Innovation Center for Cancer Medicine Guangdong Provincial Clinical Research Center for Cancer Sun Yat‐sen University Cancer Center Guangzhou 510060 P. R. China; ^2^ School of Medicine Shenzhen Campus of Sun Yat‐Sen University Shenzhen 518107 P. R. China

**Keywords:** bladder cancer (BC), methylation, MYC, SETD8, SUMOylation, tumor growth

## Abstract

Aberrant transcriptional and epigenetic landscape plays crucial roles in the progression of bladder cancer (BC). However, effective therapeutic targets derived from these processes remain undeveloped. This study pinpoints SET‐domain‐containing protein 8 (SETD8) as a pivotal gene that promotes bladder tumor growth through a screening with a CRISPR‐Cas9 library targeting transcriptional and epigenetic factors. BC patient samples display elevated SETD8 protein expression, and higher expression of SETD8 correlates with poorer prognosis. Further, MYC is identified as a novel substrate for SETD8. Specifically, SETD8 methylates MYC at lysine 412 (K412), disrupting the interaction between MYC and the E3 ubiquitin ligase CHIP, which results in MYC stabilization and ultimately promotes tumor growth both in vitro and in vivo. Moreover, this study uncovers that SUMOylation of SETD8 leads to SETD8 stabilization. The SUMOylated SETD8 further enhances MYC methylation and stabilization via SUMO‐SIM interaction. Knocking down SETD8 or using the SETD8 specific inhibitor UNC0379 substantially reduces the protein level of MYC and inhibits the bladder tumor growth in vitro and in vivo. These findings provide strong support for the idea that targeting the SETD8/MYC axis offers a promising therapeutic approach for BC patient.

## Graphical abstract

In bladder cancer (BC), MYC serves as a novel substrate of SETD8 to be methylated at lysine 412, which enhances MYC protein stability by preventing the CHIP‐mediated degradation and ultimately promotes tumor growth. SUMOylation of SETD8 leads to SETD8 stabilization and enhances MYC methylation. Targeting the SETD8/MYC axis with SETD8 inhibitor UNC0379 may provide a potential therapeutic strategy for BC.

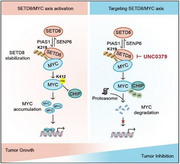

## 1 Introduction

Dysregulated landscape of epigenome and transcriptome has emerged as one of the most critical features in the development of BC.^[^ ^1^ ^]^ Increasing evidence suggests that targeting the interplay between epigenetic and transcriptional networks provides a promising approach for BC treatment.^[^ ^2^ ^]^ To systematically investigate such events in BC, our group has recently established a CRISPR‐Cas9 library based on sgRNAs targeting transcriptional and epigenetic factors, aiming to identify key genes required for the survival of BC cells.^[^ ^3^ ^]^ Among the top‐ranking genes in the screening results, we focused on SET‐domain containing protein 8 (SETD8), an important member of protein lysine methyltransferase (PKMT) family.^[^ ^4^ ^]^

SETD8 (also referred as SET8, KMT5A or PR‐Set7), has been well known so far as the sole enzyme capable of catalyzing H4K20 mono‐methylation and involved in DNA damage repair,^[^ ^5^ ^]^ cell cycle regulation,^[^ ^6^ ^]^ and chromatin remodeling.^[^ ^7^ ^]^ SETD8‐mediated methylation also installs marks on non‐histone substrates, such as p53,^[^ ^8^ ^]^ SNIP1,^[^ ^9^ ^]^ and CD147,^[^ ^10^ ^]^ which contributes to oncogenic processes. With its engagement in diverse biological processes through the methyltransferase activity, SETD8 is reported to be overexpressed in many cancers and is associated with poor prognosis.^[^ ^11^ ^]^ However, the specific role of SETD8 in BC has not yet been characterized.

In our study, we observed elevated SETD8 expression in BC samples. Notably, higher protein level of SETD8 was associated with shorter overall survival. We validated that SETD8 is essential for bladder tumor growth. Further, we discovered that MYC is a novel substrate of SETD8, underscoring the critical role of SETD8 in bladder tumor progression. By catalyzing the methylation of MYC at lysine 412 (K412), SETD8 strongly stabilizes MYC and promotes bladder tumor growth. SUMOylation of SETD8, which we have demonstrated to be modulated by PIAS1 and SENP6, stabilizes SETD8 and further enhances its binding with MYC via SUMO‐SIM interaction, leading to increased MYC methylation and tumor progression. Targeting the SETD8/MYC axis using the SETD8 specific inhibitor UNC0379 holds promise for developing novel therapeutic strategies for BC.

## 2 Results

### 2.1 SETD8 Is Required for Bladder Tumor Growth

In previous studies, our group identified key molecules essential for the survival of BC cells by CRISPR‐Cas9 screening with lentiviral library targeting factors involved in transcriptional and epigenetic regulation.^[^ ^3^, ^12^ ^]^ Among the candidate essential genes identified, SETD8 drew our attention due to its critical role in multiple biological processes and remains uncharacterized in bladder cancer (BC) (Figure S1a, Supporting Information).^[^ ^13^ ^]^ Consistently, gene effect scores of SETD8 in BC cell lines are all negative (Figure S1b, Supporting Information).

**Figure 1:**
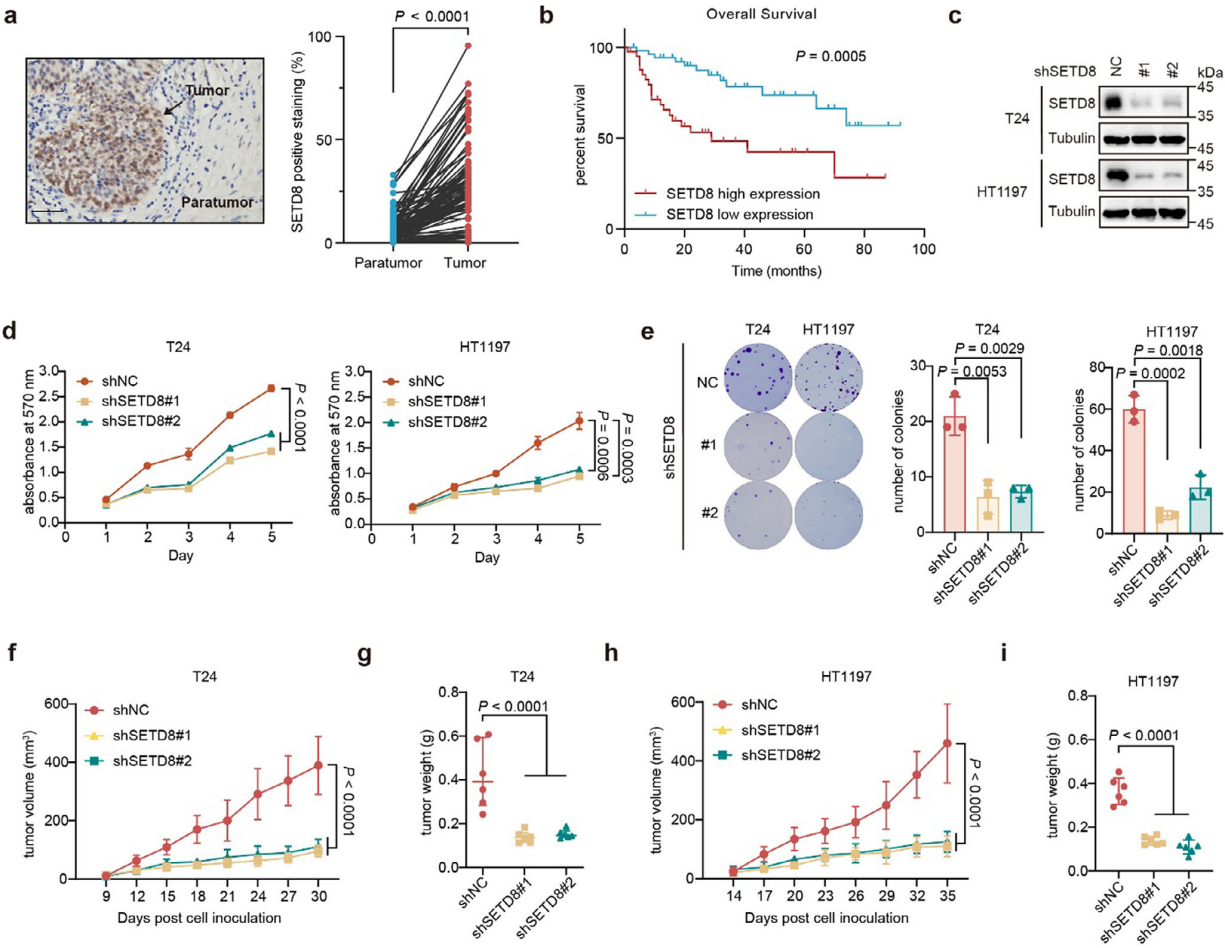
SETD8 is required for bladder tumor growth. a) The relative expression levels of SETD8 in paired tumor and paratumor tissues from 95 BC patients were assessed by analyzing immunohistochemistry (IHC) staining images via HALO system. Scale bar, 100 µm. b) Kaplan‐Meier survival analysis of BC cases, stratified by SETD8 expression levels as determined in (a). c) Stable T24 and HT1197 cells were generated with two pairs of shRNA targeting SETD8, and the relative proteins were detected by western blotting. d,e) MTT and colony formation assay of T24 as well as HT1197 cells bearing SETD8 knockdown. Statistical significance was determined from three independent experiments. f–i) Xenograft tumors were generated via subcutaneous injection of the indicated stable T24 and HT1197 cells into athymic nude mice (n = 6 per group). Tumor volumes were measured every 3 days, and tumor weights were recorded at the end of the experiment after the animals were sacrificed. Bars represent the SD. *p* values were calculated by Student's *t* test.

Immunohistochemistry (IHC) staining of bladder patient slices showed that SETD8 was elevated in tumor tissues compared to the adjacent normal tissues (**Figure** **1**a). Moreover, higher expression of SETD8 correlated with shorter survival time in BC (Figure 1b). The elevated level of SETD8 in bladder tumor tissues and the correlation with poor prognosis were also confirmed in the cohort analysis derived from TCGA (Figure S1c,d, Supporting Information). Knocking down SETD8 by shRNAs in T24 and HT1197 cells, which exhibit relatively high levels of SETD8 (Figure 1c; Figure S1e, Supporting Information), resulted in a significant reduction of BC cell viability, colony formation, and tumor growth (Figure 1d–i; Figure S1f, Supporting Information). Furthermore, rescue with wild‐type SETD8 in SETD8‐knockdown T24 and HT1197 cells restored the proliferative phenotype, whereas the R336G mutant, an inactive form of SETD8,^[^ ^9^ ^]^ could not (Figure S1g–i, Supporting Information). Together, all these results demonstrated that SETD8 is essential for bladder tumor growth both in vitro and in vivo, which is potentially associated with its methyltransferase activity.

### 2.2 SETD8 Stabilizes MYC Protein

**Figure 2:**
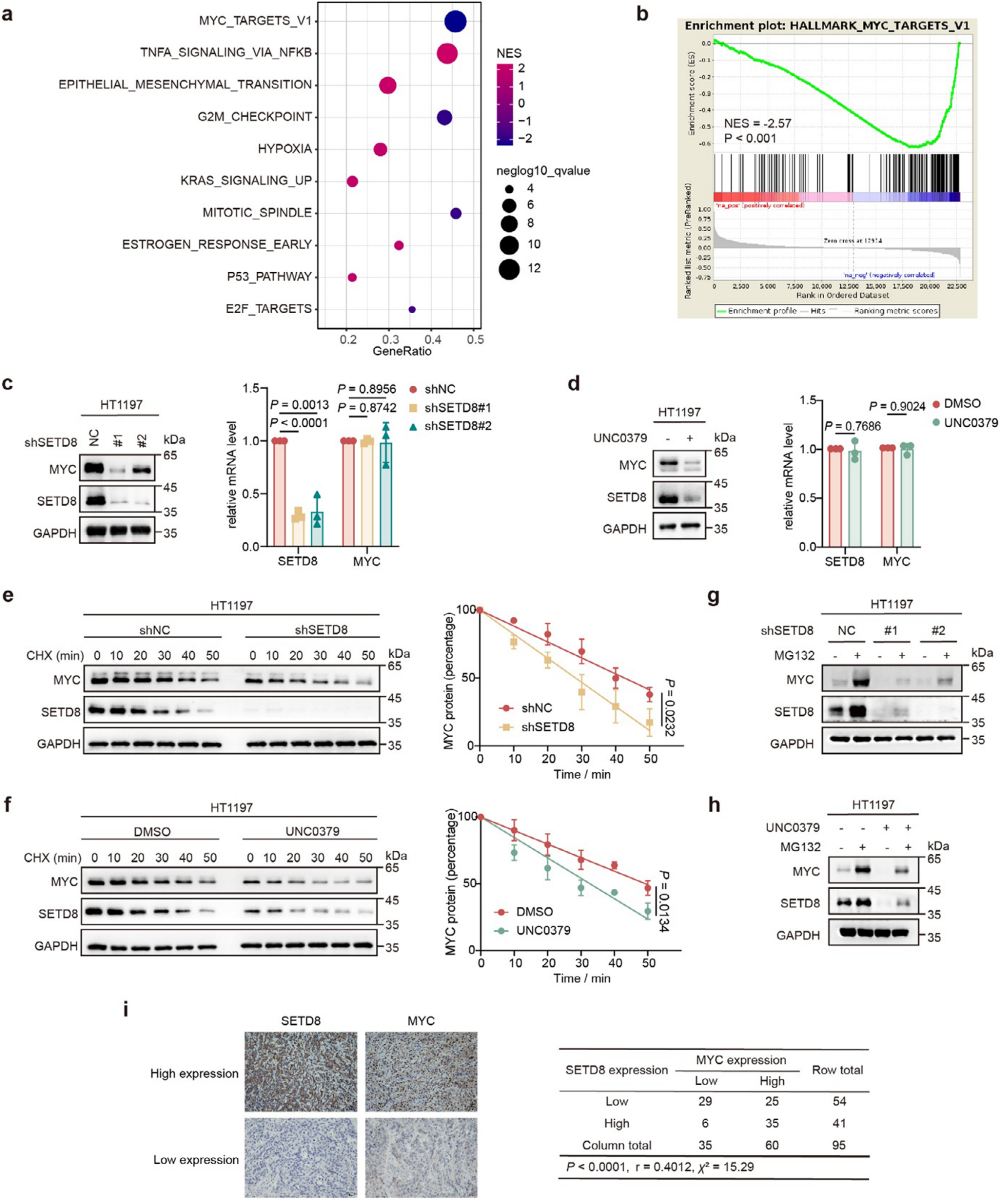
SETD8 stabilizes MYC protein. a) Bubble plots depicted enriched terms from gene set enrichment analysis (GSEA), ranked by *q* value, based on RNA‐seq data from T24 cells with SETD8 depletion. b) GSEA of MYC target genes in the expression profiles of T24 cells with SETD8 depletion. c) The protein and mRNA levels of the indicated genes in HT1197 stable cells. d) The protein and mRNA levels of the indicated genes in HT1197 cells treated with UNC0379 (5 µm, 24 h). e) Time‐course analysis of the indicated proteins in HT1197 cells bearing SETD8 knockdown following the treatment with cycloheximide (CHX, 20 µg mL⁻^1^). f) Time‐course analysis of the indicated proteins in HT1197 cells upon the treatment of UNC0379 (5 µm, 24 h) and CHX (20 µg mL⁻^1^), subsequently. Relative MYC protein levels in e) and f) were quantified by ImageJ. Three independent experiments were established to determine the statistical significance. Data are represented as means ± SD. *p* values were calculated by Student's *t* test. g) HT1197 cells expressing SETD8 shRNA were incubated with MG132 (10 µm, 6 h) and the indicated proteins were detected by western blotting. h) HT1197 cells were treated with UNC0379 (5 µm, 24 h), followed by incubation with MG132 (10 µm, 6 h). Relative proteins were detected by western blotting. i) Left, representative IHC images of SETD8 and MYC at high or low expression level in BC tissues. Scale bar, 100 µm. Right, the crosstab represents the distribution of 95 BC cases stratified by high or low expression levels of SETD8 and MYC, based on the IHC scores obtained through ImageJ. Spearman's correlation test was used to estimate the Spearman correlation coefficient, while *p* value was calculated by Pearson's chi‐squared test.

To explore how SETD8 functions in BC, we employed RNA sequencing on SETD8‐depleted cells and observed a significant down‐regulation of the MYC pathway (**Figure** **2**a,b; Table S1, Supporting Information). qPCR assay further confirmed the sequencing results (Figure S2a, Supporting Information). These data suggested that in BC, SETD8 may involve in regulating tumor growth through MYC, which was also ranked among the top candidates in our previous screening.^[^ ^3^ ^]^ SETD8 has been previously reported to regulate gene expression by catalyzing H4K20me1.^[^ ^13^ ^]^ To explore the impact of H4K20me1 on the MYC pathway, we performed a CUT&RUN assay using an anti‐H4K20me1 antibody in SETD8‐knockdown cell lines and observed reduced signals in the promoter and gene body regions of MYC target genes (Figure S2b, Supporting Information). Nevertheless, either SETD8 knockdown or treatment with the SETD8 specific inhibitor UNC0379,^[^ ^14^ ^]^ substantially reduced MYC protein levels without affecting MYC mRNA levels in both HT1197 and T24 cells (Figure 2c,d; Figure S2c,d, Supporting Information). Consistently, shortened half‐life of MYC protein was observed (Figure 2e,f; Figure S2e,f, Supporting Information). The results suggest an additional mechanism, beyond H4K20me1‐mediated chromatin remodeling, that SETD8 may regulate the MYC pathway directly through modulating of MYC protein level. Further, the reduction in MYC protein levels caused by either genetic or pharmacological inhibition of SETD8 was rescued by the proteasome inhibitor MG132 (Figure 2g,h; Figure S2g,h, Supporting Information). Most importantly, the ubiquitination level of MYC was markedly increased when SETD8 was knocked down or inhibited by UNC0379 in HT1197 cells (Figure S2i,j, Supporting Information). IHC staining on BC patient slices showed a positive correlation between SETD8 and MYC protein level in BC tissues (Figure 2i). For another, co‐transfection of wild type (WT) SETD8 but not the inactive R336G mutant with MYC significantly reduced the ubiquitination level and prolonged the half‐life of MYC (Figure S2k,l, Supporting Information).^[^ ^9^ ^]^ All these results indicated that SETD8 stabilizes MYC protein by inhibiting its ubiquitin‐proteasome degradation in BC.

### 2.3 SETD8 Methylates MYC at K412 to Inhibit CHIP‐Mediated Degradation of MYC

**Figure 3:**
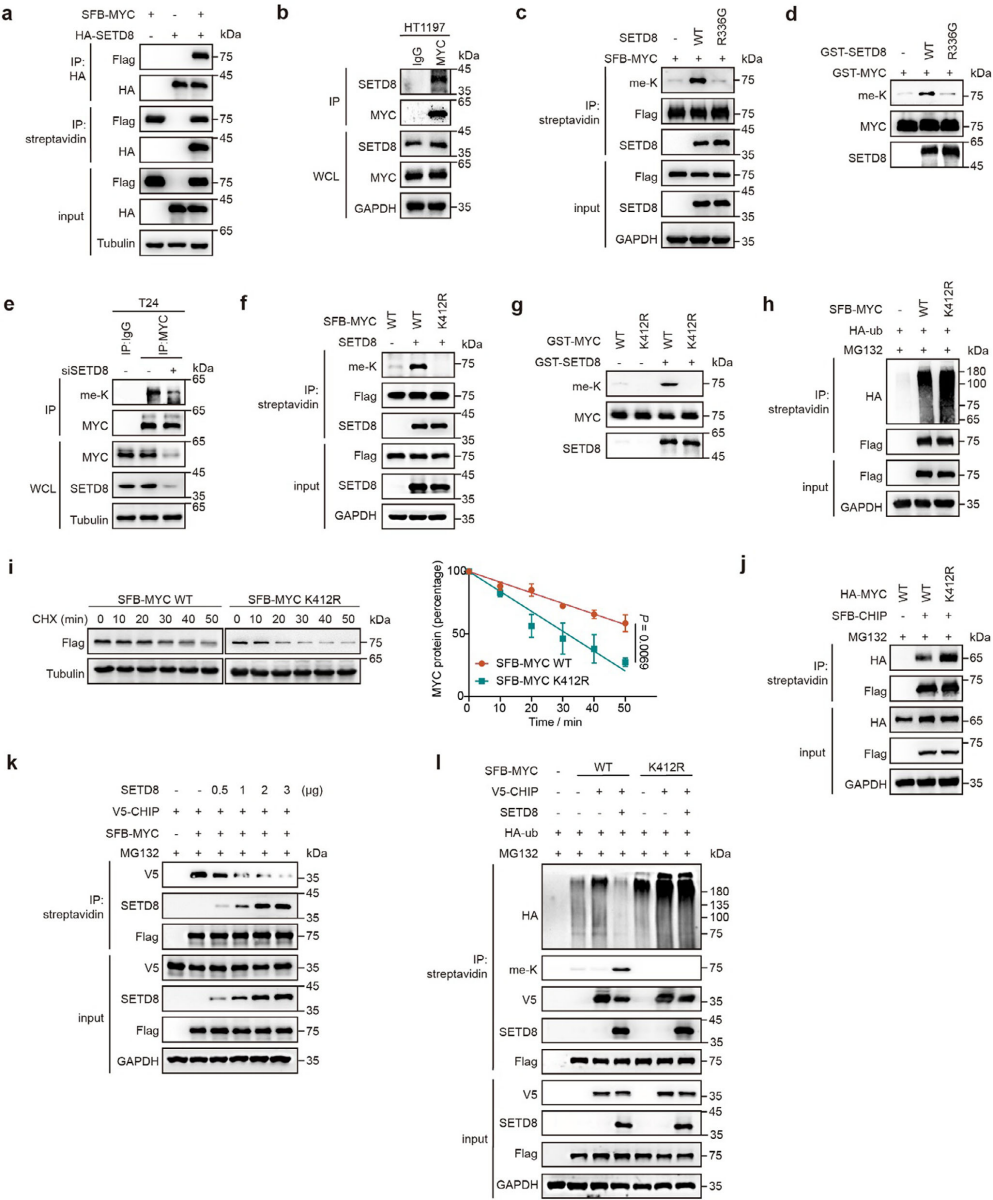
SETD8 methylates MYC at K412 to inhibit CHIP‐mediated degradation of MYC. a) HEK‐293T cells ectopically expressing SFB‐MYC and HA‐SETD8 were subjected to co‐immunoprecipitation (co‐IP) with HA‐tagged or streptavidin beads followed by western blotting. b) Co‐IP was performed in HT1197 cells using anti‐IgG or anti‐MYC antibodies, and endogenous SETD8 was detected by western blotting. c) HEK‐293T cells were co‐transfected with SFB‐MYC and SETD8 wildtype (WT) or its R336G mutant (kinase‐dead mutant), and subjected to co‐IP as well as subsequent western blotting to detect lysine methylation (me‐K) level of MYC. d) In vitro methylation assay with purified substrate GST‐MYC and lysine methyltransferase GST‐SETD8 WT or its R336G mutant. e) T24 cells transfected with SETD8 siRNA were subjected to co‐IP using anti‐IgG or anti‐MYC antibodies, and endogenous lysine methylation level of MYC was subsequently detected. f) Immunoblot analysis of lysine methylation level of ectopic SFB‐MYC WT or K412R mutant in HEK‐293T cells when co‐expressed with SETD8. g) In vitro methylation assay with purified GST‐MYC WT or K412R mutant and GST‐SETD8. h) HEK‐293T cells co‐transfected with HA‐ub and SFB‐MYC WT or K412R mutant were incubated with MG132 (10 µm, 6 h), then harvested to detect exogenous ubiquitination level of MYC via co‐IP. i) Time‐course analysis of the indicated proteins in HEK‐293T cells expressing SFB‐MYC WT or K412R mutant upon CHX (20 µg mL⁻^1^) treatment. Statistical significance was determined from three independent experiments. Data are presented as means ± SD, with *p* values calculated using Student's *t* test. j) Co‐IP of SFB‐CHIP and HA‐MYC WT or K412R mutant co‐expressed in HEK‐293T cells after treatment with MG132 (10 µm, 6 h). k) Immunoblot analysis of the interaction between SFB‐MYC and V5‐CHIP in HEK‐293T cells when co‐transfected with the increased amount of SETD8 under the treatment with MG132 (10 µm, 6 h). l) Co‐IP was performed to detect exogenous ubiquitination and methylation level of SFB‐MYC WT or K412R mutant when co‐transfected with V5‐CHIP and SETD8 in HEK‐293T cells, which were incubated with MG132 (10 µm, 6 h) prior to harvesting.

Next, we investigated how SETD8 stabilizes MYC protein. Exogenous and endogenous co‐immunoprecipitations showed that SETD8 and MYC interact with each other (**Figure** **3**a,b). Co‐transfection of SFB‐MYC full length (FL) or truncations with HA‐SETD8 identified that the amino acids 345–439 of MYC is responsible for its binding to SETD8 (Figure S3a, Supporting Information). Likewise, both the amino acids 1–101 and 192–352 of SETD8 were identified to mediate its interaction with MYC (Figure S3b, Supporting Information). These data suggested the possibility that MYC is a novel substrate of SETD8. Indeed, in vivo and in vitro methylation assays showed that the lysine methylation level of MYC increased in the presence of WT SETD8, but not its inactive R336G mutant (Figure 3c,d). Importantly, the knockdown of SETD8 using siRNAs resulted in decreased MYC methylation level in T24 cells (Figure 3e). These results demonstrated that MYC is the bona fide substrate of SETD8, and can be methylated at the lysine residue.

To identify the specific lysine residue in MYC that is methylated by SETD8, mass spectrometry was performed. Interestingly, the lysine K412 within the SETD8 binding region of MYC (amino acids 345–439) was identified to be methylated (Figure S3c, Supporting Information). Meanwhile, we constructed MYC mutants by individually mutating each lysine residue within the binding region into arginine. Co‐transfection assays showed that only K412R mutant abolished the SETD8‐meidated methylation of MYC both in cells and in vitro methylation assay (Figure 3f,g; Figure S3d, Supporting Information). Together, these results pinpointed K412 of MYC as the methylated residue regulated by SETD8.

Further, to investigate whether SETD8‐mediated methylation of MYC at K412 affects MYC stability, we performed the ubiquitination assay and the half‐life assay. Results showed that MYC‐K412R mutant exhibited increased ubiquitination and shortened half‐life compared to wild‐type MYC (Figure 3h,i). Based on this result, we reasoned that the K412R mutation in MYC might enhance its interaction with the E3 ligase. By co‐transfecting MYC wildtype or K412R mutant with the E3 ligases, which have been reported to mediate MYC degradation in previous studies,^[^ ^15^ ^]^ we found out that K412R mutant showed increased binding to CHIP (Figure 3j; Figure S3e, Supporting Information), suggesting that in BC, SETD8 inhibits CHIP‐mediated MYC degradation. Indeed, SETD8 competitively disrupted the interaction between MYC and CHIP in a dose‐dependent manner (Figure 3k). Also, CHIP‐mediated MYC ubiquitination was inhibited with the addition of SETD8, while its methylation level was enhanced (Figure 3l). All these data indicated that SETD8 methylates MYC at K412, disrupting the interaction between MYC and the E3 ligase CHIP, and promoting MYC stabilization.

### 2.4 Methylation of MYC at K412 Promotes Bladder Tumor Growth

**Figure 4:**
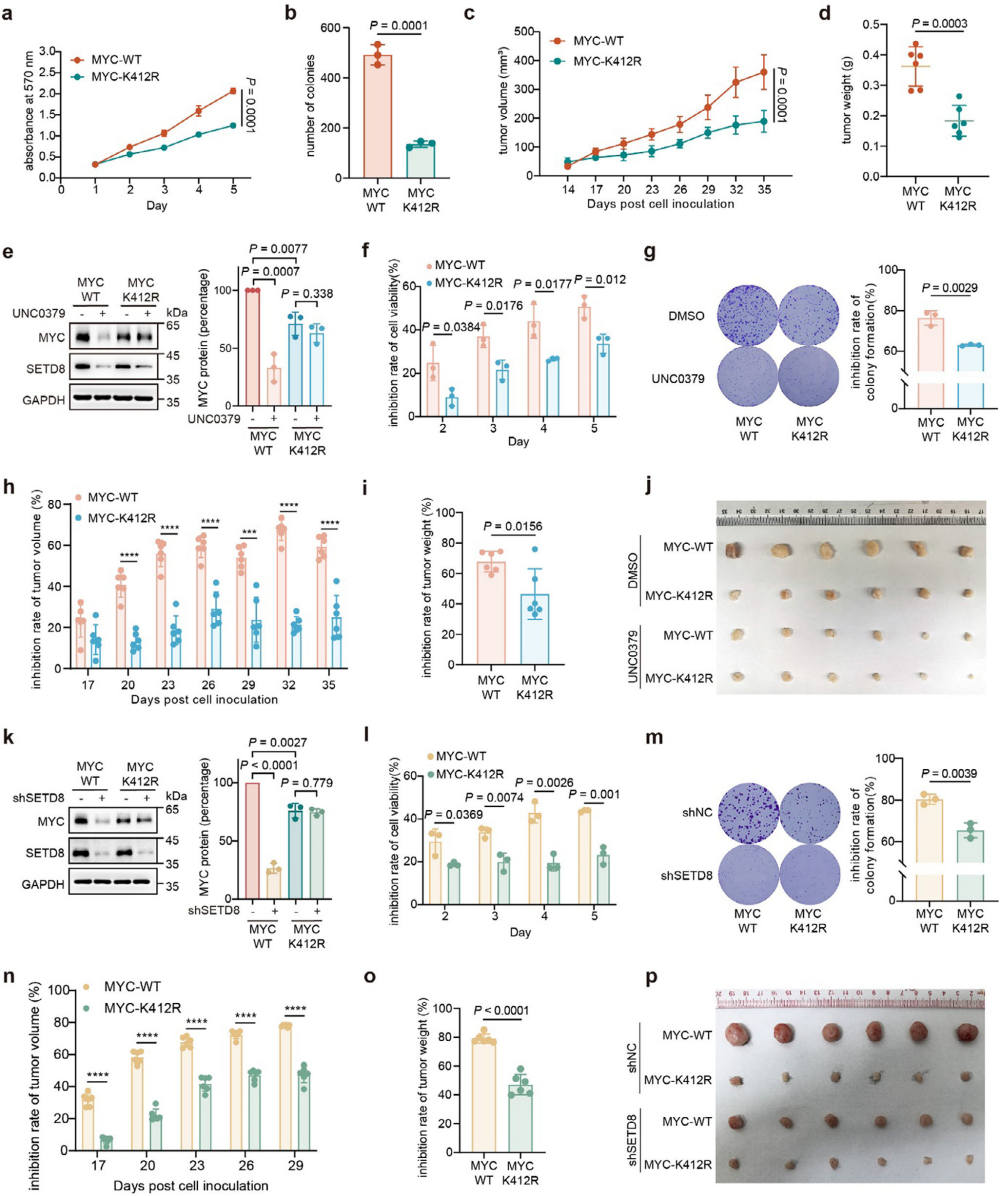
MYC‐K412 methylation promotes bladder tumor growth. A,b) MTT and colony assay of knock‐in cells. Colonies were calculated by ImageJ. c,d) Xenograft tumors were generated via subcutaneous injection of knock‐in cells into athymic nude mice (n = 6 per group). Tumor volumes were measured every 3 days, and weights were recorded after the animals were sacrificed. e) Knock‐in cells were treated with UNC0379 (5 µm, 24 h), and the indicated proteins were detected by western blotting. f,g) Inhibition rates in MTT assay and colony formation assay of knock‐in cells when exposed to UNC0379 (5 µm, 24 h). h,i) Inhibition rates of tumor volume and weight in knock‐in cell‐derived xenografts with UNC0379 arrangement (20 mg kg^−1^ d⁻^1^) since the 14^th^ day post cell inoculation (n = 6 per group). j) Xenografts excised from nude mice bearing tumors derived from knock‐in cells under UNC0379 arrangement. k) SETD8 was knocked down in knock‐in cells, and the indicated proteins were detected by western blotting. l,m) Inhibition rates in MTT assay and colony formation assay of knock‐in cells bearing SETD8 knockdown. n,o) Inhibition rates of tumor volume and weight in xenografts derived from knock‐in cells with SETD8 knockdown (n = 6 per group). p) Xenografts excised from n). Two‐tailed Student's *t*‐test determined the statistical significance of three biological replicates. Data are presented as means ± SD. *p < 0.05, **p < 0.01, ***p < 0.001, ****p < 0.0001.

To investigate the effect of MYC K412 methylation on bladder tumor growth, we mutated the endogenous MYC K412 into arginine in HT1197 cells using CRISPR‐cas9 gene editing (Figure S4a,b, Supporting Information). Consistent with the exogenous results, the generated homozygous K412R knock‐in HT1197 cell line showed increased ubiquitination level and reduced MYC half‐life (Figure S4c,d, Supporting Information). Down‐regulated MYC‐related pathway was also validated by RNA‐sequencing and qPCR assays (Figure S4e,f, Supporting Information). CUT&RUN assay was further performed in knock‐in cells to explore whether K412‐methylation affects MYC transcriptional activity. We found that the K412R mutant could still bind to the promoter of target genes, although with a decrease in signal (Figure S4g,h, Supporting Information), likely due to the reduced abundance of the MYC K412R protein. MYC‐K412R knock‐in cells exhibited reduced cell viability and colony formation in vitro (**Figure** **4**a,b). In vivo, tumor growth was inhibited, and tumor weight was decreased in MYC‐K412R knock‐in cells (Figure 4c,d). Further, MYC protein level in the knock‐in cells remained resistant to inhibition by either UNC0379 or SETD8 knockdown (Figure 4e,k). The knock‐in cells exhibited reduced inhibition rates of cell proliferation and colony formation, as well as decreased tumor growth and tumor weights following pharmacological inhibition (Figure 4f–j) or genetic knockdown of SETD8 (Figure 4l–p). These results underscored the critical role of MYC‐K412 methylation in driving bladder tumor growth and highlighted the significance of SETD8 in regulating this process.

### 2.5 SUMOylation at K219 Enhances SETD8 Protein Stability

IHC staining showed that SETD8 was overexpressed in BC tissues compared to adjacent normal tissues (Figure 1a). Notably, treatment with MG132 and Bortezomib led to a significant accumulation of SETD8 in both T24 and HT1197 cells (Figure S5a, Supporting Information), and the half‐life of SETD8 protein was ≈100 minutes (Figure S5b, Supporting Information), suggesting that the ubiquitin‐proteasome pathway regulates SETD8 degradation.

**Figure 5:**
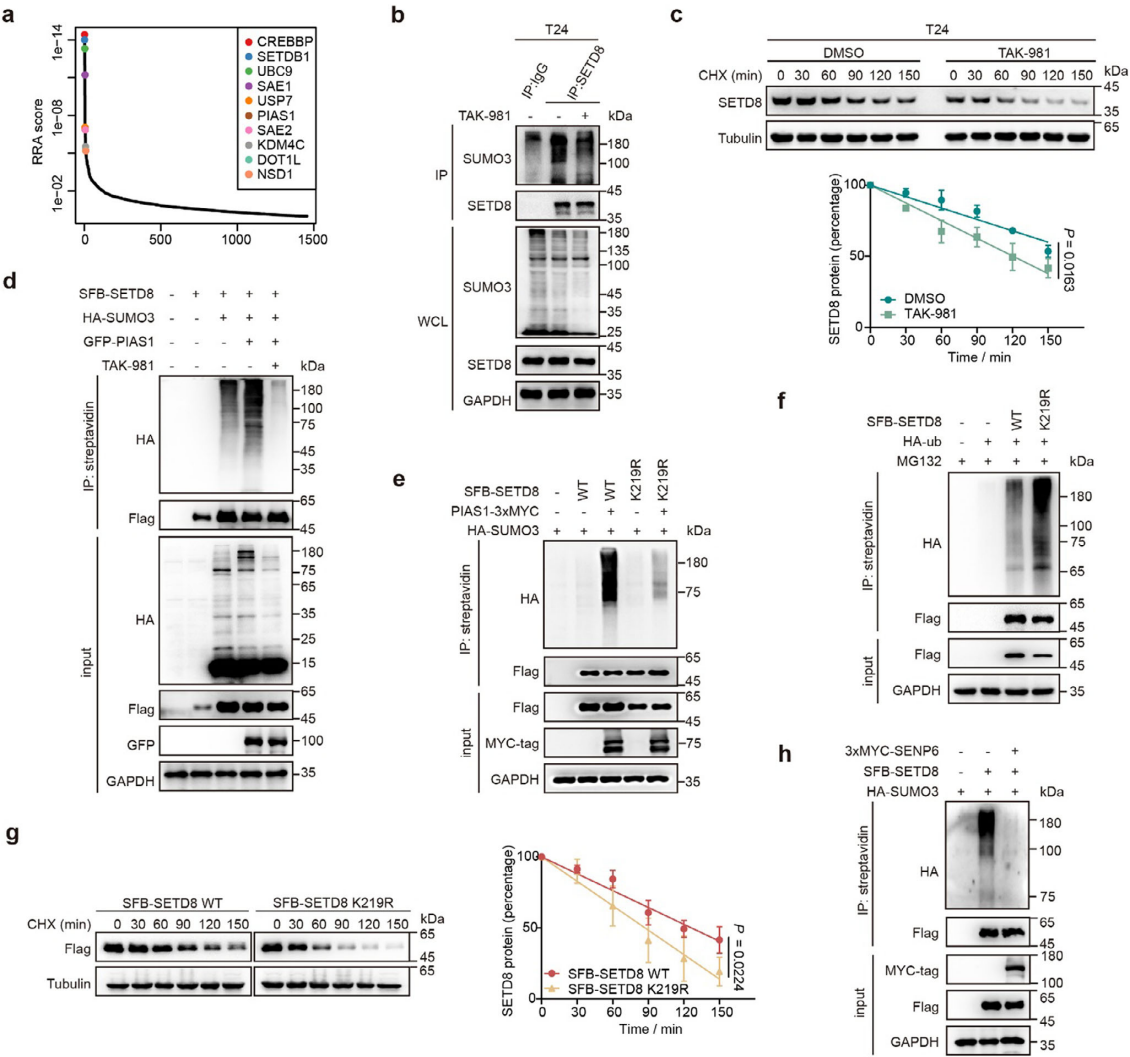
SUMOylation enhances SETD8 protein stability. a) Genes ranked at the top that regulate SETD8 stability are shown via MAGeCK analysis. The X‐axis represents the ranking, while the Y‐axis indicates the robust rank aggregation (RRA) score for each gene. b) T24 cells treated with TAK‐981 (1 µm, 6 h) were subjected to co‐IP assay to detect endogenous SUMOylation level of SETD8 via western blotting. c) Time‐course analysis of SETD8 protein in T24 cells incubated with TAK‐981 (1 µm, 6 h), followed by the addition of CHX (20 µg mL⁻^1^) at the indicated time point. d) Immunoblot analysis of exogenous SUMOylation level of SETD8 mediated by PIAS1 in HEK‐293T cells upon the treatment of TAK‐981 (1 µm, 6 h). e) Immunoblot analysis of SUMOylation level of ectopic SFB‐SETD8 WT and K219R mutant in HEK‐293T cells. f) Immunoblot analysis of ubiquitination level of SFB‐SETD8 WT and K219R mutant in HEK‐293T cells treated with MG132 (10 µm, 6 h). g) Time‐course analysis in HEK‐293T cells expressing SFB‐SETD8 WT or K219R mutant following treatment with CHX (20 µg mL⁻^1^). h) Immunoblot analysis of SUMOylation level of SFB‐SETD8 when co‐transfected with 3xMYC‐SENP6 in HEK‐293T cells. Statistical significance was determined from three independent experiments. Data are presented as means ± SD, with *p* values calculated using Student's *t* test.

To unbiasedly identify the regulators of SETD8 protein stability, we employed the Protein Stability Regulators Screening Assay (ProSRSA) based on the CRIPSR‐Cas9 library in T24 cells (Figure S5c, Supporting Information).^[^ ^16^ ^]^ Reactome enrichment analysis of the candidate factors that stabilize SETD8 exhibited a significant enrichment of the SUMOylation‐related pathway, as SUMO‐activating enzymes 1 and 2 (SAE1 and SAE2, SUMO E1), ubiquitin‐like conjugating enzyme 9 (UBC9, SUMO E2) and protein inhibitor of activated STAT 1 (PIAS1, SUMO E3) were ranked at the top list (**Figure** **5**a; Figure S5d–g, Supporting Information). Immunoprecipitation assay revealed that SETD8 preferentially interacts with SUMO3 compared to SUMO1 or SUMO2 (Figure S5h, Supporting Information). Furthermore, pharmacological inhibition of SUMOylation using TAK‐981 in T24 cells significantly reduced SETD8 SUMOylation (Figure 5b).^[^ ^17^ ^]^ Consistently, TAK‐981 treatment led to dose‐ and time‐dependent decrease in SETD8 protein levels in both T24 and HT1197 cell (Figure S5i, Supporting Information). The half‐life of SETD8 protein was also shortened upon TAK‐981 treatment in these cells (Figure 5c; Figure S5j, Supporting Information). The results determined that SUMOylation of SETD8 stabilizes its protein.

We next investigated the SUMO E3 ligase responsible for SETD8 SUMOylation. Co‐IP assays showed that among the PIAS family,^[^ ^18^ ^]^ only PIAS1 was found to enhance the SUMOylation of SETD8 (Figure S6a, Supporting Information), and this effect was inhibited by TAK‐981 (Figure 5d), further corroborating our screening results from the ProSRSA assay (Figure 5a). Endogenous Co‐IP assay confirmed the interaction between SETD8 and PIAS1 in T24 cells (Figure S6b, Supporting Information), emphasizing that PIAS1 is the E3 ligase responsible for SUMOylation of SETD8.

According to previous studies, SUMOylation typically occurs at lysine residues within the conserved amino acid sequence, ΨKxE/D (Ψ represents hydrophobic amino acids like A, I, L, M, P, F, V, and W; x means any amino acid).^[^ ^19^ ^]^ We found out that there are six potential lysine residues (K94, K110, K115, K131, K195, K219) within SETD8 that match this motif, and mutated each lysine to arginine (Figure S6c, Supporting Information). Only the K219R mutation significantly reduced the SUMOylation of SETD8 (Figure 5e; Figure S6d, Supporting Information). Correspondingly, the ubiquitination level of SETD8‐K219R was increased, and its half‐life was reduced compared to SETD8‐WT (Figure 5f,g). These results revealed that SUMOylation at K219 stabilizes SETD8.

Given that SUMOylation is a reversible process involving the balance between SUMO conjugation and deconjugation,^[^ ^20^ ^]^ we also investigated the enzymes responsible for SUMO removal from SETD8. Among the sentrin‐specific protease (SENP) family, SENP6 showed a much stronger interaction with SETD8 compared to other SENPs (Figure S6e, Supporting Information), and a significant reduced SETD8 SUMOylation (Figure 5h). Taken together, these results demonstrated that SETD8 stability is positively regulated by PIAS1‐mediated SUMOylation at K219, which is reversed by SENP6.

### 2.6 SUMOylated SETD8 Enhances MYC K412 Methylation

**Figure 6:**
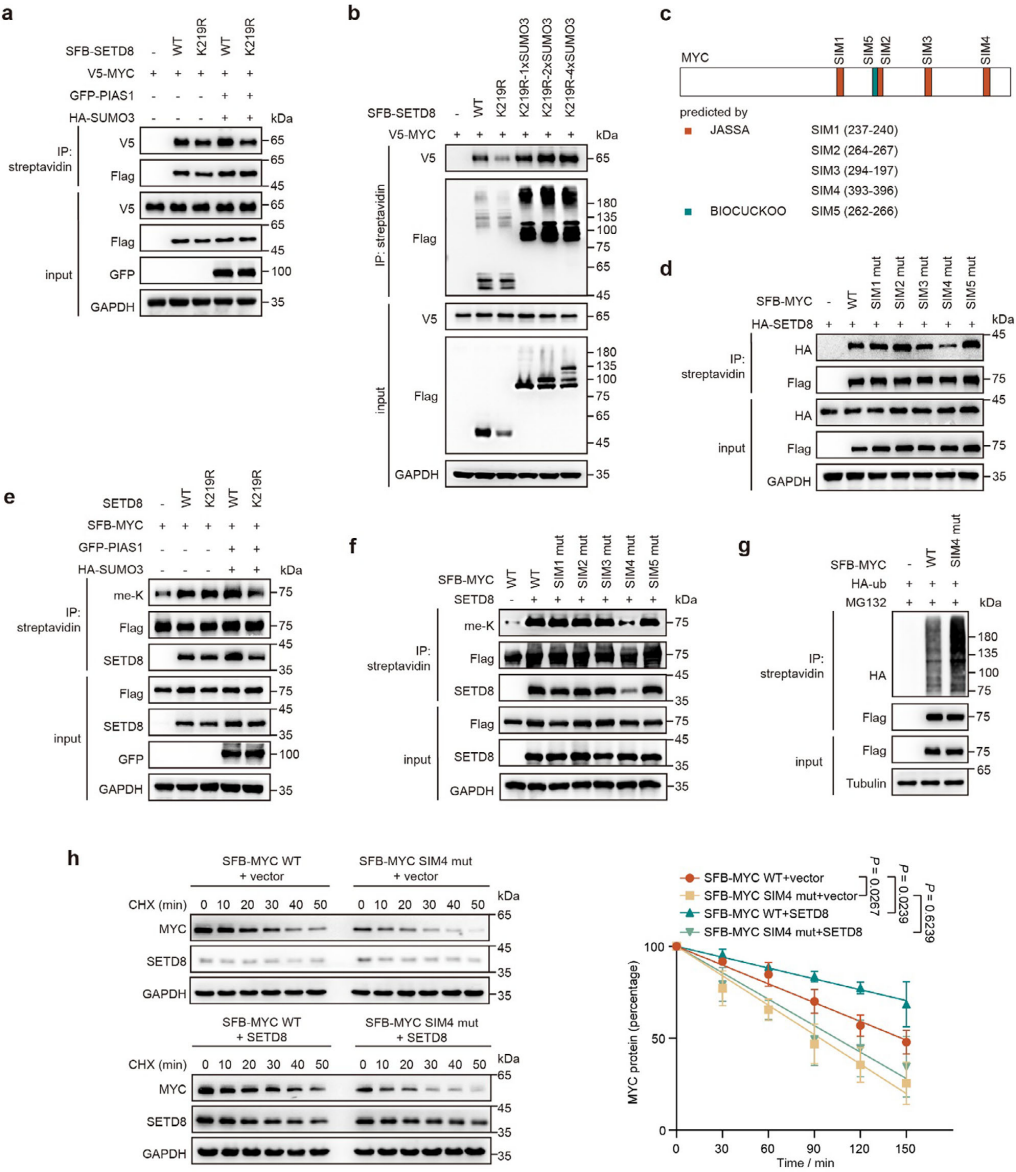
SUMOylated SETD8 enhances MYC‐K412 methylation. a) Co‐IP of V5‐MYC and SFB‐SETD8 WT or its K219R mutant in HEK‐293T cells, in the presence of GFP‐PIAS1 and HA‐SUMO3. b) Co‐IP of V5‐MYC and SFB‐SETD8 WT or its K219R mutant added with 1x, 2x, or 4x SUMO3. c) SIM domains in MYC predicted by JASSA (www.jassa.fr) and BIOCUCKOO (www.biocuckoo.org). d) Co‐IP of HA‐SETD8 and SFB‐MYC mutants with synonymous mutations in predicted SIM motifs in HEK‐293T cells. e) Immunoblot analysis of methylation level of SFB‐MYC when co‐expressed with SETD8 WT or K219R in HEK‐293T cells. f) Immunoblot analysis of lysine methylation level of SFB‐MYC with SIM mutation while co‐transfected with SETD8 in HEK‐293T cells. g) Immunoblot analysis of ubiquitination level of SFB‐MYC WT and SIM4 mutant in HEK‐293T cells following MG132 (10 µm, 6 h) treatment. h) Time‐course analysis was performed in HEK‐293T cells expressing SFB‐MYC WT or SIM4 mutant, with or without SETD8, following treatment with CHX (20 µg mL⁻^1^). Statistical significance was determined from three independent experiments. Data are presented as means ± SD, with *p* values calculated using Student's *t* test.

Since SUMO‐SIM (SUMO interacting motif) interactions are fundamental and prevalent in the protein interactome,^[^ ^21^ ^]^ we evaluated whether SUMOylation of SETD8 affects its binding to MYC. We found out that ectopic PIAS1 enhanced the interaction of MYC with SETD8‐WT, but not with SETD8‐K219R mutant (**Figure** **6**a). Further, we constructed SETD8 K219R chimeras added with 1x, 2x, or 4x SUMO3, and found that its interaction with MYC was enhanced (Figure 6b). Additionally, five SIM motifs within MYC were predicted using JASSA and Biocuckoo (Figure 6c),^[^ ^22^ ^]^ and only the SIM4 (amino acids 393–396) mutant of MYC exhibited a significant reduction in its interaction with SETD8 (Figure 6d). Consistently, SETD8‐K219R mutant showed a reduced ability to enhance the methylation of MYC compared to SETD8‐WT (Figure 6e). And a decrease in MYC methylation was observed in the SIM4 mutant (Figure 6f). Correspondingly, the ubiquitination level of the MYC SIM4 mutant was increased (Figure 6g). The MYC SIM4 mutant also exhibited a shortened half‐life, which only slightly changed even in the presence of SETD8 (Figure 6h). These results suggested that SUMOylation enhances the interaction between SETD8 and MYC, and subsequently increases the methylation of MYC.

## 3 Discussion

**Figure 7:**
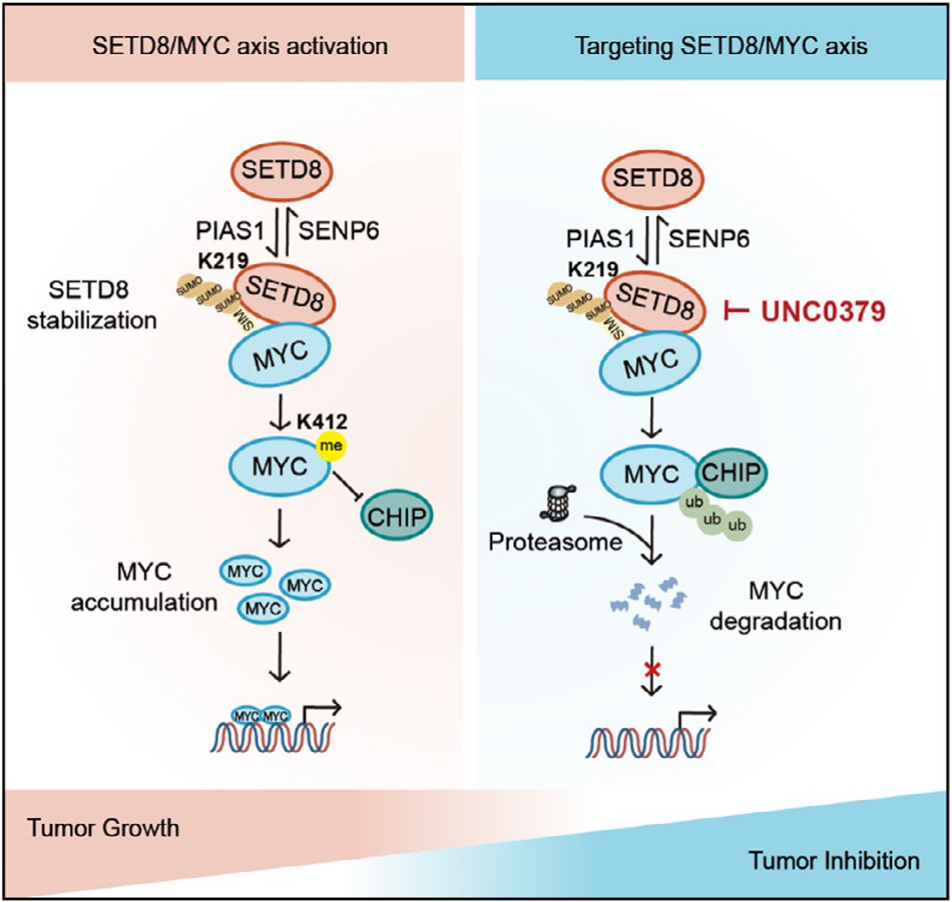
Model of the regulation of SETD/MYC axis in BC. SETD8 methylates MYC at K412, which prevents the CHIP‐mediated degradation of MYC and promotes MYC accumulation and bladder tumor growth. SUMOylation of SETD8, modulated by PIAS1 and SENP6, stabilizes SETD8 and further strengthens its interaction with MYC through the SUMO‐SIM interaction, leading to increased MYC methylation and enhanced tumor growth. Targeting the SETD8/MYC axis with SETD8 inhibitor UNC0379 may provide a potential therapeutic strategy for BC patients.

Aberrant transcriptional and epigenetic landscape is one of the common features of BC.^[^ ^2b^ ^]^ Our group focused on the key factors essential for bladder tumor growth and discovered a series of novel regulations in previous studies through utilizing CRISPR‐Cas9 screening with a library targeting epigenetic and transcriptional factors.^[^ ^3^, ^12^ ^]^ In this report, we discovered that SETD8/MYC axis is critical for BC. As illustrated in **Figure** **7**, we found that MYC, as one of the core drivers of bladder tumor growth and progression,^[^ ^23^ ^]^ is a novel substrate of SETD8 and can be methylated at K412. The methylation of MYC K412 leads to inhibition of CHIP‐mediated MYC degradation, enhancement of MYC protein stability and eventual facilitation of tumor growth. In patient samples, we observed an elevated protein level of SETD8, and the overall high SETD8 protein level correlates with poor prognosis in BC patients. By using the ProSRSA assay, we identified that SETD8 can be modified and stabilized by SUMOylation, which is modulated by PIAS1 and SENP6. SUMOylated SETD8 enhances MYC K412 methylation, further promoting bladder tumor growth. Pharmacological inhibition of SETD8 with UNC0379 ameliorates bladder tumor growth, highlighting the potential of targeting the SETD8/MYC axis as a therapeutic strategy for BC.

Abnormal expression of SETD8 has been reported in various cancers, though the mechanisms remain poorly understood.^[^ ^24^ ^]^ Previous studies have emphasized the role of ubiquitin‐mediated degradation in SETD8 regulation, with E3 ligases like APC^Cdh1^,^[^ ^25^ ^]^ SCF^SKP2^,^[^ ^26^ ^]^ CRL4^Cdt2^,^[^ ^27^ ^]^ and SCF^β‐TRCP[^ ^28^ ^]^ shown to mediate SETD8 proteolysis. Oppositely, USP17 is reported as a deubiquitinase to enhance SETD8 stability.^[^ ^29^ ^]^ In this study, we discovered that SUMOylation is a novel regulation of SETD8 stability in bladder tumor cells by utilizing our unbiased ProSRSA screening system.^[^ ^16^ ^]^ Further, we identified that the SUMOylation E3 ligase PIAS1 and the deSUMOylation enzyme SENP6 control this process. Considering that overactive SUMOylation pathway was observed in bladder tumor patient samples in recent studies^[^ ^30^ ^]^ and our unbiased screening result, it is likely that the elevated protein level of SETD8 is the result of the overactive SUMOylation in BC, which further enhances MYC methylation and tumor growth. Furthermore, a previous study has demonstrated that SETD8 is a transcriptional target of MYC,^[^ ^31^ ^]^ suggesting that MYC accumulation may promote the transcriptional regulation of SETD8. While SETD8 stabilizes MYC through methylation as demonstrated in our study, this interdependence establishes a potential positive feedback loop within the SETD8/MYC axis, which may critically sustain oncogenic signaling and tumor progression in BC.

Previous study has revealed that MYC can be methylated at two arginine residues by PRMT1, leading to the interaction between MYC and P300 and promoting downstream genes expression.^[^ ^32^ ^]^ However, methylation on a lysine residue of MYC has not been described before. Our current study pinpointed SETD8 as the bona fide lysine methyltransferase of MYC at K412 in BC cells through mass spectrometry assay, co‐transfection assays in cells, in vitro methylation assays, gene‐edited knock‐in cell lines and SETD8 specific inhibitor treatment. We further demonstrated that MYC K412 methylation antagonizes the E3 ligase CHIP‐mediated degradation, providing a novel explanation for the aberrant MYC accumulation in BC.

In summary, this study reveals that the SETD8/MYC axis is a novel regulatory mechanism essential for bladder tumor growth both in vitro and in vivo. We also provide strong supports that targeting SETD8 could be a promising therapeutic strategy for BC. The widely used substrate‐competitive inhibitor of SETD8, UNC0379, with an in vitro IC_50_ value of 7.3 µm, may exhibit off‐target effects due to its shared quinazoline core structure with high‐potency inhibitors of G9a and GLP.^[^ ^14^ ^]^ Therefore, further research is warranted to develop more effective therapeutic strategies targeting the SETD8/MYC axis in BC.

## 4 Experimental Section

### Ethics Approval

The paraffin‐embedded BC tissue samples were obtained from the Department of Pathology at Sun Yat‐sen University Cancer Center, with approval from the Ethics Committee of Sun Yat‐sen University Cancer Center (SL‐B2023‐642‐01). The animal study was approved by the Animal Research Committee of Sun Yat‐sen University Cancer Center (L025501202305020). All experiments adhered strictly to relevant ethical guidelines.

### Cell Culture

The cell lines HT1197, T24, J82, 5637, SCABER, RT4, UMUC3, and HEK‐293T were obtained from the American Type Culture Collection (ATCC). RT112 cell line was sourced from the European Collection of Authenticated Cell Cultures (ECACC), and BIU87 cell line was acquired from Kunming Cell Bank, Chinese Academy of Sciences. All cell lines were verified by short tandem repeat (STR) profiling. They were maintained in Dulbecco's modified Eagle's medium supplemented with 10% fetal bovine serum and 1% penicillin‐streptomycin solution under a 5% CO_2_ atmosphere at 37 °C. Mycoplasma contamination was monitored monthly using PCR to ensure all cultures remained negative.

### Plasmid Construction

MYC tagged with HA, V5, or SFB, along with its relative K‐to‐R mutants and truncates, was cloned into the pSIN‐EF1α‐puro vector. SFB‐ or HA‐tagged SETD8, as well as its K‐to‐R mutants and truncates, was also inserted into the pSIN‐EF1α‐puro vector. Additionally, SFB‐tagged CHIP, Fbw7, Truss, and Skp2 were cloned into the pSIN‐EF1α‐puro vector. 3xMYC‐tagged PIAS family and SENP family were inserted into the PCDNA3.1 vector. shRNAs targeting SETD8 were cloned into the pLKO.1 vector. And sgRNA targeting MYC or SETD8 was cloned into the lentiCRISPR‐V2‐puro vector. The sequences of these constructs are provided in Table S2 (Supporting Information).

### MTT Assay

The indicated cells were seeded in 96‐well plates at 2 000 cells per well and incubated for 24 h. Cells were incubated with 3‐(4,5‐dimethylthiazol‐2‐yl)‐2,5‐diphenyltetrazolium bromide (MTT) substrate (0.5 mg mL⁻^1^) for 4 h at 37 °C. The medium was removed then, and 100 µL dimethyl sulfoxide (DMSO) was added to each well. The plate was shaken for 10 min to dissolve the formazan crystals. The optical density (OD) at 570 nm was measured daily for 5 days using a microplate reader.

### Colony Formation Assay

The indicated cells were seeded in 6‐well plates at 500 cells per well for T24 cells and 1 000 cells per well for HT1197 cells. The cells were cultured for 7–10 days until visible colonies formed. Then paraformaldehyde was used to fix the colonies, which were subsequently stained with crystal violet (1%) overnight. Colony numbers were quantified by ImageJ.

### Western Blotting

Cells were harvested and lysed in RIPA buffer (50 mM Tris‐HCl, 150 mM NaCl, 5 m EDTA, 0.5% CA630, pH 8.0) supplemented with protease and phosphatase inhibitor cocktail (Roche). Cell lysates were obtained by centrifugation (12 000 x g, 4 °C) for 15 min. Samples were denatured at 100 °C for 10 min. Proteins were then separated by SDS‐PAGE and transferred to 0.45 µM polyvinylidene difluoride (PVDF) membranes (Millipore). After blocking with 5% nonfat milk at room temperature for 1 h, the immunoblots were performed referring to the standard process using the indicated primary antibodies against Flag (CST, 14 793), HA (CST, 3724), V5 (CST, 13 202), MYC‐tag (CST, 2278), SETD8 (CST, 2996), Mono‐methyl lysine (Abnova, MAB12422), GAPDH (Fudebio‐tech, FD0063), GFP (CST, 2956), β‐Tubulin (CST, 2146), SUMO2/3 (CST, 4971), PIAS1 (CST, 3550), and MYC (Abcam, ab32072).

### Co‐Immunoprecipitation (co‐IP)

The indicated cells were harvested and lysed in RIPA buffer containing inhibitor cocktail. The cell lysates were collected, with 40 µL supernatant reserved for evaluating the expression profiling in whole cell lysates. The remaining lysate was incubated with the indicated antibodies for 2 h at 4 °C, followed by the addition of protein A/G agarose and incubation overnight at 4 °C. For exogenous co‐IP, the cell lysates were directly incubated with anti‐FLAG, anti‐HA, or anti‐MYC agarose (Sigma Chemical Co.), and streptavidin beads (Cityva) at 4 °C overnight. The precipitates were washed with RIPA buffer five times and analyzed by western blotting.

### RNA Interference

siRNA oligonucleotides targeting SETD8 were transfected into the indicated cells with Lipofectamine RNAi MAX, following the manufacturer's instructions. Target sequences are the same as shRNAs targeting SETD8 listed in Table S2 (Supporting Information).

### Real‐Time qPCR

Total RNA was extracted using the Total RNA Purification Kit (TIANGEN) and then subjected to reverse transcription with the HiScript III 1st Strand cDNA Synthesis Kit (Vazyme). Real‐time qPCR was executed by a LightCycler 480 Instrument II (Roche) with SYBR Green master mix (Vazyme) according to the manufacturer's instructions. Relative sequences are listed in Table S3 (Supporting Information).

### Ubiquitination Assay

HT1197 cells were transfected with a His‐tagged ubiquitin‐expressing plasmid and incubated for 36 h. After incubation with 10 µm MG132 for 6 h, the cells were harvested with buffer A (6 m guanidine‐HCl, 0.1 m Na2HPO4/NaH2PO4, 10 mM imidazole, pH 8.0). The cell lysates were sonicated and incubated with Ni‐NTA beads (Cityva) for 3 h at room temperature. Then the precipitates were washed twice with buffer A, twice with buffer A/T1 mixture (1: 3, v/v), and once with buffer T1 (25 mM Tris‐HCl, 20 mM imidazole, pH 6.8). The samples were then subjected to western blotting to detect His‐ubiquitin‐modified proteins.

### Prokaryotic Protein Expression and Purification

Recombinant prokaryotic expression plasmids including GST‐MYC and GST‐SETD8 were transformed into *Escherichia coli* BL21(DE3) cells and expressed at 16 °C overnight with 1 mm IPTG (Biofroxx). The cells were harvested and resuspended in PBS buffer added with 500 mm NaCl, 1 mm DTT, and 1 mm PMSF, followed by lysing with an ultrahigh‐pressure homogenizer. The supernatant was collected after centrifugation at 18 000 x g for 45 min at 4 °C and filtered through a 0.22 µm filter. Proteins were enriched with pre‐equilibrated GST‐tag purification resin (Beyotime). After washing the resin with ten volumes of lysis buffer five times, proteins were eluted with elution buffer (lysis buffer supplemented with 20 mM GSH). The eluted proteins were further purified using a Superdex 200 Increase 10/300 GL column (GE Healthcare). Protein purity and size were assessed by SDS‐PAGE followed by Coomassie Blue staining. Protein concentrations were determined by measuring UV absorbance at 280 nm.

### In Vitro Methylation Assay

Prokaryotic purified lysine methyltransferase (1 µg) and substrate (2.5 µg) were combined in a reaction buffer consisting of 50 mm Tris‐HCl (pH 8.0), 20 mm KCl, 5 mm MgCl_2_, 1 mm PMSF, 1 mm DTT, and 10% glycerol. The reaction was initiated by the addition of 2 µg of S‐adenosylmethionine (SAM), the methyl donor. The mixture was incubated at 30 °C for 2 h, and subjected to western blotting to evaluate relative methylation level.

### SUMOylation Assay

For the endogenous SUMOylation assay, T24 cells were treated with 1µm TAK‐981, a SUMOylation inhibitor, or left untreated. The cells were then subjected to IP assay using either anti‐IgG or anti‐SETD8 antibody to detect the SUMOylation status of SETD8. For the exogenous SUMOylation assay, HA‐tagged SUMO1, SUMO2, or SUMO3 was co‐transfected with the indicated plasmids. After 36 h of transfection, cells were harvested for co‐IP analysis.

### Identification of Lysine‐Methylated Residue in MYC by Mass Spectrometry

HEK‐293T cells co‐expressing SFB‐MYC and SETD8 were collected and lysed. The cell lysates were incubated with streptavidin beads at 4 °C for 3 h to enrich SFB‐MYC proteins, which were eluted by biotin (2 mg mL⁻^1^) for 4 h. The elution was then incubated with pan‐mono‐methylated lysine antibody (CST, 14697S) and protein A/G agarose at 4 °C overnight. The beads were washed five times using RIPA buffer, and the proteins were separated by SDS‐PAGE. The band corresponding to 75 kDa was excised and sent for mass spectrometry to Wininnovate Bio (Shen Zhen, China).

### Immunohistochemistry Staining (IHC)

Paraffin‐embedded tissue samples were sectioned into 3 µm‐thick slices. The slides were deparaffinized and dehydrated, followed by antigen retrieval with EDTA‐Tris (pH 9.0). After blocking with goat serum for 30 min at room temperature, the slides were incubated with anti‐MYC diluted 1:100 (abcam, ab32072) or anti‐SETD8 diluted 1:400 (CST, 2996) at 4 °C overnight. After incubation, the slides were washed three times with PBS and treated with 3% hydrogen peroxide (H₂O₂) for 10 min to inhibit endogenous peroxidase activity. The slides were then incubated with anti‐rabbit IgG secondary antibody, and the staining was developed with DAB reagent (Dako Omnis). The expression levels of SETD8 in paratumor and tumor tissues were determined using the Halo system. IHC scores for the staining slides were obtained through ImageJ, with the expression levels subsequently categorized as high or low based on an established IHC cutoff.

### MYC K412R Knock‐In HT1197 Cell

An efficient sgRNA targeting MYC was selected, and a DNA donor template containing K412R mutation was cloned into the pSIN vector, omitting the EF1α promotor. To enhance selection efficiency for clones with homozygous mutations, sequences encoding EGFP were inserted into the intron between exon 3 and exon 4 of the donor template, with an internal ribosome entry site (IRES) placed upstream of EGFP. MYC‐targeting sgRNA and the corresponding donor template were co‐transfected into HT1197 cells. 48 h post‐transfection, the cells were digested and subjected to flow cytometry to sort EGFP‐positive single clones into 96‐well plates. Successful knock‐in clones were identified through PCR and Sanger sequencing. The MYC‐targeting sgRNA sequence is provided in Table S2 (Supporting Information).

### Xenograft Tumor Model

4‐week‐old male athymic nude mice were purchased from Vital River Laboratories (Beijing, China). The mice were subcutaneously injected with the indicated T24 cells (1 × 10^6^) or HT1197 cells (5 × 10^6^), mixed with Matrigel at a 1:1 ratio (n = 6 per group). For UNC0379 treatment, mice bearing knock‐in cell‐derived tumors were intraperitoneally administered UNC0379 (20 mg kg^−1^) or DMSO daily for 15 days. Tumor volume was measured every 3 days and calculated using the formula length x width^2^/2 (mm^3^). At the end of the experiment, the mice were sacrificed, and the xenografts were excised and weighed.

### RNA Sequencing

Total RNA of the indicated cells was extracted using TRIzol (Life Technologies). RNA sequencing was conducted by Novogene (Beijing, China). Data quality was validated, and clean reads were aligned to the human genome GRCh38 (Hg38) using STAR. Differentially expressed genes were identified using the DESeq2 algorithm, with criteria set to |log2 fold change| > 1 and *p* value < 0.05. sgRNA sequence targeting SETD8 is provided in Table S2 (Supporting Information).

### Protein Stability Regulators Screening Assay (ProSRSA) Targeting SETD8

ProSRSA assay was established according to a previous study.^[^ ^16^ ^]^ In short, T24 cells (2 × 10^7^) stably expressing psin‐DsRed‐P2A‐EGFP‐SETD8 were generated. The stable cells were infected with the lenti‐CRISPR library at a multiplicity of infection (MOI) of 0.3 for 48 h and selected by puromycin (0.5 µg mL⁻^1^). After a 7‐day selection, the cells were sorted by flow cytometry to isolate those exhibiting the top and bottom 5% of the EGFP/DsRed ratio.

### CUT&RUN Assay

The CUT&RUN assay was performed according to the manufacturer's instructions (Vazyme, HD102). Briefly, 250 000 cells were collected, washed with wash buffer, and incubated with ConA Beads Pro at room temperature for 10 min. The antibody was then added and incubated at 4 °C overnight. After washing, pG‐MNase Enzyme was added and incubated at 4 °C for 1 h, followed by two additional washes. CaCl_2_ was added and incubated on ice for 1 h, then stop buffer was added and incubated at 37 °C for 30 min. The supernatant, containing the released DNA, was collected and used for sequencing after library preparation. Spike‐in DNA was used for normalization.

### Statistical Analysis

All data are presented as the mean ± SD from three independent experiments, with bars representing the SD. Statistical analysis was performed with GraphPad Prism (version 10.1.2). Two‐tailed Student's *t*‐test was established to determine statistical significance between groups in three biological replicates. Spearman's correlation test was employed to estimate the Spearman correlation coefficient, while *p* values were calculated using Pearson's chi‐squared test. Overall survival was assessed using Kaplan‐Meier curves and compared with the log‐rank test. A *p* value of < 0.05 was considered statistically significant.

## Conflict of Interest

The authors declare no conflict of interest.

## Supporting information



Supporting Information

Supporting Information

## Data Availability

The data that support the findings of this study are available in the supplementary material of this article.
