# Supplementary material for: SUMOylation of SETD8 Promotes Tumor Growth by Methylating and Stabilizing MYC in Bladder Cancer
Source: Adv Sci (Weinh). 2025 Mar 16;12(18):2501734. doi: 10.1002/advs.202501734 (PMC12079334; doi:10.1002/advs.202501734)
Supplement: Supplementary file 1 — Supporting Information [file ADVS-12-2501734-s001.pdf]

ADVANCED SCIENCE
Open Access

Supporting Information

for *Adv. Sci.*, DOI 10.1002/advs.202501734

SUMOylation of SETD8 Promotes Tumor Growth by Methylating and Stabilizing MYC in Bladder Cancer

Xia Zhang, Zhenxuan Chen, Xiaobo He, Jingxuan Wang, Jianliang Zhong, Yezi Zou, Xianchong Zheng, Yujie Lin, Ruhua Zhang, Tiebang Kang, Liwen Zhou* and Yuanzhong Wu**

Supporting Information

SUMOylation of SETD8 Promotes Tumor Growth by Methylating and Stabilizing MYC in Bladder Cancer

Xia Zhang, Zhenxuan Chen, Xiaobo He, Jingxuan Wang, Jianliang Zhong, Yezi Zou, Xianchong Zheng, Yujie Lin, Ruhua Zhang, Tiebang Kang, Liwen Zhou,* and Yuanzhong Wu**

Supplementary Figures

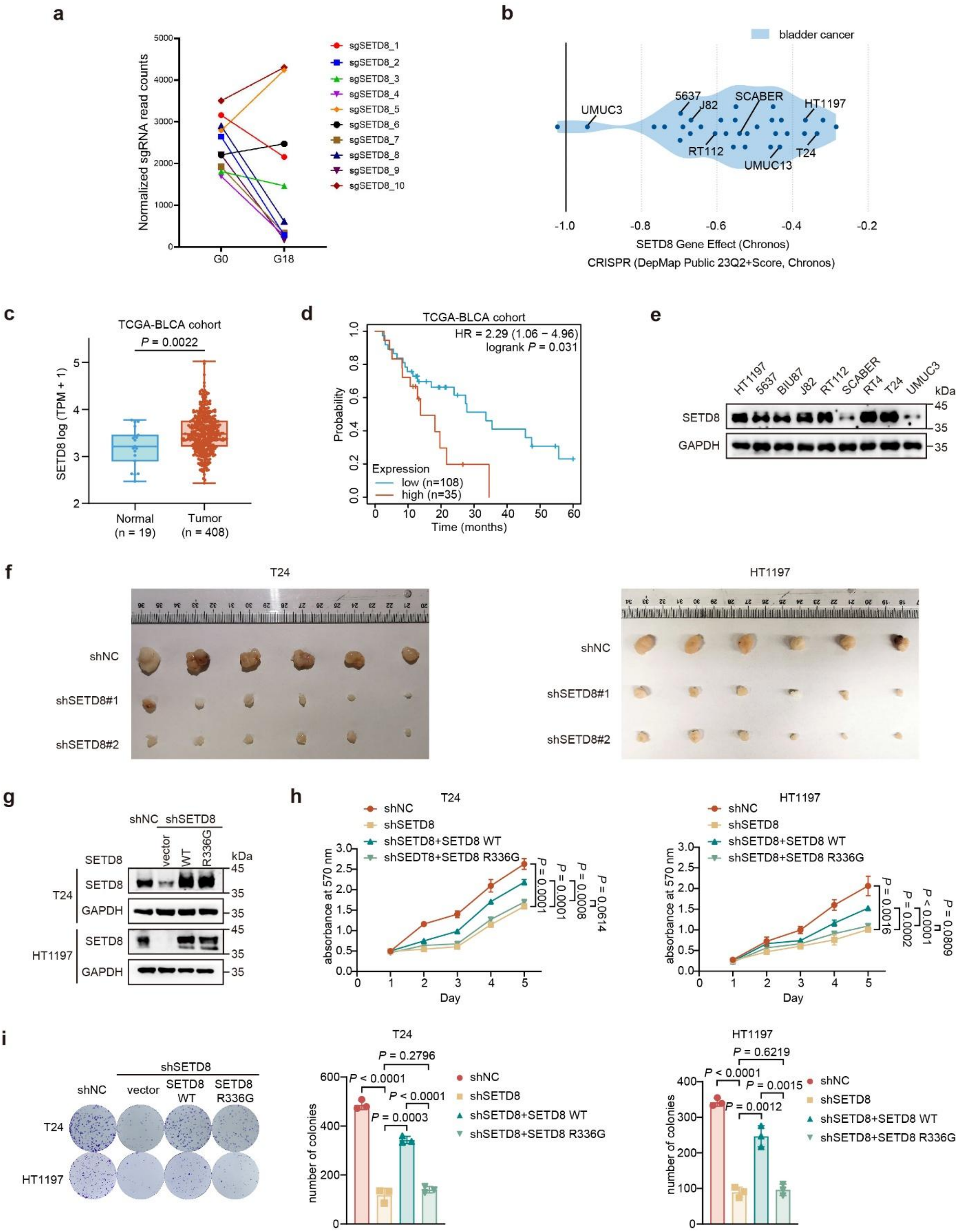

Figure S1. SETD8 promotes bladder tumor growth. a) Normalized read counts of sgRNAs targeting SETD8 in T24 cells. b) Gene effect of SETD8 in BC cell lines evaluated by CRISPR Chronos Score, as referenced from the Depmap portal. c) mRNA expression levels of SETD8 in normal and tumor tissues from the TCGA-BLCA cohort. d) Kaplan-Meier survival analysis of the TCGA-BLCA cohort, with high and low

SETD8 expression levels stratified according to the cutoff. e) Immunoblot analysis of SETD8 protein in BC cell lines. f) Xenografts excised from nude mice bearing tumors derived from the indicated T24 and HT1197 stable cell lines. g) Immunoblot analysis of the indicated protein in stable cell lines with SETD8 knockdown, and rescued with either wild-type (WT) SETD8 or its R336G mutant, the inactive form. h) and i) MTT and colony formation assay of stable cell lines with SETD8 knockdown and SETD8 rescue. Statistical significance was determined from three independent experiments. Data are presented as means ± SD, with *P* values calculated using Student's *t* test.

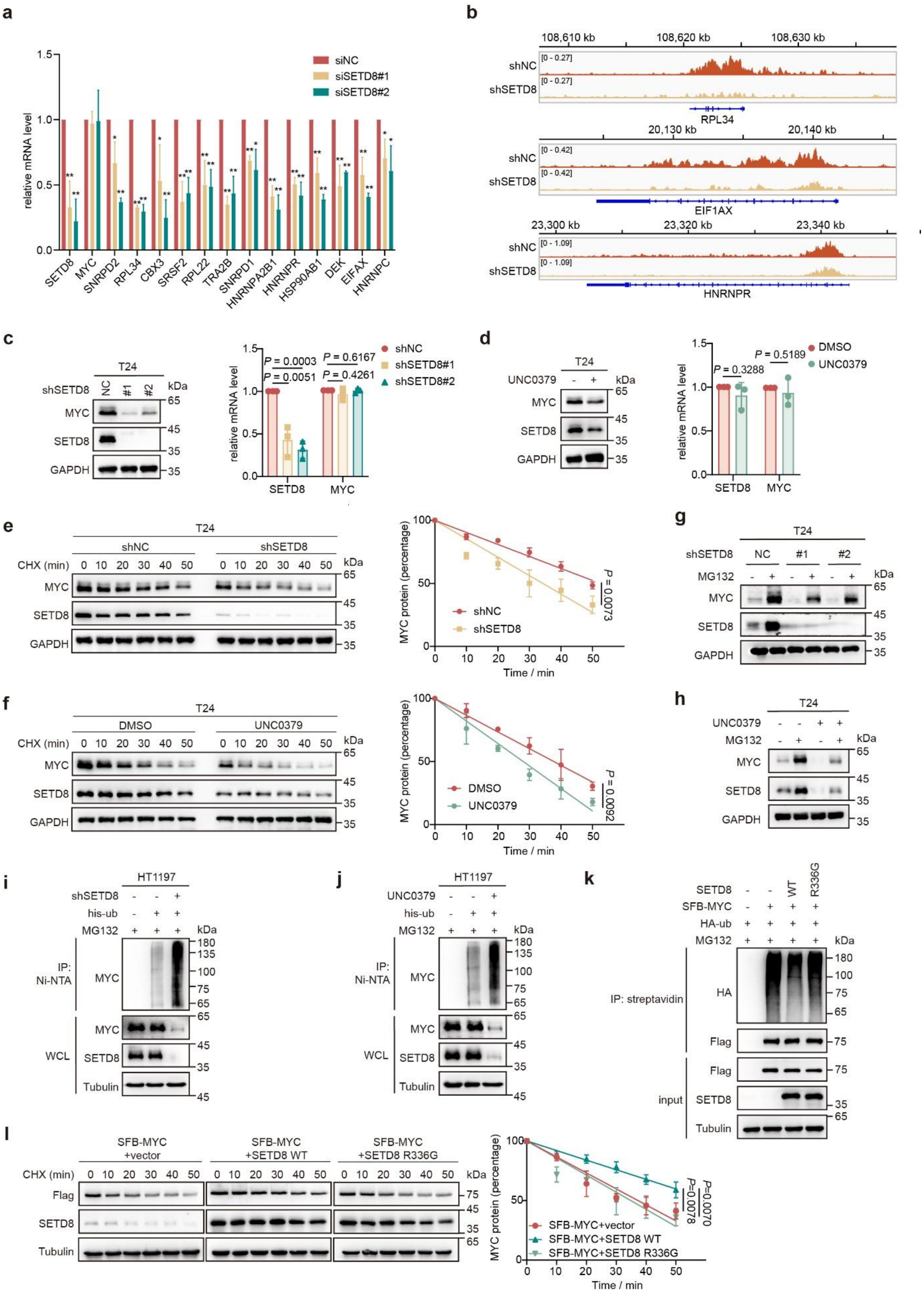

Figure S2. MYC protein is stabilized by SETD8. a) mRNA levels of MYC target genes in T24 cells transfected with SETD8 siRNA via qPCR. b) Genome browser view of H4K20me1 enrichment at selected loci of representative MYC target genes in

HT1197 cells with SETD8 knockdown. c) The protein and mRNA levels of the indicated genes in T24 stable cells. d) The protein and mRNA levels of the indicated genes in T24 cells treated with UNC0379 (5 μM, 24 h). e) Time-course analysis of the indicated proteins in T24 cells bearing SETD8 knockdown following treatment with CHX (20 μg/mL). Relative MYC protein levels were quantified using ImageJ. f) Time-course analysis of the indicated proteins in T24 cells following treatment with UNC0379 (5 μM, 24 h) and subsequent CHX (20 μg/mL). g) T24 cells expressing SETD8 shRNA were incubated with MG132 (10 μM, 6 h) and the indicated proteins were detected by western blotting. h) T24 cells were treated with UNC0379 (5 μM, 24 h) and then incubated with MG132 (10 μM, 6 h). Relative proteins were detected by western blotting. i) Immunoblot analysis of ubiquitination level of MYC in HT1197 cells with SETD8 knockdown following MG132 treatment (10 μM, 6 h). j) Immunoblot analysis of ubiquitination level of MYC in HT1197 cells treated with UNC0379 (5 μM, 24 h) followed by MG132 treatment (10 μM, 6 h). k) Immunoblot analysis of ubiquitination level of SFB-MYC when co-expressed with SETD8 WT or R336G mutant in HEK-293T cells after MG132 (10 μM, 6 h) treatment. l) Time-course analysis of SFB-MYC protein in HEK-293T cells expressing SETD8 wildtype (WT) or R336G mutant following CHX (20 μg/mL) treatment. Statistical significance was determined from three independent experiments. Data are presented as means ± SD, with *P* values calculated using Student's *t* test. **P* < 0.05, ***P* < 0.01, ****P* < 0.001, *****P* < 0.0001.

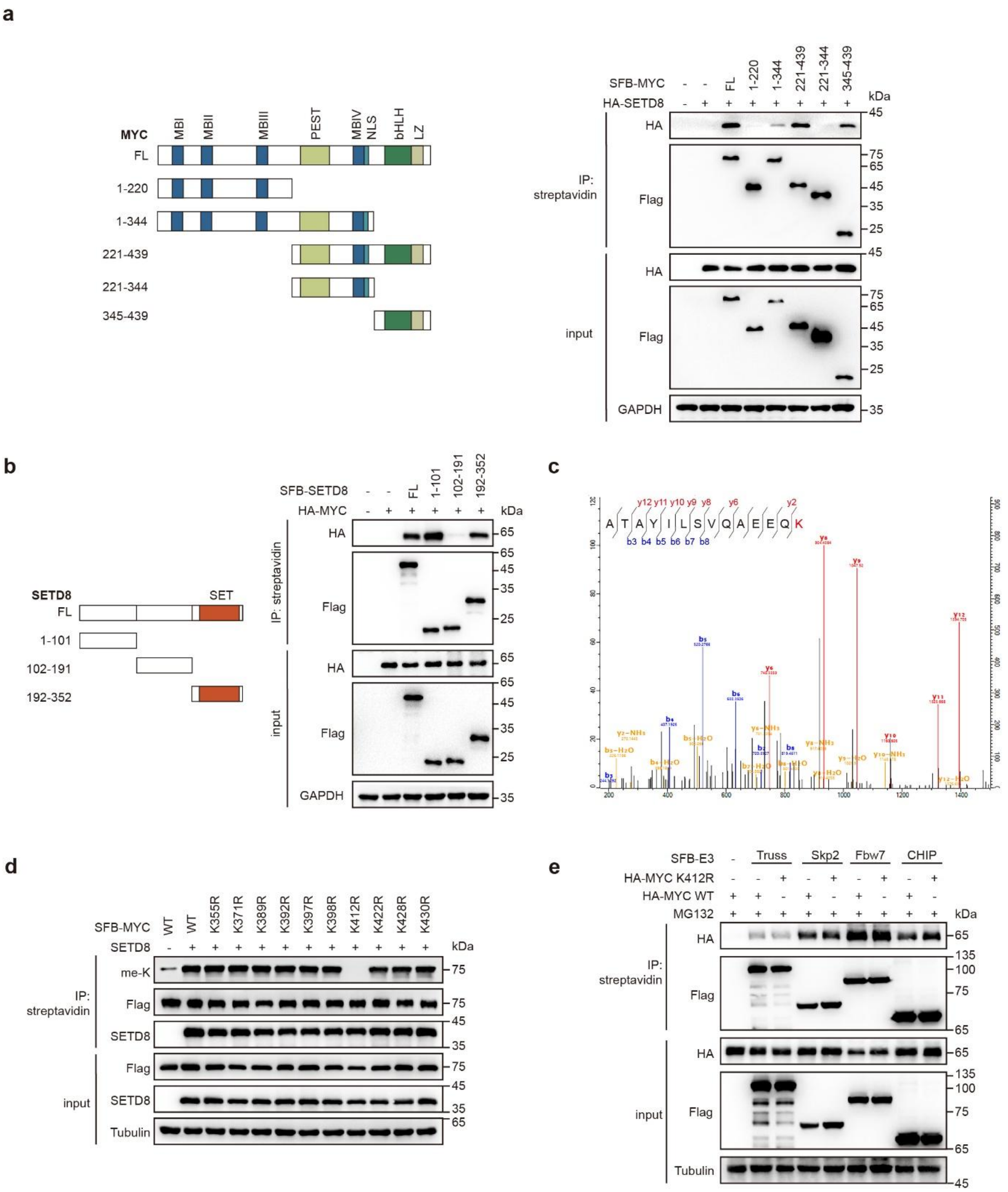

Figure S3. SETD8-mediated K412 methylation enhances MYC stability. a) Co-IP of HA-SETD8 and SFB-tagged MYC truncations in HEK-293T cells. FL, full length. b) Co-IP of SFB-MYC and HA-tagged SETD8 truncations in HEK-293T cells. c) Liquid chromatography-tandem mass spectrometry (LS-MS/MS) of the peptide ATAYILSVQAEEQK from MYC identified K412 as the mono-methylated residue. d) Immunoblot analysis of lysine methylation level of SFB-MYC with specific K-R mutations when co-expressed with SETD8 in HEK-293T cells. e) Co-IP of the ubiquitin

E3 ligases with HA-MYC WT or K412R mutant in HEK-293T cells following MG132 (10 μM, 6 h) treatment.

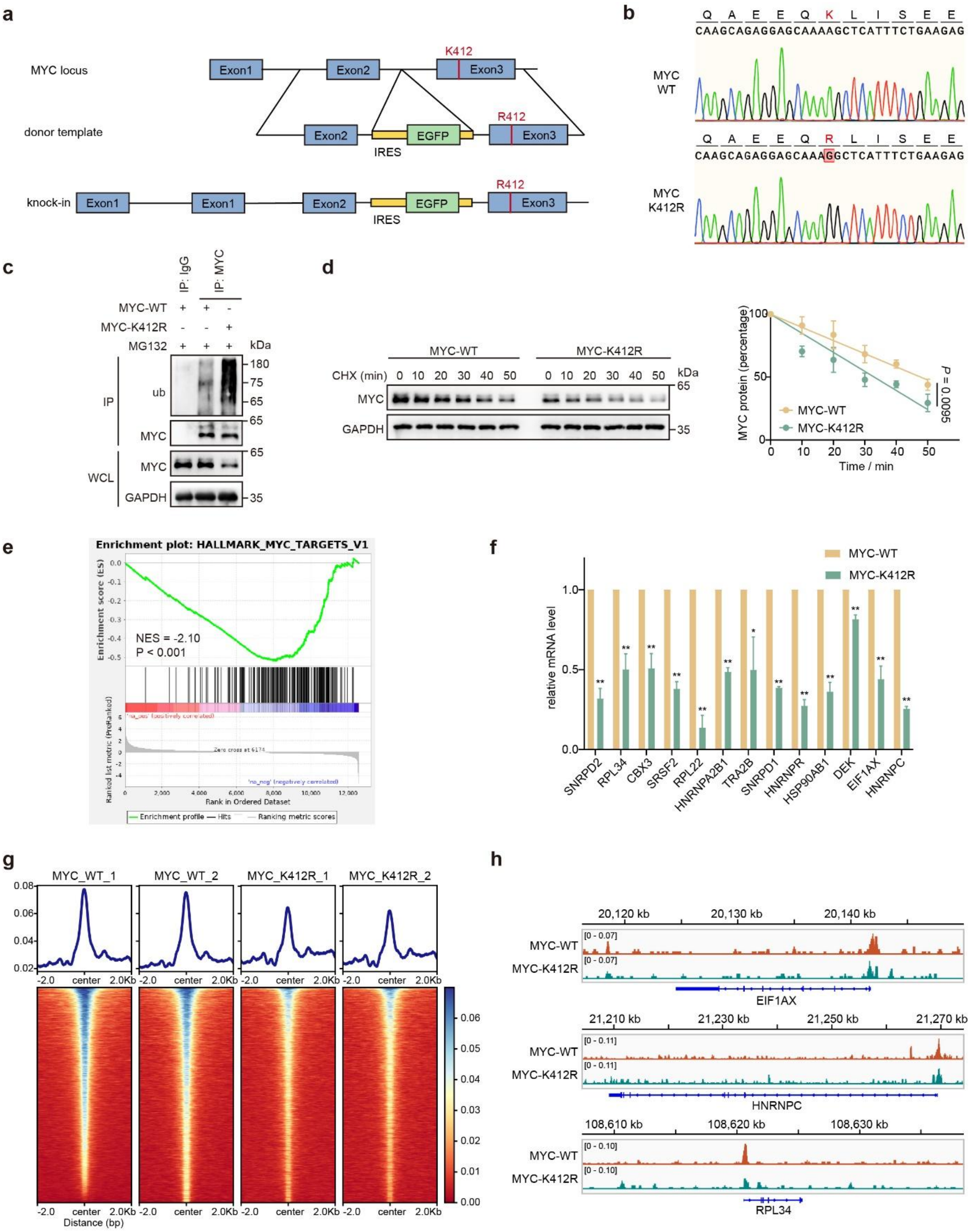

Figure S4. MYC-K412 methylation promotes tumor growth of BC. a) Scheme of endogenous edition with MYC-K412R mutation via CRISPR-Cas9 gene editing technology in HT1197 cells. b) Sanger sequencing peak map of single clone with

homozygous MYC-K412R mutation. c) Immunoblot analysis of endogenous ubiquitination level of MYC in knock-in cells following MG132 (10 μM, 6 h) treatment. d) Time-course analysis of MYC protein in MYC-WT or MYC-K412R knock-in cells upon CHX (20 μg/mL) treatment. e) GSEA of MYC target gene sets in the expression profiles of knock-in cells. f) mRNA levels of MYC target genes in knock-in cells via qPCR. g) Heat map of replicate data for MYC enrichment as detected by CUT&RUN assay in knock-in cells. h) Genome browser view of MYC enrichment at the selected loci of representatitve target genes. Three independent experiments were conducted to determine statistical significance. Data are presented as means ± SD. *P* values were calculated using Student's *t* test. $^{*}P < 0.05$, $^{**}P < 0.01$, $^{***}P < 0.001$, $^{****}P < 0.0001$.

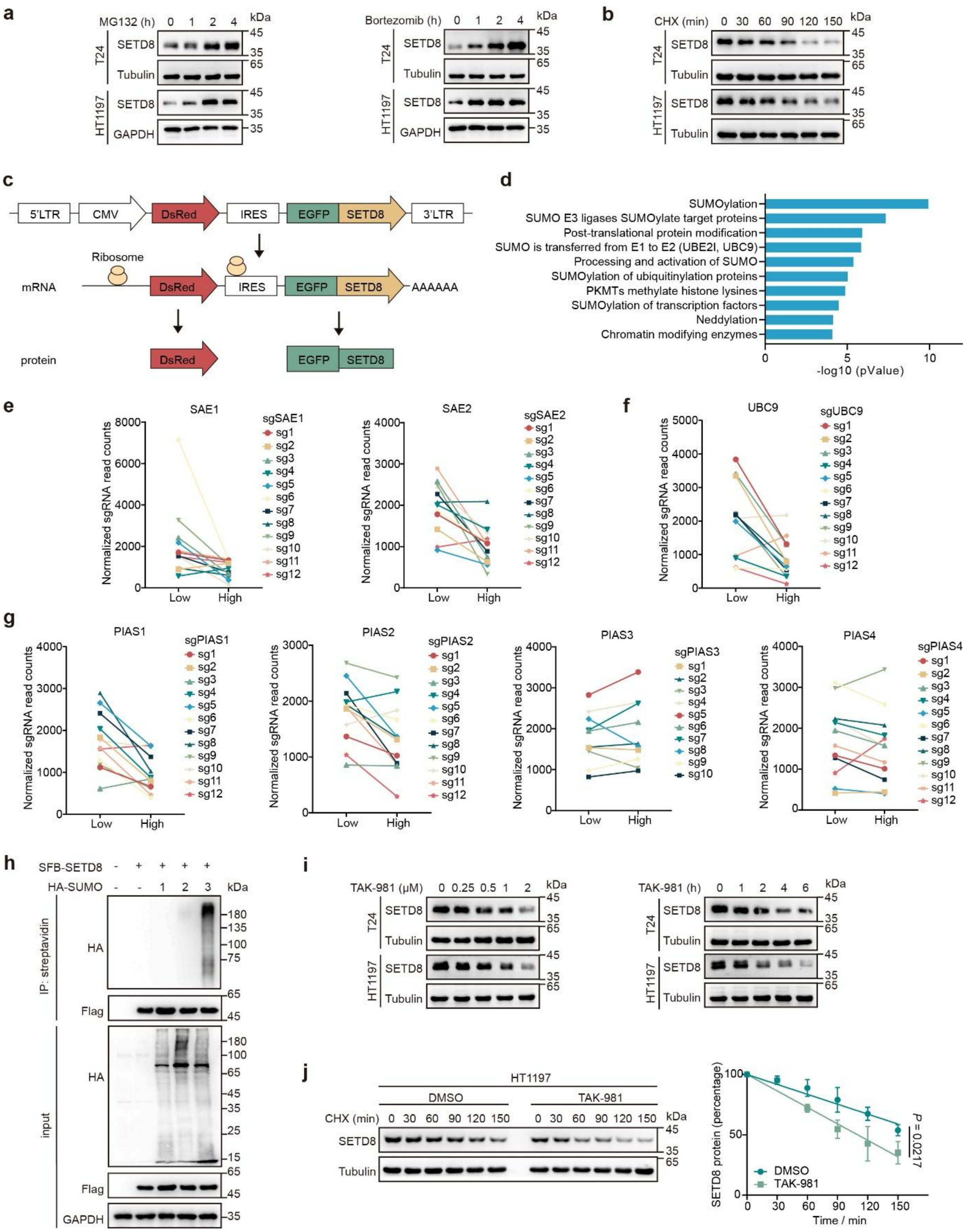

Figure S5. SUMOylation positively regulates SETD8 stability. a) Immunoblot analysis of SETD8 respond to MG132 (10 μM) and Bortezomib (100 nM) treatment for the indicated time in T24 and HT1197 cells. b) Time-course analysis of SETD8 in T24 and HT1197 cells upon CHX (20 μg/mL) treatment. c) Scheme of the Protein Stability Regulators Screening Assay (ProSRSA) targeting SETD8 in T24 cells. d) Reactome pathways enrichment for genes regulating SETD8 stability. e) Normalized read counts

of sgRNAs targeting SAE1 and SAE2 in T24 cells. f) Normalized read counts of sgRNAs targeting UBC9 in T24 cells. g) Normalized read counts of sgRNAs targeting PIAS1, PIAS2, PIAS3 and PIAS4 in T24 cells. h) Co-IP of ectopic SFB-SETD8 and HA-tagged SUMO1, SUMO2 or SUMO3 in HEK-293T cells. i) Immunoblot analysis of SETD8 protein in T24 and HT1197 cells under treatment of TAK-981 at various doses and time points. j) Time-course analysis of SETD8 protein in HT1197 cells incubated with TAK-981 (1 μM, 6 h), followed by the addition of CHX (20 μg/mL) at the indicated time points. Three independent experiments were conducted to determine statistical significance. Data are presented as means ± SD, and *P* values were calculated using Student's *t* test.

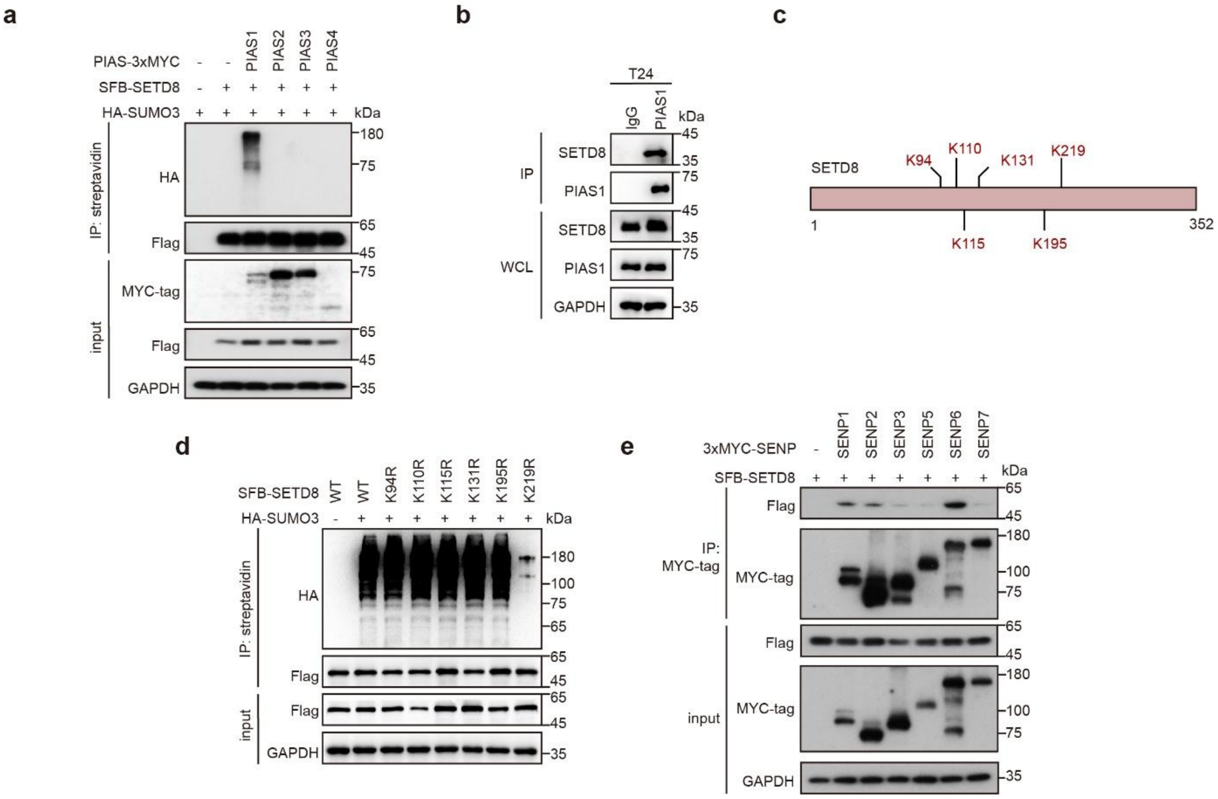

Figure S6. PIAS1 and SENP6 modulate the SUMOylation of SETD8. a) Co-IP of PIAS family proteins and SETD8 in HEK-293T cells, and SUMOylation level of SETD8 was detected by western blotting. b) Endogenous co-IP of PIAS1 and SETD8 in T24 cells. c) Predicted lysine residues responsible for SETD8 SUMOylation. d) Immunoblot analysis of SUMOylation level of SETD8 with mutations at potential residues in HEK-293T cells. e) Co-IP of SENP family proteins and SETD8 in HEK-293T cells.

Supplementary Tables

Table S1. Differential expression genes in RNA-seq analysis of T24 cells with SETD8 depletion (independent file).

Table S2. Target sequences of shRNAs and sgRNAs.

shRNA or sgRNA	Nucleotide sequence
SETD8 shRNA#1	GCAACTAGAGAGACAAATC
SETD8 shRNA#2	GTGGATGCAACTAGAGAGACA
SETD8 sgRNA#2	CTGAGTTCTCTTCCTGAAGG
SETD8 sgRNA#9	GAAGAGCAAAGCCGAGCTGC
MYC sgRNA	CAAATGCAACCTCACAACCT

Table S3 Primers used in RT-qPCR

Gene	Forward sequence (5'-3')	Reverse sequence (5'-3')
SETD8	ACAAATGCTCTGGAATGCGTT	CCGGCTAATGGTTTCCCCTG
MYC	CTGGTGCTCCATGAGGAGA	CCTGCCTCTTTTCCACAGAA
GAPDH	GAGCGAGATCCCTCCAAAAT	GGCTGTTGTCATACTTCTCATGG
SNRPD2	AGTCAAGAACAATACCCAAGTGC	ATGTTGCAGTGCCTATCGAAG
RPL34	GTTTGACATACCGACGTAGGC	GCACACATGGAACCACCATAG
CBX3	TAGATCGACGTGTAGTGAATGGG	TGTCTGTGGCACCAATTATTCTT
SRSF2	CCCGATGTGGAGGGTATGAC	GAGACTTCGAGCGGCTGTAG
RPL22	AAAGTGAACGGAAAAGCTGGG	TCACGGTGATCTTGCTCTTGC
TRA2B	GCGTCATGTTGGGAATCGG	CTTGAACGCCTAGACTGCTGG
SNRPD1	GAATTGAAGAACGGAACACAGGT	TCCACAAGTAGTGTATCCAGAGG
HNRNP-A2B1	ATTGATGGGAGAGTAGTTGAGCC	AATTCCGCCAACAAACAGCTT
HNRNPR	GCAAGGTGCAAGAGTCCACA	CACGCCAGAGTACACACTGTC
DEK	AACTGCTTTACAACAGGCCAG	ATGGTTTGCCAGAAGGCTTTG
EIF1AX	AACAGACGCAGGGGTAAGAAT	CCTGAGCATACTCCTGACCAT

HSP90A-B1	CGAAGTTGGACAGTGGTAAAGAG	TGCCCAATCATGGAGATGTCT
HNRNPC	CCCTTCTCCGTCCCCTCTAC	CCCGAGCAATAGGAGGAGGA
