# Supplementary material for: SUMOylation of SETD8 Promotes Tumor Growth by Methylating and Stabilizing MYC in Bladder Cancer
Source: Adv Sci (Weinh). 2025 Mar 16;12(18):2501734. doi: 10.1002/advs.202501734 (PMC12079334; doi:10.1002/advs.202501734)
Supplement: Supplementary file 2 — Supporting Information [file ADVS-12-2501734-s002.pdf]

ADVANCED SCIENCE
Open Access

# Supporting Information

for *Adv. Sci.*, DOI 10.1002/advs.202501734

SUMOylation of SETD8 Promotes Tumor Growth by Methylating and Stabilizing MYC in Bladder Cancer

*Xia Zhang, Zhenxuan Chen, Xiaobo He, Jingxuan Wang, Jianliang Zhong, Yezi Zou, Xianchong Zheng, Yujie Lin, Ruhua Zhang, Tiebang Kang*, Liwen Zhou* and Yuanzhong Wu**

**SUMOylation of SETD8 Promotes Tumor Growth by Methylating and Stabilizing MYC in Bladder Cancer**

*Xia Zhang, Zhenxuan Chen, Xiaobo He, Jingxuan Wang, Jianliang Zhong, Yezi Zou, Xianchong Zheng, Yujie Lin, Ruhua Zhang, Tiebang Kang,* Liwen Zhou,* and Yuanzhong Wu**

**Table S1. Differential expression genes in RNA-seq analysis of T24 cells with SETD8 depletion.**

| gene | baseMean | log2FC | lfcSE | pvalue | padj | symbol | entrez |
|---|---|---|---|---|---|---|---|
| ENSG00000227232 | 259.6178041 | 0.009172934 | 0.106211 | 0.8922604 | 0.9564219 | NA | NA |
| ENSG00000241860 | 15.23569452 | -0.00039674 | 0.1340389 | 0.9851557 | NA | NA | NA |
| ENSG00000228463 | 59.89302498 | 0.009697795 | 0.1274909 | 0.8380859 | 0.9331898 | RPL23AP21 | 728481 |
| ENSG00000237094 | 14.69254096 | -0.008835564 | 0.1343497 | 0.7379213 | NA | NA | NA |
| ENSG00000225972 | 12.47236077 | 0.006734707 | 0.1346261 | 0.7853821 | NA | MTND1P23 | 100887749 |
| ENSG00000225630 | 6401.512688 | 0.13699345 | 0.0665429 | 0.019431 | 0.1015086 | MTND2P28 | 100652939 |
| ENSG00000237973 | 712.2589181 | -0.082652616 | 0.0962724 | 0.2493182 | 0.5154909 | MTCO1P12 | 107075141 |
| ENSG00000248527 | 5047.295094 | -0.03675946 | 0.0585082 | 0.4851954 | 0.7234177 | MTATP6P1 | 106480796 |
| ENSG00000198744 | 11.92555181 | 0.030171532 | 0.1393979 | 0.2136446 | NA | MTCO3P12 | 107075270 |
| ENSG00000224956 | 40.38174984 | 0.048012176 | 0.1413012 | 0.2550224 | NA | NA | NA |
| ENSG00000240618 | 9.348154281 | 0.013064724 | 0.1358341 | 0.5460813 | NA | NA | NA |
| ENSG00000228327 | 92.48533925 | 0.016529573 | 0.1247932 | 0.7554844 | 0.8946415 | NA | NA |
| ENSG00000237491 | 51.50215039 | 0.017914923 | 0.1302134 | 0.6820906 | 0.8543094 | NA | NA |
| ENSG00000230092 | 8.167665157 | -0.008073658 | 0.1358121 | 0.6517531 | NA | NA | NA |
| ENSG00000225880 | 50.26941709 | -0.011900497 | 0.1288236 | 0.7879126 | 0.9116361 | NA | NA |
| ENSG00000228794 | 552.4575967 | 0.146556112 | 0.1134877 | 0.069856 | 0.24026 | LINC01128 | 643837 |
| ENSG00000187634 | 34.88094911 | 0.016453974 | 0.1320482 | 0.6696934 | NA | SAMD11 | 148398 |
| ENSG00000188976 | 6062.518366 | 0.041841091 | 0.0545404 | 0.4003619 | 0.6583091 | NOC2L | 26155 |
| ENSG00000187961 | 839.917073 | 0.051715614 | 0.0840166 | 0.4378245 | 0.6880813 | KLHL17 | 339451 |
| ENSG00000187583 | 213.1934681 | 0.057538404 | 0.121586 | 0.3985719 | 0.6576803 | PLEKHN1 | 84069 |
| ENSG00000272512 | 39.08872635 | 1.522817392 | 0.4889799 | 7.36E-05 | NA | NA | NA |
| ENSG00000188290 | 556.2309356 | 1.539131519 | 0.132781 | 2.52E-32 | 1.96E-29 | HES4 | 57801 |
| ENSG00000187608 | 295.0151186 | 0.095922729 | 0.1281278 | 0.2066146 | 0.4622611 | ISG15 | 9636 |
| ENSG00000188157 | 13290.65546 | -0.182309042 | 0.0588441 | 0.0006866 | 0.0082771 | AGRN | 375790 |
| ENSG00000217801 | 124.9044748 | 0.111175236 | 0.1630861 | 0.1249554 | 0.346864 | NA | NA |
| ENSG00000131591 | 612.3543008 | 0.03050883 | 0.0895483 | 0.6486562 | 0.836587 | C1orf159 | 54991 |
| ENSG00000207607 | 8.687663691 | -0.018371683 | 0.1369349 | 0.3654974 | NA | MIR200A | 406983 |
| ENSG00000198976 | 9.680975555 | -0.006785254 | 0.1351887 | 0.7478003 | NA | MIR429 | 554210 |
| ENSG00000272141 | 55.94451549 | -0.109857951 | 0.1870194 | 0.0584594 | 0.2144002 | NA | NA |
| ENSG00000078808 | 3954.348325 | 0.104460336 | 0.0603029 | 0.0535352 | 0.2019848 | SDF4 | 51150 |
| ENSG00000176022 | 490.2621357 | 0.126024458 | 0.1139294 | 0.1120214 | 0.3247758 | B3GALT6 | 126792 |
| ENSG00000184163 | 23.60728174 | 0.010813804 | 0.1329856 | 0.7441512 | NA | C1QTNF12 | 388581 |
| ENSG00000160087 | 1601.451322 | 0.032683189 | 0.0696813 | 0.5863407 | 0.7971162 | UBE2J2 | 118424 |
| ENSG00000162572 | 54.65403703 | 0.494206023 | 0.8244974 | 0.0084153 | 0.0562349 | SCNN1D | 6339 |
| ENSG00000131584 | 775.6252317 | 0.199296876 | 0.1089119 | 0.0164959 | 0.0900413 | ACAP3 | 116983 |
| ENSG00000169972 | 529.4629867 | 0.188148294 | 0.1241411 | 0.0314249 | 0.1422309 | PUSL1 | 126789 |
| ENSG00000127054 | 1842.432269 | -0.116659777 | 0.0758436 | 0.0682305 | 0.2365689 | INTS11 | 54973 |
| ENSG00000240731 | 37.14110687 | -0.029446412 | 0.1348163 | 0.4513616 | NA | NA | NA |
| ENSG00000224051 | 695.8388594 | 0.047806468 | 0.08795 | 0.4833105 | 0.7217391 | CPTP | 80772 |
| ENSG00000107404 | 3257.417728 | 0.090475153 | 0.0638671 | 0.1094491 | 0.3202262 | DVL1 | 1855 |
| ENSG00000175756 | 2867.681115 | 0.03417963 | 0.0609801 | 0.5313988 | 0.7568918 | AURKAIP1 | 54998 |
| ENSG00000221978 | 4338.961013 | 0.142111202 | 0.0620281 | 0.0105877 | 0.0658978 | CCNL2 | 81669 |
| ENSG00000224870 | 486.1881354 | 0.125139016 | 0.1137022 | 0.1147032 | 0.3296812 | MRPL20-AS | 148413 |
| ENSG00000242485 | 1412.284931 | -0.114686087 | 0.0822485 | 0.0891675 | 0.2815804 | MRPL20 | 55052 |
| ENSG00000272455 | 59.85528782 | -0.066066457 | 0.1467002 | 0.2018014 | 0.45577 | NA | NA |
| ENSG00000179403 | 123.9255514 | -0.054847721 | 0.1308893 | 0.3688934 | 0.6315515 | VWA1 | 64856 |
| ENSG00000160072 | 2214.710689 | -0.097963699 | 0.0718616 | 0.1106191 | 0.3224226 | ATAD3B | 83858 |
| ENSG00000197785 | 2640.86218 | 0.07918953 | 0.0640789 | 0.1617453 | 0.4037928 | ATAD3A | 55210 |
| ENSG00000205090 | 18.96585653 | 0.029909841 | 0.1381206 | 0.3055896 | NA | TMEM240 | 339453 |
| ENSG00000160075 | 2118.701761 | 0.081845434 | 0.0724624 | 0.1851786 | 0.435663 | SSU72 | 29101 |
| ENSG00000215014 | 9.022594516 | 0.005822994 | 0.1351609 | 0.7847768 | NA | NA | NA |
| ENSG00000228594 | 61.40894953 | 0.052752604 | 0.1394285 | 0.2927699 | 0.5621971 | FNDC10 | 643988 |
| ENSG00000272106 | 76.61406219 | 0.03393716 | 0.1292384 | 0.5216743 | 0.7493455 | NA | NA |
| ENSG00000197530 | 337.1282906 | 0.045424719 | 0.1090978 | 0.5114478 | 0.7421105 | MIB2 | 142678 |
| ENSG00000248333 | 874.9853968 | 0.044689902 | 0.0828982 | 0.4996226 | 0.734688 | CDK11B | 984 |
| ENSG00000189339 | 1504.430885 | 0.085459269 | 0.074455 | 0.175166 | 0.4234507 | SLC35E2B | 728661 |
| ENSG00000008128 | 64.68480066 | 0.005254985 | 0.1266041 | 0.9147028 | 0.9662935 | CDK11A | 728642 |
| ENSG00000268575 | 57.80505274 | -0.00961333 | 0.1280543 | 0.8320201 | 0.9304765 | NA | NA |

| | | | | | | | |
|---|---|---|---|---|---|---|---|
| ENSG00000215790 | 124.7052247 | 0.050371247 | 0.1277309 | 0.4103929 | 0.6654357 | NA | NA |
| ENSG00000008130 | 2089.174953 | 0.069474731 | 0.0691176 | 0.2455043 | 0.5110917 | NADK | 65220 |
| ENSG00000078369 | 9318.144245 | -0.168379677 | 0.055162 | 0.0009049 | 0.0102321 | GNB1 | 2782 |
| ENSG00000178821 | 69.8217592 | 0.006688317 | 0.1256649 | 0.8929206 | 0.9568118 | TMEM52 | 339456 |
| ENSG00000067606 | 729.7794281 | 0.038933032 | 0.0878155 | 0.5667921 | 0.7829587 | PRKCZ | 5590 |
| ENSG00000182873 | 8.465901271 | -0.009833192 | 0.1355812 | 0.6316835 | NA | PRKCZ-AS1 | 100506504 |
| ENSG00000162585 | 766.4725825 | -0.001169518 | 0.0831544 | 0.984993 | 0.9940394 | FAAP20 | 199990 |
| ENSG00000269554 | 19.58581446 | 0.000400571 | 0.1334359 | 0.9909372 | NA | NA | NA |
| ENSG00000157933 | 1685.581505 | -0.006894533 | 0.0701386 | 0.9079687 | 0.9638297 | SKI | 6497 |
| ENSG00000116151 | 113.9567299 | 0.085840874 | 0.1493153 | 0.1852901 | 0.435663 | MORN1 | 79906 |
| ENSG00000269896 | 10.72858682 | 0.024773189 | 0.1380727 | 0.2747649 | NA | LOC100129! | 100129534 |
| ENSG00000157916 | 2620.801299 | 0.029557751 | 0.0628822 | 0.5982049 | 0.805834 | RER1 | 11079 |
| ENSG00000157911 | 500.25883 | -0.01012425 | 0.0929121 | 0.8806257 | 0.9518649 | PEX10 | 5192 |
| ENSG00000149527 | 17.32679293 | -0.047101934 | 0.1453597 | 0.1125395 | NA | PLCH2 | 9651 |
| ENSG00000157881 | 637.3623499 | -0.083590349 | 0.0988188 | 0.2493385 | 0.5154909 | PANK4 | 55229 |
| ENSG00000272449 | 22.7531259 | 0.023716942 | 0.1354649 | 0.4664501 | NA | NA | NA |
| ENSG00000238164 | 11.96099686 | -0.003339116 | 0.1344927 | 0.8878354 | NA | NA | NA |
| ENSG00000157873 | 299.8790649 | -0.029837391 | 0.1059675 | 0.6618775 | 0.8435779 | TNFRSF14 | 8764 |
| ENSG00000157870 | 1950.731801 | 0.186479903 | 0.0809257 | 0.0066165 | 0.046926 | PRXL2B | 127281 |
| ENSG00000215912 | 12.03478728 | 0.046715871 | 0.1465282 | 0.0554859 | NA | TTC34 | 100287898 |
| ENSG00000130762 | 630.6518465 | -0.060417375 | 0.0933262 | 0.3875998 | 0.6479967 | ARHGEF16 | 27237 |
| ENSG00000162591 | 181.8675321 | 0.662609463 | 0.216233 | 0.0001128 | 0.0019234 | MEGF6 | 1953 |
| ENSG00000158109 | 867.6399903 | 0.062116979 | 0.0877406 | 0.3647218 | 0.6272549 | TPRG1L | 127262 |
| ENSG00000116213 | 748.7299092 | -0.00940547 | 0.0837649 | 0.8861794 | 0.9543156 | WRAP73 | 49856 |
| ENSG00000078900 | 576.3603635 | 0.095040436 | 0.1012342 | 0.1982126 | 0.4513782 | TP73 | 7161 |
| ENSG00000227372 | 306.8015058 | 0.083962409 | 0.1216904 | 0.2578522 | 0.5246424 | GFOD3P | 57212 |
| ENSG00000130764 | 1236.978086 | 0.144974208 | 0.0874364 | 0.0408657 | 0.1699365 | LRRC47 | 57470 |
| ENSG00000272153 | 9.111115164 | -0.001125695 | 0.1350394 | 0.9544248 | NA | NA | NA |
| ENSG00000116198 | 1148.306606 | 0.007193514 | 0.0762529 | 0.9102963 | 0.9647135 | CEP104 | 9731 |
| ENSG00000169598 | 155.6313811 | -0.108347785 | 0.1530556 | 0.145793 | 0.3783345 | DFFB | 1677 |
| ENSG00000198912 | 514.7935453 | 0.051682714 | 0.0978809 | 0.4637994 | 0.7079196 | C1orf174 | 339448 |
| ENSG00000236423 | 28.63896237 | 0.04759508 | 0.1436883 | 0.1833558 | NA | LINC01134 | 100133612 |
| ENSG00000131697 | 479.5911163 | 0.239388605 | 0.1430772 | 0.0146716 | 0.082922 | NPHP4 | 261734 |
| ENSG00000069424 | 377.0137793 | 0.047597101 | 0.1061027 | 0.4686006 | 0.7105963 | KCNAB2 | 8514 |
| ENSG00000116254 | 87.43395036 | 0.220321463 | 0.3525838 | 0.0232659 | 0.1150885 | CHD5 | 26038 |
| ENSG00000116251 | 9458.41841 | -0.237732381 | 0.0552001 | 3.81E-06 | 0.0001096 | RPL22 | 6146 |
| ENSG00000158286 | 515.6524697 | -0.382402702 | 0.1394968 | 0.0005485 | 0.0068962 | RNF207 | 388591 |
| ENSG00000116237 | 4156.049199 | 0.149003491 | 0.0624606 | 0.0077899 | 0.0529956 | ICMT | 23463 |
| ENSG00000225077 | 29.86609223 | 0.058763752 | 0.1492913 | 0.1266259 | NA | NA | NA |
| ENSG00000158292 | 124.1180865 | 0.064183622 | 0.135884 | 0.3010082 | 0.5707918 | GPR153 | 387509 |
| ENSG00000097021 | 2336.873222 | -0.028458504 | 0.0645305 | 0.6159753 | 0.8158757 | ACOT7 | 11332 |
| ENSG00000215788 | 143.428916 | 0.334237216 | 0.2821387 | 0.0129788 | 0.0764141 | TNFRSF25 | 8718 |
| ENSG00000171680 | 560.4415695 | 0.538473287 | 0.1242609 | 1.07E-06 | 3.58E-05 | PLEKHG5 | 57449 |
| ENSG00000162408 | 1421.992355 | -0.063074774 | 0.0772774 | 0.3254819 | 0.5941598 | NOL9 | 79707 |
| ENSG00000204859 | 455.3560402 | 0.262542363 | 0.1389894 | 0.0083058 | 0.0556944 | ZBTB48 | 3104 |
| ENSG00000162413 | 1605.862371 | 0.746257154 | 0.0919208 | 3.01E-17 | 6.63E-15 | KLHL21 | 9903 |
| ENSG00000116273 | 1013.132313 | 0.019391941 | 0.0804251 | 0.7666055 | 0.8994294 | PHF13 | 148479 |
| ENSG00000041988 | 340.2283712 | 0.045899876 | 0.106527 | 0.5136455 | 0.7440343 | THAP3 | 90326 |
| ENSG00000007923 | 2214.527353 | -0.027913792 | 0.0643044 | 0.6258366 | 0.8208396 | DNAJC11 | 55735 |
| ENSG00000237436 | 9.12835594 | -0.010762859 | 0.1356749 | 0.6005897 | NA | NA | NA |
| ENSG00000171735 | 877.2537267 | 0.012260774 | 0.0812311 | 0.8519881 | 0.9396727 | CAMTA1 | 23261 |
| ENSG00000049245 | 2405.610862 | -0.03105553 | 0.0638828 | 0.5807273 | 0.7928326 | VAMP3 | 9341 |
| ENSG00000269925 | 17.22277715 | 0.008090231 | 0.1339034 | 0.777398 | NA | NA | NA |
| ENSG00000049246 | 605.5947236 | -0.011901427 | 0.0901498 | 0.8588696 | 0.9430772 | PER3 | 8863 |
| ENSG00000116288 | 4515.811013 | -0.175278524 | 0.0655024 | 0.0026671 | 0.0235843 | PARK7 | 11315 |
| ENSG00000116285 | 7383.107497 | 0.411732153 | 0.0688021 | 2.38E-10 | 1.88E-08 | ERRFI1 | 54206 |
| ENSG00000162426 | 33.08470777 | 0.009520846 | 0.1313742 | 0.8020581 | NA | SLC45A1 | 50651 |
| ENSG00000142599 | 1284.823575 | 0.04260411 | 0.0781173 | 0.5083554 | 0.7401841 | RERE | 473 |
| ENSG00000074800 | 55702.16118 | -0.135120535 | 0.0479802 | 0.0026333 | 0.02332 | ENO1 | 2023 |
| ENSG00000236269 | 29.09695714 | -0.02621817 | 0.1352425 | 0.4568267 | NA | NA | NA |
| ENSG00000180758 | 80.6855234 | 0.021048641 | 0.1257696 | 0.6912376 | 0.8593387 | GPR157 | 80045 |
| ENSG00000049239 | 172.149166 | 0.402910261 | 0.2383499 | 0.0052487 | 0.0394783 | H6PD | 9563 |
| ENSG00000171621 | 375.2300857 | 0.412001637 | 0.1532555 | 0.0005713 | 0.0071217 | SPSB1 | 80176 |
| ENSG00000171612 | 509.1970354 | 0.037355044 | 0.0946179 | 0.5944069 | 0.8031487 | SLC25A33 | 84275 |
| ENSG00000188807 | 950.5489901 | 0.295960793 | 0.1003463 | 0.0004604 | 0.0060019 | TMEM201 | 199953 |
| ENSG00000171608 | 642.6785074 | -0.00917767 | 0.0912088 | 0.8894354 | 0.9549168 | PIK3CD | 5293 |
| ENSG00000231789 | 56.32837916 | 0.020496899 | 0.1293067 | 0.6611671 | 0.8431324 | PIK3CD-AS2 | 101929074 |
| ENSG00000171603 | 4718.92518 | -0.146036631 | 0.0623614 | 0.008922 | 0.0588799 | CLSTN1 | 22883 |
| ENSG00000178585 | 1111.047267 | -0.199235699 | 0.1055068 | 0.0145861 | 0.0826284 | CTNNBIP1 | 56998 |
| ENSG00000162441 | 552.8725559 | -0.148838434 | 0.1154721 | 0.0678039 | 0.235778 | LZIC | 84328 |

| | | | | | | | | |
|---|---|---|---|---|---|---|---|---|
| ENSG00000173614 | 295.1589194 | 0.268396828 | 0.1826012 | 0.0152802 | 0.0853603 | NMNAT1 | | 64802 |
| ENSG00000130939 | 1210.250367 | -0.098484968 | 0.0813802 | 0.1390631 | 0.3680101 | UBE4B | | 10277 |
| ENSG00000054523 | 2336.78387 | -0.16360761 | 0.0805472 | 0.0157085 | 0.087045 | KIF1B | | 23095 |
| ENSG00000142657 | 2959.099855 | -0.227933272 | 0.067729 | 0.0001865 | 0.0028966 | PGD | | 5226 |
| ENSG00000175279 | 187.9984934 | 0.031506655 | 0.1148323 | 0.6310664 | 0.8246434 | CENPS | | 378708 |
| ENSG00000160049 | 3734.223337 | -0.023422759 | 0.0634008 | 0.67011 | 0.8487915 | DFFA | | 1676 |
| ENSG00000203469 | 17.70890234 | 0.018289851 | 0.1354233 | 0.5157716 | NA | NA | NA | |
| ENSG00000142655 | 535.5766901 | -0.031215164 | 0.0926322 | 0.6487439 | 0.836587 | PEX14 | | 5195 |
| ENSG00000130940 | 251.8744862 | -0.068302794 | 0.117976 | 0.3407455 | 0.6080428 | CASZ1 | | 54897 |
| ENSG00000120948 | 5013.506158 | -0.266217739 | 0.06342 | 5.26E-06 | 0.0001451 | TARDBP | | 23435 |
| ENSG00000271895 | 41.1624533 | -0.061465283 | 0.1483837 | 0.1625401 | 0.4047229 | NA | NA | |
| ENSG00000116649 | 2104.343916 | 0.126114367 | 0.0720582 | 0.0419187 | 0.172595 | SRM | | 6723 |
| ENSG00000171824 | 3057.063195 | -0.213321232 | 0.0690661 | 0.0005391 | 0.006808 | EXOSC10 | | 5394 |
| ENSG00000226849 | 15.86557739 | -0.050058612 | 0.1475383 | 0.072044 | NA | NA | NA | |
| ENSG00000230337 | 15.19938096 | 0.024647629 | 0.1371308 | 0.3613113 | NA | EXOSC10-A | | 105376736 |
| ENSG00000198793 | 3549.875167 | -0.041177305 | 0.0593393 | 0.4398854 | 0.6890575 | MTOR | | 2475 |
| ENSG00000120942 | 921.5001979 | 0.020203843 | 0.0801562 | 0.7566369 | 0.8952873 | UBIAD1 | | 29914 |
| ENSG00000204624 | 62.94487327 | 0.066912697 | 0.1493034 | 0.1736651 | 0.4209549 | DISP3 | | 57540 |
| ENSG00000116661 | 1820.561972 | -0.039054416 | 0.0732415 | 0.5278593 | 0.7546809 | FBXO2 | | 26232 |
| ENSG00000132879 | 1048.027178 | -0.037712047 | 0.0832752 | 0.5691147 | 0.7847255 | FBXO44 | | 93611 |
| ENSG00000116663 | 243.1841565 | -0.006221333 | 0.1076215 | 0.9243196 | 0.9709924 | FBXO6 | | 26270 |
| ENSG00000116670 | 1396.122533 | -0.036827032 | 0.0734278 | 0.5520405 | 0.772817 | MAD2L2 | | 10459 |
| ENSG00000177674 | 439.4733984 | 0.429899401 | 0.1436145 | 0.0002198 | 0.0033081 | AGTRAP | | 57085 |
| ENSG00000177000 | 645.2096515 | 0.007844972 | 0.0869937 | 0.9078598 | 0.9638297 | MTHFR | | 4524 |
| ENSG00000011021 | 580.6874533 | -0.017569836 | 0.0928807 | 0.796017 | 0.9151845 | CLCN6 | | 1185 |
| ENSG00000242349 | 64.12064731 | 0.033721438 | 0.1315692 | 0.4925922 | 0.7284428 | NA | NA | |
| ENSG00000116685 | 1338.510805 | 0.032230446 | 0.0769761 | 0.6140111 | 0.8154834 | KIAA2013 | | 90231 |
| ENSG00000083444 | 2154.725412 | -0.071872539 | 0.0681931 | 0.2247956 | 0.4852777 | PLOD1 | | 5351 |
| ENSG00000116688 | 4465.00496 | 0.043299073 | 0.0572142 | 0.4055317 | 0.6617305 | MFN2 | | 9927 |
| ENSG00000116691 | 531.1212516 | -0.035514201 | 0.0951935 | 0.6068481 | 0.8114231 | MIIP | | 60672 |
| ENSG00000048707 | 1609.935015 | -0.004416595 | 0.069374 | 0.9403251 | 0.9766571 | VPS13D | | 55187 |
| ENSG00000162496 | 122.7037105 | 0.005950849 | 0.1320475 | 0.8665035 | 0.9460348 | DHRS3 | | 9249 |
| ENSG00000116731 | 1016.843255 | -0.085504505 | 0.0844106 | 0.2041265 | 0.4594224 | PRDM2 | | 7799 |
| ENSG00000189337 | 63.65385645 | 0.041581148 | 0.1338534 | 0.4092752 | 0.6643076 | KAZN | | 23254 |
| ENSG00000171729 | 264.0065867 | -0.030821305 | 0.1092423 | 0.6470774 | 0.8359336 | TMEM51 | | 55092 |
| ENSG00000142634 | 2176.76708 | 0.050395557 | 0.0709231 | 0.406811 | 0.6629727 | EFHD2 | | 79180 |
| ENSG00000132906 | 205.9740029 | -0.163961034 | 0.2013311 | 0.0595042 | 0.2170464 | CASP9 | | 842 |
| ENSG00000116138 | 393.9019604 | -0.01315315 | 0.1024056 | 0.8455143 | 0.936867 | DNAJC16 | | 23341 |
| ENSG00000116771 | 118.6929108 | 0.005284696 | 0.1205475 | 0.9271161 | 0.9718632 | AGMAT | | 79814 |
| ENSG00000197312 | 1410.682683 | 0.184689052 | 0.0840256 | 0.0086994 | 0.0577698 | DDI2 | | 84301 |
| ENSG00000116786 | 2157.997077 | 0.084043964 | 0.0690655 | 0.160948 | 0.4027493 | PLEKHM2 | | 23207 |
| ENSG00000162458 | 1867.08239 | 0.315453635 | 0.0793531 | 1.01E-05 | 0.0002556 | FBLIM1 | | 54751 |
| ENSG00000233954 | 46.22291706 | -0.04271881 | 0.1381729 | 0.3208219 | 0.5894181 | UQCRHL | | 440567 |
| ENSG00000179743 | 97.15308151 | -0.064231689 | 0.1400186 | 0.2683054 | 0.5361059 | SPEN-AS1 | | 729614 |
| ENSG00000065526 | 5689.197232 | -0.014781696 | 0.0637112 | 0.7923494 | 0.9130225 | SPEN | | 23013 |
| ENSG00000116809 | 739.0972222 | 0.282981437 | 0.1168927 | 0.0022062 | 0.0204172 | ZBTB17 | | 7709 |
| ENSG00000185519 | 33.51299777 | 0.046419769 | 0.1415065 | 0.2431692 | NA | FAM131C | | 348487 |
| ENSG00000142627 | 9582.544366 | 0.089623074 | 0.0591218 | 0.0923731 | 0.2874295 | EPHA2 | | 1969 |
| ENSG00000227959 | 11.41532533 | 0.059212757 | 0.1545654 | 0.011828 | NA | NA | NA | |
| ENSG00000224621 | 12.13093334 | 0.018609723 | 0.1362467 | 0.4482545 | NA | NA | NA | |
| ENSG00000142632 | 196.1410691 | 0.123031878 | 0.1546685 | 0.1215732 | 0.3414988 | ARHGEF19 | | 128272 |
| ENSG00000237276 | 9.907548043 | 0.030306966 | 0.1399374 | 0.1630263 | NA | NA | NA | |
| ENSG00000204377 | 22.36489242 | 0.021990094 | 0.1351465 | 0.4949908 | NA | NA | NA | |
| ENSG00000132881 | 65.61020433 | 0.068975156 | 0.1472064 | 0.2012489 | 0.4553999 | CPLANE2 | | 79363 |
| ENSG00000037637 | 671.9318636 | 0.032465532 | 0.0885845 | 0.6331815 | 0.8252838 | FBXO42 | | 54455 |
| ENSG00000055070 | 5025.479449 | -0.204239093 | 0.0630903 | 0.000357 | 0.004982 | SZRD1 | | 26099 |
| ENSG00000187144 | 35.42057431 | -0.032744867 | 0.1367077 | 0.3765032 | NA | SPATA21 | | 374955 |
| ENSG00000157191 | 1441.579066 | -0.084888177 | 0.0790698 | 0.1929412 | 0.4446136 | NECAP2 | | 55707 |
| ENSG00000226029 | 19.92317688 | -0.001378155 | 0.1330328 | 0.9616844 | NA | LINC01772 | | 107984921 |
| ENSG00000080947 | 91.27168134 | -0.121235071 | 0.1834893 | 0.0836261 | 0.2700934 | CROCCP3 | | 114819 |
| ENSG00000219481 | 2611.923956 | -0.092546306 | 0.0697245 | 0.1238595 | 0.3451342 | NBPF1 | | 55672 |
| ENSG00000215908 | 758.3016453 | 0.126000528 | 0.0993312 | 0.0945231 | 0.2917105 | NA | NA | |
| ENSG00000186301 | 27.90887584 | 0.022557508 | 0.1343947 | 0.5206786 | NA | NA | NA | |
| ENSG00000236698 | 13.65354447 | -0.010277942 | 0.1350398 | 0.666678 | NA | NA | NA | |
| ENSG00000058453 | 653.5757999 | -0.199558709 | 0.1147538 | 0.0192226 | 0.1009503 | CROCC | | 9696 |
| ENSG00000186715 | 19.03113578 | 0.049523493 | 0.1465759 | 0.1001736 | NA | MST1L | | 11223 |
| ENSG00000238142 | 17.30600037 | -0.003473773 | 0.1341963 | 0.8901615 | NA | LOC105376[illegible] | | 105376805 |
| ENSG00000117122 | 8.802804796 | 0.0023192 | 0.1351522 | 0.9109184 | NA | MFAP2 | | 4237 |
| ENSG00000159363 | 1656.423614 | 0.046291828 | 0.0714614 | 0.4484458 | 0.6958415 | ATP13A2 | | 23400 |
| ENSG00000117118 | 1780.084204 | 0.113513852 | 0.0746189 | 0.073008 | 0.2469116 | SDHB | | 6390 |

| | | | | | | | | |
|---|---|---|---|---|---|---|---|---|
| ENSG00000117115 | 8244.786462 | -0.260189677 | 0.23319 | 0.0231114 | 0.1145857 | PADI2 | | 11240 |
| ENSG00000204362 | 94.23352104 | 0.016753046 | 0.1252147 | 0.7509234 | 0.892513 | LINC02783 | | 400743 |
| ENSG00000142623 | 301.6872754 | 0.059308525 | 0.1109325 | 0.40642 | 0.6626733 | PADI1 | | 29943 |
| ENSG00000142619 | 23.07752237 | -0.071412826 | 0.1603613 | 0.0427283 | NA | PADI3 | | 51702 |
| ENSG00000179051 | 4809.08175 | -0.264303565 | 0.0636059 | 6.43E-06 | 0.0001716 | RCC2 | | 55920 |
| ENSG00000074964 | 159.2614689 | -0.003485365 | 0.1182433 | 0.951804 | 0.9806656 | ARHGEF10L | | 55160 |
| ENSG00000179023 | 14.42210723 | -0.067259851 | 0.1598761 | 0.0123741 | NA | KLHDC7A | | 127707 |
| ENSG00000159423 | 418.7629218 | 0.039062109 | 0.0988467 | 0.5754064 | 0.7890728 | ALDH4A1 | | 8659 |
| ENSG00000169991 | 1592.002893 | 0.072008299 | 0.0771865 | 0.2621583 | 0.5298047 | IFFO2 | | 126917 |
| ENSG00000272084 | 21.85128689 | -0.003829505 | 0.132838 | 0.9010166 | NA | NA | NA | |
| ENSG00000127481 | 11557.95496 | 0.054563092 | 0.0582379 | 0.3077659 | 0.5769089 | UBR4 | | 23352 |
| ENSG00000230424 | 22.42756614 | 0.016849995 | 0.1340662 | 0.601715 | NA | EMC1-AS1 | | 101927895 |
| ENSG00000127463 | 2509.680656 | 0.054406331 | 0.0640642 | 0.3385118 | 0.6060245 | EMC1 | | 23065 |
| ENSG00000053372 | 3004.601562 | 0.037254475 | 0.0685262 | 0.5306359 | 0.7563585 | MRTO4 | | 51154 |
| ENSG00000211454 | 18.62268346 | 0.005494625 | 0.1340633 | 0.8394114 | NA | AKR7L | | 246181 |
| ENSG00000162482 | 20.70112794 | 0.004423026 | 0.1329866 | 0.8889902 | NA | AKR7A3 | | 22977 |
| ENSG00000053371 | 1067.149607 | -0.016398439 | 0.0791375 | 0.7986561 | 0.9162772 | AKR7A2 | | 8574 |
| ENSG00000040487 | 535.3081695 | 0.317312923 | 0.1300257 | 0.0016919 | 0.0167768 | SLC66A1 | | 54896 |
| ENSG00000077549 | 4494.09648 | -0.174060415 | 0.0689967 | 0.004264 | 0.0335178 | CAPZB | | 832 |
| ENSG00000173436 | 162.6567927 | 0.140566404 | 0.1783328 | 0.0854317 | 0.2735548 | MICOS10 | | 440574 |
| ENSG00000226396 | 9.988426032 | 0.001848789 | 0.1350259 | 0.9322329 | NA | NA | NA | |
| ENSG00000158747 | 343.6354359 | 0.135971503 | 0.1314907 | 0.1015134 | 0.3050059 | NBL1 | | 4681 |
| ENSG00000162542 | 180.7799741 | 0.137744264 | 0.1658459 | 0.0956916 | 0.2938616 | TMCO4 | | 255104 |
| ENSG00000169914 | 730.7900423 | -0.059469905 | 0.0904056 | 0.3895871 | 0.6499457 | OTUD3 | | 23252 |
| ENSG00000162545 | 38.77207754 | 0.055716324 | 0.1452009 | 0.1955566 | NA | CAMK2N1 | | 55450 |
| ENSG00000090432 | 556.6082718 | 0.046082963 | 0.0936971 | 0.5075235 | 0.7397532 | MUL1 | | 79594 |
| ENSG00000183114 | 15.73978831 | 0.015847711 | 0.1350978 | 0.5655045 | NA | FAM43B | | 163933 |
| ENSG00000158828 | 682.7767412 | 0.083817566 | 0.1024766 | 0.2543296 | 0.5211459 | PINK1 | | 65018 |
| ENSG00000117242 | 172.6648282 | -0.020895978 | 0.1165734 | 0.7381676 | 0.885525 | PINK1-AS | | 100861548 |
| ENSG00000244038 | 3677.011066 | -0.102690247 | 0.0629303 | 0.0659566 | 0.2321298 | DDOST | | 1650 |
| ENSG00000117245 | 35.24125936 | 0.032734044 | 0.1356597 | 0.4123574 | NA | KIF17 | | 57576 |
| ENSG00000189410 | 1045.383127 | 0.513532835 | 0.107246 | 1.30E-07 | 5.99E-06 | SH2D5 | | 400745 |
| ENSG00000127483 | 5820.289115 | -0.220751064 | 0.0586156 | 4.38E-05 | 0.0008827 | HP1BP3 | | 50809 |
| ENSG00000203394 | 24.85150306 | -0.005103955 | 0.132719 | 0.8720689 | NA | NA | NA | |
| ENSG00000075151 | 1990.516051 | -0.207121807 | 0.076363 | 0.0018151 | 0.0176095 | EIF4G3 | | 8672 |
| ENSG00000266867 | 11.42918705 | -0.006896661 | 0.1347754 | 0.7690746 | NA | NA | NA | |
| ENSG00000117298 | 5864.410548 | -0.069635516 | 0.0553547 | 0.1679498 | 0.4131077 | ECE1 | | 1889 |
| ENSG00000227001 | 13.25350834 | -0.004645091 | 0.1343252 | 0.8520412 | NA | NA | NA | |
| ENSG00000142794 | 459.5683957 | -0.046712241 | 0.101577 | 0.5055179 | 0.738304 | NBPF3 | | 84224 |
| ENSG00000076864 | 64.47089934 | -0.027667607 | 0.1296628 | 0.5710883 | 0.786302 | RAP1GAP | | 5909 |
| ENSG00000090686 | 1681.184151 | -0.16032346 | 0.0808198 | 0.0179598 | 0.0960131 | USP48 | | 84196 |
| ENSG00000142798 | 243.9491572 | 0.938432536 | 0.1849807 | 2.09E-08 | 1.18E-06 | HSPG2 | | 3339 |
| ENSG00000218510 | 109.5617433 | 0.016940103 | 0.1214524 | 0.7704769 | 0.9020689 | LINC00339 | | 29092 |
| ENSG00000070831 | 4851.654846 | -0.396294893 | 0.0626245 | 2.74E-11 | 2.66E-09 | CDC42 | | 998 |
| ENSG00000184677 | 1607.477492 | 0.263978755 | 0.0822178 | 0.0002438 | 0.0036231 | ZBTB40 | | 9923 |
| ENSG00000237200 | 13.91449491 | 0.006196705 | 0.1344259 | 0.8080173 | NA | NA | NA | |
| ENSG00000133216 | 12.19387598 | 0.042163709 | 0.1442512 | 0.0814116 | NA | EPHB2 | | 2048 |
| ENSG00000004487 | 2225.88283 | -0.075179075 | 0.0686268 | 0.2074957 | 0.4635221 | KDM1A | | 23028 |
| ENSG00000169641 | 1606.805417 | 0.024263936 | 0.0719271 | 0.6924566 | 0.8594028 | LUZP1 | | 7798 |
| ENSG00000261326 | 55.01310242 | 0.065752241 | 0.149327 | 0.1693444 | 0.4151337 | NA | NA | |
| ENSG00000125944 | 6656.737833 | -0.285451832 | 0.0581768 | 1.66E-07 | 7.29E-06 | HNRNPR | | 10236 |
| ENSG00000125945 | 311.7439045 | 0.08142715 | 0.116659 | 0.2718455 | 0.5394882 | ZNF436 | | 80818 |
| ENSG00000249087 | 15.72747243 | 0.003517935 | 0.1340396 | 0.8963875 | NA | ZNF436-AS1 | | 148898 |
| ENSG00000088280 | 87.74994665 | 0.02495048 | 0.125522 | 0.6455749 | 0.8350083 | ASAP3 | | 55616 |
| ENSG00000007968 | 941.8304888 | 0.571438199 | 0.109576 | 1.33E-08 | 7.68E-07 | E2F2 | | 1870 |
| ENSG00000117318 | 900.9447154 | 0.636333011 | 0.1208612 | 9.24E-09 | 5.59E-07 | ID3 | | 3399 |
| ENSG00000142676 | 14774.63909 | -0.219795609 | 0.0525798 | 7.89E-06 | 0.0002063 | RPL11 | | 6135 |
| ENSG00000011007 | 1968.385402 | 0.025753715 | 0.0671886 | 0.6603577 | 0.8426737 | ELOA | | 6924 |
| ENSG00000236810 | 39.38242011 | 0.013655218 | 0.1309496 | 0.7347672 | NA | ELOA-AS1 | | 100506963 |
| ENSG00000057757 | 1222.240112 | 0.118626122 | 0.0848693 | 0.0855079 | 0.2737237 | PITHD1 | | 57095 |
| ENSG00000011009 | 1488.976134 | -0.105383373 | 0.0767328 | 0.1016839 | 0.3052607 | LYPLA2 | | 11313 |
| ENSG00000117308 | 657.6460342 | 0.180091651 | 0.1107506 | 0.0289915 | 0.1351895 | GALE | | 2582 |
| ENSG00000117305 | 369.7386659 | 0.453677043 | 0.1534545 | 0.0002295 | 0.0034419 | HMGCL | | 3155 |
| ENSG00000179163 | 335.1018413 | -0.099091166 | 0.1235303 | 0.1963599 | 0.4490367 | FUCA1 | | 2517 |
| ENSG00000189266 | 130.2551989 | -0.077123903 | 0.1412522 | 0.234566 | 0.497723 | PNRC2 | | 55629 |
| ENSG00000188529 | 653.7842836 | 0.045998669 | 0.0918212 | 0.5083545 | 0.7401841 | SRSF10 | | 10772 |
| ENSG00000142677 | 11.23724159 | 0.011588632 | 0.1354238 | 0.6124305 | NA | IL22RA1 | | 58985 |
| ENSG00000185436 | 15.22435939 | 0.01499588 | 0.1350565 | 0.5768648 | NA | IFNLR1 | | 163702 |
| ENSG00000001460 | 214.676188 | 0.123309327 | 0.1501063 | 0.1242913 | 0.3460073 | STPG1 | | 90529 |
| ENSG00000001461 | 325.6617452 | 0.017995253 | 0.1051499 | 0.7922714 | 0.9130225 | NIPAL3 | | 57185 |

| | | | | | | | | |
|---|---|---|---|---|---|---|---|---|
| ENSG00000117602 | 339.459293 | -0.087142672 | 0.115885 | 0.245479 | 0.5110917 | RCAN3 | | 11123 |
| ENSG00000133226 | 3045.662961 | 0.010862558 | 0.0604363 | 0.8393954 | 0.9341632 | SRRM1 | | 10250 |
| ENSG00000169504 | 7447.039741 | 0.068086648 | 0.0557029 | 0.1794602 | 0.4288613 | CLIC4 | | 25932 |
| ENSG00000117614 | 761.4462299 | 0.0678121 | 0.0891628 | 0.323349 | 0.5921054 | SYF2 | | 25949 |
| ENSG00000117616 | 876.6371829 | 0.004107032 | 0.1195566 | 0.9458739 | 0.9784014 | RSRP1 | | 57035 |
| ENSG00000183726 | 2771.970013 | -0.073797253 | 0.0650598 | 0.1983112 | 0.4513782 | TMEM50A | | 23585 |
| ENSG00000204178 | 444.6200442 | -0.212432122 | 0.1483199 | 0.0268333 | 0.1272591 | MACO1 | | 55219 |
| ENSG00000157978 | 850.807506 | -0.08987446 | 0.0907425 | 0.2013878 | 0.455463 | LDLRAP1 | | 26119 |
| ENSG00000162430 | 916.0954662 | 0.141229546 | 0.0965875 | 0.059313 | 0.2164435 | SELENON | | 57190 |
| ENSG00000117640 | 522.4861518 | -0.316541216 | 0.1303987 | 0.0017389 | 0.0170979 | MTFR1L | | 56181 |
| ENSG00000127423 | 309.6255516 | -0.005003318 | 0.1034107 | 0.938509 | 0.9761997 | AUNIP | | 79000 |
| ENSG00000182749 | 559.8425304 | 0.167617801 | 0.1199121 | 0.0469583 | 0.1854359 | PAQR7 | | 164091 |
| ENSG00000117632 | 8109.371029 | -0.066963456 | 0.0578219 | 0.1999164 | 0.4536176 | STMN1 | | 3925 |
| ENSG00000158006 | 193.3921437 | -0.145997053 | 0.1677374 | 0.0850644 | 0.2728275 | PAFAH2 | | 5051 |
| ENSG00000175087 | 168.4979427 | -0.061887645 | 0.1264262 | 0.355265 | 0.6192724 | PDIK1L | | 149420 |
| ENSG00000142684 | 845.3114187 | 0.128971505 | 0.0993905 | 0.0865322 | 0.2760948 | ZNF593 | | 51042 |
| ENSG00000236782 | 15.15318312 | 0.011331805 | 0.1346086 | 0.6732763 | NA | ZNF593OS | | 118568799 |
| ENSG00000130695 | 1323.886589 | 0.191923222 | 0.0912014 | 0.0099984 | 0.0635506 | CEP85 | | 64793 |
| ENSG00000142669 | 1644.207612 | 0.026347012 | 0.0699147 | 0.6616943 | 0.8434364 | SH3BGRL3 | | 83442 |
| ENSG00000158062 | 601.0378697 | 0.348348122 | 0.1238632 | 0.0005237 | 0.0067135 | UBXN11 | | 91544 |
| ENSG00000176092 | 9.263802249 | 0.009600157 | 0.1354541 | 0.6542581 | NA | CRYBG2 | | 55057 |
| ENSG00000117682 | 875.3094911 | 0.093375899 | 0.0919794 | 0.1876994 | 0.4386788 | DHDDS | | 79947 |
| ENSG00000198830 | 4951.951922 | -0.290719504 | 0.0667597 | 2.25E-06 | 6.82E-05 | HMGN2 | | 3151 |
| ENSG00000117676 | 1581.56477 | -0.179610485 | 0.0804176 | 0.0083616 | 0.0559726 | RPS6KA1 | | 6195 |
| ENSG00000260063 | 8.83938728 | 0.004806632 | 0.1354706 | 0.7991978 | NA | LOC101928 | | 101928728 |
| ENSG00000117713 | 3046.327791 | -0.015416636 | 0.0635716 | 0.7831608 | 0.9096561 | ARID1A | | 8289 |
| ENSG00000060642 | 198.137429 | 0.258812345 | 0.2147706 | 0.0215089 | 0.1092373 | PIGV | | 55650 |
| ENSG00000204160 | 1108.07975 | 0.063681219 | 0.0800781 | 0.3320613 | 0.5992396 | ZDHHC18 | | 84243 |
| ENSG00000175793 | 10146.36923 | 0.280940256 | 0.1260199 | 0.0033751 | 0.0279795 | SFN | | 2810 |
| ENSG00000142751 | 971.9561826 | 0.080259612 | 0.0842935 | 0.2374402 | 0.5019278 | GPN2 | | 54707 |
| ENSG00000198746 | 545.0495104 | 0.289119198 | 0.132726 | 0.0037754 | 0.0304936 | GPATCH3 | | 63906 |
| ENSG00000090273 | 5552.345199 | 0.044747548 | 0.0561082 | 0.3796117 | 0.6408794 | NUDC | | 10726 |
| ENSG00000253368 | 1949.759579 | 0.071906302 | 0.0734982 | 0.2524435 | 0.5191535 | TRNP1 | | 388610 |
| ENSG00000158246 | 190.780398 | -0.13965567 | 0.1669048 | 0.0921129 | 0.287261 | TENT5B | | 115572 |
| ENSG00000090020 | 957.7745897 | 0.161055662 | 0.0958612 | 0.0326986 | 0.1460619 | SLC9A1 | | 6548 |
| ENSG00000142784 | 761.6081599 | 0.065267863 | 0.0886209 | 0.339344 | 0.6066872 | WDTC1 | | 23038 |
| ENSG00000186501 | 796.0869124 | 0.378624719 | 0.1145374 | 9.47E-05 | 0.0016693 | TMEM222 | | 84065 |
| ENSG00000241547 | 21.47830548 | -0.017999732 | 0.1345059 | 0.5657925 | NA | NA | NA | |
| ENSG00000142765 | 66.34260902 | -0.077119489 | 0.1523349 | 0.1642258 | 0.4076092 | SYTL1 | | 84958 |
| ENSG00000142733 | 565.89159 | -0.161117274 | 0.1261139 | 0.0583824 | 0.2142522 | MAP3K6 | | 9064 |
| ENSG00000181773 | 168.0593082 | 0.051234474 | 0.1229302 | 0.4352739 | 0.6857883 | GPR3 | | 2827 |
| ENSG00000158195 | 3403.211003 | 0.040227796 | 0.0624432 | 0.4690815 | 0.7108642 | WASF2 | | 10163 |
| ENSG00000126705 | 598.1296598 | -0.034384776 | 0.0913398 | 0.6148082 | 0.8157779 | AHDC1 | | 27245 |
| ENSG00000126709 | 96.66626174 | -0.04042037 | 0.1289726 | 0.4706966 | 0.7115591 | IFI6 | | 2537 |
| ENSG00000009780 | 181.5826545 | 0.057999546 | 0.1227083 | 0.3920012 | 0.6522832 | FAM76A | | 199870 |
| ENSG00000117758 | 775.036991 | 0.076427774 | 0.0897248 | 0.2746809 | 0.5431373 | STX12 | | 23673 |
| ENSG00000117751 | 1361.479938 | 0.0617945 | 0.0759751 | 0.330776 | 0.5988522 | PPP1R8 | | 5511 |
| ENSG00000268552 | 33.53545703 | -0.025027416 | 0.134161 | 0.5032066 | NA | NA | NA | |
| ENSG00000130775 | 18.49672044 | 0.022539224 | 0.1359898 | 0.4456415 | NA | THEMIS2 | | 9473 |
| ENSG00000117748 | 1522.944912 | 0.058460913 | 0.0727205 | 0.3439387 | 0.6103841 | RPA2 | | 6118 |
| ENSG00000130768 | 11.06790469 | -0.031096375 | 0.1398611 | 0.1834957 | NA | SMPDL3B | | 27293 |
| ENSG00000158156 | 150.0468503 | -0.055465565 | 0.1269329 | 0.3882147 | 0.6485607 | XKR8 | | 55113 |
| ENSG00000158161 | 597.0074748 | -0.155394819 | 0.1116759 | 0.0546228 | 0.2046998 | EYA3 | | 2140 |
| ENSG00000228589 | 90.52269212 | -0.011550954 | 0.1242478 | 0.8238776 | 0.9274924 | NA | NA | |
| ENSG00000169403 | 9.557609444 | 0.012167817 | 0.1357667 | 0.5677935 | NA | PTAFR | | 5724 |
| ENSG00000126698 | 3673.699221 | -0.103509349 | 0.0636804 | 0.0662773 | 0.2326278 | DNAJC8 | | 22826 |
| ENSG00000130770 | 1043.499587 | -0.110932418 | 0.0925633 | 0.121891 | 0.3420972 | ATP5IF1 | | 93974 |
| ENSG00000270605 | 9.508197862 | -0.024391447 | 0.1381977 | 0.2502049 | NA | NA | NA | |
| ENSG00000130766 | 457.0502565 | 1.24125458 | 0.1465748 | 1.33E-18 | 3.54E-16 | SESN2 | | 83667 |
| ENSG00000130772 | 289.3623919 | 0.12607011 | 0.1378835 | 0.1226727 | 0.343137 | MED18 | | 54797 |
| ENSG00000204138 | 1185.729477 | -0.268631607 | 0.0961879 | 0.0008743 | 0.0099829 | PHACTR4 | | 65979 |
| ENSG00000180198 | 3864.779103 | -0.142698416 | 0.06304 | 0.0112579 | 0.0691758 | RCC1 | | 1104 |
| ENSG00000242125 | 1514.002003 | -0.232147722 | 0.0880639 | 0.0018123 | 0.0175974 | SNHG3 | | 8420 |
| ENSG00000200087 | 42.72364547 | -0.035203051 | 0.1357817 | 0.397138 | 0.6567942 | SNORA73B | | 26768 |
| ENSG00000180098 | 394.3902704 | -0.099585556 | 0.116995 | 0.1937357 | 0.4456981 | TRNAU1AP | | 54952 |
| ENSG00000197989 | 835.9488269 | 0.166773295 | 0.1035925 | 0.0345786 | 0.151483 | SNHG12 | | 85028 |
| ENSG00000221539 | 19.58124485 | 0.009179969 | 0.1335156 | 0.7640171 | NA | SNORD99 | | 692212 |
| ENSG00000120656 | 456.5383182 | 0.112740316 | 0.1125545 | 0.1466308 | 0.3799455 | TAF12 | | 6883 |
| ENSG00000188060 | 98.25321926 | 0.08131013 | 0.149137 | 0.1897649 | 0.440597 | RAB42 | | 115273 |
| ENSG00000162419 | 437.4157517 | -0.035966561 | 0.0974149 | 0.6035125 | 0.8089076 | GMEB1 | | 10691 |

| | | | | | | | |
|---|---|---|---|---|---|---|---|
| ENSG00000198492 | 1949.081827 | -0.017353694 | 0.0685782 | 0.787761 | 0.9116145 | YTHDF2 | 51441 |
| ENSG00000159023 | 800.835443 | -0.092722624 | 0.0914466 | 0.1894705 | 0.4403512 | EPB41 | 2035 |
| ENSG00000253304 | 476.2223266 | 0.102630183 | 0.1092117 | 0.1832974 | 0.4329849 | TMEM200B | 399474 |
| ENSG00000116350 | 2791.021964 | -0.219383991 | 0.0682974 | 0.0003246 | 0.0046129 | SRSF4 | 6429 |
| ENSG00000116353 | 429.9765985 | 0.270066562 | 0.1441104 | 0.0080724 | 0.0544419 | MECR | 51102 |
| ENSG00000060656 | 228.0815607 | 0.039345087 | 0.116161 | 0.5535316 | 0.7742549 | PTPRU | 10076 |
| ENSG00000162512 | 691.2125308 | 0.097230062 | 0.0956604 | 0.1751062 | 0.4234507 | SDC3 | 9672 |
| ENSG00000134644 | 2397.20421 | 0.033875433 | 0.0635352 | 0.5402878 | 0.7638709 | PUM1 | 9698 |
| ENSG00000060688 | 1683.892893 | -0.236586058 | 0.0806239 | 0.0007251 | 0.0086517 | SNRNP40 | 9410 |
| ENSG00000229447 | 12.16230476 | -0.026520949 | 0.1382172 | 0.2742793 | NA | NA | NA |
| ENSG00000121766 | 787.4126655 | -0.059251781 | 0.0866934 | 0.3829814 | 0.6435917 | ZCCHC17 | 51538 |
| ENSG00000229044 | 19.05177165 | 0.001102817 | 0.1331779 | 0.9726944 | NA | NA | NA |
| ENSG00000168528 | 115.5793547 | 0.070866112 | 0.1385833 | 0.2643046 | 0.5321877 | SERINC2 | 347735 |
| ENSG00000229167 | 10.28258852 | -0.008592672 | 0.1351275 | 0.7010607 | NA | NA | NA |
| ENSG00000142910 | 2213.976302 | 0.087255892 | 0.0714173 | 0.153855 | 0.3917987 | TINAGL1 | 64129 |
| ENSG00000162517 | 785.1119146 | 0.00050088 | 0.0842723 | 0.9698815 | 0.9881615 | PEF1 | 553115 |
| ENSG00000121753 | 123.771374 | 0.152066781 | 0.2089848 | 0.0595712 | 0.2170464 | ADGRB2 | 576 |
| ENSG00000184007 | 3291.347016 | -0.146098259 | 0.0644717 | 0.010707 | 0.0665256 | PTP4A2 | 8073 |
| ENSG00000121774 | 4602.782574 | -0.302902181 | 0.0613939 | 1.30E-07 | 5.99E-06 | KHDRBS1 | 10657 |
| ENSG00000121775 | 382.2119362 | -0.060181518 | 0.1060836 | 0.399805 | 0.6583091 | TMEM39B | 55116 |
| ENSG00000025800 | 2700.799898 | -0.061984152 | 0.0640683 | 0.2726451 | 0.5406158 | KPNA6 | 23633 |
| ENSG00000084652 | 4170.904232 | 0.023065246 | 0.0564954 | 0.6541469 | 0.8399571 | TXLNA | 200081 |
| ENSG00000160050 | 112.3773533 | -0.038291689 | 0.1260616 | 0.5114066 | 0.7421105 | CCDC28B | 79140 |
| ENSG00000224066 | 11.01434174 | -0.015007241 | 0.1359009 | 0.5098456 | NA | NA | NA |
| ENSG00000160051 | 187.0088984 | 0.685176587 | 0.218835 | 8.92E-05 | 0.0015883 | IQCC | 55721 |
| ENSG00000160055 | 81.34546873 | 0.114647818 | 0.1821357 | 0.0818443 | 0.2662274 | TMEM234 | 56063 |
| ENSG00000084623 | 5548.425479 | 0.074292229 | 0.0572445 | 0.1577626 | 0.3975891 | EIF3I | 8668 |
| ENSG00000220785 | 49.81963994 | 0.027969782 | 0.1322154 | 0.5287149 | 0.754996 | NA | NA |
| ENSG00000183615 | 14.52279565 | 0.067017314 | 0.1593288 | 0.0166654 | NA | FAM167B | 84734 |
| ENSG00000116478 | 3309.297084 | -0.174352382 | 0.0687581 | 0.0039813 | 0.0317229 | HDAC1 | 3065 |
| ENSG00000175130 | 1602.803473 | 0.42949096 | 0.0887776 | 1.24E-07 | 5.77E-06 | MARCKSL1 | 65108 |
| ENSG00000225828 | 157.8027319 | 0.081458003 | 0.1381819 | 0.2369821 | 0.5013017 | FAM229A | 100128071 |
| ENSG00000160058 | 1341.806418 | -0.060517646 | 0.0764494 | 0.3416539 | 0.6087837 | BSDC1 | 55108 |
| ENSG00000273274 | 9.981052296 | 0.009109208 | 0.1352437 | 0.6833424 | NA | ZBTB8B | 728116 |
| ENSG00000160062 | 124.738517 | 0.125578109 | 0.1758296 | 0.0968236 | 0.2959176 | ZBTB8A | 653121 |
| ENSG00000176261 | 647.9214208 | 0.104614095 | 0.1063891 | 0.1672758 | 0.4122331 | ZBTB8OS | 339487 |
| ENSG00000162521 | 4596.914718 | -0.053022418 | 0.0570255 | 0.3048254 | 0.5738381 | RBBP4 | 5928 |
| ENSG00000162520 | 114.5376108 | -0.048717017 | 0.130535 | 0.4055315 | 0.6617305 | SYNC | 81493 |
| ENSG00000162522 | 727.4731404 | -0.014106267 | 0.086965 | 0.8048241 | 0.9186624 | NHSL3 | 57648 |
| ENSG00000134684 | 4762.217836 | 0.106847556 | 0.0597013 | 0.0465136 | 0.1843029 | YARS1 | 8565 |
| ENSG00000116497 | 531.6734922 | 0.053324138 | 0.1002356 | 0.4517907 | 0.6981568 | S100PBP | 64766 |
| ENSG00000121905 | 10.47924044 | 0.018937063 | 0.1367418 | 0.3964085 | NA | HPCA | 3208 |
| ENSG00000121900 | 827.2006065 | 0.201379215 | 0.1029411 | 0.0125599 | 0.074815 | TMEM54 | 113452 |
| ENSG00000116514 | 706.5205228 | 0.289649199 | 0.1136367 | 0.0015155 | 0.0155284 | RNF19B | 127544 |
| ENSG00000004455 | 4547.391244 | -0.098577532 | 0.0610882 | 0.0708785 | 0.2423689 | AK2 | 204 |
| ENSG00000142920 | 19.43852841 | -0.005074173 | 0.1332404 | 0.8642472 | NA | AZIN2 | 113451 |
| ENSG00000116525 | 163.3006397 | -0.05661789 | 0.1261986 | 0.3862317 | 0.6470059 | TRIM62 | 55223 |
| ENSG00000160094 | 24.67446176 | 0.007748 | 0.1325242 | 0.8187361 | NA | ZNF362 | 149076 |
| ENSG00000134686 | 3256.082601 | 0.082196101 | 0.0621692 | 0.1375185 | 0.3657666 | PHC2 | 1912 |
| ENSG00000222112 | 12.03661067 | 0.018890942 | 0.1363415 | 0.4384639 | NA | RN7SKP16 | 106480837 |
| ENSG00000233246 | 9.764318383 | -0.004732178 | 0.1350612 | 0.8225247 | NA | PHC2-AS1 | 101929464 |
| ENSG00000121903 | 128.7032594 | 0.007707113 | 0.118158 | 0.8959981 | 0.9584283 | ZSCAN20 | 7579 |
| ENSG00000163866 | 975.1087384 | -0.148002935 | 0.0918181 | 0.0425039 | 0.1743286 | SMIM12 | 113444 |
| ENSG00000188910 | 52.48270088 | 0.584013811 | 0.6004066 | 0.0068397 | 0.0479562 | GJB3 | 2707 |
| ENSG00000116544 | 91.88009552 | 0.08344819 | 0.1508832 | 0.1789641 | 0.4279384 | DLGAP3 | 58512 |
| ENSG00000241014 | 22.3794413 | 0.028835649 | 0.1371852 | 0.3621104 | NA | GPR199P | 653160 |
| ENSG00000163867 | 192.2158749 | 0.015487119 | 0.1134826 | 0.8100138 | 0.9215712 | ZMYM6 | 9204 |
| ENSG00000197056 | 534.8204259 | -0.021945327 | 0.0920544 | 0.7473491 | 0.890205 | ZMYM1 | 79830 |
| ENSG00000116560 | 8780.008992 | -0.381985096 | 0.0579165 | 4.95E-12 | 5.51E-10 | SFPQ | 6421 |
| ENSG00000146463 | 1400.322705 | -0.19568084 | 0.0853054 | 0.0061673 | 0.0443325 | ZMYM4 | 9202 |
| ENSG00000227409 | 10.5296069 | 0.023064772 | 0.1375761 | 0.3139884 | NA | ZMYM4-AS1 | 100861513 |
| ENSG00000142687 | 1188.922744 | 0.058184377 | 0.0793827 | 0.3718705 | 0.6339491 | KIAA0319L | 79932 |
| ENSG00000020129 | 1445.453302 | 0.165026741 | 0.0832259 | 0.0172726 | 0.0933278 | NCDN | 23154 |
| ENSG00000126067 | 2463.997974 | -0.018781592 | 0.0670683 | 0.7465295 | 0.8896359 | PSMB2 | 5690 |
| ENSG00000142686 | 1251.362588 | -0.100604766 | 0.0875953 | 0.1472634 | 0.3807401 | C1orf216 | 127703 |
| ENSG00000092853 | 2992.661552 | 0.000977614 | 0.0612429 | 0.9834223 | 0.9937702 | CLSPN | 63967 |
| ENSG00000232335 | 34.51733015 | -0.000179969 | 0.1306926 | 0.9922298 | NA | NA | NA |
| ENSG00000134698 | 316.3634688 | 0.08510437 | 0.1183163 | 0.2558021 | 0.5229304 | AGO4 | 192670 |
| ENSG00000092847 | 1297.809532 | -0.237196545 | 0.094293 | 0.0024157 | 0.0218407 | AGO1 | 26523 |
| ENSG00000126070 | 878.7098502 | 0.06793211 | 0.0971164 | 0.3544977 | 0.6187516 | AGO3 | 192669 |

| | | | | | | | | |
|---|---|---|---|---|---|---|---|---|
| ENSG00000116863 | 635.3498013 | 0.102684213 | 0.1004147 | 0.1664527 | 0.4114993 | ADPRS | | 54936 |
| ENSG00000054116 | 1380.196478 | -0.101123183 | 0.0785104 | 0.1212968 | 0.3410469 | TRAPPC3 | | 27095 |
| ENSG00000116871 | 4430.389775 | 0.034448656 | 0.0576358 | 0.5099564 | 0.7415412 | MAP7D1 | | 55700 |
| ENSG00000054118 | 5294.596954 | -0.254325003 | 0.061531 | 7.45E-06 | 0.0001951 | THRAP3 | | 9967 |
| ENSG00000214193 | 460.3604493 | -0.112948462 | 0.1139215 | 0.1458654 | 0.378382 | SH3D21 | | 79729 |
| ENSG00000142694 | 280.7671289 | -0.083484209 | 0.1215038 | 0.2592273 | 0.525883 | EVA1B | | 55194 |
| ENSG00000116883 | 55.8172423 | -0.028746866 | 0.1320319 | 0.5216366 | 0.7493455 | NA | NA | |
| ENSG00000196182 | 866.5176797 | -0.114296057 | 0.0953495 | 0.1175016 | 0.3339561 | STK40 | | 83931 |
| ENSG00000181817 | 565.1524814 | -0.030673344 | 0.0923163 | 0.6538878 | 0.8399571 | LSM10 | | 84967 |
| ENSG00000116885 | 24.87570054 | 0.074806255 | 0.1624824 | 0.0453314 | NA | OSCP1 | | 127700 |
| ENSG00000116898 | 1664.503509 | -0.075981012 | 0.0721804 | 0.2193403 | 0.4793377 | MRPS15 | | 64960 |
| ENSG00000233621 | 17.01564997 | -0.016048839 | 0.1349555 | 0.5677683 | NA | LITATS1 | | 728431 |
| ENSG00000163874 | 729.8332062 | -0.193931504 | 0.1159747 | 0.0228987 | 0.1141309 | ZC3H12A | | 80149 |
| ENSG00000163875 | 969.6558677 | -0.139681601 | 0.0938311 | 0.0573012 | 0.2117479 | MEAF6 | | 64769 |
| ENSG00000163877 | 499.6024204 | -0.020468623 | 0.0931754 | 0.7636006 | 0.8982943 | SNIP1 | | 79753 |
| ENSG00000134697 | 2601.652119 | 0.293143135 | 0.0706284 | 5.47E-06 | 0.0001497 | GNL2 | | 29889 |
| ENSG00000116922 | 988.3358092 | 0.416632545 | 0.1011676 | 3.63E-06 | 0.0001051 | AIRIM | | 54955 |
| ENSG00000134690 | 2118.201828 | -0.17404995 | 0.075788 | 0.0075713 | 0.0517801 | CDCA8 | | 55143 |
| ENSG00000183317 | 54.39646444 | 0.039705424 | 0.134734 | 0.4032677 | 0.6603911 | EPHA10 | | 284656 |
| ENSG00000185090 | 630.7542325 | 0.018854047 | 0.0883558 | 0.7829851 | 0.9096561 | MANEAL | | 149175 |
| ENSG00000196449 | 910.7479667 | 0.48315686 | 0.1022939 | 1.93E-07 | 8.24E-06 | YRDC | | 79693 |
| ENSG00000197982 | 703.1513003 | 0.143968189 | 0.1027518 | 0.0625239 | 0.2236197 | C1orf122 | | 127687 |
| ENSG00000188786 | 769.6811027 | -0.007855772 | 0.0834923 | 0.9045595 | 0.962222 | MTF1 | | 4520 |
| ENSG00000204084 | 406.6306746 | -0.088304901 | 0.1111035 | 0.2378996 | 0.5025095 | INPP5B | | 3633 |
| ENSG00000183431 | 3449.40679 | -0.184852409 | 0.0664405 | 0.0018028 | 0.0175338 | SF3A3 | | 10946 |
| ENSG00000183386 | 113.5269928 | 0.044266489 | 0.1281598 | 0.4521778 | 0.6983854 | FHL3 | | 2275 |
| ENSG00000183520 | 1195.717595 | 0.044837454 | 0.0804683 | 0.4933974 | 0.7288952 | UTP11 | | 51118 |
| ENSG00000116954 | 777.6307505 | 0.444549904 | 0.1126754 | 6.85E-06 | 0.000181 | RRAGC | | 64121 |
| ENSG00000228436 | 15.96095976 | 0.051463864 | 0.1480988 | 0.0740928 | NA | RRAGC-DT | | 105378663 |
| ENSG00000214114 | 439.4528416 | 0.003193546 | 0.0968198 | 0.963866 | 0.9854626 | MYCBP | | 26292 |
| ENSG00000174574 | 1799.26406 | -0.060577095 | 0.0713921 | 0.3199558 | 0.5887529 | AKIRIN1 | | 79647 |
| ENSG00000168653 | 2516.84605 | -0.310704858 | 0.0751451 | 5.25E-06 | 0.0001451 | NDUFS5 | | 4725 |
| ENSG00000127603 | 24461.50458 | -0.226317219 | 0.0578977 | 2.13E-05 | 0.0004789 | MACF1 | | 23499 |
| ENSG00000226438 | 9.028491533 | -0.009027706 | 0.1354556 | 0.6633064 | NA | NA | NA | |
| ENSG00000255103 | 4897.757985 | -0.317277491 | 0.0678892 | 4.39E-07 | 1.67E-05 | NA | NA | |
| ENSG00000182109 | 13.06536758 | -0.003011401 | 0.1343045 | 0.9020265 | NA | NA | NA | |
| ENSG00000090621 | 3851.357036 | -0.090430189 | 0.0633834 | 0.1069283 | 0.3155708 | PABPC4 | | 8761 |
| ENSG00000228060 | 21.80793026 | -0.020411391 | 0.1349002 | 0.5186063 | NA | NA | NA | |
| ENSG00000201457 | 16.90792831 | -0.026342106 | 0.1376671 | 0.3216697 | NA | SNORA55 | | 677834 |
| ENSG00000084072 | 424.7502647 | 0.069230778 | 0.1047855 | 0.3396325 | 0.6068111 | PPIE | | 10450 |
| ENSG00000116985 | 29.36305693 | 0.006540956 | 0.1321497 | 0.851389 | NA | BMP8B | | 656 |
| ENSG00000198754 | 15.1314012 | 0.003620027 | 0.1340308 | 0.8933904 | NA | OXCT2 | | 64064 |
| ENSG00000043514 | 672.9273419 | 0.084840139 | 0.0950934 | 0.2368261 | 0.5010625 | TRIT1 | | 54802 |
| ENSG00000116990 | 10.03411065 | -0.006223769 | 0.1349891 | 0.7775835 | NA | MYCL | | 4610 |
| ENSG00000168389 | 13.66465255 | 0.019948081 | 0.1363769 | 0.4310967 | NA | MFSD2A | | 84879 |
| ENSG00000131236 | 11124.32921 | -0.191159876 | 0.0560221 | 0.0002146 | 0.0032422 | CAP1 | | 10487 |
| ENSG00000131238 | 2600.902867 | -0.197123866 | 0.0728067 | 0.0020188 | 0.0189719 | PPT1 | | 5538 |
| ENSG00000117000 | 742.8474937 | 0.054313835 | 0.0904638 | 0.4179899 | 0.6710348 | RLF | | 6018 |
| ENSG00000259943 | 54.81338483 | 0.011538104 | 0.1284907 | 0.8005934 | 0.9169162 | NA | NA | |
| ENSG00000084073 | 2225.615538 | -0.143748259 | 0.0704649 | 0.0189302 | 0.0997848 | ZMPSTE24 | | 10269 |
| ENSG00000084070 | 632.2532319 | 0.028708528 | 0.0914891 | 0.6772406 | 0.8528296 | SMAP2 | | 64744 |
| ENSG00000187801 | 237.8738663 | 0.064331461 | 0.1233646 | 0.3544667 | 0.6187516 | ZFP69B | | 65243 |
| ENSG00000260920 | 176.3251883 | 0.110129746 | 0.1487966 | 0.1496318 | 0.3849041 | NA | NA | |
| ENSG00000187815 | 142.6414879 | 0.022957186 | 0.1201966 | 0.7026486 | 0.8650963 | ZFP69 | | 339559 |
| ENSG00000164002 | 516.9026075 | 0.046542314 | 0.0952666 | 0.5063675 | 0.7389903 | EXO5 | | 64789 |
| ENSG00000227278 | 11.01114524 | 0.001643105 | 0.1349297 | 0.9417881 | NA | NA | NA | |
| ENSG00000117010 | 160.287027 | 0.045514039 | 0.121903 | 0.4804957 | 0.719573 | ZNF684 | | 127396 |
| ENSG00000117016 | 208.0877542 | -0.178478758 | 0.1882689 | 0.054602 | 0.2046873 | RIMS3 | | 9783 |
| ENSG00000272145 | 45.68380509 | 0.027363582 | 0.1324656 | 0.5294817 | 0.7554508 | NA | NA | |
| ENSG00000066136 | 1084.087897 | 0.019884877 | 0.0778723 | 0.7569201 | 0.8955316 | NFYC | | 4802 |
| ENSG00000117013 | 14.72653515 | 0.01184425 | 0.1346974 | 0.6576244 | NA | KCNQ4 | | 9132 |
| ENSG00000179862 | 3414.334277 | 0.152041768 | 0.0655572 | 0.0088813 | 0.0586555 | CITED4 | | 163732 |
| ENSG00000171793 | 5923.562038 | 0.003441046 | 0.0583096 | 0.9506954 | 0.9802173 | CTPS1 | | 1503 |
| ENSG00000171790 | 16.35101663 | 0.023407267 | 0.1366437 | 0.3997017 | NA | SLFNL1 | | 200172 |
| ENSG00000010803 | 471.3212032 | 0.08567219 | 0.1048282 | 0.2432646 | 0.5091513 | SCMH1 | | 22955 |
| ENSG00000127124 | 345.5877214 | 0.03048294 | 0.1036133 | 0.6586927 | 0.8425525 | HIVEP3 | | 59269 |
| ENSG00000198815 | 1667.252197 | -0.077627326 | 0.072466 | 0.2164934 | 0.4751709 | FOXJ3 | | 22887 |
| ENSG00000177181 | 112.6216168 | -0.044202524 | 0.1276311 | 0.455107 | 0.7001323 | RIMKLA | | 284716 |
| ENSG00000127125 | 415.1474937 | 0.189985104 | 0.1363677 | 0.0361874 | 0.1566735 | PPCS | | 79717 |
| ENSG00000186409 | 8.479085658 | 0.010097913 | 0.1357263 | 0.6148275 | NA | CCDC30 | | 728621 |

| | | | | | | | |
|---|---|---|---|---|---|---|---|
| ENSG00000171960 | 613.9676485 | -0.146350872 | 0.1096346 | 0.0659126 | 0.2320448 | PPIH | 10465 |
| ENSG00000065978 | 25444.1529 | -0.09582269 | 0.0500566 | 0.0401096 | 0.1678682 | YBX1 | 4904 |
| ENSG00000117385 | 909.5371469 | -0.069028199 | 0.0847456 | 0.3061209 | 0.5752194 | P3H1 | 64175 |
| ENSG00000164008 | 165.5269464 | 0.076665773 | 0.1340313 | 0.2651891 | 0.5327957 | C1orf50 | 79078 |
| ENSG00000177868 | 187.6540842 | -0.416503624 | 0.229278 | 0.0040192 | 0.0320033 | SVBP | 374969 |
| ENSG00000164010 | 161.8595679 | -0.050622926 | 0.1233393 | 0.4357107 | 0.6861988 | ERMAP | 114625 |
| ENSG00000228192 | 9.674573936 | 0.00740779 | 0.1351304 | 0.7381679 | NA | NA | NA |
| ENSG00000164011 | 103.5048291 | -0.000578616 | 0.1218972 | 0.99031 | 0.9957658 | ZNF691 | 51058 |
| ENSG00000117394 | 1068.084747 | -0.070784955 | 0.0820498 | 0.2872997 | 0.5571603 | SLC2A1 | 6513 |
| ENSG00000117395 | 5088.123655 | -0.026182232 | 0.058352 | 0.6216562 | 0.818389 | EBNA1BP2 | 10969 |
| ENSG00000243710 | 31.83233918 | 0.004601526 | 0.131155 | 0.9034347 | NA | CFAP57 | 149465 |
| ENSG00000234694 | 11.37526019 | 0.000303639 | 0.1345891 | 0.991972 | NA | CDC20-DT | 105378687 |
| ENSG00000117399 | 4267.55724 | -0.315743797 | 0.0694015 | 7.92E-07 | 2.80E-05 | CDC20 | 991 |
| ENSG00000066322 | 2051.842601 | 0.021079181 | 0.0665942 | 0.7175963 | 0.8722533 | ELOVL1 | 64834 |
| ENSG00000159479 | 832.4771763 | 0.088022421 | 0.088995 | 0.2075379 | 0.4635278 | MED8 | 112950 |
| ENSG00000198198 | 1162.407413 | 0.042266696 | 0.079356 | 0.5073066 | 0.7395602 | SZT2 | 23334 |
| ENSG00000178922 | 318.2308002 | -0.178210185 | 0.1521647 | 0.0515676 | 0.1961451 | HYI | 81888 |
| ENSG00000229348 | 22.59263823 | -0.042325512 | 0.1420153 | 0.196854 | NA | HYI-AS1 | 100873923 |
| ENSG00000142949 | 7271.905418 | -0.045356393 | 0.0602445 | 0.4004789 | 0.6584088 | PTPRF | 5792 |
| ENSG00000066135 | 1502.488562 | -0.10958078 | 0.0804478 | 0.0985814 | 0.2996336 | KDM4A | 9682 |
| ENSG00000236200 | 36.40432515 | -0.003265859 | 0.1303717 | 0.9317792 | NA | NA | NA |
| ENSG00000126091 | 153.3509984 | 0.011806605 | 0.1166371 | 0.8487539 | 0.9376992 | ST3GAL3 | 6487 |
| ENSG00000237950 | 19.30752826 | 0.012516587 | 0.1340767 | 0.6744946 | NA | NA | NA |
| ENSG00000117408 | 957.0147042 | 0.407241468 | 0.1109723 | 2.29E-05 | 0.0005068 | IPO13 | 9670 |
| ENSG00000132768 | 1444.399892 | 0.015522347 | 0.0712214 | 0.8008989 | 0.9169162 | DPH2 | 1802 |
| ENSG00000117410 | 2110.885506 | 0.088012495 | 0.08255 | 0.1895503 | 0.4403614 | ATP6V0B | 533 |
| ENSG00000117411 | 846.0723625 | 0.068811276 | 0.0867279 | 0.317099 | 0.5856179 | B4GALT2 | 8704 |
| ENSG00000159214 | 119.0240506 | 0.022997866 | 0.1225235 | 0.6902818 | 0.8589854 | CCDC24 | 149473 |
| ENSG00000196517 | 302.0139403 | 0.27280497 | 0.2008586 | 0.0168226 | 0.0913129 | SLC6A9 | 6536 |
| ENSG00000230615 | 69.31868678 | 0.052957336 | 0.1403686 | 0.2784205 | 0.5467586 | LOC1079849 | 107984948 |
| ENSG00000171872 | 19.52351327 | 0.032040416 | 0.1386997 | 0.2845367 | NA | KLF17 | 128209 |
| ENSG00000178028 | 981.6522664 | -0.139074569 | 0.0921505 | 0.0558863 | 0.2080666 | DMAP1 | 55929 |
| ENSG00000117419 | 805.0025458 | -0.028924378 | 0.0858531 | 0.6652738 | 0.8451364 | ERI3 | 79033 |
| ENSG00000233602 | 9.203101234 | 0.003093133 | 0.1349928 | 0.8869804 | NA | ERI3-IT1 | 100874278 |
| ENSG00000187147 | 1731.299668 | -0.037212903 | 0.0703766 | 0.5365432 | 0.7607868 | RNF220 | 55182 |
| ENSG00000126106 | 97.0525139 | -0.047275658 | 0.1309248 | 0.4085063 | 0.6640358 | TMEM53 | 79639 |
| ENSG00000142945 | 4335.86391 | 0.001157078 | 0.0564317 | 0.9825326 | 0.9934198 | KIF2C | 11004 |
| ENSG00000225721 | 24.66013994 | -0.049884934 | 0.1451132 | 0.1529382 | NA | NA | NA |
| ENSG00000142937 | 13586.5012 | -0.16304277 | 0.0546468 | 0.0011934 | 0.0128716 | RPS8 | 6202 |
| ENSG00000200913 | 12.77613862 | 0.005948016 | 0.1345139 | 0.8119467 | NA | SNORD46 | 94161 |
| ENSG00000142959 | 12.91625814 | 0.0166711 | 0.1358457 | 0.5005513 | NA | BEST4 | 266675 |
| ENSG00000173846 | 346.5008286 | 0.383712203 | 0.1593364 | 0.0013307 | 0.0140536 | PLK3 | 1263 |
| ENSG00000222009 | 57.10097967 | 0.181634162 | 0.3186834 | 0.0203835 | 0.1051576 | BTBD19 | 149478 |
| ENSG00000117425 | 24.26397624 | 0.082517117 | 0.1707592 | 0.0228584 | NA | PTCH2 | 8643 |
| ENSG00000070785 | 831.9213393 | -0.04040497 | 0.085712 | 0.5473762 | 0.7690111 | EIF2B3 | 8891 |
| ENSG00000126107 | 1420.177483 | -0.127287852 | 0.0792828 | 0.0543999 | 0.2041306 | HECTD3 | 79654 |
| ENSG00000126088 | 1140.813398 | 0.054534447 | 0.0849562 | 0.418097 | 0.6710348 | UROD | 7389 |
| ENSG00000162415 | 28.39081517 | 0.004669922 | 0.1316783 | 0.8975854 | NA | ZSWIM5 | 57643 |
| ENSG00000132781 | 279.1822216 | 0.05269409 | 0.1161004 | 0.4464378 | 0.6942938 | MUTYH | 4595 |
| ENSG00000132773 | 471.9651108 | 0.138391322 | 0.123716 | 0.0924337 | 0.287535 | TOE1 | 114034 |
| ENSG00000070759 | 74.47747174 | -0.031546414 | 0.1295527 | 0.5354008 | 0.7598124 | TESK2 | 10420 |
| ENSG00000236624 | 83.138553 | 0.038356514 | 0.1325706 | 0.4462903 | 0.6941567 | NA | NA |
| ENSG00000132763 | 162.7534348 | 0.006776281 | 0.1146562 | 0.91515 | 0.9662935 | MMACHC | 25974 |
| ENSG00000117450 | 19161.1578 | -0.146973128 | 0.0574962 | 0.0049425 | 0.0376802 | PRDX1 | 5052 |
| ENSG00000117448 | 948.6220762 | -0.021255612 | 0.0805486 | 0.7433906 | 0.8884332 | AKR1A1 | 10327 |
| ENSG00000132780 | 4594.092092 | -0.280535585 | 0.0640357 | 2.11E-06 | 6.49E-05 | NASP | 4678 |
| ENSG00000159588 | 41.66340491 | 0.005120438 | 0.1295354 | 0.9039349 | 0.9620832 | CCDC17 | 149483 |
| ENSG00000159592 | 1630.74149 | -0.134312231 | 0.07926 | 0.0426205 | 0.1745735 | GPBP1L1 | 60313 |
| ENSG00000234329 | 16.36408017 | 0.003246022 | 0.1341039 | 0.9030392 | NA | RPL7AP16 | 100270896 |
| ENSG00000159596 | 553.8134606 | 0.004777184 | 0.0906697 | 0.9447249 | 0.9784014 | TMEM69 | 51249 |
| ENSG00000197429 | 442.9197193 | 0.075508313 | 0.107506 | 0.2952639 | 0.5653135 | IPP | 3652 |
| ENSG00000230896 | 29.42895177 | 0.019642689 | 0.1334034 | 0.5879818 | NA | NA | NA |
| ENSG00000086015 | 1713.180883 | 0.12119451 | 0.0762098 | 0.0596646 | 0.2171581 | MAST2 | 23139 |
| ENSG00000117461 | 379.0756481 | 0.030784262 | 0.100324 | 0.6571168 | 0.8419409 | PIK3R3 | 8503 |
| ENSG00000117472 | 63.31560828 | -0.000522872 | 0.1265298 | 0.9898442 | 0.9955163 | TSPAN1 | 10103 |
| ENSG00000085998 | 2073.001953 | -0.044686985 | 0.0668678 | 0.4428399 | 0.6917073 | POMGNT1 | 55624 |
| ENSG00000171357 | 83.15627376 | 0.008279826 | 0.1237403 | 0.8774675 | 0.9503661 | LURAP1 | 541468 |
| ENSG00000085999 | 866.3609694 | -0.024807553 | 0.0829211 | 0.7064691 | 0.867199 | RAD54L | 8438 |
| ENSG00000132128 | 1036.328592 | -0.076854002 | 0.0832233 | 0.2503995 | 0.5164049 | LRRC41 | 10489 |
| ENSG00000173660 | 3907.356398 | -0.016514911 | 0.0581656 | 0.7530383 | 0.8932431 | UQCRH | 7388 |

| | | | | | | | | |
|---|---|---|---|---|---|---|---|---|
| ENSG00000117481 | 481.7785754 | 0.115314628 | 0.1113147 | 0.1395721 | 0.3687721 | NSUN4 | | 387338 |
| ENSG00000117480 | 13.47920369 | 0.004075675 | 0.1344987 | 0.869399 | NA | FAAH | | 2166 |
| ENSG00000232022 | 17.30356015 | 0.000957568 | 0.1335373 | 0.9749475 | NA | NA | NA | |
| ENSG00000079277 | 647.5615192 | -0.113242946 | 0.1007595 | 0.1296846 | 0.3541843 | MKNK1 | | 8569 |
| ENSG00000142961 | 190.0162282 | 0.011591972 | 0.112283 | 0.858578 | 0.9430101 | MOB3C | | 148932 |
| ENSG00000123472 | 394.7722516 | -0.079816796 | 0.1124325 | 0.2786216 | 0.5468042 | ATPAF1 | | 64756 |
| ENSG00000159658 | 2436.231511 | -0.087632661 | 0.0663897 | 0.1309164 | 0.3556358 | EFCAB14 | | 9813 |
| ENSG00000225506 | 16.22346466 | 0.01123729 | 0.134647 | 0.6723337 | NA | NA | NA | |
| ENSG00000162366 | 783.4258474 | 0.294356972 | 0.1327642 | 0.0033165 | 0.0276125 | PDZK1IP1 | | 10158 |
| ENSG00000123473 | 1910.315875 | 0.144045551 | 0.0776458 | 0.0281708 | 0.1322603 | STIL | | 6491 |
| ENSG00000162368 | 2834.469021 | -0.107940395 | 0.0659027 | 0.062085 | 0.2224145 | CMPK1 | | 51727 |
| ENSG00000237424 | 106.349728 | 0.026889827 | 0.1233114 | 0.6409208 | 0.8315061 | FOXD2-AS1 | | 84793 |
| ENSG00000186564 | 22.76717018 | 0.031101485 | 0.1375969 | 0.3410318 | NA | FOXD2 | | 2306 |
| ENSG00000132122 | 122.021042 | -0.239094354 | 0.2810511 | 0.0267929 | 0.1271708 | SPATA6 | | 54558 |
| ENSG00000185104 | 1277.505111 | -0.051101283 | 0.0773498 | 0.4244599 | 0.6765989 | FAF1 | | 11124 |
| ENSG00000123080 | 583.7455652 | -0.067421611 | 0.0979473 | 0.3433294 | 0.609766 | CDKN2C | | 1031 |
| ENSG00000123091 | 1675.675653 | -0.069127331 | 0.0738302 | 0.2667108 | 0.5346062 | RNF11 | | 26994 |
| ENSG00000085831 | 8.652818228 | -0.004922276 | 0.1354583 | 0.791802 | NA | TTC39A | | 22996 |
| ENSG00000085832 | 956.4979895 | -0.079198769 | 0.0878031 | 0.2466932 | 0.5127444 | EPS15 | | 2060 |
| ENSG00000117859 | 1363.2919 | -0.050523377 | 0.075337 | 0.4228528 | 0.6748503 | OSBPL9 | | 114883 |
| ENSG00000078618 | 4121.319305 | -0.113122727 | 0.0600106 | 0.0351112 | 0.1531493 | NRDC | | 4898 |
| ENSG00000266993 | 32.23498085 | 0.020090033 | 0.133012 | 0.5950867 | NA | NA | NA | |
| ENSG00000169213 | 1944.985029 | 0.022416792 | 0.0669613 | 0.701661 | 0.8649327 | RAB3B | | 5865 |
| ENSG00000117862 | 1691.155908 | -0.13618955 | 0.0767768 | 0.0357799 | 0.1553699 | TXNDC12 | | 51060 |
| ENSG00000198841 | 283.9243068 | 0.166817951 | 0.1561835 | 0.0637512 | 0.226985 | KTI12 | | 112970 |
| ENSG00000134717 | 1475.917761 | -0.082813981 | 0.0758487 | 0.1923073 | 0.4436149 | BTF3L4 | | 91408 |
| ENSG00000157077 | 537.192316 | -0.05298355 | 0.098035 | 0.4516041 | 0.6980532 | ZFYVE9 | | 9372 |
| ENSG00000154222 | 1640.894666 | -0.022444403 | 0.069043 | 0.7056454 | 0.8670449 | CC2D1B | | 200014 |
| ENSG00000272100 | 10.86782921 | 0.00724362 | 0.1351076 | 0.7453703 | NA | NA | NA | |
| ENSG00000085840 | 919.2806221 | 0.187392741 | 0.1057893 | 0.0207967 | 0.1065373 | ORC1 | | 4998 |
| ENSG00000134748 | 1075.70799 | -0.112392439 | 0.0858411 | 0.1029013 | 0.3074144 | PRPF38A | | 84950 |
| ENSG00000134744 | 1339.50522 | -0.224677754 | 0.0897865 | 0.0027711 | 0.024247 | TUT4 | | 23318 |
| ENSG00000162377 | 1058.779915 | 0.19452842 | 0.0965045 | 0.0118616 | 0.0717534 | COA7 | | 65260 |
| ENSG00000162378 | 859.5419056 | -0.07782597 | 0.0875483 | 0.2575605 | 0.5243228 | ZYG11B | | 79699 |
| ENSG00000121310 | 521.1919417 | -0.312953914 | 0.1311 | 0.0019612 | 0.0186405 | ECHDC2 | | 55268 |
| ENSG00000116171 | 894.1886435 | -0.004923786 | 0.0903007 | 0.9151773 | 0.9662935 | SCP2 | | 6342 |
| ENSG00000157184 | 137.0743359 | 0.170591581 | 0.2096066 | 0.0555361 | 0.2069932 | CPT2 | | 1376 |
| ENSG00000236723 | 8.874935937 | -0.028662188 | 0.1395551 | 0.1658247 | NA | NA | NA | |
| ENSG00000162384 | 405.6898613 | -0.017847652 | 0.1047052 | 0.7906976 | 0.9122112 | CZIB | | 54987 |
| ENSG00000162385 | 1021.097306 | -0.107951905 | 0.0869961 | 0.1197551 | 0.3378071 | MAGOH | | 4116 |
| ENSG00000270172 | 72.85214762 | 0.002639602 | 0.1251415 | 0.9590349 | 0.9831259 | NA | NA | |
| ENSG00000157193 | 486.3635794 | -0.067944751 | 0.1001442 | 0.3430917 | 0.6097025 | LRP8 | | 7804 |
| ENSG00000058804 | 2024.360282 | -0.099148648 | 0.0703013 | 0.101513 | 0.3050059 | NDC1 | | 55706 |
| ENSG00000058799 | 354.5531642 | -0.066493544 | 0.1090481 | 0.3564664 | 0.6197178 | YIPF1 | | 54432 |
| ENSG00000081870 | 701.6117227 | -0.063808666 | 0.0909152 | 0.3579404 | 0.621171 | IFT25 | | 51668 |
| ENSG00000116212 | 828.774818 | -0.152923626 | 0.1043225 | 0.0505963 | 0.1937121 | LRRC42 | | 115353 |
| ENSG00000272091 | 14.66990776 | 0.016050162 | 0.135409 | 0.5413469 | NA | NA | NA | |
| ENSG00000116209 | 1708.991491 | -0.026951746 | 0.0702546 | 0.6536688 | 0.8399571 | TMEM59 | | 9528 |
| ENSG00000116205 | 236.3488679 | 0.334546712 | 0.197903 | 0.007225 | 0.0499383 | TCEANC2 | | 127428 |
| ENSG00000212670 | 11.79047803 | 0.036221753 | 0.141764 | 0.1254266 | NA | NA | NA | |
| ENSG00000248835 | 142.7110764 | 0.025841595 | 0.118936 | 0.6759353 | 0.8514613 | NA | NA | |
| ENSG00000215883 | 167.4561309 | -0.015617532 | 0.1156716 | 0.801525 | 0.9170941 | CYB5RL | | 606495 |
| ENSG00000116221 | 2356.44233 | 0.090348329 | 0.0690065 | 0.130723 | 0.3554411 | MRPL37 | | 51253 |
| ENSG00000157216 | 322.0838867 | -0.043961121 | 0.1067035 | 0.5281296 | 0.754779 | SSBP3 | | 23648 |
| ENSG00000162390 | 63.26731071 | 0.085450761 | 0.1625731 | 0.1062774 | 0.3143647 | ACOT11 | | 26027 |
| ENSG00000243725 | 28.57973807 | 0.053839976 | 0.1464837 | 0.15294 | NA | TTC4 | | 7268 |
| ENSG00000162396 | 59.6191098 | 0.031276916 | 0.1317684 | 0.5080965 | 0.7401841 | PARS2 | | 25973 |
| ENSG00000116133 | 12126.54455 | -0.393364761 | 0.0560029 | 3.16E-13 | 4.35E-11 | DHCR24 | | 1718 |
| ENSG00000242396 | 41.05240365 | -0.049273513 | 0.1414135 | 0.2524943 | 0.5191652 | NA | NA | |
| ENSG00000162402 | 4183.43713 | -0.217979577 | 0.0683955 | 0.0003761 | 0.0051862 | USP24 | | 23358 |
| ENSG00000162407 | 221.0195613 | 0.28772392 | 0.2179806 | 0.0156153 | 0.0867341 | PLPP3 | | 8613 |
| ENSG00000162409 | 337.7425525 | 0.23285062 | 0.1662075 | 0.0227806 | 0.1137287 | PRKAA2 | | 5563 |
| ENSG00000162600 | 187.3988261 | -0.131342068 | 0.1589854 | 0.1061051 | 0.3139345 | OMA1 | | 115209 |
| ENSG00000185839 | 10.05822918 | -0.002316207 | 0.1351499 | 0.906577 | NA | AK2P1 | | 266920 |
| ENSG00000162601 | 2255.557876 | 0.055105522 | 0.0651934 | 0.3363555 | 0.6039921 | MYSM1 | | 114803 |
| ENSG00000177606 | 2733.496679 | 0.673819707 | 0.070452 | 7.95E-23 | 3.09E-20 | JUN | | 3725 |
| ENSG00000172456 | 50.99201889 | 0.022666404 | 0.1310328 | 0.6067796 | 0.8114231 | FGGY | | 55277 |
| ENSG00000134709 | 609.1460274 | -0.085157528 | 0.0974132 | 0.2385802 | 0.5031419 | HOOK1 | | 51361 |
| ENSG00000134716 | 22.68479085 | 0.014122794 | 0.134114 | 0.6428341 | NA | CYP2J2 | | 1573 |
| ENSG00000226476 | 12.6220698 | -0.035567731 | 0.1411618 | 0.1558111 | NA | LINC01748 | | 105378763 |

| | | | | | | | | |
|---|---|---|---|---|---|---|---|---|
| ENSG00000162604 | 320.9676739 | 0.003255088 | 0.1036353 | 0.9630958 | 0.9851357 | TM2D1 | | 83941 |
| ENSG00000132849 | 729.324788 | -0.25252997 | 0.1160776 | 0.0049266 | 0.0376622 | PATJ | | 10207 |
| ENSG00000162607 | 3260.814611 | -0.07945814 | 0.0640893 | 0.1597344 | 0.4007257 | USP1 | | 7398 |
| ENSG00000116641 | 1432.717151 | -0.081840674 | 0.075891 | 0.1986163 | 0.4518085 | DOCK7 | | 85440 |
| ENSG00000125703 | 209.2190906 | 0.004406086 | 0.1104696 | 0.9472533 | 0.9787586 | ATG4C | | 84938 |
| ENSG00000230798 | 13.09317859 | -0.03702042 | 0.1418147 | 0.1348238 | NA | FOXD3-AS1 | | 100996301 |
| ENSG00000088035 | 393.4852176 | -0.134980374 | 0.1288323 | 0.1015207 | 0.3050059 | ALG6 | | 29929 |
| ENSG00000142856 | 437.1956191 | -0.049328214 | 0.1017419 | 0.4832427 | 0.7217391 | ITGB3BP | | 23421 |
| ENSG00000203965 | 98.53472922 | -0.086623618 | 0.1511902 | 0.1749293 | 0.4232289 | EFCAB7 | | 84455 |
| ENSG00000079739 | 1023.059313 | 0.126040529 | 0.0883889 | 0.0747922 | 0.2510482 | PGM1 | | 5236 |
| ENSG00000185483 | 1246.276579 | -0.184813238 | 0.0916166 | 0.013017 | 0.0765231 | ROR1 | | 4919 |
| ENSG00000158966 | 432.0137789 | 0.018464684 | 0.096748 | 0.7883419 | 0.9116361 | CACHD1 | | 57685 |
| ENSG00000162437 | 676.0994736 | -0.08766767 | 0.0959387 | 0.223365 | 0.4836193 | RAVER2 | | 55225 |
| ENSG00000162434 | 6348.739537 | -0.359608039 | 0.0648991 | 3.76E-09 | 2.51E-07 | JAK1 | | 3716 |
| ENSG00000162433 | 1769.971059 | 0.008021823 | 0.0690637 | 0.8939197 | 0.9572676 | AK4 | | 205 |
| ENSG00000116675 | 610.4159508 | 0.424128065 | 0.1219288 | 4.39E-05 | 0.000883 | DNAJC6 | | 9829 |
| ENSG00000116678 | 235.9293436 | -0.286829358 | 0.1961178 | 0.013321 | 0.0776457 | LEPR | | 3953 |
| ENSG00000213625 | 1817.578127 | -0.050226364 | 0.0739306 | 0.4199658 | 0.672898 | LEPROT | | 54741 |
| ENSG00000152763 | 36.25848254 | 0.043587061 | 0.1404995 | 0.2615028 | NA | DNAI4 | | 79819 |
| ENSG00000198160 | 1447.039998 | -0.199108361 | 0.0870288 | 0.0060482 | 0.0436377 | MIER1 | | 57708 |
| ENSG00000116704 | 404.1825625 | -0.056284635 | 0.1029171 | 0.4284224 | 0.6796361 | SLC35D1 | | 23169 |
| ENSG00000142864 | 10947.03323 | -0.224421705 | 0.0582853 | 2.65E-05 | 0.0005745 | SERBP1 | | 26135 |
| ENSG00000116717 | 3028.06696 | 1.747647699 | 0.081549 | 3.67E-103 | 4.28E-99 | GADD45A | | 1647 |
| ENSG00000172380 | 3524.953313 | -0.231911908 | 0.0646347 | 7.91E-05 | 0.0014391 | GNG12 | | 55970 |
| ENSG00000116729 | 461.9479224 | 0.091525272 | 0.1075427 | 0.2217485 | 0.4811897 | WLS | | 79971 |
| ENSG00000024526 | 1725.835905 | -0.228905628 | 0.0973149 | 0.0039462 | 0.0315079 | DEPDC1 | | 55635 |
| ENSG00000066557 | 683.4055414 | -0.096502716 | 0.0964193 | 0.1832766 | 0.4329849 | LRRC40 | | 55631 |
| ENSG00000116754 | 4554.435117 | -0.160424025 | 0.062888 | 0.0043973 | 0.034312 | SRSF11 | | 9295 |
| ENSG00000118454 | 755.6535923 | -0.031162845 | 0.0855664 | 0.6411994 | 0.8315061 | ANKRD13C | | 81573 |
| ENSG00000197568 | 108.182648 | 0.201539414 | 0.2701331 | 0.0341995 | 0.1504084 | ANKRD13C- | | 11147 |
| ENSG00000116761 | 71.57947383 | -0.001792719 | 0.1258172 | 0.9693748 | 0.9880997 | CTH | | 1491 |
| ENSG00000235079 | 14.04116112 | -0.03151531 | 0.1394012 | 0.22932 | NA | NA | NA | |
| ENSG00000132485 | 1950.873569 | -0.090333743 | 0.0720474 | 0.1409916 | 0.3707615 | ZRANB2 | | 9406 |
| ENSG00000207721 | 9.306889958 | -0.001708976 | 0.1349584 | 0.9339191 | NA | MIR186 | | 406962 |
| ENSG00000172260 | 36.44352178 | 0.042980534 | 0.1393477 | 0.2947075 | NA | NEGR1 | | 257194 |
| ENSG00000162620 | 12.40378142 | 0.00607874 | 0.1347303 | 0.8011969 | NA | LRRIQ3 | | 127255 |
| ENSG00000254685 | 172.0861324 | -0.126726179 | 0.1658116 | 0.106361 | 0.3145324 | FPGT | | 8790 |
| ENSG00000116791 | 1363.925719 | -0.356456985 | 0.0921791 | 1.28E-05 | 0.0003117 | CRYZ | | 1429 |
| ENSG00000162623 | 658.763945 | -0.101106857 | 0.0981106 | 0.1676263 | 0.4125292 | TYW3 | | 127253 |
| ENSG00000137968 | 51.03539156 | -0.042864218 | 0.1369641 | 0.3481185 | 0.6140832 | SLC44A5 | | 204962 |
| ENSG00000117054 | 860.5746099 | 0.06339649 | 0.0855267 | 0.3528039 | 0.6185235 | ACADM | | 34 |
| ENSG00000137955 | 1397.338837 | -0.092937158 | 0.0876765 | 0.1795899 | 0.429016 | RABGGTB | | 5876 |
| ENSG00000226415 | 10.31520098 | -0.016277953 | 0.1362484 | 0.460271 | NA | NA | NA | |
| ENSG00000142892 | 898.6912693 | -0.213645764 | 0.1067246 | 0.0100818 | 0.0639333 | PIGK | | 10026 |
| ENSG00000226084 | 83.73505373 | -0.071108226 | 0.1442573 | 0.2256534 | 0.4867697 | RPL17P6 | | 645441 |
| ENSG00000154027 | 18.53954785 | 0.003355656 | 0.1335097 | 0.9093674 | NA | AK5 | | 26289 |
| ENSG00000036549 | 1081.557812 | -0.023977994 | 0.0776467 | 0.707024 | 0.8675568 | ZZZ3 | | 26009 |
| ENSG00000077254 | 1317.812148 | -0.120488411 | 0.0808398 | 0.0712583 | 0.2432314 | USP33 | | 23032 |
| ENSG00000180488 | 611.6213841 | 0.147008256 | 0.1095939 | 0.0651588 | 0.2303746 | MIGA1 | | 374986 |
| ENSG00000219201 | 16.66164636 | -0.025412688 | 0.1370038 | 0.3692818 | NA | NA | NA | |
| ENSG00000162614 | 141.3629058 | 0.074202332 | 0.1410624 | 0.2430007 | 0.5086902 | NEXN | | 91624 |
| ENSG00000162613 | 2520.668642 | -0.299817693 | 0.0774683 | 1.56E-05 | 0.0003697 | FUBP1 | | 8880 |
| ENSG00000162616 | 172.7352618 | 0.164658029 | 0.1919811 | 0.0629636 | 0.2247108 | DNAJB4 | | 11080 |
| ENSG00000117114 | 161.8500858 | -0.060251884 | 0.1263666 | 0.364096 | 0.626917 | ADGRL2 | | 23266 |
| ENSG00000137941 | 645.6546615 | 0.160522191 | 0.1085463 | 0.0457485 | 0.1827592 | TTLL7 | | 79739 |
| ENSG00000271576 | 24.78370585 | 0.043969303 | 0.1421834 | 0.2065563 | NA | NA | NA | |
| ENSG00000142875 | 384.4029433 | 0.031748541 | 0.0998547 | 0.6474468 | 0.8362299 | PRKACB | | 5567 |
| ENSG00000117133 | 796.9779892 | -0.024675271 | 0.0829532 | 0.7109906 | 0.869096 | RPF1 | | 80135 |
| ENSG00000174021 | 950.0152316 | -0.047186586 | 0.0831591 | 0.4791271 | 0.7181681 | GNG5 | | 2787 |
| ENSG00000117151 | 143.6975981 | 0.012980449 | 0.1171543 | 0.8328418 | 0.9305386 | CTBS | | 1486 |
| ENSG00000117155 | 1488.246245 | 0.065695569 | 0.0758094 | 0.3005392 | 0.57018 | SSX2IP | | 117178 |
| ENSG00000153898 | 160.4336134 | 0.027672115 | 0.1184002 | 0.6585215 | 0.8425525 | MCOLN2 | | 255231 |
| ENSG00000055732 | 444.8693274 | 0.058777147 | 0.1034788 | 0.3945661 | 0.6549057 | MCOLN3 | | 55283 |
| ENSG00000097096 | 286.2950336 | 0.026488961 | 0.1089213 | 0.6951514 | 0.8606321 | SYDE2 | | 84144 |
| ENSG00000162642 | 780.164345 | 0.373345921 | 0.1097663 | 6.94E-05 | 0.0012894 | C1orf52 | | 148423 |
| ENSG00000142867 | 708.9742003 | 0.248958833 | 0.1152141 | 0.0052709 | 0.0395691 | BCL10 | | 8915 |
| ENSG00000223653 | 8.845387904 | -0.009570166 | 0.1355175 | 0.6446844 | NA | NA | NA | |
| ENSG00000153904 | 1736.263118 | 0.069767432 | 0.0735475 | 0.2628691 | 0.5303256 | DDAH1 | | 23576 |
| ENSG00000142871 | 6442.349458 | 0.126023376 | 0.0555373 | 0.0128974 | 0.076088 | CCN1 | | 3491 |
| ENSG00000117174 | 1567.925461 | 0.32163888 | 0.0826287 | 1.37E-05 | 0.0003295 | ZNHIT6 | | 54680 |

| | | | | | | | |
|---|---|---|---|---|---|---|---|
| ENSG00000122417 | 726.7853383 | -0.003918047 | 0.0851796 | 0.9522217 | 0.9806656 | ODF2L | 57489 |
| ENSG00000097033 | 1485.205149 | -0.211070282 | 0.0834949 | 0.0029308 | 0.0253031 | SH3GLB1 | 51100 |
| ENSG00000183291 | 2706.849083 | -0.045112539 | 0.0620282 | 0.4134689 | 0.6676431 | SELENOF | 9403 |
| ENSG00000153936 | 1246.93191 | -0.021333762 | 0.0738762 | 0.7308806 | 0.8803112 | HS2ST1 | 9653 |
| ENSG00000143013 | 212.4243921 | 0.234498746 | 0.2020218 | 0.0276361 | 0.1301159 | LMO4 | 8543 |
| ENSG00000065243 | 2480.809199 | -0.173043094 | 0.0700156 | 0.0048558 | 0.0372427 | PKN2 | 5586 |
| ENSG00000241975 | 16.36322028 | 0.015500917 | 0.1351011 | 0.5693228 | NA | NA | NA |
| ENSG00000137947 | 372.8240274 | 0.15682913 | 0.1396319 | 0.0714005 | 0.2433699 | GTF2B | 2959 |
| ENSG00000137944 | 482.3016871 | -0.440819377 | 0.1388543 | 0.0001174 | 0.0019873 | KYAT3 | 56267 |
| ENSG00000213516 | 305.0890874 | -0.652568099 | 0.1731633 | 9.44E-06 | 0.0002398 | RBMXL1 | 494115 |
| ENSG00000117226 | 163.3703209 | 0.050506174 | 0.1265309 | 0.4225803 | 0.6745232 | GBP3 | 2635 |
| ENSG00000197147 | 694.8580871 | -0.071123761 | 0.0934692 | 0.3138823 | 0.5827177 | LRRC8B | 23507 |
| ENSG00000231999 | 19.81037238 | -0.026610901 | 0.136759 | 0.3834591 | NA | LRRC8C-DT | 400761 |
| ENSG00000171488 | 700.313503 | -0.083387959 | 0.0940483 | 0.2414389 | 0.5069118 | LRRC8C | 84230 |
| ENSG00000171492 | 462.6744301 | 0.025857888 | 0.095231 | 0.7071516 | 0.8675568 | LRRC8D | 55144 |
| ENSG00000228175 | 10.4905842 | 0.024751698 | 0.138081 | 0.2715109 | NA | NA | NA |
| ENSG00000162664 | 1384.857574 | -0.042144485 | 0.0734135 | 0.4963629 | 0.7316108 | ZNF326 | 284695 |
| ENSG00000233593 | 52.30854529 | 0.024519758 | 0.1313424 | 0.5799876 | 0.7922857 | LINC02609 | 105378853 |
| ENSG00000122482 | 1495.359141 | -0.010177351 | 0.072322 | 0.8674162 | 0.946589 | ZNF644 | 84146 |
| ENSG00000097046 | 733.239576 | 0.009047869 | 0.085218 | 0.8928549 | 0.9568118 | CDC7 | 8317 |
| ENSG00000069702 | 319.0877171 | -0.332585529 | 0.1678103 | 0.0043307 | 0.0339046 | TGFBR3 | 7049 |
| ENSG00000172031 | 35.41032733 | 0.0471215 | 0.1413504 | 0.2503631 | NA | EPHX4 | 253152 |
| ENSG00000189195 | 12.70669937 | 0.01847932 | 0.136135 | 0.4589876 | NA | BTBD8 | 284697 |
| ENSG00000069712 | 47.00409049 | 0.00079428 | 0.1291372 | 0.9895794 | 0.9955163 | NA | NA |
| ENSG00000174842 | 466.7009718 | -0.149537829 | 0.1214011 | 0.0711505 | 0.243052 | GLMN | 11146 |
| ENSG00000122484 | 614.6633619 | -0.083891668 | 0.0970995 | 0.244824 | 0.5104944 | RPAP2 | 79871 |
| ENSG00000162676 | 50.701624 | 0.150082474 | 0.2685211 | 0.0138169 | 0.0800173 | GFI1 | 2672 |
| ENSG00000067208 | 628.3450949 | -0.230709887 | 0.1229598 | 0.0111016 | 0.0685403 | EVI5 | 7813 |
| ENSG00000122406 | 15247.4823 | -0.227329924 | 0.054118 | 6.69E-06 | 0.0001771 | RPL5 | 6125 |
| ENSG00000251795 | 29.09402956 | 0.016533301 | 0.1328485 | 0.6479499 | NA | NA | NA |
| ENSG00000207523 | 13.12666818 | -0.016805891 | 0.1357672 | 0.5020633 | NA | SNORA66 | 26782 |
| ENSG00000154511 | 43.64101287 | -0.001850046 | 0.1291545 | 0.9635175 | 0.9851933 | DIPK1A | 388650 |
| ENSG00000143033 | 1000.089971 | -0.053726696 | 0.0807755 | 0.4121203 | 0.6662022 | MTF2 | 22823 |
| ENSG00000117500 | 2203.780233 | -0.036159347 | 0.0661696 | 0.5315293 | 0.7569513 | TMED5 | 50999 |
| ENSG00000122483 | 419.2699802 | 0.022317255 | 0.0969853 | 0.7458826 | 0.8893165 | CCDC18 | 343099 |
| ENSG00000223745 | 75.98848479 | 0.155931375 | 0.2453639 | 0.0356545 | 0.1548831 | NA | NA |
| ENSG00000117505 | 2059.898506 | 0.135238431 | 0.0740811 | 0.0328649 | 0.1463571 | DR1 | 1810 |
| ENSG00000137942 | 1347.621353 | 0.128862824 | 0.0915169 | 0.0748193 | 0.2510669 | FNBP1L | 54874 |
| ENSG00000137936 | 3642.505859 | 0.035092002 | 0.0606436 | 0.5142442 | 0.74444 | BCAR3 | 8412 |
| ENSG00000224093 | 9.710027607 | -0.005121965 | 0.1350464 | 0.8098911 | NA | NA | NA |
| ENSG00000230439 | 8.642603282 | 0.00485674 | 0.1352886 | 0.8103667 | NA | NA | NA |
| ENSG00000260464 | 14.4653775 | -0.005020704 | 0.1342663 | 0.8424733 | NA | NA | NA |
| ENSG00000067334 | 2311.144789 | 0.26803634 | 0.0743196 | 5.76E-05 | 0.0011163 | DNTTIP2 | 30836 |
| ENSG00000023909 | 2106.587258 | 0.068365859 | 0.0717778 | 0.264971 | 0.5327957 | GCLM | 2730 |
| ENSG00000137962 | 14147.06603 | 0.000386953 | 0.0488755 | 0.993881 | 0.9977151 | ARHGAP29 | 9411 |
| ENSG00000236098 | 36.79255001 | -0.020150731 | 0.1324829 | 0.6034434 | NA | NA | NA |
| ENSG00000226835 | 16.88716037 | 0.048249568 | 0.1461711 | 0.0972006 | NA | ARHGAP29- | 107985092 |
| ENSG00000117528 | 998.6109728 | 0.008858138 | 0.0789421 | 0.8915058 | 0.9562439 | ABCD3 | 5825 |
| ENSG00000117525 | 179.9851313 | 0.460283914 | 0.2307909 | 0.002552 | 0.0228126 | F3 | 2152 |
| ENSG00000117519 | 2338.210446 | -0.261766467 | 0.0772862 | 0.0001331 | 0.0021998 | CNN3 | 1266 |
| ENSG00000235501 | 40.41018506 | 0.031136662 | 0.1343547 | 0.4570617 | NA | NA | NA |
| ENSG00000172339 | 72.81441046 | -0.067230393 | 0.1457812 | 0.2117998 | 0.4684524 | ALG14 | 199857 |
| ENSG00000152078 | 331.2171761 | -0.049474382 | 0.1075978 | 0.4778017 | 0.7168253 | TLCD4 | 148534 |
| ENSG00000122481 | 76.90101629 | -0.033235554 | 0.1300142 | 0.515408 | 0.7451617 | RWDD3 | 25950 |
| ENSG00000137970 | 124.7738263 | -0.169902774 | 0.2254673 | 0.0492563 | 0.1907068 | RPL7P9 | 653702 |
| ENSG00000117569 | 387.4343237 | 0.05689777 | 0.1099569 | 0.4232877 | 0.6752831 | PTBP2 | 58155 |
| ENSG00000188641 | 286.1984385 | 0.015425897 | 0.1072723 | 0.8169196 | 0.9252377 | DPYD | 1806 |
| ENSG00000259946 | 9.276100885 | -0.019998172 | 0.1370921 | 0.3505256 | NA | NA | NA |
| ENSG00000225206 | 420.7661843 | -0.069153666 | 0.1056268 | 0.3396916 | 0.6068111 | MIR137HG | 400765 |
| ENSG00000226053 | 42.90900797 | 0.015115133 | 0.1308987 | 0.7129424 | 0.8702069 | LINC01776 | 729987 |
| ENSG00000162627 | 1081.494156 | -0.10305337 | 0.0841359 | 0.1291383 | 0.3536017 | SNX7 | 51375 |
| ENSG00000156869 | 58.62703036 | 0.034532484 | 0.1329941 | 0.463075 | 0.7074521 | FRRS1 | 391059 |
| ENSG00000162688 | 1056.182911 | -0.13124258 | 0.0921971 | 0.069971 | 0.2404621 | AGL | 178 |
| ENSG00000117620 | 555.0264823 | -0.073589122 | 0.1009655 | 0.3076108 | 0.5769089 | SLC35A3 | 23443 |
| ENSG00000156875 | 1384.968747 | -0.271856295 | 0.0872007 | 0.0003112 | 0.00445 | MFSD14A | 64645 |
| ENSG00000156876 | 504.018883 | -0.097543117 | 0.1099308 | 0.1975559 | 0.4510654 | SASS6 | 163786 |
| ENSG00000122435 | 349.9256771 | -0.005249351 | 0.0999453 | 0.9357766 | 0.9753553 | TRMT13 | 54482 |
| ENSG00000137992 | 410.0138685 | 0.130487107 | 0.1246252 | 0.109075 | 0.3197644 | DBT | 1629 |
| ENSG00000137996 | 765.4224681 | -0.026969887 | 0.0842352 | 0.6891075 | 0.858679 | RTCA | 8634 |
| ENSG00000079335 | 241.9567021 | -0.060043297 | 0.1207735 | 0.3826482 | 0.6435041 | CDC14A | 8556 |

| | | | | | | | | |
|---|---|---|---|---|---|---|---|---|
| ENSG00000162694 | 404.3456213 | -0.110209369 | 0.1196468 | 0.158876 | 0.3993092 | EXTL2 | | 2135 |
| ENSG00000273204 | 12.25197147 | 0.024576715 | 0.1376303 | 0.3187697 | NA | NA | NA | |
| ENSG00000162695 | 697.4792162 | -0.076998979 | 0.0926469 | 0.2746419 | 0.5431373 | SLC30A7 | | 148867 |
| ENSG00000117543 | 388.401078 | 0.027814395 | 0.1007684 | 0.6875918 | 0.8576358 | DPH5 | | 51611 |
| ENSG00000233184 | 104.8510416 | 0.240930598 | 0.3400051 | 0.023856 | 0.1169868 | DPH5-DT | | 102606465 |
| ENSG00000183298 | 62.52604453 | -0.035893418 | 0.1318999 | 0.4690143 | 0.7108642 | RPSAP19 | | 730029 |
| ENSG00000185946 | 235.8887897 | 0.024470434 | 0.1096568 | 0.7154402 | 0.871162 | RNPC3 | | 55599 |
| ENSG00000240038 | 22.69121709 | 0.034416948 | 0.1391966 | 0.2698597 | NA | AMY2B | | 280 |
| ENSG00000198890 | 351.2000733 | -0.13680235 | 0.1307933 | 0.0990969 | 0.3004976 | PRMT6 | | 55170 |
| ENSG00000134215 | 342.3248255 | -1.134696362 | 0.161712 | 1.23E-13 | 1.81E-11 | VAV3 | | 10451 |
| ENSG00000085491 | 2465.242144 | -0.282754639 | 0.0749856 | 2.78E-05 | 0.0005954 | SLC25A24 | | 29957 |
| ENSG00000260879 | 21.0888482 | -0.014690757 | 0.1339485 | 0.6371576 | NA | NA | NA | |
| ENSG00000162636 | 180.6744965 | -0.355459202 | 0.2553511 | 0.0094957 | 0.0615874 | EEIG2 | | 284611 |
| ENSG00000162639 | 556.914052 | -0.050156008 | 0.0940679 | 0.4706338 | 0.7115591 | HENMT1 | | 113802 |
| ENSG00000134186 | 3162.497445 | -0.012136566 | 0.0613677 | 0.8238661 | 0.9274924 | PRPF38B | | 55119 |
| ENSG00000116266 | 1141.422069 | -0.138072109 | 0.0880167 | 0.0510044 | 0.1946714 | STXBP3 | | 6814 |
| ENSG00000162641 | 32.89102528 | 0.012702119 | 0.1316246 | 0.7395629 | NA | AKNAD1 | | 254268 |
| ENSG00000121957 | 2192.71693 | -0.262981468 | 0.0764447 | 0.0001097 | 0.0018851 | GPSM2 | | 29899 |
| ENSG00000121940 | 1216.572899 | 0.02401743 | 0.075198 | 0.7031465 | 0.8651804 | CLCC1 | | 23155 |
| ENSG00000085433 | 731.7682179 | 0.126214667 | 0.1036739 | 0.0990965 | 0.3004976 | WDR47 | | 22911 |
| ENSG00000244716 | 69.79485258 | -0.085527355 | 0.1586467 | 0.1332722 | 0.3596932 | RPL17P7 | | 100132742 |
| ENSG00000197780 | 944.4685239 | 0.292692251 | 0.102857 | 0.0006419 | 0.0078191 | TAF13 | | 6884 |
| ENSG00000273382 | 29.00299837 | -0.019829071 | 0.1334908 | 0.5802307 | NA | NA | NA | |
| ENSG00000215717 | 838.9526797 | 0.178303169 | 0.1020544 | 0.0239759 | 0.1174264 | TMEM167B | | 56900 |
| ENSG00000116299 | 53.88333765 | -0.039739678 | 0.1351438 | 0.3924635 | 0.6529505 | ELAPOR1 | | 57535 |
| ENSG00000031698 | 4580.858442 | 0.245244694 | 0.060994 | 1.30E-05 | 0.0003161 | SARS1 | | 6301 |
| ENSG00000143126 | 964.1735617 | -0.005021378 | 0.0825658 | 0.9382891 | 0.9761997 | CELSR2 | | 1952 |
| ENSG00000134222 | 688.4610706 | -0.352325656 | 0.114045 | 0.0002174 | 0.0032761 | PSRC1 | | 84722 |
| ENSG00000134243 | 2704.620564 | -0.459630137 | 0.073976 | 4.78E-11 | 4.33E-09 | SORT1 | | 6272 |
| ENSG00000143106 | 3662.329533 | -0.11413616 | 0.0629235 | 0.0412258 | 0.1706444 | PSMA5 | | 5686 |
| ENSG00000162650 | 293.0959849 | 0.026561598 | 0.1053772 | 0.698251 | 0.8625201 | ATXN7L2 | | 127002 |
| ENSG00000174151 | 457.9250243 | -0.073249761 | 0.1034624 | 0.3133047 | 0.5822768 | CYB561D1 | | 284613 |
| ENSG00000181754 | 83.61553419 | 0.001159137 | 0.1237609 | 0.9839972 | 0.9939661 | AMIGO1 | | 57463 |
| ENSG00000065135 | 3178.825092 | -0.280672123 | 0.0708975 | 1.31E-05 | 0.0003179 | GNAI3 | | 2773 |
| ENSG00000116337 | 2303.972614 | -0.134316346 | 0.076813 | 0.0384605 | 0.1634846 | AMPD2 | | 271 |
| ENSG00000228703 | 13.77631961 | 0.008905365 | 0.1347627 | 0.7210262 | NA | NA | NA | |
| ENSG00000168765 | 57.16718793 | 0.038363937 | 0.134369 | 0.4155207 | 0.6697358 | GSTM4 | | 2948 |
| ENSG00000134202 | 171.5461972 | 0.504354357 | 0.232328 | 0.0015866 | 0.0159812 | GSTM3 | | 2947 |
| ENSG00000184371 | 253.1795303 | 0.696736616 | 0.1884126 | 1.19E-05 | 0.0002933 | CSF1 | | 1435 |
| ENSG00000168710 | 2880.793883 | -0.099040805 | 0.0669384 | 0.0901705 | 0.2835213 | AHCYL1 | | 10768 |
| ENSG00000143093 | 1109.93377 | -0.012164901 | 0.0758838 | 0.846416 | 0.9372292 | STRIP1 | | 85369 |
| ENSG00000258634 | 371.2763182 | 0.100857816 | 0.1169658 | 0.1905146 | 0.4412794 | NA | NA | |
| ENSG00000116396 | 200.5173925 | 0.141843006 | 0.1623162 | 0.0922632 | 0.2872944 | KCNC4 | | 3749 |
| ENSG00000162775 | 1169.391731 | 0.156708238 | 0.0877102 | 0.0282585 | 0.1324059 | RBM15 | | 64783 |
| ENSG00000273373 | 63.20175749 | -0.014171854 | 0.1293118 | 0.7469147 | 0.8899133 | NA | NA | |
| ENSG00000224699 | 59.56642277 | -0.046726519 | 0.1370443 | 0.3378653 | 0.6054931 | LAMTOR5-A | | 101410535 |
| ENSG00000134248 | 1503.001135 | -0.17241647 | 0.0824981 | 0.0125958 | 0.0749521 | LAMTOR5 | | 10542 |
| ENSG00000121931 | 679.1313605 | 0.129577241 | 0.1028198 | 0.0909573 | 0.2850744 | LRIF1 | | 55791 |
| ENSG00000156171 | 592.7923978 | -0.057852803 | 0.0950759 | 0.4091364 | 0.6643076 | DRAM2 | | 128338 |
| ENSG00000134255 | 850.7291402 | -0.151083592 | 0.0985466 | 0.04676 | 0.1849656 | CEPT1 | | 10390 |
| ENSG00000273221 | 17.08189272 | -0.010449426 | 0.1343146 | 0.7026469 | NA | NA | NA | |
| ENSG00000085465 | 50.60446873 | 0.742384499 | 0.7006187 | 0.005387 | 0.0401516 | OVGP1 | | 5016 |
| ENSG00000260948 | 36.63509472 | -0.002428705 | 0.1305138 | 0.9480443 | NA | NA | NA | |
| ENSG00000243960 | 21.11692814 | -0.020414046 | 0.1349802 | 0.5140755 | NA | NA | NA | |
| ENSG00000116455 | 1783.339372 | -0.158639476 | 0.0803093 | 0.0186462 | 0.0990466 | WDR77 | | 79084 |
| ENSG00000116459 | 3887.194318 | -0.209708759 | 0.0651288 | 0.000356 | 0.0049736 | ATP5PB | | 515 |
| ENSG00000143110 | 31.75468356 | 0.083251062 | 0.1681786 | 0.0492632 | NA | C1orf162 | | 128346 |
| ENSG00000116473 | 970.6357223 | -0.056379883 | 0.0812063 | 0.3889558 | 0.6493015 | RAP1A | | 5906 |
| ENSG00000197852 | 210.0006626 | -0.068219645 | 0.1227296 | 0.3317241 | 0.5992396 | INKA2 | | 55924 |
| ENSG00000064703 | 1080.906776 | 0.137764902 | 0.0882443 | 0.0525198 | 0.1989893 | DDX20 | | 11218 |
| ENSG00000143079 | 854.8948854 | 0.06208419 | 0.0856672 | 0.3625789 | 0.625731 | CTTNBP2NL | | 55917 |
| ENSG00000134245 | 99.96758588 | 0.073701028 | 0.1439274 | 0.2269812 | 0.4881913 | WNT2B | | 7482 |
| ENSG00000007341 | 302.0098533 | 0.069976751 | 0.1142398 | 0.3347707 | 0.6022573 | ST7L | | 54879 |
| ENSG00000116489 | 5304.981131 | -0.257589444 | 0.0616916 | 6.06E-06 | 0.0001637 | CAPZA1 | | 829 |
| ENSG00000238975 | 9.928948816 | -0.010085672 | 0.135339 | 0.6481211 | NA | NA | NA | |
| ENSG00000155363 | 2044.263101 | -0.204762192 | 0.0755599 | 0.0018632 | 0.0180137 | MOV10 | | 4343 |
| ENSG00000155366 | 2488.261187 | 0.19932331 | 0.0717852 | 0.0016206 | 0.0162407 | RHOC | | 389 |
| ENSG00000155367 | 49.62671374 | 0.020134957 | 0.1305286 | 0.6470063 | 0.8359336 | PPM1J | | 333926 |
| ENSG00000184599 | 15.82511246 | 0.022747304 | 0.1369139 | 0.3797294 | NA | TAFA3 | | 284467 |
| ENSG00000155380 | 4071.209869 | 0.003163735 | 0.0565904 | 0.9516777 | 0.9806656 | SLC16A1 | | 6566 |

| | | | | | | | |
|---|---|---|---|---|---|---|---|
| ENSG00000226419 | 166.9306189 | 0.572544448 | 0.237811 | 0.0008107 | 0.0094043 | SLC16A1-AS | 100506392 |
| ENSG00000198799 | 596.7479158 | 0.017756246 | 0.0897722 | 0.7788077 | 0.9075471 | LRIG2 | 9860 |
| ENSG00000081026 | 588.0717834 | -0.045717586 | 0.0932307 | 0.5091243 | 0.7408847 | MAGI3 | 260425 |
| ENSG00000116793 | 518.4626894 | -0.127003606 | 0.1183933 | 0.1126462 | 0.325537 | PHTF1 | 10745 |
| ENSG00000081019 | 512.7427036 | -0.128870977 | 0.1141655 | 0.1044795 | 0.3104023 | RSBN1 | 54665 |
| ENSG00000188761 | 10.41473472 | 0.027292335 | 0.1390805 | 0.1958326 | NA | BCL2L15 | 440603 |
| ENSG00000134262 | 579.5681487 | -0.058286403 | 0.0939192 | 0.4052356 | 0.6614808 | AP4B1 | 10717 |
| ENSG00000118655 | 823.5065199 | 0.122519176 | 0.0942695 | 0.093578 | 0.2901739 | DCLRE1B | 64858 |
| ENSG00000163349 | 2020.308832 | -0.162194978 | 0.0764748 | 0.0132675 | 0.0774503 | HIPK1 | 204851 |
| ENSG00000197323 | 2041.763131 | -0.069300215 | 0.0699993 | 0.2493186 | 0.5154909 | TRIM33 | 51592 |
| ENSG00000116752 | 1337.658415 | 0.124741613 | 0.0901605 | 0.0810637 | 0.2644248 | BCAS2 | 10286 |
| ENSG00000175984 | 155.5637751 | 0.006477472 | 0.115909 | 0.9175639 | 0.9678584 | DENND2C | 163259 |
| ENSG00000213281 | 3281.785088 | -0.354405534 | 0.0696468 | 4.47E-08 | 2.31E-06 | NRAS | 4893 |
| ENSG00000009307 | 14954.1224 | -0.321624593 | 0.0525544 | 1.24E-10 | 1.03E-08 | CSDE1 | 7812 |
| ENSG00000052723 | 1554.41939 | -0.203952788 | 0.0819587 | 0.0034708 | 0.0285312 | SIKE1 | 80143 |
| ENSG00000134259 | 30.71679985 | 2.711938371 | 0.6563126 | 1.81E-06 | NA | NGF | 4803 |
| ENSG00000173218 | 1375.912898 | -0.327664499 | 0.0879995 | 2.57E-05 | 0.0005587 | VANGL1 | 81839 |
| ENSG00000163393 | 65.43049455 | 0.005969967 | 0.1267163 | 0.9023628 | 0.9618324 | SLC22A15 | 55356 |
| ENSG00000163399 | 7233.988923 | -0.113653103 | 0.0561248 | 0.0261424 | 0.1250456 | ATP1A1 | 476 |
| ENSG00000269279 | 17.95233445 | -0.000719797 | 0.133362 | 0.9781814 | NA | NA | NA |
| ENSG00000203865 | 37.88905963 | 0.023289048 | 0.1327756 | 0.5635711 | NA | ATP1A1-AS1 | 84852 |
| ENSG00000224950 | 35.51297862 | 0.005769738 | 0.1307087 | 0.8831938 | NA | NA | NA |
| ENSG00000116815 | 331.0828388 | -0.089269563 | 0.1187127 | 0.2361802 | 0.5004209 | CD58 | 965 |
| ENSG00000177173 | 30.6522683 | 0.023046145 | 0.1339328 | 0.5316244 | NA | NA | NA |
| ENSG00000143061 | 1196.757817 | -0.167088982 | 0.0911262 | 0.0226206 | 0.1132097 | IGSF3 | 3321 |
| ENSG00000134247 | 188.0620653 | 0.028972565 | 0.1173353 | 0.6485348 | 0.836587 | PTGFRN | 5738 |
| ENSG00000116830 | 2255.529907 | -0.057759614 | 0.0656631 | 0.3152015 | 0.5839695 | TTF2 | 8458 |
| ENSG00000134253 | 121.2940383 | -0.011711955 | 0.1193198 | 0.8409081 | 0.9346002 | TRIM45 | 80263 |
| ENSG00000198162 | 1613.859988 | -0.293567603 | 0.0859048 | 9.74E-05 | 0.0017001 | MAN1A2 | 10905 |
| ENSG00000228217 | 29.61274135 | -0.040784997 | 0.1397739 | 0.2734093 | NA | NA | NA |
| ENSG00000196505 | 437.8614343 | -0.317972597 | 0.1452849 | 0.0030509 | 0.025987 | GDAP2 | 54834 |
| ENSG00000065183 | 1654.362585 | 0.195385816 | 0.0834069 | 0.0055019 | 0.0407531 | WDR3 | 10885 |
| ENSG00000155761 | 15.96155935 | 0.027531344 | 0.1378042 | 0.3195292 | NA | SPAG17 | 200162 |
| ENSG00000116874 | 224.7638678 | 0.003613808 | 0.1099082 | 0.9563705 | 0.9825402 | WARS2 | 10352 |
| ENSG00000231365 | 234.3549568 | 0.22141939 | 0.1876084 | 0.0311389 | 0.1416407 | WARS2-AS1 | 101929147 |
| ENSG00000143067 | 297.6589378 | -0.016827072 | 0.1040366 | 0.8032144 | 0.9180379 | ZNF697 | 90874 |
| ENSG00000092621 | 2306.56595 | 0.359048911 | 0.0793411 | 7.31E-07 | 2.62E-05 | PHGDH | 26227 |
| ENSG00000261662 | 10.07166525 | 0.004428036 | 0.1349792 | 0.8411576 | NA | NA | NA |
| ENSG00000134250 | 3665.194978 | -0.057272064 | 0.060797 | 0.2917681 | 0.5613809 | NOTCH2 | 4853 |
| ENSG00000273131 | 85.42827987 | -0.030049432 | 0.1268278 | 0.579049 | 0.7916677 | NA | NA |
| ENSG00000236933 | 71.21242268 | 0.020532588 | 0.127287 | 0.6833651 | 0.8547369 | NA | NA |
| ENSG00000188610 | 537.6390497 | 0.011148889 | 0.0949139 | 0.8715713 | 0.9483169 | FAM72B | 653820 |
| ENSG00000171943 | 204.7461931 | -0.144805841 | 0.1621663 | 0.0886117 | 0.2802783 | SRGAP2C | 653464 |
| ENSG00000269996 | 321.1014929 | -0.07089855 | 0.112265 | 0.3288276 | 0.5969902 | NA | NA |
| ENSG00000230880 | 46.62232157 | 0.037623775 | 0.1354119 | 0.3984189 | 0.657559 | NA | NA |
| ENSG00000234978 | 16.53827163 | -0.026896799 | 0.1374301 | 0.3411408 | NA | NA | NA |
| ENSG00000232745 | 14.97857342 | -0.000367097 | 0.134012 | 0.9865823 | NA | NA | NA |
| ENSG00000230850 | 17.60867402 | 0.014182986 | 0.1345685 | 0.6218298 | NA | NA | NA |
| ENSG00000185044 | 13.8163429 | -0.010055121 | 0.1349906 | 0.6742281 | NA | NA | NA |
| ENSG00000215784 | 244.3762841 | 0.067270414 | 0.1190109 | 0.3454678 | 0.6117325 | FAM72D | 728833 |
| ENSG00000196369 | 241.9464391 | -0.020118933 | 0.1089897 | 0.7619577 | 0.8979447 | SRGAP2B | 647135 |
| ENSG00000162825 | 47.98751807 | -0.021696718 | 0.1313591 | 0.609822 | 0.8128053 | NBPF20 | 100288142 |
| ENSG00000236943 | 8.876081991 | 0.030244458 | 0.1400603 | 0.1469533 | NA | NA | NA |
| ENSG00000225241 | 759.1111109 | 0.179165888 | 0.1038312 | 0.0246892 | 0.1197651 | NA | NA |
| ENSG00000168614 | 491.9648773 | -0.036682248 | 0.0948012 | 0.5950557 | 0.8031885 | NA | NA |
| ENSG00000178104 | 624.6074838 | -0.034353371 | 0.0912908 | 0.6154934 | 0.8158539 | PDE4DIP | 9659 |
| ENSG00000223380 | 1459.749879 | -0.037876382 | 0.0722187 | 0.5368454 | 0.7610305 | NA | NA |
| ENSG00000213240 | 192.99229 | 0.121008826 | 0.151475 | 0.1274989 | 0.3506746 | NA | NA |
| ENSG00000163386 | 637.6201173 | 0.093468424 | 0.0978603 | 0.2003655 | 0.4540225 | NA | NA |
| ENSG00000117289 | 10.503169 | 2.403097389 | 1.1533781 | 0.0015823 | NA | NA | NA |
| ENSG00000121851 | 133.3986201 | 0.002078846 | 0.1173599 | 0.9677384 | 0.9872153 | POLR3GL | 84265 |
| ENSG00000181039 | 16.32283309 | 0.030947397 | 0.1387232 | 0.27479 | NA | NA | NA |
| ENSG00000234222 | 59.96318466 | 0.013795868 | 0.129388 | 0.7555551 | 0.8946415 | LIX1L-AS1 | 105371260 |
| ENSG00000152022 | 701.446685 | -0.145862558 | 0.1052435 | 0.0620494 | 0.2224145 | NA | NA |
| ENSG00000131795 | 3091.627816 | -0.052504404 | 0.0630182 | 0.3466344 | 0.6132116 | NA | NA |
| ENSG00000211451 | 22.66347644 | 0.013682483 | 0.1338775 | 0.6610486 | NA | GNRHR2 | 114814 |
| ENSG00000131779 | 335.9335883 | -0.010989185 | 0.1016546 | 0.8707681 | 0.9478556 | PEX11B | 8799 |
| ENSG00000143127 | 35.61641206 | 0.012932683 | 0.1316419 | 0.7353831 | NA | ITGA10 | 8515 |
| ENSG00000131788 | 762.4045585 | -0.102731265 | 0.0935032 | 0.1522486 | 0.3895241 | PIAS3 | 10401 |
| ENSG00000186364 | 46.83830659 | 0.054843152 | 0.1438286 | 0.2199445 | 0.479502 | NUDT17 | 200035 |

| | | | | | | | | |
|---|---|---|---|---|---|---|---|---|
| ENSG00000186141 | 741.2901297 | 0.090107886 | 0.0960395 | 0.2118059 | 0.4684524 | POLR3C | | 10623 |
| ENSG00000121848 | 700.1671938 | -0.051420002 | 0.0888361 | 0.4453579 | 0.6936301 | NA | NA | |
| ENSG00000117262 | 281.7993783 | 0.024307336 | 0.1058148 | 0.7163724 | 0.8714352 | GPR89A | | 653519 |
| ENSG00000152042 | 85.1833969 | 0.031937809 | 0.127592 | 0.5578271 | 0.7769141 | NA | NA | |
| ENSG00000232637 | 193.9071629 | 0.031990275 | 0.117087 | 0.6193879 | 0.8179841 | NA | NA | |
| ENSG00000186275 | 170.0016636 | -0.021044268 | 0.1154714 | 0.7384556 | 0.885525 | NA | NA | |
| ENSG00000131791 | 424.1155199 | 0.077604928 | 0.108419 | 0.291594 | 0.5613232 | PRKAB2 | | 5565 |
| ENSG00000237188 | 20.14531183 | 0.017229828 | 0.1345729 | 0.5752898 | NA | NA | NA | |
| ENSG00000271721 | 30.78693246 | -0.004268555 | 0.1314251 | 0.9045717 | NA | NA | NA | |
| ENSG00000180867 | 35.26007184 | 0.009518728 | 0.1312585 | 0.804378 | NA | NA | NA | |
| ENSG00000131778 | 1555.162623 | -0.015293907 | 0.0715396 | 0.801134 | 0.9169162 | CHD1L | | 9557 |
| ENSG00000116128 | 537.1301595 | -0.215920677 | 0.1262054 | 0.017133 | 0.0926962 | BCL9 | | 607 |
| ENSG00000162836 | 123.2005576 | 0.050887327 | 0.1279634 | 0.4119238 | 0.6661689 | ACP6 | | 51205 |
| ENSG00000188092 | 37.3750988 | 0.010119971 | 0.1311761 | 0.793881 | NA | GPR89B | | 51463 |
| ENSG00000203836 | 35.86244108 | -0.00256202 | 0.1305257 | 0.9449327 | NA | NA | NA | |
| ENSG00000122497 | 394.4853774 | 0.062154766 | 0.1067026 | 0.3837184 | 0.6445521 | NA | NA | |
| ENSG00000223612 | 9.317365207 | -0.004792156 | 0.135063 | 0.8198928 | NA | NA | NA | |
| ENSG00000203832 | 239.423932 | -0.042790659 | 0.1131524 | 0.528004 | 0.7547252 | NA | NA | |
| ENSG00000243452 | 622.3055323 | 0.011514718 | 0.0912376 | 0.8441778 | 0.9360965 | NA | NA | |
| ENSG00000203827 | 83.72948858 | 0.016981186 | 0.1251499 | 0.7477727 | 0.8903907 | NA | NA | |
| ENSG00000232527 | 61.15621548 | -0.017107939 | 0.1280205 | 0.7163645 | 0.8714352 | LINC02802 | | 100996732 |
| ENSG00000226067 | 11.18070919 | -0.004581261 | 0.1346694 | 0.8432313 | NA | NA | NA | |
| ENSG00000235999 | 57.58742566 | 0.031454494 | 0.1323437 | 0.497122 | 0.7321753 | NA | NA | |
| ENSG00000203817 | 30.88462558 | 0.024001966 | 0.1345492 | 0.5024041 | NA | NA | NA | |
| ENSG00000269501 | 33.87129345 | -0.0018529 | 0.1312544 | 0.9568998 | NA | NA | NA | |
| ENSG00000233368 | 19.1174148 | 0.026271567 | 0.1368199 | 0.3839528 | NA | NA | NA | |
| ENSG00000203814 | 15.44611318 | -0.013726183 | 0.1348941 | 0.6062976 | NA | H2BC18 | | 440689 |
| ENSG00000183941 | 39.55730735 | 0.078968472 | 0.1617746 | 0.0834441 | NA | NA | NA | |
| ENSG00000183558 | 63.28084056 | -0.045462526 | 0.1354625 | 0.3658666 | 0.6286684 | NA | NA | |
| ENSG00000220323 | 14.20295897 | -0.010469274 | 0.1347266 | 0.6815612 | NA | H2BC19P | | 337874 |
| ENSG00000261716 | 146.9052424 | -0.004559908 | 0.1172726 | 0.9383629 | 0.9761997 | H2BC20P | | 337873 |
| ENSG00000272993 | 49.48496942 | 0.014468696 | 0.1292475 | 0.7464367 | 0.8896359 | NA | NA | |
| ENSG00000203819 | 36.06951393 | -0.010788082 | 0.1315273 | 0.771806 | NA | NA | NA | |
| ENSG00000203812 | 26.04748732 | -0.027235188 | 0.1359911 | 0.4179149 | NA | NA | NA | |
| ENSG00000182217 | 104.196511 | 0.377982885 | 0.3503723 | 0.0111138 | 0.0685738 | NA | NA | |
| ENSG00000184678 | 241.002746 | 0.055645714 | 0.1190208 | 0.4179053 | 0.6710348 | H2BC21 | | 8349 |
| ENSG00000184260 | 13.76952823 | -0.044894468 | 0.1453236 | 0.0766163 | NA | H2AC20 | | 8338 |
| ENSG00000178096 | 52.93313073 | -0.023109647 | 0.1302379 | 0.6111309 | 0.8136106 | BOLA1 | | 51027 |
| ENSG00000159164 | 15.69400008 | 0.024919758 | 0.1370863 | 0.3660365 | NA | SV2A | | 9900 |
| ENSG00000143368 | 2000.217058 | -0.027800061 | 0.0692954 | 0.6411152 | 0.8315061 | SF3B4 | | 10262 |
| ENSG00000014914 | 75.74423127 | -0.050806289 | 0.1360608 | 0.3394408 | 0.6066872 | MTMR11 | | 10903 |
| ENSG00000163113 | 912.2427109 | -0.003931602 | 0.07988 | 0.9508015 | 0.9802173 | NA | NA | |
| ENSG00000136631 | 452.018278 | -0.058502012 | 0.1009045 | 0.4103006 | 0.6653785 | VPS45 | | 11311 |
| ENSG00000023902 | 131.6498324 | -0.0551978 | 0.1291944 | 0.3780875 | 0.6395077 | PLEKHO1 | | 51177 |
| ENSG00000143401 | 3887.007649 | -0.269413039 | 0.0764414 | 7.63E-05 | 0.0014096 | ANP32E | | 81611 |
| ENSG00000117362 | 2073.927146 | -0.178032074 | 0.0737088 | 0.0053776 | 0.0401121 | APH1A | | 51107 |
| ENSG00000187145 | 403.0765103 | 0.014885385 | 0.0997204 | 0.8285175 | 0.9293175 | NA | NA | |
| ENSG00000117360 | 1091.946229 | 0.052451661 | 0.0783788 | 0.4112582 | 0.6661689 | PRPF3 | | 9129 |
| ENSG00000163125 | 1175.667991 | 0.074508094 | 0.0811449 | 0.264244 | 0.5321783 | RPRD2 | | 23248 |
| ENSG00000143374 | 484.2091139 | -0.015886451 | 0.0933313 | 0.8135391 | 0.9238507 | TARS2 | | 80222 |
| ENSG00000143369 | 12.32991326 | 0.020991988 | 0.1367537 | 0.3931765 | NA | ECM1 | | 1893 |
| ENSG00000237781 | 20.3806053 | -0.004653036 | 0.1330362 | 0.8781065 | NA | NA | NA | |
| ENSG00000143382 | 102.4898475 | 0.086473795 | 0.1538292 | 0.1636346 | 0.4063384 | ADAMTSL4 | | 54507 |
| ENSG00000143384 | 5421.415574 | -0.073485613 | 0.0597082 | 0.1724874 | 0.4194059 | MCL1 | | 4170 |
| ENSG00000143420 | 4236.678668 | -0.168965216 | 0.0632655 | 0.0028764 | 0.0249437 | ENSA | | 2029 |
| ENSG00000143457 | 304.0962651 | -0.008186245 | 0.1031257 | 0.9015715 | 0.9614952 | GOLPH3L | | 55204 |
| ENSG00000143452 | 34.67075751 | -0.014951951 | 0.1320467 | 0.6910937 | NA | HORMAD1 | | 84072 |
| ENSG00000163131 | 17.03871002 | 0.021237075 | 0.1358743 | 0.4609716 | NA | CTSS | | 1520 |
| ENSG00000143387 | 78.38102827 | 0.025719068 | 0.1275059 | 0.6201751 | 0.818389 | CTSK | | 1513 |
| ENSG00000143437 | 774.4316452 | -0.127587918 | 0.0985828 | 0.0879988 | 0.2791727 | ARNT | | 405 |
| ENSG00000143379 | 575.3367341 | -0.034572304 | 0.0946396 | 0.6157182 | 0.8158539 | SETDB1 | | 9869 |
| ENSG00000143418 | 3155.784464 | -0.002109897 | 0.0600223 | 0.9674224 | 0.9871985 | CERS2 | | 29956 |
| ENSG00000259357 | 28.14660953 | -0.002695412 | 0.13254 | 0.9317195 | NA | NA | NA | |
| ENSG00000143409 | 133.1589668 | -0.04153365 | 0.1234798 | 0.502137 | 0.7365601 | MINDY1 | | 55793 |
| ENSG00000143363 | 309.5323728 | -0.066220276 | 0.1151329 | 0.3550607 | 0.6192724 | PRUNE1 | | 58497 |
| ENSG00000163141 | 20.44935859 | 0.045711781 | 0.1440861 | 0.1477999 | NA | BNIPL | | 149428 |
| ENSG00000143443 | 45.65512556 | 0.053498734 | 0.1426526 | 0.2383847 | 0.5030834 | C1orf56 | | 54964 |
| ENSG00000197622 | 2060.079421 | -0.177276643 | 0.0761839 | 0.0067971 | 0.0476855 | CDC42SE1 | | 56882 |
| ENSG00000213190 | 85.56855733 | 0.007588798 | 0.124134 | 0.8859191 | 0.9542113 | MLLT11 | | 10962 |
| ENSG00000143458 | 244.4126668 | -0.000355258 | 0.1069906 | 0.9946187 | 0.9977447 | GABPB2 | | 126626 |

| | | | | | | | | |
|---|---|---|---|---|---|---|---|---|
| ENSG00000143434 | 221.8632261 | -0.070042099 | 0.123044 | 0.3205475 | 0.589192 | SEMA6C | | 10500 |
| ENSG00000163156 | 415.7731166 | 0.463012795 | 0.1521423 | 0.0001696 | 0.002663 | SCNM1 | | 79005 |
| ENSG00000163155 | 22.74489228 | 0.044523145 | 0.1428916 | 0.1838279 | NA | LYSMD1 | | 388695 |
| ENSG00000163159 | 722.4586201 | 0.133425919 | 0.1019324 | 0.0809493 | 0.2642336 | VPS72 | | 6944 |
| ENSG00000143398 | 2689.281174 | 0.127511973 | 0.068676 | 0.0327886 | 0.1462403 | PIP5K1A | | 8394 |
| ENSG00000159352 | 3234.50355 | -0.004849531 | 0.0624727 | 0.9297211 | 0.9728106 | PSMD4 | | 5710 |
| ENSG00000232671 | 21.5538243 | 0.02414503 | 0.1357594 | 0.4497141 | NA | ZNF687-AS1 | | 100507670 |
| ENSG00000143373 | 1044.964131 | 0.071955165 | 0.082144 | 0.2818295 | 0.5509708 | ZNF687 | | 57592 |
| ENSG00000143393 | 1657.786169 | 0.035561272 | 0.0728609 | 0.5651832 | 0.7820725 | PI4KB | | 5298 |
| ENSG00000143390 | 775.7638639 | -0.034660795 | 0.0842095 | 0.6028312 | 0.8084582 | RFX5 | | 5993 |
| ENSG00000159377 | 4020.130281 | -0.219756267 | 0.063348 | 0.0001365 | 0.0022422 | PSMB4 | | 5692 |
| ENSG00000143442 | 1507.940318 | -0.076652212 | 0.0773616 | 0.2327351 | 0.4951049 | POGZ | | 23126 |
| ENSG00000143375 | 441.4021701 | -0.103996793 | 0.1179716 | 0.1783178 | 0.4274431 | CGN | | 57530 |
| ENSG00000143367 | 494.5149298 | 0.635076262 | 0.133868 | 1.33E-07 | 6.09E-06 | TUFT1 | | 7286 |
| ENSG00000143376 | 855.3660288 | -0.126799173 | 0.0954244 | 0.0848257 | 0.2723608 | SNX27 | | 81609 |
| ENSG00000269621 | 11.2591642 | 0.012830259 | 0.135584 | 0.5743553 | NA | NA | NA | |
| ENSG00000143436 | 1240.736698 | -0.199519204 | 0.0883098 | 0.0064686 | 0.0460447 | MRPL9 | | 65005 |
| ENSG00000143450 | 25.34428155 | -0.020016675 | 0.1342341 | 0.5511519 | NA | OAZ3 | | 51686 |
| ENSG00000182134 | 273.1990882 | -0.420811495 | 0.1804896 | 0.0013725 | 0.01437 | TDRKH | | 11022 |
| ENSG00000203288 | 24.03652662 | 0.013392858 | 0.1333306 | 0.6854862 | NA | TDRKH-AS1 | | 109729141 |
| ENSG00000159445 | 135.2368821 | 0.009972438 | 0.1177659 | 0.8703758 | 0.9478556 | THEM4 | | 117145 |
| ENSG00000197747 | 6055.314634 | -0.087013994 | 0.0584553 | 0.0987597 | 0.2998731 | S100A10 | | 6281 |
| ENSG00000163191 | 4941.438244 | 0.098170483 | 0.0617055 | 0.0746808 | 0.250875 | S100A11 | | 6282 |
| ENSG00000238279 | 12.07526946 | 0.009179897 | 0.1349505 | 0.7023738 | NA | NA | NA | |
| ENSG00000197956 | 15182.79175 | 0.038489939 | 0.0614595 | 0.471023 | 0.7117749 | S100A6 | | 6277 |
| ENSG00000196154 | 108.1398847 | 0.014418837 | 0.1252868 | 0.7820397 | 0.9095461 | S100A4 | | 6275 |
| ENSG00000188015 | 308.081225 | 0.132978573 | 0.1492398 | 0.108381 | 0.3185678 | S100A3 | | 6274 |
| ENSG00000196754 | 966.9207348 | -0.356200182 | 0.1050158 | 7.66E-05 | 0.0014098 | S100A2 | | 6273 |
| ENSG00000188643 | 3670.482573 | 0.042310622 | 0.0615273 | 0.4412914 | 0.6905191 | S100A16 | | 140576 |
| ENSG00000189171 | 814.6634171 | 0.069622287 | 0.094936 | 0.3337463 | 0.601084 | S100A13 | | 6284 |
| ENSG00000160678 | 34.43165071 | 0.002331173 | 0.1306511 | 0.9535173 | NA | S100A1 | | 6271 |
| ENSG00000272030 | 32.53824547 | -0.000582094 | 0.1309742 | 0.9835463 | NA | NA | NA | |
| ENSG00000160679 | 2798.244233 | 0.077305341 | 0.06584 | 0.1804425 | 0.4299157 | CHTOP | | 26097 |
| ENSG00000143553 | 419.7317921 | -0.19529182 | 0.1356566 | 0.0320242 | 0.1442079 | SNAPIN | | 23557 |
| ENSG00000143621 | 4729.505745 | -0.136263716 | 0.0601867 | 0.0117828 | 0.0714043 | ILF2 | | 3608 |
| ENSG00000169418 | 36.53203381 | 0.015782303 | 0.1314372 | 0.6931263 | NA | NPR1 | | 4881 |
| ENSG00000143624 | 947.3888422 | -0.0476696 | 0.0822978 | 0.4701837 | 0.7115591 | INTS3 | | 65123 |
| ENSG00000143554 | 19.70301463 | -0.028327179 | 0.1378334 | 0.3175011 | NA | SLC27A3 | | 11000 |
| ENSG00000143614 | 740.9364516 | -0.060590708 | 0.0908324 | 0.3818555 | 0.642902 | GATAD2B | | 57459 |
| ENSG00000198837 | 1269.88844 | 0.089875445 | 0.0849149 | 0.1871982 | 0.4381671 | DENND4B | | 9909 |
| ENSG00000160741 | 735.1235804 | -0.110718126 | 0.0987242 | 0.1346625 | 0.3614858 | CRTC2 | | 200186 |
| ENSG00000143570 | 3045.799861 | 0.100400043 | 0.0632158 | 0.0734899 | 0.2482461 | SLC39A1 | | 27173 |
| ENSG00000273026 | 13.14747799 | -0.016478627 | 0.135662 | 0.5139727 | NA | NA | NA | |
| ENSG00000143578 | 67.93819254 | 0.023246679 | 0.1285744 | 0.636213 | 0.8275522 | CREB3L4 | | 148327 |
| ENSG00000143543 | 1523.960991 | 0.01124934 | 0.0737631 | 0.8599739 | 0.9435431 | JTB | | 10899 |
| ENSG00000143545 | 946.8131365 | -0.009510514 | 0.0795312 | 0.8822917 | 0.9525622 | RAB13 | | 5872 |
| ENSG00000177954 | 8578.533527 | -0.083964573 | 0.1265341 | 0.2673202 | 0.5355115 | RPS27 | | 6232 |
| ENSG00000143549 | 13854.93623 | -0.166558689 | 0.051793 | 0.0005349 | 0.0067838 | TPM3 | | 7170 |
| ENSG00000143612 | 2859.012063 | 0.091988361 | 0.0675951 | 0.1184461 | 0.335893 | C1orf43 | | 25912 |
| ENSG00000143569 | 3556.350322 | -0.11458895 | 0.0646419 | 0.0447263 | 0.1800304 | UBAP2L | | 9898 |
| ENSG00000252817 | 13.30656669 | -0.019536146 | 0.1362867 | 0.4361828 | NA | NA | NA | |
| ENSG00000143575 | 1657.723609 | 0.101684792 | 0.0756566 | 0.11063 | 0.3224226 | HAX1 | | 10456 |
| ENSG00000143515 | 998.6036961 | 0.110907743 | 0.0892872 | 0.1168974 | 0.33377 | ATP8B2 | | 57198 |
| ENSG00000160712 | 526.630697 | 0.975732821 | 0.1303171 | 3.90E-15 | 7.36E-13 | IL6R | | 3570 |
| ENSG00000160714 | 1547.839335 | 0.066986651 | 0.0735209 | 0.2819022 | 0.5510207 | UBE2Q1 | | 55585 |
| ENSG00000229780 | 8.77915378 | 0.005881039 | 0.1352277 | 0.7793269 | NA | UBE2Q1-AS | | 100874097 |
| ENSG00000160710 | 6792.470021 | -0.091838672 | 0.0554968 | 0.069579 | 0.2400304 | ADAR | | 103 |
| ENSG00000163344 | 684.9720839 | 0.005413989 | 0.0859544 | 0.9363278 | 0.9755316 | PMVK | | 10654 |
| ENSG00000163346 | 534.2769371 | -0.31434672 | 0.1399608 | 0.0027387 | 0.024017 | PBXIP1 | | 57326 |
| ENSG00000163348 | 985.5307069 | 0.033014868 | 0.0840784 | 0.6208 | 0.818389 | PYGO2 | | 90780 |
| ENSG00000271380 | 28.60906646 | -0.022142945 | 0.1351623 | 0.4909995 | NA | NA | NA | |
| ENSG00000160691 | 12499.57372 | -0.108207228 | 0.0659588 | 0.0615561 | 0.2214309 | SHC1 | | 6464 |
| ENSG00000173207 | 1788.977387 | 0.018360612 | 0.0682234 | 0.7585333 | 0.8963508 | CKS1B | | 1163 |
| ENSG00000264349 | 8.551990324 | 0.009557814 | 0.1355844 | 0.6414027 | NA | MIR4258 | | 100423020 |
| ENSG00000160688 | 676.054145 | 0.332002476 | 0.1164431 | 0.0005053 | 0.0065077 | FLAD1 | | 80308 |
| ENSG00000160685 | 630.6055113 | 0.043674575 | 0.0948452 | 0.5309703 | 0.756397 | ZBTB7B | | 51043 |
| ENSG00000232093 | 13.88468657 | 0.015908165 | 0.1355826 | 0.5310292 | NA | NA | NA | |
| ENSG00000143537 | 975.5015818 | 0.100136966 | 0.0900914 | 0.1543553 | 0.3926447 | ADAM15 | | 8751 |
| ENSG00000243364 | 73.48776339 | 0.036242698 | 0.130749 | 0.4833631 | 0.7217391 | EFNA4 | | 1945 |
| ENSG00000143590 | 24.65017792 | -0.023369753 | 0.1350759 | 0.4842034 | NA | EFNA3 | | 1944 |

| | | | | | | | | |
|---|---|---|---|---|---|---|---|---|
| ENSG00000169242 | 1862.35558 | -0.849511769 | 0.0858494 | 2.58E-24 | 1.12E-21 | EFNA1 | | 1942 |
| ENSG00000169241 | 591.8423867 | 0.174267658 | 0.1166171 | 0.0381025 | 0.1622588 | SLC50A1 | | 55974 |
| ENSG00000179085 | 322.1580707 | -0.015102906 | 0.1028235 | 0.8232884 | 0.92746 | DPM3 | | 54344 |
| ENSG00000163463 | 78.16816435 | -0.01476204 | 0.1263864 | 0.7662937 | 0.8994294 | KRTCAP2 | | 200185 |
| ENSG00000163462 | 61.58843842 | 0.027024784 | 0.1309987 | 0.5587263 | 0.7775341 | TRIM46 | | 80128 |
| ENSG00000185499 | 68.10518804 | -0.006371187 | 0.1263311 | 0.8945185 | 0.9577333 | MUC1 | | 4582 |
| ENSG00000169231 | 82.53760257 | 0.087161787 | 0.1558127 | 0.1532709 | 0.3908224 | THBS3 | | 7059 |
| ENSG00000231064 | 29.69749177 | 0.043949629 | 0.1420624 | 0.2121825 | NA | THBS3-AS1 | | 105371450 |
| ENSG00000173171 | 288.7839191 | -0.005824019 | 0.1051655 | 0.9279215 | 0.9723738 | MTX1 | | 4580 |
| ENSG00000160766 | 60.8785547 | 0.178987517 | 0.3271253 | 0.0163662 | 0.0895846 | NA | NA | |
| ENSG00000177628 | 522.7879959 | 0.438915165 | 0.1299228 | 5.99E-05 | 0.0011492 | GBA1 | | 2629 |
| ENSG00000160767 | 939.6170441 | 0.024558227 | 0.0806278 | 0.7072703 | 0.8675568 | ENTREP3 | | 10712 |
| ENSG00000116521 | 1476.837194 | 0.071023612 | 0.0743694 | 0.2574546 | 0.52429 | SCAMP3 | | 10067 |
| ENSG00000176444 | 496.6010086 | -0.022362376 | 0.0952282 | 0.7435105 | 0.8884855 | CLK2 | | 1196 |
| ENSG00000143630 | 260.6220747 | 0.025852369 | 0.1090549 | 0.7020684 | 0.8649636 | HCN3 | | 57657 |
| ENSG00000160752 | 2370.740095 | -0.198255758 | 0.0742421 | 0.0022182 | 0.0204734 | FDPS | | 2224 |
| ENSG00000225855 | 55.86426295 | -0.025114441 | 0.1303922 | 0.5885518 | 0.7984432 | RUSC1-AS1 | | 284618 |
| ENSG00000160753 | 1522.642733 | 0.095098726 | 0.075816 | 0.1366298 | 0.3640449 | RUSC1 | | 23623 |
| ENSG00000116539 | 2214.612351 | -0.006381425 | 0.0659689 | 0.9112695 | 0.9649147 | ASH1L | | 55870 |
| ENSG00000235919 | 56.6304446 | 0.037818627 | 0.1339128 | 0.4274416 | 0.6791494 | ASH1L-AS1 | | 645676 |
| ENSG00000125459 | 256.357701 | 0.020621791 | 0.107372 | 0.7602708 | 0.8970427 | MSTO1 | | 55154 |
| ENSG00000203761 | 35.12785527 | 0.027396313 | 0.1342224 | 0.4859567 | NA | NA | NA | |
| ENSG00000163374 | 1001.156845 | 0.054503593 | 0.0821362 | 0.4094282 | 0.664425 | YY1AP1 | | 55249 |
| ENSG00000132676 | 2460.723524 | 0.060850182 | 0.0644602 | 0.2798438 | 0.5484657 | DAP3 | | 7818 |
| ENSG00000116580 | 1161.852126 | 0.119757842 | 0.0855214 | 0.0840655 | 0.2709074 | GON4L | | 54856 |
| ENSG00000132718 | 101.6335563 | -0.081232142 | 0.1472614 | 0.1981157 | 0.4513782 | SYT11 | | 23208 |
| ENSG00000143622 | 520.7255466 | 0.040669639 | 0.0978412 | 0.5876208 | 0.7977929 | RIT1 | | 6016 |
| ENSG00000132680 | 1321.725981 | 0.229639822 | 0.2135823 | 0.03009 | 0.1387059 | KHDC4 | | 22889 |
| ENSG00000116584 | 1186.44682 | 0.346122354 | 0.0919584 | 2.03E-05 | 0.0004628 | ARHGEF2 | | 9181 |
| ENSG00000163479 | 2604.120551 | -0.040040305 | 0.0637968 | 0.4770944 | 0.7163677 | SSR2 | | 6746 |
| ENSG00000160803 | 1492.595926 | 0.00205816 | 0.0746733 | 0.974747 | 0.9905193 | UBQLN4 | | 56893 |
| ENSG00000116586 | 473.6917404 | -0.028574739 | 0.0971103 | 0.6777684 | 0.8531264 | LAMTOR2 | | 28956 |
| ENSG00000254726 | 288.0276388 | 0.328302645 | 0.1880828 | 0.006879 | 0.0480881 | MEX3A | | 92312 |
| ENSG00000160789 | 7887.137829 | -0.140217538 | 0.0658568 | 0.0162633 | 0.0891048 | LMNA | | 4000 |
| ENSG00000160785 | 970.5952695 | -0.014768761 | 0.0797839 | 0.8189513 | 0.9256091 | SLC25A44 | | 9673 |
| ENSG00000160783 | 245.7488902 | 0.001176214 | 0.1072779 | 0.9870722 | 0.994767 | PMF1 | | 11243 |
| ENSG00000160781 | 62.05182681 | 0.067406791 | 0.1473339 | 0.1980439 | 0.4513782 | PAQR6 | | 79957 |
| ENSG00000198952 | 4730.783577 | 0.029738467 | 0.0599327 | 0.5817803 | 0.7933361 | SMG5 | | 23381 |
| ENSG00000163472 | 199.4186224 | 1.480471394 | 0.2094244 | 8.15E-14 | 1.22E-11 | TMEM79 | | 84283 |
| ENSG00000198715 | 28.23482798 | 0.010853346 | 0.1325293 | 0.7544045 | NA | GLMP | | 112770 |
| ENSG00000163468 | 11371.70365 | -0.138353541 | 0.0536543 | 0.0050548 | 0.0382914 | CCT3 | | 7203 |
| ENSG00000116604 | 528.8185687 | 0.128598591 | 0.1170426 | 0.1085313 | 0.3187715 | MEF2D | | 4209 |
| ENSG00000183856 | 1719.333042 | 0.099325742 | 0.0779826 | 0.1263514 | 0.3489976 | IQGAP3 | | 128239 |
| ENSG00000163382 | 1045.149479 | -0.091463172 | 0.08487 | 0.1775859 | 0.4270449 | NAXE | | 128240 |
| ENSG00000160818 | 2082.29785 | 0.152604055 | 0.0731073 | 0.0155608 | 0.0864729 | GPATCH4 | | 54865 |
| ENSG00000272405 | 13.21536277 | 0.026127365 | 0.1379034 | 0.3026369 | NA | NA | NA | |
| ENSG00000132688 | 54.17873379 | -0.010883102 | 0.1291707 | 0.800694 | 0.9169162 | NES | | 10763 |
| ENSG00000143320 | 81.06640777 | 0.061119209 | 0.139733 | 0.2776272 | 0.5461905 | CRABP2 | | 1382 |
| ENSG00000143319 | 1133.75197 | 0.254130291 | 0.1000567 | 0.0019901 | 0.0187773 | ISG20L2 | | 81875 |
| ENSG00000143303 | 255.7473199 | 0.499466318 | 0.1940697 | 0.0006095 | 0.0074942 | METTL25B | | 51093 |
| ENSG00000143314 | 768.9216412 | -0.040783445 | 0.0865055 | 0.5497529 | 0.771065 | MRPL24 | | 79590 |
| ENSG00000143321 | 11654.17203 | -0.062705769 | 0.0505133 | 0.1832522 | 0.4329849 | HDGF | | 3068 |
| ENSG00000143294 | 1580.695974 | 0.067881625 | 0.0734484 | 0.2752864 | 0.543704 | PRCC | | 5546 |
| ENSG00000187800 | 99.40389283 | -0.112524222 | 0.1715968 | 0.1044047 | 0.3104023 | PEAR1 | | 375033 |
| ENSG00000132694 | 1506.634207 | -0.080425011 | 0.0757079 | 0.2054964 | 0.4607272 | ARHGEF11 | | 9826 |
| ENSG00000117036 | 815.9759177 | -0.007625297 | 0.0825931 | 0.9069287 | 0.963256 | ETV3 | | 2117 |
| ENSG00000183853 | 6596.928261 | -0.39006166 | 0.0587365 | 3.56E-12 | 4.07E-10 | KIRREL1 | | 55243 |
| ENSG00000226520 | 11.66718967 | -0.005669649 | 0.1347948 | 0.8048242 | NA | NA | NA | |
| ENSG00000158716 | 247.290779 | 0.086020002 | 0.1280662 | 0.2424415 | 0.5082482 | DUSP23 | | 54935 |
| ENSG00000213085 | 59.88678129 | 0.055702751 | 0.1407875 | 0.2717326 | 0.5394882 | CFAP45 | | 25790 |
| ENSG00000158710 | 7201.585966 | 0.130539056 | 0.0596612 | 0.01478 | 0.0833231 | TAGLN2 | | 8407 |
| ENSG00000260766 | 28.77167492 | 0.012594785 | 0.1324289 | 0.7241478 | NA | NA | NA | |
| ENSG00000143315 | 194.1034585 | 0.030805821 | 0.1188886 | 0.6234971 | 0.8198885 | PIGM | | 93183 |
| ENSG00000162729 | 341.7893142 | 0.019251965 | 0.1011443 | 0.7796383 | 0.9079716 | IGSF8 | | 93185 |
| ENSG00000227741 | 14.43140055 | 0.015121848 | 0.1352291 | 0.5668345 | NA | LOC729867 | | 729867 |
| ENSG00000162734 | 3497.802042 | 0.240844988 | 0.0665818 | 6.75E-05 | 0.0012674 | PEA15 | | 8682 |
| ENSG00000132716 | 866.3372173 | -0.039129444 | 0.0851356 | 0.5586573 | 0.7775341 | DCAF8 | | 50717 |
| ENSG00000162735 | 797.0323245 | -0.06179507 | 0.0863916 | 0.3626036 | 0.625731 | PEX19 | | 5824 |
| ENSG00000122218 | 3513.816687 | -0.052546163 | 0.063703 | 0.3513197 | 0.6171375 | COPA | | 1314 |
| ENSG00000162736 | 993.4665655 | -0.091722665 | 0.0849081 | 0.1776873 | 0.4270449 | NCSTN | | 23385 |

| | | | | | | | |
|---|---|---|---|---|---|---|---|
| ENSG00000158769 | 307.5799121 | -0.086372286 | 0.1182441 | 0.2483967 | 0.5148194 | F11R | 50848 |
| ENSG00000215845 | 19.45256407 | -0.017783377 | 0.134835 | 0.5505114 | NA | TSTD1 | 100131187 |
| ENSG00000158773 | 930.5768982 | -0.070364289 | 0.0839167 | 0.2919844 | 0.5617046 | USF1 | 7391 |
| ENSG00000143256 | 2114.186784 | 0.268503475 | 0.0760683 | 7.65E-05 | 0.0014096 | PFDN2 | 5202 |
| ENSG00000158793 | 382.7110262 | 0.092626447 | 0.1175138 | 0.2216652 | 0.4810983 | NIT1 | 4817 |
| ENSG00000158796 | 794.0746177 | -0.210494547 | 0.1055139 | 0.0105519 | 0.0658743 | DEDD | 9191 |
| ENSG00000143222 | 1194.412394 | -0.01217991 | 0.0744188 | 0.8445963 | 0.936404 | UFC1 | 51506 |
| ENSG00000224985 | 24.1469785 | 0.023099483 | 0.1358739 | 0.4495582 | NA | NA | NA |
| ENSG00000143258 | 527.6095283 | -0.055102794 | 0.1001249 | 0.435685 | 0.6861988 | USP21 | 27005 |
| ENSG00000143224 | 157.1057404 | -0.034700773 | 0.1190241 | 0.5808485 | 0.7928829 | PPOX | 5498 |
| ENSG00000158850 | 837.3806017 | -0.001427517 | 0.083928 | 0.9819554 | 0.9932861 | B4GALT3 | 8703 |
| ENSG00000158864 | 1540.126693 | -0.117194741 | 0.0816088 | 0.0806097 | 0.2634794 | NDUFS2 | 4720 |
| ENSG00000158882 | 321.604416 | -0.050914923 | 0.1079468 | 0.4651417 | 0.7086768 | TOMM40L | 84134 |
| ENSG00000143252 | 1604.522945 | -0.117743891 | 0.0767213 | 0.0686441 | 0.2375377 | SDHC | 6391 |
| ENSG00000188931 | 11.19709008 | -0.001693615 | 0.1346434 | 0.9392688 | NA | CFAP126 | 257177 |
| ENSG00000235477 | 123.7788873 | 0.00835161 | 0.1194878 | 0.8881274 | 0.9545517 | NA | NA |
| ENSG00000081721 | 635.4194049 | 0.051645566 | 0.0923244 | 0.4759289 | 0.7156114 | DUSP12 | 11266 |
| ENSG00000118217 | 887.4264938 | -0.091148835 | 0.0902728 | 0.1942372 | 0.4462795 | ATF6 | 22926 |
| ENSG00000162745 | 85.96443965 | -0.102900539 | 0.1683395 | 0.1104966 | 0.3222505 | OLFML2B | 25903 |
| ENSG00000198929 | 65.48337156 | 0.046644738 | 0.1363908 | 0.3503126 | 0.6163779 | NOS1AP | 9722 |
| ENSG00000239887 | 97.66396544 | -0.028933414 | 0.1248561 | 0.6070869 | 0.8115567 | C1orf226 | 400793 |
| ENSG00000152332 | 5648.443507 | -0.055709164 | 0.0562258 | 0.2757418 | 0.5439858 | UHMK1 | 127933 |
| ENSG00000117143 | 6336.293009 | 0.58631896 | 0.0696289 | 2.87E-18 | 6.84E-16 | UAP1 | 6675 |
| ENSG00000272574 | 32.09834399 | 0.064965001 | 0.1526038 | 0.1119484 | NA | NA | NA |
| ENSG00000162733 | 90.97903003 | 0.083064716 | 0.1512904 | 0.1766455 | 0.4257059 | DDR2 | 4921 |
| ENSG00000132196 | 162.9539952 | -0.316257867 | 0.2575367 | 0.0137191 | 0.0795695 | HSD17B7 | 51478 |
| ENSG00000143248 | 23.82818427 | 0.013499141 | 0.1336192 | 0.6736096 | NA | RGS5 | 8490 |
| ENSG00000232995 | 13.84332726 | 0.023975468 | 0.1371469 | 0.3594083 | NA | LOC1278142 | 127814295 |
| ENSG00000143228 | 1009.810138 | -0.217638177 | 0.0968966 | 0.005647 | 0.0414338 | NUF2 | 83540 |
| ENSG00000185630 | 12.39578347 | -0.001863146 | 0.1344802 | 0.9357164 | NA | PBX1 | 5087 |
| ENSG00000143198 | 877.664739 | -0.074464541 | 0.0860597 | 0.2719225 | 0.5395487 | MGST3 | 4259 |
| ENSG00000143149 | 1490.072901 | -0.02670413 | 0.0715736 | 0.6612635 | 0.8431632 | ALDH9A1 | 223 |
| ENSG00000143183 | 1482.090088 | -0.080227648 | 0.0753933 | 0.2054278 | 0.4607272 | TMCO1 | 54499 |
| ENSG00000224358 | 12.04534889 | -0.000166371 | 0.1345094 | 0.9920322 | NA | NA | NA |
| ENSG00000143179 | 2254.432382 | 0.005735505 | 0.0645926 | 0.9205562 | 0.9693516 | UCK2 | 7371 |
| ENSG00000143157 | 1401.359664 | -0.087982584 | 0.0799219 | 0.1813074 | 0.4308957 | POGK | 57645 |
| ENSG00000152382 | 315.6560712 | -0.118318335 | 0.1313941 | 0.1392401 | 0.3683114 | TADA1 | 117143 |
| ENSG00000143190 | 889.6439684 | -0.119293759 | 0.0958974 | 0.1043264 | 0.3104017 | POU2F1 | 5451 |
| ENSG00000143162 | 620.9086326 | 0.011670335 | 0.0925675 | 0.8650508 | 0.9457239 | CREG1 | 8804 |
| ENSG00000198771 | 10.12281386 | 1.572731092 | 1.7102063 | 0.0055088 | NA | RCSD1 | 92241 |
| ENSG00000197965 | 3393.432554 | -0.137302486 | 0.0667394 | 0.0192362 | 0.1009503 | MPZL1 | 9019 |
| ENSG00000232194 | 29.65726128 | 0.073752111 | 0.1607967 | 0.0583989 | NA | NA | NA |
| ENSG00000143158 | 2642.494109 | -0.118264243 | 0.0742718 | 0.0575272 | 0.2122584 | MPC2 | 25874 |
| ENSG00000143164 | 1482.447905 | -0.30051143 | 0.0899928 | 0.000123 | 0.0020637 | DCAF6 | 55827 |
| ENSG00000143147 | 205.3017069 | 0.053944365 | 0.1189716 | 0.4297775 | 0.6808939 | GPR161 | 23432 |
| ENSG00000143155 | 714.3730854 | -0.046749317 | 0.087641 | 0.487656 | 0.7249792 | TIPRL | 261726 |
| ENSG00000213064 | 2300.327161 | -0.156674541 | 0.0750533 | 0.0148745 | 0.0836942 | SFT2D2 | 375035 |
| ENSG00000143178 | 32.64465697 | 0.053841287 | 0.1463126 | 0.1587642 | NA | TBX19 | 9095 |
| ENSG00000143153 | 1377.208386 | -0.087325521 | 0.1484493 | 0.1856136 | 0.4358915 | ATP1B1 | 481 |
| ENSG00000143156 | 314.0527263 | -0.046304282 | 0.1078719 | 0.5072206 | 0.7395602 | NME7 | 29922 |
| ENSG00000117475 | 595.3555338 | 0.019472358 | 0.0912861 | 0.7759282 | 0.9056923 | BLZF1 | 8548 |
| ENSG00000117479 | 261.6125332 | -0.009209602 | 0.1061117 | 0.8896097 | 0.9550161 | SLC19A2 | 10560 |
| ENSG00000000460 | 318.986165 | -0.041763926 | 0.1070757 | 0.5469003 | 0.7688461 | FIRRM | 55732 |
| ENSG00000171806 | 71.51377635 | -0.057706093 | 0.1393656 | 0.2865376 | 0.5565423 | METTL18 | 92342 |
| ENSG00000000457 | 177.1510557 | -0.001542553 | 0.1130474 | 0.9794566 | 0.9923699 | SCYL3 | 57147 |
| ENSG00000075945 | 286.4655031 | -0.033876548 | 0.106662 | 0.6207849 | 0.818389 | KIFAP3 | 22920 |
| ENSG00000120370 | 203.0309147 | -0.018861178 | 0.1129826 | 0.7695058 | 0.9015177 | GORAB | 92344 |
| ENSG00000117523 | 6168.289792 | -0.189641965 | 0.0583064 | 0.0003758 | 0.0051862 | PRRC2C | 23215 |
| ENSG00000117533 | 271.4436927 | -0.035762592 | 0.1083862 | 0.6009012 | 0.8072601 | VAMP4 | 8674 |
| ENSG00000010165 | 1379.444602 | 0.173011505 | 0.0846455 | 0.0139241 | 0.0803595 | METTL13 | 51603 |
| ENSG00000197959 | 25.13423542 | 0.019949639 | 0.1341634 | 0.5585137 | NA | DNM3 | 26052 |
| ENSG00000135845 | 368.3247353 | 0.055515167 | 0.1060755 | 0.4359288 | 0.6863128 | PIGC | 5279 |
| ENSG00000094975 | 1057.976316 | -0.008401279 | 0.0787735 | 0.8953596 | 0.9581947 | SUCO | 51430 |
| ENSG00000227177 | 8.65221002 | -0.005775358 | 0.135279 | 0.7739215 | NA | NA | NA |
| ENSG00000120337 | 242.3432061 | 0.000272029 | 0.1107742 | 0.9974949 | 0.998947 | TNFSF18 | 8995 |
| ENSG00000203739 | 13.96669337 | 0.004133562 | 0.1341593 | 0.8757986 | NA | PRDX6-AS1 | 101928673 |
| ENSG00000117592 | 2470.828023 | -0.089136959 | 0.0700188 | 0.1388353 | 0.3676153 | PRDX6 | 9588 |
| ENSG00000076321 | 349.2164435 | 0.095096759 | 0.1179365 | 0.2127513 | 0.4697823 | KLHL20 | 27252 |
| ENSG00000120334 | 694.092939 | -0.080814516 | 0.0928572 | 0.2530721 | 0.5198972 | CENPL | 91687 |
| ENSG00000117593 | 959.2456261 | -0.161097854 | 0.0981149 | 0.0347616 | 0.1520217 | DARS2 | 55157 |

| | | | | | | | | |
|---|---|---|---|---|---|---|---|---|
| ENSG00000234741 | 2864.771617 | 0.467059989 | 0.0809328 | 7.04E-10 | 5.17E-08 | GAS5 | | 60674 |
| ENSG00000208317 | 16.56462317 | -0.027904413 | 0.1377079 | 0.3250185 | NA | NA | NA | |
| ENSG00000185278 | 285.2869238 | -0.093204487 | 0.123328 | 0.2178058 | 0.4771548 | ZBTB37 | | 84614 |
| ENSG00000135870 | 737.5883783 | -0.086549997 | 0.0929488 | 0.222733 | 0.4826983 | RC3H1 | | 149041 |
| ENSG00000152061 | 371.872133 | 0.056371962 | 0.106377 | 0.4288925 | 0.6798607 | RABGAP1L | | 9910 |
| ENSG00000116161 | 3584.663152 | -0.141021657 | 0.065079 | 0.0144229 | 0.0819424 | CACYBP | | 27101 |
| ENSG00000120333 | 528.5176099 | -0.025733611 | 0.0920879 | 0.7062588 | 0.8671822 | MRPS14 | | 63931 |
| ENSG00000235750 | 42.56481707 | -0.026676528 | 0.1327866 | 0.5266192 | 0.7537604 | KIAA0040 | | 9674 |
| ENSG00000143207 | 498.6410057 | -0.097885363 | 0.1130955 | 0.198581 | 0.4518085 | COP1 | | 64326 |
| ENSG00000242193 | 34.38100671 | -0.049295723 | 0.1426883 | 0.2216402 | NA | CRYZL2P | | 730102 |
| ENSG00000224687 | 48.18510145 | 0.06394033 | 0.148033 | 0.1807479 | 0.4299157 | RASAL2-AS | | 100302401 |
| ENSG00000075391 | 1951.712987 | -0.082513581 | 0.0720154 | 0.1782594 | 0.4273908 | RASAL2 | | 9462 |
| ENSG00000116191 | 743.7668942 | -0.162215201 | 0.1093988 | 0.044327 | 0.1788545 | RALGPS2 | | 55103 |
| ENSG00000116199 | 1691.764862 | -0.120077918 | 0.077095 | 0.063695 | 0.2269492 | FAM20B | | 9917 |
| ENSG00000186283 | 905.7399169 | -0.133280623 | 0.0926305 | 0.0669302 | 0.2341648 | TOR3A | | 64222 |
| ENSG00000143322 | 4235.657444 | 0.663798635 | 0.0779315 | 1.13E-18 | 3.07E-16 | ABL2 | | 27 |
| ENSG00000057252 | 1417.97802 | 0.110189426 | 0.0787682 | 0.0927623 | 0.2881032 | SOAT1 | | 6646 |
| ENSG00000169905 | 1644.001926 | -0.015029987 | 0.0723165 | 0.8056044 | 0.9187021 | TOR1AIP2 | | 163590 |
| ENSG00000272906 | 12.01173585 | 0.025763634 | 0.1382019 | 0.2723702 | NA | NA | NA | |
| ENSG00000143337 | 1558.824743 | -0.064975306 | 0.074512 | 0.3004875 | 0.5701745 | TOR1AIP1 | | 26092 |
| ENSG00000135837 | 1410.691058 | -0.151316075 | 0.0862334 | 0.0317071 | 0.1430555 | CEP350 | | 9857 |
| ENSG00000116260 | 1866.081856 | 0.106910196 | 0.0727289 | 0.0853696 | 0.273506 | QSOX1 | | 5768 |
| ENSG00000121454 | 21.51603402 | 0.038184133 | 0.1408036 | 0.2171539 | NA | LHX4 | | 89884 |
| ENSG00000230124 | 92.67858746 | 0.058900026 | 0.1364759 | 0.3124789 | 0.5818701 | ACBD6 | | 84320 |
| ENSG00000135847 | 676.4839825 | -0.043543806 | 0.0906368 | 0.5255613 | 0.7529736 | NA | NA | |
| ENSG00000143324 | 1376.647644 | -0.113384117 | 0.0799037 | 0.086844 | 0.2767116 | XPR1 | | 9213 |
| ENSG00000243155 | 15.75158234 | -0.012586949 | 0.1347782 | 0.6348407 | NA | NA | NA | |
| ENSG00000232586 | 24.45356785 | 0.023330106 | 0.1351776 | 0.4832292 | NA | KIAA1614-A | | 103344928 |
| ENSG00000135823 | 752.2600707 | 0.101552781 | 0.098716 | 0.1676463 | 0.4125292 | STX6 | | 10228 |
| ENSG00000153029 | 39.59989908 | 0.09595791 | 0.1778433 | 0.0507892 | NA | MR1 | | 3140 |
| ENSG00000162783 | 392.4360882 | 0.656254073 | 0.150201 | 7.69E-07 | 2.74E-05 | IER5 | | 51278 |
| ENSG00000272198 | 10.90908491 | 0.001897645 | 0.134652 | 0.936914 | NA | NA | NA | |
| ENSG00000135821 | 2535.103281 | -0.073322709 | 0.0663463 | 0.2053939 | 0.4607272 | GLUL | | 2752 |
| ENSG00000135829 | 7661.435575 | -0.286742773 | 0.0586286 | 1.76E-07 | 7.70E-06 | DHX9 | | 1660 |
| ENSG00000135862 | 5033.666796 | -0.003970948 | 0.0552309 | 0.9577701 | 0.9829665 | LAMC1 | | 3915 |
| ENSG00000058085 | 8533.578175 | 0.732719955 | 0.0674793 | 1.20E-28 | 7.40E-26 | LAMC2 | | 3918 |
| ENSG00000157064 | 53.41862907 | 0.941295852 | 0.4382987 | 0.0010708 | 0.011776 | NMNAT2 | | 23057 |
| ENSG00000116698 | 1917.439393 | 0.037801646 | 0.0670188 | 0.5101087 | 0.741578 | SMG7 | | 9887 |
| ENSG00000162704 | 2916.523138 | -0.06869821 | 0.0650907 | 0.22896 | 0.4902363 | ARPC5 | | 10092 |
| ENSG00000143344 | 93.46213214 | -0.040337771 | 0.1293457 | 0.4680452 | 0.7103207 | RGL1 | | 23179 |
| ENSG00000198756 | 12.42780497 | 0.029593324 | 0.13925 | 0.2188032 | NA | COLGALT2 | | 23127 |
| ENSG00000198860 | 522.5792234 | 0.087334267 | 0.1065804 | 0.2400571 | 0.5054546 | TSEN15 | | 116461 |
| ENSG00000116667 | 52.73624313 | -0.023546521 | 0.1302572 | 0.6066833 | 0.8113886 | C1orf21 | | 81563 |
| ENSG00000116406 | 669.2444854 | 0.093637268 | 0.095959 | 0.1957627 | 0.4484613 | EDEM3 | | 80267 |
| ENSG00000135842 | 1093.508099 | 0.697555192 | 0.093503 | 5.70E-15 | 1.04E-12 | NIBAN1 | | 116496 |
| ENSG00000121481 | 470.878588 | -0.094898762 | 0.1094115 | 0.2073208 | 0.4632201 | RNF2 | | 6045 |
| ENSG00000121486 | 469.9717006 | -0.024607291 | 0.0966609 | 0.7193285 | 0.873166 | TRMT1L | | 81627 |
| ENSG00000116668 | 72.62578972 | -0.03538529 | 0.13031 | 0.4941751 | 0.7297633 | SWT1 | | 54823 |
| ENSG00000116679 | 3971.920626 | 0.109683926 | 0.0648021 | 0.0552263 | 0.206258 | IVNS1ABP | | 10625 |
| ENSG00000143341 | 14.21960878 | 0.02011575 | 0.13621 | 0.4442005 | NA | HMCN1 | | 83872 |
| ENSG00000047410 | 5341.446316 | -0.156319742 | 0.0589207 | 0.0033816 | 0.027991 | TPR | | 7175 |
| ENSG00000157181 | 539.9573804 | 0.248019732 | 0.1301406 | 0.0090668 | 0.0595999 | ODR4 | | 54953 |
| ENSG00000262180 | 16.68768447 | 0.065350571 | 0.1576646 | 0.0246045 | NA | NA | NA | |
| ENSG00000073756 | 30.33435981 | 0.062000515 | 0.1510151 | 0.1178142 | NA | PTGS2 | | 5743 |
| ENSG00000116711 | 91.98479341 | 0.029068324 | 0.126879 | 0.5917662 | 0.8007903 | PLA2G4A | | 5321 |
| ENSG00000116741 | 79.55906596 | 0.15943294 | 0.2400735 | 0.0410611 | 0.1703248 | RGS2 | | 5997 |
| ENSG00000116750 | 1220.966362 | 0.024958393 | 0.0774338 | 0.6967804 | 0.8616368 | UCHL5 | | 51377 |
| ENSG00000116747 | 1239.334797 | -0.064755906 | 0.0774806 | 0.3128382 | 0.5819816 | RO60 | | 6738 |
| ENSG00000272579 | 36.56185939 | 0.04638406 | 0.1408765 | 0.2599028 | NA | NA | NA | |
| ENSG00000023572 | 338.0526099 | 0.06656381 | 0.1141404 | 0.3527619 | 0.6185235 | GLRX2 | | 51022 |
| ENSG00000134371 | 2018.388208 | 0.016576782 | 0.0659859 | 0.7729708 | 0.903811 | CDC73 | | 79577 |
| ENSG00000162687 | 15.55216077 | 0.036979815 | 0.1413863 | 0.1652155 | NA | KCNT2 | | 343450 |
| ENSG00000066279 | 4357.054797 | -0.510397985 | 0.0641761 | 1.56E-16 | 3.31E-14 | ASPM | | 259266 |
| ENSG00000177888 | 538.7008446 | 0.105155389 | 0.1102795 | 0.1734302 | 0.4206475 | ZBTB41 | | 360023 |
| ENSG00000213047 | 215.1196721 | -0.097964431 | 0.135698 | 0.1946037 | 0.4467541 | DENND1B | | 163486 |
| ENSG00000203724 | 16.93908129 | -0.004679194 | 0.1336779 | 0.8663296 | NA | C1orf53 | | 388722 |
| ENSG00000151414 | 1620.637015 | 0.06711302 | 0.0729041 | 0.2784404 | 0.5467586 | NEK7 | | 140609 |
| ENSG00000116833 | 128.5481295 | 0.073006744 | 0.1381961 | 0.2605886 | 0.5279074 | NR5A2 | | 2494 |
| ENSG00000203721 | 19.91436091 | 0.094570074 | 0.1854769 | 0.0079182 | NA | LINC00862 | | 554279 |
| ENSG00000162702 | 2003.17513 | 0.139556308 | 0.0760018 | 0.0306892 | 0.1402507 | ZNF281 | | 23528 |

| | | | | | | | |
|---|---|---|---|---|---|---|---|
| ENSG00000118193 | 2035.134651 | -0.486176973 | 0.0766026 | 1.91E-11 | 1.90E-09 | KIF14 | 9928 |
| ENSG00000118197 | 249.743399 | 0.022923887 | 0.1084574 | 0.7340621 | 0.8827032 | DDX59 | 83479 |
| ENSG00000118200 | 2117.889319 | -0.073036519 | 0.0685866 | 0.2185528 | 0.4783428 | CAMSAP2 | 23271 |
| ENSG00000163362 | 72.2236699 | -0.013260453 | 0.1257517 | 0.7921379 | 0.9130225 | INAVA | 55765 |
| ENSG00000116857 | 724.7535249 | 0.006474628 | 0.0885921 | 0.9244616 | 0.9709924 | TMEM9 | 252839 |
| ENSG00000224818 | 8.975441797 | 0.027123017 | 0.1390446 | 0.1963842 | NA | NA | NA |
| ENSG00000174307 | 199.6419421 | 0.094283119 | 0.1388567 | 0.1983635 | 0.4514094 | PHLDA3 | 23612 |
| ENSG00000159176 | 3957.687172 | 0.36033998 | 0.0685108 | 1.80E-08 | 1.02E-06 | CSRP1 | 1465 |
| ENSG00000223396 | 17.82983685 | 0.026905545 | 0.1371646 | 0.362167 | NA | NA | NA |
| ENSG00000269690 | 25.51743177 | 0.022342993 | 0.1348963 | 0.5033755 | NA | NA | NA |
| ENSG00000134369 | 948.4504122 | 0.201261535 | 0.0986927 | 0.0105528 | 0.0658743 | NAV1 | 89796 |
| ENSG00000231871 | 12.90161428 | 0.029815435 | 0.1391587 | 0.2306696 | NA | IPO9-AS1 | 100873949 |
| ENSG00000198700 | 3387.207163 | -0.041426755 | 0.0620466 | 0.449811 | 0.6966643 | IPO9 | 55705 |
| ENSG00000198892 | 111.6060283 | 0.05678425 | 0.1345443 | 0.3353471 | 0.6029229 | SHISA4 | 149345 |
| ENSG00000134375 | 2712.057645 | -0.122922824 | 0.0681093 | 0.0384946 | 0.1634859 | TIMM17A | 10440 |
| ENSG00000176393 | 1863.823458 | -0.071295101 | 0.0695393 | 0.233626 | 0.496089 | RNPEP | 6051 |
| ENSG00000273478 | 22.24088486 | -0.013483555 | 0.1337606 | 0.6645075 | NA | NA | NA |
| ENSG00000234678 | 11.40457254 | 0.009723771 | 0.1351059 | 0.6787474 | NA | NA | NA |
| ENSG00000163435 | 143.7212492 | 0.010315612 | 0.1172831 | 0.8663425 | 0.9459476 | ELF3 | 1999 |
| ENSG00000143862 | 408.9635507 | 0.048410805 | 0.1013164 | 0.4913718 | 0.7279283 | ARL8A | 127829 |
| ENSG00000077152 | 921.4983396 | 0.20299132 | 0.0980713 | 0.0097247 | 0.0625863 | UBE2T | 29089 |
| ENSG00000077157 | 389.9288037 | 0.037910042 | 0.104979 | 0.585584 | 0.7967626 | PPP1R12B | 4660 |
| ENSG00000236439 | 14.6069565 | -0.027690225 | 0.1380527 | 0.2986662 | NA | NA | NA |
| ENSG00000117139 | 592.5270205 | 0.091745621 | 0.1049034 | 0.2184899 | 0.4783119 | KDM5B | 10765 |
| ENSG00000214796 | 23.05379361 | -0.000910984 | 0.1325898 | 0.9758258 | NA | NA | NA |
| ENSG00000183155 | 526.158708 | -0.005731157 | 0.0913574 | 0.9248576 | 0.9709924 | RABIF | 5877 |
| ENSG00000117153 | 815.2249994 | -0.021360752 | 0.0824309 | 0.7482796 | 0.8907219 | KLHL12 | 59349 |
| ENSG00000159346 | 1674.498588 | 0.060981565 | 0.0711322 | 0.3166735 | 0.5852949 | ADIPOR1 | 51094 |
| ENSG00000159348 | 421.7423492 | 0.079007139 | 0.1088385 | 0.283937 | 0.5531631 | CYB5R1 | 51706 |
| ENSG00000163444 | 1086.135028 | -0.231815508 | 0.0993711 | 0.0040501 | 0.0321613 | TMEM183A | 92703 |
| ENSG00000143847 | 28.44621876 | 0.005377827 | 0.1317947 | 0.8810233 | NA | PPFIA4 | 8497 |
| ENSG00000163485 | 128.0687246 | -0.768839463 | 0.2838021 | 0.0002839 | 0.0041251 | ADORA1 | 134 |
| ENSG00000159388 | 96.37823673 | -7.95E-05 | 0.1221736 | 0.9971582 | 0.998947 | BTG2 | 7832 |
| ENSG00000058668 | 124.6423807 | 0.822080099 | 0.2852069 | 0.0001688 | 0.0026564 | ATP2B4 | 493 |
| ENSG00000058673 | 4159.225233 | -0.013250261 | 0.060965 | 0.8075001 | 0.9200093 | ZC3H11A | 9877 |
| ENSG00000257315 | 135.5318069 | 0.081387069 | 0.1403347 | 0.2292811 | 0.4903399 | ZBED6 | 100381270 |
| ENSG00000182004 | 1539.354091 | -0.069964824 | 0.0734466 | 0.2606329 | 0.5279074 | SNRPE | 6635 |
| ENSG00000143842 | 390.6150141 | 0.350865128 | 0.1568654 | 0.0023214 | 0.0212342 | SOX13 | 9580 |
| ENSG00000143845 | 241.3089148 | 0.011163603 | 0.1078453 | 0.865658 | 0.945819 | ETNK2 | 55224 |
| ENSG00000174567 | 8.693846872 | 0.037309357 | 0.142856 | 0.0662774 | NA | GOLT1A | 127845 |
| ENSG00000158615 | 1852.789463 | -0.009856179 | 0.068786 | 0.8662945 | 0.9459476 | PPP1R15B | 84919 |
| ENSG00000133056 | 243.9913263 | -0.148711186 | 0.1551858 | 0.0841622 | 0.2709755 | PIK3C2B | 5287 |
| ENSG00000198625 | 627.5921085 | 0.180010303 | 0.1162348 | 0.0329333 | 0.1464825 | MDM4 | 4194 |
| ENSG00000174529 | 50.30522605 | -0.035143373 | 0.1340347 | 0.4373591 | 0.6876822 | TMEM81 | 388730 |
| ENSG00000117222 | 958.3584425 | 0.249295979 | 0.1055766 | 0.0032798 | 0.027377 | RBBP5 | 5929 |
| ENSG00000133059 | 565.8377122 | -0.017612563 | 0.0960569 | 0.796329 | 0.9152729 | DSTYK | 25778 |
| ENSG00000133069 | 122.9989535 | 0.462514327 | 0.2919203 | 0.0050294 | 0.0381729 | TMCC2 | 9911 |
| ENSG00000163545 | 2052.086073 | -0.062599316 | 0.08017 | 0.3383265 | 0.6058756 | NUAK2 | 81788 |
| ENSG00000158711 | 1298.702819 | -0.001848382 | 0.0737144 | 0.975453 | 0.9908057 | ELK4 | 2005 |
| ENSG00000158715 | 205.9275752 | 0.094926329 | 0.1380914 | 0.1971056 | 0.4503823 | SLC45A3 | 85414 |
| ENSG00000069275 | 7725.072182 | -0.3175185 | 0.0577118 | 5.69E-09 | 3.61E-07 | NUCKS1 | 64710 |
| ENSG00000268313 | 126.5063367 | -0.063640986 | 0.1361803 | 0.2984288 | 0.56867 | NA | NA |
| ENSG00000117280 | 914.4342569 | 0.245794408 | 0.1012406 | 0.0028468 | 0.0247608 | RAB29 | 8934 |
| ENSG00000133065 | 1403.928654 | 0.236977111 | 0.0906918 | 0.0018741 | 0.0180445 | SLC41A1 | 254428 |
| ENSG00000229874 | 43.03888669 | -0.019599506 | 0.1312309 | 0.6384192 | 0.8297034 | NA | NA |
| ENSG00000196550 | 243.1133121 | 0.042008443 | 0.112253 | 0.5397997 | 0.7633657 | FAM72A | 729533 |
| ENSG00000196533 | 246.6513242 | -0.557741396 | 0.1940471 | 0.0002281 | 0.003425 | NA | NA |
| ENSG00000163486 | 1387.91937 | -0.006467176 | 0.0717389 | 0.914811 | 0.9662935 | NA | NA |
| ENSG00000143466 | 246.9918114 | -0.118748381 | 0.1397869 | 0.1369246 | 0.364664 | NA | NA |
| ENSG00000143486 | 569.298963 | -0.076415437 | 0.0977286 | 0.2878075 | 0.5576181 | EIF2D | 1939 |
| ENSG00000143479 | 188.3835303 | 0.140518967 | 0.169764 | 0.0905245 | 0.2843283 | DYRK3 | 8444 |
| ENSG00000162889 | 1354.082778 | -0.094589524 | 0.0778701 | 0.1445279 | 0.3769212 | MAPKAPK2 | 9261 |
| ENSG00000180667 | 536.7534434 | 0.098660386 | 0.1042674 | 0.1854757 | 0.4358363 | YOD1 | 55432 |
| ENSG00000123836 | 878.5198101 | 0.100807308 | 0.0935687 | 0.1606647 | 0.4022128 | PFKFB2 | 5208 |
| ENSG00000196352 | 302.3547385 | 0.671984461 | 0.171474 | 5.13E-06 | 0.0001431 | CD55 | 1604 |
| ENSG00000117335 | 2722.020324 | -0.101362735 | 0.0660286 | 0.0804182 | 0.2631274 | CD46 | 4179 |
| ENSG00000203709 | 34.38987317 | -0.031043999 | 0.1355117 | 0.4212928 | NA | NA | NA |
| ENSG00000076356 | 64.81962396 | 0.451811217 | 0.536459 | 0.0093697 | 0.0611778 | PLXNA2 | 5362 |
| ENSG00000196878 | 1178.736248 | 0.966265896 | 0.0921364 | 6.03E-27 | 3.36E-24 | LAMB3 | 3914 |
| ENSG00000162757 | 35.13930264 | 0.032065273 | 0.1354806 | 0.4206821 | NA | C1orf74 | 148304 |

| | | | | | | | | |
|---|---|---|---|---|---|---|---|---|
| ENSG00000117597 | 1076.471958 | 0.132922917 | 0.0929914 | 0.06834 | 0.2368078 | UTP25 | | 27042 |
| ENSG00000203706 | 46.11150292 | -0.023050411 | 0.1313003 | 0.5952333 | 0.8032486 | NA | NA | |
| ENSG00000082497 | 153.0600273 | 0.031276309 | 0.1205587 | 0.61217 | 0.8144372 | SERTAD4 | | 56256 |
| ENSG00000054392 | 79.03802758 | -0.008773862 | 0.1247281 | 0.8733524 | 0.948724 | HHAT | | 55733 |
| ENSG00000117625 | 485.8181127 | 0.03439666 | 0.0969312 | 0.6198513 | 0.8182262 | RCOR3 | | 55758 |
| ENSG00000082512 | 273.7931064 | 0.070362314 | 0.1193867 | 0.3269737 | 0.5951971 | TRAF5 | | 7188 |
| ENSG00000153363 | 73.86161331 | 0.060038817 | 0.1401243 | 0.2754647 | 0.5438984 | LINC00467 | | 84791 |
| ENSG00000170385 | 683.2631728 | -0.062671057 | 0.0924533 | 0.3744758 | 0.6359772 | SLC30A1 | | 7779 |
| ENSG00000117650 | 934.3579798 | -0.255265946 | 0.1006391 | 0.0019712 | 0.0186743 | NEK2 | | 4751 |
| ENSG00000123684 | 2285.23549 | -0.139147014 | 0.0706841 | 0.0230098 | 0.1142761 | LPGAT1 | | 9926 |
| ENSG00000143493 | 1266.815633 | 0.077397925 | 0.0809458 | 0.2410756 | 0.5063844 | INTS7 | | 25896 |
| ENSG00000143476 | 2507.912857 | 0.770421065 | 0.0809221 | 1.09E-22 | 3.98E-20 | DTL | | 51514 |
| ENSG00000220749 | 39.26416025 | -0.047550721 | 0.1411749 | 0.2537651 | NA | RPL21P28 | | 100131205 |
| ENSG00000066027 | 1010.863576 | -0.151070616 | 0.0919473 | 0.0388649 | 0.1645453 | PPP2R5A | | 5525 |
| ENSG00000065600 | 337.4767056 | 0.112494207 | 0.1246341 | 0.1551742 | 0.3936135 | PACC1 | | 55248 |
| ENSG00000117691 | 634.169532 | -0.015051844 | 0.0876352 | 0.8222909 | 0.9271409 | NENF | | 29937 |
| ENSG00000162772 | 216.5069338 | 2.297750923 | 0.2094329 | 3.39E-29 | 2.20E-26 | ATF3 | | 467 |
| ENSG00000123685 | 261.9911315 | 0.080121963 | 0.1221907 | 0.2734438 | 0.5416478 | BATF3 | | 55509 |
| ENSG00000117697 | 384.8073481 | -0.103735339 | 0.1165935 | 0.178779 | 0.4275952 | NSL1 | | 25936 |
| ENSG00000203705 | 175.9141972 | 0.036796031 | 0.1213582 | 0.5554664 | 0.7753828 | TATDN3 | | 128387 |
| ENSG00000198468 | 115.9417659 | 0.114329781 | 0.169868 | 0.1094652 | 0.3202262 | FLVCR1-DT | | 642946 |
| ENSG00000162769 | 634.5853135 | 0.039828138 | 0.0897392 | 0.5629561 | 0.7805304 | FLVCR1 | | 28982 |
| ENSG00000143494 | 38.92595518 | 0.00492546 | 0.1300287 | 0.9064178 | NA | VASH2 | | 79805 |
| ENSG00000174606 | 634.0483902 | -0.016806685 | 0.0880795 | 0.8020631 | 0.9173383 | ANGEL2 | | 90806 |
| ENSG00000136643 | 410.223593 | -0.307528962 | 0.1479048 | 0.0041171 | 0.032627 | RPS6KC1 | | 26750 |
| ENSG00000143499 | 919.5690571 | -0.233115904 | 0.1001192 | 0.0040224 | 0.0320066 | SMYD2 | | 56950 |
| ENSG00000228470 | 25.0359798 | 0.002885216 | 0.1325276 | 0.9311658 | NA | NA | NA | |
| ENSG00000152104 | 6042.239598 | -0.265279062 | 0.145175 | 0.0090011 | 0.0592685 | PTPN14 | | 5784 |
| ENSG00000117724 | 7515.60098 | -0.368050206 | 0.0567365 | 1.08E-11 | 1.10E-09 | CENPF | | 1063 |
| ENSG00000136636 | 1187.73944 | -0.297833187 | 0.0993487 | 0.0003872 | 0.0053027 | KCTD3 | | 51133 |
| ENSG00000092978 | 385.5667049 | 0.059416952 | 0.1045455 | 0.4063272 | 0.6626146 | GPATCH2 | | 55105 |
| ENSG00000067533 | 1040.076331 | 0.078613688 | 0.0894086 | 0.2586067 | 0.5253712 | RRP15 | | 51018 |
| ENSG00000092969 | 148.9780526 | 0.561681463 | 0.2490609 | 0.0011655 | 0.0126499 | TGFB2 | | 7042 |
| ENSG00000143353 | 141.2599217 | -0.059054006 | 0.128732 | 0.3637481 | 0.6265951 | LYPLAL1 | | 127018 |
| ENSG00000136628 | 5685.230005 | 0.030342938 | 0.0549487 | 0.5575879 | 0.7767662 | EPRS1 | | 2058 |
| ENSG00000162813 | 570.4921492 | -0.041348118 | 0.0919874 | 0.547809 | 0.7691852 | BPNT1 | | 10380 |
| ENSG00000067704 | 2852.86949 | -0.255357989 | 0.0688812 | 4.24E-05 | 0.0008559 | IARS2 | | 55699 |
| ENSG00000118873 | 1397.341537 | 0.02404529 | 0.0748193 | 0.7024642 | 0.8650963 | RAB3GAP2 | | 25782 |
| ENSG00000116141 | 115.4872366 | 0.099321606 | 0.1558492 | 0.1512858 | 0.3877924 | MARK1 | | 4139 |
| ENSG00000162817 | 319.7237721 | -0.125018464 | 0.133164 | 0.1236209 | 0.3446338 | C1orf115 | | 79762 |
| ENSG00000117791 | 153.2575231 | 0.032689249 | 0.1197034 | 0.6021211 | 0.8079281 | MTARC2 | | 54996 |
| ENSG00000186205 | 16.60181386 | -0.024036075 | 0.1366762 | 0.3921638 | NA | MTARC1 | | 64757 |
| ENSG00000136630 | 68.54982185 | 0.033361235 | 0.1301935 | 0.5145236 | 0.7447522 | HLX | | 3142 |
| ENSG00000143507 | 366.4364423 | 0.142689841 | 0.1311203 | 0.0891403 | 0.2815707 | DUSP10 | | 11221 |
| ENSG00000143498 | 285.3914267 | 0.063830994 | 0.1136606 | 0.372117 | 0.6339991 | TAF1A | | 9015 |
| ENSG00000225265 | 11.98502041 | 0.020668042 | 0.1368116 | 0.387418 | NA | TAF1A-AS1 | | 100506161 |
| ENSG00000154305 | 1037.786341 | -0.11062128 | 0.0899367 | 0.1178875 | 0.3345134 | MIA3 | | 375056 |
| ENSG00000272750 | 20.07142643 | -0.008841984 | 0.1334256 | 0.7694782 | NA | NA | NA | |
| ENSG00000186063 | 2473.376345 | -0.137191647 | 0.0697929 | 0.0237836 | 0.1168136 | AIDA | | 64853 |
| ENSG00000162819 | 1094.367445 | 0.114838509 | 0.0883859 | 0.1028556 | 0.3074144 | BROX | | 148362 |
| ENSG00000228106 | 61.78568516 | -0.034854629 | 0.1325177 | 0.4662952 | 0.7094916 | NA | NA | |
| ENSG00000154309 | 60.09348161 | 0.018593582 | 0.1286382 | 0.6947099 | 0.8605415 | DISP1 | | 84976 |
| ENSG00000162909 | 10104.44119 | -0.097720259 | 0.0541157 | 0.0484351 | 0.1885902 | CAPN2 | | 824 |
| ENSG00000143514 | 2030.904939 | 0.20529989 | 0.0821286 | 0.0033406 | 0.0277928 | TP53BP2 | | 7159 |
| ENSG00000272645 | 44.65417966 | 0.041672045 | 0.1371702 | 0.3481259 | 0.6140832 | NA | NA | |
| ENSG00000232372 | 17.58085439 | 0.025920913 | 0.1372597 | 0.3553605 | NA | NA | NA | |
| ENSG00000143756 | 1360.196815 | 0.076943735 | 0.0784407 | 0.2359695 | 0.5000653 | FBXO28 | | 23219 |
| ENSG00000143753 | 1891.573268 | -0.009074132 | 0.0668426 | 0.8756255 | 0.9496918 | DEGS1 | | 8560 |
| ENSG00000143748 | 634.2838292 | 0.002014141 | 0.0881804 | 0.9772195 | 0.9913391 | NVL | | 4931 |
| ENSG00000143771 | 870.8639852 | -0.018817543 | 0.0814363 | 0.7729522 | 0.903811 | CNIH4 | | 29097 |
| ENSG00000162923 | 2744.394367 | -0.081782166 | 0.0665764 | 0.1590417 | 0.3995197 | WDR26 | | 80232 |
| ENSG00000143786 | 11.14342351 | 0.012653661 | 0.1355948 | 0.57614 | NA | CNIH3 | | 149111 |
| ENSG00000185842 | 312.251751 | 0.338767675 | 0.1721369 | 0.0043409 | 0.0339397 | DNAH14 | | 127602 |
| ENSG00000143815 | 2489.444117 | -0.355850672 | 0.071444 | 7.82E-08 | 3.87E-06 | LBR | | 3930 |
| ENSG00000154380 | 4161.296183 | 0.097639926 | 0.0602745 | 0.070995 | 0.2426962 | ENAH | | 55740 |
| ENSG00000227496 | 8.888389247 | 0.021595011 | 0.1375408 | 0.3054729 | NA | LOC1027238 | | 102723834 |
| ENSG00000143742 | 4987.917424 | -0.092187879 | 0.058126 | 0.0789912 | 0.2599144 | SRP9 | | 6726 |
| ENSG00000143819 | 747.5145141 | 0.019643372 | 0.0841286 | 0.7681869 | 0.9005958 | EPHX1 | | 2052 |
| ENSG00000242861 | 32.10630007 | -0.005906436 | 0.1311253 | 0.8728159 | NA | NA | NA | |
| ENSG00000196187 | 856.6099094 | -0.046893033 | 0.085943 | 0.4775819 | 0.7167198 | TMEM63A | | 9725 |

| | | | | | | | |
|---|---|---|---|---|---|---|---|
| ENSG00000143811 | 498.5663682 | 0.215659076 | 0.1416892 | 0.0232213 | 0.1149488 | PYCR2 | 29920 |
| ENSG00000143751 | 1260.596005 | 0.405637169 | 0.0898901 | 6.50E-07 | 2.37E-05 | SDE2 | 163859 |
| ENSG00000163041 | 2558.94154 | -0.202075336 | 0.0752952 | 0.0020501 | 0.0192081 | H3-3A | 3020 |
| ENSG00000182827 | 1362.386771 | 0.024304789 | 0.0725434 | 0.6938202 | 0.8600779 | ACBD3 | 64746 |
| ENSG00000183814 | 495.2851189 | -0.035382543 | 0.0952058 | 0.6080311 | 0.8120753 | LIN9 | 286826 |
| ENSG00000143799 | 6806.461137 | -0.073169039 | 0.0562529 | 0.147996 | 0.3820469 | PARP1 | 142 |
| ENSG00000143772 | 320.476013 | 0.056445448 | 0.109556 | 0.4274223 | 0.6791494 | ITPKB | 3707 |
| ENSG00000143801 | 351.6500192 | -0.020527612 | 0.1045551 | 0.7613757 | 0.897621 | PSEN2 | 5664 |
| ENSG00000163050 | 559.9882337 | 0.037568811 | 0.0933579 | 0.5868894 | 0.7974241 | COQ8A | 56997 |
| ENSG00000143776 | 3326.904376 | -0.127160699 | 0.0690037 | 0.0343447 | 0.1507632 | CDC42BPA | 8476 |
| ENSG00000233706 | 8.444586864 | -0.009779193 | 0.1357517 | 0.6155268 | NA | NA | NA |
| ENSG00000181450 | 399.5759333 | 0.156368329 | 0.1361784 | 0.0702779 | 0.2411841 | ZNF678 | 339500 |
| ENSG00000143740 | 674.1826491 | -0.035252687 | 0.088373 | 0.6063801 | 0.8112617 | SNAP47 | 116841 |
| ENSG00000081692 | 332.0425831 | 0.290274016 | 0.1649422 | 0.0085041 | 0.0566668 | JMJD4 | 65094 |
| ENSG00000143816 | 60.59988735 | 0.103297783 | 0.1801406 | 0.0664699 | 0.2331637 | WNT9A | 7483 |
| ENSG00000143761 | 6118.4888 | -0.092313633 | 0.0636678 | 0.1011407 | 0.3042557 | ARF1 | 375 |
| ENSG00000143793 | 545.6126142 | -0.06226491 | 0.0972978 | 0.3787289 | 0.6400365 | C1orf35 | 79169 |
| ENSG00000162910 | 413.1916975 | 0.036261542 | 0.1024574 | 0.59857 | 0.8058894 | MRPL55 | 128308 |
| ENSG00000143774 | 2011.482613 | -0.016930879 | 0.066679 | 0.7767045 | 0.9063622 | GUK1 | 2987 |
| ENSG00000198835 | 12.52888455 | 0.002642628 | 0.1346872 | 0.9111516 | NA | GJC2 | 57165 |
| ENSG00000181873 | 310.6286182 | -0.01149349 | 0.1025223 | 0.8646537 | 0.9456272 | IBA57 | 200205 |
| ENSG00000162913 | 11.54518805 | 0.001239261 | 0.1345335 | 0.9605571 | NA | OBSCN-AS1 | 574407 |
| ENSG00000154358 | 152.4328476 | -0.075453811 | 0.133784 | 0.2693012 | 0.5372685 | OBSCN | 84033 |
| ENSG00000154370 | 1001.292884 | 0.003025251 | 0.078362 | 0.9632323 | 0.9851357 | TRIM11 | 81559 |
| ENSG00000181218 | 242.1571379 | -0.047821215 | 0.1175276 | 0.4765563 | 0.7159683 | H2AC25 | 92815 |
| ENSG00000168159 | 1994.232294 | 0.099170824 | 0.0708024 | 0.1036083 | 0.3087368 | RNF187 | 149603 |
| ENSG00000183929 | 31.20039604 | -0.023337687 | 0.1339651 | 0.5249434 | NA | NA | NA |
| ENSG00000116574 | 57.41315525 | -0.002430182 | 0.1271303 | 0.9567964 | 0.982546 | RHOU | 58480 |
| ENSG00000177788 | 12.04891792 | 0.010399302 | 0.1349997 | 0.6703766 | NA | RAB4A-AS1 | 105373159 |
| ENSG00000168118 | 496.4956054 | -0.117954009 | 0.1152391 | 0.1328957 | 0.3592582 | RAB4A | 5867 |
| ENSG00000154429 | 394.2555545 | -0.275178895 | 0.1572557 | 0.0095358 | 0.0617281 | CCSAP | 126731 |
| ENSG00000226920 | 29.73748919 | -0.029266151 | 0.1357745 | 0.4087152 | NA | NA | NA |
| ENSG00000069248 | 1779.78348 | -0.112172341 | 0.0738043 | 0.0739806 | 0.2491832 | NUP133 | 55746 |
| ENSG00000135776 | 772.487902 | -0.097963907 | 0.0944034 | 0.1731671 | 0.4203584 | ABCB10 | 23456 |
| ENSG00000135801 | 1339.048223 | -0.182392713 | 0.0879217 | 0.0117315 | 0.0712612 | TAF5L | 27097 |
| ENSG00000135763 | 1633.664592 | 0.047790447 | 0.0701396 | 0.4281559 | 0.6795115 | URB2 | 9816 |
| ENSG00000143641 | 2207.803487 | -0.135977972 | 0.0719462 | 0.0283191 | 0.1325831 | GALNT2 | 2590 |
| ENSG00000224407 | 15.90893403 | -0.031244621 | 0.1391148 | 0.2462169 | NA | NA | NA |
| ENSG00000135775 | 470.9137962 | -0.190085529 | 0.1290166 | 0.032569 | 0.1456504 | COG2 | 22796 |
| ENSG00000119280 | 664.0167959 | -0.127941536 | 0.1021284 | 0.0917751 | 0.2868223 | C1orf198 | 84886 |
| ENSG00000143643 | 251.7238742 | -0.106229678 | 0.1336782 | 0.1704336 | 0.4162313 | TTC13 | 79573 |
| ENSG00000173409 | 166.2690282 | -0.088165473 | 0.1387492 | 0.214956 | 0.473041 | ARV1 | 64801 |
| ENSG00000182118 | 176.5602128 | 0.061519163 | 0.1246119 | 0.3651512 | 0.6276526 | FAM89A | 375061 |
| ENSG00000143633 | 240.5857259 | 0.036942692 | 0.1133899 | 0.5821364 | 0.7935506 | C1orf131 | 128061 |
| ENSG00000116906 | 1012.265502 | -0.139180279 | 0.098884 | 0.0657331 | 0.2317621 | GNPAT | 8443 |
| ENSG00000116903 | 682.3970462 | 0.090030672 | 0.0946912 | 0.207775 | 0.4639686 | EXOC8 | 149371 |
| ENSG00000010072 | 544.7697209 | -0.05320399 | 0.0955234 | 0.4469194 | 0.6944817 | SPRTN | 83932 |
| ENSG00000135766 | 1626.721732 | 0.008321698 | 0.0761354 | 0.8961614 | 0.9584381 | EGLN1 | 54583 |
| ENSG00000233461 | 82.59868614 | -0.06515923 | 0.141946 | 0.2498356 | 0.5160618 | LOC1225267 | 122526782 |
| ENSG00000116918 | 532.6192769 | -0.054885678 | 0.1008672 | 0.4375871 | 0.6879482 | TSNAX | 7257 |
| ENSG00000116991 | 97.4586801 | 0.078555556 | 0.1461065 | 0.208445 | 0.4650913 | SIPA1L2 | 57568 |
| ENSG00000135778 | 478.8238577 | 0.044827276 | 0.0982931 | 0.5229661 | 0.7505426 | NTPCR | 84284 |
| ENSG00000135749 | 277.6962952 | 0.198036362 | 0.1649704 | 0.040046 | 0.1678126 | PCNX2 | 80003 |
| ENSG00000143674 | 274.8885996 | -0.111847376 | 0.1363784 | 0.1542535 | 0.3924711 | MAP3K21 | 84451 |
| ENSG00000231663 | 8.65344244 | 0.008370059 | 0.1356415 | 0.6673887 | NA | COA6-AS1 | 101927765 |
| ENSG00000168275 | 585.6959541 | 0.066396364 | 0.0967695 | 0.3262733 | 0.5943852 | COA6 | 388753 |
| ENSG00000059588 | 390.6643806 | -0.06022989 | 0.1087222 | 0.3988375 | 0.6577504 | TARBP1 | 6894 |
| ENSG00000168264 | 815.4544113 | 0.366807996 | 0.1060876 | 5.86E-05 | 0.0011293 | IRF2BP2 | 359948 |
| ENSG00000173726 | 2678.788905 | 0.042481039 | 0.069608 | 0.4855022 | 0.7234177 | TOMM20 | 9804 |
| ENSG00000188739 | 22.06897568 | -0.023462676 | 0.1355185 | 0.4625868 | NA | RBM34 | 23029 |
| ENSG00000054267 | 1054.118909 | -0.01096732 | 0.0791392 | 0.8642003 | 0.9455189 | ARID4B | 51742 |
| ENSG00000152904 | 503.6210354 | -0.093063242 | 0.1064874 | 0.2126492 | 0.4697082 | GGPS1 | 9453 |
| ENSG00000116957 | 484.9612847 | -0.207967737 | 0.1303995 | 0.0222974 | 0.1117358 | NA | NA |
| ENSG00000162885 | 412.6248843 | -0.021594256 | 0.0978688 | 0.7524451 | 0.8932431 | B3GALNT2 | 148789 |
| ENSG00000168243 | 83.07002059 | 0.093444554 | 0.160229 | 0.1365088 | 0.3638055 | GNG4 | 2786 |
| ENSG00000143669 | 1097.891488 | 0.238815837 | 0.0938161 | 0.0022198 | 0.0204734 | LYST | 1130 |
| ENSG00000116962 | 464.0960224 | -0.236964293 | 0.1353297 | 0.0132603 | 0.0774468 | NID1 | 4811 |
| ENSG00000077585 | 100.3854982 | 0.014222379 | 0.122734 | 0.7988643 | 0.9162772 | GPR137B | 7107 |
| ENSG00000086619 | 359.1174544 | -0.029362246 | 0.1019346 | 0.6693879 | 0.848177 | ERO1B | 56605 |
| ENSG00000116977 | 812.2588723 | 0.010040866 | 0.0843986 | 0.8710197 | 0.9479531 | LGALS8 | 3964 |

| | | | | | | | | |
|---|---|---|---|---|---|---|---|---|
| ENSG00000273058 | 19.46341185 | 0.006514602 | 0.1333287 | 0.8310332 | NA | NA | NA | |
| ENSG00000230325 | 9.13366199 | 0.015771168 | 0.1362894 | 0.4620744 | NA | NA | NA | |
| ENSG00000119285 | 3429.208589 | -0.05084495 | 0.0611987 | 0.3520146 | 0.6178637 | HEATR1 | | 55127 |
| ENSG00000116984 | 1187.218443 | 0.057739168 | 0.080118 | 0.3797384 | 0.6409076 | MTR | | 4548 |
| ENSG00000215808 | 34.95037974 | -0.01375916 | 0.1314864 | 0.7206666 | NA | LINC01139 | | 339535 |
| ENSG00000091483 | 1374.874074 | -0.117626514 | 0.0794766 | 0.0750004 | 0.2513858 | FH | | 2271 |
| ENSG00000054277 | 70.25655443 | 0.02494873 | 0.1283695 | 0.6187967 | 0.8177581 | OPN3 | | 23596 |
| ENSG00000203668 | 1086.501185 | -0.145334753 | 0.0918971 | 0.0461861 | 0.1839834 | CHML | | 1122 |
| ENSG00000174371 | 1320.441028 | 0.516484319 | 0.0886478 | 4.57E-10 | 3.42E-08 | EXO1 | | 9156 |
| ENSG00000214837 | 12.22611591 | 0.004688183 | 0.1347036 | 0.8429092 | NA | NA | NA | |
| ENSG00000143702 | 1829.518267 | -0.068185514 | 0.072462 | 0.2681386 | 0.5361059 | CEP170 | | 9859 |
| ENSG00000232085 | 11.54469207 | 0.018847992 | 0.1366994 | 0.4025857 | NA | NA | NA | |
| ENSG00000054282 | 189.4202864 | 0.1741255 | 0.1841137 | 0.0581182 | 0.2135512 | SDCCAG8 | | 10806 |
| ENSG00000117020 | 674.6574136 | -0.092278099 | 0.0975153 | 0.2043267 | 0.4594559 | AKT3 | | 10000 |
| ENSG00000179456 | 503.6690096 | -0.049811896 | 0.0998842 | 0.4787066 | 0.7178141 | ZBTB18 | | 10472 |
| ENSG00000035687 | 1262.729947 | -0.031926857 | 0.0744386 | 0.6088417 | 0.8123066 | ADSS2 | | 159 |
| ENSG00000121644 | 1914.149428 | -0.017633398 | 0.0669417 | 0.7618076 | 0.8978584 | DESI2 | | 51029 |
| ENSG00000203667 | 825.7875684 | -0.356737867 | 0.1095197 | 0.0001214 | 0.0020408 | COX20 | | 116228 |
| ENSG00000188206 | 430.6873433 | -0.007111506 | 0.1047395 | 0.9155611 | 0.9664559 | NA | NA | |
| ENSG00000153187 | 14947.10499 | -0.167070173 | 0.0541597 | 0.0008276 | 0.0095618 | HNRNPU | | 3192 |
| ENSG00000272195 | 14.25038923 | 0.01642738 | 0.1355891 | 0.5243162 | NA | NA | NA | |
| ENSG00000203666 | 68.88119227 | 0.068279356 | 0.1457727 | 0.2148963 | 0.4729986 | EFCAB2 | | 84288 |
| ENSG00000185420 | 308.9836378 | -0.018587856 | 0.1046173 | 0.7832292 | 0.9096561 | SMYD3 | | 64754 |
| ENSG00000162851 | 730.0053394 | -0.030127704 | 0.0857558 | 0.6524541 | 0.8389824 | TFB2M | | 64216 |
| ENSG00000162852 | 456.7059576 | 0.028389733 | 0.0971922 | 0.6810997 | 0.8538613 | CNST | | 163882 |
| ENSG00000143653 | 978.2578009 | -0.023113037 | 0.0806572 | 0.7222679 | 0.8744623 | SCCPDH | | 51097 |
| ENSG00000260855 | 16.12087511 | -0.02045436 | 0.1364196 | 0.4203687 | NA | NA | NA | |
| ENSG00000227953 | 9.328212986 | 0.020273896 | 0.1371916 | 0.3418412 | NA | NA | NA | |
| ENSG00000153207 | 3877.350908 | -0.005266918 | 0.0603436 | 0.9217586 | 0.9699282 | AHCTF1 | | 25909 |
| ENSG00000135747 | 209.6802446 | 0.576001227 | 0.2029529 | 0.0002515 | 0.0037239 | ZNF670-ZNI | | 100533111 |
| ENSG00000197472 | 112.2387634 | 0.405203144 | 0.3567963 | 0.0095473 | 0.0617509 | ZNF695 | | 57116 |
| ENSG00000188295 | 150.7200132 | 0.061624124 | 0.1300064 | 0.3424691 | 0.6089788 | ZNF669 | | 79862 |
| ENSG00000196418 | 205.5435302 | 0.053471476 | 0.1184436 | 0.4345139 | 0.6852379 | ZNF124 | | 7678 |
| ENSG00000259865 | 105.6892288 | -0.013585533 | 0.1215197 | 0.8090346 | 0.9207262 | NA | NA | |
| ENSG00000227671 | 389.0121731 | -0.017398214 | 0.0985947 | 0.7988739 | 0.9162772 | NA | NA | |
| ENSG00000162714 | 995.6012642 | 0.091125489 | 0.0883048 | 0.1906728 | 0.4413891 | ZNF496 | | 84838 |
| ENSG00000175137 | 910.4551896 | 0.45949727 | 0.1105736 | 2.73E-06 | 8.11E-05 | SH3BP5L | | 80851 |
| ENSG00000171161 | 684.3387945 | 0.287831164 | 0.1173468 | 0.0019657 | 0.0186675 | ZNF672 | | 79894 |
| ENSG00000171163 | 515.2163888 | -0.087266502 | 0.1049277 | 0.2379403 | 0.5025095 | ZNF692 | | 55657 |
| ENSG00000185220 | 151.1343872 | 0.030050787 | 0.1194871 | 0.6293747 | 0.8235526 | PGBD2 | | 267002 |
| ENSG00000184731 | 23.28666411 | 0.02220328 | 0.1350798 | 0.4958061 | NA | FAM110C | | 642273 |
| ENSG00000035115 | 173.7926241 | 0.120571835 | 0.1558879 | 0.1240377 | 0.3453836 | SH3YL1 | | 26751 |
| ENSG00000143727 | 1255.573925 | 0.022346975 | 0.073675 | 0.7197237 | 0.8733734 | ACP1 | | 52 |
| ENSG00000151353 | 490.6345459 | 0.125037381 | 0.1179911 | 0.1172176 | 0.3339557 | TMEM18 | | 129787 |
| ENSG00000228613 | 17.10174765 | 0.022479806 | 0.1362375 | 0.4309501 | NA | NA | NA | |
| ENSG00000203635 | 22.44889779 | 0.056916523 | 0.1503744 | 0.0789642 | NA | NA | NA | |
| ENSG00000130508 | 404.4997085 | -0.00120112 | 0.0987656 | 0.9846528 | 0.9940394 | PXDN | | 7837 |
| ENSG00000032389 | 357.1700372 | 0.005528215 | 0.0997929 | 0.9361357 | 0.9755069 | EIPR1 | | 7260 |
| ENSG00000171853 | 454.090781 | 3.62E-05 | 0.0948588 | 0.9990533 | 0.9993299 | TRAPPC12 | | 51112 |
| ENSG00000182551 | 854.5815859 | 0.072931797 | 0.0937535 | 0.3032863 | 0.5726018 | ADI1 | | 55256 |
| ENSG00000242282 | 25.27891593 | 0.002328502 | 0.1322363 | 0.9465875 | NA | NA | NA | |
| ENSG00000171865 | 640.4785324 | 0.015757334 | 0.0887121 | 0.8181606 | 0.9254567 | RNASEH1 | | 246243 |
| ENSG00000234171 | 197.473462 | 0.006786045 | 0.1114116 | 0.9177989 | 0.9678623 | RNASEH1-D | | 100506054 |
| ENSG00000171863 | 7594.043937 | -0.246390535 | 0.0569901 | 3.44E-06 | 0.0001008 | RPS7 | | 6201 |
| ENSG00000151692 | 168.3289636 | 0.011646241 | 0.114203 | 0.8552335 | 0.9416517 | RNF144A | | 9781 |
| ENSG00000115738 | 50.8656795 | 0.071591157 | 0.1535964 | 0.1376537 | 0.3658551 | ID2 | | 3398 |
| ENSG00000134313 | 1388.947642 | 0.071469178 | 0.0771188 | 0.265612 | 0.5333702 | KIDINS220 | | 57498 |
| ENSG00000143797 | 598.4802536 | -0.013452667 | 0.0887969 | 0.8404912 | 0.9344745 | MBOAT2 | | 129642 |
| ENSG00000151693 | 956.7740265 | 0.03563789 | 0.0807803 | 0.5837466 | 0.7951888 | ASAP2 | | 8853 |
| ENSG00000119185 | 1190.371264 | -0.144321634 | 0.0858466 | 0.038767 | 0.1643563 | ITGB1BP1 | | 9270 |
| ENSG00000119203 | 821.5912949 | -0.037465329 | 0.0850952 | 0.5750647 | 0.7888821 | CPSF3 | | 51692 |
| ENSG00000134330 | 285.3705183 | -0.097908344 | 0.1270682 | 0.1990582 | 0.4524614 | IAH1 | | 285148 |
| ENSG00000151694 | 287.0014284 | 0.016832448 | 0.1050432 | 0.8034472 | 0.9182142 | ADAM17 | | 6868 |
| ENSG00000134308 | 5671.388483 | -0.08807944 | 0.0574446 | 0.0901566 | 0.2835213 | YWHAQ | | 10971 |
| ENSG00000115750 | 283.3098661 | -0.075220834 | 0.1177597 | 0.2951427 | 0.5652292 | TAF1B | | 9014 |
| ENSG00000269973 | 30.70939166 | -0.000169708 | 0.1314312 | 0.9942011 | NA | NA | NA | |
| ENSG00000134317 | 108.2096127 | -0.10487702 | 0.16374 | 0.1252125 | 0.347221 | GRHL1 | | 29841 |
| ENSG00000260077 | 11.84439628 | 0.009254319 | 0.1349132 | 0.702962 | NA | NA | NA | |
| ENSG00000172059 | 241.1714451 | 0.261693079 | 0.1955293 | 0.0187392 | 0.0992261 | KLF11 | | 8462 |
| ENSG00000205795 | 8.987740433 | -0.002442848 | 0.1349952 | 0.9059145 | NA | CYS1 | | 192668 |

| | | | | | | | | |
|---|---|---|---|---|---|---|---|---|
| ENSG00000171848 | 5709.907155 | 0.285306759 | 0.0707663 | 9.49E-06 | 0.0002404 | RRM2 | | 6241 |
| ENSG00000163009 | 13.95941462 | -0.010190997 | 0.1346483 | 0.6914439 | NA | NA | NA | |
| ENSG00000115756 | 2483.795934 | 0.083143816 | 0.0703379 | 0.1695733 | 0.4153346 | HPCAL1 | | 3241 |
| ENSG00000115758 | 2496.842061 | 0.080505353 | 0.0718134 | 0.1844944 | 0.4347548 | ODC1 | | 4953 |
| ENSG00000257135 | 15.22783343 | 0.049403047 | 0.1471435 | 0.0769045 | NA | NA | NA | |
| ENSG00000115761 | 987.3864975 | -0.064435403 | 0.0818047 | 0.3273381 | 0.5953965 | NOL10 | | 79954 |
| ENSG00000143882 | 34.97362114 | -0.035019769 | 0.1371715 | 0.3579568 | NA | ATP6V1C2 | | 245973 |
| ENSG00000143870 | 4163.750649 | -0.19544497 | 0.0658486 | 0.000938 | 0.0105055 | PDIA6 | | 10130 |
| ENSG00000162976 | 83.63223832 | 0.059623928 | 0.1382447 | 0.2944904 | 0.5647583 | SLC66A3 | | 130814 |
| ENSG00000134318 | 1177.14096 | -0.150578519 | 0.0862194 | 0.0321012 | 0.1444432 | ROCK2 | | 9475 |
| ENSG00000169016 | 516.9377948 | -0.005931169 | 0.0929784 | 0.9293293 | 0.9726302 | E2F6 | | 1876 |
| ENSG00000134324 | 789.904613 | -0.456019541 | 0.244199 | 0.0031719 | 0.026636 | LPIN1 | | 23175 |
| ENSG00000071575 | 275.6577677 | 0.040997644 | 0.1161861 | 0.5457388 | 0.7679526 | TRIB2 | | 28951 |
| ENSG00000151779 | 532.4604468 | 0.08775325 | 0.1022245 | 0.2364824 | 0.5007816 | NBAS | | 51594 |
| ENSG00000079785 | 2272.046852 | -0.219043725 | 0.0787793 | 0.0013406 | 0.014108 | DDX1 | | 1653 |
| ENSG00000163029 | 1242.305205 | 0.059329312 | 0.0811454 | 0.3680535 | 0.6308952 | SMC6 | | 79677 |
| ENSG00000178295 | 629.133093 | 0.035904496 | 0.0895107 | 0.6021593 | 0.8079281 | GEN1 | | 348654 |
| ENSG00000240857 | 131.8410341 | -0.010583002 | 0.119227 | 0.8568985 | 0.9422695 | RDH14 | | 57665 |
| ENSG00000143867 | 35.1050309 | -0.035074351 | 0.1365629 | 0.3749953 | NA | OSR1 | | 130497 |
| ENSG00000183891 | 60.13634611 | -0.003187856 | 0.1269649 | 0.9495907 | 0.9800467 | TTC32 | | 130502 |
| ENSG00000118965 | 546.378385 | 0.234749128 | 0.1278848 | 0.0115898 | 0.0705843 | WDR35 | | 57539 |
| ENSG00000132031 | 42.49735915 | -0.007166712 | 0.1299802 | 0.8586444 | 0.9430101 | MATN3 | | 4148 |
| ENSG00000068697 | 2139.060362 | -0.037910779 | 0.065868 | 0.5103667 | 0.7416935 | LAPTM4A | | 9741 |
| ENSG00000115884 | 1374.723678 | 0.018495219 | 0.0759463 | 0.7704654 | 0.9020689 | SDC1 | | 6382 |
| ENSG00000055917 | 1887.6418 | -0.055437894 | 0.0729612 | 0.3698056 | 0.6322745 | PUM2 | | 23369 |
| ENSG00000143878 | 1219.854887 | -0.067530012 | 0.1542361 | 0.102839 | 0.3074144 | RHOB | | 388 |
| ENSG00000118960 | 399.9070422 | 0.086628734 | 0.1114086 | 0.2466101 | 0.5126628 | HS1BP3 | | 64342 |
| ENSG00000118961 | 184.9951192 | -0.120214405 | 0.1593671 | 0.1203618 | 0.3390055 | LDAH | | 60526 |
| ENSG00000119771 | 452.7075638 | 0.376694885 | 0.139498 | 0.0006328 | 0.0077242 | KLHL29 | | 114818 |
| ENSG00000119778 | 280.9291464 | -0.0312672 | 0.1068984 | 0.6463392 | 0.8355343 | ATAD2B | | 54454 |
| ENSG00000173960 | 655.8763865 | -0.034276738 | 0.0882204 | 0.6127331 | 0.8148077 | UBXN2A | | 165324 |
| ENSG00000163026 | 320.5203677 | -0.029303613 | 0.1035438 | 0.6692786 | 0.848177 | WDCP | | 80304 |
| ENSG00000119782 | 61.3572717 | -0.025936669 | 0.1296824 | 0.5893898 | 0.7988701 | FKBP1B | | 2281 |
| ENSG00000115128 | 845.9556035 | -0.083118733 | 0.0988485 | 0.2511673 | 0.5176228 | SF3B6 | | 51639 |
| ENSG00000219626 | 14.77446139 | 0.017812109 | 0.1360081 | 0.4743368 | NA | FAM228B | | 375190 |
| ENSG00000115129 | 308.6933171 | -0.025237862 | 0.1058282 | 0.7097631 | 0.8682712 | TP53I3 | | 9540 |
| ENSG00000198399 | 1091.918648 | -0.016842508 | 0.0808251 | 0.7937411 | 0.913934 | ITSN2 | | 50618 |
| ENSG00000084676 | 289.0543356 | 0.001520763 | 0.1052913 | 0.9829925 | 0.9934535 | NCOA1 | | 8648 |
| ENSG00000184924 | 234.9185799 | -0.169662472 | 0.1697014 | 0.0618563 | 0.2221161 | PTRHD1 | | 391356 |
| ENSG00000138092 | 1229.896287 | 0.079815897 | 0.0786883 | 0.2208454 | 0.4803902 | CENPO | | 79172 |
| ENSG00000138031 | 1119.634325 | -0.09881846 | 0.0838931 | 0.1448325 | 0.3772945 | ADCY3 | | 109 |
| ENSG00000271936 | 93.8901001 | 0.041283298 | 0.130875 | 0.4446679 | 0.6929249 | NA | NA | |
| ENSG00000115137 | 90.56405264 | 0.105221682 | 0.1698285 | 0.1081641 | 0.3183334 | DNAJC27 | | 51277 |
| ENSG00000084710 | 11.91963755 | 0.003174776 | 0.1346325 | 0.8942107 | NA | EFR3B | | 22979 |
| ENSG00000119772 | 126.1521591 | -0.031196318 | 0.1221923 | 0.6020976 | 0.8079281 | DNMT3A | | 1788 |
| ENSG00000138101 | 276.7777929 | -0.083204643 | 0.125997 | 0.2562686 | 0.5231846 | DTNB | | 1838 |
| ENSG00000143970 | 1182.410973 | 0.025063749 | 0.0778772 | 0.6965658 | 0.8616368 | ASXL2 | | 55252 |
| ENSG00000084731 | 395.0123484 | 0.359795637 | 0.1542215 | 0.0017717 | 0.017289 | KIF3C | | 3797 |
| ENSG00000084733 | 2236.975582 | -0.197334536 | 0.0738947 | 0.0021825 | 0.0202488 | RAB10 | | 10890 |
| ENSG00000157833 | 61.89534364 | 0.104851264 | 0.1792179 | 0.075308 | 0.2520553 | GAREM2 | | 150946 |
| ENSG00000084754 | 2122.932663 | -0.086575792 | 0.0686157 | 0.1448183 | 0.3772945 | HADHA | | 3030 |
| ENSG00000138029 | 1034.818792 | -0.068793555 | 0.0830519 | 0.3031401 | 0.5725109 | HADHB | | 3032 |
| ENSG00000138018 | 1059.802498 | -0.018763283 | 0.0793952 | 0.7708769 | 0.9023563 | SELENOI | | 85465 |
| ENSG00000213699 | 607.4397119 | -0.181135338 | 0.1168534 | 0.03217 | 0.1446412 | SLC35F6 | | 54978 |
| ENSG00000115163 | 531.5448958 | -0.325423638 | 0.1322893 | 0.0015426 | 0.0156613 | CENPA | | 1058 |
| ENSG00000084764 | 138.0753841 | 0.136176575 | 0.1804325 | 0.0863 | 0.2756984 | MAPRE3 | | 22924 |
| ENSG00000119777 | 1167.035653 | -0.099146435 | 0.0855962 | 0.1480632 | 0.3820469 | TMEM214 | | 54867 |
| ENSG00000084693 | 386.8057694 | -0.066657729 | 0.1102407 | 0.355415 | 0.6192724 | AGBL5 | | 60509 |
| ENSG00000228474 | 654.8633678 | -0.054831522 | 0.0912011 | 0.4277967 | 0.6791494 | OST4 | | 100128731 |
| ENSG00000138030 | 76.14561205 | -0.025263509 | 0.1277938 | 0.61959 | 0.8180661 | KHK | | 3795 |
| ENSG00000138028 | 33.89239804 | 0.101460658 | 0.1876062 | 0.0280333 | NA | CGREF1 | | 10669 |
| ENSG00000138073 | 868.6043935 | 0.007756755 | 0.0809554 | 0.9061841 | 0.9628103 | PREB | | 10113 |
| ENSG00000138074 | 985.1608411 | -0.219414835 | 0.1004558 | 0.0064288 | 0.0458736 | SLC5A6 | | 8884 |
| ENSG00000138085 | 357.9210816 | -0.039986373 | 0.1032241 | 0.567072 | 0.7831055 | ATRAID | | 51374 |
| ENSG00000084774 | 3348.890366 | -0.055792752 | 0.0607124 | 0.3038346 | 0.5728743 | CAD | | 790 |
| ENSG00000163794 | 11.8684112 | 0.013179221 | 0.1353784 | 0.5868911 | NA | UCN | | 7349 |
| ENSG00000115204 | 335.1963204 | 0.105206715 | 0.1221629 | 0.1771796 | 0.4267086 | MPV17 | | 4358 |
| ENSG00000115207 | 1113.448004 | -0.108477977 | 0.0848217 | 0.112832 | 0.3257515 | GTF3C2 | | 2976 |
| ENSG00000234945 | 9.906670912 | 0.002150847 | 0.1348404 | 0.9247423 | NA | GTF3C2-AS | | 100505624 |
| ENSG00000234072 | 106.4422359 | 0.065706219 | 0.1378194 | 0.2821306 | 0.5512825 | NA | NA | |

| | | | | | | | | |
|---|---|---|---|---|---|---|---|---|
| ENSG00000115211 | 676.8386733 | -0.017120922 | 0.0877577 | 0.7986461 | 0.9162772 | EIF2B4 | | 8890 |
| ENSG00000115234 | 1326.501095 | -0.019408604 | 0.0725672 | 0.7517937 | 0.8927216 | SNX17 | | 9784 |
| ENSG00000163795 | 44.20739922 | -0.03754644 | 0.1356396 | 0.3912458 | 0.6514814 | ZNF513 | | 130557 |
| ENSG00000115241 | 3828.075608 | -0.178988958 | 0.0626542 | 0.0015268 | 0.0155847 | PPM1G | | 5496 |
| ENSG00000115216 | 1111.456841 | -0.232261456 | 0.0946035 | 0.0029547 | 0.0254153 | NRBP1 | | 29959 |
| ENSG00000138002 | 210.613649 | -0.060810793 | 0.1231324 | 0.3718342 | 0.6339491 | IFT172 | | 26160 |
| ENSG00000115226 | 43.61655629 | 0.046322115 | 0.1395822 | 0.2914769 | 0.5613232 | FNDC4 | | 64838 |
| ENSG00000243943 | 223.9751724 | 0.193733181 | 0.1860103 | 0.0456061 | 0.1823775 | ZNF512 | | 84450 |
| ENSG00000176714 | 45.18579829 | -0.041630049 | 0.1369994 | 0.349515 | 0.6154182 | CCDC121 | | 79635 |
| ENSG00000198522 | 592.4754899 | -0.098549002 | 0.1016104 | 0.1831491 | 0.4329821 | GPN1 | | 11321 |
| ENSG00000119760 | 615.4823278 | 0.195919847 | 0.1147598 | 0.0213795 | 0.1087693 | SUPT7L | | 9913 |
| ENSG00000163798 | 626.4171131 | -0.007961883 | 0.0885034 | 0.9044731 | 0.9622177 | SLC4A1AP | | 22950 |
| ENSG00000243147 | 406.8730088 | -0.038298243 | 0.1000157 | 0.581501 | 0.7933122 | MRPL33 | | 9553 |
| ENSG00000171174 | 70.70598969 | 0.0960231 | 0.1685136 | 0.10084 | 0.303592 | RBKS | | 64080 |
| ENSG00000158019 | 129.2141312 | -0.015616994 | 0.11948 | 0.7905313 | 0.9121996 | BABAM2 | | 9577 |
| ENSG00000075426 | 1710.44831 | 0.253485177 | 0.0850735 | 0.0005593 | 0.0070101 | FOSL2 | | 2355 |
| ENSG00000213639 | 1569.094374 | -0.038820549 | 0.0729161 | 0.5287667 | 0.754996 | PPP1CB | | 5500 |
| ENSG00000171103 | 253.9641363 | -0.1326582 | 0.1468683 | 0.1090883 | 0.3197644 | TRMT61B | | 55006 |
| ENSG00000163811 | 1977.839738 | -0.089267165 | 0.0738172 | 0.1525946 | 0.3899492 | WDR43 | | 23160 |
| ENSG00000115295 | 552.6070787 | 0.150166136 | 0.1149555 | 0.0655343 | 0.2312931 | CLIP4 | | 79745 |
| ENSG00000119801 | 473.6162928 | 0.035389048 | 0.0957132 | 0.6098941 | 0.8128053 | YPEL5 | | 51646 |
| ENSG00000172954 | 364.3339844 | -0.044924792 | 0.1030878 | 0.52017 | 0.7486576 | LCLAT1 | | 253558 |
| ENSG00000158089 | 542.1189128 | -0.072273584 | 0.0982896 | 0.3124644 | 0.5818701 | GALNT14 | | 79623 |
| ENSG00000013016 | 31.65846976 | -0.032126268 | 0.1365567 | 0.3886945 | NA | EHD3 | | 30845 |
| ENSG00000158125 | 29.84920674 | 0.054569711 | 0.1467403 | 0.153253 | NA | XDH | | 7498 |
| ENSG00000162959 | 58.85123299 | 0.023999347 | 0.1293619 | 0.6176947 | 0.8169488 | MEMO1 | | 51072 |
| ENSG00000162961 | 474.4840639 | -0.065884363 | 0.1040179 | 0.359517 | 0.6227044 | DPY30 | | 84661 |
| ENSG00000021574 | 381.6276652 | -0.293933243 | 0.1538614 | 0.0062978 | 0.0451595 | SPAST | | 6683 |
| ENSG00000152683 | 567.1622984 | -0.056404322 | 0.0965414 | 0.4219597 | 0.6742697 | SLC30A6 | | 55676 |
| ENSG00000119820 | 589.0694291 | 0.004830932 | 0.0905063 | 0.9441745 | 0.9784014 | YIPF4 | | 84272 |
| ENSG00000115760 | 1933.369482 | -0.026828441 | 0.0681326 | 0.6491566 | 0.8366057 | BIRC6 | | 57448 |
| ENSG00000018699 | 466.4491618 | -0.088748882 | 0.1073213 | 0.2330265 | 0.4953562 | TTC27 | | 55622 |
| ENSG00000049323 | 64.55155411 | -0.06795963 | 0.1476008 | 0.1939589 | 0.4459033 | LTBP1 | | 4052 |
| ENSG00000119812 | 1004.593659 | -0.090878203 | 0.08847 | 0.1883226 | 0.4389939 | FAM98A | | 25940 |
| ENSG00000260025 | 69.87311378 | 0.028498956 | 0.1290235 | 0.5734053 | 0.7878085 | NA | NA | |
| ENSG00000150938 | 9457.120934 | -0.000855063 | 0.0630452 | 0.9871398 | 0.994767 | CRIM1 | | 51232 |
| ENSG00000273090 | 16.40348402 | 0.048608767 | 0.1467255 | 0.0815901 | NA | NA | NA | |
| ENSG00000171055 | 816.6998814 | 0.05949332 | 0.0858368 | 0.3784435 | 0.6398319 | FEZ2 | | 9637 |
| ENSG00000115808 | 1310.060086 | -0.174757331 | 0.0850901 | 0.0132972 | 0.0775848 | STRN | | 6801 |
| ENSG00000008869 | 834.192841 | 0.006026459 | 0.084749 | 0.957232 | 0.9826522 | HEATR5B | | 54497 |
| ENSG00000152133 | 582.1419143 | -0.078931006 | 0.0975048 | 0.2730526 | 0.5411481 | GPATCH11 | | 253635 |
| ENSG00000055332 | 1923.181075 | -0.1218736 | 0.0733707 | 0.0517647 | 0.1967027 | EIF2AK2 | | 5610 |
| ENSG00000218739 | 751.0878601 | -0.163948416 | 0.1051207 | 0.0387094 | 0.164233 | CEBPZOS | | 100505876 |
| ENSG00000115816 | 2023.522332 | 0.086553839 | 0.0720649 | 0.1600797 | 0.4010921 | CEBPZ | | 10153 |
| ENSG00000272054 | 27.9308762 | -0.033267697 | 0.1376816 | 0.3377122 | NA | NA | NA | |
| ENSG00000003509 | 446.4146435 | -0.162620646 | 0.1271639 | 0.0572734 | 0.2117479 | NDUFAF7 | | 55471 |
| ENSG00000115825 | 1662.536756 | -0.117661654 | 0.0757991 | 0.0659023 | 0.2320448 | PRKD3 | | 23683 |
| ENSG00000115828 | 177.2696255 | 0.012897572 | 0.1173338 | 0.8336561 | 0.9305701 | QPCT | | 25797 |
| ENSG00000163171 | 1436.81077 | 0.155510214 | 0.161958 | 0.0766266 | 0.2549883 | CDC42EP3 | | 10602 |
| ENSG00000115841 | 47.70606384 | -0.020851424 | 0.1304838 | 0.6356705 | 0.8269592 | RMDN2 | | 151393 |
| ENSG00000138061 | 27.06042705 | 0.069096946 | 0.157177 | 0.0694364 | NA | CYP1B1 | | 1545 |
| ENSG00000119787 | 1323.814136 | -0.189209759 | 0.0875662 | 0.0090243 | 0.0593878 | ATL2 | | 64225 |
| ENSG00000213553 | 879.7340022 | 0.009789023 | 0.0814595 | 0.8819088 | 0.9524387 | RPLP0P6 | | 220717 |
| ENSG00000143889 | 930.3028227 | -0.05092629 | 0.082438 | 0.439192 | 0.6886014 | HNRNPLL | | 92906 |
| ENSG00000143891 | 69.61913869 | -0.078646264 | 0.1526932 | 0.1628539 | 0.4051748 | GALM | | 130589 |
| ENSG00000115875 | 3672.159302 | -0.071045216 | 0.0651848 | 0.2142675 | 0.4719704 | SRSF7 | | 6432 |
| ENSG00000152147 | 303.0055401 | 0.18305874 | 0.1585172 | 0.0491499 | 0.1904211 | GEMIN6 | | 79833 |
| ENSG00000163214 | 914.3427834 | -0.13931165 | 0.0949501 | 0.0595545 | 0.2170464 | DHX57 | | 90957 |
| ENSG00000188010 | 202.1619164 | 0.010765898 | 0.1111233 | 0.8697924 | 0.9475003 | MORN2 | | 729967 |
| ENSG00000115904 | 1239.356564 | -0.087577961 | 0.0830845 | 0.1921453 | 0.4433917 | SOS1 | | 6654 |
| ENSG00000229692 | 28.4761307 | -0.004982316 | 0.1317178 | 0.8864847 | NA | NA | NA | |
| ENSG00000011566 | 767.7936627 | -0.087774308 | 0.093139 | 0.2168369 | 0.4757459 | MAP4K3 | | 8491 |
| ENSG00000231312 | 48.28740484 | 0.027641776 | 0.1325642 | 0.5256183 | 0.7529736 | MAP4K3-DT | | 728730 |
| ENSG00000138050 | 398.554888 | 0.492719872 | 0.1536398 | 9.28E-05 | 0.0016428 | THUMPD2 | | 80745 |
| ENSG00000183023 | 169.9126755 | -0.110284238 | 0.1518896 | 0.1450999 | 0.3776542 | SLC8A1 | | 6546 |
| ENSG00000162878 | 39.57569298 | -0.008914356 | 0.1302287 | 0.8245167 | NA | PKDCC | | 91461 |
| ENSG00000143924 | 2853.146725 | -0.071332684 | 0.0658304 | 0.2198192 | 0.479502 | EML4 | | 27436 |
| ENSG00000115944 | 1728.78197 | -0.008279298 | 0.0756359 | 0.8949986 | 0.9579839 | COX7A2L | | 9167 |
| ENSG00000057935 | 759.8559049 | -0.247961269 | 0.1121093 | 0.0047094 | 0.0363346 | MTA3 | | 57504 |
| ENSG00000115970 | 1439.866977 | 0.094279544 | 0.0778063 | 0.1456898 | 0.3782625 | THADA | | 63892 |

| | | | | | | | | |
|---|---|---|---|---|---|---|---|---|
| ENSG00000152518 | 1342.193439 | 0.232816142 | 0.0888156 | 0.0018971 | 0.0181787 | ZFP36L2 | | 678 |
| ENSG00000152527 | 153.7613664 | 0.273051568 | 0.2633807 | 0.0208875 | 0.1067779 | PLEKHH2 | | 130271 |
| ENSG00000138036 | 145.0655952 | 0.002544447 | 0.1171727 | 0.967777 | 0.9872153 | DYNC2LI1 | | 51626 |
| ENSG00000138095 | 6111.006469 | 0.084391699 | 0.0569778 | 0.1026713 | 0.3071201 | LRPPRC | | 10128 |
| ENSG00000138032 | 1414.619769 | -0.115151467 | 0.0798913 | 0.0822862 | 0.266996 | PPM1B | | 5495 |
| ENSG00000138079 | 25.20454317 | 0.015633065 | 0.1333911 | 0.6453895 | NA | SLC3A1 | | 6519 |
| ENSG00000138078 | 1930.952189 | 0.156864868 | 0.0755083 | 0.0152599 | 0.0852873 | PREPL | | 9581 |
| ENSG00000143919 | 169.0352536 | -0.018729665 | 0.1159009 | 0.7630121 | 0.8982943 | CAMKMT | | 79823 |
| ENSG00000068784 | 495.6040587 | 0.172544211 | 0.1280168 | 0.0471281 | 0.1856673 | SRBD1 | | 55133 |
| ENSG00000171132 | 565.6241781 | 0.207701916 | 0.1277612 | 0.0216384 | 0.1093783 | PRKCE | | 5581 |
| ENSG00000116016 | 1703.868039 | 0.979871792 | 0.1023076 | 5.80E-23 | 2.34E-20 | EPAS1 | | 2034 |
| ENSG00000250565 | 187.5133614 | 0.013467169 | 0.1136943 | 0.8337662 | 0.9305701 | ATP6V1E2 | | 90423 |
| ENSG00000119729 | 1592.002824 | -0.065929353 | 0.0737486 | 0.2902379 | 0.560019 | RHOQ | | 23433 |
| ENSG00000151665 | 423.431133 | -0.122133396 | 0.1182381 | 0.1248225 | 0.34677 | PIGF | | 5281 |
| ENSG00000119878 | 452.8208685 | -0.104059995 | 0.1160223 | 0.1773138 | 0.4267086 | CRIPT | | 9419 |
| ENSG00000171150 | 1416.513052 | -0.387298435 | 0.0872959 | 9.76E-07 | 3.33E-05 | SOCS5 | | 9655 |
| ENSG00000260977 | 11.28266591 | -0.003928736 | 0.1346784 | 0.8637568 | NA | NA | NA | |
| ENSG00000222005 | 10.09714828 | -0.004214474 | 0.1348705 | 0.8480395 | NA | LINC01118 | | 388948 |
| ENSG00000239332 | 14.39109972 | -0.011717628 | 0.1348993 | 0.645028 | NA | LINC01119 | | 100134259 |
| ENSG00000226548 | 10.64803087 | -0.016116441 | 0.1360786 | 0.4799363 | NA | NA | NA | |
| ENSG00000180398 | 3842.747677 | -0.10488696 | 0.0605397 | 0.053143 | 0.2006995 | MCFD2 | | 90411 |
| ENSG00000068724 | 982.3610788 | -0.005272516 | 0.0835268 | 0.9355194 | 0.9752526 | TTC7A | | 57217 |
| ENSG00000143933 | 15304.38723 | -0.153214267 | 0.0602382 | 0.0048549 | 0.0372427 | CALM2 | | 805 |
| ENSG00000119888 | 20.38641596 | 0.022212425 | 0.1354902 | 0.4774462 | NA | EPCAM | | 4072 |
| ENSG00000095002 | 3370.47947 | 0.026856662 | 0.0611257 | 0.6316062 | 0.8251641 | MSH2 | | 4436 |
| ENSG00000230979 | 13.84194553 | -0.005347029 | 0.1343731 | 0.8301368 | NA | RPL18AP6 | | 285053 |
| ENSG00000116062 | 3452.570577 | -0.110550106 | 0.0638433 | 0.0504255 | 0.1934386 | MSH6 | | 2956 |
| ENSG00000138081 | 1754.410482 | -0.217878022 | 0.0822608 | 0.0019932 | 0.01878 | FBXO11 | | 80204 |
| ENSG00000233230 | 15.22055468 | 0.030989699 | 0.1391871 | 0.2424031 | NA | LOC100506[illegible] | | 100506235 |
| ENSG00000170802 | 926.1815291 | -0.015454312 | 0.0797155 | 0.8131035 | 0.9235572 | FOXN2 | | 3344 |
| ENSG00000162869 | 520.7588474 | -0.018176161 | 0.0918076 | 0.7889175 | 0.9116361 | PPP1R21 | | 129285 |
| ENSG00000243244 | 12.91007496 | -0.035194342 | 0.1412794 | 0.1405231 | NA | STON1 | | 11037 |
| ENSG00000115239 | 27.26590357 | 0.031963231 | 0.1369109 | 0.3718747 | NA | ASB3 | | 51130 |
| ENSG00000143942 | 352.3290856 | -0.023811259 | 0.1014936 | 0.7281133 | 0.8783023 | CHAC2 | | 494143 |
| ENSG00000068912 | 909.092011 | -0.036586446 | 0.0814148 | 0.5768353 | 0.79029 | ERLEC1 | | 27248 |
| ENSG00000119737 | 60.0880892 | 0.033501206 | 0.1316293 | 0.4924673 | 0.7283747 | GPR75 | | 10936 |
| ENSG00000068878 | 2211.462585 | -0.218025954 | 0.0807494 | 0.0017063 | 0.0168905 | PSME4 | | 23198 |
| ENSG00000170634 | 138.0365009 | 0.005895471 | 0.1169338 | 0.9247803 | 0.9709924 | ACYP2 | | 98 |
| ENSG00000115306 | 8008.081631 | -0.073415002 | 0.052723 | 0.1300367 | 0.3544317 | SPTBN1 | | 6711 |
| ENSG00000214595 | 166.5521762 | 0.029212372 | 0.1168917 | 0.6480232 | 0.836587 | EML6 | | 400954 |
| ENSG00000115310 | 13063.5103 | -0.049607912 | 0.0520459 | 0.3020401 | 0.5712647 | RTN4 | | 57142 |
| ENSG00000162994 | 52.51388112 | 0.002993565 | 0.1281416 | 0.9502305 | 0.9802134 | CLHC1 | | 130162 |
| ENSG00000227799 | 19.3731628 | 0.018184166 | 0.1348215 | 0.5505815 | NA | NA | NA | |
| ENSG00000143947 | 11846.80821 | -0.098815055 | 0.0529042 | 0.0427906 | 0.1749231 | RPS27A | | 6233 |
| ENSG00000085760 | 938.2830417 | -0.064584066 | 0.0838519 | 0.3310034 | 0.5989207 | MTIF2 | | 4528 |
| ENSG00000162997 | 19.31281707 | -0.001586947 | 0.1332756 | 0.9550425 | NA | PRORSD1P | | 344405 |
| ENSG00000115355 | 2565.139324 | -0.10244524 | 0.0714991 | 0.0946336 | 0.2918558 | CCDC88A | | 55704 |
| ENSG00000240401 | 15.16227664 | -0.02062512 | 0.1361038 | 0.4456658 | NA | NA | NA | |
| ENSG00000163001 | 506.7060206 | -0.147489954 | 0.1166157 | 0.0707268 | 0.2420798 | CFAP36 | | 112942 |
| ENSG00000138041 | 2225.680115 | -0.077554862 | 0.0671 | 0.18461 | 0.4349394 | NA | NA | |
| ENSG00000272606 | 9.695478728 | -0.012286183 | 0.1358163 | 0.5575535 | NA | NA | NA | |
| ENSG00000138035 | 2091.049765 | 0.052183165 | 0.0664204 | 0.3738746 | 0.6352333 | PNPT1 | | 87178 |
| ENSG00000028116 | 774.6257472 | -0.062337097 | 0.0890396 | 0.3584659 | 0.6218058 | VRK2 | | 7444 |
| ENSG00000115392 | 543.6881016 | 0.029381942 | 0.0918952 | 0.6686945 | 0.847734 | FANCL | | 55120 |
| ENSG00000115421 | 408.0264308 | -0.047851959 | 0.1017448 | 0.4969358 | 0.7319933 | PAPOLG | | 64895 |
| ENSG00000162924 | 409.0346468 | 0.004819432 | 0.0976682 | 0.9452632 | 0.9784014 | REL | | 5966 |
| ENSG00000162927 | 142.4730652 | 0.010371186 | 0.1171706 | 0.8658329 | 0.9458332 | PUS10 | | 150962 |
| ENSG00000162928 | 784.9467535 | 0.061539666 | 0.0870169 | 0.3643205 | 0.6269614 | PEX13 | | 5194 |
| ENSG00000162929 | 474.9647237 | -0.038916279 | 0.0965568 | 0.575225 | 0.7889167 | SANBR | | 84542 |
| ENSG00000212978 | 165.964668 | -0.036700602 | 0.1181551 | 0.5685637 | 0.7844782 | C2orf74-DT | | 339803 |
| ENSG00000173209 | 1315.317855 | -0.110127707 | 0.0889242 | 0.1173424 | 0.3339561 | AHSA2P | | 130872 |
| ENSG00000115464 | 4808.23896 | -0.310026316 | 0.0627697 | 1.21E-07 | 5.70E-06 | USP34 | | 9736 |
| ENSG00000270820 | 102.8280811 | -0.072397222 | 0.1410563 | 0.2452341 | 0.5109846 | NA | NA | |
| ENSG00000082898 | 10513.46981 | -0.349711204 | 0.0556628 | 4.40E-11 | 4.04E-09 | XPO1 | | 7514 |
| ENSG00000170264 | 234.0545767 | 0.003375097 | 0.1086445 | 0.9599826 | 0.9834728 | FAM161A | | 84140 |
| ENSG00000115484 | 9391.389878 | -0.273796823 | 0.0613296 | 1.51E-06 | 4.78E-05 | CCT4 | | 10575 |
| ENSG00000236498 | 44.4356384 | -0.023920151 | 0.1340266 | 0.5192395 | 0.7478713 | NA | NA | |
| ENSG00000173163 | 373.4186358 | -0.055188443 | 0.1045095 | 0.436681 | 0.6870784 | COMMD1 | | 150684 |
| ENSG00000170340 | 739.4946324 | -0.246855915 | 0.1153167 | 0.0055909 | 0.0412779 | B3GNT2 | | 10678 |
| ENSG00000186889 | 29.54546598 | 0.047220224 | 0.1431428 | 0.197562 | NA | TMEM17 | | 200728 |

| | | | | | | | | |
|---|---|---|---|---|---|---|---|---|
| ENSG00000115504 | 1393.123508 | -0.016587965 | 0.0740403 | 0.7892973 | 0.9116361 | EHBP1 | | 23301 |
| ENSG00000231609 | 10.64803087 | -0.016116441 | 0.1360786 | 0.4799363 | NA | EHBP1-AS1 | | 100132215 |
| ENSG00000115507 | 80.15531376 | -0.014132364 | 0.125588 | 0.7806468 | 0.9087227 | OTX1 | | 5013 |
| ENSG00000143951 | 83.10618006 | -0.02058595 | 0.1265137 | 0.6870852 | 0.857187 | WDPCP | | 51057 |
| ENSG00000014641 | 2633.282713 | -0.08996518 | 0.0665049 | 0.1220141 | 0.342196 | MDH1 | | 4190 |
| ENSG00000169764 | 1516.323266 | -0.147183526 | 0.0812115 | 0.0296593 | 0.1372079 | UGP2 | | 7360 |
| ENSG00000143952 | 572.7016038 | -0.145508549 | 0.1115743 | 0.0692718 | 0.239121 | VPS54 | | 51542 |
| ENSG00000197329 | 1378.382761 | -0.311716941 | 0.0889061 | 6.35E-05 | 0.0012038 | PELI1 | | 57162 |
| ENSG00000225889 | 13.47295139 | 0.032973597 | 0.1400587 | 0.1993922 | NA | NA | NA | |
| ENSG00000119862 | 302.3220932 | -0.100527085 | 0.1251088 | 0.1908137 | 0.4415402 | LGALSL | | 29094 |
| ENSG00000119844 | 1001.369523 | -0.124339725 | 0.0903565 | 0.0815441 | 0.2655469 | AFTPH | | 54812 |
| ENSG00000179833 | 871.7170225 | 0.396958191 | 0.1074716 | 2.17E-05 | 0.0004839 | SERTAD2 | | 9792 |
| ENSG00000115902 | 410.9964235 | 0.605887257 | 0.1692686 | 2.08E-05 | 0.00047 | SLC1A4 | | 6509 |
| ENSG00000011523 | 342.11253 | 0.088512428 | 0.1172789 | 0.2401022 | 0.5054546 | CEP68 | | 23177 |
| ENSG00000138069 | 2954.830133 | -0.168223462 | 0.0667933 | 0.0044796 | 0.0347911 | RAB1A | | 5861 |
| ENSG00000172974 | 26.23503711 | 0.009945934 | 0.1324976 | 0.7733015 | NA | NA | NA | |
| ENSG00000138071 | 11581.74762 | -0.14481218 | 0.0597395 | 0.0070351 | 0.0489441 | ACTR2 | | 10097 |
| ENSG00000198369 | 1181.746794 | -0.132014328 | 0.0857644 | 0.0572454 | 0.2117429 | SPRED2 | | 200734 |
| ENSG00000143995 | 57.98741993 | -0.042250447 | 0.135105 | 0.3842306 | 0.6447633 | MEIS1 | | 4211 |
| ENSG00000143971 | 337.0447963 | -0.117146777 | 0.1271891 | 0.1415726 | 0.3718661 | ETAA1 | | 54465 |
| ENSG00000197223 | 471.284235 | -0.151638957 | 0.1239155 | 0.069681 | 0.2400304 | C1D | | 10438 |
| ENSG00000243667 | 145.633214 | -0.012913944 | 0.1172965 | 0.8318298 | 0.9304418 | DNAAF10 | | 116143 |
| ENSG00000115946 | 1178.910089 | 0.162002977 | 0.0887285 | 0.0246751 | 0.1197463 | PNO1 | | 56902 |
| ENSG00000221823 | 1428.403951 | -0.023823838 | 0.0716829 | 0.6958259 | 0.8612721 | PPP3R1 | | 5534 |
| ENSG00000273275 | 24.02162245 | 0.018811433 | 0.1340911 | 0.5741427 | NA | NA | NA | |
| ENSG00000119865 | 35.58415488 | 0.0092164 | 0.1308988 | 0.8158084 | NA | CNRIP1 | | 25927 |
| ENSG00000204923 | 100.2768166 | -0.025001097 | 0.1237123 | 0.6574598 | 0.8419413 | FBXO48 | | 554251 |
| ENSG00000169621 | 126.0502074 | 0.018655828 | 0.1216467 | 0.7464696 | 0.8896359 | APLF | | 200558 |
| ENSG00000169604 | 1545.914542 | -0.09765704 | 0.0815316 | 0.1427875 | 0.3739628 | ANTXR1 | | 84168 |
| ENSG00000198380 | 1449.463239 | -0.035421661 | 0.0718187 | 0.5621409 | 0.7800541 | GFPT1 | | 2673 |
| ENSG00000169599 | 332.8567159 | 0.003789908 | 0.1017231 | 0.9563806 | 0.9825402 | NFU1 | | 27247 |
| ENSG00000188971 | 836.0186029 | -0.009101462 | 0.083791 | 0.868961 | 0.9471245 | NA | NA | |
| ENSG00000115977 | 291.9815533 | 0.095084615 | 0.1237127 | 0.212118 | 0.468827 | AAK1 | | 22848 |
| ENSG00000196975 | 1047.225847 | -0.023596719 | 0.081366 | 0.7194941 | 0.8732763 | ANXA4 | | 307 |
| ENSG00000087338 | 634.6968611 | -0.057091414 | 0.09284 | 0.4124714 | 0.6665476 | GMCL1 | | 64395 |
| ENSG00000124380 | 547.858032 | 0.013531072 | 0.0925847 | 0.8560495 | 0.9418544 | SNRNP27 | | 11017 |
| ENSG00000059728 | 142.0752693 | 0.519911471 | 0.2648392 | 0.0023307 | 0.0212865 | MXD1 | | 4084 |
| ENSG00000179818 | 317.4355034 | -0.016334214 | 0.1043798 | 0.8086228 | 0.9204368 | PCBP1-AS1 | | 400960 |
| ENSG00000169564 | 4968.062684 | 0.012606706 | 0.0570405 | 0.8340913 | 0.9305701 | PCBP1 | | 5093 |
| ENSG00000115998 | 116.9674127 | 0.062907796 | 0.1346063 | 0.312159 | 0.5818701 | C2orf42 | | 54980 |
| ENSG00000116001 | 1467.152692 | -0.100902411 | 0.0807892 | 0.1280864 | 0.351634 | TIA1 | | 7072 |
| ENSG00000116005 | 1818.610708 | -0.275270549 | 0.0791404 | 8.75E-05 | 0.0015646 | PCYOX1 | | 51449 |
| ENSG00000143977 | 2765.547742 | -0.126872864 | 0.0698759 | 0.0362405 | 0.1568165 | SNRPG | | 6637 |
| ENSG00000035141 | 2127.881836 | -0.204061702 | 0.0733608 | 0.0015202 | 0.0155358 | FAM136A | | 84908 |
| ENSG00000233849 | 10.23098964 | -0.026648042 | 0.1387087 | 0.2243045 | NA | NA | NA | |
| ENSG00000163235 | 1083.497032 | 0.014007636 | 0.0828453 | 0.8326001 | 0.9305286 | TGFA | | 7039 |
| ENSG00000116035 | 10.43171519 | 0.01271345 | 0.1355876 | 0.5764069 | NA | VAX2 | | 25806 |
| ENSG00000144043 | 2006.347052 | -0.059516972 | 0.0688838 | 0.3168711 | 0.5853754 | TEX261 | | 113419 |
| ENSG00000124357 | 819.4054283 | -0.021441376 | 0.0836407 | 0.7454525 | 0.8893165 | NAGK | | 55577 |
| ENSG00000124370 | 58.76529325 | 0.018713158 | 0.1287402 | 0.6916829 | 0.8594028 | MCEE | | 84693 |
| ENSG00000124383 | 973.8237423 | -0.007619391 | 0.0784062 | 0.904755 | 0.962269 | MPHOSPH1 | | 10199 |
| ENSG00000124374 | 69.39305216 | 0.056760338 | 0.1393952 | 0.2881527 | 0.5578243 | PAIP2B | | 400961 |
| ENSG00000075292 | 2367.697882 | 0.019232434 | 0.0675443 | 0.744797 | 0.8891134 | ZNF638 | | 27332 |
| ENSG00000135636 | 42.19850741 | 0.116721394 | 0.2056217 | 0.0253584 | 0.1221896 | DYSF | | 8291 |
| ENSG00000003137 | 626.4834755 | -0.038854983 | 0.0894418 | 0.5608746 | 0.7795039 | CYP26B1 | | 56603 |
| ENSG00000144036 | 601.0863232 | -0.014709953 | 0.0902796 | 0.8272862 | 0.9287505 | EXOC6B | | 23233 |
| ENSG00000116096 | 1758.583836 | -0.021051889 | 0.0689122 | 0.7225717 | 0.8747394 | SPR | | 6697 |
| ENSG00000144040 | 455.6092481 | -0.039424656 | 0.0979758 | 0.5715546 | 0.7864627 | SFXN5 | | 94097 |
| ENSG00000135631 | 1008.395309 | 0.193053544 | 0.0945336 | 0.0114059 | 0.069784 | RAB11FIP5 | | 26056 |
| ENSG00000272702 | 9.943870225 | 0.026364005 | 0.1386335 | 0.2317684 | NA | NA | NA | |
| ENSG00000135632 | 1257.03634 | 0.011042364 | 0.0765911 | 0.8627295 | 0.9450945 | SMYD5 | | 10322 |
| ENSG00000135617 | 254.2018107 | 0.011521287 | 0.1076668 | 0.8594297 | 0.9433297 | PRADC1 | | 84279 |
| ENSG00000135624 | 11672.11856 | -0.144318218 | 0.057717 | 0.0059253 | 0.0430703 | CCT7 | | 10574 |
| ENSG00000163013 | 574.5741641 | 0.190605018 | 0.1268871 | 0.0312524 | 0.1419912 | FBXO41 | | 150726 |
| ENSG00000135625 | 19.91842592 | 0.080562498 | 0.1695439 | 0.0188575 | NA | EGR4 | | 1961 |
| ENSG00000116127 | 930.0847588 | 0.02781972 | 0.0826411 | 0.674012 | 0.8501975 | ALMS1 | | 7840 |
| ENSG00000230002 | 27.30223437 | 0.048960086 | 0.14408 | 0.1820577 | NA | ALMS1-IT1 | | 100874291 |
| ENSG00000144034 | 514.7420169 | -0.082357996 | 0.10186 | 0.2587015 | 0.5253712 | TPRKB | | 51002 |
| ENSG00000144048 | 567.075411 | 0.046097125 | 0.0930358 | 0.5067242 | 0.7392339 | DUSP11 | | 8446 |
| ENSG00000124356 | 769.3299512 | 0.235102211 | 0.1100903 | 0.0062648 | 0.0449783 | STAMBP | | 10617 |

| | | | | | | | | |
|---|---|---|---|---|---|---|---|---|
| ENSG00000114956 | 713.2126707 | 0.058760182 | 0.0900155 | 0.3944007 | 0.6549057 | DGUOK | | 1716 |
| ENSG00000237883 | 40.15292711 | -0.020260268 | 0.1318188 | 0.6177777 | NA | NA | NA | |
| ENSG00000235499 | 33.91781812 | 0.0220734 | 0.1333301 | 0.5620746 | NA | NA | NA | |
| ENSG00000187605 | 766.9898715 | 0.110822919 | 0.0973628 | 0.1348577 | 0.3616363 | TET3 | | 200424 |
| ENSG00000163170 | 455.7234703 | 0.067221112 | 0.1067438 | 0.3381433 | 0.6057127 | BOLA3 | | 388962 |
| ENSG00000225439 | 40.94613762 | -0.03217554 | 0.1346894 | 0.4383946 | 0.6881889 | BOLA3-DT | | 100507171 |
| ENSG00000114978 | 3834.92462 | -0.315456257 | 0.0646703 | 1.60E-07 | 7.14E-06 | MOB1A | | 55233 |
| ENSG00000065911 | 4868.506128 | 0.348424311 | 0.0646224 | 9.08E-09 | 5.52E-07 | MTHFD2 | | 10797 |
| ENSG00000264324 | 89.03436259 | 0.109438779 | 0.1800103 | 0.0814721 | 0.2654605 | NA | NA | |
| ENSG00000188687 | 10.55197979 | -0.008227918 | 0.1355112 | 0.6807498 | NA | SLC4A5 | | 57835 |
| ENSG00000204843 | 4089.031261 | -0.051557712 | 0.0577175 | 0.3236492 | 0.5924692 | DCTN1 | | 1639 |
| ENSG00000159239 | 37.19910857 | 0.135001132 | 0.2398706 | 0.0138472 | NA | NA | NA | |
| ENSG00000005448 | 608.0369378 | 0.002435627 | 0.0908585 | 0.9723706 | 0.9893759 | WDR54 | | 84058 |
| ENSG00000114993 | 245.9608067 | -0.014485645 | 0.1084984 | 0.8262499 | 0.9286016 | RTKN | | 6242 |
| ENSG00000115274 | 419.2457885 | 0.416467473 | 0.1459552 | 0.0003523 | 0.0049342 | INO80B | | 83444 |
| ENSG00000239779 | 125.2991938 | -0.074486706 | 0.1382565 | 0.2539813 | 0.5211409 | WBP1 | | 23559 |
| ENSG00000115275 | 1228.538649 | -0.030303781 | 0.0788973 | 0.6397963 | 0.8306614 | MOGS | | 7841 |
| ENSG00000204822 | 68.29051345 | 0.051438945 | 0.1369244 | 0.3295637 | 0.5977692 | MRPL53 | | 116540 |
| ENSG00000135637 | 305.1293361 | 0.015518619 | 0.1033285 | 0.8205311 | 0.9260506 | CCDC142 | | 84865 |
| ENSG00000115282 | 511.4466085 | -0.401614967 | 0.1347808 | 0.0002504 | 0.0037112 | TTC31 | | 64427 |
| ENSG00000179528 | 110.0166797 | 0.173980335 | 0.2326602 | 0.0463684 | 0.1841023 | LBX2 | | 85474 |
| ENSG00000257702 | 131.6547153 | 0.249557432 | 0.2737132 | 0.0253668 | 0.1221896 | LBX2-AS1 | | 151534 |
| ENSG00000115289 | 179.3851989 | -0.003586011 | 0.1170334 | 0.9513275 | 0.9805393 | PCGF1 | | 84759 |
| ENSG00000115307 | 2333.54664 | 0.050587525 | 0.0650639 | 0.3734575 | 0.6352333 | AUP1 | | 550 |
| ENSG00000115317 | 578.2518614 | 0.090681879 | 0.1020073 | 0.2194102 | 0.4793377 | HTRA2 | | 27429 |
| ENSG00000115318 | 52.00954428 | 0.017748777 | 0.129503 | 0.6950358 | 0.8606228 | LOXL3 | | 84695 |
| ENSG00000115325 | 135.2165484 | 0.029781637 | 0.1203615 | 0.6273048 | 0.8222551 | DOK1 | | 1796 |
| ENSG00000135622 | 464.6359756 | -0.160367551 | 0.1264856 | 0.0595475 | 0.2170464 | SEMA4F | | 10505 |
| ENSG00000272711 | 34.56476903 | 0.046242479 | 0.1414011 | 0.2463629 | NA | NA | NA | |
| ENSG00000159399 | 739.329488 | 0.601097383 | 0.1108388 | 4.06E-09 | 2.67E-07 | HK2 | | 3099 |
| ENSG00000115350 | 521.10025 | 0.118038805 | 0.1098168 | 0.1301877 | 0.3545827 | POLE4 | | 56655 |
| ENSG00000115363 | 1235.391931 | 0.177897381 | 0.0982207 | 0.021521 | 0.1092433 | EVA1A | | 84141 |
| ENSG00000115364 | 1385.040541 | -0.224578406 | 0.0849601 | 0.0018924 | 0.0181636 | MRPL19 | | 9801 |
| ENSG00000005436 | 470.1617967 | 0.057791297 | 0.0995684 | 0.4169456 | 0.6704765 | GCFC2 | | 6936 |
| ENSG00000270696 | 129.0246087 | -0.0367824 | 0.1230831 | 0.5441324 | 0.766246 | NA | NA | |
| ENSG00000271452 | 10.53194351 | 0.017667562 | 0.1364336 | 0.4342376 | NA | NA | NA | |
| ENSG00000163541 | 1583.570272 | -0.016577603 | 0.0696285 | 0.7812686 | 0.9091865 | SUCLG1 | | 8802 |
| ENSG00000186854 | 58.24905492 | 0.224957433 | 0.4664421 | 0.0144856 | 0.0822586 | TRABD2A | | 129293 |
| ENSG00000034510 | 20552.7538 | -0.029112465 | 0.0957133 | 0.6723348 | 0.8499086 | TMSB10 | | 9168 |
| ENSG00000176407 | 1855.571244 | -0.093911427 | 0.0728691 | 0.12949 | 0.353901 | KCMF1 | | 56888 |
| ENSG00000152284 | 178.4832845 | -0.030920447 | 0.1166918 | 0.6296452 | 0.8236154 | TCF7L1 | | 83439 |
| ENSG00000152291 | 5839.428946 | -0.021095388 | 0.0544292 | 0.6719999 | 0.8499086 | TGOLN2 | | 10618 |
| ENSG00000042445 | 966.2852306 | 0.144812925 | 0.092126 | 0.0475005 | 0.1861923 | RETSAT | | 54884 |
| ENSG00000115459 | 351.3039862 | 0.121339074 | 0.1320939 | 0.1330975 | 0.3595479 | ELMOD3 | | 84173 |
| ENSG00000042493 | 278.2443057 | 0.512138798 | 0.1888941 | 0.0004029 | 0.0054531 | CAPG | | 822 |
| ENSG00000168906 | 7790.964768 | -0.120239409 | 0.0696855 | 0.0440738 | 0.1780172 | MAT2A | | 4144 |
| ENSG00000115486 | 847.4110672 | -0.091834816 | 0.0896633 | 0.1881827 | 0.4389939 | GGCX | | 2677 |
| ENSG00000118640 | 241.9889163 | 0.016358681 | 0.1106505 | 0.8042948 | 0.9186624 | VAMP8 | | 8673 |
| ENSG00000168894 | 519.1090951 | 0.007948734 | 0.0921342 | 0.9081376 | 0.9638438 | RNF181 | | 51255 |
| ENSG00000168890 | 141.3913621 | 0.053349615 | 0.1266339 | 0.4049775 | 0.6614808 | TMEM150A | | 129303 |
| ENSG00000168883 | 1550.877289 | 0.123228026 | 0.0833787 | 0.0706346 | 0.2420798 | USP39 | | 10713 |
| ENSG00000168887 | 289.3860331 | 0.020051427 | 0.1051913 | 0.7644327 | 0.898595 | C2orf68 | | 388969 |
| ENSG00000115525 | 27.66149355 | -0.013065587 | 0.1326927 | 0.7056311 | NA | ST3GAL5 | | 8869 |
| ENSG00000068654 | 3659.989579 | 0.069061475 | 0.0601989 | 0.2016199 | 0.4556244 | POLR1A | | 25885 |
| ENSG00000132300 | 1460.561998 | -0.114951275 | 0.0785364 | 0.0794774 | 0.2610494 | PTCD3 | | 55037 |
| ENSG00000208772 | 14.42783152 | 0.004167511 | 0.1343531 | 0.8699546 | NA | SNORD94 | | 692225 |
| ENSG00000132305 | 2305.905421 | -0.142413492 | 0.0697308 | 0.0188884 | 0.0997748 | IMMT | | 10989 |
| ENSG00000273080 | 9.162341513 | -0.010684822 | 0.1356466 | 0.6052603 | NA | NA | NA | |
| ENSG00000132313 | 1066.384053 | -0.109542639 | 0.0853233 | 0.1100494 | 0.3212914 | MRPL35 | | 51318 |
| ENSG00000115548 | 934.141351 | 0.220473518 | 0.1029631 | 0.0070687 | 0.0491193 | KDM3A | | 55818 |
| ENSG00000115561 | 1351.91253 | -0.055458026 | 0.0748067 | 0.3770704 | 0.6388974 | CHMP3 | | 51652 |
| ENSG00000228363 | 31.63491373 | -0.052342689 | 0.1449195 | 0.1819713 | NA | CHMP3-AS1 | | 105374845 |
| ENSG00000239305 | 380.7019322 | -0.021810727 | 0.1004436 | 0.7421345 | 0.8876265 | RNF103 | | 7844 |
| ENSG00000153561 | 875.7607078 | 0.016238706 | 0.08132 | 0.8047305 | 0.9186624 | RMND5A | | 64795 |
| ENSG00000231259 | 161.1666127 | 0.12244534 | 0.1599453 | 0.1179002 | 0.3345134 | NA | NA | |
| ENSG00000204745 | 57.04127803 | -0.032626154 | 0.1322806 | 0.4867102 | 0.7239605 | LOC1027246 | | 102724642 |
| ENSG00000222041 | 697.3324285 | -0.030479462 | 0.0862584 | 0.6540628 | 0.8399571 | CYTOR | | 112597 |
| ENSG00000234231 | 50.81489865 | -0.027573936 | 0.1315474 | 0.542032 | 0.7652256 | ANAPC1P4 | | 730268 |
| ENSG00000172086 | 48.0866879 | 0.012058629 | 0.1293218 | 0.7835962 | 0.9098649 | KRCC1 | | 51315 |
| ENSG00000172071 | 315.3507178 | -0.065503387 | 0.1121079 | 0.3615875 | 0.625071 | EIF2AK3 | | 9451 |

| | | | | | | | |
|---|---|---|---|---|---|---|---|
| ENSG00000234028 | 10.82757409 | -0.00530826 | 0.13484 | 0.8134133 | NA | EIF2AK3-DT | 101928403 |
| ENSG00000153574 | 837.4705043 | 0.000807596 | 0.0815978 | 0.9910053 | 0.9962077 | RPIA | 22934 |
| ENSG00000143429 | 425.8897718 | 0.084049802 | 0.1107289 | 0.2592074 | 0.525883 | LSP1P4 | 654342 |
| ENSG00000232531 | 13.01414123 | 0.00665829 | 0.1344954 | 0.7929538 | NA | KMT5AP2 | 100421788 |
| ENSG00000144029 | 2296.101722 | -0.055682747 | 0.0674037 | 0.3451648 | 0.6114193 | MRPS5 | 64969 |
| ENSG00000144026 | 304.1320999 | 0.010963327 | 0.1029089 | 0.8727766 | 0.9484907 | ZNF514 | 84874 |
| ENSG00000163067 | 138.4031324 | 0.15891569 | 0.2020055 | 0.0631964 | 0.2254036 | NA | NA |
| ENSG00000233757 | 29.88386965 | -0.025971939 | 0.1348251 | 0.4745245 | NA | ZNF892 | 344065 |
| ENSG00000115042 | 489.339076 | 0.080975855 | 0.1053395 | 0.2717577 | 0.5394882 | FAHD2A | 51011 |
| ENSG00000229689 | 10.27262649 | 0.015508837 | 0.1361146 | 0.4850604 | NA | ANKRD33BI | 100419920 |
| ENSG00000232931 | 178.7371352 | -0.018793478 | 0.1160067 | 0.7620983 | 0.8980198 | NA | NA |
| ENSG00000174501 | 223.9977268 | -0.081937976 | 0.126483 | 0.2600994 | 0.5271028 | ANKRD36C | 400986 |
| ENSG00000231584 | 38.07110477 | 0.056760739 | 0.1465435 | 0.1727471 | NA | NA | NA |
| ENSG00000158050 | 35.34730694 | 0.100237944 | 0.1848746 | 0.0345104 | NA | DUSP2 | 1844 |
| ENSG00000084090 | 3294.380593 | -0.068473406 | 0.0613982 | 0.2111002 | 0.4679229 | STARD7 | 56910 |
| ENSG00000204685 | 86.6428223 | 0.14076398 | 0.2094763 | 0.0570615 | 0.2111963 | NA | NA |
| ENSG00000135956 | 1442.203877 | -0.110809975 | 0.0842627 | 0.1044398 | 0.3104023 | TMEM127 | 55654 |
| ENSG00000144021 | 2071.950663 | 0.092097863 | 0.0692472 | 0.1246434 | 0.3465814 | CIAO1 | 9391 |
| ENSG00000144028 | 8141.413057 | -0.118507515 | 0.0556471 | 0.0192667 | 0.1010111 | SNRNP200 | 23020 |
| ENSG00000230747 | 10.92400632 | 0.039095163 | 0.1430649 | 0.0922436 | NA | NA | NA |
| ENSG00000121152 | 1893.514523 | -0.219991327 | 0.0778207 | 0.0011556 | 0.0125573 | NCAPH | 23397 |
| ENSG00000163121 | 26.30555084 | -0.015832765 | 0.1342378 | 0.6065886 | NA | NEURL3 | 93082 |
| ENSG00000196843 | 221.2203584 | 0.554342837 | 0.2007531 | 0.0003271 | 0.004637 | ARID5A | 10865 |
| ENSG00000114982 | 1758.969138 | 0.12424037 | 0.0801263 | 0.0620653 | 0.2224145 | KANSL3 | 55683 |
| ENSG00000114988 | 591.1416352 | -0.08785773 | 0.0995242 | 0.2271521 | 0.4882146 | LMAN2L | 81562 |
| ENSG00000158158 | 520.2698588 | 0.076619322 | 0.1009604 | 0.3071317 | 0.5764148 | CNNM4 | 26504 |
| ENSG00000168763 | 623.4815998 | -0.057290246 | 0.0985623 | 0.418271 | 0.6712218 | CNNM3 | 26505 |
| ENSG00000163126 | 17.57198793 | 0.011610188 | 0.134216 | 0.6864344 | NA | ANKRD23 | 200539 |
| ENSG00000213337 | 146.4791275 | -0.022933969 | 0.1181046 | 0.7050555 | 0.8666859 | ANKRD39 | 51239 |
| ENSG00000168758 | 619.1094281 | -0.14984796 | 0.1088565 | 0.0597652 | 0.2174144 | SEMA4C | 54910 |
| ENSG00000144199 | 228.3677374 | 0.088532894 | 0.1284215 | 0.2325849 | 0.4951049 | FAHD2B | 151313 |
| ENSG00000135976 | 324.298651 | -0.046826962 | 0.1087291 | 0.4921436 | 0.7283326 | ANKRD36 | 375248 |
| ENSG00000230606 | 85.64868642 | -0.009800567 | 0.1235661 | 0.8529415 | 0.9400141 | NA | NA |
| ENSG00000196912 | 139.7656064 | -0.023725257 | 0.1190104 | 0.6963316 | 0.8615453 | ANKRD36B | 57730 |
| ENSG00000135940 | 1657.702731 | -0.164619233 | 0.0849621 | 0.0189068 | 0.0997748 | COX5B | 1329 |
| ENSG00000115073 | 703.6581404 | 0.054799545 | 0.0897103 | 0.4225711 | 0.6745232 | ACTR1B | 10120 |
| ENSG00000228486 | 17.95260337 | 0.026904871 | 0.1373063 | 0.3523951 | NA | C2orf92 | 728537 |
| ENSG00000075568 | 1211.410465 | -0.056814499 | 0.0766051 | 0.3720188 | 0.6339958 | TMEM131 | 23505 |
| ENSG00000235734 | 12.70140194 | 0.011365129 | 0.1352235 | 0.6329094 | NA | NA | NA |
| ENSG00000040933 | 723.1288892 | -0.099571928 | 0.0958132 | 0.1696042 | 0.4153346 | INPP4A | 3631 |
| ENSG00000183513 | 387.3366208 | -0.084243133 | 0.110571 | 0.257033 | 0.5240334 | COA5 | 493753 |
| ENSG00000115446 | 410.3001961 | -0.137340123 | 0.1262631 | 0.0956981 | 0.2938616 | UNC50 | 25972 |
| ENSG00000071073 | 45.48578005 | -0.005913005 | 0.1290763 | 0.8884398 | 0.9545517 | MGAT4A | 11320 |
| ENSG00000135951 | 123.5435481 | 0.014711848 | 0.1192584 | 0.8052749 | 0.9186624 | TSGA10 | 80705 |
| ENSG00000241962 | 8.614150818 | -0.017428675 | 0.1367381 | 0.3921129 | NA | NA | NA |
| ENSG00000273045 | 14.98374262 | -0.029578562 | 0.1386597 | 0.2654094 | NA | C2orf15 | 150590 |
| ENSG00000144182 | 85.81945814 | 0.04951488 | 0.1361652 | 0.3452025 | 0.6114193 | LIPT1 | 51601 |
| ENSG00000158411 | 475.4258939 | -0.025282834 | 0.0950707 | 0.7115762 | 0.869266 | MITD1 | 129531 |
| ENSG00000144214 | 18.64245082 | -0.0532912 | 0.1485023 | 0.0856278 | NA | LYG1 | 129530 |
| ENSG00000115514 | 835.2370733 | -0.064554503 | 0.0888356 | 0.348389 | 0.6143697 | TXNDC9 | 10190 |
| ENSG00000158417 | 4913.856471 | -0.16210918 | 0.0590972 | 0.0025106 | 0.0225068 | EIF5B | 9669 |
| ENSG00000135945 | 744.1394832 | -0.099420492 | 0.1002942 | 0.1776613 | 0.4270449 | REV1 | 51455 |
| ENSG00000273306 | 26.31211901 | 0.019117201 | 0.1337979 | 0.5816619 | NA | NA | NA |
| ENSG00000115526 | 567.722494 | 0.044550118 | 0.0931239 | 0.520782 | 0.7489848 | CHST10 | 9486 |
| ENSG00000115539 | 613.1378072 | -0.058788219 | 0.093133 | 0.4000807 | 0.6583091 | PDCL3 | 79031 |
| ENSG00000170485 | 169.0149544 | 0.056022264 | 0.1245089 | 0.3978073 | 0.6570683 | NPAS2 | 4862 |
| ENSG00000071082 | 7393.338788 | -0.258784872 | 0.0643368 | 1.16E-05 | 0.0002866 | RPL31 | 6160 |
| ENSG00000204634 | 1037.737536 | -0.065165906 | 0.080937 | 0.3220438 | 0.5908703 | TBC1D8 | 11138 |
| ENSG00000272902 | 15.4048661 | 0.011875128 | 0.1347103 | 0.6567399 | NA | TBC1D8-AS | 100506286 |
| ENSG00000158435 | 1023.571642 | -0.029894731 | 0.0783515 | 0.6413012 | 0.8315061 | CNOT11 | 55571 |
| ENSG00000163162 | 718.1379977 | 0.047441918 | 0.0874048 | 0.4855402 | 0.7234177 | RNF149 | 284996 |
| ENSG00000071054 | 2998.884453 | -0.008821939 | 0.062454 | 0.8759159 | 0.9497329 | MAP4K4 | 9448 |
| ENSG00000115590 | 22.07836398 | 0.033297285 | 0.1384951 | 0.3024725 | NA | IL1R2 | 7850 |
| ENSG00000115594 | 312.7876781 | -0.011957109 | 0.130303 | 0.7688946 | 0.9009387 | IL1R1 | 3554 |
| ENSG00000115602 | 36.41362708 | 0.117922658 | 0.2091238 | 0.0212611 | NA | IL1RL1 | 9173 |
| ENSG00000115604 | 42.54487578 | -0.019816703 | 0.1312846 | 0.6351255 | 0.8266184 | IL18R1 | 8809 |
| ENSG00000115616 | 29.90147433 | -0.027635652 | 0.1353982 | 0.4397002 | NA | SLC9A2 | 6549 |
| ENSG00000135953 | 456.5911138 | 0.020741463 | 0.0953626 | 0.7625398 | 0.8982682 | MFSD9 | 84804 |
| ENSG00000170417 | 111.3096094 | 0.334905848 | 0.3613393 | 0.0142523 | 0.0814886 | TMEM182 | 130827 |
| ENSG00000135972 | 912.4686655 | -0.111224395 | 0.0900342 | 0.1159699 | 0.3320889 | MRPS9 | 64965 |

| | | | | | | | | |
|---|---|---|---|---|---|---|---|---|
| ENSG00000231851 | 8.707613606 | -0.004868752 | 0.1352189 | 0.8092542 | NA | MRPS9-AS1 | 101927492 |
| ENSG00000135966 | 1112.451963 | -0.103822339 | 0.0854782 | 0.1301948 | 0.3545827 | TGFBRAP1 | 9392 |
| ENSG00000272994 | 56.07165043 | 0.003650907 | 0.1272975 | 0.9390604 | 0.9763786 | NA | NA |
| ENSG00000135974 | 570.5496669 | 0.239835832 | 0.1277021 | 0.0102568 | 0.064518 | C2orf49 | 79074 |
| ENSG00000115641 | 3786.721709 | 0.433345033 | 0.066908 | 9.43E-12 | 9.66E-10 | FHL2 | 2274 |
| ENSG00000071051 | 589.1441671 | -0.026788673 | 0.0917588 | 0.6945517 | 0.8605415 | NCK2 | 8440 |
| ENSG00000115652 | 953.1179017 | -0.04682665 | 0.081121 | 0.4740267 | 0.7140485 | UXS1 | 80146 |
| ENSG00000153165 | 9.862706072 | -0.014544182 | 0.1360711 | 0.4954373 | NA | RGPD3 | 653489 |
| ENSG00000135968 | 903.9819899 | 0.055726316 | 0.0831689 | 0.3993915 | 0.6582409 | GCC2 | 9648 |
| ENSG00000214184 | 11.21002154 | 0.004597327 | 0.1349475 | 0.8366938 | NA | GCC2-AS1 | 644903 |
| ENSG00000169756 | 1484.747611 | -0.043711415 | 0.0727323 | 0.4783847 | 0.7175397 | LIMS1 | 3987 |
| ENSG00000153201 | 4036.918164 | -0.057336815 | 0.0584991 | 0.2718195 | 0.5394882 | RANBP2 | 5903 |
| ENSG00000163006 | 406.0652989 | -0.238100528 | 0.1433604 | 0.0150708 | 0.0844328 | CCDC138 | 165055 |
| ENSG00000227470 | 9.402081148 | 0.00582259 | 0.1350683 | 0.7904697 | NA | RPL39P16 | 100271023 |
| ENSG00000186522 | 2332.760013 | -0.089863054 | 0.0692144 | 0.1330262 | 0.3595242 | SEPTIN10 | 151011 |
| ENSG00000198142 | 700.9281118 | -0.209764034 | 0.1118641 | 0.0135141 | 0.0785756 | SOWAHC | 65124 |
| ENSG00000015568 | 63.06636551 | -0.005914732 | 0.1263568 | 0.9011781 | 0.9612514 | RGPD5 | 84220 |
| ENSG00000204588 | 9.692683224 | 0.004989031 | 0.1351201 | 0.8146282 | NA | LINC01123 | 440894 |
| ENSG00000144063 | 839.1641328 | 0.049139335 | 0.094889 | 0.4820258 | 0.7211248 | MALL | 7851 |
| ENSG00000144061 | 157.0513003 | -0.045978225 | 0.1218044 | 0.4761184 | 0.7156114 | NPHP1 | 4867 |
| ENSG00000175701 | 206.7421805 | -0.013561509 | 0.1120631 | 0.8326442 | 0.9305286 | MTLN | 205251 |
| ENSG00000261760 | 12.29885526 | -0.02482511 | 0.1377854 | 0.3024334 | NA | NA | NA |
| ENSG00000175772 | 19.05757368 | 0.007258649 | 0.1335327 | 0.8080021 | NA | NA | NA |
| ENSG00000257207 | 23.41629115 | -0.003507664 | 0.1326906 | 0.9111748 | NA | NA | NA |
| ENSG00000183054 | 24.02518286 | 0.008637131 | 0.1327964 | 0.7941978 | NA | RGPD6 | 729540 |
| ENSG00000169679 | 3112.010604 | -0.37117336 | 0.0820452 | 7.07E-07 | 2.56E-05 | BUB1 | 699 |
| ENSG00000153094 | 413.3300674 | 0.242320798 | 0.1421906 | 0.0136584 | 0.0792568 | BCL2L11 | 10018 |
| ENSG00000172965 | 831.4423992 | -0.069694231 | 0.0866038 | 0.304241 | 0.5731573 | MIR4435-2H | 541471 |
| ENSG00000227992 | 60.02966184 | -0.024969105 | 0.1298959 | 0.596562 | 0.804484 | NA | NA |
| ENSG00000271590 | 10.25180806 | -0.005294394 | 0.1349541 | 0.8087226 | NA | NA | NA |
| ENSG00000153107 | 2033.724248 | -0.146380916 | 0.0725631 | 0.019337 | 0.1012441 | ANAPC1 | 64682 |
| ENSG00000153208 | 491.5674762 | -0.017426539 | 0.0956951 | 0.7981968 | 0.9162772 | MERTK | 10461 |
| ENSG00000153214 | 712.7701772 | 0.024344516 | 0.0856369 | 0.71677 | 0.8716908 | TMEM87B | 84910 |
| ENSG00000144161 | 254.2604797 | 0.257464468 | 0.1912798 | 0.019247 | 0.1009539 | ZC3H8 | 84524 |
| ENSG00000188177 | 82.33068382 | 0.697741231 | 0.3492928 | 0.0016832 | 0.0167204 | ZC3H6 | 376940 |
| ENSG00000169629 | 148.437427 | 0.00202113 | 0.1157465 | 0.9749388 | 0.9905401 | RGPD8 | 727851 |
| ENSG00000114999 | 1860.362628 | 0.040138946 | 0.0699243 | 0.5061465 | 0.7387601 | TTL | 150465 |
| ENSG00000125630 | 1706.825403 | 0.147392831 | 0.0763509 | 0.023224 | 0.1149488 | POLR1B | 84172 |
| ENSG00000125611 | 149.2896313 | -0.013511537 | 0.1174368 | 0.8240906 | 0.9274924 | CHCHD5 | 84269 |
| ENSG00000237753 | 17.69154071 | -0.009652541 | 0.1341892 | 0.7242625 | NA | NA | NA |
| ENSG00000144136 | 2353.218299 | 0.312085359 | 0.0857205 | 3.88E-05 | 0.0007999 | SLC20A1 | 6574 |
| ENSG00000169607 | 1937.813581 | 0.093428209 | 0.0703422 | 0.1233884 | 0.3443144 | CKAP2L | 150468 |
| ENSG00000125637 | 636.9153005 | -0.044107657 | 0.0921104 | 0.519761 | 0.7483099 | PSD4 | 23550 |
| ENSG00000189223 | 167.7425371 | 0.114753104 | 0.1570191 | 0.1317048 | 0.357196 | PAX8-AS1 | 654433 |
| ENSG00000125618 | 4095.359351 | -0.090979946 | 0.1174808 | 0.2246207 | 0.4852777 | PAX8 | 7849 |
| ENSG00000136682 | 471.6253031 | 0.103104839 | 0.1119934 | 0.1785015 | 0.4275952 | ZNG1B | 150472 |
| ENSG00000184492 | 18.72648943 | 0.042843194 | 0.1431081 | 0.1549684 | NA | FOXD4L1 | 200350 |
| ENSG00000146556 | 310.096991 | 0.024017319 | 0.1037347 | 0.7264845 | 0.8776165 | NA | NA |
| ENSG00000236397 | 41.1730654 | -0.007674064 | 0.1311054 | 0.8375108 | 0.9329547 | NA | NA |
| ENSG00000240356 | 226.7605386 | 0.046252671 | 0.1161345 | 0.4957603 | 0.7310915 | NA | NA |
| ENSG00000144134 | 121.5825813 | 0.028954806 | 0.1216673 | 0.628784 | 0.823051 | RABL2A | 11159 |
| ENSG00000227359 | 74.26916263 | 0.015710513 | 0.1260296 | 0.7585001 | 0.8963508 | NA | NA |
| ENSG00000115084 | 1952.117648 | -0.32690143 | 0.0850103 | 1.60E-05 | 0.0003767 | SLC35F5 | 80255 |
| ENSG00000270019 | 9.352827499 | 0.002693228 | 0.1352729 | 0.8927312 | NA | NA | NA |
| ENSG00000115091 | 8221.763643 | -0.211947115 | 0.0592397 | 9.51E-05 | 0.0016726 | ACTR3 | 10096 |
| ENSG00000234199 | 8.532648617 | -0.003443035 | 0.1353294 | 0.855677 | NA | LINC01191 | 440900 |
| ENSG00000088205 | 3248.764025 | -0.055421252 | 0.0640458 | 0.3260149 | 0.5942836 | DDX18 | 8886 |
| ENSG00000236255 | 122.0267833 | 0.779678488 | 0.2687622 | 0.0001669 | 0.0026376 | NA | NA |
| ENSG00000125633 | 1931.736865 | 0.063406819 | 0.0722374 | 0.3002645 | 0.5699365 | CCDC93 | 54520 |
| ENSG00000125629 | 269.5625342 | 0.016731153 | 0.1059675 | 0.8064008 | 0.9194308 | INSIG2 | 51141 |
| ENSG00000115107 | 906.5905098 | -0.101681299 | 0.0899301 | 0.1488672 | 0.3836131 | STEAP3 | 55240 |
| ENSG00000186132 | 120.4566964 | -0.061010194 | 0.1322921 | 0.3314945 | 0.5992244 | C2orf76 | 130355 |
| ENSG00000155368 | 2061.845177 | -0.406058894 | 0.0777084 | 1.80E-08 | 1.02E-06 | DBI | 1622 |
| ENSG00000144120 | 271.5327536 | 0.093377285 | 0.1249639 | 0.2195641 | 0.479478 | TMEM177 | 80775 |
| ENSG00000088179 | 351.2109912 | -0.065988074 | 0.1137058 | 0.3580652 | 0.6212952 | PTPN4 | 5775 |
| ENSG00000115109 | 789.4194207 | -0.216079012 | 0.1053166 | 0.0088778 | 0.0586555 | EPB41L5 | 57669 |
| ENSG00000226479 | 862.4381809 | 0.082556926 | 0.0872826 | 0.2318731 | 0.4941635 | TMEM185B | 79134 |
| ENSG00000144118 | 1818.586249 | -0.141992488 | 0.0753221 | 0.0268248 | 0.1272591 | RALB | 5899 |
| ENSG00000163083 | 34.35484635 | 0.040948551 | 0.1389403 | 0.3027313 | NA | INHBB | 3625 |
| ENSG00000074047 | 12.5924354 | 0.026781075 | 0.138165 | 0.2846943 | NA | GLI2 | 2736 |

| | | | | | | | | |
|---|---|---|---|---|---|---|---|---|
| ENSG00000115112 | 265.6079363 | 0.141578846 | 0.1460172 | 0.0921963 | 0.287262 | TFCP2L1 | | 29842 |
| ENSG00000074054 | 1381.705187 | -0.055003877 | 0.074363 | 0.3793444 | 0.6406132 | CLASP1 | | 23332 |
| ENSG00000236859 | 101.3064593 | -0.00309021 | 0.1213735 | 0.9549894 | 0.9820611 | NIFK-AS1 | | 254128 |
| ENSG00000155438 | 1551.146078 | 0.36971573 | 0.0980146 | 1.76E-05 | 0.0004094 | NIFK | | 84365 |
| ENSG00000211460 | 2241.616106 | -0.066063291 | 0.068419 | 0.266731 | 0.5346062 | TSN | | 7247 |
| ENSG00000136717 | 1559.985413 | -0.226389338 | 0.0910194 | 0.0028434 | 0.0247497 | BIN1 | | 274 |
| ENSG00000186684 | 319.7569098 | 0.073159442 | 0.114157 | 0.3163337 | 0.5849906 | CYP27C1 | | 339761 |
| ENSG00000163161 | 1816.741194 | 0.021413439 | 0.069039 | 0.7219049 | 0.8743675 | ERCC3 | | 2071 |
| ENSG00000169967 | 1630.534827 | -0.152381665 | 0.0786603 | 0.0215941 | 0.109337 | MAP3K2 | | 10746 |
| ENSG00000236682 | 10.67428742 | 0.004136754 | 0.1349098 | 0.8532959 | NA | NA | NA | |
| ENSG00000163166 | 1668.988377 | 0.050626033 | 0.072154 | 0.410086 | 0.6651964 | IWS1 | | 55677 |
| ENSG00000072163 | 107.99931 | 0.506294931 | 0.324797 | 0.0046374 | 0.0358262 | LIMS2 | | 55679 |
| ENSG00000136709 | 1827.026526 | -0.169752179 | 0.075742 | 0.0089895 | 0.0592587 | WDR33 | | 55339 |
| ENSG00000173349 | 118.6207218 | -0.040781933 | 0.1261319 | 0.4899082 | 0.7266817 | SFT2D3 | | 84826 |
| ENSG00000270081 | 212.5257555 | 0.004822593 | 0.1102665 | 0.942108 | 0.9774192 | NA | NA | |
| ENSG00000144231 | 764.7913316 | 0.069454285 | 0.088525 | 0.3126534 | 0.5819457 | POLR2D | | 5433 |
| ENSG00000144233 | 1204.666941 | 0.07272763 | 0.0799733 | 0.2698918 | 0.5378956 | AMMECR1L | | 83607 |
| ENSG00000272667 | 31.18182786 | -0.008836801 | 0.1314877 | 0.8095873 | NA | NA | NA | |
| ENSG00000136715 | 621.7710776 | -0.160220626 | 0.114265 | 0.0510135 | 0.1946714 | SAP130 | | 79595 |
| ENSG00000136731 | 2490.446622 | 0.031900669 | 0.0640951 | 0.5589397 | 0.7776292 | UGGT1 | | 56886 |
| ENSG00000136720 | 649.9285371 | 0.093022941 | 0.0992015 | 0.2049057 | 0.4602892 | HS6ST1 | | 9394 |
| ENSG00000180178 | 34.12456414 | 0.000703534 | 0.1309358 | 0.9877901 | NA | NA | NA | |
| ENSG00000196604 | 28.68229438 | 0.031804192 | 0.1367114 | 0.3795787 | NA | POTEF | | 728378 |
| ENSG00000152076 | 33.3730596 | -0.026220629 | 0.1340935 | 0.4960852 | NA | CCDC74B | | 91409 |
| ENSG00000136699 | 2712.367327 | -0.091699164 | 0.0649585 | 0.1083023 | 0.318502 | SMPD4 | | 55627 |
| ENSG00000152082 | 1615.932481 | -0.142073546 | 0.0812308 | 0.0349263 | 0.1525708 | MZT2B | | 80097 |
| ENSG00000136710 | 356.4450643 | -0.054504629 | 0.1054452 | 0.4416866 | 0.6908823 | CCDC115 | | 84317 |
| ENSG00000136718 | 2489.746301 | -0.044985695 | 0.0683453 | 0.446912 | 0.6944817 | IMP4 | | 92856 |
| ENSG00000072135 | 743.5269142 | -0.059392327 | 0.0884504 | 0.3859193 | 0.6466557 | PTPN18 | | 26469 |
| ENSG00000136002 | 115.5513279 | -0.004875621 | 0.1207673 | 0.9307006 | 0.972984 | ARHGEF4 | | 50649 |
| ENSG00000152102 | 4365.13742 | 0.005822757 | 0.0558085 | 0.9094621 | 0.9642758 | FAM168B | | 130074 |
| ENSG00000115762 | 3051.20883 | 0.024062672 | 0.0637249 | 0.6655616 | 0.8451381 | PLEKHB2 | | 55041 |
| ENSG00000173272 | 705.0258587 | -0.009757295 | 0.0851332 | 0.8827004 | 0.9527552 | MZT2A | | 653784 |
| ENSG00000152117 | 566.3645808 | 0.431879078 | 0.1356072 | 0.0001178 | 0.0019915 | SMPD4BP | | 150776 |
| ENSG00000163040 | 119.3599523 | 0.02834057 | 0.1225708 | 0.6296449 | 0.8236154 | CCDC74A | | 90557 |
| ENSG00000197927 | 26.49152154 | 0.035586527 | 0.1384811 | 0.3101798 | NA | NBEAP2 | | 100418881 |
| ENSG00000229203 | 9.328212986 | 0.020273896 | 0.1371916 | 0.3418412 | NA | NA | NA | |
| ENSG00000272769 | 13.76546322 | -0.037087676 | 0.1415187 | 0.1570462 | NA | NA | NA | |
| ENSG00000230992 | 9.357256417 | 0.001249418 | 0.1349266 | 0.9559941 | NA | NA | NA | |
| ENSG00000183840 | 272.5139929 | -0.362331359 | 0.1857266 | 0.0039435 | 0.0315079 | GPR39 | | 2863 |
| ENSG00000152127 | 1505.39919 | -0.016623594 | 0.0728994 | 0.7868719 | 0.9114885 | MGAT5 | | 4249 |
| ENSG00000224043 | 15.08666283 | -0.044572785 | 0.1446176 | 0.1062204 | NA | NA | NA | |
| ENSG00000082258 | 961.6764513 | 0.0549355 | 0.0813429 | 0.40103 | 0.6587585 | CCNT2 | | 905 |
| ENSG00000115839 | 1775.457677 | -0.035015844 | 0.069656 | 0.5586009 | 0.7775341 | RAB3GAP1 | | 22930 |
| ENSG00000121988 | 172.1079821 | -0.008120913 | 0.1138701 | 0.8963967 | 0.958602 | ZRANB3 | | 84083 |
| ENSG00000048991 | 1329.079001 | -0.107099964 | 0.0799028 | 0.1049174 | 0.3113474 | R3HDM1 | | 23518 |
| ENSG00000144224 | 2799.270599 | -0.122240567 | 0.065547 | 0.033929 | 0.149613 | UBXN4 | | 23190 |
| ENSG00000076003 | 2575.571423 | 0.011443339 | 0.0652767 | 0.8183029 | 0.9254567 | MCM6 | | 4175 |
| ENSG00000115866 | 2666.963823 | -0.138140958 | 0.0688853 | 0.0207152 | 0.1062688 | DARS1 | | 1615 |
| ENSG00000231890 | 28.24673424 | 0.002234757 | 0.1318411 | 0.9508913 | NA | NA | NA | |
| ENSG00000144228 | 568.9846505 | -0.068068067 | 0.1007816 | 0.3420381 | 0.6088612 | SPOPL | | 339745 |
| ENSG00000121964 | 296.9606619 | -0.277372247 | 0.1737796 | 0.0121962 | 0.0731708 | GTDC1 | | 79712 |
| ENSG00000169554 | 83.25702512 | 0.621241375 | 0.3682993 | 0.0031116 | 0.0262434 | ZEB2 | | 9839 |
| ENSG00000121989 | 162.6140086 | -0.04855169 | 0.1225742 | 0.4533471 | 0.6987417 | ACVR2A | | 92 |
| ENSG00000115947 | 707.0820676 | -0.291109917 | 0.1126691 | 0.0013954 | 0.0145245 | ORC4 | | 5000 |
| ENSG00000204406 | 176.4853096 | 0.025177848 | 0.1153691 | 0.6954121 | 0.8608636 | MBD5 | | 55777 |
| ENSG00000135999 | 765.8734789 | 0.005147855 | 0.0844913 | 0.9390843 | 0.9763786 | EPC2 | | 26122 |
| ENSG00000168280 | 657.8723196 | -0.030955883 | 0.0879801 | 0.6499546 | 0.8371507 | KIF5C | | 3800 |
| ENSG00000187123 | 112.391294 | -0.028540274 | 0.1251279 | 0.6009747 | 0.807266 | LYPD6 | | 130574 |
| ENSG00000168288 | 2062.933174 | -0.292187975 | 0.0770009 | 2.34E-05 | 0.0005156 | MMADHC | | 27249 |
| ENSG00000231969 | 11.99731904 | -0.007653212 | 0.1348175 | 0.7455879 | NA | MMADHC-D | | 101929231 |
| ENSG00000115963 | 334.5210327 | 1.153297544 | 0.1764919 | 3.26E-12 | 3.77E-10 | RND3 | | 390 |
| ENSG00000184898 | 147.4705617 | -0.021723836 | 0.1173787 | 0.722268 | 0.8744623 | RBM43 | | 375287 |
| ENSG00000123609 | 319.315088 | -0.338892994 | 0.1838012 | 0.0053897 | 0.0401516 | NMI | | 9111 |
| ENSG00000123610 | 164.1337847 | -0.281445954 | 0.2789061 | 0.0194254 | 0.1015086 | TNFAIP6 | | 7130 |
| ENSG00000080345 | 4046.965015 | -0.125478822 | 0.0632669 | 0.0256123 | 0.1230675 | RIF1 | | 55183 |
| ENSG00000183091 | 8.968667648 | 0.013055943 | 0.135923 | 0.5352551 | NA | NEB | | 4703 |
| ENSG00000162980 | 1206.334525 | -0.065443066 | 0.0792159 | 0.3138807 | 0.5827177 | ARL5A | | 26225 |
| ENSG00000182389 | 35.22636381 | 0.058134088 | 0.1476968 | 0.1576255 | NA | CACNB4 | | 785 |
| ENSG00000115145 | 698.6952379 | -0.087815425 | 0.0935929 | 0.2175758 | 0.4768298 | STAM2 | | 10254 |

| | | | | | | | |
|---|---|---|---|---|---|---|---|
| ENSG00000157827 | 3629.160498 | -0.05185847 | 0.062413 | 0.3471087 | 0.6134112 | FMNL2 | 114793 |
| ENSG00000196504 | 4119.339423 | -0.109477074 | 0.0612523 | 0.0454503 | 0.1819412 | PRPF40A | 55660 |
| ENSG00000177917 | 739.6225655 | -0.03305438 | 0.0852152 | 0.6237442 | 0.8200288 | ARL6IP6 | 151188 |
| ENSG00000226383 | 30.4021484 | -0.009514942 | 0.1319013 | 0.7897804 | NA | LINC01876 | 101929378 |
| ENSG00000153234 | 39.05650506 | 0.017919816 | 0.1321511 | 0.6450242 | NA | NR4A2 | 4929 |
| ENSG00000115159 | 1206.823673 | -0.01700574 | 0.0753158 | 0.7860842 | 0.9108874 | GPD2 | 2820 |
| ENSG00000115170 | 337.4635165 | 0.045467164 | 0.1060824 | 0.5166251 | 0.7458532 | ACVR1 | 90 |
| ENSG00000153237 | 21.96112194 | -0.009448124 | 0.1331278 | 0.7648519 | NA | CCDC148 | 130940 |
| ENSG00000144283 | 2438.751667 | -0.273058821 | 0.0736604 | 3.76E-05 | 0.0007814 | PKP4 | 8502 |
| ENSG00000204380 | 13.65120786 | -0.006672468 | 0.1345339 | 0.7867131 | NA | PKP4-AS1 | 100129029 |
| ENSG00000115183 | 1087.823741 | 0.025453201 | 0.0780111 | 0.6921672 | 0.8594028 | TANC1 | 85461 |
| ENSG00000196151 | 166.2079348 | -0.076780043 | 0.1325489 | 0.2679482 | 0.5361059 | WDSUB1 | 151525 |
| ENSG00000123636 | 345.1842964 | -0.071521422 | 0.1120948 | 0.3255196 | 0.5941598 | BAZ2B | 29994 |
| ENSG00000224152 | 20.55149666 | -0.016336015 | 0.1343858 | 0.5927825 | NA | BAZ2B-AS1 | 643072 |
| ENSG00000226266 | 10.79714893 | -0.015878813 | 0.1360145 | 0.4879732 | NA | BAZ2B | 29994 |
| ENSG00000136536 | 2501.31782 | -0.09265321 | 0.0686996 | 0.1192258 | 0.3371283 | MARCHF7 | 64844 |
| ENSG00000241399 | 36.54296795 | -0.000158097 | 0.1303504 | 0.9946705 | NA | CD302 | 9936 |
| ENSG00000153246 | 450.5135208 | -0.225528618 | 0.1388551 | 0.0179555 | 0.0960131 | PLA2R1 | 22925 |
| ENSG00000115221 | 32.75695208 | 0.001016539 | 0.131432 | 0.9803495 | NA | ITGB6 | 3694 |
| ENSG00000153250 | 1398.501663 | -0.267474956 | 0.0875122 | 0.000391 | 0.0053416 | RBMS1 | 5937 |
| ENSG00000136560 | 714.0543737 | -0.044250662 | 0.0877134 | 0.5132992 | 0.743625 | TANK | 10010 |
| ENSG00000115233 | 2511.184403 | -0.135964525 | 0.0693286 | 0.0243163 | 0.1183986 | PSMD14 | 10213 |
| ENSG00000197635 | 16.04996777 | 0.000823492 | 0.1340143 | 0.9772517 | NA | DPP4 | 1803 |
| ENSG00000115267 | 271.2895043 | -0.071088013 | 0.1170133 | 0.3246135 | 0.593455 | IFIH1 | 64135 |
| ENSG00000115271 | 371.8545902 | -0.111667091 | 0.1228339 | 0.1554955 | 0.3943428 | GCA | 25801 |
| ENSG00000182263 | 951.3914698 | -0.397280062 | 0.1135961 | 4.47E-05 | 0.0008917 | FIGN | 55137 |
| ENSG00000115290 | 112.5147711 | -0.284963385 | 0.3215304 | 0.0191513 | 0.100723 | GRB14 | 2888 |
| ENSG00000082438 | 299.2188051 | -0.451181841 | 0.1721032 | 0.0005998 | 0.0073981 | COBLL1 | 22837 |
| ENSG00000123607 | 453.170997 | -0.06103313 | 0.1044732 | 0.3931316 | 0.6537828 | TTC21B | 79809 |
| ENSG00000198648 | 720.0535216 | -0.086325748 | 0.0972458 | 0.2317495 | 0.4941635 | STK39 | 27347 |
| ENSG00000172292 | 1705.418838 | -0.033581577 | 0.0717249 | 0.581554 | 0.7933122 | CERS6 | 253782 |
| ENSG00000227617 | 47.87419417 | 0.062874175 | 0.1477096 | 0.1817028 | 0.4314356 | CERS6-AS1 | 100861402 |
| ENSG00000152253 | 1087.142377 | -0.282571796 | 0.0970173 | 0.0005561 | 0.0069769 | SPC25 | 57405 |
| ENSG00000163093 | 101.9274968 | 0.01100709 | 0.12372 | 0.8381037 | 0.9331898 | BBS5 | 129880 |
| ENSG00000138399 | 641.6471413 | -0.078084734 | 0.0954974 | 0.2742265 | 0.5426461 | FASTKD1 | 79675 |
| ENSG00000138398 | 2086.208285 | 0.036034926 | 0.066075 | 0.5372297 | 0.7611135 | PPIG | 9360 |
| ENSG00000154479 | 17.17786605 | 0.04793881 | 0.1458342 | 0.1065274 | NA | CFAP210 | 129881 |
| ENSG00000144362 | 65.43739215 | -0.022075198 | 0.1287128 | 0.6453011 | 0.8349316 | PHOSPHO2 | 493911 |
| ENSG00000213160 | 763.1898287 | 0.036783127 | 0.0866731 | 0.586423 | 0.7971162 | KLHL23 | 151230 |
| ENSG00000138385 | 3883.240774 | -0.088772673 | 0.0613901 | 0.1043928 | 0.3104023 | SSB | 6741 |
| ENSG00000138382 | 652.8181885 | -0.156518176 | 0.1100099 | 0.0517003 | 0.1965217 | METTL5 | 29081 |
| ENSG00000144357 | 991.2394298 | -0.183659061 | 0.0963912 | 0.0166344 | 0.0905462 | UBR3 | 130507 |
| ENSG00000128692 | 45.63084172 | 0.022061911 | 0.1310437 | 0.6140658 | 0.8154834 | NA | NA |
| ENSG00000128683 | 18.44731748 | 0.006293742 | 0.1336386 | 0.8296576 | NA | GAD1 | 2571 |
| ENSG00000115806 | 1610.31714 | -0.079791489 | 0.0727507 | 0.1940108 | 0.4459347 | GORASP2 | 26003 |
| ENSG00000198586 | 1383.574833 | -0.095333705 | 0.0774207 | 0.1400907 | 0.3695573 | TLK1 | 9874 |
| ENSG00000123600 | 437.6117758 | -0.007678772 | 0.0969727 | 0.9091083 | 0.9640923 | METTL8 | 79828 |
| ENSG00000115827 | 517.9195986 | 0.245961714 | 0.1313426 | 0.0098009 | 0.0628688 | DCAF17 | 80067 |
| ENSG00000233762 | 73.1105418 | -0.032210268 | 0.1293812 | 0.5325374 | 0.7578048 | RPS15P4 | 401019 |
| ENSG00000071967 | 89.71808856 | 0.022466014 | 0.1246436 | 0.6827839 | 0.8544725 | CYBRD1 | 79901 |
| ENSG00000077380 | 1845.5164 | -0.181764454 | 0.081904 | 0.0085209 | 0.0567462 | DYNC1I2 | 1781 |
| ENSG00000228389 | 15.84284921 | -0.003210183 | 0.1339599 | 0.9020967 | NA | NA | NA |
| ENSG00000115840 | 477.2062851 | -0.044609537 | 0.1022892 | 0.5235717 | 0.7509978 | SLC25A12 | 8604 |
| ENSG00000128708 | 2002.103349 | -0.01201308 | 0.0666991 | 0.8411595 | 0.9347236 | HAT1 | 8520 |
| ENSG00000172878 | 319.4705912 | 0.028953062 | 0.103823 | 0.674669 | 0.8505206 | METAP1D | 254042 |
| ENSG00000144355 | 260.0353511 | 0.10692567 | 0.1322656 | 0.1701452 | 0.415962 | DLX1 | 1745 |
| ENSG00000115844 | 39.12618476 | 0.062338227 | 0.1494695 | 0.1502409 | NA | DLX2 | 1746 |
| ENSG00000091409 | 3698.894328 | -0.06099936 | 0.1099884 | 0.3934448 | 0.6540243 | ITGA6 | 3655 |
| ENSG00000226963 | 9.939796594 | 0.014758482 | 0.1360418 | 0.5037522 | NA | NA | NA |
| ENSG00000225205 | 18.66378247 | -0.00743651 | 0.1336685 | 0.7959516 | NA | PDK1-AS1 | 124900513 |
| ENSG00000152256 | 246.5079869 | -0.026985907 | 0.1093038 | 0.6869307 | 0.857187 | PDK1 | 5163 |
| ENSG00000091436 | 1961.360162 | 0.003273681 | 0.0692095 | 0.9587914 | 0.9831259 | MAP3K20 | 51776 |
| ENSG00000144354 | 1328.207594 | 0.146257995 | 0.0886481 | 0.0406821 | 0.1694751 | CDCA7 | 83879 |
| ENSG00000270460 | 8.463564662 | -0.004721091 | 0.1352524 | 0.8124642 | NA | NA | NA |
| ENSG00000172845 | 2727.741818 | -0.095853679 | 0.0654329 | 0.0960064 | 0.2944213 | SP3 | 6670 |
| ENSG00000138430 | 2637.166398 | -0.168170584 | 0.0684731 | 0.0056056 | 0.041286 | OLA1 | 29789 |
| ENSG00000227180 | 100.4852997 | -0.031293507 | 0.1251734 | 0.5808999 | 0.7928829 | NA | NA |
| ENSG00000231453 | 14.69836023 | 0.040308316 | 0.1426901 | 0.1391189 | NA | LINC01305 | 285084 |
| ENSG00000138433 | 374.9360827 | -0.018921491 | 0.1007945 | 0.7813041 | 0.9091865 | CIR1 | 9541 |
| ENSG00000144306 | 213.1629702 | -0.011076289 | 0.1105296 | 0.8637844 | 0.9454522 | SCRN3 | 79634 |

| | | | | | | | | |
|---|---|---|---|---|---|---|---|---|
| ENSG00000163328 | 57.03579541 | 0.076659766 | 0.1556315 | 0.135766 | 0.3628243 | GPR155 | | 151556 |
| ENSG00000235655 | 687.3066123 | -0.114898287 | 0.1059076 | 0.1324774 | 0.358616 | NA | NA | |
| ENSG00000128656 | 592.7389346 | -0.028786431 | 0.0910679 | 0.6724709 | 0.8499086 | CHN1 | | 1123 |
| ENSG00000115966 | 1155.829545 | -0.160577423 | 0.0893596 | 0.0262022 | 0.1251813 | ATF2 | | 1386 |
| ENSG00000229750 | 12.60510656 | 0.023173625 | 0.1374167 | 0.3333524 | NA | NA | NA | |
| ENSG00000154518 | 3137.54745 | -0.220519364 | 0.0736521 | 0.0006838 | 0.0082514 | ATP5MC3 | | 518 |
| ENSG00000144320 | 782.3119713 | -0.093956926 | 0.0916407 | 0.1834796 | 0.4332373 | LNPK | | 80856 |
| ENSG00000128713 | 54.3238892 | -0.027152657 | 0.1309837 | 0.5564471 | 0.7758245 | HOXD11 | | 3237 |
| ENSG00000128710 | 111.9288617 | -0.073949303 | 0.1407078 | 0.2443665 | 0.5100385 | HOXD10 | | 3236 |
| ENSG00000237380 | 24.46957621 | -0.000431904 | 0.132455 | 0.9873442 | NA | NA | NA | |
| ENSG00000128709 | 131.6206534 | 0.016111175 | 0.1194991 | 0.7874938 | 0.9116145 | HOXD9 | | 3235 |
| ENSG00000175879 | 38.05359507 | 0.03956835 | 0.1377372 | 0.3367998 | NA | HOXD8 | | 3234 |
| ENSG00000128652 | 24.39164304 | 0.022200433 | 0.134928 | 0.5030824 | NA | HOXD3 | | 3232 |
| ENSG00000170166 | 13.10023028 | 0.026387486 | 0.1379494 | 0.3008335 | NA | HOXD4 | | 3233 |
| ENSG00000224189 | 96.12982182 | 0.046433589 | 0.1309768 | 0.4156717 | 0.6697358 | HAGLR | | 401022 |
| ENSG00000128654 | 522.3364389 | 0.06557056 | 0.0984101 | 0.3743548 | 0.6358642 | MTX2 | | 10651 |
| ENSG00000170144 | 12829.07763 | -0.38265351 | 0.0585733 | 7.55E-12 | 8.09E-10 | HNRNPA3 | | 220988 |
| ENSG00000116044 | 1613.573791 | 0.012884112 | 0.0704024 | 0.8316726 | 0.9304418 | NFE2L2 | | 4780 |
| ENSG00000213963 | 30.09324706 | 0.033426762 | 0.1368992 | 0.3702675 | NA | LOC100130( | | 100130691 |
| ENSG00000018510 | 1944.403538 | -0.111841497 | 0.0779208 | 0.0856524 | 0.2740282 | AGPS | | 8540 |
| ENSG00000196659 | 29.05742122 | -0.047760726 | 0.1431709 | 0.199175 | NA | IFT70B | | 150737 |
| ENSG00000197557 | 36.64999026 | -0.023759149 | 0.1338561 | 0.5242485 | NA | IFT70A | | 92104 |
| ENSG00000155636 | 195.7829241 | 0.000490055 | 0.1140911 | 0.9950508 | 0.9978699 | RBM45 | | 129831 |
| ENSG00000079156 | 619.452908 | 0.256547727 | 0.1193746 | 0.0051026 | 0.0386035 | OSBPL6 | | 114880 |
| ENSG00000223960 | 124.0536956 | 0.021757905 | 0.1207364 | 0.7130173 | 0.8702074 | CHROMR | | 101927027 |
| ENSG00000180228 | 639.3571889 | -0.019674916 | 0.0891214 | 0.7526759 | 0.8932431 | PRKRA | | 8575 |
| ENSG00000079150 | 26.48768359 | -0.001845298 | 0.1320821 | 0.9549547 | NA | FKBP7 | | 51661 |
| ENSG00000116095 | 513.9450299 | -0.024992932 | 0.0976732 | 0.715324 | 0.871162 | PLEKHA3 | | 65977 |
| ENSG00000237298 | 248.2793806 | 0.032339657 | 0.1118321 | 0.6302409 | 0.8239333 | TTN-AS1 | | 100506866 |
| ENSG00000155657 | 27.51611846 | 0.046310381 | 0.1426903 | 0.2062058 | NA | TTN | | 7273 |
| ENSG00000270277 | 14.03553303 | 0.012274373 | 0.1349126 | 0.6360389 | NA | NA | NA | |
| ENSG00000271011 | 12.85737189 | -0.037675291 | 0.1419587 | 0.1373702 | NA | NA | NA | |
| ENSG00000270574 | 15.62698381 | -0.009418977 | 0.1342323 | 0.7291001 | NA | NA | NA | |
| ENSG00000271141 | 14.04436624 | -0.027060914 | 0.1380915 | 0.291306 | NA | NA | NA | |
| ENSG00000187231 | 319.9574593 | -0.025131148 | 0.1043023 | 0.7121055 | 0.869458 | SESTD1 | | 91404 |
| ENSG00000144331 | 27.56510318 | 0.018735512 | 0.1335799 | 0.5938491 | NA | ZNF385B | | 151126 |
| ENSG00000163510 | 769.6167887 | -0.091810674 | 0.0966868 | 0.2042093 | 0.4594559 | CWC22 | | 57703 |
| ENSG00000170035 | 1156.141748 | -0.173364425 | 0.0891001 | 0.0169698 | 0.0919833 | UBE2E3 | | 10477 |
| ENSG00000138434 | 3313.2264 | -0.116161603 | 0.0649657 | 0.0427243 | 0.1748031 | ITPRID2 | | 6744 |
| ENSG00000077232 | 2716.464855 | -0.163669939 | 0.0747248 | 0.0108729 | 0.06727 | DNAJC10 | | 54431 |
| ENSG00000061676 | 5196.499716 | -0.252756598 | 0.0608758 | 6.96E-06 | 0.0001826 | NCKAP1 | | 10787 |
| ENSG00000162999 | 55.86558173 | -0.049234746 | 0.1388312 | 0.3040151 | 0.5728743 | DUSP19 | | 142679 |
| ENSG00000163002 | 617.5521061 | 0.054415998 | 0.0993222 | 0.4669481 | 0.7094916 | NUP35 | | 129401 |
| ENSG00000272800 | 12.70669937 | 0.01847932 | 0.136135 | 0.4589876 | NA | NA | NA | |
| ENSG00000065548 | 2511.168562 | 0.00685278 | 0.0633752 | 0.9035572 | 0.9618565 | ZC3H15 | | 55854 |
| ENSG00000268846 | 34.14607714 | -0.018436244 | 0.1323725 | 0.6292911 | NA | NA | NA | |
| ENSG00000138448 | 5698.087068 | -0.073711195 | 0.0613443 | 0.1777861 | 0.4270449 | ITGAV | | 3685 |
| ENSG00000144369 | 401.3216626 | -0.060830694 | 0.1055029 | 0.3836473 | 0.6445252 | FAM171B | | 165215 |
| ENSG00000224063 | 13.16499631 | 0.022708802 | 0.1370398 | 0.3667692 | NA | NA | NA | |
| ENSG00000003436 | 934.2832399 | -0.12399564 | 0.1781158 | 0.0919876 | 0.2872247 | TFPI | | 7035 |
| ENSG00000144366 | 707.1241682 | -0.067205811 | 0.0905485 | 0.3331028 | 0.6004589 | GULP1 | | 51454 |
| ENSG00000204262 | 9.334969893 | -0.007155297 | 0.1352508 | 0.7285793 | NA | COL5A2 | | 1290 |
| ENSG00000115368 | 1702.011527 | -0.062608219 | 0.0707532 | 0.3224563 | 0.5912121 | WDR75 | | 84128 |
| ENSG00000138381 | 1709.76952 | 0.144090956 | 0.0854125 | 0.038778 | 0.1643563 | ASNSD1 | | 54529 |
| ENSG00000151687 | 41.26675525 | -0.044730996 | 0.1392199 | 0.2974973 | 0.567914 | ANKAR | | 150709 |
| ENSG00000128694 | 76.48399968 | -0.0141981 | 0.1252468 | 0.7821475 | 0.9095461 | OSGEPL1 | | 64172 |
| ENSG00000128699 | 945.7989 | 0.005298266 | 0.0793517 | 0.9354633 | 0.9752526 | ORMDL1 | | 94101 |
| ENSG00000064933 | 475.0477863 | 0.067035074 | 0.1032505 | 0.3320959 | 0.5992396 | PMS1 | | 5378 |
| ENSG00000187699 | 29.63251853 | 0.055445053 | 0.1473463 | 0.1440964 | NA | C2orf88 | | 84281 |
| ENSG00000204253 | 120.0546457 | -0.133967406 | 0.1830281 | 0.0843133 | 0.271264 | NA | NA | |
| ENSG00000198130 | 554.6751902 | -0.165747438 | 0.1177341 | 0.0472244 | 0.1857336 | HIBCH | | 26275 |
| ENSG00000151689 | 352.4540713 | 0.123891113 | 0.1269542 | 0.1257866 | 0.3480961 | INPP1 | | 3628 |
| ENSG00000151690 | 618.1758995 | 0.00206656 | 0.0920938 | 0.9767521 | 0.9912634 | MFSD6 | | 54842 |
| ENSG00000189362 | 194.231109 | 0.072344419 | 0.1270639 | 0.3025919 | 0.5720304 | NEMP2 | | 100131211 |
| ENSG00000138386 | 1638.628614 | 0.099762555 | 0.0742253 | 0.1128005 | 0.325741 | NAB1 | | 4664 |
| ENSG00000228509 | 19.2583856 | 0.004543705 | 0.1334263 | 0.8793447 | NA | NA | NA | |
| ENSG00000235852 | 65.38769301 | 0.078610817 | 0.1556533 | 0.1414955 | 0.3718354 | NA | NA | |
| ENSG00000115419 | 14721.4126 | 0.059609554 | 0.1221923 | 0.3843908 | 0.6449396 | GLS | | 2744 |
| ENSG00000115415 | 2557.610395 | -0.15721779 | 0.0691008 | 0.0094539 | 0.0615017 | STAT1 | | 6772 |
| ENSG00000128641 | 3583.444154 | 0.076119179 | 0.0675494 | 0.1955166 | 0.4480732 | MYO1B | | 4430 |

| | | | | | | | | | |
|---|---|---|---|---|---|---|---|---|---|
| ENSG00000173559 | 1690.785259 | 1.06100749 | 0.0817572 | 9.31E-40 | 1.21E-36 | NABP1 | | | 64859 |
| ENSG00000144339 | 19.61633461 | -0.009703482 | 0.1337969 | 0.7389225 | NA | TMEFF2 | | | 23671 |
| ENSG00000196950 | 927.749581 | -0.176281303 | 0.098788 | 0.0229127 | 0.1141338 | SLC39A10 | | | 57181 |
| ENSG00000081320 | 308.6700819 | -0.096599346 | 0.1222888 | 0.2068304 | 0.4626552 | STK17B | | | 9262 |
| ENSG00000138411 | 32.11826804 | 0.011992264 | 0.1322583 | 0.7390991 | NA | HECW2 | | | 57520 |
| ENSG00000144395 | 523.7522004 | 0.075610936 | 0.0997694 | 0.3062955 | 0.5754001 | CCDC150 | | | 284992 |
| ENSG00000119041 | 821.4253595 | -0.108332701 | 0.1009087 | 0.1457604 | 0.3783345 | GTF3C3 | | | 9330 |
| ENSG00000197121 | 481.7627385 | 0.020908603 | 0.0946217 | 0.7616946 | 0.8978158 | PGAP1 | | | 80055 |
| ENSG00000065413 | 122.5533377 | 0.00771958 | 0.1188179 | 0.8793578 | 0.9513156 | ANKRD44 | | | 91526 |
| ENSG00000115524 | 6711.15919 | -0.248464346 | 0.0589581 | 5.50E-06 | 0.0001501 | SF3B1 | | | 23451 |
| ENSG00000115520 | 707.8438878 | -0.027618501 | 0.0865601 | 0.6805025 | 0.8538239 | COQ10B | | | 80219 |
| ENSG00000144381 | 18563.92025 | -0.225882197 | 0.058814 | 3.11E-05 | 0.0006607 | HSPD1 | | | 3329 |
| ENSG00000115541 | 1709.893378 | -0.281353767 | 0.0866204 | 0.000189 | 0.0029299 | HSPE1 | | | 3336 |
| ENSG00000115540 | 467.8529022 | -0.091928903 | 0.1082192 | 0.2194177 | 0.4793377 | MOB4 | | | 25843 |
| ENSG00000247626 | 374.524277 | 0.171821546 | 0.1402138 | 0.0543138 | 0.2040646 | MARS2 | | | 92935 |
| ENSG00000119042 | 472.0714397 | -0.051246343 | 0.0995561 | 0.4667004 | 0.7094916 | SATB2 | | | 23314 |
| ENSG00000225953 | 27.75713501 | -0.040025299 | 0.139897 | 0.2667656 | NA | SATB2-AS1 | | | 150538 |
| ENSG00000226124 | 16.34510237 | -0.002192114 | 0.1337229 | 0.9346045 | NA | FTCDNL1 | | | 348751 |
| ENSG00000232732 | 9.686376591 | -0.001616601 | 0.1349776 | 0.9369377 | NA | LOC101927( | | | 101927687 |
| ENSG00000178074 | 694.041138 | -0.100748439 | 0.0984735 | 0.169992 | 0.4157205 | C2orf69 | | | 205327 |
| ENSG00000162971 | 364.4683944 | 0.086646202 | 0.1219345 | 0.2459671 | 0.5116904 | TYW5 | | | 129450 |
| ENSG00000162972 | 466.8581409 | -0.136306729 | 0.1190012 | 0.0923604 | 0.2874295 | MAIP1 | | | 79568 |
| ENSG00000196141 | 2064.461021 | -0.082829013 | 0.0750447 | 0.1897366 | 0.440597 | SPATS2L | | | 26010 |
| ENSG00000155729 | 151.8613921 | 0.013358607 | 0.1171996 | 0.8281473 | 0.9291699 | KCTD18 | | | 130535 |
| ENSG00000163535 | 1170.028344 | -0.156854336 | 0.0888585 | 0.0289209 | 0.1350219 | SGO2 | | | 151246 |
| ENSG00000138356 | 136.1751875 | -0.013476168 | 0.1186411 | 0.8206329 | 0.926076 | AOX1 | | | 316 |
| ENSG00000230408 | 20.70713719 | 0.007622027 | 0.1339182 | 0.7889207 | NA | BZW1-AS1 | | | 101927795 |
| ENSG00000082153 | 9973.042553 | 0.031052362 | 0.0558394 | 0.5371808 | 0.7611135 | BZW1 | | | 9689 |
| ENSG00000260006 | 93.60692488 | -0.039368006 | 0.1286994 | 0.4811018 | 0.7202486 | NA | | NA | |
| ENSG00000013441 | 1273.13209 | 0.150663614 | 0.0969773 | 0.045965 | 0.183381 | CLK1 | | | 1195 |
| ENSG00000240344 | 243.3932183 | -0.015965862 | 0.1125713 | 0.8055715 | 0.9187021 | PPIL3 | | | 53938 |
| ENSG00000196290 | 394.4145666 | -0.451603425 | 0.147367 | 0.0001632 | 0.0025954 | NIF3L1 | | | 60491 |
| ENSG00000115942 | 1080.522807 | -0.123046328 | 0.0859724 | 0.0758063 | 0.2532152 | ORC2 | | | 4999 |
| ENSG00000183308 | 16.61440728 | 0.037101088 | 0.1410278 | 0.1908295 | NA | NA | | NA | |
| ENSG00000155744 | 842.1682093 | -0.036478012 | 0.0869017 | 0.577874 | 0.7911317 | HYCC2 | | | 285172 |
| ENSG00000119013 | 700.2605138 | -0.162067928 | 0.1057634 | 0.0410451 | 0.1703191 | NDUFB3 | | | 4709 |
| ENSG00000003402 | 3668.920495 | 0.14745991 | 0.0692096 | 0.0149171 | 0.0838127 | CFLAR | | | 8837 |
| ENSG00000232133 | 47.09625313 | -0.809154066 | 0.5198075 | 0.0030923 | 0.0262126 | IMPDH1P10 | | | 100874399 |
| ENSG00000226312 | 32.67895597 | -0.02892354 | 0.1350778 | 0.4460139 | NA | CFLAR-AS1 | | | 65072 |
| ENSG00000064012 | 726.8497479 | 0.068069769 | 0.0909232 | 0.332923 | 0.6004127 | CASP8 | | | 841 |
| ENSG00000115993 | 1307.867849 | -0.123349731 | 0.083211 | 0.0698065 | 0.2402503 | TRAK2 | | | 66008 |
| ENSG00000082146 | 378.105579 | -0.104228643 | 0.117917 | 0.1778588 | 0.4271317 | STRADB | | | 55437 |
| ENSG00000155755 | 445.0732042 | -0.182225564 | 0.1331006 | 0.0404998 | 0.1690774 | TMEM237 | | | 65062 |
| ENSG00000003393 | 1259.633558 | 0.123321099 | 0.083598 | 0.0713575 | 0.2433566 | ALS2 | | | 57679 |
| ENSG00000155760 | 15.21968617 | 0.02363421 | 0.1369634 | 0.3750572 | NA | FZD7 | | | 8324 |
| ENSG00000116030 | 2929.542172 | -0.20511909 | 0.0678162 | 0.0007088 | 0.0084909 | SUMO1 | | | 7341 |
| ENSG00000055044 | 3282.960352 | 0.093073576 | 0.0644631 | 0.101868 | 0.3055779 | NOP58 | | | 51602 |
| ENSG00000238317 | 9.196327085 | -0.010496126 | 0.1356217 | 0.611559 | NA | SNORD11 | | | 692058 |
| ENSG00000204217 | 1450.363989 | -0.406060956 | 0.0852295 | 1.92E-07 | 8.24E-06 | BMPR2 | | | 659 |
| ENSG00000138439 | 170.870092 | -0.040035838 | 0.1196369 | 0.5326237 | 0.7578048 | FAM117B | | | 150864 |
| ENSG00000163596 | 83.801062 | 0.079751649 | 0.1509433 | 0.1784446 | 0.4275716 | ICA1L | | | 130026 |
| ENSG00000138442 | 1476.98646 | 0.047124474 | 0.073187 | 0.4469748 | 0.6944817 | WDR12 | | | 55759 |
| ENSG00000138380 | 34.99054849 | 0.037205896 | 0.1376014 | 0.343178 | NA | CARF | | | 79800 |
| ENSG00000144426 | 482.4322796 | -0.067831099 | 0.1027069 | 0.3462473 | 0.6126197 | NBEAL1 | | | 65065 |
| ENSG00000119004 | 207.4071297 | 0.003436553 | 0.1110864 | 0.9588769 | 0.9831259 | CYP20A1 | | | 57404 |
| ENSG00000138443 | 1338.01672 | -0.170322767 | 0.089462 | 0.0191849 | 0.1008092 | ABI2 | | | 10152 |
| ENSG00000173166 | 1161.824788 | 0.183137987 | 0.0981464 | 0.0181782 | 0.0970029 | RAPH1 | | | 65059 |
| ENSG00000116117 | 21.49961989 | -0.017132936 | 0.1342768 | 0.5858081 | NA | PARD3B | | | 117583 |
| ENSG00000118257 | 11.72519016 | -0.001968207 | 0.1345796 | 0.9310552 | NA | NRP2 | | | 8828 |
| ENSG00000114933 | 804.0945937 | -0.035768863 | 0.0836014 | 0.5890688 | 0.798683 | INO80D | | | 54891 |
| ENSG00000227946 | 71.60614357 | -0.008580553 | 0.1254426 | 0.8633443 | 0.9453914 | NA | | NA | |
| ENSG00000023228 | 2538.956021 | -0.084677644 | 0.0668954 | 0.1467757 | 0.3800339 | NDUFS1 | | | 4719 |
| ENSG00000114942 | 7412.518156 | -0.09222568 | 0.0574472 | 0.0764576 | 0.2545912 | EEF1B2 | | | 1933 |
| ENSG00000204186 | 145.005654 | 0.027296099 | 0.1215338 | 0.6479686 | 0.836587 | ZDBF2 | | | 57683 |
| ENSG00000114948 | 29.58951719 | 0.024255262 | 0.1343099 | 0.5050527 | NA | ADAM23 | | | 8745 |
| ENSG00000138400 | 13.82556464 | -0.007032672 | 0.1347462 | 0.7666056 | NA | MDH1B | | | 130752 |
| ENSG00000118246 | 754.6836355 | 0.064293089 | 0.0954665 | 0.3633517 | 0.6263742 | FASTKD2 | | | 22868 |
| ENSG00000118263 | 710.8957479 | 0.568998627 | 0.1192961 | 1.31E-07 | 6.00E-06 | KLF7 | | | 8609 |
| ENSG00000237892 | 10.14692377 | 0.041015957 | 0.1441502 | 0.0647205 | NA | NA | | NA | |
| ENSG00000118260 | 836.0561492 | -0.017660839 | 0.0833104 | 0.7885553 | 0.9116361 | CREB1 | | | 1385 |

| | | | | | | | | |
|---|---|---|---|---|---|---|---|---|
| ENSG00000144401 | 395.6179096 | -0.050666461 | 0.1025173 | 0.4736572 | 0.7139512 | METTL21A | | 151194 |
| ENSG00000163249 | 492.0780768 | -0.132821065 | 0.11586 | 0.0971905 | 0.2965733 | CCNYL1 | | 151195 |
| ENSG00000163251 | 275.6001055 | -0.055067357 | 0.1119004 | 0.430944 | 0.6823717 | FZD5 | | 7855 |
| ENSG00000244567 | 32.23633672 | -0.010317533 | 0.1317191 | 0.7770562 | NA | NA | NA | |
| ENSG00000178385 | 80.58529508 | 0.016801586 | 0.1249689 | 0.7515844 | 0.8926359 | PLEKHM3 | | 389072 |
| ENSG00000138413 | 1190.601983 | -0.573735095 | 0.0996022 | 5.93E-10 | 4.39E-08 | IDH1 | | 3417 |
| ENSG00000115020 | 1352.9075 | -0.088978678 | 0.0773469 | 0.1675781 | 0.4125292 | PIKFYVE | | 200576 |
| ENSG00000197713 | 1533.565263 | 0.027780846 | 0.0743496 | 0.6573413 | 0.8419413 | RPE | | 6120 |
| ENSG00000144445 | 142.7791007 | -0.04628864 | 0.1235816 | 0.4652791 | 0.7086875 | KANSL1L | | 151050 |
| ENSG00000272807 | 16.09169099 | 0.001702169 | 0.1337091 | 0.955854 | NA | NA | NA | |
| ENSG00000229127 | 13.82128504 | 0.006221995 | 0.1347592 | 0.7936376 | NA | NA | NA | |
| ENSG00000115365 | 1379.964452 | -0.332286685 | 0.0930068 | 4.46E-05 | 0.0008917 | LANCL1 | | 10314 |
| ENSG00000021826 | 29.64464323 | -0.020600557 | 0.1338799 | 0.5575061 | NA | CPS1 | | 1373 |
| ENSG00000178568 | 19.20911471 | -0.038648525 | 0.1411247 | 0.2017222 | NA | ERBB4 | | 2066 |
| ENSG00000030419 | 29.22402913 | 0.015270437 | 0.1333149 | 0.6546741 | NA | IKZF2 | | 22807 |
| ENSG00000144451 | 147.9016795 | -0.065953284 | 0.1358699 | 0.2929248 | 0.5623098 | SPAG16 | | 79582 |
| ENSG00000138376 | 736.4963108 | 0.245075395 | 0.1120895 | 0.0051373 | 0.0388405 | BARD1 | | 580 |
| ENSG00000229267 | 11.8648508 | 0.022911008 | 0.1373558 | 0.3360621 | NA | SNHG31 | | 101928103 |
| ENSG00000144452 | 29.49586322 | -0.088231591 | 0.1737199 | 0.0356718 | NA | ABCA12 | | 26154 |
| ENSG00000138363 | 3004.353012 | -0.057234608 | 0.0611335 | 0.2954005 | 0.5654823 | ATIC | | 471 |
| ENSG00000115414 | 1418.034931 | 1.095921217 | 0.087222 | 1.95E-37 | 2.07E-34 | FN1 | | 2335 |
| ENSG00000118242 | 372.5119876 | -0.017068438 | 0.1025366 | 0.8013473 | 0.9169806 | MREG | | 55686 |
| ENSG00000115425 | 191.5636007 | -0.148707663 | 0.1697767 | 0.0818362 | 0.2662274 | PECR | | 55825 |
| ENSG00000163449 | 19.44887159 | 0.017554297 | 0.134959 | 0.5532019 | NA | TMEM169 | | 92691 |
| ENSG00000079246 | 8053.438061 | -0.324473751 | 0.0626181 | 3.14E-08 | 1.70E-06 | XRCC5 | | 7520 |
| ENSG00000260804 | 136.9110761 | 0.010338375 | 0.1176806 | 0.8652577 | 0.9457908 | LINC01963 | | 150967 |
| ENSG00000144583 | 447.850667 | 0.040576012 | 0.101438 | 0.5612092 | 0.7795039 | MARCHF4 | | 57574 |
| ENSG00000138375 | 364.2929669 | -0.109199351 | 0.1223509 | 0.1623192 | 0.4045339 | SMARCAL1 | | 50485 |
| ENSG00000197756 | 13676.49265 | -0.106681377 | 0.0607226 | 0.049738 | 0.1917456 | RPL37A | | 6168 |
| ENSG00000163466 | 5053.670651 | -0.157179424 | 0.0593962 | 0.0035217 | 0.0288476 | ARPC2 | | 10109 |
| ENSG00000261338 | 19.41661442 | 0.013343626 | 0.134199 | 0.6547656 | NA | NA | NA | |
| ENSG00000127837 | 2622.211995 | -0.04370902 | 0.0626304 | 0.4314704 | 0.6828658 | AAMP | | 14 |
| ENSG00000127838 | 548.5423021 | -0.17393912 | 0.1186627 | 0.0395588 | 0.1665175 | PNKD | | 25953 |
| ENSG00000135926 | 1590.495772 | 0.073958877 | 0.0724899 | 0.2307748 | 0.4924517 | TMBIM1 | | 64114 |
| ENSG00000144579 | 1277.501009 | -0.277569628 | 0.0944058 | 0.0005274 | 0.0067323 | CTDSP1 | | 58190 |
| ENSG00000127831 | 13.85095268 | 0.003645914 | 0.1344058 | 0.8847452 | NA | VIL1 | | 7429 |
| ENSG00000135913 | 930.3876627 | 0.245766141 | 0.1037015 | 0.0033056 | 0.027541 | USP37 | | 57695 |
| ENSG00000144580 | 1913.221807 | -0.029960759 | 0.0691848 | 0.6150219 | 0.8157779 | CNOT9 | | 9125 |
| ENSG00000115556 | 22.73968121 | -0.003328937 | 0.1329651 | 0.9119774 | NA | PLCD4 | | 84812 |
| ENSG00000273466 | 84.83131287 | 0.12726406 | 0.1925526 | 0.071638 | 0.2440137 | NA | NA | |
| ENSG00000115568 | 1097.096797 | 0.098247062 | 0.0864236 | 0.1550783 | 0.3935203 | ZNF142 | | 7701 |
| ENSG00000074582 | 906.8984829 | 0.217163837 | 0.1011358 | 0.0071637 | 0.0496319 | BCS1L | | 617 |
| ENSG00000163481 | 571.5147843 | 0.219050257 | 0.122492 | 0.0146376 | 0.0828001 | RNF25 | | 64320 |
| ENSG00000163482 | 343.1226445 | 0.093184564 | 0.1182035 | 0.2204311 | 0.4801345 | STK36 | | 27148 |
| ENSG00000135912 | 803.4114907 | -0.007528599 | 0.0836341 | 0.9085338 | 0.963904 | TTLL4 | | 9654 |
| ENSG00000135929 | 132.3576203 | -0.114251643 | 0.1659331 | 0.1165914 | 0.3332126 | CYP27A1 | | 1593 |
| ENSG00000171450 | 11.33477801 | 0.03693792 | 0.1420287 | 0.1190067 | NA | CDK5R2 | | 8941 |
| ENSG00000272644 | 13.522796 | -0.00623793 | 0.134406 | 0.8033096 | NA | NA | NA | |
| ENSG00000213901 | 71.39928531 | -0.00543693 | 0.1255513 | 0.9117072 | 0.9651317 | SLC23A3 | | 151295 |
| ENSG00000115649 | 910.449725 | 0.131754822 | 0.0922439 | 0.0696202 | 0.2400304 | CNPPD1 | | 27013 |
| ENSG00000144567 | 1420.28315 | 0.001189521 | 0.0729989 | 0.985708 | 0.9941336 | RETREG2 | | 79137 |
| ENSG00000158552 | 446.786454 | 0.036125519 | 0.0971127 | 0.6032504 | 0.8087419 | ZFAND2B | | 130617 |
| ENSG00000115657 | 14.21883525 | -0.00737199 | 0.1347189 | 0.7590339 | NA | ABCB6 | | 10058 |
| ENSG00000198925 | 1352.781065 | -0.022779844 | 0.0776436 | 0.7209456 | 0.8735856 | ATG9A | | 79065 |
| ENSG00000163516 | 505.7220586 | 0.027974789 | 0.0962562 | 0.6628087 | 0.8438537 | ANKZF1 | | 55139 |
| ENSG00000163521 | 70.55298196 | -0.055002583 | 0.1385309 | 0.3003763 | 0.5700562 | GLB1L | | 79411 |
| ENSG00000115661 | 321.5570767 | 0.189107108 | 0.1556787 | 0.0439002 | 0.177715 | STK16 | | 8576 |
| ENSG00000127824 | 296.4520032 | 0.322391795 | 0.1783785 | 0.0063811 | 0.0456449 | TUBA4A | | 7277 |
| ENSG00000135924 | 834.8211221 | 0.206053964 | 0.1035459 | 0.0112028 | 0.0689827 | DNAJB2 | | 3300 |
| ENSG00000123992 | 1097.066215 | -0.05496392 | 0.0785933 | 0.3945918 | 0.6549057 | DNPEP | | 23549 |
| ENSG00000144591 | 772.6197789 | 0.103436242 | 0.0937416 | 0.1522841 | 0.3895241 | GMPPA | | 29926 |
| ENSG00000123989 | 479.509114 | 0.646700695 | 0.137452 | 1.60E-07 | 7.14E-06 | CHPF | | 79586 |
| ENSG00000188760 | 45.4947427 | 0.152747557 | 0.2759976 | 0.013185 | 0.0772001 | TMEM198 | | 130612 |
| ENSG00000124006 | 217.4908553 | 0.063533408 | 0.1209139 | 0.363744 | 0.6265951 | OBSL1 | | 23363 |
| ENSG00000144589 | 482.042012 | -0.08366509 | 0.1066306 | 0.2580142 | 0.5247892 | STK11IP | | 114790 |
| ENSG00000114923 | 357.6516978 | -0.105044956 | 0.1205908 | 0.1754243 | 0.4238996 | SLC4A3 | | 6508 |
| ENSG00000261428 | 47.44025883 | 0.028716279 | 0.1323739 | 0.5191772 | 0.7478713 | NA | NA | |
| ENSG00000116120 | 1323.229656 | 0.085379527 | 0.0777076 | 0.1866614 | 0.4373905 | FARSB | | 10056 |
| ENSG00000123983 | 1670.909394 | -0.203642356 | 0.0831571 | 0.0038478 | 0.0309156 | ACSL3 | | 2181 |
| ENSG00000152056 | 440.0864825 | -0.050830292 | 0.0996829 | 0.4700853 | 0.7115546 | AP1S3 | | 130340 |

| | | | | | | | |
|---|---|---|---|---|---|---|---|
| ENSG00000085449 | 1427.577653 | 0.073062436 | 0.0748769 | 0.2465353 | 0.5125985 | WDFY1 | 57590 |
| ENSG00000135900 | 661.2179221 | -0.056149623 | 0.0920084 | 0.4208353 | 0.6735758 | MRPL44 | 65080 |
| ENSG00000135919 | 954.6421164 | 0.150726291 | 0.0942968 | 0.0423902 | 0.1740458 | SERPINE2 | 5270 |
| ENSG00000036257 | 2134.494213 | -0.143121953 | 0.0746444 | 0.0248712 | 0.1202483 | CUL3 | 8452 |
| ENSG00000135905 | 252.6504459 | 0.053168434 | 0.1145227 | 0.4438867 | 0.6921329 | DOCK10 | 55619 |
| ENSG00000169047 | 253.673909 | 0.409177294 | 0.1906116 | 0.0021923 | 0.0203236 | IRS1 | 3667 |
| ENSG00000144468 | 422.1609782 | 0.033126717 | 0.0984522 | 0.6328973 | 0.8252447 | RHBDD1 | 84236 |
| ENSG00000236432 | 8.218900127 | 0.00058491 | 0.1353642 | 0.9775655 | NA | MFF-DT | 654841 |
| ENSG00000168958 | 1080.832304 | -0.031276599 | 0.0778267 | 0.6247633 | 0.8202204 | MFF | 56947 |
| ENSG00000173744 | 1563.738345 | -0.130851937 | 0.0820307 | 0.0532999 | 0.2011621 | AGFG1 | 3267 |
| ENSG00000115009 | 278.834421 | -0.003744286 | 0.1080176 | 0.9536481 | 0.9817188 | CCL20 | 6364 |
| ENSG00000123977 | 213.7328045 | 0.122179165 | 0.1483315 | 0.1275971 | 0.3508621 | DAW1 | 164781 |
| ENSG00000153823 | 78.63263351 | 0.079947223 | 0.1541672 | 0.1555978 | 0.3943696 | PID1 | 55022 |
| ENSG00000187957 | 9.248794475 | 0.015677461 | 0.1362876 | 0.4624213 | NA | DNER | 92737 |
| ENSG00000153827 | 8455.118805 | -0.215821708 | 0.0570383 | 4.47E-05 | 0.0008917 | TRIP12 | 9320 |
| ENSG00000153832 | 61.41317247 | 0.07947153 | 0.1565245 | 0.1357679 | 0.3628243 | FBXO36 | 130888 |
| ENSG00000135899 | 183.1053299 | -0.077026472 | 0.1332926 | 0.2645413 | 0.5323182 | SP110 | 3431 |
| ENSG00000185404 | 240.6672982 | 0.423314622 | 0.2004183 | 0.0022408 | 0.0206266 | SP140L | 93349 |
| ENSG00000067066 | 592.5500807 | 0.165630242 | 0.1159625 | 0.0462792 | 0.1840517 | SP100 | 6672 |
| ENSG00000135932 | 1635.397984 | 0.153957121 | 0.0820505 | 0.0242902 | 0.1183359 | CAB39 | 51719 |
| ENSG00000135916 | 4059.423339 | 0.029458123 | 0.0572487 | 0.5713099 | 0.7864095 | ITM2C | 81618 |
| ENSG00000173692 | 3671.974755 | -0.179453955 | 0.0685765 | 0.0030778 | 0.0261275 | PSMD1 | 5707 |
| ENSG00000135931 | 353.0645141 | -0.069280833 | 0.1104557 | 0.3389428 | 0.6064835 | ARMC9 | 80210 |
| ENSG00000115053 | 28566.89853 | -0.257567864 | 0.0556736 | 7.80E-07 | 2.77E-05 | NCL | 4691 |
| ENSG00000206885 | 14.15473052 | 0.002540229 | 0.1345881 | 0.9162802 | NA | SNORA75 | 654321 |
| ENSG00000207280 | 14.9605962 | -0.025586334 | 0.1374522 | 0.3354577 | NA | SNORD20 | 6082 |
| ENSG00000181798 | 9.203978364 | 0.03179207 | 0.1405833 | 0.1288747 | NA | LINC00471 | 151477 |
| ENSG00000187514 | 27634.13693 | -0.251978507 | 0.0602573 | 6.50E-06 | 0.000173 | PTMA | 5757 |
| ENSG00000156973 | 495.7574281 | 0.000998386 | 0.0930033 | 0.9897233 | 0.9955163 | PDE6D | 5147 |
| ENSG00000144524 | 899.1034071 | -0.003973561 | 0.0807058 | 0.9505163 | 0.9802173 | COPS7B | 64708 |
| ENSG00000144535 | 392.3398662 | -0.050390245 | 0.1022407 | 0.4719711 | 0.7123777 | DIS3L2 | 129563 |
| ENSG00000221944 | 50.49787591 | 0.050471734 | 0.1401136 | 0.2827726 | 0.55226 | TIGD1 | 200765 |
| ENSG00000135930 | 1260.946989 | -0.202639011 | 0.0910273 | 0.0067845 | 0.0476548 | EIF4E2 | 9470 |
| ENSG00000204120 | 2149.653464 | -0.020278583 | 0.0678114 | 0.7299175 | 0.8797118 | GIGYF2 | 26058 |
| ENSG00000182600 | 30.70335395 | 0.019859419 | 0.1333097 | 0.5886439 | NA | SNORC | 389084 |
| ENSG00000085978 | 877.5146079 | -0.024249857 | 0.0819961 | 0.7113496 | 0.8691254 | ATG16L1 | 55054 |
| ENSG00000077044 | 1056.632052 | 0.19773394 | 0.0961537 | 0.0105489 | 0.0658743 | DGKD | 8527 |
| ENSG00000085982 | 682.9054177 | -0.146278399 | 0.1060506 | 0.0622133 | 0.2227815 | USP40 | 55230 |
| ENSG00000123485 | 4026.842249 | -0.072938382 | 0.0585197 | 0.1656347 | 0.4099107 | HJURP | 55355 |
| ENSG00000144481 | 24.38029066 | -0.002011082 | 0.1322573 | 0.9498625 | NA | TRPM8 | 79054 |
| ENSG00000188042 | 8071.681837 | -0.218210526 | 0.0600987 | 7.62E-05 | 0.0014096 | ARL4C | 10123 |
| ENSG00000130147 | 2004.99795 | -0.075516603 | 0.070997 | 0.2132233 | 0.4704065 | SH3BP4 | 23677 |
| ENSG00000157985 | 967.0135116 | -0.138754789 | 0.0918383 | 0.0559487 | 0.2080666 | AGAP1 | 116987 |
| ENSG00000168505 | 287.3311158 | 1.028779789 | 0.1682624 | 5.22E-11 | 4.69E-09 | GBX2 | 2637 |
| ENSG00000233611 | 39.20272019 | -0.003152189 | 0.1308522 | 0.9322038 | NA | NA | NA |
| ENSG00000198612 | 1313.418319 | 0.141416193 | 0.0878835 | 0.0463242 | 0.1841023 | COPS8 | 10920 |
| ENSG00000124839 | 24.89200124 | -0.021646818 | 0.1351965 | 0.4920857 | NA | RAB17 | 64284 |
| ENSG00000124831 | 3156.437136 | -0.079808412 | 0.0616028 | 0.1459427 | 0.3784983 | LRRFIP1 | 9208 |
| ENSG00000177483 | 28.89228618 | -0.04250388 | 0.1407891 | 0.2465703 | NA | RBM44 | 375316 |
| ENSG00000184182 | 181.6427659 | -0.062478856 | 0.1256285 | 0.3546393 | 0.6187516 | UBE2F | 140739 |
| ENSG00000132330 | 116.29914 | 0.045238259 | 0.1274842 | 0.4513535 | 0.6978775 | SCLY | 51540 |
| ENSG00000168427 | 13.62476996 | 0.035767409 | 0.1410931 | 0.1669806 | NA | KLHL30 | 377007 |
| ENSG00000178752 | 366.0551803 | 0.778499802 | 0.1563007 | 3.57E-08 | 1.87E-06 | ERFE | 151176 |
| ENSG00000132323 | 768.0302477 | -0.088048283 | 0.0912644 | 0.2112478 | 0.468055 | ILKAP | 80895 |
| ENSG00000144485 | 503.8212242 | 0.062052096 | 0.0985238 | 0.3841461 | 0.6447633 | HES6 | 55502 |
| ENSG00000132326 | 388.7621701 | 0.126385946 | 0.1323492 | 0.1231589 | 0.3440026 | PER2 | 8864 |
| ENSG00000204104 | 662.8445623 | -0.00061969 | 0.0903093 | 0.9895953 | 0.9955163 | TRAF3IP1 | 26146 |
| ENSG00000065802 | 1118.038778 | -0.037762403 | 0.0778686 | 0.5554144 | 0.7753828 | ASB1 | 51665 |
| ENSG00000068024 | 309.8507066 | -0.05737394 | 0.1122739 | 0.4160451 | 0.6697358 | HDAC4 | 9759 |
| ENSG00000130414 | 2010.4593 | -0.03338256 | 0.0665928 | 0.565211 | 0.7820725 | NDUFA10 | 4705 |
| ENSG00000172428 | 874.4836238 | -0.067368319 | 0.0849473 | 0.3163077 | 0.5849906 | COPS9 | 150678 |
| ENSG00000063660 | 726.5140007 | -0.105119436 | 0.0969589 | 0.1505229 | 0.3866853 | GPC1 | 2817 |
| ENSG00000144504 | 197.7596411 | 0.232893009 | 0.2176372 | 0.0292747 | 0.136184 | ANKMY1 | 51281 |
| ENSG00000188542 | 118.5490223 | 0.111189965 | 0.1653553 | 0.1203476 | 0.3390055 | DUSP28 | 285193 |
| ENSG00000142327 | 810.8320841 | -0.038465381 | 0.0838414 | 0.5615535 | 0.7797796 | RNPEPL1 | 57140 |
| ENSG00000260942 | 63.0165209 | 0.027920362 | 0.1297629 | 0.5692634 | 0.7847328 | CAPN10-DT | 101752400 |
| ENSG00000142330 | 691.6189899 | -0.332950537 | 0.1149765 | 0.0004511 | 0.0059201 | CAPN10 | 11132 |
| ENSG00000162804 | 43.04860565 | -0.010907134 | 0.1302429 | 0.7887372 | 0.9116361 | SNED1 | 25992 |
| ENSG00000122085 | 720.916945 | -0.008703803 | 0.0844034 | 0.8948613 | 0.9579703 | MTERF4 | 130916 |
| ENSG00000115687 | 984.0744338 | 0.007064428 | 0.0781614 | 0.9130802 | 0.9656034 | PASK | 23178 |

| | | | | | | | | |
|---|---|---|---|---|---|---|---|---|
| ENSG00000115685 | 1043.622854 | -0.057991275 | 0.0818404 | 0.3820214 | 0.6430282 | PPP1R7 | | 5510 |
| ENSG00000146205 | 172.546403 | -0.01312318 | 0.1150765 | 0.8326191 | 0.9305286 | ANO7 | | 50636 |
| ENSG00000115677 | 7878.64721 | -0.069417829 | 0.0531844 | 0.1548289 | 0.3932335 | HDLBP | | 3069 |
| ENSG00000268485 | 89.21300282 | 0.042729249 | 0.1305496 | 0.45498 | 0.7000292 | NA | NA | |
| ENSG00000168385 | 10202.37432 | -0.278103092 | 0.0569347 | 1.92E-07 | 8.24E-06 | SEPTIN2 | | 4735 |
| ENSG00000223374 | 24.21712832 | -0.009422513 | 0.1327899 | 0.7738478 | NA | NA | NA | |
| ENSG00000006607 | 1098.661825 | -0.197698257 | 0.09898 | 0.0119423 | 0.0721074 | FARP2 | | 9855 |
| ENSG00000115694 | 3138.197346 | -0.016518527 | 0.0605802 | 0.7602296 | 0.8970427 | STK25 | | 10494 |
| ENSG00000176720 | 1112.187375 | 0.079942407 | 0.0808953 | 0.2289271 | 0.4902363 | BOK | | 666 |
| ENSG00000176946 | 1108.183067 | -0.00709979 | 0.0774738 | 0.9106454 | 0.9648298 | THAP4 | | 51078 |
| ENSG00000168397 | 1649.88586 | -0.074937133 | 0.0727135 | 0.2208312 | 0.4803902 | ATG4B | | 23192 |
| ENSG00000168393 | 2113.271336 | 0.019822505 | 0.0687643 | 0.7393807 | 0.8857761 | DTYMK | | 1841 |
| ENSG00000228989 | 55.3321311 | 0.033855853 | 0.1325774 | 0.4754419 | 0.7152005 | NA | NA | |
| ENSG00000168395 | 1274.415898 | -0.090072753 | 0.079481 | 0.1690145 | 0.414673 | ING5 | | 84289 |
| ENSG00000180902 | 599.1479832 | 0.080750613 | 0.0975531 | 0.2630895 | 0.5306766 | D2HGDH | | 728294 |
| ENSG00000220804 | 36.53668979 | -0.031656372 | 0.1353146 | 0.4232096 | NA | NA | NA | |
| ENSG00000072756 | 903.2681879 | 0.127838295 | 0.0918777 | 0.0770904 | 0.2559674 | TRNT1 | | 51095 |
| ENSG00000113851 | 588.8719705 | -0.079295262 | 0.0987589 | 0.2704635 | 0.5382085 | CRBN | | 51185 |
| ENSG00000271870 | 42.53882083 | -0.035996431 | 0.1361355 | 0.384807 | 0.6453482 | NA | NA | |
| ENSG00000144455 | 266.0216961 | 0.026521408 | 0.1074093 | 0.6966782 | 0.8616368 | SUMF1 | | 285362 |
| ENSG00000170364 | 248.462293 | 0.04527361 | 0.1122994 | 0.5113683 | 0.7421105 | SETMAR | | 6419 |
| ENSG00000150995 | 763.4219478 | 0.175666713 | 0.1032275 | 0.0267608 | 0.1271074 | ITPR1 | | 3708 |
| ENSG00000235947 | 8.61269134 | 0.01607739 | 0.1364967 | 0.4335652 | NA | EGOT | | 100126791 |
| ENSG00000235831 | 17.49449641 | -0.016576399 | 0.135294 | 0.540307 | NA | BHLHE40-A: | | 100507582 |
| ENSG00000134107 | 4354.544722 | 0.18455515 | 0.0673492 | 0.0020673 | 0.0193499 | BHLHE40 | | 8553 |
| ENSG00000134108 | 2398.353682 | 0.144893177 | 0.0734948 | 0.0216746 | 0.1094026 | ARL8B | | 55207 |
| ENSG00000134109 | 1435.70814 | -0.101247423 | 0.0776577 | 0.1184766 | 0.335893 | EDEM1 | | 9695 |
| ENSG00000071282 | 163.9984184 | 0.221932938 | 0.2290283 | 0.0331156 | 0.1468576 | LMCD1 | | 29995 |
| ENSG00000180914 | 314.9360467 | -0.045290685 | 0.110874 | 0.5111776 | 0.7421105 | OXTR | | 5021 |
| ENSG00000070950 | 1439.056275 | -0.030299259 | 0.0719807 | 0.6203086 | 0.818389 | RAD18 | | 56852 |
| ENSG00000196220 | 50.63232304 | -0.339427808 | 1.8221555 | 0.0099696 | 0.0634631 | SRGAP3 | | 9901 |
| ENSG00000206573 | 593.1618188 | -0.094998591 | 0.100368 | 0.1972234 | 0.4504823 | THUMPD3-A | | 440944 |
| ENSG00000134077 | 1100.674469 | 0.130184006 | 0.086229 | 0.0624167 | 0.2233809 | THUMPD3 | | 25917 |
| ENSG00000168137 | 3739.377412 | -0.178035406 | 0.0664162 | 0.0026066 | 0.023151 | SETD5 | | 55209 |
| ENSG00000163719 | 1112.543441 | -0.003397083 | 0.0759085 | 0.9562995 | 0.9825402 | MTMR14 | | 64419 |
| ENSG00000156983 | 1391.425731 | 0.104039255 | 0.0825931 | 0.1226645 | 0.343137 | BRPF1 | | 7862 |
| ENSG00000114026 | 796.3909325 | -0.056697924 | 0.0859299 | 0.4015091 | 0.6592279 | OGG1 | | 4968 |
| ENSG00000134072 | 426.9316321 | -0.075220764 | 0.1110976 | 0.3048867 | 0.573861 | CAMK1 | | 8536 |
| ENSG00000171148 | 1410.836899 | -0.013969625 | 0.0724436 | 0.8194433 | 0.925668 | TADA3 | | 10474 |
| ENSG00000241553 | 1717.211254 | -0.132844224 | 0.0786281 | 0.0435935 | 0.1769341 | ARPC4 | | 10093 |
| ENSG00000214021 | 551.1400279 | 0.062176358 | 0.0987159 | 0.4017952 | 0.6594751 | TTLL3 | | 26140 |
| ENSG00000269886 | 9.637383244 | 0.003888312 | 0.1349284 | 0.8614159 | NA | NA | NA | |
| ENSG00000156990 | 1059.315546 | 0.028540055 | 0.0774088 | 0.6557583 | 0.8412864 | RPUSD3 | | 285367 |
| ENSG00000171135 | 668.6785421 | 0.005973469 | 0.0862016 | 0.9297541 | 0.9728106 | JAGN1 | | 84522 |
| ENSG00000163701 | 18.80641254 | 0.049269549 | 0.1463231 | 0.1062246 | NA | IL17RE | | 132014 |
| ENSG00000163702 | 353.8042949 | -0.067038502 | 0.1113363 | 0.3524424 | 0.6184287 | IL17RC | | 84818 |
| ENSG00000163703 | 194.4145891 | -0.021802268 | 0.115882 | 0.7265479 | 0.8776165 | CRELD1 | | 78987 |
| ENSG00000269894 | 10.99255981 | -0.022716608 | 0.1375551 | 0.3093958 | NA | NA | NA | |
| ENSG00000163704 | 62.27021018 | 0.00908432 | 0.1266648 | 0.8516535 | 0.9396585 | PRRT3 | | 285368 |
| ENSG00000230082 | 12.86762009 | 0.009367251 | 0.134801 | 0.7068136 | NA | PRRT3-AS1 | | 100874032 |
| ENSG00000125037 | 1458.30846 | 0.01966575 | 0.0712046 | 0.748154 | 0.8906631 | EMC3 | | 55831 |
| ENSG00000180385 | 537.4518461 | 0.490815646 | 0.1280722 | 9.67E-06 | 0.0002444 | NA | NA | |
| ENSG00000206567 | 16.42219289 | 0.027295439 | 0.1376544 | 0.3296483 | NA | NA | NA | |
| ENSG00000186162 | 216.8206999 | 0.698405227 | 0.1968818 | 2.09E-05 | 0.0004708 | CIDECP1 | | 152302 |
| ENSG00000144554 | 1778.249558 | -0.118380268 | 0.082101 | 0.0788597 | 0.2597745 | FANCD2 | | 2177 |
| ENSG00000163705 | 10.83201163 | 0.013294812 | 0.1355837 | 0.5675917 | NA | FANCD2OS | | 115795 |
| ENSG00000254999 | 4290.106514 | -0.063590435 | 0.0618371 | 0.2479113 | 0.5141783 | BRK1 | | 55845 |
| ENSG00000134086 | 2610.023189 | 0.080032121 | 0.0643381 | 0.1585663 | 0.39884 | VHL | | 7428 |
| ENSG00000134070 | 1552.416759 | 0.247789613 | 0.0861608 | 0.0008032 | 0.0093731 | IRAK2 | | 3656 |
| ENSG00000157014 | 979.2899875 | -0.025759153 | 0.0805618 | 0.6919576 | 0.8594028 | TATDN2 | | 9797 |
| ENSG00000231177 | 12.82928334 | -0.055379485 | 0.151344 | 0.0317126 | NA | LINC00852 | | 84657 |
| ENSG00000240288 | 17.12539004 | -0.001331659 | 0.1336236 | 0.969022 | NA | GHRLOS | | 100126793 |
| ENSG00000157020 | 2327.253121 | -0.056569592 | 0.0683761 | 0.3421349 | 0.6088638 | SEC13 | | 6396 |
| ENSG00000196639 | 219.725804 | 0.110039741 | 0.141812 | 0.156164 | 0.3950091 | HRH1 | | 3269 |
| ENSG00000197548 | 905.0417792 | -0.085833801 | 0.0864893 | 0.2096938 | 0.4663117 | ATG7 | | 10533 |
| ENSG00000144560 | 728.4125909 | -0.021232872 | 0.087123 | 0.7514779 | 0.8926188 | VGLL4 | | 9686 |
| ENSG00000144559 | 184.1106763 | -0.052976136 | 0.1206714 | 0.4283444 | 0.6796361 | TAMM41 | | 132001 |
| ENSG00000132170 | 195.8033784 | 0.016164841 | 0.1150966 | 0.7989035 | 0.9162772 | PPARG | | 5468 |
| ENSG00000154743 | 343.4160015 | 0.122135384 | 0.1269649 | 0.1300169 | 0.3544317 | TSEN2 | | 80746 |
| ENSG00000075975 | 1024.409243 | 0.041978452 | 0.0791599 | 0.5125416 | 0.742896 | MKRN2 | | 23609 |

| | | | | | | | | |
|---|---|---|---|---|---|---|---|---|
| ENSG00000132155 | 3842.973039 | -0.085570433 | 0.0639606 | 0.1294753 | 0.353901 | RAF1 | | 5894 |
| ENSG00000088726 | 1577.081131 | 0.010526009 | 0.0792194 | 0.8713689 | 0.9482448 | TMEM40 | | 55287 |
| ENSG00000144712 | 307.8615638 | 0.018451854 | 0.1056993 | 0.7863203 | 0.9110303 | CAND2 | | 23066 |
| ENSG00000272263 | 20.95637995 | 0.015586029 | 0.1340997 | 0.6200012 | NA | NA | NA | |
| ENSG00000144713 | 13131.09357 | -0.185028043 | 0.0646162 | 0.001416 | 0.0147022 | RPL32 | | 6161 |
| ENSG00000144711 | 766.7087617 | -0.103517113 | 0.0967999 | 0.1563819 | 0.3953034 | IQSEC1 | | 9922 |
| ENSG00000132182 | 4764.212981 | -0.089602155 | 0.0599546 | 0.0955975 | 0.2938201 | NUP210 | | 23225 |
| ENSG00000163517 | 353.5001592 | 0.038383528 | 0.1031326 | 0.5821324 | 0.7935506 | HDAC11 | | 79885 |
| ENSG00000154764 | 64.67107101 | 0.035977188 | 0.1321127 | 0.4680376 | 0.7103207 | WNT7A | | 7476 |
| ENSG00000163528 | 711.8923035 | 0.3079268 | 0.1130668 | 0.0008365 | 0.009624 | CHCHD4 | | 131474 |
| ENSG00000170876 | 2709.985262 | -0.045909549 | 0.0625947 | 0.4084609 | 0.6640358 | TMEM43 | | 79188 |
| ENSG00000268279 | 25.59399183 | -0.007793362 | 0.1323366 | 0.8174413 | NA | NA | NA | |
| ENSG00000154767 | 1134.467815 | -0.037021439 | 0.0793268 | 0.5670995 | 0.7831055 | XPC | | 7508 |
| ENSG00000170860 | 1915.256189 | -0.111304485 | 0.0746521 | 0.0779454 | 0.2574886 | LSM3 | | 27258 |
| ENSG00000131389 | 2768.012555 | -0.369138293 | 0.0851539 | 1.66E-06 | 5.23E-05 | SLC6A6 | | 6533 |
| ENSG00000154781 | 732.7287638 | 0.484706895 | 0.1220435 | 5.59E-06 | 0.000152 | CCDC174 | | 51244 |
| ENSG00000131379 | 24.37265662 | -0.002934868 | 0.132374 | 0.9261244 | NA | C3orf20 | | 84077 |
| ENSG00000225733 | 3946.872005 | -0.095489561 | 0.0603502 | 0.0774091 | 0.2565722 | FGD5-AS1 | | 100505641 |
| ENSG00000177463 | 611.8357741 | -0.081542829 | 0.0970635 | 0.2575402 | 0.5243228 | NR2C2 | | 7182 |
| ENSG00000131368 | 2146.383121 | 0.22992788 | 0.0740335 | 0.000447 | 0.0058794 | MRPS25 | | 64432 |
| ENSG00000131381 | 1121.039416 | -0.104105301 | 0.0832096 | 0.1233208 | 0.3442934 | RBSN | | 64145 |
| ENSG00000131375 | 1074.096795 | -0.150091032 | 0.0969209 | 0.0461122 | 0.1838349 | CAPN7 | | 23473 |
| ENSG00000224660 | 398.4527204 | 0.103090763 | 0.1150475 | 0.1806931 | 0.4299157 | SH3BP5-AS | | 100505696 |
| ENSG00000131370 | 680.0604322 | -0.006403643 | 0.0861789 | 0.922867 | 0.9702979 | SH3BP5 | | 9467 |
| ENSG00000206562 | 380.249237 | 0.108249295 | 0.1227082 | 0.1669776 | 0.4122331 | METTL6 | | 131965 |
| ENSG00000144597 | 1085.03514 | 0.342439328 | 0.09563 | 4.17E-05 | 0.0008436 | EAF1 | | 85403 |
| ENSG00000249786 | 16.57547094 | -0.002510768 | 0.1336693 | 0.9263259 | NA | NA | NA | |
| ENSG00000206561 | 18.19257007 | -0.003934446 | 0.1335572 | 0.8883831 | NA | COLQ | | 8292 |
| ENSG00000131373 | 1372.577974 | -0.134132782 | 0.0857138 | 0.0540272 | 0.2032501 | HACL1 | | 26061 |
| ENSG00000169814 | 491.4674787 | -0.069927622 | 0.1004116 | 0.3304733 | 0.5987682 | BTD | | 686 |
| ENSG00000206560 | 3250.925454 | -0.155911146 | 0.0721618 | 0.0126958 | 0.0752788 | ANKRD28 | | 23243 |
| ENSG00000263740 | 36.55488039 | -1.156898685 | 0.698259 | 0.0023112 | NA | RN7SL4P | | 6030 |
| ENSG00000224728 | 18.137634 | 0.001630412 | 0.1336998 | 0.9554994 | NA | NA | NA | |
| ENSG00000154813 | 562.1673965 | -0.009234565 | 0.0944704 | 0.891435 | 0.9562439 | DPH3 | | 285381 |
| ENSG00000154814 | 300.5569085 | 0.051924663 | 0.1091989 | 0.4644101 | 0.7082689 | OXNAD1 | | 92106 |
| ENSG00000131378 | 757.9890876 | 0.058811728 | 0.0888882 | 0.3930369 | 0.6537184 | RFTN1 | | 23180 |
| ENSG00000154822 | 57.85586206 | 0.064994955 | 0.1467712 | 0.2004058 | 0.4540225 | PLCL2 | | 23228 |
| ENSG00000131374 | 1456.267452 | -0.022522648 | 0.0709634 | 0.7099659 | 0.8682978 | TBC1D5 | | 9779 |
| ENSG00000182568 | 25.56453016 | -0.028842736 | 0.1365897 | 0.3875982 | NA | SATB1 | | 6304 |
| ENSG00000163576 | 8.839482266 | -0.015745693 | 0.1363664 | 0.4481416 | NA | EFHB | | 151651 |
| ENSG00000144566 | 1092.994585 | -0.011028134 | 0.0760314 | 0.8606763 | 0.9439076 | RAB5A | | 5868 |
| ENSG00000114166 | 300.0821656 | -0.175355284 | 0.1572152 | 0.0552329 | 0.206258 | KAT2B | | 8850 |
| ENSG00000129810 | 569.0887847 | 0.046232885 | 0.0945741 | 0.5073278 | 0.7395602 | SGO1 | | 151648 |
| ENSG00000237485 | 8.692614452 | 0.018904431 | 0.1372512 | 0.3219872 | NA | NA | NA | |
| ENSG00000132967 | 110.066401 | -0.435748662 | 0.3223887 | 0.0071256 | 0.0494307 | HMGB1P5 | | 10354 |
| ENSG00000182247 | 385.7062459 | 0.063359612 | 0.1068796 | 0.3791259 | 0.6404294 | UBE2E2 | | 7325 |
| ENSG00000170142 | 1678.503543 | -0.02800994 | 0.0697152 | 0.6394672 | 0.8306035 | UBE2E1 | | 7324 |
| ENSG00000197885 | 216.2874616 | -0.053798769 | 0.1191806 | 0.4262217 | 0.678557 | NKIRAS1 | | 28512 |
| ENSG00000174748 | 14784.19139 | -0.138525323 | 0.0561387 | 0.0068733 | 0.0480881 | RPL15 | | 6138 |
| ENSG00000174738 | 669.5000348 | 0.099070319 | 0.097058 | 0.1744705 | 0.4222939 | NR1D2 | | 9975 |
| ENSG00000151090 | 22.112473 | -0.010930207 | 0.1340207 | 0.7032856 | NA | THRB | | 7068 |
| ENSG00000077097 | 2334.171545 | -0.473294561 | 0.0832622 | 1.14E-09 | 8.28E-08 | TOP2B | | 7155 |
| ENSG00000151092 | 444.6171264 | -0.032373168 | 0.0965504 | 0.6388933 | 0.8300426 | NGLY1 | | 55768 |
| ENSG00000151093 | 233.5570893 | -0.045481765 | 0.1155549 | 0.4998507 | 0.734688 | OXSM | | 54995 |
| ENSG00000163491 | 60.49250975 | 0.212327931 | 0.4562802 | 0.0127432 | 0.0754451 | NEK10 | | 152110 |
| ENSG00000033867 | 3340.979838 | 0.062842728 | 0.0632453 | 0.2622894 | 0.5299265 | SLC4A7 | | 9497 |
| ENSG00000213846 | 34.48855564 | 0.045358509 | 0.1409208 | 0.2566457 | NA | NA | NA | |
| ENSG00000163508 | 22.21751139 | 0.037876971 | 0.140143 | 0.2466651 | NA | EOMES | | 8320 |
| ENSG00000187118 | 286.0702391 | -0.086010053 | 0.1229521 | 0.2469464 | 0.5129849 | CMC1 | | 152100 |
| ENSG00000163512 | 640.9973073 | -0.074689982 | 0.0941605 | 0.2917129 | 0.5613672 | AZI2 | | 64343 |
| ENSG00000206559 | 17.23559763 | 0.020047295 | 0.135778 | 0.4756161 | NA | ZCWPW2 | | 152098 |
| ENSG00000144642 | 97.08168801 | 0.113922273 | 0.1755639 | 0.0973791 | 0.296916 | RBMS3 | | 27303 |
| ENSG00000163513 | 912.4580267 | -0.29554739 | 0.1134394 | 0.0012402 | 0.0132905 | TGFBR2 | | 7048 |
| ENSG00000163527 | 3922.595555 | -0.15899058 | 0.0620224 | 0.0043335 | 0.0339046 | STT3B | | 201595 |
| ENSG00000144645 | 1735.240056 | -0.055250474 | 0.0705717 | 0.3608668 | 0.6241168 | OSBPL10 | | 114884 |
| ENSG00000197385 | 145.3619017 | 0.092632694 | 0.1473334 | 0.1839939 | 0.4341008 | ZNF860 | | 344787 |
| ENSG00000152642 | 687.0666661 | -0.062823347 | 0.0918905 | 0.3737831 | 0.6352333 | GPD1L | | 23171 |
| ENSG00000261572 | 9.250531496 | 0.031435265 | 0.1404734 | 0.1307974 | NA | NA | NA | |
| ENSG00000170293 | 120.7793989 | 0.020369826 | 0.1212142 | 0.7273662 | 0.8780864 | CMTM8 | | 152189 |
| ENSG00000153551 | 375.5864029 | -0.057222797 | 0.1063198 | 0.4215302 | 0.6737677 | CMTM7 | | 112616 |

| | | | | | | | |
|---|---|---|---|---|---|---|---|
| ENSG00000091317 | 2134.835634 | -0.195802387 | 0.0756179 | 0.0028927 | 0.0250481 | CMTM6 | 54918 |
| ENSG00000144635 | 1382.578734 | -0.085717701 | 0.0768288 | 0.1817562 | 0.4314356 | DYNC1LI1 | 51143 |
| ENSG00000182973 | 789.2817128 | 0.040509744 | 0.084954 | 0.545979 | 0.7680131 | CNOT10 | 25904 |
| ENSG00000170266 | 909.0576135 | -0.021446135 | 0.0824392 | 0.743875 | 0.8886411 | GLB1 | 2720 |
| ENSG00000188167 | 69.58086105 | -0.019506239 | 0.1273647 | 0.6932448 | 0.8597393 | TMPPE | 643853 |
| ENSG00000170275 | 1459.825915 | -0.05619741 | 0.073013 | 0.3638037 | 0.6265985 | CRTAP | 10491 |
| ENSG00000153558 | 125.9867083 | 0.112368189 | 0.1657193 | 0.1187392 | 0.3361592 | FBXL2 | 25827 |
| ENSG00000153560 | 2302.331871 | -0.072467768 | 0.0664303 | 0.2115871 | 0.4681851 | UBP1 | 7342 |
| ENSG00000163539 | 1323.842864 | -0.091753715 | 0.0798327 | 0.1625683 | 0.4047229 | CLASP2 | 23122 |
| ENSG00000271324 | 10.11672566 | -0.018775014 | 0.136741 | 0.3920519 | NA | NA | NA |
| ENSG00000271643 | 75.60391055 | 0.034157265 | 0.1293443 | 0.5177903 | 0.7470737 | PDCD6IP-D | 105377023 |
| ENSG00000271020 | 56.86425397 | 0.015261315 | 0.1282474 | 0.7425752 | 0.887822 | NA | NA |
| ENSG00000170248 | 3551.793955 | 0.013740769 | 0.0604732 | 0.7962551 | 0.9152729 | PDCD6IP | 10015 |
| ENSG00000168016 | 176.4660495 | -0.072202499 | 0.1295817 | 0.2949108 | 0.5650592 | TRANK1 | 9881 |
| ENSG00000178567 | 563.5340472 | 0.019232702 | 0.0904639 | 0.7781555 | 0.9075115 | EPM2AIP1 | 9852 |
| ENSG00000076242 | 843.2758109 | -0.093115771 | 0.0895818 | 0.1830667 | 0.432875 | MLH1 | 4292 |
| ENSG00000093167 | 1109.408341 | 0.098507169 | 0.0854921 | 0.1530427 | 0.3907524 | LRRFIP2 | 9209 |
| ENSG00000224080 | 44.32402566 | 0.008047579 | 0.1300493 | 0.8457066 | 0.9369039 | NA | NA |
| ENSG00000144674 | 2479.284516 | -0.039466177 | 0.0692784 | 0.5080546 | 0.7401841 | GOLGA4 | 2803 |
| ENSG00000230409 | 39.02037285 | -0.015090672 | 0.1310515 | 0.7068439 | NA | NA | NA |
| ENSG00000198590 | 11.57017509 | 0.011411624 | 0.135383 | 0.6205634 | NA | APRG1 | 339883 |
| ENSG00000235257 | 23.64039018 | -0.016714674 | 0.133862 | 0.6084982 | NA | ITGA9-AS1 | 101928153 |
| ENSG00000144677 | 915.4693779 | -0.306943325 | 0.1029321 | 0.0003853 | 0.0052889 | CTDSPL | 10217 |
| ENSG00000187091 | 103.6193745 | -0.016513811 | 0.1228614 | 0.7645207 | 0.898608 | PLCD1 | 5333 |
| ENSG00000060971 | 865.8605596 | -0.116044774 | 0.0935742 | 0.1089799 | 0.3196872 | ACAA1 | 30 |
| ENSG00000172936 | 742.934111 | -0.126843772 | 0.0987893 | 0.0899904 | 0.2831836 | MYD88 | 4615 |
| ENSG00000172939 | 1621.117549 | -0.007746843 | 0.0692502 | 0.8959708 | 0.9584283 | OXSR1 | 9943 |
| ENSG00000093217 | 220.4921943 | 0.001563676 | 0.1104836 | 0.9820616 | 0.9932861 | XYLB | 9942 |
| ENSG00000114739 | 229.4033536 | -0.032396476 | 0.1117011 | 0.6295392 | 0.8236154 | ACVR2B | 93 |
| ENSG00000157036 | 440.4995056 | 0.023683469 | 0.097349 | 0.7309424 | 0.8803112 | EXOG | 9941 |
| ENSG00000183873 | 29.13538025 | -0.009465148 | 0.1319586 | 0.7896149 | NA | SCN5A | 6331 |
| ENSG00000114742 | 727.7018412 | -0.147966494 | 0.1020367 | 0.0549624 | 0.2055108 | WDR48 | 57599 |
| ENSG00000114745 | 1265.879926 | -0.030718595 | 0.0740814 | 0.621485 | 0.818389 | GORASP1 | 64689 |
| ENSG00000168026 | 73.44569106 | -0.080362737 | 0.1527402 | 0.1650848 | 0.4090699 | TTC21A | 199223 |
| ENSG00000144655 | 507.4204585 | 0.298888988 | 0.1495979 | 0.0051917 | 0.0391759 | CSRNP1 | 64651 |
| ENSG00000144659 | 653.540036 | 0.112680526 | 0.101031 | 0.1338916 | 0.3604716 | SLC25A38 | 54977 |
| ENSG00000168028 | 22994.6259 | -0.257513043 | 0.0556434 | 5.59E-07 | 2.07E-05 | RPSA | 3921 |
| ENSG00000114784 | 439.0141245 | 0.131154486 | 0.1196928 | 0.104515 | 0.3104094 | EIF1B | 10289 |
| ENSG00000223797 | 12.61557319 | 0.001320194 | 0.1344183 | 0.9589325 | NA | ENTPD3-AS | 285266 |
| ENSG00000188846 | 9186.851666 | -0.065009497 | 0.053459 | 0.1820716 | 0.4317028 | RPL14 | 9045 |
| ENSG00000272452 | 52.19916913 | 0.018151877 | 0.1297705 | 0.6856703 | 0.8563365 | NA | NA |
| ENSG00000177873 | 79.31934232 | 0.144236401 | 0.2207575 | 0.0473957 | 0.1859327 | ZNF619 | 285267 |
| ENSG00000177842 | 103.4183675 | -0.036341294 | 0.1266024 | 0.5164227 | 0.7456659 | ZNF620 | 253639 |
| ENSG00000172888 | 991.2837756 | 0.037213657 | 0.0793557 | 0.5653629 | 0.7820725 | ZNF621 | 285268 |
| ENSG00000234287 | 19.2640137 | -0.037777775 | 0.1407645 | 0.2123124 | NA | NA | NA |
| ENSG00000168036 | 3961.945811 | -0.376379314 | 0.069181 | 6.21E-09 | 3.90E-07 | CTNNB1 | 1499 |
| ENSG00000168038 | 51.34710685 | 0.055473575 | 0.1432488 | 0.2329349 | 0.4953419 | ULK4 | 54986 |
| ENSG00000182606 | 1010.603739 | 0.230648831 | 0.1061691 | 0.0060365 | 0.0435839 | TRAK1 | 22906 |
| ENSG00000093183 | 1440.716408 | -0.232750889 | 0.0866168 | 0.0015609 | 0.0158138 | SEC22C | 9117 |
| ENSG00000008324 | 358.0381956 | -0.349820384 | 0.162679 | 0.0027824 | 0.0243271 | SS18L2 | 51188 |
| ENSG00000114857 | 2162.984536 | 0.03677088 | 0.0655912 | 0.5241961 | 0.7515816 | NKTR | 4820 |
| ENSG00000114853 | 195.8572313 | 0.012987508 | 0.1116725 | 0.8424713 | 0.9350382 | ZBTB47 | 92999 |
| ENSG00000181061 | 2253.897284 | -0.209768791 | 0.0731537 | 0.0011182 | 0.0121963 | HIGD1A | 25994 |
| ENSG00000144647 | 582.5751723 | -0.03050211 | 0.0902855 | 0.6484468 | 0.836587 | POMGNT2 | 84892 |
| ENSG00000163788 | 596.7698113 | -0.012006541 | 0.0885811 | 0.8579642 | 0.9428541 | SNRK | 54861 |
| ENSG00000271941 | 9.819626983 | 0.0173945 | 0.1364569 | 0.4329264 | NA | NA | NA |
| ENSG00000234617 | 8.610085808 | -0.007736013 | 0.1353784 | 0.7058208 | NA | NA | NA |
| ENSG00000160746 | 550.5503732 | -0.092579146 | 0.1020697 | 0.2094891 | 0.4660901 | ANO10 | 55129 |
| ENSG00000011198 | 393.6919142 | 0.049175417 | 0.1095844 | 0.4826107 | 0.7213852 | ABHD5 | 51099 |
| ENSG00000272121 | 47.7053865 | 0.059145543 | 0.1450512 | 0.2114624 | 0.4681851 | NA | NA |
| ENSG00000261786 | 135.3995113 | 0.034219413 | 0.1220985 | 0.5758592 | 0.7895084 | NA | NA |
| ENSG00000179152 | 383.146954 | -0.095347759 | 0.1139475 | 0.2089988 | 0.4654027 | TCAIM | 285343 |
| ENSG00000185219 | 1192.590282 | 0.041902724 | 0.0802239 | 0.5214418 | 0.7492879 | ZNF445 | 353274 |
| ENSG00000178917 | 113.8969134 | 0.010162137 | 0.1203413 | 0.8613568 | 0.9442993 | ZNF852 | 285346 |
| ENSG00000186448 | 293.9062276 | 0.083059828 | 0.1194534 | 0.2614815 | 0.5289859 | ZNF197 | 10168 |
| ENSG00000169981 | 356.2546116 | -0.031086482 | 0.1027728 | 0.6522517 | 0.8389068 | ZNF35 | 7584 |
| ENSG00000272077 | 110.9441892 | -0.026278859 | 0.1227764 | 0.6498773 | 0.8371434 | NA | NA |
| ENSG00000163807 | 1314.200483 | -0.013314644 | 0.0773241 | 0.8340003 | 0.9305701 | KIAA1143 | 57456 |
| ENSG00000163808 | 1291.793122 | -0.105969279 | 0.0825389 | 0.1152441 | 0.3305909 | KIF15 | 56992 |
| ENSG00000169964 | 188.7616623 | 0.273975977 | 0.223977 | 0.0188147 | 0.0995356 | TMEM42 | 131616 |

| | | | | | | | | |
|---|---|---|---|---|---|---|---|---|
| ENSG00000163810 | 114.4458592 | -0.121353618 | 0.1747514 | 0.0988961 | 0.3002092 | TGM4 | | 7047 |
| ENSG00000163812 | 1543.385503 | -0.217359762 | 0.0818634 | 0.0019483 | 0.0185504 | ZDHHC3 | | 51304 |
| ENSG00000075914 | 848.2778039 | 0.11372229 | 0.0967943 | 0.1220743 | 0.3422829 | EXOSC7 | | 23016 |
| ENSG00000163814 | 6378.333018 | 0.484445082 | 0.0577084 | 4.39E-18 | 1.00E-15 | CDCP1 | | 64866 |
| ENSG00000249992 | 95.28475686 | 0.748191968 | 0.323949 | 0.0008306 | 0.0095867 | TMEM158 | | 25907 |
| ENSG00000011376 | 882.1675372 | -0.102952555 | 0.0952511 | 0.1556547 | 0.3943696 | LARS2 | | 23395 |
| ENSG00000144791 | 1994.430914 | 0.092586201 | 0.0741244 | 0.1402198 | 0.3695639 | LIMD1 | | 8994 |
| ENSG00000230530 | 32.59643594 | -0.033870958 | 0.1372152 | 0.3563178 | NA | NA | NA | |
| ENSG00000211456 | 1101.454801 | -0.157972461 | 0.0924728 | 0.0320029 | 0.1441671 | SACM1L | | 22908 |
| ENSG00000163818 | 242.6528007 | -0.002175692 | 0.108998 | 0.9725023 | 0.9893759 | LZTFL1 | | 54585 |
| ENSG00000163820 | 720.4490487 | -0.008203461 | 0.0848849 | 0.9010214 | 0.9612514 | FYCO1 | | 79443 |
| ENSG00000178038 | 495.750096 | 0.248824997 | 0.1361603 | 0.0104022 | 0.0652215 | ALS2CL | | 259173 |
| ENSG00000160799 | 728.1722741 | -0.218626966 | 0.109222 | 0.0094186 | 0.0614142 | CCDC12 | | 151903 |
| ENSG00000160796 | 1557.729218 | -0.053899763 | 0.0772833 | 0.3859673 | 0.6466557 | NBEAL2 | | 23218 |
| ENSG00000181555 | 2247.782751 | -0.106268654 | 0.0749646 | 0.0933149 | 0.2894347 | SETD2 | | 29072 |
| ENSG00000227398 | 22.90494448 | 0.026946873 | 0.1362235 | 0.4145126 | NA | NA | NA | |
| ENSG00000088727 | 140.2933351 | 0.052750228 | 0.1271603 | 0.4042608 | 0.6612758 | KIF9 | | 64147 |
| ENSG00000114648 | 1232.190301 | 0.18942462 | 0.0939693 | 0.0125416 | 0.0747823 | KLHL18 | | 23276 |
| ENSG00000260236 | 24.85089485 | -0.005651898 | 0.1323179 | 0.8646935 | NA | NA | NA | |
| ENSG00000076201 | 1205.560443 | 0.058805751 | 0.0769661 | 0.3556637 | 0.6192724 | PTPN23 | | 25930 |
| ENSG00000114650 | 1109.967034 | -0.075079524 | 0.081757 | 0.2587502 | 0.5253712 | SCAP | | 22937 |
| ENSG00000163832 | 781.3698722 | 0.014742895 | 0.084649 | 0.8333075 | 0.9305394 | ELP6 | | 54859 |
| ENSG00000114646 | 134.6705907 | 0.022202443 | 0.1188902 | 0.7164106 | 0.8714352 | CSPG5 | | 10675 |
| ENSG00000173473 | 3470.62362 | -0.146725782 | 0.0722804 | 0.0187372 | 0.0992261 | SMARCC1 | | 6599 |
| ENSG00000132153 | 3188.609248 | -0.065646806 | 0.062546 | 0.2374499 | 0.5019278 | DHX30 | | 22907 |
| ENSG00000047849 | 5706.565772 | -0.171875121 | 0.0646595 | 0.0029374 | 0.0253409 | MAP4 | | 4134 |
| ENSG00000164045 | 1502.30481 | 0.400025398 | 0.0868349 | 4.22E-07 | 1.62E-05 | CDC25A | | 993 |
| ENSG00000164048 | 432.0958298 | 0.474943293 | 0.1430444 | 6.69E-05 | 0.0012575 | ZNF589 | | 51385 |
| ENSG00000228638 | 105.7772535 | -0.003388133 | 0.1208087 | 0.9504403 | 0.9802173 | NA | NA | |
| ENSG00000172113 | 448.3144392 | 0.039019347 | 0.1019236 | 0.5778188 | 0.7911317 | NME6 | | 10201 |
| ENSG00000164050 | 738.3879253 | -0.256646716 | 0.112155 | 0.0036721 | 0.0298398 | PLXNB1 | | 5364 |
| ENSG00000164051 | 318.2488402 | 0.211813988 | 0.1588042 | 0.030344 | 0.1393817 | CCDC51 | | 79714 |
| ENSG00000232112 | 2558.999628 | -0.189501618 | 0.0788066 | 0.0049453 | 0.0376802 | TMA7 | | 51372 |
| ENSG00000244380 | 48.28340157 | -0.029853774 | 0.133413 | 0.4846959 | 0.7231745 | NA | NA | |
| ENSG00000164053 | 326.9480278 | 0.039137499 | 0.1046135 | 0.5736488 | 0.7879576 | ATRIP | | 84126 |
| ENSG00000213689 | 68.57975965 | 0.086355195 | 0.1592982 | 0.132147 | 0.3581236 | TREX1 | | 11277 |
| ENSG00000164054 | 1937.822674 | 0.099283165 | 0.0750649 | 0.1181148 | 0.3350411 | SHISA5 | | 51246 |
| ENSG00000114268 | 145.9942077 | -0.374426742 | 0.2680175 | 0.0086099 | 0.0572084 | PFKFB4 | | 5210 |
| ENSG00000145040 | 11.34249841 | -0.005020485 | 0.1347765 | 0.8255159 | NA | UCN2 | | 90226 |
| ENSG00000114270 | 672.3313534 | -0.065911615 | 0.0974437 | 0.3542515 | 0.6187516 | COL7A1 | | 1294 |
| ENSG00000010256 | 4342.292731 | -0.103257457 | 0.0587201 | 0.0509476 | 0.1946714 | UQCRC1 | | 7384 |
| ENSG00000183396 | 9.146829137 | -0.00563713 | 0.1351917 | 0.7842034 | NA | TMEM89 | | 440955 |
| ENSG00000225697 | 802.5540515 | -0.383747106 | 0.1095784 | 4.60E-05 | 0.0009161 | SLC26A6 | | 65010 |
| ENSG00000008300 | 1559.033578 | 0.285073862 | 0.0856187 | 0.0001412 | 0.0022932 | CELSR3 | | 1951 |
| ENSG00000213672 | 1056.689663 | 0.141026577 | 0.0892329 | 0.0489309 | 0.1898875 | NCKIPSD | | 51517 |
| ENSG00000068745 | 1105.91746 | 0.013398771 | 0.0773129 | 0.8343587 | 0.930686 | IP6K2 | | 51447 |
| ENSG00000114302 | 2411.708322 | -0.224739378 | 0.0719912 | 0.0004389 | 0.0058292 | PRKAR2A | | 5576 |
| ENSG00000224424 | 19.06211483 | 0.021622539 | 0.1361222 | 0.4436099 | NA | PRKAR2A-A | | 100506637 |
| ENSG00000178537 | 395.3300127 | 0.213652396 | 0.1458075 | 0.0256003 | 0.1230675 | SLC25A20 | | 788 |
| ENSG00000221883 | 14.77803042 | 0.02900685 | 0.1386694 | 0.2616325 | NA | NA | NA | |
| ENSG00000177479 | 3179.173559 | 0.108829015 | 0.0645626 | 0.0565815 | 0.2098187 | ARIH2 | | 10425 |
| ENSG00000235236 | 14.5606723 | 0.001558067 | 0.1340289 | 0.9549508 | NA | NA | NA | |
| ENSG00000223343 | 16.37909656 | 0.018518955 | 0.1354626 | 0.5096907 | NA | NA | NA | |
| ENSG00000178467 | 309.7146273 | 0.115108357 | 0.129992 | 0.1481888 | 0.3822865 | P4HTM | | 54681 |
| ENSG00000178252 | 4027.19904 | -0.096217403 | 0.0591758 | 0.0707854 | 0.2421213 | WDR6 | | 11180 |
| ENSG00000178149 | 313.9820348 | -0.021876194 | 0.1044565 | 0.7471431 | 0.8900947 | DALRD3 | | 55152 |
| ENSG00000178057 | 460.6257719 | -0.030212054 | 0.097262 | 0.6609745 | 0.8430186 | NDUFAF3 | | 25915 |
| ENSG00000178035 | 3174.017323 | -0.019388618 | 0.0598514 | 0.7176059 | 0.8722533 | IMPDH2 | | 3615 |
| ENSG00000272434 | 19.12834033 | -0.009828136 | 0.1338871 | 0.7348557 | NA | NA | NA | |
| ENSG00000198218 | 2293.819284 | -0.153782501 | 0.0725354 | 0.0140402 | 0.0807899 | QRICH1 | | 54870 |
| ENSG00000172053 | 2673.432725 | -0.076710037 | 0.0643586 | 0.1764444 | 0.4254845 | QARS1 | | 5859 |
| ENSG00000172046 | 1358.998669 | 0.046622031 | 0.0762432 | 0.4636699 | 0.7079196 | USP19 | | 10869 |
| ENSG00000172037 | 3153.451736 | -0.058872433 | 0.0660386 | 0.3076207 | 0.5769089 | LAMB2 | | 3913 |
| ENSG00000177352 | 171.1236317 | -0.019226813 | 0.1146586 | 0.7612832 | 0.8976026 | CCDC71 | | 64925 |
| ENSG00000185909 | 144.8087133 | 0.022057424 | 0.1197752 | 0.7148464 | 0.8710746 | KLHDC8B | | 200942 |
| ENSG00000188315 | 277.7809322 | 0.124516636 | 0.1386088 | 0.126465 | 0.3490637 | C3orf62 | | 375341 |
| ENSG00000114316 | 933.7670735 | 0.025038381 | 0.080283 | 0.7011262 | 0.864704 | USP4 | | 7375 |
| ENSG00000233276 | 4850.14695 | -0.033580738 | 0.0574284 | 0.5149277 | 0.7449955 | GPX1 | | 2876 |
| ENSG00000067560 | 8375.500313 | -0.16532876 | 0.0584332 | 0.0018644 | 0.0180137 | RHOA | | 387 |
| ENSG00000145022 | 479.003667 | 0.491573564 | 0.1357738 | 2.16E-05 | 0.0004839 | TCTA | | 6988 |

| | | | | | | | |
|---|---|---|---|---|---|---|---|
| ENSG00000145020 | 17.58379058 | 0.00277596 | 0.1336638 | 0.9234589 | NA | AMT | 275 |
| ENSG00000145029 | 64.80232408 | 0.032029886 | 0.130657 | 0.5191273 | 0.7478713 | NICN1 | 84276 |
| ENSG00000173402 | 3645.398591 | -0.165680421 | 0.0649707 | 0.0042036 | 0.0331323 | DAG1 | 1605 |
| ENSG00000164061 | 41.10014594 | 0.15010862 | 0.2677963 | 0.014412 | 0.0819203 | BSN | 8927 |
| ENSG00000164062 | 2698.10934 | -0.242531099 | 0.0776648 | 0.000373 | 0.0051618 | APEH | 327 |
| ENSG00000173531 | 21.65083887 | 0.005646367 | 0.1330348 | 0.8578619 | NA | MST1 | 4485 |
| ENSG00000164068 | 1092.338709 | -0.044983733 | 0.0797724 | 0.4887415 | 0.7262421 | RNF123 | 63891 |
| ENSG00000176095 | 1284.031488 | 0.112297407 | 0.0829634 | 0.0974107 | 0.2969349 | IP6K1 | 9807 |
| ENSG00000185614 | 35.22721508 | 0.025243695 | 0.1335255 | 0.5238972 | NA | INKA1 | 389119 |
| ENSG00000182179 | 25.16231535 | 0.014569831 | 0.1332003 | 0.6690469 | NA | UBA7 | 7318 |
| ENSG00000183763 | 546.5204243 | 0.079996368 | 0.1014371 | 0.2761955 | 0.5444212 | TRAIP | 10293 |
| ENSG00000164076 | 8.81373894 | -0.012806787 | 0.1361224 | 0.5111719 | NA | CAMKV | 79012 |
| ENSG00000164078 | 1682.67596 | -0.012871295 | 0.0697568 | 0.8292205 | 0.9296029 | MST1R | 4486 |
| ENSG00000164077 | 396.832954 | 0.142155635 | 0.1273007 | 0.087847 | 0.2788425 | MON1A | 84315 |
| ENSG00000004534 | 1562.331572 | -0.17691136 | 0.0821349 | 0.0106145 | 0.0660212 | RBM6 | 10180 |
| ENSG00000003756 | 2049.790285 | -0.124417262 | 0.0720856 | 0.0447541 | 0.1800646 | RBM5 | 10181 |
| ENSG00000235016 | 18.29464763 | 0.033098295 | 0.1395053 | 0.2429513 | NA | SEMA3F-AS | 100129060 |
| ENSG00000001617 | 103.9766423 | 0.098721827 | 0.1595036 | 0.1400541 | 0.369544 | SEMA3F | 6405 |
| ENSG00000114353 | 4169.598626 | -0.123447687 | 0.0635268 | 0.0288411 | 0.1348113 | GNAI2 | 2771 |
| ENSG00000214706 | 2580.014137 | 0.073097812 | 0.0662716 | 0.2052038 | 0.4606924 | IFRD2 | 7866 |
| ENSG00000186792 | 182.647784 | 0.128202641 | 0.1583675 | 0.1116586 | 0.3242067 | HYAL3 | 8372 |
| ENSG00000243477 | 105.6480236 | 0.074203334 | 0.1418915 | 0.2385508 | 0.5031419 | NAA80 | 24142 |
| ENSG00000068001 | 539.994646 | -0.231437071 | 0.1471604 | 0.0183264 | 0.0976156 | HYAL2 | 8692 |
| ENSG00000114383 | 699.5176842 | 0.01562239 | 0.089616 | 0.8185717 | 0.9254567 | TUSC2 | 11334 |
| ENSG00000068028 | 1330.504803 | 0.897281962 | 0.0939757 | 8.24E-23 | 3.10E-20 | RASSF1 | 11186 |
| ENSG00000004838 | 36.68665651 | 0.017256995 | 0.1326846 | 0.6425842 | NA | ZMYND10 | 51364 |
| ENSG00000114388 | 369.4923855 | -0.025069783 | 0.1001573 | 0.7136641 | 0.870525 | NPRL2 | 10641 |
| ENSG00000114395 | 187.6177312 | -0.046945861 | 0.1194445 | 0.4771287 | 0.7163677 | CYB561D2 | 11068 |
| ENSG00000126062 | 844.3177652 | 0.127675743 | 0.0956467 | 0.0850578 | 0.2728275 | TMEM115 | 11070 |
| ENSG00000007402 | 20.47145394 | -0.0358977 | 0.1398195 | 0.2457169 | NA | CACNA2D2 | 9254 |
| ENSG00000088543 | 83.78510676 | 0.012866418 | 0.1240798 | 0.8104929 | 0.9218496 | C3orf18 | 51161 |
| ENSG00000114735 | 162.728977 | 0.023820369 | 0.1167994 | 0.7050865 | 0.8666859 | HEMK1 | 51409 |
| ENSG00000114737 | 52.61315456 | 0.039130209 | 0.134823 | 0.4048781 | 0.6614808 | CISH | 1154 |
| ENSG00000114738 | 1699.593759 | 0.046319232 | 0.073786 | 0.4573863 | 0.7017657 | MAPKAPK3 | 7867 |
| ENSG00000272543 | 14.78946917 | 0.014136176 | 0.13507 | 0.5926739 | NA | MIR4787 | 100616138 |
| ENSG00000088538 | 729.2497635 | 0.190795075 | 0.1068577 | 0.0195733 | 0.1019786 | DOCK3 | 1795 |
| ENSG00000145050 | 1463.705358 | -0.049677912 | 0.0741776 | 0.4263274 | 0.678557 | MANF | 7873 |
| ENSG00000179837 | 1620.756256 | -0.129520697 | 0.0783412 | 0.0504808 | 0.1935431 | NA | NA |
| ENSG00000145041 | 1400.73131 | -0.075976498 | 0.0787713 | 0.2429494 | 0.5086739 | DCAF1 | 9730 |
| ENSG00000164080 | 1233.462968 | -0.035574206 | 0.0781183 | 0.5792754 | 0.7918192 | RAD54L2 | 23132 |
| ENSG00000164081 | 844.3725692 | 0.181061996 | 0.1007488 | 0.0211962 | 0.1080247 | TEX264 | 51368 |
| ENSG00000114767 | 1633.582821 | 0.15784604 | 0.0823615 | 0.0214158 | 0.10887 | RRP9 | 9136 |
| ENSG00000041880 | 331.24219 | 0.44105059 | 0.1641766 | 0.0005219 | 0.0066989 | PARP3 | 10039 |
| ENSG00000090097 | 537.8401628 | 0.029816473 | 0.0929455 | 0.6737829 | 0.8501877 | PCBP4 | 57060 |
| ENSG00000114779 | 1053.885174 | 0.437561599 | 0.0953262 | 4.08E-07 | 1.58E-05 | ABHD14B | 84836 |
| ENSG00000248487 | 74.15290134 | 0.002524771 | 0.1248846 | 0.9619696 | 0.9844723 | ABHD14A | 25864 |
| ENSG00000243989 | 31.76461234 | -0.002445181 | 0.131567 | 0.9432893 | NA | ACY1 | 95 |
| ENSG00000162244 | 10739.25638 | -0.086427742 | 0.0555231 | 0.0886239 | 0.2802783 | RPL29 | 6159 |
| ENSG00000164086 | 1075.340873 | 0.716994953 | 0.0986075 | 2.29E-14 | 3.88E-12 | DUSP7 | 1849 |
| ENSG00000164087 | 594.8911772 | -0.059351407 | 0.0949119 | 0.3972958 | 0.6567942 | POC1A | 25886 |
| ENSG00000023330 | 2253.604465 | 0.371410354 | 0.0752596 | 9.20E-08 | 4.46E-06 | ALAS1 | 211 |
| ENSG00000247596 | 442.0375143 | 0.039437384 | 0.0976112 | 0.5717525 | 0.7864627 | TWF2 | 11344 |
| ENSG00000243224 | 8.534740927 | -0.001219201 | 0.1351198 | 0.9496196 | NA | TWF2-DT | 101929054 |
| ENSG00000164088 | 162.6853971 | 0.002340725 | 0.1164532 | 0.9707531 | 0.9883523 | PPM1M | 132160 |
| ENSG00000164091 | 3684.975991 | -0.094908727 | 0.062613 | 0.0878751 | 0.2788561 | WDR82 | 80335 |
| ENSG00000168237 | 236.9636274 | -0.037945123 | 0.1164582 | 0.5631729 | 0.780729 | GLYCTK | 132158 |
| ENSG00000114841 | 25.52478827 | -0.021842351 | 0.1347539 | 0.5109447 | NA | DNAH1 | 25981 |
| ENSG00000163930 | 3035.863738 | -0.082985709 | 0.0622993 | 0.1343641 | 0.3610553 | BAP1 | 8314 |
| ENSG00000010318 | 87.48707784 | 0.048643911 | 0.1332626 | 0.382358 | 0.6432624 | PHF7 | 51533 |
| ENSG00000010319 | 13.82928299 | 0.016199591 | 0.1354743 | 0.535189 | NA | SEMA3G | 56920 |
| ENSG00000010322 | 1867.563441 | 0.025017387 | 0.067017 | 0.6702049 | 0.8488197 | NISCH | 11188 |
| ENSG00000168268 | 1855.213009 | -0.030204362 | 0.0680305 | 0.6062467 | 0.8111761 | NT5DC2 | 64943 |
| ENSG00000168273 | 218.3180392 | 0.071060617 | 0.1264884 | 0.3100617 | 0.5796784 | UQCC5 | 440957 |
| ENSG00000163939 | 1860.163743 | -0.124918669 | 0.0757923 | 0.0511076 | 0.1949667 | PBRM1 | 55193 |
| ENSG00000163938 | 4167.944891 | -0.000354086 | 0.057408 | 0.9941646 | 0.9977151 | GNL3 | 26354 |
| ENSG00000212493 | 19.44026543 | 0.010575392 | 0.1336845 | 0.7282007 | NA | SNORD19 | 692089 |
| ENSG00000222345 | 17.55611164 | 0.009250369 | 0.1340613 | 0.7437077 | NA | SNORD19C | 109616991 |
| ENSG00000212452 | 11.62546645 | -0.006747775 | 0.1347776 | 0.7728042 | NA | SNORD69 | 692109 |
| ENSG00000016864 | 688.0407697 | -0.118444882 | 0.1025628 | 0.1171401 | 0.3339413 | GLT8D1 | 55830 |
| ENSG00000114902 | 1227.440753 | 0.026327048 | 0.0782796 | 0.6828909 | 0.8544725 | SPCS1 | 28972 |

| | | | | | | | |
|---|---|---|---|---|---|---|---|
| ENSG00000114904 | 909.5883695 | 0.040839439 | 0.0814227 | 0.5351986 | 0.7596179 | NEK4 | 6787 |
| ENSG00000248592 | 10.76820048 | -0.017097223 | 0.1363489 | 0.4451641 | NA | STIMATE-MI | 100526772 |
| ENSG00000213533 | 56.96309194 | -0.024254526 | 0.1296915 | 0.6087245 | 0.8123066 | STIMATE | 375346 |
| ENSG00000163935 | 1043.00152 | 0.022618346 | 0.0783505 | 0.7255721 | 0.8769175 | SFMBT1 | 51460 |
| ENSG00000163933 | 457.7508401 | 0.048605368 | 0.0986596 | 0.4900667 | 0.7267241 | RFT1 | 91869 |
| ENSG00000163932 | 679.3423457 | -0.034820799 | 0.087935 | 0.6107476 | 0.8135056 | PRKCD | 5580 |
| ENSG00000163931 | 5577.529256 | -0.513980435 | 0.0666749 | 1.06E-15 | 2.06E-13 | TKT | 7086 |
| ENSG00000162290 | 870.0272857 | 0.021132692 | 0.082443 | 0.7491084 | 0.8911635 | NA | NA |
| ENSG00000271976 | 51.89147559 | 0.042382873 | 0.1361018 | 0.369161 | 0.6316346 | NA | NA |
| ENSG00000113812 | 706.3805838 | -0.051313678 | 0.0880696 | 0.4444921 | 0.6928188 | ACTR8 | 93973 |
| ENSG00000113811 | 861.9629727 | -0.002383969 | 0.0834499 | 0.9703473 | 0.9882505 | SELENOK | 58515 |
| ENSG00000114251 | 9.259633633 | 0.01967109 | 0.1371031 | 0.3506243 | NA | WNT5A | 7474 |
| ENSG00000180376 | 289.6390964 | 0.404901137 | 0.1842927 | 0.0019936 | 0.01878 | CCDC66 | 285331 |
| ENSG00000163946 | 1597.611256 | -0.21210731 | 0.0824535 | 0.002559 | 0.0228353 | TASOR | 23272 |
| ENSG00000163947 | 293.3086181 | -0.085939927 | 0.1198966 | 0.2487652 | 0.5151429 | ARHGEF3 | 50650 |
| ENSG00000144730 | 164.3084179 | -0.058906698 | 0.1265364 | 0.3714379 | 0.6334891 | IL17RD | 54756 |
| ENSG00000163666 | 19.28239191 | -0.010411968 | 0.1340973 | 0.7121585 | NA | HESX1 | 8820 |
| ENSG00000157500 | 1988.87104 | -0.265934907 | 0.0765054 | 9.41E-05 | 0.0016599 | APPL1 | 26060 |
| ENSG00000239388 | 18.68727555 | -0.04585859 | 0.1446102 | 0.1259562 | NA | ASB14 | 142686 |
| ENSG00000174840 | 1001.317391 | 0.195644763 | 0.0945646 | 0.0104779 | 0.0655557 | PDE12 | 201626 |
| ENSG00000168374 | 2705.259665 | -0.003856283 | 0.061594 | 0.9433895 | 0.9782959 | ARF4 | 378 |
| ENSG00000174839 | 392.9610271 | -0.061310502 | 0.1081006 | 0.3901825 | 0.6505443 | DENND6A | 201627 |
| ENSG00000163681 | 1886.830632 | -0.078107882 | 0.0736715 | 0.2101486 | 0.4666816 | SLMAP | 7871 |
| ENSG00000136068 | 17404.62718 | 0.451098886 | 0.0600004 | 5.43E-15 | 1.01E-12 | FLNB | 2317 |
| ENSG00000244161 | 25.42902335 | 0.04538504 | 0.1434352 | 0.1732502 | NA | FLNB-AS1 | 105377105 |
| ENSG00000163686 | 173.2396479 | 0.09790168 | 0.1438497 | 0.1816193 | 0.4313736 | ABHD6 | 57406 |
| ENSG00000163684 | 860.6954173 | 0.024510106 | 0.0825308 | 0.7108179 | 0.869096 | RPP14 | 11102 |
| ENSG00000168297 | 265.632061 | 0.035524532 | 0.1093104 | 0.6002223 | 0.8069049 | PXK | 54899 |
| ENSG00000168291 | 1305.280176 | -0.338697933 | 0.089859 | 2.03E-05 | 0.0004628 | PDHB | 5162 |
| ENSG00000272182 | 31.2690211 | -0.008902266 | 0.1320449 | 0.799048 | NA | NA | NA |
| ENSG00000168301 | 123.5301194 | 0.004928157 | 0.1191101 | 0.934257 | 0.9749023 | KCTD6 | 200845 |
| ENSG00000163689 | 166.707982 | 0.073348564 | 0.1309133 | 0.2879941 | 0.5577946 | CFAP20DC | 200844 |
| ENSG00000144724 | 681.0359111 | 0.005842826 | 0.0855981 | 0.9311249 | 0.9731118 | PTPRG | 5793 |
| ENSG00000241472 | 187.5651847 | 0.178030533 | 0.1867891 | 0.0552631 | 0.206305 | PTPRG-AS1 | 100506994 |
| ENSG00000114405 | 498.9411625 | -0.114367378 | 0.1104861 | 0.1392151 | 0.3683114 | CEP15 | 57415 |
| ENSG00000163634 | 827.4626111 | -0.163220547 | 0.1047205 | 0.0388964 | 0.164619 | THOC7 | 80145 |
| ENSG00000163635 | 1032.557062 | -0.025280948 | 0.0776001 | 0.6918545 | 0.8594028 | ATXN7 | 6314 |
| ENSG00000239653 | 54.34334314 | 0.002384753 | 0.1282011 | 0.9589699 | 0.9831259 | PSMD6-AS2 | 100507062 |
| ENSG00000163636 | 1480.250766 | -0.254661142 | 0.0880853 | 0.0007194 | 0.0085926 | PSMD6 | 9861 |
| ENSG00000163638 | 342.7305892 | 0.017637379 | 0.1021675 | 0.7955926 | 0.9148766 | ADAMTS9 | 56999 |
| ENSG00000151276 | 410.1440731 | 0.034918416 | 0.0989071 | 0.6147683 | 0.8157779 | MAGI1 | 9223 |
| ENSG00000144741 | 385.6170084 | 0.032291822 | 0.0999849 | 0.6419668 | 0.8319997 | SLC25A26 | 115286 |
| ENSG00000163376 | 184.4492883 | 0.054738187 | 0.1239844 | 0.4079467 | 0.6640358 | KBTBD8 | 84541 |
| ENSG00000172340 | 774.8207053 | -0.111848381 | 0.095815 | 0.1255777 | 0.3478476 | SUCLG2 | 8801 |
| ENSG00000241316 | 27.27257411 | 0.044488797 | 0.1422429 | 0.207931 | NA | SUCLG2-DT | 101927111 |
| ENSG00000241506 | 21.83376857 | -0.043428905 | 0.1426036 | 0.1834383 | NA | PSMC1P1 | 151645 |
| ENSG00000163378 | 813.8072072 | -0.13611832 | 0.0965479 | 0.067322 | 0.2349246 | EOGT | 285203 |
| ENSG00000244513 | 23.84711636 | 0.005710278 | 0.1329185 | 0.8585122 | NA | NA | NA |
| ENSG00000144747 | 1208.710637 | -0.047514888 | 0.0817014 | 0.4679265 | 0.7103207 | TMF1 | 7110 |
| ENSG00000144744 | 1394.267868 | -0.303101001 | 0.0876179 | 7.91E-05 | 0.0014391 | UBA3 | 9039 |
| ENSG00000144746 | 853.8246339 | -0.165193476 | 0.1001848 | 0.0327419 | 0.1461994 | ARL6IP5 | 10550 |
| ENSG00000114541 | 86.94054782 | -0.08392862 | 0.1523272 | 0.1689695 | 0.4146497 | FRMD4B | 23150 |
| ENSG00000187098 | 843.2949728 | 0.45633056 | 0.1111716 | 3.43E-06 | 0.0001006 | MITF | 4286 |
| ENSG00000114861 | 78.12700006 | 0.012199726 | 0.1252292 | 0.8135992 | 0.9238507 | FOXP1 | 27086 |
| ENSG00000163602 | 845.6063403 | -0.045162813 | 0.084306 | 0.496869 | 0.7319873 | RYBP | 23429 |
| ENSG00000144736 | 654.7522159 | 0.107365786 | 0.1030419 | 0.1535002 | 0.3913077 | SHQ1 | 55164 |
| ENSG00000163605 | 2026.869752 | -0.113380326 | 0.070733 | 0.0620793 | 0.2224145 | PPP4R2 | 151987 |
| ENSG00000255423 | 25.02202189 | -0.044862041 | 0.142747 | 0.1908651 | NA | EBLN2 | 55096 |
| ENSG00000213300 | 23.36113186 | -0.011514158 | 0.1331586 | 0.7215899 | NA | NA | NA |
| ENSG00000244026 | 227.0506817 | 0.155865503 | 0.1658839 | 0.0756699 | 0.2529043 | FAM86DP | 692099 |
| ENSG00000242516 | 45.64846625 | -0.017175599 | 0.1325869 | 0.6410126 | 0.8315061 | LINC00960 | 401074 |
| ENSG00000227124 | 56.27594501 | 0.048968682 | 0.1383613 | 0.3134303 | 0.5822768 | ZNF717 | 100131827 |
| ENSG00000169855 | 1048.093394 | -0.006291734 | 0.0778542 | 0.9208578 | 0.9695818 | ROBO1 | 6091 |
| ENSG00000114480 | 926.0342299 | -0.046361127 | 0.0867212 | 0.4919218 | 0.7282321 | GBE1 | 2632 |
| ENSG00000206538 | 164.1256421 | 0.887643488 | 0.2230584 | 3.48E-06 | 0.0001015 | VGLL3 | 389136 |
| ENSG00000083937 | 901.5283792 | 0.143463196 | 0.0985758 | 0.0581148 | 0.2135512 | CHMP2B | 25978 |
| ENSG00000163320 | 1969.117492 | -0.091703587 | 0.0730779 | 0.1387799 | 0.3676153 | CGGBP1 | 8545 |
| ENSG00000229729 | 110.35591 | 0.001001091 | 0.1203035 | 0.9859879 | 0.9942441 | NA | NA |
| ENSG00000175105 | 307.1902462 | 0.041706241 | 0.1066621 | 0.5345967 | 0.7591377 | ZNF654 | 55279 |
| ENSG00000179021 | 425.2008759 | -0.03643231 | 0.0980645 | 0.5991327 | 0.80621 | C3orf38 | 285237 |

| | | | | | | | | |
|---|---|---|---|---|---|---|---|---|
| ENSG00000184500 | 544.732043 | -0.234849825 | 0.1268235 | 0.0111188 | 0.0685738 | PROS1 | | 5627 |
| ENSG00000169379 | 793.8279702 | 0.065795251 | 0.092425 | 0.3484461 | 0.6143697 | ARL13B | | 200894 |
| ENSG00000178700 | 137.4729828 | 0.456922693 | 0.2747563 | 0.0045339 | 0.0351901 | DHFR2 | | 200895 |
| ENSG00000178694 | 169.1727145 | 0.038706849 | 0.1192974 | 0.5475505 | 0.7691123 | NSUN3 | | 63899 |
| ENSG00000178660 | 8.995982679 | -0.001165735 | 0.1351858 | 0.9507832 | NA | NA | NA | |
| ENSG00000113966 | 110.1668748 | -0.053965565 | 0.131293 | 0.370586 | 0.6328676 | ARL6 | | 84100 |
| ENSG00000233280 | 419.5449096 | -0.130312945 | 0.1216369 | 0.1072668 | 0.31649 | NA | NA | |
| ENSG00000080200 | 618.8834956 | -0.003014347 | 0.089267 | 0.961668 | 0.9843697 | CRYBG3 | | 131544 |
| ENSG00000170854 | 932.7930555 | -0.053045588 | 0.0830159 | 0.4222135 | 0.6742808 | RIOX2 | | 84864 |
| ENSG00000080822 | 715.2715673 | 0.020423679 | 0.0860162 | 0.7611977 | 0.8975925 | CLDND1 | | 56650 |
| ENSG00000080819 | 845.6068794 | -0.021350362 | 0.0822027 | 0.7446921 | 0.889079 | CPOX | | 1371 |
| ENSG00000064225 | 64.3592409 | -0.000597476 | 0.1265477 | 0.9883458 | 0.9955047 | ST3GAL6 | | 10402 |
| ENSG00000057019 | 20340.6274 | -0.192462426 | 0.0603842 | 0.0004702 | 0.0061026 | DCBLD2 | | 131566 |
| ENSG00000206712 | 30.29551846 | -0.001425794 | 0.1314073 | 0.9669921 | NA | RNU6-26P | | 100873741 |
| ENSG00000239462 | 18.49178692 | 0.024548007 | 0.1363992 | 0.4131688 | NA | NA | NA | |
| ENSG00000240476 | 306.6661566 | 0.374459467 | 0.1723256 | 0.0023896 | 0.0216717 | LINC00973 | | 105374003 |
| ENSG00000184220 | 946.4736432 | 0.083159348 | 0.085138 | 0.2235298 | 0.4837969 | CMSS1 | | 84319 |
| ENSG00000261533 | 12.01569725 | 0.01646028 | 0.1362211 | 0.463564 | NA | NA | NA | |
| ENSG00000036054 | 777.2574958 | 0.019625116 | 0.0838008 | 0.7679976 | 0.90052 | TBC1D23 | | 55773 |
| ENSG00000114021 | 549.2559113 | 0.004581244 | 0.0927186 | 0.9608472 | 0.983972 | NIT2 | | 56954 |
| ENSG00000154174 | 1927.008516 | 0.017047498 | 0.0683596 | 0.7759754 | 0.9056923 | TOMM70 | | 9868 |
| ENSG00000206535 | 33.09211859 | -0.002289215 | 0.1320256 | 0.9443196 | NA | LNP1 | | 348801 |
| ENSG00000181458 | 16.58506906 | -0.033458867 | 0.1397189 | 0.2278979 | NA | TMEM45A | | 55076 |
| ENSG00000114354 | 2014.522874 | -0.055625738 | 0.0679113 | 0.3447787 | 0.610988 | TFG | | 10342 |
| ENSG00000138468 | 188.4375165 | -0.042845158 | 0.1202518 | 0.5065162 | 0.7390227 | SENP7 | | 57337 |
| ENSG00000174173 | 598.841478 | 0.000230004 | 0.0898295 | 0.9984867 | 0.9989998 | TRMT10C | | 54931 |
| ENSG00000081154 | 3163.464891 | -0.040924502 | 0.0604292 | 0.4492662 | 0.6962034 | PCNP | | 57092 |
| ENSG00000242299 | 87.71549974 | -0.109573112 | 0.1757794 | 0.0931042 | 0.2889346 | RPS18P5 | | 100131863 |
| ENSG00000066422 | 1449.078634 | 0.26748653 | 0.1017426 | 0.0014174 | 0.0147044 | ZBTB11 | | 27107 |
| ENSG00000256628 | 96.54701975 | 0.066740575 | 0.1395087 | 0.2682464 | 0.5361059 | ZBTB11-AS1 | | 100009676 |
| ENSG00000114391 | 8812.285208 | -0.188640389 | 0.0606816 | 0.0006036 | 0.0074291 | RPL24 | | 6152 |
| ENSG00000182504 | 543.5703276 | 0.131741884 | 0.1127594 | 0.0971205 | 0.2964369 | CEP97 | | 79598 |
| ENSG00000144815 | 852.4420633 | -0.335416927 | 0.1069611 | 0.000203 | 0.0030954 | NXPE3 | | 91775 |
| ENSG00000144802 | 2221.666921 | 0.03449036 | 0.067063 | 0.5569278 | 0.7762169 | NFKBIZ | | 64332 |
| ENSG00000170017 | 823.0406965 | 0.018387769 | 0.0840981 | 0.7972755 | 0.9158202 | ALCAM | | 214 |
| ENSG00000114423 | 109.4588391 | -0.044716089 | 0.1283031 | 0.445967 | 0.6940447 | CBLB | | 868 |
| ENSG00000243701 | 116.1092497 | 0.066972362 | 0.1377222 | 0.2802853 | 0.5489625 | DUBR | | 344595 |
| ENSG00000114439 | 1569.905899 | -0.091402859 | 0.0774292 | 0.1567812 | 0.3957987 | BBX | | 56987 |
| ENSG00000196776 | 762.9078973 | 0.060819252 | 0.0901887 | 0.3804081 | 0.6413895 | CD47 | | 961 |
| ENSG00000114446 | 385.6116863 | 0.064827515 | 0.1072982 | 0.3689502 | 0.6315515 | IFT57 | | 55081 |
| ENSG00000144821 | 11.99856008 | 0.02579157 | 0.1382466 | 0.2682112 | NA | MYH15 | | 22989 |
| ENSG00000241634 | 38.55359799 | 0.005215958 | 0.1301683 | 0.8981841 | NA | RPL13P8 | | 100270928 |
| ENSG00000163507 | 1886.800969 | -0.03820239 | 0.0676803 | 0.5150771 | 0.7449994 | CIP2A | | 57650 |
| ENSG00000198919 | 876.5185759 | -0.018804964 | 0.0816687 | 0.77337 | 0.9040968 | DZIP3 | | 9666 |
| ENSG00000177707 | 587.5714835 | -0.15202538 | 0.113311 | 0.0607735 | 0.2199285 | NECTIN3 | | 25945 |
| ENSG00000177494 | 91.87636855 | 0.038260969 | 0.1283006 | 0.4959399 | 0.7311922 | ZBED2 | | 79413 |
| ENSG00000240891 | 122.673828 | 0.669498703 | 0.2849323 | 0.0008044 | 0.0093773 | PLCXD2 | | 257068 |
| ENSG00000144824 | 1060.228393 | 0.573051394 | 0.0953618 | 1.38E-10 | 1.14E-08 | PHLDB2 | | 90102 |
| ENSG00000144827 | 811.5098252 | 0.013358454 | 0.0825914 | 0.8400447 | 0.9344407 | ABHD10 | | 55347 |
| ENSG00000114529 | 272.9127247 | 0.21671942 | 0.1728055 | 0.0311601 | 0.1416821 | C3orf52 | | 79669 |
| ENSG00000261488 | 17.15801112 | 0.010865659 | 0.1341815 | 0.703483 | NA | TBILA | | 112806053 |
| ENSG00000144848 | 1234.895672 | 0.003694714 | 0.0751609 | 0.9538811 | 0.9817856 | ATG3 | | 64422 |
| ENSG00000138459 | 366.0610937 | -0.010337235 | 0.1004711 | 0.8786366 | 0.9511913 | SLC35A5 | | 55032 |
| ENSG00000163607 | 213.8892977 | -0.042541792 | 0.1145483 | 0.5262423 | 0.7534979 | GTPBP8 | | 29083 |
| ENSG00000163608 | 898.4468337 | -0.015495042 | 0.0810461 | 0.8115914 | 0.9223778 | NEPRO | | 25871 |
| ENSG00000206530 | 284.2411309 | 0.034248828 | 0.1078723 | 0.617783 | 0.8169731 | CFAP44 | | 55779 |
| ENSG00000163611 | 287.0630609 | 0.057820453 | 0.1119304 | 0.4129843 | 0.6669527 | SPICE1 | | 152185 |
| ENSG00000176542 | 555.5294053 | -0.266331635 | 0.1258698 | 0.0050352 | 0.0381927 | USF3 | | 205717 |
| ENSG00000121579 | 6088.003155 | 0.074478653 | 0.0548011 | 0.1385045 | 0.3673017 | NAA50 | | 80218 |
| ENSG00000114573 | 1527.306838 | 0.002392425 | 0.06992 | 0.9689911 | 0.9880224 | ATP6V1A | | 523 |
| ENSG00000178075 | 234.9008667 | -0.024594341 | 0.1110706 | 0.7043947 | 0.8661643 | GRAMD1C | | 54762 |
| ENSG00000241889 | 11.50896085 | -0.014873902 | 0.1357931 | 0.5220589 | NA | NA | NA | |
| ENSG00000184307 | 483.8435467 | 0.181722775 | 0.1272421 | 0.0382431 | 0.1627981 | ZDHHC23 | | 254887 |
| ENSG00000163617 | 84.60549667 | 0.051798397 | 0.1362302 | 0.3362236 | 0.6038481 | CCDC191 | | 57577 |
| ENSG00000151576 | 1563.208187 | 0.273350068 | 0.0828786 | 0.0001699 | 0.0026634 | QTRT2 | | 79691 |
| ENSG00000259976 | 10.15271718 | 0.023434395 | 0.1380276 | 0.2608382 | NA | NA | NA | |
| ENSG00000181722 | 13.90461925 | -0.011122072 | 0.134728 | 0.6668963 | NA | ZBTB20 | | 26137 |
| ENSG00000198406 | 337.3351355 | -0.063593721 | 0.1094442 | 0.3755089 | 0.637176 | NA | NA | |
| ENSG00000144847 | 49.02121712 | -0.025342226 | 0.1315711 | 0.5646254 | 0.7820587 | IGSF11 | | 152404 |
| ENSG00000114638 | 11.93377682 | -0.010537003 | 0.1350161 | 0.661874 | NA | UPK1B | | 7348 |

| | | | | | | | |
|---|---|---|---|---|---|---|---|
| ENSG00000121578 | 832.6028779 | -0.001784542 | 0.0836916 | 0.9609073 | 0.983972 | B4GALT4 | 8702 |
| ENSG00000031081 | 975.4873212 | 0.001710875 | 0.1129692 | 0.9798756 | 0.9924501 | ARHGAP31 | 57514 |
| ENSG00000241155 | 10.52552465 | -0.009023779 | 0.1352075 | 0.6840294 | NA | ARHGAP31- | 100874246 |
| ENSG00000176142 | 552.0222466 | -0.059820899 | 0.0955101 | 0.3944766 | 0.6549057 | TMEM39A | 55254 |
| ENSG00000163389 | 601.6001385 | -0.084561245 | 0.1021306 | 0.2461832 | 0.5119576 | POGLUT1 | 56983 |
| ENSG00000113845 | 966.5212027 | 0.154982264 | 0.0929888 | 0.0358903 | 0.1556755 | TIMMDC1 | 51300 |
| ENSG00000144843 | 22.39184354 | 0.001168193 | 0.1326727 | 0.9728827 | NA | ADPRH | 141 |
| ENSG00000138495 | 352.1106565 | -0.000449583 | 0.099606 | 0.9938807 | 0.9977151 | COX17 | 10063 |
| ENSG00000183833 | 32.0848993 | 0.033347129 | 0.136462 | 0.3854622 | NA | CFAP91 | 89876 |
| ENSG00000082701 | 1921.583656 | 0.00570095 | 0.0698262 | 0.9250963 | 0.9710568 | GSK3B | 2932 |
| ENSG00000242622 | 25.37777976 | 0.017480231 | 0.133991 | 0.5923126 | NA | NA | NA |
| ENSG00000175697 | 32.40193927 | -0.008299728 | 0.1312954 | 0.8234978 | NA | GPR156 | 165829 |
| ENSG00000163428 | 2418.013675 | 0.111206569 | 0.0704277 | 0.0673328 | 0.2349246 | LRRC58 | 116064 |
| ENSG00000163430 | 22.32588696 | 0.072783843 | 0.161476 | 0.0406753 | NA | FSTL1 | 11167 |
| ENSG00000065518 | 868.3071174 | -0.050820658 | 0.0864942 | 0.4526083 | 0.6987417 | NDUFB4 | 4710 |
| ENSG00000144840 | 335.9102644 | -0.008713056 | 0.1028479 | 0.8966641 | 0.9588002 | RABL3 | 285282 |
| ENSG00000153767 | 301.2450448 | 0.032781819 | 0.1055401 | 0.6346833 | 0.8264113 | GTF2E1 | 2960 |
| ENSG00000051341 | 1019.864091 | 0.148690429 | 0.0916006 | 0.0416323 | 0.1717792 | POLQ | 10721 |
| ENSG00000173230 | 1610.762743 | -0.015179844 | 0.0706989 | 0.8011032 | 0.9169162 | GOLGB1 | 2804 |
| ENSG00000173226 | 780.6339819 | 0.354063585 | 0.1098239 | 0.0001397 | 0.0022762 | IQCB1 | 9657 |
| ENSG00000145088 | 77.43562281 | 0.069017902 | 0.1447931 | 0.2242944 | 0.4849126 | EAF2 | 55840 |
| ENSG00000163406 | 33.13680263 | 0.008017569 | 0.1311482 | 0.8341074 | NA | SLC15A2 | 6565 |
| ENSG00000160124 | 279.5299519 | -0.024878462 | 0.108723 | 0.6698483 | 0.8486358 | MIX23 | 131076 |
| ENSG00000114023 | 384.8710125 | -0.263051163 | 0.1516366 | 0.0107958 | 0.0669353 | FAM162A | 26355 |
| ENSG00000196981 | 70.82832458 | -0.037919175 | 0.1313834 | 0.4621598 | 0.7066085 | WDR5B | 54554 |
| ENSG00000272758 | 24.52740277 | -0.044385904 | 0.1427029 | 0.1884671 | NA | WDR5B-DT | 102723582 |
| ENSG00000114030 | 1726.645192 | -0.055679945 | 0.0760924 | 0.379816 | 0.6409076 | KPNA1 | 3836 |
| ENSG00000138496 | 175.9599596 | -0.004742494 | 0.1159015 | 0.9372993 | 0.9757234 | PARP9 | 83666 |
| ENSG00000163840 | 609.2456304 | -0.043527798 | 0.0976096 | 0.5323787 | 0.7577331 | DTX3L | 151636 |
| ENSG00000173193 | 969.390803 | -0.170121724 | 0.0959681 | 0.0248348 | 0.1202483 | PARP14 | 54625 |
| ENSG00000169087 | 146.6732995 | 0.213963735 | 0.2641919 | 0.0328984 | 0.1464505 | HSPBAP1 | 79663 |
| ENSG00000138463 | 250.416805 | 0.115992181 | 0.1389143 | 0.1444088 | 0.376779 | SLC49A4 | 84925 |
| ENSG00000273033 | 51.03124019 | 0.002583759 | 0.1285874 | 0.9546489 | 0.9819702 | LINC02035 | 100129550 |
| ENSG00000065485 | 631.0093093 | -0.195783327 | 0.1215358 | 0.0249571 | 0.1206138 | PDIA5 | 10954 |
| ENSG00000121542 | 197.535372 | 0.094954813 | 0.1367673 | 0.2003208 | 0.4540225 | SEC22A | 26984 |
| ENSG00000173175 | 10.23039005 | -0.005701598 | 0.13508 | 0.78854 | NA | ADCY5 | 111 |
| ENSG00000206527 | 2387.951444 | -0.144729965 | 0.0712298 | 0.0190022 | 0.1000744 | HACD2 | 201562 |
| ENSG00000239523 | 15.60375103 | 0.03750821 | 0.1413218 | 0.1780219 | NA | MYLK-AS1 | 100506826 |
| ENSG00000175455 | 921.3613417 | 0.027472424 | 0.0814298 | 0.6758135 | 0.8513996 | CCDC14 | 64770 |
| ENSG00000160145 | 14.05191392 | 0.014781689 | 0.1353227 | 0.5648743 | NA | KALRN | 8997 |
| ENSG00000114491 | 858.3303595 | 0.043208591 | 0.0841224 | 0.5111588 | 0.7421105 | UMPS | 7372 |
| ENSG00000272947 | 28.7295507 | 0.010956209 | 0.132159 | 0.7607216 | NA | NA | NA |
| ENSG00000082781 | 3470.429218 | 0.016489238 | 0.0626742 | 0.7674951 | 0.9002018 | ITGB5 | 3693 |
| ENSG00000173706 | 274.7006499 | 0.216345304 | 0.1774841 | 0.0320778 | 0.1443934 | HEG1 | 57493 |
| ENSG00000163848 | 823.13078 | -0.021394805 | 0.0837782 | 0.7462159 | 0.8895345 | ZNF148 | 7707 |
| ENSG00000114520 | 582.0336019 | -0.272269764 | 0.1228159 | 0.0038482 | 0.0309156 | SNX4 | 8723 |
| ENSG00000144909 | 665.8763139 | 0.020892304 | 0.0878693 | 0.757698 | 0.8961404 | OSBPL11 | 114885 |
| ENSG00000171084 | 30.79758165 | 0.039180787 | 0.1392493 | 0.2890497 | NA | NA | NA |
| ENSG00000114544 | 585.5598761 | 0.009966936 | 0.0907692 | 0.8842228 | 0.953345 | SLC41A3 | 54946 |
| ENSG00000070476 | 576.909245 | 0.059721447 | 0.0961979 | 0.4105385 | 0.6655795 | ZXDC | 79364 |
| ENSG00000197763 | 180.3668251 | 0.277789235 | 0.2288075 | 0.0183444 | 0.097667 | TXNRD3 | 114112 |
| ENSG00000159685 | 346.8860263 | 0.022700759 | 0.1024946 | 0.7396608 | 0.8859699 | CHCHD6 | 84303 |
| ENSG00000114554 | 3011.104997 | -0.045941648 | 0.0617931 | 0.4034938 | 0.6605762 | PLXNA1 | 5361 |
| ENSG00000163870 | 552.1914091 | 0.381567668 | 0.1346008 | 0.0004219 | 0.0056382 | TPRA1 | 131601 |
| ENSG00000073111 | 4327.326552 | 0.05824288 | 0.0619447 | 0.3040065 | 0.5728743 | MCM2 | 4171 |
| ENSG00000114631 | 156.9545719 | 0.017431841 | 0.115808 | 0.782254 | 0.9095461 | PODXL2 | 50512 |
| ENSG00000114626 | 232.9065694 | -0.068376063 | 0.1229668 | 0.3297857 | 0.5978967 | ABTB1 | 80325 |
| ENSG00000074416 | 837.6692917 | 0.154064926 | 0.1039986 | 0.0490651 | 0.1901557 | MGLL | 11343 |
| ENSG00000058262 | 5861.189963 | -0.167509202 | 0.0575729 | 0.0014378 | 0.0148893 | SEC61A1 | 29927 |
| ENSG00000175792 | 1994.466836 | -0.066756122 | 0.0717701 | 0.2747073 | 0.5431373 | RUVBL1 | 8607 |
| ENSG00000132394 | 215.0397687 | 0.035254253 | 0.1136549 | 0.5984659 | 0.8058763 | EEFSEC | 60678 |
| ENSG00000179348 | 178.2938321 | 0.566155668 | 0.2327995 | 0.000767 | 0.0090339 | GATA2 | 2624 |
| ENSG00000244300 | 102.3146667 | -0.014614773 | 0.1217516 | 0.7948091 | 0.9144629 | NA | NA |
| ENSG00000163902 | 4972.25036 | -0.045960239 | 0.056328 | 0.3690698 | 0.6315712 | RPN1 | 6184 |
| ENSG00000075785 | 3582.443471 | 0.087317707 | 0.0606255 | 0.1084606 | 0.3186439 | RAB7A | 7879 |
| ENSG00000231305 | 9.124559854 | 0.028001654 | 0.139257 | 0.1881728 | NA | NA | NA |
| ENSG00000177646 | 713.5660698 | -0.035669811 | 0.0867365 | 0.600137 | 0.8068831 | ACAD9 | 28976 |
| ENSG00000172780 | 74.21491252 | -0.031980225 | 0.1290949 | 0.5375866 | 0.7615266 | RAB43 | 339122 |
| ENSG00000240682 | 280.5054691 | 0.000648583 | 0.1062506 | 0.9934326 | 0.9975317 | ISY1 | 57461 |
| ENSG00000169714 | 7042.546981 | -0.023083923 | 0.0521445 | 0.5937284 | 0.8026087 | CNBP | 7555 |

| | | | | | | | | |
|---|---|---|---|---|---|---|---|---|
| ENSG00000181789 | 1391.283806 | -0.002415267 | 0.0724108 | 0.9677003 | 0.9872153 | COPG1 | | 22820 |
| ENSG00000183624 | 1642.202102 | -0.118463841 | 0.0752586 | 0.0629008 | 0.2245583 | HMCES | | 56941 |
| ENSG00000184897 | 1705.948306 | -0.04399687 | 0.0713629 | 0.4692141 | 0.7108806 | H1-10 | | 8971 |
| ENSG00000206417 | 31.77495551 | 0.018459273 | 0.1332621 | 0.6087729 | NA | NA | NA | |
| ENSG00000251474 | 186.3618195 | -0.016182967 | 0.113381 | 0.7991638 | 0.9162772 | NA | NA | |
| ENSG00000129071 | 778.567116 | 0.02474728 | 0.0836028 | 0.7096591 | 0.8682712 | MBD4 | | 8930 |
| ENSG00000163913 | 503.6151964 | 0.051602172 | 0.098107 | 0.4648001 | 0.7086048 | IFT122 | | 55764 |
| ENSG00000004399 | 420.3952471 | 0.003867754 | 0.0960036 | 0.9560134 | 0.9825402 | PLXND1 | | 23129 |
| ENSG00000172765 | 187.2134475 | -0.091025173 | 0.1371381 | 0.2100989 | 0.4666816 | TMCC1 | | 23023 |
| ENSG00000203644 | 74.54555512 | -0.015116638 | 0.1257199 | 0.7661203 | 0.8994294 | NA | NA | |
| ENSG00000271270 | 57.46183517 | 0.074270808 | 0.1540938 | 0.1428172 | 0.3739628 | TMCC1-DT | | 100507032 |
| ENSG00000253540 | 24.07104266 | 0.078279371 | 0.1666705 | 0.0273841 | NA | NA | NA | |
| ENSG00000196455 | 847.0033117 | 0.035365711 | 0.0842148 | 0.5968452 | 0.8046376 | PIK3R4 | | 30849 |
| ENSG00000017260 | 1936.680803 | -0.004503634 | 0.0665282 | 0.9370793 | 0.9756936 | ATP2C1 | | 27032 |
| ENSG00000034533 | 114.2435471 | -0.025179983 | 0.1225536 | 0.6625932 | 0.8438537 | ASTE1 | | 28990 |
| ENSG00000114670 | 97.50937722 | 0.033666956 | 0.1261963 | 0.5534243 | 0.7742395 | NEK11 | | 79858 |
| ENSG00000246082 | 13.08939113 | 0.021748561 | 0.1367614 | 0.3914516 | NA | NUDT16L2P | | 152195 |
| ENSG00000198585 | 522.3300573 | -0.100630195 | 0.1066978 | 0.1808819 | 0.4301468 | NUDT16 | | 131870 |
| ENSG00000261167 | 29.98251764 | -0.025071788 | 0.135189 | 0.4659246 | NA | NA | NA | |
| ENSG00000114686 | 2190.455493 | -0.187224197 | 0.0786751 | 0.005396 | 0.0401582 | MRPL3 | | 11222 |
| ENSG00000138246 | 1771.09888 | -0.071637497 | 0.0726737 | 0.2454275 | 0.5110917 | DNAJC13 | | 23317 |
| ENSG00000240303 | 45.46341457 | -0.090186061 | 0.1701714 | 0.0711615 | 0.243052 | ACAD11 | | 84129 |
| ENSG00000113971 | 368.7867853 | -0.063453361 | 0.1098735 | 0.3761152 | 0.6379267 | NPHP3 | | 27031 |
| ENSG00000081307 | 828.0017947 | -0.165294367 | 0.1042964 | 0.0364062 | 0.1572129 | UBA5 | | 79876 |
| ENSG00000091527 | 4753.32728 | -0.139245601 | 0.0628019 | 0.0128379 | 0.0758519 | CDV3 | | 55573 |
| ENSG00000163781 | 2807.786893 | -0.102415748 | 0.064336 | 0.0714695 | 0.2435341 | TOPBP1 | | 11073 |
| ENSG00000144867 | 1594.386235 | 0.102481128 | 0.0787652 | 0.1165095 | 0.3330758 | SRPRB | | 58477 |
| ENSG00000154917 | 677.495179 | -0.04990629 | 0.0905335 | 0.4683155 | 0.7104407 | RAB6B | | 51560 |
| ENSG00000163785 | 1062.496104 | -0.105378849 | 0.0863014 | 0.1259129 | 0.3482806 | RYK | | 6259 |
| ENSG00000114019 | 2859.528879 | 0.085157885 | 0.0633757 | 0.1294638 | 0.353901 | AMOTL2 | | 51421 |
| ENSG00000129055 | 844.4923389 | 0.574434974 | 0.1072409 | 6.04E-09 | 3.82E-07 | ANAPC13 | | 25847 |
| ENSG00000182923 | 352.4888318 | 0.009835663 | 0.1005519 | 0.8851372 | 0.9535774 | CEP63 | | 80254 |
| ENSG00000073711 | 438.9480979 | -0.185392631 | 0.1369489 | 0.0397212 | 0.1669409 | PPP2R3A | | 5523 |
| ENSG00000174579 | 528.978949 | -0.063857064 | 0.099709 | 0.3707272 | 0.6330163 | MSL2 | | 55167 |
| ENSG00000114054 | 675.9103807 | -0.303456933 | 0.1172574 | 0.001187 | 0.0128385 | PCCB | | 5096 |
| ENSG00000118007 | 1381.197588 | -0.062723357 | 0.0810208 | 0.3401902 | 0.6075159 | STAG1 | | 10274 |
| ENSG00000273486 | 23.83435021 | 0.028569524 | 0.1365112 | 0.3962149 | NA | NA | NA | |
| ENSG00000239213 | 51.98696775 | 0.003567065 | 0.129672 | 0.9331416 | 0.9743454 | NA | NA | |
| ENSG00000158092 | 427.8720704 | -0.018314022 | 0.0977557 | 0.7886289 | 0.9116361 | NCK1 | | 4690 |
| ENSG00000174564 | 15.08373526 | 0.00326614 | 0.1340057 | 0.9045139 | NA | IL20RB | | 53833 |
| ENSG00000158163 | 131.3406806 | 0.102161238 | 0.154349 | 0.1522003 | 0.3895241 | DZIP1L | | 199221 |
| ENSG00000138231 | 758.6503565 | 0.205429856 | 0.1129864 | 0.015821 | 0.0875024 | DBR1 | | 51163 |
| ENSG00000114098 | 655.3887748 | -0.150634869 | 0.1090995 | 0.0589094 | 0.2154423 | ARMC8 | | 25852 |
| ENSG00000158186 | 78.6556811 | 0.560513529 | 0.3919229 | 0.0049071 | 0.0375378 | MRAS | | 22808 |
| ENSG00000114107 | 379.9709515 | -0.410141305 | 0.1581556 | 0.0007373 | 0.0087796 | CEP70 | | 80321 |
| ENSG00000158234 | 196.8904284 | -0.152239978 | 0.1705025 | 0.0780524 | 0.2576966 | FAIM | | 55179 |
| ENSG00000051382 | 912.6343705 | -0.159768967 | 0.0951437 | 0.0330634 | 0.146682 | PIK3CB | | 5291 |
| ENSG00000183770 | 81.20017138 | 0.115447122 | 0.1818706 | 0.0833539 | 0.2698513 | FOXL2 | | 668 |
| ENSG00000206262 | 62.27950349 | 0.093360005 | 0.1667451 | 0.1035116 | 0.308606 | FOXL2NB | | 401089 |
| ENSG00000175110 | 583.0274786 | -0.107389424 | 0.1041727 | 0.1542168 | 0.3924711 | MRPS22 | | 56945 |
| ENSG00000184432 | 2790.97066 | -0.240243298 | 0.0713617 | 0.0001682 | 0.0026518 | COPB2 | | 9276 |
| ENSG00000248932 | 10.12610534 | 0.019271201 | 0.1367686 | 0.3918882 | NA | COPB2-DT | | 100507291 |
| ENSG00000114120 | 1619.231746 | -0.0504327 | 0.0734351 | 0.4158167 | 0.6697358 | SLC25A36 | | 55186 |
| ENSG00000155893 | 115.2077366 | -0.058428924 | 0.132918 | 0.3385249 | 0.6060245 | PXYLP1 | | 92370 |
| ENSG00000177311 | 814.3934868 | -0.128742488 | 0.0964909 | 0.0822761 | 0.266996 | ZBTB38 | | 253461 |
| ENSG00000155903 | 414.1304791 | 0.036645595 | 0.0987394 | 0.5981582 | 0.805834 | RASA2 | | 5922 |
| ENSG00000114125 | 885.5874781 | -0.021034588 | 0.0847062 | 0.7511677 | 0.8925687 | RNF7 | | 9616 |
| ENSG00000069849 | 3279.900052 | -0.023132025 | 0.0613346 | 0.6722795 | 0.8499086 | ATP1B3 | | 483 |
| ENSG00000114126 | 528.8612938 | -0.073894237 | 0.0990426 | 0.3038672 | 0.5728743 | TFDP2 | | 7029 |
| ENSG00000272565 | 244.106571 | -0.127778732 | 0.1439075 | 0.1178549 | 0.3345134 | NA | NA | |
| ENSG00000175066 | 427.3648457 | -0.087039187 | 0.1084763 | 0.2353898 | 0.4991085 | GK5 | | 256356 |
| ENSG00000114127 | 808.1236938 | -0.07444851 | 0.0879901 | 0.2793162 | 0.5477074 | XRN1 | | 54464 |
| ENSG00000175054 | 1299.417172 | 0.319399472 | 0.0918403 | 6.79E-05 | 0.0012698 | ATR | | 545 |
| ENSG00000120756 | 1236.72379 | -0.279689732 | 0.0975516 | 0.0006484 | 0.0078893 | PLS1 | | 5357 |
| ENSG00000144935 | 417.0025152 | 0.05282685 | 0.1013743 | 0.4558343 | 0.7007253 | TRPC1 | | 7220 |
| ENSG00000163710 | 109.631391 | 0.159661845 | 0.2158231 | 0.0554393 | 0.2066986 | PCOLCE2 | | 26577 |
| ENSG00000188582 | 10.26795327 | 0.025168368 | 0.1382523 | 0.2587408 | NA | PAQR9 | | 344838 |
| ENSG00000163714 | 3428.94749 | -0.204119367 | 0.0646519 | 0.0004654 | 0.0060536 | U2SURP | | 23350 |
| ENSG00000268129 | 8.673019826 | -0.004844998 | 0.1353133 | 0.8038661 | NA | LOC10028931 | | 100289361 |
| ENSG00000175040 | 141.6873653 | 0.055984516 | 0.128107 | 0.3810961 | 0.641901 | CHST2 | | 9435 |

| | | | | | | | | |
|---|---|---|---|---|---|---|---|---|
| ENSG00000181744 | 316.0971041 | -0.270684738 | 0.1731467 | 0.0130822 | 0.0768289 | DIPK2A | | 205428 |
| ENSG00000261051 | 10.48737908 | 0.021345278 | 0.1372227 | 0.3432962 | NA | NA | NA | |
| ENSG00000152952 | 1879.552677 | 0.029796206 | 0.0682006 | 0.615889 | 0.8158539 | PLOD2 | | 5352 |
| ENSG00000114698 | 28.55398613 | 0.032698399 | 0.1369176 | 0.3704184 | NA | PLSCR4 | | 57088 |
| ENSG00000188313 | 582.6790956 | -0.022722447 | 0.0927389 | 0.7389694 | 0.8857761 | PLSCR1 | | 5359 |
| ENSG00000163754 | 1053.595542 | 0.050867151 | 0.0797949 | 0.4293273 | 0.6803653 | GYG1 | | 2992 |
| ENSG00000071794 | 1710.035446 | -0.060237776 | 0.0702516 | 0.3176169 | 0.5862822 | HLTF | | 6596 |
| ENSG00000163755 | 604.0244874 | -0.026334671 | 0.0902625 | 0.6975377 | 0.8620332 | HPS3 | | 84343 |
| ENSG00000163762 | 1594.908926 | -0.937454821 | 0.0831592 | 1.06E-30 | 7.71E-28 | TM4SF18 | | 116441 |
| ENSG00000169908 | 10967.12946 | -0.107017139 | 0.1118906 | 0.1513842 | 0.3879595 | TM4SF1 | | 4071 |
| ENSG00000240541 | 16.28824793 | 0.053580682 | 0.1493323 | 0.0644978 | NA | TM4SF1-AS | | 100874091 |
| ENSG00000018408 | 6633.551037 | -0.282938479 | 0.0595407 | 3.59E-07 | 1.40E-05 | WWTR1 | | 25937 |
| ENSG00000241313 | 24.17334604 | 0.04273937 | 0.1418786 | 0.2079579 | NA | WWTR1-AS | | 100128025 |
| ENSG00000114744 | 759.9886863 | -0.092996147 | 0.0930913 | 0.1918749 | 0.4431181 | COMMD2 | | 51122 |
| ENSG00000082996 | 658.8240291 | -0.012910222 | 0.0874952 | 0.8469699 | 0.9372352 | RNF13 | | 11342 |
| ENSG00000070087 | 1879.192752 | -0.064541527 | 0.0700854 | 0.2833552 | 0.5528432 | PFN2 | | 5217 |
| ENSG00000268175 | 19.04317411 | 0.012539637 | 0.1341792 | 0.6693113 | NA | NA | NA | |
| ENSG00000224831 | 43.07677195 | -0.05705465 | 0.1453122 | 0.1982955 | 0.4513782 | TMEM183BF | | 653659 |
| ENSG00000196428 | 808.8347057 | 0.339279559 | 0.1117384 | 0.0002761 | 0.0040208 | TSC22D2 | | 9819 |
| ENSG00000120742 | 1944.536801 | 0.052558049 | 0.0709267 | 0.3884688 | 0.6487996 | SERP1 | | 27230 |
| ENSG00000144895 | 1406.700906 | -0.191505842 | 0.0843 | 0.0067723 | 0.0476356 | EIF2A | | 83939 |
| ENSG00000198843 | 1402.648871 | -0.0601021 | 0.0766665 | 0.3459457 | 0.6121786 | SELENOT | | 51714 |
| ENSG00000240137 | 25.02672835 | 0.000884575 | 0.1322139 | 0.9809369 | NA | ERICH6-AS1 | | 101928085 |
| ENSG00000181788 | 383.9559418 | 0.059304284 | 0.1074157 | 0.3545579 | 0.6187516 | SIAH2 | | 6478 |
| ENSG00000144893 | 239.6001603 | -0.018472626 | 0.1103874 | 0.7790734 | 0.907593 | MED12L | | 116931 |
| ENSG00000152580 | 225.5650751 | -0.220233904 | 0.1951945 | 0.0322481 | 0.1448142 | IGSF10 | | 285313 |
| ENSG00000242908 | 99.54214447 | 0.03424347 | 0.1259186 | 0.5506196 | 0.7717069 | NA | NA | |
| ENSG00000242097 | 13.85832642 | -0.00287865 | 0.1341339 | 0.9092568 | NA | NA | NA | |
| ENSG00000152601 | 2956.368001 | -0.147641386 | 0.0667061 | 0.0120766 | 0.0726775 | MBNL1 | | 4154 |
| ENSG00000229619 | 23.2082091 | 0.004904489 | 0.1326709 | 0.8813233 | NA | MBNL1-AS1 | | 401093 |
| ENSG00000169860 | 9.763094584 | 0.000276052 | 0.1350232 | 0.9918922 | NA | P2RY1 | | 5028 |
| ENSG00000181467 | 527.2439035 | 0.010075676 | 0.091578 | 0.8832366 | 0.9528582 | RAP2B | | 5912 |
| ENSG00000114790 | 409.3360227 | -0.178476041 | 0.1377821 | 0.0462926 | 0.1840517 | ARHGEF26 | | 26084 |
| ENSG00000174953 | 1470.787932 | -0.061352802 | 0.0739705 | 0.3256699 | 0.5942127 | DHX36 | | 170506 |
| ENSG00000174928 | 50.02020279 | -0.042596678 | 0.1367855 | 0.3516165 | 0.6173544 | C3orf33 | | 285315 |
| ENSG00000169359 | 617.2868151 | 0.051830647 | 0.0934458 | 0.4580626 | 0.7023667 | SLC33A1 | | 9197 |
| ENSG00000163655 | 2145.107157 | -0.143212581 | 0.0720382 | 0.0215395 | 0.1092499 | GMPS | | 8833 |
| ENSG00000114850 | 3559.215178 | 0.031386785 | 0.0585876 | 0.5559485 | 0.7757478 | SSR3 | | 6747 |
| ENSG00000163659 | 921.8544585 | -0.125174343 | 0.0943548 | 0.0867597 | 0.2765938 | TIPARP | | 25976 |
| ENSG00000240875 | 53.66187001 | 0.051830756 | 0.139902 | 0.2858902 | 0.5557469 | LINC00886 | | 730091 |
| ENSG00000197980 | 8.996242982 | 0.006439306 | 0.1352756 | 0.7575293 | NA | LEKR1 | | 389170 |
| ENSG00000163660 | 2222.573071 | 0.097588548 | 0.0732819 | 0.1175134 | 0.3339561 | CCNL1 | | 57018 |
| ENSG00000163661 | 1133.692168 | 0.442668628 | 0.0932497 | 1.90E-07 | 8.22E-06 | PTX3 | | 5806 |
| ENSG00000174899 | 43.31180633 | 0.040393849 | 0.1370191 | 0.3537927 | 0.6187516 | SLC66A1L | | 152078 |
| ENSG00000174891 | 437.5870503 | 0.001703412 | 0.0983116 | 0.9816047 | 0.9932057 | RSRC1 | | 51319 |
| ENSG00000178053 | 739.7530709 | -0.02362007 | 0.0855168 | 0.7231911 | 0.8752015 | MLF1 | | 4291 |
| ENSG00000168827 | 1552.801347 | 0.072198667 | 0.0742128 | 0.2491364 | 0.515466 | GFM1 | | 85476 |
| ENSG00000118855 | 998.3413039 | 0.024508148 | 0.0783857 | 0.7037534 | 0.8656849 | MFSD1 | | 64747 |
| ENSG00000250588 | 135.9882804 | 0.118668491 | 0.1650947 | 0.1160242 | 0.3320948 | NA | NA | |
| ENSG00000151967 | 39.9907986 | 0.006222362 | 0.1300464 | 0.8798197 | NA | SCHIP1 | | 29970 |
| ENSG00000168811 | 20.35008515 | 0.005714801 | 0.1333804 | 0.8497097 | NA | IL12A | | 3592 |
| ENSG00000180044 | 23.33295694 | 0.014187851 | 0.1336103 | 0.662744 | NA | C3orf80 | | 401097 |
| ENSG00000068885 | 348.5516202 | 0.078687876 | 0.113261 | 0.2871837 | 0.5571488 | IFT80 | | 57560 |
| ENSG00000113810 | 6166.63923 | -0.179400973 | 0.0579789 | 0.0007133 | 0.0085279 | SMC4 | | 10051 |
| ENSG00000213186 | 315.6222559 | -0.431846558 | 0.1672768 | 0.0007095 | 0.0084909 | TRIM59 | | 286827 |
| ENSG00000186432 | 2527.433338 | 0.005372666 | 0.062659 | 0.9242285 | 0.9709924 | KPNA4 | | 3840 |
| ENSG00000229320 | 83.41808532 | 0.033873092 | 0.128728 | 0.5274886 | 0.7544502 | KRT8P12 | | 90133 |
| ENSG00000163590 | 285.9518816 | -0.058008844 | 0.1125561 | 0.4090122 | 0.6643076 | PPM1L | | 151742 |
| ENSG00000169255 | 560.4095359 | 0.067130526 | 0.0967511 | 0.3529201 | 0.6185235 | B3GALNT1 | | 8706 |
| ENSG00000169251 | 1328.68764 | 0.108888969 | 0.0794432 | 0.098509 | 0.2995012 | NMD3 | | 51068 |
| ENSG00000234851 | 435.7749816 | 0.034311248 | 0.0993072 | 0.6213814 | 0.818389 | RPL23AP42 | | 647099 |
| ENSG00000244128 | 31.16066277 | 0.002982844 | 0.1315517 | 0.9354512 | NA | LINC01322 | | 103695433 |
| ENSG00000114209 | 1080.461972 | -0.207707386 | 0.094109 | 0.0067805 | 0.0476548 | PDCD10 | | 11235 |
| ENSG00000163536 | 27.24829889 | 0.034464589 | 0.1377995 | 0.3354353 | NA | SERPINI1 | | 5274 |
| ENSG00000173905 | 1201.56223 | -0.192663918 | 0.0905962 | 0.0092672 | 0.0605762 | GOLIM4 | | 27333 |
| ENSG00000085276 | 57.12706228 | 0.097588121 | 0.1730261 | 0.0822584 | 0.266996 | MECOM | | 2122 |
| ENSG00000213178 | 14.77724828 | -0.017175433 | 0.135772 | 0.4970259 | NA | NA | NA | |
| ENSG00000270141 | 9.730837412 | -0.004686751 | 0.1349498 | 0.8288708 | NA | NA | NA | |
| ENSG00000184378 | 116.7695185 | -0.110649075 | 0.1646264 | 0.1215069 | 0.3414625 | ACTRT3 | | 84517 |
| ENSG00000085274 | 660.9077498 | 0.328266192 | 0.1208717 | 0.0007611 | 0.0089802 | MYNN | | 55892 |

| | | | | | | | | |
|---|---|---|---|---|---|---|---|---|
| ENSG00000269984 | 9.606602791 | 0.006914788 | 0.1351294 | 0.7529566 | NA | NA | NA | |
| ENSG00000270096 | 9.991022944 | 0.005396792 | 0.1349436 | 0.8103986 | NA | NA | NA | |
| ENSG00000008952 | 2344.726885 | -0.080828277 | 0.0695585 | 0.1776449 | 0.4270449 | SEC62 | | 7095 |
| ENSG00000173890 | 36.20248678 | -0.040832579 | 0.1386976 | 0.3077128 | NA | GPR160 | | 26996 |
| ENSG00000173889 | 1324.168697 | 0.00500325 | 0.0731348 | 0.9363633 | 0.9755316 | PHC3 | | 80012 |
| ENSG00000163558 | 2109.190231 | -0.203858219 | 0.0754887 | 0.0019222 | 0.0183595 | PRKCI | | 5584 |
| ENSG00000136603 | 823.4025934 | -0.289157836 | 0.108752 | 0.0011183 | 0.0121963 | SKIL | | 6498 |
| ENSG00000013297 | 2530.650478 | -0.160456767 | 0.1918465 | 0.0648883 | 0.2298241 | CLDN11 | | 5010 |
| ENSG00000163584 | 512.8125212 | 0.483481621 | 0.1538982 | 0.0001185 | 0.0019968 | RPL22L1 | | 200916 |
| ENSG00000163577 | 331.054031 | -0.032675294 | 0.1035776 | 0.6353388 | 0.8266198 | EIF5A2 | | 56648 |
| ENSG00000154310 | 17.88610033 | 0.014729732 | 0.1344981 | 0.615826 | NA | TNIK | | 23043 |
| ENSG00000075651 | 73.35908261 | 0.012656957 | 0.1259648 | 0.8026363 | 0.9177708 | PLD1 | | 5337 |
| ENSG00000075420 | 1422.324113 | -0.050848145 | 0.0732487 | 0.4119197 | 0.6661689 | FNDC3B | | 64778 |
| ENSG00000121858 | 9.492330197 | -0.006099481 | 0.1350697 | 0.7770766 | NA | TNFSF10 | | 8743 |
| ENSG00000144959 | 3552.811704 | 0.207384138 | 0.0801218 | 0.0026282 | 0.0233102 | NCEH1 | | 57552 |
| ENSG00000114346 | 7254.393278 | -0.426964346 | 0.0627111 | 1.01E-12 | 1.32E-10 | ECT2 | | 1894 |
| ENSG00000177565 | 1570.56823 | -0.276284983 | 0.0825101 | 0.0001388 | 0.0022733 | TBL1XR1 | | 79718 |
| ENSG00000172667 | 693.1616667 | -0.020483358 | 0.0867136 | 0.7596625 | 0.8969311 | ZMAT3 | | 64393 |
| ENSG00000121879 | 545.012421 | 0.004807279 | 0.0945679 | 0.9449097 | 0.9784014 | PIK3CA | | 5290 |
| ENSG00000171121 | 66.79338535 | 0.075557579 | 0.1511839 | 0.1736011 | 0.4208872 | KCNMB3 | | 27094 |
| ENSG00000240429 | 88.46878685 | 0.564038537 | 0.3623109 | 0.004137 | 0.0327401 | NA | NA | |
| ENSG00000121864 | 911.1791123 | -0.078387138 | 0.0854876 | 0.2470186 | 0.5129849 | ZNF639 | | 51193 |
| ENSG00000273190 | 11.63910111 | -0.018918127 | 0.1366614 | 0.4028146 | NA | NA | NA | |
| ENSG00000171109 | 1100.930196 | -0.352227094 | 0.0972706 | 3.42E-05 | 0.0007167 | MFN1 | | 55669 |
| ENSG00000269728 | 408.390283 | -0.152171314 | 0.1290584 | 0.0723913 | 0.2457085 | NA | NA | |
| ENSG00000242539 | 11.03332815 | 0.011087097 | 0.1352697 | 0.6359116 | NA | NA | NA | |
| ENSG00000114450 | 810.9333278 | -0.158419996 | 0.1056298 | 0.0450138 | 0.1806896 | GNB4 | | 59345 |
| ENSG00000136518 | 2249.930578 | -0.090547462 | 0.0712072 | 0.1374095 | 0.3657052 | ACTL6A | | 86 |
| ENSG00000272910 | 34.48027153 | -0.028912079 | 0.1348672 | 0.4515341 | NA | NA | NA | |
| ENSG00000136522 | 750.0145761 | -0.16107943 | 0.1056848 | 0.0421132 | 0.1732129 | MRPL47 | | 57129 |
| ENSG00000136521 | 850.5344123 | 0.084444323 | 0.0882884 | 0.2243995 | 0.4850501 | NDUFB5 | | 4711 |
| ENSG00000058056 | 734.2089372 | -0.180811886 | 0.1054465 | 0.0241854 | 0.1182036 | USP13 | | 8975 |
| ENSG00000163728 | 621.5637975 | -0.158797632 | 0.1104724 | 0.0493375 | 0.1908949 | TTC14 | | 151613 |
| ENSG00000114416 | 3404.480426 | -0.225507752 | 0.0704986 | 0.0003373 | 0.0047528 | FXR1 | | 8087 |
| ENSG00000205981 | 382.0310854 | -0.173968311 | 0.1374732 | 0.0501776 | 0.1928676 | DNAJC19 | | 131118 |
| ENSG00000058063 | 1316.783375 | -0.208854082 | 0.0897126 | 0.0050082 | 0.0380369 | ATP11B | | 23200 |
| ENSG00000043093 | 932.9536025 | -0.105367271 | 0.0890126 | 0.1331876 | 0.3595479 | DCUN1D1 | | 54165 |
| ENSG00000078070 | 381.5914318 | -0.087266241 | 0.1127887 | 0.2437231 | 0.5095683 | MCCC1 | | 56922 |
| ENSG00000078081 | 51.60160518 | -0.002054503 | 0.1284263 | 0.9656179 | 0.9863288 | LAMP3 | | 27074 |
| ENSG00000053524 | 58.2734524 | -0.034316874 | 0.1321965 | 0.4756931 | 0.7154077 | MCF2L2 | | 23101 |
| ENSG00000176597 | 442.4716585 | -0.044517957 | 0.1060148 | 0.5230217 | 0.7505426 | B3GNT5 | | 84002 |
| ENSG00000240024 | 116.6321179 | 0.034865204 | 0.1249359 | 0.5498648 | 0.771065 | NA | NA | |
| ENSG00000114796 | 330.0687353 | 0.567468093 | 0.1816772 | 0.0001052 | 0.0018212 | KLHL24 | | 54800 |
| ENSG00000163872 | 1696.358786 | 0.134143819 | 0.0815728 | 0.0470741 | 0.1855728 | YEATS2 | | 55689 |
| ENSG00000233885 | 14.77342757 | -0.014317085 | 0.1354325 | 0.5609429 | NA | NA | NA | |
| ENSG00000180834 | 135.9733492 | -0.042984477 | 0.1243112 | 0.4863138 | 0.7239115 | MAP6D1 | | 79929 |
| ENSG00000175193 | 872.2107257 | -0.027690015 | 0.0815581 | 0.6740026 | 0.8501975 | PARL | | 55486 |
| ENSG00000114770 | 978.8592016 | -0.013628488 | 0.0798361 | 0.832704 | 0.9305286 | ABCC5 | | 10057 |
| ENSG00000145191 | 1204.480401 | -0.192712181 | 0.0924887 | 0.0102078 | 0.0644519 | EIF2B5 | | 8893 |
| ENSG00000161202 | 1520.468892 | -0.122044245 | 0.0789987 | 0.0640489 | 0.2277105 | DVL3 | | 1857 |
| ENSG00000161203 | 5855.402046 | -0.080272955 | 0.0556681 | 0.1139412 | 0.3281578 | AP2M1 | | 1173 |
| ENSG00000161204 | 1269.1388 | -0.057750858 | 0.0756139 | 0.3606538 | 0.6239332 | ABCF3 | | 55324 |
| ENSG00000214160 | 567.7827385 | 0.124564494 | 0.1103375 | 0.1114173 | 0.3236673 | ALG3 | | 10195 |
| ENSG00000145194 | 216.6381442 | 0.164350135 | 0.1707828 | 0.0673339 | 0.2349246 | ECE2 | | 9718 |
| ENSG00000163888 | 87.91025916 | 0.017621351 | 0.1244585 | 0.743945 | 0.8886411 | CAMK2N2 | | 94032 |
| ENSG00000175166 | 10898.53661 | 0.008537722 | 0.0494724 | 0.8523134 | 0.9396765 | PSMD2 | | 5708 |
| ENSG00000114867 | 16644.02766 | -0.206617087 | 0.0542493 | 4.08E-05 | 0.0008285 | EIF4G1 | | 1981 |
| ENSG00000175182 | 241.0254149 | 0.131241668 | 0.1474488 | 0.1118932 | 0.3244846 | FAM131A | | 131408 |
| ENSG00000114859 | 267.1259089 | -0.054229613 | 0.1138714 | 0.4337414 | 0.684513 | CLCN2 | | 1181 |
| ENSG00000163882 | 1266.719721 | -0.079731693 | 0.0787985 | 0.2200082 | 0.4795513 | POLR2H | | 5437 |
| ENSG00000177383 | 207.1518406 | 0.000314184 | 0.1101085 | 0.9981564 | 0.9989595 | MAGEF1 | | 64110 |
| ENSG00000156931 | 792.0120873 | 0.013048834 | 0.0865132 | 0.8572923 | 0.9424084 | VPS8 | | 23355 |
| ENSG00000113790 | 88.74452802 | 0.042802344 | 0.1303288 | 0.4439226 | 0.6921329 | EHHADH | | 1962 |
| ENSG00000073803 | 305.8961274 | -0.154091419 | 0.1451359 | 0.0763158 | 0.2543354 | MAP3K13 | | 9175 |
| ENSG00000163900 | 525.9449213 | 0.1002252 | 0.1068496 | 0.1904781 | 0.4412794 | TMEM41A | | 90407 |
| ENSG00000163898 | 15.85492079 | 0.016068849 | 0.1351064 | 0.5618505 | NA | LIPH | | 200879 |
| ENSG00000163904 | 1075.775696 | -0.103594725 | 0.0847723 | 0.128845 | 0.3532125 | SENP2 | | 59343 |
| ENSG00000073792 | 2140.666798 | -0.042635266 | 0.0681126 | 0.4696431 | 0.7112539 | IGF2BP2 | | 10644 |
| ENSG00000136527 | 4357.325236 | -0.28191063 | 0.0738992 | 2.35E-05 | 0.0005162 | TRA2B | | 6434 |
| ENSG00000244405 | 985.0908931 | -0.015539486 | 0.0825633 | 0.8126043 | 0.92308 | ETV5 | | 2119 |

| | | | | | | | | |
|---|---|---|---|---|---|---|---|---|
| ENSG00000213139 | 47.80963797 | -0.017650416 | 0.1306445 | 0.6756 | 0.8512224 | CRYGS | | 1427 |
| ENSG00000113838 | 286.0820637 | -0.017072105 | 0.1048103 | 0.7999762 | 0.9169162 | TBCCD1 | | 55171 |
| ENSG00000090520 | 2129.63946 | -0.105782364 | 0.0710193 | 0.0829667 | 0.2688305 | DNAJB11 | | 51726 |
| ENSG00000263826 | 15.660978 | 0.011004754 | 0.1344795 | 0.6871355 | NA | NA | NA | |
| ENSG00000156976 | 7684.189462 | -0.015278895 | 0.0517374 | 0.7490896 | 0.8911635 | EIF4A2 | | 1974 |
| ENSG00000163918 | 923.9577503 | 0.019744096 | 0.081402 | 0.76375 | 0.8982943 | RFC4 | | 5984 |
| ENSG00000163923 | 83.12648526 | -0.021518721 | 0.1260116 | 0.6801166 | 0.853781 | RPL39L | | 116832 |
| ENSG00000113916 | 26.35772588 | 0.078342071 | 0.1655962 | 0.0384313 | NA | BCL6 | | 604 |
| ENSG00000270959 | 30.01442814 | 0.008700699 | 0.1317326 | 0.8135129 | NA | LPP-AS2 | | 339929 |
| ENSG00000145012 | 1606.681805 | 0.021074065 | 0.0696381 | 0.7257955 | 0.8770967 | LPP | | 4026 |
| ENSG00000090530 | 2886.651436 | 0.062692413 | 0.0643068 | 0.2696183 | 0.5374423 | P3H2 | | 55214 |
| ENSG00000163347 | 18415.99345 | -0.36687911 | 0.0548639 | 2.83E-12 | 3.34E-10 | CLDN1 | | 9076 |
| ENSG00000113946 | 86.48950238 | 0.071590746 | 0.1442101 | 0.2271032 | 0.4882146 | CLDN16 | | 10686 |
| ENSG00000196083 | 407.9610836 | 0.044093726 | 0.1005498 | 0.5287751 | 0.754996 | IL1RAP | | 3556 |
| ENSG00000152492 | 1551.952082 | 0.136824323 | 0.0779149 | 0.0372691 | 0.159758 | CCDC50 | | 152137 |
| ENSG00000180611 | 219.4534926 | 0.058306605 | 0.118565 | 0.4012383 | 0.6590079 | MB21D2 | | 151963 |
| ENSG00000198836 | 2088.519845 | -0.135658158 | 0.081749 | 0.0449215 | 0.1805564 | OPA1 | | 4976 |
| ENSG00000224855 | 14.90936986 | -0.015577935 | 0.1352675 | 0.5541998 | NA | OPA1-AS1 | | 100873941 |
| ENSG00000114315 | 188.885316 | 0.040988643 | 0.1167626 | 0.5371526 | 0.7611135 | HES1 | | 3280 |
| ENSG00000133657 | 5153.51476 | 0.215451499 | 0.0621525 | 0.0001444 | 0.0023395 | ATP13A3 | | 79572 |
| ENSG00000244675 | 156.2718934 | 0.39783247 | 0.2915142 | 0.0080782 | 0.0544497 | NA | NA | |
| ENSG00000231770 | 38.43757975 | -0.038978573 | 0.1378949 | 0.3302152 | NA | NA | NA | |
| ENSG00000145014 | 366.7123377 | -0.00669228 | 0.1050072 | 0.9141001 | 0.9662115 | TMEM44 | | 93109 |
| ENSG00000041802 | 1972.249596 | 0.138458804 | 0.0736319 | 0.0283927 | 0.1328746 | LSG1 | | 55341 |
| ENSG00000185112 | 397.513165 | 0.088851325 | 0.1164274 | 0.2379193 | 0.5025095 | FAM43A | | 131583 |
| ENSG00000173950 | 189.7861312 | -0.111850348 | 0.1502201 | 0.1455817 | 0.3780659 | XXYLT1 | | 152002 |
| ENSG00000114331 | 1425.221932 | -0.144610275 | 0.0837208 | 0.03594 | 0.1558335 | ACAP2 | | 23527 |
| ENSG00000229325 | 11.7374888 | -0.032021567 | 0.1400498 | 0.182852 | NA | NA | NA | |
| ENSG00000184203 | 1021.515756 | 0.074165095 | 0.0856479 | 0.2788894 | 0.5470196 | PPP1R2 | | 5504 |
| ENSG00000272792 | 9.573485729 | 0.014808022 | 0.1360653 | 0.4975456 | NA | NA | NA | |
| ENSG00000229178 | 9.65375551 | -0.014143995 | 0.1359738 | 0.5109207 | NA | NA | NA | |
| ENSG00000242086 | 399.9833197 | 0.010150006 | 0.0976575 | 0.8834656 | 0.9528582 | SDHAP2 | | 727956 |
| ENSG00000207650 | 24.09366723 | 0.030584028 | 0.1370814 | 0.3671357 | NA | MIR570 | | 693155 |
| ENSG00000061938 | 733.8195659 | 0.02921557 | 0.085942 | 0.6640177 | 0.8443298 | TNK2 | | 10188 |
| ENSG00000260261 | 76.34032837 | 0.002041516 | 0.1243275 | 0.9700541 | 0.9881615 | NA | NA | |
| ENSG00000185485 | 390.2846147 | 0.023824276 | 0.1016919 | 0.7273538 | 0.8780864 | NA | NA | |
| ENSG00000072274 | 7856.357898 | -0.128260861 | 0.0539316 | 0.0099316 | 0.0633366 | TFRC | | 7037 |
| ENSG00000161217 | 1487.857378 | 0.013783242 | 0.0717504 | 0.8231303 | 0.9273712 | PCYT1A | | 5130 |
| ENSG00000213123 | 22.60832454 | 0.001674106 | 0.1332164 | 0.9592054 | NA | DYNLT2B | | 255758 |
| ENSG00000235897 | 31.75372868 | 0.942776699 | 0.6492117 | 0.0034313 | NA | TM4SF19-A! | | 100874214 |
| ENSG00000145107 | 289.6816781 | 0.732836194 | 0.170738 | 1.00E-06 | 3.40E-05 | TM4SF19 | | 116211 |
| ENSG00000163960 | 1585.706646 | -0.050834449 | 0.0737535 | 0.4142142 | 0.6685232 | UBXN7 | | 26043 |
| ENSG00000163961 | 610.6348344 | -0.027325112 | 0.0937112 | 0.6899192 | 0.8589854 | RNF168 | | 165918 |
| ENSG00000185798 | 285.0475158 | 0.224182199 | 0.1685502 | 0.0267295 | 0.1271074 | WDR53 | | 348793 |
| ENSG00000174013 | 836.4910918 | 0.246325107 | 0.106327 | 0.0037583 | 0.0304019 | FBXO45 | | 200933 |
| ENSG00000163964 | 591.1537989 | 0.135658625 | 0.1105451 | 0.0870244 | 0.2769087 | PIGX | | 54965 |
| ENSG00000174007 | 140.5502241 | 0.034658311 | 0.1220988 | 0.5717425 | 0.7864627 | CEP19 | | 84984 |
| ENSG00000180370 | 4703.06959 | -0.111116674 | 0.0580847 | 0.0342432 | 0.1504809 | PAK2 | | 5062 |
| ENSG00000119231 | 844.2352788 | -0.043894187 | 0.0842691 | 0.5093677 | 0.7410048 | SENP5 | | 205564 |
| ENSG00000230732 | 12.24702933 | 0.005011248 | 0.1345493 | 0.8389138 | NA | NA | NA | |
| ENSG00000114503 | 1812.364256 | 0.159597007 | 0.0818252 | 0.0195539 | 0.1019682 | NCBP2 | | 22916 |
| ENSG00000225578 | 9.190793977 | 0.011723007 | 0.1356736 | 0.5858894 | NA | NCBP2-AS1 | | 100874001 |
| ENSG00000270170 | 461.1711459 | 0.10624852 | 0.110837 | 0.1663067 | 0.4112342 | NCBP2AS2 | | 152217 |
| ENSG00000119227 | 47.81879059 | 0.064449489 | 0.1483061 | 0.1784439 | 0.4275716 | PIGZ | | 80235 |
| ENSG00000163975 | 832.0051777 | 0.292079526 | 0.1092935 | 0.0010661 | 0.011737 | MELTF | | 4241 |
| ENSG00000228109 | 19.38719845 | 0.004904748 | 0.1333709 | 0.8704191 | NA | MELTF-AS1 | | 100507057 |
| ENSG00000075711 | 2688.355846 | -0.078185377 | 0.0650206 | 0.1715111 | 0.4177334 | DLG1 | | 1739 |
| ENSG00000161267 | 494.5209901 | 0.013707371 | 0.0940877 | 0.8423363 | 0.9350382 | BDH1 | | 622 |
| ENSG00000214135 | 386.3718845 | -0.087904683 | 0.118603 | 0.2414568 | 0.5069118 | NA | NA | |
| ENSG00000145016 | 868.6455922 | 0.020756 | 0.0829906 | 0.7541567 | 0.8937105 | RUBCN | | 9711 |
| ENSG00000122068 | 1361.335836 | -0.080735207 | 0.0818236 | 0.2261454 | 0.4872011 | FYTTD1 | | 84248 |
| ENSG00000186001 | 1114.277358 | -0.144859669 | 0.0927267 | 0.0478843 | 0.1870785 | LRCH3 | | 84859 |
| ENSG00000114473 | 69.3424254 | 0.045772733 | 0.1344162 | 0.3816832 | 0.6427046 | IQCG | | 84223 |
| ENSG00000182899 | 7193.991772 | -0.198954538 | 0.0626282 | 0.0004546 | 0.0059537 | RPL35A | | 6165 |
| ENSG00000185621 | 198.9458337 | 0.195389432 | 0.2098955 | 0.0439146 | 0.177715 | LMLN | | 89782 |
| ENSG00000236438 | 27.02296744 | 0.03269172 | 0.1372268 | 0.358353 | NA | NA | NA | |
| ENSG00000197701 | 53.0917407 | 0.027319717 | 0.131608 | 0.5472929 | 0.7690111 | NA | NA | |
| ENSG00000131127 | 202.1547166 | 0.047007859 | 0.1179811 | 0.4815165 | 0.7206398 | ZNF141 | | 7700 |
| ENSG00000251595 | 50.94064323 | 0.019955151 | 0.130068 | 0.6569573 | 0.8419 | ABCA11P | | 79963 |
| ENSG00000182903 | 409.6287763 | 0.021595134 | 0.0978708 | 0.7537037 | 0.8933549 | ZNF721 | | 170960 |

| | | | | | | | | |
|---|---|---|---|---|---|---|---|---|
| ENSG00000174227 | 1104.302677 | 0.115041926 | 0.0868651 | 0.0995063 | 0.3010446 | PIGG | | 54872 |
| ENSG00000169020 | 760.8373302 | -0.04460385 | 0.1250755 | 0.4941303 | 0.7297633 | ATP5ME | | 521 |
| ENSG00000215375 | 46.07203857 | 0.028829856 | 0.1327946 | 0.5087902 | 0.7404907 | MYL5 | | 4636 |
| ENSG00000185619 | 984.1792551 | -0.046419131 | 0.0816237 | 0.4824441 | 0.7213706 | PCGF3 | | 10336 |
| ENSG00000249592 | 39.13318596 | 0.026854045 | 0.1335509 | 0.5093569 | NA | PCGF3-AS1 | | 100129917 |
| ENSG00000168993 | 23.59063193 | -0.019226121 | 0.1343858 | 0.5547415 | NA | CPLX1 | | 10815 |
| ENSG00000178950 | 1362.044866 | -0.002529031 | 0.0727215 | 0.9663638 | 0.9865549 | GAK | | 2580 |
| ENSG00000127419 | 176.0210922 | 0.119130205 | 0.1546975 | 0.1270921 | 0.3502981 | TMEM175 | | 84286 |
| ENSG00000145214 | 287.5274176 | 0.140548505 | 0.1437677 | 0.0965056 | 0.2953319 | DGKQ | | 1609 |
| ENSG00000127415 | 70.80904197 | 0.055381328 | 0.1384881 | 0.3016076 | 0.5710237 | IDUA | | 3425 |
| ENSG00000127418 | 418.1656698 | 0.174131327 | 0.1321234 | 0.0477684 | 0.1868392 | FGFRL1 | | 53834 |
| ENSG00000159692 | 2259.299196 | -0.067069893 | 0.0661416 | 0.246118 | 0.5119133 | CTBP1 | | 1487 |
| ENSG00000196810 | 230.4499779 | 0.056482902 | 0.1169901 | 0.4172528 | 0.6707704 | CTBP1-DT | | 92070 |
| ENSG00000090316 | 1360.193046 | 0.027226407 | 0.0724408 | 0.6588557 | 0.842576 | MAEA | | 10296 |
| ENSG00000163945 | 296.9982116 | -0.018495089 | 0.1043602 | 0.7843359 | 0.9098848 | UVSSA | | 57654 |
| ENSG00000179979 | 154.3204754 | -0.046152139 | 0.1223113 | 0.4706975 | 0.7115591 | NA | NA | |
| ENSG00000174137 | 23.86862194 | 1.187351904 | 0.7363104 | 0.0026507 | NA | FAM53A | | 152877 |
| ENSG00000163950 | 2314.697228 | 0.24020204 | 0.0744537 | 0.0002754 | 0.0040164 | SLBP | | 7884 |
| ENSG00000270195 | 8.562933088 | 0.011539189 | 0.1359956 | 0.5526688 | NA | NA | NA | |
| ENSG00000168936 | 470.3225726 | 0.361894415 | 0.1415106 | 0.0010006 | 0.0110896 | TMEM129 | | 92305 |
| ENSG00000013810 | 3031.970879 | -0.00587221 | 0.0601846 | 0.9138071 | 0.9661641 | TACC3 | | 10460 |
| ENSG00000218422 | 76.36946678 | -0.03174258 | 0.1287339 | 0.5425401 | 0.7657123 | NA | NA | |
| ENSG00000068078 | 39.87456192 | 0.022726363 | 0.1328909 | 0.5666939 | NA | FGFR3 | | 2261 |
| ENSG00000168924 | 2039.949899 | -0.002377142 | 0.0666267 | 0.9743831 | 0.9904047 | LETM1 | | 3954 |
| ENSG00000109685 | 4904.172384 | -0.146275125 | 0.0606664 | 0.0074032 | 0.0508752 | NSD2 | | 7468 |
| ENSG00000185049 | 718.2723237 | 0.013502146 | 0.0893844 | 0.8428441 | 0.9350614 | NELFA | | 7469 |
| ENSG00000243449 | 377.4368956 | 0.034345681 | 0.1055462 | 0.6131663 | 0.8149272 | NICOL1 | | 401115 |
| ENSG00000185818 | 137.7854778 | 0.456233496 | 0.2774531 | 0.0046885 | 0.0361975 | NAT8L | | 339983 |
| ENSG00000130997 | 19.04120141 | 0.024953141 | 0.1364696 | 0.4071266 | NA | POLN | | 353497 |
| ENSG00000214367 | 513.2656786 | 0.099513168 | 0.1079325 | 0.1890315 | 0.440063 | HAUS3 | | 79441 |
| ENSG00000123933 | 385.9978064 | 0.021718053 | 0.0993522 | 0.7530673 | 0.8932431 | MXD4 | | 10608 |
| ENSG00000159733 | 123.0933898 | 0.012483019 | 0.1191446 | 0.8341568 | 0.9305701 | ZFYVE28 | | 57732 |
| ENSG00000063978 | 1333.664029 | -0.030114693 | 0.0729154 | 0.62516 | 0.8202285 | RNF4 | | 6047 |
| ENSG00000125386 | 547.1887955 | 0.015002635 | 0.0917253 | 0.826508 | 0.9286016 | FAM193A | | 8603 |
| ENSG00000168884 | 1118.020831 | 0.106867196 | 0.0859081 | 0.1228983 | 0.3436858 | TNIP2 | | 79155 |
| ENSG00000087266 | 495.4742699 | 0.143517835 | 0.1167336 | 0.077919 | 0.2574745 | SH3BP2 | | 6452 |
| ENSG00000087274 | 2288.680172 | -0.18050885 | 0.0758902 | 0.0058158 | 0.0425653 | ADD1 | | 118 |
| ENSG00000109736 | 1334.116648 | -0.062969849 | 0.0759309 | 0.3206573 | 0.5893011 | MFSD10 | | 10227 |
| ENSG00000249673 | 297.2433212 | 0.629069653 | 0.1750861 | 1.89E-05 | 0.0004328 | NA | NA | |
| ENSG00000087269 | 1586.519196 | 0.033243306 | 0.0723488 | 0.5891151 | 0.798683 | NOP14 | | 8602 |
| ENSG00000125388 | 57.19067363 | 0.010545383 | 0.1276108 | 0.8233755 | 0.9274686 | GRK4 | | 2868 |
| ENSG00000197386 | 2091.240535 | -0.185781021 | 0.0783436 | 0.005601 | 0.0412779 | HTT | | 3064 |
| ENSG00000159788 | 258.0430265 | -0.015485482 | 0.1081096 | 0.8151497 | 0.9244431 | RGS12 | | 6002 |
| ENSG00000175920 | 63.37605901 | -1.055993942 | 0.3883792 | 0.0002419 | 0.0036001 | DOK7 | | 285489 |
| ENSG00000261643 | 15.95243997 | -0.001681176 | 0.1338899 | 0.9478226 | NA | NA | NA | |
| ENSG00000163956 | 1452.638539 | -0.114813721 | 0.07852 | 0.0797243 | 0.2613697 | LRPAP1 | | 4043 |
| ENSG00000184160 | 9.008558858 | 0.019091853 | 0.1369767 | 0.3663805 | NA | ADRA2C | | 152 |
| ENSG00000251669 | 23.30894202 | 0.010576018 | 0.1331172 | 0.7455029 | NA | NA | NA | |
| ENSG00000132406 | 149.5792401 | -0.003912368 | 0.1170202 | 0.9454171 | 0.9784014 | TMEM128 | | 85013 |
| ENSG00000145220 | 701.8742496 | 0.002019038 | 0.0874342 | 0.9769651 | 0.9913073 | LYAR | | 55646 |
| ENSG00000168826 | 59.25196246 | -0.039292192 | 0.1348569 | 0.4008061 | 0.6587248 | ZBTB49 | | 166793 |
| ENSG00000168818 | 459.2341558 | 0.051008609 | 0.0992671 | 0.4704369 | 0.7115591 | STX18 | | 53407 |
| ENSG00000247708 | 18.45952975 | 0.019138624 | 0.1352544 | 0.5173263 | NA | STX18-AS1 | | 100507266 |
| ENSG00000163132 | 97.93397475 | 0.115740163 | 0.1754624 | 0.0986119 | 0.2996336 | MSX1 | | 4487 |
| ENSG00000173040 | 22.52251303 | 0.027825084 | 0.1371467 | 0.3632324 | NA | EVC2 | | 132884 |
| ENSG00000072840 | 300.6901045 | 0.100263048 | 0.1324671 | 0.1892738 | 0.4402444 | EVC | | 2121 |
| ENSG00000109501 | 265.1862613 | 0.004238273 | 0.1069635 | 0.9504896 | 0.9802173 | WFS1 | | 7466 |
| ENSG00000074211 | 85.99031505 | -0.167561745 | 0.2465385 | 0.0397308 | 0.1669409 | PPP2R2C | | 5522 |
| ENSG00000013288 | 726.5233492 | -0.014074193 | 0.0844199 | 0.8314183 | 0.9304418 | MAN2B2 | | 23324 |
| ENSG00000179010 | 5706.13837 | -0.114650989 | 0.0592317 | 0.0316167 | 0.142758 | MRFAP1 | | 93621 |
| ENSG00000251580 | 19.77259934 | 0.030752188 | 0.1380766 | 0.3158997 | NA | LINC02482 | | 105374366 |
| ENSG00000170846 | 430.9760172 | 0.184457714 | 0.1333996 | 0.0392132 | 0.1656025 | MRFAP1L2 | | 93622 |
| ENSG00000178988 | 817.766324 | -0.066180833 | 0.0880056 | 0.3344279 | 0.6019835 | MRFAP1L1 | | 114932 |
| ENSG00000186222 | 157.5218095 | -0.032162135 | 0.1186366 | 0.6090627 | 0.8123386 | BLOC1S4 | | 55330 |
| ENSG00000170871 | 680.146662 | -0.196598766 | 0.1182872 | 0.0226925 | 0.1134723 | KIAA0232 | | 9778 |
| ENSG00000132405 | 1398.842932 | -0.095705399 | 0.0777709 | 0.1397609 | 0.3691041 | TBC1D14 | | 57533 |
| ENSG00000245748 | 19.8678769 | -0.002663592 | 0.1332003 | 0.9271048 | NA | LOC100129! | | 100129931 |
| ENSG00000173013 | 39.59087469 | 0.032046634 | 0.1347638 | 0.440796 | NA | CCDC96 | | 257236 |
| ENSG00000173011 | 655.2423053 | -0.013086693 | 0.08742 | 0.8448512 | 0.9364877 | TADA2B | | 93624 |
| ENSG00000109519 | 1343.773883 | 0.076685752 | 0.0793907 | 0.241003 | 0.506323 | GRPEL1 | | 80273 |

| | | | | | | | |
|---|---|---|---|---|---|---|---|
| ENSG00000272620 | 8.652218641 | 0.01509863 | 0.1362898 | 0.4667238 | NA | AFAP1-AS1 | 84740 |
| ENSG00000196526 | 1968.284517 | 0.055905439 | 0.0704232 | 0.3554515 | 0.6192724 | AFAP1 | 60312 |
| ENSG00000125089 | 26.92008788 | -0.008016117 | 0.132994 | 0.7997926 | NA | SH3TC1 | 54436 |
| ENSG00000087008 | 235.6469936 | -0.076577706 | 0.1232006 | 0.2883884 | 0.558188 | ACOX3 | 8310 |
| ENSG00000155275 | 172.3528339 | 0.102819131 | 0.148043 | 0.1642831 | 0.4076092 | TRMT44 | 152992 |
| ENSG00000261490 | 16.27045206 | -0.037566028 | 0.1412871 | 0.1799787 | NA | NA | NA |
| ENSG00000071127 | 7995.448733 | -0.072867153 | 0.0542637 | 0.1421422 | 0.3726964 | WDR1 | 9948 |
| ENSG00000178163 | 334.917536 | -0.21503956 | 0.1573361 | 0.0282503 | 0.1324059 | ZNF518B | 85460 |
| ENSG00000157869 | 242.8102462 | -0.129886347 | 0.1454529 | 0.1137127 | 0.3277271 | RAB28 | 9364 |
| ENSG00000038219 | 1827.726723 | 0.022876403 | 0.0712059 | 0.707461 | 0.8676172 | BOD1L1 | 259282 |
| ENSG00000137449 | 1025.051345 | 0.16875749 | 0.093756 | 0.024079 | 0.1178327 | CPEB2 | 132864 |
| ENSG00000048342 | 461.3784929 | -0.018382435 | 0.0961537 | 0.7877104 | 0.9116145 | CC2D2A | 57545 |
| ENSG00000118564 | 813.4754789 | 0.031677057 | 0.0842695 | 0.6350373 | 0.8266184 | FBXL5 | 26234 |
| ENSG00000237765 | 443.1097315 | 0.037007813 | 0.0971859 | 0.5947724 | 0.8031833 | FAM200B | 285550 |
| ENSG00000169762 | 243.4103724 | 0.067031749 | 0.1250845 | 0.3337583 | 0.601084 | TAPT1 | 202018 |
| ENSG00000151552 | 288.5633042 | 0.0332345 | 0.1107418 | 0.6236664 | 0.8200188 | QDPR | 5860 |
| ENSG00000002549 | 1174.552571 | -0.207543695 | 0.0971397 | 0.0079965 | 0.0541176 | LAP3 | 51056 |
| ENSG00000118579 | 873.5742682 | -0.173308168 | 0.0993928 | 0.0253011 | 0.1220558 | MED28 | 80306 |
| ENSG00000163257 | 1026.350242 | -0.034628153 | 0.0818653 | 0.5977859 | 0.8055765 | DCAF16 | 54876 |
| ENSG00000109805 | 2608.693118 | -0.044682889 | 0.0644833 | 0.4310075 | 0.6823798 | NCAPG | 64151 |
| ENSG00000178177 | 188.7332778 | -0.003930399 | 0.1129603 | 0.9497783 | 0.9801538 | LCORL | 254251 |
| ENSG00000145147 | 58.47706965 | 0.099650812 | 0.1747057 | 0.0802704 | 0.2627911 | SLIT2 | 9353 |
| ENSG00000163138 | 325.1020146 | 0.115534579 | 0.1336892 | 0.1469372 | 0.3801492 | PACRGL | 133015 |
| ENSG00000152990 | 982.0437246 | -0.149972361 | 0.0923586 | 0.0407287 | 0.1696087 | ADGRA3 | 166647 |
| ENSG00000109606 | 4046.250436 | -0.209530021 | 0.0624297 | 0.000223 | 0.0033525 | DHX15 | 1665 |
| ENSG00000181982 | 81.33508602 | -0.053412746 | 0.1367044 | 0.3249027 | 0.5936475 | CCDC149 | 91050 |
| ENSG00000109618 | 110.3913796 | 0.003096972 | 0.1202844 | 0.9566515 | 0.982546 | SEPSECS | 51091 |
| ENSG00000038210 | 727.0144374 | 0.159849082 | 0.1051241 | 0.0433203 | 0.1762261 | PI4K2B | 55300 |
| ENSG00000168228 | 194.6149421 | 0.050871164 | 0.1214193 | 0.4432591 | 0.6918756 | ZCCHC4 | 29063 |
| ENSG00000053900 | 453.3517935 | -0.056356889 | 0.0999217 | 0.426015 | 0.6784296 | ANAPC4 | 29945 |
| ENSG00000091490 | 17.01460873 | 0.063898756 | 0.1561452 | 0.0337912 | NA | SEL1L3 | 23231 |
| ENSG00000250317 | 165.373592 | -0.01469658 | 0.1175888 | 0.8076445 | 0.9200093 | SMIM20 | 389203 |
| ENSG00000168214 | 1161.763604 | -0.008514125 | 0.0783926 | 0.9227391 | 0.9702979 | RBPJ | 3516 |
| ENSG00000109680 | 29.5002144 | -0.016598206 | 0.1328491 | 0.6427072 | NA | TBC1D19 | 55296 |
| ENSG00000109689 | 431.805359 | -0.128351793 | 0.1205156 | 0.1113464 | 0.3235418 | STIM2 | 57620 |
| ENSG00000169851 | 70.31672498 | -0.021809473 | 0.1274404 | 0.6632395 | 0.8438599 | PCDH7 | 5099 |
| ENSG00000047365 | 277.3377382 | -0.052413795 | 0.1111798 | 0.4520347 | 0.6983298 | ARAP2 | 116984 |
| ENSG00000181826 | 128.9178208 | -0.057926167 | 0.1302728 | 0.3574914 | 0.6209582 | RELL1 | 768211 |
| ENSG00000169299 | 674.3169422 | -0.148868007 | 0.1068304 | 0.0591024 | 0.2158772 | PGM2 | 55276 |
| ENSG00000065882 | 1033.628514 | -0.0556627 | 0.081523 | 0.4003062 | 0.6583091 | TBC1D1 | 23216 |
| ENSG00000109787 | 502.815636 | 0.129084735 | 0.1144507 | 0.1049375 | 0.3113474 | KLF3 | 51274 |
| ENSG00000174130 | 15.31546832 | -0.008957704 | 0.134287 | 0.7374021 | NA | TLR6 | 10333 |
| ENSG00000197712 | 433.2456465 | -0.228643507 | 0.14182 | 0.017701 | 0.0950747 | FAM114A1 | 92689 |
| ENSG00000109790 | 725.951657 | -0.095108416 | 0.0955292 | 0.1876429 | 0.4386788 | KLHL5 | 51088 |
| ENSG00000157796 | 420.1281159 | 0.098342835 | 0.1142918 | 0.197837 | 0.4511888 | WDR19 | 57728 |
| ENSG00000035928 | 1596.731288 | 0.0227598 | 0.0708701 | 0.7080462 | 0.8678177 | RFC1 | 5981 |
| ENSG00000163682 | 7952.755944 | -0.112872286 | 0.0578811 | 0.0311323 | 0.1416407 | RPL9 | 6133 |
| ENSG00000121897 | 123.4736746 | 0.06358984 | 0.1341494 | 0.3129371 | 0.5820729 | LIAS | 11019 |
| ENSG00000109814 | 539.0261097 | -0.017170748 | 0.0921155 | 0.8007159 | 0.9169162 | UGDH | 7358 |
| ENSG00000249348 | 10.39539301 | 0.017453082 | 0.1363568 | 0.4436229 | NA | UGDH-AS1 | 100885776 |
| ENSG00000163683 | 61.12058908 | 0.072164493 | 0.1506914 | 0.171273 | 0.4177334 | SMIM14 | 201895 |
| ENSG00000078140 | 1823.479615 | -0.150322684 | 0.0760865 | 0.0202995 | 0.1048731 | UBE2K | 3093 |
| ENSG00000121892 | 3592.595287 | -0.182501616 | 0.065041 | 0.0017086 | 0.0168985 | PDS5A | 23244 |
| ENSG00000078177 | 359.6528419 | -0.012440927 | 0.099983 | 0.8545577 | 0.9412625 | N4BP2 | 55728 |
| ENSG00000179299 | 28.01219705 | 0.012177641 | 0.1324643 | 0.7309939 | NA | NSUN7 | 79730 |
| ENSG00000163697 | 480.8346688 | 0.069157666 | 0.1040975 | 0.3204911 | 0.5891811 | APBB2 | 323 |
| ENSG00000064042 | 27.44839161 | 0.033101451 | 0.1379659 | 0.3266275 | NA | LIMCH1 | 22998 |
| ENSG00000109133 | 1088.482838 | 0.068174203 | 0.0805952 | 0.3017172 | 0.5710237 | TMEM33 | 55161 |
| ENSG00000182308 | 9.725209318 | 0.039512953 | 0.1436074 | 0.0642119 | NA | DCAF4L1 | 285429 |
| ENSG00000014824 | 912.4625327 | -0.02394903 | 0.0801619 | 0.7135657 | 0.870525 | SLC30A9 | 10463 |
| ENSG00000124406 | 115.3557332 | -0.082330992 | 0.1446388 | 0.2100863 | 0.4666816 | ATP8A1 | 10396 |
| ENSG00000151806 | 567.560685 | 0.008757598 | 0.0968293 | 0.8991215 | 0.9602639 | GUF1 | 60558 |
| ENSG00000163281 | 431.4353296 | 0.03574369 | 0.0985739 | 0.6074256 | 0.8116379 | GNPDA2 | 132789 |
| ENSG00000249936 | 79.19497827 | -0.025134702 | 0.1268434 | 0.6306112 | 0.8242329 | NA | NA |
| ENSG00000169019 | 316.4844307 | -0.307918008 | 0.1744688 | 0.0074042 | 0.0508752 | COMMD8 | 54951 |
| ENSG00000145246 | 169.0580878 | 0.056779058 | 0.1256049 | 0.3886165 | 0.6488607 | ATP10D | 57205 |
| ENSG00000259959 | 41.52118446 | -0.026256559 | 0.1330114 | 0.5241915 | 0.7515816 | NA | NA |
| ENSG00000170448 | 524.6119841 | 0.264245311 | 0.1288606 | 0.0059521 | 0.043238 | NFXL1 | 152518 |
| ENSG00000163293 | 91.01965767 | 0.149547321 | 0.2191922 | 0.0518247 | 0.1968026 | NIPAL1 | 152519 |
| ENSG00000074966 | 8.439558357 | 0.011326475 | 0.1358783 | 0.5714867 | NA | TXK | 7294 |

| | | | | | | | | |
|---|---|---|---|---|---|---|---|---|
| ENSG00000135605 | 79.09120555 | -0.008321695 | 0.1248019 | 0.869724 | 0.9475003 | TEC | | 7006 |
| ENSG00000109171 | 660.2935548 | -0.088758081 | 0.0977666 | 0.2211085 | 0.4805935 | SLAIN2 | | 57606 |
| ENSG00000075539 | 1186.55256 | 0.025112764 | 0.0751204 | 0.670965 | 0.8493218 | FRYL | | 285527 |
| ENSG00000109180 | 1125.275329 | -0.22878376 | 0.094729 | 0.0033989 | 0.0280781 | OCIAD1 | | 54940 |
| ENSG00000145247 | 572.6645586 | -0.106910837 | 0.1032556 | 0.1546646 | 0.3930036 | OCIAD2 | | 132299 |
| ENSG00000109184 | 681.7789293 | -0.163146399 | 0.1066344 | 0.0406302 | 0.1693191 | DCUN1D4 | | 23142 |
| ENSG00000188993 | 11.59408641 | 0.016436643 | 0.1360602 | 0.4812544 | NA | LRRC66 | | 339977 |
| ENSG00000163069 | 821.8146262 | 0.089900485 | 0.0909621 | 0.2024835 | 0.4566924 | SGCB | | 6443 |
| ENSG00000109189 | 366.6404103 | -0.098595554 | 0.1184216 | 0.1976195 | 0.4511225 | USP46 | | 64854 |
| ENSG00000248866 | 12.74484494 | -0.012344536 | 0.1351032 | 0.6183723 | NA | USP46-DT | | 643783 |
| ENSG00000226950 | 217.5151554 | -0.170911802 | 0.1804501 | 0.0604295 | 0.2189445 | DANCR | | 57291 |
| ENSG00000184178 | 279.1429274 | 0.030014973 | 0.1085673 | 0.6591527 | 0.8425803 | SCFD2 | | 152579 |
| ENSG00000145216 | 786.702392 | -0.046492274 | 0.0861473 | 0.4890146 | 0.7262901 | FIP1L1 | | 81608 |
| ENSG00000072201 | 11.04824094 | 0.027292223 | 0.1386352 | 0.245652 | NA | LNX1 | | 84708 |
| ENSG00000109220 | 204.1970387 | 0.160336714 | 0.1725292 | 0.0707318 | 0.2420798 | CHIC2 | | 26511 |
| ENSG00000128039 | 70.45785371 | -0.010879778 | 0.1261468 | 0.8243669 | 0.9276013 | SRD5A3 | | 79644 |
| ENSG00000134851 | 1076.79169 | -0.146784603 | 0.094298 | 0.0473734 | 0.1859327 | TMEM165 | | 55858 |
| ENSG00000134852 | 642.193754 | -0.061197283 | 0.0970609 | 0.3870106 | 0.6475138 | CLOCK | | 9575 |
| ENSG00000109255 | 41.96521006 | 0.0151378 | 0.1306193 | 0.7170893 | 0.8718976 | NMU | | 10874 |
| ENSG00000090989 | 384.8506134 | 0.066591049 | 0.1075325 | 0.3576901 | 0.6209582 | EXOC1 | | 55763 |
| ENSG00000174799 | 708.7594306 | -0.014146171 | 0.0887299 | 0.8332961 | 0.9305394 | CEP135 | | 9662 |
| ENSG00000109265 | 163.3908208 | 0.096512459 | 0.1437574 | 0.1847297 | 0.4349649 | CRACD | | 57482 |
| ENSG00000157426 | 137.8646633 | 0.057710488 | 0.1283806 | 0.3707896 | 0.6330303 | AASDH | | 132949 |
| ENSG00000128059 | 1450.775972 | -0.017582287 | 0.0731054 | 0.7747898 | 0.905032 | PPAT | | 5471 |
| ENSG00000128050 | 5536.987057 | -0.142254839 | 0.0610275 | 0.0095026 | 0.0615983 | PAICS | | 10606 |
| ENSG00000270109 | 12.14950153 | 0.002200108 | 0.1346443 | 0.9271945 | NA | NA | NA | |
| ENSG00000174780 | 2476.694693 | -0.155657723 | 0.0739251 | 0.0143573 | 0.0818271 | SRP72 | | 6731 |
| ENSG00000084093 | 959.2301493 | -0.170437191 | 0.0942781 | 0.0231762 | 0.1148095 | REST | | 5978 |
| ENSG00000084092 | 441.6399516 | 0.068881163 | 0.1032343 | 0.3488947 | 0.6148358 | NOA1 | | 84273 |
| ENSG00000047315 | 2627.203312 | -0.293635546 | 0.0711266 | 5.93E-06 | 0.0001608 | POLR2B | | 5431 |
| ENSG00000163453 | 3111.838361 | -0.098437681 | 0.0659254 | 0.0882159 | 0.2795576 | IGFBP7 | | 3490 |
| ENSG00000245067 | 34.58109943 | -0.040706228 | 0.1395027 | 0.2823971 | NA | IGFBP7-AS1 | | 255130 |
| ENSG00000243199 | 80.20759859 | -0.050484352 | 0.1357557 | 0.3446724 | 0.610988 | RPL6P10 | | 642828 |
| ENSG00000145241 | 341.2535815 | -0.103755117 | 0.122532 | 0.1805049 | 0.4299157 | CENPC | | 1060 |
| ENSG00000033178 | 1568.96294 | -0.253828513 | 0.083354 | 0.0004465 | 0.0058794 | UBA6 | | 55236 |
| ENSG00000248049 | 126.9858629 | 0.073924791 | 0.1375002 | 0.2610662 | 0.5284205 | UBA6-DT | | 550112 |
| ENSG00000083896 | 2218.134623 | 0.008897444 | 0.0641548 | 0.8760699 | 0.9497329 | YTHDC1 | | 91746 |
| ENSG00000171234 | 129.2025333 | -0.224632417 | 0.2592673 | 0.030982 | 0.141202 | UGT2B7 | | 7364 |
| ENSG00000248763 | 11.88070984 | -0.015878541 | 0.1358559 | 0.5030491 | NA | NA | NA | |
| ENSG00000132467 | 792.219618 | 0.03594945 | 0.0843435 | 0.5905599 | 0.8001113 | UTP3 | | 57050 |
| ENSG00000018189 | 220.0683246 | -0.033740101 | 0.1140539 | 0.6088996 | 0.8123066 | RUFY3 | | 22902 |
| ENSG00000132463 | 1676.213856 | -0.166397229 | 0.0781035 | 0.0121624 | 0.073037 | GRSF1 | | 2926 |
| ENSG00000173542 | 604.5294524 | -0.096321011 | 0.0999618 | 0.190463 | 0.4412794 | MOB1B | | 92597 |
| ENSG00000156136 | 687.8410236 | 0.075691543 | 0.0953745 | 0.2888021 | 0.5587111 | DCK | | 1633 |
| ENSG00000163626 | 162.5161428 | -0.012040519 | 0.1147007 | 0.8468842 | 0.9372292 | COX18 | | 285521 |
| ENSG00000132466 | 2493.626817 | -0.080884734 | 0.0686259 | 0.1731293 | 0.4203584 | ANKRD17 | | 26057 |
| ENSG00000169429 | 2628.469398 | 1.019145954 | 0.0706833 | 2.40E-48 | 4.00E-45 | CXCL8 | | 3576 |
| ENSG00000124875 | 126.7041545 | -0.040882033 | 0.1241116 | 0.5041999 | 0.7381117 | CXCL6 | | 6372 |
| ENSG00000163739 | 5446.616494 | 0.511097379 | 0.0594781 | 7.38E-19 | 2.05E-16 | CXCL1 | | 2919 |
| ENSG00000163734 | 87.63211486 | 0.326351617 | 0.4308342 | 0.0147769 | 0.0833231 | CXCL3 | | 2921 |
| ENSG00000081041 | 550.2192183 | 0.586117791 | 0.1318104 | 5.79E-07 | 2.13E-05 | CXCL2 | | 2920 |
| ENSG00000163738 | 250.6652472 | -0.027567232 | 0.1097345 | 0.6801106 | 0.853781 | MTHFD2L | | 441024 |
| ENSG00000124882 | 2333.291214 | 0.255293268 | 0.1646479 | 0.0153229 | 0.0854762 | EREG | | 2069 |
| ENSG00000109321 | 22.28842735 | 0.032685388 | 0.1382796 | 0.3113644 | NA | AREG | | 374 |
| ENSG00000205595 | 18.8613029 | 0.027842201 | 0.1372909 | 0.3566139 | NA | NA | NA | |
| ENSG00000174808 | 24.64231682 | 0.021301159 | 0.1347572 | 0.5200694 | NA | BTC | | 685 |
| ENSG00000169116 | 12.14236347 | -0.01892253 | 0.1363432 | 0.4338414 | NA | PARM1 | | 25849 |
| ENSG00000163743 | 443.295817 | 0.031677509 | 0.0973552 | 0.64745 | 0.8362299 | RCHY1 | | 25898 |
| ENSG00000174796 | 182.0917361 | 0.100057271 | 0.1432269 | 0.1781375 | 0.4271872 | THAP6 | | 152815 |
| ENSG00000138769 | 25.43663153 | -0.009146553 | 0.132499 | 0.7857225 | NA | CDKL2 | | 8999 |
| ENSG00000138757 | 2818.616848 | -0.176110415 | 0.0680577 | 0.0034447 | 0.028376 | G3BP2 | | 9908 |
| ENSG00000138768 | 1308.543361 | -0.092253834 | 0.0790714 | 0.1578904 | 0.3976536 | USO1 | | 8615 |
| ENSG00000138744 | 373.8752512 | -0.028291432 | 0.1024393 | 0.6808724 | 0.8538613 | NAAA | | 27163 |
| ENSG00000198301 | 723.5851838 | -0.011820829 | 0.08561 | 0.8586557 | 0.9430101 | SDAD1 | | 55153 |
| ENSG00000138750 | 893.0730387 | -0.162978841 | 0.0981322 | 0.033036 | 0.1466249 | NUP54 | | 53371 |
| ENSG00000138760 | 1315.372503 | -0.126499818 | 0.0818618 | 0.0607764 | 0.2199285 | SCARB2 | | 950 |
| ENSG00000118804 | 17.9011081 | 0.00874111 | 0.1338457 | 0.7659296 | NA | STBD1 | | 8987 |
| ENSG00000138771 | 26.87109454 | -0.004035645 | 0.1323833 | 0.9011647 | NA | SHROOM3 | | 57619 |
| ENSG00000138758 | 3189.427527 | -0.159282014 | 0.0672252 | 0.0072022 | 0.0498391 | SEPTIN11 | | 55752 |
| ENSG00000118816 | 1890.981242 | -0.099937256 | 0.0759169 | 0.1170764 | 0.3339413 | CCNI | | 10983 |

| | | | | | | | | |
|---|---|---|---|---|---|---|---|---|
| ENSG00000138764 | 118.1365039 | -0.802903223 | 0.2839986 | 0.0001998 | 0.0030707 | CCNG2 | | 901 |
| ENSG00000138767 | 338.984276 | -0.148108445 | 0.1410679 | 0.083612 | 0.2700934 | CNOT6L | | 246175 |
| ENSG00000169288 | 272.2001619 | -0.375076048 | 0.1994425 | 0.0042245 | 0.0332526 | MRPL1 | | 65008 |
| ENSG00000138772 | 1334.321072 | -0.059104829 | 0.0799546 | 0.3602394 | 0.6236782 | ANXA3 | | 306 |
| ENSG00000138756 | 505.5275607 | 0.030809399 | 0.0986372 | 0.6554191 | 0.841036 | BMP2K | | 55589 |
| ENSG00000163291 | 720.3352044 | -0.065071417 | 0.0905009 | 0.3479832 | 0.6140832 | PAQR3 | | 152559 |
| ENSG00000163297 | 272.1032578 | 0.007271872 | 0.1096152 | 0.9130723 | 0.9656034 | ANTXR2 | | 118429 |
| ENSG00000152784 | 258.1268221 | 0.133814724 | 0.1543833 | 0.1056869 | 0.3130144 | PRDM8 | | 56978 |
| ENSG00000138675 | 56.34659325 | 0.733935508 | 0.4681519 | 0.0032638 | 0.0272904 | FGF5 | | 2250 |
| ENSG00000138669 | 13.06303959 | 0.022662555 | 0.1369784 | 0.3725136 | NA | PRKG2 | | 5593 |
| ENSG00000138668 | 6665.831196 | -0.277126789 | 0.060738 | 9.29E-07 | 3.18E-05 | HNRNPD | | 3184 |
| ENSG00000272677 | 11.1624963 | -0.001840246 | 0.134745 | 0.9325292 | NA | HNRNPD-DT | | 101928963 |
| ENSG00000152795 | 5871.84888 | -0.273923578 | 0.0603199 | 1.05E-06 | 3.54E-05 | HNRNPDL | | 9987 |
| ENSG00000145293 | 1168.953291 | 0.109248413 | 0.0829154 | 0.1073903 | 0.3167741 | ENOPH1 | | 58478 |
| ENSG00000249242 | 37.36314683 | 0.006273396 | 0.130549 | 0.8747519 | NA | TMEM150C | | 441027 |
| ENSG00000145284 | 341.6576456 | -0.116035635 | 0.1246209 | 0.1478299 | 0.3818666 | SCD5 | | 79966 |
| ENSG00000138674 | 1610.41332 | -0.006335078 | 0.0688685 | 0.9144204 | 0.9662935 | SEC31A | | 22872 |
| ENSG00000251022 | 552.7980136 | 0.551146862 | 0.1271624 | 1.02E-06 | 3.45E-05 | THAP9-AS1 | | 100499177 |
| ENSG00000168152 | 79.9094833 | 0.080766807 | 0.1515399 | 0.1749866 | 0.4232798 | THAP9 | | 79725 |
| ENSG00000189308 | 463.5086384 | 0.155659084 | 0.1240454 | 0.0645133 | 0.228798 | LIN54 | | 132660 |
| ENSG00000138663 | 529.3889721 | -0.123208208 | 0.111515 | 0.1154543 | 0.3309501 | COPS4 | | 51138 |
| ENSG00000173085 | 280.3802771 | -0.064772917 | 0.1147957 | 0.3595921 | 0.622742 | COQ2 | | 27235 |
| ENSG00000173083 | 153.4970568 | 0.068520301 | 0.1310157 | 0.3078232 | 0.5769089 | HPSE | | 10855 |
| ENSG00000163312 | 145.760001 | 0.071828733 | 0.1334654 | 0.2846751 | 0.5539446 | HELQ | | 113510 |
| ENSG00000163319 | 705.8531609 | 0.038215803 | 0.0867376 | 0.5719465 | 0.7865616 | MRPS18C | | 51023 |
| ENSG00000163322 | 167.2415647 | -0.014459579 | 0.1151359 | 0.8158216 | 0.9250254 | ABRAXAS1 | | 84142 |
| ENSG00000138678 | 107.4119745 | 0.025653836 | 0.1228723 | 0.6575662 | 0.8419413 | GPAT3 | | 84803 |
| ENSG00000163624 | 87.07751272 | 0.012364549 | 0.1237882 | 0.8189339 | 0.9256091 | CDS1 | | 1040 |
| ENSG00000163625 | 1152.868345 | -0.192600984 | 0.0950208 | 0.0117595 | 0.0713203 | WDFY3 | | 23001 |
| ENSG00000180769 | 22.18055637 | 0.006622345 | 0.1329501 | 0.8362329 | NA | WDFY3-AS2 | | 404201 |
| ENSG00000163629 | 547.2055319 | -0.147500036 | 0.1150678 | 0.0695994 | 0.2400304 | PTPN13 | | 5783 |
| ENSG00000163633 | 48.36184055 | 0.067524515 | 0.1514979 | 0.1449799 | 0.3774261 | C4orf36 | | 132989 |
| ENSG00000172493 | 982.3796001 | 0.007894053 | 0.0791119 | 0.9034497 | 0.9618565 | AFF1 | | 4299 |
| ENSG00000145332 | 562.0321151 | 0.051336055 | 0.0950494 | 0.4637167 | 0.7079196 | KLHL8 | | 57563 |
| ENSG00000198189 | 503.3808678 | -0.082151213 | 0.1041767 | 0.2628435 | 0.5303256 | HSD17B11 | | 51170 |
| ENSG00000170502 | 361.8441033 | -0.021317334 | 0.101503 | 0.7550033 | 0.8944415 | NUDT9 | | 53343 |
| ENSG00000118762 | 460.7358844 | 0.173136067 | 0.1303715 | 0.0479281 | 0.1871776 | PKD2 | | 5311 |
| ENSG00000163644 | 117.1242387 | -0.01708394 | 0.1231626 | 0.7553294 | 0.8945557 | PPM1K | | 152926 |
| ENSG00000138642 | 175.3304109 | -0.037742296 | 0.1186612 | 0.5561928 | 0.7757478 | HERC6 | | 55008 |
| ENSG00000138646 | 418.3103898 | 0.095504673 | 0.1152655 | 0.2090149 | 0.4654027 | HERC5 | | 51191 |
| ENSG00000145337 | 597.0064027 | -0.0260001 | 0.0913574 | 0.7023787 | 0.8650963 | PYURF | | 100996939 |
| ENSG00000138641 | 210.7961294 | 0.019979608 | 0.1122276 | 0.7601161 | 0.8970414 | HERC3 | | 8916 |
| ENSG00000177432 | 59.72904846 | 0.02740258 | 0.1300836 | 0.5712683 | 0.7864095 | NAP1L5 | | 266812 |
| ENSG00000248019 | 15.64423321 | 0.001264969 | 0.1338107 | 0.9652501 | NA | FAM13A-AS | | 285512 |
| ENSG00000138640 | 60.21905731 | 0.086613828 | 0.1642274 | 0.0981115 | 0.2986813 | FAM13A | | 10144 |
| ENSG00000271359 | 11.59702261 | -0.007777169 | 0.1350395 | 0.7289591 | NA | NA | NA | |
| ENSG00000180346 | 45.04733804 | 0.020994109 | 0.1312001 | 0.6240627 | 0.8201707 | TIGD2 | | 166815 |
| ENSG00000145335 | 67.75173832 | -0.046448316 | 0.1348944 | 0.3703151 | 0.6328256 | SNCA | | 6622 |
| ENSG00000184305 | 9.552667303 | -0.006723989 | 0.1351257 | 0.7540588 | NA | CCSER1 | | 401145 |
| ENSG00000187653 | 76.90164434 | -0.076747614 | 0.1517343 | 0.1682849 | 0.413752 | NA | NA | |
| ENSG00000163104 | 971.255126 | -0.022196975 | 0.0810888 | 0.7333837 | 0.8821599 | SMARCAD1 | | 56916 |
| ENSG00000163110 | 926.6540039 | -0.043306385 | 0.0861874 | 0.5194895 | 0.7481392 | PDLIM5 | | 10611 |
| ENSG00000138696 | 54.56180545 | 0.010535095 | 0.128155 | 0.8199478 | 0.9259577 | BMPR1B | | 658 |
| ENSG00000138698 | 833.7771666 | -0.056117617 | 0.0854773 | 0.3999717 | 0.6583091 | RAP1GDS1 | | 5910 |
| ENSG00000168785 | 621.7221272 | 0.183455231 | 0.1140036 | 0.0288669 | 0.1348777 | TSPAN5 | | 10098 |
| ENSG00000151247 | 1191.169485 | -0.208739326 | 0.0894255 | 0.0048634 | 0.0372762 | EIF4E | | 1977 |
| ENSG00000164024 | 1645.705639 | -0.174414689 | 0.0784207 | 0.0089949 | 0.0592609 | METAP1 | | 23173 |
| ENSG00000197894 | 2012.696107 | -0.353398899 | 0.0818524 | 1.91E-06 | 5.96E-05 | ADH5 | | 128 |
| ENSG00000246090 | 26.65788037 | 0.026501184 | 0.1355305 | 0.4498389 | NA | LOC1005070 | | 100507053 |
| ENSG00000145331 | 64.53847333 | -0.094569978 | 0.1684787 | 0.0969408 | 0.2961206 | TRMT10A | | 93587 |
| ENSG00000109270 | 392.4079373 | -0.025039224 | 0.1000752 | 0.7116924 | 0.8693171 | LAMTOR3 | | 8649 |
| ENSG00000164031 | 1025.666345 | -0.200466526 | 0.0938837 | 0.0085724 | 0.0570243 | DNAJB14 | | 79982 |
| ENSG00000164032 | 10444.50494 | -0.124274136 | 0.0568589 | 0.0162002 | 0.0888425 | H2AZ1 | | 3015 |
| ENSG00000245322 | 77.73476071 | 0.021345639 | 0.1293672 | 0.6500704 | 0.8372075 | NA | NA | |
| ENSG00000138814 | 624.4290372 | 0.045752622 | 0.0913263 | 0.5293511 | 0.7554508 | PPP3CA | | 5530 |
| ENSG00000254531 | 21.22644115 | 0.043136709 | 0.1427027 | 0.1767325 | NA | FLJ20021 | | 90024 |
| ENSG00000153064 | 11.49542118 | -0.022788241 | 0.1373191 | 0.3371305 | NA | BANK1 | | 55024 |
| ENSG00000138821 | 342.3502018 | 0.419043982 | 0.1597325 | 0.0006689 | 0.0081053 | SLC39A8 | | 64116 |
| ENSG00000109320 | 1456.446358 | -0.114974786 | 0.0780475 | 0.0781883 | 0.2579991 | NFKB1 | | 4790 |
| ENSG00000109323 | 180.2851922 | 0.029819724 | 0.115407 | 0.6461293 | 0.835448 | MANBA | | 4126 |

| | | | | | | | |
|---|---|---|---|---|---|---|---|
| ENSG00000230069 | 8.542019677 | 0.013171621 | 0.1360509 | 0.5176674 | NA | LRRC37A15 | 106481796 |
| ENSG00000248971 | 10.37777971 | -0.000961695 | 0.1347853 | 0.9626515 | NA | KRT8P46 | 100418744 |
| ENSG00000109332 | 3808.873787 | -0.045070102 | 0.0580212 | 0.3898618 | 0.6502297 | UBE2D3 | 7323 |
| ENSG00000246560 | 16.54321377 | -0.007443242 | 0.1339231 | 0.7880421 | NA | NA | NA |
| ENSG00000145354 | 892.2621321 | -0.128581867 | 0.0925293 | 0.0760881 | 0.2537974 | CISD2 | 493856 |
| ENSG00000164037 | 21.98403268 | 0.01299109 | 0.1337894 | 0.6770948 | NA | SLC9B1 | 150159 |
| ENSG00000164038 | 309.7169526 | 0.169904393 | 0.1541252 | 0.0603439 | 0.2188381 | SLC9B2 | 133308 |
| ENSG00000164039 | 16.39606842 | -0.017693781 | 0.1356586 | 0.5008615 | NA | BDH2 | 56898 |
| ENSG00000138778 | 1495.742779 | -0.288529535 | 0.0840848 | 9.54E-05 | 0.0016759 | CENPE | 1062 |
| ENSG00000248740 | 15.52434114 | 0.056321369 | 0.151214 | 0.0484012 | NA | NA | NA |
| ENSG00000168769 | 351.7002787 | -0.257724029 | 0.1606584 | 0.0138488 | 0.0801227 | TET2 | 54790 |
| ENSG00000138777 | 419.4523402 | -0.197429611 | 0.1414328 | 0.0329432 | 0.1464825 | PPA2 | 27068 |
| ENSG00000138785 | 318.3657185 | -0.017728742 | 0.1029916 | 0.7937019 | 0.913934 | INTS12 | 57117 |
| ENSG00000138780 | 501.0468837 | 0.022379642 | 0.0953814 | 0.7455095 | 0.8893165 | GSTCD | 79807 |
| ENSG00000168743 | 1220.500622 | -0.141046769 | 0.0935065 | 0.0546928 | 0.2048666 | NPNT | 255743 |
| ENSG00000145348 | 94.29431347 | 0.049914939 | 0.1319478 | 0.3879756 | 0.6484257 | TBCK | 93627 |
| ENSG00000164022 | 909.0303587 | 0.08333725 | 0.0871299 | 0.2277082 | 0.4888067 | AIMP1 | 9255 |
| ENSG00000138801 | 1040.452195 | -0.182114938 | 0.0937453 | 0.0156299 | 0.086774 | PAPSS1 | 9061 |
| ENSG00000164023 | 437.4785807 | 0.067662442 | 0.1030563 | 0.3536804 | 0.6187516 | SGMS2 | 166929 |
| ENSG00000155016 | 55.79387745 | 0.032967595 | 0.1326389 | 0.4806169 | 0.7196624 | CYP2U1 | 113612 |
| ENSG00000138796 | 319.4045535 | -0.065799988 | 0.1129431 | 0.3590565 | 0.6222758 | HADH | 3033 |
| ENSG00000138795 | 70.35220452 | 0.020022359 | 0.127081 | 0.692496 | 0.8594028 | LEF1 | 51176 |
| ENSG00000109475 | 3089.109596 | -0.206888688 | 0.07032 | 0.0009082 | 0.0102599 | RPL34 | 6164 |
| ENSG00000198856 | 542.0111924 | -0.065165769 | 0.0987363 | 0.3604195 | 0.623805 | OSTC | 58505 |
| ENSG00000138802 | 593.1079314 | -0.13203955 | 0.1075608 | 0.0902687 | 0.283659 | SEC24B | 10427 |
| ENSG00000005059 | 623.3666726 | -0.149327202 | 0.109603 | 0.0612264 | 0.2207364 | MCUB | 55013 |
| ENSG00000138794 | 150.8699801 | -0.315473504 | 0.2616101 | 0.0140706 | 0.0809085 | CASP6 | 839 |
| ENSG00000123739 | 398.0649249 | -0.070088399 | 0.1097928 | 0.3341526 | 0.6017014 | PLA2G12A | 81579 |
| ENSG00000109534 | 997.3426675 | -0.00194977 | 0.0788627 | 0.9750218 | 0.9905401 | GAR1 | 54433 |
| ENSG00000170522 | 63.98457679 | 0.003102777 | 0.126314 | 0.9495471 | 0.9800467 | ELOVL6 | 79071 |
| ENSG00000138792 | 21.97050162 | 0.027718838 | 0.1366116 | 0.392809 | NA | ENPEP | 2028 |
| ENSG00000174749 | 272.710076 | 0.11527114 | 0.13958 | 0.1458147 | 0.3783345 | FAM241A | 132720 |
| ENSG00000138660 | 869.7605617 | -0.022801943 | 0.0828585 | 0.7290524 | 0.8790651 | AP1AR | 55435 |
| ENSG00000145365 | 585.9558967 | -0.158398809 | 0.1138229 | 0.0527165 | 0.1995404 | TIFA | 92610 |
| ENSG00000073331 | 23.03914975 | 0.011889346 | 0.1331903 | 0.7180093 | NA | ALPK1 | 80216 |
| ENSG00000138658 | 366.1302899 | 0.048866482 | 0.1048012 | 0.4580382 | 0.7023667 | ZGRF1 | 55345 |
| ENSG00000174720 | 591.2184409 | -0.234905874 | 0.1244315 | 0.0103953 | 0.0652137 | LARP7 | 51574 |
| ENSG00000249532 | 14.78243472 | -0.006729674 | 0.134348 | 0.7919641 | NA | MIR302CHG | 109864269 |
| ENSG00000145362 | 101.2735507 | 0.145743254 | 0.2074771 | 0.0609865 | 0.2202793 | ANK2 | 287 |
| ENSG00000196656 | 328.7084874 | -0.026986559 | 0.1026766 | 0.6937949 | 0.8600779 | NA | NA |
| ENSG00000145349 | 234.6481544 | 0.662452618 | 0.1934504 | 3.40E-05 | 0.000713 | CAMK2D | 817 |
| ENSG00000180801 | 740.5328479 | -0.150487208 | 0.1046376 | 0.0544185 | 0.2041306 | ARSJ | 79642 |
| ENSG00000174607 | 611.8964751 | -0.078410689 | 0.0967718 | 0.2748227 | 0.5431817 | UGT8 | 7368 |
| ENSG00000174599 | 16.33514034 | 0.021737295 | 0.1362013 | 0.4361039 | NA | TRAM1L1 | 133022 |
| ENSG00000269893 | 542.3326214 | 0.234975905 | 0.129232 | 0.0119448 | 0.0721074 | SNHG8 | 100093630 |
| ENSG00000164099 | 72.82991037 | -0.033592966 | 0.1303164 | 0.5085161 | 0.7401841 | PRSS12 | 8492 |
| ENSG00000154608 | 16.02164353 | 0.012185729 | 0.1348767 | 0.640351 | NA | NA | NA |
| ENSG00000260404 | 91.24136961 | 0.021919888 | 0.124433 | 0.6905122 | 0.8589854 | NA | NA |
| ENSG00000225892 | 29.79751287 | 0.059790094 | 0.1498039 | 0.1240594 | NA | NA | NA |
| ENSG00000145388 | 315.7643369 | 0.038266426 | 0.1087038 | 0.5798493 | 0.7922857 | METTL14 | 57721 |
| ENSG00000150961 | 366.0591629 | 0.033236943 | 0.1011816 | 0.6325153 | 0.8251856 | SEC24D | 9871 |
| ENSG00000145390 | 617.4188812 | 0.237907292 | 0.1382232 | 0.0137503 | 0.0797107 | USP53 | 54532 |
| ENSG00000164096 | 627.7620343 | -0.131196607 | 0.1106357 | 0.0954853 | 0.2936714 | C4orf3 | 401152 |
| ENSG00000248280 | 10.729691 | 0.004662893 | 0.1349993 | 0.8319472 | NA | NA | NA |
| ENSG00000245958 | 117.6324544 | -0.058567183 | 0.1321961 | 0.3422481 | 0.6088638 | NA | NA |
| ENSG00000138735 | 14.17166529 | -0.027161667 | 0.138204 | 0.2829628 | NA | PDE5A | 8654 |
| ENSG00000164109 | 1767.641731 | -0.049206409 | 0.0694194 | 0.4098074 | 0.6648557 | MAD2L1 | 4085 |
| ENSG00000138738 | 66.40452766 | 0.054997075 | 0.1393475 | 0.2911563 | 0.5609429 | PRDM5 | 11107 |
| ENSG00000164111 | 5172.479755 | 0.025359308 | 0.0561532 | 0.6186364 | 0.8176387 | ANXA5 | 308 |
| ENSG00000123737 | 835.8141779 | -0.021564792 | 0.0858586 | 0.7384159 | 0.885525 | EXOSC9 | 5393 |
| ENSG00000145386 | 1471.040611 | -0.346600728 | 0.0848493 | 5.41E-06 | 0.0001484 | CCNA2 | 890 |
| ENSG00000138686 | 399.7432558 | -0.043425155 | 0.1037283 | 0.5338089 | 0.7585671 | BBS7 | 55212 |
| ENSG00000138688 | 1229.481487 | -0.244416679 | 0.0919219 | 0.001538 | 0.0156354 | BLTP1 | 84162 |
| ENSG00000181004 | 39.18086791 | 0.040396228 | 0.1383401 | 0.3199203 | NA | BBS12 | 166379 |
| ENSG00000138685 | 874.4469149 | 0.186806513 | 0.1079556 | 0.0225206 | 0.1127576 | FGF2 | 2247 |
| ENSG00000170917 | 64.65104215 | -0.037872081 | 0.1322393 | 0.4510211 | 0.6977059 | NUDT6 | 11162 |
| ENSG00000145375 | 299.526465 | 0.125081442 | 0.1349311 | 0.1246723 | 0.3465814 | AFG2A | 166378 |
| ENSG00000164056 | 8.662197908 | 0.031652944 | 0.140671 | 0.116757 | NA | SPRY1 | 10252 |
| ENSG00000151458 | 1560.522649 | -0.037029019 | 0.0727383 | 0.5473718 | 0.7690111 | ANKRD50 | 57182 |
| ENSG00000196159 | 314.3034977 | -0.243469429 | 0.1634704 | 0.0184007 | 0.0978625 | FAT4 | 79633 |

| | | | | | | | |
|---|---|---|---|---|---|---|---|
| ENSG00000164066 | 167.5580482 | 0.14968675 | 0.1800464 | 0.0774425 | 0.2565722 | INTU | 27152 |
| ENSG00000164070 | 695.0220966 | -0.0523243 | 0.0907345 | 0.4480084 | 0.6957175 | HSPA4L | 22824 |
| ENSG00000142731 | 1064.151419 | -0.252279242 | 0.0956812 | 0.0015459 | 0.0156755 | PLK4 | 10733 |
| ENSG00000164073 | 278.0959477 | 0.018950955 | 0.1054002 | 0.7794065 | 0.9078735 | MFSD8 | 256471 |
| ENSG00000164074 | 213.652446 | 0.045033312 | 0.1169169 | 0.502852 | 0.7371755 | ABHD18 | 80167 |
| ENSG00000138709 | 540.5744394 | -0.001724352 | 0.0904603 | 0.9788677 | 0.9921174 | LARP1B | 55132 |
| ENSG00000164040 | 1154.666589 | 0.019955616 | 0.0778778 | 0.7561671 | 0.8950034 | PGRMC2 | 10424 |
| ENSG00000251432 | 26.86084634 | -0.047377604 | 0.1437533 | 0.1780824 | NA | LINC02615 | 100507487 |
| ENSG00000077684 | 1150.765925 | -0.21208487 | 0.0935205 | 0.0056458 | 0.0414338 | JADE1 | 79960 |
| ENSG00000151466 | 469.3806086 | -0.066843632 | 0.1032012 | 0.3527797 | 0.6185235 | SCLT1 | 132320 |
| ENSG00000151470 | 82.5871364 | -0.046578023 | 0.1326734 | 0.4113645 | 0.6661689 | C4orf33 | 132321 |
| ENSG00000250102 | 25.28112047 | 0.022301876 | 0.1350235 | 0.4992268 | NA | LINC02377 | 105377424 |
| ENSG00000254535 | 49.29880137 | 0.005097434 | 0.1285024 | 0.9097473 | 0.9644906 | PABPC4L | 132430 |
| ENSG00000151012 | 733.9874195 | 0.518138325 | 0.1107869 | 2.23E-07 | 9.32E-06 | SLC7A11 | 23657 |
| ENSG00000151014 | 306.4446128 | -0.013078104 | 0.1033914 | 0.846114 | 0.937176 | NOCT | 25819 |
| ENSG00000109381 | 459.2833431 | -0.118368419 | 0.1139124 | 0.1300786 | 0.3544317 | ELF2 | 1998 |
| ENSG00000109390 | 580.2608352 | -0.177064261 | 0.1261628 | 0.0411734 | 0.1705483 | NDUFC1 | 4717 |
| ENSG00000164134 | 2139.093565 | -0.155745984 | 0.072158 | 0.0127226 | 0.0753794 | NAA15 | 80155 |
| ENSG00000273247 | 27.62612624 | -0.043177277 | 0.1412048 | 0.235404 | NA | NA | NA |
| ENSG00000172007 | 162.4542684 | 0.057907239 | 0.1249369 | 0.3849495 | 0.6453768 | RAB33B | 83452 |
| ENSG00000145391 | 1712.906208 | -0.070335443 | 0.0712766 | 0.2481215 | 0.5144316 | SETD7 | 80854 |
| ENSG00000085871 | 9.818749852 | -0.011655358 | 0.1355818 | 0.5921408 | NA | MGST2 | 4258 |
| ENSG00000178458 | 86.417977 | -0.041210015 | 0.1301131 | 0.4517627 | 0.6981568 | NA | NA |
| ENSG00000196782 | 19.13032165 | -0.001914281 | 0.1334928 | 0.9445318 | NA | MAML3 | 55534 |
| ENSG00000153130 | 754.2316722 | -0.044648157 | 0.0859998 | 0.5045863 | 0.7381431 | SCOC | 60592 |
| ENSG00000196951 | 8.65134151 | -0.013943784 | 0.1361308 | 0.4942587 | NA | SCOC-AS1 | 100129858 |
| ENSG00000153132 | 247.7535283 | -0.038463184 | 0.1116362 | 0.584215 | 0.7955327 | CLGN | 1047 |
| ENSG00000179387 | 305.8096943 | -0.245760283 | 0.1685052 | 0.0186984 | 0.099145 | ELMOD2 | 255520 |
| ENSG00000109436 | 424.6155907 | 0.010025366 | 0.096086 | 0.8842987 | 0.953345 | TBC1D9 | 23158 |
| ENSG00000170153 | 42.26154383 | -0.024928657 | 0.1325045 | 0.5496614 | 0.7710573 | RNF150 | 57484 |
| ENSG00000109445 | 607.465806 | 0.512638783 | 0.137333 | 1.35E-05 | 0.0003255 | ZNF330 | 27309 |
| ENSG00000164136 | 64.70488265 | -0.006707279 | 0.126129 | 0.8893067 | 0.9549168 | IL15 | 3600 |
| ENSG00000109452 | 53.70870068 | 0.114079802 | 0.1944512 | 0.0474024 | 0.1859327 | INPP4B | 8821 |
| ENSG00000170185 | 455.0682079 | 0.037814983 | 0.1027139 | 0.5859269 | 0.7968554 | USP38 | 84640 |
| ENSG00000109458 | 190.1870246 | 0.017553718 | 0.1125218 | 0.7876002 | 0.9116145 | GAB1 | 2549 |
| ENSG00000153147 | 2553.155007 | -0.23603767 | 0.0707502 | 0.0001948 | 0.0030053 | SMARCA5 | 8467 |
| ENSG00000164162 | 139.3798834 | 0.007403004 | 0.1170216 | 0.9042045 | 0.9622177 | ANAPC10 | 10393 |
| ENSG00000164163 | 2882.491502 | -0.058602815 | 0.0645168 | 0.3060672 | 0.5752194 | ABCE1 | 6059 |
| ENSG00000164164 | 1747.017968 | -0.042525873 | 0.0708686 | 0.4826957 | 0.721388 | OTUD4 | 54726 |
| ENSG00000170365 | 360.4882199 | -0.19136542 | 0.1444305 | 0.0385027 | 0.1634859 | SMAD1 | 4086 |
| ENSG00000151611 | 46.83996466 | 0.005363661 | 0.1291141 | 0.9018733 | 0.9616276 | MMAA | 166785 |
| ENSG00000151612 | 588.0522369 | 0.007990349 | 0.0894583 | 0.9069825 | 0.963256 | ZNF827 | 152485 |
| ENSG00000164167 | 225.2076319 | -0.056682314 | 0.1177351 | 0.4125051 | 0.6665476 | LSM6 | 11157 |
| ENSG00000120519 | 58.72657151 | -0.020811101 | 0.1292359 | 0.6549218 | 0.8405825 | SLC10A7 | 84068 |
| ENSG00000164168 | 478.7289232 | -0.082728311 | 0.1036726 | 0.2597956 | 0.5268529 | TMEM184C | 55751 |
| ENSG00000164169 | 148.0111602 | 0.037700706 | 0.1205346 | 0.5517571 | 0.7725183 | PRMT9 | 90826 |
| ENSG00000071205 | 242.7053186 | 0.007626719 | 0.1075806 | 0.9124786 | 0.9655189 | ARHGAP10 | 79658 |
| ENSG00000151623 | 11.72569477 | -0.001446817 | 0.1344964 | 0.9493872 | NA | NR3C2 | 4306 |
| ENSG00000170390 | 83.08770302 | 0.024975662 | 0.1261799 | 0.6415036 | 0.8316176 | DCLK2 | 166614 |
| ENSG00000198589 | 886.826441 | 0.091182636 | 0.0880021 | 0.1899106 | 0.4407602 | LRBA | 987 |
| ENSG00000145425 | 13434.89415 | -0.185067701 | 0.0580064 | 0.0004122 | 0.0055347 | RPS3A | 6189 |
| ENSG00000109686 | 988.4222073 | -0.084686757 | 0.0909963 | 0.2276907 | 0.4888067 | SH3D19 | 152503 |
| ENSG00000251611 | 9.12551473 | -0.006863069 | 0.1352515 | 0.7417982 | NA | NA | NA |
| ENSG00000164142 | 101.2324751 | 0.03780533 | 0.1290278 | 0.4924838 | 0.7283747 | FHIP1A | 729830 |
| ENSG00000251603 | 14.39012761 | -0.019456656 | 0.1361219 | 0.4502538 | NA | NA | NA |
| ENSG00000059691 | 227.0317977 | 0.023026504 | 0.1100168 | 0.7307101 | 0.8802394 | GATB | 5188 |
| ENSG00000109670 | 289.2308515 | -0.001581749 | 0.1042503 | 0.9801481 | 0.99264 | FBXW7 | 55294 |
| ENSG00000268471 | 49.31604214 | -0.003628976 | 0.128593 | 0.9310189 | 0.973088 | MIR4453HG | 54553 |
| ENSG00000170006 | 40.36846184 | 0.030803098 | 0.1342291 | 0.4617933 | NA | TMEM154 | 201799 |
| ENSG00000164144 | 504.781123 | -0.022657095 | 0.0936588 | 0.7397642 | 0.8860029 | ARFIP1 | 27236 |
| ENSG00000137460 | 74.75885685 | 0.035854156 | 0.1299453 | 0.4975178 | 0.7324811 | FHDC1 | 85462 |
| ENSG00000109654 | 270.6098804 | -0.015368068 | 0.1061329 | 0.804639 | 0.9186624 | TRIM2 | 23321 |
| ENSG00000121211 | 238.0017403 | 0.031981508 | 0.1116235 | 0.6339978 | 0.8257229 | MND1 | 84057 |
| ENSG00000121210 | 227.258068 | 0.061327271 | 0.1197566 | 0.3797503 | 0.6409076 | TMEM131L | 23240 |
| ENSG00000137462 | 238.0254801 | 0.122134877 | 0.1428411 | 0.1306357 | 0.3553692 | TLR2 | 7097 |
| ENSG00000145428 | 21.28947015 | 0.023565826 | 0.1358162 | 0.4495359 | NA | RNF175 | 285533 |
| ENSG00000171566 | 1113.830975 | -0.161912694 | 0.0977294 | 0.0336929 | 0.1487403 | PLRG1 | 5356 |
| ENSG00000164114 | 216.9244469 | 0.00662075 | 0.1097554 | 0.9203478 | 0.9692195 | MAP9 | 79884 |
| ENSG00000256043 | 18.10283424 | 0.02554785 | 0.1372002 | 0.3567892 | NA | CTSO | 1519 |
| ENSG00000145431 | 655.7308982 | -0.312776718 | 0.1211284 | 0.0011932 | 0.0128716 | PDGFC | 56034 |

| | | | | | | | |
|---|---|---|---|---|---|---|---|
| ENSG00000109738 | 141.8630102 | -0.072001005 | 0.1359815 | 0.2717852 | 0.5394882 | GLRB | 2743 |
| ENSG00000164124 | 94.09537682 | -0.069448346 | 0.1440657 | 0.2277276 | 0.4888067 | TMEM144 | 55314 |
| ENSG00000205208 | 660.5058363 | -0.009355586 | 0.0874002 | 0.8884107 | 0.9545517 | C4orf46 | 201725 |
| ENSG00000171503 | 184.4587583 | -0.059948394 | 0.1244068 | 0.3729422 | 0.6348496 | ETFDH | 2110 |
| ENSG00000171497 | 712.4724792 | -0.11885818 | 0.0989055 | 0.1103714 | 0.3220705 | PPID | 5481 |
| ENSG00000052795 | 325.9812265 | 0.257186573 | 0.1762959 | 0.017001 | 0.0921096 | FNIP2 | 57600 |
| ENSG00000109756 | 578.9671882 | -0.05382794 | 0.0965433 | 0.4429982 | 0.6917073 | RAPGEF2 | 9693 |
| ENSG00000145414 | 360.0590684 | 0.278420635 | 0.1617807 | 0.0098877 | 0.0631829 | NAF1 | 92345 |
| ENSG00000198498 | 478.0326853 | -0.068389734 | 0.1026161 | 0.3422111 | 0.6088638 | TMA16 | 55319 |
| ENSG00000273449 | 12.17904956 | -0.01609765 | 0.1357776 | 0.5082843 | NA | NA | NA |
| ENSG00000170088 | 398.0445258 | 0.139080211 | 0.1268969 | 0.0937913 | 0.2905267 | TMEM192 | 201931 |
| ENSG00000109466 | 396.54637 | 0.044043205 | 0.1031985 | 0.5301499 | 0.7558503 | KLHL2 | 11275 |
| ENSG00000052802 | 553.5441774 | -0.119119071 | 0.1792283 | 0.0907058 | 0.284609 | MSMO1 | 6307 |
| ENSG00000109472 | 30.16682906 | -0.05051449 | 0.1442079 | 0.1881182 | NA | CPE | 1363 |
| ENSG00000137628 | 68.34604049 | 0.007468891 | 0.1263633 | 0.8764035 | 0.949742 | DDX60 | 55601 |
| ENSG00000181381 | 151.4978162 | 0.078939293 | 0.1374096 | 0.2468754 | 0.5129849 | DDX60L | 91351 |
| ENSG00000129116 | 422.9229036 | 0.080565533 | 0.1090799 | 0.2759168 | 0.5441473 | PALLD | 23022 |
| ENSG00000145439 | 143.9326559 | 0.017149163 | 0.1171918 | 0.7816986 | 0.9094643 | CBR4 | 84869 |
| ENSG00000154447 | 319.4750164 | 0.448852802 | 0.1700497 | 0.0005781 | 0.0071824 | SH3RF1 | 57630 |
| ENSG00000137601 | 316.5399093 | 0.012110321 | 0.1027704 | 0.8581014 | 0.9428541 | NEK1 | 4750 |
| ENSG00000109572 | 968.1285856 | -0.106701596 | 0.0877726 | 0.1258416 | 0.3481656 | CLCN3 | 1182 |
| ENSG00000056050 | 277.9784492 | -0.04318017 | 0.1119188 | 0.5265934 | 0.7537604 | HPF1 | 54969 |
| ENSG00000198948 | 250.4855386 | -0.05209508 | 0.1146028 | 0.4514308 | 0.6978775 | MFAP3L | 9848 |
| ENSG00000109576 | 191.3624309 | 0.024286193 | 0.1136402 | 0.7097885 | 0.8682712 | AADAT | 51166 |
| ENSG00000245213 | 18.84543524 | 0.047544043 | 0.1453204 | 0.121891 | NA | GALNT7-DT | 101930370 |
| ENSG00000109586 | 746.1774647 | -0.176702274 | 0.1127778 | 0.0328367 | 0.1463432 | GALNT7 | 51809 |
| ENSG00000248774 | 12.93028518 | -0.01625128 | 0.1357425 | 0.5088469 | NA | NA | NA |
| ENSG00000164104 | 4359.4439 | -0.288685984 | 0.0631616 | 8.26E-07 | 2.88E-05 | HMGB2 | 3148 |
| ENSG00000272870 | 23.57366007 | 0.017382933 | 0.1340306 | 0.5974813 | NA | SAP30-DT | 105377540 |
| ENSG00000164105 | 219.401214 | -0.072879182 | 0.1246018 | 0.3034747 | 0.5727723 | SAP30 | 8819 |
| ENSG00000164117 | 122.5652624 | 0.258660297 | 0.2898429 | 0.0234698 | 0.1156754 | FBXO8 | 26269 |
| ENSG00000164118 | 223.1099579 | -0.05035795 | 0.1162891 | 0.4605756 | 0.7048321 | CEP44 | 80817 |
| ENSG00000248980 | 11.76965099 | -0.004980358 | 0.1345943 | 0.8334298 | NA | NA | NA |
| ENSG00000129128 | 3248.937163 | -0.310179184 | 0.067739 | 7.09E-07 | 2.57E-05 | SPCS3 | 60559 |
| ENSG00000150630 | 136.2651762 | 0.049562156 | 0.1269097 | 0.4257538 | 0.6782911 | VEGFC | 7424 |
| ENSG00000109674 | 351.5586588 | 0.097607037 | 0.1252859 | 0.202033 | 0.4562049 | NEIL3 | 55247 |
| ENSG00000038002 | 90.59520946 | 0.063543486 | 0.1390457 | 0.278823 | 0.5470157 | AGA | 175 |
| ENSG00000218336 | 31.03443821 | -0.013428453 | 0.132115 | 0.7117288 | NA | TENM3 | 55714 |
| ENSG00000129187 | 1298.31252 | -0.035683172 | 0.0749889 | 0.5692857 | 0.7847328 | DCTD | 1635 |
| ENSG00000251359 | 9.910744542 | 0.013604913 | 0.1357979 | 0.5417093 | NA | WWC2-AS2 | 152641 |
| ENSG00000151718 | 1780.579613 | -0.113123447 | 0.0771207 | 0.0800838 | 0.2623273 | WWC2 | 80014 |
| ENSG00000168564 | 1066.087221 | 0.016842315 | 0.0788938 | 0.7946246 | 0.9144629 | CDKN2AIP | 55602 |
| ENSG00000168556 | 252.7971535 | -0.006911467 | 0.1100168 | 0.9146205 | 0.9662935 | ING2 | 3622 |
| ENSG00000182552 | 408.7174291 | -0.017463412 | 0.1001163 | 0.7991272 | 0.9162772 | RWDD4 | 201965 |
| ENSG00000168538 | 440.8256504 | -0.090266447 | 0.1114776 | 0.2290036 | 0.4902363 | TRAPPC11 | 60684 |
| ENSG00000173320 | 41.405461 | 0.011119311 | 0.1301354 | 0.7897417 | 0.9117387 | STOX2 | 56977 |
| ENSG00000168310 | 248.9039514 | -0.078752611 | 0.1239088 | 0.2784311 | 0.5467586 | IRF2 | 3660 |
| ENSG00000271646 | 9.980175166 | -0.019132466 | 0.1368976 | 0.371407 | NA | NA | NA |
| ENSG00000164305 | 1263.687504 | -0.03626552 | 0.0757638 | 0.5653842 | 0.7820725 | CASP3 | 836 |
| ENSG00000164306 | 269.868988 | -0.002647432 | 0.1055671 | 0.9673463 | 0.9871985 | PRIMPOL | 201973 |
| ENSG00000151725 | 766.781028 | 0.784342414 | 0.1322677 | 1.76E-10 | 1.42E-08 | CENPU | 79682 |
| ENSG00000151726 | 923.3367795 | -0.050083434 | 0.0829443 | 0.4487641 | 0.6959957 | ACSL1 | 2180 |
| ENSG00000151729 | 468.5366625 | 0.114073073 | 0.1131068 | 0.1430252 | 0.374409 | SLC25A4 | 291 |
| ENSG00000164323 | 847.9571113 | -0.066588233 | 0.0866277 | 0.3280377 | 0.596112 | CFAP97 | 57587 |
| ENSG00000109762 | 279.1502384 | -0.204407648 | 0.1656428 | 0.0358284 | 0.1555225 | SNX25 | 83891 |
| ENSG00000186352 | 28.29612858 | -0.001840821 | 0.1317005 | 0.9562416 | NA | ANKRD37 | 353322 |
| ENSG00000109775 | 202.5881586 | -0.043857861 | 0.1164599 | 0.5100519 | 0.741578 | UFSP2 | 55325 |
| ENSG00000205129 | 9.600705774 | 0.021261188 | 0.1374191 | 0.318139 | NA | CFAP96 | 441054 |
| ENSG00000164342 | 10.47887653 | 0.012263137 | 0.1355535 | 0.5872911 | NA | TLR3 | 7098 |
| ENSG00000145476 | 70.45357027 | 0.060244619 | 0.1408874 | 0.2676 | 0.535818 | CYP4V2 | 285440 |
| ENSG00000083857 | 5044.98593 | 0.025637823 | 0.0598754 | 0.6340133 | 0.8257229 | FAT1 | 2195 |
| ENSG00000179046 | 1383.926598 | -0.083529142 | 0.07667 | 0.1923698 | 0.4436149 | TRIML2 | 205860 |
| ENSG00000248370 | 54.19330729 | 0.026819412 | 0.1309935 | 0.5622186 | 0.7800541 | LINC02434 | 105377606 |
| ENSG00000109536 | 394.1501197 | 0.042853151 | 0.1036718 | 0.5397131 | 0.7633355 | FRG1 | 2483 |
| ENSG00000249430 | 33.19475742 | -0.001347844 | 0.1315324 | 0.9649623 | NA | NA | NA |
| ENSG00000153404 | 82.68455675 | 0.010342088 | 0.1247453 | 0.8433018 | 0.9353791 | PLEKHG4B | 153478 |
| ENSG00000164366 | 687.6426825 | 0.008135788 | 0.0878714 | 0.9047685 | 0.962269 | CCDC127 | 133957 |
| ENSG00000260774 | 48.75087097 | 0.0064196 | 0.1288681 | 0.8843829 | 0.9533478 | NA | NA |
| ENSG00000073578 | 5001.812216 | -0.076255577 | 0.0584936 | 0.1498499 | 0.3852106 | SDHA | 6389 |
| ENSG00000249915 | 2243.298921 | -0.0664592 | 0.0670211 | 0.2563244 | 0.5231846 | PDCD6 | 10016 |

| | | | | | | | | |
|---|---|---|---|---|---|---|---|---|
| ENSG00000063438 | 153.6696777 | 0.282656529 | 0.2569396 | 0.0189021 | 0.0997748 | AHRR | | 57491 |
| ENSG00000221990 | 99.27033884 | 0.109272081 | 0.1688908 | 0.1120672 | 0.3248122 | NA | NA | |
| ENSG00000180104 | 1483.99247 | -0.099364164 | 0.0776503 | 0.1252562 | 0.347221 | EXOC3 | | 11336 |
| ENSG00000188242 | 533.6821929 | 0.17175805 | 0.1207517 | 0.0432707 | 0.176134 | SLC9A3-OT | | 25845 |
| ENSG00000225138 | 237.4212244 | 0.736903933 | 0.18686 | 4.41E-06 | 0.0001257 | NA | NA | |
| ENSG00000112877 | 775.4648567 | 0.038288449 | 0.0847472 | 0.5676133 | 0.7836298 | CEP72 | | 55722 |
| ENSG00000171368 | 82.50651872 | -0.012610594 | 0.1250136 | 0.8050353 | 0.9186624 | TPPP | | 11076 |
| ENSG00000268885 | 103.3587547 | 0.013270219 | 0.1222381 | 0.8115532 | 0.9223778 | NA | NA | |
| ENSG00000271781 | 111.6079553 | 0.037620489 | 0.1263617 | 0.5149435 | 0.7449955 | NA | NA | |
| ENSG00000188818 | 44.88367111 | 0.013665698 | 0.1297949 | 0.7516451 | 0.8926359 | ZDHHC11 | | 79844 |
| ENSG00000206077 | 9.722698772 | -0.005888915 | 0.1351446 | 0.7798536 | NA | ZDHHC11B | | 653082 |
| ENSG00000028310 | 2569.286005 | -0.075473769 | 0.0651884 | 0.1873615 | 0.4383122 | BRD9 | | 65980 |
| ENSG00000071539 | 2957.769904 | -0.050430537 | 0.0637981 | 0.3725347 | 0.6345468 | TRIP13 | | 9319 |
| ENSG00000145506 | 249.4342546 | 0.183256024 | 0.1691569 | 0.0509839 | 0.1946714 | NKD2 | | 85409 |
| ENSG00000113504 | 2166.476011 | -0.077555338 | 0.0686757 | 0.1921053 | 0.4433917 | SLC12A7 | | 10723 |
| ENSG00000164362 | 785.5791865 | 0.115171282 | 0.0955543 | 0.11638 | 0.3327869 | TERT | | 7015 |
| ENSG00000049656 | 6005.65997 | -0.059079142 | 0.0564328 | 0.24915 | 0.515466 | CLPTM1L | | 81037 |
| ENSG00000153395 | 5520.577311 | -0.141290978 | 0.0589259 | 0.0080503 | 0.0543556 | LPCAT1 | | 79888 |
| ENSG00000185986 | 127.0559177 | 0.056332577 | 0.129725 | 0.3708728 | 0.6330798 | NA | NA | |
| ENSG00000188002 | 303.9731012 | 0.205428171 | 0.1637427 | 0.0350282 | 0.1529016 | NA | NA | |
| ENSG00000171421 | 1207.073601 | 0.011772014 | 0.077814 | 0.8548323 | 0.9414385 | MRPL36 | | 64979 |
| ENSG00000145494 | 2192.595872 | -0.055342095 | 0.0667113 | 0.3431893 | 0.6097025 | NDUFS6 | | 4726 |
| ENSG00000170561 | 96.96255964 | 0.035791242 | 0.1268167 | 0.5296608 | 0.7555939 | IRX2 | | 153572 |
| ENSG00000186493 | 65.49081565 | -0.00695094 | 0.1267974 | 0.8821378 | 0.9525622 | NA | NA | |
| ENSG00000164151 | 3238.103327 | -0.12566355 | 0.0664566 | 0.0312901 | 0.1419966 | ICE1 | | 23379 |
| ENSG00000133398 | 1796.878626 | 0.340101038 | 0.0790167 | 2.18E-06 | 6.65E-05 | MED10 | | 84246 |
| ENSG00000037474 | 8059.903125 | -0.077990877 | 0.0543513 | 0.1166144 | 0.3332126 | NSUN2 | | 54888 |
| ENSG00000145545 | 912.9248664 | -0.021144333 | 0.0813623 | 0.7458539 | 0.8893165 | SRD5A1 | | 6715 |
| ENSG00000272520 | 307.9884779 | 0.026733168 | 0.1040274 | 0.6975212 | 0.8620332 | NA | NA | |
| ENSG00000112941 | 2038.131221 | -0.200585841 | 0.0761464 | 0.0024045 | 0.0217557 | TENT4A | | 11044 |
| ENSG00000124275 | 1585.741964 | 0.04424451 | 0.0710001 | 0.4667282 | 0.7094916 | MTRR | | 4552 |
| ENSG00000124279 | 530.9653878 | -0.01081043 | 0.0913619 | 0.8784609 | 0.9511774 | FASTKD3 | | 79072 |
| ENSG00000247516 | 174.5342778 | -0.137361021 | 0.169856 | 0.0929515 | 0.2886142 | MIR4458HG | | 100505738 |
| ENSG00000150756 | 627.6301113 | -0.14634219 | 0.1116718 | 0.0682023 | 0.2365412 | ATPSCKMT | | 134145 |
| ENSG00000150753 | 19982.20411 | -0.269826865 | 0.0508496 | 2.13E-08 | 1.20E-06 | CCT5 | | 22948 |
| ENSG00000271980 | 140.2781756 | -0.292648597 | 0.2819637 | 0.0179077 | 0.09591 | NA | NA | |
| ENSG00000164237 | 600.2875513 | -0.088682034 | 0.0986822 | 0.2234933 | 0.4837969 | CMBL | | 134147 |
| ENSG00000259802 | 25.02124095 | 0.032518047 | 0.1382452 | 0.3106611 | NA | MARCHF6-D | | 101929977 |
| ENSG00000145495 | 4725.691582 | -0.081585651 | 0.0570997 | 0.1147407 | 0.3296812 | MARCHF6 | | 10299 |
| ENSG00000145491 | 15.230761 | 0.001092816 | 0.1339161 | 0.9696131 | NA | ROPN1L | | 83853 |
| ENSG00000164236 | 467.8950793 | -0.074316272 | 0.1076296 | 0.3089251 | 0.5782013 | ANKRD33B | | 651746 |
| ENSG00000272016 | 1097.591061 | -0.121538286 | 0.0935341 | 0.094729 | 0.2919603 | NA | NA | |
| ENSG00000112977 | 1955.825853 | 0.062486399 | 0.0756867 | 0.3239395 | 0.5927649 | DAP | | 1611 |
| ENSG00000039139 | 52.28026556 | -0.0748614 | 0.1549951 | 0.1337408 | 0.3604584 | DNAH5 | | 1767 |
| ENSG00000038382 | 8882.009059 | -0.176546062 | 0.0608556 | 0.0013729 | 0.01437 | TRIO | | 7204 |
| ENSG00000145569 | 361.1363366 | -0.003212088 | 0.0998825 | 0.9604469 | 0.9838621 | OTULINL | | 54491 |
| ENSG00000261360 | 13.10353039 | 0.007093676 | 0.134688 | 0.7726446 | NA | OTULIN-DT | | 728178 |
| ENSG00000154124 | 1894.352573 | -0.077856091 | 0.0745873 | 0.21521 | 0.4733322 | OTULIN | | 90268 |
| ENSG00000154122 | 537.9080337 | 0.11612202 | 0.1113979 | 0.135349 | 0.362285 | ANKH | | 56172 |
| ENSG00000183580 | 35.25356661 | 0.024289857 | 0.1333218 | 0.5385847 | NA | FBXL7 | | 23194 |
| ENSG00000173545 | 1397.778747 | 0.301137195 | 0.0926717 | 0.000169 | 0.0026564 | ZNF622 | | 90441 |
| ENSG00000154153 | 113.2001277 | 0.323552946 | 0.3275289 | 0.0151084 | 0.0845214 | RETREG1 | | 54463 |
| ENSG00000145555 | 6356.512256 | -0.100622314 | 0.0642743 | 0.0771913 | 0.2562293 | MYO10 | | 4651 |
| ENSG00000176788 | 1306.256766 | -0.158931148 | 0.089813 | 0.028212 | 0.132294 | BASP1 | | 10409 |
| ENSG00000183666 | 223.3136334 | 0.014546506 | 0.1105245 | 0.8255387 | 0.9283834 | NA | NA | |
| ENSG00000213430 | 11.92824371 | 0.01101136 | 0.1352375 | 0.6406192 | NA | NA | NA | |
| ENSG00000113360 | 2484.839754 | -0.134028837 | 0.0690065 | 0.0258851 | 0.124108 | DROSHA | | 29102 |
| ENSG00000082213 | 1011.720614 | 0.120400895 | 0.0906113 | 0.0916697 | 0.2866917 | C5orf22 | | 55322 |
| ENSG00000113384 | 2987.524234 | -0.179414851 | 0.0663012 | 0.0023617 | 0.021485 | GOLPH3 | | 64083 |
| ENSG00000272086 | 43.31226523 | 0.025686307 | 0.1324676 | 0.5380078 | 0.7618965 | NA | NA | |
| ENSG00000150712 | 2076.439966 | -0.205898283 | 0.0743365 | 0.0015432 | 0.0156613 | MTMR12 | | 54545 |
| ENSG00000056097 | 5089.438557 | 0.13077689 | 0.0632724 | 0.0200399 | 0.1039918 | ZFR | | 51663 |
| ENSG00000113387 | 3989.955374 | -0.319390716 | 0.0683926 | 4.35E-07 | 1.66E-05 | SUB1 | | 10923 |
| ENSG00000113389 | 14.8929026 | 0.023219915 | 0.1367773 | 0.3887805 | NA | NPR3 | | 4883 |
| ENSG00000250697 | 25.85019992 | 1.112838408 | 0.7519781 | 0.0030905 | NA | NA | NA | |
| ENSG00000251281 | 11.71601028 | 2.334977005 | 1.0761476 | 0.0012669 | NA | NA | NA | |
| ENSG00000249572 | 17.55560704 | 0.009146987 | 0.1338602 | 0.7538745 | NA | NA | NA | |
| ENSG00000113407 | 11401.23238 | -0.103112047 | 0.0521143 | 0.0319022 | 0.1437691 | TARS1 | | 6897 |
| ENSG00000242110 | 34.68900365 | 0.034996976 | 0.1366934 | 0.3744419 | NA | AMACR | | 23600 |
| ENSG00000082196 | 15.18679616 | -0.015726135 | 0.1352447 | 0.5532641 | NA | C1QTNF3 | | 114899 |

| | | | | | | | |
|---|---|---|---|---|---|---|---|
| ENSG00000215158 | 65.99720295 | -0.108993945 | 0.1809231 | 0.0766583 | 0.2549883 | NA | NA |
| ENSG00000039560 | 4895.231331 | -0.181160906 | 0.0606178 | 0.0009925 | 0.01101 | RAI14 | 26064 |
| ENSG00000272323 | 12.93523594 | 0.023286089 | 0.1372155 | 0.3511666 | NA | TTC23L-AS1 | 101929704 |
| ENSG00000113456 | 1277.8616 | -0.033405645 | 0.0745865 | 0.5928687 | 0.8020034 | RAD1 | 5810 |
| ENSG00000113460 | 2268.218718 | -0.015897052 | 0.068569 | 0.7871911 | 0.9115956 | BRIX1 | 55299 |
| ENSG00000168724 | 2104.928948 | -0.134990302 | 0.0729292 | 0.031069 | 0.1414883 | DNAJC21 | 134218 |
| ENSG00000152582 | 56.53651553 | 0.041181922 | 0.1359699 | 0.3738432 | 0.6352333 | SPEF2 | 79925 |
| ENSG00000164187 | 760.4818242 | 0.419595466 | 0.1159947 | 2.66E-05 | 0.0005764 | LMBRD2 | 92255 |
| ENSG00000145604 | 904.2510488 | -0.255669282 | 0.1046835 | 0.0025067 | 0.0224892 | SKP2 | 6502 |
| ENSG00000152620 | 353.8331692 | -0.005394599 | 0.1005214 | 0.9349822 | 0.9752234 | NADK2 | 133686 |
| ENSG00000164190 | 2473.337189 | -0.204040865 | 0.0704243 | 0.0010717 | 0.011776 | NIPBL | 25836 |
| ENSG00000197603 | 1452.311362 | 0.102651902 | 0.0769353 | 0.1112219 | 0.3233517 | CPLANE1 | 65250 |
| ENSG00000270558 | 39.28612473 | 0.024750428 | 0.1331325 | 0.5391215 | NA | NA | NA |
| ENSG00000113569 | 4008.708525 | 0.0769531 | 0.0599769 | 0.1531907 | 0.3908224 | NUP155 | 9631 |
| ENSG00000082068 | 734.3102841 | 0.033677356 | 0.0858498 | 0.616704 | 0.816193 | WDR70 | 55100 |
| ENSG00000164318 | 44.6734795 | -0.01791099 | 0.1306809 | 0.6711901 | 0.8494227 | EGFLAM | 133584 |
| ENSG00000113594 | 460.3232153 | 0.005257496 | 0.0939793 | 0.939713 | 0.9764957 | LIFR | 3977 |
| ENSG00000249740 | 31.39711092 | 0.059456427 | 0.1492437 | 0.1335865 | NA | LINC01265 | 101926904 |
| ENSG00000145623 | 4370.109583 | 0.057839635 | 0.0588602 | 0.268402 | 0.5362073 | OSMR | 9180 |
| ENSG00000164327 | 1671.523129 | -0.012809117 | 0.0707842 | 0.8316109 | 0.9304418 | RICTOR | 253260 |
| ENSG00000153071 | 141.850107 | 0.086925896 | 0.1461115 | 0.1976946 | 0.4511271 | DAB2 | 1601 |
| ENSG00000171522 | 167.8877505 | 0.969856813 | 0.2250194 | 8.08E-07 | 2.83E-05 | PTGER4 | 5734 |
| ENSG00000113638 | 336.2839552 | -0.09078377 | 0.117576 | 0.2287494 | 0.4901894 | TTC33 | 23548 |
| ENSG00000132356 | 4274.548607 | -0.149431845 | 0.0630812 | 0.0077986 | 0.0530243 | PRKAA1 | 5562 |
| ENSG00000145592 | 14865.36481 | -0.070299403 | 0.0648928 | 0.2176744 | 0.4769565 | RPL37 | 6167 |
| ENSG00000083720 | 1105.391454 | -0.066916491 | 0.0818176 | 0.3118283 | 0.5816138 | OXCT1 | 5019 |
| ENSG00000205765 | 1429.0226 | 0.100099259 | 0.0787431 | 0.125743 | 0.3480578 | RIMOC1 | 285636 |
| ENSG00000151876 | 182.0390761 | 0.302212719 | 0.2333751 | 0.0144105 | 0.0819203 | FBXO4 | 26272 |
| ENSG00000177738 | 105.0281805 | 0.037915105 | 0.127104 | 0.5120648 | 0.7423891 | ANXA2R-OT | 648987 |
| ENSG00000177721 | 22.70484313 | 0.002943697 | 0.1331629 | 0.9244076 | NA | ANXA2R | 389289 |
| ENSG00000215068 | 36.02715788 | -0.048477681 | 0.1420251 | 0.2357191 | NA | ANXA2R-AS | 153684 |
| ENSG00000172262 | 1693.323903 | 0.024902894 | 0.0711033 | 0.6825476 | 0.8544463 | ZNF131 | 7690 |
| ENSG00000177453 | 14.21836774 | -0.015270063 | 0.1353594 | 0.5513467 | NA | NIM1K | 167359 |
| ENSG00000112972 | 1288.467143 | -0.542440254 | 0.2134594 | 0.0005883 | 0.007279 | HMGCS1 | 3157 |
| ENSG00000151882 | 53.54234569 | 0.038475681 | 0.1362881 | 0.3751908 | 0.6369138 | CCL28 | 56477 |
| ENSG00000151881 | 616.9499747 | -0.06050498 | 0.093595 | 0.387091 | 0.6475168 | TMEM267 | 64417 |
| ENSG00000172244 | 324.444914 | 0.19869058 | 0.1577746 | 0.0377559 | 0.161253 | C5orf34 | 375444 |
| ENSG00000249855 | 17.76483515 | 0.052935453 | 0.1491454 | 0.061829 | NA | EEF1A1P19 | 100421796 |
| ENSG00000172239 | 2500.133718 | -0.069492768 | 0.0645799 | 0.2209098 | 0.4804312 | PAIP1 | 10605 |
| ENSG00000248092 | 388.5320715 | 0.571536384 | 0.1556472 | 1.54E-05 | 0.0003657 | NA | NA |
| ENSG00000112992 | 1446.042014 | -0.031516029 | 0.0750077 | 0.6154731 | 0.8158539 | NNT | 23530 |
| ENSG00000112996 | 1663.717432 | 0.221089502 | 0.0893931 | 0.0031169 | 0.0262665 | MRPS30 | 10884 |
| ENSG00000272335 | 85.360759 | -0.062483866 | 0.1393033 | 0.277392 | 0.5460425 | NA | NA |
| ENSG00000170571 | 792.0872588 | -0.223208053 | 0.1100444 | 0.0087259 | 0.0579133 | EMB | 133418 |
| ENSG00000151883 | 366.0485003 | -0.040261074 | 0.1035749 | 0.5632831 | 0.7807892 | PARP8 | 79668 |
| ENSG00000213949 | 76.32272368 | 0.004180886 | 0.1245221 | 0.936813 | 0.975652 | ITGA1 | 3672 |
| ENSG00000152684 | 628.0704922 | 0.228570453 | 0.1176151 | 0.009983 | 0.0635139 | PELO | 53918 |
| ENSG00000164171 | 650.274668 | 0.951845486 | 0.1250641 | 1.55E-15 | 2.98E-13 | ITGA2 | 3673 |
| ENSG00000164172 | 853.2379271 | 0.006614867 | 0.0812302 | 0.9201979 | 0.9691785 | MOCS2 | 4338 |
| ENSG00000247796 | 106.3748804 | 0.062523168 | 0.1353967 | 0.3068834 | 0.5762265 | MOCS2-DT | 257396 |
| ENSG00000164258 | 592.8901699 | -0.059973892 | 0.0952103 | 0.3929743 | 0.6537074 | NDUFS4 | 4724 |
| ENSG00000185305 | 383.7480135 | -0.677488707 | 0.1664664 | 2.65E-06 | 7.92E-05 | ARL15 | 54622 |
| ENSG00000178996 | 449.9755042 | 0.004720294 | 0.0949751 | 0.9458653 | 0.9784014 | SNX18 | 112574 |
| ENSG00000164294 | 85.07434399 | 0.089277429 | 0.1567917 | 0.1505069 | 0.3866853 | GPX8 | 493869 |
| ENSG00000234602 | 96.24905997 | -0.005950327 | 0.1226213 | 0.9126754 | 0.9655794 | MCIDAS | 345643 |
| ENSG00000152669 | 93.24257425 | -0.0154096 | 0.1230993 | 0.778028 | 0.9074533 | CCNO | 10309 |
| ENSG00000067248 | 1357.916679 | -0.10561804 | 0.0820182 | 0.1150506 | 0.3301979 | DHX29 | 54505 |
| ENSG00000039123 | 2221.553278 | -0.234817579 | 0.0738517 | 0.0003344 | 0.0047329 | MTREX | 23517 |
| ENSG00000067113 | 186.0043208 | -0.027594536 | 0.115902 | 0.6644103 | 0.8443847 | PLPP1 | 8611 |
| ENSG00000177058 | 316.6952835 | -0.03559465 | 0.1047454 | 0.6060111 | 0.8109538 | SLC38A9 | 153129 |
| ENSG00000134352 | 6946.915812 | -0.187521347 | 0.0579856 | 0.0004148 | 0.0055562 | IL6ST | 3572 |
| ENSG00000262211 | 24.80383712 | -0.005087869 | 0.1329078 | 0.8694402 | NA | NA | NA |
| ENSG00000264281 | 44.81235799 | -0.039821856 | 0.1369931 | 0.3533827 | 0.6187516 | NA | NA |
| ENSG00000227908 | 10.9462756 | 0.005384587 | 0.1347612 | 0.819109 | NA | IL6ST-DT | 441072 |
| ENSG00000249697 | 9.879955469 | -0.00428455 | 0.1349575 | 0.8417814 | NA | NA | NA |
| ENSG00000248727 | 9.254327583 | -0.006322604 | 0.1352901 | 0.754968 | NA | LINC01948 | 102467147 |
| ENSG00000095015 | 771.1335167 | -0.525367208 | 0.1191848 | 7.53E-07 | 2.69E-05 | MAP3K1 | 4214 |
| ENSG00000155542 | 291.6603203 | -0.116580761 | 0.1314529 | 0.1436616 | 0.3754163 | SETD9 | 133383 |
| ENSG00000155545 | 859.1832773 | -0.24337317 | 0.111465 | 0.0051991 | 0.0391807 | MIER3 | 166968 |
| ENSG00000271828 | 9.097939397 | -0.000716578 | 0.1349871 | 0.9705195 | NA | NA | NA |

| | | | | | | | |
|---|---|---|---|---|---|---|---|
| ENSG00000062194 | 1989.10481 | -0.149893589 | 0.0785481 | 0.0236136 | 0.1163351 | GPBP1 | 65056 |
| ENSG00000145632 | 9566.059702 | -0.18865418 | 0.0566324 | 0.0002978 | 0.0042947 | PLK2 | 10769 |
| ENSG00000035499 | 1015.33302 | -0.28604427 | 0.0979255 | 0.00053 | 0.0067585 | DEPDC1B | 55789 |
| ENSG00000164181 | 952.8823872 | 0.132269615 | 0.0915001 | 0.0673794 | 0.2349429 | ELOVL7 | 79993 |
| ENSG00000049167 | 307.2491339 | -0.048036834 | 0.1086492 | 0.4920134 | 0.7282321 | ERCC8 | 1161 |
| ENSG00000233847 | 10.68106157 | 0.018354554 | 0.1365103 | 0.422383 | NA | NA | NA |
| ENSG00000164182 | 598.3327823 | 0.03221001 | 0.0899364 | 0.6566638 | 0.8418917 | NDUFAF2 | 91942 |
| ENSG00000188725 | 1537.411407 | -0.057508106 | 0.0721606 | 0.3490789 | 0.6148358 | SMIM15 | 643155 |
| ENSG00000251279 | 12.0639257 | 0.004116041 | 0.1347313 | 0.8611389 | NA | NA | NA |
| ENSG00000130449 | 349.9374463 | 0.187293701 | 0.1450708 | 0.0420453 | 0.1729944 | ZSWIM6 | 57688 |
| ENSG00000068796 | 1342.003813 | -0.265971564 | 0.0873156 | 0.0004093 | 0.0055075 | KIF2A | 3796 |
| ENSG00000086189 | 1012.064716 | 0.039024772 | 0.0803777 | 0.5416043 | 0.7648066 | DIMT1 | 27292 |
| ENSG00000086200 | 872.0377076 | -0.048200152 | 0.0834378 | 0.4656329 | 0.7090416 | IPO11 | 51194 |
| ENSG00000268942 | 35.07315488 | 0.006532233 | 0.1307354 | 0.8678893 | NA | NA | NA |
| ENSG00000153006 | 583.5389213 | 0.062898244 | 0.0968394 | 0.3663465 | 0.6290304 | SREK1IP1 | 285672 |
| ENSG00000153015 | 688.4252874 | -0.108985641 | 0.0980633 | 0.1389925 | 0.3679065 | CWC27 | 10283 |
| ENSG00000049192 | 28.76432704 | 0.092014387 | 0.1785097 | 0.027078 | NA | ADAMTS6 | 11174 |
| ENSG00000250081 | 19.56770517 | 0.002428946 | 0.13318 | 0.9387445 | NA | NA | NA |
| ENSG00000123219 | 764.5525138 | -0.07909022 | 0.0904468 | 0.2574416 | 0.52429 | CENPK | 64105 |
| ENSG00000113593 | 764.601976 | -0.122729249 | 0.0971282 | 0.097276 | 0.296679 | PPWD1 | 23398 |
| ENSG00000113595 | 370.0924402 | 0.082597042 | 0.1174458 | 0.2650853 | 0.5327957 | TRIM23 | 373 |
| ENSG00000113597 | 375.5446818 | 0.025727749 | 0.1002705 | 0.7097955 | 0.8682712 | TRAPPC13 | 80006 |
| ENSG00000253251 | 56.07632364 | -0.004662629 | 0.1274135 | 0.9180117 | 0.9679811 | SHLD3 | 112441434 |
| ENSG00000197860 | 523.147507 | 0.064600015 | 0.0981112 | 0.3798479 | 0.6409076 | SGTB | 54557 |
| ENSG00000123213 | 1795.782544 | -0.040100251 | 0.0718864 | 0.5107075 | 0.7419238 | NLN | 57486 |
| ENSG00000112851 | 2950.396552 | 0.043305543 | 0.0633146 | 0.4405809 | 0.6895939 | ERBIN | 55914 |
| ENSG00000153914 | 3292.579217 | 0.165721176 | 0.0689059 | 0.0063129 | 0.0452401 | SREK1 | 140890 |
| ENSG00000069020 | 134.8269701 | 0.919703486 | 0.2503117 | 1.13E-05 | 0.0002825 | MAST4 | 375449 |
| ENSG00000145675 | 427.6206145 | -0.094332597 | 0.113353 | 0.213404 | 0.4706008 | PIK3R1 | 5295 |
| ENSG00000145740 | 1101.525142 | -0.037534635 | 0.0788649 | 0.5605376 | 0.7795039 | SLC30A5 | 64924 |
| ENSG00000248664 | 14.04697177 | -0.003864944 | 0.134159 | 0.8791907 | NA | NA | NA |
| ENSG00000134057 | 6084.901278 | -0.394957023 | 0.0601739 | 5.85E-12 | 6.39E-10 | CCNB1 | 891 |
| ENSG00000153044 | 520.4088016 | -0.0468054 | 0.0982879 | 0.5032229 | 0.7374652 | CENPH | 64946 |
| ENSG00000134056 | 517.6584227 | -0.038165447 | 0.0951647 | 0.5806342 | 0.7927982 | MRPS36 | 92259 |
| ENSG00000134058 | 660.8435109 | -0.091392194 | 0.0977458 | 0.2088863 | 0.4654027 | CDK7 | 1022 |
| ENSG00000183323 | 371.9688887 | -0.064836365 | 0.1091971 | 0.3672824 | 0.6301742 | CCDC125 | 202243 |
| ENSG00000085231 | 2324.233166 | 0.060033718 | 0.0660397 | 0.2993062 | 0.5693326 | AK6 | 102157402 |
| ENSG00000152942 | 599.9086826 | -0.183151179 | 0.1194687 | 0.0322472 | 0.1448142 | RAD17 | 5884 |
| ENSG00000152939 | 236.8925768 | 0.029579934 | 0.1102476 | 0.6610056 | 0.8430186 | MARVELD2 | 153562 |
| ENSG00000197822 | 487.2215004 | 0.001043771 | 0.0943724 | 0.9891252 | 0.9955163 | OCLN | 100506658 |
| ENSG00000253203 | 32.62358686 | 0.042433503 | 0.1409241 | 0.2441198 | NA | NA | NA |
| ENSG00000183474 | 195.8281559 | 0.004442151 | 0.1146951 | 0.9446897 | 0.9784014 | GTF2H2C | 728340 |
| ENSG00000198237 | 16.09634696 | -0.050678907 | 0.1477429 | 0.0714219 | NA | NA | NA |
| ENSG00000205572 | 13.20548711 | 0.008115154 | 0.1346099 | 0.7486882 | NA | SERF1A | 8293 |
| ENSG00000205571 | 238.1581644 | -0.054951385 | 0.1168174 | 0.4259254 | 0.6784296 | SMN1 | 6606 |
| ENSG00000254701 | 29.33861515 | -0.054550423 | 0.147362 | 0.1353905 | NA | NA | NA |
| ENSG00000253816 | 52.50387723 | -0.045277975 | 0.1374484 | 0.333537 | 0.6010561 | NA | NA |
| ENSG00000226259 | 212.7329011 | 0.189535085 | 0.1826122 | 0.0478803 | 0.1870785 | NA | NA |
| ENSG00000269983 | 16.13788883 | 0.013793942 | 0.1348818 | 0.610075 | NA | NA | NA |
| ENSG00000172058 | 13.73285076 | -0.027300912 | 0.1381789 | 0.284821 | NA | SERF1A | 8293 |
| ENSG00000172062 | 637.1599861 | -0.100969231 | 0.0986896 | 0.1691484 | 0.4148273 | SMN1 | 6606 |
| ENSG00000254353 | 85.92139765 | -0.070333839 | 0.1440468 | 0.2276285 | 0.4888067 | NA | NA |
| ENSG00000145736 | 368.553452 | 0.013755191 | 0.0995867 | 0.8417499 | 0.9350382 | GTF2H2 | 2966 |
| ENSG00000230847 | 13.5599867 | -0.002876369 | 0.1342206 | 0.9068577 | NA | NA | NA |
| ENSG00000215630 | 13.19290231 | -0.032480639 | 0.1399723 | 0.1975079 | NA | GUSBP9 | 118126072 |
| ENSG00000145734 | 2393.142882 | -0.014544004 | 0.0654356 | 0.8176042 | 0.9254567 | BDP1 | 55814 |
| ENSG00000131844 | 1483.798144 | -0.073488249 | 0.0740023 | 0.2399264 | 0.5054546 | MCCC2 | 64087 |
| ENSG00000131711 | 634.3956673 | 0.603697267 | 0.1190127 | 2.66E-08 | 1.47E-06 | MAP1B | 4131 |
| ENSG00000113048 | 1817.209411 | -0.160112833 | 0.0754543 | 0.0131916 | 0.0772001 | MRPS27 | 23107 |
| ENSG00000049883 | 210.1075294 | -0.002568825 | 0.1112913 | 0.9672343 | 0.9871791 | PTCD2 | 79810 |
| ENSG00000272093 | 90.34493407 | 0.300923021 | 0.4192856 | 0.0165912 | 0.0904347 | NA | NA |
| ENSG00000272365 | 10.4774948 | -0.016809163 | 0.1362851 | 0.4524841 | NA | NA | NA |
| ENSG00000083312 | 7697.070702 | -0.13102077 | 0.0581126 | 0.0113241 | 0.0693864 | TNPO1 | 3842 |
| ENSG00000157107 | 332.5743228 | -0.024466742 | 0.1021063 | 0.7206991 | 0.8733776 | FCHO2 | 115548 |
| ENSG00000251493 | 38.77156432 | 0.032247583 | 0.1351296 | 0.4297634 | NA | FOXD1 | 2297 |
| ENSG00000145741 | 8714.502061 | -0.212729244 | 0.0559644 | 4.03E-05 | 0.0008237 | BTF3 | 689 |
| ENSG00000164331 | 117.0294078 | 0.017757752 | 0.1203175 | 0.7637105 | 0.8982943 | ANKRA2 | 57763 |
| ENSG00000164338 | 1052.500126 | 0.051267382 | 0.0791491 | 0.4239043 | 0.6759631 | UTP15 | 84135 |
| ENSG00000214944 | 1564.908839 | 0.164538485 | 0.0802009 | 0.0149019 | 0.0837675 | ARHGEF28 | 64283 |
| ENSG00000171617 | 689.5287889 | 0.706450639 | 0.1239411 | 7.40E-10 | 5.40E-08 | ENC1 | 8507 |

| | | | | | | | | |
|---|---|---|---|---|---|---|---|---|
| ENSG00000049860 | 2182.153034 | 0.049657748 | 0.0669217 | 0.3968231 | 0.6567013 | HEXB | | 3074 |
| ENSG00000164347 | 871.3562665 | -0.024435902 | 0.082203 | 0.7095127 | 0.8682712 | GFM2 | | 84340 |
| ENSG00000164346 | 1584.779144 | -0.158153184 | 0.08296 | 0.0217465 | 0.1096334 | NSA2 | | 10412 |
| ENSG00000198780 | 787.3258929 | -0.143436806 | 0.106906 | 0.0677984 | 0.235778 | FAM169A | | 26049 |
| ENSG00000113161 | 2013.106967 | -0.546476363 | 0.0877278 | 3.56E-11 | 3.38E-09 | HMGCR | | 3156 |
| ENSG00000113163 | 1019.2734 | -0.042077715 | 0.0801315 | 0.5209534 | 0.7491391 | CERT1 | | 10087 |
| ENSG00000122008 | 567.0232494 | 0.236420339 | 0.1260304 | 0.0105877 | 0.0658978 | POLK | | 51426 |
| ENSG00000152359 | 281.9255214 | -0.032910581 | 0.1081093 | 0.6285006 | 0.8230398 | POC5 | | 134359 |
| ENSG00000254893 | 125.1524999 | -0.003248615 | 0.1182887 | 0.9555195 | 0.9824331 | NA | NA | |
| ENSG00000164251 | 10.06280742 | 0.007337562 | 0.1354073 | 0.7200093 | NA | F2RL1 | | 2150 |
| ENSG00000164252 | 588.3319804 | -0.071825908 | 0.1001715 | 0.3168677 | 0.5853754 | AGGF1 | | 55109 |
| ENSG00000132846 | 222.292899 | 0.058540034 | 0.1183767 | 0.4003133 | 0.6583091 | ZBED3 | | 84327 |
| ENSG00000250802 | 28.60732203 | 0.071887841 | 0.1588666 | 0.0686581 | NA | ZBED3-AS1 | | 728723 |
| ENSG00000164253 | 762.3002699 | -0.069118627 | 0.0899045 | 0.3187049 | 0.5874843 | WDR41 | | 55255 |
| ENSG00000171530 | 1585.832459 | -0.108761523 | 0.0781726 | 0.0951782 | 0.2928811 | TBCA | | 6902 |
| ENSG00000132842 | 1512.465434 | -0.046113785 | 0.0741021 | 0.4599701 | 0.7041823 | AP3B1 | | 8546 |
| ENSG00000245556 | 179.6391702 | 0.148672614 | 0.175563 | 0.0804607 | 0.2631929 | SCAMP1-AS | | 728769 |
| ENSG00000085365 | 838.532587 | -0.007613754 | 0.0865035 | 0.944359 | 0.9784014 | SCAMP1 | | 9522 |
| ENSG00000145685 | 783.5604067 | 0.049027254 | 0.0857749 | 0.4664278 | 0.7094916 | LHFPL2 | | 10184 |
| ENSG00000113273 | 396.7135646 | -0.199773409 | 0.1490087 | 0.0341732 | 0.150349 | ARSB | | 411 |
| ENSG00000152409 | 394.0904413 | 0.097597593 | 0.1163486 | 0.2020907 | 0.4562468 | JMY | | 133746 |
| ENSG00000152413 | 389.9850415 | 0.066426856 | 0.108424 | 0.3587399 | 0.6220096 | HOMER1 | | 9456 |
| ENSG00000164329 | 701.711635 | 0.033236645 | 0.0870928 | 0.6230767 | 0.8196126 | TENT2 | | 167153 |
| ENSG00000164309 | 17.51918987 | 0.034400035 | 0.1398474 | 0.2323111 | NA | CMYA5 | | 202333 |
| ENSG00000177034 | 341.8776411 | 0.071696433 | 0.1123063 | 0.3265645 | 0.5947303 | MTX3 | | 345778 |
| ENSG00000164300 | 79.68936171 | -0.00192409 | 0.1241327 | 0.9688937 | 0.9880094 | SERINC5 | | 256987 |
| ENSG00000039319 | 1525.629381 | -0.217392531 | 0.0825758 | 0.0020795 | 0.0194321 | ZFYVE16 | | 9765 |
| ENSG00000249042 | 32.43923357 | -0.005052437 | 0.1316747 | 0.8854105 | NA | NA | NA | |
| ENSG00000152380 | 19.4752145 | -0.002300007 | 0.1331641 | 0.9369246 | NA | FAM151B | | 167555 |
| ENSG00000228716 | 2706.945205 | -0.108606707 | 0.0672122 | 0.0645202 | 0.228798 | DHFR | | 1719 |
| ENSG00000113318 | 618.3031566 | -0.120775755 | 0.1058116 | 0.1152816 | 0.3306172 | MSH3 | | 4437 |
| ENSG00000247572 | 152.936064 | 0.423697412 | 0.2579684 | 0.0052377 | 0.0394462 | CKMT2-AS1 | | 100131067 |
| ENSG00000248794 | 10.4665779 | 0.043265635 | 0.1451164 | 0.056879 | NA | NA | NA | |
| ENSG00000131732 | 492.8903364 | -0.085320147 | 0.1047145 | 0.2479105 | 0.5141783 | ZCCHC9 | | 84240 |
| ENSG00000145687 | 72.28053296 | -0.042074783 | 0.1326041 | 0.4214241 | 0.6737677 | SSBP2 | | 23635 |
| ENSG00000152348 | 532.3486245 | -0.046462132 | 0.0960839 | 0.5052563 | 0.7382367 | ATG10 | | 83734 |
| ENSG00000251495 | 9.586148274 | -0.006893011 | 0.13518 | 0.7459751 | NA | NA | NA | |
| ENSG00000186468 | 10897.36656 | -0.103101118 | 0.0537755 | 0.0362943 | 0.1568993 | RPS23 | | 6228 |
| ENSG00000205464 | 39.10981249 | 0.077786554 | 0.1618812 | 0.0758074 | NA | NA | NA | |
| ENSG00000271862 | 15.12117025 | -0.000547888 | 0.1343458 | 0.9797706 | NA | NA | NA | |
| ENSG00000174695 | 1765.046148 | 0.033976251 | 0.0705181 | 0.5747304 | 0.7887497 | TMEM167A | | 153339 |
| ENSG00000152422 | 199.238448 | 0.012505258 | 0.1120603 | 0.847738 | 0.9375782 | XRCC4 | | 7518 |
| ENSG00000038427 | 1191.543236 | 0.004458149 | 0.0823877 | 0.946815 | 0.9786934 | VCAN | | 1462 |
| ENSG00000127184 | 2913.923304 | -0.209241868 | 0.0696771 | 0.0007346 | 0.0087559 | COX7C | | 1350 |
| ENSG00000145715 | 729.3236272 | -0.110305756 | 0.0985934 | 0.1356556 | 0.3627732 | RASA1 | | 5921 |
| ENSG00000272367 | 17.100002 | -0.01332499 | 0.1344429 | 0.6389389 | NA | NA | NA | |
| ENSG00000134480 | 620.9178987 | 0.021166426 | 0.088595 | 0.7583421 | 0.8963508 | CCNH | | 902 |
| ENSG00000164180 | 559.5858696 | 0.049859136 | 0.0957001 | 0.47536 | 0.715183 | TMEM161B | | 153396 |
| ENSG00000247828 | 157.1058402 | 0.008378382 | 0.1165135 | 0.8923735 | 0.9564219 | TMEM161B- | | 100505894 |
| ENSG00000153140 | 342.6209544 | -0.122511689 | 0.1315264 | 0.1299032 | 0.3544053 | CETN3 | | 1070 |
| ENSG00000176055 | 178.1928676 | -0.043487031 | 0.1195665 | 0.5032859 | 0.7374652 | MBLAC2 | | 153364 |
| ENSG00000113356 | 592.9178327 | 0.022072419 | 0.0921919 | 0.7476088 | 0.8903758 | POLR3G | | 10622 |
| ENSG00000176018 | 756.2587006 | -0.115795263 | 0.0968842 | 0.114948 | 0.3300001 | LYSMD3 | | 116068 |
| ENSG00000164199 | 36.9288562 | -0.013426681 | 0.1310695 | 0.7335211 | NA | ADGRV1 | | 84059 |
| ENSG00000248323 | 79.14681755 | 0.144360323 | 0.218198 | 0.0501259 | 0.1927957 | LUCAT1 | | 100505994 |
| ENSG00000113369 | 206.5053929 | 0.180117961 | 0.1806396 | 0.0542242 | 0.2038458 | ARRDC3 | | 57561 |
| ENSG00000237187 | 18.7179955 | 0.056963971 | 0.1508641 | 0.0672772 | NA | NR2F1-AS1 | | 441094 |
| ENSG00000175745 | 53.99239654 | 0.125049963 | 0.2126287 | 0.0321398 | 0.1445612 | NR2F1 | | 7025 |
| ENSG00000113391 | 190.0228483 | -0.043396823 | 0.1178322 | 0.5111077 | 0.7421105 | ARB2A | | 83989 |
| ENSG00000249353 | 143.3324137 | -0.351254737 | 0.3152024 | 0.0122676 | 0.0734222 | NA | NA | |
| ENSG00000185261 | 14.57644497 | 0.004262674 | 0.1341122 | 0.8730331 | NA | KIAA0825 | | 285600 |
| ENSG00000133302 | 620.865996 | 0.395778595 | 0.1242372 | 0.0001333 | 0.0021999 | SLF1 | | 84250 |
| ENSG00000175471 | 86.3050096 | 0.330966374 | 0.4511285 | 0.0143331 | 0.0818271 | MCTP1 | | 79772 |
| ENSG00000198677 | 1631.145492 | -0.082648132 | 0.0735723 | 0.1815178 | 0.4312202 | SKIC3 | | 9652 |
| ENSG00000164291 | 250.6801983 | 0.051388063 | 0.1177326 | 0.4510136 | 0.6977059 | ARSK | | 153642 |
| ENSG00000175449 | 34.16350909 | 0.063351773 | 0.1509257 | 0.1299691 | NA | RFESD | | 317671 |
| ENSG00000164292 | 680.188396 | 0.106402351 | 0.0991148 | 0.1503731 | 0.3864704 | RHOBTB3 | | 22836 |
| ENSG00000173221 | 62.8524123 | 0.072032921 | 0.1506843 | 0.1716123 | 0.4177997 | GLRX | | 2745 |
| ENSG00000118985 | 408.9587454 | 0.122369791 | 0.1283095 | 0.1294588 | 0.353901 | ELL2 | | 22936 |
| ENSG00000153113 | 3717.588739 | -0.209748425 | 0.0644297 | 0.0003162 | 0.0045044 | CAST | | 831 |

| | | | | | | | | |
|---|---|---|---|---|---|---|---|---|
| ENSG00000249180 | 17.59558462 | -0.024238683 | 0.1367962 | 0.3848446 | NA | NA | NA | |
| ENSG00000164307 | 1361.89353 | -0.07990184 | 0.080999 | 0.2264078 | 0.4874967 | ERAP1 | | 51752 |
| ENSG00000248734 | 21.02842473 | 0.027456066 | 0.1367077 | 0.3881575 | NA | NA | NA | |
| ENSG00000247121 | 45.87053087 | -0.054889467 | 0.1433222 | 0.2286392 | 0.4900429 | NA | NA | |
| ENSG00000164308 | 95.20481053 | -0.022668608 | 0.1240506 | 0.6815614 | 0.8539085 | ERAP2 | | 64167 |
| ENSG00000113441 | 2726.654193 | -0.462510733 | 0.0770745 | 1.78E-10 | 1.43E-08 | LNPEP | | 4012 |
| ENSG00000058729 | 821.0659551 | 0.215640962 | 0.1083666 | 0.0102268 | 0.0645028 | RIOK2 | | 55781 |
| ENSG00000174136 | 1019.933894 | 0.609525202 | 0.0985261 | 4.34E-11 | 4.02E-09 | RGMB | | 285704 |
| ENSG00000153922 | 1703.472567 | -0.048755919 | 0.0704874 | 0.404729 | 0.6614808 | CHD1 | | 1105 |
| ENSG00000174132 | 164.6180629 | 0.410804838 | 0.2481002 | 0.0053715 | 0.040092 | FAM174A | | 345757 |
| ENSG00000145730 | 1893.758161 | -0.055638705 | 0.068445 | 0.3466918 | 0.6132202 | PAM | | 5066 |
| ENSG00000145723 | 85.17870644 | 0.005626967 | 0.123541 | 0.9171042 | 0.96756 | GIN1 | | 54826 |
| ENSG00000145725 | 1149.862187 | -0.189884685 | 0.0915599 | 0.0109162 | 0.0674672 | PPIP5K2 | | 23262 |
| ENSG00000181751 | 310.8163013 | 0.067112548 | 0.1141377 | 0.3513678 | 0.6171375 | MACIR | | 90355 |
| ENSG00000112874 | 188.9857862 | -0.071516172 | 0.1266826 | 0.3058197 | 0.5749687 | NUDT12 | | 83594 |
| ENSG00000184349 | 300.0986027 | 0.036375194 | 0.1137067 | 0.5851355 | 0.7963379 | EFNA5 | | 1946 |
| ENSG00000145743 | 238.5055726 | -0.040591244 | 0.1136214 | 0.5461386 | 0.7681451 | FBXL17 | | 64839 |
| ENSG00000272523 | 10.33295498 | -0.005335521 | 0.1349585 | 0.8070241 | NA | NA | NA | |
| ENSG00000151422 | 601.348763 | -0.345172161 | 0.1212233 | 0.0004805 | 0.0062153 | FER | | 2241 |
| ENSG00000198961 | 1297.248153 | -0.31201715 | 0.0972704 | 0.0001797 | 0.0028029 | PJA2 | | 9867 |
| ENSG00000112893 | 1570.079527 | -0.072244787 | 0.0731334 | 0.2442367 | 0.509998 | MAN2A1 | | 4124 |
| ENSG00000164209 | 844.6775727 | -0.049916153 | 0.0864782 | 0.463404 | 0.7077937 | SLC25A46 | | 91137 |
| ENSG00000134987 | 1780.585656 | -0.043724556 | 0.072084 | 0.4743413 | 0.7142022 | WDR36 | | 134430 |
| ENSG00000152495 | 355.5898536 | -0.053727767 | 0.1070194 | 0.4469257 | 0.6944817 | CAMK4 | | 814 |
| ENSG00000164211 | 1421.375632 | -0.531325592 | 0.0986114 | 5.45E-09 | 3.48E-07 | STARD4 | | 134429 |
| ENSG00000246859 | 22.44761104 | 0.008410126 | 0.133253 | 0.7888428 | NA | STARD4-AS | | 100505678 |
| ENSG00000134986 | 152.3424086 | -0.029625438 | 0.1188286 | 0.631841 | 0.8251856 | NREP | | 9315 |
| ENSG00000129595 | 113.7958697 | -0.083772327 | 0.1464833 | 0.2007562 | 0.4544637 | EPB41L4A | | 64097 |
| ENSG00000224032 | 542.8383732 | 0.208224211 | 0.1252557 | 0.0202506 | 0.1046671 | NA | NA | |
| ENSG00000134982 | 1104.757197 | -0.033386582 | 0.0779345 | 0.6021097 | 0.8079281 | APC | | 324 |
| ENSG00000153037 | 380.7665451 | -0.001344872 | 0.098294 | 0.9875417 | 0.9949521 | SRP19 | | 6728 |
| ENSG00000129625 | 1796.081367 | -0.237037288 | 0.0794938 | 0.0006249 | 0.0076594 | REEP5 | | 7905 |
| ENSG00000270067 | 19.32686997 | 0.026548924 | 0.1367799 | 0.3853308 | NA | NA | NA | |
| ENSG00000172795 | 738.9289297 | -0.034496893 | 0.0887665 | 0.6109053 | 0.8135885 | DCP2 | | 167227 |
| ENSG00000171444 | 103.2368973 | -0.025200477 | 0.1233871 | 0.657533 | 0.8419413 | MCC | | 4163 |
| ENSG00000047188 | 1190.428263 | -0.178477743 | 0.0918617 | 0.0160732 | 0.0885083 | YTHDC2 | | 64848 |
| ENSG00000152503 | 243.7138368 | 0.136794547 | 0.1634411 | 0.0986295 | 0.2996336 | TRIM36 | | 55521 |
| ENSG00000248175 | 25.37091063 | 0.026184847 | 0.1356926 | 0.4418436 | NA | NA | NA | |
| ENSG00000164219 | 344.1725458 | 0.017843636 | 0.1013133 | 0.793834 | 0.913934 | PGGT1B | | 5229 |
| ENSG00000164221 | 166.4819832 | -0.014633937 | 0.1149595 | 0.8142311 | 0.9240962 | CCDC112 | | 153733 |
| ENSG00000145780 | 539.9798451 | -0.167379638 | 0.1183021 | 0.0459669 | 0.183381 | FEM1C | | 56929 |
| ENSG00000134970 | 1589.487047 | -0.284133532 | 0.088494 | 0.0002111 | 0.0031983 | TMED7 | | 51014 |
| ENSG00000145782 | 1172.471229 | 0.074227056 | 0.0788911 | 0.2561944 | 0.5231846 | ATG12 | | 9140 |
| ENSG00000177879 | 1105.470601 | -0.308502317 | 0.0991026 | 0.0002526 | 0.0037349 | AP3S1 | | 1176 |
| ENSG00000145781 | 250.0776354 | -0.225224192 | 0.1800783 | 0.0282888 | 0.1324947 | COMMD10 | | 51397 |
| ENSG00000092421 | 20.29355275 | -0.010286 | 0.1335157 | 0.7354896 | NA | SEMA6A | | 57556 |
| ENSG00000248445 | 16.00856275 | -0.005714981 | 0.1343072 | 0.8217256 | NA | SEMA6A-AS | | 101927233 |
| ENSG00000169570 | 166.8642297 | -0.406991989 | 0.2418879 | 0.0051729 | 0.0390591 | DTWD2 | | 285605 |
| ENSG00000172869 | 995.1341409 | -0.099595253 | 0.0869618 | 0.1497001 | 0.3849101 | DMXL1 | | 1657 |
| ENSG00000145779 | 477.3754947 | 0.016109885 | 0.0944218 | 0.8143031 | 0.9240962 | TNFAIP8 | | 25816 |
| ENSG00000133835 | 1915.578101 | -0.212698958 | 0.0759922 | 0.001356 | 0.014244 | HSD17B4 | | 3295 |
| ENSG00000184838 | 81.97808676 | -0.095296334 | 0.1633834 | 0.1221764 | 0.3423225 | PRR16 | | 51334 |
| ENSG00000151304 | 413.7518177 | 0.298086212 | 0.1646773 | 0.0073703 | 0.0507319 | SRFBP1 | | 153443 |
| ENSG00000113083 | 22.95713432 | 0.004137097 | 0.1326045 | 0.9006124 | NA | LOX | | 4015 |
| ENSG00000205302 | 1005.977835 | -0.006398787 | 0.077979 | 0.9196007 | 0.9690041 | SNX2 | | 6643 |
| ENSG00000064652 | 442.7610004 | 0.349754776 | 0.1451305 | 0.0015348 | 0.0156172 | SNX24 | | 28966 |
| ENSG00000168938 | 293.3598124 | -0.040511969 | 0.1096576 | 0.55483 | 0.7749708 | PPIC | | 5480 |
| ENSG00000249996 | 9.457476114 | -0.014335979 | 0.1360038 | 0.5062523 | NA | NA | NA | |
| ENSG00000168944 | 458.7567726 | 0.056743662 | 0.1013981 | 0.4256323 | 0.6782776 | CEP120 | | 153241 |
| ENSG00000250539 | 9.341388753 | 0.019138629 | 0.1370128 | 0.3614555 | NA | KRT8P33 | | 100418737 |
| ENSG00000151292 | 680.3911652 | -0.182052999 | 0.1161464 | 0.0309744 | 0.141202 | CSNK1G3 | | 1456 |
| ENSG00000168916 | 34.135143 | -0.002697181 | 0.1306631 | 0.9416064 | NA | ZNF608 | | 57507 |
| ENSG00000155324 | 25.19186339 | -0.003076397 | 0.1323733 | 0.9237255 | NA | GRAMD2B | | 65983 |
| ENSG00000164904 | 1006.976825 | -0.12651459 | 0.091102 | 0.0779986 | 0.2575915 | ALDH7A1 | | 501 |
| ENSG00000164902 | 1231.884111 | 0.004402015 | 0.0746569 | 0.9447005 | 0.9784014 | PHAX | | 51808 |
| ENSG00000113368 | 2689.763742 | -0.534611207 | 0.0738301 | 3.58E-14 | 5.65E-12 | LMNB1 | | 4001 |
| ENSG00000173926 | 56.45920657 | 0.092558465 | 0.1683283 | 0.0921468 | 0.287261 | MARCHF3 | | 115123 |
| ENSG00000164241 | 16.41490552 | -0.007143043 | 0.133906 | 0.7962668 | NA | C5orf63 | | 401207 |
| ENSG00000164244 | 1315.521061 | 0.01962191 | 0.0740983 | 0.7538092 | 0.8933893 | PRRC1 | | 133619 |
| ENSG00000245937 | 263.7242046 | 0.101499338 | 0.1380132 | 0.1799444 | 0.4294914 | SLC12A2-D | | 644873 |

| | | | | | | | |
|---|---|---|---|---|---|---|---|
| ENSG00000064651 | 1174.534355 | -0.090733428 | 0.0812555 | 0.1714699 | 0.4177334 | SLC12A2 | 6558 |
| ENSG00000066583 | 618.7340447 | -0.09165488 | 0.0988084 | 0.2092653 | 0.4657317 | ISOC1 | 51015 |
| ENSG00000198108 | 78.29885492 | -0.008690552 | 0.1244345 | 0.86425 | 0.9455189 | CHSY3 | 337876 |
| ENSG00000169567 | 2326.182031 | -0.085510627 | 0.0666926 | 0.1419312 | 0.3722268 | HINT1 | 3094 |
| ENSG00000186687 | 593.6081057 | 0.008028386 | 0.0900526 | 0.9066496 | 0.9632173 | LYRM7 | 90624 |
| ENSG00000158985 | 984.9497389 | -0.10118133 | 0.0862548 | 0.1418925 | 0.3722268 | CDC42SE2 | 56990 |
| ENSG00000158987 | 267.0194356 | 0.015586931 | 0.1093273 | 0.8149116 | 0.9243526 | RAPGEF6 | 51735 |
| ENSG00000217128 | 993.1408523 | 0.237261106 | 0.0982056 | 0.0031445 | 0.0264439 | FNIP1 | 96459 |
| ENSG00000164400 | 141.1688372 | 0.800528002 | 0.2463149 | 5.49E-05 | 0.001073 | CSF2 | 1437 |
| ENSG00000072682 | 937.1978594 | 0.047764896 | 0.0814432 | 0.4660746 | 0.7093442 | P4HA2 | 8974 |
| ENSG00000131435 | 68.63531994 | 0.10316254 | 0.1740359 | 0.0908756 | 0.2849711 | PDLIM4 | 8572 |
| ENSG00000197208 | 83.41396719 | 0.270044765 | 0.405598 | 0.0189475 | 0.0998313 | SLC22A4 | 6583 |
| ENSG00000233006 | 23.17005491 | 0.013554228 | 0.1337599 | 0.6673518 | NA | MIR3936HG | 553103 |
| ENSG00000197375 | 215.3284438 | 0.053642868 | 0.117421 | 0.4334567 | 0.684513 | SLC22A5 | 6584 |
| ENSG00000197536 | 27.1598646 | 0.000102999 | 0.1318263 | 0.9996401 | NA | NA | NA |
| ENSG00000125347 | 876.9959064 | -0.10415796 | 0.0926122 | 0.1454064 | 0.3780081 | IRF1 | 3659 |
| ENSG00000113522 | 2229.127235 | 0.312729675 | 0.0811291 | 1.68E-05 | 0.0003928 | RAD50 | 10111 |
| ENSG00000223442 | 26.52550711 | 0.035760594 | 0.1385431 | 0.3079157 | NA | TH2LCRR | 101927761 |
| ENSG00000131437 | 1104.980178 | -0.084346879 | 0.0835596 | 0.2106978 | 0.4673682 | KIF3A | 11127 |
| ENSG00000164402 | 1652.913807 | 0.107109132 | 0.0772602 | 0.0977625 | 0.2978517 | SEPTIN8 | 23176 |
| ENSG00000164403 | 425.4128057 | 0.047503962 | 0.1002266 | 0.4997814 | 0.734688 | SHROOM1 | 134549 |
| ENSG00000164404 | 12.38210311 | -0.002082051 | 0.1344094 | 0.9297627 | NA | GDF9 | 2661 |
| ENSG00000164405 | 1895.88957 | -0.114970133 | 0.0752484 | 0.070451 | 0.2416852 | UQCRQ | 27089 |
| ENSG00000164406 | 35.19643463 | 0.028175624 | 0.1343207 | 0.4776028 | NA | LEAP2 | 116842 |
| ENSG00000072364 | 3734.589044 | 0.15722922 | 0.0653941 | 0.0069096 | 0.0482434 | AFF4 | 27125 |
| ENSG00000155329 | 343.6877221 | -0.021626047 | 0.1017492 | 0.7514223 | 0.8926188 | ZCCHC10 | 54819 |
| ENSG00000170606 | 5468.012729 | -0.267358156 | 0.0610769 | 2.33E-06 | 7.05E-05 | HSPA4 | 3308 |
| ENSG00000113583 | 1061.489119 | -0.145515255 | 0.0892499 | 0.0423015 | 0.1738038 | C5orf15 | 56951 |
| ENSG00000213585 | 5287.722318 | -0.161028531 | 0.0598949 | 0.0029871 | 0.025591 | VDAC1 | 7416 |
| ENSG00000271737 | 12.97695054 | 0.003218925 | 0.1343661 | 0.8991766 | NA | NA | NA |
| ENSG00000081059 | 109.5796256 | 0.054692607 | 0.1320364 | 0.3638581 | 0.6265998 | TCF7 | 6932 |
| ENSG00000113558 | 2361.133636 | -0.054509277 | 0.0657042 | 0.3448021 | 0.610988 | SKP1 | 6500 |
| ENSG00000113575 | 2876.234316 | -0.078142706 | 0.0638532 | 0.165683 | 0.4099435 | PPP2CA | 5515 |
| ENSG00000006837 | 19.30926528 | 0.028713091 | 0.1374915 | 0.3439131 | NA | CDKL3 | 51265 |
| ENSG00000266751 | 16.88491875 | 0.028419355 | 0.1380483 | 0.3069917 | NA | MIR3661 | 100500905 |
| ENSG00000119048 | 715.5709792 | 0.03899025 | 0.0871497 | 0.5649406 | 0.7820725 | UBE2B | 7320 |
| ENSG00000237190 | 459.0402998 | -0.145238317 | 0.1288007 | 0.0827108 | 0.2682245 | CDKN2AIPN | 91368 |
| ENSG00000043143 | 1042.419378 | 0.017848984 | 0.0797803 | 0.7839046 | 0.9098848 | JADE2 | 23338 |
| ENSG00000152700 | 957.2250154 | -0.063308501 | 0.083578 | 0.34028 | 0.6075474 | SAR1B | 51128 |
| ENSG00000113615 | 735.9524848 | 0.192221121 | 0.1139638 | 0.0229418 | 0.1142299 | SEC24A | 10802 |
| ENSG00000164615 | 505.9579159 | 0.222180147 | 0.1283077 | 0.0156861 | 0.0870118 | CAMLG | 819 |
| ENSG00000145833 | 3647.437463 | -0.138806212 | 0.0695723 | 0.0219054 | 0.1103393 | DDX46 | 9879 |
| ENSG00000181904 | 1903.47811 | 0.042133338 | 0.0675753 | 0.4742729 | 0.7141913 | C5orf24 | 134553 |
| ENSG00000113621 | 725.938511 | -0.141670756 | 0.1026035 | 0.0655406 | 0.2312931 | TXNDC15 | 79770 |
| ENSG00000132570 | 195.9590759 | -0.071994949 | 0.1276119 | 0.3013479 | 0.5710237 | PCBD2 | 84105 |
| ENSG00000069011 | 173.2725047 | 0.085132249 | 0.1356594 | 0.2324354 | 0.495067 | PITX1 | 5307 |
| ENSG00000224186 | 22.21341313 | -0.006850393 | 0.1337546 | 0.8080305 | NA | PITX1-AS1 | 100996485 |
| ENSG00000113648 | 4168.612131 | -0.146925796 | 0.0640547 | 0.0099666 | 0.0634631 | MACROH2A | 9555 |
| ENSG00000120708 | 350.1788773 | 0.199279398 | 0.152246 | 0.0358639 | 0.1556191 | TGFBI | 7045 |
| ENSG00000113658 | 1529.559075 | -0.01972284 | 0.0701566 | 0.7429672 | 0.8881088 | SMAD5 | 4090 |
| ENSG00000177733 | 3490.576851 | -0.055009466 | 0.0604674 | 0.3125291 | 0.5818701 | HNRNPA0 | 10949 |
| ENSG00000031003 | 960.3269999 | -0.251892522 | 0.103518 | 0.0026575 | 0.0235166 | FAM13B | 51306 |
| ENSG00000112983 | 1922.119888 | -0.134384799 | 0.0838051 | 0.0503099 | 0.1930585 | BRD8 | 10902 |
| ENSG00000112984 | 2410.664375 | -0.575842372 | 0.0830622 | 3.08E-13 | 4.34E-11 | KIF20A | 10112 |
| ENSG00000094880 | 1195.880961 | -0.125570632 | 0.0840621 | 0.066406 | 0.2330096 | CDC23 | 8697 |
| ENSG00000158402 | 301.3754886 | -0.115886882 | 0.1324005 | 0.1453894 | 0.3780081 | CDC25C | 995 |
| ENSG00000120709 | 2055.903201 | 0.500108444 | 0.0796953 | 2.96E-11 | 2.86E-09 | FAM53C | 51307 |
| ENSG00000249971 | 9.252963091 | 0.005303863 | 0.1351205 | 0.8048722 | NA | NA | NA |
| ENSG00000120733 | 2101.156512 | -0.154197026 | 0.0789529 | 0.0200825 | 0.104167 | KDM3B | 51780 |
| ENSG00000132563 | 37.22180351 | 0.000954548 | 0.1302925 | 0.9824762 | NA | REEP2 | 51308 |
| ENSG00000120738 | 479.8290598 | 0.09124123 | 0.1080992 | 0.2326186 | 0.4951049 | EGR1 | 1958 |
| ENSG00000120705 | 6233.570164 | -0.04950233 | 0.0537079 | 0.3150081 | 0.58392 | ETF1 | 2107 |
| ENSG00000113013 | 11887.68808 | 0.07575113 | 0.0503097 | 0.1047598 | 0.3110572 | HSPA9 | 3313 |
| ENSG00000222937 | 25.28916413 | 0.049017125 | 0.1445382 | 0.1674491 | NA | SNORD63B | 109617013 |
| ENSG00000044115 | 6270.815451 | -0.119431266 | 0.0580607 | 0.0230935 | 0.1145456 | CTNNA1 | 1495 |
| ENSG00000120725 | 331.5816574 | 0.034327995 | 0.1049908 | 0.6193709 | 0.8179841 | SIL1 | 64374 |
| ENSG00000015479 | 9269.038314 | -0.178749353 | 0.0541418 | 0.0003642 | 0.0050461 | MATR3 | 9782 |
| ENSG00000120727 | 1051.966706 | -0.065474152 | 0.0812293 | 0.3216366 | 0.5905434 | PAIP2 | 51247 |
| ENSG00000272742 | 21.7961276 | -0.011692554 | 0.1334723 | 0.7085459 | NA | NA | NA |
| ENSG00000228672 | 84.47554638 | 0.024098488 | 0.1260337 | 0.6525419 | 0.839003 | PROB1 | 389333 |

| | | | | | | | |
|---|---|---|---|---|---|---|---|
| ENSG00000170469 | 25.14877568 | 0.007428064 | 0.1326536 | 0.8227545 | NA | SPATA24 | 202051 |
| ENSG00000170464 | 105.9543897 | 0.053766849 | 0.1325848 | 0.3642798 | 0.6269614 | DNAJC18 | 202052 |
| ENSG00000131508 | 2001.736499 | -0.055491784 | 0.067621 | 0.3444707 | 0.610988 | UBE2D2 | 7322 |
| ENSG00000171604 | 20.53527371 | 0.013874177 | 0.1340646 | 0.6499965 | NA | CXXC5 | 51523 |
| ENSG00000158458 | 189.3816721 | 0.05272062 | 0.1199189 | 0.4359246 | 0.6863128 | NRG2 | 9542 |
| ENSG00000245146 | 19.01092556 | 0.027587994 | 0.1373748 | 0.3483421 | NA | LOC124900: | 124900193 |
| ENSG00000185129 | 446.6026235 | 0.097417575 | 0.1140862 | 0.2017791 | 0.45577 | PURA | 5813 |
| ENSG00000182700 | 22.4490174 | 0.067847397 | 0.158669 | 0.0344042 | NA | IGIP | 492311 |
| ENSG00000120306 | 818.6824214 | 0.221403037 | 0.1153561 | 0.0111694 | 0.0688493 | CYSTM1 | 84418 |
| ENSG00000113068 | 1952.621592 | 0.298699857 | 0.0783354 | 2.14E-05 | 0.0004789 | PFDN1 | 5201 |
| ENSG00000113070 | 1850.296081 | 1.467515329 | 0.086576 | 1.06E-65 | 6.17E-62 | HBEGF | 1839 |
| ENSG00000249637 | 14.17565256 | 0.023650117 | 0.137044 | 0.3683191 | NA | NA | NA |
| ENSG00000131503 | 83.11474051 | -0.027471408 | 0.1266118 | 0.6076309 | 0.8118194 | ANKHD1 | 54882 |
| ENSG00000254996 | 21.93848875 | 0.014174674 | 0.1340242 | 0.6462304 | NA | ANKHD1-EII | 404734 |
| ENSG00000213523 | 897.1616773 | 0.063464314 | 0.085627 | 0.3541231 | 0.6187516 | SRA1 | 10011 |
| ENSG00000113108 | 175.533853 | -0.000117906 | 0.1130969 | 0.9972754 | 0.998947 | APBB3 | 10307 |
| ENSG00000176087 | 1833.408827 | 0.014340429 | 0.066919 | 0.8082068 | 0.9201427 | SLC35A4 | 113829 |
| ENSG00000170458 | 12.10064887 | 0.000924877 | 0.1348689 | 0.9686158 | NA | CD14 | 929 |
| ENSG00000131495 | 970.5951266 | -0.05807667 | 0.0833272 | 0.3824 | 0.6432624 | NDUFA2 | 4695 |
| ENSG00000113119 | 155.6524352 | -0.116968722 | 0.1585518 | 0.1246755 | 0.3465814 | TMCO6 | 55374 |
| ENSG00000113141 | 2184.549852 | -0.140504767 | 0.0713134 | 0.0227567 | 0.1136958 | IK | 3550 |
| ENSG00000120314 | 1306.969732 | -0.053553475 | 0.0755721 | 0.3963353 | 0.6564005 | WDR55 | 54853 |
| ENSG00000256453 | 50.48885152 | 0.002980904 | 0.1281557 | 0.9501324 | 0.9802134 | DND1 | 373863 |
| ENSG00000170445 | 2588.127945 | -0.150631891 | 0.071567 | 0.0151407 | 0.084662 | HARS1 | 3035 |
| ENSG00000112855 | 693.9882532 | 0.048713022 | 0.0894036 | 0.4792499 | 0.7182599 | HARS2 | 23438 |
| ENSG00000146007 | 1580.40736 | -0.001764428 | 0.0710362 | 0.9759973 | 0.9911001 | ZMAT2 | 153527 |
| ENSG00000112852 | 42.50283793 | -0.056444052 | 0.1460326 | 0.1820373 | 0.4317028 | PCDHB2 | 56133 |
| ENSG00000178913 | 1331.832454 | -0.026031203 | 0.0728658 | 0.6725199 | 0.8499086 | TAF7 | 6879 |
| ENSG00000253846 | 13.69273249 | 0.014842548 | 0.1353846 | 0.5605509 | NA | PCDHGA10 | 56106 |
| ENSG00000240184 | 537.7159924 | -0.21737515 | 0.1241036 | 0.0157298 | 0.0871215 | PCDHGC3 | 5098 |
| ENSG00000131504 | 5580.931536 | -0.219429774 | 0.0625547 | 0.0001195 | 0.0020108 | DIAPH1 | 1729 |
| ENSG00000228737 | 12.43654319 | 0.012118399 | 0.135138 | 0.6241948 | NA | NA | NA |
| ENSG00000171720 | 1363.151373 | -0.229150146 | 0.0868683 | 0.0018494 | 0.017898 | HDAC3 | 8841 |
| ENSG00000164620 | 318.3875841 | 0.066865176 | 0.1147973 | 0.3525543 | 0.6185027 | RELL2 | 285613 |
| ENSG00000197948 | 455.7635354 | -0.053394805 | 0.0994326 | 0.4491525 | 0.6961982 | FCHSD1 | 89848 |
| ENSG00000120318 | 10.5322988 | 0.004307645 | 0.1349049 | 0.8483213 | NA | ARAP3 | 64411 |
| ENSG00000081791 | 849.9568366 | -0.090072793 | 0.0898582 | 0.1982526 | 0.4513782 | DELE1 | 9812 |
| ENSG00000013561 | 1164.507198 | 0.031965026 | 0.0756389 | 0.5982529 | 0.805834 | RNF14 | 9604 |
| ENSG00000113552 | 1389.500731 | 0.200464991 | 0.0856041 | 0.0052702 | 0.0395691 | GNPDA1 | 10007 |
| ENSG00000131507 | 1365.586846 | -0.137190584 | 0.0848815 | 0.0477775 | 0.1868392 | NDFIP1 | 80762 |
| ENSG00000187678 | 1329.735034 | 0.010567663 | 0.1093592 | 0.8748474 | 0.9493854 | SPRY4 | 81848 |
| ENSG00000231185 | 20.3691838 | 0.052548223 | 0.1478765 | 0.0979099 | NA | SPRY4-AS1 | 101926941 |
| ENSG00000113578 | 18.39677829 | 1.468133639 | 0.8414133 | 0.0021634 | NA | FGF1 | 2246 |
| ENSG00000145819 | 402.8108646 | -0.325675357 | 0.1469435 | 0.0027378 | 0.024017 | ARHGAP26 | 23092 |
| ENSG00000113580 | 896.1652197 | -0.066238783 | 0.0864983 | 0.325423 | 0.5941598 | NR3C1 | 2908 |
| ENSG00000145817 | 663.1392173 | -0.083221579 | 0.0947904 | 0.2438257 | 0.5095953 | YIPF5 | 81555 |
| ENSG00000186314 | 92.86498258 | 0.02523306 | 0.1249164 | 0.6487247 | 0.836587 | PRELID2 | 153768 |
| ENSG00000156463 | 9.512185126 | 0.028557325 | 0.139452 | 0.1761044 | NA | SH3RF2 | 153769 |
| ENSG00000251556 | 16.15792511 | -0.013795746 | 0.1346859 | 0.6180062 | NA | NA | NA |
| ENSG00000133706 | 3852.819733 | 0.185174847 | 0.0654871 | 0.0015822 | 0.0159598 | LARS1 | 51520 |
| ENSG00000091009 | 1547.884179 | -0.049194341 | 0.0714708 | 0.4200644 | 0.672898 | RBM27 | 54439 |
| ENSG00000113649 | 2859.610659 | -0.050124364 | 0.0632133 | 0.3737085 | 0.6352333 | TCERG1 | 10915 |
| ENSG00000113657 | 2207.195263 | -0.050543074 | 0.0755951 | 0.4229008 | 0.6748503 | DPYSL3 | 1809 |
| ENSG00000145868 | 405.9687813 | 0.058406284 | 0.1045669 | 0.4127506 | 0.6666675 | FBXO38 | 81545 |
| ENSG00000169252 | 299.3683518 | 1.397735886 | 0.1903803 | 1.07E-14 | 1.90E-12 | ADRB2 | 154 |
| ENSG00000169247 | 41.68997348 | 1.232584937 | 0.4927717 | 0.0004397 | 0.0058292 | SH3TC2 | 79628 |
| ENSG00000173210 | 21.89708374 | 0.006849102 | 0.1333447 | 0.8231443 | NA | ABLIM3 | 22885 |
| ENSG00000157510 | 219.2102689 | 0.250719143 | 0.1989 | 0.0221745 | 0.1114068 | AFAP1L1 | 134265 |
| ENSG00000164284 | 672.4134733 | 0.218475381 | 0.1136791 | 0.0114137 | 0.069784 | GRPEL2 | 134266 |
| ENSG00000145882 | 64.95231927 | 0.060310109 | 0.1423947 | 0.2499253 | 0.5161557 | PCYOX1L | 78991 |
| ENSG00000113712 | 2445.924656 | 0.103470889 | 0.072744 | 0.0956597 | 0.2938616 | CSNK1A1 | 1452 |
| ENSG00000230551 | 255.4201023 | -0.001407477 | 0.1065702 | 0.9815915 | 0.9932057 | NA | NA |
| ENSG00000155846 | 268.7065661 | 0.197642921 | 0.1709465 | 0.0414168 | 0.1711923 | PPARGC1B | 133522 |
| ENSG00000155850 | 800.9960969 | 0.146726655 | 0.0996005 | 0.0543311 | 0.2040646 | SLC26A2 | 1836 |
| ENSG00000164296 | 76.04740692 | 0.040813979 | 0.1319532 | 0.4391838 | 0.6886014 | TIGD6 | 81789 |
| ENSG00000113716 | 1839.922136 | 0.253161675 | 0.0794887 | 0.0002878 | 0.0041763 | HMGXB3 | 22993 |
| ENSG00000214485 | 147.28116 | -0.011840934 | 0.1175733 | 0.8453325 | 0.9367545 | RPL7P1 | 6174 |
| ENSG00000070814 | 4617.091062 | 0.07778069 | 0.0585173 | 0.1401834 | 0.3695639 | TCOF1 | 6949 |
| ENSG00000164587 | 18941.60359 | -0.118573074 | 0.0549869 | 0.0187186 | 0.099207 | RPS14 | 6208 |
| ENSG00000070614 | 4415.913315 | 0.052674722 | 0.0583415 | 0.3175124 | 0.5861961 | NDST1 | 3340 |

| | | | | | | | | |
|---|---|---|---|---|---|---|---|---|
| ENSG00000171992 | 481.1183929 | 0.316104861 | 0.1445281 | 0.0031189 | 0.0262665 | SYNPO | | 11346 |
| ENSG00000086589 | 1393.312344 | 0.084654082 | 0.0793411 | 0.1959008 | 0.4486016 | RBM22 | | 55696 |
| ENSG00000132912 | 1505.825331 | 0.080002673 | 0.0763694 | 0.2101154 | 0.4666816 | DCTN4 | | 51164 |
| ENSG00000145901 | 2919.383091 | 0.06336908 | 0.0718786 | 0.3014616 | 0.5710237 | TNIP1 | | 10318 |
| ENSG00000197043 | 788.3774178 | -0.081810268 | 0.0907078 | 0.2426353 | 0.508472 | ANXA6 | | 309 |
| ENSG00000198624 | 122.6964626 | -0.069834948 | 0.1361569 | 0.2777141 | 0.5461905 | CCDC69 | | 26112 |
| ENSG00000196743 | 855.6515163 | -0.149099402 | 0.0987637 | 0.049824 | 0.1919505 | GM2A | | 2760 |
| ENSG00000123643 | 415.3825515 | 0.11072015 | 0.1160701 | 0.1542478 | 0.3924711 | SLC36A1 | | 206358 |
| ENSG00000177556 | 468.6381493 | -0.060048771 | 0.1018259 | 0.3995235 | 0.6582409 | ATOX1 | | 475 |
| ENSG00000145907 | 5336.53693 | -0.149976882 | 0.0603817 | 0.0057791 | 0.0423232 | G3BP1 | | 10146 |
| ENSG00000055147 | 328.0770367 | -0.025037206 | 0.1060293 | 0.7113867 | 0.8691254 | FAM114A2 | | 10827 |
| ENSG00000037749 | 701.4487625 | -0.052847899 | 0.0892113 | 0.4333888 | 0.684513 | MFAP3 | | 4238 |
| ENSG00000164574 | 1117.806578 | -0.129864853 | 0.0877398 | 0.064964 | 0.2300227 | GALNT10 | | 55568 |
| ENSG00000245275 | 11.62497047 | 0.012438791 | 0.135464 | 0.5937646 | NA | SAP30L-AS1 | | 386627 |
| ENSG00000164576 | 538.8690639 | -0.094523209 | 0.1083302 | 0.208779 | 0.4654027 | SAP30L | | 79685 |
| ENSG00000155506 | 6879.221063 | -0.08847216 | 0.0555147 | 0.0806825 | 0.2636232 | LARP1 | | 23367 |
| ENSG00000155508 | 1148.328608 | -0.058166742 | 0.0782789 | 0.3668998 | 0.6297953 | CNOT8 | | 9337 |
| ENSG00000082516 | 1479.878723 | -0.008083985 | 0.0727458 | 0.8937494 | 0.9571796 | GEMIN5 | | 25929 |
| ENSG00000082515 | 914.8128904 | -0.144634113 | 0.0936258 | 0.0495465 | 0.1914498 | MRPL22 | | 29093 |
| ENSG00000145850 | 23.13996041 | -0.045604262 | 0.1432671 | 0.1781296 | NA | TIMD4 | | 91937 |
| ENSG00000113249 | 999.5285824 | -0.998413589 | 0.1007426 | 2.10E-24 | 9.43E-22 | HAVCR1 | | 26762 |
| ENSG00000155868 | 215.3330419 | 0.026932725 | 0.1143391 | 0.6792088 | 0.8535466 | MED7 | | 9443 |
| ENSG00000055163 | 154.5579773 | -0.10423022 | 0.1505503 | 0.1559146 | 0.3946511 | CYFIP2 | | 26999 |
| ENSG00000135074 | 58.94544946 | 0.107559703 | 0.1875911 | 0.0512086 | 0.1950344 | ADAM19 | | 8728 |
| ENSG00000172548 | 76.67883928 | 0.872695362 | 0.3734753 | 0.000707 | 0.0084829 | NIPAL4 | | 348938 |
| ENSG00000113272 | 213.155473 | 0.022030701 | 0.1127258 | 0.7379239 | 0.8854328 | THG1L | | 54974 |
| ENSG00000155858 | 151.1096061 | 0.003925114 | 0.115594 | 0.9519641 | 0.9806656 | LSM11 | | 134353 |
| ENSG00000113282 | 2575.309069 | 0.02520367 | 0.0627581 | 0.6537689 | 0.8399571 | CLINT1 | | 9685 |
| ENSG00000145860 | 2151.114948 | -0.225945506 | 0.0739266 | 0.0005367 | 0.0067985 | RNF145 | | 153830 |
| ENSG00000164332 | 555.0667336 | -0.044076598 | 0.0931272 | 0.52343 | 0.7509978 | UBLCP1 | | 134510 |
| ENSG00000113312 | 1021.409448 | -0.082924682 | 0.0849415 | 0.2199045 | 0.479502 | TTC1 | | 7265 |
| ENSG00000170234 | 467.2822238 | 0.000426389 | 0.094223 | 0.9972129 | 0.998947 | PWWP2A | | 114825 |
| ENSG00000135083 | 109.005013 | 0.00956687 | 0.1210931 | 0.8669007 | 0.9462033 | CCNJL | | 79616 |
| ENSG00000221886 | 72.63935526 | 0.022754151 | 0.1275787 | 0.6539261 | 0.8399571 | FAM200C | | 63920 |
| ENSG00000164609 | 1101.4756 | 0.148033908 | 0.0880907 | 0.0379018 | 0.1615218 | SLU7 | | 10569 |
| ENSG00000164611 | 1693.203002 | -0.369232014 | 0.090288 | 4.81E-06 | 0.0001358 | PTTG1 | | 9232 |
| ENSG00000253522 | 188.5875756 | 0.010796131 | 0.1304157 | 0.7905251 | 0.9121996 | MIR3142HG | | 107075116 |
| ENSG00000113328 | 1521.374326 | -0.310534393 | 0.0834087 | 2.82E-05 | 0.0006029 | CCNG1 | | 900 |
| ENSG00000170584 | 945.1876621 | -0.182663103 | 0.1032546 | 0.0216557 | 0.1093783 | NUDCD2 | | 134492 |
| ENSG00000072571 | 1309.555393 | -0.136809011 | 0.0857625 | 0.0496081 | 0.1915611 | HMMR | | 3161 |
| ENSG00000038274 | 1076.878225 | -0.15387785 | 0.0898827 | 0.0332596 | 0.1473287 | MAT2B | | 27430 |
| ENSG00000229119 | 37.33864715 | -0.038421188 | 0.1382333 | 0.3193219 | NA | NA | NA | |
| ENSG00000113645 | 2198.08675 | -0.022821932 | 0.0646456 | 0.6882328 | 0.858008 | WWC1 | | 23286 |
| ENSG00000113643 | 2216.835229 | -0.276216007 | 0.0765523 | 5.40E-05 | 0.0010581 | RARS1 | | 5917 |
| ENSG00000120137 | 1941.303957 | -0.136305438 | 0.0731105 | 0.0297862 | 0.1376858 | PANK3 | | 79646 |
| ENSG00000233913 | 15.93468597 | -0.01156289 | 0.1347511 | 0.6561467 | NA | RPL10P9 | | 389342 |
| ENSG00000040275 | 1159.639194 | -0.073292561 | 0.0813335 | 0.2673689 | 0.5355171 | SPDL1 | | 54908 |
| ENSG00000134516 | 24.0617321 | -0.04316629 | 0.1421375 | 0.1990108 | NA | DOCK2 | | 1794 |
| ENSG00000204767 | 34.61521323 | -0.00993204 | 0.1311569 | 0.7918313 | NA | INSYN2B | | 100131897 |
| ENSG00000204764 | 220.09136 | -0.033317398 | 0.1130645 | 0.6117527 | 0.8141485 | RANBP17 | | 64901 |
| ENSG00000181163 | 27387.1501 | -0.331127096 | 0.0540488 | 1.37E-10 | 1.14E-08 | NPM1 | | 4869 |
| ENSG00000156427 | 18.97775417 | 0.001507907 | 0.1337102 | 0.9592109 | NA | FGF18 | | 8817 |
| ENSG00000072803 | 2005.30561 | -0.16305732 | 0.0812272 | 0.0164223 | 0.0897235 | FBXW11 | | 23291 |
| ENSG00000072786 | 1724.89427 | 0.137511872 | 0.0782584 | 0.0366976 | 0.158062 | STK10 | | 6793 |
| ENSG00000168246 | 990.0442784 | -0.130116136 | 0.0894941 | 0.0677346 | 0.2357591 | UBTD2 | | 92181 |
| ENSG00000174705 | 915.0972878 | 0.026579592 | 0.0806785 | 0.6844975 | 0.8554818 | SH3PXD2B | | 285590 |
| ENSG00000214357 | 45.86813375 | 0.045034692 | 0.1391041 | 0.3017143 | 0.5710237 | NEURL1B | | 54492 |
| ENSG00000253683 | 13.3256481 | -0.014190154 | 0.1352835 | 0.5738707 | NA | NA | NA | |
| ENSG00000120129 | 866.8514436 | 0.779967345 | 0.1200768 | 5.05E-12 | 5.57E-10 | DUSP1 | | 1843 |
| ENSG00000113719 | 1431.986029 | -0.218690895 | 0.0863945 | 0.0027092 | 0.0238991 | ERGIC1 | | 57222 |
| ENSG00000204758 | 30.47534785 | 0.086071065 | 0.1713361 | 0.0409814 | NA | RPL26L1-AS | | 100268168 |
| ENSG00000037241 | 684.2623411 | -0.042269497 | 0.09147 | 0.5383991 | 0.7619385 | RPL26L1 | | 51121 |
| ENSG00000113732 | 1895.276009 | -0.078677947 | 0.0708936 | 0.1947723 | 0.4468935 | ATP6V0E1 | | 8992 |
| ENSG00000164463 | 218.0986182 | 0.165704368 | 0.1745953 | 0.0655916 | 0.2314029 | CREBRF | | 153222 |
| ENSG00000113734 | 187.143331 | -0.018555425 | 0.1139302 | 0.769925 | 0.9016938 | BNIP1 | | 662 |
| ENSG00000183072 | 260.6283099 | 0.147440533 | 0.1569377 | 0.0863608 | 0.2756984 | NKX2-5 | | 1482 |
| ENSG00000113739 | 1534.777157 | 1.41953358 | 0.0919488 | 4.79E-55 | 1.40E-51 | STC2 | | 8614 |
| ENSG00000145919 | 953.5138245 | 0.131628218 | 0.0937787 | 0.0729562 | 0.2469116 | BOD1 | | 91272 |
| ENSG00000113742 | 423.6125214 | 0.188164611 | 0.1454162 | 0.0413447 | 0.1709549 | CPEB4 | | 80315 |
| ENSG00000164466 | 1809.977337 | -0.117707436 | 0.0762912 | 0.0669558 | 0.2341648 | SFXN1 | | 94081 |

| | | | | | | | | |
|---|---|---|---|---|---|---|---|---|
| ENSG00000051596 | 910.1920428 | 0.031978292 | 0.0816777 | 0.627082 | 0.8222448 | THOC3 | | 84321 |
| ENSG00000170085 | 185.1163964 | 0.088020366 | 0.1361614 | 0.2221924 | 0.4818844 | SIMC1 | | 375484 |
| ENSG00000251667 | 8.462609786 | 0.029865308 | 0.1400514 | 0.1377449 | NA | BRCC3P1 | | 100421311 |
| ENSG00000122203 | 2294.404009 | 0.032865723 | 0.064205 | 0.5573266 | 0.7765874 | KIAA1191 | | 57179 |
| ENSG00000250909 | 16.39816935 | 0.003322674 | 0.1336964 | 0.9076455 | NA | NA | NA | |
| ENSG00000048162 | 1742.332714 | 0.038162304 | 0.0686756 | 0.5211897 | 0.749153 | NOP16 | | 51491 |
| ENSG00000146066 | 589.1620904 | -0.149135589 | 0.1118001 | 0.0637074 | 0.2269492 | HIGD2A | | 192286 |
| ENSG00000175416 | 2244.136816 | 0.134302683 | 0.0700484 | 0.0274915 | 0.1295919 | CLTB | | 1212 |
| ENSG00000113194 | 2300.384164 | -0.003696756 | 0.0650422 | 0.9488702 | 0.9798226 | FAF2 | | 23197 |
| ENSG00000146083 | 589.9210116 | 0.050223985 | 0.0925499 | 0.4698422 | 0.7112787 | RNF44 | | 22838 |
| ENSG00000169258 | 1247.15519 | 0.089880918 | 0.0837637 | 0.1847325 | 0.4349649 | GPRIN1 | | 114787 |
| ENSG00000048140 | 1680.920251 | -0.02155852 | 0.0706212 | 0.7205662 | 0.8733776 | TSPAN17 | | 26262 |
| ENSG00000113763 | 33.74327136 | 0.07017275 | 0.1561877 | 0.0935619 | NA | UNC5A | | 90249 |
| ENSG00000087206 | 1354.797037 | 0.073802074 | 0.0808439 | 0.2641161 | 0.5320125 | UIMC1 | | 51720 |
| ENSG00000113761 | 407.4713116 | 0.127785877 | 0.1242684 | 0.1149534 | 0.3300001 | ZNF346 | | 23567 |
| ENSG00000251666 | 10.84431027 | -0.016137505 | 0.1360543 | 0.4813156 | NA | NA | NA | |
| ENSG00000160867 | 367.5537649 | -0.145661428 | 0.1478875 | 0.0887151 | 0.2803792 | FGFR4 | | 2264 |
| ENSG00000165671 | 2082.50281 | -0.269761783 | 0.0798954 | 0.0001314 | 0.0021804 | NSD1 | | 64324 |
| ENSG00000169228 | 159.1503596 | -0.065124232 | 0.1304406 | 0.3231952 | 0.5919165 | RAB24 | | 53917 |
| ENSG00000213347 | 319.0136032 | -0.069210684 | 0.1142195 | 0.3383381 | 0.6058756 | MXD3 | | 83463 |
| ENSG00000169230 | 4487.149118 | -0.062741109 | 0.0582907 | 0.2326031 | 0.4951049 | PRELID1 | | 27166 |
| ENSG00000169223 | 1804.792897 | -0.003573767 | 0.069391 | 0.9499627 | 0.9801708 | LMAN2 | | 10960 |
| ENSG00000169220 | 100.6769033 | 0.043974254 | 0.1304494 | 0.4349998 | 0.685449 | RGS14 | | 10636 |
| ENSG00000131187 | 209.7253323 | 0.164017282 | 0.1747692 | 0.0671395 | 0.2346667 | F12 | | 2161 |
| ENSG00000198055 | 1508.110712 | -0.058138434 | 0.075178 | 0.3564383 | 0.6197178 | GRK6 | | 2870 |
| ENSG00000246334 | 27.84671535 | 0.050259672 | 0.1447802 | 0.1716786 | NA | NA | NA | |
| ENSG00000131188 | 282.5875689 | 0.042894428 | 0.1085284 | 0.5370536 | 0.7611135 | PRR7 | | 80758 |
| ENSG00000113758 | 3164.084877 | 0.074379017 | 0.0645612 | 0.1913191 | 0.4422717 | DBN1 | | 1627 |
| ENSG00000196923 | 1228.373468 | 0.263107139 | 0.0901753 | 0.000631 | 0.0077181 | PDLIM7 | | 9260 |
| ENSG00000146094 | 414.6182772 | -0.037014362 | 0.0997476 | 0.593884 | 0.8026852 | DOK3 | | 79930 |
| ENSG00000183258 | 1649.791334 | -0.132431635 | 0.0784152 | 0.0439379 | 0.177715 | DDX41 | | 51428 |
| ENSG00000146067 | 547.8107692 | -0.046622769 | 0.1007947 | 0.5057634 | 0.7385701 | FAM193B | | 54540 |
| ENSG00000184840 | 2871.722233 | -0.010203223 | 0.0652855 | 0.8576174 | 0.9425883 | TMED9 | | 54732 |
| ENSG00000027847 | 240.2421186 | -0.010238708 | 0.1089912 | 0.8758499 | 0.9497329 | B4GALT7 | | 11285 |
| ENSG00000247679 | 20.91624444 | 0.014805537 | 0.1345309 | 0.6151958 | NA | NA | NA | |
| ENSG00000246596 | 29.18135103 | 0.052322461 | 0.1454287 | 0.1692447 | NA | NA | NA | |
| ENSG00000170089 | 329.9992877 | -0.003681995 | 0.1014246 | 0.9553511 | 0.9823465 | NA | NA | |
| ENSG00000145911 | 498.7849626 | 0.208683501 | 0.1350937 | 0.0241308 | 0.1179876 | N4BP3 | | 23138 |
| ENSG00000145916 | 689.5463091 | -0.082326961 | 0.0952687 | 0.2442185 | 0.509998 | RMND5B | | 64777 |
| ENSG00000145912 | 2486.928343 | -0.07258238 | 0.0702033 | 0.228436 | 0.4897594 | NHP2 | | 55651 |
| ENSG00000197451 | 10017.83168 | -0.015581132 | 0.0498071 | 0.7362017 | 0.884192 | HNRNPAB | | 3182 |
| ENSG00000175309 | 409.5515512 | -0.053768858 | 0.1021915 | 0.4483356 | 0.6958415 | PHYKPL | | 85007 |
| ENSG00000113240 | 481.0102949 | 0.244325633 | 0.1380643 | 0.012022 | 0.0724273 | CLK4 | | 57396 |
| ENSG00000169131 | 453.6872866 | 0.115724742 | 0.1152969 | 0.1401998 | 0.3695639 | ZNF354A | | 6940 |
| ENSG00000178338 | 294.8559127 | 0.045612408 | 0.1081638 | 0.5139482 | 0.744196 | ZNF354B | | 117608 |
| ENSG00000234284 | 47.5899851 | 0.028395443 | 0.1324618 | 0.5207419 | 0.7489848 | ZNF879 | | 345462 |
| ENSG00000176783 | 684.2320129 | 0.139352227 | 0.1038238 | 0.0723722 | 0.2457085 | RUFY1 | | 80230 |
| ENSG00000244945 | 17.67541274 | -0.000108281 | 0.1335863 | 0.9946734 | NA | RUFY1-AS1 | | 101928445 |
| ENSG00000169045 | 13456.31514 | -0.190497897 | 0.0541675 | 0.0001664 | 0.0026334 | HNRNPH1 | | 3187 |
| ENSG00000127022 | 13571.02602 | -0.034679791 | 0.0489405 | 0.4461297 | 0.6941567 | CANX | | 821 |
| ENSG00000161021 | 1042.409033 | -0.293759086 | 0.0991267 | 0.0004439 | 0.0058728 | MAML1 | | 9794 |
| ENSG00000161013 | 3335.59172 | -0.112076293 | 0.0643642 | 0.0486847 | 0.1890888 | MGAT4B | | 11282 |
| ENSG00000161011 | 9471.542935 | 0.436142894 | 0.0588382 | 1.26E-14 | 2.20E-12 | SQSTM1 | | 8878 |
| ENSG00000161010 | 519.1434419 | 0.16641976 | 0.1307373 | 0.0552296 | 0.206258 | MRNIP | | 51149 |
| ENSG00000245317 | 9.00498983 | 0.008043548 | 0.1354183 | 0.6974234 | NA | MRNIP-DT | | 100996419 |
| ENSG00000197226 | 3551.827469 | -0.064884803 | 0.0604402 | 0.2298051 | 0.4909091 | TBC1D9B | | 23061 |
| ENSG00000113269 | 773.4571197 | -0.116680524 | 0.0975707 | 0.1143322 | 0.328805 | RNF130 | | 55819 |
| ENSG00000146090 | 13.40273 | -0.004580508 | 0.134423 | 0.8514838 | NA | RASGEF1C | | 255426 |
| ENSG00000050748 | 1203.340751 | -0.173022094 | 0.092149 | 0.0193725 | 0.1013391 | MAPK9 | | 5601 |
| ENSG00000131459 | 639.4564243 | 0.718610208 | 0.132674 | 3.78E-09 | 2.51E-07 | GFPT2 | | 9945 |
| ENSG00000113300 | 1350.723945 | -0.213663991 | 0.0854 | 0.0030638 | 0.0260518 | CNOT6 | | 57472 |
| ENSG00000260841 | 22.14716177 | -0.03225539 | 0.1383235 | 0.3041699 | NA | NA | NA | |
| ENSG00000131446 | 1829.941589 | -0.034110155 | 0.0755729 | 0.5874428 | 0.7977736 | MGAT1 | | 4245 |
| ENSG00000245060 | 59.86729889 | 0.043990214 | 0.1365618 | 0.3555978 | 0.6192724 | LINC00847 | | 729678 |
| ENSG00000196670 | 175.8229684 | 0.191269761 | 0.2048868 | 0.0464894 | 0.1842999 | ZFP62 | | 643836 |
| ENSG00000250222 | 10.44575947 | 0.020092334 | 0.1369449 | 0.3715036 | NA | NA | NA | |
| ENSG00000146054 | 421.7081074 | -0.20153454 | 0.1573657 | 0.0355239 | 0.1544689 | TRIM7 | | 81786 |
| ENSG00000248514 | 8.361607945 | -0.005418169 | 0.1352578 | 0.788105 | NA | NA | NA | |
| ENSG00000146063 | 1036.858106 | 0.063313163 | 0.081057 | 0.3357217 | 0.6031891 | TRIM41 | | 90933 |
| ENSG00000204628 | 15891.92811 | -0.063949916 | 0.0490748 | 0.161372 | 0.4033781 | RACK1 | | 10399 |

| | | | | | | | |
|---|---|---|---|---|---|---|---|
| ENSG00000233937 | 105.5888943 | 0.053420592 | 0.1319888 | 0.3704184 | 0.6328256 | CTC-338M1: | 101928649 |
| ENSG00000183718 | 210.00615 | -0.099157923 | 0.1364272 | 0.1886614 | 0.4395202 | TRIM52 | 84851 |
| ENSG00000268397 | 79.91597128 | 0.024549951 | 0.1263824 | 0.644352 | 0.8338883 | NA | NA |
| ENSG00000248275 | 68.59620967 | 0.007137282 | 0.1256913 | 0.8850729 | 0.9535774 | TRIM52-AS1 | 100507602 |
| ENSG00000238035 | 14.55060666 | 0.025076923 | 0.1375315 | 0.3289197 | NA | NA | NA |
| ENSG00000112679 | 432.6138498 | 0.023576968 | 0.0963699 | 0.7322417 | 0.8813039 | DUSP22 | 56940 |
| ENSG00000112685 | 773.6106509 | -0.13123446 | 0.0983019 | 0.0796508 | 0.2613697 | EXOC2 | 55770 |
| ENSG00000054598 | 109.8110292 | 0.134868695 | 0.1894747 | 0.0777804 | 0.2571988 | FOXC1 | 2296 |
| ENSG00000112699 | 388.4841321 | 0.038741285 | 0.1009272 | 0.5790608 | 0.7916677 | GMDS | 2762 |
| ENSG00000250903 | 19.36587543 | -0.015569534 | 0.1346035 | 0.5931457 | NA | GMDS-DT | 100508120 |
| ENSG00000124535 | 1397.338186 | 0.014518748 | 0.0732927 | 0.8156992 | 0.9249765 | WRNIP1 | 56897 |
| ENSG00000021355 | 404.5827181 | 0.134574962 | 0.1234129 | 0.0997334 | 0.3014974 | SERPINB1 | 1992 |
| ENSG00000170542 | 10.88507861 | 0.017996666 | 0.1364738 | 0.4294323 | NA | SERPINB9 | 5272 |
| ENSG00000124570 | 1211.628949 | -0.053109652 | 0.0779596 | 0.4084612 | 0.6640358 | SERPINB6 | 5269 |
| ENSG00000244041 | 15.11736554 | 0.016797531 | 0.1353811 | 0.534251 | NA | LINC01011 | 401232 |
| ENSG00000124588 | 281.4551999 | -0.024009244 | 0.1071325 | 0.7216805 | 0.8742947 | NQO2 | 4835 |
| ENSG00000137275 | 1442.224691 | -0.011660094 | 0.0718318 | 0.8478064 | 0.9375782 | RIPK1 | 8737 |
| ENSG00000272277 | 27.22514385 | 0.017764045 | 0.1334135 | 0.6127987 | NA | NA | NA |
| ENSG00000137274 | 148.2887358 | 0.048055036 | 0.1238026 | 0.4534213 | 0.6987417 | BPHL | 670 |
| ENSG00000137267 | 445.9273903 | 0.242694706 | 0.145979 | 0.014563 | 0.0825376 | TUBB2A | 7280 |
| ENSG00000137285 | 26.77146701 | 1.035814197 | 0.7485296 | 0.0035488 | NA | TUBB2B | 347733 |
| ENSG00000180822 | 313.9225574 | 0.106258444 | 0.1317437 | 0.1725622 | 0.4194205 | PSMG4 | 389362 |
| ENSG00000168994 | 523.0300548 | 0.612850504 | 0.1282316 | 1.17E-07 | 5.50E-06 | PXDC1 | 221749 |
| ENSG00000270504 | 81.60623397 | 1.066758199 | 0.3384225 | 6.84E-05 | 0.0012756 | NA | NA |
| ENSG00000260604 | 20.41487704 | 0.067352641 | 0.1579998 | 0.0391632 | NA | NA | NA |
| ENSG00000112739 | 2839.639487 | -0.133129916 | 0.0693244 | 0.0273604 | 0.1292344 | PRPF4B | 8899 |
| ENSG00000198721 | 490.4725334 | -0.033725725 | 0.095206 | 0.6245406 | 0.8202204 | ECI2 | 10455 |
| ENSG00000234817 | 13.43575208 | 0.008616324 | 0.1345531 | 0.7381807 | NA | ECI2-DT | 100507506 |
| ENSG00000153046 | 779.6342357 | -0.053148461 | 0.0862567 | 0.4261537 | 0.678557 | CDYL | 9425 |
| ENSG00000124787 | 333.1481273 | -0.053799908 | 0.1086639 | 0.451158 | 0.6978252 | RPP40 | 10799 |
| ENSG00000271978 | 9.386213483 | 0.024597085 | 0.1382464 | 0.2496874 | NA | NA | NA |
| ENSG00000214113 | 509.9597702 | -0.011429905 | 0.0932486 | 0.8655512 | 0.9457908 | LYRM4 | 57128 |
| ENSG00000145982 | 209.2413932 | 0.019627474 | 0.110962 | 0.766296 | 0.8994294 | FARS2 | 10667 |
| ENSG00000124782 | 620.5463273 | 0.01899313 | 0.0920215 | 0.7698205 | 0.9016618 | RREB1 | 6239 |
| ENSG00000124783 | 2814.519029 | -0.127083855 | 0.0661611 | 0.0290109 | 0.1352261 | SSR1 | 6745 |
| ENSG00000238221 | 16.66114176 | -0.024293918 | 0.1369063 | 0.376674 | NA | NA | NA |
| ENSG00000124784 | 978.0145155 | 0.176530345 | 0.0956159 | 0.0202259 | 0.1045853 | RIOK1 | 83732 |
| ENSG00000261189 | 8.347477301 | 0.030308981 | 0.1401774 | 0.1353238 | NA | DSP-AS1 | 101928076 |
| ENSG00000096696 | 4058.27527 | -0.098023309 | 0.06217 | 0.0764475 | 0.2545912 | DSP | 1832 |
| ENSG00000168566 | 754.6041158 | -0.059013596 | 0.0877029 | 0.3873046 | 0.6477813 | SNRNP48 | 154007 |
| ENSG00000153162 | 179.056229 | 0.387821566 | 0.2363725 | 0.0060154 | 0.043508 | BMP6 | 654 |
| ENSG00000239264 | 328.9497344 | 0.05891895 | 0.1121193 | 0.4065952 | 0.6627741 | TXNDC5 | 81567 |
| ENSG00000188428 | 232.947487 | -0.038814607 | 0.1126977 | 0.5641411 | 0.7817002 | BLOC1S5 | 63915 |
| ENSG00000124802 | 416.5600654 | -0.13489781 | 0.125714 | 0.1000001 | 0.3018463 | EEF1E1 | 9521 |
| ENSG00000124786 | 400.7979434 | -0.034358435 | 0.0992438 | 0.6195855 | 0.8180661 | SLC35B3 | 51000 |
| ENSG00000137203 | 420.9242676 | -0.109140709 | 0.1153404 | 0.1596178 | 0.4006222 | TFAP2A | 7020 |
| ENSG00000229950 | 20.77831345 | 0.010910047 | 0.134089 | 0.707 | NA | NA | NA |
| ENSG00000137434 | 19.47512813 | 0.040519355 | 0.14172 | 0.1942189 | NA | C6orf52 | 347744 |
| ENSG00000111845 | 950.9861939 | 0.219298724 | 0.1053538 | 0.0081262 | 0.054647 | PAK1IP1 | 55003 |
| ENSG00000111843 | 1211.296498 | 0.074515361 | 0.0826078 | 0.264505 | 0.5323182 | TMEM14C | 51522 |
| ENSG00000137210 | 1248.516291 | -0.132192464 | 0.0948221 | 0.0724305 | 0.2457085 | TMEM14B | 81853 |
| ENSG00000224531 | 734.9891218 | 0.317685437 | 0.1133038 | 0.0006267 | 0.0076729 | SMIM13 | 221710 |
| ENSG00000205269 | 148.0213309 | -0.259987221 | 0.2548541 | 0.0230085 | 0.1142761 | TMEM170B | 100113407 |
| ENSG00000111863 | 34.52895145 | 0.054065109 | 0.1451381 | 0.1887425 | NA | ADTRP | 84830 |
| ENSG00000095951 | 430.6016317 | 0.610825507 | 0.1406741 | 9.10E-07 | 3.14E-05 | HIVEP1 | 3096 |
| ENSG00000078401 | 27.99449738 | 0.032022834 | 0.1374778 | 0.3468604 | NA | EDN1 | 1906 |
| ENSG00000212802 | 39.61210153 | -0.052753442 | 0.1437523 | 0.2127276 | NA | RPL15P3 | 653232 |
| ENSG00000215022 | 12.08203498 | 0.002619557 | 0.1344544 | 0.9162907 | NA | LOC100130: | 100130357 |
| ENSG00000145979 | 231.7737302 | -0.106843214 | 0.1360968 | 0.1679521 | 0.4131077 | TBC1D7 | 51256 |
| ENSG00000145990 | 97.71586031 | 0.575209127 | 0.3297915 | 0.0030873 | 0.0261896 | GFOD1 | 54438 |
| ENSG00000124523 | 91.44366947 | -0.025643976 | 0.1270866 | 0.6220248 | 0.8187116 | SIRT5 | 23408 |
| ENSG00000261071 | 24.54199352 | 0.039087639 | 0.1400843 | 0.2565354 | NA | NA | NA |
| ENSG00000225921 | 1482.873123 | -0.202185341 | 0.088858 | 0.0060656 | 0.0437114 | NOL7 | 51406 |
| ENSG00000268059 | 46.65059148 | -0.006775616 | 0.1288611 | 0.8740121 | 0.9491371 | NA | NA |
| ENSG00000010017 | 1254.562141 | 0.047647027 | 0.0753136 | 0.4503399 | 0.6972061 | RANBP9 | 10048 |
| ENSG00000050393 | 1315.654554 | 0.061905008 | 0.0766673 | 0.3325227 | 0.5998623 | MCUR1 | 63933 |
| ENSG00000180537 | 1648.000443 | 0.204147561 | 0.0933094 | 0.0073856 | 0.0508077 | RNF182 | 221687 |
| ENSG00000112149 | 540.0346874 | -0.134837052 | 0.1172863 | 0.0940388 | 0.2908137 | CD83 | 9308 |
| ENSG00000008083 | 364.3377149 | 0.005882537 | 0.1005066 | 0.9315452 | 0.973389 | JARID2 | 3720 |
| ENSG00000047579 | 371.3020431 | 0.005301274 | 0.0988573 | 0.9399575 | 0.9766571 | DTNBP1 | 84062 |

| | | | | | | | | |
|---|---|---|---|---|---|---|---|---|
| ENSG00000007944 | 77.64341007 | -0.019576617 | 0.1257322 | 0.7071444 | 0.8675568 | MYLIP | | 29116 |
| ENSG00000124788 | 23.368001 | -0.017869215 | 0.1342784 | 0.5764611 | NA | ATXN1 | | 6310 |
| ENSG00000112183 | 23.49195928 | 0.045552558 | 0.1441803 | 0.1412205 | NA | RBM24 | | 221662 |
| ENSG00000112186 | 478.8692686 | -0.190955328 | 0.1281885 | 0.0313877 | 0.1422309 | CAP2 | | 10486 |
| ENSG00000137414 | 621.835101 | -0.065296138 | 0.0941938 | 0.3532751 | 0.6187516 | FAM8A1 | | 51439 |
| ENSG00000124789 | 3171.017788 | -0.019884574 | 0.0611372 | 0.7154144 | 0.871162 | NUP153 | | 9972 |
| ENSG00000272269 | 76.84075813 | 0.025170721 | 0.1268961 | 0.6325411 | 0.8251856 | NUP153-AS | | 105374952 |
| ENSG00000137177 | 1326.621392 | -0.068214515 | 0.0782548 | 0.2909972 | 0.5608991 | KIF13A | | 63971 |
| ENSG00000137364 | 626.5531543 | -0.078621949 | 0.0968998 | 0.272798 | 0.5408271 | TPMT | | 7172 |
| ENSG00000165097 | 829.5833945 | -0.218324982 | 0.1053042 | 0.0082967 | 0.0556652 | KDM1B | | 221656 |
| ENSG00000124795 | 7471.867785 | -0.221626065 | 0.0580078 | 3.51E-05 | 0.0007331 | DEK | | 7913 |
| ENSG00000137393 | 13.92507376 | 0.00269462 | 0.1342651 | 0.9172271 | NA | RNF144B | | 255488 |
| ENSG00000172201 | 22.1551511 | -0.046966974 | 0.1443241 | 0.1520579 | NA | ID4 | | 3400 |
| ENSG00000172197 | 146.4922935 | -0.22182943 | 0.2452089 | 0.032494 | 0.1453706 | MBOAT1 | | 154141 |
| ENSG00000112242 | 1386.371565 | -0.047429396 | 0.0752621 | 0.4513776 | 0.6978775 | E2F3 | | 1871 |
| ENSG00000145996 | 312.480072 | 0.00188352 | 0.1042835 | 0.9796277 | 0.9923983 | CDKAL1 | | 54901 |
| ENSG00000124766 | 201.8071642 | -0.059330565 | 0.1274562 | 0.3642945 | 0.6269614 | SOX4 | | 6659 |
| ENSG00000146038 | 666.1651772 | -0.490164556 | 0.1164508 | 1.99E-06 | 6.19E-05 | DCDC2 | | 51473 |
| ENSG00000124532 | 1390.552775 | -0.1820844 | 0.0870065 | 0.0112983 | 0.069315 | MRS2 | | 57380 |
| ENSG00000112293 | 28.53203889 | -0.003379135 | 0.1321026 | 0.9195282 | NA | GPLD1 | | 2822 |
| ENSG00000112294 | 354.7079449 | -0.044976551 | 0.1038723 | 0.5198524 | 0.7483099 | ALDH5A1 | | 7915 |
| ENSG00000137261 | 18.91792167 | 0.002743104 | 0.1334444 | 0.9268004 | NA | KIAA0319 | | 9856 |
| ENSG00000111802 | 1065.822109 | -0.142181341 | 0.0898083 | 0.0476805 | 0.1866714 | TDP2 | | 51567 |
| ENSG00000112304 | 445.5577168 | -0.117016343 | 0.1146876 | 0.1346776 | 0.3614858 | ACOT13 | | 55856 |
| ENSG00000272345 | 32.96585694 | -0.012768652 | 0.1319107 | 0.7292319 | NA | NA | NA | |
| ENSG00000112308 | 4046.991426 | -0.019991436 | 0.0592801 | 0.711909 | 0.8693997 | C6orf62 | | 81688 |
| ENSG00000112312 | 1344.677811 | -0.047763624 | 0.0754898 | 0.4488095 | 0.6959957 | GMNN | | 51053 |
| ENSG00000111913 | 22.62357661 | -0.010363477 | 0.1333481 | 0.7390267 | NA | RIPOR2 | | 9750 |
| ENSG00000079691 | 1047.866431 | -0.252587699 | 0.0955183 | 0.0015097 | 0.015496 | CARMIL1 | | 55604 |
| ENSG00000112343 | 603.5170319 | -0.079662637 | 0.0974275 | 0.2678263 | 0.5360657 | TRIM38 | | 10475 |
| ENSG00000272462 | 52.99850497 | -0.024953216 | 0.1309191 | 0.580336 | 0.7924836 | NA | NA | |
| ENSG00000198366 | 8.86262868 | -0.020109668 | 0.1372157 | 0.3342888 | NA | NA | NA | |
| ENSG00000124693 | 18.4844218 | 0.055051322 | 0.1496131 | 0.076735 | NA | NA | NA | |
| ENSG00000187837 | 1487.667518 | -0.429268266 | 0.0882569 | 1.08E-07 | 5.15E-06 | H1-2 | | 3006 |
| ENSG00000010704 | 183.5842443 | -0.557237354 | 0.2247561 | 0.0006791 | 0.008203 | HFE | | 3077 |
| ENSG00000197061 | 10.14604664 | 0.011634736 | 0.1354863 | 0.606055 | NA | H4C3 | | 8364 |
| ENSG00000180596 | 119.8215838 | 0.094574391 | 0.1516391 | 0.1684227 | 0.4140037 | H2BC4 | | 8347 |
| ENSG00000180573 | 1878.637612 | -0.209016144 | 0.0827579 | 0.0030122 | 0.0257333 | H2AC6 | | 8334 |
| ENSG00000158373 | 666.295952 | 0.060197739 | 0.0966887 | 0.3966227 | 0.6565973 | H2BC5 | | 3017 |
| ENSG00000197697 | 11.67766492 | -0.009127774 | 0.1349499 | 0.6999035 | NA | NA | NA | |
| ENSG00000197409 | 86.54235352 | 0.051573253 | 0.1355858 | 0.344386 | 0.610988 | H3C4 | | 8351 |
| ENSG00000197846 | 23.70577303 | 0.000510249 | 0.132578 | 0.9895175 | NA | NA | NA | |
| ENSG00000217275 | 11.99818755 | 8.61E-05 | 0.1344746 | 0.9994625 | NA | RPS10P1 | | 394255 |
| ENSG00000198518 | 29.19860905 | 0.097102628 | 0.1838906 | 0.0250242 | NA | NA | NA | |
| ENSG00000187990 | 74.83646677 | -0.03886578 | 0.1313977 | 0.4558689 | 0.7007253 | NA | NA | |
| ENSG00000168274 | 71.0322885 | 0.045147755 | 0.1344483 | 0.3619869 | 0.6253133 | NA | NA | |
| ENSG00000196966 | 29.74626928 | 0.025012618 | 0.134664 | 0.4901848 | NA | NA | NA | |
| ENSG00000124575 | 10.4409037 | -0.041490259 | 0.1443441 | 0.0625365 | NA | H1-3 | | 3007 |
| ENSG00000197459 | 95.49249244 | -0.087482318 | 0.1526369 | 0.1673012 | 0.4122331 | NA | NA | |
| ENSG00000256018 | 110.7072908 | -0.044031753 | 0.1271916 | 0.4597602 | 0.7041733 | NA | NA | |
| ENSG00000218690 | 12.40341751 | -0.00083803 | 0.1344518 | 0.9698627 | NA | H2AC10P | | 8333 |
| ENSG00000158406 | 367.6213899 | 0.568533409 | 0.1528738 | 1.28E-05 | 0.0003124 | H4C8 | | 8365 |
| ENSG00000186470 | 143.2043894 | -0.762655381 | 0.2601087 | 0.0001509 | 0.0024274 | BTN3A2 | | 11118 |
| ENSG00000124508 | 573.9128347 | 0.173831627 | 0.1174537 | 0.0390616 | 0.1651386 | BTN2A2 | | 10385 |
| ENSG00000026950 | 107.9429579 | -0.717617878 | 0.2886892 | 0.0005455 | 0.0068665 | BTN3A1 | | 11119 |
| ENSG00000124549 | 216.6314305 | 0.050528913 | 0.116539 | 0.4605517 | 0.7048321 | NA | NA | |
| ENSG00000111801 | 30.15713596 | 0.0029263 | 0.1313526 | 0.9393075 | NA | BTN3A3 | | 10384 |
| ENSG00000112763 | 863.4906207 | 0.221034003 | 0.1057077 | 0.0078315 | 0.0532168 | BTN2A1 | | 11120 |
| ENSG00000228223 | 265.3372719 | -0.057273224 | 0.1152599 | 0.4091654 | 0.6643076 | HCG11 | | 493812 |
| ENSG00000261353 | 9.913949662 | 0.016845688 | 0.1363502 | 0.4473131 | NA | NA | NA | |
| ENSG00000182952 | 1318.512622 | -0.128662104 | 0.0845369 | 0.061604 | 0.2214825 | HMGN4 | | 10473 |
| ENSG00000146109 | 834.3638976 | 0.049127659 | 0.0856885 | 0.4670807 | 0.7094916 | ABT1 | | 29777 |
| ENSG00000233631 | 15.74248021 | -0.001799638 | 0.1339723 | 0.9436632 | NA | NA | NA | |
| ENSG00000181315 | 191.3649402 | -0.032629451 | 0.1154704 | 0.6143268 | 0.8156344 | ZNF322 | | 79692 |
| ENSG00000261584 | 20.47085435 | -0.016238497 | 0.1342633 | 0.5991155 | NA | NA | NA | |
| ENSG00000241549 | 16.53643963 | -0.020318097 | 0.1359653 | 0.4564459 | NA | NA | NA | |
| ENSG00000224843 | 13.084354 | 0.022769859 | 0.1371615 | 0.3543717 | NA | LINC00240 | | 100133205 |
| ENSG00000272468 | 69.07318344 | 0.053213895 | 0.1398131 | 0.2876011 | 0.5574378 | NA | NA | |
| ENSG00000124635 | 456.9738314 | 0.102318934 | 0.1132965 | 0.1824469 | 0.4320381 | H2BC11 | | 8970 |
| ENSG00000196787 | 184.1860124 | 0.341786717 | 0.2295201 | 0.0092017 | 0.0603156 | H2AC11 | | 8969 |

| | | | | | | | | |
|---|---|---|---|---|---|---|---|---|
| ENSG00000197903 | 3034.682187 | 0.239547026 | 0.0733988 | 0.0002444 | 0.0036273 | H2BC12 | | 85236 |
| ENSG00000198339 | 27.140064 | 0.000489468 | 0.132493 | 0.9900862 | NA | NA | NA | |
| ENSG00000112812 | 41.62368764 | 0.043908171 | 0.1394232 | 0.2927582 | 0.5621971 | PRSS16 | | 10279 |
| ENSG00000204789 | 80.97999069 | -0.094432119 | 0.1619203 | 0.127993 | 0.3516197 | NA | NA | |
| ENSG00000124613 | 164.2584484 | 0.645261996 | 0.2302293 | 0.0002551 | 0.0037619 | ZNF391 | | 346157 |
| ENSG00000096654 | 193.8730713 | 0.226113735 | 0.234361 | 0.0315406 | 0.1425243 | ZNF184 | | 7738 |
| ENSG00000196747 | 27.56694381 | 0.03185251 | 0.1372242 | 0.3595419 | NA | H2AC13 | | 8329 |
| ENSG00000203813 | 86.70158649 | 0.004352547 | 0.1235159 | 0.9378507 | 0.9761783 | NA | NA | |
| ENSG00000197238 | 15.33628675 | 0.012192393 | 0.1346071 | 0.655817 | NA | H4C11 | | 8363 |
| ENSG00000197914 | 25.58334385 | 0.092039716 | 0.179491 | 0.0219734 | NA | NA | NA | |
| ENSG00000184348 | 16.85229767 | 0.017134205 | 0.1352841 | 0.5375614 | NA | NA | NA | |
| ENSG00000233822 | 92.63885155 | 1.118822091 | 0.3012332 | 9.18E-06 | 0.0002355 | H2BC15 | | 8341 |
| ENSG00000198374 | 13.32702983 | 0.014381418 | 0.1353858 | 0.5665696 | NA | NA | NA | |
| ENSG00000184357 | 9.621957234 | -0.028698289 | 0.1397218 | 0.1467011 | NA | H1-5 | | 3009 |
| ENSG00000196331 | 31.43882551 | 0.037307124 | 0.1380749 | 0.3276637 | NA | NA | NA | |
| ENSG00000124657 | 21.31261656 | 0.019654831 | 0.134922 | 0.5283632 | NA | OR2B6 | | 26212 |
| ENSG00000197279 | 84.41268129 | 1.293473947 | 0.3165319 | 2.02E-06 | 6.27E-05 | ZNF165 | | 7718 |
| ENSG00000219891 | 25.04553342 | 0.092207157 | 0.1818391 | 0.0114372 | NA | NA | NA | |
| ENSG00000269293 | 45.17202294 | -0.020361781 | 0.1310073 | 0.6328692 | 0.8252447 | ZSCAN16-A | | 100129195 |
| ENSG00000196812 | 71.1768037 | 0.006885784 | 0.1252532 | 0.8931403 | 0.956866 | ZSCAN16 | | 80345 |
| ENSG00000198315 | 929.817256 | 0.055061669 | 0.0822566 | 0.4025823 | 0.6601399 | ZKSCAN8 | | 7745 |
| ENSG00000226314 | 15.19357031 | -0.002267688 | 0.1338893 | 0.9306944 | NA | ZKSCAN8P1 | | 651302 |
| ENSG00000137185 | 217.6761302 | 0.125780306 | 0.1522681 | 0.1186882 | 0.3360962 | ZSCAN9 | | 7746 |
| ENSG00000187626 | 57.66034757 | 0.072344487 | 0.152219 | 0.1559212 | 0.3946511 | ZKSCAN4 | | 387032 |
| ENSG00000197062 | 172.0600152 | 0.022464578 | 0.1169528 | 0.7204332 | 0.8733776 | ZSCAN26 | | 7741 |
| ENSG00000137338 | 247.2316039 | 0.059633157 | 0.1162169 | 0.3610273 | 0.624302 | PGBD1 | | 84547 |
| ENSG00000235109 | 44.24362641 | -0.020595651 | 0.1312405 | 0.6245623 | 0.8202204 | ZSCAN31 | | 64288 |
| ENSG00000189298 | 66.08901628 | -0.010316419 | 0.1264472 | 0.831022 | 0.9304016 | ZKSCAN3 | | 80317 |
| ENSG00000158691 | 53.6504485 | 0.140752031 | 0.2343996 | 0.0294045 | 0.136516 | ZSCAN12 | | 9753 |
| ENSG00000232040 | 72.79523022 | 0.000503785 | 0.1252315 | 0.9936426 | 0.9976568 | SCAND3 | | 114821 |
| ENSG00000204713 | 1494.315172 | 0.079274443 | 0.0745875 | 0.2073101 | 0.4632201 | TRIM27 | | 5987 |
| ENSG00000227214 | 9.270203868 | -0.004598002 | 0.1350348 | 0.827935 | NA | NA | NA | |
| ENSG00000204681 | 296.4057613 | -0.189088345 | 0.1594152 | 0.0445091 | 0.1793413 | GABBR1 | | 2550 |
| ENSG00000204642 | 88.81887232 | 0.054526768 | 0.1350218 | 0.3407436 | 0.6080428 | HLA-F | | 3134 |
| ENSG00000214922 | 38.07041761 | -0.009996369 | 0.1304546 | 0.8017224 | NA | HLA-F-AS1 | | 285830 |
| ENSG00000273340 | 17.14804047 | 0.01444314 | 0.1346608 | 0.6128962 | NA | NA | NA | |
| ENSG00000230521 | 12.61877831 | 0.00448493 | 0.1344984 | 0.8561474 | NA | NA | NA | |
| ENSG00000206341 | 68.68526816 | 0.052172863 | 0.1375303 | 0.320174 | 0.5888762 | NA | NA | |
| ENSG00000206503 | 4242.486763 | 0.319298159 | 0.0668488 | 2.65E-07 | 1.08E-05 | HLA-A | | 3105 |
| ENSG00000227766 | 44.29863762 | -0.002064323 | 0.1300955 | 0.9568069 | 0.982546 | NA | NA | |
| ENSG00000204623 | 130.5863547 | 0.033169314 | 0.1225628 | 0.5818432 | 0.7933361 | POLR1HASI | | 80862 |
| ENSG00000237669 | 12.81012419 | 0.006120151 | 0.1345261 | 0.8064731 | NA | NA | NA | |
| ENSG00000066379 | 189.6909905 | -0.038763811 | 0.1167663 | 0.554813 | 0.7749708 | POLR1H | | 30834 |
| ENSG00000204619 | 982.8912427 | 0.089487113 | 0.0860294 | 0.1937552 | 0.4456981 | PPP1R11 | | 6992 |
| ENSG00000204618 | 8.527975399 | 0.005745533 | 0.135246 | 0.7819191 | NA | RNF39 | | 80352 |
| ENSG00000234127 | 1772.779881 | 0.241499882 | 0.083354 | 0.000789 | 0.0092445 | TRIM26 | | 7726 |
| ENSG00000243753 | 92.13458262 | 0.083174795 | 0.1514734 | 0.1761063 | 0.4250202 | NA | NA | |
| ENSG00000231074 | 1165.062422 | 0.177679557 | 0.1057661 | 0.0272327 | 0.1288076 | HCG18 | | 414777 |
| ENSG00000204599 | 427.0185511 | 0.167741966 | 0.1305451 | 0.0537545 | 0.2024199 | TRIM39 | | 56658 |
| ENSG00000241370 | 47.71588517 | -0.033703273 | 0.1346649 | 0.4315417 | 0.6828658 | RPP21 | | 79897 |
| ENSG00000204592 | 3257.480808 | 0.293352653 | 0.0751483 | 1.58E-05 | 0.000373 | HLA-E | | 3133 |
| ENSG00000204590 | 2178.285128 | 0.085818892 | 0.0695739 | 0.1544683 | 0.3927351 | GNL1 | | 2794 |
| ENSG00000204576 | 294.1087667 | 0.061210847 | 0.1127926 | 0.3910384 | 0.6513218 | PRR3 | | 80742 |
| ENSG00000204574 | 3303.923975 | -0.044734597 | 0.0595078 | 0.4027752 | 0.6601399 | ABCF1 | | 23 |
| ENSG00000204569 | 1831.623978 | 0.044627366 | 0.0741232 | 0.4753425 | 0.715183 | PPP1R10 | | 5514 |
| ENSG00000204568 | 999.0972214 | 0.027107444 | 0.0786565 | 0.6746132 | 0.8505206 | MRPS18B | | 28973 |
| ENSG00000137343 | 16.26066397 | 0.036003617 | 0.1407226 | 0.1943447 | NA | ATAT1 | | 79969 |
| ENSG00000204564 | 226.69956 | 0.086677559 | 0.1286108 | 0.2385843 | 0.5031419 | C6orf136 | | 221545 |
| ENSG00000204560 | 1586.579834 | -0.087919756 | 0.0739808 | 0.1598312 | 0.4008134 | DHX16 | | 8449 |
| ENSG00000146112 | 1885.046987 | 0.4299372 | 0.0788077 | 4.82E-09 | 3.11E-07 | PPP1R18 | | 170954 |
| ENSG00000137404 | 197.7832404 | -0.003574905 | 0.1110137 | 0.9541039 | 0.9818419 | NRM | | 11270 |
| ENSG00000137337 | 1459.772503 | 0.048633422 | 0.0749034 | 0.4398666 | 0.6890575 | MDC1 | | 9656 |
| ENSG00000196230 | 29640.12286 | -0.160309657 | 0.0519729 | 0.0009144 | 0.0102914 | TUBB | | 203068 |
| ENSG00000272540 | 251.5100591 | -0.017761839 | 0.1297645 | 0.687085 | 0.857187 | NA | NA | |
| ENSG00000137312 | 1795.082374 | -0.158958107 | 0.0797946 | 0.0179333 | 0.0960031 | FLOT1 | | 10211 |
| ENSG00000272273 | 129.8117949 | 0.04129034 | 0.1274812 | 0.4801249 | 0.7192022 | IER3-AS1 | | 105379695 |
| ENSG00000137331 | 7111.810229 | 0.166595508 | 0.2211652 | 0.0527655 | 0.199661 | IER3 | | 8870 |
| ENSG00000204580 | 2638.388062 | -0.11065976 | 0.0695629 | 0.0660958 | 0.2322697 | DDR1 | | 780 |
| ENSG00000137411 | 1509.008466 | -0.122201399 | 0.0777937 | 0.0610505 | 0.2204422 | VARS2 | | 57176 |
| ENSG00000204540 | 40.96505492 | 0.079508361 | 0.1612639 | 0.0921188 | 0.287261 | PSORS1C1 | | 170679 |

| | | | | | | | | |
|---|---|---|---|---|---|---|---|---|
| ENSG00000204536 | 852.1637889 | 0.118029347 | 0.0945328 | 0.1066338 | 0.3150095 | CCHCR1 | | 54535 |
| ENSG00000137310 | 2523.369616 | 0.334202327 | 0.0756139 | 1.33E-06 | 4.30E-05 | TCF19 | | 6941 |
| ENSG00000204531 | 297.0840824 | -0.405301676 | 0.1842018 | 0.0019697 | 0.0186743 | POU5F1 | | 5460 |
| ENSG00000204528 | 80.21662916 | -0.27194392 | 0.4369672 | 0.017734 | 0.0951544 | NA | NA | |
| ENSG00000272501 | 31.06531365 | -0.036831275 | 0.1380367 | 0.325345 | NA | NA | NA | |
| ENSG00000206344 | 28.3299907 | 0.039795792 | 0.1403826 | 0.2493135 | NA | HCG27 | | 253018 |
| ENSG00000204525 | 1755.707263 | 0.15160758 | 0.0763963 | 0.0197782 | 0.1028623 | HLA-C | | 3107 |
| ENSG00000234745 | 666.9212133 | 0.499428807 | 0.1206728 | 2.65E-06 | 7.93E-05 | HLA-B | | 3106 |
| ENSG00000272221 | 31.9631654 | -0.06308554 | 0.1516504 | 0.1139843 | NA | NA | NA | |
| ENSG00000204520 | 1055.928264 | 0.071830121 | 0.0821413 | 0.2832421 | 0.5528072 | MICA | | 100507436 |
| ENSG00000204516 | 836.9410209 | 0.193111236 | 0.1011481 | 0.0150192 | 0.0842244 | MICB | | 4277 |
| ENSG00000198563 | 2158.738893 | 0.287546065 | 0.0751555 | 2.17E-05 | 0.000484 | DDX39B | | 7919 |
| ENSG00000204498 | 256.6607743 | 0.26544666 | 0.1849646 | 0.0163812 | 0.0896248 | NFKBIL1 | | 4795 |
| ENSG00000232810 | 819.8245384 | -0.059153273 | 0.0860327 | 0.3821709 | 0.6430622 | TNF | | 7124 |
| ENSG00000227507 | 78.07045784 | -0.360697273 | 0.4885159 | 0.0125536 | 0.074815 | LTB | | 4050 |
| ENSG00000204482 | 15.58613773 | 0.017464999 | 0.1355822 | 0.5118007 | NA | LST1 | | 7940 |
| ENSG00000204469 | 9413.461967 | -0.061348079 | 0.0594359 | 0.2503353 | 0.5163897 | PRRC2A | | 7916 |
| ENSG00000204463 | 4758.042221 | -0.043001789 | 0.0562062 | 0.4142717 | 0.6685232 | BAG6 | | 7917 |
| ENSG00000204444 | 23.72190962 | 0.010196873 | 0.1329367 | 0.7580938 | NA | APOM | | 55937 |
| ENSG00000204439 | 471.5943104 | 0.021770207 | 0.0947592 | 0.7512065 | 0.8925687 | C6orf47 | | 57827 |
| ENSG00000204438 | 315.9486134 | 0.286563582 | 0.1725788 | 0.0102764 | 0.0646065 | GPANK1 | | 7918 |
| ENSG00000204435 | 413.2643148 | -0.004179653 | 0.1043455 | 0.94932 | 0.9799405 | CSNK2B | | 1460 |
| ENSG00000240053 | 84.83702733 | 0.025484777 | 0.125985 | 0.636268 | 0.8275522 | LY6G5B | | 58496 |
| ENSG00000204428 | 12.95293561 | -0.000498362 | 0.134341 | 0.9815792 | NA | LY6G5C | | 80741 |
| ENSG00000204427 | 24.28647258 | -0.000907032 | 0.13235 | 0.9760106 | NA | ABHD16A | | 7920 |
| ENSG00000213722 | 503.6823782 | -0.188210934 | 0.1344721 | 0.0366288 | 0.1578825 | DDAH2 | | 23564 |
| ENSG00000213719 | 8012.968171 | -0.039611873 | 0.0584678 | 0.453073 | 0.6987417 | CLIC1 | | 1192 |
| ENSG00000204410 | 64.68997848 | -0.002172833 | 0.1260538 | 0.9629082 | 0.9850876 | MSH5 | | 4439 |
| ENSG00000228727 | 10.82116385 | -0.011869055 | 0.135424 | 0.6024703 | NA | SAPCD1 | | 401251 |
| ENSG00000204396 | 16.87147406 | 0.00213898 | 0.1337015 | 0.9425859 | NA | VWA7 | | 80737 |
| ENSG00000204394 | 5053.584869 | -0.071862614 | 0.0581286 | 0.1734173 | 0.4206475 | VARS1 | | 7407 |
| ENSG00000204392 | 760.1473161 | -0.088166883 | 0.0933704 | 0.2154037 | 0.473487 | LSM2 | | 57819 |
| ENSG00000204390 | 32.85347068 | 0.002550046 | 0.1309125 | 0.9479827 | NA | HSPA1L | | 3305 |
| ENSG00000204389 | 1112.523449 | -0.050887868 | 0.0839451 | 0.4456429 | 0.6937964 | HSPA1A | | 3303 |
| ENSG00000204388 | 678.7997223 | 0.022334464 | 0.0921534 | 0.7451224 | 0.8893165 | HSPA1B | | 3304 |
| ENSG00000204387 | 2381.524852 | 1.024790314 | 0.086471 | 1.21E-33 | 1.01E-30 | SNHG32 | | 50854 |
| ENSG00000204386 | 1142.009564 | 0.409375488 | 0.0965968 | 2.20E-06 | 6.71E-05 | NEU1 | | 4758 |
| ENSG00000204371 | 1495.363051 | -0.120850896 | 0.0787569 | 0.0660852 | 0.2322697 | EHMT2 | | 10919 |
| ENSG00000237080 | 8.526238378 | -0.010442928 | 0.1356571 | 0.6096833 | NA | EHMT2-AS1 | | 107986588 |
| ENSG00000204366 | 88.14553539 | -0.031748446 | 0.1281494 | 0.5498418 | 0.771065 | ZBTB12 | | 221527 |
| ENSG00000204356 | 1286.649188 | 0.157153466 | 0.0849804 | 0.0247356 | 0.1199403 | NELFE | | 7936 |
| ENSG00000204351 | 1326.825254 | 0.009876661 | 0.0734321 | 0.874255 | 0.9491371 | SKIC2 | | 6499 |
| ENSG00000204348 | 220.1507236 | 0.116473432 | 0.1441269 | 0.1407694 | 0.3702607 | DXO | | 1797 |
| ENSG00000204344 | 196.043503 | 0.018844357 | 0.1131284 | 0.7717415 | 0.9030063 | STK19 | | 8859 |
| ENSG00000168477 | 17.9971678 | 0.02443522 | 0.1364684 | 0.408533 | NA | TNXB | | 7148 |
| ENSG00000213676 | 1031.160993 | -0.11881824 | 0.0877024 | 0.0898022 | 0.2827438 | ATF6B | | 1388 |
| ENSG00000204315 | 133.1141495 | -0.0477108 | 0.1263826 | 0.4403103 | 0.6894457 | FKBPL | | 63943 |
| ENSG00000204314 | 62.57416075 | -0.072009647 | 0.1496761 | 0.1796931 | 0.4291546 | PRRT1 | | 80863 |
| ENSG00000221988 | 252.0827754 | -0.006248173 | 0.108224 | 0.9235767 | 0.9708693 | PPT2 | | 9374 |
| ENSG00000258388 | 16.28477388 | 0.01915909 | 0.1355755 | 0.4965643 | NA | PPT2-EGFL8 | | 100532746 |
| ENSG00000241404 | 29.31477416 | -0.012448925 | 0.1322854 | 0.7244863 | NA | EGFL8 | | 80864 |
| ENSG00000204310 | 879.8959692 | 0.005906464 | 0.0817167 | 0.9290003 | 0.9726302 | AGPAT1 | | 10554 |
| ENSG00000204308 | 703.7046482 | 0.009911497 | 0.0870951 | 0.8835209 | 0.9528582 | RNF5 | | 6048 |
| ENSG00000204305 | 28.19132204 | -0.018103117 | 0.1333269 | 0.6074506 | NA | AGER | | 177 |
| ENSG00000204304 | 617.3732095 | -0.183855319 | 0.1172119 | 0.0302852 | 0.1392215 | PBX2 | | 5089 |
| ENSG00000204267 | 1193.680116 | 0.050186979 | 0.0796585 | 0.4410808 | 0.690282 | TAP2 | | 6891 |
| ENSG00000204264 | 651.5025748 | 0.022405491 | 0.0902525 | 0.7466218 | 0.8896552 | PSMB8 | | 5696 |
| ENSG00000204261 | 13.63126657 | 9.01E-05 | 0.1343184 | 0.9994661 | NA | PSMB8-AS1 | | 100507463 |
| ENSG00000240065 | 364.3076219 | -0.01549606 | 0.1033511 | 0.818281 | 0.9254567 | PSMB9 | | 5698 |
| ENSG00000168394 | 1325.274121 | 0.031426984 | 0.0731724 | 0.6124255 | 0.8145914 | TAP1 | | 6890 |
| ENSG00000204256 | 6898.028841 | 0.037003645 | 0.0541115 | 0.457084 | 0.7016487 | BRD2 | | 6046 |
| ENSG00000263756 | 10.00516221 | -0.007958106 | 0.1352383 | 0.7110844 | NA | NA | NA | |
| ENSG00000204248 | 64.11013141 | 0.100265338 | 0.1741965 | 0.0840935 | 0.2709074 | COL11A2 | | 1302 |
| ENSG00000204231 | 684.7929159 | -0.01793859 | 0.0869623 | 0.788682 | 0.9116361 | RXRB | | 6257 |
| ENSG00000112473 | 2973.628056 | 0.004709781 | 0.0611801 | 0.929292 | 0.9726302 | SLC39A7 | | 7922 |
| ENSG00000204227 | 809.6908014 | -0.017254087 | 0.0824426 | 0.7924114 | 0.9130225 | RING1 | | 6015 |
| ENSG00000232940 | 13.80292283 | -0.003428343 | 0.134362 | 0.8878325 | NA | HCG25 | | 414765 |
| ENSG00000223501 | 903.2706816 | -0.082056047 | 0.0861351 | 0.228465 | 0.4897594 | VPS52 | | 6293 |
| ENSG00000231500 | 17653.98887 | -0.069149216 | 0.0534789 | 0.1581112 | 0.3980381 | RPS18 | | 6222 |
| ENSG00000235863 | 11.88894347 | -0.037154578 | 0.1419804 | 0.1259028 | NA | B3GALT4 | | 8705 |

| | | | | | | | |
|---|---|---|---|---|---|---|---|
| ENSG00000227057 | 2027.994929 | -0.066518604 | 0.0675154 | 0.2569197 | 0.5240228 | WDR46 | 9277 |
| ENSG00000204220 | 817.8287734 | -0.071222665 | 0.0935717 | 0.3124817 | 0.5818701 | PFDN6 | 10471 |
| ENSG00000237441 | 895.7871159 | -0.124518123 | 0.0957606 | 0.0907931 | 0.2847888 | RGL2 | 5863 |
| ENSG00000231925 | 3439.251202 | -0.16142831 | 0.0651359 | 0.0053469 | 0.0399537 | TAPBP | 6892 |
| ENSG00000236104 | 219.4052493 | -0.075524574 | 0.127544 | 0.2852005 | 0.5546831 | ZBTB22 | 9278 |
| ENSG00000204209 | 2038.191494 | -0.073219649 | 0.0679821 | 0.2144509 | 0.4721961 | DAXX | 1616 |
| ENSG00000237649 | 2634.72602 | -0.100050578 | 0.0653181 | 0.081499 | 0.2654739 | KIFC1 | 3833 |
| ENSG00000112511 | 570.8025949 | -0.090787912 | 0.1062968 | 0.2227837 | 0.4827186 | PHF1 | 5252 |
| ENSG00000112514 | 1196.942758 | 0.019507687 | 0.0775244 | 0.7609203 | 0.897467 | CUTA | 51596 |
| ENSG00000197283 | 223.2158156 | 0.008919177 | 0.1090037 | 0.891026 | 0.9559869 | SYNGAP1 | 8831 |
| ENSG00000213588 | 294.0380911 | 0.171689283 | 0.1777792 | 0.0604685 | 0.2190177 | ZBTB9 | 221504 |
| ENSG00000030110 | 863.2181159 | 0.058081923 | 0.0859473 | 0.3960631 | 0.6562498 | BAK1 | 578 |
| ENSG00000096433 | 6051.393462 | 0.033405556 | 0.0575038 | 0.5235961 | 0.7509978 | ITPR3 | 3710 |
| ENSG00000137288 | 943.2816765 | 0.05397986 | 0.0891592 | 0.4201536 | 0.6729486 | UQCC2 | 84300 |
| ENSG00000161904 | 935.9297935 | 0.048723596 | 0.0818766 | 0.4563103 | 0.7008758 | LEMD2 | 221496 |
| ENSG00000137309 | 16925.96664 | 0.122996755 | 0.0559553 | 0.0159632 | 0.0881221 | HMGA1 | 3159 |
| ENSG00000186577 | 273.8219314 | 0.11251249 | 0.1339797 | 0.1548561 | 0.3932335 | SMIM29 | 221491 |
| ENSG00000225339 | 92.5563662 | 0.009528897 | 0.1233353 | 0.8601075 | 0.9435431 | NA | NA |
| ENSG00000272325 | 1402.893891 | -0.133231448 | 0.0869905 | 0.0574066 | 0.2120034 | NUDT3 | 11165 |
| ENSG00000124614 | 216.7991552 | -0.110243364 | 0.1477429 | 0.1490686 | 0.3838779 | RPS10 | 6204 |
| ENSG00000196821 | 1936.395225 | 0.087051916 | 0.0777087 | 0.1781018 | 0.4271872 | ILRUN | 64771 |
| ENSG00000272288 | 14.71152737 | 0.01782612 | 0.1356361 | 0.5046189 | NA | ILRUN-AS1 | 101929243 |
| ENSG00000124562 | 1621.93183 | -0.07093324 | 0.072211 | 0.2458134 | 0.5114617 | SNRPC | 6631 |
| ENSG00000065060 | 1523.358782 | 0.048499886 | 0.0734048 | 0.4348778 | 0.6853902 | BLTP3A | 54887 |
| ENSG00000064995 | 480.9770625 | -0.189154583 | 0.1277766 | 0.0323969 | 0.1452182 | TAF11 | 6882 |
| ENSG00000064999 | 832.140353 | -0.064231827 | 0.0875265 | 0.3473983 | 0.6135409 | ANKS1A | 23294 |
| ENSG00000272374 | 25.94947216 | 0.007915868 | 0.1325637 | 0.8148526 | NA | NA | NA |
| ENSG00000065029 | 478.7028404 | 0.089768002 | 0.1056287 | 0.2205564 | 0.4802982 | ZNF76 | 7629 |
| ENSG00000112033 | 651.2722639 | -0.013507745 | 0.0900436 | 0.8413655 | 0.9347527 | PPARD | 5467 |
| ENSG00000112039 | 321.1401158 | 0.001419811 | 0.1015387 | 0.9842108 | 0.9940394 | FANCE | 2178 |
| ENSG00000198755 | 8814.447067 | -0.129600414 | 0.0587437 | 0.0129148 | 0.0761142 | RPL10A | 4736 |
| ENSG00000007866 | 273.523082 | 0.058609095 | 0.1144484 | 0.4055585 | 0.6617305 | TEAD3 | 7005 |
| ENSG00000096060 | 1609.129042 | 0.052850996 | 0.0706633 | 0.3832644 | 0.6439746 | FKBP5 | 2289 |
| ENSG00000197753 | 8.601852181 | 0.012029003 | 0.1358342 | 0.5631108 | NA | LHFPL5 | 222662 |
| ENSG00000096063 | 4165.726514 | 0.003638526 | 0.0561909 | 0.9439416 | 0.9784014 | SRPK1 | 6732 |
| ENSG00000112062 | 1447.725034 | -0.117553043 | 0.0781492 | 0.0721343 | 0.2452272 | MAPK14 | 1432 |
| ENSG00000156711 | 182.9183931 | -0.047901636 | 0.1194625 | 0.4697227 | 0.7112787 | MAPK13 | 5603 |
| ENSG00000096070 | 1591.02909 | 0.13332064 | 0.0804538 | 0.0462474 | 0.1839973 | BRPF3 | 27154 |
| ENSG00000112078 | 1491.67902 | -0.238367207 | 0.0905042 | 0.0017303 | 0.0170566 | KCTD20 | 222658 |
| ENSG00000112079 | 1131.9393 | -0.0705906 | 0.082927 | 0.2901782 | 0.560019 | STK38 | 11329 |
| ENSG00000112081 | 8456.140316 | -0.32682692 | 0.066903 | 1.46E-07 | 6.55E-06 | SRSF3 | 6428 |
| ENSG00000124762 | 2666.169989 | 0.303930911 | 0.0801353 | 2.20E-05 | 0.0004896 | CDKN1A | 1026 |
| ENSG00000137168 | 769.1086125 | -0.027758856 | 0.0870334 | 0.6792714 | 0.8535466 | PPIL1 | 51645 |
| ENSG00000198663 | 1555.571379 | 0.071325172 | 0.0735148 | 0.2520015 | 0.5184753 | C6orf89 | 221477 |
| ENSG00000137409 | 4084.170667 | 0.073416535 | 0.0600721 | 0.173148 | 0.4203584 | MTCH1 | 23787 |
| ENSG00000137193 | 183.1649127 | 1.048313736 | 0.226717 | 1.93E-07 | 8.24E-06 | PIM1 | 5292 |
| ENSG00000065491 | 376.0684678 | -0.006140206 | 0.0983548 | 0.9275356 | 0.9720836 | TBC1D22B | 55633 |
| ENSG00000112130 | 640.9414828 | -0.016865504 | 0.090065 | 0.802745 | 0.9177708 | RNF8 | 9025 |
| ENSG00000137200 | 1501.999623 | -0.011202119 | 0.0708197 | 0.8526814 | 0.9399048 | CMTR1 | 23070 |
| ENSG00000198937 | 345.7694323 | -0.157102631 | 0.1415698 | 0.0713372 | 0.2433566 | CCDC167 | 154467 |
| ENSG00000112139 | 12.92648909 | 0.020566744 | 0.1366816 | 0.4006772 | NA | MDGA1 | 266727 |
| ENSG00000156639 | 1608.154666 | 0.15815085 | 0.0816066 | 0.0203905 | 0.1051576 | ZFAND3 | 60685 |
| ENSG00000183826 | 341.9490458 | -0.067917698 | 0.1114599 | 0.3470334 | 0.6134112 | BTBD9 | 114781 |
| ENSG00000124767 | 4784.092652 | -0.119188861 | 0.0661645 | 0.0402911 | 0.1683264 | GLO1 | 2739 |
| ENSG00000112167 | 177.6616215 | 0.033165556 | 0.1171637 | 0.6072156 | 0.8116146 | SAYSD1 | 55776 |
| ENSG00000124615 | 280.2330885 | 0.077102151 | 0.1180605 | 0.2947349 | 0.5650223 | MOCS1 | 4337 |
| ENSG00000124596 | 258.6014248 | 0.046879627 | 0.1119723 | 0.4983197 | 0.7335693 | OARD1 | 221443 |
| ENSG00000001167 | 779.1998302 | -0.005597106 | 0.0831904 | 0.9315569 | 0.973389 | NFYA | 4800 |
| ENSG00000161912 | 38.78844119 | 0.015362273 | 0.1315616 | 0.6962065 | NA | NA | NA |
| ENSG00000234753 | 16.92851106 | 0.01941886 | 0.1355393 | 0.4961481 | NA | FOXP4-AS1 | 101060264 |
| ENSG00000137166 | 894.8743313 | 0.056254871 | 0.086247 | 0.4051222 | 0.6614808 | FOXP4 | 116113 |
| ENSG00000112561 | 78.43952953 | 0.096514734 | 0.1651267 | 0.1174286 | 0.3339561 | TFEB | 7942 |
| ENSG00000137218 | 83.3512219 | 0.060685132 | 0.1413467 | 0.2619545 | 0.5296677 | FRS3 | 10817 |
| ENSG00000124593 | 156.0034249 | -0.02840035 | 0.1180262 | 0.6490633 | 0.8366057 | NA | NA |
| ENSG00000164663 | 280.6568249 | 0.122613659 | 0.1378334 | 0.1304089 | 0.3549176 | USP49 | 25862 |
| ENSG00000124641 | 506.2728094 | 0.032513787 | 0.0947379 | 0.6412605 | 0.8315061 | MED20 | 9477 |
| ENSG00000112578 | 1534.241227 | 0.147256861 | 0.0826636 | 0.0314821 | 0.1423705 | BYSL | 705 |
| ENSG00000112576 | 1131.375556 | 0.110424348 | 0.0836222 | 0.1053532 | 0.3123429 | CCND3 | 896 |
| ENSG00000137413 | 553.6423535 | -0.039511493 | 0.0925741 | 0.565724 | 0.782102 | TAF8 | 129685 |
| ENSG00000188112 | 160.9629496 | 0.708674226 | 0.2320967 | 0.0001126 | 0.001923 | C6orf132 | 647024 |

| | | | | | | | | |
|---|---|---|---|---|---|---|---|---|
| ENSG00000112599 | 41.68110579 | 0.165112111 | 0.3109057 | 0.0107689 | 0.0668037 | GUCA1B | | 2979 |
| ENSG00000048544 | 1311.930286 | -0.034025344 | 0.08009 | 0.6003641 | 0.8069949 | MRPS10 | | 55173 |
| ENSG00000124496 | 623.0565116 | -0.302964665 | 0.1281334 | 0.0022193 | 0.0204734 | TRERF1 | | 55809 |
| ENSG00000024048 | 946.9073469 | -0.014831425 | 0.0800048 | 0.8183971 | 0.9254567 | UBR2 | | 23304 |
| ENSG00000124659 | 389.3415771 | 0.104149219 | 0.1230427 | 0.1797535 | 0.429211 | TBCC | | 6903 |
| ENSG00000112624 | 471.9364498 | -0.093846744 | 0.1086168 | 0.2113709 | 0.4681824 | BICRAL | | 23506 |
| ENSG00000146223 | 4395.995153 | -0.034586505 | 0.0569865 | 0.5048143 | 0.7381515 | RPL7L1 | | 285855 |
| ENSG00000221821 | 69.85170318 | -0.072422016 | 0.1491305 | 0.1857566 | 0.4358915 | C6orf226 | | 441150 |
| ENSG00000137161 | 922.6836072 | 0.015282864 | 0.0799611 | 0.8145625 | 0.9241103 | CNPY3 | | 10695 |
| ENSG00000231113 | 40.47333752 | -0.029422636 | 0.1342822 | 0.466117 | NA | NA | NA | |
| ENSG00000124587 | 53.04164316 | 0.052538586 | 0.1408405 | 0.2709395 | 0.5386338 | PEX6 | | 5190 |
| ENSG00000112640 | 2082.670926 | -0.112441989 | 0.0712163 | 0.0662448 | 0.232594 | PPP2R5D | | 5528 |
| ENSG00000124733 | 1051.493639 | -0.014508874 | 0.0806211 | 0.8228101 | 0.9272789 | MEA1 | | 4201 |
| ENSG00000124702 | 1396.186737 | -0.082929142 | 0.0767953 | 0.1960081 | 0.4487595 | KLHDC3 | | 116138 |
| ENSG00000124541 | 1475.581679 | -0.042654715 | 0.0719675 | 0.4860421 | 0.7238885 | RRP36 | | 88745 |
| ENSG00000044090 | 862.4577497 | 0.006757222 | 0.0843304 | 0.9198115 | 0.9690041 | CUL7 | | 9820 |
| ENSG00000137171 | 224.0580565 | -0.005647584 | 0.1094829 | 0.9307431 | 0.972984 | KLC4 | | 89953 |
| ENSG00000112651 | 796.6578215 | 0.230251584 | 0.1057472 | 0.0059613 | 0.0432399 | MRPL2 | | 51069 |
| ENSG00000112655 | 1135.37078 | -0.041690987 | 0.0835033 | 0.5269956 | 0.7541143 | PTK7 | | 5754 |
| ENSG00000112658 | 1726.331918 | -0.064368936 | 0.0711618 | 0.2897919 | 0.5598841 | SRF | | 6722 |
| ENSG00000112659 | 736.0976209 | -0.013482691 | 0.0847428 | 0.8385647 | 0.9334166 | CUL9 | | 23113 |
| ENSG00000112667 | 451.9882303 | 0.038781802 | 0.1015281 | 0.5778076 | 0.7911317 | DNPH1 | | 10591 |
| ENSG00000171467 | 739.4133027 | -0.070630609 | 0.0916892 | 0.3125018 | 0.5818701 | ZNF318 | | 24149 |
| ENSG00000124574 | 645.8186368 | 0.085764937 | 0.0973891 | 0.2366448 | 0.5009511 | ABCC10 | | 89845 |
| ENSG00000171462 | 110.8741949 | -0.117115324 | 0.1716377 | 0.1060628 | 0.3138889 | DLK2 | | 65989 |
| ENSG00000137221 | 909.4224014 | 0.144810723 | 0.0987472 | 0.0563981 | 0.2092051 | TJAP1 | | 93643 |
| ENSG00000171453 | 1251.540968 | 0.206953833 | 0.0902837 | 0.0056008 | 0.0412779 | POLR1C | | 9533 |
| ENSG00000137207 | 778.1444416 | 0.01320709 | 0.0831796 | 0.8423439 | 0.9350382 | YIPF3 | | 25844 |
| ENSG00000271754 | 34.10783659 | 0.030550664 | 0.1352491 | 0.4348135 | NA | NA | NA | |
| ENSG00000124571 | 2422.850379 | 0.004231031 | 0.0636628 | 0.9409462 | 0.976977 | XPO5 | | 57510 |
| ENSG00000271970 | 8.389791486 | -0.009637377 | 0.1358676 | 0.6041761 | NA | NA | NA | |
| ENSG00000170734 | 434.1144444 | 0.27524794 | 0.1444916 | 0.0073215 | 0.0504261 | POLH | | 5429 |
| ENSG00000172432 | 1131.279392 | -0.013663138 | 0.0754156 | 0.8272964 | 0.9287505 | GTPBP2 | | 54676 |
| ENSG00000124688 | 569.8782941 | 0.09590335 | 0.1021819 | 0.1963338 | 0.4490367 | MAD2L1BP | | 9587 |
| ENSG00000096080 | 631.5539719 | -0.066691428 | 0.0974231 | 0.3484012 | 0.6143697 | MRPS18A | | 55168 |
| ENSG00000112715 | 1351.903192 | 0.406247805 | 0.0875257 | 3.53E-07 | 1.38E-05 | VEGFA | | 7422 |
| ENSG00000237686 | 15.29604025 | 0.017344952 | 0.1357757 | 0.4982762 | NA | SCIRT | | 101929705 |
| ENSG00000180992 | 1414.924925 | -0.213583863 | 0.0849437 | 0.0029674 | 0.0255059 | MRPL14 | | 64928 |
| ENSG00000137216 | 689.6382879 | -0.140835859 | 0.1047807 | 0.069756 | 0.2401473 | TMEM63B | | 55362 |
| ENSG00000112759 | 2045.21936 | 0.03672803 | 0.0660493 | 0.5307111 | 0.7563734 | SLC29A1 | | 2030 |
| ENSG00000096384 | 42008.54614 | -0.338447166 | 0.0510886 | 4.35E-12 | 4.88E-10 | HSP90AB1 | | 3326 |
| ENSG00000157593 | 1116.256199 | -0.020932883 | 0.0761708 | 0.7400598 | 0.886266 | SLC35B2 | | 347734 |
| ENSG00000146232 | 561.1452741 | 0.18734536 | 0.1215749 | 0.0305517 | 0.1401158 | NFKBIE | | 4794 |
| ENSG00000124608 | 679.7948987 | -0.00835711 | 0.0866608 | 0.8998681 | 0.9607722 | AARS2 | | 57505 |
| ENSG00000096401 | 2058.181059 | 0.035638604 | 0.0678529 | 0.5443108 | 0.7664048 | CDC5L | | 988 |
| ENSG00000196284 | 97.07296842 | -0.03450499 | 0.1271728 | 0.5350142 | 0.7594484 | SUPT3H | | 8464 |
| ENSG00000124813 | 17.38004246 | 0.031291606 | 0.1386446 | 0.2825854 | NA | RUNX2 | | 860 |
| ENSG00000001561 | 613.1060136 | 0.004542936 | 0.090603 | 0.9490968 | 0.9798833 | ENPP4 | | 22875 |
| ENSG00000146072 | 257.0118331 | 0.286048601 | 0.1888571 | 0.0126583 | 0.0751709 | TNFRSF21 | | 27242 |
| ENSG00000198087 | 1978.222179 | -0.124874902 | 0.0748605 | 0.0494659 | 0.1913249 | CD2AP | | 23607 |
| ENSG00000146085 | 626.7324503 | 0.004915449 | 0.0905796 | 0.9463419 | 0.9785959 | MMUT | | 4594 |
| ENSG00000031691 | 316.3313819 | 0.139446348 | 0.1516474 | 0.0979014 | 0.2981973 | CENPQ | | 55166 |
| ENSG00000112118 | 5555.700505 | -0.013723528 | 0.0545781 | 0.7839315 | 0.9098848 | MCM3 | | 4172 |
| ENSG00000170915 | 112.5255052 | 0.040781226 | 0.1261076 | 0.4929014 | 0.7286234 | PAQR8 | | 85315 |
| ENSG00000096093 | 356.098189 | 0.043099844 | 0.1079326 | 0.5357116 | 0.7600689 | EFHC1 | | 114327 |
| ENSG00000065308 | 1145.662538 | -0.109134721 | 0.0838091 | 0.1081521 | 0.3183334 | TRAM2 | | 9697 |
| ENSG00000225791 | 66.27310927 | 0.032235569 | 0.1303631 | 0.5218357 | 0.7494387 | TRAM2-AS1 | | 401264 |
| ENSG00000216775 | 164.7684222 | -0.043445706 | 0.1207274 | 0.5004555 | 0.7347747 | NA | NA | |
| ENSG00000096092 | 474.8960752 | 0.041264116 | 0.0972589 | 0.5545889 | 0.7749708 | TMEM14A | | 28978 |
| ENSG00000202198 | 57.6687723 | -0.016108887 | 0.1373 | 0.2756684 | 0.5439328 | RN7SK | | 125050 |
| ENSG00000112144 | 179.7917723 | 0.001972882 | 0.1130093 | 0.9765199 | 0.9912634 | CILK1 | | 22858 |
| ENSG00000112146 | 872.0121802 | -0.084312858 | 0.0893579 | 0.225844 | 0.4868367 | FBXO9 | | 26268 |
| ENSG00000012660 | 3033.37148 | -0.108914051 | 0.0634942 | 0.0528703 | 0.1999281 | ELOVL5 | | 60481 |
| ENSG00000001084 | 1033.578366 | -0.075600555 | 0.0880386 | 0.2720146 | 0.5395487 | GCLC | | 2729 |
| ENSG00000249379 | 17.67317112 | -0.015033731 | 0.1348699 | 0.5879069 | NA | NA | NA | |
| ENSG00000137269 | 323.1905914 | 0.000188599 | 0.1020522 | 0.9990733 | 0.9993299 | LRRC1 | | 55227 |
| ENSG00000137251 | 248.4025903 | -0.813505261 | 0.1835027 | 4.96E-07 | 1.87E-05 | TINAG | | 27283 |
| ENSG00000168143 | 98.55368838 | 0.083720891 | 0.1489698 | 0.1888108 | 0.4396927 | FAM83B | | 222584 |
| ENSG00000261116 | 50.37901647 | 0.007782372 | 0.1286055 | 0.8620845 | 0.9447423 | NA | NA | |
| ENSG00000151914 | 5001.714366 | -0.01384206 | 0.05478 | 0.7821623 | 0.9095461 | DST | | 667 |

| | | | | | | | | |
|---|---|---|---|---|---|---|---|---|
| ENSG00000168116 | 282.0775645 | -0.00935382 | 0.105452 | 0.8882436 | 0.9545517 | KIAA1586 | | 57691 |
| ENSG00000112200 | 1403.000689 | 0.091064282 | 0.0807674 | 0.169247 | 0.4149819 | ZNF451 | | 26036 |
| ENSG00000226803 | 17.09913349 | -0.021194829 | 0.1358887 | 0.4572401 | NA | NA | NA | |
| ENSG00000112208 | 775.2933858 | -0.117811961 | 0.0972705 | 0.1105158 | 0.3222505 | BAG2 | | 9532 |
| ENSG00000112210 | 604.0579053 | 0.043041353 | 0.0913541 | 0.5334186 | 0.7583815 | RAB23 | | 51715 |
| ENSG00000146143 | 546.9630744 | 0.0817037 | 0.102958 | 0.2734111 | 0.5416478 | PRIM2 | | 5558 |
| ENSG00000272316 | 23.13149973 | 0.02144053 | 0.1351642 | 0.4997241 | NA | NA | NA | |
| ENSG00000215190 | 168.6865089 | 0.211732515 | 0.2199078 | 0.036922 | 0.1587321 | NA | NA | |
| ENSG00000198225 | 11.43595258 | -0.013856309 | 0.1355624 | 0.557227 | NA | FKBP1C | | 642489 |
| ENSG00000112245 | 2198.456326 | 0.109100424 | 0.0688947 | 0.0686523 | 0.2375377 | PTP4A1 | | 7803 |
| ENSG00000118482 | 1695.272779 | 0.032971982 | 0.0712488 | 0.5885964 | 0.7984432 | PHF3 | | 23469 |
| ENSG00000168216 | 312.0711311 | -0.073600478 | 0.1151682 | 0.3124476 | 0.5818701 | LMBRD1 | | 55788 |
| ENSG00000082269 | 1558.042247 | -0.057026279 | 0.0748686 | 0.3645383 | 0.6271239 | FAM135A | | 57579 |
| ENSG00000154079 | 67.85658673 | 0.037613292 | 0.1316674 | 0.4644585 | 0.7082689 | SDHAF4 | | 135154 |
| ENSG00000112305 | 670.8705302 | -0.040593496 | 0.0883682 | 0.5478658 | 0.7691852 | SMAP1 | | 60682 |
| ENSG00000112309 | 36.17856684 | -0.067990492 | 0.1540768 | 0.1092001 | NA | B3GAT2 | | 135152 |
| ENSG00000232295 | 42.32595457 | -0.015333068 | 0.1306798 | 0.7096203 | 0.8682712 | NA | NA | |
| ENSG00000119900 | 10397.39432 | 0.030371805 | 0.0588751 | 0.5817734 | 0.7933361 | OGFRL1 | | 79627 |
| ENSG00000079841 | 184.7075012 | -3.27E-05 | 0.112785 | 0.9983658 | 0.9989644 | RIMS1 | | 22999 |
| ENSG00000135314 | 205.3067662 | -0.025632196 | 0.1142821 | 0.6908456 | 0.8593086 | KHDC1 | | 80759 |
| ENSG00000229852 | 32.55157917 | 0.028943418 | 0.1356573 | 0.4281333 | NA | KHDC1-AS1 | | 122539212 |
| ENSG00000235174 | 42.50533986 | -0.038271702 | 0.1364336 | 0.3704862 | 0.6328256 | NA | NA | |
| ENSG00000164430 | 1089.609696 | 0.26702289 | 0.0953645 | 0.0008755 | 0.0099868 | CGAS | | 115004 |
| ENSG00000135297 | 580.3506912 | 0.140368537 | 0.1103645 | 0.0776577 | 0.2569745 | MTO1 | | 25821 |
| ENSG00000156508 | 175516.632 | -0.166146435 | 0.0477304 | 0.0002321 | 0.0034763 | EEF1A1 | | 1915 |
| ENSG00000119899 | 345.3889955 | -0.014027839 | 0.1014777 | 0.8361038 | 0.9319207 | SLC17A5 | | 26503 |
| ENSG00000156535 | 1614.523508 | -0.086021563 | 0.0774354 | 0.1821825 | 0.431833 | CD109 | | 135228 |
| ENSG00000111799 | 4608.365373 | -0.418922573 | 0.1769747 | 0.0012558 | 0.0134084 | COL12A1 | | 1303 |
| ENSG00000112695 | 2031.48564 | -0.191344207 | 0.0778963 | 0.0042477 | 0.0334121 | COX7A2 | | 1347 |
| ENSG00000112697 | 3243.133748 | -0.13320672 | 0.0647479 | 0.0202078 | 0.1045381 | TMEM30A | | 55754 |
| ENSG00000112701 | 1378.529526 | -0.181704077 | 0.0843423 | 0.0098753 | 0.0631382 | SENP6 | | 26054 |
| ENSG00000196586 | 1221.341486 | -0.136529884 | 0.0839878 | 0.0471866 | 0.1857336 | MYO6 | | 4646 |
| ENSG00000146243 | 98.56788337 | -0.091893317 | 0.1583172 | 0.1417262 | 0.3720234 | IRAK1BP1 | | 134728 |
| ENSG00000146247 | 2555.807091 | -0.09798403 | 0.0673572 | 0.0951449 | 0.2928559 | PHIP | | 55023 |
| ENSG00000118418 | 666.1005641 | -0.290090161 | 0.1254176 | 0.0027123 | 0.0238991 | HMGN3 | | 9324 |
| ENSG00000270362 | 38.16263074 | -0.052984908 | 0.1440953 | 0.2050019 | NA | HMGN3-AS1 | | 100288198 |
| ENSG00000135338 | 111.3614169 | -0.011751369 | 0.1209695 | 0.8352964 | 0.931409 | LCA5 | | 167691 |
| ENSG00000198478 | 267.3380498 | 0.080569636 | 0.1219087 | 0.2719931 | 0.5395487 | SH3BGRL2 | | 83699 |
| ENSG00000118402 | 89.52072482 | 0.000130882 | 0.1233927 | 0.9988952 | 0.999323 | ELOVL4 | | 6785 |
| ENSG00000112742 | 1420.245443 | -0.224272277 | 0.0892578 | 0.002713 | 0.0238991 | TTK | | 7272 |
| ENSG00000083123 | 307.4548214 | -0.111577648 | 0.129879 | 0.1566272 | 0.3956066 | BCKDHB | | 594 |
| ENSG00000112773 | 70.18259746 | -0.066894488 | 0.1452015 | 0.2184809 | 0.4783119 | TENT5A | | 55603 |
| ENSG00000005700 | 1421.958621 | 0.099777494 | 0.0769024 | 0.1213124 | 0.3410469 | IBTK | | 25998 |
| ENSG00000146242 | 837.6386938 | -0.115674533 | 0.0976841 | 0.117393 | 0.3339561 | TPBG | | 7162 |
| ENSG00000118420 | 193.6440239 | 0.017228394 | 0.1135782 | 0.789525 | 0.9116361 | UBE3D | | 90025 |
| ENSG00000083097 | 362.3112506 | -0.165928815 | 0.1434131 | 0.0615192 | 0.2214309 | DOP1A | | 23033 |
| ENSG00000013375 | 1064.748532 | -0.048086019 | 0.0787162 | 0.4589317 | 0.7032377 | PGM3 | | 5238 |
| ENSG00000013392 | 161.4433014 | -0.018746916 | 0.1160105 | 0.7635312 | 0.8982943 | RWDD2A | | 112611 |
| ENSG00000065833 | 585.6930326 | -0.048373848 | 0.0977978 | 0.4893994 | 0.7263574 | ME1 | | 4199 |
| ENSG00000146250 | 35.00775094 | 0.001631045 | 0.1359742 | 0.9114803 | NA | PRSS35 | | 167681 |
| ENSG00000203877 | 26.03220678 | 0.008335984 | 0.1333007 | 0.7894517 | NA | RIPPLY2 | | 134701 |
| ENSG00000065615 | 709.4770511 | -0.155085515 | 0.1041522 | 0.0476866 | 0.1866714 | CYB5R4 | | 51167 |
| ENSG00000135324 | 54.27142181 | -0.053756637 | 0.1407829 | 0.2694837 | 0.537356 | MRAP2 | | 112609 |
| ENSG00000135315 | 340.7421829 | 0.037353926 | 0.1066519 | 0.5892563 | 0.7987818 | CEP162 | | 22832 |
| ENSG00000234155 | 27.71501942 | -0.017410447 | 0.1341422 | 0.5880102 | NA | LINC02535 | | 101928820 |
| ENSG00000135318 | 634.9626257 | 0.67077763 | 0.1278751 | 1.01E-08 | 6.03E-07 | NT5E | | 4907 |
| ENSG00000135317 | 1108.773652 | -0.270858115 | 0.0954492 | 0.0007672 | 0.0090339 | SNX14 | | 57231 |
| ENSG00000135316 | 6565.274105 | -0.232369757 | 0.0607725 | 3.18E-05 | 0.0006725 | SYNCRIP | | 10492 |
| ENSG00000203875 | 750.202489 | 0.528603655 | 0.1151891 | 3.29E-07 | 1.31E-05 | SNHG5 | | 387066 |
| ENSG00000188994 | 1068.826664 | 0.00800486 | 0.0807071 | 0.9030516 | 0.9618565 | ZNF292 | | 23036 |
| ENSG00000164411 | 54.94901249 | -0.02357264 | 0.130668 | 0.5980406 | 0.8058268 | GJB7 | | 375519 |
| ENSG00000111850 | 111.5062256 | 0.000980095 | 0.1204789 | 0.9859696 | 0.9942441 | SMIM8 | | 57150 |
| ENSG00000226524 | 10.39635651 | 0.00353255 | 0.1349196 | 0.8743772 | NA | NA | NA | |
| ENSG00000203872 | 21.23736667 | 0.005516351 | 0.1330922 | 0.8599812 | NA | C6orf163 | | 206412 |
| ENSG00000213204 | 9.277932893 | -0.023544446 | 0.1380835 | 0.2547924 | NA | NA | NA | |
| ENSG00000164414 | 102.2657684 | -0.02730585 | 0.1237828 | 0.6318088 | 0.8251856 | SLC35A1 | | 10559 |
| ENSG00000146282 | 680.9185177 | -0.163719833 | 0.1143623 | 0.0469089 | 0.1853035 | RARS2 | | 57038 |
| ENSG00000135336 | 894.03755 | -0.044165827 | 0.0831513 | 0.5048476 | 0.7381515 | ORC3 | | 23595 |
| ENSG00000135334 | 641.0848109 | 0.293944896 | 0.1203819 | 0.0019486 | 0.0185504 | AKIRIN2 | | 55122 |
| ENSG00000111880 | 847.1203295 | -0.032666615 | 0.0855601 | 0.6254199 | 0.8204773 | RNGTT | | 8732 |

| | | | | | | | | |
|---|---|---|---|---|---|---|---|---|
| ENSG00000146278 | 474.3582671 | 0.013581519 | 0.0974449 | 0.8425055 | 0.9350382 | PNRC1 | | 10957 |
| ENSG00000154548 | 127.1698252 | -0.041799505 | 0.1247339 | 0.493385 | 0.7288952 | SRSF12 | | 135295 |
| ENSG00000146281 | 1581.458054 | 0.109999275 | 0.0766679 | 0.0877243 | 0.278529 | PM20D2 | | 135293 |
| ENSG00000198833 | 1457.831062 | -0.173290888 | 0.0823633 | 0.0120963 | 0.0727209 | UBE2J1 | | 51465 |
| ENSG00000025039 | 363.3285572 | 0.84295946 | 0.150716 | 1.26E-09 | 9.01E-08 | RRAGD | | 58528 |
| ENSG00000135299 | 313.2656298 | 0.033522542 | 0.1049063 | 0.6279045 | 0.8226281 | ANKRD6 | | 22881 |
| ENSG00000083099 | 957.5452628 | -0.064853042 | 0.084309 | 0.3309729 | 0.5989207 | LYRM2 | | 57226 |
| ENSG00000112159 | 5687.428073 | 0.108694704 | 0.0621942 | 0.049789 | 0.1918791 | MDN1 | | 23195 |
| ENSG00000118412 | 1287.302946 | 0.145336112 | 0.0879688 | 0.0410137 | 0.1703096 | CASP8AP2 | | 9994 |
| ENSG00000135341 | 1142.566549 | -0.082309307 | 0.0824394 | 0.2175582 | 0.4768298 | MAP3K7 | | 6885 |
| ENSG00000172469 | 288.5510698 | -0.26889742 | 0.1752486 | 0.0139189 | 0.0803595 | MANEA | | 79694 |
| ENSG00000172461 | 45.5256885 | 0.037592296 | 0.1356207 | 0.3950083 | 0.6553177 | FUT9 | | 10690 |
| ENSG00000014123 | 331.6406712 | -0.240630321 | 0.1865744 | 0.0233738 | 0.1153771 | UFL1 | | 23376 |
| ENSG00000112218 | 124.2279414 | 0.025996898 | 0.1209065 | 0.6641522 | 0.8443298 | GPR63 | | 81491 |
| ENSG00000123545 | 448.608625 | 0.04120045 | 0.0996225 | 0.5557859 | 0.7756434 | NDUFAF4 | | 29078 |
| ENSG00000146263 | 905.0133005 | 0.095596156 | 0.0878964 | 0.1697221 | 0.4155362 | MMS22L | | 253714 |
| ENSG00000112234 | 198.30032 | 0.034237968 | 0.1146386 | 0.6048385 | 0.8100343 | FBXL4 | | 26235 |
| ENSG00000146267 | 39.66699309 | 0.056378709 | 0.145355 | 0.1957784 | NA | FAXC | | 84553 |
| ENSG00000132423 | 156.0629751 | -0.178487395 | 0.205925 | 0.0519596 | 0.1971865 | COQ3 | | 51805 |
| ENSG00000132424 | 1577.472782 | -0.110480266 | 0.0807018 | 0.0970095 | 0.2962532 | PNISR | | 25957 |
| ENSG00000228506 | 64.4363685 | 0.019882386 | 0.1279317 | 0.6853178 | 0.8560726 | NA | NA | |
| ENSG00000123552 | 349.949609 | 0.235734003 | 0.1681368 | 0.0222202 | 0.1115282 | USP45 | | 85015 |
| ENSG00000228439 | 38.45396926 | -0.018910862 | 0.1316614 | 0.6388365 | NA | NA | NA | |
| ENSG00000112237 | 978.7851326 | -0.193800834 | 0.098557 | 0.0132332 | 0.0774047 | CCNC | | 892 |
| ENSG00000112249 | 1326.209506 | 0.02493343 | 0.0733566 | 0.6882574 | 0.858008 | ASCC3 | | 10973 |
| ENSG00000260000 | 12.39404645 | -0.017268619 | 0.1360682 | 0.4704694 | NA | NA | NA | |
| ENSG00000085382 | 257.2487748 | 0.034677984 | 0.1095567 | 0.6104236 | 0.8133179 | HACE1 | | 57531 |
| ENSG00000132429 | 14.28817951 | 6.40E-05 | 0.134049 | 0.9996802 | NA | POPDC3 | | 64208 |
| ENSG00000085377 | 729.3390223 | -0.154860835 | 0.1030213 | 0.046576 | 0.1844877 | PREP | | 5550 |
| ENSG00000057663 | 522.4647447 | -0.001974648 | 0.0911876 | 0.9758345 | 0.9911001 | ATG5 | | 9474 |
| ENSG00000130347 | 169.2789202 | -0.247288657 | 0.2375002 | 0.0254262 | 0.1223242 | RTN4IP1 | | 84816 |
| ENSG00000130348 | 361.2977951 | 0.020495954 | 0.1000875 | 0.7664148 | 0.8994294 | QRSL1 | | 55278 |
| ENSG00000130349 | 98.18759334 | 0.093073759 | 0.1593453 | 0.1399181 | 0.3693538 | MTRES1 | | 51250 |
| ENSG00000178409 | 243.5408101 | -0.059040051 | 0.1169929 | 0.3973566 | 0.6567942 | BEND3 | | 57673 |
| ENSG00000164494 | 183.1919863 | -0.01688116 | 0.1135589 | 0.7906511 | 0.9122112 | PDSS2 | | 57107 |
| ENSG00000112320 | 88.71471969 | 0.052774003 | 0.1340769 | 0.3557874 | 0.6192724 | SOBP | | 55084 |
| ENSG00000025796 | 1634.173698 | 0.065208566 | 0.0726197 | 0.2910375 | 0.5608991 | SEC63 | | 11231 |
| ENSG00000272476 | 24.27920245 | 0.005182985 | 0.1324134 | 0.8792606 | NA | NA | NA | |
| ENSG00000081087 | 648.4881908 | 0.143300478 | 0.1057813 | 0.0671056 | 0.2346186 | OSTM1 | | 28962 |
| ENSG00000112335 | 2050.268162 | -0.078801207 | 0.0695697 | 0.1887824 | 0.4396927 | SNX3 | | 8724 |
| ENSG00000135537 | 68.3279312 | 0.009475243 | 0.126283 | 0.8484577 | 0.9375782 | AFG1L | | 246269 |
| ENSG00000118689 | 597.7965015 | 0.220928376 | 0.1208249 | 0.0133567 | 0.0778155 | FOXO3 | | 2309 |
| ENSG00000118690 | 38.14187142 | 0.040333587 | 0.1379279 | 0.3317002 | NA | ARMC2 | | 84071 |
| ENSG00000080546 | 147.6173838 | -0.058917197 | 0.1288516 | 0.3617747 | 0.6252242 | SESN1 | | 27244 |
| ENSG00000183137 | 335.8875755 | 0.109648279 | 0.1263903 | 0.1632135 | 0.4058104 | CEP57L1 | | 285753 |
| ENSG00000243587 | 11.2776374 | 0.018780335 | 0.1365132 | 0.4204235 | NA | NA | NA | |
| ENSG00000135535 | 3524.106451 | -0.116381967 | 0.0631858 | 0.037785 | 0.1612593 | CD164 | | 8763 |
| ENSG00000135587 | 245.5230913 | -0.069845941 | 0.1209167 | 0.3270656 | 0.5952717 | SMPD2 | | 6610 |
| ENSG00000135596 | 944.6547595 | -0.357582411 | 0.1045464 | 6.92E-05 | 0.0012874 | MICAL1 | | 64780 |
| ENSG00000112365 | 635.8326423 | 0.12190946 | 0.1056097 | 0.1125891 | 0.325537 | ZBTB24 | | 9841 |
| ENSG00000155085 | 53.06627752 | -0.005333629 | 0.1288176 | 0.8999066 | 0.9607722 | AK9 | | 221264 |
| ENSG00000112367 | 197.3871226 | -0.226361746 | 0.2056849 | 0.0305931 | 0.1401263 | FIG4 | | 9896 |
| ENSG00000112290 | 543.2752154 | -0.121944194 | 0.11148 | 0.1192083 | 0.3371283 | WASF1 | | 8936 |
| ENSG00000168438 | 503.7424105 | -0.06793112 | 0.1005048 | 0.3433996 | 0.6097979 | CDC40 | | 51362 |
| ENSG00000155111 | 438.7463371 | -0.088865886 | 0.1089663 | 0.2330778 | 0.4953752 | CDK19 | | 23097 |
| ENSG00000123505 | 2791.613541 | 0.076021321 | 0.0651583 | 0.1855871 | 0.4358915 | AMD1 | | 262 |
| ENSG00000155115 | 976.7665764 | -0.139246224 | 0.092248 | 0.0557189 | 0.2075421 | GTF3C6 | | 112495 |
| ENSG00000197498 | 995.0267395 | 0.257905451 | 0.1044819 | 0.002343 | 0.0213484 | RPF2 | | 84154 |
| ENSG00000173214 | 108.829858 | 0.045115567 | 0.127989 | 0.4475408 | 0.6951753 | MFSD4B | | 91749 |
| ENSG00000230177 | 10.97029053 | 0.009274659 | 0.1350737 | 0.6916582 | NA | NA | NA | |
| ENSG00000009413 | 867.0784293 | -0.018773405 | 0.0863247 | 0.7785345 | 0.9075471 | REV3L | | 5980 |
| ENSG00000231889 | 58.46317468 | 0.110193093 | 0.1866964 | 0.0619513 | 0.2223574 | TRAF3IP2-A | | 643749 |
| ENSG00000056972 | 46.75296643 | 0.031782986 | 0.1337191 | 0.4669148 | 0.7094916 | TRAF3IP2 | | 10758 |
| ENSG00000010810 | 103.9086699 | 0.065985265 | 0.1389914 | 0.2739085 | 0.5422925 | FYN | | 2534 |
| ENSG00000074935 | 216.6720163 | 0.102360009 | 0.1403116 | 0.1752501 | 0.4235664 | TUBE1 | | 51175 |
| ENSG00000203778 | 119.4629627 | -0.057869465 | 0.1337049 | 0.3357534 | 0.6031891 | FAM229B | | 619208 |
| ENSG00000155130 | 516.8918204 | -0.26931828 | 0.1303329 | 0.0054691 | 0.0405875 | NA | NA | |
| ENSG00000196591 | 2031.216185 | -0.095409975 | 0.0693469 | 0.109305 | 0.3200394 | HDAC2 | | 3066 |
| ENSG00000178425 | 518.792687 | -0.201066976 | 0.1247118 | 0.0233796 | 0.1153771 | NT5DC1 | | 221294 |
| ENSG00000187189 | 239.3024448 | 0.053474185 | 0.1170254 | 0.438436 | 0.6881889 | TSPYL4 | | 23270 |

| | | | | | | | |
|---|---|---|---|---|---|---|---|
| ENSG00000111817 | 542.224678 | -0.003774015 | 0.0936716 | 0.954819 | 0.9820587 | DSE | 29940 |
| ENSG00000189241 | 1046.388602 | -0.04669788 | 0.0788917 | 0.4723449 | 0.7126661 | TSPYL1 | 7259 |
| ENSG00000111832 | 627.6174943 | -0.027861593 | 0.0894459 | 0.6811061 | 0.8538613 | RWDD1 | 51389 |
| ENSG00000111834 | 16.56143529 | 0.009646147 | 0.1343258 | 0.7244982 | NA | RSPH4A | 345895 |
| ENSG00000153975 | 324.9612844 | 0.019167901 | 0.1024546 | 0.7800895 | 0.9083159 | ZUP1 | 221302 |
| ENSG00000196911 | 183.9501243 | 0.014183958 | 0.1144209 | 0.8239668 | 0.9274924 | KPNA5 | 3841 |
| ENSG00000047932 | 745.0386848 | 0.034789563 | 0.0856301 | 0.6047693 | 0.8100343 | GOPC | 57120 |
| ENSG00000164465 | 349.5229553 | -0.062459541 | 0.1099431 | 0.3827234 | 0.6435041 | DCBLD1 | 285761 |
| ENSG00000153989 | 1246.728595 | -0.282777438 | 0.0927206 | 0.0003611 | 0.0050234 | NUS1 | 116150 |
| ENSG00000230202 | 113.8471379 | -0.02325346 | 0.1219485 | 0.6879231 | 0.8578659 | NA | NA |
| ENSG00000111860 | 117.7483073 | -0.041036171 | 0.1256394 | 0.4919821 | 0.7282321 | CEP85L | 387119 |
| ENSG00000111877 | 227.264635 | 0.003160577 | 0.1084152 | 0.9627607 | 0.985023 | MCM9 | 254394 |
| ENSG00000111875 | 199.8176239 | -0.100753946 | 0.1394896 | 0.1806034 | 0.4299157 | ASF1A | 25842 |
| ENSG00000111879 | 330.2268261 | 0.327689799 | 0.1782591 | 0.0058405 | 0.0426479 | FAM184A | 79632 |
| ENSG00000111885 | 231.672569 | 0.001466694 | 0.1093712 | 0.982704 | 0.9934198 | MAN1A1 | 4121 |
| ENSG00000146350 | 88.75124307 | -0.042676019 | 0.1310588 | 0.4347119 | 0.6852726 | TBC1D32 | 221322 |
| ENSG00000152661 | 188.7628699 | 1.105215877 | 0.2112287 | 8.73E-09 | 5.37E-07 | GJA1 | 2697 |
| ENSG00000025156 | 289.3497848 | 0.022813156 | 0.1060483 | 0.7377201 | 0.8854328 | HSF2 | 3298 |
| ENSG00000111897 | 2265.160601 | -0.021449136 | 0.064047 | 0.71334 | 0.8705102 | SERINC1 | 57515 |
| ENSG00000172594 | 31.58570195 | 0.020248425 | 0.1333965 | 0.5815014 | NA | SMPDL3A | 10924 |
| ENSG00000146373 | 294.6227967 | -0.037316539 | 0.107116 | 0.5876389 | 0.7977929 | RNF217 | 154214 |
| ENSG00000111907 | 199.6643111 | 0.13506457 | 0.1581341 | 0.1025765 | 0.3069936 | TPD52L1 | 7164 |
| ENSG00000111906 | 587.6446198 | -0.025414684 | 0.0910203 | 0.7086833 | 0.8682085 | HDDC2 | 51020 |
| ENSG00000111912 | 496.6812438 | 0.029224259 | 0.0953575 | 0.6437718 | 0.8333617 | NCOA7 | 135112 |
| ENSG00000111911 | 341.6259706 | -0.006915313 | 0.1021827 | 0.9178162 | 0.9678623 | HINT3 | 135114 |
| ENSG00000066651 | 374.9417402 | 0.016728946 | 0.0995449 | 0.806681 | 0.9196604 | TRMT11 | 60487 |
| ENSG00000203760 | 455.6050761 | -0.133028529 | 0.1258073 | 0.1034541 | 0.3085136 | CENPW | 387103 |
| ENSG00000118518 | 285.8965704 | 0.039669903 | 0.108239 | 0.5664295 | 0.7826429 | RNF146 | 81847 |
| ENSG00000093144 | 1075.476803 | -0.162834137 | 0.0901401 | 0.0252218 | 0.121742 | ECHDC1 | 55862 |
| ENSG00000152894 | 891.5449199 | 0.021292061 | 0.0814884 | 0.745957 | 0.8893165 | PTPRK | 5796 |
| ENSG00000196569 | 20.46998584 | -0.024297463 | 0.1359552 | 0.4355292 | NA | LAMA2 | 3908 |
| ENSG00000146376 | 321.2795869 | -0.69677745 | 0.1614303 | 9.08E-07 | 3.14E-05 | ARHGAP18 | 93663 |
| ENSG00000198945 | 230.4890119 | 0.011412075 | 0.10961 | 0.8631266 | 0.9452636 | L3MBTL3 | 84456 |
| ENSG00000079819 | 3849.469417 | -0.137260322 | 0.0630607 | 0.0146734 | 0.082922 | EPB41L2 | 2037 |
| ENSG00000118507 | 52.56558745 | -0.035092554 | 0.1343066 | 0.4313793 | 0.6828658 | AKAP7 | 9465 |
| ENSG00000112282 | 558.3750699 | -0.023147673 | 0.0931196 | 0.7346177 | 0.882917 | MED23 | 9439 |
| ENSG00000197594 | 116.178303 | -0.042819548 | 0.1274629 | 0.4658849 | 0.7092404 | ENPP1 | 5167 |
| ENSG00000118523 | 602.2765506 | 0.588642006 | 0.1265488 | 2.21E-07 | 9.30E-06 | CCN2 | 1490 |
| ENSG00000079950 | 714.3419286 | -0.071471224 | 0.0912676 | 0.3061501 | 0.5752194 | STX7 | 8417 |
| ENSG00000112299 | 28.88663346 | -0.032537317 | 0.1372996 | 0.3532562 | NA | VNN1 | 8876 |
| ENSG00000146409 | 119.6672733 | -0.107111665 | 0.1625917 | 0.1265741 | 0.3492823 | SLC18B1 | 116843 |
| ENSG00000112306 | 7045.688016 | -0.015469717 | 0.0528855 | 0.7519374 | 0.8928015 | RPS12 | 6206 |
| ENSG00000206754 | 12.17141552 | -0.016820929 | 0.1359556 | 0.4854692 | NA | SNORD101 | 594837 |
| ENSG00000221500 | 17.16257211 | -0.016617648 | 0.1352814 | 0.5385358 | NA | SNORD100 | 594838 |
| ENSG00000200534 | 26.73061972 | -0.002686723 | 0.1319136 | 0.9368009 | NA | SNORA33 | 594839 |
| ENSG00000028839 | 243.0839614 | 0.023965449 | 0.1089829 | 0.7220051 | 0.8743675 | TBPL1 | 9519 |
| ENSG00000118515 | 3160.974705 | 0.835412394 | 0.081808 | 1.11E-25 | 5.64E-23 | SGK1 | 6446 |
| ENSG00000118514 | 9.162705422 | -0.003604831 | 0.1350124 | 0.8628301 | NA | ALDH8A1 | 64577 |
| ENSG00000112339 | 1201.211792 | -0.0795864 | 0.0803243 | 0.225865 | 0.4868367 | HBS1L | 10767 |
| ENSG00000118513 | 34.24094629 | 0.055592003 | 0.1464418 | 0.1684765 | NA | MYB | 4602 |
| ENSG00000135541 | 492.1698589 | 0.211979175 | 0.1290784 | 0.0201128 | 0.1042633 | AHI1 | 54806 |
| ENSG00000146410 | 240.4520771 | -0.099694878 | 0.1339111 | 0.188309 | 0.4389939 | MTFR2 | 113115 |
| ENSG00000029363 | 4805.707296 | -0.219372135 | 0.0612413 | 9.30E-05 | 0.0016432 | BCLAF1 | 9774 |
| ENSG00000135525 | 102.9569444 | 0.011903112 | 0.1215243 | 0.8331965 | 0.9305394 | MAP7 | 9053 |
| ENSG00000197442 | 642.6332287 | -0.065680485 | 0.0929872 | 0.3489392 | 0.6148358 | MAP3K5 | 4217 |
| ENSG00000112357 | 100.5684327 | 0.040199494 | 0.12866 | 0.4776111 | 0.7167198 | PEX7 | 5191 |
| ENSG00000027697 | 414.7936099 | -0.013785496 | 0.0974792 | 0.8395953 | 0.9342967 | IFNGR1 | 3459 |
| ENSG00000237499 | 10.14655986 | 0.029183125 | 0.1397568 | 0.1547509 | NA | WAKMAR2 | 100130476 |
| ENSG00000118503 | 601.6200336 | 0.619639725 | 0.121415 | 2.21E-08 | 1.24E-06 | TNFAIP3 | 7128 |
| ENSG00000112378 | 1057.257184 | 0.512452531 | 0.1049031 | 8.10E-08 | 3.98E-06 | PERP | 64065 |
| ENSG00000112379 | 532.7149059 | -0.052039896 | 0.0960634 | 0.4574904 | 0.7017657 | ARFGEF3 | 57221 |
| ENSG00000051620 | 839.0459864 | -0.138922094 | 0.0978614 | 0.064428 | 0.2286099 | HEBP2 | 23593 |
| ENSG00000135540 | 262.8491416 | -0.028806968 | 0.1122764 | 0.6630201 | 0.8438599 | NHSL1 | 57224 |
| ENSG00000225177 | 21.9292373 | 0.012672896 | 0.1336051 | 0.6896 | NA | LOC124900 | 124900217 |
| ENSG00000024862 | 175.3168095 | 0.009262544 | 0.1134043 | 0.887339 | 0.9545517 | CCDC28A | 25901 |
| ENSG00000135597 | 485.4246643 | -0.003553183 | 0.0931466 | 0.9572601 | 0.9826522 | REPS1 | 85021 |
| ENSG00000146386 | 476.4098938 | -0.022852959 | 0.0951917 | 0.7376713 | 0.8854328 | ABRACL | 58527 |
| ENSG00000112406 | 176.1661785 | 0.246220032 | 0.2258583 | 0.0256411 | 0.1231551 | HECA | 51696 |
| ENSG00000164442 | 629.1817302 | 1.239631418 | 0.1346598 | 1.77E-21 | 5.58E-19 | CITED2 | 10370 |
| ENSG00000009844 | 948.4975244 | -0.006463877 | 0.0787247 | 0.919187 | 0.9686957 | VTA1 | 51534 |

| | | | | | | | | |
|---|---|---|---|---|---|---|---|---|
| ENSG00000112414 | 4291.158139 | -0.367423473 | 0.0663792 | 3.76E-09 | 2.51E-07 | ADGRG6 | | 57211 |
| ENSG00000010818 | 843.1535287 | 0.193759386 | 0.1030175 | 0.0157963 | 0.087407 | HIVEP2 | | 3097 |
| ENSG00000146416 | 193.2669469 | -0.087683698 | 0.1344301 | 0.2253036 | 0.4861946 | AIG1 | | 51390 |
| ENSG00000189007 | 160.1942354 | 0.073627765 | 0.1358684 | 0.2687393 | 0.5365697 | ADAT2 | | 134637 |
| ENSG00000034693 | 245.9456199 | -0.085573167 | 0.1287784 | 0.2444402 | 0.5100385 | PEX3 | | 8504 |
| ENSG00000229036 | 63.4422745 | -0.033888142 | 0.1311832 | 0.4942349 | 0.7297633 | NA | NA | |
| ENSG00000001036 | 1388.552316 | -0.043471902 | 0.0729098 | 0.4811804 | 0.7202486 | FUCA2 | | 2519 |
| ENSG00000112419 | 491.3039044 | -0.046744687 | 0.098891 | 0.5043238 | 0.7381117 | PHACTR2 | | 9749 |
| ENSG00000135521 | 1233.080311 | -0.05331075 | 0.0765548 | 0.401805 | 0.6594751 | LTV1 | | 84946 |
| ENSG00000169976 | 1003.332991 | -0.05071106 | 0.0804949 | 0.4382572 | 0.6881889 | SF3B5 | | 83443 |
| ENSG00000135604 | 84.47526884 | -0.015633743 | 0.1247192 | 0.7653102 | 0.899174 | STX11 | | 8676 |
| ENSG00000152818 | 1714.029128 | -0.47581244 | 0.0869565 | 3.83E-09 | 2.53E-07 | UTRN | | 7402 |
| ENSG00000112425 | 40.91880659 | -0.03147327 | 0.1348916 | 0.4376038 | NA | EPM2A | | 7957 |
| ENSG00000118496 | 644.6146043 | -0.032758672 | 0.0883884 | 0.6280965 | 0.8226951 | FBXO30 | | 84085 |
| ENSG00000146414 | 575.5464592 | -0.010953101 | 0.0904721 | 0.8708489 | 0.9478556 | SHPRH | | 257218 |
| ENSG00000118508 | 534.8736717 | 0.295196426 | 0.1359425 | 0.0036618 | 0.0298072 | RAB32 | | 10981 |
| ENSG00000164506 | 440.8127839 | -0.095883433 | 0.1141206 | 0.2072543 | 0.4632201 | STXBP5 | | 134957 |
| ENSG00000225135 | 34.62829401 | 0.009233495 | 0.1310167 | 0.8127719 | NA | NA | NA | |
| ENSG00000203727 | 65.19617319 | 0.029269346 | 0.1308925 | 0.5411871 | 0.7644022 | SAMD5 | | 389432 |
| ENSG00000111961 | 517.6090035 | -0.044097941 | 0.0977617 | 0.527122 | 0.7542029 | SASH1 | | 23328 |
| ENSG00000111962 | 22.33237495 | 0.009629581 | 0.1330636 | 0.7664088 | NA | UST | | 10090 |
| ENSG00000055208 | 1049.831285 | -0.026619663 | 0.0849589 | 0.6939872 | 0.8601936 | TAB2 | | 23118 |
| ENSG00000131013 | 640.8871831 | 0.241000398 | 0.1202905 | 0.0079414 | 0.0538698 | PPIL4 | | 85313 |
| ENSG00000055211 | 502.8133796 | -0.092113873 | 0.1055244 | 0.21592 | 0.4742688 | GINM1 | | 116254 |
| ENSG00000220848 | 18.34942577 | -0.003251814 | 0.1334013 | 0.9091831 | NA | NA | NA | |
| ENSG00000186625 | 295.0749746 | 0.038394996 | 0.1072218 | 0.578149 | 0.7912547 | KATNA1 | | 11104 |
| ENSG00000131023 | 854.6864418 | -0.021377219 | 0.0832557 | 0.7457705 | 0.8893165 | LATS1 | | 9113 |
| ENSG00000120253 | 1146.673058 | -0.024430294 | 0.0764048 | 0.6993232 | 0.8632898 | NUP43 | | 348995 |
| ENSG00000120265 | 2229.489497 | -0.101015156 | 0.0713469 | 0.1000039 | 0.3018463 | PCMT1 | | 5110 |
| ENSG00000120256 | 155.6964642 | 0.048854916 | 0.1235651 | 0.4488438 | 0.6959957 | LRP11 | | 84918 |
| ENSG00000131015 | 63.49017348 | 0.177752398 | 0.3035531 | 0.0222581 | 0.1115867 | ULBP2 | | 80328 |
| ENSG00000111981 | 173.2606157 | 0.147377757 | 0.1739705 | 0.0820961 | 0.2666754 | ULBP1 | | 80329 |
| ENSG00000218358 | 14.90877889 | 0.025085784 | 0.1372778 | 0.3518513 | NA | NA | NA | |
| ENSG00000131019 | 79.18755883 | -0.030049661 | 0.1286267 | 0.5609785 | 0.7795039 | ULBP3 | | 79465 |
| ENSG00000120278 | 49.69611974 | -0.040914052 | 0.1363778 | 0.3646969 | 0.6272549 | PLEKHG1 | | 57480 |
| ENSG00000120254 | 1795.084226 | -0.047251007 | 0.0769731 | 0.45824 | 0.7025176 | MTHFD1L | | 25902 |
| ENSG00000131016 | 9222.791821 | -0.116008701 | 0.1238033 | 0.1362898 | 0.3635536 | AKAP12 | | 9590 |
| ENSG00000181472 | 628.6795373 | -0.152654119 | 0.1134566 | 0.0602647 | 0.2186866 | ZBTB2 | | 57621 |
| ENSG00000155906 | 227.9301612 | 0.480713682 | 0.2046895 | 0.0011203 | 0.0122073 | RMND1 | | 55005 |
| ENSG00000146476 | 487.1997925 | -0.047598468 | 0.096555 | 0.4954678 | 0.7308549 | ARMT1 | | 79624 |
| ENSG00000131018 | 102.9589688 | 0.673015792 | 0.3153563 | 0.0012939 | 0.0137029 | SYNE1 | | 23345 |
| ENSG00000112029 | 1147.523041 | 0.202658272 | 0.0926062 | 0.0074713 | 0.0512762 | FBXO5 | | 26271 |
| ENSG00000227627 | 11.41010565 | -0.012082798 | 0.1353821 | 0.6016235 | NA | NA | NA | |
| ENSG00000112031 | 838.8428783 | -0.034820002 | 0.0848631 | 0.6017439 | 0.8077875 | MTRF1L | | 54516 |
| ENSG00000091844 | 175.306501 | -0.443623703 | 0.2447623 | 0.0036735 | 0.0298398 | RGS17 | | 26575 |
| ENSG00000218426 | 75.29833134 | 0.026770256 | 0.1387745 | 0.2263443 | 0.4874737 | RPL27AP6 | | 389435 |
| ENSG00000153721 | 135.6046252 | 0.134213643 | 0.1778591 | 0.0895925 | 0.282485 | CNKSR3 | | 154043 |
| ENSG00000213079 | 899.1725281 | 0.023288601 | 0.0830229 | 0.7252357 | 0.8766924 | SCAF8 | | 22828 |
| ENSG00000146426 | 30.02690933 | 0.048556118 | 0.1436061 | 0.1929986 | NA | TIAM2 | | 26230 |
| ENSG00000235381 | 18.16119865 | 0.040770827 | 0.1421991 | 0.1723528 | NA | NA | NA | |
| ENSG00000029639 | 122.8520945 | -0.061736037 | 0.1330935 | 0.3244711 | 0.593455 | TFB1M | | 51106 |
| ENSG00000049618 | 1220.300121 | -0.219938793 | 0.0887807 | 0.0030965 | 0.026229 | ARID1B | | 57492 |
| ENSG00000215712 | 284.2596694 | 0.025722352 | 0.1069324 | 0.705637 | 0.8670449 | TMEM242 | | 729515 |
| ENSG00000175048 | 236.2845634 | 0.056007157 | 0.1167226 | 0.4218484 | 0.6741841 | ZDHHC14 | | 79683 |
| ENSG00000130340 | 2580.422261 | -0.487805467 | 0.076534 | 1.60E-11 | 1.61E-09 | SNX9 | | 51429 |
| ENSG00000229502 | 16.49925755 | -0.004026846 | 0.133702 | 0.8836317 | NA | NA | NA | |
| ENSG00000078269 | 2521.372443 | -0.102493677 | 0.0714322 | 0.0941498 | 0.2909429 | SYNJ2 | | 8871 |
| ENSG00000122335 | 190.9044449 | -0.219852446 | 0.2136133 | 0.0334558 | 0.1479732 | SERAC1 | | 84947 |
| ENSG00000272047 | 502.1221383 | -0.109428729 | 0.1087023 | 0.1530336 | 0.3907524 | GTF2H5 | | 404672 |
| ENSG00000236537 | 27.34888249 | 0.025956411 | 0.1351959 | 0.4629372 | NA | NA | NA | |
| ENSG00000130338 | 598.4596433 | 0.030297171 | 0.0928505 | 0.6601239 | 0.8426737 | TULP4 | | 56995 |
| ENSG00000146433 | 871.6203936 | -0.041633378 | 0.0823447 | 0.5262119 | 0.7534979 | TMEM181 | | 57583 |
| ENSG00000146425 | 1188.417105 | -0.227269213 | 0.0906089 | 0.0026311 | 0.0233186 | DYNLT1 | | 6993 |
| ENSG00000164674 | 15.5426404 | 0.007414366 | 0.134273 | 0.781611 | NA | SYTL3 | | 94120 |
| ENSG00000092820 | 7204.911026 | 0.080030731 | 0.0545754 | 0.109427 | 0.3202262 | EZR | | 7430 |
| ENSG00000233893 | 20.38478254 | 0.005064503 | 0.1335456 | 0.8663444 | NA | EZR-AS1 | | 101409257 |
| ENSG00000203711 | 10.3722466 | 0.021592198 | 0.1372559 | 0.3413244 | NA | LINC02901 | | 100130967 |
| ENSG00000130363 | 141.307342 | 0.153748511 | 0.1908636 | 0.0699533 | 0.2404621 | RSPH3 | | 83861 |
| ENSG00000112096 | 10243.58403 | -0.129532178 | 0.058351 | 0.0141366 | 0.0810877 | NA | NA | |
| ENSG00000146457 | 2084.361672 | 0.035795898 | 0.071229 | 0.5545073 | 0.7749708 | WTAP | | 9589 |

| | | | | | | | | |
|---|---|---|---|---|---|---|---|---|
| ENSG00000120437 | 1881.620293 | -0.534013175 | 0.082412 | 7.09E-12 | 7.67E-10 | ACAT2 | | 39 |
| ENSG00000120438 | 6352.612541 | -0.089039249 | 0.0552377 | 0.0723673 | 0.2457085 | TCP1 | | 6950 |
| ENSG00000112110 | 1175.248997 | -0.048194112 | 0.0776963 | 0.4519248 | 0.6982717 | MRPL18 | | 29074 |
| ENSG00000197081 | 2472.007289 | 0.201413428 | 0.0765853 | 0.0024621 | 0.022174 | IGF2R | | 3482 |
| ENSG00000213073 | 41.64052742 | 0.03522891 | 0.1355641 | 0.4049504 | 0.6614808 | NA | NA | |
| ENSG00000270949 | 41.72126856 | -0.040183189 | 0.1380271 | 0.3260824 | 0.5942836 | NA | NA | |
| ENSG00000272841 | 45.6328058 | -0.007784856 | 0.1293558 | 0.8525429 | 0.9398409 | MAP3K4-AS | | 117981787 |
| ENSG00000085511 | 511.437035 | -0.25757326 | 0.1324915 | 0.0076379 | 0.0521134 | MAP3K4 | | 4216 |
| ENSG00000026652 | 213.5720221 | 0.002955461 | 0.1097268 | 0.9653715 | 0.9862257 | AGPAT4 | | 56895 |
| ENSG00000112531 | 1811.625278 | -0.200214078 | 0.0781478 | 0.002946 | 0.0253775 | QKI | | 9444 |
| ENSG00000198818 | 721.280965 | -0.036580484 | 0.0877344 | 0.5885467 | 0.7984432 | SFT2D1 | | 113402 |
| ENSG00000060762 | 207.584282 | -0.008771194 | 0.1107587 | 0.891572 | 0.9562439 | MPC1 | | 51660 |
| ENSG00000235272 | 8.304389592 | 0.042975243 | 0.145513 | 0.0314946 | NA | RAMACL | | 353267 |
| ENSG00000026297 | 55.47750619 | -0.019335905 | 0.129315 | 0.6721204 | 0.8499086 | RNASET2 | | 8635 |
| ENSG00000227598 | 8.34166665 | 0.001948146 | 0.1351139 | 0.926356 | NA | NA | NA | |
| ENSG00000213066 | 450.5172773 | 0.006910233 | 0.0945339 | 0.9202259 | 0.9691785 | CEP43 | | 11116 |
| ENSG00000272980 | 8.983666802 | -0.013967637 | 0.1360215 | 0.5084619 | NA | NA | NA | |
| ENSG00000130396 | 1581.575261 | -0.177122205 | 0.079741 | 0.0088183 | 0.0583937 | AFDN | | 4301 |
| ENSG00000184465 | 467.8004961 | 0.429205162 | 0.1420709 | 0.0002016 | 0.0030898 | WDR27 | | 253769 |
| ENSG00000185127 | 439.8902675 | -0.146005355 | 0.1253689 | 0.0798069 | 0.261567 | C6orf120 | | 387263 |
| ENSG00000130024 | 1341.424814 | -0.098243925 | 0.0786349 | 0.1322016 | 0.3581236 | PHF10 | | 55274 |
| ENSG00000130023 | 214.6181679 | -0.106323902 | 0.1394235 | 0.1665115 | 0.4115574 | ERMARD | | 55780 |
| ENSG00000266896 | 10.28813024 | -0.009152317 | 0.1353791 | 0.6671202 | NA | NA | NA | |
| ENSG00000112584 | 417.0110635 | 0.065087888 | 0.1036862 | 0.3661672 | 0.6289998 | FAM120B | | 84498 |
| ENSG00000008018 | 2101.234913 | -0.168521912 | 0.0727688 | 0.0075902 | 0.0518486 | PSMB1 | | 5689 |
| ENSG00000112592 | 305.6002882 | 0.009075553 | 0.1031808 | 0.8932169 | 0.956866 | TBP | | 6908 |
| ENSG00000071994 | 1357.983896 | -0.123122384 | 0.0851699 | 0.0746217 | 0.2507772 | PDCD2 | | 5134 |
| ENSG00000197461 | 65.75834044 | -0.089458844 | 0.1621598 | 0.1195023 | 0.3374295 | PDGFA | | 5154 |
| ENSG00000188191 | 739.0250049 | 0.097731058 | 0.0940654 | 0.1758233 | 0.4246004 | PRKAR1B | | 5575 |
| ENSG00000164818 | 982.0138939 | -0.146010716 | 0.0933783 | 0.0472135 | 0.1857336 | DNAAF5 | | 54919 |
| ENSG00000164828 | 2188.803554 | 0.006775426 | 0.0664654 | 0.9081691 | 0.9638438 | SUN1 | | 23353 |
| ENSG00000239857 | 140.1994714 | 0.044065593 | 0.1232089 | 0.4859064 | 0.7237787 | GET4 | | 51608 |
| ENSG00000105963 | 19.00685193 | 0.017395965 | 0.1348424 | 0.5596857 | NA | ADAP1 | | 11033 |
| ENSG00000240230 | 396.8812021 | 0.445011666 | 0.1491673 | 0.0002168 | 0.0032712 | COX19 | | 90639 |
| ENSG00000146540 | 1042.571086 | -0.009066795 | 0.0806095 | 0.8884109 | 0.9545517 | C7orf50 | | 84310 |
| ENSG00000164849 | 12.14817412 | 0.008011719 | 0.1348668 | 0.7370982 | NA | GPR146 | | 115330 |
| ENSG00000178381 | 277.2433732 | 0.672766756 | 0.1722421 | 5.40E-06 | 0.0001484 | ZFAND2A | | 90637 |
| ENSG00000229043 | 29.79123471 | 0.018883121 | 0.1342525 | 0.5667323 | NA | ZFAND2A-D | | 101927021 |
| ENSG00000164877 | 314.7178112 | -0.076365104 | 0.1153113 | 0.2976476 | 0.5680006 | MICALL2 | | 79778 |
| ENSG00000273230 | 51.86755565 | 0.017480343 | 0.1296511 | 0.6967615 | 0.8616368 | NA | NA | |
| ENSG00000164880 | 2309.130219 | -0.133312329 | 0.0737498 | 0.034509 | 0.1512569 | INTS1 | | 26173 |
| ENSG00000198517 | 744.5137302 | 0.006744442 | 0.0912264 | 0.9219839 | 0.9700679 | MAFK | | 7975 |
| ENSG00000164855 | 178.5255408 | -0.050618219 | 0.1204941 | 0.4439848 | 0.6921375 | TMEM184A | | 202915 |
| ENSG00000157778 | 491.7484578 | 0.145472352 | 0.1180895 | 0.0759506 | 0.2536246 | PSMG3 | | 84262 |
| ENSG00000230487 | 18.63953186 | 0.015243301 | 0.1346266 | 0.6023283 | NA | PSMG3-AS1 | | 114796 |
| ENSG00000225968 | 108.3936712 | -0.1627981 | 0.2247765 | 0.0495758 | 0.1914995 | ELFN1 | | 392617 |
| ENSG00000002822 | 694.0235687 | 0.017263617 | 0.0858762 | 0.7972108 | 0.9158202 | MAD1L1 | | 8379 |
| ENSG00000122687 | 746.3402008 | 0.030799053 | 0.086296 | 0.6477756 | 0.8364655 | MRM2 | | 29960 |
| ENSG00000106268 | 477.5936265 | -0.123494297 | 0.114604 | 0.1175322 | 0.3339561 | NUDT1 | | 4521 |
| ENSG00000106266 | 1825.508561 | 0.127226304 | 0.0764883 | 0.0489719 | 0.1899207 | SNX8 | | 29886 |
| ENSG00000106263 | 7295.958388 | 0.028397406 | 0.0531509 | 0.5622085 | 0.7800541 | EIF3B | | 8662 |
| ENSG00000136213 | 237.6766593 | -0.079134954 | 0.1235234 | 0.2756612 | 0.5439328 | CHST12 | | 55501 |
| ENSG00000175873 | 59.56981044 | 0.017524419 | 0.1283089 | 0.7139087 | 0.8705676 | NA | NA | |
| ENSG00000106003 | 149.5114319 | -0.426179503 | 0.2639333 | 0.0053205 | 0.0398527 | LFNG | | 3955 |
| ENSG00000106009 | 1117.880128 | -0.024714435 | 0.0802073 | 0.7031952 | 0.8651804 | BRAT1 | | 221927 |
| ENSG00000106012 | 400.6318164 | -0.01321874 | 0.097745 | 0.8462723 | 0.9372292 | IQCE | | 23288 |
| ENSG00000136295 | 1982.088291 | -0.560176669 | 0.0772613 | 3.17E-14 | 5.17E-12 | TTYH3 | | 80727 |
| ENSG00000174945 | 9.057457219 | 0.036309395 | 0.1422746 | 0.0859552 | NA | AMZ1 | | 155185 |
| ENSG00000146535 | 1636.337233 | -0.210972174 | 0.0866224 | 0.0037518 | 0.030371 | GNA12 | | 2768 |
| ENSG00000146555 | 97.72004496 | 0.081131178 | 0.1515044 | 0.1754834 | 0.4239549 | SDK1 | | 221935 |
| ENSG00000164916 | 1931.166811 | 0.030337882 | 0.0681596 | 0.609439 | 0.8124694 | FOXK1 | | 221937 |
| ENSG00000242802 | 917.8186141 | 0.259469936 | 0.1056898 | 0.0023976 | 0.0217101 | AP5Z1 | | 9907 |
| ENSG00000196204 | 252.6077173 | 0.014281296 | 0.106919 | 0.8264298 | 0.9286016 | RNF216P1 | | 441191 |
| ENSG00000146587 | 547.9837998 | -0.037126491 | 0.0975202 | 0.5920546 | 0.8010877 | RBAK | | 57786 |
| ENSG00000157954 | 1172.7869 | 0.024839402 | 0.08116 | 0.7049603 | 0.8666859 | WIPI2 | | 26100 |
| ENSG00000164638 | 98.67429486 | -0.037569406 | 0.1279811 | 0.5014559 | 0.7359601 | SLC29A4 | | 222962 |
| ENSG00000182095 | 2902.019599 | -0.08479886 | 0.0685108 | 0.1532552 | 0.3908224 | TNRC18 | | 84629 |
| ENSG00000234432 | 21.92878703 | 0.047196623 | 0.1446104 | 0.1449387 | NA | LINC02983 | | 100129484 |
| ENSG00000155034 | 444.0476322 | 0.300552826 | 0.1410208 | 0.0038798 | 0.0311265 | FBXL18 | | 80028 |
| ENSG00000207973 | 16.842422 | 0.000445343 | 0.133572 | 0.9894215 | NA | MIR589 | | 693174 |

| | | | | | | | | |
|---|---|---|---|---|---|---|---|---|
| ENSG00000075624 | 44394.04196 | -0.298702818 | 0.065176 | 8.00E-07 | 2.82E-05 | ACTB | | 60 |
| ENSG00000075618 | 4848.960977 | -0.027644777 | 0.0619175 | 0.6146464 | 0.8157779 | FSCN1 | | 6624 |
| ENSG00000011275 | 980.1653574 | 0.08636411 | 0.0880755 | 0.2098048 | 0.4663614 | RNF216 | | 54476 |
| ENSG00000235944 | 17.96811575 | 0.02251127 | 0.1360504 | 0.4426931 | NA | NA | NA | |
| ENSG00000122674 | 123.0196166 | 0.00776646 | 0.1191918 | 0.9034083 | 0.9618565 | CCZ1 | | 51622 |
| ENSG00000122512 | 390.5471087 | 0.034571552 | 0.1000763 | 0.6190627 | 0.8179246 | PMS2 | | 5395 |
| ENSG00000106305 | 821.0675002 | 0.035716544 | 0.0901921 | 0.6024847 | 0.8080863 | AIMP2 | | 7965 |
| ENSG00000086232 | 2017.052416 | -0.026642954 | 0.0666983 | 0.6465641 | 0.8356474 | EIF2AK1 | | 27102 |
| ENSG00000106346 | 272.9101612 | -0.020562043 | 0.1067693 | 0.7408582 | 0.8866276 | USP42 | | 84132 |
| ENSG00000008256 | 1088.153616 | -0.055725664 | 0.0832012 | 0.4027622 | 0.6601399 | CYTH3 | | 9265 |
| ENSG00000178397 | 231.4779687 | 0.01972024 | 0.1092883 | 0.7632953 | 0.8982943 | FAM220A | | 84792 |
| ENSG00000136238 | 5278.426076 | -0.091747767 | 0.0573383 | 0.0774483 | 0.2565722 | RAC1 | | 5879 |
| ENSG00000164535 | 476.8791393 | 0.327776143 | 0.1461614 | 0.0025853 | 0.0230002 | DAGLB | | 221955 |
| ENSG00000136240 | 2297.07315 | -0.1000706 | 0.0677868 | 0.0895991 | 0.282485 | KDELR2 | | 11014 |
| ENSG00000136247 | 418.0971139 | 0.099758256 | 0.1141745 | 0.1903993 | 0.4412794 | ZDHHC4 | | 55146 |
| ENSG00000146576 | 424.2457541 | -0.060280642 | 0.1044858 | 0.3994523 | 0.6582409 | INTS15 | | 79034 |
| ENSG00000236609 | 8.406441296 | 0.018330454 | 0.1370725 | 0.3502878 | NA | ZNF853 | | 54753 |
| ENSG00000205903 | 668.4726586 | -0.058429347 | 0.0902978 | 0.3974392 | 0.6567942 | ZNF316 | | 100131017 |
| ENSG00000260054 | 410.796319 | -0.02408894 | 0.0981735 | 0.725523 | 0.8769175 | NA | NA | |
| ENSG00000164631 | 502.2162066 | 0.030331533 | 0.0951593 | 0.6635268 | 0.8440675 | ZNF12 | | 7559 |
| ENSG00000187953 | 159.1149614 | 0.064197747 | 0.1283757 | 0.3357319 | 0.6031891 | PMS2CL | | 441194 |
| ENSG00000146574 | 345.3259778 | -0.106127183 | 0.1210327 | 0.1725653 | 0.4194205 | CCZ1B | | 221960 |
| ENSG00000106392 | 565.5531762 | 0.043817602 | 0.0934967 | 0.5278642 | 0.7546809 | C1GALT1 | | 56913 |
| ENSG00000272894 | 9.16824715 | -0.004920177 | 0.1351465 | 0.8114318 | NA | NA | NA | |
| ENSG00000164654 | 630.0332603 | -0.004999606 | 0.0880526 | 0.9396802 | 0.9764957 | MIOS | | 54468 |
| ENSG00000106399 | 480.0755282 | -0.2091813 | 0.1355034 | 0.0239683 | 0.1174264 | RPA3 | | 6119 |
| ENSG00000219545 | 160.8167705 | -0.093055535 | 0.1433127 | 0.1918396 | 0.4431181 | UMAD1 | | 729852 |
| ENSG00000106415 | 67.37273765 | 0.19178522 | 0.3201208 | 0.023783 | 0.1168136 | GLCCI1 | | 113263 |
| ENSG00000003147 | 57.87565789 | -0.012011328 | 0.1281915 | 0.7918559 | 0.9129646 | ICA1 | | 3382 |
| ENSG00000189043 | 1483.455535 | -0.131564158 | 0.0812776 | 0.050724 | 0.1940738 | NDUFA4 | | 4697 |
| ENSG00000106443 | 815.4850813 | -0.049855469 | 0.0851876 | 0.4540258 | 0.6992058 | PHF14 | | 9678 |
| ENSG00000106460 | 446.8510413 | -0.023144997 | 0.0966915 | 0.7355038 | 0.8837091 | TMEM106B | | 54664 |
| ENSG00000146530 | 127.3961227 | -0.165909344 | 0.2131728 | 0.0547024 | 0.2048666 | VWDE | | 221806 |
| ENSG00000122644 | 267.8883131 | -0.030247272 | 0.1113549 | 0.6488008 | 0.836587 | ARL4A | | 10124 |
| ENSG00000006468 | 107.0767748 | -0.014971337 | 0.1219525 | 0.7891833 | 0.9116361 | ETV1 | | 2115 |
| ENSG00000214960 | 12.57258047 | -0.008385061 | 0.1347141 | 0.7320373 | NA | CRPPA | | 729920 |
| ENSG00000106524 | 345.722747 | -0.062513562 | 0.1080983 | 0.3846993 | 0.6452718 | ANKMY2 | | 57037 |
| ENSG00000136261 | 890.7949009 | 0.017696815 | 0.0811139 | 0.7872779 | 0.9115973 | BZW2 | | 28969 |
| ENSG00000106537 | 55.41765644 | -0.059782882 | 0.1438105 | 0.2291002 | 0.4903114 | TSPAN13 | | 27075 |
| ENSG00000106546 | 352.8948882 | 0.211132237 | 0.1518901 | 0.0287367 | 0.1343767 | AHR | | 196 |
| ENSG00000071189 | 614.8932754 | -0.059084218 | 0.0939261 | 0.3984421 | 0.657559 | SNX13 | | 23161 |
| ENSG00000048052 | 266.235469 | 0.469197426 | 0.1863126 | 0.0007528 | 0.0089186 | HDAC9 | | 9734 |
| ENSG00000105849 | 753.6301151 | 0.003595193 | 0.0888831 | 0.9584664 | 0.9831259 | POLR1F | | 221830 |
| ENSG00000213860 | 39.01752302 | -0.030877234 | 0.1345148 | 0.4512308 | NA | RPL21P75 | | 728501 |
| ENSG00000105855 | 337.6619805 | -0.03436249 | 0.110537 | 0.6394385 | 0.8306035 | ITGB8 | | 3696 |
| ENSG00000105866 | 300.9643716 | -0.201144621 | 0.1621383 | 0.0369301 | 0.1587321 | SP4 | | 6671 |
| ENSG00000105877 | 13.60492365 | 0.018038769 | 0.1363037 | 0.4480482 | NA | DNAH11 | | 8701 |
| ENSG00000164649 | 752.353534 | 0.02015089 | 0.0846543 | 0.7628969 | 0.8982943 | CDCA7L | | 55536 |
| ENSG00000136237 | 392.5439636 | -0.244532256 | 0.1493126 | 0.0147475 | 0.0832199 | RAPGEF5 | | 9771 |
| ENSG00000105889 | 94.67884585 | 0.067517375 | 0.1402709 | 0.2605777 | 0.5279074 | STEAP1B | | 256227 |
| ENSG00000233476 | 215.6079932 | -0.081625445 | 0.1296999 | 0.2563744 | 0.5231846 | NA | NA | |
| ENSG00000232759 | 221.8970574 | 0.108653088 | 0.1387586 | 0.1620622 | 0.4039797 | STEAP1B-A | | 100506178 |
| ENSG00000179428 | 22.90580437 | 0.014164132 | 0.1335736 | 0.6646245 | NA | IL6-AS1 | | 541472 |
| ENSG00000136244 | 1251.364596 | 1.20337803 | 0.0995905 | 7.53E-35 | 6.77E-32 | IL6 | | 3569 |
| ENSG00000196683 | 871.307377 | -0.02536595 | 0.0870383 | 0.7054135 | 0.8669966 | TOMM7 | | 54543 |
| ENSG00000228649 | 198.5615332 | 0.08891606 | 0.1363862 | 0.2191235 | 0.4793377 | SNHG26 | | 109729180 |
| ENSG00000221740 | 13.12831022 | 0.01924553 | 0.1363672 | 0.4329389 | NA | SNORD93 | | 692210 |
| ENSG00000122591 | 1113.641687 | 0.054904295 | 0.0832737 | 0.4117513 | 0.6661689 | HYCC1 | | 84668 |
| ENSG00000230658 | 23.20030614 | -0.023129298 | 0.1353597 | 0.4725301 | NA | NA | NA | |
| ENSG00000122550 | 526.2149602 | 0.015734351 | 0.0917989 | 0.8184011 | 0.9254567 | KLHL7 | | 55975 |
| ENSG00000136243 | 497.5256106 | -0.002344204 | 0.0931091 | 0.9711448 | 0.9885548 | NUP42 | | 11097 |
| ENSG00000156928 | 388.7427547 | -0.055857879 | 0.106274 | 0.4202924 | 0.6729863 | MALSU1 | | 115416 |
| ENSG00000136231 | 716.1155466 | -0.041681087 | 0.0906707 | 0.543405 | 0.7661361 | IGF2BP3 | | 10643 |
| ENSG00000232818 | 17.16380453 | -0.002767596 | 0.1337775 | 0.9174466 | NA | RPS2P32 | | 256355 |
| ENSG00000164548 | 1207.30365 | -0.126770592 | 0.0847762 | 0.065209 | 0.230401 | TRA2A | | 29896 |
| ENSG00000169193 | 93.92222399 | -0.035977129 | 0.1276523 | 0.5179934 | 0.7470898 | CCDC126 | | 90693 |
| ENSG00000188732 | 45.03104352 | -0.019212314 | 0.1307815 | 0.651662 | 0.8386102 | FAM221A | | 340277 |
| ENSG00000196335 | 12.27250373 | -0.024994264 | 0.1377265 | 0.3089961 | NA | STK31 | | 56164 |
| ENSG00000105926 | 453.8538024 | -0.051046651 | 0.1001521 | 0.4685334 | 0.7105867 | PALS2 | | 51678 |
| ENSG00000105928 | 116.359165 | -0.057773108 | 0.1324727 | 0.3438186 | 0.6102637 | GSDME | | 1687 |

| | | | | | | | | |
|---|---|---|---|---|---|---|---|---|
| ENSG00000070882 | 994.8748527 | 0.215075919 | 0.0999081 | 0.0072107 | 0.0498688 | OSBPL3 | | 26031 |
| ENSG00000172115 | 4394.073911 | 0.110293164 | 0.06141 | 0.0443921 | 0.1790227 | CYCS | | 54205 |
| ENSG00000153790 | 56.63788746 | -0.041441278 | 0.1354369 | 0.3808402 | 0.6418401 | SPMIP4 | | 136895 |
| ENSG00000050344 | 235.471297 | -0.042443513 | 0.112433 | 0.5325694 | 0.7578048 | NFE2L3 | | 9603 |
| ENSG00000273237 | 33.09677072 | 0.008442491 | 0.1313419 | 0.8237699 | NA | NA | NA | |
| ENSG00000122566 | 27586.01827 | -0.358768756 | 0.0549949 | 8.99E-12 | 9.30E-10 | HNRNPA2B1 | | 3181 |
| ENSG00000122565 | 4064.408036 | -0.297721922 | 0.0641766 | 5.70E-07 | 2.11E-05 | CBX3 | | 11335 |
| ENSG00000086300 | 286.6017342 | -0.120458502 | 0.134914 | 0.1341553 | 0.3608261 | SNX10 | | 29887 |
| ENSG00000005020 | 403.0633183 | 0.014521797 | 0.0984123 | 0.8333852 | 0.9305394 | SKAP2 | | 8935 |
| ENSG00000105991 | 49.64663041 | -0.017567794 | 0.1298338 | 0.6895734 | 0.8588425 | HOXA1 | | 3198 |
| ENSG00000233429 | 47.25654699 | 0.027868906 | 0.1323068 | 0.5285421 | 0.7549401 | HOTAIRM1 | | 100506311 |
| ENSG00000105997 | 59.82023515 | -0.050164186 | 0.1384297 | 0.3078387 | 0.5769089 | HOXA3 | | 3200 |
| ENSG00000253552 | 12.03720163 | -0.02302214 | 0.1372632 | 0.3434779 | NA | NA | NA | |
| ENSG00000197576 | 15.2079785 | 0.012092691 | 0.1346826 | 0.6513913 | NA | HOXA4 | | 3201 |
| ENSG00000254369 | 8.845892505 | -0.008787666 | 0.1354936 | 0.6659639 | NA | HOXA-AS3 | | 100133311 |
| ENSG00000106004 | 31.55074426 | 0.013978299 | 0.1321112 | 0.706916 | NA | HOXA5 | | 3202 |
| ENSG00000106006 | 9.152734775 | 9.64E-05 | 0.1349333 | 0.9993909 | NA | HOXA6 | | 3203 |
| ENSG00000122592 | 91.37839023 | -0.040750094 | 0.1308668 | 0.4488554 | 0.6959957 | HOXA7 | | 3204 |
| ENSG00000078399 | 62.11734053 | -0.06417451 | 0.1455929 | 0.2121065 | 0.468827 | HOXA9 | | 3205 |
| ENSG00000253187 | 126.742669 | -0.004181073 | 0.1199274 | 0.942528 | 0.9777681 | HOXA10-AS | | 100874323 |
| ENSG00000253293 | 75.97859573 | 0.011157555 | 0.1254238 | 0.8277775 | 0.9289335 | HOXA10 | | 3206 |
| ENSG00000233830 | 30.54276391 | -0.017112107 | 0.1330339 | 0.630559 | NA | NA | NA | |
| ENSG00000106049 | 323.5340609 | -0.058885795 | 0.1141826 | 0.4052116 | 0.6614808 | HIBADH | | 11112 |
| ENSG00000229893 | 10.21521696 | -0.004245457 | 0.1365242 | 0.5982902 | NA | NA | NA | |
| ENSG00000106052 | 1436.915148 | -0.082284324 | 0.0762497 | 0.196637 | 0.4494065 | TAX1BP1 | | 8887 |
| ENSG00000153814 | 60.40063731 | -0.05310764 | 0.1396989 | 0.2868348 | 0.5568053 | JAZF1 | | 221895 |
| ENSG00000146592 | 258.7146389 | 0.078034431 | 0.1219601 | 0.2846785 | 0.5539446 | CREB5 | | 9586 |
| ENSG00000225264 | 36.78481236 | -0.02027872 | 0.1326738 | 0.5971289 | NA | NA | NA | |
| ENSG00000227855 | 8.81904499 | 0.010287737 | 0.1358274 | 0.5992253 | NA | NA | NA | |
| ENSG00000136193 | 3134.545577 | -0.086832057 | 0.0629221 | 0.1195057 | 0.3374295 | SCRN1 | | 9805 |
| ENSG00000106080 | 447.4383806 | 0.023854517 | 0.1007478 | 0.7295231 | 0.8794775 | FKBP14 | | 55033 |
| ENSG00000106086 | 402.4946264 | -0.071529831 | 0.1074646 | 0.3257482 | 0.5942627 | PLEKHA8 | | 84725 |
| ENSG00000180354 | 128.2545568 | 0.025730102 | 0.1220074 | 0.662267 | 0.8437964 | MTURN | | 222166 |
| ENSG00000180233 | 165.0362109 | 0.034535991 | 0.1188448 | 0.5877598 | 0.7978643 | ZNRF2 | | 223082 |
| ENSG00000235859 | 33.28628459 | 0.015193005 | 0.1319422 | 0.6913401 | NA | NA | NA | |
| ENSG00000106100 | 160.5073361 | -0.034626594 | 0.1184224 | 0.5857932 | 0.7968554 | NOD1 | | 10392 |
| ENSG00000006625 | 814.5075384 | 0.067639995 | 0.0893124 | 0.3287539 | 0.5969491 | GGCT | | 79017 |
| ENSG00000196295 | 114.0343078 | -0.009735924 | 0.121166 | 0.8615279 | 0.9443096 | NA | NA | |
| ENSG00000106105 | 3466.265334 | 0.185755801 | 0.0689929 | 0.0023507 | 0.0214023 | GARS1 | | 2617 |
| ENSG00000106355 | 671.9058308 | -0.098630837 | 0.0998603 | 0.1804627 | 0.4299157 | LSM5 | | 23658 |
| ENSG00000105778 | 1083.594665 | 0.155908851 | 0.0899537 | 0.0315053 | 0.14242 | AVL9 | | 23080 |
| ENSG00000229358 | 21.58519572 | -0.01905945 | 0.134692 | 0.5424351 | NA | NA | NA | |
| ENSG00000237004 | 54.0869994 | -0.027020625 | 0.1310973 | 0.5556625 | 0.7755638 | NA | NA | |
| ENSG00000273014 | 48.70267581 | -0.026022819 | 0.1317565 | 0.5542342 | 0.7749708 | NA | NA | |
| ENSG00000231952 | 11.20065049 | -0.011985265 | 0.1353384 | 0.6077672 | NA | NA | NA | |
| ENSG00000170852 | 1674.17399 | -0.004563768 | 0.0699059 | 0.9386004 | 0.9762079 | KBTBD2 | | 25948 |
| ENSG00000205763 | 253.3999519 | 0.037580758 | 0.1129771 | 0.5779237 | 0.7911317 | NA | NA | |
| ENSG00000122642 | 1249.684238 | 0.01395457 | 0.075179 | 0.8250104 | 0.9280573 | FKBP9 | | 11328 |
| ENSG00000122643 | 885.3598913 | 0.085136428 | 0.0876633 | 0.2192125 | 0.4793377 | NT5C3A | | 51251 |
| ENSG00000164610 | 293.7759823 | 0.526887304 | 0.1747405 | 0.0001604 | 0.0025597 | RP9 | | 6100 |
| ENSG00000122507 | 230.6191657 | -0.224362315 | 0.1876731 | 0.0296258 | 0.1371792 | BBS9 | | 27241 |
| ENSG00000164619 | 8.768228256 | 0.044986463 | 0.1465072 | 0.0253429 | NA | BMPER | | 168667 |
| ENSG00000173852 | 1732.630229 | -0.204673637 | 0.0821776 | 0.0034315 | 0.0283078 | DPY19L1 | | 23333 |
| ENSG00000189212 | 375.5038814 | 0.107284532 | 0.1217855 | 0.1700109 | 0.4157205 | NA | NA | |
| ENSG00000164532 | 188.304013 | 0.103218403 | 0.1468654 | 0.1647877 | 0.4085071 | TBX20 | | 57057 |
| ENSG00000122557 | 408.2310486 | 0.004664181 | 0.096933 | 0.9469868 | 0.9786934 | HERPUD2 | | 64224 |
| ENSG00000271122 | 98.0652797 | -0.01067215 | 0.1219702 | 0.8468119 | 0.9372292 | HERPUD2-A | | 101930085 |
| ENSG00000228878 | 10.66060705 | 0.004149747 | 0.1348069 | 0.8570188 | NA | NA | NA | |
| ENSG00000122545 | 4495.200637 | -0.251497896 | 0.0659396 | 2.88E-05 | 0.0006151 | SEPTIN7 | | 989 |
| ENSG00000122547 | 86.16711065 | 0.06937427 | 0.1440822 | 0.2301325 | 0.4913503 | EEPD1 | | 80820 |
| ENSG00000164542 | 219.3628573 | -0.010190194 | 0.1108234 | 0.8732622 | 0.9487142 | MATCAP2 | | 23366 |
| ENSG00000011426 | 6096.485695 | -0.237196698 | 0.0607757 | 2.23E-05 | 0.0004947 | ANLN | | 54443 |
| ENSG00000010270 | 610.4583359 | -0.111258695 | 0.1034223 | 0.140357 | 0.3697587 | STARD3NL | | 83930 |
| ENSG00000006715 | 1209.666514 | 0.058779372 | 0.0771217 | 0.3562164 | 0.6197178 | VPS41 | | 27072 |
| ENSG00000241127 | 186.042174 | -0.003738617 | 0.1123072 | 0.952367 | 0.9806656 | YAE1 | | 57002 |
| ENSG00000006451 | 1005.804551 | -0.080873481 | 0.083709 | 0.2264817 | 0.4875659 | RALA | | 5898 |
| ENSG00000188185 | 80.1009574 | 0.122933474 | 0.1942733 | 0.0646852 | 0.2292439 | NA | NA | |
| ENSG00000065883 | 679.6021819 | 0.045987995 | 0.0892679 | 0.502284 | 0.7365601 | CDK13 | | 8621 |
| ENSG00000168303 | 586.7298666 | 0.015979799 | 0.0892827 | 0.8145691 | 0.9241103 | MPLKIP | | 136647 |
| ENSG00000175600 | 23.97271547 | -0.016717548 | 0.1337276 | 0.6139901 | NA | SUGCT | | 79783 |

| | | | | | | | |
|---|---|---|---|---|---|---|---|
| ENSG00000122641 | 379.1469493 | 2.505470924 | 0.1703268 | 2.88E-50 | 6.73E-47 | INHBA | 3624 |
| ENSG00000106571 | 49.57755508 | -0.000162698 | 0.128208 | 0.994476 | 0.9977447 | GLI3 | 2737 |
| ENSG00000136197 | 14.89697623 | 0.034099534 | 0.140233 | 0.2018865 | NA | C7orf25 | 79020 |
| ENSG00000106588 | 126.315899 | -0.217017547 | 0.2750066 | 0.0310307 | 0.1413689 | PSMA2 | 5683 |
| ENSG00000106591 | 1188.149527 | 0.084772185 | 0.0837082 | 0.2102681 | 0.4667697 | MRPL32 | 64983 |
| ENSG00000164543 | 579.7272357 | 0.452534612 | 0.1232581 | 1.98E-05 | 0.0004529 | STK17A | 9263 |
| ENSG00000106603 | 954.6987674 | -0.015026464 | 0.0788107 | 0.8169593 | 0.9252377 | COA1 | 55744 |
| ENSG00000106605 | 396.7241741 | -0.038847895 | 0.1015484 | 0.5909876 | 0.8003856 | BLVRA | 644 |
| ENSG00000062582 | 29.40374629 | 0.079141793 | 0.1657477 | 0.0434562 | NA | MRPS24 | 64951 |
| ENSG00000106608 | 665.6375933 | 0.07068442 | 0.0930628 | 0.3163587 | 0.5849906 | URGCP | 55665 |
| ENSG00000078967 | 196.8408587 | 0.073216788 | 0.1265662 | 0.2993952 | 0.5693326 | UBE2D4 | 51619 |
| ENSG00000214783 | 12.35539628 | 0.012198405 | 0.1351533 | 0.6218388 | NA | POLR2J4 | 84820 |
| ENSG00000228434 | 24.08109105 | 0.010185726 | 0.1328798 | 0.7597491 | NA | NA | NA |
| ENSG00000272655 | 104.6143432 | -0.08269828 | 0.1469616 | 0.2013926 | 0.455463 | NA | NA |
| ENSG00000136279 | 1636.053466 | -0.067429543 | 0.0734239 | 0.2777477 | 0.5461905 | DBNL | 28988 |
| ENSG00000122678 | 126.8985945 | 0.046842586 | 0.1276632 | 0.4392613 | 0.6886014 | POLM | 27434 |
| ENSG00000106628 | 2129.873075 | -0.047415584 | 0.0676984 | 0.4194553 | 0.6722911 | POLD2 | 5425 |
| ENSG00000106636 | 2444.359579 | 0.09872222 | 0.0663028 | 0.0909506 | 0.2850744 | YKT6 | 10652 |
| ENSG00000015676 | 1262.122284 | 0.008018181 | 0.076624 | 0.9003597 | 0.9609925 | NUDCD3 | 23386 |
| ENSG00000136271 | 2102.317899 | 0.026690516 | 0.0709988 | 0.6590314 | 0.842576 | DDX56 | 54606 |
| ENSG00000158604 | 1570.841648 | 0.014637687 | 0.0695711 | 0.8076183 | 0.9200093 | TMED4 | 222068 |
| ENSG00000105953 | 1904.460393 | -0.004098119 | 0.0662589 | 0.9428192 | 0.9778352 | OGDH | 4967 |
| ENSG00000122515 | 1660.561552 | -0.000744882 | 0.07102 | 0.9893712 | 0.9955163 | ZMIZ2 | 83637 |
| ENSG00000196262 | 26715.69459 | -0.273679675 | 0.0546496 | 1.05E-07 | 5.01E-06 | PPIA | 5478 |
| ENSG00000105968 | 3644.384162 | -0.157031936 | 0.0648439 | 0.0064546 | 0.0459736 | H2AZ2 | 94239 |
| ENSG00000146676 | 1607.057329 | -0.024517706 | 0.0696111 | 0.6816063 | 0.8539085 | PURB | 5814 |
| ENSG00000272768 | 12.87403032 | 0.016240229 | 0.1356631 | 0.5192804 | NA | NA | NA |
| ENSG00000232956 | 1096.943436 | 0.466099029 | 0.1022434 | 4.34E-07 | 1.66E-05 | SNHG15 | 285958 |
| ENSG00000136280 | 1003.883216 | 0.018082804 | 0.079489 | 0.7806722 | 0.9087227 | CCM2 | 83605 |
| ENSG00000136270 | 1933.83204 | 0.025558056 | 0.0681462 | 0.6670275 | 0.8463555 | TBRG4 | 9238 |
| ENSG00000201772 | 10.17829519 | -0.004345167 | 0.1348414 | 0.8447112 | NA | SNORA5C | 677796 |
| ENSG00000214765 | 205.7941176 | 0.084799744 | 0.1308519 | 0.2419003 | 0.5076604 | NA | NA |
| ENSG00000146678 | 12.51709914 | 0.05791694 | 0.1531436 | 0.0231623 | NA | IGFBP1 | 3484 |
| ENSG00000136205 | 1192.437084 | -0.066016398 | 0.0870033 | 0.3330945 | 0.6004589 | TNS3 | 64759 |
| ENSG00000136273 | 431.7299029 | 0.113909319 | 0.1153701 | 0.1454565 | 0.3780081 | HUS1 | 3364 |
| ENSG00000183696 | 1731.172287 | 0.73169662 | 0.0866639 | 1.97E-18 | 4.91E-16 | UPP1 | 7378 |
| ENSG00000132436 | 1743.48796 | -0.068756904 | 0.0703023 | 0.2545071 | 0.5211459 | FIGNL1 | 63979 |
| ENSG00000106070 | 761.0639809 | 0.258340632 | 0.112767 | 0.0036287 | 0.0295795 | GRB10 | 2887 |
| ENSG00000106078 | 30.74693764 | -0.008956282 | 0.1319808 | 0.7990812 | NA | COBL | 23242 |
| ENSG00000132432 | 1582.254186 | -0.070299604 | 0.0738171 | 0.2599651 | 0.5270137 | SEC61G | 23480 |
| ENSG00000146648 | 11870.6523 | -0.040197884 | 0.056332 | 0.427391 | 0.6791494 | EGFR | 1956 |
| ENSG00000132434 | 369.9345128 | -0.25566079 | 0.1594169 | 0.0140019 | 0.0806586 | LANCL2 | 55915 |
| ENSG00000154978 | 1444.550245 | -0.065698777 | 0.08112 | 0.318612 | 0.5874833 | VOPP1 | 81552 |
| ENSG00000178665 | 56.94432874 | -0.028863888 | 0.1326919 | 0.5092716 | 0.7410048 | ZNF713 | 349075 |
| ENSG00000146729 | 883.3344339 | -0.312243519 | 0.1048632 | 0.0003803 | 0.0052391 | NIPSNAP2 | 2631 |
| ENSG00000239789 | 135.1396318 | 0.044992226 | 0.1245999 | 0.4714661 | 0.7119839 | MRPS17 | 51373 |
| ENSG00000146733 | 519.1513796 | 0.034764708 | 0.0961672 | 0.6233053 | 0.8197286 | PSPH | 5723 |
| ENSG00000146731 | 7580.160153 | -0.307384054 | 0.0565811 | 8.80E-09 | 5.38E-07 | CCT6A | 908 |
| ENSG00000129103 | 1100.056492 | -0.048366177 | 0.0812994 | 0.4611694 | 0.7055278 | SUMF2 | 25870 |
| ENSG00000164776 | 22.41439037 | 0.017376133 | 0.1341401 | 0.5917073 | NA | PHKG1 | 5260 |
| ENSG00000106153 | 4380.619111 | -0.059497579 | 0.0573986 | 0.2520047 | 0.5184753 | CHCHD2 | 51142 |
| ENSG00000237268 | 28.66252582 | -0.04342236 | 0.1410582 | 0.2416003 | NA | NA | NA |
| ENSG00000182111 | 293.302261 | -0.185284461 | 0.1574421 | 0.0470207 | 0.185557 | ZNF716 | 441234 |
| ENSG00000214652 | 17.23361631 | 0.012052792 | 0.134374 | 0.6704991 | NA | ZNF727 | 442319 |
| ENSG00000234444 | 555.3217944 | -0.078431066 | 0.0985585 | 0.2769355 | 0.5455116 | ZNF736 | 728927 |
| ENSG00000227986 | 17.29811341 | -0.018295121 | 0.1352828 | 0.5203069 | NA | NA | NA |
| ENSG00000173041 | 219.330493 | -0.049512206 | 0.1167402 | 0.4628951 | 0.7072698 | ZNF680 | 340252 |
| ENSG00000196247 | 363.5349706 | -0.0558208 | 0.1052092 | 0.4318023 | 0.6831378 | ZNF107 | 51427 |
| ENSG00000197008 | 125.0416509 | -0.066292015 | 0.1345685 | 0.2983437 | 0.5686006 | ZNF138 | 7697 |
| ENSG00000198039 | 131.3485331 | 0.036395258 | 0.1227509 | 0.5516489 | 0.7725183 | ZNF273 | 10793 |
| ENSG00000152926 | 58.01624493 | 0.003221822 | 0.1272037 | 0.9466627 | 0.9786934 | ZNF117 | 51351 |
| ENSG00000213462 | 57.24345444 | -0.047617067 | 0.1374828 | 0.3260378 | 0.5942836 | ERV3-1 | 2086 |
| ENSG00000234585 | 44.84662973 | 0.022608313 | 0.1316553 | 0.5956238 | 0.80359 | NA | NA |
| ENSG00000226002 | 12.67272242 | 0.039828793 | 0.1429844 | 0.1122894 | NA | GTF2IP14 | 106481735 |
| ENSG00000164669 | 35.71231382 | -0.00372902 | 0.1308465 | 0.9197823 | NA | NA | NA |
| ENSG00000146757 | 207.1682535 | 0.018671706 | 0.1156655 | 0.7678106 | 0.9003911 | ZNF92 | 168374 |
| ENSG00000228409 | 136.9914174 | 0.046653402 | 0.1247443 | 0.4585461 | 0.7028312 | NA | NA |
| ENSG00000196715 | 1341.979917 | -0.19047455 | 0.0865927 | 0.0081348 | 0.0546738 | VKORC1L1 | 154807 |
| ENSG00000169919 | 1132.854991 | 0.073526132 | 0.08011 | 0.2651873 | 0.5327957 | GUSB | 2990 |
| ENSG00000126522 | 560.5049394 | 0.101763981 | 0.1033942 | 0.1740581 | 0.4217326 | ASL | 435 |

| | | | | | | | | |
|---|---|---|---|---|---|---|---|---|
| ENSG00000241258 | 682.1603476 | 0.130264988 | 0.1021514 | 0.0888976 | 0.2808797 | CRCP | | 27297 |
| ENSG00000169902 | 170.7875423 | -0.04441428 | 0.1213441 | 0.4900262 | 0.7267241 | TPST1 | | 8460 |
| ENSG00000179406 | 20.45855572 | 0.012348291 | 0.133723 | 0.6917809 | NA | LINC00174 | | 285908 |
| ENSG00000223473 | 10.87239882 | -0.000883035 | 0.1346876 | 0.9666744 | NA | NA | NA | |
| ENSG00000229180 | 81.31043947 | 0.023252596 | 0.1262767 | 0.6601872 | 0.8426737 | NA | NA | |
| ENSG00000232559 | 125.6828874 | 0.018739818 | 0.1205576 | 0.749881 | 0.8918102 | NA | NA | |
| ENSG00000243335 | 513.3761565 | 0.023945496 | 0.0933021 | 0.7278427 | 0.8782138 | KCTD7 | | 154881 |
| ENSG00000226824 | 40.37671271 | 0.052716504 | 0.1432979 | 0.2238416 | NA | LOC100996‹ | | 100996437 |
| ENSG00000154710 | 29.2589203 | 0.017996318 | 0.1335079 | 0.6051791 | NA | RABGEF1 | | 27342 |
| ENSG00000272831 | 41.76957595 | 0.00875199 | 0.1304911 | 0.8271651 | 0.9287505 | NA | NA | |
| ENSG00000230583 | 8.384754359 | -0.009610219 | 0.1355904 | 0.6363204 | NA | NA | NA | |
| ENSG00000232546 | 14.25409895 | 0.020779663 | 0.1363998 | 0.4242792 | NA | NA | NA | |
| ENSG00000230295 | 12.96108287 | 0.021835895 | 0.1368219 | 0.3866335 | NA | NA | NA | |
| ENSG00000273142 | 43.5504961 | 0.150749834 | 0.2695526 | 0.0140036 | 0.0806586 | NA | NA | |
| ENSG00000106609 | 1760.325291 | -0.050294479 | 0.0710811 | 0.4337612 | 0.684513 | TMEM248 | | 55069 |
| ENSG00000126524 | 1705.663142 | 0.123119352 | 0.0752439 | 0.0537506 | 0.2024199 | SBDS | | 51119 |
| ENSG00000198874 | 363.9310492 | -0.089290162 | 0.1160436 | 0.2350054 | 0.4984047 | TYW1 | | 55253 |
| ENSG00000067601 | 66.80436399 | -0.049835782 | 0.1372804 | 0.3265428 | 0.5947303 | NA | NA | |
| ENSG00000106610 | 196.6006836 | 0.00299614 | 0.1115454 | 0.964605 | 0.9858737 | NA | NA | |
| ENSG00000273448 | 14.6839348 | -0.015209537 | 0.1355511 | 0.5368538 | NA | NA | NA | |
| ENSG00000254184 | 49.13631757 | 0.041924918 | 0.1372661 | 0.344763 | 0.610988 | NA | NA | |
| ENSG00000225648 | 833.5079082 | 0.118037793 | 0.1016148 | 0.1173573 | 0.3339561 | NA | NA | |
| ENSG00000196313 | 1458.57906 | -0.003188545 | 0.0735425 | 0.9578984 | 0.9829756 | POM121 | | 9883 |
| ENSG00000106133 | 172.0520294 | 0.078351801 | 0.1324867 | 0.2636348 | 0.5315012 | NA | NA | |
| ENSG00000174353 | 267.5525335 | 0.000955457 | 0.1072806 | 0.9897422 | 0.9955163 | NA | NA | |
| ENSG00000229018 | 11.96629429 | 0.002343672 | 0.1346026 | 0.9226749 | NA | NA | NA | |
| ENSG00000233369 | 57.80973334 | -0.020854824 | 0.1299893 | 0.640606 | 0.8315061 | GTF2IP4 | | 100093631 |
| ENSG00000205578 | 10.83383502 | -0.012324228 | 0.1355538 | 0.5830773 | NA | NA | NA | |
| ENSG00000130305 | 1085.398435 | 0.18413559 | 0.0917563 | 0.0134558 | 0.0783146 | NSUN5 | | 55695 |
| ENSG00000009954 | 4447.320512 | -0.057736703 | 0.0588341 | 0.2758136 | 0.5440356 | BAZ1B | | 9031 |
| ENSG00000106635 | 1056.254151 | -0.073552404 | 0.0826683 | 0.2703684 | 0.5381601 | BCL7B | | 9275 |
| ENSG00000106638 | 463.7459812 | 0.030105727 | 0.0956611 | 0.6627807 | 0.8438537 | TBL2 | | 26608 |
| ENSG00000176428 | 28.15697255 | -0.025681133 | 0.1349712 | 0.4707613 | NA | VPS37D | | 155382 |
| ENSG00000176410 | 168.4014586 | -0.00400043 | 0.1137262 | 0.9481325 | 0.9794071 | DNAJC30 | | 84277 |
| ENSG00000071462 | 1526.798128 | 0.083571847 | 0.0748223 | 0.185201 | 0.435663 | BUD23 | | 114049 |
| ENSG00000106089 | 337.6883864 | 0.217266918 | 0.1556956 | 0.0267686 | 0.1271074 | STX1A | | 6804 |
| ENSG00000106077 | 303.6494672 | 0.141700616 | 0.140888 | 0.0942153 | 0.2910683 | ABHD11 | | 83451 |
| ENSG00000189143 | 91.0669745 | 0.006369141 | 0.1237793 | 0.9055334 | 0.9626443 | CLDN4 | | 1364 |
| ENSG00000165171 | 152.0691952 | 0.210431214 | 0.2294618 | 0.0370905 | 0.1591676 | METTL27 | | 155368 |
| ENSG00000106683 | 517.3535692 | -0.090176899 | 0.103471 | 0.2209465 | 0.4804312 | LIMK1 | | 3984 |
| ENSG00000106682 | 6661.231248 | -0.075545233 | 0.054707 | 0.1310917 | 0.3559467 | EIF4H | | 7458 |
| ENSG00000207741 | 16.01868271 | 0.003449487 | 0.1337986 | 0.905894 | NA | MIR590 | | 693175 |
| ENSG00000086730 | 659.3333479 | -0.423020683 | 0.1166559 | 2.54E-05 | 0.0005531 | LAT2 | | 7462 |
| ENSG00000049541 | 1378.849312 | 0.256202123 | 0.0858965 | 0.0005427 | 0.0068381 | RFC2 | | 5982 |
| ENSG00000106665 | 2738.66783 | -0.302318632 | 0.0789229 | 1.88E-05 | 0.0004316 | CLIP2 | | 7461 |
| ENSG00000006704 | 231.8873745 | 0.262931284 | 0.202732 | 0.0193519 | 0.1012764 | GTF2IRD1 | | 9569 |
| ENSG00000077809 | 2048.995712 | -0.127327987 | 0.0757876 | 0.0472137 | 0.1857336 | NA | NA | |
| ENSG00000232729 | 31.14509607 | -0.022906074 | 0.1342638 | 0.5204847 | NA | GTF2I-AS1 | | 101926943 |
| ENSG00000160828 | 224.6402661 | -0.032975251 | 0.1120872 | 0.6212352 | 0.818389 | NA | NA | |
| ENSG00000196275 | 32.44123089 | 0.016931526 | 0.1323088 | 0.65617 | NA | GTF2IRD2 | | 84163 |
| ENSG00000123965 | 72.58263289 | 0.047776222 | 0.1345568 | 0.3717271 | 0.6338897 | NA | NA | |
| ENSG00000174374 | 744.0307821 | -0.1108389 | 0.0980816 | 0.1331264 | 0.3595479 | NA | NA | |
| ENSG00000174428 | 48.62849422 | 0.071653094 | 0.1538066 | 0.1358807 | 0.3628767 | GTF2IRD2B | | 389524 |
| ENSG00000232561 | 455.7654007 | -0.075663222 | 0.1084938 | 0.3018071 | 0.5711013 | NA | NA | |
| ENSG00000230195 | 22.22450397 | -0.016445306 | 0.1341344 | 0.6014305 | NA | NA | NA | |
| ENSG00000174368 | 14.7370104 | 0.008773833 | 0.1344188 | 0.7414284 | NA | NA | NA | |
| ENSG00000205583 | 146.1309839 | -0.035971628 | 0.1208186 | 0.5644752 | 0.7820587 | NA | NA | |
| ENSG00000223705 | 198.2136474 | -0.023758898 | 0.1133268 | 0.7137048 | 0.870525 | NSUN5P1 | | 155400 |
| ENSG00000135213 | 1535.79189 | 0.057880542 | 0.0739667 | 0.3540759 | 0.6187516 | NA | NA | |
| ENSG00000127957 | 126.6718466 | 0.349615821 | 0.2990875 | 0.0120227 | 0.0724273 | NA | NA | |
| ENSG00000127946 | 974.037983 | -0.184395014 | 0.0966225 | 0.0163987 | 0.0896782 | HIP1 | | 3092 |
| ENSG00000005486 | 633.1518249 | 0.313075677 | 0.1184407 | 0.0010174 | 0.0112642 | RHBDD2 | | 57414 |
| ENSG00000127948 | 1037.642983 | -0.067476776 | 0.0813517 | 0.3065117 | 0.5757136 | POR | | 5447 |
| ENSG00000189077 | 221.5505114 | -0.040483524 | 0.1145972 | 0.5437281 | 0.7661909 | TMEM120A | | 83862 |
| ENSG00000127952 | 362.5836188 | -0.007373259 | 0.0992342 | 0.9128778 | 0.9656034 | STYXL1 | | 51657 |
| ENSG00000146701 | 2390.484947 | 0.015339788 | 0.0646626 | 0.7829863 | 0.9096561 | MDH2 | | 4191 |
| ENSG00000177679 | 13.25263983 | -0.012724888 | 0.1350498 | 0.614791 | NA | SRRM3 | | 222183 |
| ENSG00000106211 | 1157.636981 | 0.046025271 | 0.0772681 | 0.4651507 | 0.7086768 | HSPB1 | | 3315 |
| ENSG00000170027 | 4530.147894 | -0.050987007 | 0.0565141 | 0.3225568 | 0.5913034 | YWHAG | | 7532 |
| ENSG00000146700 | 12.27191276 | 0.016851275 | 0.1359175 | 0.4923426 | NA | SSC4D | | 136853 |

| | | | | | | | |
|---|---|---|---|---|---|---|---|
| ENSG00000188372 | 37.83685254 | 0.006553855 | 0.1302633 | 0.8717849 | NA | ZP3 | 7784 |
| ENSG00000091073 | 163.8239396 | 0.016926382 | 0.1152869 | 0.7892976 | 0.9116361 | DTX2 | 113878 |
| ENSG00000205485 | 88.32211639 | 0.479232932 | 0.3874979 | 0.0071188 | 0.0494307 | NA | NA |
| ENSG00000146707 | 192.3624664 | 0.04336735 | 0.1206554 | 0.5033022 | 0.7374652 | POMZP3 | 22932 |
| ENSG00000186704 | 9.598360545 | 0.005682778 | 0.1351028 | 0.7926124 | NA | NA | NA |
| ENSG00000135205 | 19.04128778 | -0.015587043 | 0.1345774 | 0.5948509 | NA | CCDC146 | 57639 |
| ENSG00000186088 | 27.22108746 | 0.04858774 | 0.1439509 | 0.183178 | NA | GSAP | 54103 |
| ENSG00000127947 | 1840.009195 | -0.272419694 | 0.0779651 | 8.53E-05 | 0.0015361 | PTPN12 | 5782 |
| ENSG00000214293 | 141.4381768 | 0.08362349 | 0.1409282 | 0.2219908 | 0.4815702 | APTR | 100505854 |
| ENSG00000187257 | 585.0229356 | -0.116294565 | 0.1055452 | 0.1277972 | 0.3512469 | RSBN1L | 222194 |
| ENSG00000135211 | 148.877859 | 0.001510517 | 0.1158547 | 0.9815231 | 0.9932057 | TMEM60 | 85025 |
| ENSG00000006576 | 710.1121436 | -0.062671095 | 0.0895824 | 0.36363 | 0.6265951 | PHTF2 | 57157 |
| ENSG00000234456 | 95.8952303 | 0.027268597 | 0.1246977 | 0.6328279 | 0.8252447 | MAGI2-AS3 | 100505881 |
| ENSG00000127955 | 250.0442923 | 0.301019366 | 0.193804 | 0.0108415 | 0.067147 | GNAI1 | 2770 |
| ENSG00000075223 | 3380.319282 | -0.273578667 | 0.0664014 | 6.92E-06 | 0.0001822 | SEMA3C | 10512 |
| ENSG00000153956 | 331.5001268 | 0.057621552 | 0.108363 | 0.4190537 | 0.6718301 | CACNA2D1 | 781 |
| ENSG00000186472 | 115.5945512 | 0.008550705 | 0.1209205 | 0.8814809 | 0.9522588 | PCLO | 27445 |
| ENSG00000075213 | 15.13721185 | 0.028646273 | 0.1385312 | 0.2686322 | NA | SEMA3A | 10371 |
| ENSG00000153993 | 9.34727715 | -0.016146394 | 0.1363309 | 0.4502724 | NA | SEMA3D | 223117 |
| ENSG00000164659 | 347.5917328 | -0.034827197 | 0.1037665 | 0.6142533 | 0.8156296 | ELAPOR2 | 222223 |
| ENSG00000135164 | 860.4355639 | 0.011099337 | 0.0824153 | 0.8668096 | 0.9461922 | DMTF1 | 9988 |
| ENSG00000135185 | 268.590497 | 0.143144379 | 0.1583616 | 0.091927 | 0.2871888 | TMEM243 | 79161 |
| ENSG00000182165 | 39.71066315 | -0.014628373 | 0.1310722 | 0.7135352 | NA | TP53TG1 | 11257 |
| ENSG00000005469 | 207.3940477 | -0.111570118 | 0.1430949 | 0.1511661 | 0.3877407 | CROT | 54677 |
| ENSG00000085563 | 72.82486707 | 0.094707396 | 0.1662583 | 0.1076051 | 0.3172477 | ABCB1 | 5243 |
| ENSG00000105784 | 8.724349782 | -0.016002901 | 0.1364075 | 0.4421341 | NA | RUNDC3B | 154661 |
| ENSG00000075303 | 436.6367383 | 0.004732195 | 0.096105 | 0.9458048 | 0.9784014 | SLC25A40 | 55972 |
| ENSG00000006634 | 676.8651126 | -0.072342187 | 0.0936124 | 0.3052837 | 0.5743305 | DBF4 | 10926 |
| ENSG00000008277 | 226.0097312 | -0.049736392 | 0.1165072 | 0.465249 | 0.7086875 | ADAM22 | 53616 |
| ENSG00000075142 | 802.0618329 | -0.122562767 | 0.0990277 | 0.1008421 | 0.303592 | SRI | 6717 |
| ENSG00000164647 | 18.30007713 | 0.014665397 | 0.1346115 | 0.6105815 | NA | STEAP1 | 26872 |
| ENSG00000157214 | 31.42614573 | 0.018179837 | 0.1327437 | 0.626733 | NA | STEAP2 | 261729 |
| ENSG00000105792 | 69.41526832 | 0.144224474 | 0.2317585 | 0.0363899 | 0.1572005 | CFAP69 | 79846 |
| ENSG00000105793 | 517.8828362 | 0.126925629 | 0.1168787 | 0.1120895 | 0.3248122 | GTPBP10 | 85865 |
| ENSG00000157224 | 545.7344679 | 0.076538652 | 0.1003433 | 0.2965482 | 0.5664726 | CLDN12 | 9069 |
| ENSG00000058091 | 52.06885494 | -0.0204827 | 0.1298234 | 0.6507508 | 0.8378754 | CDK14 | 5218 |
| ENSG00000157240 | 115.6851063 | -0.069963792 | 0.1379706 | 0.2700719 | 0.538071 | FZD1 | 8321 |
| ENSG00000127989 | 286.1781333 | 0.084994361 | 0.1201021 | 0.2608897 | 0.5281547 | MTERF1 | 7978 |
| ENSG00000127914 | 1855.948161 | -0.01132451 | 0.0712807 | 0.854008 | 0.9409946 | AKAP9 | 10142 |
| ENSG00000001630 | 226.0721659 | -0.4734211 | 0.2193406 | 0.0017235 | 0.0170032 | CYP51A1 | 1595 |
| ENSG00000001631 | 897.9069544 | -0.193016678 | 0.1026287 | 0.0160345 | 0.0883902 | KRIT1 | 889 |
| ENSG00000243107 | 12.23522667 | 0.014138706 | 0.1354778 | 0.562592 | NA | NA | NA |
| ENSG00000001629 | 1292.228396 | -0.087467753 | 0.0786907 | 0.1787612 | 0.4275952 | ANKIB1 | 54467 |
| ENSG00000157259 | 581.0690321 | 0.07794224 | 0.0980712 | 0.2794969 | 0.5478778 | GATAD1 | 57798 |
| ENSG00000244055 | 14.19376184 | 0.021969745 | 0.1366124 | 0.4036637 | NA | NA | NA |
| ENSG00000127980 | 252.8821483 | -0.039044138 | 0.1115471 | 0.5647124 | 0.7820587 | PEX1 | 5189 |
| ENSG00000127993 | 273.0307416 | 0.145924242 | 0.1561575 | 0.0883805 | 0.2798515 | RBM48 | 84060 |
| ENSG00000234545 | 801.6426742 | 0.077275524 | 0.0945862 | 0.2783184 | 0.5467586 | FAM133B | 257415 |
| ENSG00000105810 | 2754.475207 | -0.037918445 | 0.0629676 | 0.4966285 | 0.7319099 | CDK6 | 1021 |
| ENSG00000205413 | 21.08518419 | -0.003973156 | 0.133268 | 0.8916581 | NA | SAMD9 | 54809 |
| ENSG00000004766 | 416.7598693 | -0.058004208 | 0.1042051 | 0.415829 | 0.6697358 | VPS50 | 55610 |
| ENSG00000127928 | 49.55153421 | -0.042334968 | 0.1372309 | 0.3436425 | 0.6100437 | GNGT1 | 2792 |
| ENSG00000105825 | 3484.081175 | 0.457621153 | 0.2025564 | 0.0014603 | 0.0150691 | TFPI2 | 7980 |
| ENSG00000127920 | 841.3209747 | -0.259213791 | 0.1142292 | 0.0037617 | 0.030409 | GNG11 | 2791 |
| ENSG00000105829 | 447.5697568 | 0.008999421 | 0.0956838 | 0.8959264 | 0.9584283 | BET1 | 10282 |
| ENSG00000127995 | 486.8312571 | 0.049291935 | 0.1025996 | 0.4852453 | 0.7234177 | CASD1 | 64921 |
| ENSG00000127990 | 727.147671 | -0.199313553 | 0.1158437 | 0.0199767 | 0.1037561 | SGCE | 8910 |
| ENSG00000242265 | 1753.936531 | 0.010475957 | 0.0715949 | 0.8643824 | 0.9455751 | PEG10 | 23089 |
| ENSG00000158528 | 280.1222593 | -0.056293323 | 0.1157905 | 0.4127191 | 0.6666675 | PPP1R9A | 55607 |
| ENSG00000105854 | 932.3566753 | -0.014301397 | 0.0892117 | 0.7829177 | 0.9096561 | PON2 | 5445 |
| ENSG00000004864 | 1151.175576 | -0.262364437 | 0.0937741 | 0.0009016 | 0.010205 | SLC25A13 | 10165 |
| ENSG00000127922 | 1620.768729 | -0.203437383 | 0.0814495 | 0.0033985 | 0.0280781 | SEM1 | 7979 |
| ENSG00000196636 | 214.0765341 | 0.04060077 | 0.114214 | 0.5473997 | 0.7690111 | SDHAF3 | 57001 |
| ENSG00000070669 | 2209.093118 | 0.477795375 | 0.0768956 | 4.61E-11 | 4.21E-09 | ASNS | 440 |
| ENSG00000183444 | 78.51682987 | 0.033724264 | 0.1287565 | 0.5284804 | 0.7549401 | OR7E38P | 389537 |
| ENSG00000243554 | 90.01231015 | 0.149285533 | 0.2204809 | 0.0492216 | 0.1906357 | CCZ1P1 | 110440226 |
| ENSG00000164715 | 1277.156572 | 0.026651155 | 0.0757357 | 0.6737098 | 0.8501877 | LMTK2 | 22853 |
| ENSG00000205356 | 465.7124738 | -0.167001692 | 0.127591 | 0.0522884 | 0.1983034 | TECPR1 | 25851 |
| ENSG00000164713 | 1019.559119 | 0.188034318 | 0.095335 | 0.0138978 | 0.0802872 | BRI3 | 25798 |
| ENSG00000006453 | 1759.366758 | -0.139136692 | 0.0799149 | 0.0369403 | 0.1587321 | BAIAP2L1 | 55971 |

| | | | | | | | | |
|---|---|---|---|---|---|---|---|---|
| ENSG00000272782 | 10.60224264 | -0.007102376 | 0.134997 | 0.7507144 | NA | NA | NA | |
| ENSG00000214389 | 69.38147152 | -0.037321235 | 0.1327672 | 0.4455495 | 0.6937435 | RPS3AP26 | | 644972 |
| ENSG00000196367 | 4052.842711 | -0.128355859 | 0.0625431 | 0.0213451 | 0.1086887 | TRRAP | | 8295 |
| ENSG00000242687 | 62.7741214 | -0.03525567 | 0.1327507 | 0.4599039 | 0.7041733 | NA | NA | |
| ENSG00000198742 | 1607.234354 | -0.251644625 | 0.0823235 | 0.0004392 | 0.0058292 | SMURF1 | | 57154 |
| ENSG00000002079 | 31.12731003 | 0.038477145 | 0.138482 | 0.3145916 | NA | MYH16 | | 84176 |
| ENSG00000241685 | 2701.892841 | 0.037104346 | 0.0631493 | 0.5109743 | 0.7421105 | ARPC1A | | 10552 |
| ENSG00000130429 | 2934.385825 | -0.006954494 | 0.0650283 | 0.9025704 | 0.9618324 | ARPC1B | | 10095 |
| ENSG00000106244 | 4200.813138 | -0.140167426 | 0.0602731 | 0.0097426 | 0.0626671 | PDAP1 | | 11333 |
| ENSG00000106245 | 1896.81534 | 0.37742357 | 0.0798177 | 2.56E-07 | 1.06E-05 | BUD31 | | 8896 |
| ENSG00000106246 | 180.2328184 | 0.251972988 | 0.2270786 | 0.0242093 | 0.1182036 | PTCD1 | | 26024 |
| ENSG00000160917 | 956.0006553 | 0.003539798 | 0.0841088 | 0.958359 | 0.9831259 | CPSF4 | | 10898 |
| ENSG00000241468 | 1452.466543 | -0.161647399 | 0.1010707 | 0.0375154 | 0.1604604 | ATP5MF | | 9551 |
| ENSG00000198556 | 258.2822617 | 0.162141772 | 0.164056 | 0.0692704 | 0.239121 | ZNF789 | | 285989 |
| ENSG00000160908 | 464.2013848 | 0.476009355 | 0.1425665 | 6.20E-05 | 0.0011826 | ZNF394 | | 84124 |
| ENSG00000196652 | 630.659526 | 0.072973637 | 0.0961199 | 0.307239 | 0.5765235 | ZKSCAN5 | | 23660 |
| ENSG00000221909 | 114.10995 | -0.013562037 | 0.1210278 | 0.8112212 | 0.9221365 | FAM200A | | 221786 |
| ENSG00000197343 | 1555.919083 | 0.050617164 | 0.0713971 | 0.4068306 | 0.6629727 | ZNF655 | | 79027 |
| ENSG00000197037 | 461.0499982 | -8.32E-05 | 0.0959099 | 0.9976759 | 0.998947 | ZSCAN25 | | 221785 |
| ENSG00000146833 | 496.8163374 | 0.010171015 | 0.093246 | 0.8828261 | 0.9527552 | TRIM4 | | 89122 |
| ENSG00000106261 | 1563.968629 | 0.38596311 | 0.0850965 | 6.25E-07 | 2.29E-05 | ZKSCAN1 | | 7586 |
| ENSG00000166529 | 211.8839563 | 0.318664651 | 0.2105615 | 0.0102132 | 0.0644519 | ZSCAN21 | | 7589 |
| ENSG00000166526 | 642.5380285 | 0.225251283 | 0.1156561 | 0.0102377 | 0.0645075 | ZNF3 | | 7551 |
| ENSG00000168090 | 2799.663295 | -0.034977189 | 0.0613994 | 0.5179575 | 0.7470898 | COPS6 | | 10980 |
| ENSG00000166508 | 9082.820102 | 0.24104216 | 0.0565584 | 4.78E-06 | 0.0001353 | MCM7 | | 4176 |
| ENSG00000207547 | 23.02794668 | 0.000501838 | 0.1326687 | 0.9894913 | NA | MIR25 | | 407014 |
| ENSG00000221838 | 554.7751011 | 0.261963347 | 0.1275947 | 0.0060123 | 0.043508 | AP4M1 | | 9179 |
| ENSG00000106290 | 1531.292271 | -0.090458163 | 0.0747503 | 0.1515807 | 0.3882075 | TAF6 | | 6878 |
| ENSG00000242798 | 19.86256223 | -0.051975496 | 0.1474707 | 0.1023807 | NA | NA | NA | |
| ENSG00000166997 | 154.2485381 | -0.116184948 | 0.1599886 | 0.1249356 | 0.346864 | CNPY4 | | 245812 |
| ENSG00000214309 | 68.16767657 | 0.118348367 | 0.1932718 | 0.0595576 | 0.2170464 | MBLAC1 | | 255374 |
| ENSG00000188186 | 972.4900717 | -0.149521781 | 0.093751 | 0.042961 | 0.1753478 | LAMTOR4 | | 389541 |
| ENSG00000146826 | 356.7355096 | -0.003618036 | 0.1011051 | 0.9558845 | 0.9825402 | TRAPPC14 | | 55262 |
| ENSG00000213420 | 22.49095905 | 0.005511302 | 0.1328873 | 0.8637913 | NA | GPC2 | | 221914 |
| ENSG00000239521 | 62.8669107 | -0.007843298 | 0.1288142 | 0.855617 | 0.9418544 | NA | NA | |
| ENSG00000222482 | 8.721153282 | 0.001828692 | 0.1351167 | 0.9310293 | NA | NA | NA | |
| ENSG00000078319 | 203.2592132 | 0.14726669 | 0.1699682 | 0.0833843 | 0.2698513 | NA | NA | |
| ENSG00000242294 | 303.2090429 | -0.320040606 | 0.1825429 | 0.0070491 | 0.0490126 | NA | NA | |
| ENSG00000121716 | 218.2798455 | -0.094952183 | 0.1333124 | 0.2043036 | 0.4594559 | PILRB | | 29990 |
| ENSG00000078487 | 54.65285388 | 0.09864389 | 0.1747627 | 0.0773459 | 0.2565722 | ZCWPW1 | | 55063 |
| ENSG00000146834 | 1892.088253 | 0.297065718 | 0.0812136 | 3.96E-05 | 0.0008125 | MEPCE | | 56257 |
| ENSG00000241357 | 9.111975054 | -0.014173536 | 0.1360559 | 0.5013211 | NA | NA | NA | |
| ENSG00000160813 | 370.7928897 | 0.131804156 | 0.1320929 | 0.1093192 | 0.3200394 | PPP1R35 | | 221908 |
| ENSG00000240211 | 8.726955313 | 0.008299874 | 0.1354296 | 0.6895715 | NA | NA | NA | |
| ENSG00000166925 | 1070.285021 | 0.085505394 | 0.0892872 | 0.2220063 | 0.4815702 | TSC22D4 | | 81628 |
| ENSG00000166924 | 38.51916214 | 0.036992349 | 0.1368669 | 0.3641793 | NA | NYAP1 | | 222950 |
| ENSG00000106351 | 265.2479863 | 0.002841546 | 0.1097917 | 0.9653701 | 0.9862257 | AGFG2 | | 3268 |
| ENSG00000077454 | 654.6662496 | -0.056337944 | 0.0925429 | 0.4092197 | 0.6643076 | LRCH4 | | 4034 |
| ENSG00000106336 | 11.01745187 | 0.008967948 | 0.1350522 | 0.7006997 | NA | FBXO24 | | 26261 |
| ENSG00000106333 | 66.93689135 | 0.024397975 | 0.1287706 | 0.6205359 | 0.818389 | PCOLCE | | 5118 |
| ENSG00000106330 | 228.9500445 | -0.01482659 | 0.1092357 | 0.822419 | 0.9271959 | MOSPD3 | | 64598 |
| ENSG00000106327 | 50.37855757 | 0.020539534 | 0.1300916 | 0.6488836 | 0.8366015 | TFR2 | | 7036 |
| ENSG00000172354 | 5135.995421 | -0.010123345 | 0.0542734 | 0.8383646 | 0.9333718 | GNB2 | | 2783 |
| ENSG00000146830 | 1490.162209 | -0.049694029 | 0.0722072 | 0.4183487 | 0.6712543 | GIGYF1 | | 64599 |
| ENSG00000172336 | 864.7748851 | 0.14127322 | 0.0962167 | 0.0584248 | 0.2143405 | POP7 | | 10248 |
| ENSG00000196411 | 1021.874556 | -0.01545963 | 0.0809006 | 0.8118689 | 0.9224879 | EPHB4 | | 2050 |
| ENSG00000146828 | 702.7434221 | -0.035520842 | 0.0905562 | 0.6029909 | 0.8084868 | SLC12A9 | | 56996 |
| ENSG00000087077 | 1645.422718 | 0.244065238 | 0.088992 | 0.0012236 | 0.0131584 | TRIP6 | | 7205 |
| ENSG00000087087 | 3724.776305 | -0.258804702 | 0.0666141 | 2.05E-05 | 0.0004646 | SRRT | | 51593 |
| ENSG00000176125 | 19.48793999 | 0.028009535 | 0.1376733 | 0.3297113 | NA | UFSP1 | | 402682 |
| ENSG00000087085 | 12.98743441 | 0.020427888 | 0.1365638 | 0.4118684 | NA | ACHE | | 43 |
| ENSG00000205277 | 20.33440746 | -0.016336686 | 0.1345491 | 0.5855032 | NA | MUC12 | | 10071 |
| ENSG00000227053 | 18.51753886 | 0.045847934 | 0.1444485 | 0.134692 | NA | MUC12-AS1 | | 102724094 |
| ENSG00000169871 | 824.3455559 | 0.028469105 | 0.0868889 | 0.6735889 | 0.8501877 | TRIM56 | | 81844 |
| ENSG00000260336 | 403.9486654 | 0.085821188 | 0.1096062 | 0.2496207 | 0.515769 | NA | NA | |
| ENSG00000106366 | 2575.725807 | 0.770630354 | 0.0769649 | 8.56E-25 | 4.00E-22 | SERPINE1 | | 5054 |
| ENSG00000106367 | 2188.304106 | -0.059833385 | 0.0664374 | 0.3045953 | 0.5735672 | AP1S1 | | 1174 |
| ENSG00000264425 | 9.357256417 | 0.001249418 | 0.1349266 | 0.9559941 | NA | MIR4653 | | 100616117 |
| ENSG00000128564 | 73.59981157 | 0.721493133 | 0.3934148 | 0.0021959 | 0.0203416 | VGF | | 7425 |
| ENSG00000106397 | 1954.129442 | 0.064925993 | 0.0693316 | 0.278723 | 0.5469114 | PLOD3 | | 8985 |

| | | | | | | | | |
|---|---|---|---|---|---|---|---|---|
| ENSG00000106400 | 1335.209065 | 0.001023973 | 0.0745006 | 0.9878005 | 0.9950412 | ZNHIT1 | | 10467 |
| ENSG00000106404 | 199.2978237 | 0.431265678 | 0.2341338 | 0.0037038 | 0.0300446 | CLDN15 | | 24146 |
| ENSG00000214253 | 572.3135479 | 0.003518447 | 0.0915742 | 0.959614 | 0.9833539 | FIS1 | | 51024 |
| ENSG00000232445 | 102.9400269 | 0.076421668 | 0.1442341 | 0.2214668 | 0.4809358 | NA | NA | |
| ENSG00000128581 | 439.6248288 | -0.025087311 | 0.098002 | 0.7147156 | 0.8710746 | IFT22 | | 64792 |
| ENSG00000160963 | 37.98802966 | -0.045032418 | 0.1399851 | 0.27796 | NA | COL26A1 | | 136227 |
| ENSG00000257923 | 1736.36685 | 0.036249048 | 0.0731065 | 0.558604 | 0.7775341 | CUX1 | | 1523 |
| ENSG00000160999 | 24.45321257 | 0.038229944 | 0.1397299 | 0.2675503 | NA | SH2B2 | | 10603 |
| ENSG00000128563 | 723.8676778 | 0.07043868 | 0.0904657 | 0.308695 | 0.5780484 | PRKRIP1 | | 79706 |
| ENSG00000160991 | 520.9676315 | 0.099138818 | 0.1105622 | 0.1927376 | 0.444232 | ORAI2 | | 80228 |
| ENSG00000160993 | 349.6194443 | 0.058860357 | 0.1072471 | 0.4112968 | 0.6661689 | ALKBH4 | | 54784 |
| ENSG00000161036 | 949.2408164 | 0.000234766 | 0.0791747 | 0.9979207 | 0.998947 | LRWD1 | | 222229 |
| ENSG00000005075 | 660.18711 | -0.051301863 | 0.0917443 | 0.459899 | 0.7041733 | POLR2J | | 5439 |
| ENSG00000168255 | 73.80864132 | 0.03380499 | 0.1295498 | 0.5189624 | 0.747841 | POLR2J3-UI | | 123258399 |
| ENSG00000222011 | 115.9799732 | 0.012653194 | 0.120465 | 0.8292494 | 0.9296029 | FAM185A | | 222234 |
| ENSG00000161040 | 13.94762059 | 0.019421971 | 0.1361094 | 0.4551621 | NA | FBXL13 | | 222235 |
| ENSG00000170632 | 931.1747055 | -0.0759852 | 0.0868305 | 0.2666767 | 0.5346062 | ARMC10 | | 83787 |
| ENSG00000161048 | 164.1146687 | -0.048288058 | 0.1238368 | 0.4473045 | 0.6949015 | NAPEPLD | | 222236 |
| ENSG00000170629 | 315.0531959 | 0.083746042 | 0.1168441 | 0.2626317 | 0.5301194 | NA | NA | |
| ENSG00000105819 | 1191.809919 | -0.105475342 | 0.0847428 | 0.1222975 | 0.3424975 | PMPCB | | 9512 |
| ENSG00000105821 | 1547.932905 | 0.030007485 | 0.0709988 | 0.6216355 | 0.818389 | DNAJC2 | | 27000 |
| ENSG00000161057 | 1924.509232 | -0.112666671 | 0.0735158 | 0.0711917 | 0.2430839 | PSMC2 | | 5701 |
| ENSG00000164815 | 501.1839867 | 0.112380051 | 0.1091578 | 0.1443908 | 0.376779 | ORC5 | | 5001 |
| ENSG00000228393 | 37.78341303 | -0.022677735 | 0.133223 | 0.5541102 | NA | NA | NA | |
| ENSG00000239569 | 26.9678859 | -0.035521585 | 0.1384554 | 0.3077806 | NA | NA | NA | |
| ENSG00000005483 | 447.7320472 | -0.358774767 | 0.1424331 | 0.0011216 | 0.0122102 | KMT2E | | 55904 |
| ENSG00000135250 | 386.0444679 | 0.058373615 | 0.1089238 | 0.4119856 | 0.6661689 | SRPK2 | | 6733 |
| ENSG00000091127 | 1209.745058 | -0.013656094 | 0.0749664 | 0.8268795 | 0.9287505 | PUS7 | | 54517 |
| ENSG00000135249 | 787.1731071 | 0.025442112 | 0.0835474 | 0.7018575 | 0.8649636 | RINT1 | | 60561 |
| ENSG00000272604 | 31.28466171 | 0.019024932 | 0.1328696 | 0.611699 | NA | EFCAB10-A: | | 127138863 |
| ENSG00000146776 | 141.5152316 | 0.017703297 | 0.1197219 | 0.7665697 | 0.8994294 | ATXN7L1 | | 222255 |
| ENSG00000128536 | 113.5776145 | -0.000738871 | 0.1208695 | 0.9899782 | 0.9955179 | CDHR3 | | 222256 |
| ENSG00000008282 | 1961.967833 | -0.206476713 | 0.0756527 | 0.0017371 | 0.0170979 | SYPL1 | | 6856 |
| ENSG00000105835 | 1393.773755 | 0.030548705 | 0.0752946 | 0.6285795 | 0.823051 | NAMPT | | 10135 |
| ENSG00000253276 | 27.69983029 | 0.04683435 | 0.1429129 | 0.2027336 | NA | CCDC71L | | 168455 |
| ENSG00000005249 | 63.65744412 | -0.176541765 | 0.2915663 | 0.0251622 | 0.1215048 | PRKAR2B | | 5577 |
| ENSG00000105856 | 324.1810917 | 0.007503401 | 0.101968 | 0.9122083 | 0.965435 | HBP1 | | 26959 |
| ENSG00000164597 | 765.1720188 | 0.018764562 | 0.0846235 | 0.7787455 | 0.9075471 | COG5 | | 10466 |
| ENSG00000105865 | 185.5550248 | 0.070086165 | 0.1272639 | 0.3126694 | 0.5819457 | DUS4L | | 11062 |
| ENSG00000075790 | 1582.234272 | -0.03856906 | 0.0722601 | 0.5290698 | 0.7552321 | BCAP29 | | 55973 |
| ENSG00000238832 | 8.353374319 | 0.014169903 | 0.1362184 | 0.4854022 | NA | LOC1249018 | | 124901856 |
| ENSG00000250474 | 14.11937184 | -0.005065856 | 0.134455 | 0.8353722 | NA | NA | NA | |
| ENSG00000091137 | 11.56252381 | -0.031103999 | 0.1397443 | 0.1941412 | NA | SLC26A4 | | 5172 |
| ENSG00000241764 | 21.59371551 | 0.029594022 | 0.1373653 | 0.3536562 | NA | CBLL1-AS1 | | 101927974 |
| ENSG00000105879 | 608.3523047 | 0.101101255 | 0.1010201 | 0.1730852 | 0.4203584 | CBLL1 | | 79872 |
| ENSG00000091140 | 1669.74137 | -0.170673953 | 0.083633 | 0.0142781 | 0.0815962 | DLD | | 1738 |
| ENSG00000091136 | 1064.805265 | 0.012658876 | 0.0827107 | 0.8484838 | 0.9375782 | LAMB1 | | 3912 |
| ENSG00000135241 | 505.2266205 | 0.049525236 | 0.10092 | 0.4829699 | 0.7214731 | PNPLA8 | | 50640 |
| ENSG00000177683 | 458.9768818 | 0.067341426 | 0.1015379 | 0.3366702 | 0.6044302 | THAP5 | | 168451 |
| ENSG00000128590 | 301.4331238 | 0.079042124 | 0.1169189 | 0.2833092 | 0.5528432 | DNAJB9 | | 4189 |
| ENSG00000184903 | 103.4954432 | 0.079942382 | 0.1485489 | 0.1934203 | 0.4454541 | IMMP2L | | 83943 |
| ENSG00000128512 | 4975.360312 | -0.064386852 | 0.0572896 | 0.2197476 | 0.479502 | DOCK4 | | 9732 |
| ENSG00000198839 | 536.3664809 | -0.035169125 | 0.0926159 | 0.6085254 | 0.8122953 | ZNF277 | | 11179 |
| ENSG00000006652 | 2524.156162 | 0.155375733 | 0.0685751 | 0.0100118 | 0.0635934 | IFRD1 | | 3475 |
| ENSG00000181016 | 49.47174053 | 0.109012551 | 0.1940244 | 0.0339722 | 0.149747 | LSMEM1 | | 286006 |
| ENSG00000146802 | 736.7236082 | -0.177155465 | 0.1086429 | 0.0296331 | 0.1371792 | TMEM168 | | 64418 |
| ENSG00000164603 | 151.2014369 | -0.341056657 | 0.2625645 | 0.011242 | 0.0691146 | BMT2 | | 154743 |
| ENSG00000214194 | 145.1601363 | -0.044496938 | 0.1252728 | 0.4681866 | 0.7103374 | SMIM30 | | 401397 |
| ENSG00000135272 | 889.1262643 | 0.054561157 | 0.0860285 | 0.4200398 | 0.672898 | MDFIC | | 29969 |
| ENSG00000135269 | 1946.857918 | 0.572130832 | 0.0848151 | 1.15E-12 | 1.49E-10 | TES | | 26136 |
| ENSG00000237813 | 25.3044322 | 0.047918681 | 0.1443544 | 0.1633075 | NA | NA | NA | |
| ENSG00000105971 | 4095.722956 | -0.120373578 | 0.060224 | 0.0258443 | 0.1239786 | CAV2 | | 858 |
| ENSG00000105974 | 9416.054131 | -0.376557176 | 0.0576467 | 7.93E-12 | 8.42E-10 | CAV1 | | 857 |
| ENSG00000105976 | 6571.542353 | -0.01577514 | 0.0540106 | 0.7508723 | 0.892513 | MET | | 4233 |
| ENSG00000198898 | 2773.340757 | -0.190371432 | 0.0686404 | 0.00171 | 0.0168985 | CAPZA2 | | 830 |
| ENSG00000227199 | 10.34329815 | 0.017633838 | 0.1365591 | 0.4209993 | NA | ST7-AS1 | | 93653 |
| ENSG00000004866 | 420.2596515 | -0.011664208 | 0.0963902 | 0.863567 | 0.9453914 | ST7 | | 7982 |
| ENSG00000128534 | 790.9606923 | 0.044055289 | 0.085049 | 0.5118293 | 0.7422319 | LSM8 | | 51691 |
| ENSG00000106025 | 165.0111066 | -0.034952032 | 0.1183702 | 0.5841252 | 0.795519 | TSPAN12 | | 23554 |
| ENSG00000071243 | 351.9630066 | -0.006784473 | 0.0998478 | 0.9188223 | 0.9683988 | ING3 | | 54556 |

| | | | | | | | | |
|---|---|---|---|---|---|---|---|---|
| ENSG00000196937 | 1499.975807 | -0.042289108 | 0.0749407 | 0.5004796 | 0.7347747 | FAM3C | | 10447 |
| ENSG00000008311 | 383.6990087 | -0.204062951 | 0.147947 | 0.0312281 | 0.1419361 | AASS | | 10157 |
| ENSG00000081803 | 522.4661711 | -0.245009461 | 0.1287887 | 0.0092383 | 0.0604552 | CADPS2 | | 93664 |
| ENSG00000128609 | 1254.951253 | -0.131170731 | 0.0858597 | 0.0592739 | 0.2163681 | NDUFA5 | | 4698 |
| ENSG00000106299 | 1327.395666 | 0.022179457 | 0.0747298 | 0.7243151 | 0.8762143 | WASL | | 8976 |
| ENSG00000272686 | 57.54639702 | -0.003940028 | 0.1274325 | 0.9302259 | 0.972984 | WASL-DT | | 116435299 |
| ENSG00000170775 | 46.72099422 | -0.029142282 | 0.132948 | 0.4997549 | 0.734688 | GPR37 | | 2861 |
| ENSG00000128513 | 438.8943326 | -7.32E-05 | 0.0965856 | 0.9978222 | 0.998947 | POT1 | | 25913 |
| ENSG00000048405 | 1052.085826 | 0.097782542 | 0.0853623 | 0.1550447 | 0.3935203 | ZNF800 | | 168850 |
| ENSG00000179562 | 898.5348618 | 0.109712213 | 0.0905967 | 0.1231165 | 0.3439664 | GCC1 | | 79571 |
| ENSG00000004059 | 1420.514382 | -0.117536235 | 0.084303 | 0.0863494 | 0.2756984 | ARF5 | | 381 |
| ENSG00000197157 | 4330.760441 | 0.080954534 | 0.0599997 | 0.1324616 | 0.358616 | SND1 | | 27044 |
| ENSG00000106344 | 2260.990089 | -0.06952495 | 0.0660923 | 0.2290231 | 0.4902363 | RBM28 | | 55131 |
| ENSG00000106348 | 2161.577103 | -0.013219531 | 0.0689017 | 0.8234696 | 0.9274851 | IMPDH1 | | 3614 |
| ENSG00000135245 | 255.1532276 | 0.095064769 | 0.1313842 | 0.2069598 | 0.4628562 | HILPDA | | 29923 |
| ENSG00000165055 | 703.3004616 | 0.081125195 | 0.0927613 | 0.2490241 | 0.515466 | METTL2B | | 55798 |
| ENSG00000273270 | 89.20997164 | 0.092686857 | 0.1581929 | 0.1445777 | 0.3769669 | NA | NA | |
| ENSG00000242588 | 287.3967469 | 0.023415578 | 0.1072327 | 0.7300888 | 0.8798274 | NA | NA | |
| ENSG00000243302 | 125.2978219 | 0.025317655 | 0.1208453 | 0.6720274 | 0.8499086 | NA | NA | |
| ENSG00000230715 | 91.43454749 | 0.018585062 | 0.1237021 | 0.7307321 | 0.8802394 | NA | NA | |
| ENSG00000243679 | 149.1136571 | -0.013940684 | 0.1166326 | 0.8204813 | 0.9260506 | NA | NA | |
| ENSG00000128595 | 6224.664488 | -0.099073391 | 0.0556243 | 0.0512088 | 0.1950344 | CALU | | 813 |
| ENSG00000244218 | 8.233299693 | -0.004733261 | 0.1353355 | 0.807248 | NA | RN7SL81P | | 106479253 |
| ENSG00000128596 | 32.46414163 | 0.036164769 | 0.1381247 | 0.3252632 | NA | CCDC136 | | 64753 |
| ENSG00000135253 | 64.28745766 | 0.017086631 | 0.1283626 | 0.7200184 | 0.8733776 | KCP | | 375616 |
| ENSG00000128524 | 1678.071923 | 0.137937446 | 0.0762867 | 0.0330168 | 0.1466249 | ATP6V1F | | 9296 |
| ENSG00000128604 | 44.42468822 | 0.075161191 | 0.1568571 | 0.1174817 | 0.3339561 | IRF5 | | 3663 |
| ENSG00000064419 | 2269.762052 | 0.004708235 | 0.0645978 | 0.9346832 | 0.9750297 | TNPO3 | | 23534 |
| ENSG00000230359 | 10.80071796 | -0.00480379 | 0.134881 | 0.8282119 | NA | TPI1P2 | | 286016 |
| ENSG00000158457 | 463.7663789 | -0.046695843 | 0.0989376 | 0.5048648 | 0.7381515 | TSPAN33 | | 340348 |
| ENSG00000128602 | 84.50233218 | -0.016133895 | 0.1253361 | 0.7547244 | 0.8942018 | SMO | | 6608 |
| ENSG00000158467 | 487.0746991 | 0.063534394 | 0.1019258 | 0.3758607 | 0.6375877 | AHCYL2 | | 23382 |
| ENSG00000128578 | 403.6054344 | 0.023904696 | 0.0993838 | 0.7290799 | 0.8790651 | STRIP2 | | 57464 |
| ENSG00000240204 | 20.25626708 | 0.006624154 | 0.1335235 | 0.823682 | NA | SMKR1 | | 100287482 |
| ENSG00000273329 | 83.39940848 | -0.00931703 | 0.1249387 | 0.8540728 | 0.9409946 | NA | NA | |
| ENSG00000106459 | 364.6216381 | -0.069563358 | 0.1126708 | 0.3371617 | 0.6048818 | NRF1 | | 4899 |
| ENSG00000186591 | 2280.585804 | 0.354199373 | 0.0760986 | 4.05E-07 | 1.57E-05 | UBE2H | | 7328 |
| ENSG00000091732 | 635.9746077 | 0.056556396 | 0.0955066 | 0.4220722 | 0.6742808 | ZC3HC1 | | 51530 |
| ENSG00000128607 | 1253.585013 | -0.039573209 | 0.0769092 | 0.5341505 | 0.7589603 | KLHDC10 | | 23008 |
| ENSG00000146842 | 956.1297278 | 0.377332007 | 0.1147415 | 0.0001004 | 0.0017459 | TMEM209 | | 84928 |
| ENSG00000240571 | 10.55595843 | 0.021674825 | 0.1372831 | 0.3381525 | NA | NA | NA | |
| ENSG00000128510 | 86.62732098 | 1.317148082 | 0.3142999 | 1.28E-06 | 4.18E-05 | CPA4 | | 51200 |
| ENSG00000106477 | 378.3377685 | 0.109761713 | 0.1229917 | 0.1620093 | 0.4039797 | CEP41 | | 95681 |
| ENSG00000158623 | 324.5232608 | -0.160732759 | 0.145639 | 0.0678311 | 0.235778 | COPG2 | | 26958 |
| ENSG00000270953 | 9.522250759 | 0.003691897 | 0.1349791 | 0.8671852 | NA | NA | NA | |
| ENSG00000233559 | 24.0493471 | 0.024033253 | 0.1356981 | 0.452537 | NA | NA | NA | |
| ENSG00000226380 | 296.4070091 | 1.057309904 | 0.1694411 | 2.41E-11 | 2.37E-09 | NA | NA | |
| ENSG00000231721 | 196.2914466 | 0.014996241 | 0.1179206 | 0.8061058 | 0.919184 | LINC-PINT | | 378805 |
| ENSG00000128585 | 7283.860923 | -0.111345895 | 0.1574959 | 0.1277633 | 0.3512363 | MKLN1 | | 4289 |
| ENSG00000236753 | 402.6167943 | 0.008415845 | 0.1005565 | 0.9008752 | 0.9611914 | MKLN1-AS | | 100506881 |
| ENSG00000273489 | 43.37826247 | -0.070763393 | 0.1569947 | 0.0864646 | 0.2759545 | NA | NA | |
| ENSG00000128567 | 110.4839737 | 1.199708388 | 0.2719037 | 4.95E-07 | 1.87E-05 | PODXL | | 5420 |
| ENSG00000106554 | 1874.189964 | 0.042643268 | 0.070883 | 0.4829788 | 0.7214731 | CHCHD3 | | 54927 |
| ENSG00000131558 | 1057.630941 | 0.077489029 | 0.0831242 | 0.2515512 | 0.5183224 | EXOC4 | | 60412 |
| ENSG00000155530 | 17.25493071 | 0.013564366 | 0.1345206 | 0.6343623 | NA | LRGUK | | 136332 |
| ENSG00000205060 | 785.3464062 | -0.079027461 | 0.0946274 | 0.26644 | 0.5345733 | SLC35B4 | | 84912 |
| ENSG00000085662 | 10319.35153 | 0.056979573 | 0.1432684 | 0.2365219 | 0.5007816 | AKR1B1 | | 231 |
| ENSG00000172331 | 269.4962089 | 0.001838608 | 0.1061293 | 0.9792395 | 0.992236 | BPGM | | 669 |
| ENSG00000122786 | 2244.071819 | 0.368263963 | 0.0832114 | 1.11E-06 | 3.67E-05 | CALD1 | | 800 |
| ENSG00000146856 | 69.30913316 | -0.01313737 | 0.1269127 | 0.7848254 | 0.9099416 | AGBL3 | | 340351 |
| ENSG00000122783 | 1197.089345 | 0.109435336 | 0.0828732 | 0.1067547 | 0.3152179 | CYREN | | 78996 |
| ENSG00000146859 | 18.02438785 | 0.031436795 | 0.1385896 | 0.2871178 | NA | TMEM140 | | 55281 |
| ENSG00000272941 | 14.38199759 | -0.000975591 | 0.1341401 | 0.9673843 | NA | NA | NA | |
| ENSG00000105875 | 477.8509501 | -0.172253826 | 0.1328565 | 0.0496574 | 0.1916246 | WDR91 | | 29062 |
| ENSG00000080802 | 554.5295914 | 0.064255216 | 0.1002391 | 0.3916805 | 0.6521123 | CNOT4 | | 4850 |
| ENSG00000155561 | 4530.739579 | -0.033389417 | 0.056507 | 0.5152837 | 0.7451617 | NUP205 | | 23165 |
| ENSG00000243317 | 617.7566317 | -0.225409048 | 0.123388 | 0.0127256 | 0.0753794 | STMP1 | | 647087 |
| ENSG00000105887 | 5703.586737 | -0.104991171 | 0.0564334 | 0.0405435 | 0.169099 | MTPN | | 136319 |
| ENSG00000236338 | 39.89319185 | -0.060555558 | 0.1487332 | 0.1515162 | NA | NA | NA | |
| ENSG00000182158 | 561.5754211 | -0.161986658 | 0.1155485 | 0.0497276 | 0.1917456 | CREB3L2 | | 64764 |

| | | | | | | | | |
|---|---|---|---|---|---|---|---|---|
| ENSG00000122779 | 762.4976638 | -0.161880108 | 0.1057225 | 0.041263 | 0.170698 | TRIM24 | | 8805 |
| ENSG00000105929 | 108.7724019 | -0.120549159 | 0.1874702 | 0.0747568 | 0.2510015 | ATP6V0A4 | | 50617 |
| ENSG00000122778 | 652.2742635 | -0.049898857 | 0.0917065 | 0.4658668 | 0.7092404 | KIAA1549 | | 57670 |
| ENSG00000146858 | 152.2082897 | -0.141759229 | 0.1917095 | 0.0739744 | 0.2491832 | ZC3HAV1L | | 92092 |
| ENSG00000105939 | 1307.301611 | -0.168399819 | 0.0978088 | 0.0278988 | 0.1312026 | ZC3HAV1 | | 56829 |
| ENSG00000105948 | 433.8127803 | -0.094979692 | 0.1110731 | 0.2085744 | 0.4650913 | IFT56 | | 79989 |
| ENSG00000157741 | 776.2197242 | -0.046358509 | 0.086403 | 0.4914463 | 0.7279463 | UBN2 | | 254048 |
| ENSG00000164898 | 18.79922878 | 0.012160782 | 0.1341265 | 0.6796986 | NA | FMC1 | | 154791 |
| ENSG00000146963 | 1111.920552 | 0.026238245 | 0.0764818 | 0.6799342 | 0.853781 | LUC7L2 | | 51631 |
| ENSG00000273391 | 19.02628862 | 0.009629922 | 0.1337094 | 0.7480646 | NA | NA | NA | |
| ENSG00000064393 | 2277.863189 | 0.057019021 | 0.066347 | 0.325981 | 0.5942836 | HIPK2 | | 28996 |
| ENSG00000059377 | 15.32622974 | 0.061456372 | 0.1549272 | 0.0287743 | NA | TBXAS1 | | 6916 |
| ENSG00000059378 | 892.216002 | -0.085095524 | 0.0865843 | 0.2142186 | 0.4719517 | PARP12 | | 64761 |
| ENSG00000006459 | 232.2653624 | 0.089292068 | 0.1286466 | 0.2298418 | 0.4909091 | KDM7A | | 80853 |
| ENSG00000260231 | 28.85515844 | 0.007754864 | 0.1321105 | 0.8253811 | NA | KDM7A-DT | | 100134229 |
| ENSG00000157800 | 1814.556815 | -0.094709004 | 0.0737038 | 0.1296493 | 0.3541705 | SLC37A3 | | 84255 |
| ENSG00000146955 | 68.25640583 | -0.146206319 | 0.2290923 | 0.0405722 | 0.1691378 | RAB19 | | 401409 |
| ENSG00000133606 | 1935.530152 | -0.175515425 | 0.080429 | 0.0098005 | 0.0628688 | MKRN1 | | 23608 |
| ENSG00000146966 | 127.0514902 | -0.142672536 | 0.1925893 | 0.0726568 | 0.2462867 | DENND2A | | 27147 |
| ENSG00000133597 | 708.0414923 | -0.005212551 | 0.0849292 | 0.9366839 | 0.9756246 | ADCK2 | | 90956 |
| ENSG00000090266 | 1584.311466 | -0.096154613 | 0.074417 | 0.1255283 | 0.3477932 | NDUFB2 | | 4708 |
| ENSG00000240889 | 27.24432024 | 0.002463543 | 0.1320231 | 0.9447619 | NA | NDUFB2-AS | | 100134713 |
| ENSG00000157764 | 1108.319706 | 0.103867519 | 0.0870631 | 0.1358637 | 0.3628767 | BRAF | | 673 |
| ENSG00000273081 | 12.63959674 | 0.026462136 | 0.1380744 | 0.2899463 | NA | NA | NA | |
| ENSG00000090263 | 668.0323844 | 0.079866716 | 0.0936405 | 0.2613879 | 0.5288883 | MRPS33 | | 51650 |
| ENSG00000261115 | 2068.809603 | -0.133516791 | 0.0818754 | 0.0484085 | 0.1885495 | TMEM178B | | 100507421 |
| ENSG00000006530 | 844.9907723 | -0.089818512 | 0.0938799 | 0.2083366 | 0.4649561 | AGK | | 55750 |
| ENSG00000244701 | 18.43551482 | 0.015384719 | 0.1345637 | 0.6030604 | NA | NA | NA | |
| ENSG00000257093 | 1076.021624 | -0.077601801 | 0.0882526 | 0.2607223 | 0.5279074 | DENND11 | | 57189 |
| ENSG00000270157 | 39.93851256 | -0.091000154 | 0.1724544 | 0.0594797 | NA | NA | NA | |
| ENSG00000106028 | 2071.502237 | -0.132018088 | 0.0749373 | 0.0374653 | 0.1603047 | SSBP1 | | 6742 |
| ENSG00000127366 | 12.3770746 | 0.02095353 | 0.1367149 | 0.396601 | NA | TAS2R5 | | 54429 |
| ENSG00000197448 | 1007.305116 | 0.01219175 | 0.0776882 | 0.8495371 | 0.9379436 | GSTK1 | | 373156 |
| ENSG00000106144 | 1524.005498 | 0.081559693 | 0.0774723 | 0.205659 | 0.4607272 | CASP2 | | 835 |
| ENSG00000239419 | 11.35444176 | -0.021039991 | 0.1369757 | 0.3672095 | NA | RN7SL535P | | 106479417 |
| ENSG00000240322 | 8.664012675 | -0.014952956 | 0.1362743 | 0.4669297 | NA | RN7SL481P | | 106481054 |
| ENSG00000232533 | 119.0789285 | 0.041433595 | 0.1258093 | 0.490869 | 0.7273003 | NA | NA | |
| ENSG00000159840 | 4786.286184 | 0.071800202 | 0.0602002 | 0.1838511 | 0.4338885 | ZYX | | 7791 |
| ENSG00000146904 | 41.62672744 | 0.021531869 | 0.1317112 | 0.6077245 | 0.8118515 | EPHA1 | | 2041 |
| ENSG00000223459 | 89.20431647 | -0.044026915 | 0.1311609 | 0.4264173 | 0.6786077 | TCAF1P1 | | 653199 |
| ENSG00000170379 | 9.801509075 | -0.001599126 | 0.1348799 | 0.9392129 | NA | TCAF2 | | 285966 |
| ENSG00000198420 | 772.7859149 | -0.146600997 | 0.1026729 | 0.0578507 | 0.2129702 | TCAF1 | | 9747 |
| ENSG00000213214 | 183.5168182 | -0.021873915 | 0.1141532 | 0.731625 | 0.8809516 | ARHGEF35 | | 445328 |
| ENSG00000244198 | 15.14641759 | 0.017822572 | 0.1356562 | 0.5040823 | NA | NA | NA | |
| ENSG00000244479 | 89.77325647 | 0.046581563 | 0.1316872 | 0.4085004 | 0.6640358 | NA | NA | |
| ENSG00000243896 | 27.3589395 | -0.016912205 | 0.133408 | 0.6222211 | NA | OR2A7 | | 401427 |
| ENSG00000244693 | 51.98797695 | 0.004866386 | 0.1283478 | 0.9144595 | 0.9662935 | NA | NA | |
| ENSG00000204959 | 592.6321851 | -0.073478427 | 0.0968528 | 0.3024422 | 0.5718399 | NA | NA | |
| ENSG00000228960 | 22.31695756 | -0.005697791 | 0.1329732 | 0.8537912 | NA | NA | NA | |
| ENSG00000050327 | 756.4687142 | 0.01908473 | 0.0864141 | 0.7770751 | 0.9067041 | ARHGEF5 | | 7984 |
| ENSG00000196511 | 42.6227312 | 0.01575196 | 0.1304233 | 0.7159321 | 0.8713976 | TPK1 | | 27010 |
| ENSG00000273314 | 33.14530518 | 0.016569123 | 0.1321687 | 0.6647237 | NA | NA | NA | |
| ENSG00000055130 | 1794.979424 | -0.184264156 | 0.0772874 | 0.0054903 | 0.0407186 | CUL1 | | 8454 |
| ENSG00000106462 | 2410.10548 | 0.197410206 | 0.0716984 | 0.0017753 | 0.0173101 | EZH2 | | 2146 |
| ENSG00000155660 | 6681.658967 | -0.15689474 | 0.0651742 | 0.0069673 | 0.0485591 | PDIA4 | | 9601 |
| ENSG00000197362 | 193.9209198 | -0.021833662 | 0.1135914 | 0.7344621 | 0.8828208 | ZNF786 | | 136051 |
| ENSG00000204947 | 71.76551102 | 0.573970353 | 0.447393 | 0.0055282 | 0.0408959 | ZNF425 | | 155054 |
| ENSG00000197024 | 579.587336 | 0.234560634 | 0.1230507 | 0.0101613 | 0.0642979 | ZNF398 | | 57541 |
| ENSG00000170265 | 1189.096901 | 0.118501148 | 0.0870891 | 0.0899769 | 0.2831836 | ZNF282 | | 8427 |
| ENSG00000170260 | 466.3900047 | 0.23841753 | 0.1466623 | 0.0160271 | 0.0883902 | ZNF212 | | 7988 |
| ENSG00000204946 | 520.6308376 | 0.163296045 | 0.1196084 | 0.0516223 | 0.1962892 | ZNF783 | | 100289678 |
| ENSG00000244560 | 41.83562368 | -0.009836831 | 0.1309062 | 0.7991519 | 0.9162772 | NA | NA | |
| ENSG00000196453 | 504.1650622 | 0.27547406 | 0.1325218 | 0.0051454 | 0.0388769 | ZNF777 | | 27153 |
| ENSG00000181220 | 764.6315359 | 0.060953104 | 0.0897786 | 0.378604 | 0.6400105 | ZNF746 | | 155061 |
| ENSG00000133624 | 276.3613214 | 0.033857724 | 0.1127775 | 0.6136999 | 0.8152654 | NA | NA | |
| ENSG00000133619 | 303.4504861 | 0.042621107 | 0.1068498 | 0.533373 | 0.7583815 | KRBA1 | | 84626 |
| ENSG00000106479 | 30.89694145 | 0.038228051 | 0.1385625 | 0.3111537 | NA | ZNF862 | | 643641 |
| ENSG00000204934 | 27.73616971 | 0.086848613 | 0.1739084 | 0.0267169 | NA | ATP6V0E2-/ | | 401431 |
| ENSG00000171130 | 1127.450262 | -0.027244224 | 0.0799865 | 0.6741263 | 0.8501975 | ATP6V0E2 | | 155066 |
| ENSG00000106526 | 43.27434672 | 0.009783851 | 0.1297989 | 0.8168576 | 0.9252377 | ACTR3C | | 653857 |

| | | | | | | | | |
|---|---|---|---|---|---|---|---|---|
| ENSG00000127399 | 118.6658499 | 0.045271147 | 0.1277633 | 0.4493346 | 0.6962034 | LRRC61 | | 65999 |
| ENSG00000188707 | 118.8470844 | 0.055608964 | 0.1314995 | 0.3637098 | 0.6265951 | NA | NA | |
| ENSG00000214022 | 1992.852944 | 0.075893192 | 0.0697202 | 0.2070023 | 0.4628626 | REPIN1 | | 29803 |
| ENSG00000196456 | 298.0929755 | -0.012277484 | 0.1039875 | 0.8550151 | 0.9414999 | ZNF775 | | 285971 |
| ENSG00000164867 | 12.64869026 | -0.006190718 | 0.1346169 | 0.7962011 | NA | NOS3 | | 4846 |
| ENSG00000197150 | 616.1300206 | -0.094778645 | 0.1024568 | 0.2002631 | 0.4540225 | ABCB8 | | 11194 |
| ENSG00000213199 | 18.18261666 | 0.040991893 | 0.142321 | 0.1693245 | NA | ASIC3 | | 9311 |
| ENSG00000164885 | 551.5505902 | -0.005357054 | 0.0926816 | 0.924844 | 0.9709924 | CDK5 | | 1020 |
| ENSG00000164889 | 5565.845524 | 0.006653959 | 0.0568366 | 0.8978564 | 0.9594787 | SLC4A2 | | 6522 |
| ENSG00000244151 | 24.11604874 | 0.015900208 | 0.1339996 | 0.6196379 | NA | NA | NA | |
| ENSG00000164896 | 1277.536241 | 0.019141369 | 0.0738682 | 0.7591088 | 0.8964867 | FASTK | | 10922 |
| ENSG00000164897 | 1853.044775 | 0.109207326 | 0.0734098 | 0.0809605 | 0.2642336 | TMUB1 | | 83590 |
| ENSG00000133612 | 1689.581926 | -0.022003567 | 0.0719212 | 0.7182033 | 0.8722654 | AGAP3 | | 116988 |
| ENSG00000033050 | 3991.707499 | 0.06887769 | 0.0597182 | 0.1996449 | 0.4532658 | ABCF2 | | 10061 |
| ENSG00000033100 | 1018.776797 | -0.049472962 | 0.0799694 | 0.4489027 | 0.6959957 | CHPF2 | | 54480 |
| ENSG00000082014 | 107.8112742 | -0.007346054 | 0.1215962 | 0.8937555 | 0.9571796 | SMARCD3 | | 6604 |
| ENSG00000272661 | 13.06070298 | 0.026427009 | 0.1380948 | 0.2886311 | NA | NA | NA | |
| ENSG00000013374 | 1550.13143 | 0.005688496 | 0.0731057 | 0.9290005 | 0.9726302 | NUB1 | | 51667 |
| ENSG00000106615 | 2488.397078 | -0.002917979 | 0.0626332 | 0.9573308 | 0.9826522 | RHEB | | 6009 |
| ENSG00000106617 | 2754.867443 | -0.091674103 | 0.0683207 | 0.1220082 | 0.342196 | PRKAG2 | | 51422 |
| ENSG00000239911 | 9.933386355 | 0.007744572 | 0.1353288 | 0.7148146 | NA | NA | NA | |
| ENSG00000178234 | 1148.820468 | -0.081988998 | 0.080833 | 0.2141795 | 0.4719517 | GALNT11 | | 63917 |
| ENSG00000055609 | 1839.136778 | -0.007314919 | 0.0729504 | 0.9048559 | 0.9622744 | KMT2C | | 58508 |
| ENSG00000261455 | 62.55144119 | -0.005973265 | 0.1265246 | 0.8995815 | 0.9606007 | LINC01003 | | 100128822 |
| ENSG00000196584 | 1077.841533 | 0.09668636 | 0.0848054 | 0.1584215 | 0.3986051 | XRCC2 | | 7516 |
| ENSG00000133627 | 178.0675805 | 0.063205636 | 0.1255166 | 0.3528873 | 0.6185235 | ACTR3B | | 57180 |
| ENSG00000214106 | 87.57390473 | -0.110491748 | 0.1739026 | 0.0991197 | 0.3004976 | NA | NA | |
| ENSG00000157212 | 1346.8915 | -0.063661946 | 0.0808093 | 0.3336562 | 0.601084 | PAXIP1 | | 22976 |
| ENSG00000272760 | 29.69842079 | -0.042142067 | 0.1405195 | 0.2539172 | NA | NA | NA | |
| ENSG00000273344 | 195.8185527 | 0.047763641 | 0.1202886 | 0.470683 | 0.7115591 | PAXIP1-DT | | 202781 |
| ENSG00000217825 | 29.70270039 | -0.012820409 | 0.136387 | 0.4681069 | NA | NA | NA | |
| ENSG00000273117 | 41.25286028 | -0.037926095 | 0.1365334 | 0.368808 | 0.6315515 | NA | NA | |
| ENSG00000186480 | 773.848103 | -0.835202078 | 0.1257259 | 1.73E-12 | 2.08E-10 | INSIG1 | | 3638 |
| ENSG00000216895 | 79.10752593 | 0.095844296 | 0.1634431 | 0.1236711 | 0.3446914 | RBM33-DT | | 100506302 |
| ENSG00000184863 | 1902.413392 | 0.006869455 | 0.0677447 | 0.9084518 | 0.963904 | RBM33 | | 155435 |
| ENSG00000164690 | 859.8889957 | -0.35987668 | 0.1082681 | 9.60E-05 | 0.001683 | SHH | | 6469 |
| ENSG00000204876 | 385.4982178 | -0.007422681 | 0.1232504 | 0.8878002 | 0.9545517 | LOC389602 | | 389602 |
| ENSG00000244291 | 25.78438283 | 0.02668512 | 0.1356775 | 0.4402229 | NA | NA | NA | |
| ENSG00000105982 | 23.94048416 | 0.038800319 | 0.1402247 | 0.2495662 | NA | RNF32 | | 140545 |
| ENSG00000105983 | 1693.990585 | -0.030610491 | 0.0711759 | 0.6136465 | 0.8152654 | LMBR1 | | 64327 |
| ENSG00000146909 | 1581.503044 | 0.023087747 | 0.0705068 | 0.7030216 | 0.8651804 | NOM1 | | 64434 |
| ENSG00000130675 | 506.4156268 | 0.078160811 | 0.1017333 | 0.2838921 | 0.5531631 | MNX1 | | 3110 |
| ENSG00000243479 | 16.43069544 | 0.037633864 | 0.1411804 | 0.1887998 | NA | MNX1-AS1 | | 645249 |
| ENSG00000009335 | 4286.871453 | -0.078158141 | 0.0588945 | 0.1401472 | 0.3695639 | UBE3C | | 9690 |
| ENSG00000105993 | 8606.701216 | -0.117378198 | 0.0579527 | 0.0254014 | 0.1222555 | DNAJB6 | | 10049 |
| ENSG00000155093 | 50.97712335 | 0.047623955 | 0.1390118 | 0.3036162 | 0.5728743 | PTPRN2 | | 5799 |
| ENSG00000146918 | 4891.33733 | -0.114642152 | 0.0585995 | 0.0301619 | 0.1388732 | NCAPG2 | | 54892 |
| ENSG00000117868 | 1976.139385 | -0.110348793 | 0.0727769 | 0.0757326 | 0.2530413 | ESYT2 | | 57488 |
| ENSG00000126870 | 441.9640471 | 0.046219831 | 0.099268 | 0.5107276 | 0.7419238 | DYNC2I1 | | 55112 |
| ENSG00000182378 | 374.1160259 | 0.061154861 | 0.1084219 | 0.3978679 | 0.6570755 | PLCXD1 | | 55344 |
| ENSG00000178605 | 299.0884453 | -0.085643247 | 0.1214028 | 0.2491146 | 0.515466 | GTPBP6 | | 8225 |
| ENSG00000167393 | 105.5802104 | 0.117684747 | 0.1747296 | 0.0998397 | 0.3015845 | PPP2R3B | | 28227 |
| ENSG00000169100 | 3066.598934 | 0.017319277 | 0.0598022 | 0.7458428 | 0.8893165 | SLC25A6 | | 293 |
| ENSG00000236871 | 24.3953848 | -0.047218223 | 0.1441853 | 0.1615241 | NA | LINC00106 | | 751580 |
| ENSG00000236017 | 30.99983704 | -0.007513546 | 0.1322701 | 0.8245623 | NA | NA | NA | |
| ENSG00000169093 | 334.6831951 | -0.140488488 | 0.1404203 | 0.0958434 | 0.294153 | ASMTL | | 8623 |
| ENSG00000197976 | 656.1985399 | 0.042171246 | 0.0896497 | 0.5390556 | 0.7626828 | AKAP17A | | 8227 |
| ENSG00000223511 | 24.07050357 | -0.005445016 | 0.1329751 | 0.8576408 | NA | NA | NA | |
| ENSG00000169084 | 100.245254 | -0.06583033 | 0.1386048 | 0.2755986 | 0.5439328 | DHRSX | | 207063 |
| ENSG00000214717 | 396.8682758 | -0.095811632 | 0.1167883 | 0.2085689 | 0.4650913 | ZBED1 | | 9189 |
| ENSG00000223773 | 31.93932441 | -0.015790342 | 0.1324576 | 0.666123 | NA | NA | NA | |
| ENSG00000002586 | 866.6948213 | -0.103041171 | 0.0978505 | 0.1599628 | 0.4009618 | CD99 | | 4267 |
| ENSG00000006756 | 101.398264 | 0.05267226 | 0.131842 | 0.37383 | 0.6352333 | ARSD | | 414 |
| ENSG00000183943 | 374.7634235 | -0.168952368 | 0.1429177 | 0.0576955 | 0.2125996 | PRKX | | 5613 |
| ENSG00000205664 | 98.63036928 | 0.084834514 | 0.1519197 | 0.1738195 | 0.4212418 | NA | NA | |
| ENSG00000234449 | 68.77193094 | -0.003859438 | 0.1260001 | 0.9355946 | 0.9752526 | NA | NA | |
| ENSG00000130021 | 204.3213882 | -4.22E-05 | 0.111494 | 0.9982754 | 0.9989595 | PUDP | | 8226 |
| ENSG00000101846 | 96.57115308 | -0.028408786 | 0.1260199 | 0.6033361 | 0.8087639 | STS | | 412 |
| ENSG00000101849 | 340.2488146 | -0.097195299 | 0.122843 | 0.203311 | 0.4581165 | TBL1X | | 6907 |
| ENSG00000047644 | 19.14609433 | 0.00061397 | 0.1331669 | 0.9857812 | NA | WWC3 | | 55841 |

| | | | | | | | | |
|---|---|---|---|---|---|---|---|---|
| ENSG00000004961 | 444.3913904 | -0.011225592 | 0.0962354 | 0.8683533 | 0.9471139 | HCCS | | 3052 |
| ENSG00000005302 | 248.1726175 | -0.289603071 | 0.1935156 | 0.0128617 | 0.075954 | MSL3 | | 10943 |
| ENSG00000101911 | 766.2607252 | 0.010485107 | 0.0842583 | 0.875352 | 0.9494835 | PRPS2 | | 5634 |
| ENSG00000205542 | 9214.38206 | -0.29727834 | 0.1392727 | 0.0038787 | 0.0311265 | TMSB4X | | 7114 |
| ENSG00000197582 | 291.7211692 | 0.103999753 | 0.1275378 | 0.1806618 | 0.4299157 | NA | NA | |
| ENSG00000176896 | 11.40915077 | 0.019649692 | 0.1368982 | 0.3755011 | NA | TCEANC | | 170082 |
| ENSG00000123595 | 373.7397267 | 0.364659295 | 0.1600582 | 0.0019753 | 0.0186828 | RAB9A | | 9367 |
| ENSG00000196459 | 133.7975805 | -0.007819789 | 0.1177501 | 0.8960701 | 0.9584283 | TRAPPC2 | | 6399 |
| ENSG00000046651 | 230.379078 | 0.069526561 | 0.1220661 | 0.3285477 | 0.5967766 | OFD1 | | 8481 |
| ENSG00000046647 | 74.71516093 | 0.045654912 | 0.1336665 | 0.3911976 | 0.6514814 | GEMIN8 | | 54960 |
| ENSG00000231066 | 13.72498104 | -0.000442427 | 0.1341685 | 0.9838651 | NA | NA | NA | |
| ENSG00000181544 | 195.0098829 | 0.069219214 | 0.1268812 | 0.3176595 | 0.5862822 | FANCB | | 2187 |
| ENSG00000130150 | 194.1408181 | 0.025155096 | 0.1134292 | 0.7006379 | 0.86431 | MOSPD2 | | 158747 |
| ENSG00000165195 | 242.4522465 | 0.05928002 | 0.1164947 | 0.3988397 | 0.6577504 | PIGA | | 5277 |
| ENSG00000087842 | 93.44603143 | -0.541189618 | 0.3800723 | 0.0049757 | 0.0378394 | PIR | | 8544 |
| ENSG00000186312 | 120.5338029 | -0.021729733 | 0.1214553 | 0.7080412 | 0.8678177 | NA | NA | |
| ENSG00000169239 | 72.19210346 | 0.000583429 | 0.1271556 | 0.9922669 | 0.9969601 | CA5B | | 11238 |
| ENSG00000169249 | 127.4320796 | 0.041836749 | 0.124258 | 0.4974498 | 0.7324734 | ZRSR2 | | 8233 |
| ENSG00000182287 | 458.8329305 | -0.015224577 | 0.0945133 | 0.822703 | 0.9272789 | AP1S2 | | 8905 |
| ENSG00000047230 | 160.6640303 | -0.106509214 | 0.1547346 | 0.144883 | 0.377342 | CTPS2 | | 56474 |
| ENSG00000169895 | 850.3080447 | -0.047403706 | 0.0836143 | 0.4732824 | 0.7137119 | SYAP1 | | 94056 |
| ENSG00000086712 | 706.0421282 | 0.049635735 | 0.0899067 | 0.4711781 | 0.7119172 | TXLNG | | 55787 |
| ENSG00000102054 | 2376.834249 | -0.233695688 | 0.0717075 | 0.0002587 | 0.0038098 | RBBP7 | | 5931 |
| ENSG00000188158 | 174.6969293 | -0.011754186 | 0.113554 | 0.8521004 | 0.9396765 | NHS | | 4810 |
| ENSG00000047634 | 270.9198759 | 0.060775833 | 0.1157466 | 0.3879766 | 0.6484257 | SCML1 | | 6322 |
| ENSG00000102098 | 268.4935222 | 0.02161205 | 0.106321 | 0.7509304 | 0.892513 | SCML2 | | 10389 |
| ENSG00000008086 | 44.30468272 | -0.025972499 | 0.1326358 | 0.5358066 | 0.7601113 | CDKL5 | | 6792 |
| ENSG00000044446 | 367.7722157 | -0.099578221 | 0.1174346 | 0.1942965 | 0.446328 | PHKA2 | | 5256 |
| ENSG00000131828 | 1328.009778 | -0.211051069 | 0.0920968 | 0.0053486 | 0.0399537 | PDHA1 | | 5160 |
| ENSG00000180815 | 113.3473175 | 0.062687539 | 0.1342175 | 0.315564 | 0.5843537 | MAP3K15 | | 389840 |
| ENSG00000147010 | 2281.956142 | 0.13389025 | 0.0859139 | 0.0548789 | 0.2053959 | SH3KBP1 | | 30011 |
| ENSG00000173681 | 122.2707164 | -0.014964045 | 0.1199179 | 0.7974749 | 0.9159592 | BCLAF3 | | 256643 |
| ENSG00000173674 | 1742.760688 | -0.160225029 | 0.0793513 | 0.0166642 | 0.0906633 | EIF1AX | | 1964 |
| ENSG00000177189 | 726.0515387 | -0.124756581 | 0.0990456 | 0.0950725 | 0.2927871 | RPS6KA3 | | 6197 |
| ENSG00000012174 | 195.0377164 | -0.138242734 | 0.1659517 | 0.0939456 | 0.2907732 | MBTPS2 | | 51360 |
| ENSG00000230797 | 22.39096641 | -0.027009544 | 0.136381 | 0.40344 | NA | YY2 | | 404281 |
| ENSG00000102172 | 2118.86972 | -0.221163955 | 0.0829571 | 0.0018274 | 0.0176997 | SMS | | 6611 |
| ENSG00000102174 | 8.598647062 | 0.008392045 | 0.1354949 | 0.6816105 | NA | PHEX | | 5251 |
| ENSG00000174028 | 26.63222341 | -0.012622937 | 0.1327826 | 0.711847 | NA | NA | NA | |
| ENSG00000123131 | 1082.645971 | 0.026094957 | 0.0767984 | 0.682292 | 0.8543094 | PRDX4 | | 10549 |
| ENSG00000123130 | 1006.242766 | -0.11184084 | 0.0880555 | 0.1100098 | 0.3212914 | ACOT9 | | 23597 |
| ENSG00000130066 | 1304.744195 | 1.950376498 | 0.2015162 | 1.92E-23 | 8.00E-21 | SAT1 | | 6303 |
| ENSG00000184831 | 153.2705486 | -0.082407849 | 0.1406977 | 0.2247542 | 0.4852777 | APOO | | 79135 |
| ENSG00000238103 | 48.36947338 | -0.056524497 | 0.1434484 | 0.2292971 | 0.4903399 | RPL9P7 | | 6126 |
| ENSG00000174010 | 327.9042896 | 0.099823166 | 0.1259725 | 0.1939464 | 0.4459033 | KLHL15 | | 80311 |
| ENSG00000130741 | 2591.28807 | -0.126938123 | 0.070955 | 0.0383272 | 0.1630966 | EIF2S3 | | 1968 |
| ENSG00000005889 | 667.8362138 | -0.109630652 | 0.1002385 | 0.1406129 | 0.3701299 | ZFX | | 7543 |
| ENSG00000067992 | 98.56740463 | -0.036903155 | 0.1277137 | 0.5093972 | 0.7410048 | PDK3 | | 5165 |
| ENSG00000260822 | 75.22609061 | 0.004089063 | 0.1253069 | 0.9367031 | 0.9756246 | NA | NA | |
| ENSG00000101868 | 764.9255581 | -0.054091904 | 0.0873378 | 0.4211541 | 0.6736274 | POLA1 | | 5422 |
| ENSG00000232472 | 18.97478473 | -0.028181713 | 0.1374926 | 0.3408162 | NA | EEF1B2P3 | | 644820 |
| ENSG00000228933 | 31.40186188 | -0.012182659 | 0.1320151 | 0.7371112 | NA | NA | NA | |
| ENSG00000198814 | 419.7516742 | -0.319913555 | 0.1439813 | 0.002792 | 0.0243933 | GK | | 2710 |
| ENSG00000157625 | 1219.518147 | 0.011253333 | 0.0749296 | 0.8583357 | 0.9430101 | TAB3 | | 257397 |
| ENSG00000198947 | 913.083652 | 0.423865311 | 0.1052516 | 5.20E-06 | 0.0001446 | DMD | | 1756 |
| ENSG00000147027 | 211.9421705 | 0.074002196 | 0.1291603 | 0.2897162 | 0.5598305 | TMEM47 | | 83604 |
| ENSG00000130962 | 675.360433 | -0.099894163 | 0.0975254 | 0.1715097 | 0.4177334 | PRRG1 | | 5638 |
| ENSG00000147036 | 14.88293196 | 0.026845212 | 0.1377743 | 0.3179895 | NA | LANCL3 | | 347404 |
| ENSG00000047597 | 69.63343709 | 0.059839019 | 0.1427573 | 0.2444744 | 0.5100385 | XK | | 7504 |
| ENSG00000165168 | 8.448296585 | -0.00666504 | 0.1354094 | 0.7357457 | NA | CYBB | | 1536 |
| ENSG00000165169 | 1100.149017 | -0.076716588 | 0.0847935 | 0.2581392 | 0.5249519 | DYNLT3 | | 6990 |
| ENSG00000101955 | 143.0494839 | -0.084560196 | 0.1404799 | 0.2204401 | 0.4801345 | SRPX | | 8406 |
| ENSG00000156313 | 281.3301077 | -0.05410926 | 0.1121611 | 0.4379663 | 0.6880813 | RPGR | | 6103 |
| ENSG00000165175 | 966.9583073 | 0.356515531 | 0.1084655 | 0.0001105 | 0.0018962 | MID1IP1 | | 58526 |
| ENSG00000228232 | 14.22193676 | -0.005098185 | 0.1341544 | 0.8436883 | NA | NA | NA | |
| ENSG00000183337 | 234.0111249 | 0.472553328 | 0.1974501 | 0.0010282 | 0.0113521 | BCOR | | 54880 |
| ENSG00000182220 | 3145.022336 | -0.076985411 | 0.0615549 | 0.1604081 | 0.4016563 | ATP6AP2 | | 10159 |
| ENSG00000185753 | 483.2357778 | 0.010351735 | 0.0936493 | 0.8808071 | 0.9518649 | CXorf38 | | 159013 |
| ENSG00000180182 | 2622.407277 | -0.15669864 | 0.0680426 | 0.0088829 | 0.0586555 | MED14 | | 9282 |
| ENSG00000124486 | 5213.706977 | -0.177946955 | 0.0588199 | 0.00091 | 0.0102697 | USP9X | | 8239 |

| | | | | | | | | |
|---|---|---|---|---|---|---|---|---|
| ENSG00000215301 | 19727.72405 | -0.25299927 | 0.0557589 | 1.08E-06 | 3.59E-05 | DDX3X | | 1654 |
| ENSG00000147044 | 2139.010165 | -0.110057792 | 0.0721617 | 0.0746256 | 0.2507772 | CASK | | 8573 |
| ENSG00000189221 | 117.1292304 | -0.040369661 | 0.1270863 | 0.487391 | 0.7246964 | MAOA | | 4128 |
| ENSG00000069509 | 466.2140569 | 0.049808012 | 0.0981235 | 0.4796953 | 0.7187431 | FUNDC1 | | 139341 |
| ENSG00000147050 | 471.2698115 | 0.136965128 | 0.1188756 | 0.0917714 | 0.2868223 | KDM6A | | 7403 |
| ENSG00000270069 | 211.3148374 | 0.571214495 | 0.2061278 | 0.0003057 | 0.0043962 | NA | NA | |
| ENSG00000207870 | 15.26735211 | 0.02484733 | 0.1372005 | 0.3564832 | NA | MIR221 | | 407006 |
| ENSG00000231566 | 11.71078198 | -0.016858228 | 0.1360341 | 0.477091 | NA | NA | NA | |
| ENSG00000236751 | 88.97049354 | 0.109230863 | 0.1748659 | 0.0963429 | 0.2949887 | NA | NA | |
| ENSG00000147121 | 431.6417892 | 0.204342969 | 0.1354844 | 0.0266513 | 0.1268595 | KRBOX4 | | 55634 |
| ENSG00000251192 | 259.6274008 | 0.308235912 | 0.1992716 | 0.0104164 | 0.0652758 | ZNF674 | | 641339 |
| ENSG00000230844 | 224.2798991 | 0.048203757 | 0.1164036 | 0.4789329 | 0.7180127 | ZNF674-AS1 | | 401588 |
| ENSG00000147119 | 992.2268936 | 0.118755326 | 0.0881006 | 0.0915605 | 0.2865038 | CHST7 | | 56548 |
| ENSG00000065923 | 412.5383484 | 0.150940361 | 0.1286742 | 0.0743238 | 0.250052 | SLC9A7 | | 84679 |
| ENSG00000213997 | 18.70740802 | 0.036026752 | 0.14036 | 0.2205624 | NA | NA | NA | |
| ENSG00000102218 | 888.4579352 | -0.069447048 | 0.0855621 | 0.3055019 | 0.574556 | RP2 | | 6102 |
| ENSG00000102221 | 893.394117 | 0.180694738 | 0.1009647 | 0.0216256 | 0.1093783 | JADE3 | | 9767 |
| ENSG00000147123 | 1884.215423 | -0.030772443 | 0.0692699 | 0.605291 | 0.8103616 | NDUFB11 | | 54539 |
| ENSG00000182872 | 2121.863949 | -0.13937431 | 0.0774555 | 0.0322054 | 0.1447447 | RBM10 | | 8241 |
| ENSG00000130985 | 16777.68818 | -0.05459833 | 0.0516426 | 0.2522246 | 0.5187947 | UBA1 | | 7317 |
| ENSG00000224975 | 69.09709355 | -0.051698684 | 0.1381183 | 0.3108121 | 0.5809131 | INE1 | | 8552 |
| ENSG00000102225 | 4330.780983 | -0.145398042 | 0.0608639 | 0.0079046 | 0.0536514 | CDK16 | | 5127 |
| ENSG00000102226 | 2546.591718 | 0.0882906 | 0.0682132 | 0.1385735 | 0.3673017 | USP11 | | 8237 |
| ENSG00000147124 | 402.4578513 | 0.040702872 | 0.099964 | 0.5594913 | 0.7783039 | ZNF41 | | 7592 |
| ENSG00000196741 | 82.12466701 | 0.011935937 | 0.1250723 | 0.8185196 | 0.9254567 | NA | NA | |
| ENSG00000078061 | 1101.654435 | -0.111985908 | 0.0856263 | 0.1038454 | 0.3093645 | ARAF | | 369 |
| ENSG00000102265 | 2339.720558 | 0.052169279 | 0.0682051 | 0.3779871 | 0.6395077 | TIMP1 | | 7076 |
| ENSG00000126759 | 13.52564584 | 0.009062718 | 0.1348806 | 0.7091661 | NA | CFP | | 5199 |
| ENSG00000126767 | 1720.780754 | -0.081790262 | 0.0736038 | 0.1893501 | 0.4403341 | ELK1 | | 2002 |
| ENSG00000126756 | 991.2824472 | -0.05966982 | 0.0814022 | 0.3632358 | 0.6262668 | UXT | | 8409 |
| ENSG00000267064 | 24.73307047 | 0.011444897 | 0.132964 | 0.7325859 | NA | UXT-AS1 | | 100133957 |
| ENSG00000197779 | 356.0907399 | 0.043463778 | 0.1045189 | 0.5346787 | 0.7591566 | ZNF81 | | 347344 |
| ENSG00000147118 | 411.7227982 | 0.354602583 | 0.1510372 | 0.0017542 | 0.0171763 | ZNF182 | | 7569 |
| ENSG00000068438 | 1462.992087 | -0.094268366 | 0.0765229 | 0.1416026 | 0.3718661 | FTSJ1 | | 24140 |
| ENSG00000102312 | 230.8488606 | 0.092536307 | 0.1310975 | 0.2156056 | 0.4737333 | PORCN | | 64840 |
| ENSG00000147155 | 1556.366894 | -0.072402257 | 0.0771713 | 0.2592821 | 0.5259027 | EBP | | 10682 |
| ENSG00000068354 | 930.1288779 | -0.066490413 | 0.083302 | 0.3172257 | 0.5857592 | TBC1D25 | | 4943 |
| ENSG00000204620 | 12.54257354 | 0.023355152 | 0.1373653 | 0.3368524 | NA | NA | NA | |
| ENSG00000102317 | 6318.433988 | -0.233829446 | 0.0761221 | 0.0004785 | 0.0061972 | RBM3 | | 5935 |
| ENSG00000101940 | 749.6187144 | 0.005245197 | 0.0844073 | 0.9379033 | 0.9761783 | WDR13 | | 64743 |
| ENSG00000015285 | 8.984543933 | 0.01493396 | 0.1362255 | 0.4775797 | NA | WAS | | 7454 |
| ENSG00000101945 | 1270.86536 | 0.05871214 | 0.0756593 | 0.3542448 | 0.6187516 | SUV39H1 | | 6839 |
| ENSG00000094631 | 1066.934312 | -0.330070784 | 0.0971525 | 8.51E-05 | 0.0015361 | HDAC6 | | 10013 |
| ENSG00000102109 | 34.90683313 | 0.009109523 | 0.1314054 | 0.8091924 | NA | PCSK1N | | 27344 |
| ENSG00000126768 | 1345.166502 | -0.023946985 | 0.0749583 | 0.7020579 | 0.8649636 | TIMM17B | | 10245 |
| ENSG00000102103 | 3403.491491 | -0.039986083 | 0.0591956 | 0.4530397 | 0.6987417 | PQBP1 | | 10084 |
| ENSG00000102100 | 950.4991 | 0.345475305 | 0.1024186 | 8.66E-05 | 0.0015538 | SLC35A2 | | 7355 |
| ENSG00000102096 | 468.6908956 | -0.08096457 | 0.1049245 | 0.2703616 | 0.5381601 | PIM2 | | 11040 |
| ENSG00000068308 | 2172.287518 | 0.075223556 | 0.0707299 | 0.2154425 | 0.473487 | OTUD5 | | 55593 |
| ENSG00000102057 | 44.22187035 | 0.02735717 | 0.1331221 | 0.5210896 | 0.749153 | KCND1 | | 3750 |
| ENSG00000068400 | 1838.784026 | -0.057342051 | 0.0685971 | 0.3333768 | 0.6008601 | GRIPAP1 | | 56850 |
| ENSG00000068323 | 2967.649667 | 0.126524788 | 0.0665675 | 0.0306369 | 0.1401763 | TFE3 | | 7030 |
| ENSG00000147144 | 137.5535476 | -0.04246221 | 0.1233612 | 0.4954747 | 0.7308549 | CCDC120 | | 90060 |
| ENSG00000243279 | 230.6716614 | 0.076672651 | 0.1240625 | 0.2887427 | 0.5586888 | PRAF2 | | 11230 |
| ENSG00000196998 | 637.5432635 | 0.342182558 | 0.1195691 | 0.0004644 | 0.0060472 | WDR45 | | 11152 |
| ENSG00000068394 | 1412.243116 | 0.065664076 | 0.0742943 | 0.294936 | 0.5650592 | GPKOW | | 27238 |
| ENSG00000017621 | 40.84592655 | 0.000957852 | 0.1296401 | 0.9848188 | NA | NA | NA | |
| ENSG00000102007 | 4651.922809 | -0.030823047 | 0.0619482 | 0.5724012 | 0.786884 | PLP2 | | 5355 |
| ENSG00000012211 | 530.5235938 | 0.060539341 | 0.0966779 | 0.4030722 | 0.6602839 | PRICKLE3 | | 4007 |
| ENSG00000102003 | 299.3250822 | -0.094849155 | 0.1247619 | 0.2107802 | 0.4674621 | SYP | | 6855 |
| ENSG00000101997 | 824.2582416 | 0.094842563 | 0.0930776 | 0.1844175 | 0.4347246 | CCDC22 | | 28952 |
| ENSG00000049769 | 29.82101458 | 0.038922038 | 0.1391703 | 0.2915322 | NA | PPP1R3F | | 89801 |
| ENSG00000270012 | 27.87488165 | 0.003332395 | 0.131783 | 0.9263709 | NA | NA | NA | |
| ENSG00000234390 | 42.8012566 | 0.004833453 | 0.1296408 | 0.9090431 | 0.9640923 | USP27X-DT | | 158572 |
| ENSG00000242013 | 114.4571079 | -0.087847757 | 0.1482851 | 0.1873774 | 0.4383122 | NA | NA | |
| ENSG00000171365 | 1126.662393 | 0.158163498 | 0.0905596 | 0.0299589 | 0.1382656 | CLCN5 | | 1184 |
| ENSG00000147082 | 10.02450391 | 0.004524327 | 0.1350548 | 0.8346221 | NA | CCNB3 | | 85417 |
| ENSG00000158352 | 19.68613776 | -0.014916591 | 0.1342844 | 0.6190567 | NA | SHROOM4 | | 57477 |
| ENSG00000179222 | 2082.257336 | -0.110393509 | 0.0818987 | 0.0998027 | 0.3015845 | MAGED1 | | 9500 |
| ENSG00000179304 | 331.6496248 | 0.011804383 | 0.1023654 | 0.8620104 | 0.9447423 | FAM156B | | 727866 |

| | | | | | | | |
|---|---|---|---|---|---|---|---|
| ENSG00000182646 | 143.561974 | 0.007081495 | 0.1184093 | 0.9091239 | 0.9640923 | NA | NA |
| ENSG00000184194 | 25.30118522 | -0.029036922 | 0.1365573 | 0.3907865 | NA | GPR173 | 54328 |
| ENSG00000184205 | 1400.663509 | 0.230588689 | 0.0954928 | 0.0033565 | 0.0278477 | TSPYL2 | 64061 |
| ENSG00000232593 | 73.68275604 | -0.005805911 | 0.1252071 | 0.906818 | 0.963256 | KANTR | 102723508 |
| ENSG00000270189 | 76.66237961 | -0.107925751 | 0.1763324 | 0.0897566 | 0.2826765 | NA | NA |
| ENSG00000126012 | 5290.232892 | 0.008650046 | 0.0570101 | 0.897346 | 0.9593537 | KDM5C | 8242 |
| ENSG00000124313 | 325.3196428 | 0.027066046 | 0.1064756 | 0.6922017 | 0.8594028 | IQSEC2 | 23096 |
| ENSG00000072501 | 8886.960371 | -0.128506079 | 0.0544365 | 0.010135 | 0.0641666 | SMC1A | 8243 |
| ENSG00000158423 | 29.16272852 | 0.028047049 | 0.1359901 | 0.4209637 | NA | RIBC1 | 158787 |
| ENSG00000072506 | 2985.525666 | -0.136812075 | 0.075191 | 0.0323978 | 0.1452182 | HSD17B10 | 3028 |
| ENSG00000233250 | 9.881423568 | -0.016731726 | 0.1364083 | 0.4376872 | NA | NA | NA |
| ENSG00000086758 | 13738.91031 | -0.127540854 | 0.0544721 | 0.0101345 | 0.0641666 | HUWE1 | 10075 |
| ENSG00000172943 | 1491.536353 | 0.009419127 | 0.0739391 | 0.8807701 | 0.9518649 | PHF8 | 23133 |
| ENSG00000184083 | 200.5619237 | 0.071167467 | 0.1286311 | 0.3036958 | 0.5728743 | FAM120C | 54954 |
| ENSG00000196632 | 9.526315769 | -0.005857215 | 0.1350658 | 0.7846859 | NA | WNK3 | 65267 |
| ENSG00000158526 | 1968.523247 | -0.053601235 | 0.0674823 | 0.3603864 | 0.623805 | TSR2 | 90121 |
| ENSG00000102302 | 313.523395 | 0.015735101 | 0.1042767 | 0.8175003 | 0.9254567 | FGD1 | 2245 |
| ENSG00000130119 | 1969.232418 | -0.105438276 | 0.0728388 | 0.0896575 | 0.282593 | GNL3L | 54552 |
| ENSG00000102316 | 1238.078978 | -0.277162993 | 0.0956152 | 0.000598 | 0.0073842 | MAGED2 | 10916 |
| ENSG00000169188 | 514.7186124 | 0.230874007 | 0.1275372 | 0.0125868 | 0.0749369 | APEX2 | 27301 |
| ENSG00000182518 | 70.32212602 | -0.017261812 | 0.126656 | 0.7290432 | 0.8790651 | VCF2 | 90736 |
| ENSG00000247746 | 11.38870488 | 0.029275356 | 0.1391913 | 0.218048 | NA | USP51 | 158880 |
| ENSG00000083750 | 130.8470449 | -0.035188108 | 0.1239598 | 0.5531637 | 0.7740184 | RRAGB | 10325 |
| ENSG00000102349 | 12.01716535 | 0.005404602 | 0.1348499 | 0.8141779 | NA | KLF8 | 11279 |
| ENSG00000188021 | 403.0496529 | 0.003784994 | 0.1000615 | 0.9566347 | 0.982546 | UBQLN2 | 29978 |
| ENSG00000204272 | 86.76234183 | 0.039642273 | 0.1293274 | 0.474117 | 0.7140485 | NBDY | 550643 |
| ENSG00000204271 | 74.02875308 | -0.022857724 | 0.1270978 | 0.6539042 | 0.8399571 | SPIN3 | 169981 |
| ENSG00000186787 | 41.82515227 | -0.070640691 | 0.1547144 | 0.1169238 | 0.33377 | SPIN2B | 474343 |
| ENSG00000198455 | 211.6245591 | 0.125769498 | 0.1534425 | 0.1185867 | 0.3359216 | ZXDB | 158586 |
| ENSG00000198205 | 58.29750057 | 0.018632111 | 0.1290634 | 0.6900533 | 0.8589854 | ZXDA | 7789 |
| ENSG00000235437 | 201.6144845 | 0.124666424 | 0.1514963 | 0.1212683 | 0.3410469 | LINC01278 | 92249 |
| ENSG00000186767 | 598.9273845 | -0.018024404 | 0.0903254 | 0.7895748 | 0.9116361 | SPIN4 | 139886 |
| ENSG00000131089 | 175.7501453 | 0.047969819 | 0.1202573 | 0.4695395 | 0.7112477 | ARHGEF9 | 23229 |
| ENSG00000184675 | 220.9103445 | -0.017368824 | 0.1119807 | 0.7853252 | 0.9104192 | AMER1 | 139285 |
| ENSG00000102043 | 54.73925636 | 0.01772671 | 0.1289395 | 0.7015298 | 0.8648958 | MTMR8 | 55613 |
| ENSG00000126970 | 162.5120777 | -0.005456442 | 0.1143336 | 0.9293902 | 0.9726302 | ZC4H2 | 55906 |
| ENSG00000102053 | 61.42456935 | -0.006951941 | 0.1286295 | 0.8724145 | 0.9484107 | ZC3H12B | 340554 |
| ENSG00000001497 | 2051.558076 | -0.04771782 | 0.069834 | 0.4256873 | 0.6782776 | LAS1L | 81887 |
| ENSG00000147065 | 9856.816029 | 0.029204539 | 0.0506917 | 0.5221528 | 0.7495719 | MSN | 4478 |
| ENSG00000260118 | 16.14649498 | 0.022780666 | 0.1364579 | 0.4144756 | NA | NA | NA |
| ENSG00000079482 | 568.8957176 | 0.240545583 | 0.1246676 | 0.0092449 | 0.0604644 | OPHN1 | 4983 |
| ENSG00000181704 | 1588.725693 | -0.005901608 | 0.0722352 | 0.922442 | 0.9701942 | YIPF6 | 286451 |
| ENSG00000090776 | 232.8934219 | 0.071028259 | 0.1210105 | 0.3217929 | 0.5906447 | EFNB1 | 1947 |
| ENSG00000181191 | 335.3241243 | -0.099416385 | 0.12031 | 0.1952166 | 0.4475612 | PJA1 | 64219 |
| ENSG00000158813 | 12.03253704 | 0.007448914 | 0.1347654 | 0.7590695 | NA | EDA | 1896 |
| ENSG00000089289 | 791.405595 | 0.107053892 | 0.0936774 | 0.1385601 | 0.3673017 | IGBP1 | 3476 |
| ENSG00000220925 | 13.79469783 | 0.036355388 | 0.1413387 | 0.1591435 | NA | NA | NA |
| ENSG00000120509 | 775.5307011 | -0.020563706 | 0.084145 | 0.7560507 | 0.8949562 | PDZD11 | 51248 |
| ENSG00000090889 | 3990.991569 | -0.119516151 | 0.0618401 | 0.0303872 | 0.1394565 | KIF4A | 24137 |
| ENSG00000082458 | 201.8826978 | -0.141257814 | 0.1664445 | 0.0906641 | 0.284609 | DLG3 | 1741 |
| ENSG00000230629 | 13.53917689 | -0.003544645 | 0.1342242 | 0.8872962 | NA | RPS23P8 | 653658 |
| ENSG00000227536 | 10.65851474 | 0.001484578 | 0.134856 | 0.9484183 | NA | SOCS5P4 | 100132415 |
| ENSG00000147164 | 1745.971837 | -0.043029575 | 0.0713685 | 0.4786687 | 0.7178141 | SNX12 | 29934 |
| ENSG00000184481 | 120.6753771 | -0.130479763 | 0.1820053 | 0.0863212 | 0.2756984 | FOXO4 | 4303 |
| ENSG00000184634 | 1322.422959 | -0.047314216 | 0.0790638 | 0.4525929 | 0.6987417 | MED12 | 9968 |
| ENSG00000196338 | 14.92419628 | 0.038369635 | 0.1421905 | 0.1351808 | NA | NLGN3 | 54413 |
| ENSG00000147130 | 1079.233106 | -0.169793024 | 0.0917801 | 0.0214722 | 0.109098 | ZMYM3 | 9203 |
| ENSG00000147140 | 11474.67384 | -0.130159877 | 0.0550659 | 0.0097805 | 0.0628603 | NONO | 4841 |
| ENSG00000147166 | 16.7682763 | -0.033145897 | 0.1395749 | 0.235298 | NA | ITGB1BP2 | 26548 |
| ENSG00000147133 | 1236.604734 | -0.021193788 | 0.0742456 | 0.7333239 | 0.8821599 | TAF1 | 6872 |
| ENSG00000147162 | 7071.814149 | -0.020681763 | 0.0642613 | 0.7147514 | 0.8710746 | OGT | 8473 |
| ENSG00000147174 | 61.28037235 | 0.027259651 | 0.1297981 | 0.5766302 | 0.7901945 | GCNA | 93953 |
| ENSG00000102309 | 271.1978549 | 0.025768041 | 0.1067817 | 0.7025749 | 0.8650963 | PIN4 | 5303 |
| ENSG00000186871 | 1117.087552 | -0.135589847 | 0.0885001 | 0.0559475 | 0.2080666 | ERCC6L | 54821 |
| ENSG00000198034 | 15070.22921 | -0.140201593 | 0.0543234 | 0.0049462 | 0.0376802 | RPS4X | 6191 |
| ENSG00000147099 | 450.7644933 | -0.294630609 | 0.147127 | 0.0052457 | 0.0394783 | HDAC8 | 55869 |
| ENSG00000067177 | 688.4974341 | 0.154169544 | 0.1052954 | 0.0505258 | 0.1935431 | PHKA1 | 5255 |
| ENSG00000204116 | 420.1450064 | -0.030067858 | 0.0982354 | 0.6626628 | 0.8438537 | CHIC1 | 53344 |
| ENSG00000225470 | 302.0224122 | 0.056210842 | 0.1110438 | 0.4282068 | 0.6795115 | JPX | 554203 |
| ENSG00000228906 | 10.86833381 | 0.008680542 | 0.1350345 | 0.7096483 | NA | NA | NA |

| | | | | | | | |
|---|---|---|---|---|---|---|---|
| ENSG00000230590 | 47.01629414 | -0.00622257 | 0.1290471 | 0.8829359 | 0.9527552 | FTX | 100302692 |
| ENSG00000271430 | 83.34697818 | -0.037807868 | 0.1293372 | 0.4850452 | 0.7233764 | NA | NA |
| ENSG00000271533 | 39.80872759 | -0.097942519 | 0.1808954 | 0.0430139 | NA | NA | NA |
| ENSG00000147100 | 285.1663925 | 0.357077843 | 0.1794018 | 0.0037812 | 0.0305032 | SLC16A2 | 6567 |
| ENSG00000131263 | 2595.539912 | -0.115467364 | 0.0662369 | 0.0470089 | 0.185557 | RLIM | 51132 |
| ENSG00000131269 | 537.8502865 | -0.08874481 | 0.1022357 | 0.2276438 | 0.4888067 | ABCB7 | 22 |
| ENSG00000094841 | 195.0024427 | -0.001722096 | 0.1112035 | 0.9787615 | 0.9921174 | UPRT | 139596 |
| ENSG00000102383 | 19.91668889 | 0.058214256 | 0.1512397 | 0.0730071 | NA | ZDHHC15 | 158866 |
| ENSG00000215105 | 75.74566228 | -0.059003103 | 0.1393866 | 0.2832088 | 0.5528072 | NA | NA |
| ENSG00000102390 | 869.5755401 | -0.087143079 | 0.0895954 | 0.2118283 | 0.4684524 | PBDC1 | 51260 |
| ENSG00000198934 | 84.20374816 | -0.011209766 | 0.1238129 | 0.8311461 | 0.9304016 | MAGEE1 | 57692 |
| ENSG00000085224 | 2629.517623 | -0.164911607 | 0.0719601 | 0.0081195 | 0.054647 | ATRX | 546 |
| ENSG00000102158 | 2004.428782 | -0.178336691 | 0.0813264 | 0.0093199 | 0.0608868 | MAGT1 | 84061 |
| ENSG00000131174 | 1435.341615 | -0.055156829 | 0.0785845 | 0.3961626 | 0.6563006 | COX7B | 1349 |
| ENSG00000165240 | 247.730261 | -0.062250199 | 0.1165978 | 0.377547 | 0.6392414 | ATP7A | 538 |
| ENSG00000102144 | 9316.897867 | -0.152853567 | 0.0596842 | 0.0042196 | 0.0332364 | PGK1 | 5230 |
| ENSG00000187325 | 954.1039039 | -0.470123816 | 0.1175758 | 5.06E-06 | 0.0001413 | TAF9B | 51616 |
| ENSG00000165288 | 593.5058098 | -0.269242892 | 0.1251632 | 0.0045694 | 0.0354411 | BRWD3 | 254065 |
| ENSG00000198157 | 96.42080644 | -0.1109757 | 0.1732222 | 0.1009769 | 0.3038412 | HMGN5 | 79366 |
| ENSG00000131171 | 522.7517631 | -0.11128952 | 0.1089797 | 0.1467661 | 0.3800339 | SH3BGRL | 6451 |
| ENSG00000072133 | 262.8166695 | 0.038241052 | 0.1095903 | 0.577665 | 0.7911317 | RPS6KA6 | 27330 |
| ENSG00000165259 | 56.49054091 | 0.04253778 | 0.1363539 | 0.3634628 | 0.6264732 | HDX | 139324 |
| ENSG00000155008 | 161.3508353 | 0.001912834 | 0.1145165 | 0.9696175 | 0.9881436 | APOOL | 139322 |
| ENSG00000147180 | 248.0264379 | 0.337132899 | 0.1925087 | 0.0064533 | 0.0459736 | ZNF711 | 7552 |
| ENSG00000188419 | 824.5766744 | 0.159565423 | 0.1017822 | 0.0402224 | 0.1682802 | CHM | 1121 |
| ENSG00000237506 | 38.83822409 | -0.046013231 | 0.1407671 | 0.2606831 | NA | RPSAP15 | 220885 |
| ENSG00000186310 | 14.20346357 | -0.010022924 | 0.1346668 | 0.6944293 | NA | NAP1L3 | 4675 |
| ENSG00000147202 | 434.4910321 | 0.038722256 | 0.0989357 | 0.5787787 | 0.7915974 | DIAPH2 | 1730 |
| ENSG00000000003 | 233.4076543 | -0.026703184 | 0.1103601 | 0.6837058 | 0.8549799 | TSPAN6 | 7105 |
| ENSG00000102362 | 240.5778622 | 0.158965226 | 0.1597694 | 0.0727858 | 0.2466524 | SYTL4 | 94121 |
| ENSG00000101811 | 868.8297685 | -0.067657638 | 0.0869715 | 0.3214165 | 0.590367 | CSTF2 | 1478 |
| ENSG00000188917 | 531.2811768 | -0.004530256 | 0.0921601 | 0.9459025 | 0.9784014 | TRMT2B | 79979 |
| ENSG00000102384 | 1209.876155 | -0.282505855 | 0.0964484 | 0.0005262 | 0.0067243 | CENPI | 2491 |
| ENSG00000126953 | 530.7931172 | 0.045779075 | 0.0946624 | 0.5083978 | 0.7401841 | TIMM8A | 1678 |
| ENSG00000241343 | 259.5363662 | -0.085651321 | 0.1247844 | 0.247756 | 0.5140388 | RPL36A | 6173 |
| ENSG00000102393 | 1074.362446 | -0.034133268 | 0.078219 | 0.5946779 | 0.8031487 | GLA | 2717 |
| ENSG00000126945 | 1457.233681 | -0.011002562 | 0.0709202 | 0.8553654 | 0.9417081 | HNRNPH2 | 3188 |
| ENSG00000198960 | 204.687282 | 0.060966305 | 0.1210726 | 0.3766321 | 0.6384862 | ARMCX6 | 54470 |
| ENSG00000102401 | 807.0943274 | 0.071360058 | 0.0897417 | 0.3046324 | 0.5735672 | ARMCX3 | 51566 |
| ENSG00000184515 | 9.95122672 | -0.022164183 | 0.1374966 | 0.3137986 | NA | BEX5 | 340542 |
| ENSG00000125962 | 265.1153234 | -0.068719582 | 0.1168612 | 0.3387401 | 0.6062239 | ARMCX5 | 64860 |
| ENSG00000271147 | 34.05805247 | -0.030535532 | 0.1354902 | 0.4237783 | NA | NA | NA |
| ENSG00000198932 | 17.07649168 | -0.016368028 | 0.1349794 | 0.5614046 | NA | GPRASP1 | 9737 |
| ENSG00000158301 | 105.9769526 | -0.005905541 | 0.1210773 | 0.91591 | 0.9666376 | GPRASP2 | 114928 |
| ENSG00000198908 | 67.10011419 | -0.061304829 | 0.1423015 | 0.2487733 | 0.5151429 | GPRASP3 | 80823 |
| ENSG00000223546 | 81.55081578 | 0.013599096 | 0.125913 | 0.7891292 | 0.9116361 | NA | NA |
| ENSG00000239407 | 36.78814571 | 0.014373129 | 0.1312716 | 0.7179713 | NA | NA | NA |
| ENSG00000180964 | 22.50516126 | 0.043526721 | 0.1430319 | 0.1666656 | NA | TCEAL8 | 90843 |
| ENSG00000133134 | 11.36236196 | 0.05025769 | 0.148819 | 0.0304825 | NA | BEX2 | 84707 |
| ENSG00000185222 | 385.711669 | 0.150140179 | 0.1319745 | 0.0775348 | 0.2567132 | TCEAL9 | 51186 |
| ENSG00000166681 | 507.495534 | -0.0483911 | 0.1021363 | 0.4908856 | 0.7273003 | BEX3 | 27018 |
| ENSG00000133142 | 1224.062786 | 0.080541151 | 0.0820491 | 0.2270953 | 0.4882146 | TCEAL4 | 79921 |
| ENSG00000196507 | 404.9218766 | 0.356667534 | 0.1589173 | 0.0022076 | 0.0204172 | TCEAL3 | 85012 |
| ENSG00000172465 | 253.5063055 | 0.020958604 | 0.1076081 | 0.75644 | 0.8951803 | TCEAL1 | 9338 |
| ENSG00000123562 | 3931.684913 | -0.072329055 | 0.0664297 | 0.2126776 | 0.4697082 | MORF4L2 | 9643 |
| ENSG00000123570 | 25.61073663 | 0.001953058 | 0.1321496 | 0.9559613 | NA | RAB9B | 51209 |
| ENSG00000234345 | 9.844232876 | -0.020746591 | 0.1371798 | 0.3429304 | NA | NA | NA |
| ENSG00000231728 | 23.70428769 | -0.02675071 | 0.1362067 | 0.4118613 | NA | NA | NA |
| ENSG00000158427 | 155.3431083 | -0.015339261 | 0.1162804 | 0.8029252 | 0.9177972 | TMSB15B | 286527 |
| ENSG00000260831 | 13.21596236 | 0.005098125 | 0.1344387 | 0.8396413 | NA | NA | NA |
| ENSG00000176274 | 47.29348477 | 0.016031599 | 0.1312114 | 0.6932527 | 0.8597393 | NA | NA |
| ENSG00000166707 | 9.327699764 | -0.001046443 | 0.1349947 | 0.9580169 | NA | ZCCHC18 | 644353 |
| ENSG00000123575 | 3090.499675 | -0.251136417 | 0.0688618 | 5.53E-05 | 0.0010751 | FAM199X | 139231 |
| ENSG00000147231 | 8.850834646 | 0.009176977 | 0.1356612 | 0.6428275 | NA | RADX | 55086 |
| ENSG00000133138 | 225.0354749 | -0.023752803 | 0.1124275 | 0.7155606 | 0.8712178 | TBC1D8B | 54885 |
| ENSG00000133131 | 852.6307316 | 0.141712468 | 0.0956883 | 0.0570181 | 0.2111171 | MORC4 | 79710 |
| ENSG00000089682 | 344.5407898 | -0.068242083 | 0.1098397 | 0.3456549 | 0.6118494 | RBM41 | 55285 |
| ENSG00000147234 | 11.26360174 | 0.033126791 | 0.1405337 | 0.162746 | NA | FRMPD3 | 84443 |
| ENSG00000147224 | 2303.748061 | 0.058163949 | 0.0695352 | 0.3321186 | 0.5992396 | PRPS1 | 5631 |
| ENSG00000157514 | 77.93732208 | 0.115306193 | 0.1847435 | 0.0760798 | 0.2537974 | TSC22D3 | 1831 |

| | | | | | | | |
|---|---|---|---|---|---|---|---|
| ENSG00000080561 | 184.187958 | 0.029643032 | 0.1148804 | 0.649567 | 0.8369284 | MID2 | 11043 |
| ENSG00000101842 | 48.20337485 | -0.037415607 | 0.1350671 | 0.4049839 | 0.6614808 | VSIG1 | 340547 |
| ENSG00000101843 | 786.9386148 | -0.352110853 | 0.1105823 | 0.0001595 | 0.0025487 | PSMD10 | 5716 |
| ENSG00000101844 | 223.1305519 | -0.062497171 | 0.1206619 | 0.3676245 | 0.6303907 | ATG4A | 115201 |
| ENSG00000188153 | 172.4443724 | -0.014356377 | 0.1141927 | 0.8193889 | 0.925668 | COL4A5 | 1287 |
| ENSG00000101888 | 350.1837287 | -0.376446593 | 0.159602 | 0.0015288 | 0.0155847 | NXT2 | 55916 |
| ENSG00000068366 | 1494.863776 | -0.112243097 | 0.0810948 | 0.0924562 | 0.287535 | ACSL4 | 2182 |
| ENSG00000157600 | 523.0156213 | -0.049871821 | 0.0969114 | 0.4760576 | 0.7156114 | TMEM164 | 84187 |
| ENSG00000101935 | 311.1163644 | -0.029606361 | 0.1066196 | 0.6635623 | 0.8440675 | AMMECR1 | 9949 |
| ENSG00000225031 | 15.01810072 | -0.001683597 | 0.133929 | 0.9475465 | NA | NA | NA |
| ENSG00000101901 | 589.2885774 | -0.014197367 | 0.0905431 | 0.8334306 | 0.9305394 | ALG13 | 79868 |
| ENSG00000126016 | 106.7478187 | -0.115584362 | 0.1732116 | 0.1023259 | 0.3064005 | AMOT | 154796 |
| ENSG00000123496 | 55.22120431 | 0.01827814 | 0.1292388 | 0.6911666 | 0.8593387 | IL13RA2 | 3598 |
| ENSG00000130224 | 57.29125724 | 0.003508065 | 0.1270982 | 0.942024 | 0.977419 | LRCH2 | 57631 |
| ENSG00000102024 | 2275.801011 | -0.086709516 | 0.0691096 | 0.1465858 | 0.3799132 | PLS3 | 5358 |
| ENSG00000003096 | 112.4585261 | 0.008817313 | 0.1202355 | 0.8794844 | 0.9513156 | KLHL13 | 90293 |
| ENSG00000131725 | 580.4986782 | -0.078872097 | 0.0988253 | 0.2729029 | 0.5409432 | WDR44 | 54521 |
| ENSG00000147251 | 203.339863 | 0.047451588 | 0.1170416 | 0.484672 | 0.7231745 | DOCK11 | 139818 |
| ENSG00000131724 | 1391.04638 | -0.112994173 | 0.078925 | 0.0854193 | 0.2735548 | IL13RA1 | 3597 |
| ENSG00000175556 | 252.8137131 | 0.188171526 | 0.1704499 | 0.0478867 | 0.1870785 | LONRF3 | 79836 |
| ENSG00000101856 | 1798.826337 | -0.061713255 | 0.0710874 | 0.3096016 | 0.5790036 | PGRMC1 | 10857 |
| ENSG00000077713 | 412.9559573 | 0.042422766 | 0.100108 | 0.5439024 | 0.7661909 | SLC25A43 | 203427 |
| ENSG00000224281 | 40.93988532 | -0.00631231 | 0.129769 | 0.8763436 | 0.949742 | SLC25A5-AS | 100303728 |
| ENSG00000005022 | 9066.413389 | -0.210668154 | 0.062452 | 0.0002083 | 0.0031633 | SLC25A5 | 292 |
| ENSG00000018610 | 720.7636484 | 0.023579836 | 0.0875917 | 0.7275002 | 0.8781575 | STEEP1 | 63932 |
| ENSG00000077721 | 1933.674647 | 0.060710855 | 0.0682392 | 0.3054142 | 0.5744836 | UBE2A | 7319 |
| ENSG00000186416 | 848.7831018 | 0.054278581 | 0.0847726 | 0.4171416 | 0.6706994 | NKRF | 55922 |
| ENSG00000125354 | 555.3493956 | -0.054065263 | 0.0942105 | 0.4381622 | 0.6881889 | SEPTIN6 | 23157 |
| ENSG00000198918 | 9600.553514 | -0.162464391 | 0.0686541 | 0.0092123 | 0.0603189 | RPL39 | 6170 |
| ENSG00000125351 | 1243.055711 | 0.220265785 | 0.0976303 | 0.0054662 | 0.0405875 | UPF3B | 65109 |
| ENSG00000125352 | 251.5832006 | 0.069724556 | 0.11947 | 0.3308704 | 0.5989207 | RNF113A | 7737 |
| ENSG00000125356 | 1390.868542 | -0.3133468 | 0.0893203 | 6.23E-05 | 0.0011838 | NDUFA1 | 4694 |
| ENSG00000227285 | 10.58586176 | -0.009982275 | 0.1352457 | 0.6575085 | NA | NA | NA |
| ENSG00000101882 | 342.1337865 | 0.034838485 | 0.1049591 | 0.6150329 | 0.8157779 | NKAP | 79576 |
| ENSG00000233382 | 27.71550678 | -0.066034058 | 0.1546231 | 0.0840911 | NA | NKAPP1 | 158801 |
| ENSG00000177485 | 807.4805263 | 0.029508937 | 0.0847089 | 0.6800258 | 0.853781 | ZBTB33 | 10009 |
| ENSG00000005893 | 1946.462086 | -0.063998089 | 0.068129 | 0.2782357 | 0.5467586 | LAMP2 | 3920 |
| ENSG00000158290 | 997.1277448 | -0.131631372 | 0.089764 | 0.0652705 | 0.2305488 | CUL4B | 8450 |
| ENSG00000232119 | 573.7974978 | -0.064944774 | 0.0966356 | 0.3587432 | 0.6220096 | MCTS1 | 28985 |
| ENSG00000171155 | 436.4542418 | -0.220330173 | 0.1417079 | 0.0209439 | 0.1069724 | C1GALT1C1 | 29071 |
| ENSG00000125676 | 4012.541621 | -0.098113192 | 0.0628083 | 0.0784257 | 0.2585037 | THOC2 | 57187 |
| ENSG00000232149 | 15.77940197 | -0.025950505 | 0.1374645 | 0.3369667 | NA | FERP1 | 553112 |
| ENSG00000101966 | 1765.505582 | -0.053702168 | 0.0692678 | 0.3680807 | 0.6308952 | XIAP | 331 |
| ENSG00000101972 | 2510.847107 | -0.118591334 | 0.0672351 | 0.0438156 | 0.1775548 | STAG2 | 10735 |
| ENSG00000102038 | 2940.931152 | -0.07444737 | 0.0676673 | 0.2054291 | 0.4607272 | SMARCA1 | 6594 |
| ENSG00000122126 | 1271.72838 | 0.101504986 | 0.0812821 | 0.1280886 | 0.351634 | OCRL | 4952 |
| ENSG00000171388 | 22.95937595 | 0.020121379 | 0.1346391 | 0.5363635 | NA | APLN | 8862 |
| ENSG00000188706 | 717.6023316 | 0.051710407 | 0.0952196 | 0.4962794 | 0.7315799 | ZDHHC9 | 51114 |
| ENSG00000156697 | 1391.708732 | 0.147117765 | 0.0826063 | 0.0315953 | 0.1427166 | UTP14A | 10813 |
| ENSG00000085185 | 526.7972867 | 0.081289599 | 0.1062838 | 0.270734 | 0.5386338 | BCORL1 | 63035 |
| ENSG00000102034 | 1025.467965 | 0.308930595 | 0.1000179 | 0.0002726 | 0.0039825 | ELF4 | 2000 |
| ENSG00000156709 | 1985.90967 | -0.121368949 | 0.0719067 | 0.0492809 | 0.1907389 | AIFM1 | 9131 |
| ENSG00000056277 | 281.1996689 | 0.06637793 | 0.116485 | 0.3537346 | 0.6187516 | ZNF280C | 55609 |
| ENSG00000102078 | 300.0469349 | 0.076703368 | 0.1160871 | 0.295748 | 0.5658693 | SLC25A14 | 9016 |
| ENSG00000134597 | 677.0767646 | 0.140984448 | 0.1138481 | 0.0795854 | 0.2612807 | RBMX2 | 51634 |
| ENSG00000165675 | 335.7793233 | 0.061620216 | 0.1102922 | 0.3898817 | 0.6502297 | ENOX2 | 10495 |
| ENSG00000213468 | 8.48169119 | 0.03587832 | 0.1422549 | 0.0772998 | NA | FIRRE | 286467 |
| ENSG00000134602 | 1521.44596 | -0.042999074 | 0.0728069 | 0.4858015 | 0.7237147 | STK26 | 51765 |
| ENSG00000123728 | 788.7623255 | -0.25556181 | 0.1100753 | 0.0034097 | 0.0281479 | RAP2C | 57826 |
| ENSG00000232160 | 17.41732814 | 0.012859706 | 0.1343662 | 0.6555279 | NA | RAP2C-AS1 | 101928578 |
| ENSG00000076716 | 93.4963594 | 0.021793723 | 0.1259982 | 0.6804396 | 0.8538239 | GPC4 | 2239 |
| ENSG00000156531 | 1813.570065 | -0.109596424 | 0.0744022 | 0.0823636 | 0.2671729 | PHF6 | 84295 |
| ENSG00000165704 | 1280.865458 | -0.150615502 | 0.0863631 | 0.0324474 | 0.1452349 | HPRT1 | 3251 |
| ENSG00000223749 | 14.04116112 | -0.03151531 | 0.1394012 | 0.22932 | NA | NA | NA |
| ENSG00000156504 | 1516.980084 | -0.020539411 | 0.0728468 | 0.738435 | 0.885525 | PABIR2 | 159090 |
| ENSG00000156500 | 60.57089704 | 0.022859247 | 0.1289467 | 0.6363534 | 0.8275712 | PABIR3 | 159091 |
| ENSG00000101928 | 326.2283918 | 0.062695175 | 0.110012 | 0.3838171 | 0.6446251 | MOSPD1 | 56180 |
| ENSG00000212747 | 140.5791221 | -0.033684868 | 0.1205035 | 0.587944 | 0.7979376 | RTL8B | 441518 |
| ENSG00000134590 | 754.4075787 | -0.093737315 | 0.0924595 | 0.1855781 | 0.4358915 | RTL8C | 8933 |
| ENSG00000203950 | 617.9296582 | -0.015550638 | 0.0891929 | 0.8164513 | 0.9252377 | RTL8A | 26071 |

| | | | | | | | | |
|---|---|---|---|---|---|---|---|---|
| ENSG00000186376 | 327.7143748 | 0.035094068 | 0.1042692 | 0.6130066 | 0.8148077 | ZNF75D | | 7626 |
| ENSG00000173275 | 217.1371984 | 0.034270278 | 0.1130659 | 0.608613 | 0.8122953 | ZNF449 | | 203523 |
| ENSG00000165359 | 301.7294468 | -0.050346678 | 0.1133066 | 0.4644041 | 0.7082689 | INTS6L | | 203522 |
| ENSG00000169446 | 1332.684864 | -0.211655503 | 0.0860288 | 0.0034796 | 0.0285627 | MMGT1 | | 93380 |
| ENSG00000198689 | 722.7959734 | -0.25753739 | 0.1163917 | 0.0043452 | 0.033951 | SLC9A6 | | 10479 |
| ENSG00000022267 | 1274.146628 | 0.581878765 | 0.0901857 | 8.22E-12 | 8.65E-10 | FHL1 | | 2273 |
| ENSG00000129680 | 678.8706868 | -0.006667253 | 0.086965 | 0.919922 | 0.9690331 | MAP7D3 | | 79649 |
| ENSG00000102241 | 1920.693594 | -0.069273719 | 0.0701492 | 0.2494556 | 0.5155947 | HTATSF1 | | 27336 |
| ENSG00000232611 | 29.88259272 | 0.076271143 | 0.1628729 | 0.0531211 | NA | NA | NA | |
| ENSG00000147274 | 5457.473568 | -0.231352545 | 0.0673239 | 0.0001403 | 0.0022824 | RBMX | | 27316 |
| ENSG00000234062 | 24.84623025 | 0.022967329 | 0.1349894 | 0.4945657 | NA | NA | NA | |
| ENSG00000101974 | 1208.987163 | -0.028072607 | 0.0766686 | 0.6580606 | 0.8423052 | ATP11C | | 286410 |
| ENSG00000203930 | 18.22542683 | -0.018197224 | 0.1353163 | 0.5190912 | NA | LINC00632 | | 286411 |
| ENSG00000182195 | 973.1199829 | -0.126023625 | 0.092486 | 0.0816141 | 0.2656303 | LDOC1 | | 23641 |
| ENSG00000179542 | 20.38147381 | 0.003151884 | 0.1329852 | 0.9207149 | NA | SLITRK4 | | 139065 |
| ENSG00000102081 | 1305.319447 | -0.286524898 | 0.0883595 | 0.0001865 | 0.0028966 | FMR1 | | 2332 |
| ENSG00000010404 | 962.7394458 | -0.013944266 | 0.0829851 | 0.8317463 | 0.9304418 | IDS | | 3423 |
| ENSG00000241769 | 18.29201624 | -0.051405898 | 0.1474616 | 0.0944941 | NA | EOLA1-DT | | 100131434 |
| ENSG00000197620 | 212.4974523 | -0.000398077 | 0.1101184 | 0.9940877 | 0.9977151 | EOLA1 | | 91966 |
| ENSG00000155984 | 168.9460138 | -0.00982412 | 0.116382 | 0.8706302 | 0.9478556 | NA | NA | |
| ENSG00000197021 | 538.7166372 | -0.00245355 | 0.0906596 | 0.9703239 | 0.9882505 | EOLA2 | | 541578 |
| ENSG00000235703 | 27.42384622 | -0.027137254 | 0.1355 | 0.441035 | NA | EOLA2-DT | | 100272228 |
| ENSG00000013619 | 387.2400663 | -0.012478294 | 0.0979132 | 0.8544958 | 0.9412625 | MAMLD1 | | 10046 |
| ENSG00000171100 | 322.1185297 | -0.064402063 | 0.1112224 | 0.3690505 | 0.6315712 | MTM1 | | 4534 |
| ENSG00000063601 | 1521.889039 | 0.143318995 | 0.0809728 | 0.0336344 | 0.148544 | MTMR1 | | 8776 |
| ENSG00000102181 | 821.1758833 | -0.081749721 | 0.0928688 | 0.2480575 | 0.5143902 | CD99L2 | | 83692 |
| ENSG00000029993 | 1569.9058 | -0.48380587 | 0.082308 | 3.56E-10 | 2.72E-08 | HMGB3 | | 3149 |
| ENSG00000160131 | 1513.970591 | 0.018970162 | 0.0750657 | 0.7638248 | 0.8982943 | VMA21 | | 203547 |
| ENSG00000213401 | 9.914304951 | 0.00298152 | 0.1350346 | 0.8899028 | NA | MAGEA12 | | 4111 |
| ENSG00000147400 | 861.6716368 | -0.037421754 | 0.0840024 | 0.5782367 | 0.791282 | CETN2 | | 1069 |
| ENSG00000147383 | 1032.892947 | -0.064749204 | 0.0827857 | 0.3313753 | 0.5991015 | NSDHL | | 50814 |
| ENSG00000147394 | 1123.42721 | 0.854642187 | 0.106849 | 7.72E-17 | 1.67E-14 | ZNF185 | | 7739 |
| ENSG00000063587 | 301.6832463 | -0.013195745 | 0.1036185 | 0.8446569 | 0.936404 | ZNF275 | | 10838 |
| ENSG00000189420 | 15.43147794 | 0.019732304 | 0.1358739 | 0.4710214 | NA | ZFP92 | | 139735 |
| ENSG00000259886 | 30.71625218 | -0.027458558 | 0.1349034 | 0.4610263 | NA | NA | NA | |
| ENSG00000183479 | 28.76503762 | 0.051365744 | 0.145632 | 0.1558297 | NA | TREX2 | | 11219 |
| ENSG00000213397 | 34.57930067 | 0.014138628 | 0.1317522 | 0.7122458 | NA | HAUS7 | | 55559 |
| ENSG00000147382 | 428.945498 | -0.073055503 | 0.1059774 | 0.3156391 | 0.5844001 | NA | NA | |
| ENSG00000260081 | 9.383599331 | -0.020649537 | 0.1372556 | 0.332524 | NA | LOC1053733 | | 105373383 |
| ENSG00000130829 | 21.3241503 | -0.01650567 | 0.1342006 | 0.5983159 | NA | DUSP9 | | 1852 |
| ENSG00000130821 | 1202.920107 | 0.060774611 | 0.0826875 | 0.3631932 | 0.6262668 | SLC6A8 | | 6535 |
| ENSG00000185825 | 4533.746659 | -0.07457142 | 0.0587382 | 0.1584388 | 0.3986051 | BCAP31 | | 10134 |
| ENSG00000101986 | 221.155346 | 0.073932901 | 0.1268536 | 0.2958499 | 0.5659367 | ABCD1 | | 215 |
| ENSG00000198753 | 8.819540971 | -0.008274896 | 0.1353783 | 0.6904849 | NA | PLXNB3 | | 5365 |
| ENSG00000067829 | 832.3721246 | -0.035030457 | 0.0860384 | 0.6210493 | 0.818389 | IDH3G | | 3421 |
| ENSG00000180879 | 1358.030099 | 0.066595607 | 0.0767484 | 0.2977401 | 0.5680006 | SSR4 | | 6748 |
| ENSG00000067840 | 246.2746155 | 0.064354919 | 0.1219759 | 0.3569105 | 0.6201207 | PDZD4 | | 57595 |
| ENSG00000198910 | 740.6404799 | 0.676004495 | 0.1105683 | 6.43E-11 | 5.56E-09 | L1CAM | | 3897 |
| ENSG00000089820 | 8.310286609 | 0.025961827 | 0.1388515 | 0.1968753 | NA | ARHGAP4 | | 393 |
| ENSG00000102030 | 784.4853751 | -0.030141247 | 0.0874166 | 0.6540809 | 0.8399571 | NAA10 | | 8260 |
| ENSG00000172534 | 4413.218051 | -0.23659135 | 0.0630244 | 4.07E-05 | 0.0008285 | HCFC1 | | 3054 |
| ENSG00000177854 | 170.2001069 | 0.032255668 | 0.1173208 | 0.6152068 | 0.8158539 | TMEM187 | | 8269 |
| ENSG00000184216 | 10436.69711 | 0.089400229 | 0.0520034 | 0.0640927 | 0.2277105 | IRAK1 | | 3654 |
| ENSG00000169057 | 1919.443887 | -0.104106725 | 0.0751766 | 0.1003609 | 0.3025862 | MECP2 | | 4204 |
| ENSG00000196924 | 37563.81351 | 0.00367817 | 0.051925 | 0.9393395 | 0.9764939 | FLNA | | 2316 |
| ENSG00000102119 | 1334.560171 | 0.192299858 | 0.0863903 | 0.0075575 | 0.0517463 | EMD | | 2010 |
| ENSG00000147403 | 18144.38065 | -0.032953477 | 0.0530486 | 0.4989954 | 0.7341862 | RPL10 | | 6134 |
| ENSG00000013563 | 491.3480911 | 0.114474577 | 0.1103421 | 0.139491 | 0.3687247 | DNASE1L1 | | 1774 |
| ENSG00000102125 | 579.3804864 | 0.180342893 | 0.1167107 | 0.0329251 | 0.1464825 | TAFAZZIN | | 6901 |
| ENSG00000197180 | 18.72465742 | 0.04854032 | 0.1460258 | 0.1073061 | NA | ATP6AP1-DT | | 158960 |
| ENSG00000071553 | 2266.688021 | 0.123732778 | 0.070836 | 0.043155 | 0.1758285 | ATP6AP1 | | 537 |
| ENSG00000203879 | 3446.673769 | 0.113960917 | 0.0632816 | 0.0426234 | 0.1745735 | GDI1 | | 2664 |
| ENSG00000071859 | 2061.405386 | 0.030037113 | 0.0665023 | 0.6088167 | 0.8123066 | FAM50A | | 9130 |
| ENSG00000130827 | 909.2660283 | 0.03760571 | 0.0823522 | 0.5646139 | 0.7820587 | PLXNA3 | | 55558 |
| ENSG00000196976 | 476.7033361 | -0.038958771 | 0.1007453 | 0.5750284 | 0.7888821 | LAGE3 | | 8270 |
| ENSG00000102178 | 1226.368143 | 0.00868014 | 0.076596 | 0.8920608 | 0.956385 | UBL4A | | 8266 |
| ENSG00000126903 | 622.4601526 | 0.137365767 | 0.106925 | 0.0791006 | 0.2601277 | SLC10A3 | | 8273 |
| ENSG00000071889 | 520.2857671 | 0.042081571 | 0.0943861 | 0.5502708 | 0.7715069 | FAM3A | | 60343 |
| ENSG00000160211 | 1993.247437 | -0.067596631 | 0.067989 | 0.251626 | 0.5183852 | G6PD | | 2539 |
| ENSG00000073009 | 289.0455752 | 0.085993132 | 0.121934 | 0.2544533 | 0.5211459 | NA | NA | |

| | | | | | | | | |
|---|---|---|---|---|---|---|---|---|
| ENSG00000224882 | 60.79755589 | 0.018752954 | 0.1283101 | 0.696064 | 0.8613054 | NA | NA | |
| ENSG00000130826 | 5074.526607 | -0.039772499 | 0.0550343 | 0.4486574 | 0.6959957 | DKC1 | | 1736 |
| ENSG00000130830 | 320.0455136 | -0.017780148 | 0.1064985 | 0.7904352 | 0.9121996 | MPP1 | | 4354 |
| ENSG00000185010 | 125.6403992 | 0.012093364 | 0.1197625 | 0.8362533 | 0.9319553 | F8 | | 2157 |
| ENSG00000197932 | 319.3907681 | 0.053417584 | 0.108453 | 0.4503115 | 0.6972061 | NA | NA | |
| ENSG00000165775 | 395.1824284 | 0.018154893 | 0.1017183 | 0.7895726 | 0.9116361 | FUNDC2 | | 65991 |
| ENSG00000214827 | 39.2832589 | -0.003603171 | 0.1299827 | 0.9269075 | NA | MTCP1 | | 4515 |
| ENSG00000185515 | 1067.438049 | -0.23722391 | 0.0944143 | 0.0024369 | 0.0219981 | BRCC3 | | 79184 |
| ENSG00000155959 | 2139.047382 | -0.142894238 | 0.0760346 | 0.0269429 | 0.1276472 | VBP1 | | 7411 |
| ENSG00000155961 | 63.42783043 | 0.086692382 | 0.1608717 | 0.1226175 | 0.343137 | RAB39B | | 116442 |
| ENSG00000185990 | 70.72527229 | -0.002918314 | 0.125151 | 0.9524571 | 0.9806656 | NA | NA | |
| ENSG00000185978 | 11.58334224 | -0.008563051 | 0.1349177 | 0.7160222 | NA | NA | NA | |
| ENSG00000185973 | 155.5229908 | -0.028680006 | 0.1176392 | 0.6484567 | 0.836587 | TMLHE | | 55217 |
| ENSG00000168939 | 70.66694975 | 0.055285265 | 0.1387009 | 0.2992498 | 0.5693326 | SPRY3 | | 10251 |
| ENSG00000124333 | 1331.192896 | -0.121222039 | 0.0856929 | 0.0797128 | 0.2613697 | VAMP7 | | 6845 |
| ENSG00000182484 | 58.3285167 | -0.007651552 | 0.1272745 | 0.8687683 | 0.9471139 | WASH6P | | 653440 |
| ENSG00000223508 | 84.84058032 | 0.157212777 | 0.2354839 | 0.0428391 | 0.1749665 | NA | NA | |
| ENSG00000172748 | 18.20516349 | 0.062168023 | 0.1544278 | 0.0473992 | NA | ZNF596 | | 169270 |
| ENSG00000272812 | 10.59670954 | 0.015290526 | 0.1359306 | 0.5067763 | NA | NA | NA | |
| ENSG00000249868 | 19.98830681 | 0.002835367 | 0.1333087 | 0.9257325 | NA | NA | NA | |
| ENSG00000147364 | 143.7548325 | -0.008559248 | 0.1168582 | 0.887042 | 0.9545517 | FBXO25 | | 26260 |
| ENSG00000272293 | 9.462409634 | -0.015230496 | 0.1362542 | 0.4657963 | NA | NA | NA | |
| ENSG00000272005 | 30.2275387 | -0.021442039 | 0.1335903 | 0.5576831 | NA | NA | NA | |
| ENSG00000180190 | 337.9669293 | -0.054256596 | 0.1070191 | 0.442574 | 0.6916921 | TDRP | | 157695 |
| ENSG00000104714 | 315.8442019 | 0.001468908 | 0.1035423 | 0.9844804 | 0.9940394 | ERICH1 | | 157697 |
| ENSG00000182372 | 309.4946191 | 0.080150513 | 0.119518 | 0.2773375 | 0.5460273 | CLN8 | | 2055 |
| ENSG00000253982 | 29.58742488 | 0.019869962 | 0.1338697 | 0.5714806 | NA | CLN8-AS1 | | 101927752 |
| ENSG00000104728 | 1198.732202 | -0.055668074 | 0.0784236 | 0.388188 | 0.6485607 | ARHGEF10 | | 9639 |
| ENSG00000176595 | 21.22785492 | -0.003499867 | 0.133418 | 0.9023647 | NA | KBTBD11 | | 9920 |
| ENSG00000246089 | 62.53813335 | 0.017486118 | 0.1280739 | 0.716135 | 0.8714352 | MCPH1-DT | | 100287015 |
| ENSG00000147316 | 383.0213363 | 0.011097736 | 0.0985534 | 0.8721931 | 0.9484107 | MCPH1 | | 79648 |
| ENSG00000249898 | 11.35792442 | 0.032139094 | 0.140204 | 0.1724533 | NA | NA | NA | |
| ENSG00000271927 | 17.31795972 | -0.004535787 | 0.1337506 | 0.8686479 | NA | NA | NA | |
| ENSG00000155189 | 1000.035638 | 0.044247205 | 0.0803722 | 0.4927941 | 0.7286234 | AGPAT5 | | 55326 |
| ENSG00000249188 | 9.869124931 | 0.011829987 | 0.1356363 | 0.5877659 | NA | ENPP7P1 | | 100421823 |
| ENSG00000173295 | 64.29187916 | 0.221030449 | 0.4097788 | 0.0180449 | 0.096424 | FAM86B3P | | 286042 |
| ENSG00000182319 | 227.3225663 | 0.058934006 | 0.1179834 | 0.3987837 | 0.6577504 | NA | NA | |
| ENSG00000147324 | 892.7550249 | -0.036702062 | 0.0825231 | 0.5793564 | 0.7918192 | MFHAS1 | | 9258 |
| ENSG00000104626 | 764.0320192 | -0.034047004 | 0.0845677 | 0.6105584 | 0.8134047 | ERI1 | | 90459 |
| ENSG00000173281 | 101.9136929 | 0.053359258 | 0.1320253 | 0.3694194 | 0.6317991 | PPP1R3B | | 79660 |
| ENSG00000272267 | 9.139559008 | 0.000515883 | 0.1349695 | 0.9829239 | NA | NA | NA | |
| ENSG00000173273 | 671.2079146 | 0.072924953 | 0.09752 | 0.3087718 | 0.5780996 | TNKS | | 8658 |
| ENSG00000175806 | 66.27128588 | 0.057089293 | 0.1403549 | 0.2763498 | 0.5446335 | MSRA | | 4482 |
| ENSG00000183638 | 11.68936396 | -0.016765256 | 0.136146 | 0.4661701 | NA | RP1L1 | | 94137 |
| ENSG00000171056 | 14.76494964 | 0.00912173 | 0.1346252 | 0.722594 | NA | SOX7 | | 83595 |
| ENSG00000254093 | 189.6446025 | 0.521660635 | 0.2172285 | 0.0008957 | 0.0101675 | PINX1 | | 54984 |
| ENSG00000104643 | 354.3035389 | 0.019101229 | 0.1002566 | 0.7794763 | 0.9078735 | MTMR9 | | 66036 |
| ENSG00000154328 | 396.6910642 | 0.085781315 | 0.1103271 | 0.2502026 | 0.5163897 | NEIL2 | | 252969 |
| ENSG00000079459 | 1674.134983 | -0.493498748 | 0.0833226 | 2.56E-10 | 2.00E-08 | FDFT1 | | 2222 |
| ENSG00000164733 | 3518.080554 | 0.010266965 | 0.058064 | 0.8483495 | 0.9375782 | CTSB | | 1508 |
| ENSG00000186523 | 50.59740104 | 0.083939269 | 0.1621998 | 0.1040579 | 0.3098146 | FAM86B1 | | 85002 |
| ENSG00000255495 | 19.46254334 | -0.001291553 | 0.1331181 | 0.9636275 | NA | NA | NA | |
| ENSG00000154359 | 76.44159913 | 0.058057969 | 0.1389061 | 0.2914384 | 0.5613232 | LONRF1 | | 91694 |
| ENSG00000164741 | 2449.637942 | 0.088967043 | 0.0744151 | 0.1575763 | 0.3975565 | DLC1 | | 10395 |
| ENSG00000164743 | 23.90648135 | -0.001404349 | 0.1323382 | 0.9617093 | NA | C8orf48 | | 157773 |
| ENSG00000104723 | 885.2870449 | -0.133619692 | 0.0944118 | 0.0691219 | 0.2389503 | TUSC3 | | 7991 |
| ENSG00000038945 | 15.40032496 | -0.004567188 | 0.1343801 | 0.8523554 | NA | MSR1 | | 4481 |
| ENSG00000104219 | 374.9025019 | -0.358567846 | 0.1527101 | 0.0016989 | 0.016832 | ZDHHC2 | | 51201 |
| ENSG00000198791 | 1417.884252 | -0.234460058 | 0.0849843 | 0.0012523 | 0.0133952 | CNOT7 | | 29883 |
| ENSG00000155975 | 532.8459575 | 0.078728376 | 0.1056456 | 0.2839454 | 0.5531631 | VPS37A | | 137492 |
| ENSG00000003989 | 11.48165444 | 0.018824911 | 0.1365121 | 0.4220955 | NA | SLC7A2 | | 6542 |
| ENSG00000078674 | 1483.255234 | -0.10735175 | 0.0780108 | 0.0992235 | 0.3005468 | PCM1 | | 5108 |
| ENSG00000104763 | 991.7783827 | -0.017953943 | 0.0810806 | 0.7825739 | 0.9096561 | ASAH1 | | 427 |
| ENSG00000171428 | 20.27398399 | 0.023529357 | 0.1359803 | 0.4422949 | NA | NAT1 | | 9 |
| ENSG00000156011 | 1301.056647 | -0.057011834 | 0.0787659 | 0.3781763 | 0.6395653 | PSD3 | | 23362 |
| ENSG00000104611 | 920.0298963 | -0.0317874 | 0.0817213 | 0.6314716 | 0.8250805 | SH2D4A | | 63898 |
| ENSG00000147408 | 77.01164949 | 0.0144591 | 0.1251432 | 0.7822209 | 0.9095461 | CSGALNAC | | 55790 |
| ENSG00000104613 | 1036.328116 | -0.111202572 | 0.0883539 | 0.1126416 | 0.325537 | INTS10 | | 55174 |
| ENSG00000147416 | 1549.378989 | -0.000975379 | 0.070362 | 0.9863238 | 0.9944112 | ATP6V1B2 | | 526 |
| ENSG00000130227 | 1428.253101 | -0.082160537 | 0.0752666 | 0.1944602 | 0.4466162 | XPO7 | | 23039 |

| | | | | | | | | |
|---|---|---|---|---|---|---|---|---|
| ENSG00000158856 | 102.8240876 | 0.00219371 | 0.1219148 | 0.9694379 | 0.9880997 | DMTN | | 2039 |
| ENSG00000158863 | 322.6633376 | 0.068157172 | 0.1147545 | 0.344746 | 0.610988 | FHIP2B | | 64760 |
| ENSG00000173566 | 50.63408713 | 0.087790567 | 0.1656264 | 0.093662 | 0.29028 | NA | NA | |
| ENSG00000168476 | 646.1381717 | -0.155868489 | 0.1078302 | 0.0505134 | 0.1935431 | REEP4 | | 80346 |
| ENSG00000168487 | 268.3845779 | 0.101924538 | 0.1317041 | 0.1849115 | 0.4352984 | BMP1 | | 649 |
| ENSG00000168495 | 615.6167179 | 0.574917196 | 0.1198534 | 1.12E-07 | 5.32E-06 | POLR3D | | 661 |
| ENSG00000104635 | 3905.247266 | 0.075335121 | 0.0601165 | 0.1624753 | 0.4047229 | SLC39A14 | | 23516 |
| ENSG00000120910 | 208.3527668 | 0.253404133 | 0.2148949 | 0.0229875 | 0.1142625 | PPP3CC | | 5533 |
| ENSG00000120896 | 1450.309901 | -0.039584393 | 0.0720042 | 0.5179635 | 0.7470898 | SORBS3 | | 10174 |
| ENSG00000254230 | 27.75142055 | 0.044162226 | 0.141627 | 0.2279991 | NA | NA | NA | |
| ENSG00000120913 | 954.4481087 | -0.079513515 | 0.0866925 | 0.245471 | 0.5110917 | PDLIM2 | | 64236 |
| ENSG00000241852 | 447.3824081 | -0.20773061 | 0.1384935 | 0.0257529 | 0.1235907 | C8orf58 | | 541565 |
| ENSG00000158941 | 2473.204408 | -0.111840541 | 0.0680415 | 0.0589096 | 0.2154423 | CCAR2 | | 57805 |
| ENSG00000253200 | 85.31378884 | 0.016532909 | 0.1245132 | 0.7580877 | 0.8962891 | NA | NA | |
| ENSG00000147439 | 385.5617874 | 0.067634527 | 0.1073216 | 0.3504611 | 0.6163779 | BIN3 | | 55909 |
| ENSG00000245025 | 13.76044333 | 0.007565354 | 0.1344572 | 0.7699109 | NA | NA | NA | |
| ENSG00000008853 | 273.9689423 | 0.029927337 | 0.1083683 | 0.6602294 | 0.8426737 | RHOBTB2 | | 23221 |
| ENSG00000120889 | 3726.716087 | 0.132353652 | 0.0624211 | 0.0177559 | 0.0952279 | TNFRSF10B | | 8795 |
| ENSG00000173535 | 14.05954795 | 0.015952051 | 0.1354908 | 0.5363011 | NA | TNFRSF10C | | 8794 |
| ENSG00000173530 | 1295.017633 | 0.239608286 | 0.0904476 | 0.0016588 | 0.0165753 | TNFRSF10D | | 8793 |
| ENSG00000104689 | 912.534704 | 0.21218294 | 0.1014915 | 0.0085361 | 0.0568152 | TNFRSF10A | | 8797 |
| ENSG00000246582 | 170.7639554 | 0.098066907 | 0.1429911 | 0.1826286 | 0.4321888 | TNFRSF10A | | 389641 |
| ENSG00000147457 | 1126.327857 | -0.014591813 | 0.0792032 | 0.82046 | 0.9260506 | CHMP7 | | 91782 |
| ENSG00000104679 | 392.9364272 | 0.006369199 | 0.0978511 | 0.9270092 | 0.9718632 | R3HCC1 | | 203069 |
| ENSG00000134013 | 3885.800703 | -0.181001186 | 0.0730814 | 0.0044657 | 0.0347066 | LOXL2 | | 4017 |
| ENSG00000253837 | 12.12512269 | -0.008808507 | 0.1348204 | 0.7153462 | NA | LOXL2-AS1 | | 100507156 |
| ENSG00000197217 | 879.1634127 | -0.064351835 | 0.0868894 | 0.339685 | 0.6068111 | ENTPD4 | | 9583 |
| ENSG00000147454 | 1564.445619 | 0.098169386 | 0.0768602 | 0.1271529 | 0.350383 | SLC25A37 | | 51312 |
| ENSG00000167034 | 279.1113806 | 0.404509206 | 0.1794255 | 0.0017703 | 0.017289 | NKX3-1 | | 4824 |
| ENSG00000159167 | 34.7241896 | 1.111375049 | 0.6058679 | 0.0017847 | NA | STC1 | | 6781 |
| ENSG00000147459 | 583.4667198 | 0.057592892 | 0.0941241 | 0.4115894 | 0.6661689 | DOCK5 | | 80005 |
| ENSG00000147437 | 18.03644218 | 0.008431766 | 0.1341792 | 0.7588941 | NA | GNRH1 | | 2796 |
| ENSG00000104756 | 838.8103872 | -0.191449047 | 0.1023486 | 0.0164164 | 0.0897235 | KCTD9 | | 54793 |
| ENSG00000184661 | 988.3475045 | -0.249367211 | 0.0988439 | 0.0021482 | 0.0199785 | CDCA2 | | 157313 |
| ENSG00000221914 | 1288.329879 | 0.007622049 | 0.0734214 | 0.9029061 | 0.9618565 | PPP2R2A | | 5520 |
| ENSG00000228451 | 12.20974365 | 0.022895345 | 0.1372606 | 0.3458787 | NA | SDAD1P1 | | 157489 |
| ENSG00000104765 | 1136.116682 | -0.137753842 | 0.0863613 | 0.0489718 | 0.1899207 | BNIP3L | | 665 |
| ENSG00000092964 | 505.5636032 | -0.11168313 | 0.1115515 | 0.1484962 | 0.3829105 | DPYSL2 | | 1808 |
| ENSG00000104228 | 471.6104334 | 0.163870547 | 0.1238054 | 0.0541213 | 0.2035386 | TRIM35 | | 23087 |
| ENSG00000120899 | 132.6165016 | 0.129601849 | 0.1775703 | 0.0926051 | 0.2876914 | PTK2B | | 2185 |
| ENSG00000120885 | 284.6596415 | 0.045532893 | 0.1119754 | 0.5097344 | 0.7413107 | CLU | | 1191 |
| ENSG00000168077 | 85.82901179 | 0.040319231 | 0.1299817 | 0.4642376 | 0.7082689 | SCARA3 | | 51435 |
| ENSG00000147419 | 616.0219287 | -0.102266405 | 0.1010527 | 0.1681615 | 0.4135356 | CCDC25 | | 55246 |
| ENSG00000171320 | 700.3858818 | 0.180360499 | 0.1178088 | 0.0334866 | 0.1480474 | ESCO2 | | 157570 |
| ENSG00000168078 | 858.0216867 | -0.049748205 | 0.086779 | 0.4624765 | 0.7068393 | PBK | | 55872 |
| ENSG00000134014 | 394.434915 | -0.046504686 | 0.105025 | 0.506437 | 0.7389994 | ELP3 | | 55140 |
| ENSG00000186918 | 173.9911192 | 0.072524759 | 0.1299326 | 0.2927341 | 0.5621971 | ZNF395 | | 55893 |
| ENSG00000214050 | 19.51820722 | 0.007208389 | 0.1334213 | 0.8120756 | NA | FBXO16 | | 157574 |
| ENSG00000104290 | 197.6991289 | 0.028530998 | 0.1152305 | 0.6604349 | 0.8426737 | FZD3 | | 7976 |
| ENSG00000012232 | 779.6506377 | -0.326752828 | 0.108226 | 0.0003085 | 0.0044267 | EXTL3 | | 2137 |
| ENSG00000104299 | 269.0312649 | -0.068523404 | 0.1164064 | 0.3408163 | 0.6080762 | INTS9 | | 55756 |
| ENSG00000147421 | 183.4884002 | 0.015933344 | 0.1154304 | 0.8008791 | 0.9169162 | HMBOX1 | | 79618 |
| ENSG00000259366 | 23.39410823 | -0.011529322 | 0.133434 | 0.7133781 | NA | NA | NA | |
| ENSG00000197892 | 437.4348489 | 0.088381116 | 0.1103371 | 0.2437256 | 0.5095683 | KIF13B | | 23303 |
| ENSG00000133872 | 1212.118102 | -0.075925656 | 0.0797905 | 0.2457696 | 0.5114617 | SARAF | | 51669 |
| ENSG00000104660 | 589.1814639 | -0.019420412 | 0.0897342 | 0.7735452 | 0.9041206 | LEPROTL1 | | 23484 |
| ENSG00000104671 | 171.9588554 | -0.022336061 | 0.1152004 | 0.7245624 | 0.8764227 | DCTN6 | | 10671 |
| ENSG00000254109 | 23.11586774 | 0.0135782 | 0.1335306 | 0.6763213 | NA | RBPMS-AS1 | | 100128750 |
| ENSG00000157110 | 789.4216724 | -0.30994239 | 0.1077877 | 0.0005247 | 0.0067135 | RBPMS | | 11030 |
| ENSG00000197265 | 463.6025776 | -0.006317934 | 0.0938642 | 0.924741 | 0.9709924 | GTF2E2 | | 2961 |
| ENSG00000104687 | 870.7309888 | -0.082482411 | 0.0874954 | 0.2293734 | 0.4903399 | GSR | | 2936 |
| ENSG00000104691 | 100.6616464 | -0.008730251 | 0.1216461 | 0.8743014 | 0.9491371 | UBXN8 | | 7993 |
| ENSG00000104695 | 1621.951399 | -0.095387115 | 0.0747223 | 0.129586 | 0.3540805 | PPP2CB | | 5516 |
| ENSG00000172733 | 11.60908556 | -0.009132884 | 0.1350704 | 0.6908777 | NA | PURG | | 29942 |
| ENSG00000165392 | 410.1905179 | -0.003217891 | 0.0982458 | 0.9609062 | 0.983972 | WRN | | 7486 |
| ENSG00000157168 | 1813.66405 | 0.318326849 | 0.0876781 | 3.91E-05 | 0.0008036 | NRG1 | | 3084 |
| ENSG00000272327 | 454.8861825 | 0.069852793 | 0.1026273 | 0.3354776 | 0.6029717 | NA | NA | |
| ENSG00000272338 | 8.928862804 | -0.036877314 | 0.1425653 | 0.0754296 | NA | NA | NA | |
| ENSG00000172728 | 139.8041998 | -0.509679408 | 0.2747662 | 0.0028673 | 0.0248832 | FUT10 | | 84750 |
| ENSG00000129696 | 147.6280588 | 0.018593796 | 0.11769 | 0.7635398 | 0.8982943 | TTI2 | | 80185 |

| | | | | | | | | |
|---|---|---|---|---|---|---|---|---|
| ENSG00000198042 | 714.6881477 | 0.030750576 | 0.0874701 | 0.6496396 | 0.8369295 | MAK16 | | 84549 |
| ENSG00000133874 | 21.54810863 | -0.0215364 | 0.135422 | 0.4826724 | NA | RNF122 | | 79845 |
| ENSG00000183779 | 114.3983758 | -0.041789577 | 0.1270382 | 0.4770946 | 0.7163677 | ZNF703 | | 80139 |
| ENSG00000147475 | 583.7560244 | -0.074613507 | 0.0998694 | 0.2994392 | 0.5693326 | ERLIN2 | | 11160 |
| ENSG00000147471 | 633.0035453 | -0.27440253 | 0.1211083 | 0.0033836 | 0.027991 | PLPBP | | 11212 |
| ENSG00000020181 | 25.13968216 | 0.03790306 | 0.1398289 | 0.2605263 | NA | ADGRA2 | | 25960 |
| ENSG00000104221 | 229.8266395 | 0.543520729 | 0.2011582 | 0.0003942 | 0.0053727 | BRF2 | | 55290 |
| ENSG00000156675 | 500.9517433 | -0.15516832 | 0.1193467 | 0.0614329 | 0.2212758 | RAB11FIP1 | | 80223 |
| ENSG00000187840 | 986.5184781 | 0.286099151 | 0.0988426 | 0.0005786 | 0.0071824 | EIF4EBP1 | | 1978 |
| ENSG00000129691 | 581.8558039 | 0.082220792 | 0.0980566 | 0.2556274 | 0.5226648 | ASH2L | | 9070 |
| ENSG00000253356 | 10.21548588 | -0.000859053 | 0.1347469 | 0.967011 | NA | NA | NA | |
| ENSG00000175324 | 580.0060515 | 0.038343045 | 0.0920581 | 0.6244259 | 0.8202204 | LSM1 | | 27257 |
| ENSG00000156735 | 451.7565911 | -0.086885243 | 0.1065462 | 0.2409256 | 0.506323 | BAG4 | | 9530 |
| ENSG00000085788 | 1273.707656 | -0.14084035 | 0.0840029 | 0.041029 | 0.1703126 | DDHD2 | | 23259 |
| ENSG00000147535 | 237.8036845 | 0.077630906 | 0.1238229 | 0.284048 | 0.5531785 | PLPP5 | | 84513 |
| ENSG00000147548 | 1343.368117 | 0.052786496 | 0.0752978 | 0.4032648 | 0.6603911 | NSD3 | | 54904 |
| ENSG00000255487 | 8.69616624 | -0.008787828 | 0.1355894 | 0.6559906 | NA | NA | NA | |
| ENSG00000165046 | 20.55045542 | 0.062656296 | 0.1541845 | 0.0579797 | NA | LETM2 | | 137994 |
| ENSG00000077782 | 726.8709019 | 0.004589945 | 0.0842468 | 0.9457124 | 0.9784014 | FGFR1 | | 2260 |
| ENSG00000147526 | 1286.585715 | 0.075965036 | 0.0782666 | 0.2416812 | 0.5072918 | TACC1 | | 6867 |
| ENSG00000169499 | 90.63121701 | 0.020360117 | 0.124506 | 0.7092969 | 0.8682712 | PLEKHA2 | | 59339 |
| ENSG00000169490 | 699.4830621 | -0.045442404 | 0.0883371 | 0.5025259 | 0.7367899 | TM2D2 | | 83877 |
| ENSG00000168615 | 4718.191124 | -0.009025056 | 0.0560552 | 0.8592252 | 0.9433297 | ADAM9 | | 8754 |
| ENSG00000197140 | 16.294372 | -0.011770395 | 0.1345917 | 0.6601544 | NA | ADAM32 | | 203102 |
| ENSG00000147533 | 575.603506 | 0.012370745 | 0.0897584 | 0.8560608 | 0.9418544 | GOLGA7 | | 51125 |
| ENSG00000147536 | 1056.002164 | 0.042809952 | 0.0841823 | 0.5220211 | 0.7495672 | GINS4 | | 84296 |
| ENSG00000253174 | 10.58353377 | 0.015513939 | 0.1359979 | 0.4975703 | NA | NA | NA | |
| ENSG00000158669 | 1491.079564 | -0.016387135 | 0.0721042 | 0.7885051 | 0.9116361 | GPAT4 | | 137964 |
| ENSG00000264578 | 8.682999093 | 0.012050293 | 0.1358171 | 0.5642959 | NA | NA | NA | |
| ENSG00000029534 | 106.1859575 | 0.020831409 | 0.1221033 | 0.7179559 | 0.8722654 | ANK1 | | 286 |
| ENSG00000083168 | 1005.366872 | -0.076366231 | 0.0844536 | 0.2555974 | 0.5226648 | KAT6A | | 7994 |
| ENSG00000070718 | 544.8469111 | 0.024744625 | 0.0918367 | 0.7180864 | 0.8722654 | AP3M2 | | 10947 |
| ENSG00000104368 | 79.61723177 | 0.413927998 | 0.4547624 | 0.0102949 | 0.0646879 | PLAT | | 5327 |
| ENSG00000104365 | 704.3529142 | -0.091968613 | 0.0943281 | 0.1991749 | 0.4526385 | IKBKB | | 3551 |
| ENSG00000070501 | 298.4067992 | -0.000668533 | 0.1036386 | 0.9905257 | 0.9958969 | POLB | | 5423 |
| ENSG00000078668 | 2140.840626 | -0.225043168 | 0.0754888 | 0.0006878 | 0.0082824 | VDAC3 | | 7419 |
| ENSG00000168575 | 512.6709991 | 0.069504689 | 0.0999267 | 0.3342558 | 0.6017943 | SLC20A2 | | 6575 |
| ENSG00000254165 | 8.609217297 | -0.015397733 | 0.1363931 | 0.4483157 | NA | NA | NA | |
| ENSG00000176209 | 58.84816856 | 0.014446995 | 0.1281727 | 0.7583093 | 0.8963508 | SMIM19 | | 114926 |
| ENSG00000131931 | 250.9380618 | 0.201628484 | 0.1757014 | 0.0395573 | 0.1665175 | THAP1 | | 55145 |
| ENSG00000120925 | 211.0228265 | 0.119187992 | 0.1461726 | 0.134214 | 0.3608746 | RNF170 | | 81790 |
| ENSG00000168172 | 992.5540006 | -0.079286636 | 0.0885991 | 0.2519329 | 0.5184753 | HOOK3 | | 84376 |
| ENSG00000168522 | 586.0921571 | 0.087930968 | 0.0992019 | 0.2280009 | 0.4890685 | FNTA | | 2339 |
| ENSG00000185900 | 201.8000371 | -0.152928548 | 0.1691319 | 0.0777365 | 0.2571623 | POMK | | 84197 |
| ENSG00000165102 | 297.3898992 | 0.025118124 | 0.1048492 | 0.7140305 | 0.8706253 | HGSNAT | | 138050 |
| ENSG00000164808 | 581.0093502 | -0.154427017 | 0.1120983 | 0.0563901 | 0.2092051 | SPIDR | | 23514 |
| ENSG00000221869 | 148.2492334 | -0.456924717 | 0.2679398 | 0.0041897 | 0.0330899 | CEBPD | | 1052 |
| ENSG00000253729 | 12497.3306 | -0.057044907 | 0.052107 | 0.2400724 | 0.5054546 | PRKDC | | 5591 |
| ENSG00000104738 | 4999.059866 | 0.056834453 | 0.0569907 | 0.2716341 | 0.5394882 | MCM4 | | 4173 |
| ENSG00000169139 | 842.9322899 | -0.09766768 | 0.0918282 | 0.1688809 | 0.4145971 | UBE2V2 | | 7336 |
| ENSG00000168300 | 173.896646 | -0.025388141 | 0.1162804 | 0.6330473 | 0.8252838 | PCMTD1 | | 115294 |
| ENSG00000228801 | 11.57866902 | 0.0007922 | 0.1346683 | 0.9749363 | NA | NA | NA | |
| ENSG00000023287 | 863.0791928 | 0.001279159 | 0.0825497 | 0.9853567 | 0.9940394 | RB1CC1 | | 9821 |
| ENSG00000047249 | 595.849156 | 0.103117039 | 0.1023172 | 0.1672379 | 0.4122331 | ATP6V1H | | 51606 |
| ENSG00000147509 | 403.3605158 | -0.040037765 | 0.1015287 | 0.5658372 | 0.782102 | RGS20 | | 8601 |
| ENSG00000187735 | 2477.821498 | -0.075979155 | 0.0654384 | 0.1852267 | 0.435663 | TCEA1 | | 6917 |
| ENSG00000120992 | 1851.946997 | -0.088398568 | 0.075125 | 0.1619645 | 0.4039797 | LYPLA1 | | 10434 |
| ENSG00000137547 | 1161.441549 | 0.085933032 | 0.0841908 | 0.2042314 | 0.4594559 | MRPL15 | | 29088 |
| ENSG00000167904 | 177.9859859 | 0.022630011 | 0.1161829 | 0.7206342 | 0.8733776 | TMEM68 | | 137695 |
| ENSG00000137574 | 578.4578846 | -0.07169697 | 0.0974346 | 0.3145849 | 0.583373 | TGS1 | | 96764 |
| ENSG00000254087 | 386.0530597 | -0.002391402 | 0.1003561 | 0.9705685 | 0.9882505 | LYN | | 4067 |
| ENSG00000008988 | 5460.33585 | -0.146288663 | 0.0586707 | 0.0059107 | 0.0429902 | RPS20 | | 6224 |
| ENSG00000181690 | 33.92878935 | 0.001172428 | 0.1308958 | 0.9771079 | NA | PLAG1 | | 5324 |
| ENSG00000170791 | 488.8993597 | 0.007607105 | 0.0932302 | 0.9125356 | 0.9655189 | CHCHD7 | | 79145 |
| ENSG00000104331 | 1768.664535 | -0.078737794 | 0.0719142 | 0.1992497 | 0.4527204 | BPNT2 | | 54928 |
| ENSG00000215114 | 439.9494805 | -0.104407449 | 0.1116794 | 0.1717455 | 0.4179776 | UBXN2B | | 137886 |
| ENSG00000137575 | 2475.518135 | -0.011131872 | 0.0664889 | 0.8794432 | 0.9513156 | SDCBP | | 6386 |
| ENSG00000035681 | 556.5444497 | -0.079878269 | 0.0993814 | 0.2687139 | 0.5365697 | NSMAF | | 8439 |
| ENSG00000104388 | 1246.114142 | -0.166470393 | 0.0891687 | 0.0215288 | 0.1092433 | RAB2A | | 5862 |
| ENSG00000171316 | 657.5465597 | -0.065495671 | 0.0959966 | 0.3554769 | 0.6192724 | CHD7 | | 55636 |

| | | | | | | | | |
|---|---|---|---|---|---|---|---|---|
| ENSG00000198363 | 2299.446917 | 0.159838025 | 0.0725591 | 0.0110307 | 0.0681385 | ASPH | | 444 |
| ENSG00000254285 | 23.36114048 | 0.008359544 | 0.1328499 | 0.7990471 | NA | KRT8P3 | | 728638 |
| ENSG00000137563 | 977.9198541 | -0.126556642 | 0.0908184 | 0.0774166 | 0.2565722 | GGH | | 8836 |
| ENSG00000270673 | 12.95778277 | 0.040603544 | 0.1433253 | 0.1057171 | NA | YTHDF3-DT | | 101410533 |
| ENSG00000185728 | 1376.367947 | 0.006763481 | 0.0718695 | 0.9129269 | 0.9656034 | YTHDF3 | | 253943 |
| ENSG00000261542 | 8.625867107 | 0.016053011 | 0.1364463 | 0.4402222 | NA | NA | NA | |
| ENSG00000104442 | 726.552965 | 0.024440343 | 0.0851498 | 0.7151737 | 0.8711099 | ARMC1 | | 55156 |
| ENSG00000066855 | 854.6823438 | -0.037865333 | 0.0864423 | 0.5734967 | 0.7878413 | MTFR1 | | 9650 |
| ENSG00000205268 | 275.3176926 | 0.002993352 | 0.1048473 | 0.9656527 | 0.9863288 | PDE7A | | 5150 |
| ENSG00000179041 | 867.6055512 | 0.05089023 | 0.0863948 | 0.4533496 | 0.6987417 | RRS1 | | 23212 |
| ENSG00000147576 | 9.403462881 | 0.030342575 | 0.1403525 | 0.1148543 | NA | ADHFE1 | | 137872 |
| ENSG00000169085 | 21.14122922 | 0.055879361 | 0.1494169 | 0.0932595 | NA | VXN | | 254778 |
| ENSG00000185697 | 874.226603 | 0.494314516 | 0.1143238 | 1.20E-06 | 3.96E-05 | MYBL1 | | 4603 |
| ENSG00000175073 | 589.6019864 | 0.140018091 | 0.1147991 | 0.0827717 | 0.2683476 | VCPIP1 | | 80124 |
| ENSG00000213865 | 34.68501639 | -0.013838218 | 0.1318937 | 0.7112338 | NA | NA | NA | |
| ENSG00000104205 | 64.97504745 | 0.016687824 | 0.1271126 | 0.7363656 | 0.8842898 | SGK3 | | 23678 |
| ENSG00000178460 | 22.62420944 | 0.023857612 | 0.1355626 | 0.4615761 | NA | MCMDC2 | | 157777 |
| ENSG00000245910 | 852.5223186 | 0.38815009 | 0.1112841 | 4.80E-05 | 0.0009503 | NA | NA | |
| ENSG00000121022 | 776.9782398 | -0.001504484 | 0.0831234 | 0.9809423 | 0.9930139 | COPS5 | | 10987 |
| ENSG00000104218 | 434.0592642 | 0.09085999 | 0.1096565 | 0.2263554 | 0.4874737 | CSPP1 | | 79848 |
| ENSG00000066777 | 1104.710014 | -0.201229769 | 0.0948492 | 0.0087946 | 0.0583029 | ARFGEF1 | | 10565 |
| ENSG00000165084 | 8.815736264 | 0.00812965 | 0.1356277 | 0.675082 | NA | C8orf34 | | 116328 |
| ENSG00000140396 | 917.4087708 | -0.161627014 | 0.099301 | 0.0354172 | 0.1542648 | NCOA2 | | 10499 |
| ENSG00000067167 | 2335.55497 | -0.197368276 | 0.0730776 | 0.0020506 | 0.0192081 | TRAM1 | | 23471 |
| ENSG00000213002 | 22.61406486 | -0.019438945 | 0.1347942 | 0.5343272 | NA | NA | NA | |
| ENSG00000147592 | 287.1571159 | 0.053325212 | 0.1123541 | 0.4429945 | 0.6917073 | LACTB2 | | 51110 |
| ENSG00000221947 | 39.79667446 | 0.061478931 | 0.1490832 | 0.1502711 | NA | XKR9 | | 389668 |
| ENSG00000147601 | 646.2411759 | -0.143298241 | 0.1072625 | 0.0683926 | 0.2369198 | TERF1 | | 7013 |
| ENSG00000253636 | 10.20378683 | 0.006475328 | 0.1352492 | 0.7588269 | NA | NA | NA | |
| ENSG00000147604 | 21814.31667 | -0.246909695 | 0.0601252 | 8.63E-06 | 0.0002224 | RPL7 | | 6129 |
| ENSG00000121039 | 300.7720595 | 0.118831571 | 0.1313792 | 0.138851 | 0.3676153 | RDH10 | | 157506 |
| ENSG00000040341 | 522.2722566 | -0.05065813 | 0.0965245 | 0.4691873 | 0.7108806 | STAU2 | | 27067 |
| ENSG00000104343 | 501.927945 | 0.003255883 | 0.09309 | 0.9632925 | 0.9851357 | UBE2W | | 55284 |
| ENSG00000154582 | 1771.9956 | -0.027909977 | 0.0709469 | 0.6438024 | 0.8333617 | ELOC | | 6921 |
| ENSG00000175606 | 645.1940083 | 0.027747746 | 0.0891024 | 0.6846198 | 0.8554818 | TMEM70 | | 54968 |
| ENSG00000104381 | 361.795021 | 0.109514764 | 0.1222352 | 0.163016 | 0.4054918 | GDAP1 | | 54332 |
| ENSG00000253341 | 42.88696451 | -0.020237938 | 0.1319445 | 0.6150634 | 0.8157779 | NA | NA | |
| ENSG00000249395 | 12.10604129 | -0.014210078 | 0.1355205 | 0.5543206 | NA | CASC9 | | 101805492 |
| ENSG00000164749 | 32.65067744 | -0.00292068 | 0.1311544 | 0.9349875 | NA | HNF4G | | 3174 |
| ENSG00000091656 | 326.0582888 | -0.035261701 | 0.1047576 | 0.6093407 | 0.8124311 | ZFHX4 | | 79776 |
| ENSG00000164751 | 478.3614736 | -0.115600908 | 0.1131773 | 0.1376194 | 0.3658471 | PEX2 | | 5828 |
| ENSG00000171033 | 170.6680711 | -0.153860469 | 0.1815791 | 0.0737587 | 0.2487945 | PKIA | | 5569 |
| ENSG00000104427 | 263.8752472 | -0.004677711 | 0.1065804 | 0.9438689 | 0.9784014 | ZC2HC1A | | 51101 |
| ENSG00000104432 | 21.43201265 | -0.010069479 | 0.133361 | 0.7454609 | NA | IL7 | | 3574 |
| ENSG00000261618 | 9.782436291 | 0.013563967 | 0.1358553 | 0.5369624 | NA | NA | NA | |
| ENSG00000147586 | 317.6739503 | -0.105327881 | 0.1238459 | 0.1765751 | 0.4256732 | MRPS28 | | 28957 |
| ENSG00000272518 | 14.89212908 | -0.005562061 | 0.1342962 | 0.8266355 | NA | NA | NA | |
| ENSG00000076554 | 1267.633283 | -0.103661212 | 0.0833797 | 0.1252921 | 0.347221 | TPD52 | | 7163 |
| ENSG00000251867 | 9.894876878 | 0.032188652 | 0.1405751 | 0.1381131 | NA | NA | NA | |
| ENSG00000205189 | 474.7575633 | -0.074890401 | 0.1048937 | 0.3039246 | 0.5728743 | ZBTB10 | | 65986 |
| ENSG00000164684 | 34.18915623 | -0.052016964 | 0.1440973 | 0.2002679 | NA | ZNF704 | | 619279 |
| ENSG00000076641 | 24.7040098 | -0.00970073 | 0.1328228 | 0.7668598 | NA | PAG1 | | 55824 |
| ENSG00000254027 | 11.31171796 | -0.002207311 | 0.1345962 | 0.9227534 | NA | NA | NA | |
| ENSG00000164687 | 1781.824593 | 0.021899656 | 0.0694511 | 0.7151639 | 0.8711099 | FABP5 | | 2171 |
| ENSG00000133731 | 982.4400003 | -0.070515772 | 0.0835003 | 0.2892141 | 0.5592864 | IMPA1 | | 3612 |
| ENSG00000253598 | 10.65654204 | 0.012812452 | 0.1356972 | 0.565556 | NA | SLC10A5 | | 347051 |
| ENSG00000104231 | 561.187999 | 0.067098931 | 0.0974459 | 0.3545473 | 0.6187516 | ZFAND1 | | 79752 |
| ENSG00000164695 | 347.1247739 | 0.118388782 | 0.1292629 | 0.1394647 | 0.3687247 | CHMP4C | | 92421 |
| ENSG00000104497 | 204.9693198 | 0.125430128 | 0.1510089 | 0.1203122 | 0.3390055 | SNX16 | | 64089 |
| ENSG00000133739 | 71.50435217 | 0.015070523 | 0.1264867 | 0.7641681 | 0.8983744 | LRRCC1 | | 85444 |
| ENSG00000133740 | 59.68727092 | -0.004297233 | 0.1269036 | 0.9258485 | 0.971576 | E2F5 | | 1875 |
| ENSG00000176731 | 586.9598051 | -0.373971949 | 0.1277329 | 0.0003252 | 0.0046157 | RBIS | | 401466 |
| ENSG00000185015 | 250.1634049 | -0.001139168 | 0.1067883 | 0.9851774 | 0.9940394 | CA13 | | 377677 |
| ENSG00000104267 | 56.28799196 | 0.03237362 | 0.1323893 | 0.4897902 | 0.7266633 | CA2 | | 760 |
| ENSG00000123124 | 934.3787463 | -0.11236511 | 0.0923178 | 0.1173107 | 0.3339561 | WWP1 | | 11059 |
| ENSG00000176623 | 612.2417259 | 0.010309263 | 0.0930583 | 0.8807701 | 0.9518649 | RMDN1 | | 51115 |
| ENSG00000085719 | 2008.92271 | -0.219029698 | 0.0763043 | 0.0010214 | 0.011298 | CPNE3 | | 8895 |
| ENSG00000156103 | 48.74827406 | 0.003768163 | 0.1283802 | 0.9338157 | 0.9746159 | MMP16 | | 4325 |
| ENSG00000251136 | 49.29120919 | 0.085321575 | 0.1638839 | 0.0966374 | 0.2955472 | PARAIL | | 101929709 |
| ENSG00000104312 | 336.442317 | 0.034162293 | 0.1073951 | 0.6163432 | 0.8159003 | RIPK2 | | 8767 |

| | | | | | | | | |
|---|---|---|---|---|---|---|---|---|
| ENSG00000164823 | 543.3292149 | 0.067889725 | 0.0978235 | 0.3504515 | 0.6163779 | OSGIN2 | | 734 |
| ENSG00000104320 | 1528.68155 | -0.190986304 | 0.082032 | 0.0058886 | 0.0428834 | NBN | | 4683 |
| ENSG00000104325 | 473.3337099 | -0.143289507 | 0.1186981 | 0.0794926 | 0.2610494 | DECR1 | | 1666 |
| ENSG00000180694 | 896.4204989 | -0.04786998 | 0.0834289 | 0.4695781 | 0.7112477 | TMEM64 | | 169200 |
| ENSG00000155099 | 173.1175009 | -0.031133617 | 0.1177112 | 0.6225778 | 0.8191407 | PIP4P2 | | 55529 |
| ENSG00000253738 | 336.9681784 | -0.162829907 | 0.1482839 | 0.0660655 | 0.2322697 | OTUD6B-AS | | 100506365 |
| ENSG00000155100 | 589.1895644 | 0.019625161 | 0.0915651 | 0.7743662 | 0.9046394 | OTUD6B | | 51633 |
| ENSG00000214954 | 32.93607707 | -0.028774727 | 0.1350662 | 0.446131 | NA | LRRC69 | | 100130742 |
| ENSG00000205133 | 166.3793652 | -0.020434693 | 0.1166589 | 0.7422146 | 0.8876265 | TRIQK | | 286144 |
| ENSG00000188343 | 310.5098292 | -0.069438604 | 0.1131426 | 0.3369276 | 0.6047405 | CIBAR1 | | 137392 |
| ENSG00000271971 | 11.29168168 | 0.026038747 | 0.1383331 | 0.2591115 | NA | NA | NA | |
| ENSG00000183808 | 827.5643208 | -0.011486906 | 0.0825645 | 0.8605972 | 0.9439076 | RBM12B | | 389677 |
| ENSG00000164953 | 237.8781065 | 0.12600008 | 0.1481591 | 0.1206018 | 0.3394578 | TMEM67 | | 91147 |
| ENSG00000164951 | 791.042867 | -0.010327082 | 0.0836615 | 0.8751303 | 0.9494835 | PDP1 | | 54704 |
| ENSG00000164949 | 34.75501192 | 2.597541229 | 0.5863542 | 5.69E-07 | NA | GEM | | 2669 |
| ENSG00000197275 | 247.1715963 | 0.017585686 | 0.1079831 | 0.7936407 | 0.913934 | RAD54B | | 25788 |
| ENSG00000164944 | 1502.098262 | -0.015233542 | 0.0713487 | 0.8018163 | 0.9172909 | VIRMA | | 25962 |
| ENSG00000104413 | 34.78763564 | 0.017597722 | 0.1324342 | 0.643195 | NA | ESRP1 | | 54845 |
| ENSG00000156162 | 582.4877659 | -0.125129179 | 0.1072222 | 0.1059517 | 0.313719 | DPY19L4 | | 286148 |
| ENSG00000164941 | 950.3148673 | -0.328705052 | 0.1009016 | 0.0001397 | 0.0022762 | INTS8 | | 55656 |
| ENSG00000175305 | 915.0031589 | 0.057476867 | 0.08436 | 0.3954009 | 0.6555966 | CCNE2 | | 9134 |
| ENSG00000156170 | 333.3723769 | -0.038110182 | 0.1066215 | 0.5802703 | 0.7924836 | NDUFAF6 | | 137682 |
| ENSG00000164938 | 105.3669617 | 0.052092643 | 0.1332691 | 0.3655721 | 0.6282547 | TP53INP1 | | 94241 |
| ENSG00000175895 | 262.362117 | -0.05915046 | 0.1152841 | 0.3999206 | 0.6583091 | PLEKHF2 | | 79666 |
| ENSG00000156172 | 195.2315901 | -0.054901117 | 0.1224246 | 0.4106675 | 0.6656962 | CFAP418 | | 157657 |
| ENSG00000156467 | 2489.79459 | -0.151996992 | 0.0693556 | 0.0121676 | 0.073037 | UQCRB | | 7381 |
| ENSG00000156469 | 441.7955341 | 0.390074674 | 0.1416033 | 0.0005139 | 0.0066111 | MTERF3 | | 51001 |
| ENSG00000156471 | 2287.749412 | -0.086203036 | 0.0669239 | 0.1394806 | 0.3687247 | PTDSS1 | | 9791 |
| ENSG00000169439 | 144.9023022 | -0.26926242 | 0.2671665 | 0.0213613 | 0.108724 | SDC2 | | 6383 |
| ENSG00000104324 | 50.74789962 | -0.020248169 | 0.1302645 | 0.6469653 | 0.8359336 | CPQ | | 10404 |
| ENSG00000147649 | 2870.928094 | -0.24576096 | 0.0688477 | 7.68E-05 | 0.0014099 | MTDH | | 92140 |
| ENSG00000104341 | 4888.337469 | -0.256486386 | 0.0629703 | 9.42E-06 | 0.0002398 | LAPTM4B | | 55353 |
| ENSG00000132561 | 49.6225205 | -0.002088205 | 0.1285917 | 0.9585223 | 0.9831259 | MATN2 | | 4147 |
| ENSG00000156482 | 7856.687063 | -0.103610933 | 0.062062 | 0.0611169 | 0.2205456 | RPL30 | | 6156 |
| ENSG00000245970 | 96.39273252 | -0.00074961 | 0.1323139 | 0.9855091 | 0.9940394 | NA | NA | |
| ENSG00000207067 | 14.82222232 | -0.000554448 | 0.134233 | 0.9800068 | NA | SNORA72 | | 26775 |
| ENSG00000132541 | 278.3523731 | -0.032533331 | 0.1070858 | 0.6335375 | 0.8254975 | RIDA | | 10247 |
| ENSG00000104356 | 1084.599738 | -0.077485038 | 0.0820881 | 0.244769 | 0.5104707 | POP1 | | 10940 |
| ENSG00000104361 | 32.77303435 | -0.020343529 | 0.1334899 | 0.5731332 | NA | NIPAL2 | | 79815 |
| ENSG00000104375 | 293.6250657 | -0.015168931 | 0.1084207 | 0.8185194 | 0.9254567 | STK3 | | 6788 |
| ENSG00000164920 | 37.40701669 | 0.043691996 | 0.1394774 | 0.2916172 | NA | OSR2 | | 116039 |
| ENSG00000132549 | 1110.610666 | 0.092194465 | 0.0825484 | 0.1715117 | 0.4177334 | VPS13B | | 157680 |
| ENSG00000164919 | 1301.86037 | -0.150882597 | 0.0890368 | 0.0354462 | 0.1542648 | COX6C | | 1345 |
| ENSG00000156509 | 174.1411922 | 0.045625015 | 0.120436 | 0.4877054 | 0.7249792 | FBXO43 | | 286151 |
| ENSG00000147669 | 884.8083327 | -0.049467089 | 0.0830168 | 0.4533438 | 0.6987417 | POLR2K | | 5440 |
| ENSG00000104450 | 620.8324205 | -0.017272391 | 0.0879067 | 0.7970053 | 0.9157658 | SPAG1 | | 6674 |
| ENSG00000034677 | 678.4418958 | -0.036265905 | 0.0877108 | 0.5933783 | 0.802414 | RNF19A | | 25897 |
| ENSG00000186106 | 112.1344935 | 0.102316046 | 0.1589097 | 0.1406563 | 0.3701299 | ANKRD46 | | 157567 |
| ENSG00000070756 | 17436.8768 | -0.038196558 | 0.0480782 | 0.395065 | 0.6553186 | PABPC1 | | 26986 |
| ENSG00000264991 | 11.28449791 | -0.008901539 | 0.135071 | 0.697565 | NA | NA | NA | |
| ENSG00000212994 | 23.86106565 | 0.034928929 | 0.1386206 | 0.3022263 | NA | NA | NA | |
| ENSG00000164924 | 12795.5267 | -0.311808471 | 0.0619495 | 7.53E-08 | 3.76E-06 | YWHAZ | | 7534 |
| ENSG00000120963 | 609.2744823 | -0.004131641 | 0.0887283 | 0.9487376 | 0.9797722 | ZNF706 | | 51123 |
| ENSG00000048392 | 869.9554706 | -0.112126503 | 0.1013627 | 0.1347376 | 0.3614858 | RRM2B | | 50484 |
| ENSG00000246263 | 15.44264776 | -0.021445694 | 0.1367267 | 0.3924256 | NA | NA | NA | |
| ENSG00000104517 | 3270.399831 | -0.160073327 | 0.0683462 | 0.0077812 | 0.0529676 | UBR5 | | 51366 |
| ENSG00000155090 | 1109.513677 | 0.145470415 | 0.0956616 | 0.051153 | 0.1950344 | KLF10 | | 7071 |
| ENSG00000253669 | 37.41546013 | -0.064227782 | 0.1509679 | 0.1337309 | NA | NA | NA | |
| ENSG00000155096 | 2798.077098 | 0.042620343 | 0.064446 | 0.4574111 | 0.7017657 | AZIN1 | | 51582 |
| ENSG00000253320 | 17.45857522 | -0.012943089 | 0.1344689 | 0.6439047 | NA | NA | NA | |
| ENSG00000155097 | 2092.43059 | 0.104292654 | 0.0696201 | 0.0835446 | 0.2700934 | ATP6V1C1 | | 528 |
| ENSG00000247081 | 27.66358586 | -0.010839789 | 0.1323389 | 0.755215 | NA | LOC105369 | | 105369147 |
| ENSG00000164930 | 1570.501013 | -0.150346928 | 0.0791469 | 0.0245419 | 0.1192982 | FZD6 | | 8323 |
| ENSG00000164933 | 976.3728269 | -0.017304962 | 0.0789684 | 0.7876353 | 0.9116145 | SLC25A32 | | 81034 |
| ENSG00000164934 | 1590.088649 | 0.065353854 | 0.0741228 | 0.2961673 | 0.5661152 | DCAF13 | | 25879 |
| ENSG00000271830 | 11.98180667 | -0.002735826 | 0.1344583 | 0.9078591 | NA | NA | NA | |
| ENSG00000253477 | 28.6704374 | 0.006223436 | 0.1319979 | 0.8603254 | NA | NA | NA | |
| ENSG00000176406 | 177.1378393 | 0.036958248 | 0.1173617 | 0.5708256 | 0.7862989 | RIMS2 | | 9699 |
| ENSG00000147650 | 705.6137961 | -0.0175201 | 0.0872153 | 0.7937773 | 0.913934 | LRP12 | | 29967 |
| ENSG00000169946 | 159.575622 | 0.51673987 | 0.2439991 | 0.0017172 | 0.016956 | ZFPM2 | | 23414 |

| | | | | | | | | |
|---|---|---|---|---|---|---|---|---|
| ENSG00000251003 | 120.1741798 | 0.018485109 | 0.1207418 | 0.752878 | 0.8932431 | NA | NA | |
| ENSG00000254615 | 60.42163829 | 0.027338005 | 0.1304134 | 0.5657411 | 0.782102 | NA | NA | |
| ENSG00000164830 | 489.6243029 | 0.058836188 | 0.0983019 | 0.4081265 | 0.6640358 | OXR1 | | 55074 |
| ENSG00000104408 | 4130.129342 | -0.236927264 | 0.0639543 | 4.84E-05 | 0.0009572 | EIF3E | | 3646 |
| ENSG00000104412 | 498.7663418 | -0.146863534 | 0.1189708 | 0.0736902 | 0.2486353 | EMC2 | | 9694 |
| ENSG00000164841 | 26.37810883 | 0.010369627 | 0.1328478 | 0.757227 | NA | TMEM74 | | 157753 |
| ENSG00000120526 | 964.1303299 | 0.044704474 | 0.0809283 | 0.491598 | 0.7279863 | NUDCD1 | | 84955 |
| ENSG00000120533 | 1580.293049 | -0.092301295 | 0.0753448 | 0.1455715 | 0.3780659 | ENY2 | | 56943 |
| ENSG00000147654 | 260.096357 | 0.032695583 | 0.1087904 | 0.6320388 | 0.8251856 | EBAG9 | | 9166 |
| ENSG00000147642 | 76.94716963 | 0.086798823 | 0.1569076 | 0.1471171 | 0.3805302 | SYBU | | 55638 |
| ENSG00000104447 | 88.45804024 | 0.00487176 | 0.1238194 | 0.9251526 | 0.9710568 | TRPS1 | | 7227 |
| ENSG00000147677 | 3628.13886 | 0.000356841 | 0.0581097 | 0.9957815 | 0.9983455 | EIF3H | | 8667 |
| ENSG00000147679 | 630.4686858 | 0.212439018 | 0.1173112 | 0.0150084 | 0.0842043 | UTP23 | | 84294 |
| ENSG00000164754 | 5697.279898 | -0.284949669 | 0.0592098 | 2.64E-07 | 1.08E-05 | RAD21 | | 5885 |
| ENSG00000164758 | 180.9464379 | -0.048437235 | 0.1195764 | 0.4649846 | 0.7086768 | MED30 | | 90390 |
| ENSG00000182197 | 905.3142073 | 0.188853422 | 0.0983634 | 0.0153713 | 0.0856234 | EXT1 | | 2131 |
| ENSG00000177570 | 197.931205 | -0.035905257 | 0.1159151 | 0.5812381 | 0.7932518 | SAMD12 | | 401474 |
| ENSG00000147676 | 13.00173899 | 0.036125488 | 0.1414544 | 0.1455999 | NA | MAL2 | | 114569 |
| ENSG00000064313 | 1995.034865 | -0.067509346 | 0.0678834 | 0.2517016 | 0.5184493 | TAF2 | | 6873 |
| ENSG00000136982 | 934.8624409 | 0.005723715 | 0.0802362 | 0.9306029 | 0.972984 | DSCC1 | | 79075 |
| ENSG00000155792 | 110.0827546 | -0.026862239 | 0.123227 | 0.6409642 | 0.8315061 | DEPTOR | | 64798 |
| ENSG00000172172 | 1128.45855 | -0.092658093 | 0.0825498 | 0.1668889 | 0.4122285 | MRPL13 | | 28998 |
| ENSG00000172167 | 534.7537813 | -0.084622247 | 0.1037958 | 0.2477021 | 0.5140182 | MTBP | | 27085 |
| ENSG00000178764 | 128.1505546 | 0.228531464 | 0.2794432 | 0.0291534 | 0.135728 | ZHX2 | | 22882 |
| ENSG00000136986 | 1863.591323 | 0.024336789 | 0.0675107 | 0.6805154 | 0.8538239 | DERL1 | | 79139 |
| ENSG00000156787 | 427.6777554 | -0.037806955 | 0.1015086 | 0.5858889 | 0.7968554 | TBC1D31 | | 93594 |
| ENSG00000147689 | 27.28810373 | 0.096128617 | 0.18385 | 0.0206623 | NA | FAM83A | | 84985 |
| ENSG00000189376 | 84.71510346 | -0.020022485 | 0.1248067 | 0.7080752 | 0.8678177 | C8orf76 | | 84933 |
| ENSG00000259305 | 9.016184277 | -0.000594043 | 0.1351288 | 0.9734326 | NA | ZHX1-C8orf7 | | 100533106 |
| ENSG00000165156 | 1053.900439 | -0.008940572 | 0.0767771 | 0.8872881 | 0.9545517 | ZHX1 | | 11244 |
| ENSG00000156802 | 7328.177418 | 0.182586021 | 0.0606349 | 0.0008807 | 0.0100167 | ATAD2 | | 29028 |
| ENSG00000156795 | 274.1744485 | 0.011841825 | 0.1051333 | 0.8635633 | 0.9453914 | NTAQ1 | | 55093 |
| ENSG00000156804 | 19.18685405 | 0.01424468 | 0.1342233 | 0.6370469 | NA | FBXO32 | | 114907 |
| ENSG00000176853 | 2938.703791 | 0.008110135 | 0.0608246 | 0.8807157 | 0.9518649 | FAM91A1 | | 157769 |
| ENSG00000271975 | 68.87935906 | -0.048407134 | 0.136069 | 0.3476793 | 0.6138516 | NA | NA | |
| ENSG00000164983 | 542.4918869 | -0.063211883 | 0.0967726 | 0.3713302 | 0.6334284 | TMEM65 | | 157378 |
| ENSG00000183665 | 426.0732606 | -0.03605107 | 0.0987005 | 0.6029524 | 0.8084868 | TRMT12 | | 55039 |
| ENSG00000245149 | 40.61391594 | -0.033166815 | 0.1349846 | 0.4260169 | NA | RNF139-DT | | 101927612 |
| ENSG00000170881 | 654.7640452 | -0.225157943 | 0.1200738 | 0.0116482 | 0.070829 | RNF139 | | 11236 |
| ENSG00000147687 | 716.1957274 | 0.023750103 | 0.0868203 | 0.7248077 | 0.876447 | TATDN1 | | 83940 |
| ENSG00000253106 | 23.22838607 | -0.029049675 | 0.1369228 | 0.3717999 | NA | NA | NA | |
| ENSG00000147684 | 3425.229072 | -0.025859286 | 0.0597294 | 0.6302233 | 0.8239333 | NDUFB9 | | 4715 |
| ENSG00000170873 | 37.70342942 | 0.075837021 | 0.1593406 | 0.0912562 | NA | MTSS1 | | 9788 |
| ENSG00000104549 | 1210.825443 | -0.321903999 | 0.0922889 | 6.39E-05 | 0.0012101 | SQLE | | 6713 |
| ENSG00000164961 | 1449.490113 | -0.124445394 | 0.083265 | 0.0681242 | 0.2363406 | WASHC5 | | 9897 |
| ENSG00000253167 | 8.574268228 | -0.002569324 | 0.1351093 | 0.8979065 | NA | WASHC5-AS | | 106479020 |
| ENSG00000156831 | 657.3928857 | 0.004173842 | 0.0874743 | 0.9514111 | 0.9805393 | NSMCE2 | | 286053 |
| ENSG00000173334 | 439.1383333 | 0.245890113 | 0.1556798 | 0.0159498 | 0.0880899 | TRIB1 | | 10221 |
| ENSG00000168672 | 4108.834402 | 0.043637227 | 0.1094345 | 0.5345615 | 0.7591377 | LRATD2 | | 157638 |
| ENSG00000254286 | 14.76335331 | -0.011796837 | 0.134867 | 0.6439563 | NA | NA | NA | |
| ENSG00000254166 | 199.4801072 | 0.087611392 | 0.1328949 | 0.2288576 | 0.4902363 | PCAT2 | | 103164619 |
| ENSG00000247844 | 591.1362893 | 0.267803015 | 0.1291944 | 0.0055056 | 0.0407549 | NA | NA | |
| ENSG00000246228 | 52.26848015 | -0.022907654 | 0.130628 | 0.6078812 | 0.811968 | CASC8 | | 727677 |
| ENSG00000136997 | 2219.65876 | 0.613422128 | 0.0736649 | 6.79E-18 | 1.53E-15 | MYC | | 4609 |
| ENSG00000249859 | 726.1466256 | 0.380415434 | 0.1142341 | 8.66E-05 | 0.0015538 | PVT1 | | 5820 |
| ENSG00000207110 | 18.16476768 | 0.052100729 | 0.1480296 | 0.0861531 | NA | RNVU1-32 | | 106481624 |
| ENSG00000153310 | 1948.281451 | -0.258906982 | 0.0775416 | 0.0001615 | 0.0025743 | CYRIB | | 51571 |
| ENSG00000153317 | 3196.656003 | -0.007388792 | 0.0597244 | 0.8899156 | 0.955169 | ASAP1 | | 50807 |
| ENSG00000132294 | 1847.751907 | -0.096243786 | 0.074419 | 0.1259918 | 0.3483265 | EFR3A | | 23167 |
| ENSG00000129295 | 66.09909174 | 0.130250881 | 0.210448 | 0.0452743 | 0.1812991 | DNAAF11 | | 23639 |
| ENSG00000129292 | 1287.810398 | -0.050250435 | 0.0776978 | 0.4331471 | 0.6844685 | PHF20L1 | | 51105 |
| ENSG00000223697 | 10.43440709 | -0.005496909 | 0.1348994 | 0.8050109 | NA | NA | NA | |
| ENSG00000104419 | 479.1529406 | 0.379959815 | 0.1380053 | 0.0005372 | 0.0067985 | NDRG1 | | 10397 |
| ENSG00000008513 | 1231.983691 | -0.02134223 | 0.0824222 | 0.7450516 | 0.8893165 | ST3GAL1 | | 6482 |
| ENSG00000261220 | 26.51284456 | 0.061776042 | 0.151864 | 0.0974136 | NA | NA | NA | |
| ENSG00000066827 | 266.5943668 | -0.056033779 | 0.1138216 | 0.4237423 | 0.6758239 | ZFAT | | 57623 |
| ENSG00000259820 | 95.44871998 | 0.105019561 | 0.1680836 | 0.1132193 | 0.3266274 | NA | NA | |
| ENSG00000131773 | 234.7673287 | 0.155259328 | 0.1611195 | 0.0767566 | 0.255149 | KHDRBS3 | | 10656 |
| ENSG00000167632 | 277.4519244 | -0.022017045 | 0.1057255 | 0.7638693 | 0.8982943 | TRAPPC9 | | 83696 |
| ENSG00000259891 | 32.80841889 | 0.044558586 | 0.1409761 | 0.2521517 | NA | NA | NA | |

| | | | | | | | | |
|---|---|---|---|---|---|---|---|---|
| ENSG00000104472 | 1383.479789 | -0.071107214 | 0.0772666 | 0.2682732 | 0.5361059 | CHRAC1 | | 54108 |
| ENSG00000259758 | 2871.840848 | -0.14424717 | 0.0667748 | 0.0142049 | 0.0813367 | NA | NA | |
| ENSG00000123908 | 1816.639377 | -0.158471045 | 0.0819857 | 0.0204666 | 0.1054086 | AGO2 | | 27161 |
| ENSG00000169398 | 3717.799456 | -0.223096106 | 0.0637533 | 0.0001184 | 0.0019968 | PTK2 | | 5747 |
| ENSG00000105339 | 2572.034039 | 0.032686509 | 0.0635883 | 0.5620268 | 0.7800541 | DENND3 | | 22898 |
| ENSG00000253210 | 25.4521439 | -0.014102947 | 0.1330955 | 0.6762829 | NA | DENND3-AS | | 101927963 |
| ENSG00000022567 | 417.4575482 | -0.228273821 | 0.140074 | 0.0173513 | 0.0937034 | SLC45A4 | | 57210 |
| ENSG00000184489 | 111.9112568 | 0.769522853 | 0.2856784 | 0.0003051 | 0.0043938 | PTP4A3 | | 11156 |
| ENSG00000171045 | 119.152946 | 0.042171839 | 0.1254534 | 0.487346 | 0.7246964 | TSNARE1 | | 203062 |
| ENSG00000181790 | 76.7548344 | 0.032077751 | 0.1294542 | 0.5345932 | 0.7591377 | ADGRB1 | | 575 |
| ENSG00000198576 | 8.370623716 | 0.025866602 | 0.1387752 | 0.2039118 | NA | ARC | | 23237 |
| ENSG00000234616 | 2010.038188 | -0.102957 | 0.0703728 | 0.0895002 | 0.2823258 | JRK | | 8629 |
| ENSG00000167653 | 113.0884348 | 0.118803109 | 0.1767496 | 0.0961034 | 0.2946 | PSCA | | 8000 |
| ENSG00000160886 | 4582.148773 | 0.176836417 | 0.0653843 | 0.0024667 | 0.0221814 | LY6K | | 54742 |
| ENSG00000253741 | 100.4869145 | 0.035711562 | 0.1284916 | 0.5149898 | 0.7449955 | LNCOC1 | | 100288181 |
| ENSG00000130193 | 410.5532322 | 0.373966106 | 0.1498382 | 0.0011082 | 0.0121208 | THEM6 | | 51337 |
| ENSG00000180155 | 17.3906127 | 0.006658537 | 0.1337111 | 0.8183608 | NA | LYNX1 | | 66004 |
| ENSG00000160932 | 1454.558025 | 0.059475629 | 0.0774758 | 0.3545896 | 0.6187516 | LY6E | | 4061 |
| ENSG00000181638 | 179.3400397 | 0.07236713 | 0.1293537 | 0.2967224 | 0.566626 | ZFP41 | | 286128 |
| ENSG00000250571 | 199.0700226 | 0.175101194 | 0.1848642 | 0.0573848 | 0.2119898 | GLI4 | | 2738 |
| ENSG00000253716 | 28.8381952 | 0.073096771 | 0.1597006 | 0.0670275 | NA | MINCR | | 100507316 |
| ENSG00000185730 | 317.0114164 | 0.058549656 | 0.1109845 | 0.4112942 | 0.6661689 | ZNF696 | | 79943 |
| ENSG00000272172 | 11.594686 | -0.003655359 | 0.1346463 | 0.8734956 | NA | NA | NA | |
| ENSG00000184428 | 1045.975661 | -0.024366999 | 0.0777562 | 0.7027283 | 0.8650963 | TOP1MT | | 116447 |
| ENSG00000254389 | 24.24288027 | 0.009547863 | 0.1327328 | 0.7764615 | NA | RHPN1-AS1 | | 78998 |
| ENSG00000158106 | 358.0077318 | 0.157589685 | 0.1365969 | 0.068993 | 0.2385751 | RHPN1 | | 114822 |
| ENSG00000182759 | 10.01773839 | 0.011566769 | 0.135555 | 0.6015749 | NA | MAFA | | 389692 |
| ENSG00000014164 | 787.8371701 | 0.371641485 | 0.1114155 | 8.81E-05 | 0.0015703 | ZC3H3 | | 23144 |
| ENSG00000104518 | 1142.810381 | -0.06329941 | 0.0788566 | 0.3286077 | 0.5967766 | GSDMD | | 79792 |
| ENSG00000204839 | 237.6502387 | -0.022286218 | 0.1090888 | 0.7388084 | 0.8857662 | MROH6 | | 642475 |
| ENSG00000147813 | 1194.253006 | -0.139357042 | 0.0934553 | 0.057301 | 0.2117479 | NAPRT | | 93100 |
| ENSG00000254741 | 58.82333441 | -0.025006071 | 0.1303797 | 0.5889654 | 0.798683 | NA | NA | |
| ENSG00000104529 | 9351.329131 | -0.008176806 | 0.0511687 | 0.8687112 | 0.9471139 | EEF1D | | 1936 |
| ENSG00000179886 | 221.5096962 | 0.058596639 | 0.1197213 | 0.3966172 | 0.6565973 | TIGD5 | | 84948 |
| ENSG00000271912 | 35.99101826 | 0.022240339 | 0.1333654 | 0.56293 | NA | NA | NA | |
| ENSG00000104524 | 664.9104897 | 0.241561617 | 0.1160676 | 0.0066885 | 0.0472362 | PYCR3 | | 65263 |
| ENSG00000104522 | 1493.857181 | -0.02467134 | 0.0731564 | 0.6894116 | 0.8588425 | GFUS | | 7264 |
| ENSG00000183309 | 441.0689167 | 0.109783817 | 0.1171912 | 0.1595841 | 0.4006222 | ZNF623 | | 9831 |
| ENSG00000181135 | 404.9976325 | 0.084954189 | 0.1096462 | 0.2538306 | 0.5210248 | ZNF707 | | 286075 |
| ENSG00000181097 | 27.86247079 | 0.011116635 | 0.1327032 | 0.7453578 | NA | NA | NA | |
| ENSG00000180921 | 1348.981428 | 0.21850293 | 0.0874348 | 0.0029799 | 0.0255751 | FAM83H | | 286077 |
| ENSG00000265660 | 17.82022149 | 0.015711005 | 0.1349121 | 0.578792 | NA | MIR4664 | | 100616318 |
| ENSG00000203499 | 49.05219738 | 0.074102533 | 0.1559704 | 0.1210887 | 0.340664 | IQANK1 | | 642574 |
| ENSG00000180900 | 3957.112393 | -0.091876265 | 0.0622035 | 0.0971 | 0.2964369 | SCRIB | | 23513 |
| ENSG00000179950 | 4009.602441 | -0.090967133 | 0.059293 | 0.0880734 | 0.279248 | PUF60 | | 22827 |
| ENSG00000185189 | 531.499681 | -0.012067908 | 0.0953089 | 0.8586644 | 0.9430101 | NRBP2 | | 340371 |
| ENSG00000178209 | 28242.58724 | 0.070812063 | 0.0507644 | 0.1300426 | 0.3544317 | PLEC | | 5339 |
| ENSG00000178685 | 285.7437096 | -0.001382897 | 0.1088581 | 0.9819909 | 0.9932861 | PARP10 | | 84875 |
| ENSG00000178719 | 1818.302059 | 0.208051559 | 0.0831463 | 0.0032353 | 0.0271297 | GRINA | | 2907 |
| ENSG00000178814 | 522.4860051 | -0.061282587 | 0.1021726 | 0.3904074 | 0.6505443 | OPLAH | | 26873 |
| ENSG00000255224 | 29.55171829 | 0.01955989 | 0.1337101 | 0.5791169 | NA | NA | NA | |
| ENSG00000178896 | 745.3456611 | 0.192849095 | 0.110794 | 0.020814 | 0.1065373 | EXOSC4 | | 54512 |
| ENSG00000197858 | 1283.960627 | 0.252765578 | 0.0893706 | 0.0008914 | 0.0101294 | GPAA1 | | 8733 |
| ENSG00000179091 | 4863.975078 | -0.039958339 | 0.0573446 | 0.4421349 | 0.6912833 | CYC1 | | 1537 |
| ENSG00000179526 | 551.9065995 | 0.184858175 | 0.1237565 | 0.0336357 | 0.148544 | SHARPIN | | 81858 |
| ENSG00000179632 | 2605.329942 | -0.02884411 | 0.064214 | 0.609909 | 0.8128053 | MAF1 | | 84232 |
| ENSG00000179698 | 24.28372636 | -0.016365999 | 0.1339081 | 0.6127391 | NA | WDR97 | | 340390 |
| ENSG00000235173 | 68.25614673 | 0.00645298 | 0.1258704 | 0.8949039 | 0.9579703 | HGH1 | | 51236 |
| ENSG00000179832 | 337.113252 | 0.122114016 | 0.1274474 | 0.1303293 | 0.3547836 | MROH1 | | 727957 |
| ENSG00000204775 | 34.10929606 | -0.001289637 | 0.1307983 | 0.9708928 | NA | NA | NA | |
| ENSG00000204771 | 15.81156416 | -0.000932801 | 0.1338486 | 0.9703121 | NA | NA | NA | |
| ENSG00000170727 | 1079.064302 | -0.016985268 | 0.0770376 | 0.7888648 | 0.9116361 | NA | NA | |
| ENSG00000185122 | 2073.668832 | 0.01499039 | 0.0667966 | 0.7949804 | 0.9144634 | HSF1 | | 3297 |
| ENSG00000254690 | 35.02156462 | 0.008686997 | 0.1311012 | 0.8258525 | NA | NA | NA | |
| ENSG00000185000 | 629.5236817 | -0.069014355 | 0.0949427 | 0.3291476 | 0.5971072 | DGAT1 | | 8694 |
| ENSG00000214597 | 14.95593161 | 0.004303195 | 0.133987 | 0.8746872 | NA | NA | NA | |
| ENSG00000271698 | 17.51918125 | 0.012507362 | 0.1346986 | 0.643971 | NA | NA | NA | |
| ENSG00000185803 | 1937.439141 | 0.120003277 | 0.0747007 | 0.0587292 | 0.215052 | SLC52A2 | | 79581 |
| ENSG00000182325 | 933.6259362 | -0.012501549 | 0.0791361 | 0.8476305 | 0.9375782 | FBXL6 | | 26233 |
| ENSG00000173137 | 391.4642517 | -0.016850343 | 0.0985191 | 0.8050657 | 0.9186624 | ADCK5 | | 203054 |

| | | | | | | | | |
|---|---|---|---|---|---|---|---|---|
| ENSG00000071894 | 3877.249203 | -0.152208341 | 0.0619189 | 0.0061588 | 0.0442984 | CPSF1 | | 29894 |
| ENSG00000147804 | 351.1351096 | -0.02638399 | 0.1036718 | 0.6994813 | 0.8633391 | SLC39A4 | | 55630 |
| ENSG00000160948 | 1693.112392 | 0.040132754 | 0.0746467 | 0.5213773 | 0.7492875 | VPS28 | | 51160 |
| ENSG00000160949 | 2245.222053 | 0.002197888 | 0.0661346 | 0.970425 | 0.9882505 | TONSL | | 4796 |
| ENSG00000187954 | 955.4225718 | 0.066720165 | 0.0837028 | 0.3228245 | 0.5915158 | ZFTRAF1 | | 50626 |
| ENSG00000167702 | 1360.845917 | 0.059902382 | 0.0749666 | 0.3418688 | 0.6087837 | KIFC2 | | 90990 |
| ENSG00000160973 | 10.72268118 | 0.019391231 | 0.136742 | 0.3947524 | NA | FOXH1 | | 8928 |
| ENSG00000160972 | 1315.841741 | 0.23524316 | 0.0870872 | 0.0014834 | 0.0152692 | PPP1R16A | | 84988 |
| ENSG00000255182 | 10.54598779 | 0.025071604 | 0.1381899 | 0.2634972 | NA | NA | NA | |
| ENSG00000167700 | 147.8526526 | -0.02053705 | 0.1196125 | 0.7262205 | 0.8775196 | MFSD3 | | 113655 |
| ENSG00000160957 | 3702.121035 | 0.084243176 | 0.0625507 | 0.129772 | 0.35434 | RECQL4 | | 9401 |
| ENSG00000265393 | 31.29376385 | 0.007874874 | 0.1313877 | 0.8339024 | NA | NA | NA | |
| ENSG00000160959 | 1692.358414 | 0.139963979 | 0.0840851 | 0.0425895 | 0.174557 | LRRC14 | | 9684 |
| ENSG00000254402 | 23.17596917 | 0.041739174 | 0.141507 | 0.2156599 | NA | LRRC24 | | 441381 |
| ENSG00000213563 | 638.4681162 | -0.146865573 | 0.107183 | 0.0624189 | 0.2233809 | C8orf82 | | 414919 |
| ENSG00000147799 | 716.7082212 | 0.000509395 | 0.0887841 | 0.9949587 | 0.9978632 | ARHGAP39 | | 80728 |
| ENSG00000198169 | 312.1746519 | 0.137384759 | 0.1381199 | 0.1012265 | 0.3044354 | ZNF251 | | 90987 |
| ENSG00000196378 | 64.63291682 | 0.043162623 | 0.135217 | 0.3800837 | 0.6412129 | ZNF34 | | 80778 |
| ENSG00000161016 | 27891.49353 | -0.033801651 | 0.0524905 | 0.4905049 | 0.7271973 | RPL8 | | 6132 |
| ENSG00000197363 | 58.65463155 | 0.098716635 | 0.1744341 | 0.0788409 | 0.2597745 | ZNF517 | | 340385 |
| ENSG00000147789 | 639.0300385 | 0.280754381 | 0.1200164 | 0.0027376 | 0.024017 | ZNF7 | | 7553 |
| ENSG00000170619 | 517.4730787 | 0.007693475 | 0.0933921 | 0.9113038 | 0.9649147 | COMMD5 | | 28991 |
| ENSG00000196150 | 189.2002869 | 0.383341516 | 0.225993 | 0.0056467 | 0.0414338 | ZNF250 | | 58500 |
| ENSG00000170631 | 240.6115714 | 0.302272638 | 0.1957083 | 0.0108592 | 0.067221 | ZNF16 | | 7564 |
| ENSG00000196922 | 384.0089176 | 0.115273292 | 0.1206973 | 0.1451847 | 0.3776933 | NA | NA | |
| ENSG00000182307 | 1783.447002 | 0.298630997 | 0.0805532 | 3.25E-05 | 0.000686 | C8orf33 | | 65265 |
| ENSG00000181404 | 181.3166903 | -0.020254962 | 0.1142194 | 0.749862 | 0.8918102 | WASHC1 | | 100287171 |
| ENSG00000170122 | 15.85222889 | 0.034680468 | 0.1401821 | 0.2132515 | NA | FOXD4 | | 2298 |
| ENSG00000172785 | 339.4479134 | -0.011410483 | 0.1006534 | 0.8662224 | 0.9459476 | ZNG1A | | 55871 |
| ENSG00000107099 | 16.62976172 | -0.002094798 | 0.1338574 | 0.9359269 | NA | DOCK8 | | 81704 |
| ENSG00000107104 | 441.6224038 | -0.120320396 | 0.1193952 | 0.1293781 | 0.353901 | KANK1 | | 23189 |
| ENSG00000226403 | 12.31672887 | 0.000691186 | 0.1346318 | 0.9785787 | NA | NA | NA | |
| ENSG00000080503 | 250.8025219 | 0.009607332 | 0.1089188 | 0.8881422 | 0.9545517 | SMARCA2 | | 6595 |
| ENSG00000147852 | 16.41102307 | 0.079289561 | 0.1700667 | 0.0081844 | NA | VLDLR | | 7436 |
| ENSG00000080608 | 663.5048495 | 0.025421058 | 0.0869577 | 0.7064333 | 0.867199 | PUM3 | | 9933 |
| ENSG00000080298 | 72.65400775 | 0.027751734 | 0.1292127 | 0.5793749 | 0.7918192 | RFX3 | | 5991 |
| ENSG00000107249 | 102.8598423 | -0.046146001 | 0.1295855 | 0.4276268 | 0.6791494 | GLIS3 | | 169792 |
| ENSG00000205808 | 55.60548377 | 0.023515265 | 0.1298294 | 0.6166177 | 0.8161713 | PLPP6 | | 403313 |
| ENSG00000106993 | 56.87888922 | -0.013915158 | 0.1282759 | 0.7616326 | 0.8978158 | CDC37L1 | | 55664 |
| ENSG00000147853 | 329.7758106 | 0.047743011 | 0.1084615 | 0.4861798 | 0.7239089 | AK3 | | 50808 |
| ENSG00000120158 | 228.3399005 | 0.065281173 | 0.1203622 | 0.3546027 | 0.6187516 | RCL1 | | 10171 |
| ENSG00000096968 | 105.7716241 | 0.00905927 | 0.1230018 | 0.868419 | 0.9471139 | JAK2 | | 3717 |
| ENSG00000107020 | 81.68660137 | 0.008471079 | 0.1243623 | 0.8723809 | 0.9484107 | PLGRKT | | 55848 |
| ENSG00000107036 | 226.5454309 | -0.120952346 | 0.1438515 | 0.1314131 | 0.3567363 | RIC1 | | 57589 |
| ENSG00000099219 | 655.6438332 | -0.291524246 | 0.1188168 | 0.0019055 | 0.0182446 | ERMP1 | | 79956 |
| ENSG00000183354 | 130.548335 | -0.142731144 | 0.1937698 | 0.0719104 | 0.2446795 | KIAA2026 | | 158358 |
| ENSG00000137040 | 230.4286143 | 0.080128299 | 0.1264255 | 0.2695164 | 0.537356 | RANBP6 | | 26953 |
| ENSG00000147854 | 410.7606259 | 0.260727925 | 0.1488331 | 0.0107599 | 0.0667837 | UHRF2 | | 115426 |
| ENSG00000107077 | 127.7017398 | 0.064724564 | 0.1328907 | 0.3155452 | 0.5843537 | KDM4C | | 23081 |
| ENSG00000137038 | 169.2215511 | 0.029782847 | 0.116415 | 0.6437437 | 0.8333617 | DMAC1 | | 90871 |
| ENSG00000153714 | 57.56888334 | 0.153621921 | 0.2530322 | 0.0280523 | 0.1318096 | LURAP1L | | 286343 |
| ENSG00000107186 | 1082.630773 | -0.126354796 | 0.0984173 | 0.0907114 | 0.284609 | MPDZ | | 8777 |
| ENSG00000147862 | 696.6531809 | -0.095413318 | 0.0964536 | 0.1882137 | 0.4389939 | NFIB | | 4781 |
| ENSG00000175893 | 291.0478337 | -0.211752394 | 0.1643493 | 0.0312852 | 0.1419966 | ZDHHC21 | | 340481 |
| ENSG00000155158 | 79.51359977 | 0.056203673 | 0.1382153 | 0.3026675 | 0.5720807 | TTC39B | | 158219 |
| ENSG00000164975 | 794.8005721 | 0.02427763 | 0.0836448 | 0.7150436 | 0.8711099 | SNAPC3 | | 6619 |
| ENSG00000164985 | 2097.203092 | -0.294346401 | 0.0754759 | 1.49E-05 | 0.000356 | PSIP1 | | 11168 |
| ENSG00000164989 | 77.49406496 | 0.020532308 | 0.1263083 | 0.6923844 | 0.8594028 | CCDC171 | | 203238 |
| ENSG00000044459 | 370.7197041 | -0.002309366 | 0.1018233 | 0.9700276 | 0.9881615 | CNTLN | | 54875 |
| ENSG00000155876 | 778.9659447 | -0.183880482 | 0.1068905 | 0.0234648 | 0.1156754 | RRAGA | | 10670 |
| ENSG00000147874 | 945.1489093 | 0.005845992 | 0.0789604 | 0.9285379 | 0.9726302 | HAUS6 | | 54801 |
| ENSG00000147872 | 427.305625 | 0.102643918 | 0.1181808 | 0.1838668 | 0.4338885 | PLIN2 | | 123 |
| ENSG00000137145 | 716.3928398 | -0.140851702 | 0.1015656 | 0.0658849 | 0.2320448 | DENND4C | | 55667 |
| ENSG00000272842 | 13.89967711 | -0.030483434 | 0.1390902 | 0.2416831 | NA | NA | NA | |
| ENSG00000137154 | 11303.45535 | -0.113299519 | 0.0539808 | 0.0220621 | 0.1109373 | RPS6 | | 6194 |
| ENSG00000177076 | 19.21058281 | -0.054615987 | 0.1491581 | 0.0840073 | NA | ACER2 | | 340485 |
| ENSG00000260912 | 17.57752966 | 0.008880258 | 0.1342892 | 0.7434471 | NA | NA | NA | |
| ENSG00000171843 | 184.135419 | -0.044589118 | 0.1182182 | 0.5483686 | 0.7697985 | MLLT3 | | 4300 |
| ENSG00000188352 | 778.7434272 | -0.116973173 | 0.0976142 | 0.1134342 | 0.3270052 | FOCAD | | 54914 |
| ENSG00000198642 | 557.6993904 | -0.014565812 | 0.0919647 | 0.8298048 | 0.930047 | KLHL9 | | 55958 |

| | | | | | | | | |
|---|---|---|---|---|---|---|---|---|
| ENSG00000171889 | 32.57217054 | 0.135677976 | 0.246233 | 0.0092866 | NA | MIR31HG | | 554202 |
| ENSG00000099810 | 901.8960813 | 0.226286801 | 0.1028699 | 0.0058623 | 0.0427195 | MTAP | | 4507 |
| ENSG00000265194 | 12.3802711 | 0.003133843 | 0.1345113 | 0.898428 | NA | NA | NA | |
| ENSG00000147889 | 868.297138 | -0.017136234 | 0.0812119 | 0.7924169 | 0.9130225 | CDKN2A | | 1029 |
| ENSG00000240498 | 36.8059947 | 0.005224418 | 0.1303659 | 0.8966118 | NA | CDKN2B-AS | | 100048912 |
| ENSG00000147883 | 96.04322937 | 0.355553648 | 0.384284 | 0.0129463 | 0.0762608 | CDKN2B | | 1030 |
| ENSG00000176399 | 45.70105448 | 0.037539866 | 0.1356477 | 0.3938501 | 0.6544181 | DMRTA1 | | 63951 |
| ENSG00000198680 | 383.665288 | 0.008055622 | 0.099463 | 0.9060932 | 0.9628103 | TUSC1 | | 286319 |
| ENSG00000120159 | 482.4730874 | 0.057915515 | 0.0984605 | 0.4143136 | 0.6685232 | CAAP1 | | 79886 |
| ENSG00000137055 | 1100.370098 | -0.053428605 | 0.079888 | 0.4119818 | 0.6661689 | PLAA | | 9373 |
| ENSG00000096872 | 215.2831874 | -0.041384679 | 0.1158698 | 0.5320395 | 0.7574349 | IFT74 | | 80173 |
| ENSG00000120162 | 128.9104311 | -0.066659667 | 0.1337134 | 0.3012614 | 0.5710237 | MOB3B | | 79817 |
| ENSG00000147894 | 255.271775 | 0.114515162 | 0.1400251 | 0.1472538 | 0.3807401 | C9orf72 | | 203228 |
| ENSG00000122729 | 2037.116905 | -0.322871687 | 0.0769577 | 3.73E-06 | 0.0001076 | ACO1 | | 48 |
| ENSG00000107201 | 1222.58101 | -0.279150158 | 0.0999007 | 0.0008095 | 0.0094043 | RIGI | | 23586 |
| ENSG00000197579 | 1055.008583 | 0.030946446 | 0.0784034 | 0.624873 | 0.8202204 | TOPORS | | 10210 |
| ENSG00000235453 | 35.88732452 | 0.008181491 | 0.1309111 | 0.8344084 | NA | SMIM27 | | 100129250 |
| ENSG00000165264 | 875.638815 | -0.062613927 | 0.0854862 | 0.3540147 | 0.6187516 | NDUFB6 | | 4712 |
| ENSG00000232303 | 22.51877868 | -0.007380982 | 0.1328836 | 0.8161487 | NA | DFFBP1 | | 100422581 |
| ENSG00000137074 | 562.6820121 | 0.345097071 | 0.1386039 | 0.0012812 | 0.0136177 | APTX | | 54840 |
| ENSG00000236184 | 17.15073237 | -0.002948356 | 0.1336861 | 0.9138239 | NA | NA | NA | |
| ENSG00000086061 | 10630.98914 | -0.226232036 | 0.0571055 | 1.75E-05 | 0.0004084 | DNAJA1 | | 3301 |
| ENSG00000122692 | 1575.238654 | -0.117526216 | 0.0773776 | 0.0701362 | 0.2408884 | SMU1 | | 55234 |
| ENSG00000086062 | 725.7925115 | 0.003333466 | 0.0852255 | 0.9609846 | 0.983972 | B4GALT1 | | 2683 |
| ENSG00000107262 | 1076.081749 | 0.286559947 | 0.1029483 | 0.0008006 | 0.0093519 | BAG1 | | 573 |
| ENSG00000086065 | 1505.540111 | -0.014365777 | 0.0722541 | 0.814224 | 0.9240962 | CHMP5 | | 51510 |
| ENSG00000086102 | 1059.858674 | 0.278301424 | 0.0962304 | 0.0006127 | 0.0075262 | NFX1 | | 4799 |
| ENSG00000165271 | 2838.376843 | 0.203506016 | 0.0692711 | 0.0009565 | 0.0106616 | NOL6 | | 65083 |
| ENSG00000226823 | 14.29669068 | 0.028495176 | 0.138501 | 0.2722421 | NA | NA | NA | |
| ENSG00000230453 | 168.6749654 | 0.055203558 | 0.1245164 | 0.4029903 | 0.6602839 | ANKRD18B | | 441459 |
| ENSG00000234373 | 31.87950052 | 0.004378206 | 0.1311242 | 0.9083525 | NA | NA | NA | |
| ENSG00000226562 | 31.24033296 | -0.004219787 | 0.1314655 | 0.9051991 | NA | CYP4F26P | | 106478958 |
| ENSG00000231991 | 29.01202276 | 0.02795092 | 0.1356041 | 0.4361491 | NA | NA | NA | |
| ENSG00000107341 | 1352.160422 | -0.137920566 | 0.0818899 | 0.041831 | 0.1723554 | UBE2R2 | | 54926 |
| ENSG00000137073 | 1550.3782 | 0.229036246 | 0.0834178 | 0.0013716 | 0.01437 | UBAP2 | | 55833 |
| ENSG00000198876 | 804.0798289 | -0.010635054 | 0.086828 | 0.8728129 | 0.9484907 | DCAF12 | | 25853 |
| ENSG00000165006 | 1144.183521 | 0.022630821 | 0.0763278 | 0.7217749 | 0.8743185 | UBAP1 | | 51271 |
| ENSG00000186638 | 507.436641 | 0.395735872 | 0.1566541 | 0.0009421 | 0.0105404 | KIF24 | | 347240 |
| ENSG00000164978 | 275.0197292 | -0.016400297 | 0.1058076 | 0.8070328 | 0.9198819 | NUDT2 | | 318 |
| ENSG00000164976 | 448.0808321 | -0.094076932 | 0.1085013 | 0.2095231 | 0.4660901 | MYORG | | 57462 |
| ENSG00000164970 | 757.5477402 | 0.044315162 | 0.0857668 | 0.5110682 | 0.7421105 | FAM219A | | 203259 |
| ENSG00000122735 | 20.18277144 | 0.051837458 | 0.1472787 | 0.1077617 | NA | DNAI1 | | 27019 |
| ENSG00000168913 | 73.22783816 | 0.035943945 | 0.1302366 | 0.4937462 | 0.7293183 | ENHO | | 375704 |
| ENSG00000164967 | 259.6895625 | 0.323357432 | 0.1871447 | 0.0072647 | 0.0501296 | RPP25L | | 138716 |
| ENSG00000137100 | 829.681692 | -0.047314073 | 0.0893374 | 0.4894409 | 0.7263574 | DCTN3 | | 11258 |
| ENSG00000147955 | 1505.408278 | 0.263418584 | 0.0848018 | 0.0003462 | 0.0048609 | SIGMAR1 | | 10280 |
| ENSG00000213930 | 147.0817877 | 0.08047119 | 0.1394705 | 0.2350153 | 0.4984047 | GALT | | 2592 |
| ENSG00000137070 | 121.135459 | 0.051608573 | 0.1306021 | 0.3906721 | 0.6508044 | IL11RA | | 3590 |
| ENSG00000187186 | 14.78453565 | 0.016053648 | 0.1352835 | 0.5506354 | NA | LOC730098 | | 730098 |
| ENSG00000230074 | 8.470843412 | 0.009807524 | 0.1355874 | 0.6354258 | NA | NA | NA | |
| ENSG00000137094 | 153.147761 | -0.071033159 | 0.1318108 | 0.2924095 | 0.561967 | DNAJB5 | | 25822 |
| ENSG00000165280 | 9821.061555 | -0.306406141 | 0.0555065 | 5.31E-09 | 3.41E-07 | VCP | | 7415 |
| ENSG00000221829 | 1190.661239 | -0.037187678 | 0.079219 | 0.5649709 | 0.7820725 | FANCG | | 2189 |
| ENSG00000165282 | 619.4746766 | -0.098851146 | 0.1011866 | 0.1817211 | 0.4314356 | PIGO | | 84720 |
| ENSG00000165283 | 1951.757532 | 0.022566042 | 0.0681974 | 0.7042008 | 0.8660529 | STOML2 | | 30968 |
| ENSG00000005238 | 194.1604632 | 0.135442681 | 0.1592023 | 0.1015677 | 0.3050688 | ATOSB | | 80256 |
| ENSG00000198722 | 1194.998179 | -0.256015185 | 0.0934053 | 0.0010875 | 0.0119168 | UNC13B | | 10497 |
| ENSG00000198853 | 1243.552121 | 0.388162003 | 0.0896343 | 1.59E-06 | 5.02E-05 | RUSC2 | | 9853 |
| ENSG00000107140 | 301.289608 | 0.016602031 | 0.1038695 | 0.8080109 | 0.9200093 | TESK1 | | 7016 |
| ENSG00000269900 | 26.72289183 | -0.016580625 | 0.1380747 | 0.0167708 | NA | NA | NA | |
| ENSG00000159884 | 256.8912997 | -0.040243528 | 0.1110868 | 0.5561681 | 0.7757478 | CCDC107 | | 203260 |
| ENSG00000137135 | 413.3235551 | -0.108317394 | 0.1163086 | 0.1627744 | 0.4050634 | ARHGEF39 | | 84904 |
| ENSG00000198467 | 8506.245133 | 0.144385172 | 0.0651531 | 0.012075 | 0.0726775 | TPM2 | | 7169 |
| ENSG00000137076 | 7964.446578 | -0.0723615 | 0.0538534 | 0.1287067 | 0.3529163 | TLN1 | | 7094 |
| ENSG00000107175 | 945.5113662 | 0.01979354 | 0.0798979 | 0.76106 | 0.8975206 | CREB3 | | 10488 |
| ENSG00000070610 | 1540.153693 | -0.025133595 | 0.0774166 | 0.6949675 | 0.8606228 | GBA2 | | 57704 |
| ENSG00000107185 | 1370.359689 | -0.034632201 | 0.0736749 | 0.5769385 | 0.7903388 | RGP1 | | 9827 |
| ENSG00000215183 | 12.44070319 | -0.018832649 | 0.1362515 | 0.4436472 | NA | MSMP | | 692094 |
| ENSG00000228843 | 11.37206369 | 0.016697681 | 0.1361814 | 0.4683994 | NA | NA | NA | |
| ENSG00000227388 | 29.48729155 | -0.003384291 | 0.1318938 | 0.9208 | NA | NA | NA | |

| | | | | | | | | |
|---|---|---|---|---|---|---|---|---|
| ENSG00000159899 | 133.2480426 | 0.026802044 | 0.120046 | 0.660119 | 0.8426737 | NPR2 | | 4882 |
| ENSG00000137133 | 239.1364525 | -0.054748559 | 0.1158963 | 0.4287703 | 0.6797592 | HINT2 | | 84681 |
| ENSG00000137103 | 53.11982063 | 0.023287523 | 0.130311 | 0.6118651 | 0.8141485 | TMEM8B | | 51754 |
| ENSG00000196196 | 27.11736786 | -0.029114636 | 0.1360754 | 0.4093431 | NA | HRCT1 | | 646962 |
| ENSG00000122707 | 189.5346727 | -0.001767568 | 0.1117262 | 0.9769131 | 0.9913073 | RECK | | 8434 |
| ENSG00000122694 | 295.9433546 | 0.154779827 | 0.1462301 | 0.0760892 | 0.2537974 | GLIPR2 | | 152007 |
| ENSG00000122705 | 3612.708513 | 0.045628411 | 0.0657476 | 0.4296543 | 0.6807911 | CLTA | | 1211 |
| ENSG00000159921 | 729.5178363 | -0.064996153 | 0.0898436 | 0.3471673 | 0.6134112 | GNE | | 10020 |
| ENSG00000137075 | 622.2050194 | -0.301316005 | 0.1252182 | 0.0020186 | 0.0189719 | RNF38 | | 152006 |
| ENSG00000165304 | 2121.201508 | -0.09656558 | 0.0718291 | 0.1159937 | 0.3320889 | MELK | | 9833 |
| ENSG00000196092 | 20.85702877 | 0.03964186 | 0.1410746 | 0.2167669 | NA | PAX5 | | 5079 |
| ENSG00000233137 | 1142.510953 | 0.148616137 | 0.0887382 | 0.0380746 | 0.1621989 | NA | NA | |
| ENSG00000147905 | 538.9120799 | 0.177324965 | 0.1229352 | 0.0394554 | 0.1663822 | ZCCHC7 | | 84186 |
| ENSG00000137106 | 1454.176636 | -0.059603836 | 0.0738304 | 0.338989 | 0.6064835 | GRHPR | | 9380 |
| ENSG00000168795 | 616.2144461 | -0.054262151 | 0.129975 | 0.3780163 | 0.6395077 | ZBTB5 | | 9925 |
| ENSG00000137054 | 1638.560951 | 0.046891537 | 0.070558 | 0.4386861 | 0.6882367 | POLR1E | | 64425 |
| ENSG00000234160 | 193.9144145 | -0.012364196 | 0.1128301 | 0.8466893 | 0.9372292 | NA | NA | |
| ENSG00000147912 | 84.65651199 | 0.010806638 | 0.1236904 | 0.8412592 | 0.9347236 | FBXO10 | | 26267 |
| ENSG00000175768 | 60.24704105 | -0.017895043 | 0.1287658 | 0.697655 | 0.862087 | TOMM5 | | 401505 |
| ENSG00000165275 | 187.9689911 | 0.055177268 | 0.1209918 | 0.415778 | 0.6697358 | TRMT10B | | 158234 |
| ENSG00000107371 | 682.2698005 | -0.068571163 | 0.0923786 | 0.3276551 | 0.5956947 | EXOSC3 | | 51010 |
| ENSG00000122741 | 506.9934703 | 0.146562779 | 0.1163321 | 0.0724105 | 0.2457085 | DCAF10 | | 79269 |
| ENSG00000122696 | 374.8087846 | 0.052820463 | 0.1045096 | 0.4582813 | 0.7025176 | SLC25A51 | | 92014 |
| ENSG00000107338 | 727.96279 | -0.011146515 | 0.0889473 | 0.8681169 | 0.9471139 | SHB | | 6461 |
| ENSG00000137124 | 770.2435737 | -0.080112194 | 0.0981926 | 0.2665002 | 0.5346023 | ALDH1B1 | | 219 |
| ENSG00000180071 | 310.3745529 | -0.016820232 | 0.1034896 | 0.8036849 | 0.9183959 | ANKRD18A | | 253650 |
| ENSG00000225345 | 163.0633156 | -0.02270561 | 0.1160813 | 0.7182132 | 0.8722654 | NA | NA | |
| ENSG00000204860 | 113.1546517 | -0.025027902 | 0.1223239 | 0.6659385 | 0.8452861 | NA | NA | |
| ENSG00000106714 | 1071.336699 | -0.0700008 | 0.0828152 | 0.2951715 | 0.5652292 | CNTNAP3 | | 79937 |
| ENSG00000227921 | 64.01273447 | -0.059754205 | 0.1421171 | 0.2510138 | 0.517489 | NA | NA | |
| ENSG00000185020 | 152.568659 | -0.027544272 | 0.1199712 | 0.6492008 | 0.8366057 | NA | NA | |
| ENSG00000215142 | 207.1670236 | 0.043488843 | 0.116361 | 0.5060774 | 0.7387601 | NA | NA | |
| ENSG00000196409 | 126.1922712 | 0.040140777 | 0.1246518 | 0.5084357 | 0.7401841 | NA | NA | |
| ENSG00000204837 | 11.4754885 | 0.00510542 | 0.1348522 | 0.8244066 | NA | NA | NA | |
| ENSG00000223839 | 20.91882535 | 0.004970694 | 0.1330834 | 0.8737116 | NA | NA | NA | |
| ENSG00000215126 | 34.36889063 | 0.048851411 | 0.1425741 | 0.2250241 | NA | ZNG1F | | 644019 |
| ENSG00000132498 | 14.62631545 | 0.02708956 | 0.1379148 | 0.3094067 | NA | NA | NA | |
| ENSG00000154529 | 521.0511226 | -0.097869633 | 0.1079635 | 0.1938122 | 0.4457414 | CNTNAP3B | | 728577 |
| ENSG00000237357 | 33.19934427 | 0.02712139 | 0.1348208 | 0.4699834 | NA | NA | NA | |
| ENSG00000204805 | 10.51789923 | 0.010157157 | 0.1353015 | 0.6539503 | NA | NA | NA | |
| ENSG00000182368 | 66.94739984 | 0.076575157 | 0.1527574 | 0.1613443 | 0.4033781 | NA | NA | |
| ENSG00000204807 | 8.367418596 | 0.022161368 | 0.1377999 | 0.2756275 | NA | NA | NA | |
| ENSG00000204802 | 21.76546676 | 0.004802541 | 0.1333413 | 0.8739094 | NA | FAM88C | | 102724238 |
| ENSG00000226007 | 14.58875223 | -0.003990536 | 0.1340891 | 0.8769613 | NA | NA | NA | |
| ENSG00000236029 | 35.53696768 | -0.049140142 | 0.1427254 | 0.2196801 | NA | NA | NA | |
| ENSG00000237792 | 31.43670734 | -0.024380141 | 0.1341124 | 0.5096416 | NA | NA | NA | |
| ENSG00000238113 | 57.78996722 | 0.051025705 | 0.139122 | 0.2993316 | 0.5693326 | LINC01410 | | 103352539 |
| ENSG00000202474 | 23.22632701 | 0.023090897 | 0.1352535 | 0.4809803 | NA | RNA5SP283 | | 100873536 |
| ENSG00000229422 | 24.39973597 | 0.009523345 | 0.1330443 | 0.770038 | NA | NA | NA | |
| ENSG00000170161 | 65.87792771 | -0.023922864 | 0.1285645 | 0.6250784 | 0.8202285 | FAM88B | | 728673 |
| ENSG00000198312 | 12.19309384 | -0.008525051 | 0.1347648 | 0.7254503 | NA | BMS1P9 | | 653458 |
| ENSG00000186466 | 9.873189941 | 0.002482689 | 0.134948 | 0.9102377 | NA | NA | NA | |
| ENSG00000237238 | 46.67503326 | 0.055057711 | 0.1435969 | 0.2247609 | 0.4852777 | BMS1P10 | | 728611 |
| ENSG00000232833 | 56.61678147 | 0.076407577 | 0.1559024 | 0.1321126 | 0.3581236 | NA | NA | |
| ENSG00000236233 | 9.543565167 | 0.004972967 | 0.135037 | 0.8195946 | NA | NA | NA | |
| ENSG00000170215 | 15.17952603 | -0.009766748 | 0.1343548 | 0.7156643 | NA | NA | NA | |
| ENSG00000196774 | 12.99013493 | 0.021518302 | 0.1369539 | 0.3746388 | NA | NA | NA | |
| ENSG00000232815 | 23.47100878 | 0.061229744 | 0.1525288 | 0.078141 | NA | NA | NA | |
| ENSG00000266017 | 30.00517669 | 0.00603485 | 0.1319187 | 0.8655861 | NA | MIR4477B | | 100616194 |
| ENSG00000233961 | 30.86449431 | 0.020371967 | 0.1375355 | 0.2956039 | NA | NA | NA | |
| ENSG00000204790 | 161.0764008 | 0.119745254 | 0.1620612 | 0.1185978 | 0.3359216 | LOC728877 | | 728877 |
| ENSG00000172014 | 10.5723307 | 0.003580146 | 0.1349052 | 0.8726511 | NA | NA | NA | |
| ENSG00000224185 | 10.45261998 | -0.007566223 | 0.1351366 | 0.7297009 | NA | NA | NA | |
| ENSG00000204778 | 10.42764156 | 0.001396088 | 0.1347041 | 0.9536227 | NA | ZNG1DP | | 653510 |
| ENSG00000147996 | 446.1219625 | 0.122392659 | 0.119197 | 0.1247419 | 0.3466834 | ZNG1E | | 220869 |
| ENSG00000196873 | 184.4271239 | 0.309904451 | 0.2481869 | 0.0142127 | 0.0813418 | ZNG1C | | 445571 |
| ENSG00000187866 | 190.6315548 | 0.049488447 | 0.1189567 | 0.4624923 | 0.7068393 | PABIR1 | | 116224 |
| ENSG00000165060 | 325.4933227 | -0.14480738 | 0.1399408 | 0.0883385 | 0.2797944 | FXN | | 2395 |
| ENSG00000119139 | 960.1671759 | -0.357045515 | 0.099767 | 3.93E-05 | 0.0008073 | TJP2 | | 9414 |
| ENSG00000188647 | 1313.898144 | 0.119403272 | 0.083185 | 0.079252 | 0.2605462 | PTAR1 | | 375743 |

| | | | | | | | | |
|---|---|---|---|---|---|---|---|---|
| ENSG00000165072 | 16.06855319 | 0.025482059 | 0.1372465 | 0.3559252 | NA | MAMDC2 | | 256691 |
| ENSG00000268364 | 15.84321312 | 0.003921368 | 0.1338056 | 0.8891607 | NA | NA | NA | |
| ENSG00000198887 | 1522.113699 | -0.035686018 | 0.0735997 | 0.5653615 | 0.7820725 | SMC5 | | 23137 |
| ENSG00000135048 | 645.5320853 | -0.096430413 | 0.0978481 | 0.1862328 | 0.4368804 | CEMIP2 | | 23670 |
| ENSG00000107362 | 294.1608987 | -0.003565022 | 0.1039504 | 0.9558479 | 0.9825402 | ABHD17B | | 51104 |
| ENSG00000155621 | 143.1258354 | 0.064213532 | 0.1303833 | 0.3111989 | 0.5812103 | C9orf85 | | 138241 |
| ENSG00000107372 | 3615.151458 | -0.421697748 | 0.0678581 | 5.27E-11 | 4.70E-09 | ZFAND5 | | 7763 |
| ENSG00000135046 | 1843.559681 | 0.334678665 | 0.0791702 | 3.13E-06 | 9.24E-05 | ANXA1 | | 301 |
| ENSG00000135045 | 351.7534538 | 0.116037215 | 0.1250368 | 0.1451614 | 0.3776933 | C9orf40 | | 55071 |
| ENSG00000156017 | 294.9499293 | 0.031933775 | 0.1061652 | 0.6425822 | 0.8325202 | CARNMT1 | | 138199 |
| ENSG00000106733 | 55.06214765 | -0.046106886 | 0.1376618 | 0.3290315 | 0.5970416 | NMRK1 | | 54981 |
| ENSG00000134996 | 383.2703706 | -0.050074102 | 0.1027907 | 0.4772911 | 0.7165169 | OSTF1 | | 26578 |
| ENSG00000135002 | 845.4486326 | -0.271975509 | 0.108499 | 0.0018965 | 0.0181787 | RFK | | 55312 |
| ENSG00000234618 | 12.11885315 | -0.019985367 | 0.1368327 | 0.3790898 | NA | NA | NA | |
| ENSG00000187210 | 89.91061758 | 0.067168716 | 0.1421617 | 0.2470399 | 0.5129849 | GCNT1 | | 2650 |
| ENSG00000197969 | 1348.206155 | -0.02049673 | 0.0811502 | 0.7527054 | 0.8932431 | VPS13A | | 23230 |
| ENSG00000156052 | 670.1702916 | -0.270317076 | 0.1164947 | 0.0030696 | 0.0260773 | GNAQ | | 2776 |
| ENSG00000148019 | 1054.188204 | -0.120083759 | 0.0864839 | 0.0841179 | 0.2709074 | CEP78 | | 84131 |
| ENSG00000135069 | 2929.725703 | 0.30497194 | 0.0770599 | 1.15E-05 | 0.000285 | PSAT1 | | 29968 |
| ENSG00000106829 | 476.4375753 | -0.197747342 | 0.1425494 | 0.0333145 | 0.1475158 | TLE4 | | 7091 |
| ENSG00000196781 | 416.7699704 | 0.284833988 | 0.1482758 | 0.0066018 | 0.0468502 | TLE1 | | 7088 |
| ENSG00000165105 | 618.7016347 | -0.125799042 | 0.1070859 | 0.1039041 | 0.3094604 | RASEF | | 158158 |
| ENSG00000172159 | 52.67259853 | -0.001657958 | 0.1289041 | 0.9670833 | 0.9871111 | FRMD3 | | 257019 |
| ENSG00000148057 | 18.47504212 | 0.014157723 | 0.1343534 | 0.6327105 | NA | IDNK | | 414328 |
| ENSG00000135018 | 2524.345634 | -0.124308855 | 0.0675995 | 0.0354437 | 0.1542648 | UBQLN1 | | 29979 |
| ENSG00000165113 | 55.42324865 | 0.030496241 | 0.1315471 | 0.5198643 | 0.7483099 | GKAP1 | | 80318 |
| ENSG00000165115 | 73.40663248 | 0.050520628 | 0.1361675 | 0.3416301 | 0.6087837 | KIF27 | | 55582 |
| ENSG00000165118 | 407.7363398 | -0.132702788 | 0.127829 | 0.1056197 | 0.3128946 | QNG1 | | 84267 |
| ENSG00000165119 | 13748.24842 | -0.293162436 | 0.0536779 | 8.69E-09 | 5.37E-07 | HNRNPK | | 3190 |
| ENSG00000235298 | 62.13019426 | -0.001228792 | 0.1264415 | 0.9779825 | 0.9917623 | NA | NA | |
| ENSG00000178966 | 357.1499267 | 0.083666592 | 0.1147087 | 0.2621436 | 0.5298047 | RMI1 | | 80010 |
| ENSG00000135049 | 1000.775668 | -0.026243215 | 0.0784563 | 0.6853857 | 0.8560726 | AGTPBP1 | | 23287 |
| ENSG00000165121 | 55.75090935 | 0.057265915 | 0.143113 | 0.2384273 | 0.5030834 | NA | NA | |
| ENSG00000135040 | 1134.051133 | -0.204016398 | 0.0910187 | 0.0064891 | 0.0461624 | NAA35 | | 60560 |
| ENSG00000135052 | 2665.81922 | -0.120742148 | 0.0671001 | 0.0411149 | 0.1703803 | GOLM1 | | 51280 |
| ENSG00000135070 | 1300.115448 | -0.050872097 | 0.0751323 | 0.418551 | 0.6713944 | ISCA1 | | 81689 |
| ENSG00000083223 | 835.8912608 | -0.009401402 | 0.0816687 | 0.8850357 | 0.9535774 | TUT7 | | 79670 |
| ENSG00000196730 | 452.2187174 | -0.073526852 | 0.1042121 | 0.3114516 | 0.5813465 | DAPK1 | | 1612 |
| ENSG00000135047 | 1147.530385 | 0.123173472 | 0.0852271 | 0.0752009 | 0.251855 | CTSL | | 1514 |
| ENSG00000156345 | 183.8450106 | 0.186596313 | 0.1959243 | 0.0494814 | 0.1913249 | CDK20 | | 23552 |
| ENSG00000106723 | 2663.961703 | -0.159219697 | 0.0691454 | 0.0086091 | 0.0572084 | SPIN1 | | 10927 |
| ENSG00000213694 | 10.85688645 | 0.004021196 | 0.1348788 | 0.8585038 | NA | S1PR3 | | 1903 |
| ENSG00000123975 | 2730.296169 | -0.180779829 | 0.0706699 | 0.0035722 | 0.0292002 | CKS2 | | 1164 |
| ENSG00000265112 | 15.89627148 | -0.011113259 | 0.1343675 | 0.6856574 | NA | MIR3153 | | 100422936 |
| ENSG00000187742 | 827.4710006 | 0.035030992 | 0.0881596 | 0.6073387 | 0.8116146 | SECISBP2 | | 79048 |
| ENSG00000187764 | 460.8932163 | -0.178638907 | 0.1351014 | 0.0446791 | 0.1799023 | SEMA4D | | 10507 |
| ENSG00000148090 | 292.4269534 | -0.066981297 | 0.1149733 | 0.3504822 | 0.6163779 | AUH | | 549 |
| ENSG00000165030 | 295.7872564 | 0.457534906 | 0.2004083 | 0.0013757 | 0.0143859 | NFIL3 | | 4783 |
| ENSG00000169071 | 136.9809422 | 0.049662205 | 0.125452 | 0.4336184 | 0.684513 | ROR2 | | 4920 |
| ENSG00000090054 | 1852.420576 | 0.002464852 | 0.0677818 | 0.9691396 | 0.9880876 | SPTLC1 | | 10558 |
| ENSG00000196305 | 7053.03888 | 0.031819168 | 0.052298 | 0.5103866 | 0.7416935 | IARS1 | | 3376 |
| ENSG00000239183 | 11.37648399 | -0.004764334 | 0.1347883 | 0.8331173 | NA | SNORA84 | | 100124534 |
| ENSG00000198000 | 1751.699267 | -0.018261924 | 0.0683154 | 0.7250349 | 0.8766311 | NOL8 | | 55035 |
| ENSG00000188312 | 481.6174843 | 0.063445723 | 0.1020642 | 0.3769173 | 0.6387307 | CENPP | | 401541 |
| ENSG00000127080 | 550.1398727 | 0.146313212 | 0.1130412 | 0.0698373 | 0.24026 | IPPK | | 64768 |
| ENSG00000185963 | 2270.837924 | 0.017714192 | 0.06545 | 0.7588729 | 0.8963893 | BICD2 | | 23299 |
| ENSG00000226721 | 13.37047283 | -0.009649994 | 0.1347269 | 0.6999992 | NA | EEF1DP2 | | 442429 |
| ENSG00000127081 | 245.6354737 | -0.036400339 | 0.1141581 | 0.583026 | 0.7946706 | ZNF484 | | 83744 |
| ENSG00000127084 | 14.47804867 | -0.006184112 | 0.1342438 | 0.8106136 | NA | FGD3 | | 89846 |
| ENSG00000165233 | 603.5958642 | 0.009894502 | 0.0890891 | 0.8867195 | 0.9545517 | CARD19 | | 84270 |
| ENSG00000131669 | 303.5456873 | -0.064074864 | 0.112662 | 0.3698638 | 0.6322814 | NINJ1 | | 4814 |
| ENSG00000165238 | 687.7869051 | 0.152771947 | 0.107347 | 0.0528251 | 0.199822 | WNK2 | | 65268 |
| ENSG00000227603 | 8.596823675 | 0.036424361 | 0.1424406 | 0.0752841 | NA | NA | NA | |
| ENSG00000188938 | 670.3282562 | 0.058072817 | 0.0901638 | 0.4009645 | 0.6587435 | FAM120AOS | | 158293 |
| ENSG00000048828 | 4773.354102 | -0.215240473 | 0.0620702 | 0.0001426 | 0.0023136 | FAM120A | | 23196 |
| ENSG00000263875 | 9.679611063 | 0.004820573 | 0.135085 | 0.8231399 | NA | NA | NA | |
| ENSG00000197724 | 1118.957521 | -0.141734943 | 0.0867913 | 0.0438733 | 0.1776992 | PHF2 | | 5253 |
| ENSG00000158079 | 582.581713 | 0.158562187 | 0.116649 | 0.0553711 | 0.2065101 | PTPDC1 | | 138639 |
| ENSG00000269929 | 95.49286019 | -0.193495291 | 0.2803421 | 0.0322581 | 0.1448142 | MIRLET7A1I | | 112903833 |
| ENSG00000199133 | 14.49269253 | -0.019509917 | 0.1360032 | 0.4599818 | NA | MIRLET7D | | 406886 |

| | | | | | | | | |
|---|---|---|---|---|---|---|---|---|
| ENSG00000175787 | 109.6920044 | 0.044675027 | 0.1280211 | 0.450542 | 0.6973716 | ZNF169 | | 169841 |
| ENSG00000148110 | 2711.294132 | 0.003719345 | 0.0617622 | 0.9469026 | 0.9786934 | MFSD14B | | 84641 |
| ENSG00000148120 | 281.7011447 | 0.199725132 | 0.1695952 | 0.039774 | 0.1670022 | AOPEP | | 84909 |
| ENSG00000158169 | 580.5567007 | -0.026135508 | 0.0939779 | 0.7027565 | 0.8650963 | FANCC | | 2176 |
| ENSG00000237857 | 38.59583442 | 0.025343175 | 0.1331622 | 0.5330141 | NA | NA | NA | |
| ENSG00000185920 | 210.1379508 | 0.044408019 | 0.115636 | 0.5116678 | 0.7421745 | PTCH1 | | 5727 |
| ENSG00000271155 | 9.344949161 | 0.009764497 | 0.1354206 | 0.6529717 | NA | NA | NA | |
| ENSG00000175611 | 59.28717428 | 0.124716447 | 0.2038825 | 0.048082 | 0.1877157 | ERCC6L2-A | | 100128782 |
| ENSG00000182150 | 855.2303765 | -0.01625266 | 0.0826592 | 0.8042969 | 0.9186624 | ERCC6L2 | | 375748 |
| ENSG00000130958 | 368.8966684 | -0.10953991 | 0.1200983 | 0.1614786 | 0.4035192 | SLC35D2 | | 11046 |
| ENSG00000165244 | 1301.771576 | 0.19621375 | 0.0869513 | 0.0067736 | 0.0476356 | ZNF367 | | 195828 |
| ENSG00000130956 | 782.048726 | 0.005655084 | 0.0858793 | 0.9290548 | 0.9726302 | HABP4 | | 22927 |
| ENSG00000081377 | 735.9334953 | -0.094870774 | 0.0961036 | 0.1898184 | 0.4406336 | CDC14B | | 8555 |
| ENSG00000158122 | 579.6787772 | -0.116406339 | 0.1071083 | 0.1293285 | 0.353901 | PRXL2C | | 195827 |
| ENSG00000081386 | 622.2334085 | 0.063543608 | 0.0935101 | 0.3674201 | 0.6302253 | ZNF510 | | 22869 |
| ENSG00000196597 | 147.9862595 | -0.000915218 | 0.1158327 | 0.9867671 | 0.994767 | ZNF782 | | 158431 |
| ENSG00000196312 | 252.3468827 | 0.045165901 | 0.1162672 | 0.5042545 | 0.7381117 | NA | NA | |
| ENSG00000136943 | 898.0914371 | -0.107202662 | 0.0921685 | 0.1338969 | 0.3604716 | CTSV | | 1515 |
| ENSG00000159712 | 9.072960975 | 0.008503911 | 0.1354351 | 0.6829297 | NA | NA | NA | |
| ENSG00000203279 | 16.43681952 | -0.0258725 | 0.1372938 | 0.3484384 | NA | NA | NA | |
| ENSG00000254876 | 37.16589532 | -0.000389181 | 0.1301731 | 0.9903473 | NA | SUGT1P4-S | | 100499484 |
| ENSG00000197816 | 38.6844699 | -0.044430548 | 0.1399332 | 0.2780206 | NA | CCDC180 | | 100499483 |
| ENSG00000196116 | 729.2722329 | -0.046142634 | 0.0870506 | 0.4928426 | 0.7286234 | TDRD7 | | 23424 |
| ENSG00000136842 | 44.32523222 | -0.034579472 | 0.1346384 | 0.4271813 | 0.6791209 | TMOD1 | | 7111 |
| ENSG00000136925 | 589.5225643 | 0.111915255 | 0.1051015 | 0.1406399 | 0.3701299 | TSTD2 | | 158427 |
| ENSG00000136937 | 2119.516054 | 0.00448276 | 0.0646977 | 0.9381588 | 0.9761997 | NCBP1 | | 4686 |
| ENSG00000136936 | 344.8713288 | -0.000341658 | 0.1042765 | 0.9947162 | 0.9977447 | XPA | | 7507 |
| ENSG00000136932 | 194.2740142 | 0.014528637 | 0.1119968 | 0.8237315 | 0.9274924 | TRMO | | 51531 |
| ENSG00000136938 | 7275.934645 | -0.31117884 | 0.0605203 | 4.19E-08 | 2.19E-06 | ANP32B | | 10541 |
| ENSG00000095380 | 1497.359073 | 0.015427938 | 0.0737399 | 0.8047811 | 0.9186624 | NANS | | 54187 |
| ENSG00000106785 | 994.35343 | -0.065150838 | 0.081641 | 0.321697 | 0.5905614 | TRIM14 | | 9830 |
| ENSG00000106789 | 147.8838612 | 0.038894439 | 0.1208851 | 0.5395399 | 0.7631831 | CORO2A | | 7464 |
| ENSG00000095383 | 1497.263609 | 0.354116038 | 0.0860946 | 4.69E-06 | 0.0001329 | TBC1D2 | | 55357 |
| ENSG00000136928 | 37.35271729 | 0.022883485 | 0.1327335 | 0.5685289 | NA | GABBR2 | | 9568 |
| ENSG00000165138 | 669.739425 | 0.37505138 | 0.1172925 | 0.0001397 | 0.0022762 | ANKS6 | | 203286 |
| ENSG00000106799 | 718.1113254 | -0.07737714 | 0.0956183 | 0.2777438 | 0.5461905 | TGFBR1 | | 7046 |
| ENSG00000119523 | 1016.426082 | -0.084990976 | 0.0840698 | 0.2055033 | 0.4607272 | ALG2 | | 85365 |
| ENSG00000106803 | 1689.230045 | -0.159819198 | 0.0784866 | 0.0161163 | 0.0885906 | SEC61B | | 10952 |
| ENSG00000119508 | 21.67866713 | 0.012477074 | 0.1336137 | 0.6929103 | NA | NR4A3 | | 8013 |
| ENSG00000136874 | 416.9421267 | -0.055725045 | 0.1075403 | 0.4318371 | 0.6831378 | STX17 | | 55014 |
| ENSG00000023318 | 844.0374215 | -0.000505042 | 0.0813936 | 0.9929895 | 0.997344 | ERP44 | | 23071 |
| ENSG00000119509 | 336.3999683 | -0.037952687 | 0.1055659 | 0.5831823 | 0.7947909 | INVS | | 27130 |
| ENSG00000136891 | 1711.56122 | -0.060306621 | 0.0717184 | 0.3152065 | 0.5839695 | TEX10 | | 54881 |
| ENSG00000066697 | 2436.074163 | -0.14031911 | 0.0706577 | 0.0219844 | 0.1106417 | MSANTD3 | | 91283 |
| ENSG00000241697 | 24.34434101 | 0.026938107 | 0.1364246 | 0.4050556 | NA | TMEFF1 | | 8577 |
| ENSG00000170681 | 68.16032749 | 0.03825511 | 0.1330648 | 0.4392995 | 0.6886014 | CAVIN4 | | 347273 |
| ENSG00000136897 | 805.5698401 | -0.029225913 | 0.0871156 | 0.6637332 | 0.8441929 | MRPL50 | | 54534 |
| ENSG00000136870 | 394.4071296 | 0.053220169 | 0.1043933 | 0.4539044 | 0.6991498 | ZNF189 | | 7743 |
| ENSG00000165152 | 297.4600912 | 0.036324061 | 0.1097422 | 0.59512 | 0.8031885 | PGAP4 | | 84302 |
| ENSG00000155827 | 2093.899608 | 0.027830525 | 0.0652152 | 0.6287909 | 0.823051 | RNF20 | | 56254 |
| ENSG00000136824 | 4408.22029 | -0.113328346 | 0.0605548 | 0.0364464 | 0.1573281 | SMC2 | | 10592 |
| ENSG00000136783 | 275.0335405 | -0.123882206 | 0.1404345 | 0.1260736 | 0.3483265 | NIPSNAP3A | | 25934 |
| ENSG00000165028 | 31.69580593 | 0.044007658 | 0.140842 | 0.2536998 | NA | NIPSNAP3B | | 55335 |
| ENSG00000165029 | 165.2007117 | 0.648396756 | 0.2468308 | 0.0004054 | 0.0054744 | ABCA1 | | 19 |
| ENSG00000070214 | 1147.636588 | -0.28557902 | 0.0931718 | 0.0003357 | 0.0047366 | SLC44A1 | | 23446 |
| ENSG00000106701 | 468.8390486 | 0.243055375 | 0.1352509 | 0.0115569 | 0.070457 | FSD1L | | 83856 |
| ENSG00000229419 | 45.40528321 | 0.066052635 | 0.1508047 | 0.1496053 | 0.3849041 | RALGAPA1F | | 26134 |
| ENSG00000106692 | 1116.65436 | 0.036181813 | 0.0773087 | 0.5601529 | 0.7791315 | FKTN | | 2218 |
| ENSG00000095209 | 1047.382028 | 0.145311015 | 0.0998659 | 0.056862 | 0.2107249 | TMEM38B | | 55151 |
| ENSG00000148143 | 545.197537 | 0.178859041 | 0.1276641 | 0.0408247 | 0.1698292 | ZNF462 | | 58499 |
| ENSG00000119318 | 7282.216192 | -0.338402385 | 0.0571048 | 4.32E-10 | 3.28E-08 | RAD23B | | 5887 |
| ENSG00000136826 | 669.4101847 | 0.718576526 | 0.1323755 | 3.53E-09 | 2.38E-07 | KLF4 | | 9314 |
| ENSG00000070061 | 2296.807352 | -0.183262068 | 0.0731432 | 0.0040316 | 0.0320581 | ELP1 | | 8518 |
| ENSG00000119328 | 353.2930506 | -0.009481293 | 0.1043938 | 0.8869308 | 0.9545517 | ABITRAM | | 54942 |
| ENSG00000119326 | 6862.892552 | -0.231040919 | 0.0579065 | 1.68E-05 | 0.0003935 | CTNNAL1 | | 8727 |
| ENSG00000106771 | 3083.840224 | 0.022692735 | 0.0602599 | 0.6724935 | 0.8499086 | TMEM245 | | 23731 |
| ENSG00000095203 | 437.7384146 | -0.056003192 | 0.1037972 | 0.4303521 | 0.6816257 | EPB41L4B | | 54566 |
| ENSG00000070159 | 2163.203023 | -0.196941516 | 0.074089 | 0.0023234 | 0.0212363 | PTPN3 | | 5774 |
| ENSG00000241978 | 21.56561833 | -0.004654786 | 0.1330742 | 0.877538 | NA | NA | NA | |
| ENSG00000136810 | 5534.89903 | -0.293994476 | 0.0609236 | 2.34E-07 | 9.78E-06 | TXN | | 7295 |

| | | | | | | | | |
|---|---|---|---|---|---|---|---|---|
| ENSG00000198121 | 808.624496 | 0.0880595 | 0.0965732 | 0.2304238 | 0.4917926 | LPAR1 | | 1902 |
| ENSG00000136813 | 3908.110335 | -0.167955241 | 0.0623009 | 0.0027297 | 0.024017 | ECPAS | | 23392 |
| ENSG00000173258 | 220.6669174 | 0.036039082 | 0.1143592 | 0.5885409 | 0.7984432 | ZNF483 | | 158399 |
| ENSG00000106853 | 1056.859586 | 0.084237097 | 0.0824874 | 0.2100356 | 0.4666816 | PTGR1 | | 22949 |
| ENSG00000059769 | 434.491914 | 0.250723187 | 0.1558354 | 0.0146845 | 0.0829445 | DNAJC25 | | 548645 |
| ENSG00000148154 | 5118.832543 | -0.260864072 | 0.0680141 | 2.49E-05 | 0.0005446 | UGCG | | 7357 |
| ENSG00000259953 | 25.40360945 | -0.021632511 | 0.1345761 | 0.5208009 | NA | NA | NA | |
| ENSG00000106868 | 180.3066349 | 0.05583425 | 0.1225965 | 0.4059883 | 0.6622468 | SUSD1 | | 64420 |
| ENSG00000119314 | 5208.427787 | -0.366316446 | 0.0647365 | 1.85E-09 | 1.30E-07 | PTBP3 | | 9991 |
| ENSG00000119471 | 1334.623559 | -0.118725934 | 0.0826024 | 0.0790857 | 0.2601277 | HSDL2 | | 84263 |
| ENSG00000230185 | 13.78261763 | -0.003482917 | 0.1343854 | 0.8854565 | NA | NA | NA | |
| ENSG00000165185 | 629.7787126 | -0.048253557 | 0.0964549 | 0.4901235 | 0.7267241 | KIAA1958 | | 158405 |
| ENSG00000148153 | 837.9830848 | -0.069157351 | 0.0878648 | 0.313385 | 0.5822768 | INIP | | 58493 |
| ENSG00000148158 | 741.5876883 | 0.049099874 | 0.0894274 | 0.4748918 | 0.7147547 | SNX30 | | 401548 |
| ENSG00000136866 | 194.5373482 | 0.047531754 | 0.1204632 | 0.4720577 | 0.7123777 | ZFP37 | | 7539 |
| ENSG00000225684 | 41.4044518 | 0.009750189 | 0.1301697 | 0.8143063 | 0.9240962 | FAM225B | | 100128385 |
| ENSG00000231528 | 72.65290616 | -0.026767126 | 0.1288341 | 0.5908457 | 0.8002862 | FAM225A | | 286333 |
| ENSG00000136867 | 334.7308299 | 0.048509362 | 0.1063675 | 0.4829545 | 0.7214731 | SLC31A2 | | 1318 |
| ENSG00000119321 | 1393.294155 | -0.001267312 | 0.0722157 | 0.9825602 | 0.9934198 | FKBP15 | | 23307 |
| ENSG00000136868 | 1617.805472 | -0.056619965 | 0.0718292 | 0.3551422 | 0.6192724 | SLC31A1 | | 1317 |
| ENSG00000176386 | 271.338912 | -0.009908215 | 0.1056399 | 0.8816546 | 0.9523403 | CDC26 | | 246184 |
| ENSG00000136875 | 2744.439611 | 0.038147799 | 0.0618131 | 0.4894327 | 0.7263574 | PRPF4 | | 9128 |
| ENSG00000148225 | 87.21425439 | 0.106075404 | 0.1705217 | 0.1068806 | 0.3155099 | WDR31 | | 114987 |
| ENSG00000119431 | 433.8817664 | 0.014251838 | 0.099054 | 0.8354826 | 0.9314949 | HDHD3 | | 81932 |
| ENSG00000148218 | 985.0400372 | -0.081881056 | 0.0848948 | 0.227201 | 0.4882146 | ALAD | | 210 |
| ENSG00000148229 | 3246.149667 | 0.020992743 | 0.0609993 | 0.7041857 | 0.8660529 | POLE3 | | 54107 |
| ENSG00000157653 | 15.80653565 | 0.020928418 | 0.1361904 | 0.4405844 | NA | C9orf43 | | 257169 |
| ENSG00000138835 | 122.9942372 | 0.012887491 | 0.1201711 | 0.8265278 | 0.9286016 | RGS3 | | 5998 |
| ENSG00000157657 | 958.0125712 | -0.442243359 | 0.1028941 | 1.54E-06 | 4.89E-05 | ZNF618 | | 114991 |
| ENSG00000136883 | 96.88527794 | -0.066130043 | 0.1391007 | 0.2676108 | 0.535818 | KIF12 | | 113220 |
| ENSG00000196739 | 1060.560275 | -0.04229708 | 0.0822583 | 0.5211696 | 0.749153 | COL27A1 | | 85301 |
| ENSG00000106948 | 317.8063432 | 0.00748863 | 0.1029499 | 0.9121618 | 0.965435 | AKNA | | 80709 |
| ENSG00000095397 | 213.9553467 | 0.026899634 | 0.1124452 | 0.6843933 | 0.8554818 | WHRN | | 25861 |
| ENSG00000136888 | 2118.67761 | 0.160631596 | 0.0752069 | 0.0128343 | 0.0758519 | ATP6V1G1 | | 9550 |
| ENSG00000157693 | 731.3683347 | 0.151473104 | 0.1168985 | 0.065162 | 0.2303746 | TMEM268 | | 203197 |
| ENSG00000181634 | 35.63514555 | 0.02584218 | 0.1338516 | 0.509591 | NA | TNFSF15 | | 9966 |
| ENSG00000041982 | 1800.589431 | 0.210546973 | 0.0840056 | 0.0031385 | 0.0264125 | TNC | | 3371 |
| ENSG00000182752 | 240.124765 | 0.548890042 | 0.2046404 | 0.0004117 | 0.0055344 | PAPPA | | 5069 |
| ENSG00000256040 | 9.636506113 | -0.024349177 | 0.138204 | 0.2487282 | NA | PAPPA-AS1 | | 493913 |
| ENSG00000148219 | 225.5164852 | -0.043832825 | 0.1152979 | 0.5141785 | 0.7444372 | ASTN2 | | 23245 |
| ENSG00000119401 | 1068.540419 | 0.019844905 | 0.0768 | 0.7556345 | 0.8946449 | TRIM32 | | 22954 |
| ENSG00000136861 | 2556.600927 | -0.029413405 | 0.0633064 | 0.5986813 | 0.8059463 | CDK5RAP2 | | 55755 |
| ENSG00000106780 | 498.783901 | -0.247170598 | 0.135595 | 0.0105786 | 0.0658978 | MEGF9 | | 1955 |
| ENSG00000119402 | 2535.687821 | -0.101921098 | 0.0687 | 0.0872327 | 0.2772694 | FBXW2 | | 26190 |
| ENSG00000214654 | 81.24073993 | 0.027705789 | 0.1268018 | 0.60592 | 0.8109248 | B3GALT9 | | 100288842 |
| ENSG00000095261 | 2195.54065 | -0.066984542 | 0.0677742 | 0.2566828 | 0.5236312 | PSMD5 | | 5711 |
| ENSG00000226752 | 247.4289677 | 0.206212183 | 0.1785948 | 0.0373342 | 0.1599782 | CUTALP | | 253039 |
| ENSG00000119403 | 2704.393071 | -0.093946174 | 0.0679478 | 0.1118588 | 0.3244846 | PHF19 | | 26147 |
| ENSG00000056558 | 927.0218272 | 2.069675094 | 0.1256512 | 2.94E-62 | 1.14E-58 | TRAF1 | | 7185 |
| ENSG00000106804 | 37.988507 | 0.650461072 | 0.7778422 | 0.0066077 | NA | C5 | | 727 |
| ENSG00000119397 | 626.4732374 | -0.040801154 | 0.0957335 | 0.5565443 | 0.7758558 | CNTRL | | 11064 |
| ENSG00000119396 | 3272.785143 | -0.050180091 | 0.0613974 | 0.3592239 | 0.6224233 | RAB14 | | 51552 |
| ENSG00000148180 | 1496.514997 | -0.183399289 | 0.0850176 | 0.0096593 | 0.0623373 | GSN | | 2934 |
| ENSG00000148175 | 1736.865195 | 0.191402077 | 0.0830241 | 0.0062816 | 0.0450705 | STOM | | 2040 |
| ENSG00000267987 | 11.45969858 | -0.034965625 | 0.1415389 | 0.1150072 | NA | NA | NA | |
| ENSG00000136848 | 869.1932589 | -0.190781268 | 0.1063438 | 0.019172 | 0.1007869 | DAB2IP | | 153090 |
| ENSG00000175764 | 75.93262111 | 0.011871026 | 0.1256241 | 0.8163311 | 0.9252377 | TTLL11 | | 158135 |
| ENSG00000119421 | 1332.843917 | -0.032835273 | 0.074557 | 0.5992291 | 0.80621 | NDUFA8 | | 4702 |
| ENSG00000119446 | 1079.551131 | 0.202871482 | 0.099537 | 0.0104319 | 0.0653376 | RBM18 | | 92400 |
| ENSG00000148187 | 640.4315452 | 0.065957158 | 0.0962691 | 0.3536177 | 0.6187516 | MRRF | | 92399 |
| ENSG00000269979 | 64.34317464 | 0.031879032 | 0.1316068 | 0.506023 | 0.7387601 | NA | NA | |
| ENSG00000270102 | 9.446037368 | 0.002248102 | 0.134928 | 0.9193502 | NA | NA | NA | |
| ENSG00000095303 | 10.28936266 | 0.00532951 | 0.1349028 | 0.8149099 | NA | PTGS1 | | 5742 |
| ENSG00000136940 | 1059.58379 | -0.233255535 | 0.1005747 | 0.0041209 | 0.0326348 | PDCL | | 5082 |
| ENSG00000056586 | 2534.768039 | -0.003219136 | 0.0665013 | 0.9549369 | 0.9820611 | RC3H2 | | 54542 |
| ENSG00000186130 | 520.8262534 | 0.067786132 | 0.0995392 | 0.3449204 | 0.6111049 | ZBTB6 | | 10773 |
| ENSG00000171448 | 371.2587506 | -0.076218899 | 0.1146909 | 0.2996065 | 0.5694273 | ZBTB26 | | 57684 |
| ENSG00000261094 | 62.06579214 | 0.007330747 | 0.1270283 | 0.8793265 | 0.9513156 | NA | NA | |
| ENSG00000011454 | 1459.418076 | -0.019000485 | 0.0747442 | 0.7609377 | 0.897467 | RABGAP1 | | 23637 |
| ENSG00000236901 | 212.3103848 | -0.105059562 | 0.1421701 | 0.1671054 | 0.4122331 | MIR600HG | | 81571 |

| | | | | | | | | |
|---|---|---|---|---|---|---|---|---|
| ENSG00000165209 | 1991.905468 | -0.160544138 | 0.074579 | 0.0124634 | 0.0744298 | STRBP | | 55342 |
| ENSG00000119522 | 504.7406939 | -0.004929704 | 0.0922592 | 0.9408753 | 0.976977 | DENND1A | | 57706 |
| ENSG00000119408 | 1031.949704 | -0.213803126 | 0.1013738 | 0.0080106 | 0.0541505 | NEK6 | | 10783 |
| ENSG00000136930 | 4490.533982 | -0.164204068 | 0.064071 | 0.0040726 | 0.0323179 | PSMB7 | | 5695 |
| ENSG00000148200 | 237.7623252 | 0.083148332 | 0.1259241 | 0.2573706 | 0.52429 | NR6A1 | | 2649 |
| ENSG00000224020 | 19.74033355 | 0.005733422 | 0.1333291 | 0.8503166 | NA | MIR181A2H( | | 100379345 |
| ENSG00000185585 | 23.84900269 | 0.0333446 | 0.1384261 | 0.3055243 | NA | OLFML2A | | 169611 |
| ENSG00000136942 | 15638.5373 | -0.195480481 | 0.0566492 | 0.0001815 | 0.0028264 | RPL35 | | 11224 |
| ENSG00000136950 | 1860.479785 | -0.058397881 | 0.0685106 | 0.324074 | 0.5928753 | ARPC5L | | 81873 |
| ENSG00000136935 | 607.8026411 | 0.027517028 | 0.0905902 | 0.7044398 | 0.8661643 | GOLGA1 | | 2800 |
| ENSG00000173611 | 399.0568934 | -0.073416955 | 0.1073788 | 0.3143641 | 0.5831486 | SCAI | | 286205 |
| ENSG00000119414 | 2211.065008 | -0.072519016 | 0.0673306 | 0.2173993 | 0.4767113 | PPP6C | | 5537 |
| ENSG00000136933 | 902.8902954 | -0.019563539 | 0.0804822 | 0.7656362 | 0.8994294 | RABEPK | | 10244 |
| ENSG00000044574 | 18902.07485 | -0.118714932 | 0.0568039 | 0.0227397 | 0.1136593 | HSPA5 | | 3309 |
| ENSG00000165219 | 2156.091362 | -0.017066317 | 0.0687758 | 0.7732942 | 0.9040968 | GAPVD1 | | 26130 |
| ENSG00000119487 | 2144.791098 | -0.077215228 | 0.070438 | 0.2015812 | 0.4556244 | MAPKAP1 | | 79109 |
| ENSG00000167081 | 506.9654177 | 0.065507206 | 0.0985909 | 0.3786922 | 0.6400365 | PBX3 | | 5090 |
| ENSG00000196814 | 262.3554749 | -0.085818814 | 0.1260084 | 0.2457131 | 0.5114352 | MVB12B | | 89853 |
| ENSG00000136944 | 52.21885013 | 0.005311821 | 0.128027 | 0.9093199 | 0.9642125 | LMX1B | | 4010 |
| ENSG00000169155 | 582.6559291 | 0.292267952 | 0.1241014 | 0.0024409 | 0.0220168 | ZBTB43 | | 23099 |
| ENSG00000177125 | 563.6276161 | -0.098711922 | 0.1100701 | 0.1926712 | 0.4441667 | ZBTB34 | | 403341 |
| ENSG00000136828 | 199.656317 | -0.257343365 | 0.2316389 | 0.0229024 | 0.1141309 | RALGPS1 | | 9649 |
| ENSG00000136895 | 134.081908 | -0.075189048 | 0.1376204 | 0.2551755 | 0.5221979 | GARNL3 | | 84253 |
| ENSG00000271833 | 8.893417754 | -0.001925591 | 0.1350003 | 0.9250542 | NA | NA | NA | |
| ENSG00000136856 | 520.8651715 | -0.154786135 | 0.1179895 | 0.0609108 | 0.2200738 | SLC2A8 | | 29988 |
| ENSG00000196152 | 210.8087266 | 0.097255168 | 0.1369761 | 0.1923575 | 0.4436149 | ZNF79 | | 7633 |
| ENSG00000197958 | 14772.72587 | 0.009786814 | 0.0491865 | 0.8178351 | 0.9254567 | RPL12 | | 6136 |
| ENSG00000201302 | 17.7484124 | -0.022197942 | 0.1360212 | 0.4425403 | NA | SNORA65 | | 26783 |
| ENSG00000148356 | 454.9999542 | -0.055599792 | 0.1025217 | 0.4332193 | 0.6844899 | LRSAM1 | | 90678 |
| ENSG00000136830 | 12586.07704 | -0.005171149 | 0.0530233 | 0.9149935 | 0.9662935 | NIBAN2 | | 64855 |
| ENSG00000136854 | 1102.882272 | 0.112762473 | 0.0844721 | 0.1003485 | 0.3025862 | STXBP1 | | 6812 |
| ENSG00000187024 | 317.3374526 | 0.149038776 | 0.1474831 | 0.0837725 | 0.2703169 | PTRH1 | | 138428 |
| ENSG00000160404 | 288.9213405 | 0.206928364 | 0.1654679 | 0.0345865 | 0.151483 | TOR2A | | 27433 |
| ENSG00000136807 | 1666.106758 | -0.06964747 | 0.0715057 | 0.2550595 | 0.5220518 | CDK9 | | 1025 |
| ENSG00000136877 | 1910.567405 | -0.138626145 | 0.0756369 | 0.0306843 | 0.1402507 | FPGS | | 2356 |
| ENSG00000106991 | 1426.742554 | 0.002317777 | 0.0731896 | 0.9712058 | 0.9885548 | ENG | | 2022 |
| ENSG00000225032 | 39.12087871 | 0.035515027 | 0.1360446 | 0.3915992 | NA | LOC102723! | | 102723566 |
| ENSG00000106992 | 200.3333117 | 0.060017458 | 0.1230915 | 0.3789837 | 0.6403732 | AK1 | | 203 |
| ENSG00000257524 | 16.65750361 | 0.044351849 | 0.1444685 | 0.111576 | NA | NA | NA | |
| ENSG00000160408 | 1620.782589 | -0.054821844 | 0.0818913 | 0.4030892 | 0.6602839 | ST6GALNAC | | 30815 |
| ENSG00000136840 | 1307.876144 | -0.061349689 | 0.0798236 | 0.3470672 | 0.6134112 | ST6GALNAC | | 27090 |
| ENSG00000167103 | 81.69998435 | 0.006507024 | 0.1252314 | 0.8991838 | 0.9602639 | PIP5KL1 | | 138429 |
| ENSG00000136908 | 1278.114818 | 0.267512918 | 0.0911216 | 0.0005799 | 0.0071906 | DPM2 | | 8818 |
| ENSG00000167106 | 755.3135462 | 0.320842584 | 0.1145292 | 0.0006217 | 0.007628 | EEIG1 | | 399665 |
| ENSG00000171169 | 217.0807363 | 0.056579581 | 0.1190534 | 0.4115661 | 0.6661689 | NAIF1 | | 203245 |
| ENSG00000148339 | 548.5145412 | 0.25281266 | 0.1249887 | 0.0069014 | 0.0482147 | SLC25A25 | | 114789 |
| ENSG00000269988 | 14.72617124 | 0.004440245 | 0.1341756 | 0.8663548 | NA | NA | NA | |
| ENSG00000234771 | 119.5195011 | 0.035069108 | 0.1241041 | 0.5548007 | 0.7749708 | NA | NA | |
| ENSG00000148334 | 2209.418116 | 0.091685223 | 0.070841 | 0.1325353 | 0.358616 | PTGES2 | | 80142 |
| ENSG00000148346 | 54.70068394 | -0.011139157 | 0.1279746 | 0.8072479 | 0.9200019 | LCN2 | | 3934 |
| ENSG00000171159 | 777.3193984 | 0.09503963 | 0.0943255 | 0.1862567 | 0.4368804 | BBLN | | 79095 |
| ENSG00000148337 | 3527.640561 | -0.007699602 | 0.0583986 | 0.8842726 | 0.953345 | CIZ1 | | 25792 |
| ENSG00000106976 | 379.0182398 | 0.021269134 | 0.0996309 | 0.7589834 | 0.8964293 | DNM1 | | 1759 |
| ENSG00000167110 | 2345.691434 | -0.001779584 | 0.0650932 | 0.9855295 | 0.9940394 | GOLGA2 | | 2801 |
| ENSG00000175854 | 385.2353369 | 0.138623726 | 0.1285123 | 0.0951223 | 0.2928559 | SWI5 | | 375757 |
| ENSG00000272960 | 33.81070466 | 0.009192669 | 0.1318732 | 0.8005301 | NA | NA | NA | |
| ENSG00000273281 | 58.63514437 | 0.005737076 | 0.1274879 | 0.9030007 | 0.9618565 | NA | NA | |
| ENSG00000167112 | 993.421434 | -0.192863774 | 0.1016223 | 0.015299 | 0.0854243 | TRUB2 | | 26995 |
| ENSG00000167113 | 830.9575162 | 0.217476399 | 0.1124991 | 0.0112279 | 0.0690646 | COQ4 | | 51117 |
| ENSG00000167114 | 1479.174994 | -0.086261429 | 0.0748689 | 0.1718178 | 0.4180386 | SLC27A4 | | 10999 |
| ENSG00000167118 | 1470.025351 | -0.016724079 | 0.0724263 | 0.7848354 | 0.9099416 | URM1 | | 81605 |
| ENSG00000272593 | 28.53239418 | -0.016208596 | 0.1329149 | 0.6468133 | NA | NA | NA | |
| ENSG00000167123 | 462.4316346 | 0.072227381 | 0.103827 | 0.3220675 | 0.5908703 | CERCAM | | 51148 |
| ENSG00000273186 | 15.20316843 | -0.031919769 | 0.1395903 | 0.2183576 | NA | NA | NA | |
| ENSG00000136811 | 2739.065892 | -0.12883345 | 0.0705784 | 0.0349569 | 0.1526474 | ODF2 | | 4957 |
| ENSG00000225951 | 21.64797304 | -0.022467528 | 0.1357913 | 0.4555716 | NA | ODF2-AS1 | | 107080620 |
| ENSG00000119392 | 1606.317272 | 0.014101088 | 0.0693625 | 0.8142258 | 0.9240962 | GLE1 | | 2733 |
| ENSG00000228395 | 17.28544224 | -0.017721169 | 0.1351115 | 0.5365073 | NA | NA | NA | |
| ENSG00000197694 | 8941.783304 | 0.089385758 | 0.0521062 | 0.0628793 | 0.2245583 | SPTAN1 | | 6709 |
| ENSG00000119333 | 1487.433876 | 0.192397086 | 0.0990741 | 0.0141405 | 0.0810877 | DYNC2I2 | | 89891 |

| | | | | | | | |
|---|---|---|---|---|---|---|---|
| ENSG00000119335 | 15444.74561 | -0.14293561 | 0.0557511 | 0.0050528 | 0.0382914 | SET | 6418 |
| ENSG00000160447 | 1543.301745 | -0.201134356 | 0.0869973 | 0.0056159 | 0.0413353 | PKN3 | 29941 |
| ENSG00000160446 | 465.5894889 | -0.063796815 | 0.1010151 | 0.3720164 | 0.6339958 | ZDHHC12 | 84885 |
| ENSG00000223478 | 214.2705486 | -0.193091022 | 0.1841795 | 0.0452299 | 0.1812453 | ZDHHC12-D | 100506100 |
| ENSG00000160445 | 675.7006364 | 0.062960393 | 0.0909004 | 0.365168 | 0.6276526 | ZER1 | 10444 |
| ENSG00000107021 | 1263.707783 | -0.003865242 | 0.0745323 | 0.9499087 | 0.9801708 | TBC1D13 | 54662 |
| ENSG00000167136 | 635.7108864 | 0.032920262 | 0.0895658 | 0.6322668 | 0.8251856 | ENDOG | 2021 |
| ENSG00000198917 | 1864.996466 | -0.05730756 | 0.0707847 | 0.3441172 | 0.610608 | SPOUT1 | 51490 |
| ENSG00000171097 | 155.4438623 | -0.025144425 | 0.11883 | 0.6814224 | 0.8538613 | KYAT1 | 883 |
| ENSG00000136802 | 1475.635251 | 0.267809103 | 0.0955459 | 0.0008639 | 0.0098741 | LRRC8A | 56262 |
| ENSG00000175283 | 595.1995491 | 0.038713103 | 0.0922386 | 0.5527075 | 0.7735653 | DOLK | 22845 |
| ENSG00000095319 | 5423.983792 | -0.089212276 | 0.0561725 | 0.0805446 | 0.2633934 | NUP188 | 23511 |
| ENSG00000148341 | 1764.990624 | -0.078977208 | 0.072186 | 0.1990385 | 0.4524614 | SH3GLB2 | 56904 |
| ENSG00000148343 | 901.5839677 | 0.072678085 | 0.0851094 | 0.2862884 | 0.5561508 | MIGA2 | 84895 |
| ENSG00000167130 | 692.2828678 | 0.069594981 | 0.0943163 | 0.3252028 | 0.5939431 | DOLPP1 | 57171 |
| ENSG00000095321 | 1042.795598 | 0.168748922 | 0.0929976 | 0.0234159 | 0.1155074 | CRAT | 1384 |
| ENSG00000119383 | 5017.576174 | -0.12259776 | 0.059798 | 0.0229797 | 0.1142625 | PTPA | 5524 |
| ENSG00000188483 | 263.8630635 | -0.089636789 | 0.1307518 | 0.2252629 | 0.4861946 | IER5L | 389792 |
| ENSG00000233901 | 16.02361623 | 0.001675384 | 0.1339728 | 0.9518977 | NA | LINC01503 | 100506119 |
| ENSG00000204054 | 1051.783426 | -0.131005869 | 0.0876924 | 0.0628605 | 0.2245583 | LOC124900: | 124900275 |
| ENSG00000148335 | 1323.719741 | 0.377313419 | 0.0908401 | 3.58E-06 | 0.0001039 | NTMT1 | 28989 |
| ENSG00000148331 | 836.424994 | -0.033106453 | 0.083026 | 0.6180891 | 0.8171928 | ASB6 | 140459 |
| ENSG00000136816 | 1511.789409 | 0.055776433 | 0.0732002 | 0.3686169 | 0.6315361 | TOR1B | 27348 |
| ENSG00000136827 | 1770.572043 | -0.027356602 | 0.069974 | 0.6476998 | 0.83646 | TOR1A | 1861 |
| ENSG00000136819 | 2106.567953 | 0.001151321 | 0.0646562 | 0.9847006 | 0.9940394 | C9orf78 | 51759 |
| ENSG00000136878 | 603.6989905 | 0.085643856 | 0.097932 | 0.2375542 | 0.5020572 | USP20 | 10868 |
| ENSG00000187239 | 2129.715933 | 0.063494946 | 0.069734 | 0.2902932 | 0.560019 | FNBP1 | 23048 |
| ENSG00000148358 | 2842.866401 | -0.064934905 | 0.063855 | 0.2544562 | 0.5211459 | GPR107 | 57720 |
| ENSG00000107130 | 1592.23002 | 0.167602849 | 0.0790887 | 0.0124841 | 0.0745153 | NCS1 | 23413 |
| ENSG00000130707 | 33.10514504 | -0.015871869 | 0.1321654 | 0.6721184 | NA | ASS1 | 445 |
| ENSG00000107164 | 2880.31648 | -0.109303818 | 0.0648844 | 0.0560386 | 0.208314 | FUBP3 | 8939 |
| ENSG00000130713 | 2581.217084 | -0.003789691 | 0.0618916 | 0.9470226 | 0.9786934 | EXOSC2 | 23404 |
| ENSG00000097007 | 2196.926735 | 0.21480466 | 0.0735459 | 0.0009145 | 0.0102914 | ABL1 | 25 |
| ENSG00000130720 | 18.56826923 | 0.055376195 | 0.1499962 | 0.0704321 | NA | FIBCD1 | 84929 |
| ENSG00000126878 | 25.38887922 | 0.030584495 | 0.1369888 | 0.370469 | NA | AIF1L | 83543 |
| ENSG00000126883 | 2060.752128 | 0.137322973 | 0.0721046 | 0.0272475 | 0.1288076 | NUP214 | 8021 |
| ENSG00000236986 | 9.4311332 | 0.0070839 | 0.1352564 | 0.7396942 | NA | NA | NA |
| ENSG00000126882 | 97.7222521 | 0.030305585 | 0.12538 | 0.5916038 | 0.800756 | FAM78A | 286336 |
| ENSG00000130723 | 9198.463082 | 0.043185145 | 0.0511025 | 0.3618943 | 0.6252459 | NA | NA |
| ENSG00000231587 | 14.65915497 | -0.028298294 | 0.138422 | 0.2785314 | NA | SNORD62B | 692093 |
| ENSG00000130714 | 1200.560662 | -0.086981591 | 0.0806961 | 0.1877523 | 0.4387148 | POMT1 | 10585 |
| ENSG00000130717 | 1437.827443 | -0.023368699 | 0.0759506 | 0.7094813 | 0.8682712 | UCK1 | 83549 |
| ENSG00000107263 | 2741.513481 | -0.179292157 | 0.0801683 | 0.0082719 | 0.0555313 | RAPGEF1 | 2889 |
| ENSG00000160563 | 534.7250053 | 0.069737851 | 0.0988606 | 0.3423433 | 0.6089405 | MED27 | 9442 |
| ENSG00000107290 | 4487.701178 | -0.073146718 | 0.0574178 | 0.1589545 | 0.3993864 | SETX | 23064 |
| ENSG00000125482 | 809.0119621 | 0.111884275 | 0.0936084 | 0.1222612 | 0.3424779 | TTF1 | 7270 |
| ENSG00000125485 | 1061.992211 | 0.282411041 | 0.0965055 | 0.0005385 | 0.0068072 | DDX31 | 64794 |
| ENSG00000125484 | 2633.122598 | -0.119575902 | 0.0691552 | 0.0464286 | 0.1842788 | GTF3C4 | 9329 |
| ENSG00000165695 | 10.69286422 | 0.008871206 | 0.1351547 | 0.6970467 | NA | AK8 | 158067 |
| ENSG00000165698 | 130.3602694 | 0.382074691 | 0.3074026 | 0.0097133 | 0.0625471 | SPACA9 | 11092 |
| ENSG00000165699 | 1070.728124 | -0.055127785 | 0.0792115 | 0.3975851 | 0.6567942 | TSC1 | 7248 |
| ENSG00000196205 | 5974.95769 | -0.22294527 | 0.0630872 | 0.0001058 | 0.0018256 | NA | NA |
| ENSG00000148308 | 2170.673928 | -0.023039217 | 0.0652948 | 0.6869465 | 0.857187 | GTF3C5 | 9328 |
| ENSG00000160271 | 622.1512697 | 0.036017857 | 0.0928242 | 0.6024572 | 0.8080863 | RALGDS | 5900 |
| ENSG00000148296 | 1964.665692 | 0.062942494 | 0.0741193 | 0.3168869 | 0.5853754 | SURF6 | 6838 |
| ENSG00000148297 | 1456.164669 | -0.10204388 | 0.0773528 | 0.1147576 | 0.3296812 | MED22 | 6837 |
| ENSG00000148303 | 22938.89381 | -0.160628666 | 0.0553504 | 0.0015718 | 0.0158958 | RPL7A | 6130 |
| ENSG00000148290 | 522.2923866 | -0.085972583 | 0.1024111 | 0.2423353 | 0.5081168 | SURF1 | 6834 |
| ENSG00000148291 | 632.7905886 | 0.006147452 | 0.0892549 | 0.9309142 | 0.9730657 | SURF2 | 6835 |
| ENSG00000148248 | 7109.863242 | -0.08499656 | 0.0549686 | 0.086972 | 0.2768173 | SURF4 | 6836 |
| ENSG00000198870 | 13.91509449 | -0.013721316 | 0.1351843 | 0.5877793 | NA | STKLD1 | 169436 |
| ENSG00000148300 | 1372.881447 | 0.15355923 | 0.0836599 | 0.0264974 | 0.1263332 | REXO4 | 57109 |
| ENSG00000160323 | 14.19645374 | 0.003586201 | 0.1342161 | 0.8908369 | NA | ADAMTS13 | 11093 |
| ENSG00000160325 | 90.70941049 | 0.007646019 | 0.1229662 | 0.8885575 | 0.9545884 | CACFD1 | 11094 |
| ENSG00000160326 | 697.2873301 | -0.031789547 | 0.0892872 | 0.6396474 | 0.8306614 | SLC2A6 | 11182 |
| ENSG00000160293 | 1933.029953 | -0.102785088 | 0.0723567 | 0.0963263 | 0.2949887 | VAV2 | 7410 |
| ENSG00000235106 | 620.1159909 | 0.007606765 | 0.0887543 | 0.9139859 | 0.9662036 | BRD3OS | 266655 |
| ENSG00000169925 | 1203.694379 | -0.010181267 | 0.0743401 | 0.8696573 | 0.9475003 | BRD3 | 8019 |
| ENSG00000196363 | 2935.531211 | -0.060869325 | 0.0615836 | 0.2713044 | 0.5389637 | WDR5 | 11091 |
| ENSG00000186350 | 1480.095938 | -0.267903246 | 0.0875517 | 0.0003866 | 0.0053005 | RXRA | 6256 |

| | | | | | | | |
|---|---|---|---|---|---|---|---|
| ENSG00000225361 | 38.77042688 | -0.001408577 | 0.12987 | 0.97033 | NA | PPP1R26-A! | 100506599 |
| ENSG00000196422 | 924.0586346 | 0.036984793 | 0.0836276 | 0.5787599 | 0.7915974 | PPP1R26 | 9858 |
| ENSG00000160345 | 78.60491625 | 0.071265972 | 0.1470161 | 0.2043613 | 0.4594559 | PIERCE1 | 138162 |
| ENSG00000122140 | 2144.536991 | 0.07455179 | 0.0699836 | 0.2160615 | 0.4744013 | MRPS2 | 51116 |
| ENSG00000226706 | 14.96539765 | 7.37E-05 | 0.1340932 | 0.9999672 | NA | LOC101928! | 101928525 |
| ENSG00000130559 | 2075.712612 | -0.026024534 | 0.0668722 | 0.6547871 | 0.8405019 | CAMSAP1 | 157922 |
| ENSG00000238058 | 46.93237758 | 0.075822409 | 0.1564869 | 0.1231001 | 0.3439664 | NA | NA |
| ENSG00000130560 | 1421.05496 | -0.136461459 | 0.0833218 | 0.0464751 | 0.1842999 | UBAC1 | 10422 |
| ENSG00000148411 | 1981.492133 | -0.105883267 | 0.0723258 | 0.0867946 | 0.2766296 | NACC2 | 138151 |
| ENSG00000238227 | 1168.543383 | 0.118723041 | 0.0840648 | 0.0833972 | 0.2698513 | TMEM250 | 90120 |
| ENSG00000165661 | 2897.530966 | 0.104258103 | 0.0667662 | 0.0745051 | 0.2505164 | QSOX2 | 169714 |
| ENSG00000160360 | 570.287521 | 0.308569017 | 0.1328528 | 0.0023915 | 0.0216724 | GPSM1 | 26086 |
| ENSG00000213221 | 21.47093174 | -0.011543755 | 0.1334879 | 0.7110375 | NA | DNLZ | 728489 |
| ENSG00000187796 | 26.54732612 | 0.036991314 | 0.1391563 | 0.2850237 | NA | CARD9 | 64170 |
| ENSG00000165684 | 1693.987747 | 0.102837995 | 0.0770064 | 0.1108895 | 0.3229259 | SNAPC4 | 6621 |
| ENSG00000165689 | 2806.068548 | 0.155985491 | 0.0693559 | 0.0102482 | 0.0645075 | ENTR1 | 10807 |
| ENSG00000165688 | 2649.216909 | -0.021817288 | 0.062338 | 0.6933836 | 0.8598104 | PMPCA | 23203 |
| ENSG00000148384 | 707.5914091 | 0.002316513 | 0.0849597 | 0.9731219 | 0.989729 | INPP5E | 56623 |
| ENSG00000148396 | 4616.742714 | -0.072947001 | 0.0588538 | 0.1695743 | 0.4153346 | SEC16A | 9919 |
| ENSG00000196366 | 11.32896736 | 0.008533221 | 0.1349431 | 0.7194055 | NA | C9orf163 | 158055 |
| ENSG00000148400 | 559.6165549 | 0.192199702 | 0.1195347 | 0.0261524 | 0.1250456 | NOTCH1 | 4851 |
| ENSG00000172889 | 450.8555478 | 0.079846424 | 0.1075986 | 0.2885051 | 0.5583214 | EGFL7 | 51162 |
| ENSG00000169692 | 1249.995391 | 0.124536331 | 0.0859157 | 0.0735273 | 0.2482906 | AGPAT2 | 10555 |
| ENSG00000165716 | 46.37540679 | 0.008156142 | 0.1290578 | 0.8523002 | 0.9396765 | DIPK1B | 138311 |
| ENSG00000233016 | 922.0235289 | 0.439655414 | 0.1113796 | 6.93E-06 | 0.0001822 | SNHG7 | 84973 |
| ENSG00000244187 | 259.4703331 | 0.071552064 | 0.1216965 | 0.3169175 | 0.5853754 | TMEM141 | 85014 |
| ENSG00000213213 | 14.20876962 | 0.015845527 | 0.1353844 | 0.5466762 | NA | CCDC183 | 84960 |
| ENSG00000273066 | 57.65101239 | -0.077565695 | 0.1552674 | 0.1399982 | 0.36948 | NA | NA |
| ENSG00000228544 | 231.8858362 | -0.011616448 | 0.1104344 | 0.8595116 | 0.9433297 | CCDC183-A | 100131193 |
| ENSG00000196642 | 4800.363426 | 0.027986429 | 0.0551209 | 0.5799737 | 0.7922857 | RABL6 | 55684 |
| ENSG00000265806 | 28.09934459 | -0.00245094 | 0.1317495 | 0.9423718 | NA | MIR4292 | 100422860 |
| ENSG00000232434 | 51.25413142 | 0.009176543 | 0.1283264 | 0.8403319 | 0.9344745 | AJM1 | 389813 |
| ENSG00000054148 | 1299.829032 | 0.007210923 | 0.0730732 | 0.9079234 | 0.9638297 | PHPT1 | 29085 |
| ENSG00000177943 | 267.0424428 | -0.283677983 | 0.1832781 | 0.0122131 | 0.0732349 | MAMDC4 | 158056 |
| ENSG00000107223 | 4537.146471 | 0.131965267 | 0.0594585 | 0.0138938 | 0.0802872 | EDF1 | 8721 |
| ENSG00000127191 | 1871.065993 | 0.1086808 | 0.0751958 | 0.087483 | 0.2778381 | TRAF2 | 7186 |
| ENSG00000266507 | 14.61043917 | 0.025571189 | 0.1374313 | 0.340342 | NA | MIR4479 | 100616480 |
| ENSG00000159069 | 3036.503022 | -0.057028249 | 0.0624969 | 0.3040192 | 0.5728743 | FBXW5 | 54461 |
| ENSG00000176919 | 14.9726764 | 0.014137076 | 0.1349895 | 0.5986586 | NA | C8G | 733 |
| ENSG00000148362 | 1149.800315 | 0.300723516 | 0.1008886 | 0.0004039 | 0.0054601 | PAXX | 286257 |
| ENSG00000107331 | 1447.89093 | 0.042686122 | 0.0756082 | 0.5003877 | 0.7347747 | ABCA2 | 20 |
| ENSG00000107281 | 629.6060989 | 0.074175989 | 0.098969 | 0.3036701 | 0.5728743 | NPDC1 | 56654 |
| ENSG00000054179 | 25.31125085 | -0.056961936 | 0.1491137 | 0.1127754 | NA | ENTPD2 | 954 |
| ENSG00000186193 | 1963.760603 | -0.267954219 | 0.0804591 | 0.0001564 | 0.0025098 | SAPCD2 | 89958 |
| ENSG00000231864 | 18.76757981 | 0.007150562 | 0.133787 | 0.8043217 | NA | NA | NA |
| ENSG00000197355 | 855.4630085 | 0.184667797 | 0.1014021 | 0.0194725 | 0.1016347 | UAP1L1 | 91373 |
| ENSG00000268996 | 35.03979352 | 0.018653389 | 0.132898 | 0.6166322 | NA | MAN1B1-DT | 100289341 |
| ENSG00000177239 | 1937.108154 | 0.01263857 | 0.0668178 | 0.8291148 | 0.9296029 | MAN1B1 | 11253 |
| ENSG00000176978 | 1717.44338 | 0.110803824 | 0.0783125 | 0.0896849 | 0.2826029 | DPP7 | 29952 |
| ENSG00000176884 | 11.40517213 | -0.010727213 | 0.1351542 | 0.6481244 | NA | GRIN1 | 2902 |
| ENSG00000176248 | 1716.704958 | 0.022155593 | 0.070573 | 0.71481 | 0.8710746 | ANAPC2 | 29882 |
| ENSG00000176101 | 1688.034629 | -0.011890317 | 0.0690112 | 0.8408934 | 0.9346002 | SSNA1 | 8636 |
| ENSG00000176058 | 728.2371165 | -0.141424926 | 0.1008469 | 0.0638229 | 0.2270144 | TPRN | 286262 |
| ENSG00000187713 | 820.9755324 | 0.029420902 | 0.083078 | 0.6573525 | 0.8419413 | TMEM203 | 94107 |
| ENSG00000188566 | 1058.197282 | 0.356495253 | 0.0967858 | 2.65E-05 | 0.0005751 | NDOR1 | 27158 |
| ENSG00000212864 | 176.7726202 | 0.052851543 | 0.1218367 | 0.4285785 | 0.6796361 | RNF208 | 727800 |
| ENSG00000197191 | 9.709428019 | 0.015661531 | 0.1361803 | 0.4756176 | NA | CYSRT1 | 375791 |
| ENSG00000188229 | 20366.23299 | -0.073782941 | 0.0514509 | 0.1248324 | 0.34677 | TUBB4B | 10383 |
| ENSG00000188163 | 63.34805542 | -0.032886252 | 0.1312159 | 0.502229 | 0.7365601 | CIMIP2A | 401565 |
| ENSG00000188986 | 3538.138857 | -0.073689448 | 0.0602304 | 0.1715493 | 0.4177334 | NELFB | 25920 |
| ENSG00000198113 | 2638.987134 | -0.063792554 | 0.0720566 | 0.2978608 | 0.568051 | TOR4A | 54863 |
| ENSG00000198435 | 83.06971839 | 0.102664632 | 0.1735649 | 0.0913646 | 0.2860247 | NRARP | 441478 |
| ENSG00000187609 | 131.1429949 | 0.03917802 | 0.1232483 | 0.5253175 | 0.7527274 | EXD3 | 54932 |
| ENSG00000188747 | 31.76438528 | 0.043253507 | 0.1406528 | 0.2568842 | NA | NOXA1 | 10811 |
| ENSG00000165802 | 3279.16643 | 0.306170768 | 0.0675858 | 9.26E-07 | 3.18E-05 | NSMF | 26012 |
| ENSG00000130653 | 38.1438934 | -0.004189737 | 0.1312969 | 0.9079196 | NA | PNPLA7 | 375775 |
| ENSG00000182154 | 1996.391264 | 0.004507568 | 0.0702771 | 0.9411799 | 0.976977 | MRPL41 | 64975 |
| ENSG00000148399 | 870.8563213 | 0.085529892 | 0.0875188 | 0.2165421 | 0.4751885 | DPH7 | 92715 |
| ENSG00000165724 | 1829.480326 | 0.21057478 | 0.0770655 | 0.0016711 | 0.0166551 | ZMYND19 | 116225 |
| ENSG00000197070 | 706.5601874 | -0.000593609 | 0.0852768 | 0.9920013 | 0.9968657 | ARRDC1 | 92714 |

| | | | | | | | |
|---|---|---|---|---|---|---|---|
| ENSG00000203993 | 346.6811948 | 0.043587592 | 0.1046241 | 0.5336083 | 0.7584666 | ARRDC1-AS | 85026 |
| ENSG00000181090 | 2022.178396 | -0.172013618 | 0.0771577 | 0.0091202 | 0.059899 | EHMT1 | 79813 |
| ENSG00000173876 | 9.018520886 | -0.005465556 | 0.1351504 | 0.792595 | NA | NA | NA |
| ENSG00000015171 | 1027.734511 | -0.175104827 | 0.0948889 | 0.0204753 | 0.1054086 | ZMYND11 | 10771 |
| ENSG00000151240 | 137.477629 | 0.0232858 | 0.118913 | 0.7033648 | 0.8652979 | DIP2C | 22982 |
| ENSG00000107929 | 1431.385251 | 0.006327484 | 0.0760351 | 0.9210641 | 0.9697015 | LARP4B | 23185 |
| ENSG00000107937 | 2674.46015 | 0.076096456 | 0.0655245 | 0.185263 | 0.435663 | GTPBP4 | 23560 |
| ENSG00000067064 | 852.3305039 | -0.314092562 | 0.1097197 | 0.0005315 | 0.00677 | IDI1 | 3422 |
| ENSG00000047056 | 389.2287862 | -0.028827802 | 0.1000305 | 0.6642023 | 0.8443298 | WDR37 | 22884 |
| ENSG00000067057 | 5560.156205 | -0.070821112 | 0.0564912 | 0.1695749 | 0.4153346 | PFKP | 5214 |
| ENSG00000107959 | 1727.762673 | 0.061864853 | 0.0709323 | 0.3089194 | 0.5782013 | PITRM1 | 10531 |
| ENSG00000237399 | 33.93253973 | -0.049512355 | 0.1430772 | 0.2131236 | NA | PITRM1-AS1 | 100507034 |
| ENSG00000067082 | 6197.699529 | 0.389637332 | 0.0651879 | 2.65E-10 | 2.06E-08 | KLF6 | 1316 |
| ENSG00000173848 | 833.241394 | 0.040479652 | 0.0847641 | 0.5401456 | 0.7637624 | NET1 | 10276 |
| ENSG00000196372 | 377.0745198 | 0.164257122 | 0.1353861 | 0.0601361 | 0.2183368 | ASB13 | 79754 |
| ENSG00000108021 | 2100.963146 | 0.0713554 | 0.0743757 | 0.250331 | 0.5163897 | TASOR2 | 54906 |
| ENSG00000057608 | 3675.095807 | -0.216033787 | 0.0642653 | 0.0002061 | 0.0031346 | GDI2 | 2665 |
| ENSG00000272764 | 10.16968903 | -0.010943079 | 0.1357395 | 0.5885764 | NA | NA | NA |
| ENSG00000134461 | 99.80357104 | 0.074871275 | 0.1442066 | 0.2238339 | 0.4842378 | ANKRD16 | 54522 |
| ENSG00000134452 | 982.1729332 | -0.059592159 | 0.0822337 | 0.3666392 | 0.6294405 | FBH1 | 84893 |
| ENSG00000134470 | 69.09637172 | 0.238384429 | 0.4508453 | 0.0172738 | 0.0933278 | IL15RA | 3601 |
| ENSG00000134453 | 1628.064609 | -0.018834616 | 0.0691205 | 0.7513475 | 0.8926188 | RBM17 | 84991 |
| ENSG00000170525 | 6830.908642 | 0.074887886 | 0.0623374 | 0.1798648 | 0.429389 | PFKFB3 | 5209 |
| ENSG00000198879 | 230.6762889 | 0.05928931 | 0.1176987 | 0.3969105 | 0.6567013 | SFMBT2 | 57713 |
| ENSG00000151657 | 230.0129386 | -0.068308698 | 0.121719 | 0.333097 | 0.6004589 | KIN | 22944 |
| ENSG00000165629 | 1716.766157 | -0.335766062 | 0.0806178 | 4.05E-06 | 0.0001158 | ATP5F1C | 509 |
| ENSG00000165632 | 392.4616419 | 0.032929923 | 0.0996465 | 0.6352512 | 0.8266198 | TAF3 | 83860 |
| ENSG00000048740 | 37.2607226 | -0.000280101 | 0.1302235 | 0.992504 | NA | CELF2 | 10659 |
| ENSG00000148429 | 443.4556646 | -0.059904949 | 0.1046735 | 0.4013031 | 0.6590218 | USP6NL | 9712 |
| ENSG00000148426 | 359.4987442 | 0.646358915 | 0.1559796 | 2.09E-06 | 6.43E-05 | PROSER2 | 254427 |
| ENSG00000151461 | 1069.888391 | -0.015712569 | 0.0772574 | 0.8045775 | 0.9186624 | UPF2 | 26019 |
| ENSG00000181192 | 384.6381974 | 0.060756482 | 0.1067281 | 0.3998838 | 0.6583091 | DHTKD1 | 55526 |
| ENSG00000065665 | 229.1389933 | 0.052467954 | 0.1168579 | 0.4462564 | 0.6941567 | SEC61A2 | 55176 |
| ENSG00000165609 | 926.5047178 | -0.096258752 | 0.0896414 | 0.1699937 | 0.4157205 | NUDT5 | 11164 |
| ENSG00000151465 | 1436.753973 | -0.091378779 | 0.0760275 | 0.1522949 | 0.3895241 | CDC123 | 8872 |
| ENSG00000228302 | 21.98056726 | 0.002783715 | 0.1329501 | 0.9304822 | NA | NA | NA |
| ENSG00000183049 | 29.4513419 | 0.025057298 | 0.1350561 | 0.476581 | NA | CAMK1D | 57118 |
| ENSG00000123240 | 1707.542143 | -0.123602856 | 0.0760282 | 0.0542379 | 0.2038458 | OPTN | 10133 |
| ENSG00000065328 | 1060.541409 | 0.130015286 | 0.0889162 | 0.0674899 | 0.2351177 | MCM10 | 55388 |
| ENSG00000107537 | 204.1443137 | -0.025982891 | 0.1140014 | 0.6877649 | 0.8577602 | PHYH | 5264 |
| ENSG00000086475 | 1181.810177 | -0.026703786 | 0.0753046 | 0.670476 | 0.8489788 | SEPHS1 | 22929 |
| ENSG00000165626 | 192.0776578 | -0.032294779 | 0.1145278 | 0.6205908 | 0.818389 | BEND7 | 222389 |
| ENSG00000165630 | 135.3206752 | -0.013331314 | 0.1184289 | 0.8230419 | 0.9273611 | PRPF18 | 8559 |
| ENSG00000239665 | 145.3313963 | 0.008608034 | 0.1170555 | 0.8887158 | 0.9546623 | NA | NA |
| ENSG00000151474 | 1828.237588 | -0.873592982 | 0.0919925 | 1.30E-22 | 4.59E-20 | FRMD4A | 55691 |
| ENSG00000065809 | 184.1833365 | 0.426145171 | 0.2323697 | 0.0037989 | 0.0306037 | FAM107B | 83641 |
| ENSG00000187522 | 814.353646 | -0.058099212 | 0.0860513 | 0.3904603 | 0.6505443 | HSPA14 | 51182 |
| ENSG00000272853 | 19.32193645 | 0.027400893 | 0.1371025 | 0.3664715 | NA | NA | NA |
| ENSG00000152455 | 445.757407 | 0.04208877 | 0.0992211 | 0.5478029 | 0.7691852 | SUV39H2 | 79723 |
| ENSG00000152457 | 195.4702181 | -0.027543079 | 0.1148663 | 0.6683088 | 0.8475209 | DCLRE1C | 64421 |
| ENSG00000176244 | 11.63360509 | -0.004437909 | 0.1349333 | 0.8371867 | NA | ACBD7 | 414149 |
| ENSG00000152464 | 203.348294 | 0.00292731 | 0.1108414 | 0.9658102 | 0.9863288 | RPP38 | 10557 |
| ENSG00000152465 | 517.7048613 | -0.279962653 | 0.130768 | 0.0043096 | 0.0337851 | NMT2 | 9397 |
| ENSG00000148468 | 638.3156808 | -0.220705977 | 0.1168764 | 0.0118683 | 0.0717565 | FAM171A1 | 221061 |
| ENSG00000148481 | 289.4855754 | 0.059349421 | 0.1127119 | 0.4015415 | 0.6592279 | MINDY3 | 80013 |
| ENSG00000165983 | 334.8919112 | -0.038524031 | 0.1046817 | 0.5785135 | 0.7915681 | PTER | 9317 |
| ENSG00000148484 | 1232.608458 | -0.005307933 | 0.0765052 | 0.932514 | 0.9738664 | RSU1 | 6251 |
| ENSG00000107614 | 77.42246184 | -0.031815578 | 0.1298058 | 0.5304291 | 0.7561561 | TRDMT1 | 1787 |
| ENSG00000229124 | 76.20783549 | -0.001974307 | 0.1257872 | 0.9663264 | 0.9865549 | VIM-AS1 | 100507347 |
| ENSG00000026025 | 13005.04191 | 0.223303084 | 0.0584438 | 3.55E-05 | 0.00074 | VIM | 7431 |
| ENSG00000234961 | 56.22063765 | 0.041916092 | 0.1375628 | 0.3398097 | 0.6069292 | NA | NA |
| ENSG00000165996 | 75.18116227 | 0.001082592 | 0.1247044 | 0.9847535 | 0.9940394 | HACD1 | 9200 |
| ENSG00000136738 | 441.5401329 | 0.077062132 | 0.1055162 | 0.3000944 | 0.5697989 | STAM | 8027 |
| ENSG00000240291 | 31.2339572 | 0.071861056 | 0.1585439 | 0.0730709 | NA | NA | NA |
| ENSG00000241058 | 68.91814728 | 0.108178899 | 0.1798195 | 0.0796794 | 0.2613697 | NSUN6 | 221078 |
| ENSG00000165997 | 509.661249 | 0.133138599 | 0.1132492 | 0.0966519 | 0.2955472 | ARL5B | 221079 |
| ENSG00000078114 | 143.6022724 | -0.091621977 | 0.1479021 | 0.1824688 | 0.4320381 | NEBL | 10529 |
| ENSG00000204682 | 576.6644905 | -0.357947863 | 0.1474814 | 0.0014101 | 0.0146548 | MIR1915HG | 399726 |
| ENSG00000222071 | 12.78436362 | -0.034472984 | 0.140819 | 0.1617456 | NA | MIR1915 | 100302129 |
| ENSG00000180592 | 56.41384155 | -0.326304606 | 0.897296 | 0.0113278 | 0.0693864 | SKIDA1 | 387640 |

| | | | | | | | | |
|---|---|---|---|---|---|---|---|---|
| ENSG00000078403 | 873.0993075 | -0.170609932 | 0.1032525 | 0.030708 | 0.1402816 | MLLT10 | | 8028 |
| ENSG00000136770 | 334.4200508 | 0.068868759 | 0.1123559 | 0.3422415 | 0.6088638 | DNAJC1 | | 64215 |
| ENSG00000148444 | 123.0681498 | -0.005713204 | 0.1196404 | 0.9217681 | 0.9699282 | COMMD3 | | 23412 |
| ENSG00000168283 | 654.9439471 | -0.07642042 | 0.0944499 | 0.281805 | 0.5509708 | BMI1 | | 648 |
| ENSG00000150867 | 467.2837462 | 0.00982343 | 0.0938761 | 0.887853 | 0.9545517 | PIP4K2A | | 5305 |
| ENSG00000148450 | 34.55763959 | 0.046950178 | 0.1415969 | 0.243594 | NA | MSRB2 | | 22921 |
| ENSG00000165312 | 40.70347903 | 0.013640326 | 0.1305966 | 0.7412081 | NA | OTUD1 | | 220213 |
| ENSG00000107863 | 897.3960553 | -0.082099527 | 0.089334 | 0.237893 | 0.5025095 | ARHGAP21 | | 57584 |
| ENSG00000099256 | 63.29700301 | 0.018265739 | 0.1278798 | 0.7074783 | 0.8676172 | PRTFDC1 | | 56952 |
| ENSG00000185875 | 85.39312961 | 0.081304867 | 0.1506839 | 0.1806254 | 0.4299157 | THNSL1 | | 79896 |
| ENSG00000151025 | 96.44917732 | 0.024082683 | 0.1247542 | 0.6622579 | 0.8437964 | GPR158 | | 57512 |
| ENSG00000148459 | 206.817502 | 0.044304745 | 0.1172216 | 0.5212196 | 0.749153 | PDSS1 | | 23590 |
| ENSG00000136754 | 1096.594015 | 0.00171925 | 0.0770065 | 0.9792374 | 0.992236 | ABI1 | | 10006 |
| ENSG00000107890 | 263.409722 | 0.041141924 | 0.1100572 | 0.550923 | 0.7718703 | ANKRD26 | | 22852 |
| ENSG00000136758 | 2605.205856 | -0.03968794 | 0.0642922 | 0.4829997 | 0.7214731 | YME1L1 | | 10730 |
| ENSG00000120539 | 893.6423778 | 0.086244964 | 0.0874122 | 0.2128603 | 0.4698453 | MASTL | | 84930 |
| ENSG00000107897 | 468.5283747 | -0.056929087 | 0.0998294 | 0.4215024 | 0.6737677 | ACBD5 | | 91452 |
| ENSG00000099246 | 1060.157031 | -0.08957604 | 0.0853159 | 0.1883228 | 0.4389939 | RAB18 | | 22931 |
| ENSG00000150054 | 8.405564165 | -0.008831521 | 0.1355369 | 0.6612564 | NA | MPP7 | | 143098 |
| ENSG00000254635 | 154.0444826 | 0.062509608 | 0.1299837 | 0.3394664 | 0.6066872 | NA | NA | |
| ENSG00000095787 | 2197.150782 | -0.19224346 | 0.0752258 | 0.0032469 | 0.0271875 | WAC | | 51322 |
| ENSG00000224597 | 281.6358989 | -0.100697508 | 0.1289404 | 0.1891438 | 0.4402049 | NA | NA | |
| ENSG00000197321 | 3555.741667 | -0.193536729 | 0.0735227 | 0.0025821 | 0.0229891 | SVIL | | 6840 |
| ENSG00000165757 | 139.2846947 | 0.15141859 | 0.1981256 | 0.0669398 | 0.2341648 | JCAD | | 57608 |
| ENSG00000259994 | 18.59053852 | 0.021250605 | 0.1356882 | 0.4730566 | NA | NA | NA | |
| ENSG00000107951 | 441.4175602 | 0.024660805 | 0.0968907 | 0.7204737 | 0.8733776 | MTPAP | | 55149 |
| ENSG00000107968 | 31.50211482 | 0.024944535 | 0.1345725 | 0.4942479 | NA | MAP3K8 | | 1326 |
| ENSG00000183621 | 72.52252284 | 0.004684913 | 0.1251437 | 0.9271846 | 0.9718632 | ZNF438 | | 220929 |
| ENSG00000237036 | 60.49382376 | 0.033169363 | 0.1314986 | 0.4967208 | 0.7319535 | ZEB1-AS1 | | 220930 |
| ENSG00000148516 | 446.2539682 | -0.025872623 | 0.0970677 | 0.706307 | 0.8671822 | ZEB1 | | 6935 |
| ENSG00000165322 | 452.4103779 | -0.050997052 | 0.1004836 | 0.4689681 | 0.7108642 | ARHGAP12 | | 94134 |
| ENSG00000170759 | 4747.424595 | -0.253952012 | 0.0615932 | 8.09E-06 | 0.0002095 | KIF5B | | 3799 |
| ENSG00000217094 | 15.38032934 | -0.032505014 | 0.1395636 | 0.2278323 | NA | NA | NA | |
| ENSG00000120616 | 362.4927331 | 0.011356383 | 0.0996069 | 0.8690859 | 0.9471723 | EPC1 | | 80314 |
| ENSG00000216937 | 9.73674305 | 0.001168064 | 0.1349247 | 0.9591116 | NA | CCDC7 | | 79741 |
| ENSG00000273038 | 55.0464293 | -0.016689209 | 0.1292987 | 0.7094221 | 0.8682712 | NA | NA | |
| ENSG00000150093 | 20845.63705 | 0.000666143 | 0.0511404 | 0.9767223 | 0.9912634 | ITGB1 | | 3688 |
| ENSG00000229656 | 66.21916516 | 0.002454787 | 0.1257643 | 0.9619614 | 0.9844723 | ITGB1-DT | | 101929475 |
| ENSG00000099250 | 54.8859773 | 0.220345162 | 0.4758629 | 0.0135498 | 0.0787436 | NRP1 | | 8829 |
| ENSG00000148498 | 2062.238529 | -0.292219985 | 0.0763681 | 2.08E-05 | 0.00047 | PARD3 | | 56288 |
| ENSG00000108094 | 1358.961153 | -0.218448974 | 0.0857007 | 0.0025892 | 0.0230168 | CUL2 | | 8453 |
| ENSG00000095794 | 236.0087963 | 0.465329654 | 0.2336838 | 0.0024984 | 0.0224317 | CREM | | 1390 |
| ENSG00000108100 | 1185.281701 | -0.060526812 | 0.0796121 | 0.3525905 | 0.6185027 | CCNY | | 219771 |
| ENSG00000271335 | 46.42346989 | 0.068163197 | 0.1521198 | 0.1404297 | 0.3698667 | NA | NA | |
| ENSG00000177283 | 28.90555814 | 0.068590598 | 0.1566525 | 0.0733192 | NA | FZD8 | | 8325 |
| ENSG00000198105 | 143.9743939 | -0.065242648 | 0.1335978 | 0.3085387 | 0.5779411 | ZNF248 | | 57209 |
| ENSG00000175395 | 45.59706596 | -0.0625702 | 0.1483336 | 0.169057 | 0.4146903 | ZNF25 | | 219749 |
| ENSG00000189180 | 337.1051468 | -0.050332635 | 0.1068015 | 0.4773508 | 0.7165169 | ZNF33A | | 7581 |
| ENSG00000075407 | 740.113158 | -0.023017651 | 0.0860598 | 0.7304258 | 0.880052 | ZNF37A | | 7587 |
| ENSG00000099251 | 26.87384076 | 0.011902853 | 0.1325473 | 0.7342244 | NA | NA | NA | |
| ENSG00000234420 | 735.9697785 | 0.100470083 | 0.0999364 | 0.1746376 | 0.4226108 | ZNF37BP | | 100129482 |
| ENSG00000272373 | 17.54164913 | -0.039725027 | 0.1419403 | 0.1715701 | NA | NA | NA | |
| ENSG00000196693 | 227.0329378 | -0.081816991 | 0.1263809 | 0.2638759 | 0.531712 | ZNF33B | | 7582 |
| ENSG00000165733 | 2775.192095 | -0.204626635 | 0.0697628 | 0.0009558 | 0.0106616 | BMS1 | | 9790 |
| ENSG00000169826 | 653.8003187 | 0.5428962 | 0.1206455 | 4.89E-07 | 1.86E-05 | CSGALNAC | | 55454 |
| ENSG00000169813 | 8329.138446 | -0.004653976 | 0.0523509 | 0.9228638 | 0.9702979 | HNRNPF | | 3185 |
| ENSG00000230555 | 48.67847091 | 0.008227154 | 0.1287134 | 0.8536638 | 0.9407214 | NA | NA | |
| ENSG00000243660 | 70.56653887 | -0.011161966 | 0.1259059 | 0.8220334 | 0.9271099 | ZNF487 | | 642819 |
| ENSG00000196793 | 354.7250398 | 0.614677495 | 0.16818 | 1.52E-05 | 0.0003613 | ZNF239 | | 8187 |
| ENSG00000198298 | 109.0834778 | 0.363489274 | 0.3366208 | 0.0118576 | 0.0717534 | ZNF485 | | 220992 |
| ENSG00000169740 | 212.5594967 | 0.009396891 | 0.1109825 | 0.8869045 | 0.9545517 | ZNF32 | | 7580 |
| ENSG00000107551 | 73.78354084 | -0.163024889 | 0.2513767 | 0.0356085 | 0.1547408 | RASSF4 | | 83937 |
| ENSG00000165512 | 328.034979 | -0.024673487 | 0.1031468 | 0.7178491 | 0.8722654 | ZNF22 | | 7570 |
| ENSG00000165406 | 494.9315976 | -0.148119498 | 0.1237016 | 0.0754074 | 0.2521711 | MARCHF8 | | 220972 |
| ENSG00000172671 | 57.7400795 | -0.006497924 | 0.1272798 | 0.8828785 | 0.9527552 | ZFAND4 | | 93550 |
| ENSG00000237840 | 10.98112969 | 0.013438107 | 0.1356163 | 0.561793 | NA | FAM21FP | | 100288690 |
| ENSG00000172661 | 854.1206426 | 0.139170491 | 0.095412 | 0.060819 | 0.2199465 | WASHC2C | | 253725 |
| ENSG00000239883 | 16.58041309 | 0.016388111 | 0.135036 | 0.5615875 | NA | NA | NA | |
| ENSG00000223477 | 176.3979089 | 0.740238035 | 0.235124 | 8.08E-05 | 0.0014673 | NA | NA | |
| ENSG00000213412 | 23.57329616 | 0.010585279 | 0.1329837 | 0.7488324 | NA | HNRNPA1P: | | 728643 |

| | | | | | | | | |
|---|---|---|---|---|---|---|---|---|
| ENSG00000150165 | 9.557973353 | 0.019570978 | 0.1370143 | 0.3630316 | NA | NA | NA | |
| ENSG00000204172 | 14.08365787 | -0.001096279 | 0.1340573 | 0.9642244 | NA | AGAP9 | | 642517 |
| ENSG00000215033 | 24.81127256 | 0.015429737 | 0.1336626 | 0.6405478 | NA | NA | NA | |
| ENSG00000152726 | 597.1980972 | 0.253161615 | 0.1318476 | 0.0084463 | 0.0563781 | NA | NA | |
| ENSG00000198035 | 31.42907331 | -0.024884994 | 0.1343789 | 0.4977219 | NA | NA | NA | |
| ENSG00000165388 | 84.57612998 | 0.017553424 | 0.1248418 | 0.7422597 | 0.8876265 | NA | NA | |
| ENSG00000204164 | 45.68389145 | -0.010660884 | 0.129429 | 0.8020008 | 0.9173383 | NA | NA | |
| ENSG00000188279 | 20.06086481 | 0.036397793 | 0.1399116 | 0.246454 | NA | NA | NA | |
| ENSG00000107643 | 897.7747694 | 0.073441177 | 0.0910214 | 0.2911171 | 0.5609429 | MAPK8 | | 5599 |
| ENSG00000225830 | 415.0134288 | 0.195846737 | 0.1465678 | 0.036263 | 0.1568264 | ERCC6 | | 2074 |
| ENSG00000227345 | 390.2319526 | -0.00616302 | 0.0983795 | 0.9271239 | 0.9718632 | PARG | | 8505 |
| ENSG00000174194 | 129.3457987 | 0.061700123 | 0.1314691 | 0.3354162 | 0.6029542 | NA | NA | |
| ENSG00000225784 | 34.93770857 | -0.012662722 | 0.1313926 | 0.7394941 | NA | NA | NA | |
| ENSG00000204152 | 75.38569992 | 0.014383163 | 0.1258496 | 0.7786137 | 0.9075471 | TIMM23B | | 100652748 |
| ENSG00000204169 | 16.42035226 | 0.011619539 | 0.1345765 | 0.6681327 | NA | NA | NA | |
| ENSG00000138293 | 2428.076341 | -0.042514994 | 0.0673379 | 0.4673275 | 0.7097164 | NA | NA | |
| ENSG00000138297 | 1074.563439 | -0.081393615 | 0.08376 | 0.2268308 | 0.4881315 | NA | NA | |
| ENSG00000214982 | 252.7645929 | -0.011947122 | 0.108059 | 0.8561064 | 0.9418544 | NA | NA | |
| ENSG00000178440 | 52.28282659 | 0.056104176 | 0.1439688 | 0.2207888 | 0.4803902 | TIMM23B-A( | | 113218477 |
| ENSG00000204149 | 509.6422121 | 0.117739855 | 0.113284 | 0.1328364 | 0.3591811 | AGAP6 | | 414189 |
| ENSG00000235618 | 12.27866967 | -0.009329551 | 0.1351321 | 0.6818136 | NA | NA | NA | |
| ENSG00000099290 | 253.6840957 | 0.07231668 | 0.120027 | 0.2920747 | 0.5617705 | WASHC2A | | 387680 |
| ENSG00000188611 | 20.09778658 | 0.009944396 | 0.1337097 | 0.7411554 | NA | ASAH2 | | 56624 |
| ENSG00000198964 | 649.9244341 | 0.043878068 | 0.089351 | 0.5226017 | 0.7501242 | SGMS1 | | 259230 |
| ENSG00000226200 | 27.64996844 | 0.042717205 | 0.1412206 | 0.2331627 | NA | NA | NA | |
| ENSG00000204147 | 99.86620165 | -0.015359179 | 0.1230753 | 0.7786677 | 0.9075471 | ASAH2B | | 653308 |
| ENSG00000177613 | 622.412757 | 0.051356648 | 0.092141 | 0.4612114 | 0.7055278 | CSTF2T | | 23283 |
| ENSG00000107984 | 303.8733105 | 0.876322978 | 0.1776225 | 4.36E-08 | 2.27E-06 | DKK1 | | 22943 |
| ENSG00000122952 | 4072.567051 | -0.052885383 | 0.0584586 | 0.3162195 | 0.5849906 | ZWINT | | 11130 |
| ENSG00000151151 | 306.1384785 | 0.014574061 | 0.1045511 | 0.8306678 | 0.9304016 | IPMK | | 253430 |
| ENSG00000122873 | 529.506763 | 0.043763085 | 0.0944665 | 0.5282908 | 0.754858 | CISD1 | | 55847 |
| ENSG00000072401 | 587.2194088 | 0.09888623 | 0.1019595 | 0.1832986 | 0.4329849 | UBE2D1 | | 7321 |
| ENSG00000108064 | 3208.449574 | -0.052445323 | 0.0613111 | 0.3379483 | 0.6055491 | TFAM | | 7019 |
| ENSG00000122870 | 2252.605347 | -0.24471789 | 0.0737312 | 0.0001921 | 0.0029677 | BICC1 | | 80114 |
| ENSG00000165449 | 14.33291787 | 0.048503715 | 0.1469076 | 0.0707949 | NA | SLC16A9 | | 220963 |
| ENSG00000108091 | 1172.707686 | 0.008683449 | 0.0746539 | 0.8903397 | 0.9554911 | CCDC6 | | 8030 |
| ENSG00000170312 | 2924.818337 | 0.0098806 | 0.0637723 | 0.8635144 | 0.9453914 | CDK1 | | 983 |
| ENSG00000182010 | 119.8809131 | -0.108033975 | 0.1611489 | 0.1308257 | 0.3555548 | RTKN2 | | 219790 |
| ENSG00000181915 | 759.6642154 | 0.093539562 | 0.0934432 | 0.1911833 | 0.4421326 | ADO | | 84890 |
| ENSG00000148572 | 336.4496872 | 0.004242387 | 0.1005985 | 0.9508469 | 0.9802173 | NRBF2 | | 29982 |
| ENSG00000171988 | 2008.480559 | -0.024218785 | 0.0710149 | 0.6891524 | 0.858679 | JMJD1C | | 221037 |
| ENSG00000272767 | 24.053871 | 0.00348992 | 0.1323685 | 0.9197834 | NA | JMJD1C-AS | | 84989 |
| ENSG00000165476 | 1024.699684 | -0.107373546 | 0.0871148 | 0.1219816 | 0.342196 | REEP3 | | 221035 |
| ENSG00000228065 | 83.49725091 | 0.027891082 | 0.1265318 | 0.6066595 | 0.8113886 | NA | NA | |
| ENSG00000096717 | 1279.01533 | 0.027575319 | 0.0780596 | 0.6681327 | 0.8474588 | SIRT1 | | 23411 |
| ENSG00000148634 | 1949.633832 | 0.045413157 | 0.0681002 | 0.4427259 | 0.6917073 | HERC4 | | 26091 |
| ENSG00000138347 | 12.77110149 | 0.007300014 | 0.1348466 | 0.7576806 | NA | MYPN | | 84665 |
| ENSG00000108187 | 61.64901983 | 0.023328733 | 0.1288432 | 0.6323379 | 0.8251856 | PBLD | | 64081 |
| ENSG00000096746 | 3675.746263 | -0.198569959 | 0.070249 | 0.0013949 | 0.0145245 | HNRNPH3 | | 3189 |
| ENSG00000204130 | 607.0514919 | 0.011505515 | 0.0893046 | 0.8672856 | 0.946535 | RUFY2 | | 55680 |
| ENSG00000138346 | 863.8459913 | 0.026546791 | 0.0838738 | 0.6901191 | 0.8589854 | DNA2 | | 1763 |
| ENSG00000122912 | 300.4951502 | 0.08400446 | 0.1197922 | 0.2571543 | 0.5241352 | SLC25A16 | | 8034 |
| ENSG00000138336 | 22.09437233 | 0.00859997 | 0.1331848 | 0.785783 | NA | TET1 | | 80312 |
| ENSG00000060339 | 2901.692898 | -0.078249105 | 0.0633336 | 0.1625248 | 0.4047229 | CCAR1 | | 55749 |
| ENSG00000221184 | 24.07910111 | -0.015986558 | 0.1341392 | 0.608236 | NA | NA | NA | |
| ENSG00000165730 | 19.38981261 | 0.051179986 | 0.147187 | 0.1016135 | NA | STOX1 | | 219736 |
| ENSG00000107625 | 708.7832126 | -0.026835979 | 0.0881742 | 0.6909808 | 0.8593387 | DDX50 | | 79009 |
| ENSG00000165732 | 10885.42438 | 0.080837921 | 0.0539578 | 0.1004137 | 0.3026141 | DDX21 | | 9188 |
| ENSG00000198954 | 935.6860741 | -0.184100994 | 0.09865 | 0.0180807 | 0.0965711 | KIFBP | | 26128 |
| ENSG00000122958 | 1740.792557 | -0.076525661 | 0.071205 | 0.2089841 | 0.4654027 | VPS26A | | 9559 |
| ENSG00000156502 | 750.2721184 | 0.304831539 | 0.1137062 | 0.0009585 | 0.0106734 | SUPV3L1 | | 6832 |
| ENSG00000156510 | 21.69706258 | 0.09678005 | 0.1872886 | 0.0100382 | NA | HKDC1 | | 80201 |
| ENSG00000156515 | 2650.497597 | -0.124563008 | 0.069676 | 0.0393091 | 0.1658251 | HK1 | | 3098 |
| ENSG00000099282 | 10.97385094 | -0.000825025 | 0.1348021 | 0.9678913 | NA | TSPAN15 | | 23555 |
| ENSG00000171224 | 405.7061556 | 0.006060477 | 0.0970609 | 0.9307528 | 0.972984 | FAM241B | | 219738 |
| ENSG00000197467 | 14.80941908 | 0.028221959 | 0.1381633 | 0.2962701 | NA | COL13A1 | | 1305 |
| ENSG00000099284 | 126.6049773 | 0.01453165 | 0.1190183 | 0.8079449 | 0.9200093 | MACROH2A | | 55506 |
| ENSG00000042286 | 926.3166398 | 0.017998778 | 0.0813802 | 0.7841263 | 0.9098848 | AIFM2 | | 84883 |
| ENSG00000156521 | 217.1939837 | 0.054913909 | 0.1178391 | 0.4259957 | 0.6784296 | TYSND1 | | 219743 |
| ENSG00000079332 | 2827.044269 | -0.115846956 | 0.0652145 | 0.0438225 | 0.1775548 | SAR1A | | 56681 |

| | | | | | | | | |
|---|---|---|---|---|---|---|---|---|
| ENSG00000180817 | 3074.561249 | -0.142086301 | 0.0680484 | 0.0170286 | 0.0922163 | PPA1 | | 5464 |
| ENSG00000172731 | 274.0248813 | 0.12329974 | 0.1400088 | 0.1285168 | 0.3524782 | LRRC20 | | 55222 |
| ENSG00000148730 | 1733.609697 | 0.051453056 | 0.0700975 | 0.3935298 | 0.6540725 | EIF4EBP2 | | 1979 |
| ENSG00000166224 | 1451.22704 | -0.029851686 | 0.0769166 | 0.6321998 | 0.8251856 | SGPL1 | | 8879 |
| ENSG00000166228 | 1582.788373 | -0.187649159 | 0.0849632 | 0.0082046 | 0.0551112 | PCBD1 | | 5092 |
| ENSG00000198246 | 16.95806771 | 0.021213916 | 0.1360256 | 0.451859 | NA | SLC29A3 | | 55315 |
| ENSG00000107738 | 294.7664187 | -0.028379791 | 0.1051592 | 0.6748915 | 0.8506969 | VSIR | | 64115 |
| ENSG00000197746 | 12173.09958 | 0.022561791 | 0.0502334 | 0.6350903 | 0.8266184 | PSAP | | 5660 |
| ENSG00000122863 | 988.0419976 | -0.01296577 | 0.079096 | 0.8399671 | 0.9344407 | CHST3 | | 9469 |
| ENSG00000107742 | 26.26227439 | 0.056432188 | 0.1490208 | 0.1103916 | NA | SPOCK2 | | 9806 |
| ENSG00000138303 | 783.6877074 | -0.194225219 | 0.1039852 | 0.0156876 | 0.0870118 | ASCC1 | | 51008 |
| ENSG00000166295 | 1246.186682 | -0.057670969 | 0.0769921 | 0.3663412 | 0.6290304 | ANAPC16 | | 119504 |
| ENSG00000168209 | 829.0967907 | 0.018353318 | 0.1236123 | 0.7408931 | 0.8866276 | DDIT4 | | 54541 |
| ENSG00000269926 | 29.61494589 | -0.015909635 | 0.1331706 | 0.6450618 | NA | NA | NA | |
| ENSG00000148719 | 1233.529273 | -0.086049352 | 0.0791732 | 0.18743 | 0.4383122 | DNAJB12 | | 54788 |
| ENSG00000107745 | 851.1513899 | -0.096876706 | 0.0897136 | 0.1673142 | 0.4122331 | MICU1 | | 10367 |
| ENSG00000156026 | 673.0625996 | 0.026850027 | 0.0871888 | 0.6910516 | 0.8593387 | MCU | | 90550 |
| ENSG00000122884 | 438.8521799 | 0.010879161 | 0.0952005 | 0.8742106 | 0.9491371 | P4HA1 | | 5033 |
| ENSG00000166321 | 73.04430768 | -0.018664514 | 0.1264752 | 0.7122364 | 0.869527 | NUDT13 | | 25961 |
| ENSG00000272599 | 66.56104287 | -0.036736164 | 0.1319961 | 0.4620076 | 0.7064682 | NA | NA | |
| ENSG00000221164 | 20.24640004 | 0.010205622 | 0.1335038 | 0.742392 | NA | SNORA11F | | 109617000 |
| ENSG00000122882 | 1035.243817 | -0.092361766 | 0.0853859 | 0.1744098 | 0.4222509 | ECD | | 11319 |
| ENSG00000138286 | 163.3518254 | -0.025139367 | 0.1161516 | 0.6911782 | 0.8593387 | FAM149B1 | | 317662 |
| ENSG00000213551 | 2261.712199 | 0.023400405 | 0.0643908 | 0.6808549 | 0.8538613 | DNAJC9 | | 23234 |
| ENSG00000182180 | 1736.064169 | 0.061947414 | 0.0717835 | 0.3120711 | 0.5818519 | MRPS16 | | 51021 |
| ENSG00000236756 | 21.98636929 | 0.009004615 | 0.1331634 | 0.7776654 | NA | DNAJC9-AS | | 414245 |
| ENSG00000227540 | 42.85917073 | 0.048529231 | 0.1407506 | 0.2682665 | 0.5361059 | DNAJC9-AS | | 414245 |
| ENSG00000138279 | 2418.219597 | -0.138662396 | 0.0687239 | 0.0208222 | 0.1065373 | ANXA7 | | 310 |
| ENSG00000233144 | 24.76471081 | -0.003505804 | 0.132288 | 0.9143491 | NA | NA | NA | |
| ENSG00000166343 | 22.45871912 | 0.039935045 | 0.1411546 | 0.2157709 | NA | MSS51 | | 118490 |
| ENSG00000107758 | 707.4283532 | -0.120644884 | 0.1012312 | 0.1091859 | 0.3199178 | PPP3CB | | 5532 |
| ENSG00000221817 | 58.19042898 | 0.087349358 | 0.1630977 | 0.1093055 | 0.3200394 | PPP3CB-AS | | 101929145 |
| ENSG00000166348 | 612.89995 | 0.008497812 | 0.0887669 | 0.9034509 | 0.9618565 | USP54 | | 159195 |
| ENSG00000272140 | 15.69729157 | -0.013776417 | 0.1347588 | 0.6142246 | NA | NA | NA | |
| ENSG00000242338 | 24.5230071 | 0.011927447 | 0.1329894 | 0.7221306 | NA | NA | NA | |
| ENSG00000250959 | 49.63235046 | 0.000564621 | 0.1282209 | 0.9925219 | 0.9969601 | NA | NA | |
| ENSG00000235316 | 26.07274328 | 0.010167887 | 0.13249 | 0.7691501 | NA | NA | NA | |
| ENSG00000176986 | 2811.87302 | -0.133192611 | 0.0660989 | 0.0217838 | 0.109774 | SEC24C | | 9632 |
| ENSG00000196968 | 586.8626149 | -0.16206109 | 0.1152268 | 0.0497095 | 0.1917456 | FUT11 | | 170384 |
| ENSG00000172586 | 799.7885721 | -0.115056522 | 0.0950761 | 0.1142884 | 0.328805 | CHCHD1 | | 118487 |
| ENSG00000214655 | 1441.597597 | 0.063996948 | 0.0765789 | 0.3164912 | 0.5851429 | ZSWIM8 | | 23053 |
| ENSG00000166507 | 36.54962987 | -0.005615354 | 0.1308551 | 0.8812291 | NA | NDST2 | | 8509 |
| ENSG00000148660 | 1154.833131 | -0.094039674 | 0.0828684 | 0.161798 | 0.4038382 | CAMK2G | | 818 |
| ENSG00000122861 | 13490.70346 | 0.119757951 | 0.1135355 | 0.1274851 | 0.3506746 | PLAU | | 5328 |
| ENSG00000222047 | 157.574304 | 0.043601363 | 0.1239095 | 0.4861256 | 0.7239089 | C10orf55 | | 414236 |
| ENSG00000035403 | 8801.768843 | -0.02111545 | 0.0606147 | 0.7011794 | 0.864704 | VCL | | 7414 |
| ENSG00000267957 | 26.60331205 | -0.019781199 | 0.1342429 | 0.5533448 | NA | NA | NA | |
| ENSG00000269772 | 9.857772552 | -0.013507516 | 0.1358216 | 0.5373888 | NA | NA | NA | |
| ENSG00000185009 | 1665.3295 | -0.04544115 | 0.0701441 | 0.4496582 | 0.6965805 | AP3M1 | | 26985 |
| ENSG00000156110 | 1447.958842 | -0.004849336 | 0.0706881 | 0.9353484 | 0.9752526 | ADK | | 132 |
| ENSG00000156650 | 457.6670272 | -0.135851076 | 0.1286469 | 0.0994492 | 0.3009499 | KAT6B | | 23522 |
| ENSG00000156671 | 1011.228895 | 0.027735526 | 0.0789396 | 0.6681873 | 0.8474588 | SAMD8 | | 142891 |
| ENSG00000165637 | 3758.451121 | -0.01123649 | 0.0620911 | 0.8405782 | 0.9344745 | VDAC2 | | 7417 |
| ENSG00000165644 | 248.8976952 | -0.020946667 | 0.1082338 | 0.7534532 | 0.8933224 | COMTD1 | | 118881 |
| ENSG00000165655 | 43.71966768 | 0.719461362 | 0.5760395 | 0.0048119 | 0.0370031 | ZNF503 | | 84858 |
| ENSG00000237149 | 14.04784029 | 0.004023435 | 0.1341753 | 0.878614 | NA | ZNF503-AS2 | | 100131213 |
| ENSG00000213513 | 23.0871796 | 0.019503606 | 0.1344049 | 0.5537685 | NA | NA | NA | |
| ENSG00000228748 | 27.52649751 | -0.073178918 | 0.1604201 | 0.0576391 | NA | NA | NA | |
| ENSG00000151208 | 3903.793875 | -0.320131337 | 0.0679114 | 3.53E-07 | 1.38E-05 | DLG5 | | 9231 |
| ENSG00000233871 | 8.787900628 | 0.006385457 | 0.1355084 | 0.7411025 | NA | NA | NA | |
| ENSG00000148606 | 1376.930371 | 0.384096023 | 0.0880763 | 1.40E-06 | 4.47E-05 | POLR3A | | 11128 |
| ENSG00000138326 | 10462.18395 | -0.207816583 | 0.0558415 | 5.82E-05 | 0.0011255 | RPS24 | | 6229 |
| ENSG00000108175 | 226.934207 | -0.011710019 | 0.1106924 | 0.8570046 | 0.9422695 | ZMIZ1 | | 57178 |
| ENSG00000108179 | 2678.93519 | 0.157171342 | 0.0696107 | 0.0097926 | 0.0628688 | PPIF | | 10105 |
| ENSG00000165424 | 17.78857253 | 0.011782334 | 0.1341558 | 0.6855784 | NA | ZCCHC24 | | 219654 |
| ENSG00000253626 | 9.400335507 | -0.031548326 | 0.1404373 | 0.1356127 | NA | EIF5AL1 | | 143244 |
| ENSG00000244733 | 12.13733496 | 0.003889023 | 0.1346004 | 0.8718327 | NA | NA | NA | |
| ENSG00000226381 | 40.32497314 | 0.038272998 | 0.1372118 | 0.3537107 | NA | NA | NA | |
| ENSG00000224886 | 31.09332445 | 0.038231818 | 0.1383963 | 0.3170086 | NA | NA | NA | |
| ENSG00000225484 | 164.9697437 | 0.003620887 | 0.1166665 | 0.9537855 | 0.9817737 | NUTM2B-AS | | 101060691 |

| | | | | | | | |
|---|---|---|---|---|---|---|---|
| ENSG00000272447 | 117.5445765 | 0.012028573 | 0.1199165 | 0.8384879 | 0.9334166 | LOC642361 | 642361 |
| ENSG00000133678 | 136.9078303 | 0.049265961 | 0.1265144 | 0.4304843 | 0.6817363 | TMEM254 | 80195 |
| ENSG00000122359 | 2319.343767 | -0.142330707 | 0.0703142 | 0.0198088 | 0.1029757 | ANXA11 | 311 |
| ENSG00000237523 | 243.1184183 | -0.00894415 | 0.1075136 | 0.8920642 | 0.956385 | LINC00857 | 439990 |
| ENSG00000234743 | 23.79107994 | -0.027842861 | 0.1366205 | 0.3894636 | NA | NA | NA |
| ENSG00000122378 | 840.6537256 | 0.019704992 | 0.0852398 | 0.7688518 | 0.9009387 | PRXL2A | 84293 |
| ENSG00000271738 | 80.44395052 | -0.008344668 | 0.1259813 | 0.8641389 | 0.9455189 | NA | NA |
| ENSG00000108219 | 1638.766891 | -0.116425326 | 0.0830728 | 0.0847556 | 0.2722107 | TSPAN14 | 81619 |
| ENSG00000226659 | 8.451770628 | 0.025358245 | 0.1386536 | 0.209251 | NA | TSPAN14-A | 101929574 |
| ENSG00000271933 | 19.37508979 | -0.006864865 | 0.133868 | 0.8048488 | NA | NA | NA |
| ENSG00000165678 | 4405.260418 | -0.073380921 | 0.0578044 | 0.1597485 | 0.4007257 | GHITM | 27069 |
| ENSG00000107771 | 602.1974915 | -0.006029687 | 0.088565 | 0.9266459 | 0.9718632 | CCSER2 | 54462 |
| ENSG00000062650 | 2459.036646 | -0.075355775 | 0.0653327 | 0.1884761 | 0.4391761 | WAPL | 23063 |
| ENSG00000227896 | 24.88488904 | 0.014474628 | 0.1332634 | 0.6702165 | NA | NA | NA |
| ENSG00000272631 | 73.90055582 | -0.026373736 | 0.128444 | 0.599755 | 0.8065338 | NA | NA |
| ENSG00000107779 | 1176.807154 | -0.235815101 | 0.0963014 | 0.0028957 | 0.0250552 | BMPR1A | 657 |
| ENSG00000272734 | 345.8161822 | 0.790807312 | 0.1575987 | 3.02E-08 | 1.66E-06 | NA | NA |
| ENSG00000148671 | 9.971081649 | 0.012761931 | 0.1356943 | 0.5650493 | NA | ADIRF | 10974 |
| ENSG00000148672 | 3156.65102 | 0.035796776 | 0.0611713 | 0.5103608 | 0.7416935 | GLUD1 | 2746 |
| ENSG00000122376 | 1389.165954 | -0.148642287 | 0.0909454 | 0.0405484 | 0.169099 | SHLD2 | 54537 |
| ENSG00000223482 | 377.7230192 | 0.010796537 | 0.0990067 | 0.8760312 | 0.9497329 | NUTM2A-AS | 728190 |
| ENSG00000224914 | 73.00916863 | 0.027624561 | 0.1285097 | 0.5887788 | 0.7985979 | LINC00863 | 439994 |
| ENSG00000214562 | 11.82540986 | -0.017111627 | 0.136083 | 0.4711859 | NA | NUTM2D | 728130 |
| ENSG00000107789 | 444.6560726 | 0.054181591 | 0.1001595 | 0.4447892 | 0.6929291 | MINPP1 | 9562 |
| ENSG00000198682 | 282.2307364 | 0.398900557 | 0.1847711 | 0.0022207 | 0.0204734 | PAPSS2 | 9060 |
| ENSG00000138138 | 1161.711333 | -0.080359698 | 0.0803818 | 0.2216509 | 0.4810983 | ATAD1 | 84896 |
| ENSG00000227268 | 11.47141487 | -0.005021702 | 0.1348585 | 0.822171 | NA | KLLN | 100144748 |
| ENSG00000171862 | 973.055107 | -0.16097163 | 0.0935218 | 0.0302538 | 0.1391865 | PTEN | 5728 |
| ENSG00000224745 | 9.010886847 | -0.006605635 | 0.1352085 | 0.7528779 | NA | NA | NA |
| ENSG00000184719 | 39.31655851 | 0.058997985 | 0.1469028 | 0.1792407 | NA | RNLS | 55328 |
| ENSG00000138134 | 163.1389651 | 0.25291899 | 0.2383631 | 0.0244288 | 0.1188473 | STAMBPL1 | 57559 |
| ENSG00000107796 | 40.84340859 | 0.067964433 | 0.1528501 | 0.1283782 | NA | ACTA2 | 59 |
| ENSG00000026103 | 936.7550382 | -0.051860407 | 0.0828866 | 0.4328193 | 0.6841524 | FAS | 355 |
| ENSG00000261438 | 19.72332845 | -0.011403072 | 0.1338666 | 0.7006057 | NA | NA | NA |
| ENSG00000107798 | 1002.919354 | -0.407199719 | 0.0992662 | 3.95E-06 | 0.0001135 | LIPA | 3988 |
| ENSG00000119922 | 11.103272 | -0.000604681 | 0.1348267 | 0.9755816 | NA | IFIT2 | 3433 |
| ENSG00000119917 | 20.42951229 | 0.031630216 | 0.1381233 | 0.3155225 | NA | IFIT3 | 3437 |
| ENSG00000152778 | 224.6968071 | -0.009878598 | 0.1092932 | 0.8786055 | 0.9511913 | IFIT5 | 24138 |
| ENSG00000152782 | 55.95598748 | -0.050006833 | 0.1397465 | 0.2868211 | 0.5568053 | PANK1 | 53354 |
| ENSG00000138182 | 896.9226623 | -0.127809153 | 0.0990403 | 0.0880942 | 0.279248 | KIF20B | 9585 |
| ENSG00000148680 | 13.36901335 | 0.020981561 | 0.1368305 | 0.3868518 | NA | HTR7 | 3363 |
| ENSG00000148688 | 530.2204467 | -0.037240003 | 0.0957479 | 0.58986 | 0.7994146 | RPP30 | 10556 |
| ENSG00000148677 | 180.7073663 | 2.915185498 | 0.4462434 | 3.05E-12 | 3.57E-10 | ANKRD1 | 27063 |
| ENSG00000180628 | 637.6178426 | -0.02547222 | 0.0880232 | 0.7097746 | 0.8682712 | PCGF5 | 84333 |
| ENSG00000165338 | 294.2370417 | 0.028343317 | 0.1054062 | 0.6796778 | 0.853781 | HECTD2 | 143279 |
| ENSG00000119938 | 619.6167637 | -0.093560807 | 0.1514656 | 0.1689126 | 0.4145971 | PPP1R3C | 5507 |
| ENSG00000228701 | 8.503091963 | -0.006303241 | 0.1352685 | 0.7578938 | NA | TNKS2-DT | 100507633 |
| ENSG00000107854 | 1381.514822 | 0.049056281 | 0.0763884 | 0.4413915 | 0.6905832 | TNKS2 | 80351 |
| ENSG00000180581 | 71.18701002 | -0.019630046 | 0.1270063 | 0.6946945 | 0.8605415 | NA | NA |
| ENSG00000174721 | 18.59337973 | 0.016698448 | 0.1349018 | 0.564493 | NA | FGFBP3 | 143282 |
| ENSG00000095564 | 1462.105124 | -0.019601388 | 0.0717116 | 0.747684 | 0.8903758 | BTAF1 | 9044 |
| ENSG00000107864 | 46.27093212 | 0.076373992 | 0.1577148 | 0.112917 | 0.3258358 | CPEB3 | 22849 |
| ENSG00000198060 | 710.1582489 | 0.036789228 | 0.0873658 | 0.5872587 | 0.7976592 | MARCHF5 | 54708 |
| ENSG00000119912 | 1112.468998 | -0.104481558 | 0.084153 | 0.1244139 | 0.346266 | IDE | 3416 |
| ENSG00000138160 | 2058.989455 | -0.229496663 | 0.0795849 | 0.0008988 | 0.010183 | KIF11 | 3832 |
| ENSG00000152804 | 140.6723344 | 0.094752587 | 0.1469111 | 0.1819782 | 0.4317028 | HHEX | 3087 |
| ENSG00000138190 | 192.3359682 | -0.29268549 | 0.2268305 | 0.0153314 | 0.085479 | EXOC6 | 54536 |
| ENSG00000138119 | 7493.38248 | -0.033540858 | 0.0537126 | 0.498653 | 0.7339675 | MYOF | 26509 |
| ENSG00000138180 | 2613.51998 | -0.046214355 | 0.0646839 | 0.4165496 | 0.6700325 | CEP55 | 55165 |
| ENSG00000148690 | 183.4647221 | -0.03839214 | 0.1172731 | 0.5560802 | 0.7757478 | FRA10AC1 | 118924 |
| ENSG00000176273 | 460.297361 | 0.007893645 | 0.094969 | 0.9087697 | 0.9640667 | SLC35G1 | 159371 |
| ENSG00000138193 | 29.21557229 | 0.021793334 | 0.1341144 | 0.5392 | NA | PLCE1 | 51196 |
| ENSG00000173145 | 909.1128565 | 0.122870908 | 0.0964293 | 0.0966068 | 0.2955472 | NOC3L | 64318 |
| ENSG00000108239 | 156.2613774 | -0.017852402 | 0.1171558 | 0.7710069 | 0.902418 | TBC1D12 | 23232 |
| ENSG00000119969 | 1846.528808 | 0.027113043 | 0.0711656 | 0.6563092 | 0.8417183 | HELLS | 3070 |
| ENSG00000107438 | 511.883038 | 0.054294925 | 0.0996744 | 0.4435578 | 0.692026 | PDLIM1 | 9124 |
| ENSG00000095637 | 80.37431272 | -0.079427984 | 0.1507765 | 0.1780772 | 0.4271872 | SORBS1 | 10580 |
| ENSG00000059573 | 1329.76307 | -0.25389747 | 0.0875731 | 0.0007073 | 0.0084829 | ALDH18A1 | 5832 |
| ENSG00000119977 | 440.5388233 | -0.102711214 | 0.1120975 | 0.1779838 | 0.4271872 | TCTN3 | 26123 |
| ENSG00000138185 | 36.45740074 | 0.019514871 | 0.1326527 | 0.6124932 | NA | ENTPD1 | 953 |

| | | | | | | | | |
|---|---|---|---|---|---|---|---|---|
| ENSG00000226688 | 249.7488828 | 0.030884338 | 0.1096979 | 0.6484638 | 0.836587 | ENTPD1-AS | | 728558 |
| ENSG00000107443 | 394.8780053 | -0.027303129 | 0.0988381 | 0.6922461 | 0.8594028 | CCNJ | | 54619 |
| ENSG00000177853 | 420.2968965 | 0.005486954 | 0.0966027 | 0.9371442 | 0.9756936 | ZNF518A | | 9849 |
| ENSG00000077147 | 4534.844369 | -0.177914557 | 0.0646997 | 0.0020821 | 0.0194414 | TM9SF3 | | 56889 |
| ENSG00000236552 | 71.94040447 | -0.011191746 | 0.1274222 | 0.8116791 | 0.9223877 | RPL13AP5 | | 728658 |
| ENSG00000196233 | 594.1574264 | -0.061245085 | 0.0945765 | 0.3828191 | 0.6435041 | LCOR | | 84458 |
| ENSG00000155640 | 278.6085244 | -0.186783882 | 0.1657877 | 0.0475819 | 0.1863864 | NA | NA | |
| ENSG00000231025 | 163.7511743 | -0.004238601 | 0.1160344 | 0.9435596 | 0.978317 | NA | NA | |
| ENSG00000213390 | 639.8902288 | -0.195015972 | 0.1148381 | 0.0216584 | 0.1093783 | ARHGAP19 | | 84986 |
| ENSG00000165879 | 27.62603126 | -0.021324429 | 0.134028 | 0.5439799 | NA | FRAT1 | | 10023 |
| ENSG00000181274 | 83.0231813 | 0.021022464 | 0.1255682 | 0.6931778 | 0.8597393 | FRAT2 | | 23401 |
| ENSG00000052749 | 1521.814886 | 0.394393992 | 0.0866838 | 5.55E-07 | 2.07E-05 | RRP12 | | 23223 |
| ENSG00000224474 | 10.54226945 | 0.000847259 | 0.1348348 | 0.971698 | NA | NA | NA | |
| ENSG00000171314 | 6580.014655 | -0.109922608 | 0.0553626 | 0.029295 | 0.1362243 | PGAM1 | | 5223 |
| ENSG00000171311 | 342.4437858 | -0.045820409 | 0.10484 | 0.507137 | 0.739559 | EXOSC1 | | 51013 |
| ENSG00000171307 | 485.0415493 | -0.006258822 | 0.0960986 | 0.9247504 | 0.9709924 | ZDHHC16 | | 84287 |
| ENSG00000155229 | 978.6717976 | -0.184520727 | 0.0994917 | 0.0182124 | 0.0971414 | MMS19 | | 64210 |
| ENSG00000165886 | 371.9248176 | 0.022351472 | 0.1002867 | 0.7458328 | 0.8893165 | UBTD1 | | 80019 |
| ENSG00000155252 | 386.3558629 | 0.035157892 | 0.1013721 | 0.613342 | 0.815068 | PI4K2A | | 55361 |
| ENSG00000171160 | 149.5261631 | 0.222583076 | 0.243831 | 0.0326349 | 0.1458578 | MORN4 | | 118812 |
| ENSG00000119986 | 62.05960154 | 0.058715039 | 0.1427303 | 0.2429286 | 0.5086739 | AVPI1 | | 60370 |
| ENSG00000155254 | 354.3248644 | 0.228097781 | 0.1593438 | 0.0229646 | 0.1142625 | MARVELD1 | | 83742 |
| ENSG00000155256 | 476.4559471 | 0.054579354 | 0.1026106 | 0.4427558 | 0.6917073 | ZFYVE27 | | 118813 |
| ENSG00000166024 | 345.8960786 | -0.013332116 | 0.1028012 | 0.8434243 | 0.9353791 | R3HCC1L | | 27291 |
| ENSG00000107521 | 502.785618 | 0.056471722 | 0.0974019 | 0.4429792 | 0.6917073 | HPS1 | | 3257 |
| ENSG00000120053 | 825.7515046 | 0.094677284 | 0.0899738 | 0.178616 | 0.4275952 | GOT1 | | 2805 |
| ENSG00000155287 | 309.5947352 | 0.16166926 | 0.148724 | 0.0679772 | 0.2359709 | SLC25A28 | | 81894 |
| ENSG00000198018 | 417.0758492 | 0.200806078 | 0.1397138 | 0.030396 | 0.1394565 | ENTPD7 | | 57089 |
| ENSG00000119929 | 213.8689839 | -0.056746905 | 0.1187199 | 0.4084184 | 0.6640358 | CUTC | | 51076 |
| ENSG00000014919 | 393.3594124 | 0.02145857 | 0.1014328 | 0.7533163 | 0.8932584 | COX15 | | 1355 |
| ENSG00000023839 | 47.40946976 | 0.012499676 | 0.1292244 | 0.7779807 | 0.9074533 | ABCC2 | | 1244 |
| ENSG00000107554 | 1230.256355 | 0.100925354 | 0.0819322 | 0.1333409 | 0.3597957 | DNMBP | | 23268 |
| ENSG00000227695 | 15.75764591 | 0.022851556 | 0.1369746 | 0.3739262 | NA | DNMBP-AS1 | | 100188954 |
| ENSG00000107566 | 1085.220064 | 0.014350606 | 0.0763608 | 0.8215404 | 0.9267922 | ERLIN1 | | 10613 |
| ENSG00000213341 | 851.2653381 | -0.01427452 | 0.0836552 | 0.8286565 | 0.929341 | CHUK | | 1147 |
| ENSG00000095485 | 533.6665537 | 0.000171037 | 0.0913509 | 0.9895488 | 0.9955163 | CWF19L1 | | 55280 |
| ENSG00000196072 | 514.9298889 | 0.045355488 | 0.0987269 | 0.518065 | 0.7471008 | BLOC1S2 | | 282991 |
| ENSG00000099194 | 1653.077985 | -0.698191331 | 0.2350854 | 0.0001386 | 0.0022733 | SCD | | 6319 |
| ENSG00000235823 | 94.03917509 | -0.224244731 | 0.324919 | 0.0253965 | 0.1222555 | OLMALINC | | 90271 |
| ENSG00000075826 | 78.64817792 | 0.010448109 | 0.1250805 | 0.839985 | 0.9344407 | SEC31B | | 25956 |
| ENSG00000166136 | 170.7388131 | -0.089802278 | 0.1382831 | 0.2111081 | 0.4679229 | NDUFB8 | | 4714 |
| ENSG00000166135 | 967.7982995 | -0.107377192 | 0.0894238 | 0.1273745 | 0.3505038 | HIF1AN | | 55662 |
| ENSG00000075891 | 1747.390615 | -0.625183367 | 0.0893712 | 1.85E-13 | 2.67E-11 | PAX2 | | 5076 |
| ENSG00000119906 | 992.541675 | 0.079695977 | 0.0866166 | 0.2406348 | 0.5060129 | SLF2 | | 55719 |
| ENSG00000055950 | 544.4346309 | -0.090872667 | 0.1028677 | 0.21862 | 0.4784003 | MRPL43 | | 84545 |
| ENSG00000095539 | 46.59580472 | 0.010713275 | 0.1295516 | 0.8038019 | 0.9184398 | SEMA4G | | 57715 |
| ENSG00000107815 | 791.6329727 | 0.445391754 | 0.1107456 | 5.01E-06 | 0.0001403 | TWNK | | 56652 |
| ENSG00000107816 | 335.2373345 | -0.006427307 | 0.1018416 | 0.9225193 | 0.9701942 | LZTS2 | | 84445 |
| ENSG00000186862 | 17.58812452 | 0.021491092 | 0.1359308 | 0.4556942 | NA | PDZD7 | | 79955 |
| ENSG00000107819 | 318.1909471 | 0.054470016 | 0.1123732 | 0.4387133 | 0.6882367 | SFXN3 | | 81855 |
| ENSG00000273162 | 26.27937448 | 0.054373529 | 0.1475737 | 0.1290078 | NA | NA | NA | |
| ENSG00000107821 | 38.86425358 | 0.016311721 | 0.1313256 | 0.6868894 | NA | KAZALD1 | | 81621 |
| ENSG00000138136 | 9.158640412 | 0.005505766 | 0.1352319 | 0.7918523 | NA | LBX1 | | 10660 |
| ENSG00000166167 | 396.1022829 | -0.120302844 | 0.1223277 | 0.1316737 | 0.3571947 | BTRC | | 8945 |
| ENSG00000166171 | 200.2246895 | 0.112117189 | 0.1460393 | 0.1480193 | 0.3820469 | DPCD | | 25911 |
| ENSG00000166169 | 253.6905418 | -0.005863295 | 0.1072462 | 0.9285887 | 0.9726302 | POLL | | 27343 |
| ENSG00000107829 | 213.6223453 | 0.109973523 | 0.1426521 | 0.1558286 | 0.394588 | FBXW4 | | 6468 |
| ENSG00000107833 | 594.3063669 | -0.019928291 | 0.0907011 | 0.7685218 | 0.900773 | NPM3 | | 10360 |
| ENSG00000198408 | 2083.99317 | -0.101929611 | 0.0694282 | 0.0894271 | 0.2821711 | OGA | | 10724 |
| ENSG00000120049 | 35.83482004 | -0.00945223 | 0.1312225 | 0.7996562 | NA | KCNIP2 | | 30819 |
| ENSG00000120029 | 493.0129415 | -0.138776491 | 0.1161728 | 0.0855646 | 0.2738302 | ARMH3 | | 79591 |
| ENSG00000166189 | 289.2545824 | 0.053910706 | 0.1144461 | 0.4396047 | 0.6888948 | HPS6 | | 79803 |
| ENSG00000198728 | 587.3216211 | 0.023301234 | 0.0897764 | 0.7232531 | 0.8752015 | LDB1 | | 8861 |
| ENSG00000148840 | 1589.130962 | 0.114431931 | 0.0787524 | 0.081231 | 0.2647853 | PPRC1 | | 23082 |
| ENSG00000166197 | 4902.064346 | 0.077311104 | 0.0563813 | 0.1326171 | 0.3586711 | NOLC1 | | 9221 |
| ENSG00000119915 | 14.56154081 | 0.009471272 | 0.134451 | 0.7224636 | NA | ELOVL3 | | 83401 |
| ENSG00000107862 | 1598.857567 | -0.027200369 | 0.0729256 | 0.6585966 | 0.8425525 | GBF1 | | 8729 |
| ENSG00000077150 | 2440.737138 | 0.271890475 | 0.0723401 | 3.18E-05 | 0.0006722 | NFKB2 | | 4791 |
| ENSG00000059915 | 11.23514066 | -0.010673974 | 0.1352931 | 0.6376405 | NA | PSD | | 5662 |
| ENSG00000107872 | 202.9351252 | 0.079862326 | 0.1289446 | 0.2648595 | 0.5327749 | FBXL15 | | 79176 |

| | | | | | | | |
|---|---|---|---|---|---|---|---|
| ENSG00000107874 | 469.4988771 | -0.028162097 | 0.0975347 | 0.6822526 | 0.8543094 | CUEDC2 | 79004 |
| ENSG00000269609 | 101.8150363 | 0.03666864 | 0.1263063 | 0.5262041 | 0.7534979 | C10orf95-AS | 100505761 |
| ENSG00000138111 | 201.4576905 | 0.050357965 | 0.1196015 | 0.4533396 | 0.6987417 | MFSD13A | 79847 |
| ENSG00000138107 | 2070.18929 | -0.118706588 | 0.0723991 | 0.0560791 | 0.2083524 | ACTR1A | 10121 |
| ENSG00000107882 | 273.7253276 | 0.002721112 | 0.1059094 | 0.9687559 | 0.9879551 | SUFU | 51684 |
| ENSG00000171206 | 568.6325306 | 0.26737418 | 0.1250267 | 0.0048108 | 0.0370031 | TRIM8 | 81603 |
| ENSG00000138175 | 484.8531348 | -0.054177773 | 0.0981528 | 0.4418192 | 0.6908823 | ARL3 | 403 |
| ENSG00000156398 | 194.5482799 | -0.069586754 | 0.1253688 | 0.3181802 | 0.5871506 | SFXN2 | 118980 |
| ENSG00000166272 | 377.4173822 | -0.027758676 | 0.1008618 | 0.6875826 | 0.8576358 | WBP1L | 54838 |
| ENSG00000166275 | 103.2917384 | -0.01284494 | 0.1218246 | 0.8176467 | 0.9254567 | BORCS7 | 119032 |
| ENSG00000148842 | 315.3096891 | -0.094300665 | 0.121131 | 0.2156356 | 0.4737333 | CNNM2 | 54805 |
| ENSG00000076685 | 1084.991794 | -0.069895342 | 0.0827808 | 0.2960887 | 0.566076 | NT5C2 | 22978 |
| ENSG00000156374 | 394.8404062 | 0.023296745 | 0.0988293 | 0.7358104 | 0.8838957 | PCGF6 | 84108 |
| ENSG00000148835 | 212.9389614 | -0.073580101 | 0.1249173 | 0.300041 | 0.5697989 | TAF5 | 6877 |
| ENSG00000173915 | 1152.445906 | -0.083605833 | 0.0840887 | 0.2153352 | 0.4734294 | ATP5MK | 84833 |
| ENSG00000148843 | 2036.431618 | 0.157967461 | 0.0739193 | 0.0131612 | 0.077138 | PDCD11 | 22984 |
| ENSG00000107960 | 110.093901 | 0.013006069 | 0.1213695 | 0.8201114 | 0.9260167 | STN1 | 79991 |
| ENSG00000065613 | 1294.221906 | 0.001439755 | 0.0728945 | 0.9821758 | 0.9933157 | SLK | 9748 |
| ENSG00000065618 | 54.70643408 | 0.160192881 | 0.2723213 | 0.0228052 | 0.1137921 | COL17A1 | 1308 |
| ENSG00000156384 | 155.4644522 | 0.435563582 | 0.2563374 | 0.0046129 | 0.0357079 | SFR1 | 119392 |
| ENSG00000148834 | 908.2929946 | 0.011562248 | 0.0802313 | 0.8595841 | 0.9433297 | GSTO1 | 9446 |
| ENSG00000148841 | 795.776058 | 0.333183165 | 0.1158537 | 0.0004675 | 0.0060741 | ITPRIP | 85450 |
| ENSG00000108039 | 1181.057928 | -0.021408052 | 0.0750612 | 0.7322948 | 0.8813039 | XPNPEP1 | 7511 |
| ENSG00000272160 | 12.51952211 | 0.006575593 | 0.1345788 | 0.7918136 | NA | NA | NA |
| ENSG00000148700 | 489.8726355 | -0.080922096 | 0.1053955 | 0.2703842 | 0.5381601 | ADD3 | 120 |
| ENSG00000119950 | 341.5849122 | 0.074197293 | 0.1201625 | 0.3022503 | 0.5715697 | MXI1 | 4601 |
| ENSG00000119953 | 1059.053843 | 0.065104816 | 0.0822289 | 0.331236 | 0.5990297 | SMNDC1 | 10285 |
| ENSG00000138166 | 1844.900011 | 0.631866214 | 0.0864487 | 1.88E-14 | 3.23E-12 | DUSP5 | 1847 |
| ENSG00000108055 | 3978.191514 | -0.148012201 | 0.0640642 | 0.0094722 | 0.0615035 | SMC3 | 9126 |
| ENSG00000203497 | 9.082940242 | 0.025243325 | 0.1385556 | 0.2205033 | NA | NA | NA |
| ENSG00000150593 | 173.6756462 | 0.029878036 | 0.1178964 | 0.637256 | 0.8285605 | PDCD4 | 27250 |
| ENSG00000214413 | 212.9468557 | -0.063700705 | 0.1227751 | 0.3558405 | 0.6192724 | BBIP1 | 92482 |
| ENSG00000108061 | 597.57478 | -0.01533315 | 0.0895409 | 0.8194789 | 0.925668 | SHOC2 | 8036 |
| ENSG00000119927 | 574.4233835 | -0.068254403 | 0.0972634 | 0.3370519 | 0.6048343 | GPAM | 57678 |
| ENSG00000023041 | 593.7264791 | -0.090382881 | 0.1029253 | 0.2191794 | 0.4793377 | ZDHHC6 | 64429 |
| ENSG00000151532 | 586.422411 | -0.0131327 | 0.0894201 | 0.845133 | 0.9366632 | VTI1A | 143187 |
| ENSG00000260917 | 77.57817653 | -0.021854419 | 0.1268717 | 0.6686872 | 0.847734 | LOC103344 | 103344931 |
| ENSG00000148737 | 183.7956103 | -0.011682115 | 0.1160969 | 0.8484175 | 0.9375782 | TCF7L2 | 6934 |
| ENSG00000165806 | 509.9240401 | 0.153365112 | 0.1174849 | 0.0629017 | 0.2245583 | CASP7 | 840 |
| ENSG00000198924 | 311.9096206 | -0.030162207 | 0.1045928 | 0.6598435 | 0.8426737 | DCLRE1A | 9937 |
| ENSG00000196865 | 762.8848596 | -0.15219956 | 0.1013703 | 0.0481608 | 0.1878532 | NHLRC2 | 374354 |
| ENSG00000043591 | 13.67301825 | -0.023883764 | 0.1374806 | 0.3249835 | NA | ADRB1 | 153 |
| ENSG00000165813 | 491.4246932 | -0.026675824 | 0.0942973 | 0.6969219 | 0.8616368 | CCDC186 | 55088 |
| ENSG00000169129 | 871.2392551 | -0.101568584 | 0.0902591 | 0.1495627 | 0.3848957 | AFAP1L2 | 84632 |
| ENSG00000099204 | 77.96779653 | 0.080614213 | 0.1525094 | 0.1688599 | 0.4145971 | ABLIM1 | 3983 |
| ENSG00000151553 | 277.3239973 | 0.007122355 | 0.1047424 | 0.9169479 | 0.9675582 | FHIP2A | 57700 |
| ENSG00000165832 | 644.7601791 | 0.022134839 | 0.0890973 | 0.7457462 | 0.8893165 | TRUB1 | 142940 |
| ENSG00000107518 | 69.3470455 | 0.283958381 | 0.5236376 | 0.0155426 | 0.0864125 | ATRNL1 | 26033 |
| ENSG00000165868 | 231.0190636 | 0.017047417 | 0.1094527 | 0.7970714 | 0.9157658 | HSPA12A | 259217 |
| ENSG00000187164 | 854.89002 | -0.111061135 | 0.0915704 | 0.1199079 | 0.3380749 | SHTN1 | 57698 |
| ENSG00000165650 | 935.2951042 | -0.131966008 | 0.0942504 | 0.0719853 | 0.2448634 | PDZD8 | 118987 |
| ENSG00000107560 | 562.5102286 | 0.058055398 | 0.0975569 | 0.4116733 | 0.6661689 | RAB11FIP2 | 22841 |
| ENSG00000177640 | 18.62859772 | 0.032932006 | 0.1390144 | 0.2708856 | NA | CASC2 | 255082 |
| ENSG00000165669 | 498.2049332 | -0.012922935 | 0.0933582 | 0.8485833 | 0.9375994 | FAM204A | 63877 |
| ENSG00000151893 | 905.1505088 | -0.259854467 | 0.1070823 | 0.0025586 | 0.0228353 | CACUL1 | 143384 |
| ENSG00000188613 | 14.99407718 | -0.026622332 | 0.1376479 | 0.3244643 | NA | NANOS1 | 340719 |
| ENSG00000107581 | 8833.714403 | -0.085395867 | 0.052048 | 0.0749393 | 0.251253 | EIF3A | 8661 |
| ENSG00000119979 | 382.4613543 | -0.293661289 | 0.1522434 | 0.0060659 | 0.0437114 | DENND10 | 404636 |
| ENSG00000183605 | 451.1466003 | -0.045777685 | 0.1006381 | 0.5132372 | 0.743625 | SFXN4 | 119559 |
| ENSG00000165672 | 3019.768494 | -0.195698742 | 0.067746 | 0.0011894 | 0.0128525 | PRDX3 | 10935 |
| ENSG00000198873 | 246.6526402 | 0.058544539 | 0.116494 | 0.4057181 | 0.6618984 | GRK5 | 2869 |
| ENSG00000148908 | 572.2150244 | 0.001014215 | 0.0904988 | 0.9890041 | 0.9955163 | RGS10 | 6001 |
| ENSG00000151923 | 1926.675842 | -0.063901016 | 0.0718282 | 0.2964392 | 0.5664726 | TIAL1 | 7073 |
| ENSG00000151929 | 738.2870219 | -0.008309255 | 0.0861923 | 0.8887897 | 0.9546623 | BAG3 | 9531 |
| ENSG00000198825 | 309.4355072 | -0.042282924 | 0.1071279 | 0.5424928 | 0.7657123 | INPP5F | 22876 |
| ENSG00000197771 | 2404.233207 | -0.140042493 | 0.0690899 | 0.020119 | 0.1042633 | MCMBP | 79892 |
| ENSG00000107651 | 1290.168553 | -0.260248908 | 0.0933754 | 0.0009445 | 0.0105573 | SEC23IP | 11196 |
| ENSG00000220842 | 529.9019964 | -0.19330415 | 0.1296947 | 0.030591 | 0.1401263 | RPL21P16 | 729402 |
| ENSG00000120008 | 892.9657496 | -0.065481854 | 0.0846483 | 0.3275705 | 0.5956338 | WDR11 | 55717 |
| ENSG00000107669 | 445.0525844 | -0.220178005 | 0.1380936 | 0.0197761 | 0.1028623 | ATE1 | 11101 |

| | | | | | | | | |
|---|---|---|---|---|---|---|---|---|
| ENSG00000107672 | 539.5628544 | -0.116236978 | 0.1075961 | 0.1306828 | 0.3554145 | NSMCE4A | | 54780 |
| ENSG00000138162 | 467.1995249 | 0.044270099 | 0.0994613 | 0.5284438 | 0.7549401 | TACC2 | | 10579 |
| ENSG00000107679 | 764.5881414 | -0.030696463 | 0.085155 | 0.6453984 | 0.834965 | PLEKHA1 | | 59338 |
| ENSG00000138161 | 58.44984098 | 0.062225247 | 0.1448954 | 0.2192589 | 0.4793377 | CUZD1 | | 50624 |
| ENSG00000213185 | 19.00730221 | -0.015841843 | 0.1346061 | 0.58966 | NA | FAM24B | | 196792 |
| ENSG00000255624 | 128.8486948 | 0.3469822 | 0.2997847 | 0.0122695 | 0.0734222 | C10orf88B | | 399815 |
| ENSG00000119965 | 235.7730971 | 0.170719705 | 0.1719444 | 0.0612689 | 0.2208215 | C10orf88 | | 80007 |
| ENSG00000179988 | 81.89642907 | 0.031845028 | 0.1279692 | 0.5534543 | 0.7742395 | PSTK | | 118672 |
| ENSG00000095574 | 390.955482 | 0.074704029 | 0.1097328 | 0.3086444 | 0.5780464 | IKZF5 | | 64376 |
| ENSG00000196177 | 358.2884524 | -0.243818664 | 0.1542645 | 0.0161637 | 0.0887673 | ACADSB | | 36 |
| ENSG00000154473 | 3342.360331 | -0.035944123 | 0.0608058 | 0.5081921 | 0.7401841 | BUB3 | | 9184 |
| ENSG00000182022 | 706.3837324 | -0.379372349 | 0.1152781 | 0.0001009 | 0.0017513 | CHST15 | | 51363 |
| ENSG00000065154 | 1316.368975 | -0.120067168 | 0.081023 | 0.0726497 | 0.2462867 | OAT | | 4942 |
| ENSG00000107902 | 20.56023489 | -0.036653796 | 0.1398915 | 0.2473873 | NA | LHPP | | 64077 |
| ENSG00000189319 | 328.0995153 | 0.114763174 | 0.1271549 | 0.1493818 | 0.3845148 | FAM53B | | 9679 |
| ENSG00000203791 | 128.3940301 | -0.072823162 | 0.137578 | 0.2620748 | 0.5298047 | EEF1AKMT2 | | 399818 |
| ENSG00000165660 | 603.9797118 | 0.093330188 | 0.1002125 | 0.2044168 | 0.4594559 | ABRAXAS2 | | 23172 |
| ENSG00000249456 | 20.2156282 | 0.031671832 | 0.1384906 | 0.2960783 | NA | NA | NA | |
| ENSG00000019995 | 496.6869065 | -0.052480922 | 0.0987875 | 0.4559724 | 0.7007253 | ZRANB1 | | 54764 |
| ENSG00000175029 | 1070.232026 | -0.293295486 | 0.0978937 | 0.0004027 | 0.0054531 | CTBP2 | | 1488 |
| ENSG00000107938 | 598.385203 | 0.019690461 | 0.0911923 | 0.7734751 | 0.9041206 | EDRF1 | | 26098 |
| ENSG00000188690 | 770.9328728 | 0.13172795 | 0.0977583 | 0.0784297 | 0.2585037 | UROS | | 7390 |
| ENSG00000107949 | 1495.461783 | -0.297957617 | 0.0893303 | 0.0001256 | 0.0020955 | BCCIP | | 56647 |
| ENSG00000089876 | 454.1971518 | 0.05141104 | 0.0992408 | 0.4668491 | 0.7094916 | DHX32 | | 55760 |
| ENSG00000150760 | 1311.910791 | -0.151332472 | 0.0892537 | 0.0352959 | 0.1538399 | DOCK1 | | 1793 |
| ENSG00000132334 | 230.733067 | -0.078305001 | 0.1247031 | 0.2789186 | 0.5470196 | PTPRE | | 5791 |
| ENSG00000227076 | 25.83857862 | 0.048876337 | 0.1444669 | 0.1684693 | NA | NA | NA | |
| ENSG00000148773 | 14767.03119 | -0.199763189 | 0.0539859 | 6.78E-05 | 0.0012698 | MKI67 | | 4288 |
| ENSG00000170430 | 343.8879012 | -0.041878624 | 0.104105 | 0.5471969 | 0.7690111 | MGMT | | 4255 |
| ENSG00000237489 | 10.50229187 | 0.03691728 | 0.1423205 | 0.0961504 | NA | C10orf143 | | 387723 |
| ENSG00000108010 | 2640.828897 | -0.188686572 | 0.0698784 | 0.0022475 | 0.0206552 | GLRX3 | | 10539 |
| ENSG00000175470 | 687.4537143 | -0.106336648 | 0.0997722 | 0.1512553 | 0.3877924 | PPP2R2D | | 55844 |
| ENSG00000176171 | 1067.588331 | -0.048370233 | 0.0794642 | 0.4559521 | 0.7007253 | BNIP3 | | 664 |
| ENSG00000165752 | 634.5765347 | 0.028014565 | 0.0887685 | 0.6825416 | 0.8544463 | STK32C | | 282974 |
| ENSG00000148814 | 79.14455007 | 0.036273391 | 0.1301429 | 0.4923875 | 0.7283747 | LRRC27 | | 80313 |
| ENSG00000171813 | 227.9297555 | 0.311131171 | 0.202408 | 0.010349 | 0.0649931 | PWWP2B | | 170394 |
| ENSG00000068383 | 559.2999483 | -0.157766479 | 0.1143785 | 0.0539507 | 0.203093 | INPP5A | | 3632 |
| ENSG00000171798 | 15.80922755 | 0.003733236 | 0.1338181 | 0.8946843 | NA | KNDC1 | | 85442 |
| ENSG00000151651 | 25.82165127 | -0.031365553 | 0.1369861 | 0.3672807 | NA | ADAM8 | | 101 |
| ENSG00000130640 | 1826.094064 | -0.100633945 | 0.0716528 | 0.1007247 | 0.303395 | TUBGCP2 | | 10844 |
| ENSG00000235245 | 40.8517372 | 0.026004797 | 0.1328312 | 0.5345945 | NA | NA | NA | |
| ENSG00000198546 | 660.0137477 | -0.360276157 | 0.116266 | 0.0002029 | 0.0030954 | ZNF511 | | 118472 |
| ENSG00000148803 | 273.172618 | 0.052046856 | 0.1128579 | 0.4524958 | 0.6987417 | FUOM | | 282969 |
| ENSG00000127884 | 2504.743578 | -0.012333267 | 0.063679 | 0.8259609 | 0.9286016 | ECHS1 | | 1892 |
| ENSG00000148832 | 92.42108521 | 0.016899532 | 0.1234339 | 0.7597873 | 0.8969311 | PAOX | | 196743 |
| ENSG00000148824 | 297.63863 | 0.09932889 | 0.1252144 | 0.1960625 | 0.4487959 | MTG1 | | 92170 |
| ENSG00000214279 | 43.04159582 | 0.001294462 | 0.1297032 | 0.9766805 | 0.9912634 | SCART1 | | 619207 |
| ENSG00000130649 | 8.365690195 | 0.025324098 | 0.1387485 | 0.1978985 | NA | CYP2E1 | | 1571 |
| ENSG00000230724 | 73.80505505 | -0.010585435 | 0.1253659 | 0.8334068 | 0.9305394 | NA | NA | |
| ENSG00000177951 | 303.0950058 | 0.033786508 | 0.1055525 | 0.6246746 | 0.8202204 | BET1L | | 51272 |
| ENSG00000177963 | 1878.879785 | 0.027092182 | 0.0681749 | 0.6005257 | 0.8069949 | RIC8A | | 60626 |
| ENSG00000142082 | 174.2407963 | 0.03909861 | 0.1180284 | 0.5489995 | 0.7704064 | SIRT3 | | 23410 |
| ENSG00000185627 | 1874.367268 | -0.115792066 | 0.0738463 | 0.0650809 | 0.2302969 | PSMD13 | | 5719 |
| ENSG00000142102 | 385.8509437 | 0.018736412 | 0.1041511 | 0.7845527 | 0.9098848 | PGGHG | | 80162 |
| ENSG00000185201 | 14.15534735 | 0.021879515 | 0.1368462 | 0.3832357 | NA | IFITM2 | | 10581 |
| ENSG00000254910 | 8.843919805 | 0.002935929 | 0.1351587 | 0.8874474 | NA | NA | NA | |
| ENSG00000142089 | 903.6704207 | 0.006895727 | 0.0897373 | 0.9197929 | 0.9690041 | IFITM3 | | 10410 |
| ENSG00000182272 | 561.5890456 | 0.074509341 | 0.0978375 | 0.2954908 | 0.5655625 | B4GALNT4 | | 338707 |
| ENSG00000184363 | 916.9779716 | -0.100578186 | 0.0895234 | 0.1521071 | 0.3894703 | PKP3 | | 11187 |
| ENSG00000185187 | 183.4248974 | -0.035957013 | 0.118489 | 0.5739578 | 0.7881967 | SIGIRR | | 59307 |
| ENSG00000185101 | 94.28984747 | 0.055312048 | 0.1348867 | 0.3376627 | 0.6054088 | ANO9 | | 338440 |
| ENSG00000174915 | 789.6971553 | -0.043841891 | 0.0858077 | 0.5157983 | 0.7452598 | PTDSS2 | | 81490 |
| ENSG00000023191 | 2026.91974 | 0.116583226 | 0.0750173 | 0.0666707 | 0.2334472 | RNH1 | | 6050 |
| ENSG00000174775 | 1679.109343 | -0.000976084 | 0.069113 | 0.9861166 | 0.9942882 | HRAS | | 3265 |
| ENSG00000161328 | 14.13576997 | 0.039119396 | 0.142351 | 0.1394552 | NA | LRRC56 | | 115399 |
| ENSG00000185522 | 42.9810355 | -0.010147802 | 0.1298407 | 0.7948414 | 0.9144629 | LMNTD2 | | 256329 |
| ENSG00000099849 | 609.4986934 | 0.092855777 | 0.0998046 | 0.2059854 | 0.4609417 | RASSF7 | | 8045 |
| ENSG00000070047 | 1619.71856 | 0.008790493 | 0.0701435 | 0.8846916 | 0.9535046 | PHRF1 | | 57661 |
| ENSG00000185507 | 162.3583283 | 0.053981042 | 0.1252836 | 0.4076385 | 0.6640113 | IRF7 | | 3665 |
| ENSG00000069696 | 9.226247648 | -0.001021472 | 0.1349648 | 0.9586128 | NA | DRD4 | | 1815 |

| | | | | | | | | |
|---|---|---|---|---|---|---|---|---|
| ENSG00000177030 | 417.5535299 | 0.033929702 | 0.0982303 | 0.6248609 | 0.8202204 | DEAF1 | | 10522 |
| ENSG00000177106 | 1532.258141 | -0.16187245 | 0.0804673 | 0.0166908 | 0.0906816 | EPS8L2 | | 64787 |
| ENSG00000177042 | 197.1159657 | 0.117715912 | 0.1486218 | 0.1353825 | 0.3622916 | TMEM80 | | 283232 |
| ENSG00000177156 | 1657.651709 | -0.145419639 | 0.0776756 | 0.0266076 | 0.1267553 | TALDO1 | | 6888 |
| ENSG00000177225 | 1269.62622 | 0.059137449 | 0.0766031 | 0.3545106 | 0.6187516 | GATD1 | | 347862 |
| ENSG00000255284 | 22.72270936 | 0.033631218 | 0.1384872 | 0.3040422 | NA | GATD1-DT | | 171391 |
| ENSG00000177236 | 9.311832099 | 0.017658989 | 0.1366172 | 0.4126519 | NA | NA | NA | |
| ENSG00000177542 | 613.8283923 | -0.024819484 | 0.0890119 | 0.713478 | 0.870525 | SLC25A22 | | 79751 |
| ENSG00000177595 | 358.4672305 | 0.064058922 | 0.1099257 | 0.3735577 | 0.6352333 | PIDD1 | | 55367 |
| ENSG00000177600 | 6255.215515 | 0.051007152 | 0.0561833 | 0.3188944 | 0.5875404 | RPLP2 | | 6181 |
| ENSG00000177666 | 700.3679723 | 0.046092282 | 0.0880803 | 0.4991497 | 0.7342359 | PNPLA2 | | 57104 |
| ENSG00000177685 | 31.9774786 | -0.025245586 | 0.1342908 | 0.4970388 | NA | CRACR2B | | 283229 |
| ENSG00000177697 | 2764.178525 | 0.071235932 | 0.0641287 | 0.2085265 | 0.4650913 | CD151 | | 977 |
| ENSG00000177700 | 2494.068891 | -0.197312762 | 0.0871882 | 0.0063885 | 0.0456555 | POLR2L | | 5441 |
| ENSG00000214063 | 1004.960823 | -0.029016789 | 0.0824566 | 0.6589885 | 0.842576 | TSPAN4 | | 7106 |
| ENSG00000250397 | 8.210066913 | 0.041189369 | 0.1447779 | 0.033239 | NA | NA | NA | |
| ENSG00000177830 | 642.1918134 | -0.00485601 | 0.0874081 | 0.9426229 | 0.9777798 | CHID1 | | 66005 |
| ENSG00000183020 | 1255.189542 | -0.051559032 | 0.0762166 | 0.4162814 | 0.6698695 | AP2A2 | | 161 |
| ENSG00000078902 | 612.6605482 | 0.121828962 | 0.1069191 | 0.11434 | 0.328805 | TOLLIP | | 54472 |
| ENSG00000174672 | 331.4933662 | -0.087398684 | 0.1170421 | 0.2442865 | 0.5100108 | BRSK2 | | 9024 |
| ENSG00000182208 | 333.3623122 | 0.047850512 | 0.106273 | 0.488395 | 0.725912 | MOB2 | | 81532 |
| ENSG00000184545 | 131.1614372 | 0.585671074 | 0.2959552 | 0.0019676 | 0.0186704 | DUSP8 | | 1850 |
| ENSG00000117984 | 1597.828208 | 0.121630664 | 0.0767356 | 0.0598829 | 0.2176391 | CTSD | | 1509 |
| ENSG00000235027 | 10.99169992 | -0.010943866 | 0.1352557 | 0.6355363 | NA | PRADX | | 126568849 |
| ENSG00000229512 | 14.05287741 | 0.00190846 | 0.1341074 | 0.943569 | NA | NA | NA | |
| ENSG00000214026 | 562.4033202 | 0.028076699 | 0.0926321 | 0.682931 | 0.8544725 | MRPL23 | | 6150 |
| ENSG00000130600 | 124.488463 | -2.282435415 | 0.2933524 | 3.70E-16 | 7.44E-14 | H19 | | 283120 |
| ENSG00000167244 | 12.19743639 | 0.030331104 | 0.139533 | 0.2027618 | NA | IGF2 | | 3481 |
| ENSG00000236264 | 26.30570877 | 0.012942481 | 0.1327585 | 0.7099807 | NA | NA | NA | |
| ENSG00000110651 | 1389.51747 | -0.102936005 | 0.0779535 | 0.1133431 | 0.3268581 | CD81 | | 975 |
| ENSG00000184281 | 597.4118631 | 0.069027194 | 0.0956148 | 0.3312843 | 0.5990297 | TSSC4 | | 10078 |
| ENSG00000110628 | 88.66569046 | 0.676749632 | 0.3324081 | 0.0015913 | 0.0160107 | SLC22A18 | | 5002 |
| ENSG00000181649 | 679.3821442 | 0.43357177 | 0.1296313 | 6.79E-05 | 0.0012698 | PHLDA2 | | 7262 |
| ENSG00000205531 | 2441.011224 | -0.059500247 | 0.0658347 | 0.3017005 | 0.5710237 | NAP1L4 | | 4676 |
| ENSG00000110619 | 922.1532021 | 0.100025415 | 0.0912341 | 0.160854 | 0.4026002 | CARS1 | | 833 |
| ENSG00000021762 | 236.7574784 | 0.012250892 | 0.1082678 | 0.8560451 | 0.9418544 | OSBPL5 | | 114879 |
| ENSG00000005801 | 469.7565999 | 0.087440984 | 0.1055156 | 0.2408988 | 0.506323 | ZNF195 | | 7748 |
| ENSG00000129749 | 15.50585932 | 0.025824853 | 0.1374621 | 0.3378734 | NA | CHRNA10 | | 57053 |
| ENSG00000110713 | 2824.62953 | -0.116995057 | 0.0652009 | 0.0415178 | 0.1714886 | NUP98 | | 4928 |
| ENSG00000148985 | 225.9149199 | -0.040999233 | 0.1132729 | 0.5440277 | 0.7661909 | PGAP2 | | 27315 |
| ENSG00000250404 | 15.78630819 | -0.038170649 | 0.1419042 | 0.1500014 | NA | NA | NA | |
| ENSG00000177105 | 425.7114636 | 0.043414756 | 0.1004151 | 0.535666 | 0.7600689 | RHOG | | 391 |
| ENSG00000167323 | 553.5444099 | 0.004510514 | 0.0905107 | 0.9478285 | 0.9791797 | STIM1 | | 6786 |
| ENSG00000229368 | 17.52161285 | -0.011531955 | 0.134138 | 0.6870977 | NA | NA | NA | |
| ENSG00000167325 | 2395.52546 | -0.139575878 | 0.0688417 | 0.0201927 | 0.1045381 | RRM1 | | 6240 |
| ENSG00000132109 | 283.0268476 | -0.161313846 | 0.1526323 | 0.068683 | 0.2375735 | TRIM21 | | 6737 |
| ENSG00000167333 | 177.7156445 | 0.120896969 | 0.1593599 | 0.1196307 | 0.3376193 | TRIM68 | | 55128 |
| ENSG00000132256 | 371.894095 | 0.016847209 | 0.0990835 | 0.8067933 | 0.9196987 | TRIM5 | | 85363 |
| ENSG00000051009 | 272.4891796 | 0.024397935 | 0.1066853 | 0.717332 | 0.8721019 | FHIP1B | | 84067 |
| ENSG00000166311 | 136.370253 | 0.132300557 | 0.1758623 | 0.0925473 | 0.2876914 | SMPD1 | | 6609 |
| ENSG00000166313 | 513.0647272 | 0.113885576 | 0.1145021 | 0.1443893 | 0.376779 | APBB1 | | 322 |
| ENSG00000110171 | 302.4852123 | -0.007877046 | 0.1033043 | 0.9050834 | 0.9623051 | TRIM3 | | 10612 |
| ENSG00000132254 | 893.5250283 | 0.091241519 | 0.0896142 | 0.1934807 | 0.4454937 | ARFIP2 | | 23647 |
| ENSG00000132286 | 438.247689 | 0.009587591 | 0.0984456 | 0.8892573 | 0.9549168 | TIMM10B | | 26515 |
| ENSG00000179532 | 163.8524449 | 0.561352715 | 0.2366298 | 0.0008986 | 0.010183 | DNHD1 | | 144132 |
| ENSG00000132275 | 342.4856062 | 0.344908441 | 0.1613481 | 0.0029704 | 0.0255123 | RRP8 | | 23378 |
| ENSG00000166333 | 2017.994977 | -0.080324843 | 0.0697564 | 0.1812009 | 0.43073 | ILK | | 3611 |
| ENSG00000166337 | 448.487587 | -0.007145916 | 0.0964619 | 0.9152531 | 0.9662935 | TAF10 | | 6881 |
| ENSG00000166340 | 1275.095094 | 0.141717163 | 0.0838999 | 0.0398974 | 0.1674554 | TPP1 | | 1200 |
| ENSG00000166341 | 15.09100539 | -0.002788819 | 0.1340093 | 0.9135335 | NA | DCHS1 | | 8642 |
| ENSG00000158042 | 616.9732964 | 0.0905152 | 0.1023385 | 0.2200879 | 0.4795932 | MRPL17 | | 63875 |
| ENSG00000254838 | 12.56877576 | 0.007812963 | 0.1349802 | 0.7367867 | NA | GVINP1 | | 387751 |
| ENSG00000149054 | 364.4742444 | 0.169387579 | 0.1389531 | 0.0559203 | 0.2080666 | ZNF215 | | 7762 |
| ENSG00000149050 | 23.87926992 | 0.011519274 | 0.1330626 | 0.7284438 | NA | ZNF214 | | 7761 |
| ENSG00000166387 | 87.26076566 | 0.024449119 | 0.125325 | 0.6524311 | 0.8389824 | PPFIBP2 | | 8495 |
| ENSG00000166394 | 20.79632775 | 0.03220444 | 0.1383211 | 0.306364 | NA | CYB5R2 | | 51700 |
| ENSG00000175390 | 1597.65708 | -0.028554627 | 0.0718799 | 0.6397646 | 0.8306614 | EIF3F | | 8665 |
| ENSG00000166436 | 134.2070999 | 0.013589903 | 0.1185019 | 0.8222384 | 0.9271409 | TRIM66 | | 9866 |
| ENSG00000166441 | 7372.332343 | -0.188479055 | 0.0609659 | 0.0006621 | 0.0080313 | RPL27A | | 6157 |
| ENSG00000166444 | 35.60895195 | 0.064826584 | 0.1516822 | 0.1267265 | NA | DENND2B | | 6764 |

| | | | | | | | |
|---|---|---|---|---|---|---|---|
| ENSG00000166452 | 413.2091096 | 0.107541339 | 0.1171624 | 0.1665971 | 0.411682 | AKIP1 | 56672 |
| ENSG00000175348 | 505.3079461 | 0.063075828 | 0.1005037 | 0.3780547 | 0.6395077 | TMEM9B | 56674 |
| ENSG00000254860 | 11.72336678 | 0.024149898 | 0.1376381 | 0.3152448 | NA | TMEM9B-AS | 493900 |
| ENSG00000175352 | 75.6601049 | 0.120105209 | 0.1896425 | 0.0706584 | 0.2420798 | NRIP3 | 56675 |
| ENSG00000184014 | 1336.163653 | 0.033640132 | 0.0743817 | 0.5912995 | 0.8006223 | DENND5A | 23258 |
| ENSG00000166471 | 873.8608103 | -0.097361453 | 0.0950784 | 0.1771108 | 0.4266511 | TMEM41B | 440026 |
| ENSG00000205339 | 5705.189303 | -0.177922612 | 0.0577802 | 0.0007574 | 0.0089633 | IPO7 | 10527 |
| ENSG00000268403 | 14.71890111 | 0.010477074 | 0.1346215 | 0.6910589 | NA | LOC644656 | 644656 |
| ENSG00000166478 | 495.0237697 | 0.31465552 | 0.1361737 | 0.0023673 | 0.0215191 | ZNF143 | 7702 |
| ENSG00000166483 | 914.2590874 | -0.046554541 | 0.081821 | 0.47767 | 0.7167198 | WEE1 | 7465 |
| ENSG00000133789 | 1184.208267 | 0.07438133 | 0.0786744 | 0.2543329 | 0.5211459 | SWAP70 | 23075 |
| ENSG00000245522 | 13.37038646 | 0.032803304 | 0.1399878 | 0.2023051 | NA | LINC02709 | 440028 |
| ENSG00000246273 | 93.34134309 | 0.015564689 | 0.1232371 | 0.7782804 | 0.9075471 | SBF2-AS1 | 283104 |
| ENSG00000133812 | 681.2848407 | -0.13162175 | 0.1028891 | 0.0853159 | 0.2734087 | SBF2 | 81846 |
| ENSG00000148926 | 1010.992328 | -0.299772366 | 0.1016379 | 0.0004445 | 0.0058739 | ADM | 133 |
| ENSG00000133805 | 29.69311594 | 0.057773067 | 0.1494365 | 0.1132528 | NA | AMPD3 | 272 |
| ENSG00000110315 | 355.2500943 | -0.005201814 | 0.1024136 | 0.9358972 | 0.9753939 | RNF141 | 50862 |
| ENSG00000198730 | 1053.073002 | -0.053098287 | 0.0795216 | 0.4159776 | 0.6697358 | CTR9 | 9646 |
| ENSG00000110321 | 12597.66999 | -0.262657176 | 0.0539338 | 2.07E-07 | 8.78E-06 | EIF4G2 | 1982 |
| ENSG00000236287 | 511.0680244 | -0.025890075 | 0.0968848 | 0.7058034 | 0.8670449 | ZBED5 | 58486 |
| ENSG00000247271 | 31.61981959 | -0.004317235 | 0.1312746 | 0.9044642 | NA | ZBED5-AS1 | 729013 |
| ENSG00000110328 | 35.75078264 | 0.02670736 | 0.1340478 | 0.4962759 | NA | GALNT18 | 374378 |
| ENSG00000170242 | 1285.697982 | 0.002006433 | 0.0750226 | 0.9753226 | 0.9907595 | USP47 | 55031 |
| ENSG00000050165 | 2147.003031 | -0.226661904 | 0.0773421 | 0.000817 | 0.0094581 | DKK3 | 27122 |
| ENSG00000254991 | 32.65191848 | 0.030383427 | 0.1356592 | 0.4228082 | NA | NA | NA |
| ENSG00000133816 | 4593.166946 | -0.051930542 | 0.0649699 | 0.362072 | 0.6253679 | MICAL2 | 9645 |
| ENSG00000254680 | 30.10418121 | 0.015795408 | 0.13282 | 0.6607297 | NA | NA | NA |
| ENSG00000133808 | 32.20610153 | -0.064017863 | 0.1522216 | 0.1104694 | NA | NA | NA |
| ENSG00000197702 | 678.219012 | -0.077464075 | 0.0948637 | 0.2710539 | 0.5387409 | PARVA | 55742 |
| ENSG00000187079 | 3286.17817 | -0.067652181 | 0.0676228 | 0.2511154 | 0.5176071 | TEAD1 | 7003 |
| ENSG00000251381 | 222.1946087 | 0.457822859 | 0.2116272 | 0.0018002 | 0.0175237 | LINC00958 | 100506305 |
| ENSG00000133794 | 171.8732671 | 0.007424554 | 0.114073 | 0.9076809 | 0.963787 | BMAL1 | 406 |
| ENSG00000148925 | 478.2732489 | 0.051018462 | 0.1003463 | 0.4713459 | 0.7119839 | BTBD10 | 84280 |
| ENSG00000197601 | 1788.272482 | -0.01663137 | 0.067327 | 0.7743607 | 0.9046394 | FAR1 | 84188 |
| ENSG00000133818 | 1438.373324 | -0.051509326 | 0.0775336 | 0.4213076 | 0.6737677 | RRAS2 | 22800 |
| ENSG00000129083 | 2094.100072 | -0.208464916 | 0.0738868 | 0.0013152 | 0.0139026 | COPB1 | 1315 |
| ENSG00000129084 | 428.2527871 | 0.021647219 | 0.0964161 | 0.753227 | 0.8932431 | PSMA1 | 5682 |
| ENSG00000152270 | 166.8521481 | -0.05138966 | 0.1232156 | 0.4315482 | 0.6828658 | PDE3B | 5140 |
| ENSG00000186104 | 161.0992028 | 0.071247727 | 0.1329143 | 0.2894776 | 0.5595695 | CYP2R1 | 120227 |
| ENSG00000110696 | 2513.780736 | -0.008253948 | 0.0634021 | 0.8823592 | 0.9525622 | C11orf58 | 10944 |
| ENSG00000244398 | 627.2762099 | -0.098565116 | 0.0995409 | 0.1804313 | 0.4299157 | RPL36AP37 | 729362 |
| ENSG00000110700 | 4797.668981 | -0.004095772 | 0.0654791 | 0.8977399 | 0.9594787 | RPS13 | 6207 |
| ENSG00000272034 | 10.55741791 | -0.011113537 | 0.1354768 | 0.6132291 | NA | SNORD14A | 26822 |
| ENSG00000011405 | 1043.051046 | -0.234792844 | 0.0971193 | 0.0031798 | 0.026683 | PIK3C2A | 5286 |
| ENSG00000070081 | 368.1711479 | -0.091959317 | 0.1202703 | 0.223336 | 0.4836193 | NUCB2 | 4925 |
| ENSG00000188211 | 268.7341797 | 0.389075398 | 0.1983589 | 0.0034395 | 0.0283536 | NCR3LG1 | 374383 |
| ENSG00000260196 | 40.15017347 | 0.074207382 | 0.1576638 | 0.1014626 | NA | NA | NA |
| ENSG00000187486 | 15.65159832 | -0.024559994 | 0.1371832 | 0.3554548 | NA | KCNJ11 | 3767 |
| ENSG00000129158 | 104.8777597 | -0.013028174 | 0.1215691 | 0.816349 | 0.9252377 | SERGEF | 26297 |
| ENSG00000129167 | 9.733442944 | 0.019508966 | 0.1369337 | 0.3717289 | NA | TPH1 | 7166 |
| ENSG00000166788 | 466.5909195 | -0.036258737 | 0.0958857 | 0.6005172 | 0.8069949 | SAAL1 | 113174 |
| ENSG00000173432 | 103.7773331 | -0.055063859 | 0.1329375 | 0.353554 | 0.6187516 | SAA1 | 6288 |
| ENSG00000110756 | 1208.210303 | 0.319948786 | 0.0991798 | 0.0001644 | 0.0026097 | HPS5 | 11234 |
| ENSG00000110768 | 1123.99453 | 0.237315864 | 0.0948778 | 0.0025293 | 0.0226569 | GTF2H1 | 2965 |
| ENSG00000134333 | 25608.72349 | -0.149246443 | 0.0525074 | 0.0021666 | 0.0201178 | LDHA | 3939 |
| ENSG00000256006 | 13.65527287 | -0.016800639 | 0.1356227 | 0.5130979 | NA | NA | NA |
| ENSG00000074319 | 515.3495454 | -0.013937344 | 0.0919617 | 0.8368105 | 0.9322636 | TSG101 | 7251 |
| ENSG00000151116 | 338.3873707 | -0.031336794 | 0.1031069 | 0.6490317 | 0.8366057 | UEVLD | 55293 |
| ENSG00000247595 | 15.04384405 | -0.002540991 | 0.1340098 | 0.9209081 | NA | MISFA | 100506540 |
| ENSG00000179119 | 565.4355995 | 0.212466743 | 0.1209254 | 0.0165276 | 0.0901725 | SPTY2D1 | 144108 |
| ENSG00000177054 | 391.6738214 | -0.042739733 | 0.1026194 | 0.5406245 | 0.7640695 | ZDHHC13 | 54503 |
| ENSG00000129173 | 526.3246666 | 0.535549941 | 0.1295676 | 2.54E-06 | 7.64E-05 | E2F8 | 79733 |
| ENSG00000166833 | 697.0188731 | 0.69753531 | 0.114418 | 6.90E-11 | 5.90E-09 | NAV2 | 89797 |
| ENSG00000270607 | 36.28384211 | 0.100514477 | 0.1850937 | 0.035056 | NA | NA | NA |
| ENSG00000109854 | 442.7567837 | 0.055409725 | 0.1031848 | 0.4365043 | 0.686893 | HTATIP2 | 10553 |
| ENSG00000185238 | 466.0321783 | 0.058864084 | 0.100054 | 0.4091648 | 0.6643076 | PRMT3 | 10196 |
| ENSG00000183161 | 93.21306331 | -0.005394719 | 0.1228626 | 0.9186857 | 0.9683988 | FANCF | 2188 |
| ENSG00000198168 | 485.6086429 | -0.062243116 | 0.1028806 | 0.3839594 | 0.6447633 | SVIP | 258010 |
| ENSG00000187398 | 71.7320992 | -0.016154994 | 0.127341 | 0.7378721 | 0.8854328 | LUZP2 | 338645 |
| ENSG00000254560 | 64.21684771 | 0.042257937 | 0.1343314 | 0.398115 | 0.6572977 | BBOX1-AS1 | 103695435 |

| | | | | | | | | |
|---|---|---|---|---|---|---|---|---|
| ENSG00000109881 | 720.9956297 | -0.085215426 | 0.0944291 | 0.2326661 | 0.4951049 | CCDC34 | | 91057 |
| ENSG00000205213 | 1000.453726 | -0.074374142 | 0.0874281 | 0.2779755 | 0.5464543 | LGR4 | | 55366 |
| ENSG00000148943 | 1607.627864 | 0.056323706 | 0.0719724 | 0.35863 | 0.6219981 | LIN7C | | 55327 |
| ENSG00000245573 | 12.38261633 | 0.01887509 | 0.1363401 | 0.4380121 | NA | BDNF-AS | | 497258 |
| ENSG00000176697 | 69.45631421 | 0.966105815 | 0.3883425 | 0.0004777 | 0.0061936 | BDNF | | 627 |
| ENSG00000121621 | 920.7984396 | -0.110697998 | 0.0914866 | 0.1208129 | 0.3399699 | KIF18A | | 81930 |
| ENSG00000169519 | 357.9693199 | 0.085996607 | 0.1168043 | 0.2502789 | 0.5163897 | METTL15 | | 196074 |
| ENSG00000152219 | 693.194479 | 0.19595399 | 0.1153663 | 0.021581 | 0.1093182 | ARL14EP | | 120534 |
| ENSG00000066382 | 9.054843067 | -0.010220017 | 0.1355231 | 0.6280706 | NA | MPPED2 | | 744 |
| ENSG00000170946 | 458.2746792 | 0.02183294 | 0.0974364 | 0.7506606 | 0.8924648 | DNAJC24 | | 120526 |
| ENSG00000148950 | 204.3956945 | -0.011674523 | 0.1128092 | 0.8542038 | 0.9410501 | IMMP1L | | 196294 |
| ENSG00000109911 | 378.4363398 | 0.029443219 | 0.1004097 | 0.6707244 | 0.849168 | ELP4 | | 26610 |
| ENSG00000007372 | 759.9471014 | -0.214838186 | 0.10887 | 0.010589 | 0.0658978 | PAX6 | | 5080 |
| ENSG00000049449 | 4562.026088 | -0.022338561 | 0.0601526 | 0.6785132 | 0.853512 | RCN1 | | 5954 |
| ENSG00000255252 | 39.88304847 | 0.018575385 | 0.1315464 | 0.6497508 | NA | NA | NA | |
| ENSG00000184937 | 290.9691985 | -0.047008509 | 0.1114095 | 0.4968415 | 0.7319873 | WT1 | | 7490 |
| ENSG00000183242 | 34.10583926 | 0.00680082 | 0.1314714 | 0.8547492 | NA | WT1-AS | | 51352 |
| ENSG00000149100 | 3886.520617 | -0.027622945 | 0.0575721 | 0.5960413 | 0.8039675 | EIF3M | | 10480 |
| ENSG00000186714 | 17.12895907 | 0.009479599 | 0.1339632 | 0.7427881 | NA | CCDC73 | | 493860 |
| ENSG00000135378 | 35.03711024 | 0.058314814 | 0.1479816 | 0.1529725 | NA | PRRG4 | | 79056 |
| ENSG00000060749 | 3335.696655 | -0.188809725 | 0.0658517 | 0.0013481 | 0.0141743 | QSER1 | | 79832 |
| ENSG00000121690 | 213.7237284 | -0.0074373 | 0.1105422 | 0.9075249 | 0.963709 | DEPDC7 | | 91614 |
| ENSG00000176148 | 468.8784253 | 0.127786507 | 0.1167282 | 0.1098081 | 0.3209078 | TCP11L1 | | 55346 |
| ENSG00000176102 | 1158.202267 | 0.026360812 | 0.0766293 | 0.6613871 | 0.8432289 | CSTF3 | | 1479 |
| ENSG00000247151 | 19.87039607 | 0.069184693 | 0.159277 | 0.0377834 | NA | CSTF3-DT | | 338739 |
| ENSG00000110422 | 1793.419023 | -0.296313073 | 0.0797496 | 3.14E-05 | 0.0006673 | HIPK3 | | 10114 |
| ENSG00000110427 | 216.9566139 | 0.081492348 | 0.127711 | 0.2616799 | 0.5292042 | KIAA1549L | | 25758 |
| ENSG00000205177 | 13.61218516 | -0.005281957 | 0.1342813 | 0.8349726 | NA | C11orf91 | | 100131378 |
| ENSG00000085063 | 10336.81187 | -0.012295241 | 0.0519261 | 0.8151287 | 0.9244431 | CD59 | | 966 |
| ENSG00000110429 | 842.9979073 | -0.199262507 | 0.1032085 | 0.0134641 | 0.0783239 | FBXO3 | | 26273 |
| ENSG00000135387 | 8183.352493 | -0.170019601 | 0.0583594 | 0.0013894 | 0.0144905 | CAPRIN1 | | 4076 |
| ENSG00000135372 | 2392.299276 | -0.08690505 | 0.0669274 | 0.1364981 | 0.3638055 | NAT10 | | 55226 |
| ENSG00000166016 | 1323.274272 | 0.077716863 | 0.0788876 | 0.2331821 | 0.4955067 | ABTB2 | | 25841 |
| ENSG00000121691 | 825.5430332 | -0.204806905 | 0.1049979 | 0.0122371 | 0.0733408 | CAT | | 847 |
| ENSG00000149089 | 426.3244553 | -0.031366684 | 0.1033116 | 0.6486056 | 0.836587 | APIP | | 51074 |
| ENSG00000110435 | 1002.218816 | -0.10073825 | 0.0861018 | 0.1431677 | 0.3745698 | PDHX | | 8050 |
| ENSG00000255521 | 15.94603835 | 0.012232937 | 0.134554 | 0.6580697 | NA | CD44-DT | | 100507144 |
| ENSG00000026508 | 16277.97451 | 0.10028412 | 0.0554493 | 0.0473909 | 0.1859327 | CD44 | | 960 |
| ENSG00000255443 | 21.32850147 | 0.043998698 | 0.1429424 | 0.1768777 | NA | NA | NA | |
| ENSG00000251194 | 24.59566008 | 0.022952917 | 0.1349901 | 0.494461 | NA | NA | NA | |
| ENSG00000179431 | 1980.869745 | 0.042198007 | 0.0711227 | 0.494729 | 0.7302112 | FJX1 | | 24147 |
| ENSG00000166326 | 2760.510866 | -0.031733362 | 0.062181 | 0.565826 | 0.782102 | TRIM44 | | 54765 |
| ENSG00000261355 | 54.65760485 | 0.017336347 | 0.1288329 | 0.7093108 | 0.8682712 | NA | NA | |
| ENSG00000179241 | 779.9109968 | -0.036625719 | 0.0850082 | 0.5865826 | 0.7971319 | LDLRAD3 | | 143458 |
| ENSG00000196559 | 13.71057285 | -0.015000176 | 0.1353988 | 0.5522186 | NA | NA | NA | |
| ENSG00000110442 | 810.9189433 | -0.12490524 | 0.0982256 | 0.093644 | 0.29028 | COMMD9 | | 29099 |
| ENSG00000135362 | 67.70095483 | 0.030725973 | 0.1300774 | 0.5382919 | 0.7619385 | PRR5L | | 79899 |
| ENSG00000175104 | 360.1021367 | -0.03183178 | 0.1039941 | 0.6433625 | 0.833254 | TRAF6 | | 7189 |
| ENSG00000166352 | 151.4922029 | 0.021239533 | 0.1168675 | 0.7347502 | 0.8829853 | IFTAP | | 119710 |
| ENSG00000166181 | 5552.438374 | -0.192977086 | 0.0588319 | 0.0003347 | 0.0047329 | API5 | | 8539 |
| ENSG00000254907 | 99.24320395 | -0.057025972 | 0.1349356 | 0.3297876 | 0.5978967 | NA | NA | |
| ENSG00000052841 | 1847.491587 | 0.093243665 | 0.0733346 | 0.1349021 | 0.3616363 | TTC17 | | 55761 |
| ENSG00000254463 | 9.83279413 | -0.004072243 | 0.1349358 | 0.8499656 | NA | PPIAP41 | | 111082977 |
| ENSG00000149084 | 2255.952683 | -0.164755476 | 0.0761236 | 0.0114166 | 0.069784 | HSD17B12 | | 51144 |
| ENSG00000246250 | 55.2734262 | -0.075921385 | 0.1549677 | 0.1381475 | 0.3667502 | NA | NA | |
| ENSG00000166199 | 132.0459084 | 0.045016729 | 0.1255656 | 0.4660683 | 0.7093442 | ALKBH3 | | 221120 |
| ENSG00000110455 | 64.63010147 | 0.142590993 | 0.2281143 | 0.0383903 | 0.1633055 | ACCS | | 84680 |
| ENSG00000151348 | 1902.01629 | -0.042361135 | 0.069073 | 0.476801 | 0.7161907 | EXT2 | | 2132 |
| ENSG00000085117 | 980.3982878 | -0.144098245 | 0.0916666 | 0.0475395 | 0.1862827 | CD82 | | 3732 |
| ENSG00000157570 | 128.7577871 | 0.006233014 | 0.1200313 | 0.921258 | 0.9697409 | TSPAN18 | | 90139 |
| ENSG00000019485 | 634.7207602 | -0.20147286 | 0.1214127 | 0.0216524 | 0.1093783 | PRDM11 | | 56981 |
| ENSG00000181830 | 493.2056244 | 0.09877998 | 0.1066697 | 0.1901351 | 0.4411059 | SLC35C1 | | 55343 |
| ENSG00000121671 | 463.8151723 | 0.055117293 | 0.1008147 | 0.438374 | 0.6881889 | CRY2 | | 1408 |
| ENSG00000121653 | 839.5219091 | -0.195226807 | 0.1014048 | 0.0141757 | 0.0812495 | MAPK8IP1 | | 9479 |
| ENSG00000255498 | 13.23591227 | 0.017644949 | 0.1360103 | 0.4766618 | NA | NA | NA | |
| ENSG00000234776 | 15.02216573 | -0.011256721 | 0.1345441 | 0.6738659 | NA | FREY1 | | 143678 |
| ENSG00000121680 | 731.8489037 | 0.12158012 | 0.0998704 | 0.1055858 | 0.3128736 | PEX16 | | 9409 |
| ENSG00000165905 | 90.48114284 | 0.025007852 | 0.1261702 | 0.6369794 | 0.828293 | LARGE2 | | 120071 |
| ENSG00000135365 | 500.9410225 | -0.247551833 | 0.1308179 | 0.0092066 | 0.0603156 | PHF21A | | 51317 |
| ENSG00000149091 | 2576.975583 | 0.090900243 | 0.0692192 | 0.1290869 | 0.3535437 | DGKZ | | 8525 |

| | | | | | | | |
|---|---|---|---|---|---|---|---|
| ENSG00000110492 | 677.187831 | 0.137258184 | 0.1160591 | 0.0886808 | 0.2803467 | MDK | 4192 |
| ENSG00000180720 | 14.33941448 | 0.010707516 | 0.1346776 | 0.6826728 | NA | CHRM4 | 1132 |
| ENSG00000110497 | 879.0537019 | 0.18580752 | 0.103632 | 0.020364 | 0.1051136 | AMBRA1 | 55626 |
| ENSG00000244313 | 119.6659335 | -0.058437916 | 0.131672 | 0.3473603 | 0.6135409 | NA | NA |
| ENSG00000180423 | 85.43561773 | 0.092375346 | 0.1591294 | 0.1403498 | 0.3697587 | HARBI1 | 283254 |
| ENSG00000175224 | 1577.608213 | 0.079131469 | 0.0753845 | 0.2115491 | 0.4681851 | ATG13 | 9776 |
| ENSG00000175220 | 1336.806679 | -0.004351127 | 0.0740174 | 0.9434554 | 0.9782959 | ARHGAP1 | 392 |
| ENSG00000175213 | 610.220325 | 0.584957325 | 0.1271406 | 2.87E-07 | 1.16E-05 | ZNF408 | 79797 |
| ENSG00000175216 | 8934.477898 | -0.135201771 | 0.0544272 | 0.0067538 | 0.0475823 | CKAP5 | 9793 |
| ENSG00000247675 | 14.42238478 | -0.015163035 | 0.1351959 | 0.5651005 | NA | LRP4-AS1 | 100507401 |
| ENSG00000134569 | 675.8027186 | 0.052182384 | 0.1010964 | 0.4548909 | 0.6999843 | LRP4 | 4038 |
| ENSG00000149179 | 368.1472401 | -0.067476105 | 0.1077912 | 0.3504698 | 0.6163779 | CSTPP1 | 79096 |
| ENSG00000149182 | 1881.107048 | 0.05343341 | 0.0753995 | 0.3989039 | 0.6577635 | ARFGAP2 | 84364 |
| ENSG00000270060 | 22.09583181 | -0.022501696 | 0.1355292 | 0.468136 | NA | NA | NA |
| ENSG00000165912 | 687.3214988 | -0.002130366 | 0.0855392 | 0.973637 | 0.9899943 | PACSIN3 | 29763 |
| ENSG00000134574 | 697.5447061 | 0.120020847 | 0.1013909 | 0.1115389 | 0.3239397 | DDB2 | 1643 |
| ENSG00000134575 | 427.2165495 | -0.187221686 | 0.1428083 | 0.0407913 | 0.1698088 | ACP2 | 53 |
| ENSG00000025434 | 181.5021861 | 0.099930527 | 0.1435839 | 0.1777536 | 0.4270449 | NR1H3 | 10062 |
| ENSG00000110514 | 1657.372561 | -0.046717618 | 0.0752443 | 0.4484264 | 0.6958415 | MADD | 8567 |
| ENSG00000165915 | 769.8309857 | -0.038585532 | 0.0848167 | 0.5629471 | 0.7805304 | SLC39A13 | 91252 |
| ENSG00000165916 | 5602.837366 | -0.053378642 | 0.0551515 | 0.2891995 | 0.5592864 | PSMC3 | 5702 |
| ENSG00000165917 | 13.27345825 | 0.008635425 | 0.1346033 | 0.7356207 | NA | RAPSN | 5913 |
| ENSG00000149187 | 4818.009026 | -0.130538154 | 0.0633438 | 0.0205257 | 0.1055283 | CELF1 | 10658 |
| ENSG00000213619 | 1459.835304 | -0.001424876 | 0.0728118 | 0.9781133 | 0.9917831 | NDUFS3 | 4722 |
| ENSG00000110536 | 303.9827462 | 0.025886066 | 0.1043466 | 0.7062424 | 0.8671822 | PTPMT1 | 114971 |
| ENSG00000123444 | 757.7283249 | -0.092500106 | 0.09222 | 0.1905499 | 0.4412794 | KBTBD4 | 55709 |
| ENSG00000231880 | 10.02094351 | 0.015080532 | 0.1360259 | 0.4994133 | NA | NA | NA |
| ENSG00000109919 | 2302.531045 | -0.028190539 | 0.0671838 | 0.6293151 | 0.8235526 | MTCH2 | 23788 |
| ENSG00000165923 | 14.76372584 | 0.015360104 | 0.1351842 | 0.5669285 | NA | AGBL2 | 79841 |
| ENSG00000109920 | 2531.733892 | 0.057671348 | 0.0644212 | 0.3130347 | 0.5821263 | FNBP4 | 23360 |
| ENSG00000200090 | 11.59183617 | -0.017656471 | 0.1365094 | 0.4213115 | NA | NA | NA |
| ENSG00000030066 | 2437.321912 | -0.210458083 | 0.0709386 | 0.0008145 | 0.0094384 | NUP160 | 23279 |
| ENSG00000149177 | 632.2913897 | 0.214679387 | 0.1155149 | 0.013451 | 0.0783146 | PTPRJ | 5795 |
| ENSG00000149115 | 2662.419001 | -0.098394174 | 0.0649328 | 0.0852333 | 0.2732189 | TNKS1BP1 | 85456 |
| ENSG00000254662 | 62.82507883 | -0.072353679 | 0.1514618 | 0.1630603 | 0.4055156 | NA | NA |
| ENSG00000149136 | 10482.724 | -0.268686019 | 0.0541949 | 1.41E-07 | 6.37E-06 | SSRP1 | 6749 |
| ENSG00000186907 | 19.50919145 | -0.022226913 | 0.1357386 | 0.4589116 | NA | RTN4RL2 | 349667 |
| ENSG00000149150 | 67.23704704 | 0.140539002 | 0.2218048 | 0.0429625 | 0.1753478 | SLC43A1 | 8501 |
| ENSG00000134809 | 1123.068078 | 0.000430592 | 0.0755756 | 0.9953187 | 0.9980528 | TIMM10 | 26519 |
| ENSG00000156587 | 256.933625 | 0.039950587 | 0.1114686 | 0.5582072 | 0.7773508 | UBE2L6 | 9246 |
| ENSG00000254602 | 20.61764442 | 0.00905289 | 0.1333401 | 0.7718085 | NA | NA | NA |
| ENSG00000172409 | 740.8254251 | -0.091827634 | 0.0927832 | 0.1965457 | 0.4493738 | CLP1 | 10978 |
| ENSG00000156599 | 2971.488134 | 0.033943174 | 0.0608463 | 0.531842 | 0.757246 | ZDHHC5 | 25921 |
| ENSG00000156603 | 512.2475316 | 0.010245344 | 0.09337 | 0.8818346 | 0.9524387 | MED19 | 219541 |
| ENSG00000213593 | 2063.651772 | -0.021672101 | 0.0674441 | 0.7202237 | 0.8733776 | TMX2 | 51075 |
| ENSG00000211450 | 489.1284713 | -0.013129203 | 0.0957592 | 0.846752 | 0.9372292 | SELENOH | 280636 |
| ENSG00000198561 | 3950.231223 | -0.150206311 | 0.0658881 | 0.0099997 | 0.0635506 | CTNND1 | 1500 |
| ENSG00000110031 | 56.11419027 | 0.211361328 | 0.4532804 | 0.012778 | 0.0756129 | LPXN | 9404 |
| ENSG00000186660 | 2815.11383 | -0.154712933 | 0.0708704 | 0.0121157 | 0.0728004 | ZFP91 | 80829 |
| ENSG00000242689 | 59.138551 | -0.041487796 | 0.1349899 | 0.3890463 | 0.6493015 | CNTF | 1270 |
| ENSG00000189057 | 2353.796514 | 0.303802368 | 0.07849 | 1.66E-05 | 0.0003892 | FAM111B | 374393 |
| ENSG00000245571 | 114.5803963 | -0.045133958 | 0.1270134 | 0.4531202 | 0.6987417 | FAM111A-D | 101927204 |
| ENSG00000166801 | 2712.933477 | 0.019164288 | 0.064952 | 0.7384138 | 0.885525 | FAM111A | 63901 |
| ENSG00000110042 | 37.69960008 | 0.04853471 | 0.1422837 | 0.2306397 | NA | DTX4 | 23220 |
| ENSG00000110048 | 2136.328382 | -0.078998366 | 0.0699244 | 0.1892588 | 0.4402444 | OSBP | 5007 |
| ENSG00000255139 | 12.08290349 | 0.010667702 | 0.1350082 | 0.6636983 | NA | NA | NA |
| ENSG00000166889 | 2904.342061 | 0.028952204 | 0.0621715 | 0.6015452 | 0.8077538 | PATL1 | 219988 |
| ENSG00000166900 | 1219.187094 | 0.290915672 | 0.0941896 | 0.000306 | 0.0043962 | STX3 | 6809 |
| ENSG00000234964 | 149.7136983 | -0.005514984 | 0.1162333 | 0.9268601 | 0.9718632 | NA | NA |
| ENSG00000166902 | 956.0750306 | -0.160045962 | 0.0961311 | 0.0337772 | 0.1490562 | MRPL16 | 54948 |
| ENSG00000255959 | 11.87577632 | -0.014444943 | 0.135578 | 0.5468679 | NA | NA | NA |
| ENSG00000256813 | 16.88232183 | 0.024228598 | 0.1369864 | 0.3741785 | NA | CCDC86-AS | 127138859 |
| ENSG00000110104 | 3842.577844 | 0.253641115 | 0.0704393 | 6.51E-05 | 0.0012298 | CCDC86 | 79080 |
| ENSG00000110107 | 3427.319818 | -0.040361495 | 0.061033 | 0.4589984 | 0.7032477 | PRPF19 | 27339 |
| ENSG00000110108 | 4047.546434 | 0.038860628 | 0.057439 | 0.4560946 | 0.7007999 | TMEM109 | 79073 |
| ENSG00000256196 | 8.720189786 | 0.015542337 | 0.1363496 | 0.4552754 | NA | NA | NA |
| ENSG00000006118 | 3419.187662 | 0.224191343 | 0.0683418 | 0.0002653 | 0.0038936 | TMEM132A | 54972 |
| ENSG00000110446 | 13.89602171 | 0.001051216 | 0.1342719 | 0.9688054 | NA | SLC15A3 | 51296 |
| ENSG00000167987 | 447.6622042 | 0.144671657 | 0.1228763 | 0.080616 | 0.2634794 | VPS37C | 55048 |
| ENSG00000167986 | 7225.767901 | -0.078564924 | 0.0540471 | 0.1079911 | 0.3180646 | DDB1 | 1642 |

| | | | | | | | |
|---|---|---|---|---|---|---|---|
| ENSG00000149476 | 630.4455752 | 0.036389832 | 0.0907232 | 0.5983418 | 0.8058608 | TKFC | 26007 |
| ENSG00000162144 | 1010.912476 | 0.065087717 | 0.0848276 | 0.3392084 | 0.6066573 | CYB561A3 | 220002 |
| ENSG00000149483 | 626.2417003 | 0.060979225 | 0.0944966 | 0.3867748 | 0.647427 | TMEM138 | 51524 |
| ENSG00000187049 | 124.8140778 | -0.086487887 | 0.1451577 | 0.199704 | 0.4533117 | TMEM216 | 51259 |
| ENSG00000149532 | 2561.79136 | -0.086495826 | 0.0684375 | 0.1442425 | 0.3765973 | CPSF7 | 79869 |
| ENSG00000167985 | 181.5255237 | 0.24290138 | 0.2299487 | 0.0266303 | 0.1268116 | SDHAF2 | 54949 |
| ENSG00000011347 | 142.0741811 | 0.194210394 | 0.2433297 | 0.0402844 | 0.1683264 | SYT7 | 9066 |
| ENSG00000134780 | 196.0467194 | -0.021909167 | 0.1134608 | 0.7337322 | 0.8823974 | DAGLA | 747 |
| ENSG00000124920 | 414.9629937 | 0.022988267 | 0.0980908 | 0.7383254 | 0.885525 | MYRF | 745 |
| ENSG00000134825 | 741.6678477 | -0.125941915 | 0.1047001 | 0.1003147 | 0.3025862 | TMEM258 | 746 |
| ENSG00000168496 | 3206.540088 | -0.015680546 | 0.0596743 | 0.7696447 | 0.9015463 | FEN1 | 2237 |
| ENSG00000134824 | 252.4744054 | -0.252819669 | 0.1886915 | 0.0200194 | 0.1039319 | FADS2 | 9415 |
| ENSG00000149485 | 2307.203577 | -0.204513992 | 0.0740512 | 0.0016031 | 0.0161017 | FADS1 | 3992 |
| ENSG00000221968 | 625.849713 | -0.252400755 | 0.119582 | 0.0056919 | 0.0417369 | FADS3 | 3995 |
| ENSG00000167994 | 39.29245722 | 0.119516611 | 0.2093082 | 0.0250536 | NA | RAB3IL1 | 5866 |
| ENSG00000167995 | 320.7383612 | -0.009372634 | 0.125439 | 0.8505193 | 0.9387618 | BEST1 | 7439 |
| ENSG00000167996 | 13063.57717 | 0.022434037 | 0.0576072 | 0.6692402 | 0.848177 | FTH1 | 2495 |
| ENSG00000149503 | 2672.875305 | -0.103890529 | 0.0659202 | 0.0724442 | 0.2457085 | INCENP | 3619 |
| ENSG00000162174 | 784.3105284 | -0.024721973 | 0.0837988 | 0.7087037 | 0.8682085 | ASRGL1 | 80150 |
| ENSG00000124942 | 76460.51302 | -0.143548665 | 0.0520898 | 0.0028371 | 0.0247128 | AHNAK | 79026 |
| ENSG00000257058 | 10.2975013 | 0.007053476 | 0.13503 | 0.7545799 | NA | NA | NA |
| ENSG00000149016 | 106.059185 | 0.178819637 | 0.2468037 | 0.0415113 | 0.1714886 | TUT1 | 64852 |
| ENSG00000149480 | 3860.869751 | -0.117448946 | 0.061799 | 0.0331581 | 0.1469345 | MTA2 | 9219 |
| ENSG00000149499 | 1056.94459 | -0.067956883 | 0.0824358 | 0.3078395 | 0.5769089 | EML3 | 256364 |
| ENSG00000149489 | 41.46839502 | 0.012852027 | 0.1306517 | 0.7530915 | 0.8932431 | ROM1 | 6094 |
| ENSG00000149541 | 715.0130386 | 0.173790116 | 0.1092665 | 0.0328524 | 0.1463571 | B3GAT3 | 26229 |
| ENSG00000089597 | 9461.28354 | -0.168969364 | 0.0549262 | 0.0008371 | 0.009624 | GANAB | 23193 |
| ENSG00000185085 | 491.80539 | 0.078890944 | 0.1026494 | 0.2801265 | 0.5488355 | INTS5 | 80789 |
| ENSG00000162194 | 473.6935512 | 0.021813606 | 0.0963959 | 0.7505233 | 0.8924648 | LBHD1 | 79081 |
| ENSG00000214756 | 80.0576699 | 0.017077968 | 0.1260148 | 0.7403497 | 0.8863406 | CSKMT | 751071 |
| ENSG00000204922 | 297.3426542 | -0.0588704 | 0.1127197 | 0.4041333 | 0.6612662 | UQCC3 | 790955 |
| ENSG00000162191 | 1832.107901 | 0.22799435 | 0.0779659 | 0.0008101 | 0.0094043 | UBXN1 | 51035 |
| ENSG00000168000 | 44.64281128 | 0.002393222 | 0.1299607 | 0.9545982 | 0.9819702 | BSCL2 | 26580 |
| ENSG00000214753 | 1146.086649 | -0.067056632 | 0.081214 | 0.3091294 | 0.5784909 | HNRNPUL2 | 221092 |
| ENSG00000162222 | 355.6905775 | 0.548539683 | 0.1615018 | 4.41E-05 | 0.0008842 | TTC9C | 283237 |
| ENSG00000185670 | 47.26327928 | -0.029139968 | 0.13279 | 0.5045667 | 0.7381431 | ZBTB3 | 79842 |
| ENSG00000168002 | 974.0441886 | -0.025582421 | 0.0807362 | 0.694206 | 0.8602822 | POLR2G | 5436 |
| ENSG00000162227 | 478.3441736 | -0.045429285 | 0.0971469 | 0.5150106 | 0.7449955 | TAF6L | 10629 |
| ENSG00000168569 | 198.0885727 | -0.04022493 | 0.115569 | 0.5434713 | 0.7661361 | TMEM223 | 79064 |
| ENSG00000269176 | 21.17762915 | -0.013807063 | 0.1337861 | 0.6583051 | NA | NA | NA |
| ENSG00000185475 | 219.8652883 | -0.017716828 | 0.1105987 | 0.7845426 | 0.9098848 | TMEM179B | 374395 |
| ENSG00000162231 | 1731.870345 | 0.056368726 | 0.0709359 | 0.3537912 | 0.6187516 | NXF1 | 10482 |
| ENSG00000162236 | 816.9398751 | 0.15802226 | 0.10195 | 0.0421605 | 0.1733465 | STX5 | 6811 |
| ENSG00000133316 | 1149.425602 | 0.170752984 | 0.0919429 | 0.0211342 | 0.1078029 | WDR74 | 54663 |
| ENSG00000255717 | 3874.658523 | 0.638723055 | 0.1491009 | 1.19E-06 | 3.92E-05 | SNHG1 | 23642 |
| ENSG00000168003 | 6687.785006 | 1.012902221 | 0.0723099 | 8.26E-46 | 1.21E-42 | SLC3A2 | 6520 |
| ENSG00000176485 | 369.0667382 | -0.034184984 | 0.1022916 | 0.6214755 | 0.818389 | PLAAT3 | 11145 |
| ENSG00000184743 | 2452.941498 | -0.007783707 | 0.0657628 | 0.9082865 | 0.9638438 | ATL3 | 25923 |
| ENSG00000133318 | 3743.507857 | -0.04723733 | 0.0595839 | 0.3772766 | 0.6391542 | RTN3 | 10313 |
| ENSG00000188070 | 379.6946769 | -0.10196092 | 0.1253702 | 0.186507 | 0.4373797 | ZFTA | 65998 |
| ENSG00000168005 | 906.7042728 | -0.018227882 | 0.080476 | 0.7787117 | 0.9075471 | SPINDOC | 144097 |
| ENSG00000072518 | 1221.097115 | -0.026852497 | 0.0787 | 0.6775214 | 0.8529634 | MARK2 | 2011 |
| ENSG00000167771 | 16.61179313 | -0.006767386 | 0.1339593 | 0.8042744 | NA | RCOR2 | 283248 |
| ENSG00000110583 | 1633.094167 | 0.041945616 | 0.0711471 | 0.4908052 | 0.7273003 | NAA40 | 79829 |
| ENSG00000176340 | 2028.975258 | 0.043083031 | 0.074972 | 0.4990144 | 0.7341862 | COX8A | 1351 |
| ENSG00000167770 | 2031.155986 | -0.031639362 | 0.0666036 | 0.585688 | 0.7968113 | OTUB1 | 55611 |
| ENSG00000133315 | 237.8303111 | 0.005880581 | 0.1077135 | 0.9304017 | 0.972984 | MACROD1 | 28992 |
| ENSG00000168439 | 8776.837497 | -0.129253953 | 0.0568552 | 0.0115301 | 0.0703699 | STIP1 | 10963 |
| ENSG00000149781 | 28.94905426 | -0.056309358 | 0.1477839 | 0.1374271 | NA | FERMT3 | 83706 |
| ENSG00000149743 | 202.257885 | -0.005444655 | 0.1119648 | 0.9288954 | 0.9726302 | TRPT1 | 83707 |
| ENSG00000149761 | 411.1661903 | -0.028121138 | 0.0991438 | 0.6828817 | 0.8544725 | NUDT22 | 84304 |
| ENSG00000256116 | 10.98937193 | 0.014476315 | 0.1357623 | 0.533665 | NA | NA | NA |
| ENSG00000110011 | 175.3331447 | 0.00127202 | 0.1131998 | 0.9852939 | 0.9940394 | DNAJC4 | 3338 |
| ENSG00000173511 | 421.4908576 | -0.042980952 | 0.1013127 | 0.5381404 | 0.7619385 | VEGFB | 7423 |
| ENSG00000173486 | 1275.945353 | -0.082086446 | 0.0783925 | 0.205437 | 0.4607272 | FKBP2 | 2286 |
| ENSG00000173457 | 3511.551541 | -0.030258449 | 0.058568 | 0.5665581 | 0.782728 | PPP1R14B | 26472 |
| ENSG00000256940 | 82.17035407 | 0.454185047 | 0.4081433 | 0.0083144 | 0.0557204 | PPP1R14B-/ | 105369340 |
| ENSG00000257086 | 50.96781279 | -0.064907335 | 0.1483383 | 0.1774418 | 0.4268322 | NA | NA |
| ENSG00000149782 | 2114.214198 | -0.314101496 | 0.0828612 | 2.13E-05 | 0.0004789 | PLCB3 | 5331 |
| ENSG00000002330 | 881.9376647 | 0.019131941 | 0.0809684 | 0.7702771 | 0.9020157 | BAD | 572 |

| | | | | | | | |
|---|---|---|---|---|---|---|---|
| ENSG00000173264 | 701.0096293 | 0.10855032 | 0.1055954 | 0.1531145 | 0.3908224 | GPR137 | 56834 |
| ENSG00000173153 | 1264.438807 | 0.446447363 | 0.0915461 | 9.85E-08 | 4.76E-06 | ESRRA | 2101 |
| ENSG00000173113 | 1358.39791 | -0.434520581 | 0.0933925 | 3.01E-07 | 1.21E-05 | TRMT112 | 51504 |
| ENSG00000126432 | 3497.574394 | -0.040446585 | 0.0656431 | 0.4822629 | 0.7213706 | PRDX5 | 25824 |
| ENSG00000168071 | 102.6324556 | -0.053691106 | 0.1333726 | 0.3558921 | 0.6192724 | CCDC88B | 283234 |
| ENSG00000162302 | 2191.645838 | -0.092813009 | 0.0698138 | 0.1233219 | 0.3442934 | RPS6KA4 | 8986 |
| ENSG00000068831 | 20.50926884 | -0.016762063 | 0.1346549 | 0.5733386 | NA | RASGRP2 | 10235 |
| ENSG00000068976 | 10.27348638 | 0.003216742 | 0.1348006 | 0.8884136 | NA | PYGM | 5837 |
| ENSG00000168066 | 5110.420672 | 0.030466807 | 0.0560213 | 0.5506529 | 0.7717069 | SF1 | 7536 |
| ENSG00000269290 | 14.52927502 | -0.014349482 | 0.1353878 | 0.5623422 | NA | NA | NA |
| ENSG00000168067 | 789.9823357 | -0.350948177 | 0.1081421 | 0.0001302 | 0.002166 | MAP4K2 | 5871 |
| ENSG00000133895 | 1071.343264 | 0.01696259 | 0.0778872 | 0.7918977 | 0.9129646 | MEN1 | 4221 |
| ENSG00000171219 | 256.360804 | -0.264180332 | 0.2002775 | 0.0185428 | 0.0985432 | CDC42BPG | 55561 |
| ENSG00000110047 | 7978.675203 | -0.077442641 | 0.0570017 | 0.133803 | 0.3604596 | EHD1 | 10938 |
| ENSG00000110046 | 853.2619666 | 0.018030273 | 0.0813466 | 0.7836914 | 0.9098797 | ATG2A | 23130 |
| ENSG00000068971 | 436.0324976 | 0.108868511 | 0.1138132 | 0.1599934 | 0.4009618 | PPP2R5B | 5526 |
| ENSG00000168062 | 10.02120381 | 0.019722419 | 0.1372433 | 0.3318036 | NA | BATF2 | 116071 |
| ENSG00000213465 | 241.072933 | -0.032708983 | 0.1104187 | 0.6239507 | 0.8201157 | ARL2 | 402 |
| ENSG00000110025 | 14.56066368 | -0.01929761 | 0.135929 | 0.4667891 | NA | SNX15 | 29907 |
| ENSG00000168061 | 522.2371602 | 0.286701595 | 0.1341262 | 0.0041995 | 0.0331227 | SAC3D1 | 29901 |
| ENSG00000146670 | 3760.435567 | 0.267407102 | 0.0671928 | 1.31E-05 | 0.000317 | CDCA5 | 113130 |
| ENSG00000162300 | 493.8829215 | 0.140054493 | 0.1167909 | 0.083989 | 0.2707908 | ZFPL1 | 7542 |
| ENSG00000187066 | 10.531075 | 0.009466098 | 0.1352781 | 0.6722726 | NA | TMEM262 | 100130348 |
| ENSG00000149823 | 1280.229327 | -0.14437518 | 0.0843665 | 0.0369689 | 0.1587622 | VPS51 | 738 |
| ENSG00000149809 | 84.56648138 | -0.037395594 | 0.12907 | 0.4924552 | 0.7283747 | TM7SF2 | 7108 |
| ENSG00000174276 | 182.4066206 | 0.704812143 | 0.2155564 | 5.51E-05 | 0.0010743 | ZNHIT2 | 741 |
| ENSG00000149806 | 4993.668981 | 0.054059562 | 0.0635088 | 0.3370834 | 0.6048343 | FAU | 2197 |
| ENSG00000162298 | 712.1298992 | 0.029155095 | 0.0928526 | 0.6721483 | 0.8499086 | SYVN1 | 84447 |
| ENSG00000149792 | 1952.555426 | 0.026004571 | 0.0684945 | 0.6631882 | 0.8438599 | MRPL49 | 740 |
| ENSG00000254614 | 63.86678566 | 0.076598864 | 0.1526014 | 0.1635233 | 0.4062348 | CAPN1-AS1 | 728975 |
| ENSG00000014216 | 3454.966345 | -0.066546476 | 0.0633064 | 0.2355576 | 0.499283 | CAPN1 | 823 |
| ENSG00000197847 | 10.52379625 | -0.004361681 | 0.1348473 | 0.8441162 | NA | SLC22A20P | 440044 |
| ENSG00000014138 | 1285.456769 | 0.247182919 | 0.0914285 | 0.0013387 | 0.0141002 | POLA2 | 23649 |
| ENSG00000149798 | 347.895312 | -0.023027987 | 0.1045941 | 0.7341483 | 0.8827161 | CDC42EP2 | 10435 |
| ENSG00000133884 | 1585.643489 | 0.230118017 | 0.0832444 | 0.0012866 | 0.0136374 | DPF2 | 5977 |
| ENSG00000173825 | 33.4370521 | -0.058435817 | 0.1482288 | 0.1469059 | NA | TIGD3 | 220359 |
| ENSG00000162241 | 98.55227216 | -0.014437682 | 0.1235473 | 0.7880325 | 0.9116361 | SLC25A45 | 283130 |
| ENSG00000126391 | 3405.589727 | -0.044376677 | 0.0591332 | 0.4045091 | 0.6614808 | FRMD8 | 83786 |
| ENSG00000245532 | 8113.648821 | 0.02374972 | 0.0600605 | 0.6603508 | 0.8426737 | NEAT1 | 283131 |
| ENSG00000173727 | 55.09155619 | 0.062154932 | 0.1462768 | 0.2008084 | 0.454494 | NA | NA |
| ENSG00000251562 | 7148.293476 | -0.111300636 | 0.1149185 | 0.1527681 | 0.3902217 | MALAT1 | 378938 |
| ENSG00000142186 | 1818.823192 | 0.001287153 | 0.0670324 | 0.9848641 | 0.9940394 | SCYL1 | 57410 |
| ENSG00000168056 | 724.6428298 | -0.017014195 | 0.0858527 | 0.7984396 | 0.9162772 | LTBP3 | 4054 |
| ENSG00000173465 | 709.4501204 | 0.349381204 | 0.1184587 | 0.000343 | 0.0048209 | ZNRD2 | 10534 |
| ENSG00000176973 | 138.3503975 | -0.005434983 | 0.1196297 | 0.9246467 | 0.9709924 | FAM89B | 23625 |
| ENSG00000173442 | 3808.112543 | -0.012841518 | 0.0630751 | 0.8178181 | 0.9254567 | EHBP1L1 | 254102 |
| ENSG00000173327 | 2083.298944 | 0.116900512 | 0.0703302 | 0.0546005 | 0.2046873 | MAP3K11 | 4296 |
| ENSG00000197136 | 2158.332061 | 0.038635044 | 0.0656634 | 0.5037695 | 0.7379648 | PCNX3 | 399909 |
| ENSG00000213445 | 788.8342935 | -0.15258175 | 0.0994953 | 0.0456625 | 0.1825407 | SIPA1 | 6494 |
| ENSG00000173039 | 2125.420106 | 0.179260683 | 0.0740954 | 0.0052938 | 0.0396998 | RELA | 5970 |
| ENSG00000172977 | 500.2820395 | -0.096133028 | 0.10835 | 0.2017207 | 0.455764 | KAT5 | 10524 |
| ENSG00000172922 | 1003.547228 | 0.059182942 | 0.0808478 | 0.3643364 | 0.6269614 | RNASEH2C | 84153 |
| ENSG00000254470 | 291.0958398 | -0.082410269 | 0.122958 | 0.2624279 | 0.5300012 | AP5B1 | 91056 |
| ENSG00000172757 | 18759.20819 | -0.097229894 | 0.057641 | 0.0608389 | 0.2199504 | CFL1 | 1072 |
| ENSG00000172803 | 80.30723594 | -0.01212432 | 0.1255053 | 0.8104953 | 0.9218496 | SNX32 | 254122 |
| ENSG00000172732 | 861.0057597 | 0.050061112 | 0.0840517 | 0.4496973 | 0.6965805 | MUS81 | 80198 |
| ENSG00000172638 | 18.96376422 | 0.028628083 | 0.1376049 | 0.3382846 | NA | EFEMP2 | 30008 |
| ENSG00000172500 | 555.8561282 | -0.015369062 | 0.0906662 | 0.8201839 | 0.9260167 | FIBP | 9158 |
| ENSG00000175602 | 1176.979333 | 0.005936928 | 0.0860085 | 0.9214141 | 0.9698178 | CCDC85B | 11007 |
| ENSG00000175592 | 2719.803541 | 0.317646902 | 0.0708431 | 1.07E-06 | 3.59E-05 | FOSL1 | 8061 |
| ENSG00000175573 | 1056.972333 | 0.671255272 | 0.0970663 | 3.17E-13 | 4.35E-11 | C11orf68 | 83638 |
| ENSG00000175550 | 5152.271676 | 0.107385547 | 0.0588636 | 0.0431574 | 0.1758285 | DRAP1 | 10589 |
| ENSG00000175467 | 2288.825571 | -0.004626972 | 0.0636067 | 0.9449295 | 0.9784014 | SART1 | 9092 |
| ENSG00000175376 | 753.7618563 | 0.124291053 | 0.0995056 | 0.0981506 | 0.2987225 | EIF1AD | 84285 |
| ENSG00000175334 | 3346.804717 | -0.137873298 | 0.0632819 | 0.0143524 | 0.0818271 | BANF1 | 8815 |
| ENSG00000087365 | 5893.123123 | 0.031521425 | 0.0535832 | 0.5221415 | 0.7495719 | SF3B2 | 10992 |
| ENSG00000175115 | 1255.976595 | -0.028948705 | 0.0756422 | 0.6456533 | 0.8350173 | PACS1 | 55690 |
| ENSG00000174996 | 1289.105076 | 0.084287019 | 0.0856397 | 0.2172114 | 0.4764782 | KLC2 | 64837 |
| ENSG00000174903 | 5534.214645 | -0.129416961 | 0.0599777 | 0.0166878 | 0.0906816 | RAB1B | 81876 |
| ENSG00000245156 | 29.41820759 | -0.004541441 | 0.1315488 | 0.8975583 | NA | NA | NA |

| | | | | | | | |
|---|---|---|---|---|---|---|---|
| ENSG00000254452 | 16.31156707 | -0.0357941 | 0.1406516 | 0.1955239 | NA | NA | NA |
| ENSG00000174871 | 28.33542021 | 0.023824713 | 0.1344429 | 0.5078959 | NA | CNIH2 | 254263 |
| ENSG00000174851 | 1241.918008 | 0.246854924 | 0.0933802 | 0.001587 | 0.0159812 | YIF1A | 10897 |
| ENSG00000179292 | 136.0412019 | 0.045321427 | 0.124276 | 0.4708044 | 0.7116286 | TMEM151A | 256472 |
| ENSG00000174791 | 402.2009917 | 0.121937647 | 0.1208787 | 0.1272023 | 0.3504362 | RIN1 | 9610 |
| ENSG00000174744 | 1516.01514 | 0.081753084 | 0.0747547 | 0.1945561 | 0.4467485 | BRMS1 | 25855 |
| ENSG00000174684 | 330.9309623 | -0.082394153 | 0.1158694 | 0.2671998 | 0.535362 | B4GAT1 | 11041 |
| ENSG00000255468 | 22.75641739 | -0.013326583 | 0.133607 | 0.6736533 | NA | B4GAT1-DT | 102724064 |
| ENSG00000174669 | 186.394786 | -0.161955301 | 0.1794983 | 0.0675539 | 0.2352704 | SLC29A2 | 3177 |
| ENSG00000174547 | 1233.566302 | -0.082819847 | 0.0818661 | 0.2128449 | 0.4698453 | MRPL11 | 65003 |
| ENSG00000174516 | 160.9604837 | -0.042655321 | 0.121399 | 0.5000231 | 0.734688 | PELI3 | 246330 |
| ENSG00000255517 | 39.10303834 | 0.066716292 | 0.1524971 | 0.1268159 | NA | DPP3-DT | 101928069 |
| ENSG00000254986 | 1373.146055 | -0.039154453 | 0.0761736 | 0.5366642 | 0.7608659 | DPP3 | 10072 |
| ENSG00000174483 | 23.20030614 | -0.023129298 | 0.1353597 | 0.4725301 | NA | BBS1 | 582 |
| ENSG00000174165 | 189.337431 | 0.106677244 | 0.1466998 | 0.1580216 | 0.3978983 | ZDHHC24 | 254359 |
| ENSG00000269990 | 69.51271717 | 0.053917442 | 0.1379601 | 0.3108216 | 0.5809131 | NA | NA |
| ENSG00000174080 | 17.39467771 | -0.002373608 | 0.1335879 | 0.9232121 | NA | CTSF | 8722 |
| ENSG00000173992 | 507.0261546 | 0.00646503 | 0.0921772 | 0.9256019 | 0.9714246 | CCS | 9973 |
| ENSG00000239306 | 1337.894697 | -0.119817557 | 0.0862788 | 0.0842267 | 0.2711082 | RBM14 | 10432 |
| ENSG00000173933 | 249.1893213 | -0.104040878 | 0.1321571 | 0.1773173 | 0.4267086 | RBM4 | 5936 |
| ENSG00000173914 | 453.9576181 | -0.117230196 | 0.1261989 | 0.1415326 | 0.3718494 | RBM4B | 83759 |
| ENSG00000258297 | 66.00746838 | -0.0111357 | 0.1265711 | 0.8176869 | 0.9254567 | NA | NA |
| ENSG00000173898 | 801.447933 | 0.207195681 | 0.1113694 | 0.0143804 | 0.0819001 | SPTBN2 | 6712 |
| ENSG00000173715 | 206.4356861 | -0.025182104 | 0.1120622 | 0.7008738 | 0.8645097 | TOP6BL | 79703 |
| ENSG00000173653 | 532.3397037 | -0.024796536 | 0.0921104 | 0.7162276 | 0.8714352 | RCE1 | 9986 |
| ENSG00000173599 | 475.6782888 | -0.434939478 | 0.1517955 | 0.0003104 | 0.0044482 | PC | 5091 |
| ENSG00000173621 | 910.5488421 | -0.068368354 | 0.0853988 | 0.3124327 | 0.5818701 | LRFN4 | 78999 |
| ENSG00000173227 | 53.35745689 | -0.518905634 | 0.7205135 | 0.0079924 | 0.0541176 | SYT12 | 91683 |
| ENSG00000173156 | 213.2743423 | 0.238673118 | 0.2028033 | 0.0263418 | 0.1257453 | RHOD | 29984 |
| ENSG00000173120 | 2477.089796 | -0.029404939 | 0.0627424 | 0.5968825 | 0.8046376 | KDM2A | 22992 |
| ENSG00000173020 | 2069.271294 | 0.018448697 | 0.0653197 | 0.7492213 | 0.8912072 | GRK2 | 156 |
| ENSG00000172932 | 545.7074525 | -0.136187575 | 0.1184035 | 0.0922654 | 0.2872944 | ANKRD13D | 338692 |
| ENSG00000172830 | 540.6349926 | -0.099392932 | 0.1039522 | 0.1838156 | 0.4338885 | SSH3 | 54961 |
| ENSG00000175482 | 73.44329395 | 0.021370615 | 0.1268734 | 0.6775659 | 0.8529634 | POLD4 | 57804 |
| ENSG00000175505 | 610.1560479 | 0.019779384 | 0.0957761 | 0.7741397 | 0.9046394 | CLCF1 | 23529 |
| ENSG00000172613 | 450.8522019 | 0.146804259 | 0.124903 | 0.0784066 | 0.2585037 | RAD9A | 5883 |
| ENSG00000172531 | 4567.516536 | -0.132598764 | 0.0615248 | 0.0160845 | 0.0885083 | PPP1CA | 5499 |
| ENSG00000172508 | 8.389183278 | -0.010068951 | 0.1358687 | 0.592849 | NA | CARNS1 | 57571 |
| ENSG00000175634 | 980.3672579 | 0.008142459 | 0.0785535 | 0.9000118 | 0.9607968 | RPS6KB2 | 6199 |
| ENSG00000213402 | 16.36494868 | 0.009974947 | 0.1346378 | 0.7005488 | NA | PTPRCAP | 5790 |
| ENSG00000172725 | 1944.841458 | -0.157625112 | 0.0771031 | 0.016199 | 0.0888425 | CORO1B | 57175 |
| ENSG00000172663 | 173.1505376 | 0.228389867 | 0.2210893 | 0.0309492 | 0.1411627 | TMEM134 | 80194 |
| ENSG00000110711 | 1141.494917 | -0.05787667 | 0.0786343 | 0.3705072 | 0.6328256 | AIP | 9049 |
| ENSG00000110697 | 1088.922525 | 0.081200549 | 0.083178 | 0.2302936 | 0.4916046 | PITPNM1 | 9600 |
| ENSG00000167797 | 635.1350381 | -0.107445014 | 0.102011 | 0.1508971 | 0.3873059 | CDK2AP2 | 10263 |
| ENSG00000084207 | 2749.168408 | -0.159615538 | 0.0735394 | 0.0118928 | 0.0718676 | GSTP1 | 2950 |
| ENSG00000167792 | 2216.819347 | 0.065432868 | 0.0663351 | 0.260097 | 0.5271028 | NDUFV1 | 4723 |
| ENSG00000167799 | 71.6127105 | 0.003720974 | 0.1259384 | 0.9402359 | 0.9766571 | NUDT8 | 254552 |
| ENSG00000160172 | 68.21020057 | -0.014322989 | 0.1271979 | 0.7658931 | 0.8994294 | NA | NA |
| ENSG00000110057 | 340.4168253 | -0.000327154 | 0.1013267 | 0.9947551 | 0.9977447 | UNC93B1 | 81622 |
| ENSG00000006534 | 12.80555458 | 0.013693096 | 0.1354984 | 0.5696478 | NA | ALDH3B1 | 221 |
| ENSG00000110717 | 943.7133239 | -0.067294706 | 0.0881741 | 0.3267055 | 0.5948626 | NDUFS8 | 4728 |
| ENSG00000110719 | 1106.520784 | 0.00951195 | 0.0788662 | 0.8834501 | 0.9528582 | TCIRG1 | 10312 |
| ENSG00000255031 | 84.76187242 | -0.003709522 | 0.1241241 | 0.9411753 | 0.976977 | NA | NA |
| ENSG00000110721 | 398.4373018 | 0.022369416 | 0.098399 | 0.7457425 | 0.8893165 | CHKA | 1119 |
| ENSG00000110066 | 902.9402538 | -0.272605654 | 0.1026342 | 0.001262 | 0.0134506 | KMT5B | 51111 |
| ENSG00000171067 | 506.9006442 | 0.122172134 | 0.1113343 | 0.1185014 | 0.335893 | C11orf24 | 53838 |
| ENSG00000162337 | 936.2348951 | -0.031052041 | 0.0801021 | 0.6335575 | 0.8254975 | LRP5 | 4041 |
| ENSG00000110075 | 3207.85727 | -0.156525171 | 0.0644854 | 0.0064224 | 0.0458557 | PPP6R3 | 55291 |
| ENSG00000069482 | 9.608339812 | 0.023498035 | 0.1378642 | 0.2830899 | NA | GAL | 51083 |
| ENSG00000132749 | 62.61512027 | 0.045982088 | 0.1358532 | 0.3607888 | 0.6240743 | TESMIN | 9633 |
| ENSG00000110090 | 693.8654752 | 0.235466429 | 0.1219036 | 0.0096552 | 0.0623373 | CPT1A | 1374 |
| ENSG00000197345 | 818.3417977 | 0.02224831 | 0.0867442 | 0.7417103 | 0.887242 | MRPL21 | 219927 |
| ENSG00000132740 | 696.6847345 | 0.131102822 | 0.1030938 | 0.087073 | 0.2769879 | IGHMBP2 | 3508 |
| ENSG00000162341 | 388.6142675 | -0.131777794 | 0.1279489 | 0.1078309 | 0.317753 | TPCN2 | 219931 |
| ENSG00000172927 | 4090.713913 | -0.00623879 | 0.0572328 | 0.9037705 | 0.961996 | MYEOV | 26579 |
| ENSG00000260877 | 23.89215953 | -0.062164224 | 0.1528145 | 0.0808792 | NA | NA | NA |
| ENSG00000110092 | 12192.73518 | 0.014156045 | 0.059442 | 0.8000415 | 0.9169162 | CCND1 | 595 |
| ENSG00000149716 | 505.9501906 | 0.056940832 | 0.0981332 | 0.4447406 | 0.6929291 | LTO1 | 220064 |
| ENSG00000168040 | 811.8813065 | 0.04362797 | 0.0853759 | 0.5117261 | 0.7421745 | FADD | 8772 |

| | | | | | | | |
|---|---|---|---|---|---|---|---|
| ENSG00000254721 | 15.93347079 | 0.01243457 | 0.134609 | 0.6497696 | NA | NA | NA |
| ENSG00000131626 | 2247.655545 | -0.082188622 | 0.0684393 | 0.1657848 | 0.4101084 | PPFIA1 | 8500 |
| ENSG00000246889 | 66.61729772 | -0.070660965 | 0.1482364 | 0.1919737 | 0.4432584 | NA | NA |
| ENSG00000085733 | 7023.612574 | -0.051115712 | 0.053616 | 0.2975979 | 0.5680006 | CTTN | 2017 |
| ENSG00000162105 | 495.4229303 | -0.091029364 | 0.1048843 | 0.2206891 | 0.4803184 | SHANK2 | 22941 |
| ENSG00000172893 | 2645.968114 | -0.410179114 | 0.0802166 | 3.09E-08 | 1.68E-06 | DHCR7 | 1717 |
| ENSG00000254682 | 57.70569294 | -0.00769862 | 0.1276275 | 0.8661896 | 0.9459476 | NA | NA |
| ENSG00000172890 | 643.4219154 | -0.061882588 | 0.091835 | 0.3741304 | 0.6355756 | NADSYN1 | 55191 |
| ENSG00000158483 | 190.1633748 | 0.136995052 | 0.1618694 | 0.0983812 | 0.2991904 | FAM86C1P | 55199 |
| ENSG00000254469 | 33.49227433 | 0.004373383 | 0.1308603 | 0.910493 | NA | XNDC1N | 100133315 |
| ENSG00000137522 | 587.2314053 | 0.050534714 | 0.092393 | 0.467024 | 0.7094916 | RNF121 | 55298 |
| ENSG00000137496 | 278.0849468 | 0.029925292 | 0.1105392 | 0.6571334 | 0.8419409 | IL18BP | 10068 |
| ENSG00000137497 | 3872.132281 | -0.00984314 | 0.0578201 | 0.8503163 | 0.9386264 | NUMA1 | 4926 |
| ENSG00000251143 | 42.08648386 | -0.004841187 | 0.1294971 | 0.9059115 | 0.9628103 | LOC100128 | 100128494 |
| ENSG00000184154 | 142.5218612 | 0.041580004 | 0.1226439 | 0.508368 | 0.7401841 | LRRC51 | 120356739 |
| ENSG00000149357 | 850.2303918 | 0.014232245 | 0.08535 | 0.8319172 | 0.9304505 | LAMTOR1 | 55004 |
| ENSG00000110200 | 288.4925042 | -0.141495726 | 0.1415647 | 0.093898 | 0.2907029 | ANAPC15 | 25906 |
| ENSG00000165458 | 1888.498059 | -0.085397221 | 0.073485 | 0.170396 | 0.4162267 | INPPL1 | 3636 |
| ENSG00000162129 | 550.1063402 | -0.062203907 | 0.0960802 | 0.3774567 | 0.6392414 | CLPB | 81570 |
| ENSG00000186635 | 885.8308718 | -0.053183044 | 0.0868437 | 0.4328756 | 0.6841524 | ARAP1 | 116985 |
| ENSG00000214530 | 733.5952263 | -0.021704037 | 0.089945 | 0.7487451 | 0.8910111 | STARD10 | 10809 |
| ENSG00000168010 | 396.8907621 | 0.015235496 | 0.0980057 | 0.8253015 | 0.9282059 | ATG16L2 | 89849 |
| ENSG00000137478 | 695.1181006 | 0.013271662 | 0.0899951 | 0.8458408 | 0.9369622 | FCHSD2 | 9873 |
| ENSG00000175591 | 237.2948347 | 0.065592823 | 0.1199187 | 0.3533703 | 0.6187516 | P2RY2 | 5029 |
| ENSG00000260401 | 81.42591986 | 0.016978812 | 0.1250402 | 0.7486371 | 0.8910111 | NA | NA |
| ENSG00000171631 | 62.68101895 | 0.001295008 | 0.1277947 | 0.9788019 | 0.9921174 | P2RY6 | 5031 |
| ENSG00000257038 | 29.46637814 | -0.003412276 | 0.1319557 | 0.91915 | NA | ARHGEF17- | 100287837 |
| ENSG00000110237 | 843.4272739 | -0.11626784 | 0.0974501 | 0.1153967 | 0.3308662 | ARHGEF17 | 9828 |
| ENSG00000054967 | 904.0268711 | 0.201778159 | 0.1073888 | 0.0146331 | 0.0828001 | RELT | 84957 |
| ENSG00000054965 | 1537.699384 | -0.152138826 | 0.0812454 | 0.0248705 | 0.1202483 | FAM168A | 23201 |
| ENSG00000021300 | 47.37580623 | -0.051421186 | 0.1415842 | 0.2545276 | 0.5211459 | PLEKHB1 | 58473 |
| ENSG00000175582 | 2313.418429 | -0.039565123 | 0.0657179 | 0.4915154 | 0.7279563 | RAB6A | 5870 |
| ENSG00000256034 | 10.97002161 | -0.018023754 | 0.1365577 | 0.4157586 | NA | NA | NA |
| ENSG00000175581 | 319.9788291 | -0.14240543 | 0.1375258 | 0.0902871 | 0.283659 | MRPL48 | 51642 |
| ENSG00000181924 | 1149.141705 | 0.086657577 | 0.085306 | 0.2038401 | 0.4589703 | COA4 | 51287 |
| ENSG00000175575 | 164.6284458 | 0.053891615 | 0.1250041 | 0.4089751 | 0.6643076 | PAAF1 | 80227 |
| ENSG00000175567 | 620.2403171 | -0.072285006 | 0.1008427 | 0.3150299 | 0.58392 | UCP2 | 7351 |
| ENSG00000175564 | 20.49582415 | -0.047928434 | 0.1450771 | 0.1352677 | NA | UCP3 | 7352 |
| ENSG00000168014 | 1555.485465 | -0.085504498 | 0.0792037 | 0.1902515 | 0.4411132 | C2CD3 | 26005 |
| ENSG00000214517 | 4944.183016 | -0.571615154 | 0.065491 | 2.09E-19 | 6.09E-17 | PPME1 | 51400 |
| ENSG00000149380 | 22.32102257 | -0.015067461 | 0.1338392 | 0.6352018 | NA | P4HA3 | 283208 |
| ENSG00000165434 | 519.706005 | 0.196711852 | 0.1248538 | 0.0261956 | 0.1251813 | PGM2L1 | 283209 |
| ENSG00000175536 | 10.09618478 | 0.008969958 | 0.1352732 | 0.6843381 | NA | LIPT2 | 387787 |
| ENSG00000254837 | 25.1392553 | -0.022246249 | 0.1346248 | 0.5125069 | NA | LIPT2-AS1 | 100287896 |
| ENSG00000077514 | 1237.801279 | 0.157873606 | 0.0870118 | 0.0263988 | 0.1259641 | POLD3 | 10714 |
| ENSG00000166439 | 1225.461512 | 0.009760951 | 0.0790022 | 0.8807187 | 0.9518649 | RNF169 | 254225 |
| ENSG00000166435 | 180.1811283 | -0.046207665 | 0.1226208 | 0.4703045 | 0.7115591 | XRRA1 | 143570 |
| ENSG00000241170 | 25.55662841 | 0.077320097 | 0.1647915 | 0.0390323 | NA | NA | NA |
| ENSG00000118363 | 2032.096147 | -0.089323108 | 0.0691034 | 0.1349209 | 0.3616363 | SPCS2 | 9789 |
| ENSG00000162139 | 261.5799899 | -0.054176417 | 0.1131594 | 0.4379132 | 0.6880813 | NEU3 | 10825 |
| ENSG00000254429 | 9.393073997 | -0.003494954 | 0.13514 | 0.8627737 | NA | NA | NA |
| ENSG00000137486 | 1302.828126 | -0.330405781 | 0.0996063 | 0.0001125 | 0.001923 | ARRB1 | 408 |
| ENSG00000149273 | 23060.32687 | -0.094630968 | 0.050183 | 0.0420061 | 0.1728938 | RPS3 | 6188 |
| ENSG00000207445 | 15.57304832 | -0.02326243 | 0.1366367 | 0.3963235 | NA | SNORD15B | 114599 |
| ENSG00000149243 | 19.12147981 | 0.017122814 | 0.1347056 | 0.5705058 | NA | KLHL35 | 283212 |
| ENSG00000158555 | 141.4195828 | 0.04424756 | 0.1231406 | 0.484849 | 0.7233041 | GDPD5 | 81544 |
| ENSG00000149257 | 435.7662992 | 0.062292703 | 0.1067344 | 0.3854314 | 0.6459432 | SERPINH1 | 871 |
| ENSG00000062282 | 301.7284692 | 0.590307726 | 0.1710629 | 3.36E-05 | 0.0007055 | DGAT2 | 84649 |
| ENSG00000198382 | 515.2119522 | 0.11937772 | 0.1105524 | 0.128438 | 0.3524276 | UVRAG | 7405 |
| ENSG00000137492 | 1680.922551 | -0.045037151 | 0.0702052 | 0.4539296 | 0.6991498 | THAP12 | 5612 |
| ENSG00000179240 | 63.24759143 | -0.012377104 | 0.1276307 | 0.7914386 | 0.9127055 | GVQW3 | 100506127 |
| ENSG00000255135 | 51.22744184 | 0.059939171 | 0.1455365 | 0.205853 | 0.4608333 | EMSY-DT | 124902718 |
| ENSG00000158636 | 677.4947521 | -0.034082724 | 0.089374 | 0.6162565 | 0.815878 | EMSY | 56946 |
| ENSG00000182704 | 282.3826118 | -0.005116247 | 0.104488 | 0.938354 | 0.9761997 | TSKU | 25987 |
| ENSG00000261578 | 10.95807826 | -0.003461658 | 0.1347904 | 0.8760868 | NA | NA | NA |
| ENSG00000078124 | 963.7446566 | -0.120615381 | 0.0893822 | 0.0886352 | 0.2802783 | ACER3 | 55331 |
| ENSG00000149260 | 343.0573433 | -0.040834862 | 0.1049658 | 0.5574041 | 0.7766028 | CAPN5 | 726 |
| ENSG00000137474 | 28.46935655 | -0.01832097 | 0.1332381 | 0.606915 | NA | MYO7A | 4647 |
| ENSG00000149269 | 836.6230325 | 0.114069202 | 0.0957173 | 0.1191042 | 0.337029 | PAK1 | 5058 |
| ENSG00000074201 | 1342.616858 | -0.073862434 | 0.0825182 | 0.2679412 | 0.5361059 | CLNS1A | 1207 |

| | | | | | | | | |
|---|---|---|---|---|---|---|---|---|
| ENSG00000178301 | 43.65906783 | -0.031866426 | 0.1349572 | 0.433985 | 0.684681 | AQP11 | | 282679 |
| ENSG00000048649 | 1519.748551 | -0.11708473 | 0.0771341 | 0.070717 | 0.2420798 | RSF1 | | 51773 |
| ENSG00000087884 | 189.1467612 | -0.050494735 | 0.1193869 | 0.4509256 | 0.6977059 | AAMDC | | 28971 |
| ENSG00000255449 | 8.491748204 | -0.010403726 | 0.1357592 | 0.6002058 | NA | NA | NA | |
| ENSG00000149262 | 448.7334803 | -0.161832518 | 0.1252188 | 0.0569728 | 0.2110687 | INTS4 | | 92105 |
| ENSG00000151364 | 164.5086056 | 0.000567019 | 0.1160702 | 0.9562069 | 0.9825402 | KCTD14 | | 65987 |
| ENSG00000151366 | 472.9647529 | -0.060282217 | 0.1030232 | 0.3980456 | 0.6572761 | NDUFC2 | | 4718 |
| ENSG00000159063 | 368.1062608 | -0.118267149 | 0.1226878 | 0.1374561 | 0.365746 | ALG8 | | 79053 |
| ENSG00000246174 | 21.43192629 | 0.0312898 | 0.1378115 | 0.3311561 | NA | KCTD21-AS | | 100289388 |
| ENSG00000188997 | 202.5405704 | -0.094694056 | 0.1354212 | 0.2023231 | 0.4565069 | KCTD21 | | 283219 |
| ENSG00000118369 | 279.5900322 | -0.002947621 | 0.1072473 | 0.9634746 | 0.9851933 | USP35 | | 57558 |
| ENSG00000033327 | 50.59212823 | 0.125688342 | 0.2140295 | 0.0308239 | 0.1407013 | GAB2 | | 9846 |
| ENSG00000251323 | 15.00369253 | -0.01585815 | 0.1353464 | 0.5450074 | NA | LINC02728 | | 101928865 |
| ENSG00000137513 | 766.2417081 | -0.008931771 | 0.0841502 | 0.8920019 | 0.956385 | NARS2 | | 79731 |
| ENSG00000227097 | 113.4097544 | -0.025964691 | 0.1230738 | 0.6513933 | 0.8384491 | NA | NA | |
| ENSG00000182103 | 15.59940848 | -0.003009866 | 0.1339817 | 0.9076578 | NA | FAM181B | | 220382 |
| ENSG00000137509 | 1317.165671 | -0.075431817 | 0.0776273 | 0.2412362 | 0.5066307 | PRCP | | 5547 |
| ENSG00000165490 | 702.1783716 | 0.145154263 | 0.1107771 | 0.0693423 | 0.2392872 | DDIAS | | 220042 |
| ENSG00000137502 | 282.8234101 | 0.096658894 | 0.1256341 | 0.2056051 | 0.4607272 | RAB30 | | 27314 |
| ENSG00000246067 | 120.0338246 | 0.028855125 | 0.1241079 | 0.6158605 | 0.8158539 | NA | NA | |
| ENSG00000165494 | 1204.476654 | -0.017390774 | 0.0765565 | 0.7832608 | 0.9096561 | PCF11 | | 51585 |
| ENSG00000247137 | 38.14474467 | -0.033486735 | 0.1361214 | 0.3940076 | NA | ANKRD42-D | | 100506282 |
| ENSG00000269939 | 15.37086329 | -0.029451896 | 0.1384274 | 0.2819834 | NA | NA | NA | |
| ENSG00000137494 | 381.665117 | 0.358526051 | 0.1556629 | 0.0019135 | 0.0183057 | ANKRD42 | | 338699 |
| ENSG00000137500 | 434.9603792 | 0.062530252 | 0.104385 | 0.384048 | 0.6447633 | CCDC90B | | 60492 |
| ENSG00000150672 | 14.89965951 | -0.004180641 | 0.1341888 | 0.8690773 | NA | DLG2 | | 1740 |
| ENSG00000171204 | 859.1808685 | 0.086963077 | 0.0923161 | 0.2196681 | 0.479502 | TMEM126B | | 55863 |
| ENSG00000171202 | 451.4802456 | -0.047206926 | 0.0993234 | 0.5005224 | 0.7347747 | TMEM126A | | 84233 |
| ENSG00000137504 | 2063.072386 | 0.110546903 | 0.0711944 | 0.0712765 | 0.2432314 | CREBZF | | 58487 |
| ENSG00000137501 | 35.01852482 | 0.032451191 | 0.135984 | 0.4032755 | NA | SYTL2 | | 54843 |
| ENSG00000073921 | 2815.611607 | 0.013508281 | 0.0610491 | 0.8053294 | 0.9186624 | PICALM | | 8301 |
| ENSG00000074266 | 716.2349333 | -0.17959849 | 0.1128367 | 0.0306047 | 0.1401263 | EED | | 8726 |
| ENSG00000254783 | 22.26343169 | 0.001922462 | 0.1329104 | 0.9525863 | NA | LOC100289! | | 100289518 |
| ENSG00000149196 | 483.3236522 | -0.005666473 | 0.0954475 | 0.9317646 | 0.9735189 | HIKESHI | | 51501 |
| ENSG00000149201 | 11.37552911 | 0.029367141 | 0.1392515 | 0.2138996 | NA | CCDC81 | | 60494 |
| ENSG00000151376 | 228.491581 | -0.053645476 | 0.1159284 | 0.4372348 | 0.6876434 | ME3 | | 10873 |
| ENSG00000150687 | 1290.262305 | -0.181205056 | 0.194665 | 0.0518826 | 0.1969581 | PRSS23 | | 11098 |
| ENSG00000174804 | 198.5351249 | -0.039682391 | 0.1163754 | 0.5457251 | 0.7679526 | FZD4 | | 8322 |
| ENSG00000166575 | 394.0022506 | -0.20518542 | 0.1617871 | 0.0343643 | 0.1507922 | TMEM135 | | 65084 |
| ENSG00000123892 | 12.52455924 | 0.00395345 | 0.1346888 | 0.8678344 | NA | RAB38 | | 23682 |
| ENSG00000109861 | 910.7236084 | 0.062775785 | 0.0938946 | 0.3733179 | 0.6352113 | CTSC | | 1075 |
| ENSG00000110172 | 2473.703202 | -0.083578237 | 0.0672247 | 0.1532384 | 0.3908224 | CHORDC1 | | 26973 |
| ENSG00000180773 | 859.5706207 | -0.092117118 | 0.0896139 | 0.1866094 | 0.4373905 | SLC36A4 | | 120103 |
| ENSG00000166002 | 145.6176843 | -0.039792703 | 0.1215892 | 0.5261919 | 0.7534979 | SMCO4 | | 56935 |
| ENSG00000166004 | 821.7190069 | 0.093643892 | 0.0921806 | 0.187797 | 0.4387313 | CEP295 | | 85459 |
| ENSG00000254911 | 8.415534812 | -0.012311349 | 0.1359638 | 0.5386013 | NA | SCARNA9 | | 619383 |
| ENSG00000166012 | 2114.595834 | 0.030633217 | 0.0669298 | 0.6041982 | 0.8094551 | TAF1D | | 79101 |
| ENSG00000202314 | 11.54692507 | 0.016861194 | 0.1361177 | 0.4721623 | NA | SNORD6 | | 692075 |
| ENSG00000182919 | 239.0962701 | -0.208877851 | 0.1813377 | 0.0360884 | 0.1563609 | C11orf54 | | 28970 |
| ENSG00000042429 | 946.6095292 | -0.233805162 | 0.0987978 | 0.0036918 | 0.0299683 | MED17 | | 9440 |
| ENSG00000110218 | 677.8423039 | -0.065751757 | 0.0930214 | 0.3496489 | 0.6155613 | PANX1 | | 24145 |
| ENSG00000020922 | 1112.418204 | -0.036589886 | 0.0771054 | 0.5655655 | 0.782102 | MRE11 | | 4361 |
| ENSG00000255893 | 21.45652356 | -0.026378693 | 0.1364102 | 0.4046216 | NA | NA | NA | |
| ENSG00000168876 | 228.9154606 | 0.085342744 | 0.1283788 | 0.2440854 | 0.5098718 | ANKRD49 | | 54851 |
| ENSG00000196371 | 200.7684722 | -0.067080395 | 0.1240852 | 0.3344639 | 0.6019835 | FUT4 | | 2526 |
| ENSG00000166025 | 918.1244369 | 0.774539865 | 0.1003232 | 7.31E-16 | 1.45E-13 | AMOTL1 | | 154810 |
| ENSG00000150316 | 688.079489 | -0.171149543 | 0.1126466 | 0.0377513 | 0.161253 | CWC15 | | 51503 |
| ENSG00000186280 | 21.31444857 | 0.014743057 | 0.1339524 | 0.639763 | NA | KDM4D | | 55693 |
| ENSG00000180771 | 160.01893 | -0.355668302 | 0.2767277 | 0.0104465 | 0.0653939 | NA | NA | |
| ENSG00000149218 | 309.029805 | -0.098267288 | 0.1243352 | 0.1987938 | 0.4520362 | ENDOD1 | | 23052 |
| ENSG00000077458 | 554.7744462 | 0.039174911 | 0.0929128 | 0.5709453 | 0.786302 | FAM76B | | 143684 |
| ENSG00000166037 | 1195.196231 | 0.02116859 | 0.0764979 | 0.7393866 | 0.8857761 | CEP57 | | 9702 |
| ENSG00000087053 | 884.9945011 | -0.325914381 | 0.1032784 | 0.0001994 | 0.0030684 | MTMR2 | | 8898 |
| ENSG00000184384 | 15.84544613 | -0.000200357 | 0.1340758 | 0.991471 | NA | MAML2 | | 84441 |
| ENSG00000149231 | 676.5077792 | -0.113966086 | 0.099309 | 0.1251312 | 0.3471045 | CCDC82 | | 79780 |
| ENSG00000183340 | 161.9004743 | 0.02900734 | 0.1188925 | 0.6425599 | 0.8325202 | JRKL | | 8690 |
| ENSG00000165895 | 63.1017242 | 0.001661608 | 0.1262412 | 0.9740745 | 0.9902667 | ARHGAP42 | | 143872 |
| ENSG00000170647 | 11.03972977 | -0.003153467 | 0.1346676 | 0.8895105 | NA | NA | NA | |
| ENSG00000110318 | 27.33515284 | 0.051835547 | 0.1469879 | 0.1163794 | NA | CEP126 | | 57562 |
| ENSG00000137693 | 4495.525144 | -0.20379194 | 0.0627302 | 0.0003398 | 0.0047816 | YAP1 | | 10413 |

| | | | | | | | | |
|---|---|---|---|---|---|---|---|---|
| ENSG00000254422 | 83.6073623 | -0.045124515 | 0.1329358 | 0.4000621 | 0.6583091 | NA | NA | |
| ENSG00000023445 | 5048.526764 | 0.167298124 | 0.0694532 | 0.0060792 | 0.0437802 | BIRC3 | | 330 |
| ENSG00000110330 | 4114.357665 | 0.273200481 | 0.072117 | 2.76E-05 | 0.0005947 | BIRC2 | | 329 |
| ENSG00000152558 | 9371.461995 | -0.221854475 | 0.0595705 | 5.27E-05 | 0.0010349 | TMEM123 | | 114908 |
| ENSG00000196611 | 31.40648198 | 0.083927715 | 0.1685842 | 0.0498434 | NA | MMP1 | | 4312 |
| ENSG00000149968 | 25.13434885 | 3.879923057 | 0.7987838 | 1.48E-07 | NA | MMP3 | | 4314 |
| ENSG00000260966 | 30.62331123 | 0.001361249 | 0.1312461 | 0.9723925 | NA | NA | NA | |
| ENSG00000137692 | 1217.940423 | -0.053229071 | 0.0766621 | 0.4028841 | 0.6602257 | DCUN1D5 | | 84259 |
| ENSG00000187240 | 744.2264598 | -0.077543812 | 0.0948232 | 0.2752757 | 0.543704 | DYNC2H1 | | 79659 |
| ENSG00000170962 | 25.84195647 | -0.030366886 | 0.136811 | 0.3769029 | NA | PDGFD | | 80310 |
| ENSG00000196954 | 414.5610204 | 0.519948466 | 0.1469018 | 2.74E-05 | 0.0005913 | CASP4 | | 837 |
| ENSG00000170903 | 512.4975395 | -0.15009019 | 0.1180527 | 0.0678409 | 0.235778 | MSANTD4 | | 84437 |
| ENSG00000182359 | 46.22937301 | -0.024802805 | 0.1322009 | 0.5566023 | 0.7758558 | KBTBD3 | | 143879 |
| ENSG00000149313 | 1275.306416 | -0.004616827 | 0.0730585 | 0.9396579 | 0.9764957 | AASDHPPT | | 60496 |
| ENSG00000152402 | 14.60811118 | 0.053481746 | 0.1496951 | 0.0519151 | NA | GUCY1A2 | | 2977 |
| ENSG00000261098 | 26.65084592 | 0.005535257 | 0.1321146 | 0.8741233 | NA | NA | NA | |
| ENSG00000152404 | 407.2360416 | 0.027488357 | 0.0984602 | 0.6903653 | 0.8589854 | CWF19L2 | | 143884 |
| ENSG00000137760 | 606.539041 | 0.071372056 | 0.0959916 | 0.3165519 | 0.5851626 | ALKBH8 | | 91801 |
| ENSG00000110675 | 49.4661237 | -0.015437939 | 0.1294112 | 0.7269217 | 0.8779128 | ELMOD1 | | 55531 |
| ENSG00000110660 | 1977.041512 | -0.122118345 | 0.0762948 | 0.0570221 | 0.2111171 | SLC35F2 | | 54733 |
| ENSG00000166266 | 1242.891018 | -0.028133706 | 0.0745587 | 0.6522287 | 0.8389068 | CUL5 | | 8065 |
| ENSG00000075239 | 1640.238914 | -0.051309664 | 0.0714368 | 0.3994873 | 0.6582409 | ACAT1 | | 38 |
| ENSG00000149308 | 802.4465301 | 0.043205275 | 0.0849012 | 0.5191243 | 0.7478713 | NPAT | | 4863 |
| ENSG00000149311 | 2430.264713 | -0.111212127 | 0.0675042 | 0.0589041 | 0.2154423 | ATM | | 472 |
| ENSG00000166323 | 11.5302925 | 0.024955046 | 0.1380977 | 0.2741626 | NA | C11orf65 | | 160140 |
| ENSG00000178202 | 1267.026915 | -0.072249369 | 0.0784749 | 0.2643398 | 0.5321877 | POGLUT3 | | 143888 |
| ENSG00000110723 | 53.87096988 | 0.063795195 | 0.14695 | 0.1949756 | 0.4471845 | EXPH5 | | 23086 |
| ENSG00000178105 | 937.5760902 | 0.183974485 | 0.103152 | 0.0211049 | 0.1077003 | DDX10 | | 1662 |
| ENSG00000149289 | 807.4441342 | -0.064022023 | 0.0879315 | 0.3497398 | 0.6156284 | ZC3H12C | | 85463 |
| ENSG00000137710 | 3515.248887 | -0.019858067 | 0.05925 | 0.7079831 | 0.8678177 | RDX | | 5962 |
| ENSG00000137714 | 454.3222896 | -0.073458256 | 0.1030743 | 0.3116565 | 0.5815431 | FDX1 | | 2230 |
| ENSG00000170145 | 732.9810607 | 0.013453369 | 0.0847447 | 0.8406048 | 0.9344745 | SIK2 | | 23235 |
| ENSG00000137713 | 1208.098338 | -0.145505017 | 0.0880704 | 0.0402604 | 0.1683264 | PPP2R1B | | 5519 |
| ENSG00000086848 | 166.2145471 | -0.035761848 | 0.1217894 | 0.5611547 | 0.7795039 | ALG9 | | 79796 |
| ENSG00000255561 | 33.87618988 | 0.002291428 | 0.1307675 | 0.9539814 | NA | FDXACB1 | | 91893 |
| ENSG00000137720 | 46.36482794 | 0.011453644 | 0.1293611 | 0.7930709 | 0.9135057 | CFAP68 | | 64776 |
| ENSG00000150764 | 234.8556988 | 0.026231255 | 0.1098572 | 0.6964397 | 0.8615878 | DIXDC1 | | 85458 |
| ENSG00000150768 | 2054.748817 | -0.077897917 | 0.0681211 | 0.1874193 | 0.4383122 | DLAT | | 1737 |
| ENSG00000150773 | 18.80859122 | 0.008411848 | 0.1338594 | 0.7710227 | NA | PIH1D2 | | 120379 |
| ENSG00000150776 | 751.4498059 | 0.100796211 | 0.0989536 | 0.1707646 | 0.4168656 | NKAPD1 | | 55216 |
| ENSG00000150779 | 694.4342642 | -0.12832121 | 0.1058471 | 0.0964943 | 0.2953319 | TIMM8B | | 26521 |
| ENSG00000204370 | 936.6909296 | -0.03189531 | 0.0803689 | 0.6251294 | 0.8202285 | SDHD | | 6392 |
| ENSG00000150782 | 29.11337989 | 0.052173482 | 0.1453207 | 0.1711026 | NA | IL18 | | 3606 |
| ENSG00000150787 | 514.7934086 | -0.073194848 | 0.099703 | 0.3073443 | 0.5766285 | PTS | | 5805 |
| ENSG00000255129 | 11.98328339 | 0.004700679 | 0.1347788 | 0.8403362 | NA | NA | NA | |
| ENSG00000149292 | 144.7418662 | -0.024183635 | 0.1189082 | 0.691505 | 0.8594028 | TTC12 | | 54970 |
| ENSG00000166682 | 12.67046356 | -0.018487988 | 0.136231 | 0.4472332 | NA | TMPRSS5 | | 80975 |
| ENSG00000086827 | 804.5633574 | -0.174563126 | 0.1015312 | 0.0260934 | 0.124916 | ZW10 | | 9183 |
| ENSG00000048028 | 701.6157741 | -0.150127236 | 0.1057034 | 0.0561135 | 0.2084139 | USP28 | | 57646 |
| ENSG00000166741 | 454.5657934 | 0.093073796 | 0.1220166 | 0.2196223 | 0.479502 | NNMT | | 4837 |
| ENSG00000180425 | 27.37545246 | -0.041182842 | 0.1406612 | 0.242992 | NA | C11orf71 | | 54494 |
| ENSG00000076053 | 392.2788393 | 0.009592701 | 0.0992364 | 0.8894219 | 0.9549168 | RBM7 | | 10179 |
| ENSG00000076043 | 904.3926384 | -0.059468417 | 0.0840063 | 0.3704194 | 0.6328256 | REXO2 | | 25996 |
| ENSG00000137656 | 447.2486852 | -0.051595555 | 0.1004009 | 0.4640698 | 0.708231 | BUD13 | | 84811 |
| ENSG00000231611 | 15.29284375 | 0.034753708 | 0.1405829 | 0.1863482 | NA | NA | NA | |
| ENSG00000109917 | 1313.594065 | 0.33451278 | 0.0904788 | 2.77E-05 | 0.000595 | ZPR1 | | 8882 |
| ENSG00000160584 | 668.3196777 | -0.040410342 | 0.0904073 | 0.5549059 | 0.7749708 | SIK3 | | 23387 |
| ENSG00000168092 | 2624.112287 | -0.128006362 | 0.0739523 | 0.0424536 | 0.1741833 | PAFAH1B2 | | 5049 |
| ENSG00000149577 | 273.1085454 | -0.045587899 | 0.1103798 | 0.5084694 | 0.7401841 | SIDT2 | | 51092 |
| ENSG00000149591 | 427.2320256 | 0.36141281 | 0.1527734 | 0.0016165 | 0.0162219 | TAGLN | | 6876 |
| ENSG00000160613 | 1020.038996 | -0.04696756 | 0.0802648 | 0.4738079 | 0.7139512 | PCSK7 | | 9159 |
| ENSG00000167257 | 367.58434 | 0.007338687 | 0.1028695 | 0.9151734 | 0.9662935 | RNF214 | | 257160 |
| ENSG00000186318 | 51.34849596 | 0.087433985 | 0.1658818 | 0.0906817 | 0.284609 | BACE1 | | 23621 |
| ENSG00000110274 | 767.2029739 | -0.026764592 | 0.0845387 | 0.6870355 | 0.857187 | CEP164 | | 22897 |
| ENSG00000160588 | 11.10537293 | 0.023208738 | 0.13749 | 0.3241216 | NA | MPZL3 | | 196264 |
| ENSG00000110344 | 1643.614523 | 0.050118248 | 0.0706 | 0.4080462 | 0.6640358 | UBE4A | | 9354 |
| ENSG00000254873 | 53.38463367 | 0.009250383 | 0.1295027 | 0.8295987 | 0.9299052 | NA | NA | |
| ENSG00000167283 | 3405.264863 | -0.094659665 | 0.0621989 | 0.0872711 | 0.2773161 | ATP5MG | | 10632 |
| ENSG00000118058 | 4068.659464 | 0.026530241 | 0.0622574 | 0.6324852 | 0.8251856 | KMT2A | | 4297 |
| ENSG00000149582 | 108.6650994 | 0.020095129 | 0.1219354 | 0.7276522 | 0.8782138 | TMEM25 | | 84866 |

| | | | | | | | | |
|---|---|---|---|---|---|---|---|---|
| ENSG00000118096 | 126.0693209 | -0.033094343 | 0.1221607 | 0.5833483 | 0.7949132 | IFT46 | | 56912 |
| ENSG00000095139 | 4413.904835 | 0.112142244 | 0.0623385 | 0.0434839 | 0.1766733 | ARCN1 | | 372 |
| ENSG00000019144 | 631.4614305 | -0.063061303 | 0.0926923 | 0.3670317 | 0.629929 | PHLDB1 | | 23187 |
| ENSG00000110367 | 3095.786385 | -0.304688399 | 0.0709047 | 2.63E-06 | 7.89E-05 | DDX6 | | 1656 |
| ENSG00000186174 | 3915.289659 | -0.064965304 | 0.0619921 | 0.2395738 | 0.5051375 | BCL9L | | 283149 |
| ENSG00000255121 | 35.33804687 | 0.057984971 | 0.1482223 | 0.1452613 | NA | NA | NA | |
| ENSG00000186166 | 436.770111 | 0.033655026 | 0.1015095 | 0.6271192 | 0.8222448 | CENATAC | | 338657 |
| ENSG00000118181 | 8734.172806 | -0.06299088 | 0.0536676 | 0.2005681 | 0.454126 | RPS25 | | 6230 |
| ENSG00000196655 | 649.5004954 | -0.034064826 | 0.0886146 | 0.6149544 | 0.8157779 | TRAPPC4 | | 51399 |
| ENSG00000137700 | 554.3545393 | -0.13015458 | 0.1097057 | 0.0968516 | 0.2959256 | SLC37A4 | | 2542 |
| ENSG00000255114 | 16.53791634 | -0.012569482 | 0.1346071 | 0.6437942 | NA | NA | NA | |
| ENSG00000149428 | 5215.870319 | -0.171181355 | 0.0602357 | 0.0017404 | 0.0170985 | HYOU1 | | 10525 |
| ENSG00000160695 | 679.7291975 | 0.070885673 | 0.0952618 | 0.3190976 | 0.5876606 | VPS11 | | 55823 |
| ENSG00000256269 | 522.6894224 | -0.113151896 | 0.112798 | 0.1455298 | 0.3780659 | HMBS | | 3145 |
| ENSG00000188486 | 3462.039726 | -0.130605897 | 0.0699589 | 0.0313905 | 0.1422309 | H2AX | | 3014 |
| ENSG00000172269 | 597.3586987 | -0.02475628 | 0.0896112 | 0.7141761 | 0.8707119 | DPAGT1 | | 1798 |
| ENSG00000172375 | 382.293933 | 0.030557334 | 0.1010427 | 0.6593795 | 0.8426512 | C2CD2L | | 9854 |
| ENSG00000172273 | 480.2751777 | 0.120099891 | 0.1135961 | 0.1300777 | 0.3544317 | HINFP | | 25988 |
| ENSG00000172350 | 14.84903275 | -0.015159682 | 0.1351591 | 0.567918 | NA | ABCG4 | | 64137 |
| ENSG00000160703 | 289.7422338 | 0.114818568 | 0.1327192 | 0.148976 | 0.383724 | NLRX1 | | 79671 |
| ENSG00000248712 | 9.508702463 | -0.023686476 | 0.1380407 | 0.2599469 | NA | CCDC153 | | 283152 |
| ENSG00000110395 | 3270.35727 | 0.007050718 | 0.063134 | 0.900549 | 0.9610169 | CBL | | 867 |
| ENSG00000076706 | 1132.970548 | 0.251985701 | 0.1064066 | 0.0031526 | 0.0264934 | MCAM | | 4162 |
| ENSG00000173456 | 1372.813278 | -0.185498065 | 0.0851303 | 0.0089629 | 0.0591166 | RNF26 | | 79102 |
| ENSG00000036672 | 31.25758236 | 0.005304977 | 0.1317136 | 0.8833538 | NA | USP2 | | 9099 |
| ENSG00000245248 | 128.0732531 | 0.092598004 | 0.1485209 | 0.1804537 | 0.4299157 | NA | NA | |
| ENSG00000110400 | 358.9629834 | -0.140341089 | 0.1309759 | 0.0925788 | 0.2876914 | NECTIN1 | | 5818 |
| ENSG00000184232 | 42.77555898 | 0.018597248 | 0.1311911 | 0.6559114 | 0.8413904 | OAF | | 220323 |
| ENSG00000181264 | 179.9770864 | 0.307716842 | 0.2440369 | 0.0143606 | 0.0818271 | TLCD5 | | 219902 |
| ENSG00000196914 | 4817.460572 | -0.386977031 | 0.0616458 | 3.91E-11 | 3.69E-09 | ARHGEF12 | | 23365 |
| ENSG00000154114 | 134.0402982 | 0.043801188 | 0.1239871 | 0.4839004 | 0.7223311 | TBCEL | | 219899 |
| ENSG00000109929 | 784.5267064 | -0.149884739 | 0.1092779 | 0.0600295 | 0.2180361 | SC5D | | 6309 |
| ENSG00000137642 | 210.2190931 | -0.759068924 | 0.2307842 | 4.86E-05 | 0.0009599 | SORL1 | | 6653 |
| ENSG00000255248 | 17.445677 | 0.034429435 | 0.139976 | 0.2243867 | NA | MIR100HG | | 399959 |
| ENSG00000154127 | 5557.106546 | -0.240495205 | 0.0645704 | 4.44E-05 | 0.0008894 | UBASH3B | | 84959 |
| ENSG00000109944 | 73.34480265 | 0.031161153 | 0.1288364 | 0.5507543 | 0.7717564 | JHY | | 79864 |
| ENSG00000109971 | 34192.80059 | -0.259440863 | 0.0512001 | 8.43E-08 | 4.12E-06 | HSPA8 | | 3312 |
| ENSG00000200879 | 11.15217036 | 0.012607902 | 0.1358159 | 0.5537383 | NA | SNORD14E | | 85391 |
| ENSG00000166261 | 457.0334036 | 0.098564792 | 0.111068 | 0.1950648 | 0.4473011 | ZNF202 | | 7753 |
| ENSG00000154144 | 767.9456953 | 0.385283648 | 0.1196524 | 0.0001233 | 0.0020667 | TBRG1 | | 84897 |
| ENSG00000110013 | 257.4703977 | 0.135497288 | 0.1468289 | 0.1050667 | 0.3116517 | SIAE | | 54414 |
| ENSG00000064199 | 282.0157657 | -0.204717532 | 0.1709077 | 0.0366838 | 0.1580609 | SPA17 | | 53340 |
| ENSG00000154146 | 332.4164863 | -0.039397189 | 0.10481 | 0.5703314 | 0.7857107 | NRGN | | 4900 |
| ENSG00000149564 | 1590.115434 | 0.156221495 | 0.0806835 | 0.020875 | 0.1067604 | ESAM | | 90952 |
| ENSG00000250073 | 22.49008192 | -0.020945189 | 0.1350924 | 0.5035972 | NA | ESAM-AS1 | | 101929340 |
| ENSG00000120458 | 178.8447248 | 0.006535705 | 0.1139703 | 0.9187925 | 0.9683988 | MSANTD2 | | 79684 |
| ENSG00000245498 | 11.29264518 | 0.013094532 | 0.13548 | 0.5801964 | NA | MSANTD2-A | | 100507283 |
| ENSG00000154134 | 192.3794898 | 0.957256499 | 0.205526 | 1.66E-07 | 7.29E-06 | ROBO3 | | 64221 |
| ENSG00000154133 | 65.84253434 | 1.249714434 | 0.3778316 | 4.01E-05 | 0.0008213 | ROBO4 | | 54538 |
| ENSG00000149548 | 121.6780944 | 0.934703295 | 0.2629678 | 1.76E-05 | 0.0004085 | CCDC15 | | 80071 |
| ENSG00000134955 | 15.40563963 | 0.041591266 | 0.1432972 | 0.1250261 | NA | SLC37A2 | | 219855 |
| ENSG00000150433 | 207.348009 | 0.003868693 | 0.1109853 | 0.9536147 | 0.9817188 | TMEM218 | | 219854 |
| ENSG00000149557 | 53.26353765 | 0.018356615 | 0.1296462 | 0.684119 | 0.8553135 | FEZ1 | | 9638 |
| ENSG00000149547 | 2712.228295 | -0.161607948 | 0.0690181 | 0.007484 | 0.051333 | EI24 | | 9538 |
| ENSG00000134910 | 3021.169428 | -0.114964337 | 0.0641887 | 0.0428059 | 0.1749231 | STT3A | | 3703 |
| ENSG00000149554 | 1532.045947 | 0.066210313 | 0.0729932 | 0.2851925 | 0.5546831 | CHEK1 | | 1111 |
| ENSG00000198331 | 657.1615058 | -0.147641668 | 0.1087589 | 0.0630755 | 0.2250413 | HYLS1 | | 219844 |
| ENSG00000110060 | 124.7534371 | 0.138982361 | 0.1893628 | 0.0766646 | 0.2549883 | PUS3 | | 83480 |
| ENSG00000064309 | 164.7610571 | -0.023585408 | 0.1166365 | 0.7066688 | 0.8672619 | CDON | | 50937 |
| ENSG00000165526 | 1106.932271 | -0.166538475 | 0.0919054 | 0.0240496 | 0.1177381 | RPUSD4 | | 84881 |
| ENSG00000254694 | 37.55536984 | 0.032400455 | 0.1357686 | 0.4115008 | NA | NA | NA | |
| ENSG00000197798 | 651.8676741 | -0.021944852 | 0.0876398 | 0.7440825 | 0.8887145 | FAM118B | | 79607 |
| ENSG00000182934 | 2707.220088 | -0.153210859 | 0.0693289 | 0.0112957 | 0.069315 | SRPRA | | 6734 |
| ENSG00000110074 | 970.3677372 | 0.026300714 | 0.0790539 | 0.6845776 | 0.8554818 | FOXRED1 | | 55572 |
| ENSG00000150455 | 192.3745936 | 0.026920452 | 0.1162359 | 0.6741314 | 0.8501975 | TIRAP | | 114609 |
| ENSG00000255062 | 19.30824746 | 0.006343788 | 0.1337334 | 0.8261168 | NA | NA | NA | |
| ENSG00000110063 | 427.0797004 | -0.224242374 | 0.1403834 | 0.0189197 | 0.0997748 | DCPS | | 28960 |
| ENSG00000110080 | 379.8671659 | 0.164720584 | 0.1355225 | 0.0596183 | 0.2171093 | ST3GAL4 | | 6484 |
| ENSG00000254612 | 210.1964496 | -0.079606337 | 0.1327995 | 0.2570594 | 0.5240334 | DNAJB6P1 | | 387820 |
| ENSG00000134954 | 8225.354934 | 0.288512893 | 0.0562346 | 5.13E-08 | 2.63E-06 | ETS1 | | 2113 |

| | | | | | | | |
|---|---|---|---|---|---|---|---|
| ENSG00000174370 | 30.41335147 | 0.001097826 | 0.1315257 | 0.977572 | NA | KCNJ5-AS1 | 219833 |
| ENSG00000134909 | 593.1012979 | -0.083025175 | 0.1030323 | 0.2554164 | 0.5225076 | ARHGAP32 | 9743 |
| ENSG00000170322 | 1299.602374 | -0.06358747 | 0.0783073 | 0.3246871 | 0.593455 | NFRKB | 4798 |
| ENSG00000170325 | 690.2957608 | -0.108369802 | 0.0977961 | 0.1406061 | 0.3701299 | PRDM10 | 56980 |
| ENSG00000084234 | 8712.849527 | 0.053447121 | 0.0511834 | 0.2585971 | 0.5253712 | APLP2 | 334 |
| ENSG00000196323 | 1104.47622 | -0.11754851 | 0.0890719 | 0.0956091 | 0.2938201 | ZBTB44 | 29068 |
| ENSG00000120451 | 239.2295918 | 0.179857472 | 0.1688656 | 0.0536545 | 0.2022387 | SNX19 | 399979 |
| ENSG00000151503 | 3344.644871 | -0.126798106 | 0.0632609 | 0.0240934 | 0.1178539 | NCAPD3 | 23310 |
| ENSG00000151502 | 1294.140035 | -0.034066368 | 0.0746915 | 0.5860615 | 0.7968554 | VPS26B | 112936 |
| ENSG00000151500 | 341.6107804 | -0.24780402 | 0.1572765 | 0.015705 | 0.087045 | THYN1 | 29087 |
| ENSG00000151498 | 846.6696563 | 0.026456377 | 0.0830164 | 0.6897922 | 0.8589854 | ACAD8 | 27034 |
| ENSG00000226210 | 512.0716783 | 0.073890652 | 0.1002113 | 0.3069982 | 0.5762568 | WASH8P | 100288778 |
| ENSG00000261799 | 51.2359099 | -0.006765697 | 0.1285805 | 0.8759239 | 0.9497329 | NA | NA |
| ENSG00000073614 | 1580.367008 | -0.068298334 | 0.0720735 | 0.2661261 | 0.5340997 | KDM5A | 5927 |
| ENSG00000120647 | 346.7827802 | 0.110225996 | 0.1226708 | 0.1612722 | 0.4033013 | CCDC77 | 84318 |
| ENSG00000139044 | 125.4758638 | 0.000580192 | 0.1184367 | 0.9933507 | 0.9975317 | B4GALNT3 | 283358 |
| ENSG00000177406 | 12.00115699 | 0.030037844 | 0.1394758 | 0.2042306 | NA | NINJ2-AS1 | 100049716 |
| ENSG00000060237 | 5212.83507 | -0.101456829 | 0.0607096 | 0.0608657 | 0.2199792 | WNK1 | 65125 |
| ENSG00000002016 | 189.7659257 | 0.01284039 | 0.1130264 | 0.8423423 | 0.9350382 | RAD52 | 5893 |
| ENSG00000250132 | 32.38876351 | -0.007913443 | 0.1312225 | 0.8313841 | NA | NA | NA |
| ENSG00000082805 | 1671.989323 | -0.152139092 | 0.0797576 | 0.0230369 | 0.114362 | ERC1 | 23085 |
| ENSG00000111186 | 1672.312365 | -0.073058428 | 0.0717726 | 0.2399771 | 0.5054546 | WNT5B | 81029 |
| ENSG00000171823 | 163.7600607 | -0.126556917 | 0.1621706 | 0.1101158 | 0.3214049 | FBXL14 | 144699 |
| ENSG00000006831 | 2939.070286 | -0.166178558 | 0.0667272 | 0.0049483 | 0.0376802 | ADIPOR2 | 79602 |
| ENSG00000004478 | 3452.239825 | 0.001580346 | 0.0587419 | 0.9747325 | 0.9905193 | FKBP4 | 2288 |
| ENSG00000258092 | 43.21326194 | 0.032672738 | 0.1360147 | 0.4027445 | 0.6601399 | NA | NA |
| ENSG00000111203 | 276.286807 | -0.032468516 | 0.1073518 | 0.6339843 | 0.8257229 | ITFG2 | 55846 |
| ENSG00000111206 | 3350.145006 | -0.110476238 | 0.0623149 | 0.0463599 | 0.1841023 | FOXM1 | 2305 |
| ENSG00000171792 | 651.2054922 | 0.003631108 | 0.0873862 | 0.9578053 | 0.9829665 | RHNO1 | 83695 |
| ENSG00000078246 | 768.5990384 | -0.019853303 | 0.0841522 | 0.7641482 | 0.8983744 | TULP3 | 7289 |
| ENSG00000197905 | 1177.642091 | 0.1068173 | 0.082216 | 0.1133552 | 0.3268581 | TEAD4 | 7004 |
| ENSG00000250899 | 777.8460463 | -0.038045292 | 0.0860017 | 0.576719 | 0.7902235 | NA | NA |
| ENSG00000011105 | 299.0680671 | -0.002028464 | 0.1032327 | 0.9744646 | 0.9904047 | TSPAN9 | 10867 |
| ENSG00000227081 | 132.8966593 | -0.049240202 | 0.1268472 | 0.4278042 | 0.6791494 | NA | NA |
| ENSG00000130038 | 103.280275 | -0.026876541 | 0.1249471 | 0.6280688 | 0.8226951 | CRACR2A | 84766 |
| ENSG00000111224 | 92.13872922 | -0.027337176 | 0.1282589 | 0.5912272 | 0.8006172 | PARP11 | 57097 |
| ENSG00000118971 | 8.745941732 | 0.035201478 | 0.1420159 | 0.0789293 | NA | CCND2 | 894 |
| ENSG00000078237 | 568.7079165 | -0.104978038 | 0.1059467 | 0.164286 | 0.4076092 | TIGAR | 57103 |
| ENSG00000047621 | 475.6798331 | 0.086876395 | 0.1069903 | 0.2476403 | 0.5139811 | C12orf4 | 57102 |
| ENSG00000111247 | 1046.261955 | 0.064183296 | 0.0848569 | 0.3420362 | 0.6088612 | RAD51AP1 | 10635 |
| ENSG00000010219 | 87.84447053 | -0.071429181 | 0.1440772 | 0.2268373 | 0.4881315 | DYRK4 | 8798 |
| ENSG00000139180 | 273.7258188 | -0.057020679 | 0.113142 | 0.4143574 | 0.6685232 | NDUFA9 | 4704 |
| ENSG00000010278 | 6451.114996 | 0.176417816 | 0.0660819 | 0.002738 | 0.024017 | CD9 | 928 |
| ENSG00000067182 | 1061.670955 | 0.195444216 | 0.0924307 | 0.0094887 | 0.0615764 | TNFRSF1A | 7132 |
| ENSG00000111321 | 393.7205442 | 0.015630153 | 0.0979237 | 0.8208092 | 0.9261855 | LTBR | 4055 |
| ENSG00000215039 | 22.33411197 | 0.025511637 | 0.1359334 | 0.4337389 | NA | CD27-AS1 | 678655 |
| ENSG00000139192 | 35.11062312 | 0.059773527 | 0.1485017 | 0.1520039 | NA | TAPBPL | 55080 |
| ENSG00000139190 | 182.6114693 | 0.030266786 | 0.115664 | 0.6410384 | 0.8315061 | VAMP1 | 6843 |
| ENSG00000111639 | 1922.138595 | -0.14020849 | 0.073192 | 0.0256913 | 0.1233455 | MRPL51 | 51258 |
| ENSG00000010292 | 5736.665155 | -0.309527113 | 0.0648165 | 2.80E-07 | 1.14E-05 | NCAPD2 | 9918 |
| ENSG00000111640 | 38625.07874 | -0.13268943 | 0.0497615 | 0.0043507 | 0.0339708 | GAPDH | 2597 |
| ENSG00000269968 | 187.5223104 | -0.010287849 | 0.1296942 | 0.8050938 | 0.9186624 | NA | NA |
| ENSG00000111641 | 3285.454911 | 0.08519081 | 0.0613984 | 0.1200883 | 0.3385017 | NOP2 | 4839 |
| ENSG00000111642 | 5759.605545 | -0.325528649 | 0.0638342 | 4.88E-08 | 2.51E-06 | CHD4 | 1108 |
| ENSG00000247853 | 40.61039262 | -0.026605289 | 0.1339109 | 0.4974253 | NA | NA | NA |
| ENSG00000111653 | 171.5560298 | 0.073494169 | 0.1310898 | 0.2860529 | 0.5559707 | ING4 | 51147 |
| ENSG00000126746 | 1283.583478 | 0.093882119 | 0.0793633 | 0.1509325 | 0.3873116 | ZNF384 | 171017 |
| ENSG00000111652 | 943.2150666 | -0.019767319 | 0.0838242 | 0.7646067 | 0.8986187 | COPS7A | 50813 |
| ENSG00000089693 | 3796.931633 | -0.095941254 | 0.0609211 | 0.0780903 | 0.2577489 | MLF2 | 8079 |
| ENSG00000159335 | 3367.124437 | -0.137793186 | 0.0633813 | 0.0145017 | 0.08227 | PTMS | 5763 |
| ENSG00000089692 | 13.77708452 | 0.018170489 | 0.1359322 | 0.4784473 | NA | LAG3 | 3902 |
| ENSG00000110811 | 117.9770006 | -0.030533594 | 0.1227389 | 0.605243 | 0.8103616 | P3H3 | 10536 |
| ENSG00000111664 | 13.41900728 | -0.001503932 | 0.1342827 | 0.9496155 | NA | GNB3 | 2784 |
| ENSG00000111665 | 1100.232551 | -0.092373649 | 0.0822027 | 0.1671053 | 0.4122331 | CDCA3 | 83461 |
| ENSG00000111667 | 2545.240466 | -0.171185057 | 0.0736862 | 0.0072863 | 0.0502427 | USP5 | 8078 |
| ENSG00000111669 | 16634.61102 | -0.175879293 | 0.0527157 | 0.0003211 | 0.0045685 | TPI1 | 7167 |
| ENSG00000111671 | 112.2494075 | 0.294030405 | 0.3340357 | 0.018268 | 0.097393 | SPSB2 | 84727 |
| ENSG00000240370 | 46.63356914 | -0.066158577 | 0.1498346 | 0.1616403 | 0.4037019 | RPL13P5 | 283345 |
| ENSG00000010626 | 90.48216929 | 0.066520796 | 0.1405244 | 0.2613178 | 0.5288381 | LRRC23 | 10233 |
| ENSG00000248593 | 75.8918109 | -0.096925412 | 0.165759 | 0.1141551 | 0.3285968 | NA | NA |

| | | | | | | | |
|---|---|---|---|---|---|---|---|
| ENSG00000111674 | 703.3436824 | 0.248758055 | 0.1120287 | 0.0046186 | 0.0357287 | ENO2 | 2026 |
| ENSG00000111676 | 2269.051313 | -0.011776749 | 0.0698236 | 0.8420965 | 0.9350382 | ATN1 | 1822 |
| ENSG00000111678 | 573.6289156 | 0.157469722 | 0.1146309 | 0.0549542 | 0.2055108 | C12orf57 | 113246 |
| ENSG00000126749 | 1136.730657 | -0.014714665 | 0.0815723 | 0.8248877 | 0.9280086 | EMG1 | 10436 |
| ENSG00000215021 | 3742.024571 | -0.155937907 | 0.0632087 | 0.0058226 | 0.0425844 | PHB2 | 11331 |
| ENSG00000111684 | 679.4478176 | -0.147996324 | 0.1084285 | 0.0620918 | 0.2224145 | LPCAT3 | 10162 |
| ENSG00000272201 | 137.8585306 | 0.10174311 | 0.1518522 | 0.1578466 | 0.3976292 | NA | NA |
| ENSG00000182326 | 9.802890808 | 0.02756314 | 0.1390552 | 0.2027184 | NA | C1S | 716 |
| ENSG00000139178 | 170.991346 | 0.05197234 | 0.1218077 | 0.4344172 | 0.6852379 | C1RL | 51279 |
| ENSG00000205885 | 275.1802085 | -0.33840337 | 0.1852513 | 0.0055735 | 0.0411794 | C1RL-AS1 | 283314 |
| ENSG00000139182 | 744.0296179 | -0.230099282 | 0.1086425 | 0.0066861 | 0.0472362 | CLSTN3 | 9746 |
| ENSG00000139197 | 963.1416788 | 0.082750452 | 0.0847152 | 0.2246788 | 0.4852777 | PEX5 | 5830 |
| ENSG00000177675 | 67.88495283 | 0.11709384 | 0.1898037 | 0.0659016 | 0.2320448 | CD163L1 | 283316 |
| ENSG00000065970 | 517.2672172 | -0.016226578 | 0.0918912 | 0.810856 | 0.9219906 | FOXJ2 | 55810 |
| ENSG00000089818 | 629.7051199 | -0.00773211 | 0.0887224 | 0.9070159 | 0.963256 | NECAP1 | 25977 |
| ENSG00000166523 | 20.06009129 | 0.006271713 | 0.1333524 | 0.8369811 | NA | CLEC4E | 26253 |
| ENSG00000166532 | 758.7091114 | -0.100523336 | 0.0981202 | 0.1695587 | 0.4153346 | RIMKLB | 57494 |
| ENSG00000111752 | 553.7793258 | -0.041992468 | 0.093106 | 0.5428267 | 0.7657126 | PHC1 | 1911 |
| ENSG00000003056 | 2632.828495 | -0.079573077 | 0.0678022 | 0.1765955 | 0.4256732 | M6PR | 4074 |
| ENSG00000231503 | 56.8067815 | -0.082655358 | 0.1597246 | 0.1193636 | 0.3373545 | NA | NA |
| ENSG00000111788 | 564.0067622 | 0.043712196 | 0.0942517 | 0.529776 | 0.7555939 | NA | NA |
| ENSG00000256673 | 17.0791922 | -0.013770985 | 0.1345479 | 0.6258482 | NA | NA | NA |
| ENSG00000214826 | 852.3548268 | -0.082331222 | 0.0880348 | 0.2313761 | 0.4934643 | NA | NA |
| ENSG00000256594 | 54.95004755 | 0.030266371 | 0.1317529 | 0.5162218 | 0.7456396 | NA | NA |
| ENSG00000257027 | 36.47142778 | -0.010346102 | 0.1307896 | 0.7913245 | NA | NA | NA |
| ENSG00000069493 | 36.26690614 | -0.011514426 | 0.1309105 | 0.7687657 | NA | CLEC2D | 29121 |
| ENSG00000213443 | 8.521660146 | -0.020668195 | 0.1375455 | 0.2930759 | NA | NA | NA |
| ENSG00000139112 | 222.092409 | 0.537295607 | 0.1990384 | 0.0003983 | 0.0054168 | GABARAPL: | 23710 |
| ENSG00000245648 | 12.43123714 | -0.013364663 | 0.1355184 | 0.5676075 | NA | KLRK1-AS1 | 101928100 |
| ENSG00000205810 | 10.14873854 | -0.006111886 | 0.1351133 | 0.7742396 | NA | KLRC3 | 3823 |
| ENSG00000205809 | 29.7206037 | -0.033818507 | 0.1371362 | 0.3583523 | NA | KLRC2 | 3822 |
| ENSG00000256667 | 55.7737696 | -0.008531158 | 0.1277518 | 0.8516079 | 0.9396585 | NA | NA |
| ENSG00000111196 | 426.9523408 | -0.06866117 | 0.1076354 | 0.3511925 | 0.6171375 | MAGOHB | 55110 |
| ENSG00000060140 | 23.97766623 | 0.02224096 | 0.1348369 | 0.5072222 | NA | STYK1 | 55359 |
| ENSG00000060138 | 3915.295875 | 0.037745892 | 0.0609092 | 0.488972 | 0.7262901 | YBX3 | 8531 |
| ENSG00000111215 | 32.52471442 | 0.012387106 | 0.1316312 | 0.7448517 | NA | PRH1 | 5554 |
| ENSG00000212127 | 18.43144119 | 0.004339312 | 0.1334754 | 0.8833688 | NA | TAS2R14 | 50840 |
| ENSG00000256537 | 179.8057955 | -0.044336803 | 0.1231339 | 0.4823971 | 0.7213706 | SMIM10L1 | 100129361 |
| ENSG00000139083 | 271.1535706 | -0.057029638 | 0.1131145 | 0.4152115 | 0.6694756 | ETV6 | 2120 |
| ENSG00000198134 | 55.51393936 | -0.02774924 | 0.131437 | 0.5432561 | 0.7660278 | NA | NA |
| ENSG00000070018 | 1026.905391 | -0.05855066 | 0.0876958 | 0.3862947 | 0.6470187 | LRP6 | 4040 |
| ENSG00000111261 | 78.0790652 | -0.011598927 | 0.1246927 | 0.8226319 | 0.9272789 | MANSC1 | 54682 |
| ENSG00000205791 | 9.451579096 | 0.000735987 | 0.1349324 | 0.9749429 | NA | LOH12CR2 | 503693 |
| ENSG00000165714 | 82.14101586 | 0.090540928 | 0.1589082 | 0.1399187 | 0.3693538 | BORCS5 | 118426 |
| ENSG00000111266 | 260.6754822 | 0.102095491 | 0.1306586 | 0.1850767 | 0.4355996 | DUSP16 | 80824 |
| ENSG00000111269 | 413.9931749 | 0.001012391 | 0.0970389 | 0.9896242 | 0.9955163 | CREBL2 | 1389 |
| ENSG00000183150 | 15.22019077 | 0.025431695 | 0.1373215 | 0.348297 | NA | GPR19 | 2842 |
| ENSG00000111276 | 576.9909373 | -0.055944902 | 0.0946413 | 0.4240032 | 0.6759631 | CDKN1B | 1027 |
| ENSG00000178878 | 74.4026621 | 0.116163105 | 0.1875922 | 0.0697201 | 0.2400946 | APOLD1 | 81575 |
| ENSG00000213782 | 48.44108512 | 0.024421887 | 0.1318629 | 0.5713837 | 0.7864184 | DDX47 | 51202 |
| ENSG00000234498 | 34.66780408 | -0.029043033 | 0.1352053 | 0.4403912 | NA | RPL13AP20 | 387841 |
| ENSG00000013588 | 5205.527542 | 0.344955993 | 0.0733466 | 3.49E-07 | 1.37E-05 | GPRC5A | 9052 |
| ENSG00000247498 | 89.96239781 | 0.029154311 | 0.1259211 | 0.5989656 | 0.8061432 | GPRC5D-AS | 100506314 |
| ENSG00000013583 | 329.6494599 | 0.253781829 | 0.1611767 | 0.0150855 | 0.0844742 | HEBP1 | 50865 |
| ENSG00000183935 | 20.4558552 | 0.009824137 | 0.1334481 | 0.751941 | NA | HTR7P1 | 93164 |
| ENSG00000084444 | 330.1072795 | 0.096224139 | 0.1196206 | 0.2089418 | 0.4654027 | FAM234B | 57613 |
| ENSG00000134531 | 517.6481536 | 0.890130928 | 0.3514166 | 0.0004224 | 0.0056395 | EMP1 | 2012 |
| ENSG00000171681 | 1067.269195 | 0.060147262 | 0.0796238 | 0.3535968 | 0.6187516 | ATF7IP | 55729 |
| ENSG00000121316 | 14.53431214 | -0.018066205 | 0.1357357 | 0.4908853 | NA | PLBD1 | 79887 |
| ENSG00000261324 | 106.3854235 | -0.174203844 | 0.2338436 | 0.0450726 | 0.1808632 | NA | NA |
| ENSG00000197837 | 65.56653567 | -0.069452523 | 0.148187 | 0.1900024 | 0.4408856 | H4C16 | 121504 |
| ENSG00000246705 | 1158.158272 | -0.083517972 | 0.0852746 | 0.2192777 | 0.4793377 | H2AJ | 55766 |
| ENSG00000084463 | 2311.120683 | -0.044156398 | 0.0648143 | 0.4382054 | 0.6881889 | WBP11 | 51729 |
| ENSG00000182993 | 16.64293749 | -0.002589051 | 0.133796 | 0.9226184 | NA | C12orf60 | 144608 |
| ENSG00000111348 | 60.69806786 | -0.009319765 | 0.1271483 | 0.8426943 | 0.9350382 | ARHGDIB | 397 |
| ENSG00000151490 | 22.37277076 | 0.015749043 | 0.133971 | 0.6225303 | NA | PTPRO | 5800 |
| ENSG00000151491 | 594.6479322 | 0.163573122 | 0.1123898 | 0.0458111 | 0.182938 | EPS8 | 2059 |
| ENSG00000023734 | 3480.273278 | -0.105608609 | 0.0649035 | 0.0661414 | 0.2323603 | STRAP | 11171 |
| ENSG00000023697 | 346.65095 | -0.012031388 | 0.1032346 | 0.8580447 | 0.9428541 | DERA | 51071 |
| ENSG00000008394 | 225.0249762 | -0.028399879 | 0.1124332 | 0.6643651 | 0.8443847 | MGST1 | 4257 |

| | | | | | | | | |
|---|---|---|---|---|---|---|---|---|
| ENSG00000052126 | 334.4445904 | -0.317116368 | 0.1629085 | 0.0050858 | 0.0385011 | PLEKHA5 | | 54477 |
| ENSG00000139154 | 820.5590971 | -0.096911619 | 0.0937225 | 0.1762805 | 0.4252072 | AEBP2 | | 121536 |
| ENSG00000256663 | 60.10454783 | -0.019565799 | 0.1283231 | 0.6821832 | 0.8543094 | NA | NA | |
| ENSG00000121350 | 163.2952167 | -0.267406338 | 0.2478056 | 0.0211923 | 0.1080247 | PYROXD1 | | 79912 |
| ENSG00000004700 | 2375.214299 | 0.159676129 | 0.0700363 | 0.0091458 | 0.0600181 | RECQL | | 5965 |
| ENSG00000111711 | 1146.393005 | 0.106147843 | 0.0877223 | 0.1298457 | 0.3543757 | GOLT1B | | 51026 |
| ENSG00000111716 | 12661.48293 | -0.200917001 | 0.0586873 | 0.0001891 | 0.0029299 | LDHB | | 3945 |
| ENSG00000111726 | 755.2627768 | 0.037013622 | 0.0851988 | 0.581316 | 0.7932654 | CMAS | | 55907 |
| ENSG00000111731 | 643.7224722 | -0.188394056 | 0.1132581 | 0.024664 | 0.1197422 | C2CD5 | | 9847 |
| ENSG00000256973 | 10.79125191 | -0.000834046 | 0.1346551 | 0.9687065 | NA | NA | NA | |
| ENSG00000139163 | 1160.727022 | -0.014932125 | 0.0772298 | 0.81404 | 0.9240962 | ETNK1 | | 55500 |
| ENSG00000060982 | 1876.33548 | 0.144185048 | 0.0741878 | 0.0233566 | 0.1153771 | BCAT1 | | 586 |
| ENSG00000256482 | 11.21333027 | 0.006392906 | 0.135168 | 0.7675226 | NA | NA | NA | |
| ENSG00000205707 | 77.89190518 | 0.012774403 | 0.1252619 | 0.8053337 | 0.9186624 | ETFRF1 | | 144363 |
| ENSG00000133703 | 1169.200207 | -0.259410861 | 0.0918673 | 0.0008435 | 0.0096883 | KRAS | | 3845 |
| ENSG00000246695 | 126.0647883 | -0.029968114 | 0.1221799 | 0.6140735 | 0.8154834 | NA | NA | |
| ENSG00000123094 | 915.0883521 | 0.010249279 | 0.0827192 | 0.8772089 | 0.950252 | RASSF8 | | 11228 |
| ENSG00000123095 | 50.65448114 | 1.414134426 | 0.4265854 | 3.81E-05 | 0.0007868 | BHLHE41 | | 79365 |
| ENSG00000256185 | 10.5592413 | -0.039074546 | 0.1431378 | 0.0856188 | NA | NA | NA | |
| ENSG00000123104 | 742.3101658 | -0.135153579 | 0.1009566 | 0.0753796 | 0.2521581 | ITPR2 | | 3709 |
| ENSG00000064102 | 1117.798113 | -0.030637297 | 0.0780253 | 0.6323684 | 0.8251856 | INTS13 | | 55726 |
| ENSG00000111790 | 951.7795058 | -0.076254233 | 0.0842572 | 0.2580029 | 0.5247892 | FGFR1OP2 | | 26127 |
| ENSG00000064115 | 907.4768244 | -0.215722445 | 0.0994451 | 0.0068693 | 0.0480881 | TM7SF3 | | 51768 |
| ENSG00000152944 | 522.9059762 | -0.121256798 | 0.1105352 | 0.1194672 | 0.3374295 | MED21 | | 9412 |
| ENSG00000211455 | 652.7499248 | 0.048403256 | 0.0915916 | 0.486424 | 0.7239115 | STK38L | | 23012 |
| ENSG00000029153 | 1008.140876 | 0.054165921 | 0.0811457 | 0.406514 | 0.6627341 | BMAL2 | | 56938 |
| ENSG00000110841 | 1134.361405 | 0.026963003 | 0.0767168 | 0.6574505 | 0.8419413 | PPFIBP1 | | 8496 |
| ENSG00000174236 | 8.523033258 | -0.013906029 | 0.1361029 | 0.4988977 | NA | REP15 | | 387849 |
| ENSG00000061794 | 1211.384969 | -0.06981502 | 0.0781432 | 0.2794159 | 0.5478109 | MRPS35 | | 60488 |
| ENSG00000087448 | 727.0041734 | 0.003474866 | 0.0854427 | 0.959321 | 0.9832262 | KLHL42 | | 57542 |
| ENSG00000123106 | 341.56429 | 0.042634214 | 0.1052945 | 0.5420215 | 0.7652256 | CCDC91 | | 55297 |
| ENSG00000087502 | 750.4172113 | -0.09169003 | 0.0931464 | 0.1981205 | 0.4513782 | ERGIC2 | | 51290 |
| ENSG00000133704 | 1652.785706 | -0.261846989 | 0.08054 | 0.0002138 | 0.0032348 | IPO8 | | 10526 |
| ENSG00000110888 | 1215.724433 | 0.250468312 | 0.1005349 | 0.0023355 | 0.0213132 | CAPRIN2 | | 65981 |
| ENSG00000235884 | 243.078242 | 0.511821774 | 0.1941763 | 0.0004982 | 0.0064237 | NA | NA | |
| ENSG00000245614 | 66.74622401 | 0.076193111 | 0.1515784 | 0.1714735 | 0.4177334 | DDX11-AS1 | | 100506660 |
| ENSG00000013573 | 1581.663638 | 0.084070886 | 0.0749171 | 0.1829398 | 0.4326625 | DDX11 | | 1663 |
| ENSG00000139146 | 2196.506335 | 0.085409549 | 0.0693446 | 0.1563198 | 0.395232 | SINHCAF | | 58516 |
| ENSG00000170456 | 449.1497171 | -0.048023579 | 0.0987796 | 0.4946482 | 0.7301889 | DENND5B | | 160518 |
| ENSG00000233381 | 8.925674925 | 0.00275901 | 0.1351919 | 0.8932093 | NA | NA | NA | |
| ENSG00000139160 | 22.01011529 | -0.014646644 | 0.1338232 | 0.6428186 | NA | ETFBKMT | | 254013 |
| ENSG00000151743 | 569.2358957 | -0.075593644 | 0.0983032 | 0.2915691 | 0.5613232 | AMN1 | | 196394 |
| ENSG00000223722 | 16.16330891 | -0.048447314 | 0.1466998 | 0.0780263 | NA | NA | NA | |
| ENSG00000174718 | 2067.401013 | -0.370780008 | 0.0882915 | 2.97E-06 | 8.79E-05 | RESF1 | | 55196 |
| ENSG00000151746 | 714.6931587 | 0.048301963 | 0.0915583 | 0.4850789 | 0.7233764 | BICD1 | | 636 |
| ENSG00000139132 | 173.3716056 | -0.092493332 | 0.1390563 | 0.2027088 | 0.4569858 | FGD4 | | 121512 |
| ENSG00000087470 | 2402.530525 | -0.263997799 | 0.0761862 | 9.95E-05 | 0.0017317 | DNM1L | | 10059 |
| ENSG00000257511 | 15.40252087 | -0.003282824 | 0.1338639 | 0.9016648 | NA | LOC100420¦ | | 100420981 |
| ENSG00000139131 | 926.6042367 | -0.118752024 | 0.0929423 | 0.1003785 | 0.3025862 | YARS2 | | 51067 |
| ENSG00000057294 | 182.8191886 | 0.141123279 | 0.1662083 | 0.0916572 | 0.2866917 | PKP2 | | 5318 |
| ENSG00000139133 | 116.7786391 | 0.032894511 | 0.1242519 | 0.5728746 | 0.7872645 | ALG10 | | 84920 |
| ENSG00000175548 | 281.9056419 | -0.079107791 | 0.1184805 | 0.2813283 | 0.5504516 | ALG10B | | 144245 |
| ENSG00000139117 | 1343.893476 | -0.140923361 | 0.0825736 | 0.0388462 | 0.1645453 | CPNE8 | | 144402 |
| ENSG00000257718 | 25.49437172 | -0.012234095 | 0.1330445 | 0.7110044 | NA | CPNE8-AS1 | | 107984504 |
| ENSG00000139116 | 1333.318947 | 0.103539915 | 0.0817435 | 0.1221627 | 0.3423225 | KIF21A | | 55605 |
| ENSG00000151229 | 337.8213159 | 0.033812932 | 0.1071926 | 0.6193794 | 0.8179841 | SLC2A13 | | 114134 |
| ENSG00000151233 | 1080.764709 | -0.166711259 | 0.0931302 | 0.024985 | 0.1206988 | GXYLT1 | | 283464 |
| ENSG00000015153 | 434.4913149 | -0.06556814 | 0.107833 | 0.3754282 | 0.6371317 | YAF2 | | 10138 |
| ENSG00000134283 | 1528.659112 | -0.115379581 | 0.0806633 | 0.0830641 | 0.2690716 | PPHLN1 | | 51535 |
| ENSG00000139168 | 1126.515245 | -0.164585001 | 0.0934653 | 0.0270189 | 0.12788 | ZCRB1 | | 85437 |
| ENSG00000139174 | 118.577893 | 0.314306545 | 0.3184913 | 0.0159496 | 0.0880899 | PRICKLE1 | | 144165 |
| ENSG00000257742 | 196.3219373 | -0.069845785 | 0.1258619 | 0.3159972 | 0.5849704 | NA | NA | |
| ENSG00000129317 | 517.3822832 | -0.017993652 | 0.0934004 | 0.7910501 | 0.9124377 | PUS7L | | 83448 |
| ENSG00000198001 | 399.2725713 | 0.026846718 | 0.0987693 | 0.6975368 | 0.8620332 | IRAK4 | | 51135 |
| ENSG00000151239 | 4057.068798 | -0.191787092 | 0.0634183 | 0.0007907 | 0.0092546 | TWF1 | | 5756 |
| ENSG00000139173 | 20.7179591 | -0.026193638 | 0.1365478 | 0.3968761 | NA | TMEM117 | | 84216 |
| ENSG00000184613 | 92.46596306 | -0.060063115 | 0.1377827 | 0.2959922 | 0.5660584 | NELL2 | | 4753 |
| ENSG00000134297 | 211.9146566 | 0.035269335 | 0.1160084 | 0.5879504 | 0.7979376 | PLEKHA8P1 | | 51054 |
| ENSG00000177119 | 7720.693196 | -0.28512919 | 0.071912 | 1.24E-05 | 0.0003039 | ANO6 | | 196527 |
| ENSG00000273015 | 164.970886 | 0.012197426 | 0.1153663 | 0.8464085 | 0.9372292 | NA | NA | |

| | | | | | | | | |
|---|---|---|---|---|---|---|---|---|
| ENSG00000189079 | 1278.924498 | -0.287951755 | 0.0898803 | 0.0002111 | 0.0031983 | ARID2 | | 196528 |
| ENSG00000139218 | 2971.153004 | -0.067073719 | 0.0623465 | 0.2268698 | 0.4881315 | SCAF11 | | 9169 |
| ENSG00000111371 | 11854.30724 | -0.047365663 | 0.0515576 | 0.3272383 | 0.5953077 | SLC38A1 | | 81539 |
| ENSG00000134294 | 23238.80224 | -0.097515577 | 0.1208066 | 0.1645432 | 0.4081607 | SLC38A2 | | 54407 |
| ENSG00000257261 | 27.40011746 | 0.037766773 | 0.1389422 | 0.2969911 | NA | NA | NA | |
| ENSG00000139211 | 260.7017622 | 0.044973029 | 0.112851 | 0.5138047 | 0.7440804 | AMIGO2 | | 347902 |
| ENSG00000005175 | 1596.169698 | -0.093109071 | 0.0756577 | 0.1431773 | 0.3745698 | RPAP3 | | 79657 |
| ENSG00000257433 | 27.21751843 | 0.036588677 | 0.1387352 | 0.301479 | NA | NA | NA | |
| ENSG00000079337 | 3501.416038 | -0.289143579 | 0.0735446 | 1.39E-05 | 0.000333 | RAPGEF3 | | 10411 |
| ENSG00000211584 | 4803.384648 | -0.344984298 | 0.0779973 | 1.22E-06 | 4.01E-05 | SLC48A1 | | 55652 |
| ENSG00000061273 | 2407.395644 | -0.433622921 | 0.0744983 | 5.73E-10 | 4.26E-08 | HDAC7 | | 51564 |
| ENSG00000268069 | 69.34807073 | 0.039446026 | 0.1332624 | 0.429107 | 0.6801084 | NA | NA | |
| ENSG00000111424 | 159.144091 | 0.813036661 | 0.2337066 | 2.48E-05 | 0.0005421 | VDR | | 7421 |
| ENSG00000134291 | 2335.232848 | 0.103791505 | 0.0687407 | 0.0819789 | 0.2665171 | TMEM106C | | 79022 |
| ENSG00000079387 | 931.2789554 | 0.028923013 | 0.081021 | 0.6590208 | 0.842576 | SENP1 | | 29843 |
| ENSG00000240399 | 11.79860805 | 0.017744268 | 0.1362412 | 0.4531943 | NA | NA | NA | |
| ENSG00000152556 | 1115.866989 | 0.011200763 | 0.0788139 | 0.8627008 | 0.9450945 | PFKM | | 5213 |
| ENSG00000177981 | 300.1967034 | -0.55208376 | 0.1671986 | 5.94E-05 | 0.0011435 | ASB8 | | 140461 |
| ENSG00000177875 | 33.23419835 | 0.035406355 | 0.1373002 | 0.354416 | NA | CCDC184 | | 387856 |
| ENSG00000167528 | 377.7415701 | 0.034346751 | 0.1024789 | 0.6215632 | 0.818389 | ZNF641 | | 121274 |
| ENSG00000139620 | 974.82531 | -0.071570975 | 0.0831861 | 0.2844339 | 0.5536531 | KANSL2 | | 54934 |
| ENSG00000221491 | 14.08919959 | -0.00247213 | 0.1342005 | 0.9203617 | NA | SNORA2C | | 677815 |
| ENSG00000207313 | 8.937878575 | -0.004604354 | 0.135196 | 0.8193092 | NA | SNORA2B | | 677794 |
| ENSG00000129315 | 2208.183469 | 0.387755349 | 0.0789474 | 9.93E-08 | 4.77E-06 | CCNT1 | | 904 |
| ENSG00000174233 | 1111.541012 | -0.054792497 | 0.0813144 | 0.4048312 | 0.6614808 | ADCY6 | | 112 |
| ENSG00000257653 | 12.47002417 | 0.011093015 | 0.1351086 | 0.6487337 | NA | NA | NA | |
| ENSG00000167535 | 439.2677665 | 0.066474582 | 0.1049662 | 0.3471316 | 0.6134112 | CACNB3 | | 784 |
| ENSG00000174243 | 4131.608985 | -0.155353113 | 0.0613175 | 0.0048408 | 0.0371766 | DDX23 | | 9416 |
| ENSG00000172602 | 29.97836626 | 0.019705565 | 0.1335461 | 0.5829023 | NA | RND1 | | 27289 |
| ENSG00000134285 | 405.2660086 | -0.070398177 | 0.1110813 | 0.331928 | 0.5992396 | FKBP11 | | 51303 |
| ENSG00000201678 | 9.21132624 | -0.039127549 | 0.1434823 | 0.0629219 | NA | NA | NA | |
| ENSG00000134287 | 3762.59878 | -0.114142 | 0.0644802 | 0.0451194 | 0.1809269 | ARF3 | | 377 |
| ENSG00000169884 | 31.38531688 | 0.091328067 | 0.1776715 | 0.0281169 | NA | WNT10B | | 7480 |
| ENSG00000181418 | 69.75860674 | 0.067230085 | 0.1457451 | 0.2150554 | 0.4731705 | DDN | | 23109 |
| ENSG00000181929 | 1386.550644 | -0.035506849 | 0.0729632 | 0.5648015 | 0.7820587 | PRKAG1 | | 5571 |
| ENSG00000167548 | 6281.205956 | -0.037587846 | 0.0591965 | 0.4717702 | 0.712259 | KMT2D | | 8085 |
| ENSG00000167550 | 169.6520098 | 0.335735037 | 0.2534627 | 0.0114632 | 0.070032 | RHEBL1 | | 121268 |
| ENSG00000139636 | 248.7854731 | -0.078308417 | 0.1224182 | 0.2823623 | 0.5516258 | LMBR1L | | 55716 |
| ENSG00000258017 | 1133.797651 | -0.117421124 | 0.1715714 | 0.1066572 | 0.3150095 | NA | NA | |
| ENSG00000123416 | 37213.55755 | -0.284604348 | 0.058246 | 1.83E-07 | 7.95E-06 | TUBA1B | | 10376 |
| ENSG00000167552 | 708.2012448 | 0.012713827 | 0.0872475 | 0.8508313 | 0.9389287 | TUBA1A | | 7846 |
| ENSG00000167553 | 14830.32821 | -0.002901991 | 0.0536462 | 0.941135 | 0.976977 | TUBA1C | | 84790 |
| ENSG00000258232 | 120.1272702 | -0.072529002 | 0.1404196 | 0.249477 | 0.5155947 | NA | NA | |
| ENSG00000135451 | 1405.02294 | -0.228787737 | 0.0872846 | 0.0019416 | 0.0185141 | TROAP | | 10024 |
| ENSG00000186897 | 104.7460563 | 0.017876972 | 0.1218667 | 0.7552665 | 0.8945557 | C1QL4 | | 338761 |
| ENSG00000178401 | 21.98272251 | 0.065009792 | 0.1555599 | 0.0568587 | NA | DNAJC22 | | 79962 |
| ENSG00000123352 | 1114.62277 | 0.032988605 | 0.0802276 | 0.6157952 | 0.8158539 | SPATS2 | | 65244 |
| ENSG00000135519 | 12.21390364 | -0.007709136 | 0.1347461 | 0.7478808 | NA | KCNH3 | | 23416 |
| ENSG00000187778 | 1412.168687 | -0.237686009 | 0.0863291 | 0.0012381 | 0.0132799 | MCRS1 | | 10445 |
| ENSG00000110844 | 361.8298443 | -0.177200087 | 0.144108 | 0.0500414 | 0.1926611 | PRPF40B | | 25766 |
| ENSG00000161791 | 1001.107828 | -0.005899255 | 0.0786051 | 0.9260884 | 0.9716733 | FMNL3 | | 91010 |
| ENSG00000139644 | 11961.00783 | -0.031348701 | 0.049276 | 0.5119576 | 0.7423258 | TMBIM6 | | 7009 |
| ENSG00000167566 | 594.4615902 | -0.011019637 | 0.0922318 | 0.8705668 | 0.9478556 | NCKAP5L | | 57701 |
| ENSG00000258057 | 12.73080066 | -0.019932981 | 0.1364296 | 0.4221258 | NA | BCDIN3D-A! | | 100286844 |
| ENSG00000186666 | 87.41463588 | 0.076577811 | 0.1493328 | 0.1879126 | 0.4389135 | BCDIN3D | | 144233 |
| ENSG00000161800 | 4447.81041 | -0.078724037 | 0.0574015 | 0.1294349 | 0.353901 | RACGAP1 | | 29127 |
| ENSG00000110881 | 403.3996162 | -0.072437089 | 0.1082458 | 0.3200995 | 0.5888319 | ASIC1 | | 41 |
| ENSG00000066117 | 2342.131798 | -0.248963353 | 0.0755067 | 0.0002019 | 0.0030905 | SMARCD1 | | 6602 |
| ENSG00000178449 | 634.8708825 | -0.067808816 | 0.097596 | 0.3409154 | 0.6081036 | COX14 | | 84987 |
| ENSG00000139624 | 518.7254079 | -0.000520539 | 0.0957042 | 0.9924411 | 0.9969601 | CERS5 | | 91012 |
| ENSG00000050405 | 170.0571993 | -0.040186622 | 0.1188527 | 0.5342576 | 0.7590201 | LIMA1 | | 51474 |
| ENSG00000161813 | 2677.308108 | 0.106103846 | 0.0656941 | 0.0662478 | 0.232594 | LARP4 | | 113251 |
| ENSG00000066084 | 2009.720171 | 0.103443104 | 0.0763245 | 0.1065364 | 0.3148121 | DIP2B | | 57609 |
| ENSG00000123268 | 764.3336146 | -0.002455617 | 0.0834343 | 0.9694899 | 0.9880997 | ATF1 | | 466 |
| ENSG00000110911 | 992.1951774 | -0.047500414 | 0.0857444 | 0.478962 | 0.7180127 | SLC11A2 | | 4891 |
| ENSG00000050426 | 963.1065719 | 0.151012205 | 0.0949895 | 0.0431706 | 0.1758285 | LETMD1 | | 25875 |
| ENSG00000110925 | 682.6933058 | 0.075393489 | 0.0947496 | 0.2899415 | 0.5599126 | CSRNP2 | | 81566 |
| ENSG00000135457 | 983.9750483 | -0.106763901 | 0.0871087 | 0.1240278 | 0.3453836 | TFCP2 | | 7024 |
| ENSG00000184271 | 26.00376293 | 0.007519408 | 0.1328818 | 0.8174501 | NA | POU6F1 | | 5463 |
| ENSG00000183283 | 3487.66685 | -0.231689632 | 0.0656944 | 9.95E-05 | 0.0017317 | DAZAP2 | | 9802 |

| | | | | | | | | |
|---|---|---|---|---|---|---|---|---|
| ENSG00000170545 | 160.0794816 | 0.074713311 | 0.1351211 | 0.2682067 | 0.5361059 | SMAGP | | 57228 |
| ENSG00000139629 | 25.54908073 | 0.028429866 | 0.1362363 | 0.4080649 | NA | GALNT6 | | 11226 |
| ENSG00000135503 | 721.9798452 | 0.122382158 | 0.0985946 | 0.1015015 | 0.3050059 | ACVR1B | | 91 |
| ENSG00000161835 | 11.72716286 | -0.012913179 | 0.1355911 | 0.5693365 | NA | TAMALIN | | 160622 |
| ENSG00000123358 | 131.2379602 | 0.512751309 | 0.2740776 | 0.0027996 | 0.0244407 | NR4A1 | | 3164 |
| ENSG00000123395 | 1812.585405 | 0.372731319 | 0.0830045 | 8.10E-07 | 2.83E-05 | ATG101 | | 60673 |
| ENSG00000257663 | 23.52023781 | 0.025235264 | 0.1357232 | 0.4452186 | NA | NA | NA | |
| ENSG00000167767 | 2286.051292 | -0.599940403 | 0.1625607 | 1.33E-05 | 0.000321 | KRT80 | | 144501 |
| ENSG00000135480 | 10986.49229 | -0.017202132 | 0.1091805 | 0.7663658 | 0.8994294 | KRT7 | | 3855 |
| ENSG00000257671 | 54.8459899 | 0.008588977 | 0.1285134 | 0.8489377 | 0.9377876 | KRT7-AS | | 109729127 |
| ENSG00000170442 | 39.75124894 | -0.050424071 | 0.1422084 | 0.2384811 | NA | KRT86 | | 3892 |
| ENSG00000257829 | 13.31792769 | 0.025971161 | 0.137907 | 0.3014133 | NA | NA | NA | |
| ENSG00000170421 | 33639.78614 | 0.172407813 | 0.0522948 | 0.0003847 | 0.0052865 | KRT8 | | 3856 |
| ENSG00000111057 | 18979.40596 | 0.081280786 | 0.1025295 | 0.2741402 | 0.5425779 | KRT18 | | 3875 |
| ENSG00000225410 | 67.03415884 | 0.016803282 | 0.128589 | 0.705824 | 0.8670449 | NA | NA | |
| ENSG00000063046 | 8861.920698 | -0.067053131 | 0.0524485 | 0.1730536 | 0.4203584 | EIF4B | | 1975 |
| ENSG00000257337 | 133.9112966 | 0.108976482 | 0.1595734 | 0.1337818 | 0.3604596 | TNS2-AS1 | | 283335 |
| ENSG00000111077 | 32.04508583 | -0.03309633 | 0.1364067 | 0.3848271 | NA | TNS2 | | 23371 |
| ENSG00000167778 | 518.7160676 | -0.048217756 | 0.0960726 | 0.4895945 | 0.7264931 | SPRYD3 | | 84926 |
| ENSG00000167779 | 113.4614519 | 0.681956492 | 0.2894374 | 0.0007868 | 0.0092273 | IGFBP6 | | 3489 |
| ENSG00000139631 | 107.3653264 | 0.042691415 | 0.1276303 | 0.4662769 | 0.7094916 | CSAD | | 51380 |
| ENSG00000139651 | 839.6676328 | -0.034319475 | 0.0859936 | 0.6090269 | 0.8123386 | ZNF740 | | 283337 |
| ENSG00000172819 | 175.9242133 | 0.061197171 | 0.1258664 | 0.3777114 | 0.6393344 | RARG | | 5916 |
| ENSG00000182544 | 551.7658643 | 0.058302808 | 0.0968596 | 0.4082799 | 0.6640358 | MFSD5 | | 84975 |
| ENSG00000135476 | 2792.081662 | 0.029200446 | 0.0612857 | 0.5945647 | 0.8031487 | ESPL1 | | 9700 |
| ENSG00000123349 | 2087.770455 | -0.141575718 | 0.0782113 | 0.0314661 | 0.1423529 | PFDN5 | | 5204 |
| ENSG00000257605 | 17.56054918 | 0.028643252 | 0.1377332 | 0.3285216 | NA | NA | NA | |
| ENSG00000139637 | 706.9107662 | 0.050101331 | 0.0905732 | 0.4689352 | 0.7108642 | MYG1 | | 60314 |
| ENSG00000094914 | 495.8789204 | -0.084885957 | 0.1050565 | 0.250348 | 0.5163897 | AAAS | | 8086 |
| ENSG00000185591 | 2114.729444 | -0.023879941 | 0.065825 | 0.678181 | 0.8532595 | SP1 | | 6667 |
| ENSG00000205352 | 1271.65796 | 0.065652801 | 0.0823409 | 0.3251444 | 0.5939431 | PRR13 | | 54458 |
| ENSG00000197111 | 4110.831742 | -0.146142829 | 0.0609868 | 0.0076613 | 0.0522119 | PCBP2 | | 5094 |
| ENSG00000139625 | 370.6359983 | 0.010843565 | 0.1011954 | 0.8737294 | 0.9490453 | MAP3K12 | | 7786 |
| ENSG00000139546 | 759.8935073 | -0.107441133 | 0.0947018 | 0.1375265 | 0.3657666 | TARBP2 | | 6895 |
| ENSG00000267281 | 13.66020639 | -0.017913479 | 0.1359229 | 0.4776806 | NA | ATF7-NPFF | | 114108587 |
| ENSG00000170653 | 855.0525061 | -0.006767117 | 0.0853505 | 0.9182243 | 0.9680305 | ATF7 | | 11016 |
| ENSG00000257550 | 10.05405195 | -0.013532876 | 0.1357962 | 0.5387431 | NA | LOC100652[illegible] | | 100652999 |
| ENSG00000135390 | 1678.58744 | -0.225500415 | 0.0954921 | 0.0039404 | 0.0315044 | ATP5MC2 | | 517 |
| ENSG00000012822 | 605.6872081 | 0.039689863 | 0.0906062 | 0.5647848 | 0.7820587 | CALCOCO1 | | 57658 |
| ENSG00000123364 | 51.33746563 | -0.007455433 | 0.1283703 | 0.8654769 | 0.9457908 | HOXC13 | | 3229 |
| ENSG00000180818 | 67.05972082 | 0.08094111 | 0.1560374 | 0.1435027 | 0.3751142 | HOXC10 | | 3226 |
| ENSG00000197757 | 26.8044965 | 0.003190221 | 0.1320163 | 0.9280523 | NA | HOXC6 | | 3223 |
| ENSG00000250133 | 14.90704187 | 0.008864091 | 0.1344321 | 0.7381921 | NA | HOXC-AS2 | | 100874364 |
| ENSG00000180806 | 139.7442302 | 0.031830983 | 0.1202305 | 0.6081255 | 0.8121054 | HOXC9 | | 3225 |
| ENSG00000037965 | 59.97057082 | 0.065329849 | 0.1481613 | 0.1824909 | 0.4320381 | HOXC8 | | 3224 |
| ENSG00000123415 | 378.0724644 | -0.012384993 | 0.0988903 | 0.8477707 | 0.9375782 | SMUG1 | | 23583 |
| ENSG00000094916 | 4557.105899 | 0.071726259 | 0.057202 | 0.1663103 | 0.4112342 | CBX5 | | 23468 |
| ENSG00000135486 | 19955.37706 | -0.448980852 | 0.0619771 | 6.02E-14 | 9.13E-12 | HNRNPA1 | | 3178 |
| ENSG00000111481 | 1664.719937 | -0.058836257 | 0.0732443 | 0.3418693 | 0.6087837 | COPZ1 | | 22818 |
| ENSG00000161642 | 113.1899845 | -0.072410321 | 0.1394655 | 0.2540128 | 0.5211409 | ZNF385A | | 25946 |
| ENSG00000161638 | 1481.417875 | 0.119138666 | 0.0804092 | 0.0735754 | 0.2483198 | ITGA5 | | 3678 |
| ENSG00000179899 | 43.20215263 | -0.012869582 | 0.1302282 | 0.7557353 | 0.8946735 | NA | NA | |
| ENSG00000135424 | 8.238241834 | 0.013590302 | 0.1361955 | 0.4952687 | NA | ITGA7 | | 3679 |
| ENSG00000258311 | 16.48165287 | -0.001388181 | 0.1337483 | 0.9574769 | NA | NA | NA | |
| ENSG00000135441 | 87.3033069 | -0.048779772 | 0.133266 | 0.3777 | 0.6393344 | BLOC1S1 | | 2647 |
| ENSG00000135404 | 4047.219416 | -0.008580602 | 0.0606692 | 0.8725474 | 0.9484215 | CD63 | | 967 |
| ENSG00000258056 | 66.54470769 | -0.027260951 | 0.1290146 | 0.5834762 | 0.7949132 | CD63-AS1 | | 105369779 |
| ENSG00000135414 | 344.3551422 | 0.06468881 | 0.1098614 | 0.3699998 | 0.6324213 | GDF11 | | 10220 |
| ENSG00000205323 | 35.36551122 | 0.064749349 | 0.1517068 | 0.126169 | NA | SARNP | | 84324 |
| ENSG00000257390 | 9.546770286 | 0.007801068 | 0.1353214 | 0.712385 | NA | NA | NA | |
| ENSG00000123353 | 548.2691124 | -0.098450855 | 0.1101993 | 0.1935524 | 0.4454947 | ORMDL2 | | 29095 |
| ENSG00000135392 | 402.732756 | -0.065922813 | 0.1055005 | 0.3564625 | 0.6197178 | DNAJC14 | | 85406 |
| ENSG00000182796 | 97.21065251 | 0.044081491 | 0.130133 | 0.4386761 | 0.6882367 | TMEM198B | | 440104 |
| ENSG00000170473 | 284.2537849 | 0.011168911 | 0.1052494 | 0.8721704 | 0.9484107 | PYM1 | | 84305 |
| ENSG00000065357 | 154.2717448 | 0.083597435 | 0.1390426 | 0.2279496 | 0.4890685 | DGKA | | 1606 |
| ENSG00000185664 | 21.63877592 | 0.006789955 | 0.133046 | 0.8304225 | NA | PMEL | | 6490 |
| ENSG00000123374 | 2027.027103 | 0.093753243 | 0.0697752 | 0.1196235 | 0.3376193 | CDK2 | | 1017 |
| ENSG00000258554 | 29.98547969 | 0.00609225 | 0.131826 | 0.8654363 | NA | NA | NA | |
| ENSG00000111540 | 284.0750968 | -0.114211183 | 0.1398788 | 0.146795 | 0.3800339 | RAB5B | | 5869 |
| ENSG00000139531 | 312.1719159 | -0.318649655 | 0.1706315 | 0.0058257 | 0.0425844 | SUOX | | 6821 |

| | | | | | | | |
|---|---|---|---|---|---|---|---|
| ENSG00000123411 | 150.2459498 | -0.04257766 | 0.1221913 | 0.5000218 | 0.734688 | IKZF4 | 64375 |
| ENSG00000197728 | 5042.988987 | -0.129599161 | 0.0600635 | 0.0165466 | 0.0902339 | RPS26 | 6231 |
| ENSG00000065361 | 528.971795 | -0.081714158 | 0.1025777 | 0.2655656 | 0.5333686 | ERBB3 | 2065 |
| ENSG00000170515 | 4767.1797 | 0.001960224 | 0.0564186 | 0.9726957 | 0.9893868 | PA2G4 | 5036 |
| ENSG00000257553 | 75.5914911 | -0.058427288 | 0.1423594 | 0.2485898 | 0.5150369 | NA | NA |
| ENSG00000229117 | 13264.09206 | -0.140502939 | 0.0623511 | 0.0102496 | 0.0645075 | RPL41 | 6171 |
| ENSG00000135482 | 38.3574679 | 0.01768926 | 0.131707 | 0.6584679 | NA | ZC3H10 | 84872 |
| ENSG00000139641 | 3434.498232 | -0.033700021 | 0.059499 | 0.5293795 | 0.7554508 | ESYT1 | 23344 |
| ENSG00000257809 | 13.33475885 | -0.004688419 | 0.1345523 | 0.8442167 | NA | NA | NA |
| ENSG00000196465 | 464.090657 | -0.034877219 | 0.0970962 | 0.6145199 | 0.8157779 | MYL6B | 140465 |
| ENSG00000092841 | 7075.591196 | -0.174611499 | 0.0620801 | 0.0019547 | 0.0185939 | MYL6 | 4637 |
| ENSG00000258199 | 98.23888882 | 0.013921954 | 0.1228321 | 0.8021311 | 0.9173383 | NA | NA |
| ENSG00000139613 | 2361.995428 | -0.061786613 | 0.065353 | 0.2805913 | 0.5492856 | SMARCC2 | 6601 |
| ENSG00000181852 | 560.8427767 | 0.157521586 | 0.1178242 | 0.0575313 | 0.2122584 | RNF41 | 10193 |
| ENSG00000139579 | 1036.128882 | 0.000270929 | 0.0791341 | 0.9974832 | 0.998947 | NABP2 | 79035 |
| ENSG00000139645 | 2473.602692 | -0.222887231 | 0.072331 | 0.0005339 | 0.0067838 | ANKRD52 | 283373 |
| ENSG00000135469 | 258.1548183 | -0.035207799 | 0.1099341 | 0.6036898 | 0.8090122 | COQ10A | 93058 |
| ENSG00000062485 | 3610.132014 | 0.003390634 | 0.0599522 | 0.9543084 | 0.9819659 | CS | 1431 |
| ENSG00000257303 | 15.26291457 | 0.005783313 | 0.1343598 | 0.8201056 | NA | NA | NA |
| ENSG00000257727 | 228.6791148 | 0.028038416 | 0.1119055 | 0.673635 | 0.8501877 | CNPY2 | 10330 |
| ENSG00000135473 | 246.7432876 | 0.123812336 | 0.1420847 | 0.1268165 | 0.349786 | PAN2 | 9924 |
| ENSG00000170581 | 518.658035 | -0.128455752 | 0.1128682 | 0.1041747 | 0.3100292 | STAT2 | 6773 |
| ENSG00000111602 | 2604.050293 | 0.092198072 | 0.0671218 | 0.1143274 | 0.328805 | TIMELESS | 8914 |
| ENSG00000176422 | 262.4204924 | 0.054233688 | 0.1130864 | 0.4397661 | 0.6890554 | SPRYD4 | 283377 |
| ENSG00000135423 | 47.88511108 | 0.006944792 | 0.1288244 | 0.8753242 | 0.9494835 | GLS2 | 27165 |
| ENSG00000076067 | 1333.094268 | -0.012547693 | 0.0761547 | 0.842215 | 0.9350382 | RBMS2 | 5939 |
| ENSG00000076108 | 4424.592642 | -0.078292447 | 0.060528 | 0.1482824 | 0.3824434 | BAZ2A | 11176 |
| ENSG00000110955 | 13975.49677 | -0.056340781 | 0.0539092 | 0.2540531 | 0.5211409 | ATP5F1B | 506 |
| ENSG00000110958 | 6669.497172 | -0.055319133 | 0.0537732 | 0.2626234 | 0.5301194 | PTGES3 | 10728 |
| ENSG00000196531 | 7130.193981 | -0.184330545 | 0.0565348 | 0.0003897 | 0.0053301 | NACA | 4666 |
| ENSG00000198056 | 656.7178586 | 0.223109287 | 0.1224994 | 0.0132489 | 0.0774187 | PRIM1 | 5557 |
| ENSG00000025423 | 16.80080239 | -0.000598685 | 0.1336145 | 0.9762541 | NA | HSD17B6 | 8630 |
| ENSG00000166860 | 339.9842643 | -0.000546482 | 0.1007009 | 0.9924836 | 0.9969601 | ZBTB39 | 9880 |
| ENSG00000166881 | 1028.082032 | 0.27030563 | 0.0962712 | 0.0008338 | 0.0096055 | NEMP1 | 23306 |
| ENSG00000166886 | 254.7464101 | -0.057337707 | 0.1147436 | 0.4115989 | 0.6661689 | NAB2 | 4665 |
| ENSG00000166888 | 27.60819089 | 0.007852582 | 0.1321577 | 0.8230815 | NA | STAT6 | 6778 |
| ENSG00000123384 | 578.1273887 | 0.001490406 | 0.0920024 | 0.983476 | 0.9937702 | LRP1 | 4035 |
| ENSG00000182379 | 67.24376208 | 0.009804904 | 0.1262603 | 0.8412417 | 0.9347236 | NXPH4 | 11247 |
| ENSG00000182199 | 2773.400729 | -0.006742866 | 0.0611457 | 0.9011784 | 0.9612514 | SHMT2 | 6472 |
| ENSG00000185633 | 10.91810068 | 0.033158202 | 0.1406572 | 0.1518485 | NA | NDUFA4L2 | 56901 |
| ENSG00000185482 | 10.81302521 | -0.013266972 | 0.1356351 | 0.5595948 | NA | STAC3 | 246329 |
| ENSG00000179912 | 267.0292841 | 0.059514825 | 0.1141402 | 0.4008939 | 0.6587248 | R3HDM2 | 22864 |
| ENSG00000166986 | 5024.158961 | 0.003622646 | 0.0544153 | 0.9428437 | 0.9778352 | MARS1 | 4141 |
| ENSG00000175197 | 443.8049397 | 1.574083641 | 0.1481307 | 1.17E-27 | 6.83E-25 | DDIT3 | 1649 |
| ENSG00000166987 | 669.8999723 | -0.00274632 | 0.0862707 | 0.9663694 | 0.9865549 | MBD6 | 114785 |
| ENSG00000175203 | 2295.548495 | -0.057623469 | 0.0650437 | 0.3130654 | 0.5821263 | DCTN2 | 10540 |
| ENSG00000166908 | 1209.944536 | 0.239995401 | 0.0920755 | 0.0018667 | 0.0180202 | PIP4K2C | 79837 |
| ENSG00000178498 | 68.96207026 | 0.038315537 | 0.1317846 | 0.4586929 | 0.702964 | DTX3 | 196403 |
| ENSG00000240771 | 145.7809749 | -0.008022693 | 0.1165916 | 0.8941697 | 0.9574476 | ARHGEF25 | 115557 |
| ENSG00000135502 | 27.50474145 | -0.012877259 | 0.1332323 | 0.6938983 | NA | SLC26A10P | 65012 |
| ENSG00000135454 | 1103.889804 | -0.040821924 | 0.0780528 | 0.5246195 | 0.752004 | B4GALNT1 | 2583 |
| ENSG00000135506 | 3082.905394 | 0.011300936 | 0.0619732 | 0.8333824 | 0.9305394 | OS9 | 10956 |
| ENSG00000257342 | 19.14030092 | 0.012167077 | 0.134496 | 0.662713 | NA | NA | NA |
| ENSG00000135439 | 19.62319512 | -0.036535099 | 0.1403392 | 0.2230682 | NA | AGAP2 | 116986 |
| ENSG00000255737 | 285.6322114 | -0.006393295 | 0.1046131 | 0.9232561 | 0.9706196 | AGAP2-AS1 | 100130776 |
| ENSG00000135452 | 91.47851233 | 0.033302454 | 0.1267808 | 0.5503121 | 0.7715069 | TSPAN31 | 6302 |
| ENSG00000135446 | 2685.009493 | -0.001419248 | 0.0667661 | 0.9814235 | 0.9932057 | CDK4 | 1019 |
| ENSG00000139266 | 76.11133768 | 0.079164459 | 0.1544104 | 0.1561191 | 0.394981 | MARCHF9 | 92979 |
| ENSG00000111012 | 211.949845 | 0.658027516 | 0.2001845 | 5.44E-05 | 0.0010645 | CYP27B1 | 1594 |
| ENSG00000037897 | 375.7388223 | -0.000839903 | 0.1008231 | 0.9888229 | 0.9955163 | METTL1 | 4234 |
| ENSG00000123427 | 104.1860443 | 0.51857778 | 0.3238514 | 0.004271 | 0.0335383 | EEF1AKMT3 | 25895 |
| ENSG00000123297 | 376.7831236 | -0.051108332 | 0.1046751 | 0.4690663 | 0.7108642 | TSFM | 10102 |
| ENSG00000175215 | 1219.027834 | -0.211136684 | 0.0915987 | 0.0051965 | 0.0391807 | CTDSP2 | 10106 |
| ENSG00000257698 | 67.78888451 | 0.064990845 | 0.1444528 | 0.2271877 | 0.4882146 | GIHCG | 100506844 |
| ENSG00000166896 | 83.0693619 | -0.001962906 | 0.1238424 | 0.9685079 | 0.9877884 | ATP23 | 91419 |
| ENSG00000139263 | 239.8395559 | -0.040397083 | 0.1142369 | 0.5459117 | 0.768011 | LRIG3 | 121227 |
| ENSG00000118596 | 143.3465812 | 0.060919002 | 0.129725 | 0.3478686 | 0.6140002 | SLC16A7 | 9194 |
| ENSG00000135655 | 906.6699109 | 0.088852489 | 0.0876275 | 0.2002892 | 0.4540225 | USP15 | 9958 |
| ENSG00000061987 | 1037.916227 | 0.028375978 | 0.0785747 | 0.6601964 | 0.8426737 | MON2 | 23041 |
| ENSG00000257354 | 9.812852835 | 0.003288325 | 0.1349053 | 0.8827583 | NA | MIRLET7IHG | 120766144 |

| | | | | | | | | |
|---|---|---|---|---|---|---|---|---|
| ENSG00000111110 | 121.5161288 | 0.113210915 | 0.1653182 | 0.1194487 | 0.3374295 | PPM1H | | 57460 |
| ENSG00000177990 | 43.70437374 | 0.060091294 | 0.1471696 | 0.1781379 | 0.4271872 | DPY19L2 | | 283417 |
| ENSG00000118600 | 401.4824143 | 0.12661824 | 0.122512 | 0.1166789 | 0.3333153 | RXYLT1 | | 10329 |
| ENSG00000196935 | 1117.791295 | 0.395850305 | 0.093563 | 2.40E-06 | 7.23E-05 | SRGAP1 | | 57522 |
| ENSG00000174206 | 136.3512739 | 0.086981394 | 0.144438 | 0.2034955 | 0.4583554 | KICS2 | | 144577 |
| ENSG00000184575 | 3585.722642 | 0.004471979 | 0.0608244 | 0.9336871 | 0.9745688 | XPOT | | 11260 |
| ENSG00000183735 | 1004.443747 | -0.026628511 | 0.0787523 | 0.678788 | 0.8535466 | TBK1 | | 29110 |
| ENSG00000153179 | 1432.896167 | 0.031585325 | 0.0729573 | 0.6099693 | 0.8128053 | RASSF3 | | 283349 |
| ENSG00000135677 | 2643.260857 | -0.104456983 | 0.0655795 | 0.069871 | 0.24026 | GNS | | 2799 |
| ENSG00000174106 | 872.175351 | -0.004091537 | 0.0805777 | 0.9490355 | 0.9798833 | LEMD3 | | 23592 |
| ENSG00000174099 | 1100.781191 | -0.200502385 | 0.1015823 | 0.0120134 | 0.0724273 | MSRB3 | | 253827 |
| ENSG00000250280 | 10.34561752 | -0.02793617 | 0.1390017 | 0.2132085 | NA | NA | NA | |
| ENSG00000256268 | 30.38850512 | -0.014861295 | 0.1327919 | 0.6718465 | NA | NA | NA | |
| ENSG00000241749 | 13.29419031 | 1.740998421 | 1.0193107 | 0.002459 | NA | RPSAP52 | | 204010 |
| ENSG00000149948 | 1505.634205 | 0.145188509 | 0.0880621 | 0.041268 | 0.170698 | HMGA2 | | 8091 |
| ENSG00000139233 | 513.1881393 | 0.16582825 | 0.1300189 | 0.0556495 | 0.2073499 | LLPH | | 84298 |
| ENSG00000155957 | 118.2430499 | -0.086481998 | 0.1473077 | 0.1935138 | 0.4454937 | TMBIM4 | | 51643 |
| ENSG00000239335 | 16.03891017 | 0.072720771 | 0.1637095 | 0.0147297 | NA | LLPH-DT | | 103625681 |
| ENSG00000090376 | 25.40759672 | 0.029789944 | 0.1365839 | 0.3924236 | NA | IRAK3 | | 11213 |
| ENSG00000127311 | 104.3103235 | 0.063159561 | 0.1360443 | 0.2946055 | 0.5648862 | HELB | | 92797 |
| ENSG00000155974 | 90.14540525 | -0.067787472 | 0.1411484 | 0.2530651 | 0.5198972 | GRIP1 | | 23426 |
| ENSG00000111530 | 3181.016517 | -0.172107596 | 0.0652969 | 0.0031024 | 0.026241 | CAND1 | | 55832 |
| ENSG00000127334 | 390.1009785 | -0.27376295 | 0.1584332 | 0.0100468 | 0.0637458 | DYRK2 | | 8445 |
| ENSG00000111554 | 282.1918753 | 0.072054259 | 0.1168868 | 0.3207177 | 0.5893195 | MDM1 | | 56890 |
| ENSG00000240087 | 17.35291741 | 0.002551784 | 0.1336837 | 0.9295111 | NA | RPSAP12 | | 387867 |
| ENSG00000127314 | 2194.966997 | -0.004970177 | 0.0652141 | 0.929962 | 0.972941 | RAP1B | | 5908 |
| ENSG00000111581 | 1579.659675 | -0.323699523 | 0.089835 | 4.12E-05 | 0.0008364 | NUP107 | | 57122 |
| ENSG00000175782 | 324.3202578 | -0.092772858 | 0.1194047 | 0.2214424 | 0.4809358 | SLC35E3 | | 55508 |
| ENSG00000135679 | 1998.145039 | -0.077657889 | 0.0687953 | 0.1917896 | 0.4430962 | MDM2 | | 4193 |
| ENSG00000257181 | 22.37728604 | -0.02710807 | 0.1364014 | 0.4026765 | NA | NA | NA | |
| ENSG00000111605 | 2123.480331 | -0.297074415 | 0.0747244 | 1.07E-05 | 0.0002694 | CPSF6 | | 11052 |
| ENSG00000127337 | 427.7079621 | -0.050792011 | 0.1035001 | 0.4712958 | 0.7119839 | YEATS4 | | 8089 |
| ENSG00000166225 | 515.1747507 | -0.125070247 | 0.1129674 | 0.1123449 | 0.3254406 | FRS2 | | 10818 |
| ENSG00000166226 | 6724.098702 | -0.134789696 | 0.0560964 | 0.0079015 | 0.0536514 | CCT2 | | 10576 |
| ENSG00000127325 | 12.42336743 | 0.012633068 | 0.135191 | 0.610517 | NA | BEST3 | | 144453 |
| ENSG00000127328 | 871.868647 | -0.08758673 | 0.0886119 | 0.2072535 | 0.4632201 | RAB3IP | | 117177 |
| ENSG00000257815 | 21.34643682 | -0.007362999 | 0.1334206 | 0.8042439 | NA | PRANCR | | 101928062 |
| ENSG00000111596 | 1006.230497 | -0.144606078 | 0.0912228 | 0.0462365 | 0.1839973 | CNOT2 | | 4848 |
| ENSG00000133858 | 810.7255218 | -0.131605995 | 0.1074945 | 0.0911207 | 0.2855098 | ZFC3H1 | | 196441 |
| ENSG00000173451 | 64.14996829 | 0.059208866 | 0.1431346 | 0.2403249 | 0.5055349 | THAP2 | | 83591 |
| ENSG00000139291 | 594.3504157 | -0.21095919 | 0.1224635 | 0.0176876 | 0.0950747 | TMEM19 | | 55266 |
| ENSG00000080371 | 1240.372137 | 0.217306596 | 0.0891004 | 0.0035308 | 0.0289021 | RAB21 | | 23011 |
| ENSG00000121749 | 952.9474975 | 0.039692288 | 0.0803941 | 0.5435297 | 0.7661361 | TBC1D15 | | 64786 |
| ENSG00000253719 | 918.2763631 | -0.262502741 | 0.1017185 | 0.0016602 | 0.0165753 | ATXN7L3B | | 552889 |
| ENSG00000180881 | 21.55959308 | -0.022083495 | 0.13582 | 0.4550343 | NA | CAPS2 | | 84698 |
| ENSG00000139278 | 37.56737369 | -0.092872334 | 0.1752596 | 0.0507451 | NA | GLIPR1 | | 11010 |
| ENSG00000257497 | 26.44852019 | 0.006010928 | 0.1323943 | 0.859824 | NA | NA | NA | |
| ENSG00000111615 | 1282.520836 | 0.001377456 | 0.0784839 | 0.9836986 | 0.9938231 | KRR1 | | 11103 |
| ENSG00000139289 | 2588.638266 | 0.5415878 | 0.1920541 | 0.0002796 | 0.0040673 | PHLDA1 | | 22822 |
| ENSG00000257453 | 15.89680194 | 0.045073453 | 0.1453201 | 0.0820148 | NA | NA | NA | |
| ENSG00000187109 | 7603.584215 | -0.150846312 | 0.0570097 | 0.003478 | 0.0285627 | NAP1L1 | | 4673 |
| ENSG00000179941 | 123.4859744 | 0.062129081 | 0.1329147 | 0.3256549 | 0.5942127 | BBS10 | | 79738 |
| ENSG00000091039 | 2381.032686 | -0.191054729 | 0.0716112 | 0.0023875 | 0.0216717 | OSBPL8 | | 114882 |
| ENSG00000186908 | 601.6994785 | -0.116546624 | 0.1066743 | 0.1284746 | 0.3524451 | ZDHHC17 | | 23390 |
| ENSG00000175183 | 284.9699133 | 0.187217482 | 0.1606009 | 0.0467332 | 0.1849224 | CSRP2 | | 1466 |
| ENSG00000165891 | 1678.717072 | 0.246306622 | 0.0810091 | 0.0004871 | 0.0062934 | E2F7 | | 144455 |
| ENSG00000067798 | 1525.124272 | 0.140260215 | 0.0853112 | 0.0439406 | 0.177715 | NAV3 | | 89795 |
| ENSG00000177425 | 1976.097765 | -0.03615722 | 0.0665471 | 0.5332309 | 0.7582993 | PAWR | | 5074 |
| ENSG00000058272 | 1785.320994 | -0.031915646 | 0.0681755 | 0.5874881 | 0.7977736 | PPP1R12A | | 4659 |
| ENSG00000257557 | 22.83931856 | 0.039129943 | 0.1408957 | 0.2210759 | NA | NA | NA | |
| ENSG00000111052 | 8.615019328 | -0.009111332 | 0.1355625 | 0.6505678 | NA | LIN7A | | 8825 |
| ENSG00000133773 | 704.238312 | 0.090134651 | 0.0957371 | 0.2113855 | 0.4681824 | CCDC59 | | 29080 |
| ENSG00000127720 | 34.45713373 | -0.005772062 | 0.1309466 | 0.8770616 | NA | METTL25 | | 84190 |
| ENSG00000179104 | 253.8495688 | -0.024918609 | 0.1085918 | 0.7097943 | 0.8682712 | TMTC2 | | 160335 |
| ENSG00000133640 | 14.20971587 | -0.038215982 | 0.1420465 | 0.1413202 | NA | LRRIQ1 | | 84125 |
| ENSG00000133641 | 677.3811952 | 0.053461498 | 0.0920965 | 0.442453 | 0.6915956 | RLIG1 | | 91298 |
| ENSG00000198707 | 576.8991719 | 0.18520623 | 0.1189795 | 0.030614 | 0.1401263 | CEP290 | | 80184 |
| ENSG00000139324 | 945.6943032 | -0.088042507 | 0.0872081 | 0.2014547 | 0.4555154 | TMTC3 | | 160418 |
| ENSG00000049130 | 744.672927 | 0.79563606 | 0.1136891 | 1.58E-13 | 2.30E-11 | KITLG | | 4254 |
| ENSG00000139318 | 741.0553842 | -0.210783481 | 0.1115913 | 0.0130242 | 0.076527 | DUSP6 | | 1848 |

| | | | | | | | | |
|---|---|---|---|---|---|---|---|---|
| ENSG00000139323 | 354.6725084 | -0.017410717 | 0.1003808 | 0.7982945 | 0.9162772 | POC1B | | 282809 |
| ENSG00000257594 | 24.35009255 | -0.064160445 | 0.1542571 | 0.0719733 | NA | GALNT4 | | 8693 |
| ENSG00000270344 | 8.367418596 | 0.022161368 | 0.1377999 | 0.2756275 | NA | NA | NA | |
| ENSG00000070961 | 1315.796592 | 0.056334357 | 0.0778612 | 0.3820956 | 0.6430282 | ATP2B1 | | 490 |
| ENSG00000133639 | 249.7193051 | 0.04242731 | 0.11213 | 0.5361944 | 0.7604767 | BTG1 | | 694 |
| ENSG00000102189 | 813.1717429 | 0.055600816 | 0.0860233 | 0.4083376 | 0.6640358 | EEA1 | | 8411 |
| ENSG00000256393 | 104.8533636 | -0.0578632 | 0.133533 | 0.3373168 | 0.6049743 | NA | NA | |
| ENSG00000173598 | 563.7865681 | -0.032422382 | 0.0915824 | 0.6352992 | 0.8266198 | NUDT4 | | 11163 |
| ENSG00000177889 | 1959.43707 | -0.302331626 | 0.0766031 | 1.21E-05 | 0.0002975 | UBE2N | | 7334 |
| ENSG00000198015 | 1226.791209 | -0.039690038 | 0.0752711 | 0.5277323 | 0.7546809 | MRPL42 | | 28977 |
| ENSG00000243015 | 11.17663556 | -0.015874453 | 0.13593 | 0.4966537 | NA | RN7SL737P | | 106481123 |
| ENSG00000246985 | 86.45241406 | 0.038762626 | 0.1303358 | 0.4714127 | 0.7119839 | SOCS2-AS1 | | 144481 |
| ENSG00000120833 | 1481.513875 | -0.151900066 | 0.0894524 | 0.0349073 | 0.152545 | SOCS2 | | 8835 |
| ENSG00000186076 | 231.5046485 | -0.129412229 | 0.1490925 | 0.1139745 | 0.3281578 | NA | NA | |
| ENSG00000169372 | 69.92850874 | 0.011682543 | 0.1259882 | 0.8167984 | 0.9252377 | CRADD | | 8738 |
| ENSG00000258365 | 8.782358899 | 0.009110977 | 0.1354897 | 0.6630047 | NA | NA | NA | |
| ENSG00000173588 | 624.0889582 | 0.080286096 | 0.0957462 | 0.2631383 | 0.5306834 | CEP83 | | 51134 |
| ENSG00000057704 | 20.55411944 | 0.043876436 | 0.1435434 | 0.1498375 | NA | TMCC3 | | 57458 |
| ENSG00000184752 | 900.2474669 | -0.01246082 | 0.0799665 | 0.8490873 | 0.9377876 | NDUFA12 | | 55967 |
| ENSG00000120798 | 646.5592445 | -0.028152601 | 0.0877116 | 0.6787641 | 0.8535466 | NR2C1 | | 7181 |
| ENSG00000180263 | 1347.848528 | 0.056882119 | 0.0754398 | 0.3683392 | 0.631153 | FGD6 | | 55785 |
| ENSG00000028203 | 1505.725143 | -0.085009752 | 0.0785852 | 0.1906275 | 0.4413717 | VEZT | | 55591 |
| ENSG00000265917 | 15.74482543 | 0.012679094 | 0.1348329 | 0.6341617 | NA | MIR3685 | | 100500802 |
| ENSG00000111142 | 2529.347103 | -0.261792464 | 0.0745447 | 8.53E-05 | 0.0015361 | METAP2 | | 10988 |
| ENSG00000074527 | 231.6463075 | 0.254756276 | 0.1939904 | 0.0204002 | 0.1051609 | NTN4 | | 59277 |
| ENSG00000139343 | 1186.381947 | -0.128295188 | 0.0842893 | 0.0614282 | 0.2212758 | SNRPF | | 6636 |
| ENSG00000084110 | 20.83482861 | -0.010705281 | 0.1335611 | 0.7257656 | NA | HAL | | 3034 |
| ENSG00000111144 | 886.444278 | -0.208521575 | 0.1029376 | 0.0100465 | 0.0637458 | LTA4H | | 4048 |
| ENSG00000111145 | 4000.180533 | 0.08450431 | 0.0623024 | 0.1273768 | 0.3505038 | ELK3 | | 2004 |
| ENSG00000258177 | 36.40405622 | -0.027883337 | 0.1346191 | 0.4666549 | NA | NA | NA | |
| ENSG00000059758 | 610.0891604 | -0.065691148 | 0.0959552 | 0.3529931 | 0.6185587 | CDK17 | | 5128 |
| ENSG00000139350 | 1072.236953 | 0.006347041 | 0.0791588 | 0.9224674 | 0.9701942 | NEDD1 | | 121441 |
| ENSG00000227825 | 9.61831046 | 0.019640284 | 0.1369476 | 0.3709142 | NA | SLC9A7P1 | | 121456 |
| ENSG00000257167 | 194.5894985 | -0.069549038 | 0.1252464 | 0.3188577 | 0.5875404 | TMPO-AS1 | | 100128191 |
| ENSG00000120802 | 7054.868104 | -0.229291216 | 0.0614276 | 4.67E-05 | 0.0009276 | TMPO | | 7112 |
| ENSG00000075415 | 7432.031998 | -0.126827829 | 0.0598529 | 0.0183041 | 0.0975414 | SLC25A3 | | 5250 |
| ENSG00000221625 | 14.33473264 | -0.000197683 | 0.1341876 | 0.9914542 | NA | NA | NA | |
| ENSG00000166130 | 1220.614892 | -0.232739989 | 0.0914359 | 0.0023112 | 0.0211573 | IKBIP | | 121457 |
| ENSG00000120868 | 775.9612787 | -0.469684168 | 0.1094509 | 1.45E-06 | 4.64E-05 | APAF1 | | 317 |
| ENSG00000111647 | 791.9354325 | -0.122449756 | 0.0960769 | 0.0962797 | 0.2949496 | BLTP3B | | 23074 |
| ENSG00000238105 | 192.8480006 | 0.011373622 | 0.1121035 | 0.8614567 | 0.9443096 | NA | NA | |
| ENSG00000257489 | 18.13543808 | -0.00023074 | 0.1334442 | 0.9915103 | NA | NA | NA | |
| ENSG00000075089 | 418.5023453 | -0.060144677 | 0.102529 | 0.4000121 | 0.6583091 | ACTR6 | | 64431 |
| ENSG00000166153 | 23.96302237 | 0.035248024 | 0.138756 | 0.2964349 | NA | DEPDC4 | | 120863 |
| ENSG00000136021 | 1094.128672 | -0.029004053 | 0.0780496 | 0.6504937 | 0.8376603 | SCYL2 | | 55681 |
| ENSG00000139354 | 656.7006949 | -0.292222628 | 0.1213346 | 0.0021174 | 0.0197079 | GAS2L3 | | 283431 |
| ENSG00000120800 | 3070.897132 | -0.002578218 | 0.0609574 | 0.962314 | 0.9847385 | UTP20 | | 27340 |
| ENSG00000120805 | 986.0652452 | -0.18231349 | 0.0994368 | 0.0194065 | 0.1014713 | ARL1 | | 400 |
| ENSG00000111666 | 754.1300304 | -0.034021313 | 0.0887011 | 0.6156511 | 0.8158539 | CHPT1 | | 56994 |
| ENSG00000111670 | 750.0428015 | -0.17716915 | 0.1087005 | 0.0296006 | 0.137154 | GNPTAB | | 79158 |
| ENSG00000136048 | 558.7813486 | 0.034272254 | 0.0915475 | 0.6180659 | 0.8171928 | DRAM1 | | 55332 |
| ENSG00000120860 | 192.759881 | 0.016776567 | 0.1123166 | 0.7969053 | 0.9157552 | WASHC3 | | 51019 |
| ENSG00000075188 | 515.0049724 | -0.186561673 | 0.1329338 | 0.0367655 | 0.158238 | NUP37 | | 79023 |
| ENSG00000185480 | 739.618138 | -0.156191441 | 0.1059097 | 0.0481654 | 0.1878532 | PARPBP | | 55010 |
| ENSG00000183395 | 11.85766703 | -0.010870521 | 0.1353589 | 0.6298232 | NA | PMCH | | 5367 |
| ENSG00000257681 | 15.45522394 | -0.004830312 | 0.1340421 | 0.8541502 | NA | NA | NA | |
| ENSG00000111696 | 316.5022055 | 0.139454638 | 0.1458479 | 0.0982926 | 0.298999 | NT5DC3 | | 51559 |
| ENSG00000166598 | 9354.383055 | -0.193771823 | 0.0672198 | 0.0012277 | 0.013181 | HSP90B1 | | 7184 |
| ENSG00000204954 | 114.6293033 | -0.015755869 | 0.1203774 | 0.7865974 | 0.911261 | UQCC6 | | 728568 |
| ENSG00000139372 | 729.6761056 | 0.071638266 | 0.0903741 | 0.3001727 | 0.5698549 | TDG | | 6996 |
| ENSG00000216285 | 8.125445959 | 0.008201616 | 0.1356734 | 0.6692855 | NA | NA | NA | |
| ENSG00000111727 | 192.9599983 | 0.043048082 | 0.1172529 | 0.5184383 | 0.7474909 | HCFC2 | | 29915 |
| ENSG00000120837 | 430.0671339 | 0.053112611 | 0.1013165 | 0.4538648 | 0.6991498 | NFYB | | 4801 |
| ENSG00000198431 | 10348.95304 | 0.741032585 | 0.0668487 | 9.81E-30 | 6.74E-27 | TXNRD1 | | 7296 |
| ENSG00000257732 | 11.24045533 | 0.037510644 | 0.1422742 | 0.1137165 | NA | LOC1249030 | | 124903002 |
| ENSG00000213442 | 148.8173726 | -0.007728797 | 0.1161015 | 0.8986445 | 0.9599512 | RPL18AP3 | | 390354 |
| ENSG00000255150 | 21.66219988 | 0.054702088 | 0.1485827 | 0.1027513 | NA | EID3 | | 493861 |
| ENSG00000171310 | 500.733209 | 0.226518206 | 0.1336189 | 0.0159818 | 0.0881829 | CHST11 | | 50515 |
| ENSG00000136052 | 275.6452138 | 0.111348127 | 0.1344857 | 0.158244 | 0.3982865 | SLC41A2 | | 84102 |
| ENSG00000151131 | 221.7823173 | 0.088287013 | 0.1301959 | 0.2293537 | 0.4903399 | NOPCHAP1 | | 121053 |

| | | | | | | | | |
|---|---|---|---|---|---|---|---|---|
| ENSG00000136010 | 188.3244267 | 1.122833057 | 0.2071683 | 3.13E-09 | 2.12E-07 | ALDH1L2 | | 160428 |
| ENSG00000136051 | 1526.295148 | -0.148259564 | 0.0816839 | 0.0291389 | 0.1357145 | WASHC4 | | 23325 |
| ENSG00000136044 | 414.317279 | -0.231354088 | 0.1425348 | 0.017039 | 0.09223 | APPL2 | | 55198 |
| ENSG00000235162 | 1540.147461 | -0.03567931 | 0.0721224 | 0.5606093 | 0.7795039 | C12orf75 | | 387882 |
| ENSG00000074590 | 12.48579684 | 0.013015922 | 0.1355019 | 0.5796996 | NA | NUAK1 | | 9891 |
| ENSG00000136026 | 387.554946 | 0.071381464 | 0.107974 | 0.3272142 | 0.5953077 | CKAP4 | | 10970 |
| ENSG00000166046 | 16.99250356 | -0.011916172 | 0.1343858 | 0.6685844 | NA | TCP11L2 | | 255394 |
| ENSG00000013503 | 361.8765148 | -0.197191928 | 0.1472877 | 0.0354273 | 0.1542648 | POLR3B | | 55703 |
| ENSG00000111785 | 390.7521239 | -0.114020066 | 0.1194641 | 0.1473834 | 0.3809661 | RIC8B | | 55188 |
| ENSG00000260329 | 10.37814361 | 0.006185304 | 0.1349914 | 0.783478 | NA | NA | NA | |
| ENSG00000151135 | 706.5130001 | -0.148197244 | 0.1084385 | 0.0619806 | 0.2223574 | TMEM263 | | 90488 |
| ENSG00000120832 | 189.8941749 | -0.189741719 | 0.201988 | 0.0461613 | 0.1839681 | MTERF2 | | 80298 |
| ENSG00000008405 | 1245.687842 | 0.082041252 | 0.0833168 | 0.2226537 | 0.4826328 | CRY1 | | 1407 |
| ENSG00000136045 | 1732.120039 | -0.155404338 | 0.0788517 | 0.019463 | 0.1016303 | PWP1 | | 11137 |
| ENSG00000110851 | 826.7853801 | -0.023011512 | 0.083774 | 0.7278476 | 0.8782138 | PRDM4 | | 11108 |
| ENSG00000198855 | 102.1896512 | 0.063949836 | 0.1374428 | 0.289941 | 0.5599126 | FICD | | 11153 |
| ENSG00000075856 | 2119.612746 | 0.029636047 | 0.0688875 | 0.6191617 | 0.8179629 | SART3 | | 9733 |
| ENSG00000136003 | 808.7759644 | -0.029752707 | 0.0831336 | 0.6543023 | 0.8400643 | ISCU | | 23479 |
| ENSG00000110880 | 3627.6544 | 0.032509964 | 0.0619556 | 0.5564088 | 0.7758245 | CORO1C | | 23603 |
| ENSG00000084112 | 1665.789341 | 0.237548493 | 0.0818452 | 0.0008058 | 0.0093847 | SSH1 | | 54434 |
| ENSG00000135093 | 148.3996899 | -0.059132591 | 0.1274373 | 0.3663051 | 0.6290304 | USP30 | | 84749 |
| ENSG00000189046 | 539.1623442 | 0.338106989 | 0.1314558 | 0.0010727 | 0.011776 | ALKBH2 | | 121642 |
| ENSG00000076248 | 2224.833832 | 0.067445293 | 0.0672282 | 0.2505838 | 0.5166937 | UNG | | 7374 |
| ENSG00000076555 | 171.4761167 | -0.078472467 | 0.1323263 | 0.2624824 | 0.5300012 | ACACB | | 32 |
| ENSG00000110906 | 1147.248982 | -0.019852773 | 0.0763278 | 0.7532241 | 0.8932431 | KCTD10 | | 83892 |
| ENSG00000151148 | 663.2021387 | 0.020832156 | 0.0871499 | 0.7577415 | 0.8961404 | UBE3B | | 89910 |
| ENSG00000139428 | 134.3828942 | -0.117623816 | 0.1699377 | 0.1076913 | 0.3174217 | MMAB | | 326625 |
| ENSG00000110921 | 464.2811963 | -0.347295729 | 0.1382384 | 0.0011753 | 0.0127358 | MVK | | 4598 |
| ENSG00000139438 | 232.1116242 | 0.094084823 | 0.1361758 | 0.2038977 | 0.4589957 | FAM222A | | 84915 |
| ENSG00000139433 | 938.2277367 | 0.035003649 | 0.0824729 | 0.5864581 | 0.7971162 | GLTP | | 51228 |
| ENSG00000139437 | 815.9526146 | 0.02254582 | 0.082752 | 0.7333558 | 0.8821599 | TCHP | | 84260 |
| ENSG00000139436 | 995.4652682 | -0.049243876 | 0.0826767 | 0.4601229 | 0.7043239 | GIT2 | | 9815 |
| ENSG00000076513 | 976.8779785 | 0.191469417 | 0.1010755 | 0.0158547 | 0.0876471 | ANKRD13A | | 88455 |
| ENSG00000174456 | 95.89714867 | -0.010764522 | 0.1227565 | 0.8426591 | 0.9350382 | C12orf76 | | 400073 |
| ENSG00000122970 | 321.7236221 | -0.044792014 | 0.1069223 | 0.5207354 | 0.7489848 | IFT81 | | 28981 |
| ENSG00000174437 | 7198.360186 | -0.061699004 | 0.0547663 | 0.2180788 | 0.4776634 | ATP2A2 | | 488 |
| ENSG00000196510 | 1087.437524 | -0.037540415 | 0.0778592 | 0.5576807 | 0.7768029 | ANAPC7 | | 51434 |
| ENSG00000111229 | 2944.953344 | -0.049166644 | 0.0617432 | 0.3725471 | 0.6345468 | ARPC3 | | 10094 |
| ENSG00000111231 | 598.662247 | -0.04505362 | 0.0937265 | 0.5153861 | 0.7451617 | GPN3 | | 51184 |
| ENSG00000204856 | 189.3253555 | 0.170528907 | 0.1842218 | 0.0606398 | 0.2195703 | FAM216A | | 29902 |
| ENSG00000111237 | 1592.200385 | -0.16427541 | 0.087377 | 0.0216052 | 0.1093458 | VPS29 | | 51699 |
| ENSG00000151164 | 19.99791355 | -0.0077026 | 0.1333551 | 0.7975654 | NA | RAD9B | | 144715 |
| ENSG00000196850 | 1000.730823 | 0.109050077 | 0.0887542 | 0.1216349 | 0.3414988 | PPTC7 | | 160760 |
| ENSG00000204852 | 116.148243 | 0.05252085 | 0.1320762 | 0.3736442 | 0.6352333 | TCTN1 | | 79600 |
| ENSG00000186298 | 4730.926264 | -0.237144933 | 0.0633579 | 4.08E-05 | 0.0008285 | PPP1CC | | 5501 |
| ENSG00000198324 | 251.7733202 | -0.022934163 | 0.1077847 | 0.7322215 | 0.8813039 | PHETA1 | | 144717 |
| ENSG00000111252 | 1924.120809 | 0.026788489 | 0.0673114 | 0.6293857 | 0.8235526 | SH2B3 | | 10019 |
| ENSG00000204842 | 753.7293707 | 0.012013028 | 0.0856207 | 0.8623917 | 0.9449018 | ATXN2 | | 6311 |
| ENSG00000089234 | 668.3507245 | 0.1806073 | 0.1121234 | 0.0295489 | 0.1370229 | BRAP | | 8315 |
| ENSG00000111271 | 179.8047335 | 0.005111929 | 0.1155104 | 0.9355122 | 0.9752526 | ACAD10 | | 80724 |
| ENSG00000111275 | 26.08939309 | 0.041879601 | 0.1409638 | 0.2378368 | NA | ALDH2 | | 217 |
| ENSG00000234608 | 100.8853086 | 0.109888057 | 0.1692472 | 0.1118669 | 0.3244846 | MAPKAPK5- | | 51275 |
| ENSG00000089022 | 482.1944619 | 0.13230333 | 0.1209582 | 0.1028822 | 0.3074144 | MAPKAPK5 | | 8550 |
| ENSG00000229186 | 33.76270806 | 0.06153088 | 0.1498586 | 0.1375642 | NA | NA | NA | |
| ENSG00000198270 | 69.4212357 | 0.050784562 | 0.1364118 | 0.3377313 | 0.6054388 | TMEM116 | | 89894 |
| ENSG00000089248 | 2137.527286 | -0.014157432 | 0.0671233 | 0.808002 | 0.9200093 | ERP29 | | 10961 |
| ENSG00000111300 | 1448.966842 | 0.084266124 | 0.0786107 | 0.1955568 | 0.4480776 | NAA25 | | 80018 |
| ENSG00000135148 | 429.1585837 | 0.060534715 | 0.1021914 | 0.3975457 | 0.6567942 | TRAFD1 | | 10906 |
| ENSG00000173064 | 1454.208732 | -0.049082761 | 0.0745999 | 0.4335001 | 0.684513 | HECTD4 | | 283450 |
| ENSG00000089009 | 14629.95647 | -0.217804954 | 0.053346 | 1.22E-05 | 0.0002995 | RPL6 | | 6128 |
| ENSG00000179295 | 7603.685345 | -0.066159158 | 0.0539356 | 0.1806672 | 0.4299157 | PTPN11 | | 5781 |
| ENSG00000111331 | 2290.132624 | -0.062120895 | 0.0671265 | 0.2876667 | 0.5574378 | OAS3 | | 4940 |
| ENSG00000123064 | 2738.963123 | -0.120413284 | 0.0680267 | 0.0422527 | 0.1736642 | DDX54 | | 79039 |
| ENSG00000139405 | 607.9070144 | 0.065366701 | 0.0958327 | 0.3568247 | 0.6201207 | RITA1 | | 84934 |
| ENSG00000166578 | 68.97344726 | 0.111329056 | 0.1831544 | 0.0744398 | 0.2503689 | IQCD | | 115811 |
| ENSG00000186815 | 400.2248519 | 0.119498858 | 0.1199061 | 0.1330556 | 0.3595242 | TPCN1 | | 53373 |
| ENSG00000089060 | 540.717104 | 0.052986975 | 0.0950677 | 0.4469245 | 0.6944817 | SLC8B1 | | 80024 |
| ENSG00000151176 | 853.2577239 | 0.024021666 | 0.0834599 | 0.7177055 | 0.8722654 | PLBD2 | | 196463 |
| ENSG00000139410 | 15.26995764 | 0.048820395 | 0.1470584 | 0.0701716 | NA | SDSL | | 113675 |
| ENSG00000122965 | 1383.169832 | 0.024957791 | 0.072132 | 0.68499 | 0.8558528 | RBM19 | | 9904 |

| | | | | | | | | |
|---|---|---|---|---|---|---|---|---|
| ENSG00000123066 | 1847.25744 | -0.04025982 | 0.0734677 | 0.5157195 | 0.7452598 | MED13L | | 23389 |
| ENSG00000171471 | 67.98810872 | 0.061405725 | 0.1420034 | 0.2541821 | 0.5211409 | NA | NA | |
| ENSG00000111412 | 754.297899 | -0.014074149 | 0.0836748 | 0.830827 | 0.9304016 | SPRING1 | | 79794 |
| ENSG00000135119 | 105.3775752 | -0.486572114 | 0.3488996 | 0.0059635 | 0.0432399 | RNFT2 | | 84900 |
| ENSG00000174989 | 293.2608842 | 0.038484852 | 0.1074768 | 0.5662575 | 0.7824977 | FBXW8 | | 26259 |
| ENSG00000088992 | 234.7827721 | -0.048889236 | 0.1148444 | 0.4744777 | 0.7142337 | TESC | | 54997 |
| ENSG00000135108 | 1453.72111 | -0.071776216 | 0.0747562 | 0.2541163 | 0.5211409 | FBXO21 | | 23014 |
| ENSG00000171435 | 19.40513381 | -0.046730662 | 0.1448664 | 0.1282318 | NA | KSR2 | | 283455 |
| ENSG00000111445 | 1409.552012 | -0.025275853 | 0.0720149 | 0.67918 | 0.8535466 | RFC5 | | 5985 |
| ENSG00000176871 | 1243.556567 | -0.175009438 | 0.0981242 | 0.0230623 | 0.1144398 | WSB2 | | 55884 |
| ENSG00000176834 | 640.9417127 | -0.323351934 | 0.1184292 | 0.0007601 | 0.0089778 | VSIG10 | | 54621 |
| ENSG00000089220 | 4065.840751 | -0.117361485 | 0.0621349 | 0.0340896 | 0.1501235 | PEBP1 | | 5037 |
| ENSG00000135090 | 344.1238409 | 0.017089748 | 0.1026795 | 0.8012315 | 0.9169379 | TAOK3 | | 51347 |
| ENSG00000240342 | 608.9594248 | -0.104801583 | 0.1053926 | 0.164586 | 0.4081803 | RPS2P5 | | 100130562 |
| ENSG00000111707 | 1212.983605 | -0.221608339 | 0.0931783 | 0.0039402 | 0.0315044 | SUDS3 | | 64426 |
| ENSG00000111725 | 727.5712665 | 0.00331806 | 0.084255 | 0.9609856 | 0.983972 | PRKAB1 | | 5564 |
| ENSG00000122966 | 2004.938004 | -0.108530403 | 0.0737844 | 0.0834453 | 0.2699322 | CIT | | 11113 |
| ENSG00000135127 | 244.2284676 | 0.023377467 | 0.1086878 | 0.7287636 | 0.8789559 | BICDL1 | | 92558 |
| ENSG00000111737 | 1744.722634 | 0.047928136 | 0.0703791 | 0.4280011 | 0.6793696 | RAB35 | | 11021 |
| ENSG00000089154 | 6779.440987 | -0.039233719 | 0.0566281 | 0.4458117 | 0.6939667 | GCN1 | | 10985 |
| ENSG00000089157 | 21885.1125 | -0.087222314 | 0.0498005 | 0.0596762 | 0.2171581 | RPLP0 | | 6175 |
| ENSG00000255857 | 130.6706252 | 0.044169275 | 0.12781 | 0.4570774 | 0.7016487 | PXN-AS1 | | 100506649 |
| ENSG00000089159 | 8002.562835 | 0.190173614 | 0.0552871 | 0.0001967 | 0.0030309 | PXN | | 5829 |
| ENSG00000089163 | 20.60099461 | -0.021758974 | 0.1353475 | 0.4841279 | NA | SIRT4 | | 23409 |
| ENSG00000111775 | 908.4550098 | 0.015749948 | 0.0855385 | 0.8143692 | 0.9240962 | COX6A1 | | 1337 |
| ENSG00000170855 | 489.0540874 | -0.037838705 | 0.0957834 | 0.5843679 | 0.7955712 | TRIAP1 | | 51499 |
| ENSG00000257218 | 537.437162 | 0.101071454 | 0.1077595 | 0.1842567 | 0.4345452 | GATC | | 283459 |
| ENSG00000111786 | 4563.575681 | -0.164805096 | 0.0650301 | 0.0044171 | 0.0344434 | SRSF9 | | 8683 |
| ENSG00000088986 | 2799.367371 | -0.23145242 | 0.0803804 | 0.0008794 | 0.0100119 | DYNLL1 | | 8655 |
| ENSG00000248008 | 206.3790056 | 0.040380703 | 0.1155149 | 0.5458064 | 0.7679553 | NRAV | | 100506668 |
| ENSG00000110871 | 231.3713156 | -0.184763559 | 0.1750844 | 0.0499912 | 0.1925311 | COQ5 | | 84274 |
| ENSG00000022840 | 2179.112405 | 0.012352069 | 0.0654627 | 0.8306904 | 0.9304016 | RNF10 | | 9921 |
| ENSG00000167272 | 484.1225047 | -0.152344013 | 0.135294 | 0.0746976 | 0.250875 | POP5 | | 51367 |
| ENSG00000110917 | 5044.740147 | -0.111101202 | 0.0584888 | 0.0348706 | 0.1524414 | MLEC | | 9761 |
| ENSG00000256364 | 69.3982374 | -0.046321034 | 0.1364734 | 0.348044 | 0.6140832 | NA | NA | |
| ENSG00000175970 | 1571.24934 | -0.010325039 | 0.0710816 | 0.8641429 | 0.9455189 | UNC119B | | 84747 |
| ENSG00000122971 | 190.236741 | -0.031006143 | 0.1148443 | 0.6326835 | 0.8252447 | ACADS | | 35 |
| ENSG00000157837 | 571.8020545 | -0.189250566 | 0.1195224 | 0.0278515 | 0.131077 | SPPL3 | | 121665 |
| ENSG00000271769 | 19.11333255 | -0.004660598 | 0.13341 | 0.8718867 | NA | NA | NA | |
| ENSG00000157895 | 458.9754423 | -0.101578572 | 0.1108788 | 0.1815139 | 0.4312202 | C12orf43 | | 64897 |
| ENSG00000135124 | 183.0272707 | 0.128210164 | 0.1581511 | 0.1118291 | 0.3244846 | P2RX4 | | 5025 |
| ENSG00000110931 | 1147.814348 | -0.013177135 | 0.075818 | 0.83382 | 0.9305701 | CAMKK2 | | 10645 |
| ENSG00000089053 | 1784.968282 | -0.011973068 | 0.0673502 | 0.8362944 | 0.9319553 | ANAPC5 | | 51433 |
| ENSG00000170633 | 986.9891459 | -0.065689902 | 0.0824193 | 0.3200531 | 0.5888319 | RNF34 | | 80196 |
| ENSG00000089094 | 601.8829523 | -0.07159242 | 0.0960049 | 0.3140104 | 0.5827728 | KDM2B | | 84678 |
| ENSG00000182500 | 622.2994093 | 0.289419383 | 0.1238437 | 0.0026082 | 0.023151 | NA | NA | |
| ENSG00000188735 | 743.7382899 | -0.018350235 | 0.0855892 | 0.7828279 | 0.9096561 | TMEM120B | | 144404 |
| ENSG00000139725 | 1951.220942 | -0.004951035 | 0.0720421 | 0.934713 | 0.9750297 | RHOF | | 54509 |
| ENSG00000212694 | 96.50781329 | -0.127180676 | 0.1888541 | 0.0770512 | 0.2559674 | LINC01089 | | 338799 |
| ENSG00000139718 | 869.3500777 | -0.154902954 | 0.0970074 | 0.0399106 | 0.1674554 | SETD1B | | 23067 |
| ENSG00000110801 | 214.6739526 | 0.006306676 | 0.1104003 | 0.923942 | 0.9709924 | PSMD9 | | 5715 |
| ENSG00000158023 | 15.55140325 | 0.018731038 | 0.1364469 | 0.4295801 | NA | CFAP251 | | 144406 |
| ENSG00000110987 | 215.721522 | -0.021378017 | 0.1108371 | 0.7432793 | 0.8883911 | BCL7A | | 605 |
| ENSG00000175727 | 1691.494014 | -0.15341659 | 0.0767882 | 0.0187589 | 0.0992851 | MLXIP | | 22877 |
| ENSG00000176383 | 40.1133998 | -0.019311065 | 0.1315231 | 0.6355101 | NA | B3GNT4 | | 79369 |
| ENSG00000184047 | 136.4701852 | 0.019782695 | 0.1200565 | 0.7402193 | 0.8863406 | DIABLO | | 56616 |
| ENSG00000139719 | 270.1161841 | 0.006947711 | 0.1071155 | 0.918121 | 0.968009 | VPS33A | | 65082 |
| ENSG00000130779 | 2111.154354 | -0.164929498 | 0.0741383 | 0.0098656 | 0.0631108 | CLIP1 | | 6249 |
| ENSG00000256304 | 31.06979689 | -0.004994711 | 0.1316323 | 0.8867159 | NA | NA | NA | |
| ENSG00000033030 | 760.701692 | 0.106919114 | 0.0946925 | 0.1416859 | 0.3720011 | ZCCHC8 | | 55596 |
| ENSG00000111011 | 1801.144647 | 0.168670583 | 0.0864094 | 0.0177214 | 0.0951305 | RSRC2 | | 65117 |
| ENSG00000184445 | 1403.742254 | -0.1990508 | 0.0885805 | 0.0066778 | 0.0472175 | KNTC1 | | 9735 |
| ENSG00000196917 | 170.3994187 | 0.017531615 | 0.11641 | 0.787097 | 0.9115956 | HCAR1 | | 27198 |
| ENSG00000139726 | 2672.521266 | -0.200039503 | 0.0701028 | 0.0012639 | 0.0134576 | DENR | | 8562 |
| ENSG00000130783 | 10.7874472 | 0.014924463 | 0.136104 | 0.4916617 | NA | CCDC62 | | 84660 |
| ENSG00000130787 | 694.2016538 | -0.235531214 | 0.1241063 | 0.0101981 | 0.0644519 | HIP1R | | 9026 |
| ENSG00000139722 | 834.743415 | 0.050680832 | 0.0862883 | 0.4546856 | 0.6997605 | VPS37B | | 79720 |
| ENSG00000150967 | 210.8549579 | -0.286488715 | 0.2101943 | 0.0149408 | 0.0839056 | ABCB9 | | 23457 |
| ENSG00000111325 | 61.72598212 | 0.016951257 | 0.128643 | 0.7170799 | 0.8718976 | OGFOD2 | | 79676 |
| ENSG00000182196 | 15.36583478 | -0.005979777 | 0.1340631 | 0.8217348 | NA | ARL6IP4 | | 51329 |

| | | | | | | | |
|---|---|---|---|---|---|---|---|
| ENSG00000090975 | 289.5808939 | -0.32625578 | 0.1750261 | 0.0055944 | 0.0412779 | PITPNM2 | 57605 |
| ENSG00000051825 | 1015.584293 | -0.392596963 | 0.0980675 | 6.31E-06 | 0.0001698 | MPHOSPH9 | 10198 |
| ENSG00000130921 | 465.204264 | 0.065649243 | 0.1060011 | 0.3623752 | 0.6257065 | MTRFR | 91574 |
| ENSG00000235423 | 185.7705262 | -0.264064697 | 0.2503734 | 0.0220917 | 0.1110384 | NA | NA |
| ENSG00000111328 | 2022.55826 | -0.394611602 | 0.0865083 | 5.31E-07 | 1.99E-05 | CDK2AP1 | 8099 |
| ENSG00000139697 | 2231.6734 | -0.342345642 | 0.0777394 | 1.36E-06 | 4.38E-05 | SBNO1 | 55206 |
| ENSG00000256092 | 21.25122839 | -0.060827028 | 0.1526419 | 0.0700545 | NA | SBNO1-AS1 | 112268105 |
| ENSG00000183955 | 934.5611473 | -0.597656712 | 0.1015545 | 2.73E-10 | 2.11E-08 | KMT5A | 387893 |
| ENSG00000150977 | 497.9654061 | -0.65526301 | 0.1374218 | 1.15E-07 | 5.45E-06 | RILPL2 | 196383 |
| ENSG00000184209 | 235.2595425 | -0.589753749 | 0.1914886 | 0.0001157 | 0.0019652 | SNRNP35 | 11066 |
| ENSG00000188026 | 129.0834813 | -0.062814871 | 0.1344519 | 0.3123343 | 0.5818701 | RILPL1 | 353116 |
| ENSG00000247373 | 20.9700517 | -0.004191804 | 0.1331794 | 0.8873732 | NA | NA | NA |
| ENSG00000086598 | 3422.221698 | -0.478531901 | 0.0687798 | 3.07E-13 | 4.34E-11 | TMED2 | 10959 |
| ENSG00000111364 | 939.8612516 | -0.144077661 | 0.0961767 | 0.0536132 | 0.2021486 | DDX55 | 57696 |
| ENSG00000111361 | 822.3837162 | -0.305884103 | 0.1122579 | 0.0008332 | 0.0096055 | EIF2B1 | 1967 |
| ENSG00000111358 | 725.7978179 | -0.066582286 | 0.097696 | 0.3418903 | 0.6087837 | GTF2H3 | 2967 |
| ENSG00000168778 | 258.468667 | -0.206428439 | 0.1935294 | 0.0397642 | 0.1670022 | TCTN2 | 79867 |
| ENSG00000185344 | 426.6895445 | 0.095237818 | 0.1168414 | 0.2115276 | 0.4681851 | ATP6V0A2 | 23545 |
| ENSG00000119242 | 137.6185615 | -0.006135248 | 0.1193349 | 0.9156661 | 0.9664675 | CCDC92 | 80212 |
| ENSG00000179195 | 3553.834233 | -0.282006309 | 0.0697473 | 9.09E-06 | 0.0002339 | ZNF664 | 144348 |
| ENSG00000196498 | 3480.605108 | -0.379453592 | 0.0679969 | 2.77E-09 | 1.90E-07 | NCOR2 | 9612 |
| ENSG00000073060 | 799.3932176 | 0.006170784 | 0.0831305 | 0.9263467 | 0.9717697 | SCARB1 | 949 |
| ENSG00000150991 | 11036.78121 | -0.121213165 | 0.1201978 | 0.1291811 | 0.3536359 | UBC | 7316 |
| ENSG00000150990 | 2212.115992 | -0.034629548 | 0.0694253 | 0.5619624 | 0.7800541 | DHX37 | 57647 |
| ENSG00000184992 | 1476.131636 | -0.146311635 | 0.0842573 | 0.0347134 | 0.1519535 | BRI3BP | 140707 |
| ENSG00000081760 | 712.3362024 | -0.035564478 | 0.0880265 | 0.5991988 | 0.80621 | AACS | 65985 |
| ENSG00000256128 | 25.84655194 | 0.020152798 | 0.1342075 | 0.5544495 | NA | NA | NA |
| ENSG00000139370 | 373.9805348 | 0.131642744 | 0.13007 | 0.1097136 | 0.3207922 | SLC15A4 | 121260 |
| ENSG00000111450 | 1030.036255 | -0.056129799 | 0.0815952 | 0.3964571 | 0.6565092 | STX2 | 2054 |
| ENSG00000256299 | 8.573763626 | -0.00310028 | 0.1351462 | 0.876997 | NA | NA | NA |
| ENSG00000132341 | 13193.39956 | -0.341957635 | 0.0548326 | 6.14E-11 | 5.36E-09 | RAN | 5901 |
| ENSG00000061936 | 1054.883904 | 0.012199598 | 0.080079 | 0.8517804 | 0.9396727 | SFSWAP | 6433 |
| ENSG00000198598 | 255.5109017 | 0.000559437 | 0.1063877 | 0.995756 | 0.9983455 | MMP17 | 4326 |
| ENSG00000177169 | 1084.484134 | 0.233038776 | 0.0956709 | 0.003109 | 0.026241 | ULK1 | 8408 |
| ENSG00000255992 | 12.71676501 | -0.006322398 | 0.1345944 | 0.7940637 | NA | NA | NA |
| ENSG00000177192 | 1291.130809 | 0.053754076 | 0.0760601 | 0.3975402 | 0.6567942 | PUS1 | 80324 |
| ENSG00000183495 | 2806.738316 | -0.213683843 | 0.0696306 | 0.0005736 | 0.0071432 | EP400 | 57634 |
| ENSG00000185684 | 218.1985616 | 0.124571572 | 0.147657 | 0.1235267 | 0.344475 | NA | NA |
| ENSG00000185163 | 1450.860247 | 0.040145693 | 0.072044 | 0.513012 | 0.7434855 | DDX51 | 317781 |
| ENSG00000184967 | 796.9037287 | -0.04565781 | 0.0873025 | 0.5003098 | 0.7347747 | NOC4L | 79050 |
| ENSG00000256576 | 37.27070187 | 0.015536799 | 0.1313761 | 0.6992046 | NA | LINC02361 | 100996246 |
| ENSG00000182870 | 24.36504721 | 0.028030619 | 0.1367802 | 0.3834386 | NA | GALNT9 | 50614 |
| ENSG00000112787 | 1182.782962 | 0.116904997 | 0.0840184 | 0.0880663 | 0.279248 | FBRSL1 | 57666 |
| ENSG00000177084 | 4602.367066 | -0.060633444 | 0.0569373 | 0.2438069 | 0.5095953 | POLE | 5426 |
| ENSG00000176894 | 116.3688629 | -0.033011633 | 0.1236187 | 0.5762633 | 0.7897843 | PXMP2 | 5827 |
| ENSG00000247077 | 1550.456234 | -0.124385148 | 0.081167 | 0.0637593 | 0.226985 | PGAM5 | 192111 |
| ENSG00000176915 | 3293.967482 | 0.035388822 | 0.0680454 | 0.5509678 | 0.7718703 | ANKLE2 | 23141 |
| ENSG00000090615 | 2034.253139 | -0.07623903 | 0.0682105 | 0.1972946 | 0.4505568 | GOLGA3 | 2802 |
| ENSG00000072609 | 1084.11205 | 0.014763025 | 0.0765531 | 0.8167613 | 0.9252377 | CHFR | 55743 |
| ENSG00000196458 | 268.62546 | -0.031625212 | 0.1080329 | 0.6416007 | 0.8316176 | ZNF605 | 100289635 |
| ENSG00000198393 | 84.92540849 | 0.385411232 | 0.4290925 | 0.0115305 | 0.0703699 | ZNF26 | 7574 |
| ENSG00000198040 | 169.1640354 | 0.196574875 | 0.2155635 | 0.043155 | 0.1758285 | ZNF84 | 7637 |
| ENSG00000196387 | 247.9895865 | 0.277773206 | 0.189133 | 0.0142888 | 0.0816173 | ZNF140 | 7699 |
| ENSG00000214029 | 93.25218099 | -0.024601262 | 0.1244711 | 0.6564552 | 0.8417183 | ZNF891 | 101060200 |
| ENSG00000256223 | 188.2374791 | 0.569979928 | 0.2161403 | 0.0004464 | 0.0058794 | ZNF10 | 7556 |
| ENSG00000090612 | 386.1680575 | 0.054341985 | 0.1034263 | 0.4445405 | 0.6928188 | ZNF268 | 10795 |
| ENSG00000227879 | 11.89979124 | -0.010663944 | 0.1350462 | 0.6571388 | NA | PSPC1P1 | 642395 |
| ENSG00000196199 | 874.3583773 | -0.007293618 | 0.0811613 | 0.91025 | 0.9647135 | MPHOSPH8 | 54737 |
| ENSG00000121390 | 1623.942255 | -0.04077436 | 0.071329 | 0.5023061 | 0.7365601 | PSPC1 | 55269 |
| ENSG00000132950 | 193.3457791 | 0.074541966 | 0.1309552 | 0.2824007 | 0.5516258 | ZMYM5 | 9205 |
| ENSG00000121741 | 692.8477566 | -0.14156199 | 0.1078589 | 0.0722745 | 0.245561 | ZMYM2 | 7750 |
| ENSG00000121743 | 83.92611324 | 0.752149263 | 0.3354671 | 0.0009611 | 0.0106923 | GJA3 | 2700 |
| ENSG00000165475 | 104.814722 | -0.014678025 | 0.1215827 | 0.794633 | 0.9144629 | CRYL1 | 51084 |
| ENSG00000032742 | 205.4811613 | -0.015760816 | 0.1109956 | 0.8084863 | 0.9203711 | IFT88 | 8100 |
| ENSG00000150456 | 91.77914556 | -0.056538826 | 0.1357007 | 0.3246957 | 0.593455 | EEF1AKMT1 | 221143 |
| ENSG00000132953 | 1707.977771 | -0.094103026 | 0.0732818 | 0.1305348 | 0.3551774 | XPO4 | 64328 |
| ENSG00000150457 | 906.8711177 | -0.039204292 | 0.0814946 | 0.5492219 | 0.770626 | LATS2 | 26524 |
| ENSG00000150459 | 2291.087307 | 0.060687938 | 0.0673984 | 0.3015471 | 0.5710237 | SAP18 | 10284 |
| ENSG00000165480 | 849.0687211 | 0.064519478 | 0.087777 | 0.3470558 | 0.6134112 | SKA3 | 221150 |
| ENSG00000173141 | 605.1913431 | -0.00383551 | 0.0888492 | 0.9523757 | 0.9806656 | MRPL57 | 78988 |

| | | | | | | | | |
|---|---|---|---|---|---|---|---|---|
| ENSG00000233325 | 11.60049665 | 0.021307456 | 0.1372154 | 0.3432608 | NA | NA | NA | |
| ENSG00000180776 | 539.4711396 | -0.205580702 | 0.1317563 | 0.0242553 | 0.1182672 | ZDHHC20 | | 253832 |
| ENSG00000165487 | 496.6680349 | -0.098312891 | 0.108646 | 0.1932808 | 0.4452204 | MICU2 | | 221154 |
| ENSG00000151835 | 2227.007724 | -0.27224444 | 0.0741776 | 4.38E-05 | 0.0008827 | SACS | | 26278 |
| ENSG00000027001 | 232.9626934 | -0.057241503 | 0.1171767 | 0.4092504 | 0.6643076 | MIPEP | | 4285 |
| ENSG00000182957 | 247.8797071 | 0.02781811 | 0.1089906 | 0.6812771 | 0.8538613 | SPATA13 | | 221178 |
| ENSG00000102699 | 1746.698881 | -0.077625789 | 0.0718027 | 0.2051143 | 0.4606691 | PARP4 | | 143 |
| ENSG00000151849 | 713.7091303 | 0.021180217 | 0.0887569 | 0.7551671 | 0.8945449 | CENPJ | | 55835 |
| ENSG00000139505 | 758.3307533 | -0.054301879 | 0.0872923 | 0.4178994 | 0.6710348 | MTMR6 | | 9107 |
| ENSG00000139496 | 1990.938026 | -0.00835163 | 0.0657412 | 0.8923929 | 0.9564219 | NUP58 | | 9818 |
| ENSG00000180730 | 153.1979447 | 0.247396999 | 0.2446896 | 0.0259169 | 0.1241734 | SHISA2 | | 387914 |
| ENSG00000127870 | 1125.352582 | -0.045072669 | 0.0783997 | 0.483749 | 0.7222229 | RNF6 | | 6049 |
| ENSG00000132964 | 418.1809701 | -0.059922171 | 0.103548 | 0.4019477 | 0.659524 | CDK8 | | 1024 |
| ENSG00000132970 | 342.8243469 | -0.054798717 | 0.1083779 | 0.4372755 | 0.6876434 | WASF3 | | 10810 |
| ENSG00000152484 | 1231.009677 | -0.057823646 | 0.077614 | 0.3673411 | 0.6301824 | USP12 | | 219333 |
| ENSG00000122026 | 7599.654784 | -0.204464917 | 0.0556947 | 7.39E-05 | 0.0013709 | RPL21 | | 6144 |
| ENSG00000122035 | 20.54117935 | 0.018702003 | 0.1349958 | 0.5349422 | NA | RASL11A | | 387496 |
| ENSG00000122034 | 1643.764967 | 0.081934541 | 0.0777177 | 0.2038471 | 0.4589703 | GTF3A | | 2971 |
| ENSG00000203602 | 16.27663524 | 0.016294141 | 0.135226 | 0.5517774 | NA | NA | NA | |
| ENSG00000122033 | 138.1824497 | -0.12402497 | 0.16828 | 0.1064423 | 0.3146931 | MTIF3 | | 219402 |
| ENSG00000139517 | 317.4312113 | 0.072009781 | 0.1148337 | 0.3227411 | 0.5915158 | LNX2 | | 222484 |
| ENSG00000186184 | 896.2125265 | -0.015770772 | 0.0800998 | 0.8094932 | 0.9210686 | POLR1D | | 51082 |
| ENSG00000152520 | 633.9489503 | -0.094560099 | 0.0980052 | 0.194657 | 0.4467541 | PAN3 | | 255967 |
| ENSG00000132963 | 2087.124747 | -0.152022589 | 0.0723765 | 0.0150974 | 0.0845006 | POMP | | 51371 |
| ENSG00000139514 | 3679.04868 | 0.186868901 | 0.0682767 | 0.0020093 | 0.0189125 | SLC7A1 | | 6541 |
| ENSG00000122042 | 248.5229842 | -0.043443115 | 0.1115788 | 0.5267014 | 0.7537857 | UBL3 | | 5412 |
| ENSG00000102781 | 671.3009802 | 0.019605241 | 0.0869385 | 0.7714089 | 0.902798 | KATNAL1 | | 84056 |
| ENSG00000189403 | 11136.97028 | -0.362855387 | 0.053827 | 1.86E-12 | 2.22E-10 | HMGB1 | | 3146 |
| ENSG00000132952 | 423.6509756 | 0.011695739 | 0.0959843 | 0.8650567 | 0.9457239 | USPL1 | | 10208 |
| ENSG00000120694 | 6295.110839 | -0.062497676 | 0.1295487 | 0.3405864 | 0.6079446 | HSPH1 | | 10808 |
| ENSG00000187676 | 449.538771 | -0.140221459 | 0.1208261 | 0.0865779 | 0.276165 | B3GLCT | | 145173 |
| ENSG00000073910 | 31.17551261 | -0.034897291 | 0.1373475 | 0.3503705 | NA | FRY | | 10129 |
| ENSG00000139618 | 1074.321161 | 0.328883606 | 0.100305 | 0.0001307 | 0.002172 | BRCA2 | | 675 |
| ENSG00000139597 | 20.7852443 | 0.033578331 | 0.1390073 | 0.2761687 | NA | N4BP2L1 | | 90634 |
| ENSG00000244754 | 775.8235842 | -0.041104429 | 0.0847936 | 0.5364 | 0.7606761 | N4BP2L2 | | 10443 |
| ENSG00000083642 | 1038.973512 | -0.157725549 | 0.0926318 | 0.0324513 | 0.1452349 | PDS5B | | 23047 |
| ENSG00000133121 | 558.8433462 | 0.131549681 | 0.1096953 | 0.094326 | 0.2912561 | STARD13 | | 90627 |
| ENSG00000133119 | 1488.241237 | 0.087403054 | 0.0752838 | 0.1676037 | 0.4125292 | RFC3 | | 5983 |
| ENSG00000172915 | 148.2661248 | -0.02045056 | 0.119557 | 0.7281256 | 0.8783023 | NBEA | | 26960 |
| ENSG00000242715 | 23.12524742 | 0.053983917 | 0.1477873 | 0.1178406 | NA | CCDC169 | | 728591 |
| ENSG00000133104 | 605.4626748 | 0.020776671 | 0.0892038 | 0.7344108 | 0.8828208 | SPART | | 23111 |
| ENSG00000133101 | 27.92089693 | -0.053718127 | 0.1466694 | 0.145971 | NA | CCNA1 | | 8900 |
| ENSG00000133111 | 171.8103431 | -0.206397407 | 0.2110534 | 0.0388648 | 0.1645453 | RFXAP | | 5994 |
| ENSG00000120693 | 23.27400157 | 0.040791913 | 0.1416169 | 0.2011653 | NA | SMAD9 | | 4093 |
| ENSG00000120697 | 398.5390243 | -0.070706902 | 0.1069613 | 0.3304054 | 0.598738 | ALG5 | | 29880 |
| ENSG00000120699 | 951.0067465 | -0.270346698 | 0.101635 | 0.001286 | 0.0136374 | EXOSC8 | | 11340 |
| ENSG00000102710 | 569.7411849 | 0.029693137 | 0.091526 | 0.6651871 | 0.8451219 | SUPT20H | | 55578 |
| ENSG00000120686 | 1205.187191 | 0.280305558 | 0.0933212 | 0.0004295 | 0.0057207 | UFM1 | | 51569 |
| ENSG00000120685 | 723.3188634 | -0.056425728 | 0.0909076 | 0.4142304 | 0.6685232 | PROSER1 | | 80209 |
| ENSG00000188811 | 195.9752285 | -0.053855451 | 0.1203067 | 0.4246129 | 0.6767504 | NHLRC3 | | 387921 |
| ENSG00000183722 | 279.2161182 | 0.172312402 | 0.1575772 | 0.0585477 | 0.2145892 | LHFPL6 | | 10186 |
| ENSG00000133103 | 424.122436 | -0.15513449 | 0.1282733 | 0.067946 | 0.2359327 | COG6 | | 57511 |
| ENSG00000150907 | 187.6902091 | -0.296647401 | 0.2316594 | 0.0150451 | 0.0843289 | FOXO1 | | 2308 |
| ENSG00000102738 | 252.5732444 | 0.03916353 | 0.1103415 | 0.5682424 | 0.7841275 | MRPS31 | | 10240 |
| ENSG00000102743 | 258.7327396 | -0.003793129 | 0.1064213 | 0.9780078 | 0.9917623 | SLC25A15 | | 10166 |
| ENSG00000207652 | 42.42324669 | 0.01215965 | 0.1301209 | 0.7725204 | 0.9036003 | MIR621 | | 693206 |
| ENSG00000120690 | 678.8233067 | -0.08446611 | 0.1068657 | 0.2519513 | 0.5184753 | ELF1 | | 1997 |
| ENSG00000120688 | 427.1864559 | -0.065473785 | 0.1038852 | 0.3631015 | 0.6262607 | WBP4 | | 11193 |
| ENSG00000165572 | 381.4105079 | 0.088571154 | 0.1129374 | 0.2384257 | 0.5030834 | KBTBD6 | | 89890 |
| ENSG00000120696 | 142.7804824 | -0.023618276 | 0.1183724 | 0.6999836 | 0.8636853 | KBTBD7 | | 84078 |
| ENSG00000120662 | 140.8137172 | 0.02219765 | 0.1222657 | 0.6995857 | 0.8633767 | MTRF1 | | 9617 |
| ENSG00000172766 | 557.9192947 | -0.034410972 | 0.0928795 | 0.616202 | 0.815878 | NAA16 | | 79612 |
| ENSG00000102763 | 409.058852 | -0.065892087 | 0.109785 | 0.3596675 | 0.6227803 | VWA8 | | 23078 |
| ENSG00000102780 | 1417.243095 | -0.069919725 | 0.074379 | 0.2649839 | 0.5327957 | DGKH | | 160851 |
| ENSG00000023516 | 1094.849113 | 0.001146984 | 0.0838535 | 0.9870867 | 0.994767 | AKAP11 | | 11215 |
| ENSG00000120675 | 129.5547346 | -0.152679929 | 0.2054296 | 0.0615629 | 0.2214309 | DNAJC15 | | 29103 |
| ENSG00000151773 | 51.89494102 | 0.053085128 | 0.1411182 | 0.2661583 | 0.5340997 | CCDC122 | | 160857 |
| ENSG00000179630 | 134.2858313 | 0.044225764 | 0.1249007 | 0.4754942 | 0.7152005 | LACC1 | | 144811 |
| ENSG00000179611 | 8.419608442 | -0.001375045 | 0.1350973 | 0.9440349 | NA | DGKZP1 | | 400126 |
| ENSG00000102804 | 430.4406211 | 0.140366689 | 0.1240617 | 0.0897209 | 0.2826401 | TSC22D1 | | 8848 |

| | | | | | | | | |
|---|---|---|---|---|---|---|---|---|
| ENSG00000083635 | 287.1018159 | 0.137207993 | 0.1420194 | 0.1017478 | 0.3053742 | NUFIP1 | | 26747 |
| ENSG00000133114 | 431.4557039 | -0.000814132 | 0.0966405 | 0.9891305 | 0.9955163 | GPALPP1 | | 55425 |
| ENSG00000188342 | 590.7805203 | -0.017005702 | 0.0898381 | 0.800889 | 0.9169162 | GTF2F2 | | 2963 |
| ENSG00000253051 | 14.35527352 | -0.028636498 | 0.138396 | 0.2779799 | NA | SNORA31B | | 109616966 |
| ENSG00000133112 | 15014.16437 | -0.105827389 | 0.0504535 | 0.0236974 | 0.1166497 | TPT1 | | 7178 |
| ENSG00000273149 | 939.1109198 | -0.027710278 | 0.0854387 | 0.6780165 | 0.8532068 | NA | NA | |
| ENSG00000170919 | 106.347918 | 0.176986263 | 0.2556357 | 0.0384432 | 0.1634712 | NA | NA | |
| ENSG00000214455 | 11.91974978 | 0.01994158 | 0.1369379 | 0.3743342 | NA | NA | NA | |
| ENSG00000174032 | 260.505014 | -0.033909634 | 0.1100944 | 0.615584 | 0.8158539 | SLC25A30 | | 253512 |
| ENSG00000136152 | 378.6195399 | -0.178861888 | 0.1399943 | 0.0467287 | 0.1849224 | COG3 | | 83548 |
| ENSG00000123200 | 1465.867566 | -0.166796302 | 0.0816558 | 0.0148396 | 0.0835783 | ZC3H13 | | 23091 |
| ENSG00000136141 | 625.3410271 | -0.068840465 | 0.0948872 | 0.330244 | 0.5985902 | LRCH1 | | 23143 |
| ENSG00000139684 | 1118.860083 | -0.140064417 | 0.0884382 | 0.0486735 | 0.1890888 | ESD | | 2098 |
| ENSG00000136143 | 805.6150423 | -0.155231091 | 0.0993255 | 0.0424417 | 0.1741833 | SUCLA2 | | 8803 |
| ENSG00000136159 | 621.3250511 | -0.118234554 | 0.1040995 | 0.1198982 | 0.3380749 | NUDT15 | | 55270 |
| ENSG00000136146 | 683.3660819 | -0.082596841 | 0.094563 | 0.2469502 | 0.5129849 | MED4 | | 29079 |
| ENSG00000136156 | 1574.670041 | -0.092897606 | 0.075246 | 0.1426783 | 0.3738506 | ITM2B | | 9445 |
| ENSG00000139687 | 1463.90124 | -0.066113357 | 0.0746367 | 0.2926181 | 0.5621828 | RB1 | | 5925 |
| ENSG00000136161 | 152.1035681 | 0.069246109 | 0.132366 | 0.2994589 | 0.5693326 | RCBTB2 | | 1102 |
| ENSG00000102531 | 1263.135699 | -0.100881287 | 0.0803066 | 0.126937 | 0.3499531 | FNDC3A | | 22862 |
| ENSG00000102543 | 185.1399438 | 0.086373742 | 0.1349492 | 0.2297425 | 0.4908765 | CDADC1 | | 81602 |
| ENSG00000102547 | 153.196858 | -0.076456935 | 0.1344256 | 0.2634225 | 0.5311649 | CAB39L | | 81617 |
| ENSG00000136169 | 136.1648714 | 0.152746302 | 0.1938333 | 0.0691551 | 0.2389944 | SETDB2 | | 83852 |
| ENSG00000136147 | 147.3333252 | 0.022267337 | 0.1175946 | 0.7205634 | 0.8733776 | PHF11 | | 51131 |
| ENSG00000136144 | 672.1882184 | 0.115982981 | 0.1007674 | 0.1234993 | 0.344475 | RCBTB1 | | 55213 |
| ENSG00000123179 | 558.4859081 | -0.070063424 | 0.0976267 | 0.325217 | 0.5939431 | EBPL | | 84650 |
| ENSG00000102753 | 1294.757489 | -0.171898417 | 0.0870387 | 0.0162632 | 0.0891048 | KPNA3 | | 3839 |
| ENSG00000123178 | 269.0623081 | 0.028486515 | 0.1079112 | 0.6728747 | 0.8500061 | SPRYD7 | | 57213 |
| ENSG00000204977 | 306.8227005 | -0.128025747 | 0.1351069 | 0.1177193 | 0.3343251 | TRIM13 | | 10206 |
| ENSG00000231607 | 85.3394298 | 0.038189435 | 0.1294215 | 0.4854043 | 0.7234177 | DLEU2 | | 8847 |
| ENSG00000176124 | 199.5066905 | 0.124078527 | 0.1515086 | 0.1223741 | 0.34263 | DLEU1 | | 10301 |
| ENSG00000136104 | 294.1011267 | -0.061502497 | 0.1126037 | 0.3848554 | 0.6453482 | RNASEH2B | | 79621 |
| ENSG00000150510 | 61.42514786 | -0.007439574 | 0.126661 | 0.8753148 | 0.9494835 | FAM124A | | 220108 |
| ENSG00000102786 | 817.1386658 | -0.095519263 | 0.096268 | 0.1870944 | 0.4380536 | INTS6 | | 26512 |
| ENSG00000270101 | 40.22584039 | -0.002644318 | 0.1297363 | 0.9460003 | NA | NA | NA | |
| ENSG00000139668 | 432.7135391 | -0.010882567 | 0.0955078 | 0.8723835 | 0.9484107 | WDFY2 | | 115825 |
| ENSG00000102796 | 37.63544965 | 0.049343677 | 0.1421742 | 0.2365522 | NA | DHRS12 | | 79758 |
| ENSG00000123191 | 228.3125742 | 0.138424443 | 0.1554162 | 0.0990218 | 0.3004976 | ATP7B | | 540 |
| ENSG00000253710 | 215.8658434 | 0.014161296 | 0.1110578 | 0.8313849 | 0.9304418 | ALG11 | | 440138 |
| ENSG00000253797 | 518.501136 | -0.011532349 | 0.0937226 | 0.8645523 | 0.9456272 | UTP14C | | 9724 |
| ENSG00000136098 | 216.8063031 | -0.05974543 | 0.1219554 | 0.3805159 | 0.6414787 | NEK3 | | 4752 |
| ENSG00000243406 | 11.37845668 | -0.01708212 | 0.1361598 | 0.462952 | NA | NA | NA | |
| ENSG00000136114 | 25.80555654 | 0.034156276 | 0.1380677 | 0.3246303 | NA | THSD1 | | 55901 |
| ENSG00000136100 | 744.0707848 | -0.166272209 | 0.1106451 | 0.0409222 | 0.1701112 | VPS36 | | 51028 |
| ENSG00000136108 | 2200.794537 | -0.156130536 | 0.0723094 | 0.0126432 | 0.0751199 | CKAP2 | | 26586 |
| ENSG00000139675 | 88.66308633 | -0.031033063 | 0.1273274 | 0.5639388 | 0.7815717 | HNRNPA1L2 | | 144983 |
| ENSG00000165416 | 1067.873502 | -0.082196036 | 0.0818763 | 0.2162963 | 0.4748274 | SUGT1 | | 10910 |
| ENSG00000136149 | 72.87406037 | -0.02975196 | 0.1310635 | 0.5299466 | 0.7556526 | RPL13AP25 | | 100287887 |
| ENSG00000139734 | 1166.85099 | 0.054049276 | 0.0772753 | 0.3959249 | 0.6562498 | DIAPH3 | | 81624 |
| ENSG00000227528 | 12.14863302 | -0.005020577 | 0.1347252 | 0.8277987 | NA | DIAPH3-AS1 | | 100874195 |
| ENSG00000083544 | 175.2825983 | -0.136772526 | 0.1676549 | 0.0946701 | 0.2918558 | TDRD3 | | 81550 |
| ENSG00000236565 | 9.481491039 | -0.010464425 | 0.1354671 | 0.628191 | NA | HNRNPA3P5 | | 387933 |
| ENSG00000165659 | 26.26262106 | 0.024271168 | 0.1348837 | 0.4878767 | NA | NA | NA | |
| ENSG00000204899 | 1070.44505 | -0.035174524 | 0.0785064 | 0.5842714 | 0.7955327 | MZT1 | | 440145 |
| ENSG00000136122 | 365.7676839 | -0.216586903 | 0.1481344 | 0.0247674 | 0.1200448 | BORA | | 79866 |
| ENSG00000083520 | 1611.45313 | 0.164890607 | 0.0865028 | 0.0203345 | 0.1050079 | DIS3 | | 22894 |
| ENSG00000083535 | 211.4105468 | 0.098565919 | 0.1355757 | 0.1910433 | 0.4419839 | PIBF1 | | 10464 |
| ENSG00000102554 | 301.2421541 | 0.482250663 | 0.1695507 | 0.0002977 | 0.0042947 | KLF5 | | 688 |
| ENSG00000118922 | 181.1657403 | -0.017141638 | 0.1136414 | 0.7873796 | 0.9116145 | KLF12 | | 11278 |
| ENSG00000136111 | 985.9748065 | 0.112354836 | 0.0914202 | 0.1160549 | 0.3321012 | TBC1D4 | | 9882 |
| ENSG00000188243 | 348.8394085 | -0.159739047 | 0.1410394 | 0.0674699 | 0.2351177 | COMMD6 | | 170622 |
| ENSG00000118939 | 113.7619372 | -0.071113809 | 0.1401159 | 0.2535126 | 0.520619 | UCHL3 | | 7347 |
| ENSG00000261105 | 10.70603999 | 0.008447822 | 0.1351133 | 0.7109793 | NA | LMO7-AS1 | | 101927155 |
| ENSG00000136153 | 970.8878681 | -0.158363355 | 0.094828 | 0.034109 | 0.1501235 | LMO7 | | 4008 |
| ENSG00000178695 | 1149.541366 | -0.289376288 | 0.0962626 | 0.0003958 | 0.0053885 | KCTD12 | | 115207 |
| ENSG00000102805 | 413.2880668 | -0.015719233 | 0.0970028 | 0.817938 | 0.9254567 | CLN5 | | 1203 |
| ENSG00000005812 | 988.8338809 | -0.114289118 | 0.0884116 | 0.103608 | 0.3087368 | FBXL3 | | 26224 |
| ENSG00000005810 | 2090.634095 | -0.003864435 | 0.0677139 | 0.9559547 | 0.9825402 | MYCBP2 | | 23077 |
| ENSG00000236051 | 11.7532701 | -0.007602824 | 0.1348319 | 0.7462876 | NA | MYCBP2-AS | | 100874212 |
| ENSG00000139737 | 57.96220583 | -0.008011612 | 0.1273727 | 0.8622691 | 0.944856 | SLAIN1 | | 122060 |

| | | | | | | | |
|---|---|---|---|---|---|---|---|
| ENSG00000234377 | 11.11557926 | -0.007587183 | 0.1352834 | 0.7172528 | NA | OBI1-AS1 | 100874222 |
| ENSG00000152193 | 474.8062518 | 0.016652101 | 0.0940288 | 0.8106698 | 0.9219583 | OBI1 | 79596 |
| ENSG00000139746 | 1424.065249 | -0.067109243 | 0.0776828 | 0.2964614 | 0.5664726 | RBM26 | 64062 |
| ENSG00000227354 | 27.95136396 | 0.030943037 | 0.1367769 | 0.3798375 | NA | RBM26-AS1 | 100505538 |
| ENSG00000102471 | 1043.211255 | -0.103287518 | 0.085472 | 0.1307655 | 0.3554741 | NDFIP2 | 54602 |
| ENSG00000136158 | 821.7580471 | -0.066223797 | 0.0890551 | 0.332021 | 0.5992396 | SPRY2 | 10253 |
| ENSG00000215417 | 98.93017109 | -0.016921657 | 0.1230252 | 0.758747 | 0.8963893 | MIR17HG | 407975 |
| ENSG00000088451 | 225.3425639 | 0.074957623 | 0.1263462 | 0.2922197 | 0.5617739 | TGDS | 23483 |
| ENSG00000152749 | 565.5611658 | -0.102817723 | 0.1046434 | 0.1702458 | 0.4161207 | GPR180 | 160897 |
| ENSG00000125257 | 1508.732016 | -0.523565717 | 0.0861474 | 9.74E-11 | 8.24E-09 | ABCC4 | 10257 |
| ENSG00000134874 | 1115.40347 | -0.102158284 | 0.0843206 | 0.1331633 | 0.3595479 | DZIP1 | 22873 |
| ENSG00000247400 | 28.36358651 | -0.021242021 | 0.134055 | 0.5437938 | NA | DNAJC3-DT | 100289274 |
| ENSG00000102580 | 850.2972377 | -0.003299879 | 0.0821897 | 0.9590548 | 0.9831259 | DNAJC3 | 5611 |
| ENSG00000102595 | 477.1462166 | -0.029712623 | 0.0955908 | 0.6655238 | 0.8451381 | UGGT2 | 55757 |
| ENSG00000139793 | 1374.321837 | -0.02014729 | 0.0817435 | 0.7317909 | 0.8810606 | MBNL2 | 10150 |
| ENSG00000125249 | 668.2574112 | -0.086936455 | 0.0996399 | 0.2332527 | 0.4955666 | RAP2A | 5911 |
| ENSG00000065150 | 6075.103924 | -0.21926204 | 0.0576091 | 3.80E-05 | 0.0007866 | IPO5 | 3843 |
| ENSG00000152767 | 821.2421645 | -0.124816014 | 0.0982636 | 0.0939782 | 0.2907971 | FARP1 | 10160 |
| ENSG00000102572 | 1863.834565 | -0.153022137 | 0.0746611 | 0.0167874 | 0.0911641 | STK24 | 8428 |
| ENSG00000088387 | 924.100837 | -0.080863248 | 0.0854117 | 0.2324666 | 0.495067 | DOCK9 | 23348 |
| ENSG00000228889 | 22.84300242 | -0.013621559 | 0.1338807 | 0.6573809 | NA | UBAC2-AS1 | 100289373 |
| ENSG00000134882 | 807.0409569 | -0.033857963 | 0.0838733 | 0.6118834 | 0.8141485 | UBAC2 | 337867 |
| ENSG00000125304 | 1784.230569 | -0.215471376 | 0.0779229 | 0.0014463 | 0.0149371 | TM9SF2 | 9375 |
| ENSG00000125246 | 44.03147073 | -0.022562054 | 0.1317221 | 0.5915469 | 0.800756 | CLYBL | 171425 |
| ENSG00000139800 | 91.89868091 | 0.69701776 | 0.3671488 | 0.0019882 | 0.0187749 | ZIC5 | 85416 |
| ENSG00000043355 | 111.3100166 | -0.042957304 | 0.127515 | 0.4647574 | 0.7086048 | ZIC2 | 7546 |
| ENSG00000175198 | 171.3743659 | -0.104179156 | 0.1469223 | 0.1619059 | 0.4039797 | PCCA | 5095 |
| ENSG00000134864 | 15.68845835 | 0.02584227 | 0.1374133 | 0.3442397 | NA | GGACT | 87769 |
| ENSG00000125247 | 223.9345942 | -0.066693828 | 0.1208092 | 0.3435545 | 0.6099803 | TMTC4 | 84899 |
| ENSG00000102452 | 71.78038936 | -0.004550983 | 0.1251984 | 0.92649 | 0.9718328 | NALCN | 259232 |
| ENSG00000198542 | 96.93079461 | 0.066522051 | 0.1407037 | 0.2607102 | 0.5279074 | ITGBL1 | 9358 |
| ENSG00000134900 | 1198.956784 | -0.083260565 | 0.0804176 | 0.205972 | 0.4609417 | TPP2 | 7174 |
| ENSG00000151287 | 550.7171759 | 0.149559245 | 0.1168455 | 0.0680482 | 0.2361472 | TEX30 | 93081 |
| ENSG00000134901 | 178.6621197 | -0.069084035 | 0.1286169 | 0.311566 | 0.5814671 | POGLUT2 | 79070 |
| ENSG00000134897 | 266.1083601 | 0.004948306 | 0.1069713 | 0.9442641 | 0.9784014 | BIVM | 54841 |
| ENSG00000134899 | 113.7997866 | 0.009006898 | 0.1203191 | 0.8768033 | 0.949999 | ERCC5 | 2073 |
| ENSG00000125266 | 154.3543905 | 0.387712347 | 0.2660944 | 0.00772 | 0.0525817 | EFNB2 | 1948 |
| ENSG00000134884 | 1666.126789 | -0.156917912 | 0.0872915 | 0.0273925 | 0.1292816 | ARGLU1 | 55082 |
| ENSG00000204442 | 11.91914157 | 0.021937435 | 0.1372057 | 0.3497215 | NA | NALF1 | 728215 |
| ENSG00000174405 | 437.2948542 | -0.041881752 | 0.098327 | 0.5476972 | 0.7691852 | LIG4 | 3981 |
| ENSG00000139826 | 399.7664702 | -0.304218085 | 0.1524147 | 0.0049737 | 0.0378394 | ABHD13 | 84945 |
| ENSG00000185950 | 2463.195701 | -0.023653204 | 0.1047901 | 0.7270782 | 0.8779948 | IRS2 | 8660 |
| ENSG00000187498 | 1942.788636 | -0.265639391 | 0.0834199 | 0.0002677 | 0.0039188 | COL4A1 | 1282 |
| ENSG00000134871 | 4168.238246 | -0.211936983 | 0.0683043 | 0.0005244 | 0.0067135 | COL4A2 | 1284 |
| ENSG00000139832 | 46.3116746 | 0.044351316 | 0.1384796 | 0.3154257 | 0.5842828 | RAB20 | 55647 |
| ENSG00000213995 | 472.2412342 | -0.00418546 | 0.0964057 | 0.949212 | 0.9799156 | NAXD | 55739 |
| ENSG00000134905 | 524.1713268 | -0.066027118 | 0.098273 | 0.3562705 | 0.6197178 | CARS2 | 79587 |
| ENSG00000153487 | 306.1067532 | -0.078369837 | 0.1161858 | 0.2868636 | 0.5568053 | ING1 | 3621 |
| ENSG00000255874 | 8.985403824 | 0.002339908 | 0.1350216 | 0.9137273 | NA | PRECSIT | 283487 |
| ENSG00000088448 | 821.1343681 | -0.072011053 | 0.0944438 | 0.3095194 | 0.5790036 | ANKRD10 | 55608 |
| ENSG00000229152 | 83.12877617 | -0.039351707 | 0.1297546 | 0.470516 | 0.7115591 | NA | NA |
| ENSG00000102606 | 1242.817281 | -0.113907505 | 0.0862674 | 0.0999932 | 0.3018463 | ARHGEF7 | 8874 |
| ENSG00000126216 | 765.5016373 | -0.079564635 | 0.0923687 | 0.259183 | 0.525883 | TUBGCP3 | 10426 |
| ENSG00000068650 | 1356.5542 | -0.022986968 | 0.0786129 | 0.7203073 | 0.8733776 | ATP11A | 23250 |
| ENSG00000126217 | 22.57155088 | -0.127152766 | 0.2375613 | 0.0031806 | NA | MCF2L | 23263 |
| ENSG00000126226 | 1006.049912 | -0.050787151 | 0.0802861 | 0.4371289 | 0.687598 | PCID2 | 55795 |
| ENSG00000139842 | 2544.474502 | -0.143379979 | 0.0688716 | 0.0171867 | 0.0929434 | CUL4A | 8451 |
| ENSG00000185896 | 5152.396813 | 0.131790939 | 0.0595079 | 0.0141367 | 0.0810877 | LAMP1 | 3916 |
| ENSG00000139835 | 133.0839268 | -0.175094729 | 0.2154548 | 0.0511777 | 0.1950344 | GRTP1 | 79774 |
| ENSG00000153531 | 11.04970904 | 0.013632074 | 0.1356531 | 0.5557461 | NA | ADPRHL1 | 113622 |
| ENSG00000150401 | 478.681078 | 0.061881967 | 0.1036798 | 0.3649767 | 0.6276009 | DCUN1D2 | 55208 |
| ENSG00000150403 | 791.255014 | -0.132321517 | 0.0985721 | 0.0778136 | 0.2571988 | TMCO3 | 55002 |
| ENSG00000198176 | 3509.675305 | -0.026995558 | 0.0589173 | 0.6107734 | 0.8135056 | TFDP1 | 7027 |
| ENSG00000184497 | 18.15428381 | 0.02826153 | 0.1380892 | 0.3032119 | NA | TMEM255B | 348013 |
| ENSG00000183087 | 483.5765612 | 0.066567474 | 0.1003108 | 0.3538706 | 0.6187516 | GAS6 | 2621 |
| ENSG00000272695 | 13.32235661 | 0.024653694 | 0.137442 | 0.336248 | NA | GAS6-DT | 100506394 |
| ENSG00000260910 | 20.419628 | -0.006332417 | 0.1332291 | 0.8336492 | NA | NA | NA |
| ENSG00000185989 | 1334.758015 | -0.26135276 | 0.0896888 | 0.0006398 | 0.0078008 | RASA3 | 22821 |
| ENSG00000130177 | 964.0934281 | -0.163482419 | 0.0948599 | 0.0293157 | 0.136266 | CDC16 | 8881 |
| ENSG00000169062 | 1182.705889 | 0.063554764 | 0.0822941 | 0.3394497 | 0.6066872 | UPF3A | 65110 |

| | | | | | | | | |
|---|---|---|---|---|---|---|---|---|
| ENSG00000198824 | 722.2077721 | 0.112367622 | 0.1029194 | 0.1363332 | 0.3635864 | CHAMP1 | | 283489 |
| ENSG00000225210 | 574.1517159 | 0.030208844 | 0.0906373 | 0.6590717 | 0.842576 | DUXAP9 | | 503638 |
| ENSG00000244306 | 405.7484832 | 0.034903246 | 0.1000339 | 0.6148143 | 0.8157779 | DUXAP10 | | 503639 |
| ENSG00000136319 | 435.7861048 | -0.057693037 | 0.1011693 | 0.4165548 | 0.6700325 | TTC5 | | 91875 |
| ENSG00000258768 | 17.66813399 | -0.014267159 | 0.1344969 | 0.6197647 | NA | NA | NA | |
| ENSG00000258459 | 13.78358974 | 0.003515371 | 0.1342412 | 0.8926838 | NA | NA | NA | |
| ENSG00000100814 | 740.0962572 | 0.441989297 | 0.1197416 | 1.87E-05 | 0.0004316 | CCNB1IP1 | | 57820 |
| ENSG00000259001 | 48.8911507 | -0.013028606 | 0.1375478 | 0.0143413 | 0.0818271 | NA | NA | |
| ENSG00000129484 | 890.3882905 | 0.016807375 | 0.081649 | 0.7983813 | 0.9162772 | PARP2 | | 10038 |
| ENSG00000254846 | 25.72820572 | -0.002419987 | 0.1320798 | 0.9412508 | NA | NA | NA | |
| ENSG00000129566 | 556.0846686 | -0.038020562 | 0.0922674 | 0.5798886 | 0.7922857 | TEP1 | | 7011 |
| ENSG00000092094 | 570.3617376 | 0.016774096 | 0.0944925 | 0.7976805 | 0.9161052 | OSGEP | | 55644 |
| ENSG00000100823 | 3616.82904 | -0.054103093 | 0.0599043 | 0.3135283 | 0.5823381 | APEX1 | | 328 |
| ENSG00000165782 | 630.693552 | 0.227384985 | 0.1202377 | 0.0111832 | 0.0688982 | PIP4P1 | | 90809 |
| ENSG00000198805 | 1341.559634 | 0.290198125 | 0.0902841 | 0.0002022 | 0.0030916 | PNP | | 4860 |
| ENSG00000258908 | 10.85749465 | 0.004242699 | 0.1347755 | 0.8550808 | NA | NA | NA | |
| ENSG00000214274 | 17.40959912 | 0.032639362 | 0.1392295 | 0.2541762 | NA | ANG | | 283 |
| ENSG00000165792 | 792.0850291 | -0.05524766 | 0.0857397 | 0.4082834 | 0.6640358 | METTL17 | | 64745 |
| ENSG00000258471 | 11.23698129 | 0.004070633 | 0.1347565 | 0.8615529 | NA | LOC101929[illegible] | | 101929718 |
| ENSG00000165799 | 11.992837 | 6.560852449 | 2.5840465 | 1.28E-05 | NA | RNASE7 | | 84659 |
| ENSG00000165801 | 154.2720927 | 0.073367179 | 0.1347592 | 0.2744247 | 0.5429463 | ARHGEF40 | | 55701 |
| ENSG00000165804 | 244.7224414 | 0.025927361 | 0.1105949 | 0.6982865 | 0.8625201 | ZNF219 | | 51222 |
| ENSG00000258441 | 187.5388951 | -0.299261977 | 0.2350286 | 0.0148515 | 0.0836051 | LINC00641 | | 283624 |
| ENSG00000092199 | 11534.20827 | -0.203031369 | 0.0583462 | 0.0001335 | 0.0021999 | HNRNPC | | 3183 |
| ENSG00000092201 | 5933.848329 | -0.165299178 | 0.0627369 | 0.0033439 | 0.0278006 | SUPT16H | | 11198 |
| ENSG00000100888 | 2872.309296 | -0.026862811 | 0.0629842 | 0.6204781 | 0.818389 | CHD8 | | 57680 |
| ENSG00000129472 | 459.7069204 | -0.048201931 | 0.1000702 | 0.4922386 | 0.7283747 | RAB2B | | 84932 |
| ENSG00000092203 | 1428.403928 | -0.002563212 | 0.0712168 | 0.9651783 | 0.9862008 | TOX4 | | 9878 |
| ENSG00000165819 | 1126.133909 | -0.098009534 | 0.0861332 | 0.1540447 | 0.3921962 | METTL3 | | 56339 |
| ENSG00000165821 | 26.18070062 | -0.003815065 | 0.1323494 | 0.9066852 | NA | SALL2 | | 6297 |
| ENSG00000129562 | 1534.615408 | 0.02812452 | 0.0702846 | 0.6415479 | 0.8316176 | DAD1 | | 1603 |
| ENSG00000100439 | 160.6845267 | 0.038986792 | 0.1210699 | 0.5371863 | 0.7611135 | ABHD4 | | 63874 |
| ENSG00000155463 | 1190.102832 | 0.011659088 | 0.0764481 | 0.8548787 | 0.9414385 | OXA1L | | 5018 |
| ENSG00000172590 | 936.8594585 | -0.082436119 | 0.0855722 | 0.2260703 | 0.4871293 | MRPL52 | | 122704 |
| ENSG00000157227 | 965.8192811 | 0.164392989 | 0.0988423 | 0.0327884 | 0.1462403 | MMP14 | | 4323 |
| ENSG00000197324 | 1765.87911 | 0.018916038 | 0.0679675 | 0.7506175 | 0.8924648 | LRP10 | | 26020 |
| ENSG00000100461 | 855.8798784 | 0.021310978 | 0.0830773 | 0.7478643 | 0.890409 | RBM23 | | 55147 |
| ENSG00000237054 | 77.99298476 | -0.010246473 | 0.1246239 | 0.8434508 | 0.9353791 | PRMT5-AS1 | | 100505758 |
| ENSG00000100462 | 3318.984104 | 0.040278894 | 0.0614884 | 0.4650555 | 0.7086768 | PRMT5 | | 10419 |
| ENSG00000092036 | 158.4122516 | 0.054282038 | 0.1249272 | 0.4062965 | 0.6626146 | HAUS4 | | 54930 |
| ENSG00000129474 | 1759.793512 | -0.101291696 | 0.0773646 | 0.1169551 | 0.3337779 | AJUBA | | 84962 |
| ENSG00000100802 | 49.85521707 | -0.024420395 | 0.1313082 | 0.5751417 | 0.7888951 | C14orf93 | | 60686 |
| ENSG00000100804 | 2387.246755 | -0.073910625 | 0.0659743 | 0.200489 | 0.4541229 | PSMB5 | | 5693 |
| ENSG00000139880 | 308.4562361 | -0.017269974 | 0.1040408 | 0.7982073 | 0.9162772 | CDH24 | | 64403 |
| ENSG00000100813 | 5538.177183 | -0.095993968 | 0.058365 | 0.0696611 | 0.2400304 | ACIN1 | | 22985 |
| ENSG00000179933 | 942.0483978 | 0.108638507 | 0.089804 | 0.1251123 | 0.3471045 | C14orf119 | | 55017 |
| ENSG00000215271 | 60.684855 | -0.003366363 | 0.1273939 | 0.9402168 | 0.9766571 | NA | NA | |
| ENSG00000235194 | 199.4772745 | 0.269082294 | 0.2419687 | 0.0207859 | 0.1065373 | PPP1R3E | | 90673 |
| ENSG00000129473 | 699.8963393 | 0.05926631 | 0.0895587 | 0.390427 | 0.6505443 | BCL2L2 | | 599 |
| ENSG00000258643 | 18.22395011 | -0.027544401 | 0.1373613 | 0.3465955 | NA | BCL2L2-PAI[illegible] | | 100529063 |
| ENSG00000100836 | 2261.551903 | -0.25147004 | 0.0734282 | 0.0001256 | 0.0020955 | PABPN1 | | 8106 |
| ENSG00000268702 | 132.2712573 | -0.520724939 | 0.2870338 | 0.0030137 | 0.0257333 | NA | NA | |
| ENSG00000129460 | 597.999907 | 0.018393675 | 0.090266 | 0.7754854 | 0.9053735 | NGDN | | 25983 |
| ENSG00000157306 | 24.15263984 | 0.040937194 | 0.1410963 | 0.2270606 | NA | NA | NA | |
| ENSG00000136367 | 41.17668876 | 0.031489199 | 0.1346259 | 0.4475998 | 0.6951753 | ZFHX2 | | 85446 |
| ENSG00000259431 | 48.67354601 | 0.028278338 | 0.1321803 | 0.5273304 | 0.7544086 | THTPA | | 79178 |
| ENSG00000213983 | 297.2927478 | 0.00471685 | 0.1035526 | 0.9446203 | 0.9784014 | AP1G2 | | 8906 |
| ENSG00000258727 | 106.3039312 | 0.017234791 | 0.1219803 | 0.7627392 | 0.8982943 | AP1G2-AS1 | | 102724814 |
| ENSG00000100867 | 15.03969267 | 0.051824294 | 0.1485606 | 0.0632204 | NA | DHRS2 | | 10202 |
| ENSG00000215256 | 100.3692468 | 0.064626489 | 0.1380524 | 0.2839061 | 0.5531631 | DHRS4-AS1 | | 55449 |
| ENSG00000157326 | 153.3512711 | -0.010237458 | 0.1195089 | 0.8594156 | 0.9433297 | DHRS4 | | 10901 |
| ENSG00000187630 | 53.1280284 | -0.052179501 | 0.1402492 | 0.2781524 | 0.5467102 | DHRS4L2 | | 317749 |
| ENSG00000129535 | 16.32699308 | -0.000176327 | 0.1336535 | 0.9907123 | NA | NRL | | 4901 |
| ENSG00000100889 | 468.2399051 | 0.88112173 | 0.1347952 | 3.59E-12 | 4.07E-10 | PCK2 | | 5106 |
| ENSG00000100897 | 773.4797261 | -0.226186887 | 0.1069885 | 0.0069202 | 0.0482882 | DCAF11 | | 80344 |
| ENSG00000092010 | 1289.512177 | -0.150736689 | 0.0846597 | 0.0302754 | 0.1392215 | PSME1 | | 5720 |
| ENSG00000100908 | 436.6867779 | -0.047367034 | 0.1005976 | 0.4997546 | 0.734688 | EMC9 | | 51016 |
| ENSG00000100911 | 2143.043898 | -0.053106504 | 0.0677087 | 0.3659563 | 0.62873 | PSME2 | | 5721 |
| ENSG00000092098 | 352.7786086 | -0.148681057 | 0.1338453 | 0.0799908 | 0.262096 | RNF31 | | 55072 |
| ENSG00000259529 | 30.53451304 | -0.039716235 | 0.1392465 | 0.2883967 | NA | NA | NA | |

| | | | | | | | | |
|---|---|---|---|---|---|---|---|---|
| ENSG00000213928 | 31.29830499 | 0.021617431 | 0.1341978 | 0.53929 | NA | IRF9 | | 10379 |
| ENSG00000100918 | 59.36611545 | -0.045884621 | 0.1361972 | 0.3532953 | 0.6187516 | REC8 | | 9985 |
| ENSG00000196497 | 25.76530143 | 0.021010756 | 0.1343762 | 0.5368492 | NA | IPO4 | | 79711 |
| ENSG00000100926 | 224.9613957 | -0.044234067 | 0.1141648 | 0.5147192 | 0.744943 | TM9SF1 | | 10548 |
| ENSG00000254505 | 19.07207685 | 0.001061148 | 0.1334866 | 0.9723677 | NA | CHMP4A | | 29082 |
| ENSG00000260669 | 9.22070592 | 0.000450261 | 0.1349556 | 0.9854517 | NA | NA | NA | |
| ENSG00000129559 | 747.3372845 | -0.170218057 | 0.1048997 | 0.032358 | 0.145151 | NEDD8 | | 4738 |
| ENSG00000100938 | 659.6340672 | -0.010962027 | 0.0877752 | 0.8697449 | 0.9475003 | GMPR2 | | 51292 |
| ENSG00000092330 | 341.1935466 | -0.12291037 | 0.1273045 | 0.1272355 | 0.3504452 | TINF2 | | 26277 |
| ENSG00000092295 | 41.30464172 | 0.046211298 | 0.1398336 | 0.2853124 | 0.5548084 | TGM1 | | 7051 |
| ENSG00000100949 | 400.0068523 | 0.066686631 | 0.1054226 | 0.3558627 | 0.6192724 | RABGGTA | | 5875 |
| ENSG00000157379 | 229.6384657 | -0.007879031 | 0.1083292 | 0.9050848 | 0.9623051 | DHRS1 | | 115817 |
| ENSG00000196943 | 1687.966857 | -0.275679942 | 0.0836848 | 0.0001681 | 0.0026518 | NOP9 | | 161424 |
| ENSG00000136305 | 44.59979269 | -0.065416072 | 0.150324 | 0.1514742 | 0.3880199 | CIDEB | | 27141 |
| ENSG00000272658 | 77.27166971 | -0.001572686 | 0.124738 | 0.973459 | 0.9899767 | NA | NA | |
| ENSG00000213903 | 336.5312471 | 0.253554057 | 0.1654066 | 0.0160473 | 0.0884189 | LTB4R | | 1241 |
| ENSG00000100441 | 1141.956146 | 0.15405458 | 0.0924642 | 0.0363067 | 0.1568993 | KHNYN | | 23351 |
| ENSG00000100445 | 461.1226866 | 0.113445399 | 0.115168 | 0.1469101 | 0.3801492 | SDR39U1 | | 56948 |
| ENSG00000184304 | 429.9546478 | -0.139692046 | 0.126366 | 0.0920631 | 0.287261 | PRKD1 | | 5587 |
| ENSG00000092140 | 955.1690576 | -0.464067375 | 0.1063838 | 1.10E-06 | 3.64E-05 | G2E3 | | 55632 |
| ENSG00000092108 | 769.057872 | -0.078653708 | 0.0899032 | 0.2586675 | 0.5253712 | SCFD1 | | 23256 |
| ENSG00000100473 | 931.0649648 | 0.089916514 | 0.0884225 | 0.1979919 | 0.4513782 | COCH | | 1690 |
| ENSG00000258525 | 23.7187045 | 0.006955159 | 0.1327509 | 0.8324311 | NA | LOC1005060 | | 100506071 |
| ENSG00000196792 | 806.2988964 | -0.084976305 | 0.0892422 | 0.2214657 | 0.4809358 | STRN3 | | 29966 |
| ENSG00000100478 | 78.18339918 | -0.064025366 | 0.1423569 | 0.2475431 | 0.5138709 | AP4S1 | | 11154 |
| ENSG00000092148 | 3449.71621 | -0.182292433 | 0.0681904 | 0.0025404 | 0.0227392 | HECTD1 | | 25831 |
| ENSG00000129493 | 273.8067152 | 0.025431347 | 0.1077506 | 0.7020709 | 0.8649636 | HEATR5A | | 25938 |
| ENSG00000129480 | 313.4667332 | -0.089434684 | 0.1194918 | 0.2350128 | 0.4984047 | DTD2 | | 112487 |
| ENSG00000151413 | 183.3906061 | -0.062888857 | 0.1259702 | 0.3513895 | 0.6171375 | NUBPL | | 80224 |
| ENSG00000258655 | 11.29585029 | 0.016414029 | 0.1360065 | 0.48786 | NA | NA | NA | |
| ENSG00000100852 | 1532.518735 | -0.056653584 | 0.0726652 | 0.3584388 | 0.6218058 | ARHGAP5 | | 394 |
| ENSG00000151320 | 8.660452267 | -0.004660995 | 0.1351977 | 0.8178122 | NA | AKAP6 | | 9472 |
| ENSG00000165389 | 655.2649928 | -0.047657238 | 0.0896504 | 0.4864332 | 0.7239115 | SPTSSA | | 171546 |
| ENSG00000129518 | 351.3735281 | 0.031341776 | 0.1021708 | 0.6515172 | 0.8385162 | EAPP | | 55837 |
| ENSG00000129515 | 1887.13765 | -0.069704488 | 0.0726046 | 0.2583163 | 0.5252207 | SNX6 | | 58533 |
| ENSG00000165410 | 742.3834679 | -0.214340562 | 0.1109257 | 0.0115687 | 0.0704924 | CFL2 | | 1073 |
| ENSG00000198604 | 2516.152643 | 0.099987247 | 0.0664224 | 0.0852158 | 0.2732189 | BAZ1A | | 11177 |
| ENSG00000258738 | 53.70925841 | 0.005092657 | 0.1280385 | 0.9117566 | 0.9651317 | BAZ1A-AS1 | | 112268124 |
| ENSG00000258704 | 14.51844448 | 0.000301568 | 0.1339803 | 0.9931416 | NA | NA | NA | |
| ENSG00000100883 | 1447.722292 | -0.012834355 | 0.0735899 | 0.835235 | 0.931409 | SRP54 | | 6729 |
| ENSG00000151327 | 979.4292917 | 0.009075314 | 0.0795848 | 0.8892678 | 0.9549168 | FAM177A1 | | 283635 |
| ENSG00000092020 | 436.6189695 | 0.053195197 | 0.1005367 | 0.4529014 | 0.6987417 | PPP2R3C | | 55012 |
| ENSG00000100890 | 16.30782531 | 0.034815753 | 0.1403468 | 0.2032932 | NA | PRORP | | 9692 |
| ENSG00000100902 | 125.635821 | 0.003958996 | 0.1186298 | 0.947813 | 0.9791797 | PSMA6 | | 5687 |
| ENSG00000100906 | 3166.34775 | 0.008326572 | 0.0707748 | 0.8762541 | 0.949742 | NFKBIA | | 4792 |
| ENSG00000168348 | 14.93279381 | 0.029320961 | 0.1384887 | 0.2796682 | NA | INSM2 | | 84684 |
| ENSG00000174373 | 621.0527839 | -0.006453869 | 0.0908338 | 0.9217185 | 0.9699282 | RALGAPA1 | | 253959 |
| ENSG00000258938 | 13.95708664 | 0.014487851 | 0.1352963 | 0.5729774 | NA | NA | NA | |
| ENSG00000100916 | 464.0614152 | -0.330994734 | 0.138625 | 0.0017491 | 0.0171549 | BRMS1L | | 84312 |
| ENSG00000151332 | 348.4298173 | -0.053642258 | 0.1059502 | 0.4052349 | 0.6614808 | MBIP | | 51562 |
| ENSG00000198807 | 76.07091247 | -0.024189558 | 0.128097 | 0.6272816 | 0.8222551 | PAX9 | | 5083 |
| ENSG00000183032 | 14.00164244 | -0.008710719 | 0.1345279 | 0.7332202 | NA | SLC25A21 | | 89874 |
| ENSG00000258708 | 12.26011011 | 0.025796568 | 0.1380446 | 0.2863357 | NA | SLC25A21-A | | 100129794 |
| ENSG00000151338 | 140.2821395 | -0.037272271 | 0.1223867 | 0.5439408 | 0.7661909 | MIPOL1 | | 145282 |
| ENSG00000129514 | 19.56720057 | 0.001790284 | 0.133119 | 0.9548524 | NA | FOXA1 | | 3169 |
| ENSG00000100934 | 2090.970369 | -0.265187721 | 0.0797975 | 0.0001633 | 0.0025954 | SEC23A | | 10484 |
| ENSG00000092208 | 224.8972218 | 0.014726747 | 0.1097591 | 0.8246558 | 0.9278371 | GEMIN2 | | 8487 |
| ENSG00000182400 | 604.8560901 | 0.17082645 | 0.1142986 | 0.0396845 | 0.1669263 | TRAPPC6B | | 122553 |
| ENSG00000100941 | 5038.228727 | 0.017107268 | 0.0558095 | 0.7379112 | 0.8854328 | PNN | | 5411 |
| ENSG00000150527 | 28.83100282 | 0.010716958 | 0.1321843 | 0.7647867 | NA | MIA2 | | 4253 |
| ENSG00000165355 | 384.8784985 | -0.147887844 | 0.1292338 | 0.0789541 | 0.259901 | FBXO33 | | 254170 |
| ENSG00000179476 | 39.55896542 | 0.018549146 | 0.1318889 | 0.6429385 | NA | C14orf28 | | 122525 |
| ENSG00000179454 | 192.9560111 | 0.007024822 | 0.1118616 | 0.906146 | 0.9628103 | KLHL28 | | 54813 |
| ENSG00000198718 | 557.7092003 | 0.239875017 | 0.1295304 | 0.0107475 | 0.0667418 | TOGARAM1 | | 23116 |
| ENSG00000185246 | 982.151236 | 0.145024726 | 0.0962842 | 0.0526472 | 0.1993426 | PRPF39 | | 55015 |
| ENSG00000100442 | 1614.585622 | -0.156504015 | 0.0793004 | 0.0191484 | 0.100723 | FKBP3 | | 2287 |
| ENSG00000187790 | 349.752697 | 0.026588841 | 0.104407 | 0.6988491 | 0.8629314 | FANCM | | 57697 |
| ENSG00000129534 | 1168.960372 | -0.050564031 | 0.0769601 | 0.4277134 | 0.6791494 | MIS18BP1 | | 55320 |
| ENSG00000213741 | 4236.935006 | -0.40173921 | 0.0787956 | 3.52E-08 | 1.85E-06 | RPS29 | | 6235 |
| ENSG00000258486 | 7251.463761 | -0.020901822 | 0.1372161 | 0.316156 | 0.5849906 | NA | NA | |

| | | | | | | | | |
|---|---|---|---|---|---|---|---|---|
| ENSG00000253459 | 25.77320439 | 0.048433478 | 0.1446694 | 0.1570695 | NA | NA | NA | |
| ENSG00000165501 | 616.2702459 | 0.052444821 | 0.0921317 | 0.452082 | 0.6983298 | LRR1 | | 122769 |
| ENSG00000258568 | 23.23923385 | -0.004897531 | 0.1327101 | 0.8772738 | NA | NA | NA | |
| ENSG00000165502 | 2462.493297 | -0.112209526 | 0.0733191 | 0.0718503 | 0.2445465 | RPL36AL | | 6166 |
| ENSG00000168282 | 1068.739107 | -0.151525814 | 0.0896421 | 0.0355223 | 0.1544689 | MGAT2 | | 4247 |
| ENSG00000258377 | 42.6288639 | -0.023298436 | 0.1321471 | 0.5748809 | 0.7888151 | NA | NA | |
| ENSG00000165506 | 346.1861811 | -0.033239551 | 0.1031913 | 0.6301391 | 0.8239333 | DNAAF2 | | 55172 |
| ENSG00000100479 | 511.2760326 | 0.117110964 | 0.1123273 | 0.1335674 | 0.3601571 | POLE2 | | 5427 |
| ENSG00000165516 | 345.5042414 | 0.064797883 | 0.1097695 | 0.3689384 | 0.6315515 | KLHDC2 | | 23588 |
| ENSG00000165525 | 1736.033433 | 0.047233397 | 0.0711256 | 0.4379354 | 0.6880813 | NEMF | | 9147 |
| ENSG00000266037 | 424.3945651 | -0.020502557 | 0.1380643 | 0.1981992 | 0.4513782 | NA | NA | |
| ENSG00000265150 | 16267.56421 | -0.022410981 | 0.1377026 | 0.2808425 | 0.5496851 | NA | NA | |
| ENSG00000165527 | 5356.655219 | 0.193060085 | 0.0598202 | 0.0004059 | 0.0054747 | ARF6 | | 382 |
| ENSG00000214900 | 59.35515404 | 0.20717104 | 0.3864067 | 0.017588 | 0.0948063 | LINC01599 | | 196913 |
| ENSG00000258929 | 9.640596984 | 0.026992504 | 0.1389513 | 0.2052413 | NA | NA | NA | |
| ENSG00000100483 | 258.4904289 | 0.094475741 | 0.1276153 | 0.2118858 | 0.4684911 | VCPKMT | | 79609 |
| ENSG00000100485 | 702.2442465 | 0.038576166 | 0.0896307 | 0.572973 | 0.7873071 | SOS2 | | 6655 |
| ENSG00000087299 | 724.8004577 | 0.161794199 | 0.105694 | 0.0416919 | 0.1719642 | L2HGDH | | 79944 |
| ENSG00000125375 | 241.8890727 | 0.013072715 | 0.1080773 | 0.8381212 | 0.9331898 | DMAC2L | | 27109 |
| ENSG00000259071 | 11.46550923 | -0.011171891 | 0.1352495 | 0.6312847 | NA | NA | NA | |
| ENSG00000100490 | 77.63631388 | 0.027673821 | 0.1275104 | 0.5993603 | 0.80621 | CDKL1 | | 8814 |
| ENSG00000012983 | 1330.383791 | 0.03901488 | 0.0782601 | 0.5454747 | 0.7678585 | MAP4K5 | | 11183 |
| ENSG00000198513 | 9.183515228 | -0.002851379 | 0.1350565 | 0.8891486 | NA | ATL1 | | 51062 |
| ENSG00000151748 | 916.2536568 | 0.281392027 | 0.1015118 | 0.0008586 | 0.0098328 | SAV1 | | 60485 |
| ENSG00000269906 | 27.50501037 | 0.012438545 | 0.1326157 | 0.7229682 | NA | NA | NA | |
| ENSG00000100503 | 2105.749663 | -0.142757609 | 0.0747101 | 0.0253077 | 0.1220558 | NIN | | 51199 |
| ENSG00000270062 | 13.29747318 | 0.012578717 | 0.1350311 | 0.6237264 | NA | NA | NA | |
| ENSG00000100504 | 4147.097614 | -0.22445105 | 0.0789194 | 0.0010563 | 0.0116398 | PYGL | | 5836 |
| ENSG00000258745 | 10.76229484 | -0.024563525 | 0.1379081 | 0.2872147 | NA | NA | NA | |
| ENSG00000100505 | 146.8362745 | 0.040890333 | 0.122154 | 0.5164315 | 0.7456659 | TRIM9 | | 114088 |
| ENSG00000139921 | 2699.819376 | -0.124482047 | 0.0682246 | 0.036483 | 0.1574281 | TMX1 | | 81542 |
| ENSG00000139926 | 2029.840477 | -0.093360501 | 0.0743112 | 0.1361287 | 0.3632667 | FRMD6 | | 122786 |
| ENSG00000258535 | 10.25971964 | 0.045061848 | 0.146216 | 0.0396003 | NA | NA | NA | |
| ENSG00000087302 | 1364.796886 | -0.078851532 | 0.0759505 | 0.215263 | 0.4733597 | RTRAF | | 51637 |
| ENSG00000087301 | 265.5603692 | 0.154216532 | 0.1601291 | 0.0776108 | 0.256892 | TXNDC16 | | 57544 |
| ENSG00000180998 | 146.5632487 | 0.057487452 | 0.1290833 | 0.3687301 | 0.6315515 | GPR137C | | 283554 |
| ENSG00000197930 | 2119.221771 | -0.065295356 | 0.069407 | 0.2748109 | 0.5431817 | ERO1A | | 30001 |
| ENSG00000100519 | 2337.975788 | -0.406438141 | 0.0741791 | 4.44E-09 | 2.88E-07 | PSMC6 | | 5706 |
| ENSG00000198252 | 561.928001 | -0.077381982 | 0.1025526 | 0.2873256 | 0.5571603 | STYX | | 6815 |
| ENSG00000100522 | 1636.904205 | -0.103285256 | 0.0748719 | 0.1024886 | 0.3068091 | GNPNAT1 | | 64841 |
| ENSG00000259049 | 34.21638489 | -0.022635442 | 0.1332013 | 0.5562251 | NA | NA | NA | |
| ENSG00000073712 | 1278.710248 | -0.234598177 | 0.0880869 | 0.0016444 | 0.0164596 | FERMT2 | | 10979 |
| ENSG00000100523 | 481.3870494 | -0.095782371 | 0.1065536 | 0.2012867 | 0.4553999 | DDHD1 | | 80821 |
| ENSG00000100526 | 953.1220693 | -0.092591675 | 0.086715 | 0.1785815 | 0.4275952 | CDKN3 | | 1033 |
| ENSG00000100528 | 1358.304476 | -0.008561388 | 0.0755916 | 0.8910869 | 0.9559869 | CNIH1 | | 10175 |
| ENSG00000197045 | 2248.170787 | -0.073588546 | 0.0680495 | 0.2140854 | 0.4718872 | GMFB | | 2764 |
| ENSG00000100532 | 231.6259519 | -0.052987143 | 0.1189774 | 0.4346245 | 0.6852726 | CGRRF1 | | 10668 |
| ENSG00000020577 | 1587.396601 | -0.016879433 | 0.0695615 | 0.7772935 | 0.9068683 | SAMD4A | | 23034 |
| ENSG00000131979 | 652.6434104 | 0.272418581 | 0.1175114 | 0.0030644 | 0.0260518 | GCH1 | | 2643 |
| ENSG00000198554 | 1714.370141 | 0.009986177 | 0.0728174 | 0.872246 | 0.9484107 | WDHD1 | | 11169 |
| ENSG00000180008 | 1723.921259 | 0.265268226 | 0.0937033 | 0.0008083 | 0.0094038 | SOCS4 | | 122809 |
| ENSG00000168175 | 2026.860247 | -0.141467457 | 0.0724079 | 0.0233669 | 0.1153771 | MAPK1IP1L | | 93487 |
| ENSG00000131981 | 285.0056619 | 0.042120136 | 0.1092842 | 0.5428965 | 0.7657126 | LGALS3 | | 3958 |
| ENSG00000126787 | 2162.220289 | -0.410352523 | 0.0875314 | 2.76E-07 | 1.12E-05 | DLGAP5 | | 9787 |
| ENSG00000178974 | 672.343388 | -0.117460151 | 0.1017478 | 0.1188862 | 0.3364938 | FBXO34 | | 55030 |
| ENSG00000126775 | 550.8190044 | 0.077862034 | 0.0986829 | 0.2804517 | 0.5491044 | ATG14 | | 22863 |
| ENSG00000186615 | 99.83072196 | 0.354192526 | 0.3804729 | 0.0131183 | 0.0769253 | KTN1-AS1 | | 100129075 |
| ENSG00000126777 | 6555.65378 | -0.263131236 | 0.0582634 | 1.24E-06 | 4.06E-05 | KTN1 | | 3895 |
| ENSG00000070269 | 236.4342552 | 0.068619894 | 0.1238976 | 0.3289668 | 0.5970416 | TMEM260 | | 54916 |
| ENSG00000070367 | 2113.335925 | -0.172474754 | 0.0735762 | 0.0067964 | 0.0476855 | EXOC5 | | 10640 |
| ENSG00000053770 | 559.2055898 | -0.136580191 | 0.1118453 | 0.0859193 | 0.2746016 | AP5M1 | | 55745 |
| ENSG00000139977 | 1029.942842 | 0.053438976 | 0.0813215 | 0.4154808 | 0.6697358 | NAA30 | | 122830 |
| ENSG00000100557 | 92.41358204 | -0.153349059 | 0.2269498 | 0.0457337 | 0.1827592 | CCDC198 | | 55195 |
| ENSG00000139971 | 52.25476269 | -0.016346646 | 0.1299458 | 0.7059953 | 0.8671642 | ARMH4 | | 145407 |
| ENSG00000131966 | 863.5819501 | -0.206037575 | 0.1107281 | 0.014401 | 0.0819203 | ACTR10 | | 55860 |
| ENSG00000100567 | 2051.313209 | -0.170253402 | 0.0765539 | 0.0093839 | 0.0612364 | PSMA3 | | 5684 |
| ENSG00000257621 | 620.1584986 | 0.036519458 | 0.0914239 | 0.5970007 | 0.8047041 | PSMA3-AS1 | | 379025 |
| ENSG00000032219 | 549.9696723 | 4.57E-05 | 0.0917498 | 0.9997041 | 0.9997897 | ARID4A | | 5926 |
| ENSG00000100575 | 304.8851958 | 0.303808206 | 0.1747356 | 0.0080228 | 0.0542016 | TIMM9 | | 26520 |
| ENSG00000100578 | 405.5790922 | 0.041142406 | 0.1033989 | 0.554242 | 0.7749708 | KIAA0586 | | 9786 |

| | | | | | | | | |
|---|---|---|---|---|---|---|---|---|
| ENSG00000258900 | 25.36487292 | 0.046929504 | 0.1437506 | 0.1758635 | NA | NA | NA | |
| ENSG00000100592 | 356.8268404 | 0.109457296 | 0.1256037 | 0.1636933 | 0.4063978 | DAAM1 | | 23002 |
| ENSG00000181619 | 55.89581693 | -0.00231944 | 0.1273191 | 0.9583211 | 0.9831259 | GPR135 | | 64582 |
| ENSG00000126790 | 97.0188736 | 0.144331715 | 0.2059913 | 0.0618221 | 0.2220614 | L3HYPDH | | 112849 |
| ENSG00000050130 | 831.0490116 | -0.103038309 | 0.0940556 | 0.1525018 | 0.3898572 | JKAMP | | 51528 |
| ENSG00000126773 | 1446.003871 | -0.017344914 | 0.0718631 | 0.7760739 | 0.9057168 | PCNX4 | | 64430 |
| ENSG00000100612 | 544.3609937 | -0.207110821 | 0.1303015 | 0.0227827 | 0.1137287 | DHRS7 | | 51635 |
| ENSG00000100614 | 849.4806907 | -0.054062566 | 0.0843102 | 0.4159599 | 0.6697358 | PPM1A | | 5494 |
| ENSG00000126778 | 136.2222883 | 0.255473185 | 0.2793258 | 0.0242155 | 0.1182036 | SIX1 | | 6495 |
| ENSG00000100625 | 251.9374976 | 0.747590773 | 0.1818365 | 2.17E-06 | 6.63E-05 | SIX4 | | 51804 |
| ENSG00000020426 | 448.8655339 | -0.035180067 | 0.0970267 | 0.6110459 | 0.8135902 | MNAT1 | | 4331 |
| ENSG00000126814 | 615.6334644 | 0.066720421 | 0.0979061 | 0.3498924 | 0.6158043 | TRMT5 | | 57570 |
| ENSG00000139974 | 123.2787498 | -0.067675347 | 0.1355487 | 0.2876422 | 0.5574378 | SLC38A6 | | 145389 |
| ENSG00000027075 | 237.9414588 | 0.073831428 | 0.1219116 | 0.2989759 | 0.5691553 | PRKCH | | 5583 |
| ENSG00000250548 | 10.72822291 | 0.017702972 | 0.1364239 | 0.4342172 | NA | LINC01303 | | 101927780 |
| ENSG00000258777 | 27.04948308 | -0.080275579 | 0.1665884 | 0.0427332 | NA | NA | NA | |
| ENSG00000100644 | 11016.5756 | -0.347572646 | 0.0673633 | 3.23E-08 | 1.73E-06 | HIF1A | | 3091 |
| ENSG00000023608 | 529.4044767 | 0.020578971 | 0.0978044 | 0.7627452 | 0.8982943 | SNAPC1 | | 6617 |
| ENSG00000154001 | 1497.722764 | -0.155193629 | 0.0811856 | 0.0222343 | 0.1115282 | PPP2R5E | | 5529 |
| ENSG00000140006 | 280.0875493 | 0.259247224 | 0.1759607 | 0.0164405 | 0.0897807 | WDR89 | | 112840 |
| ENSG00000258800 | 8.465032761 | -0.017975662 | 0.1368358 | 0.3798129 | NA | NA | NA | |
| ENSG00000126821 | 720.057446 | 0.023480575 | 0.0858055 | 0.7266012 | 0.8776165 | SGPP1 | | 81537 |
| ENSG00000054654 | 6753.20482 | -0.262214535 | 0.0692136 | 3.07E-05 | 0.0006547 | SYNE2 | | 23224 |
| ENSG00000214770 | 14.2409318 | 0.04368502 | 0.144417 | 0.101838 | NA | NA | NA | |
| ENSG00000100714 | 5164.909678 | -0.02198701 | 0.0556249 | 0.6645006 | 0.8443847 | MTHFD1 | | 4522 |
| ENSG00000272909 | 20.26460431 | -0.012348381 | 0.1337995 | 0.6848298 | NA | NA | NA | |
| ENSG00000089775 | 287.9158176 | 0.210253336 | 0.1661608 | 0.0329662 | 0.1465289 | ZBTB25 | | 7597 |
| ENSG00000179841 | 99.05204925 | 0.014522453 | 0.1227714 | 0.7944271 | 0.9144367 | AKAP5 | | 9495 |
| ENSG00000126804 | 1058.735701 | -0.024188294 | 0.0796984 | 0.7082883 | 0.8679727 | ZBTB1 | | 22890 |
| ENSG00000126803 | 65.69472047 | -0.024572137 | 0.1284453 | 0.6197428 | 0.8181754 | HSPA2 | | 3306 |
| ENSG00000126822 | 905.2198008 | 0.211345734 | 0.1034514 | 0.0095244 | 0.0617051 | PLEKHG3 | | 26030 |
| ENSG00000070182 | 586.2172609 | 0.053337255 | 0.1331333 | 0.3625449 | 0.625731 | SPTB | | 6710 |
| ENSG00000258289 | 399.9902309 | 0.0359125 | 0.1010932 | 0.6055371 | 0.8105052 | CHURC1 | | 91612 |
| ENSG00000257365 | 111.262743 | -0.061348032 | 0.1345474 | 0.3162719 | 0.5849906 | FNTB | | 2342 |
| ENSG00000139998 | 370.7753606 | -0.048638314 | 0.1041502 | 0.4890305 | 0.7262901 | RAB15 | | 376267 |
| ENSG00000125952 | 880.1005138 | 0.121082721 | 0.0952032 | 0.0990633 | 0.3004976 | MAX | | 4149 |
| ENSG00000259118 | 16.49519254 | 0.005327742 | 0.1337992 | 0.8508969 | NA | LOC100506: | | 100506321 |
| ENSG00000033170 | 399.776865 | -0.08519271 | 0.1099886 | 0.2519411 | 0.5184753 | FUT8 | | 2530 |
| ENSG00000224861 | 166.505116 | -0.085784504 | 0.1389597 | 0.2216049 | 0.4810983 | NA | NA | |
| ENSG00000171723 | 401.8138318 | -0.020934064 | 0.0994785 | 0.7600293 | 0.8970296 | GPHN | | 10243 |
| ENSG00000072415 | 1379.147994 | -0.018319915 | 0.0729717 | 0.7660558 | 0.8994294 | PALS1 | | 64398 |
| ENSG00000100554 | 1064.274222 | 0.052636264 | 0.0800601 | 0.4160836 | 0.6697358 | ATP6V1D | | 51382 |
| ENSG00000134001 | 4222.056249 | 0.047499677 | 0.0568834 | 0.3576423 | 0.6209582 | EIF2S1 | | 1965 |
| ENSG00000100558 | 1145.49477 | 0.328020611 | 0.0996721 | 0.000126 | 0.0020996 | PLEK2 | | 26499 |
| ENSG00000054690 | 264.8303592 | -0.059706074 | 0.1181957 | 0.389976 | 0.6502941 | PLEKHH1 | | 57475 |
| ENSG00000100564 | 342.291238 | 0.060526583 | 0.1078552 | 0.3995879 | 0.6582409 | PIGH | | 5283 |
| ENSG00000081181 | 158.7572754 | -0.064259279 | 0.1284536 | 0.345484 | 0.6117325 | ARG2 | | 384 |
| ENSG00000100568 | 1637.535682 | -0.200221885 | 0.0822187 | 0.0041106 | 0.0325977 | VTI1B | | 10490 |
| ENSG00000072042 | 1073.363743 | -0.088692452 | 0.0904454 | 0.2058581 | 0.4608333 | RDH11 | | 51109 |
| ENSG00000072121 | 969.88932 | 0.088666365 | 0.0861322 | 0.1978417 | 0.4511888 | ZFYVE26 | | 23503 |
| ENSG00000182185 | 135.2640045 | 0.114615412 | 0.1633494 | 0.1216485 | 0.3414988 | RAD51B | | 5890 |
| ENSG00000185650 | 3592.705286 | -0.153040218 | 0.0706487 | 0.0130998 | 0.076855 | ZFP36L1 | | 677 |
| ENSG00000072110 | 11016.20656 | -0.029647915 | 0.054377 | 0.5517354 | 0.7725183 | ACTN1 | | 87 |
| ENSG00000139990 | 697.5687951 | 0.029431879 | 0.0867119 | 0.6627019 | 0.8438537 | DCAF5 | | 8816 |
| ENSG00000081177 | 450.6129645 | 0.001470905 | 0.0946325 | 0.9840103 | 0.9939661 | EXD2 | | 55218 |
| ENSG00000100632 | 3621.758679 | -0.204159259 | 0.0659675 | 0.0005658 | 0.0070767 | ERH | | 2079 |
| ENSG00000029364 | 1039.088391 | -0.027200128 | 0.0818491 | 0.6780514 | 0.8532068 | SLC39A9 | | 55334 |
| ENSG00000100647 | 445.1839667 | 0.067501279 | 0.1033627 | 0.3552528 | 0.6192724 | SUSD6 | | 9766 |
| ENSG00000100650 | 4460.744981 | -0.202077687 | 0.0669647 | 0.0007484 | 0.0088838 | SRSF5 | | 6430 |
| ENSG00000242071 | 473.1359291 | -0.460001943 | 0.1486296 | 0.0001455 | 0.0023541 | RPL7AP6 | | 326310 |
| ENSG00000133983 | 269.5388095 | -0.322064319 | 0.1856624 | 0.0071547 | 0.0495991 | COX16 | | 51241 |
| ENSG00000213463 | 281.9326818 | -0.018841229 | 0.1064397 | 0.7787682 | 0.9075471 | SYNJ2BP | | 55333 |
| ENSG00000133997 | 320.4378157 | 0.006488844 | 0.1020546 | 0.9240895 | 0.9709924 | MED6 | | 10001 |
| ENSG00000006432 | 685.7419947 | 0.160601953 | 0.1077404 | 0.0440459 | 0.1779662 | MAP3K9 | | 4293 |
| ENSG00000259153 | 48.83472702 | 0.027520466 | 0.1319736 | 0.5383601 | 0.7619385 | MAP3K9-DT | | 100506411 |
| ENSG00000100731 | 1712.838418 | -0.06268303 | 0.0702383 | 0.2981448 | 0.5683515 | PCNX1 | | 22990 |
| ENSG00000259146 | 9.688973502 | 0.001347584 | 0.1350626 | 0.9504492 | NA | NA | NA | |
| ENSG00000197555 | 972.6581021 | -0.062154222 | 0.0841818 | 0.3529064 | 0.6185235 | SIPA1L1 | | 26037 |
| ENSG00000205683 | 232.0062306 | 0.002549469 | 0.1097427 | 0.9692789 | 0.9880997 | DPF3 | | 8110 |
| ENSG00000119599 | 891.1301672 | 0.004151062 | 0.0814617 | 0.9502557 | 0.9802134 | DCAF4 | | 26094 |

| | | | | | | | |
|---|---|---|---|---|---|---|---|
| ENSG00000259015 | 11.20879775 | 0.010622904 | 0.1351791 | 0.6522631 | NA | NA | NA |
| ENSG00000165861 | 413.1710626 | 0.084170571 | 0.109543 | 0.2578391 | 0.5246424 | ZFYVE1 | 53349 |
| ENSG00000119707 | 4208.375698 | -0.158510232 | 0.0607599 | 0.0038233 | 0.0307574 | RBM25 | 58517 |
| ENSG00000080815 | 1383.898468 | -0.130420678 | 0.0805711 | 0.0512966 | 0.1953051 | PSEN1 | 5663 |
| ENSG00000100767 | 146.1662686 | -0.025788104 | 0.121973 | 0.657946 | 0.8423052 | PAPLN | 89932 |
| ENSG00000258376 | 67.61084647 | 0.034713378 | 0.1314318 | 0.4866045 | 0.7239115 | NA | NA |
| ENSG00000258944 | 11.13995809 | 0.002136666 | 0.1346636 | 0.9285437 | NA | NA | NA |
| ENSG00000133961 | 1608.121853 | -0.189683608 | 0.0806191 | 0.005623 | 0.0413368 | NUMB | 8650 |
| ENSG00000184227 | 40.51682362 | 0.042160653 | 0.1384044 | 0.3191891 | NA | ACOT1 | 641371 |
| ENSG00000119673 | 452.7316479 | 0.19946481 | 0.1351605 | 0.0293752 | 0.1364342 | ACOT2 | 10965 |
| ENSG00000177465 | 89.14936561 | 0.0604974 | 0.1376215 | 0.2985619 | 0.5686822 | ACOT4 | 122970 |
| ENSG00000119661 | 391.4421044 | 0.086232306 | 0.1115867 | 0.2485774 | 0.5150369 | DNAL1 | 83544 |
| ENSG00000176903 | 1020.467109 | -0.093758998 | 0.0856137 | 0.1705359 | 0.4163942 | PNMA1 | 9240 |
| ENSG00000156030 | 1336.466351 | -0.249806279 | 0.1017848 | 0.0025525 | 0.0228126 | MIDEAS | 91748 |
| ENSG00000259065 | 10.34292562 | -0.009053535 | 0.1352169 | 0.682385 | NA | NA | NA |
| ENSG00000140043 | 106.8092672 | 0.511729023 | 0.3201545 | 0.0043218 | 0.0338583 | PTGR2 | 145482 |
| ENSG00000119725 | 20.10047848 | -0.007125923 | 0.1332744 | 0.8135377 | NA | ZNF410 | 57862 |
| ENSG00000156050 | 136.7769522 | 0.541955672 | 0.2747598 | 0.0021661 | 0.0201178 | FAM161B | 145483 |
| ENSG00000119723 | 252.1741088 | 0.002746563 | 0.1074729 | 0.9717383 | 0.9889245 | COQ6 | 51004 |
| ENSG00000187097 | 427.8865158 | 0.092845337 | 0.1144527 | 0.2201095 | 0.4795932 | ENTPD5 | 957 |
| ENSG00000119636 | 58.55565674 | 0.079436373 | 0.1576097 | 0.1259708 | 0.3483265 | BBOF1 | 80127 |
| ENSG00000119711 | 94.45344668 | -0.051445928 | 0.1332061 | 0.3671715 | 0.6300764 | ALDH6A1 | 4329 |
| ENSG00000205659 | 362.1542962 | 0.021760746 | 0.100196 | 0.7527169 | 0.8932431 | LIN52 | 91750 |
| ENSG00000119688 | 387.3652359 | -0.011650071 | 0.0991529 | 0.8701419 | 0.9477925 | ABCD4 | 5826 |
| ENSG00000258559 | 12.65139077 | -0.003683089 | 0.13445 | 0.8780591 | NA | NA | NA |
| ENSG00000119655 | 1262.259692 | 0.071676451 | 0.0779231 | 0.264056 | 0.531983 | NPC2 | 10577 |
| ENSG00000165898 | 462.0992306 | -0.132195284 | 0.1174427 | 0.0995414 | 0.3010729 | ISCA2 | 122961 |
| ENSG00000119681 | 1419.019886 | 0.390656863 | 0.0878233 | 9.21E-07 | 3.17E-05 | LTBP2 | 4053 |
| ENSG00000258976 | 53.56987532 | 0.027142531 | 0.1309063 | 0.5608182 | 0.7795039 | NA | NA |
| ENSG00000119682 | 1700.938705 | -0.00732757 | 0.0682342 | 0.9001701 | 0.9608779 | AREL1 | 9870 |
| ENSG00000119616 | 986.6742279 | -0.025621688 | 0.0789871 | 0.6935395 | 0.8599124 | FCF1 | 51077 |
| ENSG00000119596 | 2183.406503 | -0.094478744 | 0.0744226 | 0.1325317 | 0.358616 | YLPM1 | 56252 |
| ENSG00000259606 | 9.320065725 | -0.002156835 | 0.1349542 | 0.9175319 | NA | NA | NA |
| ENSG00000119689 | 2475.360579 | 0.053621749 | 0.0676976 | 0.3624373 | 0.6257212 | DLST | 1743 |
| ENSG00000119630 | 75.17170484 | 0.020906256 | 0.127109 | 0.6809271 | 0.8538613 | PGF | 5228 |
| ENSG00000119718 | 1073.62444 | 0.003326937 | 0.0800331 | 0.9599525 | 0.9834728 | EIF2B2 | 8892 |
| ENSG00000258646 | 14.3643929 | 0.001377809 | 0.1342441 | 0.9588179 | NA | NA | NA |
| ENSG00000119684 | 414.7770832 | 0.36675809 | 0.145802 | 0.0010888 | 0.0119201 | MLH3 | 27030 |
| ENSG00000119640 | 252.1550868 | -0.001852473 | 0.1096789 | 0.9761333 | 0.9911521 | ACYP1 | 97 |
| ENSG00000119703 | 74.66767755 | 0.133977604 | 0.2105552 | 0.0503056 | 0.1930585 | ZC2HC1C | 79696 |
| ENSG00000119638 | 256.0754214 | 0.194179422 | 0.176079 | 0.0440321 | 0.1779662 | NEK9 | 91754 |
| ENSG00000170348 | 2593.331594 | -0.114451637 | 0.0674753 | 0.052073 | 0.1975529 | TMED10 | 10972 |
| ENSG00000170345 | 31.90197822 | 0.698053199 | 0.9214525 | 0.0065049 | NA | FOS | 2353 |
| ENSG00000140044 | 111.2416961 | 0.275814909 | 0.3245584 | 0.0204947 | 0.1054617 | JDP2 | 122953 |
| ENSG00000119685 | 643.4008241 | -0.01738474 | 0.0886033 | 0.795718 | 0.9149307 | TTLL5 | 23093 |
| ENSG00000133935 | 654.5002734 | -0.260496068 | 0.1245354 | 0.0055456 | 0.0409986 | ERG28 | 11161 |
| ENSG00000119650 | 274.1127062 | 0.051189685 | 0.1118342 | 0.4609982 | 0.7053865 | IFT43 | 112752 |
| ENSG00000119699 | 17.55068214 | 0.027148674 | 0.1377825 | 0.3208915 | NA | TGFB3 | 7043 |
| ENSG00000089916 | 1434.931664 | 0.084460539 | 0.0768257 | 0.1882371 | 0.4389939 | GPATCH2L | 55668 |
| ENSG00000071246 | 35.1880012 | -0.065034126 | 0.1517965 | 0.1251934 | NA | VASH1 | 22846 |
| ENSG00000258301 | 85.63199211 | 0.066307629 | 0.1415869 | 0.2532607 | 0.520193 | VASH1-AS1 | 100506603 |
| ENSG00000013523 | 473.4710309 | 0.455612221 | 0.1369328 | 6.85E-05 | 0.0012756 | ANGEL1 | 23357 |
| ENSG00000258602 | 24.17445885 | 0.039672039 | 0.1409751 | 0.2205717 | NA | LINC01629 | 105370578 |
| ENSG00000119669 | 1949.858347 | -0.538981785 | 0.1940786 | 0.0003134 | 0.004475 | IRF2BPL | 64207 |
| ENSG00000198894 | 574.556714 | 0.016618397 | 0.0897716 | 0.8072956 | 0.9200019 | CIPC | 85457 |
| ENSG00000165548 | 39.63631625 | 0.056731678 | 0.1458798 | 0.187431 | NA | TMEM63C | 57156 |
| ENSG00000177108 | 13.74935987 | 0.009216044 | 0.1349517 | 0.7019315 | NA | ZDHHC22 | 283576 |
| ENSG00000009830 | 681.6969099 | -0.000112374 | 0.0868558 | 0.9977334 | 0.998947 | POMT2 | 29954 |
| ENSG00000100577 | 137.1328237 | 0.10443438 | 0.1556491 | 0.1449198 | 0.3773536 | GSTZ1 | 2954 |
| ENSG00000100580 | 1256.295407 | 0.257742558 | 0.0895635 | 0.0007409 | 0.008813 | TMED8 | 283578 |
| ENSG00000100583 | 30.63685953 | 0.02697313 | 0.1349985 | 0.464653 | NA | SAMD15 | 161394 |
| ENSG00000151445 | 253.3554456 | 0.023635595 | 0.1108891 | 0.722651 | 0.8747447 | VIPAS39 | 63894 |
| ENSG00000100591 | 2959.590321 | -0.135641882 | 0.0653866 | 0.0189167 | 0.0997748 | AHSA1 | 10598 |
| ENSG00000100596 | 1530.145577 | -0.215737025 | 0.0842773 | 0.0025668 | 0.0228873 | SPTLC2 | 9517 |
| ENSG00000100601 | 286.4808773 | 0.056875886 | 0.1118937 | 0.4196485 | 0.6725085 | ALKBH1 | 8846 |
| ENSG00000119705 | 1048.065044 | -0.080922455 | 0.0840276 | 0.2294074 | 0.4903399 | SLIRP | 81892 |
| ENSG00000100603 | 2065.138112 | 0.026187197 | 0.0684413 | 0.6586224 | 0.8425525 | SNW1 | 22938 |
| ENSG00000063761 | 91.75013661 | 0.02516515 | 0.1249367 | 0.6171439 | 0.8165812 | ADCK1 | 57143 |
| ENSG00000100629 | 486.8684847 | -0.030174982 | 0.097992 | 0.6615944 | 0.8434011 | CEP128 | 145508 |
| ENSG00000165417 | 1202.37854 | 0.024565204 | 0.0777364 | 0.7019111 | 0.8649636 | GTF2A1 | 2957 |

| | | | | | | | | |
|---|---|---|---|---|---|---|---|---|
| ENSG00000140022 | 43.68022675 | 0.093602054 | 0.1741521 | 0.061763 | 0.2219176 | STON2 | | 85439 |
| ENSG00000071537 | 1619.010117 | -0.151668385 | 0.0786863 | 0.0222064 | 0.1115191 | SEL1L | | 6400 |
| ENSG00000185070 | 12.0280995 | -0.011040767 | 0.135066 | 0.6472381 | NA | FLRT2 | | 23768 |
| ENSG00000042317 | 65.76089409 | 0.051367459 | 0.1383074 | 0.3112411 | 0.5812103 | SPATA7 | | 55812 |
| ENSG00000070778 | 224.7576315 | -0.036106949 | 0.1121807 | 0.59084 | 0.8002862 | PTPN21 | | 11099 |
| ENSG00000100722 | 1253.33667 | -0.190976854 | 0.0904622 | 0.0099335 | 0.0633366 | ZC3H14 | | 79882 |
| ENSG00000165521 | 82.30531442 | 0.004816708 | 0.1236185 | 0.9280837 | 0.9724566 | EML5 | | 161436 |
| ENSG00000165533 | 128.2886843 | 0.04164604 | 0.1249593 | 0.4951989 | 0.7306326 | TTC8 | | 123016 |
| ENSG00000053254 | 379.3229713 | -0.007368699 | 0.0984135 | 0.9114376 | 0.964969 | FOXN3 | | 1112 |
| ENSG00000140025 | 132.9953444 | -0.053102438 | 0.1295504 | 0.386871 | 0.647427 | EFCAB11 | | 90141 |
| ENSG00000042088 | 404.9871242 | -0.12988383 | 0.1219269 | 0.1083279 | 0.318502 | TDP1 | | 55775 |
| ENSG00000100764 | 2578.878082 | -0.235461589 | 0.0776543 | 0.0005398 | 0.0068099 | PSMC1 | | 5700 |
| ENSG00000119720 | 277.1250015 | -0.201156258 | 0.167364 | 0.0377784 | 0.1612593 | NRDE2 | | 55051 |
| ENSG00000198668 | 5718.830403 | -0.093723 | 0.0697124 | 0.1172934 | 0.3339561 | CALM1 | | 801 |
| ENSG00000258424 | 30.42886384 | -0.003501625 | 0.1316392 | 0.9194349 | NA | NA | NA | |
| ENSG00000165914 | 304.7914937 | -0.021347847 | 0.1048439 | 0.7526267 | 0.8932431 | TTC7B | | 145567 |
| ENSG00000100784 | 160.8984687 | 0.02695484 | 0.1182942 | 0.6663227 | 0.845553 | RPS6KA5 | | 9252 |
| ENSG00000133943 | 93.48440744 | 0.019401436 | 0.124847 | 0.7191649 | 0.8731289 | DGLUCY | | 80017 |
| ENSG00000015133 | 438.3217158 | 0.395485764 | 0.1411324 | 0.0004401 | 0.0058292 | CCDC88C | | 440193 |
| ENSG00000100796 | 1635.09349 | -0.193021307 | 0.0857303 | 0.0070339 | 0.0489441 | PPP4R3A | | 55671 |
| ENSG00000165929 | 188.4030659 | -0.015736019 | 0.1142105 | 0.8028558 | 0.9177972 | TC2N | | 123036 |
| ENSG00000100815 | 609.2615185 | 0.026914692 | 0.0907622 | 0.7156422 | 0.8712264 | TRIP11 | | 9321 |
| ENSG00000066427 | 169.9567107 | -0.05051928 | 0.1215969 | 0.4433465 | 0.6918811 | ATXN3 | | 4287 |
| ENSG00000183648 | 548.2500096 | -0.001364934 | 0.0970906 | 0.9828907 | 0.9934365 | NDUFB1 | | 4707 |
| ENSG00000165934 | 1347.915997 | -0.206841374 | 0.0868682 | 0.0044573 | 0.034664 | CPSF2 | | 53981 |
| ENSG00000100599 | 169.2208972 | 0.018887074 | 0.1157392 | 0.7651815 | 0.8991133 | RIN3 | | 79890 |
| ENSG00000100600 | 488.7955153 | 0.093419195 | 0.1069785 | 0.2132354 | 0.4704065 | LGMN | | 5641 |
| ENSG00000066455 | 443.2987122 | 0.106990901 | 0.1152406 | 0.167399 | 0.4123551 | GOLGA5 | | 9950 |
| ENSG00000100605 | 742.5227828 | -0.07715572 | 0.0907442 | 0.2694943 | 0.537356 | ITPK1 | | 3705 |
| ENSG00000165943 | 328.2738513 | 0.052089868 | 0.107888 | 0.486516 | 0.7239115 | MOAP1 | | 64112 |
| ENSG00000153485 | 93.31196301 | -0.143798573 | 0.2085689 | 0.0584828 | 0.2144185 | LYSET | | 26175 |
| ENSG00000170270 | 279.6539767 | 0.076840994 | 0.1182109 | 0.2828628 | 0.5522993 | GON7 | | 84520 |
| ENSG00000012963 | 711.870856 | 0.076721615 | 0.0922129 | 0.2769131 | 0.5455116 | UBR7 | | 55148 |
| ENSG00000011114 | 854.651917 | -0.115929986 | 0.0998864 | 0.121599 | 0.3414988 | BTBD7 | | 55727 |
| ENSG00000089723 | 154.2827407 | 0.009369864 | 0.1167705 | 0.879345 | 0.9513156 | OTUB2 | | 78990 |
| ENSG00000089737 | 2458.629202 | 0.05845949 | 0.0652117 | 0.3113428 | 0.5812363 | DDX24 | | 57062 |
| ENSG00000165948 | 102.8271076 | 0.14197595 | 0.2002445 | 0.0665727 | 0.2333139 | IFI27L1 | | 122509 |
| ENSG00000119632 | 40.08652025 | 0.077714738 | 0.1607152 | 0.0872671 | NA | IFI27L2 | | 83982 |
| ENSG00000119698 | 102.6601592 | -0.031558582 | 0.1247394 | 0.5817195 | 0.7933361 | PPP4R4 | | 57718 |
| ENSG00000170099 | 26.10140556 | -0.053988937 | 0.1477156 | 0.1179074 | NA | SERPINA6 | | 866 |
| ENSG00000197249 | 39.41365946 | -0.001535185 | 0.1299965 | 0.9675747 | NA | SERPINA1 | | 5265 |
| ENSG00000100697 | 1117.43013 | -0.113090529 | 0.0862898 | 0.1020981 | 0.3060324 | DICER1 | | 23405 |
| ENSG00000235706 | 9.685413094 | 0.011750603 | 0.1355901 | 0.5937785 | NA | DICER1-AS1 | | 400242 |
| ENSG00000165959 | 509.9938721 | -0.31679658 | 0.1368572 | 0.0022933 | 0.0210102 | CLMN | | 79789 |
| ENSG00000247092 | 125.8704235 | -0.016704587 | 0.1213229 | 0.7695431 | 0.9015177 | SNHG10 | | 283596 |
| ENSG00000252481 | 10.50384633 | -0.0183455 | 0.1365013 | 0.4214988 | NA | SCARNA13 | | 677768 |
| ENSG00000182512 | 711.723088 | -0.137934506 | 0.1041032 | 0.0743242 | 0.250052 | GLRX5 | | 51218 |
| ENSG00000227051 | 381.8641649 | 0.062723778 | 0.1095091 | 0.3827648 | 0.6435041 | C14orf132 | | 56967 |
| ENSG00000066739 | 558.290454 | -0.009494662 | 0.0900084 | 0.8876622 | 0.9545517 | ATG2B | | 55102 |
| ENSG00000100744 | 334.737292 | -0.069893096 | 0.1114457 | 0.335336 | 0.6029229 | GSKIP | | 51527 |
| ENSG00000140057 | 14.88709195 | -0.003968638 | 0.1339968 | 0.8795572 | NA | AK7 | | 122481 |
| ENSG00000260806 | 9.38803687 | -0.001817582 | 0.1349155 | 0.9306223 | NA | NA | NA | |
| ENSG00000090060 | 3691.556856 | -0.337524128 | 0.0695591 | 1.64E-07 | 7.28E-06 | PAPOLA | | 10914 |
| ENSG00000100749 | 438.6197683 | -0.00567408 | 0.0952187 | 0.9324486 | 0.9738664 | VRK1 | | 7443 |
| ENSG00000232573 | 43.5945461 | -0.037646162 | 0.1365679 | 0.3694155 | 0.6317991 | RPL3P4 | | 326307 |
| ENSG00000183576 | 763.1189526 | 0.013641331 | 0.0883227 | 0.8406351 | 0.9344745 | SETD3 | | 84193 |
| ENSG00000090061 | 923.4412037 | -0.067382244 | 0.0835879 | 0.3119174 | 0.5816582 | CCNK | | 8812 |
| ENSG00000205476 | 482.8002583 | -0.024365308 | 0.0964408 | 0.7213563 | 0.8739926 | CCDC85C | | 317762 |
| ENSG00000196405 | 313.682797 | 0.112443754 | 0.1278083 | 0.1564287 | 0.3953362 | EVL | | 51466 |
| ENSG00000258982 | 9.133062403 | 0.034294244 | 0.1416701 | 0.0878006 | NA | NA | NA | |
| ENSG00000100811 | 2219.15559 | -0.08539259 | 0.0695405 | 0.156254 | 0.395151 | YY1 | | 7528 |
| ENSG00000197119 | 303.9997637 | 0.012749648 | 0.1034845 | 0.8519708 | 0.9396727 | SLC25A29 | | 123096 |
| ENSG00000140105 | 1357.146585 | 0.152867881 | 0.082945 | 0.0262977 | 0.1255859 | WARS1 | | 7453 |
| ENSG00000176473 | 38.99617657 | 0.038590019 | 0.1375571 | 0.3420027 | NA | WDR25 | | 79446 |
| ENSG00000183092 | 20.97449786 | 0.034527667 | 0.1390701 | 0.2778968 | NA | BEGAIN | | 57596 |
| ENSG00000078304 | 985.2346047 | -0.285530794 | 0.1009274 | 0.0007023 | 0.0084485 | PPP2R5C | | 5527 |
| ENSG00000271780 | 10.51963625 | 0.026940851 | 0.1386437 | 0.2391014 | NA | NA | NA | |
| ENSG00000197102 | 13978.55362 | 0.115363901 | 0.0554458 | 0.0224923 | 0.1126638 | DYNC1H1 | | 1778 |
| ENSG00000258959 | 13.36605253 | 0.011753747 | 0.1351393 | 0.6325231 | NA | NA | NA | |
| ENSG00000080824 | 31629.28465 | -0.135070987 | 0.0484218 | 0.0017584 | 0.0172023 | HSP90AA1 | | 3320 |

| | | | | | | | | |
|---|---|---|---|---|---|---|---|---|
| ENSG00000140153 | 594.8318849 | -0.116678282 | 0.1042033 | 0.1245874 | 0.3465814 | WDR20 | | 91833 |
| ENSG00000080823 | 431.8085401 | 0.766175651 | 0.1393531 | 2.29E-09 | 1.59E-07 | MOK | | 5891 |
| ENSG00000022976 | 157.7424331 | 0.108346488 | 0.1520676 | 0.1467725 | 0.3800339 | ZNF839 | | 55778 |
| ENSG00000100865 | 344.6113948 | 0.060416596 | 0.1084774 | 0.3992272 | 0.6582037 | CINP | | 51550 |
| ENSG00000196663 | 225.2084239 | 0.075234853 | 0.124196 | 0.2951106 | 0.5652292 | TECPR2 | | 9895 |
| ENSG00000156381 | 251.4164334 | 0.197499274 | 0.172689 | 0.0415878 | 0.1716561 | ANKRD9 | | 122416 |
| ENSG00000089902 | 1094.30464 | -0.0188538 | 0.0766821 | 0.7658281 | 0.8994294 | RCOR1 | | 23186 |
| ENSG00000131323 | 1779.850069 | -0.031550827 | 0.0692881 | 0.5968652 | 0.8046376 | TRAF3 | | 7187 |
| ENSG00000198752 | 2259.25779 | -0.192214642 | 0.0763176 | 0.00361 | 0.0294474 | CDC42BPB | | 9578 |
| ENSG00000185215 | 3417.152529 | -0.413046958 | 0.0823438 | 5.28E-08 | 2.69E-06 | TNFAIP2 | | 7127 |
| ENSG00000100664 | 6192.161082 | -0.00119435 | 0.05524 | 0.9796546 | 0.9923983 | EIF5 | | 1983 |
| ENSG00000075413 | 1114.808136 | -0.086800358 | 0.0812437 | 0.1907558 | 0.4414936 | MARK3 | | 4140 |
| ENSG00000166165 | 244.9356865 | 0.096367635 | 0.1306261 | 0.2034664 | 0.4583554 | CKB | | 1152 |
| ENSG00000260285 | 24.74920706 | 0.021358864 | 0.1345621 | 0.5271608 | NA | NA | NA | |
| ENSG00000166166 | 1116.466547 | 0.428668557 | 0.0966088 | 8.56E-07 | 2.97E-05 | TRMT61A | | 115708 |
| ENSG00000166170 | 1026.857644 | 0.018593201 | 0.0793237 | 0.7743762 | 0.9046394 | BAG5 | | 9529 |
| ENSG00000126214 | 638.2187744 | -0.009830714 | 0.0884974 | 0.8600759 | 0.9435431 | KLC1 | | 3831 |
| ENSG00000256053 | 204.8253537 | 0.05819581 | 0.1205966 | 0.3947258 | 0.654942 | COA8 | | 84334 |
| ENSG00000270108 | 17.14447145 | 0.004185307 | 0.1336852 | 0.8839995 | NA | NA | NA | |
| ENSG00000269940 | 160.0213564 | 0.008702642 | 0.1164714 | 0.8689157 | 0.9471245 | NA | NA | |
| ENSG00000269958 | 163.6662709 | 0.165879814 | 0.2022842 | 0.0590453 | 0.2158711 | NA | NA | |
| ENSG00000126215 | 1146.732506 | 0.195081959 | 0.0945801 | 0.0107057 | 0.0665256 | XRCC3 | | 7517 |
| ENSG00000224997 | 17.41636465 | 0.026574954 | 0.13719 | 0.3602215 | NA | NA | NA | |
| ENSG00000100711 | 430.1017626 | -0.024967112 | 0.101094 | 0.7137247 | 0.870525 | ZFYVE21 | | 79038 |
| ENSG00000088808 | 395.0197497 | -0.076965817 | 0.1099775 | 0.2947732 | 0.5650223 | PPP1R13B | | 23368 |
| ENSG00000156411 | 773.3693467 | -0.126516082 | 0.0997032 | 0.0921943 | 0.287262 | ATP5MJ | | 9556 |
| ENSG00000203485 | 1481.726367 | -0.005840628 | 0.0708341 | 0.9224678 | 0.9701942 | INF2 | | 64423 |
| ENSG00000184990 | 997.6888596 | -0.027177793 | 0.0797721 | 0.674523 | 0.8505206 | SIVA1 | | 10572 |
| ENSG00000258430 | 56.96260458 | 0.011338342 | 0.1283269 | 0.8055509 | 0.9187021 | NA | NA | |
| ENSG00000142208 | 3853.770903 | -0.061727692 | 0.0644052 | 0.2765202 | 0.5448773 | AKT1 | | 207 |
| ENSG00000179627 | 94.74225861 | 0.014511548 | 0.1236432 | 0.7899525 | 0.9118019 | ZBTB42 | | 100128927 |
| ENSG00000099814 | 1561.538065 | 0.057219114 | 0.0741998 | 0.3605272 | 0.6238323 | CEP170B | | 283638 |
| ENSG00000185567 | 1405.620065 | -0.322730507 | 0.1082492 | 0.0003531 | 0.0049401 | AHNAK2 | | 113146 |
| ENSG00000140104 | 144.9344702 | 0.968900747 | 0.2406259 | 2.79E-06 | 8.26E-05 | CLBA1 | | 122616 |
| ENSG00000170779 | 1182.59914 | 0.056765245 | 0.079758 | 0.3853926 | 0.6459432 | CDCA4 | | 55038 |
| ENSG00000257556 | 9.245589355 | 0.012026448 | 0.1357834 | 0.5684548 | NA | NA | NA | |
| ENSG00000184916 | 1119.923542 | 0.054024419 | 0.0836734 | 0.4191102 | 0.6718301 | JAG2 | | 3714 |
| ENSG00000183828 | 280.3266895 | -0.014683065 | 0.1050193 | 0.8268338 | 0.9287505 | NUDT14 | | 256281 |
| ENSG00000185024 | 791.5071176 | 0.006637298 | 0.0841566 | 0.9211375 | 0.9697015 | BRF1 | | 2972 |
| ENSG00000184887 | 785.0173464 | -0.002628743 | 0.0881232 | 0.973535 | 0.9899767 | BTBD6 | | 90135 |
| ENSG00000179364 | 516.0317969 | 0.115039505 | 0.1111824 | 0.1385133 | 0.3673017 | PACS2 | | 23241 |
| ENSG00000251602 | 32.75260953 | -0.033168343 | 0.137079 | 0.3609845 | NA | MTA1-DT | | 100507437 |
| ENSG00000182979 | 1269.818089 | -0.105007164 | 0.0816794 | 0.1162512 | 0.3325516 | MTA1 | | 9112 |
| ENSG00000182809 | 266.9558933 | 0.246878415 | 0.1790798 | 0.0205237 | 0.1055283 | CRIP2 | | 1397 |
| ENSG00000213145 | 12.25343956 | 0.011125981 | 0.135131 | 0.6454181 | NA | CRIP1 | | 1396 |
| ENSG00000185347 | 599.1909376 | 0.034145512 | 0.092612 | 0.6324305 | 0.8251856 | TEDC1 | | 283643 |
| ENSG00000184986 | 61.36986512 | 0.025943624 | 0.1301122 | 0.586055 | 0.7968554 | TMEM121 | | 80757 |
| ENSG00000180229 | 23.99871172 | -0.004159901 | 0.1328451 | 0.8940149 | NA | NA | NA | |
| ENSG00000258488 | 15.095174 | -0.012867501 | 0.1347499 | 0.6311489 | NA | LOC100996 | | 100996379 |
| ENSG00000153575 | 635.567658 | 0.029553972 | 0.088205 | 0.6632031 | 0.8438599 | NA | NA | |
| ENSG00000068793 | 2692.306436 | -0.18430498 | 0.0700632 | 0.0027619 | 0.0241845 | NA | NA | |
| ENSG00000140157 | 1333.614392 | 0.002296204 | 0.0739672 | 0.971462 | 0.9887295 | NIPA2 | | 81614 |
| ENSG00000170113 | 1660.021894 | 0.060130252 | 0.0745388 | 0.3381402 | 0.6057127 | NIPA1 | | 123606 |
| ENSG00000187667 | 85.19704279 | -0.03615999 | 0.1288111 | 0.5052939 | 0.7382367 | NA | NA | |
| ENSG00000140181 | 1051.401336 | 0.004456535 | 0.0778847 | 0.9453087 | 0.9784014 | NA | NA | |
| ENSG00000128739 | 22.58025322 | 0.027711827 | 0.136532 | 0.3965146 | NA | SNRPN | | 6638 |
| ENSG00000224078 | 476.3257356 | -0.069722486 | 0.1038337 | 0.3347664 | 0.6022573 | NA | NA | |
| ENSG00000270704 | 9.160977021 | 0.00103731 | 0.1351273 | 0.9607873 | NA | NA | NA | |
| ENSG00000239014 | 9.742631447 | -0.035273133 | 0.141742 | 0.1036747 | NA | SNORD108 | | 338427 |
| ENSG00000271347 | 14.39429622 | -0.03041697 | 0.1389589 | 0.2510963 | NA | NA | NA | |
| ENSG00000257151 | 54.97574039 | -0.059357635 | 0.1441805 | 0.2231606 | 0.4834315 | NA | NA | |
| ENSG00000270246 | 9.896786631 | -0.036534004 | 0.1422522 | 0.0924114 | NA | NA | NA | |
| ENSG00000261069 | 23.52984455 | 0.013156513 | 0.1337261 | 0.6758005 | NA | NA | NA | |
| ENSG00000114062 | 1439.167015 | -0.057434028 | 0.0735576 | 0.3557097 | 0.6192724 | UBE3A | | 7337 |
| ENSG00000128731 | 2743.7318 | -0.043893885 | 0.0641718 | 0.4377494 | 0.6880813 | HERC2 | | 8924 |
| ENSG00000206149 | 384.3519514 | 0.010372149 | 0.0991509 | 0.8812628 | 0.9522588 | NA | NA | |
| ENSG00000248334 | 20.28445062 | 0.001003048 | 0.1330014 | 0.9739039 | NA | NA | NA | |
| ENSG00000034053 | 94.29330687 | -0.020079146 | 0.1265867 | 0.6922143 | 0.8594028 | APBA2 | | 321 |
| ENSG00000185115 | 191.9085824 | 0.032079236 | 0.1156182 | 0.6229301 | 0.819512 | NSMCE3 | | 56160 |
| ENSG00000256802 | 17.39554622 | 0.004988133 | 0.1337182 | 0.8614632 | NA | LCIIAR | | 100130111 |

| | | | | | | | | |
|---|---|---|---|---|---|---|---|---|
| ENSG00000104067 | 1823.422244 | -0.181277351 | 0.0765906 | 0.0059071 | 0.0429902 | TJP1 | | 7082 |
| ENSG00000215302 | 40.61950337 | -0.019182257 | 0.1321452 | 0.6242597 | NA | NA | NA | |
| ENSG00000270055 | 86.14936049 | 0.005937832 | 0.1240436 | 0.911261 | 0.9649147 | NA | NA | |
| ENSG00000261794 | 29.43187934 | -0.024095247 | 0.1343891 | 0.5035604 | NA | GOLGA8H | | 728498 |
| ENSG00000187951 | 715.73191 | -0.065746626 | 0.0901149 | 0.3424197 | 0.6089788 | LOC100288( | | 100288637 |
| ENSG00000269974 | 37.75048594 | -0.062660499 | 0.1495193 | 0.1497016 | NA | NA | NA | |
| ENSG00000198690 | 397.4298222 | 0.089972961 | 0.1152674 | 0.2334636 | 0.4958342 | FAN1 | | 22909 |
| ENSG00000270015 | 62.3823201 | 0.073613096 | 0.151455 | 0.1669274 | 0.4122331 | NA | NA | |
| ENSG00000166912 | 644.3585441 | 0.048504786 | 0.0906428 | 0.4824988 | 0.7213706 | MTMR10 | | 54893 |
| ENSG00000169926 | 531.49645 | 0.003319144 | 0.090837 | 0.9563793 | 0.9825402 | KLF13 | | 51621 |
| ENSG00000169918 | 15.37996543 | -0.042398478 | 0.1434793 | 0.1277949 | NA | OTUD7A | | 161725 |
| ENSG00000175344 | 20.13853768 | 0.003933419 | 0.1330913 | 0.8997128 | NA | CHRNA7 | | 1139 |
| ENSG00000223509 | 83.44959359 | 0.045220357 | 0.1319742 | 0.4113891 | 0.6661689 | NA | NA | |
| ENSG00000198826 | 2599.392459 | -0.358455181 | 0.0711846 | 5.86E-08 | 2.96E-06 | ARHGAP11/ | | 9824 |
| ENSG00000166922 | 21.7269659 | 0.051206438 | 0.1465422 | 0.1244588 | NA | SCG5 | | 6447 |
| ENSG00000169857 | 124.7032102 | 0.000502146 | 0.1184412 | 0.9943837 | 0.9977447 | AVEN | | 57099 |
| ENSG00000134153 | 902.3342264 | 0.076433384 | 0.0872941 | 0.2690727 | 0.536996 | EMC7 | | 56851 |
| ENSG00000182405 | 29.40346874 | 0.027357189 | 0.1354218 | 0.4466713 | NA | PGBD4 | | 161779 |
| ENSG00000134152 | 416.9886908 | -0.091994535 | 0.1106033 | 0.2211953 | 0.4806143 | KATNBL1 | | 79768 |
| ENSG00000128463 | 912.4492138 | 0.02767079 | 0.0810965 | 0.6729889 | 0.8500105 | EMC4 | | 51234 |
| ENSG00000140199 | 209.0482473 | -0.026091625 | 0.1126093 | 0.6900259 | 0.8589854 | SLC12A6 | | 9990 |
| ENSG00000182117 | 1230.945846 | -0.083203742 | 0.0793273 | 0.2025822 | 0.4568267 | NOP10 | | 55505 |
| ENSG00000176454 | 397.9184778 | -0.009308812 | 0.0996353 | 0.8921129 | 0.956385 | LPCAT4 | | 254531 |
| ENSG00000175265 | 753.6631909 | 0.21511891 | 0.1163455 | 0.013604 | 0.0790196 | GOLGA8A | | 23015 |
| ENSG00000215252 | 538.1053704 | 0.088509832 | 0.103305 | 0.2339832 | 0.4966028 | GOLGA8B | | 440270 |
| ENSG00000021776 | 1021.260954 | -0.201400036 | 0.0955592 | 0.0090409 | 0.0594636 | AQR | | 9716 |
| ENSG00000198146 | 1159.479252 | 0.182428051 | 0.096811 | 0.0176218 | 0.0948571 | ZNF770 | | 54989 |
| ENSG00000212768 | 17.82993184 | 0.005083025 | 0.1336689 | 0.8605474 | NA | NA | NA | |
| ENSG00000134146 | 100.3306067 | -0.08432913 | 0.1495676 | 0.1844444 | 0.4347246 | DPH6 | | 89978 |
| ENSG00000186073 | 134.473555 | 0.029908215 | 0.1213324 | 0.6221816 | 0.8187116 | CDIN1 | | 84529 |
| ENSG00000134138 | 240.4622131 | -0.178185643 | 0.1822513 | 0.0550334 | 0.2057103 | MEIS2 | | 4212 |
| ENSG00000166068 | 562.035212 | -0.129432741 | 0.1101413 | 0.0991861 | 0.3005468 | SPRED1 | | 161742 |
| ENSG00000171262 | 809.6612414 | -0.161001025 | 0.1033495 | 0.0401007 | 0.1678682 | FAM98B | | 283742 |
| ENSG00000259345 | 29.15582615 | -0.015265288 | 0.1328784 | 0.6617657 | NA | NA | NA | |
| ENSG00000137801 | 4136.558542 | 0.034146131 | 0.1035557 | 0.6212828 | 0.818389 | THBS1 | | 7057 |
| ENSG00000150667 | 22.13573164 | 0.002273363 | 0.1328817 | 0.9443487 | NA | FSIP1 | | 161835 |
| ENSG00000261136 | 8.893417754 | -0.001925591 | 0.1350003 | 0.9250542 | NA | LOC105370! | | 105370941 |
| ENSG00000166073 | 318.297361 | 0.118906257 | 0.1329092 | 0.1395642 | 0.3687721 | GPR176 | | 11245 |
| ENSG00000128829 | 1322.093726 | -0.121361951 | 0.0811599 | 0.0699929 | 0.2404666 | EIF2AK4 | | 440275 |
| ENSG00000140319 | 2677.782523 | -0.137405948 | 0.0679112 | 0.0208221 | 0.1065373 | SRP14 | | 6727 |
| ENSG00000248508 | 29.00708062 | 0.009857991 | 0.1322437 | 0.7806453 | NA | SRP14-DT | | 100131089 |
| ENSG00000104081 | 14.81175569 | 0.023369501 | 0.1368117 | 0.3862752 | NA | BMF | | 90427 |
| ENSG00000156970 | 2153.691133 | -0.127373445 | 0.0716485 | 0.0390132 | 0.1650538 | BUB1B | | 701 |
| ENSG00000137841 | 21.62559153 | -0.01274268 | 0.1336105 | 0.6836815 | NA | PLCB2 | | 5330 |
| ENSG00000259330 | 326.4317033 | -0.070791739 | 0.1120775 | 0.3297016 | 0.5978967 | INAFM2 | | 100505573 |
| ENSG00000188549 | 1665.039894 | 0.022119395 | 0.0686809 | 0.7102321 | 0.8685323 | CCDC9B | | 388115 |
| ENSG00000140323 | 245.7048797 | -0.016662962 | 0.1090007 | 0.8006589 | 0.9169162 | DISP2 | | 85455 |
| ENSG00000128944 | 830.3992504 | -0.101606634 | 0.0939358 | 0.1577389 | 0.3975891 | KNSTRN | | 90417 |
| ENSG00000128928 | 504.0361325 | 0.005829549 | 0.0951324 | 0.9334207 | 0.974432 | IVD | | 3712 |
| ENSG00000140320 | 614.0376548 | 0.017995133 | 0.0894851 | 0.7531853 | 0.8932431 | BAHD1 | | 22893 |
| ENSG00000169105 | 335.1556719 | -0.040735624 | 0.1082758 | 0.5543918 | 0.7749708 | CHST14 | | 113189 |
| ENSG00000128891 | 297.022103 | 0.009401659 | 0.1035006 | 0.8906666 | 0.9557116 | CCDC32 | | 90416 |
| ENSG00000166133 | 214.4375932 | 0.046556068 | 0.115807 | 0.4932123 | 0.7288856 | RPUSD2 | | 27079 |
| ENSG00000137812 | 1873.323172 | -0.141442689 | 0.0778921 | 0.0311134 | 0.141635 | KNL1 | | 57082 |
| ENSG00000245849 | 50.74868176 | 0.023505924 | 0.1307546 | 0.602314 | 0.808043 | RAD51-AS1 | | 100505648 |
| ENSG00000051180 | 511.8993978 | 0.126109509 | 0.1115495 | 0.1112534 | 0.3233517 | RAD51 | | 5888 |
| ENSG00000137824 | 775.6415412 | 0.033162804 | 0.0868825 | 0.6243109 | 0.8202204 | RMDN3 | | 55177 |
| ENSG00000137880 | 326.8395672 | -0.072673529 | 0.1130871 | 0.3183467 | 0.5871795 | GCHFR | | 2644 |
| ENSG00000104129 | 194.19087 | -0.000927042 | 0.1112876 | 0.9887266 | 0.9955163 | DNAJC17 | | 55192 |
| ENSG00000188277 | 179.4424407 | -0.109215272 | 0.1525691 | 0.1452119 | 0.3776933 | C15orf62 | | 643338 |
| ENSG00000166140 | 448.4181597 | 0.273164298 | 0.1394435 | 0.006701 | 0.0472732 | ZFYVE19 | | 84936 |
| ENSG00000166145 | 12.03981579 | 0.022379838 | 0.1371056 | 0.3600581 | NA | SPINT1 | | 6692 |
| ENSG00000104140 | 23.44854832 | 0.00110311 | 0.1324929 | 0.9750605 | NA | RHOV | | 171177 |
| ENSG00000104142 | 813.7246645 | 0.186464401 | 0.1016738 | 0.0186544 | 0.0990466 | VPS18 | | 57617 |
| ENSG00000128917 | 29.32146194 | 0.041078341 | 0.140009 | 0.2669753 | NA | DLL4 | | 54567 |
| ENSG00000128965 | 151.2282383 | 1.086434583 | 0.2365231 | 2.21E-07 | 9.30E-06 | CHAC1 | | 79094 |
| ENSG00000128908 | 1030.350269 | -0.068834118 | 0.0842498 | 0.3067737 | 0.5761131 | INO80 | | 54617 |
| ENSG00000187446 | 1013.977792 | -0.139170169 | 0.0915115 | 0.0547488 | 0.2049746 | CHP1 | | 11261 |
| ENSG00000247556 | 1416.13758 | -0.122108528 | 0.0807253 | 0.0677598 | 0.2357767 | OIP5-AS1 | | 729082 |
| ENSG00000104147 | 318.3634549 | -0.201713319 | 0.1615649 | 0.0364975 | 0.1574324 | OIP5 | | 11339 |

| | | | | | | | | |
|---|---|---|---|---|---|---|---|---|
| ENSG00000137804 | 3074.681633 | -0.005127349 | 0.0626082 | 0.9285091 | 0.9726302 | NUSAP1 | | 51203 |
| ENSG00000137806 | 244.9363751 | -0.002903733 | 0.1075979 | 0.9639496 | 0.9854626 | NDUFAF1 | | 51103 |
| ENSG00000137815 | 1325.370828 | -0.202157399 | 0.0895267 | 0.0063254 | 0.0453014 | RTF1 | | 23168 |
| ENSG00000137825 | 142.7563096 | -0.053525076 | 0.1261563 | 0.4033836 | 0.6604883 | ITPKA | | 3706 |
| ENSG00000103932 | 631.3602656 | 0.107008969 | 0.100594 | 0.1507774 | 0.3871206 | RPAP1 | | 26015 |
| ENSG00000092445 | 475.0674599 | 0.068216168 | 0.1008221 | 0.3432952 | 0.609766 | TYRO3 | | 7301 |
| ENSG00000174197 | 1670.708258 | 0.077605686 | 0.0738512 | 0.2144371 | 0.4721961 | MGA | | 23269 |
| ENSG00000137802 | 540.4625552 | 0.038936837 | 0.0938043 | 0.5699648 | 0.7854543 | MAPKBP1 | | 23005 |
| ENSG00000168970 | 56.9399369 | -0.039606644 | 0.1342148 | 0.4101314 | 0.6651964 | JMJD7-PLA2 | | 8681 |
| ENSG00000272003 | 13.11669754 | -0.013062249 | 0.1351872 | 0.5997428 | NA | NA | NA | |
| ENSG00000103966 | 762.7866026 | -0.070836409 | 0.0894594 | 0.3061231 | 0.5752194 | EHD4 | | 30844 |
| ENSG00000166887 | 593.8630309 | 0.045033707 | 0.0951979 | 0.5184634 | 0.7474909 | VPS39 | | 23339 |
| ENSG00000103978 | 670.7757797 | -0.076420164 | 0.0956482 | 0.2834352 | 0.5529069 | TMEM87A | | 25963 |
| ENSG00000214013 | 122.7286951 | -0.006124214 | 0.1201112 | 0.9151807 | 0.9662935 | GANC | | 2595 |
| ENSG00000103994 | 2000.236522 | -0.06653345 | 0.070017 | 0.2686492 | 0.5365697 | ZNF106 | | 64397 |
| ENSG00000092531 | 739.2401527 | -0.09415758 | 0.0937597 | 0.1880678 | 0.4389252 | SNAP23 | | 8773 |
| ENSG00000180979 | 340.730638 | 0.103604741 | 0.1221863 | 0.182243 | 0.4318741 | LRRC57 | | 255252 |
| ENSG00000137814 | 751.8529053 | 0.05626912 | 0.0898448 | 0.4150617 | 0.6693823 | HAUS2 | | 55142 |
| ENSG00000159433 | 495.6321534 | -0.097182252 | 0.1103302 | 0.1995259 | 0.4530837 | STARD9 | | 57519 |
| ENSG00000140326 | 409.583716 | -0.084489633 | 0.1117087 | 0.2559462 | 0.5230675 | CDAN1 | | 146059 |
| ENSG00000128881 | 522.435858 | 0.008835617 | 0.0918248 | 0.8977236 | 0.9594787 | TTBK2 | | 146057 |
| ENSG00000159459 | 624.5463976 | 0.055541244 | 0.0933821 | 0.4285243 | 0.6796361 | UBR1 | | 197131 |
| ENSG00000137842 | 162.7221153 | -0.050594839 | 0.1224756 | 0.4394215 | 0.6887002 | TMEM62 | | 80021 |
| ENSG00000166946 | 491.264868 | -0.040667399 | 0.0953608 | 0.5572404 | 0.77656 | CCNDBP1 | | 23582 |
| ENSG00000168806 | 135.3105232 | 0.03488981 | 0.1219776 | 0.5700108 | 0.7854543 | LCMT2 | | 9836 |
| ENSG00000168803 | 205.9666769 | 0.190814033 | 0.1898651 | 0.0473843 | 0.1859327 | ADAL | | 161823 |
| ENSG00000140265 | 555.9138584 | 0.018371645 | 0.0940545 | 0.7984682 | 0.9162772 | ZSCAN29 | | 146050 |
| ENSG00000137822 | 956.2219817 | -0.01257682 | 0.0802154 | 0.8457079 | 0.9369039 | TUBGCP4 | | 27229 |
| ENSG00000067369 | 1266.654486 | -0.117988002 | 0.0817371 | 0.0789649 | 0.259901 | TP53BP1 | | 7158 |
| ENSG00000166963 | 63.11682696 | -0.021482455 | 0.1284461 | 0.6567346 | 0.8418917 | MAP1A | | 4130 |
| ENSG00000168781 | 212.0434474 | -0.039019331 | 0.1152626 | 0.5550263 | 0.7750463 | PPIP5K1 | | 9677 |
| ENSG00000166762 | 49.40179933 | -0.069905431 | 0.1514814 | 0.1548901 | 0.3932344 | CATSPER2 | | 117155 |
| ENSG00000249839 | 9.74487307 | -0.017208038 | 0.1365689 | 0.414783 | NA | NA | NA | |
| ENSG00000205771 | 14.14892849 | -0.003246723 | 0.1341801 | 0.8971113 | NA | NA | NA | |
| ENSG00000167004 | 5334.469586 | -0.185186003 | 0.0633949 | 0.0012148 | 0.0130783 | PDIA3 | | 2923 |
| ENSG00000128886 | 8.637305852 | -0.000631943 | 0.1350572 | 0.9732184 | NA | ELL3 | | 80237 |
| ENSG00000140264 | 3962.98788 | -0.0570356 | 0.0617844 | 0.3020261 | 0.5712647 | SERF2 | | 10169 |
| ENSG00000221792 | 41.93659227 | -0.029601679 | 0.1342689 | 0.4665472 | 0.7094916 | MIR1282 | | 100302254 |
| ENSG00000242028 | 42.63269323 | -0.006956279 | 0.12953 | 0.86627 | 0.9459476 | HYPK | | 25764 |
| ENSG00000140259 | 934.1463686 | 0.193543723 | 0.0971196 | 0.0126039 | 0.0749626 | MFAP1 | | 4236 |
| ENSG00000092470 | 786.6862268 | 0.068327842 | 0.0880386 | 0.3219725 | 0.5908703 | WDR76 | | 79968 |
| ENSG00000171877 | 34.86775611 | -0.021335108 | 0.1327658 | 0.5812382 | NA | FRMD5 | | 84978 |
| ENSG00000166734 | 1025.193355 | -0.132720291 | 0.0894831 | 0.0627441 | 0.2242697 | GOLM2 | | 113201 |
| ENSG00000259595 | 8.667217795 | -0.011838754 | 0.1357915 | 0.5673124 | NA | NA | NA | |
| ENSG00000137770 | 872.7621018 | -0.050477264 | 0.0847849 | 0.4493056 | 0.6962034 | CTDSPL2 | | 51496 |
| ENSG00000179523 | 109.9186608 | -0.080930974 | 0.1488267 | 0.1902924 | 0.4411203 | EIF3J-DT | | 645212 |
| ENSG00000104131 | 2141.240375 | -0.05925709 | 0.0676417 | 0.3131807 | 0.582248 | EIF3J | | 8669 |
| ENSG00000104133 | 403.1735184 | 0.082946584 | 0.1093866 | 0.2637985 | 0.5316478 | SPG11 | | 80208 |
| ENSG00000166710 | 4212.603086 | -0.031762069 | 0.0599769 | 0.5548578 | 0.7749708 | B2M | | 567 |
| ENSG00000185880 | 92.76849602 | -0.039211052 | 0.1282444 | 0.4866153 | 0.7239115 | TRIM69 | | 140691 |
| ENSG00000259479 | 15.97188528 | 0.01042909 | 0.1344041 | 0.7031741 | NA | NA | NA | |
| ENSG00000140263 | 801.7087357 | -0.000649584 | 0.0839647 | 0.9764548 | 0.9912634 | SORD | | 6652 |
| ENSG00000138606 | 11.93699056 | 0.012683443 | 0.1354356 | 0.590763 | NA | SHF | | 90525 |
| ENSG00000171763 | 249.0872447 | 0.293730266 | 0.1996028 | 0.012612 | 0.0749725 | AFG2B | | 79029 |
| ENSG00000166920 | 1089.221479 | 0.14511498 | 0.0890037 | 0.0428136 | 0.1749231 | C15orf48 | | 84419 |
| ENSG00000259354 | 26.81610919 | 0.036180187 | 0.1385333 | 0.308372 | NA | NA | NA | |
| ENSG00000104154 | 153.2849788 | 0.01851901 | 0.1166285 | 0.7671372 | 0.8999629 | SLC30A4 | | 7782 |
| ENSG00000179362 | 10.92312919 | 0.009508664 | 0.1351055 | 0.6838325 | NA | NA | NA | |
| ENSG00000104164 | 907.8714036 | -0.001759094 | 0.0798177 | 0.9775111 | 0.9914307 | BLOC1S6 | | 26258 |
| ENSG00000137767 | 1071.976413 | -0.074867985 | 0.0887529 | 0.2785256 | 0.5467586 | SQOR | | 58472 |
| ENSG00000128951 | 1559.782726 | 0.069195639 | 0.081432 | 0.2936124 | 0.563537 | DUT | | 1854 |
| ENSG00000166147 | 173.1026804 | 0.082146664 | 0.1348109 | 0.2443963 | 0.5100385 | FBN1 | | 2200 |
| ENSG00000103995 | 527.4302958 | 0.312996894 | 0.1300477 | 0.0018905 | 0.0181606 | CEP152 | | 22995 |
| ENSG00000255302 | 725.2599385 | -0.377669426 | 0.1144136 | 9.61E-05 | 0.001683 | EID1 | | 23741 |
| ENSG00000138593 | 752.3301137 | -0.243340543 | 0.1161299 | 0.0063904 | 0.0456555 | SECISBP2L | | 9728 |
| ENSG00000166200 | 1107.853985 | -0.096655243 | 0.0850728 | 0.1566373 | 0.3956066 | COPS2 | | 9318 |
| ENSG00000156958 | 136.5701939 | -0.02075238 | 0.1195843 | 0.7281465 | 0.8783023 | GALK2 | | 2585 |
| ENSG00000166262 | 23.5547366 | 0.041755919 | 0.1421354 | 0.1872977 | NA | FAM227B | | 196951 |
| ENSG00000104047 | 141.1598401 | 0.108902076 | 0.1575655 | 0.1375832 | 0.3658342 | DTWD1 | | 56986 |
| ENSG00000261597 | 8.412842914 | 0.005662807 | 0.1352408 | 0.7853118 | NA | NA | NA | |

| | | | | | | | | |
|---|---|---|---|---|---|---|---|---|
| ENSG00000104064 | 454.2217034 | 0.018757582 | 0.095474 | 0.7845202 | 0.9098848 | GABPB1 | | 2553 |
| ENSG00000244879 | 127.6292607 | 0.103656469 | 0.1570913 | 0.1442095 | 0.3765953 | NA | NA | |
| ENSG00000138592 | 985.5786645 | -0.082031352 | 0.085318 | 0.2275544 | 0.4888067 | USP8 | | 9101 |
| ENSG00000092439 | 1275.685397 | 0.097967484 | 0.0834125 | 0.147444 | 0.3810382 | TRPM7 | | 54822 |
| ENSG00000138600 | 828.7868649 | -0.083316653 | 0.0893565 | 0.2312254 | 0.493233 | SPPL2A | | 84888 |
| ENSG00000081014 | 647.2585319 | 0.0460051 | 0.0897363 | 0.5034388 | 0.7375729 | AP4E1 | | 23431 |
| ENSG00000183578 | 8.254023133 | 0.036580383 | 0.1427441 | 0.0569945 | NA | TNFAIP8L3 | | 388121 |
| ENSG00000104093 | 819.8778088 | 0.064292864 | 0.086024 | 0.3447911 | 0.610988 | DMXL2 | | 23312 |
| ENSG00000140280 | 168.2106826 | -0.016761124 | 0.1167672 | 0.7841868 | 0.9098848 | LYSMD2 | | 256586 |
| ENSG00000128872 | 220.6842926 | -0.032281264 | 0.1122403 | 0.6255544 | 0.8205615 | TMOD2 | | 29767 |
| ENSG00000138594 | 1409.869537 | 0.097157663 | 0.0834721 | 0.1510797 | 0.3876043 | TMOD3 | | 29766 |
| ENSG00000166477 | 524.584841 | -0.233732241 | 0.1280279 | 0.011802 | 0.0714666 | LEO1 | | 123169 |
| ENSG00000069956 | 644.2786397 | -0.093773382 | 0.101982 | 0.2047268 | 0.4599758 | MAPK6 | | 5597 |
| ENSG00000069966 | 632.8753665 | -0.23438702 | 0.1198129 | 0.0091529 | 0.0600307 | GNB5 | | 10681 |
| ENSG00000128833 | 12.49687168 | -0.008915056 | 0.1349008 | 0.7070322 | NA | MYO5C | | 55930 |
| ENSG00000197535 | 1493.004532 | -0.047909613 | 0.0720861 | 0.434661 | 0.6852726 | MYO5A | | 4644 |
| ENSG00000128989 | 3741.556254 | -0.15243064 | 0.0634948 | 0.0071753 | 0.0496824 | ARPP19 | | 10776 |
| ENSG00000047346 | 121.0007072 | 0.092941097 | 0.1539483 | 0.1615808 | 0.403641 | ATOSA | | 56204 |
| ENSG00000169856 | 13.5594821 | -0.003268713 | 0.1343595 | 0.8930578 | NA | ONECUT1 | | 3175 |
| ENSG00000166415 | 72.00261929 | -0.005876797 | 0.1259186 | 0.9034453 | 0.9618565 | WDR72 | | 256764 |
| ENSG00000137766 | 30.02855878 | -0.024232933 | 0.1342974 | 0.5120065 | NA | UNC13C | | 440279 |
| ENSG00000137876 | 1796.604641 | 0.052028677 | 0.0691075 | 0.3842288 | 0.6447633 | RSL24D1 | | 51187 |
| ENSG00000069974 | 315.1567305 | 0.050800475 | 0.1119777 | 0.4670825 | 0.7094916 | RAB27A | | 5873 |
| ENSG00000225973 | 56.29669571 | -0.060594597 | 0.1442386 | 0.2241457 | 0.4847707 | PIGBOS1 | | 101928527 |
| ENSG00000069943 | 134.2530363 | -0.001382724 | 0.1174019 | 0.9804483 | 0.9927719 | PIGB | | 9488 |
| ENSG00000260916 | 119.7569733 | -0.011050829 | 0.1194838 | 0.849155 | 0.9377876 | CCPG1 | | 9236 |
| ENSG00000261652 | 11.01891997 | -0.003668038 | 0.1347536 | 0.8699624 | NA | PIERCE2 | | 145788 |
| ENSG00000171016 | 209.8408952 | 0.189043739 | 0.1853421 | 0.0483537 | 0.1884003 | PYGO1 | | 26108 |
| ENSG00000166450 | 250.4194625 | -0.022243542 | 0.1081429 | 0.7393445 | 0.8857761 | PRTG | | 283659 |
| ENSG00000069869 | 4027.439229 | -0.398369234 | 0.0737237 | 6.99E-09 | 4.37E-07 | NEDD4 | | 4734 |
| ENSG00000181827 | 611.5475186 | -0.188407268 | 0.1147186 | 0.0256038 | 0.1230675 | RFX7 | | 64864 |
| ENSG00000151575 | 63.24002896 | 0.051391274 | 0.1381865 | 0.290278 | 0.560019 | TEX9 | | 374618 |
| ENSG00000138587 | 66.69723066 | 0.081526684 | 0.1559098 | 0.1460436 | 0.3786761 | MNS1 | | 55329 |
| ENSG00000137871 | 301.0002335 | -0.01954254 | 0.104117 | 0.7728733 | 0.903811 | ZNF280D | | 54816 |
| ENSG00000140262 | 1059.101105 | 0.002389258 | 0.0783431 | 0.9711351 | 0.9885548 | TCF12 | | 6938 |
| ENSG00000247982 | 11.19068846 | 0.012135436 | 0.1354293 | 0.6019757 | NA | LINC00926 | | 283663 |
| ENSG00000255529 | 394.5964475 | -0.078983282 | 0.1105859 | 0.2839967 | 0.5531709 | POLR2M | | 81488 |
| ENSG00000166035 | 291.8965528 | -0.567779216 | 0.173176 | 6.23E-05 | 0.0011838 | LIPC | | 3990 |
| ENSG00000137845 | 2892.708061 | -0.187242619 | 0.0674595 | 0.0017518 | 0.0171672 | ADAM10 | | 102 |
| ENSG00000128923 | 524.1930471 | -0.147371385 | 0.1195964 | 0.0732983 | 0.2476708 | MINDY2 | | 54629 |
| ENSG00000157450 | 625.9090678 | 0.0054918 | 0.0878451 | 0.9371386 | 0.9756936 | RNF111 | | 54778 |
| ENSG00000137776 | 2796.717812 | -0.102390656 | 0.0667085 | 0.0792724 | 0.2605462 | SLTM | | 79811 |
| ENSG00000157456 | 1092.336425 | -0.393390432 | 0.1052399 | 1.83E-05 | 0.0004229 | CCNB2 | | 9133 |
| ENSG00000157483 | 886.4967336 | 0.015409088 | 0.0830253 | 0.8162596 | 0.9252377 | MYO1E | | 4643 |
| ENSG00000157470 | 145.6156832 | -0.010788569 | 0.1173545 | 0.859323 | 0.9433297 | FAM81A | | 145773 |
| ENSG00000140307 | 979.1583841 | -0.093791901 | 0.0894229 | 0.1800295 | 0.429519 | GTF2A2 | | 2958 |
| ENSG00000140299 | 1060.359792 | -0.225783615 | 0.0965745 | 0.0041956 | 0.0331137 | BNIP2 | | 663 |
| ENSG00000234797 | 44.06226843 | 0.011943219 | 0.1298065 | 0.7798626 | 0.9081422 | RPS3AP6 | | 145767 |
| ENSG00000182718 | 18599.68557 | 0.041542828 | 0.0524365 | 0.3933518 | 0.6540086 | ANXA2 | | 302 |
| ENSG00000128915 | 923.9095897 | 0.023206643 | 0.0823626 | 0.7251933 | 0.8766924 | ICE2 | | 79664 |
| ENSG00000129003 | 1119.314635 | -0.036893708 | 0.0766327 | 0.5610477 | 0.7795039 | VPS13C | | 54832 |
| ENSG00000171914 | 154.6730134 | 0.09475786 | 0.1440644 | 0.1879902 | 0.4389252 | TLN2 | | 83660 |
| ENSG00000259727 | 8.610085808 | -0.007736013 | 0.1353784 | 0.7058208 | NA | NA | NA | |
| ENSG00000140416 | 9028.784038 | -0.54687893 | 0.0696582 | 3.26E-16 | 6.80E-14 | TPM1 | | 7168 |
| ENSG00000259498 | 43.65564811 | -0.033716197 | 0.1344383 | 0.4359594 | 0.6863128 | NA | NA | |
| ENSG00000259627 | 47.6088667 | -0.069036061 | 0.1524975 | 0.1385754 | 0.3673017 | NA | NA | |
| ENSG00000103642 | 298.6593148 | 0.084143053 | 0.1203436 | 0.2563363 | 0.5231846 | LACTB | | 114294 |
| ENSG00000185088 | 577.6061952 | -0.102925564 | 0.1100526 | 0.1767638 | 0.4259029 | RPS27L | | 51065 |
| ENSG00000166128 | 416.9367316 | -0.141596451 | 0.12478 | 0.0869397 | 0.2767899 | RAB8B | | 51762 |
| ENSG00000138613 | 103.7703664 | 0.040234831 | 0.1269988 | 0.489303 | 0.7263574 | APH1B | | 83464 |
| ENSG00000140455 | 317.4765679 | -0.011543737 | 0.1025766 | 0.8640653 | 0.9455189 | USP3 | | 9960 |
| ENSG00000103657 | 917.3470148 | 0.010522529 | 0.0795257 | 0.8715975 | 0.9483169 | HERC1 | | 8925 |
| ENSG00000035664 | 18.13197266 | -0.010819842 | 0.1340633 | 0.7051232 | NA | DAPK2 | | 23604 |
| ENSG00000166797 | 535.1983114 | -0.021130074 | 0.0929477 | 0.7564699 | 0.8951803 | CIAO2A | | 84191 |
| ENSG00000028528 | 1350.582491 | -0.207551528 | 0.0876795 | 0.004596 | 0.035601 | SNX1 | | 6642 |
| ENSG00000157734 | 169.3348047 | -0.065803206 | 0.129806 | 0.3140535 | 0.5827728 | SNX22 | | 79856 |
| ENSG00000166794 | 4012.275994 | -0.219481035 | 0.0687599 | 0.0003587 | 0.0050002 | PPIB | | 5479 |
| ENSG00000169118 | 447.6253799 | -0.065622771 | 0.1039577 | 0.3615528 | 0.625071 | CSNK1G1 | | 53944 |
| ENSG00000166803 | 994.9493257 | 0.073407829 | 0.082911 | 0.2741456 | 0.5425779 | PCLAF | | 9768 |
| ENSG00000103671 | 428.7028431 | 0.034367531 | 0.0987226 | 0.6206924 | 0.818389 | TRIP4 | | 9325 |

| | | | | | | | |
|---|---|---|---|---|---|---|---|
| ENSG00000180357 | 1170.30896 | -0.111027918 | 0.0871022 | 0.110043 | 0.3212914 | ZNF609 | 23060 |
| ENSG00000180304 | 853.839101 | -0.143727318 | 0.1020508 | 0.0616295 | 0.2215059 | OAZ2 | 4947 |
| ENSG00000166831 | 188.8612899 | 0.085753021 | 0.136199 | 0.229231 | 0.4903399 | RBPMS2 | 348093 |
| ENSG00000140451 | 305.4081656 | -0.783060553 | 0.1692547 | 2.03E-07 | 8.64E-06 | PIF1 | 80119 |
| ENSG00000241839 | 130.0910829 | 0.04352335 | 0.12464 | 0.4826321 | 0.7213852 | PLEKHO2 | 80301 |
| ENSG00000166839 | 35.47336495 | 0.048910229 | 0.1422392 | 0.2345097 | NA | ANKDD1A | 348094 |
| ENSG00000090487 | 835.9276842 | 0.01062172 | 0.0820974 | 0.872308 | 0.9484107 | SPG21 | 51324 |
| ENSG00000103707 | 237.2135622 | -0.010731492 | 0.108691 | 0.8705193 | 0.9478556 | MTFMT | 123263 |
| ENSG00000246922 | 20.284041 | -0.018470622 | 0.1350576 | 0.5317685 | NA | UBAP1L | 390595 |
| ENSG00000090470 | 252.8180989 | 0.010094134 | 0.1071115 | 0.8788476 | 0.9512796 | PDCD7 | 10081 |
| ENSG00000166855 | 804.1601344 | -0.092770556 | 0.0939263 | 0.1949257 | 0.4471577 | CLPX | 10845 |
| ENSG00000138617 | 129.6765264 | 0.021527793 | 0.1202275 | 0.7188365 | 0.8729316 | PARP16 | 54956 |
| ENSG00000074603 | 716.1477051 | -0.061003782 | 0.0897822 | 0.3764811 | 0.6384547 | DPP8 | 54878 |
| ENSG00000074696 | 2276.534609 | 0.095966659 | 0.0674753 | 0.1028912 | 0.3074144 | HACD3 | 51495 |
| ENSG00000138614 | 504.6516034 | 0.005694012 | 0.0924098 | 0.9346648 | 0.9750297 | INTS14 | 81556 |
| ENSG00000074621 | 57.40543484 | 0.034368142 | 0.1323087 | 0.4744845 | 0.7142337 | SLC24A1 | 9187 |
| ENSG00000174485 | 481.083735 | 0.091852273 | 0.1118389 | 0.2238579 | 0.4842378 | DENND4A | 10260 |
| ENSG00000103769 | 1417.378488 | -0.067035869 | 0.0748395 | 0.2869306 | 0.5568427 | RAB11A | 8766 |
| ENSG00000166938 | 300.1414983 | -0.103174232 | 0.1252212 | 0.1822737 | 0.4318741 | DIS3L | 115752 |
| ENSG00000075131 | 433.4452553 | 0.172239894 | 0.1359286 | 0.0514761 | 0.1959247 | TIPIN | 54962 |
| ENSG00000169032 | 1013.320386 | 0.103825438 | 0.0872105 | 0.1359822 | 0.3630647 | MAP2K1 | 5604 |
| ENSG00000261351 | 15.22297765 | -0.013066199 | 0.1348923 | 0.6193782 | NA | NA | NA |
| ENSG00000174446 | 243.103767 | 0.072364249 | 0.1212513 | 0.3318705 | 0.5992396 | SNAPC5 | 10302 |
| ENSG00000174444 | 16300.21112 | -0.120388518 | 0.0560059 | 0.0184063 | 0.0978625 | RPL4 | 6124 |
| ENSG00000174442 | 1050.926244 | -0.0897842 | 0.0853355 | 0.1868705 | 0.4377049 | ZWILCH | 55055 |
| ENSG00000188501 | 14.32807072 | 0.005611217 | 0.1343516 | 0.8280504 | NA | LCTL | 197021 |
| ENSG00000137834 | 115.5267873 | 0.007476856 | 0.1206746 | 0.8968134 | 0.9588721 | SMAD6 | 4091 |
| ENSG00000166949 | 5538.705944 | -0.087186564 | 0.0595667 | 0.1031351 | 0.3079552 | SMAD3 | 4088 |
| ENSG00000103591 | 1159.936806 | 0.033873969 | 0.0781217 | 0.5834368 | 0.7949132 | AAGAB | 79719 |
| ENSG00000103599 | 21.90215795 | -0.000402605 | 0.1328377 | 0.9875087 | NA | IQCH | 64799 |
| ENSG00000259673 | 35.05389092 | -0.069288637 | 0.1552787 | 0.1004074 | NA | IQCH-AS1 | 100506686 |
| ENSG00000189227 | 73.09784337 | 0.137520948 | 0.216154 | 0.0467823 | 0.1849913 | C15orf61 | 145853 |
| ENSG00000137764 | 134.8968943 | 0.028710643 | 0.1202617 | 0.6387094 | 0.829896 | MAP2K5 | 5607 |
| ENSG00000033800 | 297.7534837 | -0.009470542 | 0.1034306 | 0.8877144 | 0.9545517 | PIAS1 | 8554 |
| ENSG00000129007 | 124.631607 | -0.051191849 | 0.1280558 | 0.4077374 | 0.6640358 | CALML4 | 91860 |
| ENSG00000128973 | 1039.158525 | 0.015240199 | 0.0773331 | 0.8119252 | 0.9224879 | CLN6 | 54982 |
| ENSG00000169018 | 641.0648143 | -0.004189262 | 0.0894439 | 0.9484302 | 0.9796055 | FEM1B | 10116 |
| ENSG00000140350 | 2379.508397 | -0.265469654 | 0.0804856 | 0.0001781 | 0.0027809 | ANP32A | 8125 |
| ENSG00000138604 | 491.2966847 | -0.052785616 | 0.1030729 | 0.4551809 | 0.7001538 | GLCE | 26035 |
| ENSG00000259426 | 21.1297991 | 1.142352938 | 0.8664227 | 0.0038581 | NA | KIF23-AS1 | 145694 |
| ENSG00000137807 | 2470.110343 | -0.207757936 | 0.076985 | 0.0018799 | 0.0180735 | KIF23 | 9493 |
| ENSG00000137818 | 8513.331286 | -0.040103065 | 0.0532293 | 0.4122802 | 0.6663685 | RPLP1 | 6176 |
| ENSG00000140332 | 363.4499597 | 0.075589742 | 0.1102903 | 0.3037693 | 0.5728743 | TLE3 | 7090 |
| ENSG00000137831 | 800.6790215 | 0.072692556 | 0.0923968 | 0.3018653 | 0.571119 | UACA | 55075 |
| ENSG00000166173 | 285.0243754 | 0.393322073 | 0.1838471 | 0.0023886 | 0.0216717 | LARP6 | 55323 |
| ENSG00000137821 | 114.9475338 | -0.119859626 | 0.1736221 | 0.1009248 | 0.3037626 | LRRC49 | 54839 |
| ENSG00000129028 | 40.33846354 | 0.089426132 | 0.1716705 | 0.0573124 | NA | THAP10 | 56906 |
| ENSG00000187720 | 1228.611873 | -0.010470239 | 0.0750984 | 0.8667652 | 0.9461922 | THSD4 | 79875 |
| ENSG00000225362 | 16.72226101 | 0.023121835 | 0.136389 | 0.4178783 | NA | CT62 | 196993 |
| ENSG00000259781 | 49.81810412 | -0.106368827 | 0.1854337 | 0.0543591 | 0.2041044 | NA | NA |
| ENSG00000066933 | 692.554671 | -0.076145587 | 0.0966335 | 0.2816691 | 0.5508784 | MYO9A | 4649 |
| ENSG00000166192 | 31.18000447 | 0.015787167 | 0.1324168 | 0.6706921 | NA | SENP8 | 123228 |
| ENSG00000067225 | 31185.61341 | -0.106955665 | 0.0491193 | 0.0186751 | 0.0991115 | PKM | 5315 |
| ENSG00000137817 | 503.985166 | 0.214235003 | 0.1301325 | 0.0195643 | 0.101977 | PARP6 | 56965 |
| ENSG00000213614 | 79.55052893 | 0.030845518 | 0.1280337 | 0.5620775 | 0.7800541 | HEXA | 3073 |
| ENSG00000261423 | 28.59899221 | -0.023526437 | 0.1344889 | 0.5063327 | NA | TMEM202-A | 105370888 |
| ENSG00000166233 | 852.6306319 | -0.09802446 | 0.0901324 | 0.1634112 | 0.4061289 | ARIH1 | 25820 |
| ENSG00000260534 | 18.08924024 | -0.013022296 | 0.1342426 | 0.6537886 | NA | NA | NA |
| ENSG00000140463 | 252.6743079 | 0.084793709 | 0.1256687 | 0.2702597 | 0.5381601 | BBS4 | 585 |
| ENSG00000159322 | 885.9747912 | -0.019771296 | 0.0806798 | 0.763213 | 0.8982943 | ADPGK | 83440 |
| ENSG00000067141 | 642.2736673 | 0.003283122 | 0.0889603 | 0.9631748 | 0.9851357 | NEO1 | 4756 |
| ENSG00000156642 | 951.7747462 | -0.060910962 | 0.0824681 | 0.3556543 | 0.6192724 | NPTN | 27020 |
| ENSG00000103855 | 498.990214 | -0.202149338 | 0.1335875 | 0.0266847 | 0.1269671 | CD276 | 80381 |
| ENSG00000067221 | 50.51712263 | 0.096577293 | 0.1741792 | 0.0731553 | 0.2472589 | STOML1 | 9399 |
| ENSG00000140464 | 389.2758683 | 0.11961766 | 0.1216161 | 0.1342351 | 0.3608746 | PML | 5371 |
| ENSG00000138623 | 29.15151684 | 4.77E-05 | 0.1315525 | 0.9990521 | NA | SEMA7A | 8482 |
| ENSG00000138629 | 438.340093 | 0.037106176 | 0.0976088 | 0.5939991 | 0.802696 | UBL7 | 84993 |
| ENSG00000247240 | 36.05881547 | -0.02264432 | 0.1328246 | 0.565356 | NA | UBL7-DT | 440288 |
| ENSG00000179361 | 229.5955766 | -0.013771868 | 0.1096875 | 0.8347739 | 0.9310603 | ARID3B | 10620 |
| ENSG00000179335 | 579.6969724 | 0.014870818 | 0.0912481 | 0.8279699 | 0.9290601 | CLK3 | 1198 |

| | | | | | | | | |
|---|---|---|---|---|---|---|---|---|
| ENSG00000179151 | 639.4017001 | 0.049675951 | 0.0909986 | 0.4741088 | 0.7140485 | EDC3 | | 80153 |
| ENSG00000103653 | 1360.259907 | 0.017526938 | 0.07678 | 0.7836008 | 0.9098649 | CSK | | 1445 |
| ENSG00000140474 | 598.0584489 | 0.023891465 | 0.089251 | 0.7229093 | 0.8749667 | ULK3 | | 25989 |
| ENSG00000140497 | 751.9272413 | 0.01622208 | 0.0842709 | 0.8078333 | 0.9200093 | SCAMP2 | | 10066 |
| ENSG00000178802 | 196.9247717 | 0.044893722 | 0.1202437 | 0.4931736 | 0.7288856 | MPI | | 4351 |
| ENSG00000178761 | 512.9695819 | 0.053361878 | 0.0984701 | 0.4505662 | 0.6973716 | FAM219B | | 57184 |
| ENSG00000178741 | 1149.216646 | -0.162960994 | 0.0895555 | 0.024459 | 0.1189447 | COX5A | | 9377 |
| ENSG00000178718 | 286.445616 | 0.32114372 | 0.1795243 | 0.006627 | 0.0469722 | RPP25 | | 54913 |
| ENSG00000198794 | 166.7608418 | 0.078118758 | 0.1333946 | 0.2573285 | 0.52429 | SCAMP5 | | 192683 |
| ENSG00000138621 | 322.6397184 | 0.531608359 | 0.1639926 | 7.72E-05 | 0.001412 | PPCDC | | 60490 |
| ENSG00000167173 | 253.4041229 | 0.172528726 | 0.1628905 | 0.0603647 | 0.2188456 | C15orf39 | | 56905 |
| ENSG00000140365 | 862.0815842 | 0.058014414 | 0.0854994 | 0.394417 | 0.6549057 | COMMD4 | | 54939 |
| ENSG00000140398 | 20.78620779 | 0.019627059 | 0.1352866 | 0.5103759 | NA | NEIL1 | | 79661 |
| ENSG00000140400 | 323.8111132 | -0.187919166 | 0.151428 | 0.0432758 | 0.176134 | MAN2C1 | | 4123 |
| ENSG00000260274 | 17.45355534 | 0.029479621 | 0.1381265 | 0.3055147 | NA | NA | NA | |
| ENSG00000169375 | 1282.109193 | -0.17314557 | 0.0862964 | 0.0149833 | 0.0841038 | SIN3A | | 25942 |
| ENSG00000169410 | 551.1730068 | 0.070382537 | 0.0980604 | 0.3310633 | 0.5989207 | PTPN9 | | 5780 |
| ENSG00000169371 | 265.8765629 | -0.107515843 | 0.133635 | 0.1673132 | 0.4122331 | SNUPN | | 10073 |
| ENSG00000177971 | 633.4330503 | -0.075402374 | 0.096182 | 0.2909145 | 0.5608471 | IMP3 | | 55272 |
| ENSG00000173548 | 239.0787417 | -0.042208395 | 0.1141652 | 0.53084 | 0.756397 | SNX33 | | 257364 |
| ENSG00000140367 | 493.1657013 | -0.027155746 | 0.0940563 | 0.6916786 | 0.8594028 | UBE2Q2 | | 92912 |
| ENSG00000167196 | 541.7501948 | 0.052205882 | 0.0945516 | 0.4534852 | 0.6987417 | FBXO22 | | 26263 |
| ENSG00000140374 | 1292.164691 | -0.174361731 | 0.0869298 | 0.0148258 | 0.0835407 | ETFA | | 2108 |
| ENSG00000140386 | 212.7183462 | -0.010603471 | 0.1106359 | 0.8695747 | 0.9475003 | SCAPER | | 49855 |
| ENSG00000117906 | 1084.442163 | -0.282937449 | 0.0950444 | 0.0004552 | 0.0059537 | RCN2 | | 5955 |
| ENSG00000140391 | 1101.027616 | 0.142454803 | 0.0919062 | 0.0502305 | 0.192944 | TSPAN3 | | 10099 |
| ENSG00000269951 | 9.821954972 | -0.008476679 | 0.1352132 | 0.6976901 | NA | NA | NA | |
| ENSG00000173517 | 932.2797699 | 0.078581047 | 0.0945942 | 0.269529 | 0.537356 | PEAK1 | | 79834 |
| ENSG00000140382 | 669.8231926 | 0.022365577 | 0.0884037 | 0.7416979 | 0.887242 | HMG20A | | 10363 |
| ENSG00000167202 | 794.0895968 | -0.058405717 | 0.0861926 | 0.3884408 | 0.6487996 | TBC1D2B | | 23102 |
| ENSG00000136425 | 283.3548648 | -0.040836954 | 0.109572 | 0.5515948 | 0.7725183 | CIB2 | | 10518 |
| ENSG00000166411 | 1331.369858 | -0.090509874 | 0.0775656 | 0.1611018 | 0.4030478 | IDH3A | | 3419 |
| ENSG00000103740 | 23.71340707 | 0.001564737 | 0.1324225 | 0.9640988 | NA | ACSBG1 | | 23205 |
| ENSG00000140395 | 657.0085471 | -0.038245429 | 0.088445 | 0.5701995 | 0.7856217 | SKIC8 | | 80349 |
| ENSG00000259562 | 14.00573331 | 0.036767514 | 0.1419262 | 0.1252354 | NA | NA | NA | |
| ENSG00000136381 | 1040.741376 | -0.160181766 | 0.0908343 | 0.0282049 | 0.132294 | IREB2 | | 3658 |
| ENSG00000188266 | 22.30983674 | 0.012363801 | 0.1337576 | 0.6901696 | NA | HYKK | | 123688 |
| ENSG00000041357 | 3182.131368 | -0.31122319 | 0.0709505 | 1.72E-06 | 5.38E-05 | PSMA4 | | 5685 |
| ENSG00000169684 | 414.030262 | -0.046008186 | 0.1017628 | 0.511555 | 0.7421105 | CHRNA5 | | 1138 |
| ENSG00000261762 | 22.78399272 | -0.019962915 | 0.1345888 | 0.5379543 | NA | NA | NA | |
| ENSG00000136378 | 108.3118595 | -0.098625971 | 0.1577529 | 0.1444549 | 0.3768151 | ADAMTS7 | | 11173 |
| ENSG00000185787 | 3701.410831 | -0.375880301 | 0.0656093 | 1.19E-09 | 8.57E-08 | MORF4L1 | | 10933 |
| ENSG00000103811 | 143.2188294 | -0.030348273 | 0.1193674 | 0.6246509 | 0.8202204 | CTSH | | 1512 |
| ENSG00000166557 | 1193.685741 | 0.117813107 | 0.0837261 | 0.0849934 | 0.2727493 | TMED3 | | 23423 |
| ENSG00000261712 | 11.50219532 | -0.008236609 | 0.1349618 | 0.722064 | NA | NA | NA | |
| ENSG00000169330 | 31.09844795 | -0.004399613 | 0.131618 | 0.9001298 | NA | MINAR1 | | 23251 |
| ENSG00000136371 | 45.87954664 | -0.023931622 | 0.1317968 | 0.5747657 | 0.7887497 | MTHFS | | 10588 |
| ENSG00000180953 | 22.62508657 | 0.055131459 | 0.1486538 | 0.1061159 | NA | ST20 | | 400410 |
| ENSG00000086666 | 571.0260085 | -0.131229535 | 0.1184327 | 0.1026492 | 0.3071201 | ZFAND6 | | 54469 |
| ENSG00000103876 | 131.7809499 | -0.0008643 | 0.1181745 | 0.9871877 | 0.994767 | FAH | | 2184 |
| ENSG00000172379 | 420.3312472 | 0.020272184 | 0.0974762 | 0.7682165 | 0.9005958 | ARNT2 | | 9915 |
| ENSG00000136379 | 141.8848266 | -0.004381718 | 0.1166125 | 0.9414676 | 0.9771609 | ABHD17C | | 58489 |
| ENSG00000117899 | 818.0650747 | -0.187781881 | 0.1018235 | 0.0179447 | 0.0960131 | MESD | | 23184 |
| ENSG00000140406 | 540.8448164 | -0.055326185 | 0.0972326 | 0.4320159 | 0.6832356 | TLNRD1 | | 59274 |
| ENSG00000172345 | 27.92040095 | -0.028966351 | 0.1365166 | 0.3921207 | NA | STARD5 | | 80765 |
| ENSG00000183496 | 65.99777527 | 0.033609066 | 0.1311389 | 0.4999235 | 0.734688 | MEX3B | | 84206 |
| ENSG00000140598 | 550.371961 | 0.194571231 | 0.1246123 | 0.0273822 | 0.1292816 | EFL1 | | 79631 |
| ENSG00000188659 | 16.24177254 | -0.010987647 | 0.1343625 | 0.6888009 | NA | SAXO2 | | 283726 |
| ENSG00000237550 | 11.60735716 | -0.004795998 | 0.1346226 | 0.8383461 | NA | RPL9P8 | | 254948 |
| ENSG00000255769 | 8.747496196 | -0.020213146 | 0.1372515 | 0.3309164 | NA | NA | NA | |
| ENSG00000184779 | 40.92057565 | -0.097521993 | 0.1812528 | 0.0396997 | 0.1669303 | NA | NA | |
| ENSG00000259328 | 16.90004997 | -0.021506618 | 0.136303 | 0.4252656 | NA | NA | NA | |
| ENSG00000182774 | 287.2536977 | -0.121904234 | 0.1412489 | 0.1299173 | 0.3544053 | RPS17 | | 6218 |
| ENSG00000250988 | 15.86549103 | -0.007983475 | 0.134086 | 0.7691927 | NA | SNHG21 | | 100505616 |
| ENSG00000252690 | 23.70109119 | -0.010267737 | 0.1329402 | 0.7523017 | NA | NA | NA | |
| ENSG00000156232 | 451.3872584 | 0.207720973 | 0.1491728 | 0.0298259 | 0.1378149 | WHAMM | | 123720 |
| ENSG00000103942 | 470.563487 | 0.05717909 | 0.1001599 | 0.422313 | 0.6742808 | HOMER2 | | 9455 |
| ENSG00000169612 | 304.2468931 | 0.026143421 | 0.104875 | 0.703059 | 0.8651804 | RAMAC | | 83640 |
| ENSG00000169609 | 194.1767936 | 0.04455193 | 0.1179372 | 0.5040384 | 0.7381071 | C15orf40 | | 123207 |
| ENSG00000064726 | 1897.269634 | -0.063278604 | 0.0695452 | 0.2901789 | 0.560019 | BTBD1 | | 53339 |

| | | | | | | | | |
|---|---|---|---|---|---|---|---|---|
| ENSG00000260579 | 9.024331537 | 0.021552643 | 0.1375822 | 0.2983461 | NA | NA | NA | |
| ENSG00000166503 | 649.5675799 | -0.107014806 | 0.1045348 | 0.1565868 | 0.3956066 | HDGFL3 | | 50810 |
| ENSG00000169594 | 559.1387347 | 0.486740392 | 0.1266132 | 9.27E-06 | 0.0002371 | BNC1 | | 646 |
| ENSG00000259726 | 23.47316283 | -0.014844878 | 0.1336183 | 0.6471703 | NA | NA | NA | |
| ENSG00000225151 | 61.82362091 | 0.015975081 | 0.1274666 | 0.7444842 | 0.8889216 | NA | NA | |
| ENSG00000184206 | 12.11515205 | -0.005115036 | 0.1345557 | 0.8309133 | NA | GOLGA6L4 | | 643707 |
| ENSG00000230373 | 40.91257891 | 0.02582965 | 0.1328368 | 0.5355078 | NA | GOLGA6L17 | | 642402 |
| ENSG00000189136 | 29.47168419 | 0.021212617 | 0.1337977 | 0.556501 | NA | NA | NA | |
| ENSG00000176371 | 98.8745651 | -0.035326312 | 0.1270573 | 0.5289207 | 0.7551115 | ZSCAN2 | | 54993 |
| ENSG00000176700 | 82.53093203 | 0.070364757 | 0.1442397 | 0.2279657 | 0.4890685 | NA | NA | |
| ENSG00000177082 | 484.6112349 | 0.029086242 | 0.0945298 | 0.6750678 | 0.8507355 | WDR73 | | 84942 |
| ENSG00000197696 | 179.7697508 | 0.057715291 | 0.1242956 | 0.3885906 | 0.6488607 | NMB | | 4828 |
| ENSG00000140612 | 1450.453503 | -0.163060949 | 0.0823271 | 0.0176634 | 0.0949934 | SEC11A | | 23478 |
| ENSG00000166716 | 964.2162884 | -0.035876104 | 0.0831288 | 0.5872673 | 0.7976592 | ZNF592 | | 9640 |
| ENSG00000136383 | 11.18478282 | 0.006753151 | 0.134812 | 0.7760388 | NA | ALPK3 | | 57538 |
| ENSG00000073417 | 688.6247432 | 0.067831387 | 0.0913951 | 0.341702 | 0.6087837 | PDE8A | | 5151 |
| ENSG00000229212 | 99.79129225 | 0.05713162 | 0.1347598 | 0.3325664 | 0.5998623 | NA | NA | |
| ENSG00000170776 | 1755.423105 | 0.172489498 | 0.0778353 | 0.0094217 | 0.0614142 | AKAP13 | | 11214 |
| ENSG00000259375 | 9.291890804 | 0.025517937 | 0.1384822 | 0.2366783 | NA | NA | NA | |
| ENSG00000259407 | 9.016697499 | 0.020352214 | 0.1373209 | 0.3240496 | NA | NA | NA | |
| ENSG00000183655 | 105.8002838 | 0.024731545 | 0.124425 | 0.6580869 | 0.8423052 | KLHL25 | | 64410 |
| ENSG00000259494 | 130.17534 | 0.065731973 | 0.1328153 | 0.3118438 | 0.5816138 | MRPL46 | | 26589 |
| ENSG00000181991 | 550.6456877 | 0.035309815 | 0.0921678 | 0.6081927 | 0.8121054 | MRPS11 | | 64963 |
| ENSG00000140543 | 32.31638807 | 0.02660207 | 0.1347686 | 0.4747713 | NA | DET1 | | 55070 |
| ENSG00000181026 | 1765.756431 | 0.506401428 | 0.080751 | 2.99E-11 | 2.86E-09 | AEN | | 64782 |
| ENSG00000172183 | 33.07663084 | 0.031741509 | 0.1367898 | 0.3763436 | NA | ISG20 | | 3669 |
| ENSG00000140511 | 130.4784196 | -0.184686706 | 0.2344208 | 0.043712 | 0.1773534 | HAPLN3 | | 145864 |
| ENSG00000140545 | 1876.685839 | -0.06636235 | 0.1283707 | 0.3282567 | 0.5964172 | MFGE8 | | 4240 |
| ENSG00000140526 | 1421.260353 | -0.106214567 | 0.0787638 | 0.1049121 | 0.3113474 | ABHD2 | | 11057 |
| ENSG00000140525 | 2681.591745 | 0.039899639 | 0.0625307 | 0.4733808 | 0.7137681 | FANCI | | 55215 |
| ENSG00000140521 | 2121.929525 | -0.008891413 | 0.0653399 | 0.8761669 | 0.949742 | POLG | | 5428 |
| ENSG00000140534 | 1013.918847 | 0.073955994 | 0.0836602 | 0.2750733 | 0.5434931 | TICRR | | 90381 |
| ENSG00000166813 | 293.7404425 | 0.037725697 | 0.1072565 | 0.5845199 | 0.7956855 | KIF7 | | 374654 |
| ENSG00000166821 | 49.58320042 | -0.001808286 | 0.1292202 | 0.9625757 | 0.98492 | PEX11A | | 8800 |
| ENSG00000166823 | 20.0573044 | 0.045546831 | 0.144156 | 0.1427149 | NA | MESP1 | | 55897 |
| ENSG00000157823 | 116.0353052 | -0.046798447 | 0.1273025 | 0.4400163 | 0.6891701 | AP3S2 | | 10239 |
| ENSG00000242498 | 28.37096886 | -0.008408753 | 0.1320556 | 0.8093814 | NA | ARPIN | | 348110 |
| ENSG00000140548 | 153.0645178 | 0.651941081 | 0.2591741 | 0.0005532 | 0.0069479 | ZNF710 | | 374655 |
| ENSG00000182054 | 336.4997364 | 0.478200252 | 0.172129 | 0.0003612 | 0.0050234 | IDH2 | | 3418 |
| ENSG00000185033 | 1798.510718 | 0.135807506 | 0.0775713 | 0.0378377 | 0.1614253 | SEMA4B | | 10509 |
| ENSG00000185043 | 1147.877056 | 0.042056323 | 0.0822848 | 0.5247781 | 0.752139 | CIB1 | | 10519 |
| ENSG00000183208 | 48.94826318 | 0.006958879 | 0.1289489 | 0.8744194 | 0.9491769 | GDPGP1 | | 390637 |
| ENSG00000182768 | 496.1678677 | 0.06985691 | 0.1007057 | 0.3336547 | 0.601084 | NGRN | | 51335 |
| ENSG00000228998 | 133.4646631 | -0.085280139 | 0.1427997 | 0.21031 | 0.4667738 | NA | NA | |
| ENSG00000196391 | 23.67870968 | 0.001660322 | 0.1332627 | 0.9575956 | NA | ZNF774 | | 342132 |
| ENSG00000140575 | 7630.002976 | -0.202823848 | 0.0590026 | 0.0001769 | 0.0027668 | IQGAP1 | | 8826 |
| ENSG00000140577 | 547.9102144 | -0.148039636 | 0.1147321 | 0.0684268 | 0.2369682 | CRTC3 | | 64784 |
| ENSG00000197299 | 657.6413328 | 0.038776034 | 0.0884043 | 0.5690831 | 0.7847255 | BLM | | 641 |
| ENSG00000140564 | 1520.880386 | -0.045197234 | 0.1182157 | 0.5021635 | 0.7365601 | FURIN | | 5045 |
| ENSG00000196547 | 1145.825891 | -0.077661707 | 0.0820594 | 0.2427798 | 0.5085703 | MAN2A2 | | 4122 |
| ENSG00000259661 | 18.45147747 | -0.022429973 | 0.1361048 | 0.4365208 | NA | NA | NA | |
| ENSG00000140553 | 2097.35732 | -0.08350601 | 0.0703405 | 0.1672229 | 0.4122331 | UNC45A | | 55898 |
| ENSG00000184508 | 64.41055482 | 0.069194657 | 0.1494954 | 0.176293 | 0.4252072 | HDDC3 | | 374659 |
| ENSG00000166965 | 361.3213155 | -0.082567897 | 0.112754 | 0.2652569 | 0.5328402 | RCCD1 | | 91433 |
| ENSG00000198901 | 3855.061858 | -0.265696999 | 0.0664416 | 1.21E-05 | 0.0002975 | PRC1 | | 9055 |
| ENSG00000258725 | 56.49995647 | -0.042852108 | 0.1364951 | 0.3568284 | 0.6201207 | PRC1-AS1 | | 100507118 |
| ENSG00000184056 | 171.3540053 | 0.025700805 | 0.118244 | 0.6799669 | 0.853781 | VPS33B | | 26276 |
| ENSG00000176463 | 208.2679596 | -0.026681208 | 0.1129625 | 0.6829343 | 0.8544725 | SLCO3A1 | | 28232 |
| ENSG00000185442 | 9.758066077 | 0.021437042 | 0.1375272 | 0.3054123 | NA | FAM174B | | 400451 |
| ENSG00000272888 | 365.9277974 | 0.322720497 | 0.1563258 | 0.003903 | 0.0312907 | NA | NA | |
| ENSG00000173575 | 2583.379676 | 0.337917813 | 0.0730584 | 4.97E-07 | 1.87E-05 | CHD2 | | 1106 |
| ENSG00000185551 | 298.3074898 | 0.053096742 | 0.1117036 | 0.450106 | 0.6970288 | NR2F2 | | 7026 |
| ENSG00000140450 | 50.32185244 | 0.08847669 | 0.1678055 | 0.0801069 | 0.2623293 | ARRDC4 | | 91947 |
| ENSG00000140443 | 629.8621577 | 0.176736729 | 0.1152699 | 0.0351586 | 0.1532989 | IGF1R | | 3480 |
| ENSG00000182253 | 157.2565079 | -0.102914136 | 0.1483816 | 0.1620609 | 0.4039797 | SYNM | | 23336 |
| ENSG00000103852 | 177.1301103 | 0.050689333 | 0.1217492 | 0.443883 | 0.6921329 | TTC23 | | 64927 |
| ENSG00000168904 | 202.5073325 | -0.13026785 | 0.1547926 | 0.1099653 | 0.321287 | LRRC28 | | 123355 |
| ENSG00000068305 | 774.2505038 | 0.091112441 | 0.0949354 | 0.2056923 | 0.4607272 | MEF2A | | 4205 |
| ENSG00000183060 | 158.2634717 | -0.061902589 | 0.1283173 | 0.3478058 | 0.6139822 | LYSMD4 | | 145748 |
| ENSG00000259363 | 20.42593464 | 0.000337142 | 0.1330867 | 0.9933101 | NA | NA | NA | |

| | | | | | | | | |
|---|---|---|---|---|---|---|---|---|
| ENSG00000270127 | 10.51556262 | 0.015112034 | 0.1359427 | 0.5079727 | NA | NA | NA | |
| ENSG00000140471 | 250.811772 | 0.20622448 | 0.1774213 | 0.037132 | 0.1592871 | LINS1 | | 55180 |
| ENSG00000183475 | 335.1325267 | -0.028467853 | 0.102964 | 0.6782394 | 0.8532595 | ASB7 | | 140460 |
| ENSG00000232386 | 17.15064601 | 0.039393678 | 0.1417791 | 0.1777219 | NA | NA | NA | |
| ENSG00000272808 | 23.28364897 | -0.020392521 | 0.1348695 | 0.5206363 | NA | GCAWKR | | 105369201 |
| ENSG00000184254 | 2018.372067 | 0.274746205 | 0.0962881 | 0.0007057 | 0.0084811 | ALDH1A3 | | 220 |
| ENSG00000259583 | 70.28485758 | 0.033337528 | 0.1300587 | 0.5172332 | 0.7465465 | ALDH1A3-A | | 101927751 |
| ENSG00000154237 | 294.509421 | -0.056510251 | 0.110775 | 0.4222422 | 0.6742808 | LRRK1 | | 79705 |
| ENSG00000131873 | 423.2719442 | -0.151798786 | 0.1285108 | 0.0729879 | 0.2469116 | CHSY1 | | 22856 |
| ENSG00000131871 | 519.8505376 | 0.009231429 | 0.0915853 | 0.8931146 | 0.956866 | SELENOS | | 55829 |
| ENSG00000131876 | 1278.35928 | -0.124918909 | 0.0819857 | 0.0640697 | 0.2277105 | SNRPA1 | | 6627 |
| ENSG00000184277 | 354.2271243 | 0.078791029 | 0.1119029 | 0.2870066 | 0.5568977 | TM2D3 | | 80213 |
| ENSG00000185418 | 148.4588352 | -0.138328649 | 0.1774768 | 0.0871453 | 0.2770669 | TARS3 | | 123283 |
| ENSG00000259658 | 9.13279348 | 0.007566718 | 0.1352436 | 0.7257625 | NA | NA | NA | |
| ENSG00000185596 | 455.6728827 | 0.082622075 | 0.1115958 | 0.2660718 | 0.5340997 | NA | NA | |
| ENSG00000234769 | 182.6385896 | -0.005859474 | 0.1137498 | 0.9251683 | 0.9710568 | NA | NA | |
| ENSG00000161980 | 312.9121938 | 0.06070847 | 0.11077 | 0.3967304 | 0.6566825 | POLR3K | | 51728 |
| ENSG00000161981 | 503.8445978 | -0.063686289 | 0.0993678 | 0.3712217 | 0.6334284 | SNRNP25 | | 79622 |
| ENSG00000007384 | 211.352703 | 0.020195768 | 0.1122789 | 0.7575256 | 0.8960665 | RHBDF1 | | 64285 |
| ENSG00000103152 | 962.9197489 | 0.000267772 | 0.0791747 | 0.9975144 | 0.998947 | MPG | | 4350 |
| ENSG00000103148 | 1130.808563 | 0.055324262 | 0.0821777 | 0.4042582 | 0.6612758 | NPRL3 | | 8131 |
| ENSG00000007392 | 1239.179476 | 0.03364165 | 0.0767305 | 0.5977082 | 0.8055647 | LUC7L | | 55692 |
| ENSG00000167930 | 373.0704375 | 0.180954019 | 0.1497743 | 0.049017 | 0.1900324 | FAM234A | | 83986 |
| ENSG00000076344 | 22.5419251 | -0.011285041 | 0.1332706 | 0.7228739 | NA | RGS11 | | 8786 |
| ENSG00000103126 | 1119.75356 | 0.021087071 | 0.0817429 | 0.7487514 | 0.8910111 | AXIN1 | | 8312 |
| ENSG00000086504 | 2023.744842 | -0.090845391 | 0.0707443 | 0.1347322 | 0.3614858 | MRPL28 | | 10573 |
| ENSG00000129925 | 1716.499325 | 0.024052525 | 0.0689023 | 0.6868634 | 0.857187 | PGAP6 | | 58986 |
| ENSG00000236829 | 10.94133346 | -0.014031898 | 0.1356691 | 0.5444171 | NA | RPL23AP5 | | 729480 |
| ENSG00000103202 | 1611.800176 | -0.146407308 | 0.0782311 | 0.0264092 | 0.1259641 | NME4 | | 4833 |
| ENSG00000242612 | 249.3759939 | -0.094357893 | 0.1280044 | 0.2108229 | 0.4674681 | DECR2 | | 26063 |
| ENSG00000090565 | 1292.589436 | 0.006608841 | 0.072997 | 0.9155724 | 0.9664559 | RAB11FIP3 | | 9727 |
| ENSG00000103326 | 2710.689841 | -0.05534067 | 0.0656799 | 0.3360462 | 0.6036223 | CAPN15 | | 6650 |
| ENSG00000266124 | 20.04332925 | -0.043922026 | 0.1434112 | 0.1527754 | NA | MIR5587 | | 100847028 |
| ENSG00000266235 | 30.62504825 | 0.016650912 | 0.1325867 | 0.6551617 | NA | MIR3176 | | 100423037 |
| ENSG00000261691 | 17.75655104 | -0.020008233 | 0.1356432 | 0.4806145 | NA | NA | NA | |
| ENSG00000007541 | 1152.196906 | 0.220380629 | 0.0925593 | 0.0039797 | 0.0317229 | PIGQ | | 9091 |
| ENSG00000197562 | 501.318329 | -0.0283083 | 0.0936528 | 0.6792599 | 0.8535466 | RAB40C | | 57799 |
| ENSG00000127578 | 32.33313286 | 0.035363601 | 0.1376374 | 0.34246 | NA | WFIKKN1 | | 117166 |
| ENSG00000130731 | 626.4377796 | 0.025895987 | 0.089025 | 0.7066216 | 0.8672619 | METTL26 | | 84326 |
| ENSG00000172366 | 547.3869809 | 0.18195814 | 0.1195444 | 0.0334171 | 0.147858 | MCRIP2 | | 84331 |
| ENSG00000161996 | 852.8607594 | 0.300879766 | 0.105412 | 0.0005958 | 0.0073643 | WDR90 | | 197335 |
| ENSG00000140983 | 2696.794189 | 0.031904166 | 0.0632012 | 0.5722776 | 0.7868143 | RHOT2 | | 89941 |
| ENSG00000103269 | 46.76892908 | -0.003762239 | 0.1286903 | 0.9292203 | 0.9726302 | RHBDL1 | | 9028 |
| ENSG00000103266 | 2049.718166 | 0.0333254 | 0.0659868 | 0.569146 | 0.7847255 | STUB1 | | 10273 |
| ENSG00000161999 | 1015.067614 | -0.103535384 | 0.0907251 | 0.1435139 | 0.3751142 | JMJD8 | | 339123 |
| ENSG00000127580 | 425.3128636 | 0.079594986 | 0.1071319 | 0.2746812 | 0.5431373 | WDR24 | | 84219 |
| ENSG00000261659 | 26.36009453 | -0.00618626 | 0.1327535 | 0.8460416 | NA | NA | NA | |
| ENSG00000127585 | 1005.51418 | -0.040961012 | 0.081921 | 0.5335071 | 0.7584151 | FBXL16 | | 146330 |
| ENSG00000103260 | 815.6808366 | -0.068895459 | 0.0941094 | 0.3285831 | 0.5967766 | METRN | | 79006 |
| ENSG00000103254 | 208.2128214 | -0.046280344 | 0.1170189 | 0.4887403 | 0.7262421 | ANTKMT | | 65990 |
| ENSG00000162004 | 52.28376546 | 0.015179114 | 0.1289167 | 0.7394232 | 0.8857761 | CCDC78 | | 124093 |
| ENSG00000103253 | 163.7706197 | -0.000706106 | 0.1159642 | 0.950599 | 0.9802173 | HAGHL | | 84264 |
| ENSG00000103245 | 874.1058182 | 0.134947933 | 0.09551 | 0.0686105 | 0.2375338 | CIAO3 | | 64428 |
| ENSG00000007376 | 1517.281573 | 0.074258972 | 0.0747784 | 0.2382997 | 0.5030834 | RPUSD1 | | 113000 |
| ENSG00000127586 | 1783.262737 | -0.093888987 | 0.0796554 | 0.1524666 | 0.3898572 | CHTF18 | | 63922 |
| ENSG00000103227 | 54.56996133 | 0.05158983 | 0.1396771 | 0.2899504 | 0.5599126 | LMF1 | | 64788 |
| ENSG00000261713 | 53.25043963 | -0.03672569 | 0.1340004 | 0.4285167 | 0.6796361 | SSTR5-AS1 | | 146336 |
| ENSG00000162009 | 37.87491174 | 0.017156308 | 0.1314818 | 0.6708381 | NA | SSTR5 | | 6755 |
| ENSG00000103275 | 3024.306472 | -0.125396008 | 0.0662998 | 0.0313378 | 0.1421028 | UBE2I | | 7329 |
| ENSG00000261505 | 53.86735515 | 0.038010149 | 0.1344218 | 0.4173006 | 0.6707704 | NA | NA | |
| ENSG00000007516 | 43.17506465 | -0.047117777 | 0.1398476 | 0.2838475 | 0.5531631 | BAIAP3 | | 8938 |
| ENSG00000007520 | 1088.711126 | 0.025363336 | 0.077386 | 0.6919317 | 0.8594028 | TSR3 | | 115939 |
| ENSG00000090581 | 618.938048 | 0.150622784 | 0.1125591 | 0.062463 | 0.2234702 | GNPTG | | 84572 |
| ENSG00000059145 | 865.6707208 | 0.135707904 | 0.1027429 | 0.077089 | 0.2559674 | UNKL | | 64718 |
| ENSG00000174109 | 311.8728283 | 0.076275566 | 0.1159969 | 0.2981643 | 0.5683515 | UQCC4 | | 283951 |
| ENSG00000103249 | 2127.478485 | 0.398058028 | 0.0770467 | 2.55E-08 | 1.42E-06 | CLCN7 | | 1186 |
| ENSG00000100726 | 1644.349898 | -0.007591616 | 0.069093 | 0.8978736 | 0.9594787 | TELO2 | | 9894 |
| ENSG00000187535 | 307.2486379 | -0.037103627 | 0.1060008 | 0.5905795 | 0.8001113 | IFT140 | | 9742 |
| ENSG00000007545 | 482.2341507 | 0.067787594 | 0.1019056 | 0.3472522 | 0.6134684 | CRAMP1 | | 57585 |
| ENSG00000261732 | 24.31171993 | 0.017588223 | 0.1338623 | 0.5999812 | NA | NA | NA | |

| | | | | | | | | |
|---|---|---|---|---|---|---|---|---|
| ENSG00000206053 | 4947.902647 | -0.180521368 | 0.0632849 | 0.001529 | 0.0155847 | JPT2 | | 90861 |
| ENSG00000138834 | 1141.520547 | -0.035087593 | 0.0782952 | 0.5848022 | 0.795977 | MAPK8IP3 | | 23162 |
| ENSG00000103024 | 498.8485829 | 0.095495179 | 0.1076647 | 0.2046183 | 0.4598204 | NME3 | | 4832 |
| ENSG00000074071 | 2047.15987 | -0.077787823 | 0.0686193 | 0.1902194 | 0.4411132 | MRPS34 | | 65993 |
| ENSG00000197774 | 596.2193359 | -0.058908358 | 0.0942678 | 0.4000638 | 0.6583091 | EME2 | | 197342 |
| ENSG00000162032 | 468.5869437 | 0.030385278 | 0.0983654 | 0.6605369 | 0.8426903 | SPSB3 | | 90864 |
| ENSG00000095906 | 972.7072096 | 0.11603013 | 0.0882753 | 0.0992055 | 0.3005468 | NUBP2 | | 10101 |
| ENSG00000063854 | 577.3043019 | 0.09541323 | 0.1036884 | 0.200567 | 0.454126 | HAGH | | 3029 |
| ENSG00000180185 | 636.915503 | 0.042174641 | 0.094096 | 0.5438779 | 0.7661909 | FAHD1 | | 81889 |
| ENSG00000198736 | 488.3976559 | -0.26237832 | 0.1400776 | 0.0083864 | 0.0561065 | MSRB1 | | 51734 |
| ENSG00000140990 | 1324.12196 | 0.086775889 | 0.0779945 | 0.1807381 | 0.4299157 | NDUFB10 | | 4716 |
| ENSG00000140988 | 44612.43909 | -0.077254268 | 0.0508026 | 0.0972739 | 0.296679 | RPS2 | | 6187 |
| ENSG00000255198 | 46.50465389 | 0.053003733 | 0.1441393 | 0.205194 | 0.4606924 | NA | NA | |
| ENSG00000183751 | 1363.695887 | 0.024092622 | 0.0746816 | 0.7014681 | 0.8648958 | TBL3 | | 10607 |
| ENSG00000127554 | 878.1037618 | 0.065846708 | 0.0848875 | 0.3318586 | 0.5992396 | GFER | | 2671 |
| ENSG00000261790 | 8.687072724 | 0.02362352 | 0.1381081 | 0.2529284 | NA | NA | NA | |
| ENSG00000127561 | 179.2512477 | -0.05441058 | 0.1222219 | 0.4147546 | 0.6690718 | SYNGR3 | | 9143 |
| ENSG00000167962 | 2080.622055 | 0.123230555 | 0.0742291 | 0.0515125 | 0.1959993 | ZNF598 | | 90850 |
| ENSG00000065054 | 362.5142313 | 0.061227352 | 0.1071138 | 0.3936664 | 0.6542065 | NHERF2 | | 9351 |
| ENSG00000065057 | 469.1011221 | -0.110444749 | 0.1124582 | 0.1525252 | 0.3898572 | NTHL1 | | 4913 |
| ENSG00000103197 | 1471.022416 | -0.062094091 | 0.0744887 | 0.3221707 | 0.5909668 | TSC2 | | 7249 |
| ENSG00000008710 | 2116.935733 | 0.021904687 | 0.066294 | 0.7072805 | 0.8675568 | PKD1 | | 5310 |
| ENSG00000261123 | 9.540959635 | -0.018229647 | 0.1367941 | 0.3857757 | NA | NA | NA | |
| ENSG00000167964 | 30.15314869 | -0.049352482 | 0.1437524 | 0.1932325 | NA | RAB26 | | 25837 |
| ENSG00000260260 | 249.9557891 | -0.007034796 | 0.1071184 | 0.9147523 | 0.9662935 | SNHG19 | | 100507303 |
| ENSG00000131653 | 2507.670398 | 0.043805595 | 0.0631891 | 0.4344768 | 0.6852379 | TRAF7 | | 84231 |
| ENSG00000167971 | 55.22928124 | -0.005084954 | 0.1293731 | 0.901427 | 0.9614288 | CASKIN1 | | 57524 |
| ENSG00000167965 | 716.3906227 | 0.07668477 | 0.0926856 | 0.2778582 | 0.5463156 | MLST8 | | 64223 |
| ENSG00000182685 | 105.0524717 | -0.024039218 | 0.1233985 | 0.6707707 | 0.849168 | BRICD5 | | 283870 |
| ENSG00000184207 | 1061.387392 | -0.078950641 | 0.083778 | 0.2406388 | 0.5060129 | PGP | | 283871 |
| ENSG00000167967 | 981.6531581 | -0.057609388 | 0.0813683 | 0.3796115 | 0.6408794 | E4F1 | | 1877 |
| ENSG00000167968 | 32.91284429 | 0.013478061 | 0.1321613 | 0.7144224 | NA | DNASE1L2 | | 1775 |
| ENSG00000167969 | 1332.7439 | -0.044730892 | 0.0742108 | 0.4734985 | 0.7138534 | ECI1 | | 1632 |
| ENSG00000205937 | 4371.09872 | 0.029553577 | 0.0576475 | 0.5719591 | 0.7865616 | RNPS1 | | 10921 |
| ENSG00000167970 | 23.72251783 | 0.009588917 | 0.1331345 | 0.7661597 | NA | NA | NA | |
| ENSG00000260778 | 20.77648144 | 0.016976123 | 0.134637 | 0.5757488 | NA | MIR3677HG | | 106660606 |
| ENSG00000167972 | 892.0316624 | 0.350440989 | 0.1191052 | 0.0003521 | 0.0049342 | ABCA3 | | 21 |
| ENSG00000162063 | 1952.188769 | -0.124479782 | 0.0739705 | 0.0483541 | 0.1884003 | CCNF | | 899 |
| ENSG00000162062 | 424.9786876 | 0.217460969 | 0.1393629 | 0.0215603 | 0.1092606 | TEDC2 | | 80178 |
| ENSG00000162065 | 556.4969883 | 0.038924469 | 0.0941297 | 0.5744774 | 0.7887248 | TBC1D24 | | 57465 |
| ENSG00000185883 | 138.3786686 | -0.022166885 | 0.1188188 | 0.7146027 | 0.8710746 | ATP6V0C | | 527 |
| ENSG00000162066 | 783.4018093 | -0.014340641 | 0.0843797 | 0.828234 | 0.929178 | AMDHD2 | | 51005 |
| ENSG00000140992 | 1438.204765 | 0.07560307 | 0.0775529 | 0.2409788 | 0.506323 | PDPK1 | | 5170 |
| ENSG00000269937 | 19.93314753 | -0.00499376 | 0.1331376 | 0.8680354 | NA | NA | NA | |
| ENSG00000215154 | 163.6188109 | 0.127836796 | 0.169002 | 0.1033096 | 0.3081614 | NA | NA | |
| ENSG00000205918 | 22.7558178 | 0.006163058 | 0.1327225 | 0.8510071 | NA | PDPK2P | | 653650 |
| ENSG00000260565 | 340.913516 | -0.050315513 | 0.1058349 | 0.4740644 | 0.7140485 | NA | NA | |
| ENSG00000167977 | 1384.759902 | 0.119146002 | 0.0797263 | 0.0720479 | 0.2450048 | KCTD5 | | 54442 |
| ENSG00000172382 | 18.35107642 | 0.056787028 | 0.150867 | 0.0643246 | NA | PRSS27 | | 83886 |
| ENSG00000205913 | 10.16009092 | 0.019435247 | 0.1368023 | 0.3878467 | NA | SRRM2-AS1 | | 100128788 |
| ENSG00000167978 | 17007.47944 | 0.01395593 | 0.049899 | 0.7632829 | 0.8982943 | SRRM2 | | 23524 |
| ENSG00000103363 | 2808.239693 | -0.051138845 | 0.0662398 | 0.3767184 | 0.6384862 | ELOB | | 6923 |
| ENSG00000005001 | 233.6412316 | 0.201096091 | 0.2012348 | 0.0417316 | 0.1720672 | PRSS22 | | 64063 |
| ENSG00000263325 | 15.50178569 | 0.015178084 | 0.1350517 | 0.5775083 | NA | NA | NA | |
| ENSG00000162076 | 364.1304731 | 0.020522725 | 0.1009218 | 0.7638569 | 0.8982943 | FLYWCH2 | | 114984 |
| ENSG00000059122 | 1587.423414 | 0.200183954 | 0.0838503 | 0.0047146 | 0.0363506 | FLYWCH1 | | 84256 |
| ENSG00000131650 | 327.7866025 | -0.576637177 | 0.159517 | 1.86E-05 | 0.0004299 | KREMEN2 | | 79412 |
| ENSG00000127564 | 2167.525797 | 0.257748545 | 0.0744332 | 0.0001054 | 0.0018218 | PKMYT1 | | 9088 |
| ENSG00000162073 | 1272.474161 | -0.056607691 | 0.0765607 | 0.3736493 | 0.6352333 | PAQR4 | | 124222 |
| ENSG00000272079 | 13.13481544 | 0.005163782 | 0.1344492 | 0.8374629 | NA | NA | NA | |
| ENSG00000184697 | 9.922452211 | 0.025638336 | 0.1384511 | 0.2413148 | NA | CLDN6 | | 9074 |
| ENSG00000006327 | 4130.40252 | 0.116347859 | 0.0723965 | 0.0608183 | 0.2199465 | TNFRSF12A | | 51330 |
| ENSG00000103145 | 304.5865256 | 0.018850559 | 0.1055001 | 0.78162 | 0.9094634 | HCFC1R1 | | 54985 |
| ENSG00000131652 | 563.1413961 | -0.006184488 | 0.0900955 | 0.9263306 | 0.9717697 | THOC6 | | 79228 |
| ENSG00000261971 | 76.17379559 | -0.028586126 | 0.1292732 | 0.5658109 | 0.782102 | LOC124900372 | | 124900372 |
| ENSG00000008517 | 180.4016088 | 0.448776762 | 0.2393977 | 0.0032812 | 0.027377 | IL32 | | 9235 |
| ENSG00000122386 | 360.75382 | 0.090192985 | 0.1172445 | 0.231866 | 0.4941635 | ZNF205 | | 7755 |
| ENSG00000263072 | 78.59147156 | 0.045424891 | 0.1332491 | 0.395835 | 0.6562232 | NA | NA | |
| ENSG00000085644 | 153.2452283 | 0.009562811 | 0.1158415 | 0.8784295 | 0.9511774 | ZNF213 | | 7760 |
| ENSG00000010539 | 411.110926 | 0.071880327 | 0.1070253 | 0.3239629 | 0.5927649 | ZNF200 | | 7752 |

| | | | | | | | | |
|---|---|---|---|---|---|---|---|---|
| ENSG00000006194 | 1242.864333 | 0.315455373 | 0.0914868 | 7.76E-05 | 0.0014159 | ZNF263 | | 10127 |
| ENSG00000140993 | 117.3046516 | 0.113651028 | 0.1685405 | 0.1129105 | 0.3258358 | TIGD7 | | 91151 |
| ENSG00000162086 | 315.3082294 | 0.27000459 | 0.17502 | 0.0137838 | 0.0798654 | ZNF75A | | 7627 |
| ENSG00000140987 | 557.9323482 | 0.033482126 | 0.0933303 | 0.6275754 | 0.8224737 | ZSCAN32 | | 54925 |
| ENSG00000103343 | 209.750714 | 0.069220296 | 0.1266545 | 0.3187634 | 0.5874843 | ZNF174 | | 7727 |
| ENSG00000122390 | 73.84920986 | -0.031864603 | 0.1293039 | 0.5361393 | 0.7604767 | NAA60 | | 79903 |
| ENSG00000103351 | 198.3116921 | 0.360946772 | 0.2302413 | 0.0075072 | 0.0514619 | CLUAP1 | | 23059 |
| ENSG00000188827 | 934.989879 | 0.036846508 | 0.0828715 | 0.5785868 | 0.7915757 | SLX4 | | 84464 |
| ENSG00000213918 | 407.9478646 | 0.223025271 | 0.1412625 | 0.0198651 | 0.1032226 | DNASE1 | | 1773 |
| ENSG00000263235 | 21.12951294 | 0.019795677 | 0.1348221 | 0.5300515 | NA | NA | NA | |
| ENSG00000126602 | 2914.978663 | -0.053332745 | 0.063409 | 0.3416269 | 0.6087837 | TRAP1 | | 10131 |
| ENSG00000178430 | 16.16926887 | -0.008575662 | 0.1342123 | 0.7495357 | NA | NA | NA | |
| ENSG00000005339 | 2836.879198 | -0.092749309 | 0.0736058 | 0.1373293 | 0.3655822 | CREBBP | | 1387 |
| ENSG00000162104 | 516.3756595 | 0.127236391 | 0.1135907 | 0.1084074 | 0.3185678 | ADCY9 | | 115 |
| ENSG00000262468 | 15.31167224 | 0.027322625 | 0.1380182 | 0.3022233 | NA | NA | NA | |
| ENSG00000090447 | 188.3253153 | 0.04383177 | 0.1194068 | 0.5044619 | 0.7381431 | TFAP4 | | 7023 |
| ENSG00000126603 | 462.0753056 | -0.199654939 | 0.133683 | 0.0284245 | 0.1329703 | GLIS2 | | 84662 |
| ENSG00000217930 | 70.52856724 | 0.05914157 | 0.1409598 | 0.2676879 | 0.5358804 | PAM16 | | 51025 |
| ENSG00000262712 | 109.1405739 | 1.016159279 | 0.2829381 | 1.51E-05 | 0.0003609 | NA | NA | |
| ENSG00000262246 | 135.9455963 | -0.036633509 | 0.1235004 | 0.5431795 | 0.7660123 | CORO7 | | 79585 |
| ENSG00000168140 | 69.68691726 | 0.041176404 | 0.1334963 | 0.4165519 | 0.6700325 | VASN | | 114990 |
| ENSG00000103423 | 2040.229429 | 0.301963854 | 0.0800217 | 2.45E-05 | 0.0005366 | DNAJA3 | | 9093 |
| ENSG00000153406 | 864.0284596 | 0.161191376 | 0.0975264 | 0.0342547 | 0.1504809 | NMRAL1 | | 57407 |
| ENSG00000103415 | 1425.667251 | 0.154672121 | 0.0816011 | 0.0231583 | 0.1147694 | HMOX2 | | 3163 |
| ENSG00000089486 | 548.9530154 | 0.228545821 | 0.1363104 | 0.0161272 | 0.0886084 | CDIP1 | | 29965 |
| ENSG00000153443 | 550.2706927 | 0.18955274 | 0.1213629 | 0.0289443 | 0.1350234 | UBALD1 | | 124402 |
| ENSG00000102858 | 1521.625683 | 0.032438186 | 0.0712364 | 0.5944797 | 0.8031487 | MGRN1 | | 23295 |
| ENSG00000168101 | 427.6578609 | -0.148041071 | 0.1266731 | 0.0775288 | 0.2567132 | NUDT16L1 | | 84309 |
| ENSG00000168096 | 577.4659741 | 0.050082063 | 0.0928158 | 0.4481372 | 0.6957325 | ANKS3 | | 124401 |
| ENSG00000103199 | 281.5902413 | 0.020021641 | 0.1064582 | 0.7631811 | 0.8982943 | ZNF500 | | 26048 |
| ENSG00000067836 | 281.8041713 | -0.280404326 | 0.1761593 | 0.0117856 | 0.0714043 | ROGDI | | 79641 |
| ENSG00000140632 | 2253.236489 | -0.122540929 | 0.0690967 | 0.0411971 | 0.1705862 | GLYR1 | | 84656 |
| ENSG00000118900 | 1744.954096 | 0.000507897 | 0.0705563 | 0.9942131 | 0.9977151 | UBN1 | | 29855 |
| ENSG00000118898 | 1536.242106 | -0.600814357 | 0.0970163 | 4.21E-11 | 3.93E-09 | PPL | | 5493 |
| ENSG00000103174 | 546.76934 | 0.037051755 | 0.0930764 | 0.5917657 | 0.8007903 | NAGPA | | 51172 |
| ENSG00000033011 | 935.4923966 | 0.019131459 | 0.0813023 | 0.7707272 | 0.9022714 | ALG1 | | 56052 |
| ENSG00000118894 | 400.0134379 | -0.012927327 | 0.099144 | 0.8493269 | 0.9378167 | EEF2KMT | | 196483 |
| ENSG00000260289 | 10.27261787 | -0.004831575 | 0.1348652 | 0.8276934 | NA | NA | NA | |
| ENSG00000067365 | 355.6240229 | 0.046994521 | 0.1055821 | 0.5041194 | 0.7381071 | METTL22 | | 79091 |
| ENSG00000184857 | 178.2807454 | 0.067400878 | 0.1264507 | 0.3278764 | 0.5960044 | TMEM186 | | 25880 |
| ENSG00000140650 | 722.6787668 | -0.027930447 | 0.0890771 | 0.6801717 | 0.853781 | PMM2 | | 5373 |
| ENSG00000260350 | 13.33242225 | -0.000472808 | 0.1343919 | 0.9822025 | NA | NA | NA | |
| ENSG00000260276 | 94.02616444 | 0.72222108 | 0.3517297 | 0.0014347 | 0.0148704 | NA | NA | |
| ENSG00000153048 | 867.336651 | -0.158422571 | 0.0999465 | 0.0396338 | 0.1667733 | CARHSP1 | | 23589 |
| ENSG00000259939 | 30.53897644 | 0.036248121 | 0.138161 | 0.3237547 | NA | NA | NA | |
| ENSG00000187555 | 3466.97435 | -0.192846684 | 0.0641924 | 0.0008476 | 0.0097163 | USP7 | | 7874 |
| ENSG00000182831 | 942.0324689 | -0.027647461 | 0.0814164 | 0.6723704 | 0.8499086 | HAPSTR1 | | 29035 |
| ENSG00000262944 | 550.9048991 | -0.293104723 | 0.133298 | 0.0034615 | 0.0284944 | NA | NA | |
| ENSG00000263244 | 1058.535473 | -0.33502504 | 0.0992798 | 9.05E-05 | 0.0016062 | NA | NA | |
| ENSG00000166669 | 147.1326712 | 0.04860113 | 0.1249964 | 0.4435269 | 0.692026 | ATF7IP2 | | 80063 |
| ENSG00000213853 | 955.6240455 | -0.058421202 | 0.0839946 | 0.3816015 | 0.6426596 | EMP2 | | 2013 |
| ENSG00000103274 | 289.2883562 | -0.314923457 | 0.1765402 | 0.0068791 | 0.0480881 | NUBP1 | | 4682 |
| ENSG00000182108 | 314.8526012 | -0.028535284 | 0.1042925 | 0.6766777 | 0.8523045 | DEXI | | 28955 |
| ENSG00000263013 | 63.64126303 | -0.012758931 | 0.1268123 | 0.7909178 | 0.9123751 | NA | NA | |
| ENSG00000038532 | 817.69988 | -0.067869886 | 0.0870339 | 0.3202453 | 0.5889146 | CLEC16A | | 23274 |
| ENSG00000175643 | 257.8866607 | -0.009546023 | 0.1091247 | 0.883054 | 0.9527945 | RMI2 | | 116028 |
| ENSG00000185338 | 142.5877959 | -1.278385885 | 0.2648901 | 6.44E-08 | 3.23E-06 | SOCS1 | | 8651 |
| ENSG00000189067 | 2330.889953 | -0.038316236 | 0.0715585 | 0.5297569 | 0.7555939 | LITAF | | 9516 |
| ENSG00000184602 | 849.8506077 | -0.052529778 | 0.0842473 | 0.4286344 | 0.6796361 | SNN | | 8303 |
| ENSG00000153066 | 695.095077 | -0.051506875 | 0.0929853 | 0.458005 | 0.7023667 | TXNDC11 | | 51061 |
| ENSG00000122299 | 1592.666496 | -0.004455395 | 0.0704621 | 0.94037 | 0.9766571 | ZC3H7A | | 29066 |
| ENSG00000171490 | 5158.000608 | -6.60E-05 | 0.0546036 | 0.9979155 | 0.998947 | RSL1D1 | | 26156 |
| ENSG00000103342 | 10772.63448 | -0.031375405 | 0.1188482 | 0.6160836 | 0.815878 | GSPT1 | | 2935 |
| ENSG00000261560 | 47.84411568 | 0.018879591 | 0.1305403 | 0.6639868 | 0.8443298 | NA | NA | |
| ENSG00000260318 | 12.05247833 | 0.000279957 | 0.1345106 | 0.9929518 | NA | NA | NA | |
| ENSG00000234719 | 26.8598051 | 0.024544736 | 0.1349307 | 0.4857346 | NA | NPIPB2 | | 729978 |
| ENSG00000048462 | 9.662353045 | -0.026090037 | 0.138646 | 0.2228616 | NA | TNFRSF17 | | 608 |
| ENSG00000048471 | 1026.519033 | 0.322543001 | 0.1180083 | 0.0007519 | 0.0089165 | SNX29 | | 92017 |
| ENSG00000103381 | 98.98870823 | -0.082594022 | 0.1491489 | 0.1872179 | 0.4381671 | CPPED1 | | 55313 |
| ENSG00000175595 | 269.8809719 | 0.017146486 | 0.1067188 | 0.8009918 | 0.9169162 | ERCC4 | | 2072 |

| | | | | | | | |
|---|---|---|---|---|---|---|---|
| ENSG00000186260 | 1131.577757 | -0.281774106 | 0.0969961 | 0.0005744 | 0.007145 | MRTFB | 57496 |
| ENSG00000262454 | 18.85278311 | -0.022108903 | 0.1357566 | 0.4584247 | NA | MIR193BHG | 100129781 |
| ENSG00000140694 | 862.3707037 | -0.063268216 | 0.0940716 | 0.3674947 | 0.6302608 | PARN | 5073 |
| ENSG00000103429 | 1548.784666 | 0.03394865 | 0.0704376 | 0.5746057 | 0.7887497 | BFAR | 51283 |
| ENSG00000103512 | 1838.436863 | 0.000395975 | 0.0672239 | 0.9968189 | 0.998947 | NOMO1 | 23420 |
| ENSG00000183458 | 328.0993452 | 0.042447087 | 0.1053569 | 0.5429015 | 0.7657126 | PKD1P3 | 339039 |
| ENSG00000207425 | 20.46435775 | 0.018415286 | 0.1347541 | 0.5495522 | NA | NA | NA |
| ENSG00000183426 | 89.42853756 | 0.106050383 | 0.1691729 | 0.1107808 | 0.3227008 | NPIPA1 | 9284 |
| ENSG00000179889 | 2302.323858 | -0.157619262 | 0.0722821 | 0.0116133 | 0.0706906 | PDXDC1 | 23042 |
| ENSG00000260872 | 14.19965886 | 0.006888945 | 0.1343105 | 0.7935092 | NA | NA | NA |
| ENSG00000157045 | 776.3397153 | -0.200492211 | 0.1056493 | 0.0141891 | 0.0812861 | NTAN1 | 123803 |
| ENSG00000085721 | 2041.834162 | 0.312077723 | 0.0762178 | 6.25E-06 | 0.0001686 | RRN3 | 54700 |
| ENSG00000188599 | 33.47685694 | -0.010225831 | 0.1314725 | 0.7818058 | NA | NPIPP1 | 100874381 |
| ENSG00000270580 | 29.86616997 | -0.003669388 | 0.1313987 | 0.9175646 | NA | PKD1P6-NP | 105369154 |
| ENSG00000250251 | 421.2246896 | 0.042778552 | 0.1010673 | 0.540905 | 0.7642142 | PKD1P6 | 353511 |
| ENSG00000183793 | 19.48839027 | -0.002725134 | 0.1331706 | 0.9257331 | NA | NPIPA5 | 100288332 |
| ENSG00000166780 | 332.0766823 | 0.022891025 | 0.1029084 | 0.7391554 | 0.8857761 | BMERB1 | 89927 |
| ENSG00000166783 | 712.2451592 | -0.248567392 | 0.1111045 | 0.0044361 | 0.0345225 | MARF1 | 9665 |
| ENSG00000072864 | 956.9509328 | -0.398237705 | 0.1009527 | 7.96E-06 | 0.0002071 | NDE1 | 54820 |
| ENSG00000263335 | 36.15365137 | -0.002855429 | 0.1307359 | 0.9379836 | NA | NA | NA |
| ENSG00000133393 | 850.6895758 | -0.192213516 | 0.1034854 | 0.01669 | 0.0906816 | CEP20 | 123811 |
| ENSG00000103222 | 1468.193397 | 0.128266351 | 0.0809636 | 0.055917 | 0.2080666 | ABCC1 | 4363 |
| ENSG00000091262 | 9.404913738 | -0.016923745 | 0.136745 | 0.3903726 | NA | ABCC6 | 368 |
| ENSG00000103226 | 86.7624282 | 0.004267725 | 0.1230421 | 0.9381123 | 0.9761997 | NOMO3 | 408050 |
| ENSG00000183889 | 35.61026212 | 0.014930508 | 0.1316433 | 0.7019796 | NA | NPIPA9 | 105376752 |
| ENSG00000227827 | 520.8561998 | 0.104834574 | 0.1065865 | 0.1703375 | 0.4162267 | PKD1P2 | 283955 |
| ENSG00000214940 | 37.41281872 | 0.050862662 | 0.1428656 | 0.2259785 | NA | NPIPA8 | 101059953 |
| ENSG00000205746 | 163.3721306 | -0.024995935 | 0.1162287 | 0.6925399 | 0.8594028 | PKD1P4 | 353512 |
| ENSG00000254681 | 806.45119 | -0.009293731 | 0.0831221 | 0.8871481 | 0.9545517 | PKD1P5 | 348156 |
| ENSG00000185164 | 983.3925273 | 0.042270302 | 0.0827401 | 0.5241182 | 0.7515816 | NOMO2 | 283820 |
| ENSG00000134419 | 2533.212756 | -0.276539678 | 0.0828479 | 0.0001393 | 0.0022762 | RPS15A | 6210 |
| ENSG00000170540 | 4549.989543 | -0.196624724 | 0.0727393 | 0.0020452 | 0.0191888 | ARL6IP1 | 23204 |
| ENSG00000157106 | 7238.597984 | 0.073591747 | 0.0564325 | 0.1489544 | 0.383724 | SMG1 | 23049 |
| ENSG00000170537 | 51.60048375 | -0.020807308 | 0.1300359 | 0.6430538 | 0.8330388 | TMC7 | 79905 |
| ENSG00000167186 | 208.7991651 | -0.066040492 | 0.1227917 | 0.3272062 | 0.5953077 | COQ7 | 10229 |
| ENSG00000205730 | 1291.071985 | 0.049134196 | 0.0749329 | 0.4349039 | 0.6853902 | ITPRIPL2 | 162073 |
| ENSG00000261759 | 9.839204369 | 0.002295076 | 0.1349503 | 0.9170179 | NA | NA | NA |
| ENSG00000103534 | 12.4382716 | 0.006785776 | 0.1347855 | 0.7756857 | NA | TMC5 | 79838 |
| ENSG00000006007 | 1228.134028 | -0.159901677 | 0.0858401 | 0.02328 | 0.1150885 | GDE1 | 51573 |
| ENSG00000103540 | 1162.677912 | 0.003319069 | 0.0758529 | 0.9588275 | 0.9831259 | CCP110 | 9738 |
| ENSG00000103544 | 810.4022683 | -0.223293687 | 0.104813 | 0.0069981 | 0.0487445 | VPS35L | 57020 |
| ENSG00000103550 | 1201.893004 | 0.037253322 | 0.0763934 | 0.5428065 | 0.7657126 | KNOP1 | 400506 |
| ENSG00000261312 | 12.76469987 | 0.022246524 | 0.1370026 | 0.3689053 | NA | NA | NA |
| ENSG00000174628 | 129.6888755 | 0.076457752 | 0.1403965 | 0.2408542 | 0.506323 | IQCK | 124152 |
| ENSG00000167191 | 1863.205508 | -0.402108701 | 0.0786269 | 3.31E-08 | 1.76E-06 | GPRC5B | 51704 |
| ENSG00000066654 | 1231.161767 | -0.127061504 | 0.082829 | 0.0614711 | 0.2213454 | THUMPD1 | 55623 |
| ENSG00000196678 | 253.3619557 | 0.003047308 | 0.1072899 | 0.9668899 | 0.987 | ERI2 | 112479 |
| ENSG00000005189 | 169.8157212 | 0.106388134 | 0.1476654 | 0.1578178 | 0.3976292 | REXO5 | 81691 |
| ENSG00000188215 | 275.401587 | 0.0263409 | 0.107612 | 0.6983009 | 0.8625201 | DCUN1D3 | 123879 |
| ENSG00000102897 | 416.0433519 | 0.170549818 | 0.1426321 | 0.0562473 | 0.2088445 | LYRM1 | 57149 |
| ENSG00000158486 | 10.24124645 | 0.041431295 | 0.1442754 | 0.0653951 | NA | DNAH3 | 55567 |
| ENSG00000011638 | 176.9497256 | 0.345798576 | 0.2465579 | 0.0099247 | 0.0633366 | LDAF1 | 57146 |
| ENSG00000189149 | 13.50643236 | 0.029610932 | 0.1391331 | 0.2305518 | NA | NA | NA |
| ENSG00000169246 | 138.474964 | -0.030236942 | 0.1214179 | 0.617207 | 0.8165812 | NPIPB3 | 23117 |
| ENSG00000180747 | 126.1137989 | -0.003900834 | 0.1183274 | 0.9468267 | 0.9786934 | SMG1P3 | 100271836 |
| ENSG00000197006 | 1784.409906 | -0.111117906 | 0.0733179 | 0.0752051 | 0.251855 | METTL9 | 51108 |
| ENSG00000185864 | 36.21137048 | 0.011383001 | 0.1319667 | 0.75707 | NA | NPIPB4 | 440345 |
| ENSG00000140740 | 2870.794649 | 0.0171366 | 0.0628398 | 0.7595773 | 0.8969311 | UQCRC2 | 7385 |
| ENSG00000185716 | 322.1666902 | 0.04352823 | 0.1062891 | 0.5330982 | 0.758237 | MOSMO | 730094 |
| ENSG00000103319 | 657.273771 | -0.02718336 | 0.087631 | 0.6918896 | 0.8594028 | EEF2K | 29904 |
| ENSG00000058600 | 1212.773232 | 0.04968529 | 0.0780325 | 0.4336403 | 0.684513 | POLR3E | 55718 |
| ENSG00000140743 | 927.2668481 | -0.017190614 | 0.0797928 | 0.7926209 | 0.9131541 | CDR2 | 1039 |
| ENSG00000257122 | 269.8725804 | -0.051361878 | 0.1130931 | 0.4562009 | 0.7007999 | NA | NA |
| ENSG00000237296 | 219.2796392 | -0.025248732 | 0.111469 | 0.6984553 | 0.8626196 | SMG1P1 | 641298 |
| ENSG00000243716 | 248.1736105 | 0.031152545 | 0.1106652 | 0.6442903 | 0.8338883 | NPIPB5 | 100132247 |
| ENSG00000103404 | 985.1212602 | 0.08152468 | 0.0881742 | 0.2400725 | 0.5054546 | USP31 | 57478 |
| ENSG00000168434 | 242.5304845 | -0.095694776 | 0.1320943 | 0.2056802 | 0.4607272 | COG7 | 91949 |
| ENSG00000260136 | 76.48208993 | 0.044583418 | 0.1326385 | 0.4083921 | 0.6640358 | NA | NA |
| ENSG00000103365 | 2522.480715 | -0.263270977 | 0.0748074 | 8.21E-05 | 0.0014883 | GGA2 | 23062 |
| ENSG00000103356 | 832.4186097 | -0.029735946 | 0.0838096 | 0.6536712 | 0.8399571 | EARS2 | 124454 |

| | | | | | | | | |
|---|---|---|---|---|---|---|---|---|
| ENSG00000103353 | 1891.727247 | -0.05811048 | 0.068219 | 0.3245259 | 0.593455 | UBFD1 | | 56061 |
| ENSG00000260751 | 22.03585862 | -0.015735991 | 0.1339661 | 0.6193112 | NA | NA | NA | |
| ENSG00000004779 | 1183.698419 | -0.059560017 | 0.0785086 | 0.3563401 | 0.6197178 | NDUFAB1 | | 4706 |
| ENSG00000083093 | 616.3332488 | 0.183157856 | 0.1160251 | 0.0303855 | 0.1394565 | PALB2 | | 79728 |
| ENSG00000166847 | 1931.500565 | 0.017852085 | 0.0667361 | 0.7598893 | 0.896955 | DCTN5 | | 84516 |
| ENSG00000260482 | 231.0065862 | -0.046143366 | 0.1148879 | 0.4988205 | 0.7341215 | NA | NA | |
| ENSG00000259806 | 43.39570688 | -0.044128199 | 0.1397044 | 0.2842408 | 0.5534617 | NA | NA | |
| ENSG00000166851 | 3471.552969 | -0.40968267 | 0.0737046 | 2.83E-09 | 1.93E-07 | PLK1 | | 5347 |
| ENSG00000122257 | 1849.9227 | -0.022455153 | 0.0669605 | 0.6997772 | 0.8635218 | RBBP6 | | 5930 |
| ENSG00000090905 | 2072.942701 | -0.261898922 | 0.0757419 | 0.000103 | 0.0017855 | TNRC6A | | 27327 |
| ENSG00000140750 | 638.6856739 | -0.115874303 | 0.1075149 | 0.1316096 | 0.3571039 | ARHGAP17 | | 55114 |
| ENSG00000205629 | 330.4664413 | -0.015967535 | 0.1033826 | 0.8132576 | 0.9236424 | LCMT1 | | 51451 |
| ENSG00000155592 | 379.4085496 | 0.070964929 | 0.1078417 | 0.326739 | 0.5948626 | ZKSCAN2 | | 342357 |
| ENSG00000230330 | 29.64327012 | -0.028230924 | 0.1357851 | 0.4252209 | NA | NA | NA | |
| ENSG00000155666 | 48.9994192 | -0.043556421 | 0.1383104 | 0.3182312 | 0.5871518 | KDM8 | | 79831 |
| ENSG00000169189 | 369.2144685 | -0.206183647 | 0.1534662 | 0.0324398 | 0.1452349 | NSMCE1 | | 197370 |
| ENSG00000077238 | 1268.728011 | 0.058343374 | 0.0774363 | 0.3543146 | 0.6187516 | IL4R | | 3566 |
| ENSG00000077235 | 4138.223648 | -0.172101597 | 0.0649305 | 0.0029486 | 0.0253818 | GTF3C1 | | 2975 |
| ENSG00000047578 | 756.8537119 | -0.060937407 | 0.0880679 | 0.3732861 | 0.6352113 | KATNIP | | 23247 |
| ENSG00000169180 | 3710.590638 | 0.04593964 | 0.0602661 | 0.396889 | 0.6567013 | XPO6 | | 23214 |
| ENSG00000188322 | 144.5976446 | -0.020704639 | 0.1183912 | 0.7295726 | 0.8794775 | SBK1 | | 388228 |
| ENSG00000205609 | 1231.31871 | 0.033615851 | 0.0751849 | 0.5936621 | 0.8026087 | EIF3CL | | 728689 |
| ENSG00000188603 | 64.50909923 | -0.024296771 | 0.1286257 | 0.6205672 | 0.818389 | CLN3 | | 1201 |
| ENSG00000176476 | 273.5495829 | 0.160209183 | 0.156386 | 0.0710201 | 0.2427109 | SGF29 | | 112869 |
| ENSG00000184110 | 1632.667711 | -0.011610776 | 0.0736183 | 0.8508173 | 0.9389287 | EIF3C | | 8663 |
| ENSG00000168488 | 4767.108689 | -0.014365203 | 0.0576991 | 0.7811788 | 0.9091865 | ATXN2L | | 11273 |
| ENSG00000260570 | 13.66117851 | -0.010097749 | 0.1348324 | 0.6839761 | NA | NA | NA | |
| ENSG00000178952 | 5644.786355 | -0.081351651 | 0.0559231 | 0.1097891 | 0.3209078 | TUFM | | 7284 |
| ENSG00000178188 | 1029.910397 | 0.007860242 | 0.0772321 | 0.9026288 | 0.9618324 | SH2B1 | | 25970 |
| ENSG00000196296 | 57.05595514 | 0.00517189 | 0.127187 | 0.9137349 | 0.9661641 | ATP2A1 | | 487 |
| ENSG00000260442 | 53.48592428 | 0.023608258 | 0.1308168 | 0.599613 | 0.806457 | ATP2A1-AS: | | 100289092 |
| ENSG00000177548 | 391.1492004 | 0.004718916 | 0.0985468 | 0.9460548 | 0.9784722 | RABEP2 | | 79874 |
| ENSG00000176953 | 2774.10899 | 0.105099134 | 0.0654478 | 0.0678865 | 0.2357962 | NFATC2IP | | 84901 |
| ENSG00000169682 | 44.60134836 | 0.009024474 | 0.1297494 | 0.830572 | 0.9304016 | SPNS1 | | 83985 |
| ENSG00000261067 | 16.77348736 | 0.013165902 | 0.1345274 | 0.6410318 | NA | NA | NA | |
| ENSG00000213658 | 14.29544964 | -0.006111782 | 0.1342558 | 0.8121611 | NA | LAT | | 27040 |
| ENSG00000103472 | 91.56001453 | 0.021712232 | 0.1242035 | 0.6895886 | 0.8588425 | RRN3P2 | | 653390 |
| ENSG00000260953 | 69.39410323 | 0.233288587 | 0.4350204 | 0.017643 | 0.0949274 | NA | NA | |
| ENSG00000254206 | 22.79968765 | 0.0457639 | 0.1434441 | 0.1746075 | NA | NPIPB11 | | 728888 |
| ENSG00000254634 | 20.34272866 | 0.055454891 | 0.1495822 | 0.0830294 | NA | SMG1P6 | | 100422558 |
| ENSG00000169203 | 16.06991768 | 0.013492293 | 0.1348318 | 0.6182184 | NA | NPIPB12 | | 440353 |
| ENSG00000205534 | 88.33521581 | 0.016461452 | 0.1240178 | 0.760463 | 0.8971789 | NA | NA | |
| ENSG00000260727 | 40.20973705 | 0.041272436 | 0.1379421 | 0.330884 | NA | SLC7A5P1 | | 81893 |
| ENSG00000103485 | 841.6998973 | -0.040453751 | 0.0922786 | 0.5561664 | 0.7757478 | QPRT | | 23475 |
| ENSG00000079616 | 1613.79216 | -0.153512836 | 0.0806129 | 0.0228832 | 0.1141309 | KIF22 | | 3835 |
| ENSG00000103495 | 2854.169899 | -0.002294151 | 0.0622791 | 0.9662015 | 0.9865549 | MAZ | | 4150 |
| ENSG00000259952 | 13.76806875 | -0.011531206 | 0.1348992 | 0.6478035 | NA | NA | NA | |
| ENSG00000238045 | 183.0051483 | -0.041291051 | 0.1199593 | 0.5207702 | 0.7489848 | MVP-DT | | 112268170 |
| ENSG00000167371 | 11.34890003 | -0.019577379 | 0.1366649 | 0.4030378 | NA | PRRT2 | | 112476 |
| ENSG00000185928 | 111.0524526 | -0.020224572 | 0.121819 | 0.7239669 | 0.8758838 | NA | NA | |
| ENSG00000013364 | 1362.434556 | -0.044017835 | 0.07672 | 0.4891469 | 0.7262901 | MVP | | 9961 |
| ENSG00000103502 | 1285.695664 | 0.094837351 | 0.0797207 | 0.148051 | 0.3820469 | CDIPT | | 10423 |
| ENSG00000174938 | 244.2073949 | 0.18118518 | 0.1744256 | 0.0531746 | 0.2007538 | SEZ6L2 | | 26470 |
| ENSG00000174939 | 267.778922 | 0.286740113 | 0.1821274 | 0.0116412 | 0.0708231 | ASPHD1 | | 253982 |
| ENSG00000174943 | 177.701842 | 0.275436462 | 0.2401524 | 0.0195934 | 0.1020375 | KCTD13 | | 253980 |
| ENSG00000260114 | 47.1830255 | 0.009532207 | 0.1295821 | 0.8229056 | 0.927297 | NA | NA | |
| ENSG00000247735 | 12.99446886 | 0.041980325 | 0.1440464 | 0.0909878 | NA | NA | NA | |
| ENSG00000149932 | 384.8195417 | -0.000603025 | 0.098037 | 0.9919155 | 0.9968652 | TMEM219 | | 124446 |
| ENSG00000149930 | 1660.229646 | 0.026210232 | 0.0695127 | 0.6621882 | 0.8437964 | TAOK2 | | 9344 |
| ENSG00000149929 | 527.7469536 | -0.016941109 | 0.0915824 | 0.8110636 | 0.9221365 | HIRIP3 | | 8479 |
| ENSG00000169592 | 1013.214113 | 0.050732995 | 0.0800888 | 0.4321603 | 0.6833714 | INO80E | | 283899 |
| ENSG00000149927 | 16.21826222 | -0.014206057 | 0.1348006 | 0.6045395 | NA | DOC2A | | 8448 |
| ENSG00000149926 | 24.90300695 | 0.033102602 | 0.1377911 | 0.3348433 | NA | TLCD3B | | 83723 |
| ENSG00000149925 | 11500.74134 | 0.037755819 | 0.0525014 | 0.4390828 | 0.6886014 | ALDOA | | 226 |
| ENSG00000149923 | 1670.195847 | -0.070539921 | 0.0735268 | 0.2549273 | 0.5218727 | PPP4C | | 5531 |
| ENSG00000102882 | 433.3224753 | -0.060329838 | 0.101749 | 0.3973457 | 0.6567942 | MAPK3 | | 5595 |
| ENSG00000102879 | 136.4243626 | -0.156358783 | 0.1960314 | 0.0656751 | 0.2316277 | CORO1A | | 11151 |
| ENSG00000169627 | 23.73664847 | -0.020272503 | 0.1350006 | 0.5157132 | NA | BOLA2 | | 552900 |
| ENSG00000261052 | 10.92226068 | 0.001478446 | 0.1346175 | 0.9516145 | NA | SULT1A3 | | 6818 |
| ENSG00000183604 | 48.10174138 | 0.01970094 | 0.1305602 | 0.6520062 | 0.8388682 | NA | NA | |

| | | | | | | | |
|---|---|---|---|---|---|---|---|
| ENSG00000169217 | 2095.931788 | 0.098299954 | 0.0697585 | 0.1031964 | 0.3079809 | CD2BP2 | 10421 |
| ENSG00000169221 | 989.8694386 | 0.13744869 | 0.091327 | 0.0577892 | 0.2128107 | TBC1D10B | 26000 |
| ENSG00000180035 | 231.9592984 | 0.214146308 | 0.1876095 | 0.0344872 | 0.1512181 | ZNF48 | 197407 |
| ENSG00000179965 | 173.3690764 | 0.010800834 | 0.1139429 | 0.8648431 | 0.9457239 | ZNF771 | 51333 |
| ENSG00000179958 | 2128.868172 | 0.047615121 | 0.0677957 | 0.423573 | 0.6756461 | DCTPP1 | 79077 |
| ENSG00000179918 | 824.6284387 | 0.094033122 | 0.0924797 | 0.1867875 | 0.4375983 | SEPHS2 | 22928 |
| ENSG00000169957 | 544.6344406 | 0.178765547 | 0.1210232 | 0.0370284 | 0.1589594 | ZNF768 | 79724 |
| ENSG00000169955 | 54.48893787 | 0.005995778 | 0.1281122 | 0.8957797 | 0.9584283 | ZNF747 | 65988 |
| ENSG00000235560 | 13.93860482 | -0.010980903 | 0.1346984 | 0.6715255 | NA | ZNF747-DT | 107984875 |
| ENSG00000169951 | 75.55866017 | 0.10249833 | 0.1728125 | 0.0940459 | 0.2908137 | ZNF764 | 92595 |
| ENSG00000229809 | 35.68775364 | 0.045786444 | 0.1409144 | 0.2579129 | NA | ZNF688 | 146542 |
| ENSG00000156853 | 392.7852587 | 0.099412818 | 0.1169403 | 0.1954463 | 0.448 | ZNF689 | 115509 |
| ENSG00000156858 | 505.9958654 | -0.012028597 | 0.0934218 | 0.8587277 | 0.9430101 | PRR14 | 78994 |
| ENSG00000156860 | 1093.915383 | 0.071686076 | 0.0851561 | 0.2907915 | 0.560795 | FBRS | 64319 |
| ENSG00000261840 | 9.097939397 | -0.000716578 | 0.1349871 | 0.9705195 | NA | LOC730183 | 730183 |
| ENSG00000080603 | 2662.418326 | 0.028461392 | 0.07258 | 0.6459932 | 0.8353643 | SRCAP | 10847 |
| ENSG00000156873 | 400.4541176 | 0.009427328 | 0.098084 | 0.8918152 | 0.956385 | PHKG2 | 5261 |
| ENSG00000196118 | 77.84986854 | 0.111371154 | 0.1786244 | 0.0882739 | 0.2796657 | CFAP119 | 90835 |
| ENSG00000103549 | 2291.520394 | 0.015762353 | 0.0670946 | 0.7884454 | 0.9116361 | RNF40 | 9810 |
| ENSG00000102870 | 542.3138445 | 0.148860419 | 0.1273384 | 0.076635 | 0.2549883 | ZNF629 | 23361 |
| ENSG00000099385 | 644.5176194 | -0.027067976 | 0.0880771 | 0.6926892 | 0.8594968 | BCL7C | 9274 |
| ENSG00000260083 | 31.43708849 | 0.021184024 | 0.133444 | 0.5688558 | NA | MIR762HG | 101928736 |
| ENSG00000260852 | 36.08502631 | -0.016227068 | 0.131876 | 0.6733132 | NA | FBXL19-AS1 | 283932 |
| ENSG00000099364 | 673.9114567 | -0.15973182 | 0.1124855 | 0.0501021 | 0.1927678 | FBXL19 | 54620 |
| ENSG00000175938 | 22.6360121 | 0.014450906 | 0.1338508 | 0.6490754 | NA | ORAI3 | 93129 |
| ENSG00000099381 | 1166.264867 | -0.139220801 | 0.0900916 | 0.0523048 | 0.1983034 | SETD1A | 9739 |
| ENSG00000099377 | 49.83469343 | 0.036107449 | 0.1346865 | 0.4210058 | 0.6735758 | HSD3B7 | 80270 |
| ENSG00000099365 | 12.47789388 | -0.015213469 | 0.1356079 | 0.5336379 | NA | STX1B | 112755 |
| ENSG00000103496 | 552.7136198 | 0.037368767 | 0.0933909 | 0.589003 | 0.798683 | STX4 | 6810 |
| ENSG00000167394 | 291.6398976 | 0.03971617 | 0.1090246 | 0.5196247 | 0.7482416 | ZNF668 | 79759 |
| ENSG00000167395 | 411.9353769 | 0.482139093 | 0.1567243 | 0.0001471 | 0.0023736 | ZNF646 | 9726 |
| ENSG00000151006 | 37.79903764 | -0.010172604 | 0.1309243 | 0.7915618 | NA | PRSS53 | 339105 |
| ENSG00000167397 | 137.6202 | -0.021041438 | 0.1195227 | 0.7247497 | 0.876447 | VKORC1 | 79001 |
| ENSG00000103507 | 457.6532979 | -0.214011146 | 0.1386804 | 0.0226561 | 0.1133386 | BCKDK | 10295 |
| ENSG00000103510 | 393.2572645 | -0.018297188 | 0.1018232 | 0.7881072 | 0.9116361 | KAT8 | 84148 |
| ENSG00000262766 | 8.391528507 | 0.003883521 | 0.1353248 | 0.8445498 | NA | NA | NA |
| ENSG00000089280 | 12728.01154 | -0.235258539 | 0.0549771 | 3.70E-06 | 0.0001069 | FUS | 2521 |
| ENSG00000260304 | 11.71260536 | -0.044832921 | 0.1455976 | 0.0630646 | NA | NA | NA |
| ENSG00000260060 | 8.349900276 | -0.016941249 | 0.1367582 | 0.3910593 | NA | NA | NA |
| ENSG00000260267 | 30.88879419 | 0.0159192 | 0.1325607 | 0.6643627 | NA | NA | NA |
| ENSG00000140691 | 333.9579532 | -0.188999874 | 0.1503126 | 0.0418182 | 0.1723554 | ARMC5 | 79798 |
| ENSG00000140682 | 443.7681582 | 0.037200643 | 0.0979486 | 0.5930912 | 0.8022115 | TGFB1I1 | 7041 |
| ENSG00000140688 | 503.6685866 | 0.081179302 | 0.1057125 | 0.2703931 | 0.5381601 | RUSF1 | 64755 |
| ENSG00000131797 | 152.2839219 | 0.103668296 | 0.1516324 | 0.1556307 | 0.3943696 | NA | NA |
| ENSG00000197302 | 265.8301392 | 0.000745697 | 0.1057981 | 0.9922721 | 0.9969601 | KRBOX5 | 124411 |
| ENSG00000185947 | 305.4197779 | 0.036280343 | 0.1076571 | 0.5984913 | 0.8058763 | ZNF267 | 10308 |
| ENSG00000230267 | 41.29397529 | -0.037914263 | 0.1363665 | 0.3731232 | 0.6350652 | NA | NA |
| ENSG00000260644 | 10.27929703 | 0.029716129 | 0.1397062 | 0.1723788 | NA | HERC2P5 | 388254 |
| ENSG00000171241 | 1701.456622 | -0.062589285 | 0.0735331 | 0.3133915 | 0.5822768 | SHCBP1 | 79801 |
| ENSG00000261512 | 34.66606705 | -0.045756109 | 0.1413066 | 0.2436527 | NA | NA | NA |
| ENSG00000069329 | 4825.152205 | -0.082647145 | 0.0571617 | 0.1112459 | 0.3233517 | VPS35 | 55737 |
| ENSG00000091651 | 2616.737683 | 0.208170657 | 0.0817836 | 0.0028632 | 0.0248832 | ORC6 | 23594 |
| ENSG00000155330 | 368.4362789 | 0.153671561 | 0.1356402 | 0.0738332 | 0.2489019 | C16orf87 | 388272 |
| ENSG00000166123 | 1511.879682 | 0.269328075 | 0.1032269 | 0.0014836 | 0.0152692 | GPT2 | 84706 |
| ENSG00000069345 | 3633.134509 | -0.003524336 | 0.0576418 | 0.944804 | 0.9784014 | DNAJA2 | 10294 |
| ENSG00000171208 | 4570.27538 | -0.064363807 | 0.0588859 | 0.2242807 | 0.4849126 | NETO2 | 81831 |
| ENSG00000129636 | 842.0781253 | -0.023581251 | 0.0817743 | 0.7206851 | 0.8733776 | ITFG1 | 81533 |
| ENSG00000102893 | 1612.56209 | -0.135039112 | 0.0795442 | 0.0418509 | 0.1723769 | PHKB | 5257 |
| ENSG00000102910 | 1716.670297 | -0.178476583 | 0.0820633 | 0.0096914 | 0.062441 | LONP2 | 83752 |
| ENSG00000196470 | 604.7510184 | -0.021708452 | 0.0892312 | 0.7473881 | 0.890205 | SIAH1 | 6477 |
| ENSG00000102921 | 940.3085484 | -0.016877328 | 0.0806207 | 0.7948263 | 0.9144629 | N4BP1 | 9683 |
| ENSG00000205423 | 407.2042164 | -0.266999236 | 0.1535117 | 0.0103629 | 0.0650448 | CNEP1R1 | 255919 |
| ENSG00000155393 | 942.05984 | -0.465677761 | 0.0994585 | 2.39E-07 | 9.94E-06 | HEATR3 | 55027 |
| ENSG00000121274 | 971.8410314 | 0.032507761 | 0.083742 | 0.6157099 | 0.8158539 | TENT4B | 64282 |
| ENSG00000121281 | 241.7925467 | 0.014034813 | 0.1099346 | 0.8215849 | 0.9267922 | ADCY7 | 113 |
| ENSG00000166164 | 2624.362422 | -0.092016854 | 0.0652359 | 0.1086072 | 0.3188079 | BRD7 | 29117 |
| ENSG00000261393 | 9.511402981 | -0.021895309 | 0.1374852 | 0.3120024 | NA | NA | NA |
| ENSG00000140807 | 9.139559008 | 0.000515883 | 0.1349695 | 0.9829239 | NA | NKD1 | 85407 |
| ENSG00000083799 | 983.5189347 | 0.12537972 | 0.0945011 | 0.0869317 | 0.2767899 | CYLD | 1540 |
| ENSG00000224578 | 75.03298185 | -0.069091318 | 0.144928 | 0.2211446 | 0.4805935 | HNRNPA1L3 | 642659 |

| | | | | | | | | |
|---|---|---|---|---|---|---|---|---|
| ENSG00000177200 | 1434.60789 | -0.024198619 | 0.0737003 | 0.6959346 | 0.8612721 | CHD9 | | 80205 |
| ENSG00000103479 | 1311.523685 | -0.163820546 | 0.0842091 | 0.0188367 | 0.099607 | RBL2 | | 5934 |
| ENSG00000166971 | 399.1416835 | -0.144696038 | 0.128673 | 0.0838561 | 0.2705118 | AKTIP | | 64400 |
| ENSG00000103494 | 591.1388433 | 0.029526294 | 0.0937453 | 0.6684105 | 0.8475579 | RPGRIP1L | | 23322 |
| ENSG00000140718 | 642.6251703 | -0.029913944 | 0.0897527 | 0.6581388 | 0.8423052 | FTO | | 79068 |
| ENSG00000177508 | 187.8825775 | -0.017293041 | 0.1145707 | 0.7832652 | 0.9096561 | IRX3 | | 79191 |
| ENSG00000245694 | 472.8062685 | -0.032965664 | 0.0964207 | 0.6329396 | 0.8252447 | CRNDE | | 643911 |
| ENSG00000176842 | 103.2528439 | 0.015602404 | 0.1225262 | 0.780453 | 0.9086486 | IRX5 | | 10265 |
| ENSG00000087253 | 601.8105993 | -0.230556036 | 0.132044 | 0.0140233 | 0.0807324 | LPCAT2 | | 54947 |
| ENSG00000159461 | 2299.056554 | -0.05113364 | 0.0654434 | 0.3727158 | 0.6345995 | AMFR | | 267 |
| ENSG00000167005 | 4677.462925 | -0.160574398 | 0.0604792 | 0.0032864 | 0.0274004 | NUDT21 | | 11051 |
| ENSG00000087263 | 2444.661464 | 0.212953828 | 0.0758921 | 0.001336 | 0.0140851 | OGFOD1 | | 55239 |
| ENSG00000125124 | 1111.573015 | 0.016103643 | 0.0767649 | 0.8006812 | 0.9169162 | BBS2 | | 583 |
| ENSG00000125148 | 32039.66031 | -0.189809723 | 0.0676611 | 0.0011144 | 0.0121767 | MT2A | | 4502 |
| ENSG00000260823 | 92.63282125 | 0.542553982 | 0.3903274 | 0.0053226 | 0.0398527 | NA | NA | |
| ENSG00000169715 | 6861.209889 | -0.025209371 | 0.1051066 | 0.7120549 | 0.869458 | MT1E | | 4493 |
| ENSG00000205364 | 117.4908235 | 0.059239789 | 0.1320268 | 0.3431702 | 0.6097025 | MT1M | | 4499 |
| ENSG00000255986 | 17.1613988 | 1.599562087 | 0.8475533 | 0.0017038 | NA | NA | NA | |
| ENSG00000198417 | 65.19814589 | 0.020868953 | 0.1283071 | 0.6694071 | 0.848177 | MT1F | | 4494 |
| ENSG00000187193 | 285.4395516 | 0.055117087 | 0.1111691 | 0.4328885 | 0.6841524 | MT1X | | 4501 |
| ENSG00000102900 | 2729.531686 | -0.045795864 | 0.0658634 | 0.4266351 | 0.6787658 | NUP93 | | 9688 |
| ENSG00000051108 | 1150.585723 | 0.148150061 | 0.0938119 | 0.0450016 | 0.1806896 | HERPUD1 | | 9709 |
| ENSG00000261270 | 13.2649557 | -0.00030222 | 0.1343318 | 0.9878998 | NA | NA | NA | |
| ENSG00000261114 | 9.433730112 | 0.01018273 | 0.1355608 | 0.6298657 | NA | NA | NA | |
| ENSG00000140853 | 13.84566387 | 0.018536045 | 0.1359849 | 0.471588 | NA | NLRC5 | | 84166 |
| ENSG00000140848 | 1164.269556 | -0.059546092 | 0.0782906 | 0.3557409 | 0.6192724 | CPNE2 | | 221184 |
| ENSG00000172775 | 1469.805324 | -0.023711505 | 0.0739474 | 0.7024128 | 0.8650963 | PSME3IP1 | | 80011 |
| ENSG00000159579 | 1527.046778 | 0.047232038 | 0.0747104 | 0.4512752 | 0.6978775 | RSPRY1 | | 89970 |
| ENSG00000102931 | 1440.374974 | -0.013525597 | 0.0719 | 0.8241112 | 0.9274924 | ARL2BP | | 23568 |
| ENSG00000260946 | 19.64158195 | 0.008550212 | 0.1334347 | 0.7804381 | NA | NA | NA | |
| ENSG00000102934 | 15.66270641 | 0.00669301 | 0.1340269 | 0.8128681 | NA | PLLP | | 51090 |
| ENSG00000006210 | 107.1787264 | 0.011143203 | 0.1247709 | 0.8317581 | 0.9304418 | CX3CL1 | | 6376 |
| ENSG00000005194 | 2217.656247 | 0.029052325 | 0.0648298 | 0.6112658 | 0.8136975 | CIAPIN1 | | 57019 |
| ENSG00000088682 | 962.7272926 | 0.071259986 | 0.084918 | 0.2965313 | 0.5664726 | COQ9 | | 57017 |
| ENSG00000102978 | 2399.093406 | -0.033855065 | 0.0654621 | 0.5548985 | 0.7749708 | POLR2C | | 5432 |
| ENSG00000125170 | 184.5039517 | 0.003880505 | 0.1136953 | 0.9523604 | 0.9806656 | DOK4 | | 55715 |
| ENSG00000135736 | 365.2133357 | 0.019338695 | 0.1001657 | 0.7789966 | 0.907593 | CCDC102A | | 92922 |
| ENSG00000205336 | 198.3536397 | 0.966518641 | 0.2146873 | 3.38E-07 | 1.34E-05 | ADGRG1 | | 9289 |
| ENSG00000140854 | 738.2481398 | -0.090982724 | 0.0926549 | 0.200237 | 0.4540225 | KATNB1 | | 10300 |
| ENSG00000140859 | 402.6896928 | 0.379096761 | 0.155639 | 0.0012579 | 0.0134185 | KIFC3 | | 3801 |
| ENSG00000166188 | 175.8858864 | 0.172623552 | 0.1897892 | 0.0586336 | 0.2148365 | ZNF319 | | 57567 |
| ENSG00000103005 | 1649.76883 | -0.048109818 | 0.0725706 | 0.4339738 | 0.684681 | USB1 | | 79650 |
| ENSG00000102996 | 129.0983189 | -0.079604489 | 0.1410183 | 0.2288741 | 0.4902363 | MMP15 | | 4324 |
| ENSG00000070761 | 917.3893354 | -0.041411806 | 0.082413 | 0.5309442 | 0.756397 | CFAP20 | | 29105 |
| ENSG00000070770 | 2475.338558 | -0.108098247 | 0.0674198 | 0.0658759 | 0.2320448 | CSNK2A2 | | 1459 |
| ENSG00000103021 | 190.9862356 | 0.579922137 | 0.2189647 | 0.0004293 | 0.0057207 | CCDC113 | | 29070 |
| ENSG00000181938 | 718.8889891 | 0.184324763 | 0.1172949 | 0.0301119 | 0.1387524 | GINS3 | | 64785 |
| ENSG00000103034 | 8.90572501 | -0.0102413 | 0.1355883 | 0.622026 | NA | NDRG4 | | 65009 |
| ENSG00000103037 | 534.1560729 | -0.210996352 | 0.1237614 | 0.0181339 | 0.096811 | SETD6 | | 79918 |
| ENSG00000125107 | 8450.109768 | -0.04902095 | 0.0535767 | 0.2804177 | 0.5491044 | CNOT1 | | 23019 |
| ENSG00000103042 | 693.2019785 | 0.133581601 | 0.1041337 | 0.0841138 | 0.2709074 | SLC38A7 | | 55238 |
| ENSG00000125166 | 5010.371052 | -0.157923808 | 0.0588935 | 0.0031062 | 0.026241 | GOT2 | | 2806 |
| ENSG00000224631 | 65.44591936 | -0.081844635 | 0.1577085 | 0.1326122 | 0.3586711 | RPS27AP16 | | 643358 |
| ENSG00000150394 | 60.14860886 | 0.126762742 | 0.2061988 | 0.0470554 | 0.1855687 | CDH8 | | 1006 |
| ENSG00000260115 | 10.11795808 | -0.003554112 | 0.1348594 | 0.8706494 | NA | LOC124903 | | 124903776 |
| ENSG00000246898 | 14.01211769 | -0.011844981 | 0.1348566 | 0.6443529 | NA | NA | NA | |
| ENSG00000166546 | 110.9842297 | -0.010902949 | 0.1204328 | 0.8483837 | 0.9375782 | BEAN1 | | 146227 |
| ENSG00000166548 | 354.1048825 | -0.159541214 | 0.1387668 | 0.0668823 | 0.234118 | TK2 | | 7084 |
| ENSG00000217555 | 394.0037419 | -0.046389249 | 0.1060647 | 0.505354 | 0.7382367 | CKLF | | 51192 |
| ENSG00000254788 | 34.78268488 | -0.01821594 | 0.1323346 | 0.6322411 | NA | CKLF-CMTM | | 100529251 |
| ENSG00000089505 | 153.9888385 | 0.022229723 | 0.1177555 | 0.7204528 | 0.8733776 | CMTM1 | | 113540 |
| ENSG00000140931 | 2488.998197 | -0.598099263 | 0.0893995 | 1.59E-12 | 1.95E-10 | CMTM3 | | 123920 |
| ENSG00000183723 | 2208.16782 | -0.060267524 | 0.0665067 | 0.3016048 | 0.5710237 | CMTM4 | | 146223 |
| ENSG00000135720 | 5530.701547 | -0.097525031 | 0.0559596 | 0.0553296 | 0.2064872 | DYNC1LI2 | | 1783 |
| ENSG00000260465 | 57.96326432 | -0.03885387 | 0.1339493 | 0.4188903 | 0.6717512 | NA | NA | |
| ENSG00000159593 | 2386.899149 | -0.088336918 | 0.0676557 | 0.1349748 | 0.3616424 | NAE1 | | 8883 |
| ENSG00000172840 | 508.6772723 | 0.235163093 | 0.1287044 | 0.0117463 | 0.0712983 | PDP2 | | 57546 |
| ENSG00000261705 | 103.0052272 | 0.028501583 | 0.1242528 | 0.6172923 | 0.8166015 | NA | NA | |
| ENSG00000261088 | 182.1785443 | 0.115425504 | 0.1517051 | 0.1361511 | 0.3632667 | NA | NA | |
| ENSG00000166592 | 23.49685571 | 0.055823441 | 0.1493083 | 0.0962327 | NA | RRAD | | 6236 |

| | | | | | | | | |
|---|---|---|---|---|---|---|---|---|
| ENSG00000166595 | 963.3661556 | 0.007967777 | 0.0793014 | 0.9026273 | 0.9618324 | CIAO2B | | 51647 |
| ENSG00000172831 | 624.5775542 | 0.215029252 | 0.1170248 | 0.0139391 | 0.0804064 | CES2 | | 8824 |
| ENSG00000067955 | 2775.022151 | -0.034026595 | 0.0627746 | 0.5409231 | 0.7642142 | CBFB | | 865 |
| ENSG00000125149 | 781.8349148 | 0.028096926 | 0.0841639 | 0.6734648 | 0.8501877 | PHAF1 | | 80262 |
| ENSG00000237172 | 222.8901548 | -0.044382756 | 0.118825 | 0.4994814 | 0.7346313 | B3GNT9 | | 84752 |
| ENSG00000102871 | 482.0879468 | 0.048983296 | 0.0977652 | 0.486843 | 0.7240658 | TRADD | | 8717 |
| ENSG00000135722 | 85.30405387 | -0.003178396 | 0.1231588 | 0.9510361 | 0.9803259 | FBXL8 | | 55336 |
| ENSG00000102878 | 103.6830535 | 0.028082485 | 0.124267 | 0.6215177 | 0.818389 | HSF4 | | 3299 |
| ENSG00000140939 | 126.2352725 | 0.065559773 | 0.1346769 | 0.3032271 | 0.5725825 | NOL3 | | 8996 |
| ENSG00000196123 | 241.1196342 | -0.128168132 | 0.1446003 | 0.1170724 | 0.3339413 | MATCAP1 | | 653319 |
| ENSG00000205250 | 1683.727599 | -0.067176329 | 0.0714456 | 0.2772873 | 0.5460273 | E2F4 | | 1874 |
| ENSG00000102890 | 123.3782071 | 0.037799707 | 0.1244527 | 0.5294379 | 0.7554508 | ELMO3 | | 79767 |
| ENSG00000168701 | 658.2154594 | -0.004565227 | 0.0865843 | 0.9447828 | 0.9784014 | TMEM208 | | 29100 |
| ENSG00000135723 | 989.6871088 | -0.01421732 | 0.08167 | 0.8274039 | 0.928782 | FHOD1 | | 29109 |
| ENSG00000135740 | 251.0833221 | 0.208534647 | 0.1812814 | 0.0365844 | 0.1577493 | SLC9A5 | | 6553 |
| ENSG00000262691 | 31.81693041 | 0.008286737 | 0.1318062 | 0.8199996 | NA | NA | NA | |
| ENSG00000196155 | 109.2646705 | 0.117046389 | 0.1720444 | 0.1051326 | 0.3117679 | PLEKHG4 | | 25894 |
| ENSG00000176387 | 35.26660169 | 0.028264616 | 0.1348024 | 0.4632756 | NA | HSD11B2 | | 3291 |
| ENSG00000203401 | 10.58440228 | 0.023419955 | 0.1377041 | 0.3024275 | NA | NA | NA | |
| ENSG00000159720 | 2252.794419 | 0.143936199 | 0.0720215 | 0.0207382 | 0.10634 | ATP6V0D1 | | 9114 |
| ENSG00000039523 | 1419.902825 | 0.129213877 | 0.0810666 | 0.0544044 | 0.2041306 | RIPOR1 | | 79567 |
| ENSG00000261396 | 10.76317198 | 0.004724047 | 0.134813 | 0.8382004 | NA | NA | NA | |
| ENSG00000259804 | 10.02848256 | 0.036593746 | 0.1423547 | 0.0861729 | NA | CTCF-DT | | 107984813 |
| ENSG00000102974 | 2510.797138 | 0.022941802 | 0.0689816 | 0.7021304 | 0.8649636 | CTCF | | 10664 |
| ENSG00000159753 | 218.4405416 | 0.125063446 | 0.1514462 | 0.1204112 | 0.3390055 | CARMIL2 | | 146206 |
| ENSG00000102977 | 1448.26957 | 0.169394766 | 0.0829365 | 0.0144997 | 0.08227 | ACD | | 65057 |
| ENSG00000102981 | 97.41580962 | -0.09757368 | 0.1596515 | 0.1382979 | 0.3669827 | PARD6A | | 50855 |
| ENSG00000124074 | 578.8025883 | 0.316314496 | 0.1262164 | 0.0014449 | 0.0149358 | ENKD1 | | 84080 |
| ENSG00000159761 | 19.62190958 | 0.044951159 | 0.1438661 | 0.1478749 | NA | C16orf86 | | 388284 |
| ENSG00000141098 | 942.893111 | 0.043358006 | 0.082637 | 0.5061408 | 0.7387601 | GFOD2 | | 81577 |
| ENSG00000141084 | 995.2675192 | 0.148103443 | 0.093516 | 0.0448104 | 0.1801826 | RANBP10 | | 57610 |
| ENSG00000270165 | 13.57820821 | 0.013950872 | 0.1353739 | 0.5750874 | NA | NA | NA | |
| ENSG00000102904 | 29.2534908 | 0.040839878 | 0.1399073 | 0.2706992 | NA | TSNAXIP1 | | 55815 |
| ENSG00000102901 | 1468.163201 | 0.025797206 | 0.0713542 | 0.6735498 | 0.8501877 | CENPT | | 80152 |
| ENSG00000168286 | 1107.785265 | -0.029457572 | 0.0780383 | 0.6455048 | 0.8350083 | THAP11 | | 57215 |
| ENSG00000102898 | 2752.036635 | -0.043295099 | 0.064158 | 0.4439035 | 0.6921329 | NUTF2 | | 10204 |
| ENSG00000038358 | 2180.672579 | -0.130106019 | 0.0738275 | 0.039052 | 0.1651386 | EDC4 | | 23644 |
| ENSG00000263126 | 12.05358252 | -0.018874221 | 0.1364707 | 0.4221412 | NA | NA | NA | |
| ENSG00000159792 | 599.5981613 | 0.104357584 | 0.1067909 | 0.168565 | 0.4142664 | PSKH1 | | 5681 |
| ENSG00000261884 | 77.36391608 | 0.008391734 | 0.1246425 | 0.8725867 | 0.9484215 | NA | NA | |
| ENSG00000205220 | 96.91627543 | 0.062204444 | 0.137088 | 0.2976927 | 0.5680006 | PSMB10 | | 5699 |
| ENSG00000213398 | 214.9511358 | 0.027595959 | 0.1115531 | 0.67974 | 0.853781 | LCAT | | 3931 |
| ENSG00000124067 | 1798.451529 | 0.156237406 | 0.0759648 | 0.0160862 | 0.0885083 | SLC12A4 | | 6560 |
| ENSG00000167264 | 344.8215928 | -0.000112607 | 0.1029243 | 0.9974595 | 0.998947 | DUS2 | | 54920 |
| ENSG00000182810 | 410.161003 | 0.076520531 | 0.1144085 | 0.2958801 | 0.5659367 | DDX28 | | 55794 |
| ENSG00000072736 | 758.6125973 | -0.068341639 | 0.0901599 | 0.3245535 | 0.593455 | NFATC3 | | 4775 |
| ENSG00000262160 | 11.09280537 | 0.021151027 | 0.1372214 | 0.3440096 | NA | NA | NA | |
| ENSG00000103066 | 585.4839265 | -0.005088799 | 0.0923913 | 0.9394286 | 0.9764939 | PLA2G15 | | 23659 |
| ENSG00000103064 | 3577.016458 | -0.144838163 | 0.0659808 | 0.0129884 | 0.0764318 | SLC7A6 | | 9057 |
| ENSG00000103061 | 1033.16924 | 0.104952629 | 0.0886863 | 0.135563 | 0.3626084 | SLC7A6OS | | 84138 |
| ENSG00000132600 | 954.6033718 | -0.032414714 | 0.0873966 | 0.6305573 | 0.8242329 | PRMT7 | | 54496 |
| ENSG00000184939 | 496.4970188 | 0.217268199 | 0.1314089 | 0.0186944 | 0.099145 | ZFP90 | | 146198 |
| ENSG00000103047 | 557.0715171 | -0.039334536 | 0.0928191 | 0.5677689 | 0.7837518 | TANGO6 | | 79613 |
| ENSG00000103044 | 5290.089023 | 0.010031127 | 0.1096655 | 0.8805562 | 0.9518649 | HAS3 | | 3038 |
| ENSG00000168802 | 983.3417295 | -0.025942936 | 0.0801368 | 0.6891632 | 0.858679 | CHTF8 | | 54921 |
| ENSG00000141076 | 4600.537977 | 0.113378054 | 0.0602824 | 0.0361821 | 0.1566735 | UTP4 | | 84916 |
| ENSG00000168807 | 1442.185392 | -0.099889635 | 0.0822293 | 0.136111 | 0.3632667 | SNTB2 | | 6645 |
| ENSG00000132612 | 1822.146574 | 0.045278176 | 0.0685092 | 0.4465232 | 0.6943341 | VPS4A | | 27183 |
| ENSG00000213380 | 454.3066168 | 0.116762972 | 0.1161912 | 0.1377205 | 0.3659493 | COG8 | | 84342 |
| ENSG00000258429 | 295.9912292 | 0.060147389 | 0.1127625 | 0.3981912 | 0.6573306 | PDF | | 64146 |
| ENSG00000132603 | 2610.714293 | 0.111737644 | 0.0709531 | 0.0675799 | 0.2352909 | NIP7 | | 51388 |
| ENSG00000132604 | 1080.841011 | 0.227115376 | 0.0993486 | 0.0047559 | 0.0366206 | TERF2 | | 7014 |
| ENSG00000103018 | 4364.227874 | -0.073233926 | 0.0622239 | 0.1856826 | 0.4358915 | CYB5B | | 80777 |
| ENSG00000102908 | 4382.412974 | -0.209242566 | 0.0657358 | 0.0004065 | 0.0054765 | NFAT5 | | 10725 |
| ENSG00000181019 | 4331.772437 | -0.022404866 | 0.0589692 | 0.6728273 | 0.8500061 | NQO1 | | 1728 |
| ENSG00000262136 | 26.39509793 | 0.01378003 | 0.1329065 | 0.6913642 | NA | NA | NA | |
| ENSG00000141101 | 2128.059468 | 0.310023507 | 0.0810111 | 1.90E-05 | 0.0004347 | NOB1 | | 28987 |
| ENSG00000198373 | 1300.007264 | -0.147910754 | 0.0832463 | 0.0314269 | 0.1422309 | WWP2 | | 11060 |
| ENSG00000196696 | 79.98481835 | -0.065325402 | 0.1426382 | 0.2446384 | 0.5102895 | PDXDC2P-N | | 283970 |
| ENSG00000090857 | 2036.820857 | 0.165512846 | 0.0790213 | 0.0133194 | 0.0776457 | PDPR | | 55066 |

| | | | | | | | |
|---|---|---|---|---|---|---|---|
| ENSG00000247228 | 23.33576107 | 0.014877423 | 0.1339686 | 0.6377004 | NA | LOC400541 | 400541 |
| ENSG00000261556 | 182.7806508 | -0.020422051 | 0.1166508 | 0.7407098 | 0.88659 | SMG1P7 | 100506060 |
| ENSG00000223496 | 935.823513 | -0.078657142 | 0.0860528 | 0.2486571 | 0.5150848 | EXOSC6 | 118460 |
| ENSG00000090861 | 7504.325484 | 0.243061262 | 0.0559565 | 3.25E-06 | 9.56E-05 | AARS1 | 16 |
| ENSG00000157349 | 399.2024793 | 0.096419246 | 0.1139041 | 0.205243 | 0.4606924 | DDX19B | 11269 |
| ENSG00000261777 | 17.3353041 | -0.015141141 | 0.1346803 | 0.5963254 | NA | DDX19A-DT | 100506083 |
| ENSG00000168872 | 1511.494925 | 0.022224366 | 0.0713084 | 0.7166449 | 0.8716295 | DDX19A | 55308 |
| ENSG00000157350 | 926.7156752 | -0.011231536 | 0.0827973 | 0.8637451 | 0.9454522 | ST3GAL2 | 6483 |
| ENSG00000260111 | 12.71433341 | 0.018815447 | 0.1362969 | 0.4425087 | NA | NA | NA |
| ENSG00000157353 | 394.2449544 | -0.00825788 | 0.098415 | 0.9028837 | 0.9618565 | FCSK | 197258 |
| ENSG00000103051 | 1357.064671 | 0.005667791 | 0.0751612 | 0.9290649 | 0.9726302 | COG4 | 25839 |
| ENSG00000189091 | 8155.05381 | -0.072739421 | 0.0571863 | 0.1601414 | 0.4011606 | SF3B3 | 23450 |
| ENSG00000260023 | 39.28553376 | 0.064503692 | 0.1513493 | 0.1308708 | NA | NA | NA |
| ENSG00000132613 | 552.2427762 | 0.212552246 | 0.1241976 | 0.0178742 | 0.0957747 | MTSS2 | 92154 |
| ENSG00000268927 | 11.92060967 | 0.010329302 | 0.1350873 | 0.6664244 | NA | NA | NA |
| ENSG00000103043 | 1247.565502 | -0.121231821 | 0.0829418 | 0.0739619 | 0.2491832 | VAC14 | 55697 |
| ENSG00000180917 | 845.6644431 | 0.131651417 | 0.0947395 | 0.0737921 | 0.2488353 | CMTR2 | 55783 |
| ENSG00000167377 | 40.61807714 | 0.065455563 | 0.1512188 | 0.1385974 | NA | ZNF23 | 7571 |
| ENSG00000157429 | 27.55206811 | 0.011856524 | 0.1329108 | 0.7255999 | NA | ZNF19 | 7567 |
| ENSG00000040199 | 1276.570631 | -0.213140309 | 0.0893645 | 0.0041728 | 0.0329786 | PHLPP2 | 23035 |
| ENSG00000166747 | 3843.944294 | -0.092816 | 0.0599103 | 0.0844231 | 0.2713742 | AP1G1 | 164 |
| ENSG00000224470 | 2505.209995 | -0.085700466 | 0.0662737 | 0.1388015 | 0.3676153 | ATXN1L | 342371 |
| ENSG00000182149 | 3869.662333 | -0.05201284 | 0.0597408 | 0.3319734 | 0.5992396 | IST1 | 9798 |
| ENSG00000102984 | 90.09303405 | 0.00764871 | 0.1229567 | 0.8884416 | 0.9545517 | ZNF821 | 55565 |
| ENSG00000263320 | 13.66428002 | -0.006918336 | 0.134464 | 0.7820358 | NA | NA | NA |
| ENSG00000102967 | 386.9950601 | 0.037124248 | 0.1039099 | 0.5939224 | 0.8026852 | DHODH | 1723 |
| ENSG00000140830 | 673.0073868 | 0.508708061 | 0.1201151 | 1.71E-06 | 5.38E-05 | TXNL4B | 54957 |
| ENSG00000140829 | 3506.694159 | 0.001995831 | 0.0597014 | 0.9675888 | 0.9872153 | DHX38 | 9785 |
| ENSG00000118557 | 63.95858055 | -0.007566767 | 0.1263365 | 0.8748553 | 0.9493854 | PMFBP1 | 83449 |
| ENSG00000261008 | 31.90549677 | 0.015629669 | 0.1325171 | 0.6752985 | NA | LINC01572 | 101927957 |
| ENSG00000259768 | 39.83847542 | 0.003042426 | 0.1298644 | 0.9421983 | NA | ZFHX3-AS1 | 101927978 |
| ENSG00000140836 | 943.8412951 | -0.162988196 | 0.0965662 | 0.0314076 | 0.1422309 | ZFHX3 | 463 |
| ENSG00000259901 | 186.2893192 | 0.395152368 | 0.2439602 | 0.006037 | 0.0435839 | NA | NA |
| ENSG00000259209 | 12.35045414 | -0.006971108 | 0.1346358 | 0.7737482 | NA | NA | NA |
| ENSG00000103035 | 1956.143218 | -0.098856265 | 0.0731263 | 0.1111947 | 0.3233517 | PSMD7 | 5713 |
| ENSG00000259972 | 47.46020875 | 0.04095921 | 0.1365663 | 0.3630181 | 0.6262607 | NA | NA |
| ENSG00000239763 | 13.89281659 | -0.001989182 | 0.1341989 | 0.9343047 | NA | NA | NA |
| ENSG00000214331 | 134.9220997 | 0.062029888 | 0.1339115 | 0.3197697 | 0.5886556 | NA | NA |
| ENSG00000260539 | 447.8818967 | -0.175815037 | 0.1293358 | 0.0443268 | 0.1788545 | NA | NA |
| ENSG00000090863 | 3925.814447 | -0.209762858 | 0.0674016 | 0.0005165 | 0.0066369 | GLG1 | 2734 |
| ENSG00000168411 | 3968.30491 | 0.323588226 | 0.0682404 | 3.06E-07 | 1.23E-05 | RFWD3 | 55159 |
| ENSG00000168404 | 570.7329091 | 0.03244678 | 0.0929133 | 0.6377652 | 0.8291302 | MLKL | 197259 |
| ENSG00000103091 | 1516.34298 | -0.149084676 | 0.0883375 | 0.0367322 | 0.1581528 | WDR59 | 79726 |
| ENSG00000186187 | 434.0179049 | 0.039405177 | 0.0987733 | 0.572463 | 0.786884 | ZNRF1 | 84937 |
| ENSG00000166816 | 27.56572001 | 0.037685369 | 0.1392331 | 0.2856764 | NA | LDHD | 197257 |
| ENSG00000184517 | 382.6446377 | 0.058106924 | 0.1045864 | 0.4158882 | 0.6697358 | ZFP1 | 162239 |
| ENSG00000050820 | 4101.810308 | 0.079888715 | 0.06102 | 0.1430517 | 0.374409 | BCAR1 | 9564 |
| ENSG00000153774 | 785.1687048 | 0.116302679 | 0.0978229 | 0.1162692 | 0.3325516 | CFDP1 | 10428 |
| ENSG00000261783 | 9.932649917 | -0.024473819 | 0.1383457 | 0.2316743 | NA | NA | NA |
| ENSG00000166822 | 978.7977137 | 0.036877621 | 0.0808816 | 0.5670403 | 0.7831055 | TMEM170A | 124491 |
| ENSG00000205084 | 109.2329093 | 0.082477333 | 0.1462698 | 0.202731 | 0.4569858 | TMEM231 | 79583 |
| ENSG00000034713 | 1159.503545 | -0.063115268 | 0.0803711 | 0.3350558 | 0.6026773 | GABARAPL: | 11345 |
| ENSG00000065457 | 1136.478698 | 0.214052402 | 0.0917296 | 0.0047478 | 0.0365823 | ADAT1 | 23536 |
| ENSG00000065427 | 5865.433728 | -0.025395171 | 0.0564425 | 0.6202874 | 0.818389 | KARS1 | 3735 |
| ENSG00000166848 | 1347.116248 | 0.114725913 | 0.0794714 | 0.0822699 | 0.266996 | TERF2IP | 54386 |
| ENSG00000103111 | 1768.716249 | 0.00662769 | 0.0680532 | 0.9131114 | 0.9656034 | MON1B | 22879 |
| ENSG00000205078 | 27.42220419 | -0.066976209 | 0.1555933 | 0.0758377 | NA | SYCE1L | 100130958 |
| ENSG00000140876 | 65.09707753 | -0.012364461 | 0.1271632 | 0.7949984 | 0.9144634 | NUDT7 | 283927 |
| ENSG00000186153 | 337.0252286 | 0.131360684 | 0.1314773 | 0.1107117 | 0.3225799 | WWOX | 51741 |
| ENSG00000166446 | 61.45708299 | 0.061145499 | 0.1435829 | 0.2355288 | 0.499283 | CDYL2 | 124359 |
| ENSG00000103121 | 1122.168678 | 0.065440509 | 0.0826836 | 0.3261264 | 0.5942836 | CMC2 | 56942 |
| ENSG00000166451 | 1767.783945 | -0.082349911 | 0.0731344 | 0.184437 | 0.4347246 | CENPN | 55839 |
| ENSG00000261061 | 309.7543978 | -0.120238771 | 0.1347695 | 0.1345364 | 0.3614351 | NA | NA |
| ENSG00000166454 | 1326.341523 | -0.133912368 | 0.0842312 | 0.0517944 | 0.1967512 | ATMIN | 23300 |
| ENSG00000166455 | 59.46671629 | -0.014034066 | 0.1280703 | 0.7624369 | 0.8982682 | C16orf46 | 123775 |
| ENSG00000140905 | 333.4921369 | -0.051713206 | 0.1084312 | 0.4637117 | 0.7079196 | GCSH | 2653 |
| ENSG00000261609 | 657.6229751 | 0.238478796 | 0.1200511 | 0.00841 | 0.0562318 | GAN | 8139 |
| ENSG00000153815 | 4292.725128 | -0.011898153 | 0.0606095 | 0.8261256 | 0.9286016 | CMIP | 80790 |
| ENSG00000197943 | 376.845448 | 0.058675381 | 0.1058466 | 0.4119041 | 0.6661689 | PLCG2 | 5336 |
| ENSG00000260682 | 46.9639933 | 0.018492522 | 0.1308298 | 0.6641906 | 0.8443298 | NA | NA |

| | | | | | | | |
|---|---|---|---|---|---|---|---|
| ENSG00000135698 | 873.8763468 | -0.01389052 | 0.080526 | 0.8325266 | 0.9305286 | MPHOSPH6 | 10200 |
| ENSG00000140945 | 236.4430686 | 0.081715981 | 0.1251929 | 0.2702987 | 0.5381601 | CDH13 | 1012 |
| ENSG00000230989 | 3527.084918 | -0.173294703 | 0.0645301 | 0.0026737 | 0.0236249 | HSBP1 | 3281 |
| ENSG00000103150 | 72.48884067 | 0.125821238 | 0.1998405 | 0.0575484 | 0.2122584 | MLYCD | 23417 |
| ENSG00000140961 | 258.7262315 | 0.932690697 | 0.1976842 | 1.24E-07 | 5.77E-06 | OSGIN1 | 29948 |
| ENSG00000140943 | 2631.797788 | -0.058508112 | 0.0645549 | 0.3030394 | 0.5724132 | MBTPS1 | 8720 |
| ENSG00000260018 | 30.42720577 | 0.050308851 | 0.1442645 | 0.1864253 | NA | NA | NA |
| ENSG00000103160 | 975.3939467 | 0.018064469 | 0.0810643 | 0.7828736 | 0.9096561 | HSDL1 | 83693 |
| ENSG00000103168 | 1454.95388 | 0.030396924 | 0.074222 | 0.6273382 | 0.8222551 | TAF1C | 9013 |
| ENSG00000261286 | 64.91151384 | 0.032406062 | 0.1306918 | 0.5159812 | 0.7453842 | ATP2C2-AS1 | 105371374 |
| ENSG00000140950 | 743.775034 | 0.090602299 | 0.09356 | 0.2092822 | 0.4657317 | MEAK7 | 57707 |
| ENSG00000103187 | 16327.35293 | 0.156503921 | 0.0607127 | 0.004309 | 0.0337851 | COTL1 | 23406 |
| ENSG00000135686 | 1740.635591 | 0.045368423 | 0.0707138 | 0.4545168 | 0.6995929 | KLHL36 | 79786 |
| ENSG00000103194 | 3010.765516 | -0.116072844 | 0.0652413 | 0.0434623 | 0.1766467 | USP10 | 9100 |
| ENSG00000103196 | 16.35645475 | 0.020536904 | 0.1362781 | 0.4350068 | NA | CRISPLD2 | 83716 |
| ENSG00000153786 | 3050.578246 | 0.154820926 | 0.0663027 | 0.0083495 | 0.0559233 | ZDHHC7 | 55625 |
| ENSG00000135709 | 444.8368764 | 0.057440496 | 0.1033253 | 0.4210898 | 0.6736168 | KIAA0513 | 9764 |
| ENSG00000131149 | 442.6315184 | -0.189512559 | 0.1457007 | 0.0400532 | 0.1678126 | GSE1 | 23199 |
| ENSG00000131153 | 1662.121567 | 0.036606844 | 0.0731789 | 0.5547417 | 0.7749708 | GINS2 | 51659 |
| ENSG00000154102 | 34.15473762 | 0.020876792 | 0.13322 | 0.5787273 | NA | C16orf74 | 404550 |
| ENSG00000131148 | 1287.751566 | 0.04968657 | 0.0767413 | 0.436382 | 0.6867931 | EMC8 | 10328 |
| ENSG00000131143 | 5943.082874 | -0.093540704 | 0.0567356 | 0.0691911 | 0.2390478 | COX4I1 | 1327 |
| ENSG00000103248 | 440.8463315 | 0.061950092 | 0.1019336 | 0.3810443 | 0.641901 | MTHFSD | 64779 |
| ENSG00000260944 | 23.20831271 | 0.004188426 | 0.1327987 | 0.8976899 | NA | NA | NA |
| ENSG00000176692 | 291.419961 | 0.298912082 | 0.1829713 | 0.0098075 | 0.0628769 | FOXC2 | 2303 |
| ENSG00000176678 | 662.1753034 | 0.55197189 | 0.115909 | 1.39E-07 | 6.31E-06 | FOXL1 | 2300 |
| ENSG00000260456 | 36.33132549 | 0.017354855 | 0.1318695 | 0.6604312 | NA | C16orf95 | 100506581 |
| ENSG00000103264 | 1260.026933 | 0.122887308 | 0.0835702 | 0.0722745 | 0.245561 | FBXO31 | 79791 |
| ENSG00000140941 | 4303.234674 | 0.20506931 | 0.0646202 | 0.0004382 | 0.0058292 | MAP1LC3B | 81631 |
| ENSG00000140948 | 1333.320581 | -0.055880828 | 0.0786732 | 0.3875817 | 0.6479967 | ZCCHC14 | 23174 |
| ENSG00000260750 | 36.35134453 | -0.050377082 | 0.142788 | 0.2241653 | NA | NA | NA |
| ENSG00000154118 | 9.00178471 | 0.005183981 | 0.1351292 | 0.8077959 | NA | JPH3 | 57338 |
| ENSG00000226180 | 64.40293681 | -0.010088022 | 0.126894 | 0.832581 | 0.9305286 | LOC100129 | 100129215 |
| ENSG00000104731 | 1120.770316 | -0.003734759 | 0.0767409 | 0.9523214 | 0.9806656 | KLHDC4 | 54758 |
| ENSG00000205047 | 269.6327975 | 0.019251074 | 0.1088782 | 0.7755472 | 0.9053735 | NA | NA |
| ENSG00000260671 | 51.5340238 | 0.054538011 | 0.1434996 | 0.2258929 | 0.4868367 | NA | NA |
| ENSG00000103257 | 12862.99284 | 0.698916455 | 0.0539061 | 1.34E-39 | 1.57E-36 | SLC7A5 | 8140 |
| ENSG00000260466 | 14.90791038 | 0.016828309 | 0.1354645 | 0.5287467 | NA | NA | NA |
| ENSG00000172530 | 251.3685537 | 0.638077655 | 0.2131785 | 0.0001457 | 0.0023541 | BANP | 54971 |
| ENSG00000225614 | 96.85155989 | 0.357166764 | 0.3810582 | 0.0129059 | 0.0760998 | ZNF469 | 84627 |
| ENSG00000179588 | 235.866682 | 0.527562283 | 0.2051425 | 0.0005699 | 0.0071193 | ZFPM1 | 161882 |
| ENSG00000158545 | 3870.600888 | 0.094374192 | 0.0619147 | 0.0866938 | 0.2764594 | ZC3H18 | 124245 |
| ENSG00000051523 | 4161.492938 | 0.18167429 | 0.0684714 | 0.0027341 | 0.024017 | CYBA | 1535 |
| ENSG00000167508 | 2276.135847 | -0.209420018 | 0.0839359 | 0.0032743 | 0.0273587 | MVD | 4597 |
| ENSG00000260630 | 70.46595527 | -0.004588827 | 0.1252508 | 0.9259125 | 0.971576 | SNAI3-AS1 | 197187 |
| ENSG00000158717 | 828.9181903 | 0.284690292 | 0.1095464 | 0.0013679 | 0.0143561 | RNF166 | 115992 |
| ENSG00000174177 | 1037.109724 | -0.020385782 | 0.0774887 | 0.748859 | 0.8910484 | CTU2 | 348180 |
| ENSG00000103335 | 11432.35516 | 0.008895982 | 0.0498685 | 0.8483448 | 0.9375782 | PIEZO1 | 9780 |
| ENSG00000260121 | 62.21524948 | -0.014672502 | 0.1287594 | 0.745294 | 0.8893165 | NA | NA |
| ENSG00000224888 | 17.9532202 | 0.048022282 | 0.1459223 | 0.1045951 | NA | NA | NA |
| ENSG00000167513 | 2599.992741 | 0.378905632 | 0.0719742 | 1.62E-08 | 9.26E-07 | CDT1 | 81620 |
| ENSG00000198931 | 2095.809505 | -0.039658096 | 0.0689158 | 0.5043753 | 0.7381117 | APRT | 353 |
| ENSG00000141012 | 1511.813441 | -0.026671681 | 0.0707034 | 0.6592193 | 0.8425803 | GALNS | 2588 |
| ENSG00000167515 | 1793.598149 | 0.110679225 | 0.0752311 | 0.0819401 | 0.2664649 | TRAPPC2L | 51693 |
| ENSG00000176715 | 632.3684864 | 0.151057247 | 0.1086545 | 0.0581635 | 0.2136501 | ACSF3 | 197322 |
| ENSG00000129910 | 8.927907928 | -0.001045165 | 0.1351169 | 0.9563504 | NA | CDH15 | 1013 |
| ENSG00000259877 | 34.45272944 | 0.029330998 | 0.1350372 | 0.4481401 | NA | NA | NA |
| ENSG00000170100 | 430.1562319 | -0.008665656 | 0.0997486 | 0.8980112 | 0.959538 | ZNF778 | 197320 |
| ENSG00000167522 | 7984.474187 | 0.018157922 | 0.0565171 | 0.7197018 | 0.8733734 | ANKRD11 | 29123 |
| ENSG00000261253 | 43.85538555 | -0.015099952 | 0.1313847 | 0.7005737 | 0.86431 | NA | NA |
| ENSG00000197912 | 2672.978415 | 0.037317982 | 0.0671711 | 0.5243052 | 0.7516456 | SPG7 | 6687 |
| ENSG00000167526 | 25427.3982 | -0.042814589 | 0.0514951 | 0.3710158 | 0.6332313 | RPL13 | 6137 |
| ENSG00000178773 | 1095.865479 | 0.025289236 | 0.0781536 | 0.6813285 | 0.8538613 | CPNE7 | 27132 |
| ENSG00000131165 | 5112.77437 | 0.001517046 | 0.0580774 | 0.9773041 | 0.9913391 | CHMP1A | 5119 |
| ENSG00000167523 | 701.4933441 | 0.241035168 | 0.116793 | 0.0069531 | 0.048489 | SPATA33 | 124045 |
| ENSG00000185324 | 1805.384805 | 0.05284368 | 0.0725676 | 0.3918843 | 0.6522832 | CDK10 | 8558 |
| ENSG00000260259 | 16.69198516 | 0.024066415 | 0.137001 | 0.3708144 | NA | NA | NA |
| ENSG00000158792 | 528.3605256 | 0.328784902 | 0.1297978 | 0.0012553 | 0.0134084 | SPATA2L | 124044 |
| ENSG00000075399 | 139.1394701 | 0.017606817 | 0.1199195 | 0.7672964 | 0.9000591 | VPS9D1 | 9605 |
| ENSG00000261373 | 480.4877837 | 0.046491861 | 0.0977595 | 0.5082778 | 0.7401841 | VPS9D1-AS | 100128881 |

| | | | | | | | |
|---|---|---|---|---|---|---|---|
| ENSG00000158805 | 991.7468 | -0.014667239 | 0.0789177 | 0.8192242 | 0.925668 | ZNF276 | 92822 |
| ENSG00000187741 | 3509.577021 | 0.026780236 | 0.0609318 | 0.6239246 | 0.8201157 | FANCA | 2175 |
| ENSG00000204991 | 473.3263482 | 0.061981154 | 0.1028366 | 0.3616325 | 0.625071 | SPIRE2 | 84501 |
| ENSG00000141002 | 3115.54217 | -0.137199441 | 0.0667123 | 0.0192377 | 0.1009503 | TCF25 | 22980 |
| ENSG00000258839 | 176.5994453 | -0.003538149 | 0.112906 | 0.9545406 | 0.9819702 | MC1R | 4157 |
| ENSG00000259006 | 9.248794475 | 0.015677461 | 0.1362876 | 0.4624213 | NA | NA | NA |
| ENSG00000258947 | 164.8135714 | 0.021335135 | 0.1160035 | 0.7362085 | 0.884192 | TUBB3 | 10381 |
| ENSG00000140995 | 3399.57477 | 0.074825813 | 0.0614742 | 0.1698844 | 0.4156722 | DEF8 | 54849 |
| ENSG00000261317 | 11.08812353 | 0.011632122 | 0.1353795 | 0.6157043 | NA | NA | NA |
| ENSG00000177946 | 346.1727546 | 0.104065459 | 0.1279752 | 0.1802411 | 0.4297604 | CENPBD1P | 92806 |
| ENSG00000223959 | 993.1770117 | 0.225084953 | 0.1046688 | 0.0066451 | 0.047015 | AFG3L1P | 172 |
| ENSG00000003249 | 1180.646547 | -0.051413494 | 0.0766334 | 0.4188972 | 0.6717512 | DBNDD1 | 79007 |
| ENSG00000141013 | 1168.98963 | 0.02292893 | 0.0751264 | 0.6989293 | 0.8629314 | GAS8 | 2622 |
| ENSG00000222019 | 9.427564171 | -0.003055991 | 0.1349925 | 0.8837557 | NA | NA | NA |
| ENSG00000261812 | 17.83833078 | 0.01340907 | 0.1346862 | 0.6283367 | NA | TUBB8P7 | 197331 |
| ENSG00000260528 | 29.96066659 | 0.04083855 | 0.1403581 | 0.2543692 | NA | NA | NA |
| ENSG00000181031 | 96.99771853 | -0.047967264 | 0.1312504 | 0.4027621 | 0.6601399 | RPH3AL | 9501 |
| ENSG00000187624 | 12.79548894 | 0.041611453 | 0.1437446 | 0.1009344 | NA | LIAT1 | 400566 |
| ENSG00000183688 | 1696.910868 | -0.132725555 | 0.0769987 | 0.0409409 | 0.1701284 | RFLNB | 359845 |
| ENSG00000141252 | 902.4078479 | -0.030317244 | 0.0810316 | 0.6436368 | 0.8333617 | VPS53 | 55275 |
| ENSG00000167695 | 812.9162218 | 0.130067812 | 0.0963541 | 0.0797068 | 0.2613697 | TLCD3A | 79850 |
| ENSG00000179409 | 1629.889509 | 0.003095139 | 0.0702806 | 0.9598452 | 0.9834728 | GEMIN4 | 50628 |
| ENSG00000231784 | 9.04981456 | 0.01267535 | 0.1359043 | 0.5430309 | NA | DBIL5P | 100131454 |
| ENSG00000167699 | 1648.865276 | -0.018510867 | 0.0711769 | 0.7597923 | 0.8969311 | GLOD4 | 51031 |
| ENSG00000171861 | 845.3497664 | 0.330601798 | 0.1173164 | 0.0005613 | 0.0070278 | MRM3 | 55178 |
| ENSG00000262434 | 8.889344123 | -0.013133133 | 0.1359481 | 0.5284668 | NA | NA | NA |
| ENSG00000167693 | 1794.07994 | -0.146734081 | 0.0787225 | 0.0267544 | 0.1271074 | NXN | 64359 |
| ENSG00000177370 | 855.7486096 | 0.014547924 | 0.0828211 | 0.826147 | 0.9286016 | TIMM22 | 29928 |
| ENSG00000159842 | 2685.918272 | -0.273016763 | 0.0712714 | 2.29E-05 | 0.0005068 | ABR | 29 |
| ENSG00000108953 | 13985.53606 | -0.359155874 | 0.052784 | 1.33E-12 | 1.68E-10 | YWHAE | 7531 |
| ENSG00000167193 | 3506.734936 | -0.091430174 | 0.0606555 | 0.0917851 | 0.2868223 | CRK | 1398 |
| ENSG00000197879 | 8602.642602 | -0.162073941 | 0.0547516 | 0.0011799 | 0.0127731 | MYO1C | 4641 |
| ENSG00000132376 | 512.480232 | 0.017700199 | 0.0937057 | 0.7968621 | 0.9157552 | INPP5K | 51763 |
| ENSG00000236618 | 68.68083062 | 0.035437873 | 0.1308119 | 0.4907035 | 0.7273003 | PITPNA-AS1 | 100306951 |
| ENSG00000174238 | 1658.098781 | -0.028329066 | 0.0703928 | 0.6379876 | 0.8293271 | PITPNA | 5306 |
| ENSG00000167703 | 248.3638017 | 0.051645934 | 0.1142791 | 0.4571138 | 0.7016487 | SLC43A2 | 124935 |
| ENSG00000074660 | 40.30447796 | 0.089399161 | 0.1716172 | 0.0575488 | NA | SCARF1 | 8578 |
| ENSG00000167705 | 302.1634237 | 0.012301098 | 0.1060271 | 0.8562728 | 0.9418544 | RILP | 83547 |
| ENSG00000174231 | 9278.901486 | -0.073449911 | 0.0518824 | 0.1250768 | 0.3471045 | PRPF8 | 10594 |
| ENSG00000185561 | 23.95867981 | -0.003736905 | 0.1324375 | 0.9076306 | NA | TLCD2 | 727910 |
| ENSG00000186594 | 434.832366 | -0.05567196 | 0.1022506 | 0.4323725 | 0.6836144 | MIR22HG | 84981 |
| ENSG00000167716 | 1335.1298 | 0.295544775 | 0.0877848 | 0.0001162 | 0.0019708 | WDR81 | 124997 |
| ENSG00000186532 | 739.8710973 | 0.099807051 | 0.0964796 | 0.1717569 | 0.4179776 | SMYD4 | 114826 |
| ENSG00000132383 | 4426.450905 | -0.080100952 | 0.0597199 | 0.1350161 | 0.3616424 | RPA1 | 6117 |
| ENSG00000108958 | 8.742467689 | 0.003185025 | 0.1350872 | 0.8804174 | NA | NA | NA |
| ENSG00000185924 | 8.630904233 | 0.01253577 | 0.1360374 | 0.5306845 | NA | RTN4RL1 | 146760 |
| ENSG00000262445 | 14.04376666 | -0.007124806 | 0.1343326 | 0.7819299 | NA | NA | NA |
| ENSG00000108963 | 524.530497 | 0.170452092 | 0.12462 | 0.0470882 | 0.1855728 | DPH1 | 1801 |
| ENSG00000177374 | 107.4025948 | -0.003760283 | 0.1205697 | 0.9455588 | 0.9784014 | HIC1 | 3090 |
| ENSG00000070366 | 830.7727401 | -0.155822459 | 0.1022946 | 0.0449343 | 0.1805564 | SMG6 | 23293 |
| ENSG00000167720 | 311.921142 | 0.097759944 | 0.1290596 | 0.1998124 | 0.4534698 | SRR | 63826 |
| ENSG00000262333 | 23.00896026 | -0.02520197 | 0.1358299 | 0.4373146 | NA | NA | NA |
| ENSG00000167721 | 4295.390212 | 0.082846938 | 0.0586743 | 0.117115 | 0.3339413 | TSR1 | 55720 |
| ENSG00000141258 | 1288.7945 | -0.194643395 | 0.0919423 | 0.0094643 | 0.0615017 | SGSM2 | 9905 |
| ENSG00000263345 | 15.30055554 | -0.024810445 | 0.1370936 | 0.3618174 | NA | NA | NA |
| ENSG00000070444 | 423.7141281 | -0.01811939 | 0.0969532 | 0.7911413 | 0.9124528 | MNT | 4335 |
| ENSG00000127804 | 1017.103643 | -0.012240259 | 0.0783848 | 0.848082 | 0.9375782 | METTL16 | 79066 |
| ENSG00000007168 | 3323.727816 | -0.215446667 | 0.0653271 | 0.000261 | 0.0038348 | PAFAH1B1 | 5048 |
| ENSG00000272770 | 8.642239373 | -0.002161863 | 0.1351196 | 0.913043 | NA | NA | NA |
| ENSG00000132361 | 5023.910773 | -0.054278315 | 0.0560732 | 0.2873327 | 0.5571603 | CLUH | 23277 |
| ENSG00000262050 | 16.43448291 | -0.021678298 | 0.136158 | 0.4355791 | NA | NA | NA |
| ENSG00000132359 | 45.64577175 | 0.083773088 | 0.1638268 | 0.0902231 | 0.2836104 | RAP1GAP2 | 23108 |
| ENSG00000197417 | 174.1840024 | -0.068107917 | 0.1303218 | 0.3110382 | 0.581132 | SHPK | 23729 |
| ENSG00000262248 | 10.36053031 | -0.011672646 | 0.1355071 | 0.5986129 | NA | NA | NA |
| ENSG00000040531 | 642.156267 | 0.24734865 | 0.1165614 | 0.0058417 | 0.0426479 | CTNS | 1497 |
| ENSG00000262903 | 19.08983085 | 0.01234607 | 0.1339451 | 0.683019 | NA | NA | NA |
| ENSG00000213977 | 161.6144444 | 0.030192111 | 0.1233395 | 0.6069328 | 0.8114435 | TAX1BP3 | 30851 |
| ENSG00000257950 | 21.30420038 | -0.030437995 | 0.1375489 | 0.3402812 | NA | P2RX5-TAX1 | 100533970 |
| ENSG00000127774 | 809.6372375 | -0.023986487 | 0.0847408 | 0.718007 | 0.8722654 | EMC6 | 83460 |
| ENSG00000083454 | 162.2281673 | -0.009595739 | 0.1159943 | 0.8750356 | 0.9494835 | P2RX5 | 5026 |

| | | | | | | | | |
|---|---|---|---|---|---|---|---|---|
| ENSG00000083457 | 607.1892218 | -0.035895265 | 0.091402 | 0.5998082 | 0.8065338 | ITGAE | | 3682 |
| ENSG00000177602 | 630.8422028 | 0.011182539 | 0.0873534 | 0.8687891 | 0.9471139 | HASPIN | | 83903 |
| ENSG00000262358 | 8.558400563 | 0.014039791 | 0.1363796 | 0.4609077 | NA | NA | NA | |
| ENSG00000262194 | 22.08398345 | -0.02927877 | 0.137086 | 0.3638771 | NA | NA | NA | |
| ENSG00000262758 | 252.2396338 | -0.160229023 | 0.1570756 | 0.0707229 | 0.2420798 | NA | NA | |
| ENSG00000074356 | 1474.932037 | -0.297752433 | 0.0896058 | 0.0001317 | 0.0021819 | NCBP3 | | 55421 |
| ENSG00000004660 | 70.92397466 | -0.04434907 | 0.1334975 | 0.3974681 | 0.6567942 | CAMKK1 | | 84254 |
| ENSG00000074370 | 17.18202605 | 0.014767979 | 0.1346726 | 0.6065781 | NA | ATP2A3 | | 489 |
| ENSG00000074755 | 1533.16796 | 0.066647062 | 0.0740994 | 0.2867597 | 0.5568053 | ZZEF1 | | 23140 |
| ENSG00000167740 | 133.8831908 | 0.053015196 | 0.1272474 | 0.399566 | 0.6582409 | CYB5D2 | | 124936 |
| ENSG00000185722 | 2498.713457 | -0.063191181 | 0.063997 | 0.2624521 | 0.5300012 | ANKFY1 | | 51479 |
| ENSG00000132388 | 1903.810781 | -0.127890675 | 0.0779776 | 0.0505321 | 0.1935431 | UBE2G1 | | 7326 |
| ENSG00000183018 | 934.3316288 | -0.453133279 | 0.1134145 | 5.38E-06 | 0.0001481 | SPNS2 | | 124976 |
| ENSG00000132382 | 3407.636664 | -0.06369445 | 0.0633169 | 0.2559587 | 0.5230675 | MYBBP1A | | 10514 |
| ENSG00000141456 | 2336.375289 | 0.116441201 | 0.0681605 | 0.0500646 | 0.1926868 | PELP1 | | 27043 |
| ENSG00000141480 | 1279.942797 | -0.085168517 | 0.0785179 | 0.1896623 | 0.440534 | ARRB2 | | 409 |
| ENSG00000161920 | 162.3720903 | -0.032100413 | 0.1182847 | 0.6122493 | 0.8144498 | MED11 | | 400569 |
| ENSG00000161921 | 130.3085286 | 0.225078468 | 0.2662356 | 0.0307913 | 0.1406073 | CXCL16 | | 58191 |
| ENSG00000182853 | 43.48810735 | 0.050424739 | 0.1420617 | 0.244089 | 0.5098718 | VMO1 | | 284013 |
| ENSG00000142507 | 2202.400901 | -0.115690616 | 0.07069 | 0.0575735 | 0.212284 | PSMB6 | | 5694 |
| ENSG00000129219 | 827.2538798 | -0.213441577 | 0.1133329 | 0.0128791 | 0.0760183 | PLD2 | | 5338 |
| ENSG00000141503 | 2874.79074 | -0.049256889 | 0.0648928 | 0.3880229 | 0.6484257 | MINK1 | | 50488 |
| ENSG00000205710 | 15.40573461 | 0.019951775 | 0.1360069 | 0.4605502 | NA | C17orf107 | | 100130311 |
| ENSG00000108528 | 713.4285918 | 0.031876595 | 0.0912932 | 0.6437042 | 0.8333617 | SLC25A11 | | 8402 |
| ENSG00000108523 | 1001.826995 | 0.037371113 | 0.0800891 | 0.5612795 | 0.7795039 | RNF167 | | 26001 |
| ENSG00000108518 | 31269.69969 | -0.154399935 | 0.0571287 | 0.0030523 | 0.025987 | PFN1 | | 5216 |
| ENSG00000108515 | 458.856865 | 0.051525761 | 0.0999178 | 0.4665754 | 0.7094916 | ENO3 | | 2027 |
| ENSG00000091640 | 938.730999 | -0.086022181 | 0.0861839 | 0.2088562 | 0.4654027 | SPAG7 | | 9552 |
| ENSG00000108509 | 895.6055738 | 0.062741967 | 0.0867581 | 0.3545139 | 0.6187516 | CAMTA2 | | 23125 |
| ENSG00000262429 | 19.7725821 | -0.009412251 | 0.1335663 | 0.7530339 | NA | NA | NA | |
| ENSG00000262227 | 19.86728593 | 0.035369413 | 0.1399337 | 0.2385958 | NA | NA | NA | |
| ENSG00000196388 | 11.50617397 | 0.023589675 | 0.1376119 | 0.3127785 | NA | INCA1 | | 388324 |
| ENSG00000129250 | 6551.053091 | -0.022396637 | 0.0536922 | 0.6517952 | 0.8386891 | KIF1C | | 10749 |
| ENSG00000227495 | 44.49458636 | 0.042779739 | 0.1379599 | 0.3290603 | 0.5970416 | KIF1C-AS1 | | 102724009 |
| ENSG00000167840 | 365.9150348 | 0.280780779 | 0.1614129 | 0.0094499 | 0.0615017 | ZNF232 | | 7775 |
| ENSG00000234327 | 43.43737698 | 0.042378955 | 0.1379778 | 0.3290532 | 0.5970416 | ZNF232-AS1 | | 101928000 |
| ENSG00000180626 | 207.1616026 | 0.217129555 | 0.1976684 | 0.0340965 | 0.1501235 | ZNF594 | | 84622 |
| ENSG00000261879 | 30.8066924 | 0.051187197 | 0.1445086 | 0.1862898 | NA | ZNF594-DT | | 100130950 |
| ENSG00000029725 | 1608.718649 | -0.06832854 | 0.0750818 | 0.2785515 | 0.5467586 | RABEP1 | | 9135 |
| ENSG00000108559 | 1200.658251 | -0.222021447 | 0.0922816 | 0.0035892 | 0.0293189 | NUP88 | | 4927 |
| ENSG00000129197 | 857.0544729 | 0.081386025 | 0.0877495 | 0.2397934 | 0.505418 | RPAIN | | 84268 |
| ENSG00000263272 | 10.52786126 | -0.014296014 | 0.135784 | 0.5294688 | NA | NA | NA | |
| ENSG00000108561 | 4919.994789 | -0.200335989 | 0.0620423 | 0.0003758 | 0.0051862 | C1QBP | | 708 |
| ENSG00000005100 | 2912.917155 | 0.153301393 | 0.0660675 | 0.0088076 | 0.0583558 | DHX33 | | 56919 |
| ENSG00000072849 | 848.0408032 | -0.02872745 | 0.082654 | 0.6657888 | 0.8452861 | DERL2 | | 51009 |
| ENSG00000167842 | 851.9181768 | 0.164088514 | 0.1099442 | 0.0430382 | 0.1755951 | MIS12 | | 79003 |
| ENSG00000091592 | 50.66524873 | 0.162470547 | 0.2942586 | 0.0146376 | 0.0828001 | NLRP1 | | 22861 |
| ENSG00000179314 | 4231.407357 | -0.184934536 | 0.0617008 | 0.0009265 | 0.0103887 | WSCD1 | | 23302 |
| ENSG00000129195 | 1331.602433 | -0.166094536 | 0.0950855 | 0.027248 | 0.1288076 | PIMREG | | 54478 |
| ENSG00000091622 | 13.29720425 | -0.014298204 | 0.1355616 | 0.5506122 | NA | PITPNM3 | | 83394 |
| ENSG00000198920 | 411.3884806 | 0.034323284 | 0.1020011 | 0.6214572 | 0.818389 | KIAA0753 | | 9851 |
| ENSG00000129235 | 1258.835125 | -0.152264633 | 0.0885679 | 0.0333915 | 0.1478008 | TXNDC17 | | 84817 |
| ENSG00000108590 | 205.7605677 | -0.11185759 | 0.1441965 | 0.1496746 | 0.3849101 | MED31 | | 51003 |
| ENSG00000215067 | 84.90083848 | 0.054238771 | 0.1352179 | 0.3391975 | 0.6066573 | ALOX12-AS1 | | 100506713 |
| ENSG00000262089 | 26.15467234 | 0.060009311 | 0.151203 | 0.0942581 | NA | NA | NA | |
| ENSG00000219200 | 82.47865582 | 0.218936723 | 0.335299 | 0.024871 | 0.1202483 | RNASEK | | 440400 |
| ENSG00000269871 | 33.66065515 | -0.045317462 | 0.1418627 | 0.226133 | NA | NA | NA | |
| ENSG00000258315 | 18.31101128 | -0.001334074 | 0.1333759 | 0.9615026 | NA | C17orf49 | | 124944 |
| ENSG00000267532 | 11.34718025 | 0.005142446 | 0.1349589 | 0.8187802 | NA | MIR497HG | | 100506755 |
| ENSG00000174327 | 80.4199787 | 0.083474277 | 0.1533123 | 0.1655168 | 0.4097476 | SLC16A13 | | 201232 |
| ENSG00000141505 | 16.56664635 | 0.061929305 | 0.1548451 | 0.0364473 | NA | ASGR1 | | 432 |
| ENSG00000132535 | 191.8932452 | 0.01321229 | 0.1153939 | 0.8338906 | 0.9305701 | DLG4 | | 1742 |
| ENSG00000072778 | 4004.077099 | 0.172748866 | 0.0670916 | 0.0037089 | 0.0300649 | ACADVL | | 37 |
| ENSG00000004975 | 983.690903 | 0.136585162 | 0.0912435 | 0.0590829 | 0.2158733 | DVL2 | | 1856 |
| ENSG00000040633 | 1240.888748 | -0.150057517 | 0.0874056 | 0.0344136 | 0.1509518 | PHF23 | | 79142 |
| ENSG00000170296 | 102.4204121 | 0.04146158 | 0.1297274 | 0.4574494 | 0.7017657 | GABARAP | | 11337 |
| ENSG00000175826 | 1553.405812 | -0.064828375 | 0.0736203 | 0.2977858 | 0.5680006 | CTDNEP1 | | 23399 |
| ENSG00000170291 | 732.3194225 | -0.049287975 | 0.0926445 | 0.4768269 | 0.7161907 | ELP5 | | 23587 |
| ENSG00000132507 | 20253.40048 | -0.15368154 | 0.0556137 | 0.0025699 | 0.0228981 | EIF5A | | 1984 |
| ENSG00000132522 | 528.4659271 | -0.134706597 | 0.1280239 | 0.1021929 | 0.3061593 | GPS2 | | 2874 |

| | | | | | | | | |
|---|---|---|---|---|---|---|---|---|
| ENSG00000215041 | 1050.326688 | 0.142852803 | 0.0980127 | 0.0571874 | 0.2115954 | NEURL4 | | 84461 |
| ENSG00000072818 | 16.46345721 | 0.040403854 | 0.1426541 | 0.1421655 | NA | ACAP1 | | 9744 |
| ENSG00000213859 | 391.9662122 | 0.072750603 | 0.1110939 | 0.3198423 | 0.5886556 | KCTD11 | | 147040 |
| ENSG00000174292 | 21.15352786 | 0.023610928 | 0.1356915 | 0.4544036 | NA | TNK1 | | 8711 |
| ENSG00000205544 | 77.08874864 | 0.059820173 | 0.1397691 | 0.2800122 | 0.5487036 | TMEM256 | | 254863 |
| ENSG00000169992 | 811.5550679 | 0.0302417 | 0.0862756 | 0.6539124 | 0.8399571 | NLGN2 | | 57555 |
| ENSG00000181284 | 145.6991174 | 0.010860509 | 0.1165133 | 0.8608504 | 0.9439242 | TMEM102 | | 284114 |
| ENSG00000161958 | 10.31867502 | 0.013097063 | 0.1359425 | 0.534123 | NA | FGF11 | | 2256 |
| ENSG00000170175 | 341.832412 | -0.18761432 | 0.1515823 | 0.0435483 | 0.176812 | CHRNB1 | | 1140 |
| ENSG00000174282 | 998.3148435 | -0.015271677 | 0.0837409 | 0.8169004 | 0.9252377 | ZBTB4 | | 57659 |
| ENSG00000181222 | 7741.611468 | 0.021639385 | 0.0580808 | 0.6813077 | 0.8538613 | POLR2A | | 5430 |
| ENSG00000239697 | 94.8422328 | -0.126335908 | 0.1882443 | 0.0773699 | 0.2565722 | TNFSF12 | | 8742 |
| ENSG00000161956 | 856.3955899 | 0.05655533 | 0.0860122 | 0.4074595 | 0.6638123 | SENP3 | | 26168 |
| ENSG00000265500 | 16.84475861 | -0.004189355 | 0.1336532 | 0.8797793 | NA | NA | NA | |
| ENSG00000161960 | 246.0323284 | -0.138220341 | 0.15009 | 0.0993526 | 0.3007353 | EIF4A1 | | 1973 |
| ENSG00000233223 | 81.46018298 | 0.062928968 | 0.1401453 | 0.2708034 | 0.5386338 | MPDU1-AS1 | | 100996842 |
| ENSG00000129255 | 1161.250115 | -0.013386823 | 0.0750364 | 0.8302624 | 0.9302425 | MPDU1 | | 9526 |
| ENSG00000129194 | 9.975746247 | -0.016393878 | 0.1364332 | 0.4359299 | NA | SOX15 | | 6665 |
| ENSG00000129245 | 1520.585766 | 0.203913674 | 0.0907755 | 0.0064514 | 0.0459736 | FXR2 | | 9513 |
| ENSG00000141504 | 305.7801276 | 0.002055246 | 0.1049278 | 0.9773359 | 0.9913391 | SAT2 | | 112483 |
| ENSG00000141510 | 290.6634677 | 0.008650468 | 0.1067565 | 0.8981105 | 0.9595563 | TP53 | | 7157 |
| ENSG00000262251 | 59.87748798 | -0.016405862 | 0.1281571 | 0.7271399 | 0.8779948 | NA | NA | |
| ENSG00000141499 | 1037.670139 | 0.127922774 | 0.0906172 | 0.0748888 | 0.251156 | WRAP53 | | 55135 |
| ENSG00000108947 | 50.92144222 | 0.002203628 | 0.1285564 | 0.961623 | 0.9843697 | EFNB3 | | 1949 |
| ENSG00000132510 | 548.0378459 | 0.69421085 | 0.1337359 | 1.30E-08 | 7.57E-07 | KDM6B | | 23135 |
| ENSG00000183011 | 522.7135558 | -0.058852606 | 0.0976143 | 0.4048765 | 0.6614808 | NAA38 | | 84316 |
| ENSG00000182224 | 951.4664303 | 0.401901375 | 0.0994274 | 5.26E-06 | 0.0001451 | CYB5D1 | | 124637 |
| ENSG00000170004 | 3178.903903 | -0.101744991 | 0.0654559 | 0.0766869 | 0.2549899 | CHD3 | | 1107 |
| ENSG00000179859 | 86.26413505 | 0.7675048 | 0.3297682 | 0.0007838 | 0.0092112 | RNF227 | | 284023 |
| ENSG00000170049 | 58.02111934 | -0.148643497 | 0.2446749 | 0.0289429 | 0.1350234 | KCNAB3 | | 9196 |
| ENSG00000170043 | 923.3668669 | -0.035246294 | 0.0841727 | 0.5958294 | 0.8037745 | TRAPPC1 | | 58485 |
| ENSG00000170037 | 1843.26997 | -0.130019842 | 0.078378 | 0.0477104 | 0.1867019 | CNTROB | | 116840 |
| ENSG00000179148 | 21.82998231 | 3.647864679 | 0.8279451 | 1.20E-06 | NA | ALOXE3 | | 59344 |
| ENSG00000179111 | 34.6145359 | 0.074407814 | 0.1593477 | 0.0820311 | NA | HES7 | | 84667 |
| ENSG00000179094 | 286.3520199 | 0.154904221 | 0.1478499 | 0.0762149 | 0.254144 | PER1 | | 5187 |
| ENSG00000220205 | 292.520616 | 0.002630348 | 0.104927 | 0.9698539 | 0.9881615 | VAMP2 | | 6844 |
| ENSG00000179029 | 65.37845402 | 0.039577802 | 0.1327118 | 0.4367443 | 0.6870856 | TMEM107 | | 84314 |
| ENSG00000196544 | 147.0056473 | -0.027809071 | 0.1194883 | 0.6465697 | 0.8356474 | BORCS6 | | 54785 |
| ENSG00000178999 | 2057.323907 | 0.023680819 | 0.068645 | 0.6750372 | 0.8507355 | AURKB | | 9212 |
| ENSG00000269928 | 24.45087596 | 0.04335652 | 0.1419233 | 0.2119553 | NA | NA | NA | |
| ENSG00000178971 | 685.1529778 | 0.101786402 | 0.0966075 | 0.165345 | 0.4095409 | CTC1 | | 80169 |
| ENSG00000178921 | 2408.286745 | -0.070376107 | 0.0682479 | 0.2339955 | 0.4966028 | PFAS | | 5198 |
| ENSG00000125434 | 113.5488193 | -0.038679618 | 0.1265259 | 0.504908 | 0.7381515 | SLC25A35 | | 399512 |
| ENSG00000108961 | 395.4784856 | -0.005464298 | 0.0997463 | 0.9361726 | 0.9755069 | RANGRF | | 29098 |
| ENSG00000161970 | 754.2937676 | -0.140619918 | 0.1006693 | 0.0651248 | 0.2303746 | RPL26 | | 6154 |
| ENSG00000166579 | 837.0353189 | 0.049405968 | 0.0863932 | 0.4664381 | 0.7094916 | NDEL1 | | 81565 |
| ENSG00000133026 | 3151.076811 | -0.274702943 | 0.067421 | 8.30E-06 | 0.0002146 | MYH10 | | 4628 |
| ENSG00000065320 | 17.26500497 | 0.008896779 | 0.1341066 | 0.7511202 | NA | NTN1 | | 9423 |
| ENSG00000170310 | 216.9324351 | -0.095048065 | 0.1329848 | 0.2021457 | 0.4562828 | STX8 | | 9482 |
| ENSG00000271851 | 29.74098048 | 0.040144488 | 0.139602 | 0.2789548 | NA | NA | NA | |
| ENSG00000154914 | 316.1711151 | -0.109529814 | 0.1259009 | 0.1626301 | 0.4047906 | USP43 | | 124739 |
| ENSG00000109063 | 45.78022131 | 0.049316181 | 0.141522 | 0.2520249 | 0.5184753 | MYH3 | | 4621 |
| ENSG00000273290 | 157.7971064 | -0.030165015 | 0.1176487 | 0.6331444 | 0.8252838 | NA | NA | |
| ENSG00000133028 | 663.5506772 | 0.121384572 | 0.104618 | 0.1126077 | 0.325537 | SCO1 | | 6341 |
| ENSG00000170222 | 86.65541572 | 0.5490799 | 0.3751705 | 0.0048184 | 0.037029 | ADPRM | | 56985 |
| ENSG00000154957 | 114.8952835 | 0.00065794 | 0.1207455 | 0.9894587 | 0.9955163 | ZNF18 | | 7566 |
| ENSG00000065559 | 622.2004991 | -0.251879533 | 0.1198763 | 0.0058475 | 0.0426637 | MAP2K4 | | 6416 |
| ENSG00000006740 | 380.2033997 | 0.003083055 | 0.1003934 | 0.964762 | 0.9859238 | ARHGAP44 | | 9912 |
| ENSG00000006744 | 2659.693982 | -0.117198097 | 0.0666124 | 0.0447657 | 0.1800646 | ELAC2 | | 60528 |
| ENSG00000236088 | 115.5987137 | 0.150628191 | 0.2021348 | 0.0645702 | 0.2289057 | COX10-DT | | 100874058 |
| ENSG00000006695 | 349.4504182 | 0.034237481 | 0.1034024 | 0.6220692 | 0.8187116 | COX10 | | 1352 |
| ENSG00000231595 | 24.64898736 | 0.036942353 | 0.1392549 | 0.2813918 | NA | NA | NA | |
| ENSG00000109099 | 521.5492917 | 0.421996705 | 0.1318482 | 0.0001156 | 0.0019652 | PMP22 | | 5376 |
| ENSG00000175106 | 32.36008398 | 0.016897441 | 0.1323456 | 0.6558648 | NA | TVP23C | | 201158 |
| ENSG00000221926 | 62.97932158 | 0.006977606 | 0.1264257 | 0.88658 | 0.9545517 | TRIM16 | | 10626 |
| ENSG00000187607 | 175.0030326 | 0.029128968 | 0.1170318 | 0.6485297 | 0.836587 | ZNF286A | | 57335 |
| ENSG00000179277 | 83.03138906 | -0.04880146 | 0.1332905 | 0.3846527 | 0.6452718 | NA | NA | |
| ENSG00000170425 | 1049.030088 | 0.008178051 | 0.0800052 | 0.9005258 | 0.9610169 | ADORA2B | | 136 |
| ENSG00000214941 | 205.8395148 | 0.010576167 | 0.1142833 | 0.868091 | 0.9471139 | ZSWIM7 | | 125150 |
| ENSG00000011295 | 1402.994579 | -0.009610889 | 0.0722436 | 0.8746796 | 0.9493711 | TTC19 | | 54902 |

| | | | | | | | | |
|---|---|---|---|---|---|---|---|---|
| ENSG00000141027 | 3270.339476 | -0.187777329 | 0.0724521 | 0.0030504 | 0.025987 | NCOR1 | | 9611 |
| ENSG00000108474 | 224.5173687 | 0.068923053 | 0.1217046 | 0.3318493 | 0.5992396 | PIGL | | 9487 |
| ENSG00000166582 | 459.2399564 | 0.284901339 | 0.1407151 | 0.0053975 | 0.0401582 | CENPV | | 201161 |
| ENSG00000170315 | 12546.10382 | -0.108547031 | 0.0545432 | 0.0290785 | 0.1354871 | UBB | | 7314 |
| ENSG00000265401 | 145.7692673 | -0.091248068 | 0.150576 | 0.1744166 | 0.4222509 | NA | NA | |
| ENSG00000187688 | 17.05093091 | 0.055683179 | 0.1506359 | 0.0552324 | NA | TRPV2 | | 51393 |
| ENSG00000175061 | 4866.075124 | 0.277176846 | 0.0712034 | 1.77E-05 | 0.0004098 | SNHG29 | | 125144 |
| ENSG00000181350 | 69.49676194 | -0.010767443 | 0.1265202 | 0.8243454 | 0.9276013 | LRRC75A | | 388341 |
| ENSG00000197566 | 130.910935 | 0.037207178 | 0.1234402 | 0.5405917 | 0.7640695 | ZNF624 | | 57547 |
| ENSG00000133030 | 4991.464623 | 0.081068229 | 0.0584526 | 0.1246478 | 0.3465814 | MPRIP | | 23164 |
| ENSG00000179598 | 207.2187196 | 1.268347919 | 0.2035743 | 2.35E-11 | 2.32E-09 | PLD6 | | 201164 |
| ENSG00000154803 | 881.2661211 | 0.327432331 | 0.1077428 | 0.0002894 | 0.0041889 | FLCN | | 201163 |
| ENSG00000141030 | 2134.997556 | -0.095262611 | 0.0684523 | 0.1085641 | 0.3187877 | COPS3 | | 8533 |
| ENSG00000205309 | 25.9518791 | 0.049433173 | 0.1453649 | 0.1430721 | NA | NT5M | | 56953 |
| ENSG00000215030 | 81.01725652 | -0.04714367 | 0.135952 | 0.3536206 | 0.6187516 | RPL13P12 | | 388344 |
| ENSG00000141026 | 325.1936848 | 0.078877472 | 0.1153008 | 0.286187 | 0.5561387 | MED9 | | 55090 |
| ENSG00000133027 | 343.7384106 | -0.057276594 | 0.1075375 | 0.4202684 | 0.6729863 | PEMT | | 10400 |
| ENSG00000108557 | 1353.056052 | -0.184775919 | 0.0862592 | 0.0098472 | 0.063028 | RAI1 | | 10743 |
| ENSG00000072310 | 1741.71331 | -0.466149566 | 0.1759751 | 0.0005348 | 0.0067838 | SREBF1 | | 6720 |
| ENSG00000175662 | 612.8731332 | 0.258701538 | 0.119953 | 0.0049316 | 0.0376753 | TOM1L2 | | 146691 |
| ENSG00000171962 | 28.11972136 | 0.075254976 | 0.1623898 | 0.0502768 | NA | DRC3 | | 83450 |
| ENSG00000171953 | 223.5423673 | -0.011055174 | 0.1091225 | 0.8657603 | 0.9458332 | ATPAF2 | | 91647 |
| ENSG00000141034 | 197.368553 | 0.012987022 | 0.1114679 | 0.8427431 | 0.9350382 | GID4 | | 79018 |
| ENSG00000108591 | 861.1880327 | 0.141415872 | 0.0983891 | 0.0611083 | 0.2205456 | DRG2 | | 1819 |
| ENSG00000091542 | 1753.320756 | -0.024614784 | 0.0685039 | 0.6814095 | 0.8538613 | ALKBH5 | | 54890 |
| ENSG00000131899 | 1380.226206 | -0.149042886 | 0.083188 | 0.0302473 | 0.1391865 | LLGL1 | | 3996 |
| ENSG00000177731 | 5463.863497 | -0.032148356 | 0.0551066 | 0.5234899 | 0.7509978 | FLII | | 2314 |
| ENSG00000177427 | 256.4981658 | 0.192784498 | 0.1699411 | 0.0442174 | 0.1785355 | MIEF2 | | 125170 |
| ENSG00000177302 | 1682.31706 | 0.32639066 | 0.0827905 | 1.10E-05 | 0.0002741 | TOP3A | | 7156 |
| ENSG00000176994 | 784.4413396 | 0.151005785 | 0.0995062 | 0.0482309 | 0.1880457 | SMCR8 | | 140775 |
| ENSG00000267350 | 435.0341991 | 0.171616583 | 0.1304708 | 0.0495301 | 0.1914498 | NA | NA | |
| ENSG00000176974 | 1427.2164 | -0.003892338 | 0.0710128 | 0.9759633 | 0.9911001 | SHMT1 | | 6470 |
| ENSG00000174977 | 210.4660225 | -0.062933492 | 0.1238489 | 0.3576281 | 0.6209582 | NA | NA | |
| ENSG00000249459 | 61.22539562 | 0.095732455 | 0.1700083 | 0.0919573 | 0.2872067 | NA | NA | |
| ENSG00000264885 | 71.47844473 | 0.160367673 | 0.2493219 | 0.0360222 | 0.1561316 | NA | NA | |
| ENSG00000240445 | 53.57707632 | 0.127597928 | 0.2125596 | 0.0373715 | 0.1600793 | FOXO3B | | 2310 |
| ENSG00000224126 | 9.753115316 | -0.016519336 | 0.1363697 | 0.444089 | NA | NA | NA | |
| ENSG00000108448 | 71.49253351 | 0.009827112 | 0.1259898 | 0.8446955 | 0.936404 | NA | NA | |
| ENSG00000171928 | 476.8656247 | 0.137368012 | 0.1247175 | 0.095053 | 0.2927871 | TVP23B | | 51030 |
| ENSG00000141127 | 534.6229906 | -0.064691889 | 0.0975935 | 0.3622297 | 0.6255478 | PRPSAP2 | | 5636 |
| ENSG00000188522 | 1228.811458 | 0.439505927 | 0.1011262 | 1.25E-06 | 4.07E-05 | FAM83G | | 644815 |
| ENSG00000228157 | 16.77164674 | -0.001530483 | 0.134248 | 0.9488081 | NA | NA | NA | |
| ENSG00000072134 | 901.7144511 | 0.075682535 | 0.085739 | 0.2687672 | 0.5365697 | EPN2 | | 22905 |
| ENSG00000265263 | 14.43460567 | 0.017706422 | 0.1357822 | 0.4946126 | NA | NA | NA | |
| ENSG00000108641 | 158.9820874 | 0.023247906 | 0.1170894 | 0.7111553 | 0.8691154 | B9D1 | | 27077 |
| ENSG00000166484 | 418.8919186 | -0.029598284 | 0.0987799 | 0.6674701 | 0.8468251 | MAPK7 | | 5598 |
| ENSG00000189343 | 35.03248153 | -0.047749615 | 0.1418282 | 0.2385913 | NA | RPS2P46 | | 125208 |
| ENSG00000072210 | 750.4023177 | -0.418953122 | 0.1272702 | 8.51E-05 | 0.0015361 | ALDH3A2 | | 224 |
| ENSG00000108599 | 580.8418576 | 0.008785246 | 0.0903653 | 0.8978464 | 0.9594787 | AKAP10 | | 11216 |
| ENSG00000128487 | 733.8217802 | 0.135957885 | 0.100545 | 0.0740024 | 0.2491846 | SPECC1 | | 92521 |
| ENSG00000154898 | 18.55161942 | 0.022173929 | 0.1359567 | 0.452366 | NA | NA | NA | |
| ENSG00000214832 | 11.79489833 | 0.013229606 | 0.1355741 | 0.5699317 | NA | NA | NA | |
| ENSG00000189423 | 13.83023786 | -0.017622569 | 0.1357929 | 0.4918607 | NA | NA | NA | |
| ENSG00000233098 | 9.506974062 | -0.017655949 | 0.1367609 | 0.3903922 | NA | LOC339260 | | 339260 |
| ENSG00000124422 | 5087.700257 | -0.079653189 | 0.0575755 | 0.1264573 | 0.3490637 | USP22 | | 23326 |
| ENSG00000263986 | 14.32536158 | -0.017343003 | 0.1356 | 0.5087233 | NA | NA | NA | |
| ENSG00000109016 | 290.1379188 | 0.06583917 | 0.1139383 | 0.3592482 | 0.6224233 | DHRS7B | | 25979 |
| ENSG00000178307 | 703.1847924 | 0.157480529 | 0.1098178 | 0.0505356 | 0.1935431 | TMEM11 | | 8834 |
| ENSG00000235530 | 15.36212506 | -0.00944638 | 0.1344109 | 0.7207258 | NA | TMEM11-DT | | 107984969 |
| ENSG00000154035 | 270.4373331 | 0.297843112 | 0.193616 | 0.0113153 | 0.0693829 | NA | NA | |
| ENSG00000034152 | 1697.82623 | 0.036106267 | 0.0693066 | 0.5463103 | 0.7682016 | MAP2K3 | | 5606 |
| ENSG00000109046 | 1547.163283 | -0.134596935 | 0.0799611 | 0.0435428 | 0.176812 | WSB1 | | 26118 |
| ENSG00000264469 | 30.31499224 | -0.014183235 | 0.1325405 | 0.6884298 | NA | NA | NA | |
| ENSG00000265683 | 11.58151023 | -0.003285747 | 0.1345928 | 0.8869938 | NA | SYPL1P2 | | 100132972 |
| ENSG00000141068 | 419.5331572 | 0.106078992 | 0.1162808 | 0.1688863 | 0.4145971 | KSR1 | | 8844 |
| ENSG00000232859 | 13.39992588 | -0.005943433 | 0.1347588 | 0.7969275 | NA | LYRM9 | | 201229 |
| ENSG00000087095 | 418.3280354 | 0.009510599 | 0.097088 | 0.8903793 | 0.9554911 | NLK | | 51701 |
| ENSG00000109084 | 1011.464733 | -0.129398098 | 0.0947692 | 0.0778065 | 0.2571988 | TMEM97 | | 27346 |
| ENSG00000109083 | 266.6815254 | 0.038852682 | 0.10953 | 0.5678821 | 0.7838155 | IFT20 | | 90410 |
| ENSG00000109079 | 1999.289805 | 0.081402548 | 0.0692901 | 0.1741524 | 0.4218736 | TNFAIP1 | | 7126 |

| | | | | | | | | |
|---|---|---|---|---|---|---|---|---|
| ENSG00000004142 | 2064.06972 | 0.070171187 | 0.0696534 | 0.2421409 | 0.5078915 | POLDIP2 | | 26073 |
| ENSG00000244045 | 389.1566376 | 0.165058981 | 0.1365538 | 0.0596256 | 0.2171093 | TMEM199 | | 147007 |
| ENSG00000265618 | 11.63901475 | 0.020028091 | 0.1367515 | 0.3949456 | NA | NA | NA | |
| ENSG00000004139 | 104.2891732 | -0.023131824 | 0.1230907 | 0.6831803 | 0.8545972 | SARM1 | | 23098 |
| ENSG00000076351 | 452.8610496 | -0.287027308 | 0.1414336 | 0.0052574 | 0.0395188 | SLC46A1 | | 113235 |
| ENSG00000258472 | 72.86834594 | -0.053455714 | 0.1383639 | 0.3046286 | 0.5735672 | NA | NA | |
| ENSG00000183405 | 17.08006071 | -0.006046249 | 0.1337135 | 0.8297225 | NA | NA | NA | |
| ENSG00000109103 | 485.3791414 | -0.1250361 | 0.1145065 | 0.1139528 | 0.3281578 | UNC119 | | 9094 |
| ENSG00000087111 | 830.593082 | -0.095303879 | 0.0903151 | 0.1751468 | 0.4234507 | PIGS | | 94005 |
| ENSG00000109107 | 31.34779432 | 0.000555412 | 0.1314488 | 0.990316 | NA | ALDOC | | 230 |
| ENSG00000076382 | 2921.900099 | -0.295836154 | 0.0710206 | 5.00E-06 | 0.0001403 | SPAG5 | | 10615 |
| ENSG00000227543 | 50.65001653 | -0.010593174 | 0.1292439 | 0.8052581 | 0.9186624 | NA | NA | |
| ENSG00000264608 | 19.42577088 | 0.030661966 | 0.1388479 | 0.2645915 | NA | NA | NA | |
| ENSG00000167524 | 36.56721856 | -0.021956952 | 0.1328556 | 0.5728629 | NA | RSKR | | 124923 |
| ENSG00000007202 | 7558.571866 | -0.123406027 | 0.0560285 | 0.0157387 | 0.0871299 | BLTP2 | | 9703 |
| ENSG00000264044 | 38.02665137 | -0.041552699 | 0.1394634 | 0.2870955 | NA | NA | NA | |
| ENSG00000132581 | 763.0397732 | -0.049345734 | 0.0870208 | 0.4634197 | 0.7077937 | SDF2 | | 6388 |
| ENSG00000109111 | 4636.150522 | 0.086825514 | 0.0589323 | 0.101902 | 0.3056014 | SUPT6H | | 6830 |
| ENSG00000167525 | 29.10574585 | 0.050694847 | 0.1445581 | 0.1809958 | NA | PROCA1 | | 147011 |
| ENSG00000109113 | 2200.017638 | -0.118536812 | 0.0701621 | 0.0506921 | 0.1940151 | RAB34 | | 83871 |
| ENSG00000198242 | 10651.79894 | 0.049930273 | 0.0548471 | 0.3183398 | 0.5871795 | RPL23A | | 6147 |
| ENSG00000264577 | 55.22669172 | -0.013543938 | 0.1282866 | 0.7676902 | 0.9003403 | NA | NA | |
| ENSG00000238578 | 26.38988686 | -0.02819169 | 0.1366313 | 0.3884518 | NA | SNORD4A | | 26773 |
| ENSG00000160606 | 109.5784795 | 0.008628714 | 0.1207276 | 0.8805527 | 0.9518649 | TLCD1 | | 116238 |
| ENSG00000160602 | 110.4702849 | -0.009577846 | 0.120633 | 0.868344 | 0.9471139 | NEK8 | | 284086 |
| ENSG00000076604 | 1813.547162 | 0.042725903 | 0.0683486 | 0.4720927 | 0.7123777 | TRAF4 | | 9618 |
| ENSG00000173065 | 340.1842288 | -0.016381558 | 0.1022629 | 0.8091922 | 0.9208158 | FAM222B | | 55731 |
| ENSG00000132591 | 1347.313812 | 0.114027765 | 0.0841048 | 0.094805 | 0.2921174 | ERAL1 | | 26284 |
| ENSG00000132589 | 1645.84048 | 0.016299209 | 0.0708453 | 0.7886574 | 0.9116361 | FLOT2 | | 2319 |
| ENSG00000167536 | 198.1512352 | 0.072619935 | 0.128109 | 0.2987991 | 0.5689115 | DHRS13 | | 147015 |
| ENSG00000109118 | 1280.781186 | -0.040088376 | 0.0742196 | 0.5202508 | 0.7486818 | PHF12 | | 57649 |
| ENSG00000196535 | 818.6957438 | -0.390759729 | 0.107409 | 2.70E-05 | 0.0005832 | MYO18A | | 399687 |
| ENSG00000221995 | 33.51774012 | -0.044388546 | 0.1406181 | 0.259023 | NA | NA | NA | |
| ENSG00000108256 | 2682.027604 | -0.127666731 | 0.0674906 | 0.030662 | 0.1402362 | NUFIP2 | | 57532 |
| ENSG00000160551 | 2760.519893 | -0.29532155 | 0.0705936 | 4.55E-06 | 0.0001293 | TAOK1 | | 57551 |
| ENSG00000168792 | 352.1303535 | -0.000322054 | 0.0999043 | 0.9945393 | 0.9977447 | ABHD15 | | 116236 |
| ENSG00000167543 | 484.484913 | -0.086781878 | 0.1044512 | 0.2401412 | 0.5054546 | TP53I13 | | 90313 |
| ENSG00000264290 | 9.709428019 | 0.015661531 | 0.1361803 | 0.4756176 | NA | NA | NA | |
| ENSG00000108262 | 1764.994558 | 0.021384335 | 0.0681063 | 0.7192232 | 0.8731289 | GIT1 | | 28964 |
| ENSG00000198720 | 604.4805141 | 0.070006385 | 0.0963434 | 0.3261572 | 0.5942836 | ANKRD13B | | 124930 |
| ENSG00000264647 | 12.15407976 | 0.014317991 | 0.1354901 | 0.5584921 | NA | NA | NA | |
| ENSG00000167549 | 70.73177872 | 0.1332711 | 0.2091686 | 0.0511907 | 0.1950344 | CORO6 | | 84940 |
| ENSG00000141298 | 1100.145147 | 0.072357397 | 0.081049 | 0.2761717 | 0.5444212 | SSH2 | | 85464 |
| ENSG00000176927 | 18.76600811 | 0.020653167 | 0.1355464 | 0.4855072 | NA | EFCAB5 | | 374786 |
| ENSG00000126653 | 795.8468225 | 0.191014097 | 0.1037485 | 0.0175724 | 0.0947659 | NSRP1 | | 84081 |
| ENSG00000108578 | 840.4206774 | -0.22948348 | 0.1041744 | 0.0056232 | 0.0413368 | BLMH | | 642 |
| ENSG00000108582 | 1948.390708 | -0.212336707 | 0.0778775 | 0.0016863 | 0.0167357 | CPD | | 1362 |
| ENSG00000108587 | 1096.17776 | 0.122802 | 0.0891158 | 0.0835715 | 0.2700934 | GOSR1 | | 9527 |
| ENSG00000214719 | 9.845378929 | 0.038343773 | 0.1429763 | 0.0811919 | NA | NA | NA | |
| ENSG00000248121 | 69.80782591 | -0.004221762 | 0.1261197 | 0.9291279 | 0.9726302 | NA | NA | |
| ENSG00000264943 | 10.59584102 | 0.007144377 | 0.1349462 | 0.7573435 | NA | NA | NA | |
| ENSG00000250462 | 185.4834575 | -0.07689026 | 0.1298501 | 0.2749298 | 0.5433015 | NA | NA | |
| ENSG00000264538 | 240.2307054 | 0.038390647 | 0.1165112 | 0.5611252 | 0.7795039 | NA | NA | |
| ENSG00000176390 | 850.5396531 | -0.043875884 | 0.0848362 | 0.5132468 | 0.743625 | CRLF3 | | 51379 |
| ENSG00000266490 | 26.98333653 | 0.031077228 | 0.1372996 | 0.3551715 | NA | NA | NA | |
| ENSG00000176208 | 897.9449955 | 0.376753088 | 0.1095153 | 5.99E-05 | 0.0011492 | ATAD5 | | 79915 |
| ENSG00000172171 | 239.1288431 | -0.031561033 | 0.1110986 | 0.6334145 | 0.8254954 | TEFM | | 79736 |
| ENSG00000184060 | 102.4606328 | -0.118674744 | 0.1767546 | 0.0954778 | 0.2936714 | ADAP2 | | 55803 |
| ENSG00000181481 | 139.2980061 | 0.249958112 | 0.2718365 | 0.0253323 | 0.1221238 | RNF135 | | 84282 |
| ENSG00000264456 | 10.72328077 | -0.001199824 | 0.1346738 | 0.9558866 | NA | NA | NA | |
| ENSG00000266865 | 21.59924862 | 0.008256715 | 0.1330621 | 0.7966955 | NA | NA | NA | |
| ENSG00000196712 | 3150.366611 | -0.206616636 | 0.0719569 | 0.001133 | 0.0123232 | NF1 | | 4763 |
| ENSG00000131242 | 158.963741 | 0.527670681 | 0.2432093 | 0.0015152 | 0.0155284 | RAB11FIP4 | | 84440 |
| ENSG00000172301 | 1089.855924 | -0.025494413 | 0.0781717 | 0.6904792 | 0.8589854 | COPRS | | 55352 |
| ENSG00000108651 | 1558.11067 | -0.085412765 | 0.0765309 | 0.1820367 | 0.4317028 | UTP6 | | 55813 |
| ENSG00000178691 | 3127.587107 | -0.093535754 | 0.0646938 | 0.1002964 | 0.3025862 | SUZ12 | | 23512 |
| ENSG00000185158 | 146.8311386 | 0.023699569 | 0.1194963 | 0.6974408 | 0.8620332 | LRRC37B | | 114659 |
| ENSG00000266777 | 15.83731611 | 0.018812833 | 0.1356129 | 0.4988793 | NA | NA | NA | |
| ENSG00000126858 | 435.7406621 | -0.155003665 | 0.1251314 | 0.0660437 | 0.2322697 | RHOT1 | | 55288 |
| ENSG00000108666 | 266.5799685 | -0.036233368 | 0.1097131 | 0.594989 | 0.8031885 | C17orf75 | | 64149 |

| | | | | | | | | |
|---|---|---|---|---|---|---|---|---|
| ENSG00000010244 | 3664.302195 | -0.141612848 | 0.0637338 | 0.0125289 | 0.074745 | ZNF207 | | 7756 |
| ENSG00000108671 | 4142.406754 | -0.172367395 | 0.0613522 | 0.0018684 | 0.0180223 | PSMD11 | | 5717 |
| ENSG00000176749 | 176.6896647 | -0.009880202 | 0.1149861 | 0.8731939 | 0.9487142 | CDK5R1 | | 8851 |
| ENSG00000176658 | 613.7294382 | 0.006492021 | 0.0887757 | 0.9268622 | 0.9718632 | MYO1D | | 4642 |
| ENSG00000006042 | 40.10125048 | 0.018605414 | 0.1319384 | 0.6407905 | NA | TMEM98 | | 26022 |
| ENSG00000226049 | 14.07135061 | 0.007124132 | 0.1343751 | 0.785326 | NA | TLK2P1 | | 646202 |
| ENSG00000108691 | 303.6263493 | -0.103290179 | 0.1884881 | 0.0318054 | 0.1434436 | CCL2 | | 6347 |
| ENSG00000198783 | 321.1774953 | -0.032067956 | 0.1047299 | 0.6405598 | 0.8315061 | ZNF830 | | 91603 |
| ENSG00000005156 | 669.1199305 | -0.272560679 | 0.115126 | 0.0026922 | 0.0237697 | LIG3 | | 3980 |
| ENSG00000224113 | 185.0200187 | -0.311118953 | 0.2297186 | 0.0126912 | 0.0752788 | NA | NA | |
| ENSG00000092871 | 506.1288962 | 0.065360803 | 0.1003142 | 0.3618888 | 0.6252459 | RFFL | | 117584 |
| ENSG00000185379 | 231.6889092 | 0.038081932 | 0.1118619 | 0.5755867 | 0.7892275 | RAD51D | | 5892 |
| ENSG00000073536 | 1053.514029 | 0.220372314 | 0.0974823 | 0.0053495 | 0.0399537 | NLE1 | | 54475 |
| ENSG00000166750 | 401.8176592 | 1.207484343 | 0.1435533 | 2.22E-18 | 5.40E-16 | SLFN5 | | 162394 |
| ENSG00000172716 | 765.3041834 | 0.307977721 | 0.12127 | 0.0013798 | 0.0144167 | SLFN11 | | 91607 |
| ENSG00000267547 | 21.21396857 | 0.020710949 | 0.1352855 | 0.5002475 | NA | NA | NA | |
| ENSG00000267745 | 47.51559509 | 0.00376217 | 0.1286224 | 0.9331159 | 0.9743454 | NA | NA | |
| ENSG00000267648 | 13.45126445 | 0.003312843 | 0.1342953 | 0.8975163 | NA | NA | NA | |
| ENSG00000267102 | 60.31078925 | -0.04191807 | 0.1345445 | 0.3939058 | 0.6544181 | NA | NA | |
| ENSG00000172123 | 618.922849 | -0.060370843 | 0.0943892 | 0.3896002 | 0.6499457 | SLFN12 | | 55106 |
| ENSG00000154760 | 221.8881995 | 0.112605831 | 0.1409156 | 0.1512821 | 0.3877924 | SLFN13 | | 146857 |
| ENSG00000267321 | 232.7875655 | 0.102309543 | 0.1341848 | 0.1823731 | 0.4320218 | NA | NA | |
| ENSG00000108733 | 73.33587445 | 0.097591954 | 0.1675564 | 0.1083305 | 0.318502 | PEX12 | | 5193 |
| ENSG00000006125 | 7077.650103 | -0.089748154 | 0.0597974 | 0.0946444 | 0.2918558 | AP2B1 | | 163 |
| ENSG00000141150 | 8.742736612 | 0.031529648 | 0.1406357 | 0.1163062 | NA | NA | NA | |
| ENSG00000172660 | 6093.130978 | -0.153871287 | 0.0576544 | 0.0033774 | 0.0279795 | NA | NA | |
| ENSG00000161570 | 27.25205047 | 1.377809877 | 0.6260205 | 0.0008913 | NA | NA | NA | |
| ENSG00000187456 | 13.38304901 | 0.009433974 | 0.1348835 | 0.7003501 | NA | NA | NA | |
| ENSG00000108278 | 513.8615354 | -0.003816582 | 0.0923416 | 0.954009 | 0.9818307 | NA | NA | |
| ENSG00000141140 | 2956.938476 | 0.016822839 | 0.0602326 | 0.7545293 | 0.8940614 | NA | NA | |
| ENSG00000184886 | 951.4658311 | 0.087301298 | 0.0860834 | 0.204403 | 0.4594559 | NA | NA | |
| ENSG00000005955 | 2064.870746 | 0.008845935 | 0.0671592 | 0.8742406 | 0.9491371 | NA | NA | |
| ENSG00000108272 | 320.6129386 | -0.029154664 | 0.108233 | 0.665921 | 0.8452861 | NA | NA | |
| ENSG00000129282 | 371.5112365 | 0.227277876 | 0.1506524 | 0.0210274 | 0.1073518 | NA | NA | |
| ENSG00000255509 | 187.339514 | 0.2286092 | 0.2146483 | 0.0305692 | 0.1401263 | NA | NA | |
| ENSG00000132130 | 185.9235833 | 0.25340658 | 0.2261588 | 0.023797 | 0.1168136 | NA | NA | |
| ENSG00000108270 | 2215.270323 | 0.083162284 | 0.067473 | 0.1577321 | 0.3975891 | NA | NA | |
| ENSG00000132142 | 2967.2993 | -0.119024738 | 0.0659505 | 0.0402842 | 0.1683264 | NA | NA | |
| ENSG00000108264 | 309.4783829 | -0.102146821 | 0.1253449 | 0.1853727 | 0.4357694 | NA | NA | |
| ENSG00000161326 | 942.9147413 | 0.097703185 | 0.0871922 | 0.159343 | 0.4001903 | NA | NA | |
| ENSG00000006114 | 889.3472746 | 0.004567471 | 0.0833972 | 0.9457529 | 0.9784014 | NA | NA | |
| ENSG00000141141 | 2067.777191 | -0.148157081 | 0.075377 | 0.0212356 | 0.1081785 | NA | NA | |
| ENSG00000108753 | 1610.706552 | -0.294758065 | 0.0846794 | 7.69E-05 | 0.0014108 | NA | NA | |
| ENSG00000259549 | 36.6657431 | 0.019650484 | 0.1323298 | 0.6191807 | NA | NA | NA | |
| ENSG00000185128 | 32.74501736 | 0.036454778 | 0.1374639 | 0.3474232 | NA | NA | NA | |
| ENSG00000197681 | 11.18245483 | 0.032608495 | 0.1403992 | 0.1651264 | NA | NA | NA | |
| ENSG00000174093 | 200.9416756 | -0.113255587 | 0.147138 | 0.1441022 | 0.3763992 | NA | NA | |
| ENSG00000174100 | 770.7032511 | -0.182992285 | 0.1035105 | 0.021418 | 0.10887 | NA | NA | |
| ENSG00000174111 | 188.3797663 | 0.016715141 | 0.1149677 | 0.7926875 | 0.9131541 | NA | NA | |
| ENSG00000225485 | 2800.868859 | -0.108905947 | 0.0718659 | 0.0770045 | 0.2559001 | NA | NA | |
| ENSG00000260833 | 73.31274528 | 0.278522143 | 0.4778848 | 0.016617 | 0.0905334 | NA | NA | |
| ENSG00000017373 | 25.57194961 | -0.021825949 | 0.1348014 | 0.5089768 | NA | NA | NA | |
| ENSG00000179294 | 416.604254 | 0.375019948 | 0.1651075 | 0.0019209 | 0.0183595 | NA | NA | |
| ENSG00000261005 | 25.91469703 | 0.075519367 | 0.1631295 | 0.0432465 | NA | NA | NA | |
| ENSG00000108292 | 1966.977693 | -0.004815059 | 0.0670831 | 0.9334727 | 0.974432 | NA | NA | |
| ENSG00000230055 | 484.1192288 | -0.007918522 | 0.0929458 | 0.904328 | 0.9622177 | NA | NA | |
| ENSG00000056661 | 716.1970859 | -0.048895686 | 0.0901984 | 0.4764266 | 0.7158655 | NA | NA | |
| ENSG00000108294 | 2916.181744 | -0.077661896 | 0.0662169 | 0.1794377 | 0.4288613 | NA | NA | |
| ENSG00000141720 | 1685.435414 | -0.21862981 | 0.0798306 | 0.001517 | 0.0155299 | NA | NA | |
| ENSG00000108296 | 680.4108703 | 0.012206485 | 0.0863985 | 0.8563046 | 0.9418544 | NA | NA | |
| ENSG00000125691 | 9268.268959 | -0.143958431 | 0.0583461 | 0.0067639 | 0.0476246 | RPL23 | | 9349 |
| ENSG00000002834 | 4339.878991 | -0.137807518 | 0.0629528 | 0.0141212 | 0.0810877 | LASP1 | | 3927 |
| ENSG00000265784 | 10.23309057 | -0.003406608 | 0.1348581 | 0.8755896 | NA | NA | NA | |
| ENSG00000067191 | 159.0018794 | 0.008870151 | 0.1151073 | 0.8880949 | 0.9545517 | CACNB1 | | 782 |
| ENSG00000108298 | 11297.52281 | -0.093560295 | 0.0557051 | 0.0682635 | 0.2366129 | RPL19 | | 6143 |
| ENSG00000266753 | 30.03350092 | -0.005749524 | 0.1315136 | 0.8720896 | NA | NA | NA | |
| ENSG00000108306 | 111.4662554 | -0.06306615 | 0.1346521 | 0.3096015 | 0.5790036 | FBXL20 | | 84961 |
| ENSG00000266469 | 16.74540743 | 0.01898231 | 0.1354629 | 0.50597 | NA | NA | NA | |
| ENSG00000125686 | 2667.841136 | -0.005635554 | 0.0617288 | 0.9178153 | 0.9678623 | MED1 | | 5469 |
| ENSG00000167258 | 3523.753812 | -0.087440103 | 0.0649128 | 0.1257059 | 0.3480376 | CDK12 | | 51755 |

| | | | | | | | | |
|---|---|---|---|---|---|---|---|---|
| ENSG00000131748 | 900.1612925 | 0.144804772 | 0.0947524 | 0.0510028 | 0.1946714 | STARD3 | | 10948 |
| ENSG00000173991 | 18.06463435 | 0.022253395 | 0.136189 | 0.4354859 | NA | TCAP | | 8557 |
| ENSG00000161395 | 149.8917031 | -0.02462046 | 0.1178081 | 0.6882486 | 0.858008 | PGAP3 | | 93210 |
| ENSG00000141736 | 1060.602358 | -0.096560565 | 0.0848547 | 0.1557289 | 0.3944209 | ERBB2 | | 2064 |
| ENSG00000141741 | 612.8517156 | -0.066553087 | 0.0944683 | 0.3455861 | 0.6118205 | MIEN1 | | 84299 |
| ENSG00000141738 | 501.4716258 | -0.692975204 | 0.1381212 | 3.19E-08 | 1.72E-06 | GRB7 | | 2886 |
| ENSG00000073605 | 100.4690396 | -0.000924198 | 0.1214987 | 0.9852353 | 0.9940394 | GSDMB | | 55876 |
| ENSG00000172057 | 1019.987145 | -0.021050499 | 0.0777924 | 0.7415235 | 0.8872002 | ORMDL3 | | 94103 |
| ENSG00000108344 | 3629.600185 | -0.026531089 | 0.0621982 | 0.6309897 | 0.8246354 | PSMD3 | | 5709 |
| ENSG00000108342 | 94.49536228 | 0.227008234 | 0.3460312 | 0.0238505 | 0.1169868 | CSF3 | | 1440 |
| ENSG00000008838 | 1072.809748 | -0.037009414 | 0.0814877 | 0.5721762 | 0.7867675 | MED24 | | 9862 |
| ENSG00000126351 | 343.2195125 | -0.08473818 | 0.1196694 | 0.2553464 | 0.5224559 | THRA | | 7067 |
| ENSG00000126368 | 396.1534774 | 2.003529484 | 0.1646038 | 2.49E-35 | 2.42E-32 | NR1D1 | | 9572 |
| ENSG00000188895 | 2324.713618 | -0.100798952 | 0.068918 | 0.0911591 | 0.2855535 | MSL1 | | 339287 |
| ENSG00000108349 | 2301.976743 | -0.086851232 | 0.0673383 | 0.1382612 | 0.3669688 | CASC3 | | 22794 |
| ENSG00000108352 | 78.55724048 | -0.06053852 | 0.1407245 | 0.2665561 | 0.5346062 | RAPGEFL1 | | 51195 |
| ENSG00000171475 | 1099.250217 | 0.038504832 | 0.0776613 | 0.5383272 | 0.7619385 | WIPF2 | | 147179 |
| ENSG00000094804 | 2814.317628 | 0.406100464 | 0.0726654 | 2.42E-09 | 1.67E-07 | CDC6 | | 990 |
| ENSG00000270145 | 65.1959746 | 0.051315827 | 0.1381962 | 0.312798 | 0.5819816 | NA | NA | |
| ENSG00000131759 | 315.2346353 | 0.28771459 | 0.1698747 | 0.0096373 | 0.062268 | RARA | | 5914 |
| ENSG00000265666 | 34.03025008 | 0.020577422 | 0.1327782 | 0.5947037 | NA | RARA-AS1 | | 101929693 |
| ENSG00000266208 | 78.16321603 | 0.09708168 | 0.1657581 | 0.115216 | 0.3305909 | NA | NA | |
| ENSG00000131747 | 13863.47283 | -0.284808805 | 0.0611105 | 5.50E-07 | 2.05E-05 | TOP2A | | 7153 |
| ENSG00000141753 | 2842.719898 | 1.042083366 | 0.0714949 | 2.39E-49 | 4.66E-46 | IGFBP4 | | 3487 |
| ENSG00000073584 | 783.1270245 | -0.13972442 | 0.1012393 | 0.0673176 | 0.2349246 | SMARCE1 | | 6605 |
| ENSG00000186395 | 403.9816396 | -0.050030491 | 0.103654 | 0.4761961 | 0.7156114 | KRT10 | | 3858 |
| ENSG00000167920 | 93.65947864 | -0.054990484 | 0.1340904 | 0.3446501 | 0.610988 | KRT10-AS1 | | 147184 |
| ENSG00000212724 | 78.74075477 | 1.634884369 | 0.3286796 | 3.25E-08 | 1.74E-06 | KRTAP2-3 | | 730755 |
| ENSG00000213417 | 8.521478794 | 0.040652093 | 0.1445212 | 0.0343337 | NA | KRTAP2-4 | | 85294 |
| ENSG00000108759 | 25.29048291 | 0.018960574 | 0.134344 | 0.5634931 | NA | KRT32 | | 3882 |
| ENSG00000171346 | 826.2332144 | -0.013466692 | 0.0849161 | 0.8571419 | 0.9423317 | KRT15 | | 3866 |
| ENSG00000186832 | 29.90710363 | 0.059205994 | 0.1497032 | 0.12116 | NA | KRT16 | | 3868 |
| ENSG00000173801 | 1939.468005 | -0.581428384 | 0.0800997 | 2.91E-14 | 4.85E-12 | JUP | | 3728 |
| ENSG00000173812 | 6262.501408 | 0.100982046 | 0.0612761 | 0.0651851 | 0.2303864 | EIF1 | | 10209 |
| ENSG00000173805 | 119.0020131 | 0.08456018 | 0.1458313 | 0.2021943 | 0.4563044 | HAP1 | | 9001 |
| ENSG00000141696 | 457.8296676 | 0.071124736 | 0.1031221 | 0.3302726 | 0.5985902 | P3H4 | | 10609 |
| ENSG00000141756 | 2114.002257 | -0.116400112 | 0.0721835 | 0.0598257 | 0.217499 | FKBP10 | | 60681 |
| ENSG00000141698 | 1567.450146 | -0.076281191 | 0.0735134 | 0.2206394 | 0.4802998 | NT5C3B | | 115024 |
| ENSG00000259623 | 246.2599235 | 0.035275177 | 0.1117879 | 0.6017726 | 0.8077875 | NA | NA | |
| ENSG00000178502 | 185.2812366 | 0.017678303 | 0.113407 | 0.7844355 | 0.9098848 | KLHL11 | | 55175 |
| ENSG00000131473 | 9622.502275 | -0.133126662 | 0.056011 | 0.0076467 | 0.0521434 | ACLY | | 47 |
| ENSG00000204815 | 14.07272372 | 0.015494657 | 0.1354157 | 0.5475255 | NA | ODAD4 | | 83538 |
| ENSG00000173786 | 1981.201338 | -0.050244805 | 0.0699767 | 0.4022858 | 0.6598007 | CNP | | 1267 |
| ENSG00000168259 | 2588.748373 | -0.136867072 | 0.067398 | 0.0206561 | 0.1060587 | DNAJC7 | | 7266 |
| ENSG00000168256 | 523.8825663 | 0.166680654 | 0.1195227 | 0.0472849 | 0.1859087 | NKIRAS2 | | 28511 |
| ENSG00000108773 | 1648.817913 | 0.004699114 | 0.0708007 | 0.9389715 | 0.9763786 | KAT2A | | 2648 |
| ENSG00000108774 | 1141.269004 | 0.042545866 | 0.0783228 | 0.5009854 | 0.735362 | RAB5C | | 5878 |
| ENSG00000089558 | 9.471789314 | 0.021559444 | 0.1375176 | 0.3079319 | NA | KCNH4 | | 23415 |
| ENSG00000167925 | 267.0385762 | 0.025811043 | 0.1076516 | 0.7030133 | 0.8651804 | GHDC | | 84514 |
| ENSG00000173757 | 1079.769538 | 0.00021839 | 0.0762493 | 0.9980407 | 0.9989595 | STAT5B | | 6777 |
| ENSG00000126561 | 48.16770658 | -0.018993807 | 0.1304628 | 0.6599746 | 0.8426737 | STAT5A | | 6776 |
| ENSG00000168610 | 5156.404467 | -0.124898193 | 0.0751845 | 0.0502125 | 0.1929381 | STAT3 | | 6774 |
| ENSG00000177469 | 20340.28565 | -0.027927467 | 0.055891 | 0.6008987 | 0.8072601 | CAVIN1 | | 284119 |
| ENSG00000033627 | 1753.352106 | -0.040923992 | 0.0705657 | 0.4818443 | 0.7209457 | ATP6V0A1 | | 535 |
| ENSG00000267632 | 25.43393101 | -0.011418726 | 0.1328573 | 0.7314373 | NA | NA | NA | |
| ENSG00000108784 | 284.2170775 | 0.413173818 | 0.1827311 | 0.0016833 | 0.0167204 | NAGLU | | 4669 |
| ENSG00000108786 | 79.95934515 | 0.083776443 | 0.1536451 | 0.1636168 | 0.4063384 | HSD17B1 | | 3292 |
| ENSG00000266962 | 495.699251 | 0.015034173 | 0.093548 | 0.8271564 | 0.9287505 | HSD17B1-A: | | 108783654 |
| ENSG00000068120 | 1782.813348 | 0.026632279 | 0.0705397 | 0.6603634 | 0.8426737 | COASY | | 80347 |
| ENSG00000108788 | 908.9252093 | -0.026040754 | 0.0802674 | 0.6895014 | 0.8588425 | MLX | | 6945 |
| ENSG00000131470 | 1444.574365 | 0.769593437 | 0.0911203 | 1.92E-18 | 4.87E-16 | PSMC3IP | | 29893 |
| ENSG00000141699 | 723.597565 | -0.014205877 | 0.0856921 | 0.8308413 | 0.9304016 | RETREG3 | | 162427 |
| ENSG00000131462 | 1915.709036 | -0.036778041 | 0.0692175 | 0.5265326 | 0.7537604 | TUBG1 | | 7283 |
| ENSG00000037042 | 345.0647397 | 0.0279389 | 0.1022255 | 0.6859397 | 0.8565814 | TUBG2 | | 27175 |
| ENSG00000068137 | 528.1372299 | -0.03533391 | 0.0971354 | 0.609274 | 0.8124311 | PLEKHH3 | | 79990 |
| ENSG00000184451 | 18.83458746 | 0.021372656 | 0.1356211 | 0.4751269 | NA | CCR10 | | 2826 |
| ENSG00000108797 | 655.2799686 | -0.057832985 | 0.0952831 | 0.4092922 | 0.6643076 | CNTNAP1 | | 8506 |
| ENSG00000108799 | 546.4185341 | 0.083281152 | 0.1002802 | 0.2519259 | 0.5184753 | EZH1 | | 2145 |
| ENSG00000131475 | 791.2487558 | -0.039289489 | 0.0876077 | 0.5610839 | 0.7795039 | VPS25 | | 84313 |
| ENSG00000126562 | 59.50488031 | 0.206801852 | 0.3853753 | 0.0176156 | 0.0948571 | WNK4 | | 65266 |

| | | | | | | | |
|---|---|---|---|---|---|---|---|
| ENSG00000183978 | 511.7850048 | -0.106276378 | 0.1162007 | 0.1697767 | 0.4155828 | COA3 | 28958 |
| ENSG00000176563 | 16.54418589 | -0.00017086 | 0.1338456 | 0.9941172 | NA | CNTD1 | 124817 |
| ENSG00000126581 | 1122.307079 | 0.125541703 | 0.0853973 | 0.0702765 | 0.2411841 | BECN1 | 8678 |
| ENSG00000131467 | 4458.873868 | 0.065650711 | 0.0583138 | 0.2123508 | 0.4691639 | PSME3 | 10197 |
| ENSG00000131480 | 201.534714 | 1.036117303 | 0.2273181 | 2.61E-07 | 1.07E-05 | AOC2 | 314 |
| ENSG00000131471 | 63.35001069 | 1.583703611 | 0.3658844 | 7.13E-07 | 2.57E-05 | AOC3 | 8639 |
| ENSG00000266967 | 59.79702823 | 0.047562827 | 0.136866 | 0.3403119 | 0.6075474 | AARSD1 | 80755 |
| ENSG00000267060 | 17.61940957 | 0.018553239 | 0.1354271 | 0.5111887 | NA | PTGES3L | 100885848 |
| ENSG00000198863 | 714.1245033 | 0.239245102 | 0.1177726 | 0.0075709 | 0.0517801 | RUNDC1 | 146923 |
| ENSG00000131469 | 9876.709573 | -0.114982103 | 0.056004 | 0.0237575 | 0.1168136 | RPL27 | 6155 |
| ENSG00000068079 | 367.8165866 | -0.046128835 | 0.1059064 | 0.5158228 | 0.7452598 | IFI35 | 3430 |
| ENSG00000108828 | 3233.481404 | 0.182540606 | 0.0661197 | 0.0019853 | 0.0187621 | VAT1 | 10493 |
| ENSG00000108830 | 47.36029385 | -0.048309374 | 0.1396294 | 0.2896618 | 0.559818 | RND2 | 8153 |
| ENSG00000012048 | 1931.345926 | 0.258923956 | 0.0828615 | 0.0003357 | 0.0047366 | BRCA1 | 672 |
| ENSG00000198496 | 120.4326222 | 0.188407322 | 0.2382244 | 0.0423457 | 0.1739241 | NBR2 | 10230 |
| ENSG00000267002 | 49.55816887 | 0.122964516 | 0.2088786 | 0.034733 | 0.1519535 | NA | NA |
| ENSG00000188554 | 2173.680361 | 0.339132492 | 0.0750285 | 8.08E-07 | 2.83E-05 | NBR1 | 4077 |
| ENSG00000188825 | 34.34808082 | 0.048459741 | 0.1423786 | 0.2306941 | NA | LINC00910 | 100130581 |
| ENSG00000175906 | 80.21321065 | -0.01029843 | 0.1255414 | 0.8366947 | 0.9322234 | ARL4D | 379 |
| ENSG00000067596 | 1291.484015 | -0.035043685 | 0.0758606 | 0.5788061 | 0.7915974 | DHX8 | 1659 |
| ENSG00000175832 | 632.2432194 | 0.003917927 | 0.0907991 | 0.9570925 | 0.9826522 | ETV4 | 2118 |
| ENSG00000108861 | 2574.405513 | -0.047653563 | 0.0647714 | 0.4019004 | 0.659524 | DUSP3 | 1845 |
| ENSG00000161647 | 399.7434532 | -0.14113818 | 0.1261914 | 0.0885391 | 0.2802019 | MPP3 | 4356 |
| ENSG00000108852 | 291.6567217 | 0.021378053 | 0.1085762 | 0.7510959 | 0.8925687 | MPP2 | 4355 |
| ENSG00000161653 | 16.11259578 | -0.017282631 | 0.1355056 | 0.5173595 | NA | NAGS | 162417 |
| ENSG00000091947 | 602.8076439 | 0.193769878 | 0.1279283 | 0.0295791 | 0.1371085 | TMEM101 | 84336 |
| ENSG00000161654 | 942.7886576 | -0.009013513 | 0.0791238 | 0.8880925 | 0.9545517 | LSM12 | 124801 |
| ENSG00000141349 | 601.4931086 | 0.237515743 | 0.1244621 | 0.0098473 | 0.063028 | G6PC3 | 92579 |
| ENSG00000108840 | 228.9266376 | 0.405956244 | 0.2035086 | 0.0030413 | 0.0259495 | HDAC5 | 10014 |
| ENSG00000125319 | 313.3318652 | 0.110297835 | 0.1264456 | 0.1625237 | 0.4047229 | HROB | 78995 |
| ENSG00000267080 | 155.3464897 | 0.216901691 | 0.233623 | 0.0347254 | 0.1519535 | ASB16-AS1 | 339201 |
| ENSG00000168591 | 646.6987063 | 0.125671698 | 0.1032817 | 0.0998248 | 0.3015845 | TMUB2 | 79089 |
| ENSG00000087152 | 1837.373303 | -0.069826112 | 0.0746578 | 0.2660295 | 0.5340997 | ATXN7L3 | 56970 |
| ENSG00000108312 | 2486.853324 | -0.220917447 | 0.0728809 | 0.0006327 | 0.0077242 | UBTF | 7343 |
| ENSG00000260793 | 11.00116597 | -0.014372991 | 0.1358301 | 0.5248693 | NA | ATXN7L3-AS | 101926967 |
| ENSG00000267750 | 65.96795952 | 0.641578122 | 0.4250841 | 0.0039354 | 0.0315044 | NA | NA |
| ENSG00000108309 | 8.644584602 | 0.01257468 | 0.1360762 | 0.5227717 | NA | RUNDC3A | 10900 |
| ENSG00000013306 | 2793.613496 | 0.002485095 | 0.0609991 | 0.964381 | 0.9857311 | SLC25A39 | 51629 |
| ENSG00000030582 | 3363.169367 | 0.11020255 | 0.0625595 | 0.047049 | 0.1855687 | GRN | 2896 |
| ENSG00000161682 | 237.0144131 | 0.007498155 | 0.1080897 | 0.9109461 | 0.9648392 | FAM171A2 | 284069 |
| ENSG00000005961 | 33.50137647 | -0.006482535 | 0.1309898 | 0.8625138 | NA | ITGA2B | 3674 |
| ENSG00000186566 | 1580.519497 | -0.073431072 | 0.0791125 | 0.2591113 | 0.525883 | GPATCH8 | 23131 |
| ENSG00000180340 | 103.1363914 | -0.076681939 | 0.1435284 | 0.2269808 | 0.4881913 | FZD2 | 2535 |
| ENSG00000180336 | 14.76545424 | 0.010421572 | 0.1345668 | 0.6941079 | NA | MEIOC | 284071 |
| ENSG00000180329 | 670.3887799 | -0.062595705 | 0.0924618 | 0.3712519 | 0.6334284 | CCDC43 | 124808 |
| ENSG00000267160 | 11.74243956 | 0.008549808 | 0.1348954 | 0.7221767 | NA | NA | NA |
| ENSG00000161692 | 891.8296149 | 0.051315195 | 0.0845307 | 0.4462005 | 0.6941567 | DBF4B | 80174 |
| ENSG00000073670 | 144.3783162 | 0.089794125 | 0.1440219 | 0.1987231 | 0.4519634 | ADAM11 | 4185 |
| ENSG00000267405 | 36.42687197 | 0.012133678 | 0.1309647 | 0.7605281 | NA | NA | NA |
| ENSG00000182963 | 3935.096264 | -0.140075153 | 0.0653872 | 0.0154579 | 0.0860238 | GJC1 | 10052 |
| ENSG00000108883 | 3905.055902 | -0.145918484 | 0.0622319 | 0.0088593 | 0.0585987 | EFTUD2 | 9343 |
| ENSG00000186185 | 2168.154108 | -0.05553664 | 0.0658291 | 0.3372805 | 0.6049743 | KIF18B | 146909 |
| ENSG00000131094 | 118.0625492 | 0.104582351 | 0.1608312 | 0.1337094 | 0.360457 | C1QL1 | 10882 |
| ENSG00000172992 | 238.8840846 | 0.433657181 | 0.2011278 | 0.0019753 | 0.0186828 | DCAKD | 79877 |
| ENSG00000136448 | 3362.599164 | -0.070809409 | 0.060713 | 0.1917221 | 0.4430278 | NMT1 | 4836 |
| ENSG00000161714 | 691.0397197 | 0.239815981 | 0.1129929 | 0.0062128 | 0.0446318 | PLCD3 | 113026 |
| ENSG00000181513 | 71.94035519 | 0.016802176 | 0.1262354 | 0.7424043 | 0.8877086 | ACBD4 | 79777 |
| ENSG00000186834 | 2169.003608 | 0.073904674 | 0.0695704 | 0.2197292 | 0.479502 | HEXIM1 | 10614 |
| ENSG00000224505 | 21.64686023 | -0.024127355 | 0.136008 | 0.4339987 | NA | NA | NA |
| ENSG00000168517 | 39.12805385 | 0.050430011 | 0.1425553 | 0.2315997 | NA | HEXIM2 | 124790 |
| ENSG00000184922 | 60.66237974 | 0.042615121 | 0.136557 | 0.358874 | 0.6220519 | FMNL1 | 752 |
| ENSG00000233175 | 8.844433027 | 0.024925549 | 0.1384338 | 0.231085 | NA | NA | NA |
| ENSG00000267278 | 35.59315341 | -0.00087181 | 0.1305206 | 0.9767801 | NA | NA | NA |
| ENSG00000006062 | 1201.434234 | 0.243413689 | 0.0922584 | 0.001657 | 0.0165719 | MAP3K14 | 9020 |
| ENSG00000159314 | 422.8882914 | -0.051230894 | 0.1007113 | 0.467352 | 0.7097164 | ARHGAP27 | 201176 |
| ENSG00000225190 | 803.6315217 | 0.30317917 | 0.1127722 | 0.0009537 | 0.0106499 | PLEKHM1 | 9842 |
| ENSG00000214425 | 371.0806262 | -0.073573853 | 0.1109674 | 0.3142013 | 0.5829393 | NA | NA |
| ENSG00000264070 | 10.42123132 | -0.004884792 | 0.1349292 | 0.8234983 | NA | NA | NA |
| ENSG00000204650 | 199.8659473 | -0.04072555 | 0.1169487 | 0.5346003 | 0.7591377 | LINC02210 | 147081 |
| ENSG00000186868 | 64.3317273 | -0.059094638 | 0.1434574 | 0.2338448 | 0.4964632 | MAPT | 4137 |

| | | | | | | | | |
|---|---|---|---|---|---|---|---|---|
| ENSG00000120071 | 770.7619606 | -0.014109127 | 0.085618 | 0.8314673 | 0.9304418 | KANSL1 | | 284058 |
| ENSG00000228696 | 54.68541586 | -0.012890338 | 0.1283053 | 0.7775069 | 0.9070266 | ARL17A | | 51326 |
| ENSG00000176681 | 15.98835254 | -0.027145136 | 0.1377385 | 0.3209752 | NA | LRRC37A | | 9884 |
| ENSG00000260075 | 28.17124005 | -0.00373385 | 0.1325525 | 0.9066062 | NA | LRRC37A2 | | 474170 |
| ENSG00000238083 | 48.020967 | 0.048208708 | 0.1392948 | 0.2979929 | 0.56821 | LRRC37A2 | | 474170 |
| ENSG00000185829 | 35.64865199 | -0.019369182 | 0.1324786 | 0.6140334 | NA | ARL17A | | 51326 |
| ENSG00000073969 | 1067.389307 | 0.00248994 | 0.0792812 | 0.970058 | 0.9881615 | NSF | | 4905 |
| ENSG00000213326 | 28.84806966 | -0.04303548 | 0.1415288 | 0.2232928 | NA | RPS7P11 | | 644315 |
| ENSG00000108379 | 91.4442997 | 0.07960044 | 0.1484888 | 0.1940896 | 0.446028 | WNT3 | | 7473 |
| ENSG00000108433 | 577.0002209 | 0.048619558 | 0.0935082 | 0.5185738 | 0.7475577 | GOSR2 | | 9570 |
| ENSG00000262879 | 133.3933387 | 0.009340554 | 0.1179768 | 0.8776419 | 0.9504668 | NA | NA | |
| ENSG00000004897 | 2948.327545 | -0.33588196 | 0.0694729 | 1.80E-07 | 7.83E-06 | CDC27 | | 996 |
| ENSG00000261872 | 19.09856908 | -0.005990355 | 0.1335851 | 0.8339129 | NA | NA | NA | |
| ENSG00000259207 | 667.5589107 | 0.201986215 | 0.120101 | 0.0209399 | 0.1069724 | ITGB3 | | 3690 |
| ENSG00000178852 | 13.74136193 | 0.002344171 | 0.1341404 | 0.9297701 | NA | EFCAB13 | | 124989 |
| ENSG00000228782 | 59.51679779 | 0.0434902 | 0.1357968 | 0.3692619 | 0.6317147 | NA | NA | |
| ENSG00000141279 | 2162.222556 | -0.25777169 | 0.0742026 | 9.73E-05 | 0.0017001 | NPEPPS | | 9520 |
| ENSG00000263766 | 58.73378114 | 0.069080659 | 0.1490467 | 0.1820906 | 0.4317028 | KPNB1-DT | | 112268184 |
| ENSG00000108424 | 15518.961 | -0.202999872 | 0.0546387 | 6.21E-05 | 0.0011826 | KPNB1 | | 3837 |
| ENSG00000264558 | 238.2054454 | -0.136772488 | 0.150687 | 0.1021518 | 0.3061148 | NA | NA | |
| ENSG00000198933 | 101.3789482 | 0.099982592 | 0.1624964 | 0.1298254 | 0.3543757 | TBKBP1 | | 9755 |
| ENSG00000006025 | 139.2419364 | 0.075340438 | 0.1377881 | 0.2554917 | 0.5225702 | OSBPL7 | | 114881 |
| ENSG00000159111 | 668.7659043 | 0.12736127 | 0.1042107 | 0.0967914 | 0.2958965 | MRPL10 | | 124995 |
| ENSG00000141294 | 29.9235795 | 0.041014252 | 0.1397513 | 0.276389 | NA | LRRC46 | | 90506 |
| ENSG00000141295 | 207.2084422 | -0.130706657 | 0.1579327 | 0.1080233 | 0.3180793 | SCRN2 | | 90507 |
| ENSG00000189120 | 64.41238806 | 0.106690869 | 0.1802439 | 0.0753819 | 0.2521581 | SP6 | | 80320 |
| ENSG00000264920 | 21.37763551 | 0.027986686 | 0.1372409 | 0.3575424 | NA | SP2-DT | | 102724532 |
| ENSG00000167182 | 290.877491 | -0.001844596 | 0.1074044 | 0.9743599 | 0.9904047 | SP2 | | 6668 |
| ENSG00000234494 | 44.52568023 | -0.044600017 | 0.1384133 | 0.3144414 | 0.5831994 | SP2-AS1 | | 100506325 |
| ENSG00000108439 | 365.7311431 | 0.358358905 | 0.1753861 | 0.0033567 | 0.0278477 | PNPO | | 55163 |
| ENSG00000264019 | 8.86585104 | 0.021431009 | 0.1378184 | 0.2626656 | NA | NA | NA | |
| ENSG00000108465 | 840.3141309 | 0.112488333 | 0.0948825 | 0.1225035 | 0.34291 | CDK5RAP3 | | 80279 |
| ENSG00000263412 | 10.41853943 | 0.013253241 | 0.1356488 | 0.5618004 | NA | NFE2L1-DT | | 116435298 |
| ENSG00000082641 | 3854.441899 | 0.099346785 | 0.0617905 | 0.0713757 | 0.2433566 | NFE2L1 | | 4779 |
| ENSG00000266341 | 9.053974556 | -0.018463208 | 0.1368166 | 0.3824974 | NA | NA | NA | |
| ENSG00000108468 | 3009.231095 | -0.119276282 | 0.0691042 | 0.0468661 | 0.1851973 | CBX1 | | 10951 |
| ENSG00000002919 | 465.6332798 | -0.080722305 | 0.1042143 | 0.2709539 | 0.5386338 | SNX11 | | 29916 |
| ENSG00000173917 | 451.6550264 | 0.341936267 | 0.14154 | 0.0015745 | 0.0159096 | HOXB2 | | 3212 |
| ENSG00000230148 | 79.02320116 | -0.059833974 | 0.1391908 | 0.2828873 | 0.5522993 | HOXB-AS1 | | 100874362 |
| ENSG00000120093 | 439.2654435 | 0.127429461 | 0.1181543 | 0.1117395 | 0.3243612 | HOXB3 | | 3213 |
| ENSG00000182742 | 244.801185 | 0.512631886 | 0.1916647 | 0.0004509 | 0.0059201 | HOXB4 | | 3214 |
| ENSG00000120075 | 42.68490032 | 0.009645253 | 0.1298296 | 0.8191719 | 0.925668 | HOXB5 | | 3215 |
| ENSG00000108511 | 156.4958048 | 0.075524835 | 0.1347127 | 0.2671091 | 0.5352721 | HOXB6 | | 3216 |
| ENSG00000260027 | 283.2262604 | 0.031841624 | 0.1069926 | 0.6425392 | 0.8325202 | HOXB7 | | 3217 |
| ENSG00000170689 | 83.33459178 | 0.982054729 | 0.3336418 | 0.0001359 | 0.0022353 | HOXB9 | | 3219 |
| ENSG00000272763 | 134.4676779 | 0.006771716 | 0.1177419 | 0.910453 | 0.9647135 | NA | NA | |
| ENSG00000244649 | 18.70366506 | -0.022238726 | 0.1358315 | 0.4538676 | NA | NA | NA | |
| ENSG00000159184 | 73.41499818 | 0.009449054 | 0.1256835 | 0.8519329 | 0.9396727 | HOXB13 | | 10481 |
| ENSG00000136436 | 1024.34611 | 0.079613467 | 0.0850176 | 0.2451343 | 0.5108911 | CALCOCO2 | | 10241 |
| ENSG00000248278 | 9.289285272 | 0.000823821 | 0.1350167 | 0.9710452 | NA | LOC1053718 | | 105371814 |
| ENSG00000159199 | 661.2305897 | -0.078960109 | 0.0950998 | 0.2682001 | 0.5361059 | ATP5MC1 | | 516 |
| ENSG00000159202 | 2858.636757 | -0.083739763 | 0.0649797 | 0.1427418 | 0.373933 | UBE2Z | | 65264 |
| ENSG00000159210 | 1021.055703 | -0.09622324 | 0.0852414 | 0.1588581 | 0.3993092 | SNF8 | | 11267 |
| ENSG00000230532 | 35.5992404 | -0.055627753 | 0.1462332 | 0.171917 | NA | NA | NA | |
| ENSG00000159217 | 1267.982845 | 0.163326953 | 0.0854041 | 0.0204153 | 0.1051921 | IGF2BP1 | | 10642 |
| ENSG00000198740 | 415.3366536 | -0.113550272 | 0.1193879 | 0.1486389 | 0.3831938 | ZNF652 | | 22834 |
| ENSG00000167085 | 5955.954681 | -0.068281774 | 0.0573684 | 0.1880642 | 0.4389252 | PHB1 | | 5245 |
| ENSG00000250186 | 58.7778151 | 0.005871377 | 0.127272 | 0.9018023 | 0.9616276 | NA | NA | |
| ENSG00000121067 | 865.1730784 | -0.057630351 | 0.0841424 | 0.386532 | 0.6473234 | SPOP | | 8405 |
| ENSG00000121073 | 841.3830443 | 0.056383511 | 0.085223 | 0.4021136 | 0.6596487 | SLC35B1 | | 10237 |
| ENSG00000121104 | 132.0188673 | 0.175445253 | 0.2317185 | 0.046487 | 0.1842999 | FAM117A | | 81558 |
| ENSG00000136504 | 871.3208574 | -0.08841396 | 0.0997291 | 0.225887 | 0.4868367 | KAT7 | | 11143 |
| ENSG00000262837 | 18.72361497 | -0.008584927 | 0.1337657 | 0.765643 | NA | NA | NA | |
| ENSG00000204584 | 87.36014147 | 0.033673029 | 0.1283 | 0.5337192 | 0.758532 | NA | NA | |
| ENSG00000108813 | 13.06451631 | 0.028636468 | 0.1388406 | 0.2431454 | NA | DLX4 | | 1748 |
| ENSG00000246640 | 18.33306212 | 0.030725382 | 0.1387483 | 0.2741291 | NA | PICART1 | | 284080 |
| ENSG00000005884 | 13786.94338 | -0.072875655 | 0.055657 | 0.1535282 | 0.3913077 | ITGA3 | | 3675 |
| ENSG00000005882 | 306.1569051 | -0.072314435 | 0.1141821 | 0.3197819 | 0.5886556 | PDK2 | | 5164 |
| ENSG00000167100 | 15.28469649 | 0.014158201 | 0.1349614 | 0.5968997 | NA | SAMD14 | | 201191 |
| ENSG00000108819 | 1825.300669 | -0.010981144 | 0.069141 | 0.8357108 | 0.9315873 | PPP1R9B | | 84687 |

| | | | | | | | | |
|---|---|---|---|---|---|---|---|---|
| ENSG00000236472 | 16.12074304 | 0.003123538 | 0.1339012 | 0.9103573 | NA | NA | NA | |
| ENSG00000015532 | 428.3950176 | -0.054620611 | 0.1009147 | 0.4401043 | 0.6892155 | XYLT2 | | 64132 |
| ENSG00000108826 | 867.4483023 | -0.174609374 | 0.1024917 | 0.0269801 | 0.1277481 | MRPL27 | | 51264 |
| ENSG00000154920 | 301.0921455 | 0.260412434 | 0.1847059 | 0.0175098 | 0.0945158 | EME1 | | 146956 |
| ENSG00000108829 | 5876.574464 | 0.12143621 | 0.0564034 | 0.0183919 | 0.0978625 | LRRC59 | | 55379 |
| ENSG00000253102 | 9.52102696 | 0.007255309 | 0.1355211 | 0.7121565 | NA | NA | NA | |
| ENSG00000167107 | 201.5603923 | 0.098039344 | 0.1381651 | 0.1895127 | 0.4403614 | ACSF2 | | 80221 |
| ENSG00000136444 | 621.3897329 | -0.030546582 | 0.0906494 | 0.6539974 | 0.8399571 | RSAD1 | | 55316 |
| ENSG00000006282 | 717.1969078 | 0.134008352 | 0.1051776 | 0.0845444 | 0.2716391 | SPATA20 | | 64847 |
| ENSG00000108846 | 317.1279589 | 0.471319069 | 0.1644383 | 0.0002888 | 0.0041849 | ABCC3 | | 8714 |
| ENSG00000154945 | 1818.671237 | -0.12449797 | 0.0746957 | 0.0496544 | 0.1916246 | ANKRD40 | | 91369 |
| ENSG00000262967 | 49.44135249 | -0.006986895 | 0.1295732 | 0.8646729 | 0.9456272 | NA | NA | |
| ENSG00000108848 | 4100.871307 | -0.021971644 | 0.0617802 | 0.690334 | 0.8589854 | LUC7L3 | | 51747 |
| ENSG00000141232 | 295.4777334 | -0.030242834 | 0.1071579 | 0.6563931 | 0.8417183 | TOB1 | | 10140 |
| ENSG00000008294 | 4465.645186 | 0.09929659 | 0.0676481 | 0.0922805 | 0.2872944 | SPAG9 | | 9043 |
| ENSG00000239672 | 1679.175164 | -0.132723745 | 0.0789502 | 0.0443993 | 0.1790227 | NME1 | | 4830 |
| ENSG00000243678 | 151.2009421 | -0.049922247 | 0.1236515 | 0.4390149 | 0.6886014 | NME2 | | 4831 |
| ENSG00000011258 | 465.0035373 | -0.005230267 | 0.097013 | 0.9371871 | 0.9756936 | MBTD1 | | 54799 |
| ENSG00000011260 | 1574.937965 | -0.282880992 | 0.0844853 | 0.0001324 | 0.0021909 | UTP18 | | 51096 |
| ENSG00000154975 | 156.881048 | -0.425818869 | 0.274899 | 0.0058625 | 0.0427195 | CA10 | | 56934 |
| ENSG00000141198 | 1146.863369 | 0.014466448 | 0.0759526 | 0.8196271 | 0.9257459 | TOM1L1 | | 10040 |
| ENSG00000166260 | 927.8628293 | -0.054543553 | 0.0850942 | 0.4178936 | 0.6710348 | COX11 | | 1353 |
| ENSG00000166263 | 381.8199128 | -0.21742536 | 0.1497296 | 0.0248483 | 0.1202483 | STXBP4 | | 252983 |
| ENSG00000261589 | 26.59101342 | 0.006455464 | 0.1323919 | 0.8494651 | NA | NA | NA | |
| ENSG00000108960 | 254.232089 | 0.116614397 | 0.1410895 | 0.1418541 | 0.3722268 | MMD | | 23531 |
| ENSG00000141179 | 273.6737584 | -0.00791246 | 0.1053975 | 0.9051319 | 0.9623051 | PCTP | | 58488 |
| ENSG00000183691 | 206.4751479 | 0.441730353 | 0.2480247 | 0.0039111 | 0.0313343 | NOG | | 9241 |
| ENSG00000214226 | 69.96882561 | -0.042118964 | 0.133644 | 0.4069051 | 0.6630015 | C17orf67 | | 339210 |
| ENSG00000153933 | 599.9906178 | 0.073688185 | 0.0955417 | 0.3011989 | 0.5710237 | DGKE | | 8526 |
| ENSG00000121060 | 2523.845654 | 0.128839037 | 0.0728252 | 0.0392138 | 0.1656025 | TRIM25 | | 7706 |
| ENSG00000262112 | 9.734406441 | 0.005757544 | 0.1350803 | 0.7923192 | NA | NA | NA | |
| ENSG00000121058 | 654.5059086 | -0.008189915 | 0.0878354 | 0.9060539 | 0.9628103 | COIL | | 8161 |
| ENSG00000121064 | 510.4333157 | -0.037749818 | 0.0961597 | 0.5852609 | 0.7964158 | SCPEP1 | | 59342 |
| ENSG00000263004 | 11.87839047 | 0.02979692 | 0.1393309 | 0.2140931 | NA | NA | NA | |
| ENSG00000121057 | 1909.422721 | 0.055460475 | 0.0681366 | 0.3489868 | 0.6148358 | AKAP1 | | 8165 |
| ENSG00000153944 | 1756.80061 | -0.446672946 | 0.0848289 | 1.22E-08 | 7.13E-07 | MSI2 | | 124540 |
| ENSG00000181610 | 1112.048383 | 0.041518691 | 0.0799571 | 0.5175124 | 0.7467649 | MRPS23 | | 51649 |
| ENSG00000180891 | 1337.711194 | -0.052973465 | 0.0751939 | 0.4001804 | 0.6583091 | CUEDC1 | | 404093 |
| ENSG00000136451 | 1536.161624 | -0.226823356 | 0.0855132 | 0.0017992 | 0.0175237 | VEZF1 | | 7716 |
| ENSG00000264112 | 445.0848277 | 0.110779449 | 0.1196204 | 0.1577215 | 0.3975891 | NA | NA | |
| ENSG00000136450 | 6674.235944 | -0.279188719 | 0.0589329 | 4.16E-07 | 1.60E-05 | SRSF1 | | 6426 |
| ENSG00000264364 | 2524.208114 | 0.036187237 | 0.0642175 | 0.5215969 | 0.7493455 | DYNLL2 | | 140735 |
| ENSG00000011143 | 259.5905188 | 0.016375418 | 0.1075458 | 0.8077588 | 0.9200093 | MKS1 | | 54903 |
| ENSG00000265148 | 9.625675575 | -0.008353745 | 0.1352576 | 0.6981227 | NA | TSPOAP1-A | | 100506779 |
| ENSG00000213246 | 814.0050163 | -0.085238301 | 0.0930504 | 0.2294637 | 0.4903706 | SUPT4H1 | | 6827 |
| ENSG00000108389 | 1285.450379 | -0.26589911 | 0.089759 | 0.0005341 | 0.0067838 | MTMR4 | | 9110 |
| ENSG00000108384 | 578.5826525 | -0.090020623 | 0.105328 | 0.2246996 | 0.4852777 | RAD51C | | 5889 |
| ENSG00000108395 | 1545.392096 | -0.058949238 | 0.072488 | 0.3386548 | 0.6061641 | TRIM37 | | 4591 |
| ENSG00000224738 | 26.7604453 | 0.027570437 | 0.1356603 | 0.4352263 | NA | NA | NA | |
| ENSG00000182628 | 1398.756308 | -0.032013218 | 0.0760357 | 0.612583 | 0.8147082 | SKA2 | | 348235 |
| ENSG00000068489 | 2103.644461 | -0.345347628 | 0.0905572 | 1.66E-05 | 0.0003899 | PRR11 | | 55771 |
| ENSG00000265415 | 8.380689349 | 1.77E-05 | 0.1352695 | 0.9982109 | NA | NA | NA | |
| ENSG00000167447 | 523.8213966 | 0.140855824 | 0.1153837 | 0.0809317 | 0.2642336 | SMG8 | | 55181 |
| ENSG00000153982 | 39.82348489 | 0.049512042 | 0.1415786 | 0.2518541 | NA | GDPD1 | | 284161 |
| ENSG00000175155 | 46.84366576 | -0.009677698 | 0.1290561 | 0.8221047 | 0.9271099 | YPEL2 | | 388403 |
| ENSG00000108406 | 1056.580867 | -0.147734685 | 0.0897161 | 0.0400853 | 0.1678682 | DHX40 | | 79665 |
| ENSG00000141367 | 12864.20581 | -0.295006473 | 0.0544388 | 1.03E-08 | 6.12E-07 | CLTC | | 1213 |
| ENSG00000141378 | 967.320024 | 0.156397386 | 0.0959439 | 0.0378866 | 0.1615157 | PTRH2 | | 51651 |
| ENSG00000062716 | 3167.1938 | -0.05986423 | 0.0683374 | 0.3111273 | 0.5812055 | VMP1 | | 81671 |
| ENSG00000108423 | 173.9339688 | -0.049099649 | 0.1208511 | 0.4561713 | 0.7007999 | TUBD1 | | 51174 |
| ENSG00000108443 | 1378.614587 | -0.104210192 | 0.0791591 | 0.1125412 | 0.325537 | RPS6KB1 | | 6198 |
| ENSG00000189050 | 147.4374312 | -0.096901194 | 0.150957 | 0.1655337 | 0.4097476 | RNFT1 | | 51136 |
| ENSG00000263422 | 9.305057951 | 0.003643102 | 0.1350067 | 0.8667372 | NA | NA | NA | |
| ENSG00000068097 | 539.8893566 | 0.035201463 | 0.0943029 | 0.6092876 | 0.8124311 | HEATR6 | | 63897 |
| ENSG00000261040 | 160.2577676 | -0.029952604 | 0.135831 | 0.4152342 | 0.6694756 | WFDC21P | | 645638 |
| ENSG00000267248 | 13.23704108 | 0.034176376 | 0.1406393 | 0.172792 | NA | LOC100996 | | 100996660 |
| ENSG00000170832 | 1284.912262 | 0.002225304 | 0.074909 | 0.9725207 | 0.9893759 | USP32 | | 84669 |
| ENSG00000062725 | 889.2771653 | -0.066084078 | 0.0847395 | 0.3239001 | 0.5927649 | APPBP2 | | 10513 |
| ENSG00000170836 | 310.3548853 | 0.042444127 | 0.1112498 | 0.5377264 | 0.7616323 | PPM1D | | 8493 |
| ENSG00000141376 | 286.273596 | -0.011756313 | 0.1054642 | 0.8601824 | 0.9435431 | BCAS3 | | 54828 |

| | | | | | | | | |
|---|---|---|---|---|---|---|---|---|
| ENSG00000267280 | 25.92906335 | 0.008251745 | 0.132721 | 0.8035805 | NA | TBX2-AS1 | | 103689912 |
| ENSG00000121068 | 111.3722287 | 0.176662682 | 0.233385 | 0.0458245 | 0.182938 | TBX2 | | 6909 |
| ENSG00000187013 | 11.33339628 | 0.006064432 | 0.1348513 | 0.7944849 | NA | LINC02875 | | 388407 |
| ENSG00000136492 | 1105.650104 | 0.231225791 | 0.0946984 | 0.0031014 | 0.026241 | BRIP1 | | 83990 |
| ENSG00000108506 | 799.0615621 | -0.091338698 | 0.0922846 | 0.1978374 | 0.4511888 | INTS2 | | 57508 |
| ENSG00000108510 | 2193.390946 | -0.092621205 | 0.0753528 | 0.1434587 | 0.3751142 | MED13 | | 9969 |
| ENSG00000087995 | 398.2468036 | 0.028790619 | 0.1002989 | 0.6773465 | 0.852871 | METTL2A | | 339175 |
| ENSG00000146872 | 908.0414496 | -0.054636024 | 0.0904705 | 0.4272049 | 0.6791209 | TLK2 | | 11011 |
| ENSG00000011028 | 1221.995511 | -0.084202861 | 0.0789943 | 0.1962074 | 0.4489518 | MRC2 | | 9902 |
| ENSG00000170921 | 709.6466501 | 0.211698296 | 0.1139926 | 0.0138681 | 0.0801949 | TANC2 | | 26115 |
| ENSG00000008283 | 636.1179585 | 0.040122096 | 0.0934195 | 0.5627433 | 0.7805304 | CYB561 | | 1534 |
| ENSG00000136485 | 2305.019479 | -0.165962552 | 0.0716137 | 0.0076039 | 0.0519121 | DCAF7 | | 10238 |
| ENSG00000136463 | 546.0488308 | -0.023366691 | 0.0930216 | 0.7324659 | 0.8813282 | TACO1 | | 51204 |
| ENSG00000198909 | 720.0895798 | 0.005925199 | 0.0858621 | 0.9302399 | 0.972984 | MAP3K3 | | 4215 |
| ENSG00000136490 | 484.0868828 | -0.115556469 | 0.113014 | 0.1379594 | 0.3663342 | LIMD2 | | 80774 |
| ENSG00000266173 | 73.87250559 | -0.021976959 | 0.1278503 | 0.6563958 | 0.8417183 | STRADA | | 92335 |
| ENSG00000108588 | 3330.898731 | 0.072903597 | 0.0606601 | 0.1786666 | 0.4275952 | CCDC47 | | 57003 |
| ENSG00000198231 | 4073.977248 | -0.033630707 | 0.0592751 | 0.5281708 | 0.754779 | DDX42 | | 11325 |
| ENSG00000108592 | 2626.962511 | -0.090566426 | 0.0689189 | 0.1289093 | 0.3532231 | FTSJ3 | | 117246 |
| ENSG00000087191 | 3784.239484 | -0.174856141 | 0.0632824 | 0.0021019 | 0.0195951 | PSMC5 | | 5705 |
| ENSG00000108604 | 1229.587159 | -0.020181745 | 0.0796578 | 0.7580974 | 0.8962891 | SMARCD2 | | 6603 |
| ENSG00000178607 | 399.8465185 | 0.414908404 | 0.1482049 | 0.0004159 | 0.0055645 | ERN1 | | 2081 |
| ENSG00000266402 | 26.22861825 | -0.01511051 | 0.133426 | 0.6486348 | NA | SNHG25 | | 105376843 |
| ENSG00000199753 | 63.02770194 | -0.067406198 | 0.1490374 | 0.1759666 | 0.4248586 | SNORD104 | | 692227 |
| ENSG00000136478 | 981.6107535 | 0.106763077 | 0.088305 | 0.1280744 | 0.351634 | TEX2 | | 55852 |
| ENSG00000256525 | 398.5378622 | -0.048731799 | 0.1037852 | 0.4726468 | 0.7130295 | POLG2 | | 11232 |
| ENSG00000108654 | 10696.39416 | -0.205541192 | 0.0554908 | 5.85E-05 | 0.0011293 | DDX5 | | 1655 |
| ENSG00000258890 | 529.3300584 | 0.077961049 | 0.1002753 | 0.2915374 | 0.5613232 | CEP95 | | 90799 |
| ENSG00000108854 | 6735.296366 | 0.05071548 | 0.0587524 | 0.3306208 | 0.5988497 | SMURF2 | | 64750 |
| ENSG00000215769 | 49.46758916 | 0.986473258 | 0.5171962 | 0.0016773 | 0.016696 | ARHGAP27I | | 109286553 |
| ENSG00000265982 | 10.95945137 | 0.004941762 | 0.1347375 | 0.8338529 | NA | NA | NA | |
| ENSG00000214176 | 264.2096276 | 0.39239636 | 0.184558 | 0.0024548 | 0.0221256 | NA | NA | |
| ENSG00000176809 | 15.28875288 | -0.014726371 | 0.1351578 | 0.5732201 | NA | LRRC37A3 | | 374819 |
| ENSG00000214174 | 163.9223854 | -0.007785159 | 0.1183238 | 0.89349 | 0.9570708 | NA | NA | |
| ENSG00000120063 | 1556.900444 | -0.147918925 | 0.0814439 | 0.0291711 | 0.135756 | GNA13 | | 10672 |
| ENSG00000263470 | 30.28572175 | 0.051432067 | 0.1448423 | 0.1786961 | NA | NA | NA | |
| ENSG00000168646 | 17.10754968 | 0.029507347 | 0.1381383 | 0.3055479 | NA | AXIN2 | | 8313 |
| ENSG00000154240 | 78.99647709 | -0.090030091 | 0.1595385 | 0.1346677 | 0.3614858 | CEP112 | | 201134 |
| ENSG00000154229 | 759.2151467 | -0.148515965 | 0.1047216 | 0.0574562 | 0.2121194 | PRKCA | | 5578 |
| ENSG00000198265 | 1041.353992 | -0.073066192 | 0.0862855 | 0.2836774 | 0.5531382 | HELZ | | 9931 |
| ENSG00000197170 | 2145.982858 | -0.162189905 | 0.074984 | 0.0117498 | 0.0712983 | PSMD12 | | 5718 |
| ENSG00000154217 | 324.1108098 | 0.041388427 | 0.1067457 | 0.5524078 | 0.7732385 | PITPNC1 | | 26207 |
| ENSG00000130935 | 2028.932883 | -0.046360593 | 0.0670424 | 0.4267434 | 0.6787658 | NOL11 | | 25926 |
| ENSG00000171634 | 2213.308867 | -0.198251489 | 0.0770769 | 0.0029181 | 0.0252113 | BPTF | | 2186 |
| ENSG00000186665 | 200.4363272 | 0.026982351 | 0.1160272 | 0.6738325 | 0.8501877 | C17orf58 | | 284018 |
| ENSG00000182481 | 7770.528273 | -0.26072455 | 0.0614017 | 4.40E-06 | 0.0001257 | KPNA2 | | 3838 |
| ENSG00000265055 | 9.426096073 | 0.009772633 | 0.1353999 | 0.6542743 | NA | LOC1249040 | | 124904048 |
| ENSG00000237854 | 81.56390398 | -0.065474092 | 0.14197 | 0.2491228 | 0.515466 | NA | NA | |
| ENSG00000267023 | 13.26434749 | -0.00037037 | 0.1342652 | 0.9865612 | NA | NA | NA | |
| ENSG00000196704 | 964.0688928 | -0.17526715 | 0.0969806 | 0.0220112 | 0.1107289 | AMZ2 | | 51321 |
| ENSG00000141337 | 14.16064478 | 0.030105083 | 0.1389018 | 0.2539887 | NA | ARSG | | 22901 |
| ENSG00000070540 | 66.93629176 | 0.043375514 | 0.1342537 | 0.3947153 | 0.654942 | WIPI1 | | 55062 |
| ENSG00000108946 | 3463.087925 | -0.111757285 | 0.0641058 | 0.0486608 | 0.1890888 | PRKAR1A | | 5573 |
| ENSG00000154263 | 45.68562847 | 0.003478174 | 0.1290819 | 0.9340306 | 0.9747531 | ABCA10 | | 10349 |
| ENSG00000154265 | 525.5894383 | 0.206461633 | 0.1315153 | 0.0237763 | 0.1168136 | ABCA5 | | 23461 |
| ENSG00000153822 | 83.7782426 | -0.9679071 | 0.3458461 | 0.0002013 | 0.0030898 | KCNJ16 | | 3773 |
| ENSG00000123700 | 64.86286716 | 0.007369332 | 0.1267503 | 0.8792232 | 0.9513156 | KCNJ2 | | 3759 |
| ENSG00000125398 | 1092.956897 | -0.216008366 | 0.0985744 | 0.0065203 | 0.0463567 | SOX9 | | 6662 |
| ENSG00000227036 | 178.455027 | -0.026907607 | 0.1161621 | 0.6724561 | 0.8499086 | LINC00511 | | 400619 |
| ENSG00000133195 | 264.7401594 | -0.014302257 | 0.1063754 | 0.8302978 | 0.9302425 | SLC39A11 | | 201266 |
| ENSG00000166685 | 470.1189534 | -0.100475465 | 0.1104557 | 0.1857605 | 0.4358915 | COG1 | | 9382 |
| ENSG00000133193 | 836.4087713 | -0.142492426 | 0.0983149 | 0.0590725 | 0.2158733 | VCF1 | | 84923 |
| ENSG00000141219 | 650.1433136 | -0.073459887 | 0.0938581 | 0.2987419 | 0.5688953 | C17orf80 | | 55028 |
| ENSG00000179604 | 530.7481048 | 0.031194574 | 0.0935876 | 0.6595034 | 0.8426667 | CDC42EP4 | | 23580 |
| ENSG00000172809 | 6370.52798 | -0.153608493 | 0.0680741 | 0.0102074 | 0.0644519 | RPL38 | | 6169 |
| ENSG00000170412 | 432.9769964 | -0.128207959 | 0.1221188 | 0.1126136 | 0.325537 | GPRC5C | | 55890 |
| ENSG00000109062 | 1427.905563 | -0.098345076 | 0.0792033 | 0.1339 | 0.3604716 | NHERF1 | | 9368 |
| ENSG00000109065 | 951.8757835 | 0.142480392 | 0.0940604 | 0.0535986 | 0.2021486 | NAT9 | | 26151 |
| ENSG00000109066 | 808.9937778 | -0.123459428 | 0.0953695 | 0.0925977 | 0.2876914 | TMEM104 | | 54868 |
| ENSG00000161513 | 711.6239246 | -0.174234431 | 0.113512 | 0.0355327 | 0.1544689 | FDXR | | 2232 |

| | | | | | | | | |
|---|---|---|---|---|---|---|---|---|
| ENSG00000167861 | 46.39986337 | -0.038995845 | 0.1359885 | 0.3792209 | 0.6404973 | HID1 | | 283987 |
| ENSG00000109089 | 683.1491968 | 0.371965783 | 0.1185405 | 0.0001723 | 0.0026972 | CDR2L | | 30850 |
| ENSG00000167862 | 306.4606384 | -0.004612601 | 0.1027546 | 0.9437982 | 0.9784014 | MRPL58 | | 3396 |
| ENSG00000180901 | 945.1033254 | 0.032613125 | 0.0859092 | 0.6284328 | 0.8230398 | KCTD2 | | 23510 |
| ENSG00000167863 | 2310.458189 | -0.047605575 | 0.0648951 | 0.4037492 | 0.6609017 | ATP5PD | | 10476 |
| ENSG00000170190 | 784.2106975 | -0.01089822 | 0.0840387 | 0.8685577 | 0.9471139 | SLC16A5 | | 9121 |
| ENSG00000125449 | 478.3829163 | -0.164387357 | 0.1228538 | 0.0523871 | 0.198551 | ARMC7 | | 79637 |
| ENSG00000125458 | 394.0182609 | 0.074108292 | 0.1086998 | 0.3112791 | 0.5812103 | NT5C | | 30833 |
| ENSG00000189159 | 3864.504818 | -0.002780111 | 0.0619881 | 0.9588665 | 0.9831259 | JPT1 | | 51155 |
| ENSG00000265800 | 42.31499456 | -0.057907566 | 0.1463337 | 0.1819591 | 0.4317028 | NA | NA | |
| ENSG00000263786 | 41.3364548 | -0.045646386 | 0.1402524 | 0.2721842 | 0.5397934 | NA | NA | |
| ENSG00000265242 | 53.89638134 | -0.015505084 | 0.1286862 | 0.7323725 | 0.8813066 | NA | NA | |
| ENSG00000188612 | 6204.522945 | -0.199262682 | 0.0642603 | 0.0005829 | 0.0072202 | SUMO2 | | 6613 |
| ENSG00000125450 | 1723.297179 | -0.005239779 | 0.0736288 | 0.9319255 | 0.9735366 | NUP85 | | 79902 |
| ENSG00000125447 | 1004.494771 | 0.084785532 | 0.0863457 | 0.2194879 | 0.4794014 | GGA3 | | 23163 |
| ENSG00000125445 | 1683.962805 | 0.008801139 | 0.0697659 | 0.8841435 | 0.953345 | MRPS7 | | 51081 |
| ENSG00000125457 | 157.9228091 | -0.048377394 | 0.1223455 | 0.4558525 | 0.7007253 | MIF4GD | | 57409 |
| ENSG00000263843 | 45.85651121 | -0.023744336 | 0.1323154 | 0.5654141 | 0.7820725 | MIF4GD-DT | | 100287042 |
| ENSG00000125454 | 730.1547344 | 0.545457421 | 0.1118228 | 7.90E-08 | 3.89E-06 | SLC25A19 | | 60386 |
| ENSG00000177885 | 2189.403665 | -0.018897453 | 0.0645423 | 0.7390655 | 0.8857761 | GRB2 | | 2885 |
| ENSG00000177728 | 832.9151732 | -0.105554871 | 0.0917903 | 0.1387049 | 0.3675616 | TMEM94 | | 9772 |
| ENSG00000177303 | 794.7496096 | 0.045730508 | 0.0889256 | 0.5048009 | 0.7381515 | CASKIN2 | | 57513 |
| ENSG00000182173 | 860.2724296 | 0.157555307 | 0.0979057 | 0.0384744 | 0.1634846 | TSEN54 | | 283989 |
| ENSG00000073350 | 596.7225956 | -0.064137797 | 0.0965176 | 0.3644039 | 0.6269851 | LLGL2 | | 3993 |
| ENSG00000108469 | 368.9724399 | 0.11904006 | 0.1239002 | 0.1367647 | 0.3643212 | RECQL5 | | 9400 |
| ENSG00000161526 | 1840.031225 | 0.187907752 | 0.0770718 | 0.0046365 | 0.0358262 | SAP30BP | | 29115 |
| ENSG00000132470 | 162.633977 | 0.091536866 | 0.1418561 | 0.200244 | 0.4540225 | ITGB4 | | 3691 |
| ENSG00000108479 | 329.1017147 | 0.121467272 | 0.1282947 | 0.1322307 | 0.3581236 | GALK1 | | 2584 |
| ENSG00000132475 | 8176.688025 | -0.117426286 | 0.0593273 | 0.0279534 | 0.1313979 | H3-3B | | 3021 |
| ENSG00000132478 | 845.2633519 | 0.045510935 | 0.0894683 | 0.5069925 | 0.7394584 | UNK | | 85451 |
| ENSG00000092929 | 88.31933091 | -0.001842964 | 0.1235703 | 0.9705097 | 0.9882505 | UNC13D | | 201294 |
| ENSG00000132471 | 1430.452279 | 0.08192147 | 0.0754096 | 0.1963467 | 0.4490367 | WBP2 | | 23558 |
| ENSG00000132481 | 1344.372027 | -0.077967349 | 0.0768247 | 0.2236019 | 0.4838633 | TRIM47 | | 91107 |
| ENSG00000267801 | 16.07667459 | -0.012094513 | 0.1347508 | 0.6447446 | NA | NA | NA | |
| ENSG00000141569 | 695.8222355 | 0.099435068 | 0.096682 | 0.1722812 | 0.4189915 | TRIM65 | | 201292 |
| ENSG00000204316 | 106.8477681 | 0.039607482 | 0.1289405 | 0.4795384 | 0.7186001 | MRPL38 | | 64978 |
| ENSG00000188878 | 165.2940819 | 0.512937687 | 0.2588467 | 0.0022832 | 0.0209337 | FBF1 | | 85302 |
| ENSG00000161533 | 645.4042818 | -0.007358683 | 0.090336 | 0.9103814 | 0.9647135 | ACOX1 | | 51 |
| ENSG00000261408 | 51.92431511 | -0.01024341 | 0.1283667 | 0.8189199 | 0.9256091 | TEN1-CDK3 | | 100529145 |
| ENSG00000250506 | 13.41850268 | -0.002117098 | 0.1342487 | 0.930754 | NA | CDK3 | | 1018 |
| ENSG00000167880 | 1157.298455 | 0.003213765 | 0.0788678 | 0.9610596 | 0.983972 | EVPL | | 2125 |
| ENSG00000167881 | 2323.02752 | -0.046899601 | 0.0673026 | 0.4277337 | 0.6791494 | SRP68 | | 6730 |
| ENSG00000186919 | 56.03589075 | -0.004316254 | 0.1285593 | 0.919761 | 0.9690041 | ZACN | | 353174 |
| ENSG00000182473 | 3567.063834 | -0.144615464 | 0.0667493 | 0.0139497 | 0.0804274 | EXOC7 | | 23265 |
| ENSG00000185262 | 528.8243989 | 0.203548703 | 0.1238707 | 0.0219296 | 0.1104135 | UBALD2 | | 283991 |
| ENSG00000129646 | 23.20144357 | 0.011705348 | 0.133174 | 0.7218241 | NA | QRICH2 | | 84074 |
| ENSG00000161542 | 1173.430624 | -0.073246265 | 0.0817398 | 0.2690413 | 0.536996 | PRPSAP1 | | 5635 |
| ENSG00000176170 | 2898.591284 | 0.623781385 | 0.0702601 | 4.93E-20 | 1.48E-17 | SPHK1 | | 8877 |
| ENSG00000175931 | 1180.544577 | 0.013793032 | 0.075382 | 0.8272472 | 0.9287505 | UBE2O | | 63893 |
| ENSG00000129667 | 873.0920547 | -0.072581162 | 0.0867898 | 0.2881298 | 0.5578243 | RHBDF2 | | 79651 |
| ENSG00000163597 | 1626.787867 | -0.06606155 | 0.0780286 | 0.3052721 | 0.5743305 | SNHG16 | | 100507246 |
| ENSG00000070731 | 23.23786936 | 0.00618881 | 0.1329576 | 0.8468275 | NA | ST6GALNAC | | 10610 |
| ENSG00000182534 | 752.9432725 | 0.122491001 | 0.0973214 | 0.0992395 | 0.3005468 | MXRA7 | | 439921 |
| ENSG00000070495 | 1188.373062 | 0.057996014 | 0.077515 | 0.3650578 | 0.627648 | JMJD6 | | 23210 |
| ENSG00000181038 | 201.6024277 | 0.012981323 | 0.1111046 | 0.8433175 | 0.9353791 | METTL23 | | 124512 |
| ENSG00000161547 | 6752.824549 | -0.272859222 | 0.0628709 | 2.67E-06 | 7.95E-05 | SRSF2 | | 6427 |
| ENSG00000092931 | 280.6502606 | -0.012479982 | 0.1049193 | 0.8521946 | 0.9396765 | MFSD11 | | 79157 |
| ENSG00000167889 | 87.04296344 | -0.103957711 | 0.1679892 | 0.1125074 | 0.325537 | MGAT5B | | 146664 |
| ENSG00000234912 | 171.2177778 | 0.08452172 | 0.1377988 | 0.229242 | 0.4903399 | SNHG20 | | 654434 |
| ENSG00000129657 | 3627.493708 | -0.006742462 | 0.0677857 | 0.908312 | 0.9638438 | SEC14L1 | | 6397 |
| ENSG00000184640 | 4715.131348 | -0.084121899 | 0.0585508 | 0.1109133 | 0.3229259 | SEPTIN9 | | 10801 |
| ENSG00000078687 | 318.9108914 | 0.007477029 | 0.1035729 | 0.9122936 | 0.9654377 | TNRC6C | | 57690 |
| ENSG00000204282 | 35.19399441 | 0.030953332 | 0.1356946 | 0.4188612 | NA | NA | NA | |
| ENSG00000141524 | 592.3311327 | -0.062370805 | 0.0954047 | 0.3753969 | 0.6371317 | TMC6 | | 11322 |
| ENSG00000167895 | 15.06402964 | -0.015711082 | 0.1354595 | 0.5386147 | NA | TMC8 | | 147138 |
| ENSG00000108639 | 2866.087431 | -0.093166648 | 0.0649421 | 0.1030141 | 0.3076727 | SYNGR2 | | 9144 |
| ENSG00000167900 | 5263.081647 | 0.067774676 | 0.0562313 | 0.1847006 | 0.4349649 | TK1 | | 7083 |
| ENSG00000183077 | 567.5335855 | 0.298179394 | 0.1254812 | 0.0022368 | 0.0206056 | AFMID | | 125061 |
| ENSG00000089685 | 3649.651225 | -0.213024418 | 0.0677465 | 0.0004451 | 0.0058743 | BIRC5 | | 332 |
| ENSG00000268310 | 48.13697541 | -0.044074973 | 0.1389222 | 0.3042675 | 0.5731573 | NA | NA | |

| | | | | | | | | |
|---|---|---|---|---|---|---|---|---|
| ENSG00000184557 | 654.1297536 | 0.13126391 | 0.1128275 | 0.0982674 | 0.298999 | SOCS3 | | 9021 |
| ENSG00000087157 | 552.0025702 | 0.029904135 | 0.0977857 | 0.6632546 | 0.8438599 | PGS1 | | 9489 |
| ENSG00000187775 | 21.24805034 | 3.025962362 | 0.7861771 | 7.39E-06 | NA | DNAH17 | | 8632 |
| ENSG00000267432 | 28.56085526 | 0.023293309 | 0.1345743 | 0.5058838 | NA | NA | NA | |
| ENSG00000108669 | 889.2489571 | -0.042678876 | 0.0842288 | 0.5251125 | 0.752526 | CYTH1 | | 9267 |
| ENSG00000055483 | 1875.250787 | 0.822900934 | 0.0874333 | 2.96E-22 | 1.02E-19 | USP36 | | 57602 |
| ENSG00000035862 | 358.4924949 | 0.210961524 | 0.1645726 | 0.0323485 | 0.145151 | TIMP2 | | 7077 |
| ENSG00000108679 | 65.46221508 | 0.090330071 | 0.1630396 | 0.1176197 | 0.3341236 | LGALS3BP | | 3959 |
| ENSG00000171302 | 1646.764568 | -0.095017749 | 0.075339 | 0.1340418 | 0.3606039 | CANT1 | | 124583 |
| ENSG00000173918 | 55.82203633 | 0.101661498 | 0.1803564 | 0.0601496 | 0.2183368 | C1QTNF1 | | 114897 |
| ENSG00000167280 | 532.6514834 | -0.093531982 | 0.1057457 | 0.2097906 | 0.4663614 | ENGASE | | 64772 |
| ENSG00000173894 | 464.0600577 | 0.099734851 | 0.1095004 | 0.1890451 | 0.440063 | CBX2 | | 84733 |
| ENSG00000141570 | 222.021609 | -0.00080804 | 0.1118946 | 0.9877743 | 0.9950412 | CBX8 | | 57332 |
| ENSG00000141582 | 523.3172743 | 0.147470006 | 0.1165981 | 0.0705791 | 0.2419827 | CBX4 | | 8535 |
| ENSG00000167291 | 929.4340447 | 0.129125747 | 0.0988523 | 0.0859192 | 0.2746016 | TBC1D16 | | 125058 |
| ENSG00000141519 | 70.46111794 | 0.122179161 | 0.1940219 | 0.0643496 | 0.2284011 | CCDC40 | | 55036 |
| ENSG00000171298 | 551.5794987 | 0.219199046 | 0.124427 | 0.0153381 | 0.085479 | GAA | | 2548 |
| ENSG00000141543 | 2742.880296 | 0.029454543 | 0.0659803 | 0.6113913 | 0.8137719 | EIF4A3 | | 9775 |
| ENSG00000262580 | 92.04184765 | 0.083744937 | 0.1524699 | 0.1709072 | 0.4170393 | NA | NA | |
| ENSG00000181523 | 593.771597 | 0.132608361 | 0.1112728 | 0.0943027 | 0.2912561 | SGSH | | 6448 |
| ENSG00000181045 | 341.5097166 | 0.036291472 | 0.1039872 | 0.601423 | 0.8076825 | SLC26A11 | | 284129 |
| ENSG00000173821 | 5948.735013 | -0.251944419 | 0.0666115 | 3.27E-05 | 0.000688 | RNF213 | | 57674 |
| ENSG00000263069 | 32.79990772 | 0.016126013 | 0.1321684 | 0.6718422 | NA | RNF213-AS: | | 100294362 |
| ENSG00000173818 | 189.2422891 | 0.070799572 | 0.1265368 | 0.3079961 | 0.5771098 | ENDOV | | 284131 |
| ENSG00000171246 | 123.3761308 | 0.050230758 | 0.1275119 | 0.4189459 | 0.6717512 | NPTX1 | | 4884 |
| ENSG00000141564 | 979.1835722 | 0.034514899 | 0.0799669 | 0.5962381 | 0.8041401 | RPTOR | | 57521 |
| ENSG00000176108 | 399.8073235 | -0.049047548 | 0.1015253 | 0.4862593 | 0.7239115 | CHMP6 | | 79643 |
| ENSG00000175911 | 24.3101236 | -0.003939566 | 0.1328956 | 0.8987313 | NA | NA | NA | |
| ENSG00000226137 | 320.3780351 | -0.058117263 | 0.1106724 | 0.4112378 | 0.6661689 | BAIAP2-DT | | 440465 |
| ENSG00000175866 | 622.9087825 | -0.002437223 | 0.0902598 | 0.9684109 | 0.9877758 | BAIAP2 | | 10458 |
| ENSG00000181409 | 17.57815387 | 0.02546002 | 0.1368849 | 0.3811369 | NA | AATK | | 9625 |
| ENSG00000141577 | 1319.88357 | 0.045167647 | 0.0742625 | 0.4705323 | 0.7115591 | CEP131 | | 22994 |
| ENSG00000167302 | 554.3635041 | 0.001900976 | 0.0918078 | 0.9785743 | 0.9920784 | TEPSIN | | 146705 |
| ENSG00000224877 | 914.5971002 | -0.000230366 | 0.0817796 | 0.9963301 | 0.9987241 | NDUFAF8 | | 284184 |
| ENSG00000157637 | 1166.300334 | 0.108162695 | 0.0830426 | 0.1111604 | 0.3233517 | SLC38A10 | | 124565 |
| ENSG00000185168 | 37.86860511 | 0.010174256 | 0.1307903 | 0.7982542 | NA | NA | NA | |
| ENSG00000262877 | 9.636514733 | -0.004166143 | 0.1349397 | 0.8469537 | NA | NA | NA | |
| ENSG00000263053 | 37.86823258 | -0.015581133 | 0.1311655 | 0.6971744 | NA | NA | NA | |
| ENSG00000171282 | 972.9535511 | -0.076825195 | 0.0860313 | 0.2560545 | 0.5231717 | NA | NA | |
| ENSG00000184009 | 53206.02866 | -0.146788109 | 0.1060858 | 0.0641703 | 0.2279032 | ACTG1 | | 71 |
| ENSG00000185504 | 792.8835467 | 0.066693432 | 0.0873345 | 0.3312662 | 0.5990297 | FAAP100 | | 80233 |
| ENSG00000182446 | 3683.601892 | 0.011543123 | 0.0593006 | 0.8301422 | 0.9302425 | NPLOC4 | | 55666 |
| ENSG00000182612 | 16.29510844 | 0.020361986 | 0.1361694 | 0.4465471 | NA | TSPAN10 | | 83882 |
| ENSG00000204237 | 313.7662544 | 0.483663695 | 0.1673836 | 0.000259 | 0.0038098 | OXLD1 | | 339229 |
| ENSG00000185298 | 1712.781235 | 0.199150085 | 0.0794034 | 0.003467 | 0.0285197 | CCDC137 | | 339230 |
| ENSG00000214087 | 739.7701833 | 0.119200998 | 0.098198 | 0.1091955 | 0.3199178 | ARL16 | | 339231 |
| ENSG00000185359 | 2750.138482 | 0.118904356 | 0.0654335 | 0.039164 | 0.1655117 | HGS | | 9146 |
| ENSG00000262049 | 150.5075257 | 0.000316306 | 0.1158125 | 0.9998063 | 0.9998063 | NA | NA | |
| ENSG00000262814 | 625.2328824 | -0.131226933 | 0.1070321 | 0.091383 | 0.2860247 | MRPL12 | | 6182 |
| ENSG00000183048 | 426.2225132 | 0.022724823 | 0.0974568 | 0.7413789 | 0.8871181 | SLC25A10 | | 1468 |
| ENSG00000225663 | 78.01393526 | -0.014795208 | 0.1256143 | 0.7716222 | 0.9029572 | MCRIP1 | | 348262 |
| ENSG00000185624 | 11771.87103 | 0.062731189 | 0.0514596 | 0.187682 | 0.4386788 | P4HB | | 5034 |
| ENSG00000262831 | 127.8794843 | 0.029650537 | 0.1239843 | 0.6085559 | 0.8122953 | NA | NA | |
| ENSG00000262413 | 31.78267592 | -0.016068268 | 0.1329508 | 0.6484354 | NA | NA | NA | |
| ENSG00000141522 | 5841.100249 | -0.101707915 | 0.0589787 | 0.0553536 | 0.2065101 | ARHGDIA | | 396 |
| ENSG00000263731 | 48.83322568 | -0.011867012 | 0.1290675 | 0.7856854 | 0.9107464 | NA | NA | |
| ENSG00000183684 | 3164.317978 | -0.031188792 | 0.0601511 | 0.5628047 | 0.7805304 | ALYREF | | 10189 |
| ENSG00000141552 | 1041.547308 | 0.025230013 | 0.0786427 | 0.6959633 | 0.8612721 | ANAPC11 | | 51529 |
| ENSG00000185813 | 687.7142973 | -0.17881183 | 0.1128393 | 0.0312686 | 0.1419966 | PCYT2 | | 5833 |
| ENSG00000187531 | 1156.942913 | 0.174031078 | 0.0903907 | 0.0177799 | 0.0953131 | SIRT7 | | 51547 |
| ENSG00000197063 | 1518.048791 | 0.55862215 | 0.0906588 | 5.44E-11 | 4.82E-09 | MAFG | | 4097 |
| ENSG00000265688 | 50.4458169 | 0.016512453 | 0.1315494 | 0.6789552 | 0.8535466 | MILIP | | 92659 |
| ENSG00000183010 | 2029.831187 | 0.17160143 | 0.0791258 | 0.010557 | 0.0658743 | PYCR1 | | 5831 |
| ENSG00000263585 | 9.96343899 | -0.008758385 | 0.1353289 | 0.6809055 | NA | NA | NA | |
| ENSG00000185269 | 507.8198386 | -0.032569151 | 0.1282759 | 0.5394204 | 0.7631064 | NOTUM | | 147111 |
| ENSG00000169696 | 657.0540181 | 0.011074255 | 0.0887152 | 0.8707194 | 0.9478556 | ASPSCR1 | | 79058 |
| ENSG00000169689 | 3316.454944 | 0.111729847 | 0.0681826 | 0.0604261 | 0.2189445 | CENPX | | 201254 |
| ENSG00000169683 | 835.2179016 | 0.026171124 | 0.0838658 | 0.6941738 | 0.8602822 | LRRC45 | | 201255 |
| ENSG00000169750 | 437.4237709 | -0.299826203 | 0.1429519 | 0.004154 | 0.0328523 | RAC3 | | 5881 |
| ENSG00000169738 | 1149.009857 | -0.02475729 | 0.0761182 | 0.6948462 | 0.8606191 | DCXR | | 51181 |

| | | | | | | | | |
|---|---|---|---|---|---|---|---|---|
| ENSG00000169733 | 491.5969168 | 0.106994086 | 0.1096843 | 0.1633016 | 0.4059431 | RFNG | | 5986 |
| ENSG00000169727 | 2219.759811 | -0.068648623 | 0.0700422 | 0.2542129 | 0.5211409 | GPS1 | | 2873 |
| ENSG00000169718 | 2039.842429 | 0.063097805 | 0.068022 | 0.2857883 | 0.5556414 | DUS1L | | 64118 |
| ENSG00000169710 | 8929.429524 | -0.278459735 | 0.0621577 | 1.35E-06 | 4.36E-05 | FASN | | 2194 |
| ENSG00000176155 | 669.8160584 | -0.042072524 | 0.0884584 | 0.5323488 | 0.7577331 | CCDC57 | | 284001 |
| ENSG00000141526 | 4025.24959 | 0.060513399 | 0.0581719 | 0.2500444 | 0.5162193 | SLC16A3 | | 9123 |
| ENSG00000141551 | 3357.304839 | 0.040633335 | 0.0605348 | 0.4574646 | 0.7017657 | CSNK1D | | 1453 |
| ENSG00000184551 | 22.22515405 | 0.058624959 | 0.1513661 | 0.0753016 | NA | NA | NA | |
| ENSG00000260563 | 24.94548645 | 0.02111883 | 0.1345611 | 0.529645 | NA | NA | NA | |
| ENSG00000141574 | 117.3277463 | -0.001731771 | 0.1212251 | 0.9740664 | 0.9902667 | SECTM1 | | 6398 |
| ENSG00000181396 | 781.4410585 | -0.120510154 | 0.0988807 | 0.1059913 | 0.3137568 | OGFOD3 | | 79701 |
| ENSG00000169660 | 220.7958745 | 0.074858439 | 0.123981 | 0.2972754 | 0.5675833 | HEXD | | 284004 |
| ENSG00000178927 | 577.6736059 | 0.063584431 | 0.094775 | 0.3682185 | 0.6310387 | CYBC1 | | 79415 |
| ENSG00000265458 | 17.03106736 | -0.000533796 | 0.1335487 | 0.9827698 | NA | NARF-AS2 | | 105371941 |
| ENSG00000141562 | 770.7359078 | -0.086952188 | 0.0909211 | 0.2159716 | 0.474293 | NARF | | 26502 |
| ENSG00000141568 | 2018.024264 | 0.052527414 | 0.0679631 | 0.3727409 | 0.6345995 | FOXK2 | | 3607 |
| ENSG00000261845 | 12.80432216 | -0.000215621 | 0.1343914 | 0.9906042 | NA | NA | NA | |
| ENSG00000141580 | 3280.170128 | -0.010334523 | 0.0597037 | 0.8467499 | 0.9372292 | WDR45B | | 56270 |
| ENSG00000141542 | 219.2690345 | -0.065572471 | 0.1209836 | 0.3485512 | 0.6144622 | RAB40B | | 10966 |
| ENSG00000141560 | 1085.594823 | 0.008392555 | 0.0760727 | 0.8951436 | 0.9580514 | FN3KRP | | 79672 |
| ENSG00000167363 | 152.4804604 | -0.073780152 | 0.1367038 | 0.264478 | 0.5323182 | FN3K | | 64122 |
| ENSG00000141556 | 1675.134732 | -0.184251631 | 0.0810833 | 0.007256 | 0.0501227 | TBCD | | 6904 |
| ENSG00000175711 | 270.8400664 | -0.056851912 | 0.1143611 | 0.4126901 | 0.6666675 | B3GNTL1 | | 146712 |
| ENSG00000176845 | 60.85252644 | 0.099546429 | 0.1746044 | 0.0803493 | 0.2629757 | METRNL | | 284207 |
| ENSG00000263006 | 19.30468705 | 0.016342935 | 0.1346573 | 0.5841451 | NA | NA | NA | |
| ENSG00000101557 | 2669.118372 | -0.102819641 | 0.0659603 | 0.0755168 | 0.2524647 | USP14 | | 9097 |
| ENSG00000079134 | 829.1144802 | -0.121707798 | 0.0945471 | 0.0955796 | 0.2938201 | THOC1 | | 9984 |
| ENSG00000176912 | 44.49655906 | 0.032048142 | 0.1338741 | 0.4622573 | 0.7066651 | NA | NA | |
| ENSG00000176890 | 4715.338687 | -0.162938943 | 0.0630693 | 0.0039582 | 0.0315815 | TYMS | | 7298 |
| ENSG00000132199 | 1021.694696 | 0.015695837 | 0.0793188 | 0.808932 | 0.9206991 | ENOSF1 | | 55556 |
| ENSG00000176105 | 1885.21012 | -0.267838584 | 0.0788675 | 0.0001243 | 0.0020794 | YES1 | | 7525 |
| ENSG00000266171 | 8.769183132 | 0.00955162 | 0.1355343 | 0.6478209 | NA | NA | NA | |
| ENSG00000101574 | 314.6661419 | -0.121490547 | 0.134795 | 0.1320113 | 0.3579442 | METTL4 | | 64863 |
| ENSG00000266783 | 14.62543832 | -0.0012592 | 0.1339525 | 0.9599748 | NA | NA | NA | |
| ENSG00000080986 | 1765.889721 | -0.135704604 | 0.0749631 | 0.0334979 | 0.1480474 | NDC80 | | 10403 |
| ENSG00000101596 | 2711.029759 | -0.070722145 | 0.0636245 | 0.2085963 | 0.4650913 | SMCHD1 | | 23347 |
| ENSG00000101577 | 991.2896637 | 0.114497764 | 0.0992402 | 0.1239928 | 0.3453836 | LPIN2 | | 9663 |
| ENSG00000272688 | 38.01676829 | 0.044133086 | 0.139507 | 0.2912315 | NA | NA | NA | |
| ENSG00000101608 | 5451.649092 | 0.036884075 | 0.0544452 | 0.4594085 | 0.7037836 | MYL12A | | 10627 |
| ENSG00000264235 | 132.547979 | -0.005375681 | 0.1188222 | 0.9269002 | 0.9718632 | MYL12-AS1 | | 104968399 |
| ENSG00000118680 | 5601.817502 | -0.212958421 | 0.0591298 | 8.80E-05 | 0.0015703 | MYL12B | | 103910 |
| ENSG00000177426 | 1211.729135 | 0.010565613 | 0.0837554 | 0.874229 | 0.9491371 | TGIF1 | | 7050 |
| ENSG00000177337 | 114.1035805 | -0.066145629 | 0.1363769 | 0.2908968 | 0.5608471 | NA | NA | |
| ENSG00000262001 | 712.8679661 | 0.184381182 | 0.1130612 | 0.027512 | 0.1296359 | NA | NA | |
| ENSG00000266401 | 38.48171976 | 0.044195778 | 0.1400852 | 0.2757857 | NA | LOC1053719 | | 105371967 |
| ENSG00000263753 | 73.65552737 | -0.029621441 | 0.1285411 | 0.5660541 | 0.7823092 | LINC00667 | | 339290 |
| ENSG00000198081 | 334.2377851 | -0.003251735 | 0.1021774 | 0.9593158 | 0.9832262 | ZBTB14 | | 7541 |
| ENSG00000235552 | 242.4650313 | -0.15559963 | 0.1674672 | 0.0752285 | 0.2518614 | RPL6P27 | | 645387 |
| ENSG00000173482 | 385.4095626 | 0.286975046 | 0.1722665 | 0.0101778 | 0.0643674 | PTPRM | | 5797 |
| ENSG00000206418 | 577.8844798 | -0.101884424 | 0.1072902 | 0.1773085 | 0.4267086 | RAB12 | | 201475 |
| ENSG00000168502 | 1420.227232 | -0.075318383 | 0.0797296 | 0.2492257 | 0.5154909 | MTCL1 | | 23255 |
| ENSG00000178127 | 38.44399861 | -0.015483848 | 0.1310845 | 0.7000781 | NA | NDUFV2 | | 4729 |
| ENSG00000101745 | 848.5996425 | 0.023245717 | 0.089777 | 0.7350968 | 0.8833109 | ANKRD12 | | 23253 |
| ENSG00000128791 | 1821.846999 | -0.217052394 | 0.0777754 | 0.0013091 | 0.0138513 | TWSG1 | | 57045 |
| ENSG00000017797 | 1989.982992 | -0.070800795 | 0.0685012 | 0.2323619 | 0.4950245 | RALBP1 | | 10928 |
| ENSG00000273352 | 15.54990191 | -0.018973611 | 0.1356878 | 0.4867342 | NA | NA | NA | |
| ENSG00000154845 | 1651.692149 | -0.116120764 | 0.0750064 | 0.0674813 | 0.2351177 | PPP4R1 | | 9989 |
| ENSG00000168461 | 520.8514255 | 0.072179281 | 0.1062058 | 0.3229876 | 0.5916289 | RAB31 | | 11031 |
| ENSG00000101558 | 2346.409357 | -0.025207471 | 0.0669199 | 0.6653788 | 0.8451364 | VAPA | | 9218 |
| ENSG00000134265 | 797.9997748 | -0.188623709 | 0.1083142 | 0.021682 | 0.1094026 | NAPG | | 8774 |
| ENSG00000255112 | 781.9576478 | 0.177037193 | 0.1084259 | 0.0296413 | 0.1371792 | CHMP1B | | 57132 |
| ENSG00000267165 | 19.25725679 | -0.009502108 | 0.1340157 | 0.7362299 | NA | NA | NA | |
| ENSG00000154889 | 229.5809339 | 0.082519438 | 0.1259206 | 0.259945 | 0.5270137 | MPPE1 | | 65258 |
| ENSG00000141401 | 286.3670449 | 0.19350038 | 0.1636583 | 0.0427553 | 0.1748687 | IMPA2 | | 3613 |
| ENSG00000176014 | 5034.688723 | 0.001485954 | 0.0565573 | 0.9808248 | 0.9930139 | TUBB6 | | 84617 |
| ENSG00000141385 | 2248.786529 | -0.062005905 | 0.0686373 | 0.2967256 | 0.566626 | AFG3L2 | | 10939 |
| ENSG00000141391 | 93.14724481 | 0.165596694 | 0.2387904 | 0.0437634 | 0.1774383 | PRELID3A | | 10650 |
| ENSG00000267199 | 49.42834204 | 0.018841504 | 0.1298235 | 0.675403 | 0.851066 | NA | NA | |
| ENSG00000134278 | 1233.527514 | 0.070997469 | 0.0775115 | 0.2692917 | 0.5372685 | SPIRE1 | | 56907 |
| ENSG00000215527 | 17.00202393 | 0.017947253 | 0.135305 | 0.5254547 | NA | NA | NA | |

| | | | | | | | | |
|---|---|---|---|---|---|---|---|---|
| ENSG00000128789 | 893.0253493 | -0.221715142 | 0.1006359 | 0.0060154 | 0.043508 | PSMG2 | | 56984 |
| ENSG00000101624 | 289.9381208 | 0.135517741 | 0.1394478 | 0.1044738 | 0.3104023 | CEP76 | | 79959 |
| ENSG00000267249 | 19.17504277 | 0.002769467 | 0.1333445 | 0.9269376 | NA | NA | NA | |
| ENSG00000175354 | 697.5936752 | -0.069260623 | 0.0916983 | 0.3215213 | 0.5904246 | PTPN2 | | 5771 |
| ENSG00000085415 | 2121.037337 | -0.162898387 | 0.0729001 | 0.0098977 | 0.063212 | SEH1L | | 81929 |
| ENSG00000101639 | 1297.159566 | 0.020619701 | 0.073662 | 0.7406867 | 0.88659 | CEP192 | | 55125 |
| ENSG00000177150 | 565.892959 | 0.109662673 | 0.1055787 | 0.1488564 | 0.3836131 | FAM210A | | 125228 |
| ENSG00000101654 | 1243.109243 | -0.004199051 | 0.0735379 | 0.9452533 | 0.9784014 | RNMT | | 8731 |
| ENSG00000175322 | 96.04459266 | 0.020675245 | 0.1237567 | 0.7098712 | 0.8682728 | ZNF519 | | 162655 |
| ENSG00000267756 | 8.420121664 | 0.018804064 | 0.1371683 | 0.334958 | NA | NA | NA | |
| ENSG00000067900 | 2315.460256 | -0.125878865 | 0.0743033 | 0.0466335 | 0.1846527 | ROCK1 | | 6093 |
| ENSG00000141449 | 249.8665862 | 0.201222476 | 0.1798919 | 0.0403067 | 0.1683313 | GREB1L | | 80000 |
| ENSG00000141446 | 646.3099694 | 0.036830112 | 0.0925137 | 0.5934506 | 0.8024189 | ESCO1 | | 114799 |
| ENSG00000167088 | 2225.423378 | -0.174456595 | 0.0756 | 0.0073208 | 0.0504261 | SNRPD1 | | 6632 |
| ENSG00000263350 | 16.55812656 | 0.007742293 | 0.1339909 | 0.7825598 | NA | NA | NA | |
| ENSG00000158201 | 697.1032811 | 0.032783198 | 0.0877242 | 0.6287779 | 0.823051 | ABHD3 | | 171586 |
| ENSG00000101752 | 1301.015516 | -0.013671562 | 0.0729115 | 0.8239183 | 0.9274924 | MIB1 | | 57534 |
| ENSG00000141448 | 70.90676095 | 0.483050379 | 0.4612177 | 0.0080036 | 0.0541342 | GATA6 | | 2627 |
| ENSG00000101773 | 1908.383765 | 0.209765453 | 0.0788489 | 0.0020707 | 0.0193662 | RBBP8 | | 5932 |
| ENSG00000134508 | 33.99677773 | 0.041640011 | 0.1392838 | 0.2921716 | NA | CABLES1 | | 91768 |
| ENSG00000134490 | 340.2626135 | 0.021148887 | 0.1015054 | 0.7586581 | 0.8963893 | TMEM241 | | 85019 |
| ENSG00000101782 | 1358.744362 | 0.465021499 | 0.0933723 | 5.55E-08 | 2.82E-06 | RIOK3 | | 8780 |
| ENSG00000141452 | 673.5248812 | 0.079889164 | 0.0941636 | 0.2623093 | 0.5299265 | RMC1 | | 29919 |
| ENSG00000141458 | 1714.429638 | 0.125130922 | 0.0825755 | 0.0653042 | 0.2305982 | NPC1 | | 4864 |
| ENSG00000154065 | 65.14818905 | 0.039444616 | 0.1326582 | 0.4384472 | 0.6881889 | ANKRD29 | | 147463 |
| ENSG00000053747 | 589.2899503 | 0.084380276 | 0.098525 | 0.2450295 | 0.5108316 | LAMA3 | | 3909 |
| ENSG00000168234 | 341.5190224 | 0.055109882 | 0.1085773 | 0.4356545 | 0.6861988 | TTC39C | | 125488 |
| ENSG00000154040 | 148.5140673 | 0.049436742 | 0.1242334 | 0.4417133 | 0.6908823 | CABYR | | 26256 |
| ENSG00000141447 | 548.0858554 | 0.017144461 | 0.0940883 | 0.8111915 | 0.9221365 | OSBPL1A | | 114876 |
| ENSG00000154059 | 328.4863795 | 0.067489111 | 0.1110121 | 0.3518803 | 0.6177207 | IMPACT | | 55364 |
| ENSG00000141380 | 2412.554354 | -0.211352336 | 0.0717269 | 0.0008628 | 0.0098706 | SS18 | | 6760 |
| ENSG00000188985 | 19.66153187 | 0.021597673 | 0.1355079 | 0.4802826 | NA | NA | NA | |
| ENSG00000141384 | 416.5973234 | 0.471801263 | 0.1454403 | 8.67E-05 | 0.0015538 | TAF4B | | 6875 |
| ENSG00000134504 | 475.9995259 | -0.314150674 | 0.1368111 | 0.0024287 | 0.0219406 | KCTD1 | | 284252 |
| ENSG00000170558 | 330.5279986 | -0.050683507 | 0.106871 | 0.4705225 | 0.7115591 | CDH2 | | 1000 |
| ENSG00000134762 | 1104.966239 | -0.317619867 | 0.0984141 | 0.0001662 | 0.0026334 | DSC3 | | 1825 |
| ENSG00000134755 | 415.0624892 | -0.012925142 | 0.0965723 | 0.8493419 | 0.9378167 | DSC2 | | 1824 |
| ENSG00000046604 | 2093.365864 | 0.047691475 | 0.0691115 | 0.4232644 | 0.6752831 | DSG2 | | 1829 |
| ENSG00000118276 | 246.7327112 | 0.392353376 | 0.1917004 | 0.0028883 | 0.0250285 | B4GALT6 | | 9331 |
| ENSG00000259985 | 19.19143228 | 0.024340226 | 0.1365268 | 0.4043029 | NA | NA | NA | |
| ENSG00000153339 | 1411.785094 | -0.023229152 | 0.0737604 | 0.7080876 | 0.8678177 | TRAPPC8 | | 22878 |
| ENSG00000101695 | 39.67806793 | 0.030505047 | 0.1344639 | 0.4574013 | NA | RNF125 | | 54941 |
| ENSG00000134758 | 1066.625438 | -0.044016182 | 0.0805689 | 0.500152 | 0.7347747 | RNF138 | | 51444 |
| ENSG00000141441 | 177.9510933 | 0.363121181 | 0.2390625 | 0.0079785 | 0.0540587 | GAREM1 | | 64762 |
| ENSG00000215492 | 505.5208684 | -0.398137194 | 0.1366413 | 0.0003109 | 0.00445 | NA | NA | |
| ENSG00000262477 | 14.91170646 | -0.018587968 | 0.1360526 | 0.462611 | NA | NA | NA | |
| ENSG00000197705 | 53.06007589 | -1.72398638 | 0.4156887 | 1.48E-06 | 4.70E-05 | KLHL14 | | 57565 |
| ENSG00000166974 | 565.8984146 | -0.061722978 | 0.0955764 | 0.380643 | 0.6416005 | MAPRE2 | | 10982 |
| ENSG00000186812 | 188.7604632 | 0.38653487 | 0.226243 | 0.0054641 | 0.0405875 | ZNF397 | | 84307 |
| ENSG00000186814 | 201.4056561 | -0.01407739 | 0.1118332 | 0.8269813 | 0.9287505 | ZSCAN30 | | 100101467 |
| ENSG00000268573 | 100.2811272 | 0.071350888 | 0.1422879 | 0.2402842 | 0.5055349 | NA | NA | |
| ENSG00000257267 | 668.8988984 | -0.166869941 | 0.1075953 | 0.037464 | 0.1603047 | ZNF271P | | 10778 |
| ENSG00000172466 | 2613.414843 | -0.004063392 | 0.0621318 | 0.9407943 | 0.976977 | ZNF24 | | 7572 |
| ENSG00000186496 | 23.99230148 | -0.0104697 | 0.132994 | 0.7470362 | NA | ZNF396 | | 252884 |
| ENSG00000153391 | 154.6481991 | 0.015499789 | 0.1159132 | 0.8048506 | 0.9186624 | INO80C | | 125476 |
| ENSG00000141429 | 1009.12545 | -0.095724864 | 0.0847853 | 0.159762 | 0.4007257 | GALNT1 | | 2589 |
| ENSG00000141428 | 307.1438412 | 0.035676666 | 0.1068022 | 0.6051882 | 0.8103616 | C18orf21 | | 83608 |
| ENSG00000141425 | 1895.284966 | -0.056193706 | 0.0684421 | 0.3418358 | 0.6087837 | RPRD1A | | 55197 |
| ENSG00000141424 | 2135.183335 | -0.081090208 | 0.0684596 | 0.1713459 | 0.4177334 | SLC39A6 | | 25800 |
| ENSG00000134759 | 1259.966263 | -0.089325368 | 0.078674 | 0.1698656 | 0.4156722 | ELP2 | | 55250 |
| ENSG00000260552 | 22.83070379 | 0.01238013 | 0.133645 | 0.6928087 | NA | COSMOC | | 101927809 |
| ENSG00000075643 | 426.4831737 | -0.01986952 | 0.0966232 | 0.7719487 | 0.9031583 | MOCOS | | 55034 |
| ENSG00000134775 | 560.9504589 | 0.224926211 | 0.1227768 | 0.0127853 | 0.0756175 | FHOD3 | | 80206 |
| ENSG00000134779 | 2404.27791 | -0.062889394 | 0.0669356 | 0.2816879 | 0.5508784 | TPGS2 | | 25941 |
| ENSG00000150477 | 123.1980212 | -0.031746794 | 0.1224563 | 0.5946681 | 0.8031487 | KIAA1328 | | 57536 |
| ENSG00000267374 | 82.67059266 | 0.104211872 | 0.1697493 | 0.1075167 | 0.3170671 | MIR924HG | | 647946 |
| ENSG00000078142 | 554.2093813 | -0.031997122 | 0.0916288 | 0.6396708 | 0.8306614 | PIK3C3 | | 5289 |
| ENSG00000152223 | 824.7271364 | 0.578083426 | 0.1143963 | 3.07E-08 | 1.68E-06 | EPG5 | | 57724 |
| ENSG00000152234 | 9002.921944 | -0.262724131 | 0.0575402 | 1.01E-06 | 3.41E-05 | ATP5F1A | | 498 |
| ENSG00000152240 | 899.1160728 | -0.196936921 | 0.1020804 | 0.0138463 | 0.0801227 | HAUS1 | | 115106 |

| | | | | | | | |
|---|---|---|---|---|---|---|---|
| ENSG00000152242 | 1057.210715 | 0.220521445 | 0.094313 | 0.0044203 | 0.0344451 | ARK2N | 147339 |
| ENSG00000078043 | 521.6925663 | -0.048786184 | 0.0973381 | 0.4855094 | 0.7234177 | PIAS2 | 9063 |
| ENSG00000167220 | 433.2000702 | -0.154433381 | 0.1258706 | 0.0673637 | 0.2349429 | HDHD2 | 84064 |
| ENSG00000134049 | 600.3090052 | -0.156830991 | 0.1133577 | 0.0539953 | 0.2031955 | IER3IP1 | 51124 |
| ENSG00000175387 | 1654.261 | -0.212783772 | 0.083895 | 0.0028025 | 0.0244482 | SMAD2 | 4087 |
| ENSG00000134030 | 1286.355562 | -0.1342179 | 0.0835976 | 0.0501561 | 0.1928482 | CTIF | 9811 |
| ENSG00000101665 | 334.0679436 | -0.025061849 | 0.1029452 | 0.7138321 | 0.8705651 | SMAD7 | 4092 |
| ENSG00000141627 | 515.3137091 | 0.064906243 | 0.0984317 | 0.3803417 | 0.6413815 | DYM | 54808 |
| ENSG00000177576 | 22.72581087 | 0.037770402 | 0.1400728 | 0.248341 | NA | C18orf32 | 497661 |
| ENSG00000263916 | 8.928862804 | -0.036877314 | 0.1425653 | 0.0754296 | NA | NA | NA |
| ENSG00000265496 | 39.68737728 | 0.015190363 | 0.1322372 | 0.6856276 | NA | NA | NA |
| ENSG00000265681 | 727.9747779 | 0.019144682 | 0.0850721 | 0.774871 | 0.9050363 | RPL17 | 6139 |
| ENSG00000101670 | 64.93337472 | 0.135187639 | 0.2169633 | 0.0427145 | 0.1748031 | LIPG | 9388 |
| ENSG00000167315 | 1278.611085 | -0.004061481 | 0.0732564 | 0.9468983 | 0.9786934 | ACAA2 | 10449 |
| ENSG00000167306 | 350.1430252 | 0.128711634 | 0.129692 | 0.1157542 | 0.3317283 | MYO5B | 4645 |
| ENSG00000172361 | 24.75155229 | 0.037396429 | 0.1394235 | 0.2764142 | NA | CFAP53 | 220136 |
| ENSG00000141644 | 1613.43605 | 0.007407049 | 0.0698057 | 0.9025454 | 0.9618324 | MBD1 | 4152 |
| ENSG00000154832 | 1503.788983 | -0.089408259 | 0.0750684 | 0.1575795 | 0.3975565 | CXXC1 | 30827 |
| ENSG00000154839 | 1167.228915 | 0.071749281 | 0.0803322 | 0.2785151 | 0.5467586 | SKA1 | 220134 |
| ENSG00000082212 | 1212.481982 | -0.008785298 | 0.075019 | 0.8878842 | 0.9545517 | ME2 | 4200 |
| ENSG00000141642 | 74.66419489 | 0.060265944 | 0.1411769 | 0.2637967 | 0.5316478 | ELAC1 | 55520 |
| ENSG00000141646 | 1191.85263 | -0.039024782 | 0.080821 | 0.5487122 | 0.7701883 | SMAD4 | 4089 |
| ENSG00000176624 | 739.1522673 | -0.152513466 | 0.1037212 | 0.0507725 | 0.194132 | MEX3C | 51320 |
| ENSG00000134046 | 1514.17201 | -0.098762415 | 0.0774155 | 0.1266952 | 0.349534 | MBD2 | 8932 |
| ENSG00000101751 | 237.9830958 | -0.051651032 | 0.1173556 | 0.4482116 | 0.6957554 | POLI | 11201 |
| ENSG00000166845 | 925.7616302 | 0.205885693 | 0.0980335 | 0.0088508 | 0.0585757 | C18orf54 | 162681 |
| ENSG00000041353 | 33.78859207 | 0.044424782 | 0.1416183 | 0.2291242 | NA | RAB27B | 5874 |
| ENSG00000166510 | 133.9755322 | 0.046333297 | 0.1246866 | 0.4613314 | 0.705619 | CCDC68 | 80323 |
| ENSG00000091164 | 1201.25211 | -0.223412805 | 0.0915704 | 0.0032463 | 0.0271875 | TXNL1 | 9352 |
| ENSG00000091157 | 509.2209393 | 0.039696765 | 0.0944783 | 0.5531435 | 0.7740184 | WDR7 | 23335 |
| ENSG00000119547 | 138.2396999 | 0.074756711 | 0.1377653 | 0.2577573 | 0.5246319 | ONECUT2 | 9480 |
| ENSG00000066926 | 1228.313899 | -0.145454643 | 0.0847614 | 0.0362473 | 0.1568165 | FECH | 2235 |
| ENSG00000134440 | 6471.691878 | 0.045891321 | 0.0533473 | 0.3476184 | 0.6138368 | NARS1 | 4677 |
| ENSG00000267040 | 45.74005132 | -0.018811235 | 0.1314083 | 0.6450699 | 0.8347249 | ATP8B1-AS1 | 100505549 |
| ENSG00000081923 | 436.467304 | 0.02833023 | 0.0978965 | 0.6818528 | 0.8541257 | ATP8B1 | 5205 |
| ENSG00000049759 | 5305.680259 | 0.391528699 | 0.0647029 | 1.66E-10 | 1.35E-08 | NEDD4L | 23327 |
| ENSG00000198796 | 927.8156985 | 1.004043432 | 0.1075089 | 5.79E-22 | 1.93E-19 | ALPK2 | 115701 |
| ENSG00000267257 | 17.80471774 | 0.035373805 | 0.1407513 | 0.1859142 | NA | NA | NA |
| ENSG00000267226 | 12.83094261 | 0.028104684 | 0.1385224 | 0.2650446 | NA | MALT1-AS1 | 101927322 |
| ENSG00000172175 | 1204.462445 | -0.140590782 | 0.0856585 | 0.0433718 | 0.1763404 | MALT1 | 10892 |
| ENSG00000267010 | 65.70409153 | -0.009677817 | 0.1262286 | 0.8423468 | 0.9350382 | NA | NA |
| ENSG00000074657 | 1142.508802 | -0.149602836 | 0.0893565 | 0.0372615 | 0.159758 | ZNF532 | 55205 |
| ENSG00000166562 | 648.8563326 | -0.047939509 | 0.0897146 | 0.4840883 | 0.7224525 | SEC11C | 90701 |
| ENSG00000074695 | 4137.973664 | -0.107539162 | 0.0595821 | 0.0437602 | 0.1774383 | LMAN1 | 3998 |
| ENSG00000183287 | 17.87699819 | 0.026517613 | 0.1370647 | 0.367703 | NA | CCBE1 | 147372 |
| ENSG00000141682 | 1261.667262 | 0.714098859 | 0.099424 | 4.38E-14 | 6.73E-12 | PMAIP1 | 5366 |
| ENSG00000267316 | 19.20166323 | 0.027938199 | 0.1376055 | 0.3338802 | NA | NA | NA |
| ENSG00000267279 | 258.8532817 | 0.01291787 | 0.1070598 | 0.8480143 | 0.9375782 | NA | NA |
| ENSG00000176641 | 57.75227193 | 0.044310638 | 0.1367821 | 0.3506942 | 0.6164725 | RNF152 | 220441 |
| ENSG00000197563 | 729.0333225 | -0.284296272 | 0.1108436 | 0.0014994 | 0.0154038 | PIGN | 23556 |
| ENSG00000134444 | 910.1575994 | 0.063808841 | 0.091354 | 0.3588283 | 0.6220519 | RELCH | 57614 |
| ENSG00000141655 | 86.57013607 | 0.050358945 | 0.1332179 | 0.3745414 | 0.6359963 | TNFRSF11A | 8792 |
| ENSG00000141664 | 286.9300105 | -0.100954344 | 0.1259284 | 0.1894296 | 0.4403437 | ZCCHC2 | 54877 |
| ENSG00000081913 | 358.9951614 | 0.084447887 | 0.1150482 | 0.2587481 | 0.5253712 | PHLPP1 | 23239 |
| ENSG00000171791 | 168.7877526 | 0.043449526 | 0.1208089 | 0.501826 | 0.7364107 | BCL2 | 596 |
| ENSG00000119537 | 1562.117723 | 0.090672717 | 0.0751473 | 0.1521572 | 0.389513 | KDSR | 2531 |
| ENSG00000119541 | 1575.081249 | -0.02688752 | 0.0701532 | 0.6545225 | 0.8402547 | VPS4B | 9525 |
| ENSG00000221887 | 62.4114499 | 0.014181145 | 0.1277129 | 0.7664639 | 0.8994294 | HMSD | 284293 |
| ENSG00000166401 | 462.7917535 | 0.183422181 | 0.128253 | 0.0374122 | 0.160195 | SERPINB8 | 5271 |
| ENSG00000171451 | 173.9393019 | 0.127604934 | 0.1606935 | 0.1105115 | 0.3222505 | DSEL | 92126 |
| ENSG00000166479 | 2618.751374 | -0.170438313 | 0.0722066 | 0.0066451 | 0.047015 | TMX3 | 54495 |
| ENSG00000150636 | 19.04760303 | 0.010846358 | 0.133859 | 0.7161891 | NA | CCDC102B | 79839 |
| ENSG00000176225 | 505.5992995 | 0.148054518 | 0.1172702 | 0.0707707 | 0.2421213 | RTTN | 25914 |
| ENSG00000170677 | 525.1861111 | -0.178058048 | 0.1215678 | 0.0376701 | 0.161004 | SOCS6 | 9306 |
| ENSG00000075336 | 679.1699145 | 0.11287504 | 0.10109 | 0.1335313 | 0.3601571 | TIMM21 | 29090 |
| ENSG00000166347 | 768.7669173 | 0.006202085 | 0.087781 | 0.9275614 | 0.9720836 | CYB5A | 1528 |
| ENSG00000133313 | 1931.480422 | -0.073188289 | 0.0692682 | 0.2199255 | 0.479502 | CNDP2 | 55748 |
| ENSG00000264247 | 100.1944792 | 0.023782479 | 0.123867 | 0.672782 | 0.8500061 | ZNF407-AS1 | 400657 |
| ENSG00000215421 | 473.7725874 | -0.108450196 | 0.110772 | 0.157663 | 0.3975891 | ZNF407 | 55628 |
| ENSG00000180011 | 556.3345031 | 0.053228234 | 0.1015909 | 0.4531408 | 0.6987417 | PTGR3 | 284273 |

| | | | | | | | | |
|---|---|---|---|---|---|---|---|---|
| ENSG00000179981 | 105.6095672 | 0.008134566 | 0.12147 | 0.8832803 | 0.9528582 | TSHZ1 | | 10194 |
| ENSG00000101493 | 30.65080882 | 0.059484117 | 0.149305 | 0.1323484 | NA | ZNF516 | | 9658 |
| ENSG00000130856 | 418.5486207 | 0.087238549 | 0.1090392 | 0.2397228 | 0.5053605 | ZNF236 | | 7776 |
| ENSG00000197971 | 937.935611 | -0.13472664 | 0.0959368 | 0.0692736 | 0.239121 | MBP | | 4155 |
| ENSG00000166377 | 292.74548 | 4.30E-05 | 0.1041352 | 0.9992393 | 0.9994104 | ATP9B | | 374868 |
| ENSG00000267655 | 10.96128338 | -0.000422932 | 0.1346932 | 0.9821459 | NA | NA | NA | |
| ENSG00000131196 | 278.2202919 | 0.174142571 | 0.161989 | 0.0577263 | 0.2126462 | NFATC1 | | 4772 |
| ENSG00000060069 | 1046.958995 | 0.00676849 | 0.0769387 | 0.9160265 | 0.9666732 | CTDP1 | | 9150 |
| ENSG00000122490 | 1205.843585 | -0.091811528 | 0.0802512 | 0.1635001 | 0.4062348 | SLC66A2 | | 80148 |
| ENSG00000226742 | 341.3313087 | -0.097547008 | 0.122839 | 0.2015548 | 0.4556244 | HSBP1L1 | | 440498 |
| ENSG00000141759 | 2220.754852 | 0.014386802 | 0.0644065 | 0.8010633 | 0.9169162 | TXNL4A | | 10907 |
| ENSG00000267127 | 9.059776588 | -0.010096624 | 0.1357809 | 0.6017076 | NA | NA | NA | |
| ENSG00000101546 | 478.0231205 | -0.028931326 | 0.0964691 | 0.673623 | 0.8501877 | RBFA | | 79863 |
| ENSG00000261126 | 36.76705836 | -0.032927762 | 0.1354428 | 0.4143101 | NA | RBFADN | | 100506070 |
| ENSG00000101544 | 1261.355389 | -0.15296073 | 0.0842606 | 0.0275814 | 0.1299105 | ADNP2 | | 22850 |
| ENSG00000267270 | 37.24334975 | 0.030868036 | 0.1356282 | 0.4225356 | NA | PARD6G-AS | | 100130522 |
| ENSG00000178184 | 228.7301548 | -0.046055464 | 0.1158139 | 0.4959538 | 0.7311922 | PARD6G | | 84552 |
| ENSG00000196476 | 223.3382354 | 0.031121309 | 0.1113848 | 0.6432769 | 0.8332355 | C20orf96 | | 140680 |
| ENSG00000177764 | 121.9979399 | 0.054487521 | 0.1299947 | 0.3945769 | 0.6549057 | NA | NA | |
| ENSG00000225377 | 67.10732381 | 0.026916451 | 0.128815 | 0.5924228 | 0.801493 | NRSN2-AS1 | | 100507459 |
| ENSG00000177732 | 1003.342208 | -0.098728977 | 0.0874321 | 0.1544168 | 0.3927156 | SOX12 | | 6666 |
| ENSG00000125841 | 959.8992443 | 0.116614938 | 0.0904772 | 0.1023179 | 0.3064005 | NRSN2 | | 80023 |
| ENSG00000101255 | 1269.256764 | 0.755243118 | 0.0965374 | 3.36E-16 | 6.89E-14 | TRIB3 | | 57761 |
| ENSG00000125826 | 1750.496395 | 0.322375631 | 0.0801653 | 8.06E-06 | 0.0002091 | RBCK1 | | 10616 |
| ENSG00000125875 | 1468.120452 | -0.070498075 | 0.0745163 | 0.2616578 | 0.5292042 | TBC1D20 | | 128637 |
| ENSG00000101266 | 4477.861335 | -0.165503256 | 0.0605938 | 0.0024644 | 0.0221778 | CSNK2A1 | | 1457 |
| ENSG00000271303 | 199.1154431 | 0.277957785 | 0.2189583 | 0.0175708 | 0.0947659 | SRXN1 | | 140809 |
| ENSG00000125898 | 297.3014674 | 0.216727954 | 0.1676633 | 0.0299483 | 0.1382656 | FAM110A | | 83541 |
| ENSG00000125818 | 2150.384116 | 0.073677935 | 0.0731775 | 0.2327387 | 0.4951049 | PSMF1 | | 9491 |
| ENSG00000215388 | 9.244356936 | -0.002831385 | 0.1351412 | 0.8866561 | NA | NA | NA | |
| ENSG00000125895 | 34.87001497 | 0.033627095 | 0.1360717 | 0.3968334 | NA | TMEM74B | | 55321 |
| ENSG00000244588 | 17.5528979 | -0.013145713 | 0.1345553 | 0.6356032 | NA | RAD21L1 | | 642636 |
| ENSG00000101298 | 334.4461533 | -0.02718729 | 0.1028677 | 0.6916408 | 0.8594028 | SNPH | | 9751 |
| ENSG00000125775 | 27.46576062 | 0.066400297 | 0.1550146 | 0.0813938 | NA | SDCBP2 | | 27111 |
| ENSG00000234684 | 115.0162055 | 0.03827659 | 0.1252924 | 0.5187735 | 0.7476611 | SDCBP2-AS | | 100507495 |
| ENSG00000088832 | 12770.54511 | -0.038059194 | 0.054501 | 0.4459806 | 0.6940447 | FKBP1A | | 2280 |
| ENSG00000088833 | 3528.631062 | -0.103311545 | 0.0631924 | 0.0650225 | 0.2301601 | NSFL1C | | 55968 |
| ENSG00000196209 | 36.16348132 | -0.000151758 | 0.1303378 | 0.9949786 | NA | SIRPB2 | | 284759 |
| ENSG00000101307 | 121.8464148 | 0.116470718 | 0.1701636 | 0.1086256 | 0.3188079 | SIRPB1 | | 10326 |
| ENSG00000198053 | 2995.560041 | -0.295297087 | 0.0676619 | 2.08E-06 | 6.43E-05 | SIRPA | | 140885 |
| ENSG00000125834 | 1492.613952 | 0.05707982 | 0.0724573 | 0.3555868 | 0.6192724 | STK35 | | 140901 |
| ENSG00000125835 | 5554.639752 | -0.13037756 | 0.060198 | 0.0161017 | 0.0885515 | SNRPB | | 6628 |
| ENSG00000088876 | 246.0553537 | 0.164303871 | 0.1757169 | 0.0666151 | 0.2333811 | ZNF343 | | 79175 |
| ENSG00000101361 | 9133.750935 | 0.044054789 | 0.0551436 | 0.3810685 | 0.641901 | NOP56 | | 10528 |
| ENSG00000101365 | 2507.030548 | -0.072959927 | 0.0663581 | 0.2078872 | 0.4641304 | IDH3B | | 3420 |
| ENSG00000132635 | 471.9357979 | 0.45005152 | 0.1406425 | 0.0001072 | 0.0018469 | PCED1A | | 64773 |
| ENSG00000215305 | 818.9792083 | -0.032991981 | 0.0832011 | 0.6184917 | 0.817617 | VPS16 | | 64601 |
| ENSG00000132670 | 1690.352841 | -0.095428378 | 0.0731642 | 0.1249357 | 0.346864 | PTPRA | | 5786 |
| ENSG00000125787 | 8.986280955 | 0.031526998 | 0.1405006 | 0.13159 | NA | GNRH2 | | 2797 |
| ENSG00000125901 | 1190.308942 | -0.064212921 | 0.0779435 | 0.3185603 | 0.5874807 | MRPS26 | | 64949 |
| ENSG00000185019 | 240.1262628 | 0.540316095 | 0.1891296 | 0.0002534 | 0.0037423 | UBOX5 | | 22888 |
| ENSG00000215251 | 893.3875999 | -0.179500872 | 0.1001725 | 0.0217408 | 0.1096334 | FASTKD5 | | 60493 |
| ENSG00000088899 | 630.2340622 | 0.065043372 | 0.0956337 | 0.3593525 | 0.6225117 | LZTS3 | | 9762 |
| ENSG00000198171 | 1627.252621 | 0.038162213 | 0.0713653 | 0.5315702 | 0.7569513 | DDRGK1 | | 65992 |
| ENSG00000125877 | 1222.1156 | 0.210983261 | 0.0918209 | 0.0053258 | 0.0398527 | ITPA | | 3704 |
| ENSG00000088836 | 447.7965281 | -0.101944001 | 0.1104015 | 0.1795984 | 0.429016 | SLC4A11 | | 83959 |
| ENSG00000088854 | 533.4658032 | 0.016680635 | 0.0925366 | 0.807957 | 0.9200093 | DNAAF9 | | 25943 |
| ENSG00000088812 | 1714.644123 | -0.129616355 | 0.0784596 | 0.0484974 | 0.1887699 | ATRN | | 8455 |
| ENSG00000101220 | 1635.967475 | 0.054855206 | 0.0736451 | 0.3782366 | 0.6395747 | ADISSP | | 54976 |
| ENSG00000101222 | 10.76083537 | 0.009400256 | 0.1351632 | 0.682902 | NA | SPEF1 | | 25876 |
| ENSG00000125817 | 3606.943788 | 0.038619208 | 0.0630226 | 0.4898336 | 0.7266633 | CENPB | | 1059 |
| ENSG00000101224 | 2959.035946 | -0.036818065 | 0.0629507 | 0.5113604 | 0.7421105 | CDC25B | | 994 |
| ENSG00000125843 | 283.326865 | 0.014756397 | 0.1062421 | 0.827619 | 0.9288449 | AP5S1 | | 55317 |
| ENSG00000088888 | 1503.034958 | -0.034486838 | 0.0729007 | 0.5762554 | 0.7897843 | MAVS | | 57506 |
| ENSG00000229539 | 14.51583895 | -0.023511016 | 0.1369464 | 0.3727585 | NA | NA | NA | |
| ENSG00000125779 | 1184.562081 | 0.155612865 | 0.0864925 | 0.0279006 | 0.1312026 | PANK2 | | 80025 |
| ENSG00000101236 | 334.5559685 | 0.110879708 | 0.1306705 | 0.1594513 | 0.4003764 | RNF24 | | 11237 |
| ENSG00000088826 | 1497.456331 | 0.252571202 | 0.0864255 | 0.0006736 | 0.0081512 | SMOX | | 54498 |
| ENSG00000171867 | 5115.58128 | 0.114693442 | 0.0584932 | 0.0303387 | 0.1393817 | PRNP | | 5621 |
| ENSG00000101265 | 213.1772009 | -0.001831704 | 0.11107 | 0.9762858 | 0.9912207 | RASSF2 | | 9770 |

| | | | | | | | |
|---|---|---|---|---|---|---|---|
| ENSG00000089057 | 1095.827879 | 0.046280383 | 0.0779167 | 0.4642269 | 0.7082689 | SLC23A2 | 9962 |
| ENSG00000089063 | 1558.059294 | 0.036356337 | 0.0722608 | 0.5542792 | 0.7749708 | TMEM230 | 29058 |
| ENSG00000132646 | 5999.355845 | -0.182218667 | 0.0599068 | 0.000826 | 0.0095528 | PCNA | 5111 |
| ENSG00000101290 | 1815.781549 | -0.256185211 | 0.0832989 | 0.0004013 | 0.0054472 | CDS2 | 8760 |
| ENSG00000125772 | 264.7686885 | 0.17194087 | 0.1787459 | 0.0597999 | 0.2174729 | GPCPD1 | 56261 |
| ENSG00000171984 | 48.19218903 | -0.009569588 | 0.1291911 | 0.8227324 | 0.9272789 | SHLD1 | 149840 |
| ENSG00000089195 | 1396.593632 | 0.072681858 | 0.075815 | 0.2526797 | 0.5193651 | TRMT6 | 51605 |
| ENSG00000125885 | 2299.744388 | 0.086214461 | 0.0676933 | 0.1434005 | 0.3750698 | MCM8 | 84515 |
| ENSG00000088766 | 1145.845995 | -0.120965741 | 0.0862458 | 0.0812332 | 0.2647853 | CRLS1 | 54675 |
| ENSG00000125872 | 2127.763453 | -0.762989094 | 0.0886171 | 4.59E-19 | 1.31E-16 | LRRN4 | 164312 |
| ENSG00000101311 | 928.4625629 | 0.480877485 | 0.11465 | 2.16E-06 | 6.63E-05 | FERMT1 | 55612 |
| ENSG00000125827 | 1198.703031 | -0.029932614 | 0.0753854 | 0.6338035 | 0.8257229 | TMX4 | 56255 |
| ENSG00000182621 | 93.97597813 | 0.025322235 | 0.1247361 | 0.649245 | 0.8366057 | PLCB1 | 23236 |
| ENSG00000101333 | 402.5022018 | -0.504598817 | 0.178527 | 0.0002912 | 0.0042093 | PLCB4 | 5332 |
| ENSG00000132623 | 153.8234876 | 0.011863826 | 0.1166112 | 0.8479953 | 0.9375782 | ANKEF1 | 63926 |
| ENSG00000125863 | 931.147152 | 0.055988033 | 0.0827813 | 0.3960455 | 0.6562498 | MKKS | 8195 |
| ENSG00000149346 | 44.33993041 | -0.011987791 | 0.1307505 | 0.7625209 | 0.8982682 | SLX4IP | 128710 |
| ENSG00000101384 | 868.3886359 | 0.631133652 | 0.1029992 | 6.13E-11 | 5.36E-09 | JAG1 | 182 |
| ENSG00000132640 | 879.2631575 | -0.03927443 | 0.0840199 | 0.5587382 | 0.7775341 | BTBD3 | 22903 |
| ENSG00000101230 | 11.84846991 | 0.020795328 | 0.1367642 | 0.3910359 | NA | ISM1 | 140862 |
| ENSG00000089123 | 175.3900004 | 0.060185067 | 0.1242932 | 0.3866231 | 0.6473831 | TASP1 | 55617 |
| ENSG00000089048 | 1205.068405 | 0.18004219 | 0.0890999 | 0.0136374 | 0.0791741 | ESF1 | 51575 |
| ENSG00000101247 | 338.525467 | -0.013932715 | 0.1025384 | 0.8366795 | 0.9322234 | NDUFAF5 | 79133 |
| ENSG00000172264 | 243.0422049 | -0.009847997 | 0.1074485 | 0.8814498 | 0.9522588 | MACROD2 | 140733 |
| ENSG00000089177 | 516.2986162 | -0.1494059 | 0.1161919 | 0.0672009 | 0.2347409 | KIF16B | 55614 |
| ENSG00000125870 | 1469.961547 | -0.054119494 | 0.0743055 | 0.386841 | 0.647427 | SNRPB2 | 6629 |
| ENSG00000125864 | 59.22165935 | 0.115595874 | 0.1965993 | 0.0455558 | 0.1822781 | BFSP1 | 631 |
| ENSG00000125868 | 6666.821987 | -0.0290695 | 0.0572106 | 0.58389 | 0.7952914 | DSTN | 11034 |
| ENSG00000125844 | 4432.69314 | -0.129122821 | 0.0645921 | 0.0239561 | 0.1174264 | RRBP1 | 6238 |
| ENSG00000089006 | 3550.254275 | -0.074130549 | 0.0606366 | 0.1710985 | 0.4174188 | SNX5 | 27131 |
| ENSG00000212232 | 140.6692132 | -0.067990956 | 0.13404 | 0.2949376 | 0.5650592 | SNORD17 | 692086 |
| ENSG00000125871 | 1340.885822 | 0.579764404 | 0.096733 | 1.50E-10 | 1.23E-08 | MGME1 | 92667 |
| ENSG00000232838 | 55.48644421 | 0.064384688 | 0.1472782 | 0.1923939 | 0.4436149 | PET117 | 100303755 |
| ENSG00000149474 | 234.2138578 | 0.201942136 | 0.1797885 | 0.039954 | 0.1675182 | KAT14 | 57325 |
| ENSG00000125846 | 200.7709235 | 0.15756918 | 0.1711996 | 0.073146 | 0.2472589 | ZNF133 | 7692 |
| ENSG00000089091 | 152.1058097 | 0.087064417 | 0.1406107 | 0.2145195 | 0.4722583 | DZANK1 | 55184 |
| ENSG00000132664 | 541.6434513 | 0.230938088 | 0.1296458 | 0.0132412 | 0.0774127 | POLR3F | 10621 |
| ENSG00000089050 | 619.564113 | -0.042617728 | 0.0942175 | 0.5380433 | 0.7618965 | RBBP9 | 10741 |
| ENSG00000101310 | 1905.085979 | -0.017466348 | 0.0672142 | 0.7647244 | 0.8986666 | SEC23B | 10483 |
| ENSG00000232388 | 426.4538553 | -0.07737712 | 0.1072452 | 0.2940695 | 0.5640436 | SMIM26 | 388789 |
| ENSG00000125821 | 849.0413012 | -0.103991593 | 0.0933221 | 0.147662 | 0.3815174 | DTD1 | 92675 |
| ENSG00000132669 | 1401.158553 | 0.150678377 | 0.0833756 | 0.0289157 | 0.1350219 | RIN2 | 54453 |
| ENSG00000173418 | 1823.618945 | 0.015715451 | 0.0695986 | 0.794296 | 0.9143759 | NAA20 | 51126 |
| ENSG00000101343 | 973.856825 | -0.05936197 | 0.0826677 | 0.3695266 | 0.63189 | CRNKL1 | 51340 |
| ENSG00000188559 | 869.399203 | -0.077467459 | 0.0873127 | 0.259217 | 0.525883 | RALGAPA2 | 57186 |
| ENSG00000088970 | 246.0068157 | -0.125716468 | 0.1448066 | 0.1215187 | 0.3414625 | KIZ | 55857 |
| ENSG00000088930 | 3981.369232 | -0.118504398 | 0.0628067 | 0.0338756 | 0.1494339 | XRN2 | 22803 |
| ENSG00000227063 | 10.38454523 | -0.007730277 | 0.1351136 | 0.7250773 | NA | NA | NA |
| ENSG00000132661 | 634.0369391 | 0.137056854 | 0.1112681 | 0.0844255 | 0.2713742 | NXT1 | 29107 |
| ENSG00000125812 | 729.4448574 | 0.009574442 | 0.0860804 | 0.8870636 | 0.9545517 | GZF1 | 64412 |
| ENSG00000125814 | 289.2867051 | 0.28971709 | 0.1759647 | 0.0102441 | 0.0645075 | NAPB | 63908 |
| ENSG00000101439 | 635.5102286 | 0.070008304 | 0.0945988 | 0.3236491 | 0.5924692 | CST3 | 1471 |
| ENSG00000101474 | 2965.24979 | -0.027811737 | 0.0620723 | 0.6161239 | 0.815878 | APMAP | 57136 |
| ENSG00000154930 | 238.3970341 | 0.016942226 | 0.1110956 | 0.788076 | 0.9116361 | ACSS1 | 84532 |
| ENSG00000197586 | 2223.659844 | -0.041727793 | 0.0648324 | 0.4680815 | 0.7103207 | ENTPD6 | 955 |
| ENSG00000100994 | 6425.876206 | 0.19252136 | 0.063404 | 0.0007722 | 0.0090837 | PYGB | 5834 |
| ENSG00000100997 | 1247.153829 | 0.053950415 | 0.0764193 | 0.3870337 | 0.6475138 | ABHD12 | 26090 |
| ENSG00000101003 | 1200.653591 | -0.154987942 | 0.0867679 | 0.0281978 | 0.132294 | GINS1 | 9837 |
| ENSG00000101004 | 294.1725348 | -0.0393353 | 0.1069136 | 0.5689122 | 0.7847255 | NINL | 22981 |
| ENSG00000170191 | 534.1679072 | 0.090447031 | 0.1027442 | 0.2226614 | 0.4826328 | NANP | 140838 |
| ENSG00000213742 | 44.39203005 | 0.062221152 | 0.1484151 | 0.1675805 | 0.4125292 | ZNF337-AS1 | 102724826 |
| ENSG00000130684 | 375.8520029 | 0.000850114 | 0.0984173 | 0.9907609 | 0.9960477 | ZNF337 | 26152 |
| ENSG00000226465 | 11.78456377 | 0.010520707 | 0.1350916 | 0.6606999 | NA | NA | NA |
| ENSG00000175170 | 8.518873262 | 0.017428766 | 0.1367629 | 0.3916213 | NA | NA | NA |
| ENSG00000204556 | 19.29297938 | 0.004707535 | 0.1334261 | 0.874922 | NA | NA | NA |
| ENSG00000149531 | 207.2878865 | 0.000425265 | 0.1100351 | 0.9967689 | 0.998947 | NA | NA |
| ENSG00000101294 | 3524.794626 | 0.170222941 | 0.0640596 | 0.0030047 | 0.0256938 | HM13 | 81502 |
| ENSG00000101898 | 22.30973314 | 0.013624782 | 0.1337584 | 0.666129 | NA | MCTS2 | 100101490 |
| ENSG00000235313 | 15.31400885 | 0.024079947 | 0.1369467 | 0.3758376 | NA | NA | NA |
| ENSG00000125968 | 1602.470875 | 0.121507288 | 0.0826302 | 0.072931 | 0.2469116 | ID1 | 3397 |

| | | | | | | | |
|---|---|---|---|---|---|---|---|
| ENSG00000171552 | 6879.892282 | 0.104565913 | 0.05877 | 0.0485838 | 0.1890328 | BCL2L1 | 598 |
| ENSG00000088325 | 10847.12229 | -0.021533774 | 0.0514841 | 0.6508042 | 0.8378754 | TPX2 | 22974 |
| ENSG00000101306 | 14.79093727 | 0.001820055 | 0.1339708 | 0.9475099 | NA | MYLK2 | 85366 |
| ENSG00000088356 | 1603.95728 | 0.331429589 | 0.0845014 | 1.15E-05 | 0.0002858 | PDRG1 | 81572 |
| ENSG00000101337 | 2647.886478 | 0.043581668 | 0.0644315 | 0.4431181 | 0.6918021 | TM9SF4 | 9777 |
| ENSG00000235217 | 10.24626633 | -0.00393675 | 0.1348335 | 0.858459 | NA | TSPY26P | 128854 |
| ENSG00000126003 | 1540.134152 | -0.013208406 | 0.0707075 | 0.8264671 | 0.9286016 | PLAGL2 | 5326 |
| ENSG00000101346 | 2720.129386 | 0.093893896 | 0.0680666 | 0.1127357 | 0.325715 | POFUT1 | 23509 |
| ENSG00000101350 | 2989.530248 | -0.136915112 | 0.0681297 | 0.0215533 | 0.1092606 | KIF3B | 9371 |
| ENSG00000171456 | 5517.209145 | -0.161230302 | 0.0617154 | 0.0036726 | 0.0298398 | ASXL1 | 171023 |
| ENSG00000197183 | 43.00714274 | -0.004959826 | 0.1294824 | 0.903538 | 0.9618565 | NOL4L | 140688 |
| ENSG00000149600 | 471.7277537 | -0.074601744 | 0.108786 | 0.3076783 | 0.5769089 | COMMD7 | 149951 |
| ENSG00000088305 | 265.8106248 | 0.040880747 | 0.1100039 | 0.549509 | 0.7709362 | DNMT3B | 1789 |
| ENSG00000101367 | 7365.025724 | -0.099813631 | 0.0551728 | 0.0474385 | 0.1860119 | MAPRE1 | 22919 |
| ENSG00000260257 | 62.71410754 | 0.018295401 | 0.1277261 | 0.7085579 | 0.8682085 | LOC119746! | 119746555 |
| ENSG00000101391 | 1421.85233 | -0.025100111 | 0.0718126 | 0.6807998 | 0.8538613 | CDK5RAP1 | 51654 |
| ENSG00000101400 | 590.2439732 | 0.001636026 | 0.0894588 | 0.9817099 | 0.9932057 | SNTA1 | 6640 |
| ENSG00000078699 | 807.5080916 | 0.08806489 | 0.0936501 | 0.2175191 | 0.4768298 | CBFA2T2 | 9139 |
| ENSG00000125967 | 493.842236 | 0.017722454 | 0.0971882 | 0.7955821 | 0.9148766 | NECAB3 | 63941 |
| ENSG00000182584 | 8.329359394 | 0.01022472 | 0.135701 | 0.6144166 | NA | NA | NA |
| ENSG00000101412 | 3011.065354 | 0.506837287 | 0.0733949 | 3.93E-13 | 5.33E-11 | E2F1 | 1869 |
| ENSG00000101417 | 278.7063286 | 0.006615219 | 0.1064609 | 0.9223191 | 0.9701942 | PXMP4 | 11264 |
| ENSG00000131061 | 167.2698666 | -0.024776994 | 0.1178111 | 0.6890128 | 0.858679 | ZNF341 | 84905 |
| ENSG00000101421 | 2587.414076 | 0.003563309 | 0.0618585 | 0.9461552 | 0.9784895 | CHMP4B | 128866 |
| ENSG00000228265 | 25.20851319 | 0.025269972 | 0.1357882 | 0.4412079 | NA | NA | NA |
| ENSG00000125970 | 4557.232179 | -0.006637913 | 0.0563723 | 0.8959129 | 0.9584283 | RALY | 22913 |
| ENSG00000229472 | 43.76576071 | 0.007106563 | 0.1293659 | 0.8686374 | 0.9471139 | NA | NA |
| ENSG00000125977 | 8169.182063 | 0.092771718 | 0.0537399 | 0.0615707 | 0.2214309 | EIF2S2 | 8894 |
| ENSG00000101444 | 7267.884447 | -0.008195217 | 0.0526691 | 0.860853 | 0.9439242 | AHCY | 191 |
| ENSG00000272945 | 18.29599488 | -0.015349747 | 0.1347282 | 0.5895527 | NA | NA | NA |
| ENSG00000078747 | 5428.516441 | -0.021723651 | 0.0580811 | 0.6807891 | 0.8538613 | ITCH | 83737 |
| ENSG00000125971 | 1973.993752 | -0.098807443 | 0.0735754 | 0.1126405 | 0.325537 | DYNLRB1 | 83658 |
| ENSG00000101460 | 342.839874 | 0.281914122 | 0.1628551 | 0.009463 | 0.0615017 | MAP1LC3A | 84557 |
| ENSG00000101464 | 565.6333091 | -0.076083663 | 0.1023991 | 0.2939803 | 0.5639652 | PIGU | 128869 |
| ENSG00000198646 | 2155.964745 | -0.142674945 | 0.0735412 | 0.0236839 | 0.1166324 | NCOA6 | 23054 |
| ENSG00000078804 | 521.4574094 | 0.145083463 | 0.1162804 | 0.0751404 | 0.2517827 | TP53INP2 | 58476 |
| ENSG00000131067 | 600.4311707 | -0.044835463 | 0.0920354 | 0.5154445 | 0.7451617 | GGT7 | 2686 |
| ENSG00000131069 | 769.6119722 | 0.043424719 | 0.092178 | 0.5298835 | 0.7556526 | ACSS2 | 55902 |
| ENSG00000100983 | 2889.04912 | -0.001186747 | 0.064885 | 0.981727 | 0.9932057 | GSS | 2937 |
| ENSG00000100991 | 2392.313229 | -0.058816237 | 0.0658952 | 0.3083961 | 0.5777667 | TRPC4AP | 26133 |
| ENSG00000088298 | 454.7842593 | 0.034521109 | 0.1019204 | 0.6189976 | 0.8179246 | EDEM2 | 55741 |
| ENSG00000101000 | 1165.191257 | 0.188554007 | 0.0971466 | 0.0147403 | 0.0832198 | PROCR | 10544 |
| ENSG00000126005 | 705.5223566 | 0.132921562 | 0.1015846 | 0.0812422 | 0.2647853 | MMP24OS | 101410538 |
| ENSG00000125966 | 16.50862861 | 0.012165388 | 0.1345746 | 0.6578182 | NA | MMP24 | 10893 |
| ENSG00000242372 | 2665.680206 | 0.080272432 | 0.0664533 | 0.1686115 | 0.4142934 | EIF6 | 3692 |
| ENSG00000101019 | 1425.874274 | 0.029491493 | 0.0723068 | 0.6320307 | 0.8251856 | UQCC1 | 55245 |
| ENSG00000126001 | 2861.152381 | 0.187940276 | 0.0689947 | 0.0020994 | 0.0195876 | CEP250 | 11190 |
| ENSG00000125991 | 4074.359775 | -0.011886111 | 0.0589867 | 0.8263739 | 0.9286016 | ERGIC3 | 51614 |
| ENSG00000088340 | 42.00083646 | -0.082238825 | 0.1637916 | 0.0820258 | 0.266521 | FER1L4 | 80307 |
| ENSG00000061656 | 110.2659385 | 0.050285291 | 0.1296178 | 0.4044917 | 0.6614808 | SPAG4 | 6676 |
| ENSG00000214078 | 3021.498714 | -0.167004878 | 0.0681993 | 0.0054608 | 0.0405875 | CPNE1 | 8904 |
| ENSG00000244462 | 2693.802422 | -0.356771703 | 0.0733986 | 1.44E-07 | 6.49E-06 | RBM12 | 10137 |
| ENSG00000244005 | 893.5128943 | -0.066234124 | 0.0846238 | 0.3222535 | 0.5910188 | NFS1 | 9054 |
| ENSG00000125995 | 1583.721916 | -0.074811183 | 0.0774975 | 0.2451455 | 0.5108911 | ROMO1 | 140823 |
| ENSG00000131051 | 6596.546948 | 0.041754812 | 0.0551693 | 0.409299 | 0.6643076 | RBM39 | 9584 |
| ENSG00000025293 | 1437.424677 | 0.063956251 | 0.078326 | 0.3229003 | 0.5915619 | PHF20 | 51230 |
| ENSG00000171222 | 947.9097521 | 0.107342462 | 0.0918617 | 0.1350777 | 0.3617245 | SCAND1 | 51282 |
| ENSG00000260032 | 7696.086402 | -0.030324745 | 0.0575925 | 0.5611842 | 0.7795039 | NORAD | 647979 |
| ENSG00000088367 | 481.5162844 | -0.164257592 | 0.1323984 | 0.0582215 | 0.2137962 | EPB41L1 | 2036 |
| ENSG00000131043 | 1154.483251 | 0.004222414 | 0.0751622 | 0.9471291 | 0.9787169 | AAR2 | 25980 |
| ENSG00000080845 | 1011.999953 | 0.1674682 | 0.0923033 | 0.0237743 | 0.1168136 | DLGAP4 | 22839 |
| ENSG00000101335 | 2149.455094 | 0.073323453 | 0.1250939 | 0.3029599 | 0.5723556 | MYL9 | 10398 |
| ENSG00000259716 | 21.92896838 | -0.014300077 | 0.1338586 | 0.647452 | NA | NA | NA |
| ENSG00000118707 | 413.950784 | 0.01401578 | 0.1036198 | 0.8376766 | 0.9330505 | TGIF2 | 60436 |
| ENSG00000101084 | 1128.704131 | 0.132072032 | 0.0858341 | 0.0582477 | 0.2138249 | RAB5IF | 55969 |
| ENSG00000101082 | 8.837249263 | -0.009250888 | 0.1357633 | 0.6267272 | NA | SLA2 | 84174 |
| ENSG00000101079 | 967.0097791 | -0.534398752 | 0.1011635 | 9.81E-09 | 5.91E-07 | NDRG3 | 57446 |
| ENSG00000149636 | 1858.906695 | 0.208105313 | 0.0774101 | 0.0019393 | 0.0185069 | DSN1 | 79980 |
| ENSG00000149639 | 1364.49124 | 0.047703137 | 0.0762753 | 0.4534508 | 0.6987417 | MTCL2 | 140710 |
| ENSG00000101342 | 13.78221663 | -0.004300896 | 0.1344873 | 0.8581474 | NA | TLDC2 | 140711 |

| | | | | | | | | |
|---|---|---|---|---|---|---|---|---|
| ENSG00000101347 | 2467.318 | -0.208819361 | 0.0729608 | 0.0011663 | 0.0126499 | SAMHD1 | | 25939 |
| ENSG00000080839 | 1208.311619 | 0.031362086 | 0.0777812 | 0.6044293 | 0.8096718 | RBL1 | | 5933 |
| ENSG00000101353 | 18.45139111 | 0.017233065 | 0.134905 | 0.5583329 | NA | MROH8 | | 140699 |
| ENSG00000118705 | 7248.833768 | -0.08003457 | 0.0540712 | 0.1018078 | 0.3054758 | RPN2 | | 6185 |
| ENSG00000101363 | 1302.912402 | 0.067415284 | 0.0762954 | 0.2902634 | 0.560019 | MANBAL | | 63905 |
| ENSG00000197122 | 1124.288596 | -0.027573652 | 0.0774637 | 0.6654156 | 0.8451364 | SRC | | 6714 |
| ENSG00000166619 | 1569.148049 | 0.06682521 | 0.0722714 | 0.27767 | 0.5461905 | BLCAP | | 10904 |
| ENSG00000132792 | 2156.267131 | 0.014852879 | 0.0647508 | 0.7954068 | 0.9148431 | CTNNBL1 | | 56259 |
| ENSG00000101407 | 1582.555346 | 0.031478227 | 0.0718292 | 0.6072978 | 0.8116146 | TTI1 | | 9675 |
| ENSG00000101413 | 1630.259833 | 0.146807716 | 0.0825754 | 0.0318431 | 0.143558 | RPRD1B | | 58490 |
| ENSG00000198959 | 12771.21016 | 0.658720075 | 0.0710577 | 1.31E-21 | 4.26E-19 | TGM2 | | 7052 |
| ENSG00000149633 | 11.85844918 | 0.037804791 | 0.142323 | 0.1165675 | NA | KIAA1755 | | 85449 |
| ENSG00000224635 | 35.62715623 | 0.038738743 | 0.137809 | 0.3355775 | NA | NA | NA | |
| ENSG00000196756 | 1472.119957 | 0.634351193 | 0.0881685 | 4.36E-14 | 6.73E-12 | SNHG17 | | 388796 |
| ENSG00000235408 | 31.10275726 | -0.017849948 | 0.1335533 | 0.6039645 | NA | NA | NA | |
| ENSG00000225091 | 23.68523215 | 0.028057619 | 0.136401 | 0.4020619 | NA | SNORA71A | | 26777 |
| ENSG00000201512 | 46.57370938 | 0.110579213 | 0.1920376 | 0.0449667 | 0.1806246 | SNORA71C | | 677839 |
| ENSG00000174365 | 152.8482219 | 0.611815678 | 0.2483942 | 0.0006568 | 0.0079834 | SNHG11 | | 128439 |
| ENSG00000170471 | 2522.356435 | -0.024546968 | 0.0668341 | 0.6730372 | 0.8500105 | RALGAPB | | 57148 |
| ENSG00000101442 | 820.5284772 | -0.210963987 | 0.1043591 | 0.0099505 | 0.0634104 | ACTR5 | | 79913 |
| ENSG00000199691 | 14.71038994 | -0.017250912 | 0.1357524 | 0.4985768 | NA | NA | NA | |
| ENSG00000101447 | 2344.867346 | -0.301079864 | 0.0776621 | 1.62E-05 | 0.0003813 | FAM83D | | 81610 |
| ENSG00000101452 | 676.8425459 | -0.02314776 | 0.0880661 | 0.7310849 | 0.880392 | DHX35 | | 60625 |
| ENSG00000198900 | 4808.34267 | -0.022378594 | 0.0577028 | 0.6468981 | 0.8359336 | TOP1 | | 7150 |
| ENSG00000226648 | 43.61856965 | -0.015285158 | 0.1312 | 0.7015571 | 0.8648958 | PLCG1-AS1 | | 101927117 |
| ENSG00000124181 | 3253.066054 | -0.032739031 | 0.0596191 | 0.5411644 | 0.7644022 | PLCG1 | | 5335 |
| ENSG00000174306 | 1806.738668 | 0.404031896 | 0.0808056 | 6.01E-08 | 3.03E-06 | ZHX3 | | 23051 |
| ENSG00000272407 | 8.672159935 | 0.007696424 | 0.1353387 | 0.7144911 | NA | NA | NA | |
| ENSG00000132793 | 668.7368042 | -0.017099488 | 0.0885334 | 0.799409 | 0.9164684 | LPIN3 | | 64900 |
| ENSG00000124177 | 1586.75157 | -0.008475516 | 0.0699753 | 0.8870786 | 0.9545517 | CHD6 | | 84181 |
| ENSG00000124193 | 5770.066916 | -0.226837971 | 0.0664688 | 0.0001581 | 0.0025294 | SRSF6 | | 6431 |
| ENSG00000185513 | 56.43564192 | 0.046130657 | 0.1369589 | 0.3427457 | 0.6092767 | L3MBTL1 | | 26013 |
| ENSG00000101049 | 61.78716806 | -0.788085827 | 0.4349503 | 0.0021069 | 0.019626 | SGK2 | | 10110 |
| ENSG00000101052 | 744.6793595 | -0.269992166 | 0.1122631 | 0.0024934 | 0.0224043 | IFT52 | | 51098 |
| ENSG00000101057 | 5631.30258 | 0.115124877 | 0.0587635 | 0.0300247 | 0.1384595 | MYBL2 | | 4605 |
| ENSG00000124191 | 72.97261201 | 0.087988433 | 0.1595812 | 0.133557 | 0.3601571 | TOX2 | | 84969 |
| ENSG00000149596 | 28.04084069 | 1.549524992 | 0.609628 | 0.0003958 | NA | JPH2 | | 57158 |
| ENSG00000132823 | 958.3800378 | 0.440713887 | 0.1139192 | 9.43E-06 | 0.0002398 | OSER1 | | 51526 |
| ENSG00000223891 | 57.6071696 | 0.072889894 | 0.1526446 | 0.1527221 | 0.3901897 | OSER1-DT | | 100505783 |
| ENSG00000197296 | 714.5923062 | 0.008235208 | 0.0850498 | 0.9024628 | 0.9618324 | FITM2 | | 128486 |
| ENSG00000124120 | 1496.566605 | 0.087303199 | 0.0767033 | 0.1732622 | 0.420502 | TTPAL | | 79183 |
| ENSG00000132824 | 3389.240432 | 0.028058372 | 0.0606815 | 0.6064541 | 0.8112678 | SERINC3 | | 10955 |
| ENSG00000168734 | 346.4008029 | -0.003761944 | 0.1010229 | 0.9543934 | 0.9819668 | PKIG | | 11142 |
| ENSG00000196839 | 202.1767998 | -0.053557603 | 0.1217793 | 0.4214697 | 0.6737677 | ADA | | 100 |
| ENSG00000166913 | 11466.43736 | -0.153535664 | 0.0614588 | 0.0055014 | 0.0407531 | YWHAB | | 7529 |
| ENSG00000101104 | 750.7524394 | -0.001195055 | 0.0854121 | 0.986878 | 0.994767 | PABPC1L | | 80336 |
| ENSG00000025772 | 2412.836377 | 0.286949693 | 0.0727105 | 1.33E-05 | 0.0003213 | TOMM34 | | 10953 |
| ENSG00000101109 | 3920.086452 | -0.044326052 | 0.0614353 | 0.4185168 | 0.6713944 | STK4 | | 6789 |
| ENSG00000124159 | 11.80720559 | 0.005688072 | 0.1346849 | 0.8132961 | NA | MATN4 | | 8785 |
| ENSG00000124145 | 6472.567806 | 0.019995838 | 0.0588011 | 0.7111022 | 0.8691154 | SDC4 | | 6385 |
| ENSG00000204070 | 775.629259 | -0.066235949 | 0.0891435 | 0.3367029 | 0.6044302 | SYS1 | | 90196 |
| ENSG00000244274 | 145.3951742 | 0.068901173 | 0.1341674 | 0.2925006 | 0.5620496 | DBNDD2 | | 55861 |
| ENSG00000124155 | 1979.574355 | 0.09762907 | 0.0717632 | 0.1124186 | 0.3255233 | PIGT | | 51604 |
| ENSG00000101443 | 64.54514387 | -0.078448365 | 0.1542385 | 0.1505829 | 0.3867543 | WFDC2 | | 10406 |
| ENSG00000101457 | 1901.14456 | 0.543509268 | 0.0805616 | 1.19E-12 | 1.52E-10 | DNTTIP1 | | 116092 |
| ENSG00000175063 | 5027.31348 | 0.175855174 | 0.0682952 | 0.0035926 | 0.0293256 | UBE2C | | 11065 |
| ENSG00000124104 | 668.4418521 | 0.044354786 | 0.0885039 | 0.5158313 | 0.7452598 | SNX21 | | 90203 |
| ENSG00000101473 | 307.9945574 | 0.058823538 | 0.1101952 | 0.4107947 | 0.6658101 | ACOT8 | | 10005 |
| ENSG00000132801 | 52.11550426 | 0.084744015 | 0.1641523 | 0.0921292 | 0.287261 | ZSWIM3 | | 140831 |
| ENSG00000168612 | 268.0170124 | 0.020472289 | 0.1062167 | 0.7638201 | 0.8982943 | ZSWIM1 | | 90204 |
| ENSG00000149634 | 11.1170806 | 0.035589514 | 0.1415686 | 0.1265921 | NA | SPATA25 | | 128497 |
| ENSG00000124257 | 25.36157281 | 0.069907309 | 0.1584533 | 0.0573926 | NA | NEURL2 | | 140825 |
| ENSG00000064601 | 4346.801743 | 0.179859739 | 0.0632271 | 0.0015683 | 0.0158743 | CTSA | | 5476 |
| ENSG00000271984 | 20.97015531 | -0.004530341 | 0.1333243 | 0.8767263 | NA | NA | NA | |
| ENSG00000100979 | 9.103741428 | 0.005616027 | 0.1351827 | 0.7900796 | NA | PLTP | | 5360 |
| ENSG00000100982 | 693.3024845 | -0.097144757 | 0.0993257 | 0.1854556 | 0.4358363 | PCIF1 | | 63935 |
| ENSG00000198026 | 1225.167208 | 0.05624013 | 0.0764694 | 0.3720607 | 0.6339958 | ZNF335 | | 63925 |
| ENSG00000124140 | 29.78492808 | 0.016399235 | 0.1328063 | 0.651055 | NA | SLC12A5 | | 57468 |
| ENSG00000124160 | 1381.499687 | -0.025651944 | 0.0735622 | 0.6788816 | 0.8535466 | NCOA5 | | 57727 |
| ENSG00000101017 | 1219.463692 | -0.043482183 | 0.0760789 | 0.4919165 | 0.7282321 | CD40 | | 958 |

| | | | | | | | |
|---|---|---|---|---|---|---|---|
| ENSG00000080189 | 1211.708547 | 0.000753823 | 0.074619 | 0.9911488 | 0.9962622 | SLC35C2 | 51006 |
| ENSG00000062598 | 915.7667551 | -0.070290294 | 0.0855243 | 0.2995877 | 0.5694273 | ELMO2 | 63916 |
| ENSG00000172315 | 703.0288337 | 0.101994785 | 0.0983708 | 0.1647659 | 0.4085071 | TP53RK | 112858 |
| ENSG00000101040 | 901.8088983 | -0.126711755 | 0.0997926 | 0.0920474 | 0.287261 | ZMYND8 | 23613 |
| ENSG00000124151 | 7462.887106 | -0.534414108 | 0.0736297 | 3.23E-14 | 5.17E-12 | NCOA3 | 8202 |
| ENSG00000196562 | 34.42680355 | -0.035265211 | 0.1369178 | 0.3642583 | NA | SULF2 | 55959 |
| ENSG00000124126 | 36.0935918 | 0.045290794 | 0.1405095 | 0.2674511 | NA | PREX1 | 57580 |
| ENSG00000124198 | 3391.02162 | 0.097062593 | 0.0632361 | 0.0835485 | 0.2700934 | ARFGEF2 | 10564 |
| ENSG00000124207 | 8509.497698 | -0.289346839 | 0.0584022 | 1.25E-07 | 5.77E-06 | CSE1L | 1434 |
| ENSG00000124214 | 5281.175137 | -0.135872709 | 0.0590767 | 0.0109163 | 0.0674672 | STAU1 | 6780 |
| ENSG00000124228 | 4580.204311 | 0.109939509 | 0.0583919 | 0.0369483 | 0.1587321 | DDX27 | 55661 |
| ENSG00000124201 | 3146.465575 | 0.102511157 | 0.0647064 | 0.0728701 | 0.2468371 | ZNFX1 | 57169 |
| ENSG00000177410 | 3550.617022 | 0.60545787 | 0.071225 | 1.37E-18 | 3.55E-16 | ZFAS1 | 441951 |
| ENSG00000209042 | 13.05860205 | 0.003800997 | 0.1343733 | 0.8806667 | NA | SNORD12C | 26765 |
| ENSG00000222365 | 29.41872082 | 0.015252222 | 0.1327301 | 0.6714572 | NA | SNORD12B | 100113393 |
| ENSG00000212304 | 36.77087169 | -0.026555949 | 0.1343307 | 0.4863558 | NA | SNORD12 | 692057 |
| ENSG00000158445 | 637.8961414 | -0.499148844 | 0.1259582 | 5.55E-06 | 0.000151 | KCNB1 | 3745 |
| ENSG00000158470 | 2289.338108 | 0.029387421 | 0.0640514 | 0.5988752 | 0.8061145 | B4GALT5 | 9334 |
| ENSG00000197818 | 377.4754639 | 0.447663563 | 0.1594721 | 0.0003621 | 0.0050299 | SLC9A8 | 23315 |
| ENSG00000158480 | 501.0994818 | 0.127619358 | 0.1159078 | 0.1090143 | 0.3197079 | SPATA2 | 9825 |
| ENSG00000124226 | 2264.904966 | 0.249637751 | 0.0753344 | 0.0001901 | 0.0029414 | RNF114 | 55905 |
| ENSG00000124216 | 83.70008602 | 0.122367854 | 0.1888475 | 0.0748733 | 0.251156 | SNAI1 | 6615 |
| ENSG00000244687 | 129.1551301 | -0.004689711 | 0.118102 | 0.9365276 | 0.9756157 | UBE2V1 | 7335 |
| ENSG00000240849 | 365.7513128 | -0.087999447 | 0.1133309 | 0.2402449 | 0.5055349 | PEDS1 | 387521 |
| ENSG00000231742 | 20.21743435 | -0.033442118 | 0.1387878 | 0.2844669 | NA | LINC01273 | 101927541 |
| ENSG00000172216 | 1782.945911 | 1.735771095 | 0.1708166 | 1.50E-25 | 7.29E-23 | CEBPB | 1051 |
| ENSG00000203999 | 50.47611123 | 0.088925039 | 0.167455 | 0.0845544 | 0.2716391 | LINC01270 | 284751 |
| ENSG00000196396 | 2294.726884 | 0.119569824 | 0.0766877 | 0.0640966 | 0.2277105 | PTPN1 | 5770 |
| ENSG00000232043 | 21.12457941 | 0.021064231 | 0.1351382 | 0.5041941 | NA | NA | NA |
| ENSG00000042062 | 10.40829124 | -0.032427197 | 0.1405859 | 0.141302 | NA | RIPOR3 | 140876 |
| ENSG00000124171 | 1359.829627 | -0.469347932 | 0.1006297 | 2.60E-07 | 1.07E-05 | PARD6B | 84612 |
| ENSG00000124243 | 480.9174372 | -0.11626863 | 0.1119312 | 0.1347409 | 0.3614858 | BCAS4 | 55653 |
| ENSG00000101126 | 3006.78959 | -0.212700935 | 0.0764922 | 0.0013964 | 0.0145245 | ADNP | 23394 |
| ENSG00000259456 | 11.94105557 | 0.003954051 | 0.1345609 | 0.8710732 | NA | ADNP-AS1 | 101927631 |
| ENSG00000000419 | 1507.313299 | -0.094159558 | 0.0762927 | 0.1411381 | 0.3710633 | DPM1 | 8813 |
| ENSG00000124217 | 226.3222606 | 0.070814226 | 0.1230825 | 0.3191106 | 0.5876606 | MOCS3 | 27304 |
| ENSG00000101096 | 74.11772521 | 0.058947814 | 0.1394331 | 0.2844325 | 0.5536531 | NFATC2 | 4773 |
| ENSG00000054793 | 262.0331459 | 0.104205381 | 0.1334618 | 0.1772621 | 0.4267086 | ATP9A | 10079 |
| ENSG00000101115 | 43.63998887 | 0.170446 | 0.320424 | 0.0123291 | 0.0737034 | SALL4 | 57167 |
| ENSG00000020256 | 782.9818847 | -0.035577687 | 0.0854712 | 0.5950755 | 0.8031885 | ZFP64 | 55734 |
| ENSG00000259723 | 15.65794682 | 0.057594542 | 0.1524147 | 0.0354207 | NA | NA | NA |
| ENSG00000171940 | 4967.735505 | 0.100350158 | 0.1181014 | 0.220634 | 0.4802998 | ZNF217 | 7764 |
| ENSG00000019186 | 63.24978117 | 0.153593184 | 0.2515701 | 0.0294624 | 0.1366977 | CYP24A1 | 1591 |
| ENSG00000101132 | 1183.703771 | -0.013316498 | 0.07757 | 0.8341751 | 0.9305701 | PFDN4 | 5203 |
| ENSG00000101134 | 44.50570306 | 0.102940954 | 0.1861192 | 0.0415595 | 0.1716 | DOK5 | 55816 |
| ENSG00000185834 | 44.18467966 | 0.024353397 | 0.1322089 | 0.5629628 | 0.7805304 | NA | NA |
| ENSG00000124098 | 776.3917042 | -0.213652324 | 0.1099532 | 0.01137 | 0.0696084 | FAM210B | 116151 |
| ENSG00000087586 | 3840.703733 | -0.390957994 | 0.073808 | 1.31E-08 | 7.59E-07 | AURKA | 6790 |
| ENSG00000101138 | 1031.535907 | 0.061554631 | 0.0812993 | 0.3505649 | 0.6164306 | CSTF1 | 1477 |
| ENSG00000022277 | 2365.016992 | 0.117991953 | 0.0746458 | 0.0627092 | 0.2242137 | RTF2 | 51507 |
| ENSG00000087510 | 643.8464263 | 0.117628645 | 0.101466 | 0.1233857 | 0.3443144 | TFAP2C | 7022 |
| ENSG00000101146 | 1600.661022 | 0.090479179 | 0.0761679 | 0.1567223 | 0.3957358 | RAE1 | 8480 |
| ENSG00000132819 | 985.1726878 | -0.032093274 | 0.081772 | 0.6246673 | 0.8202204 | RBM38 | 55544 |
| ENSG00000124225 | 235.3369756 | -0.006173695 | 0.109411 | 0.924464 | 0.9709924 | PMEPA1 | 56937 |
| ENSG00000124224 | 232.945576 | -0.087622836 | 0.1274931 | 0.2367067 | 0.5009914 | NA | NA |
| ENSG00000124209 | 1743.818279 | -0.057300353 | 0.070251 | 0.3412868 | 0.6086369 | RAB22A | 57403 |
| ENSG00000124164 | 1966.346183 | 0.104526178 | 0.0710633 | 0.0873949 | 0.2776338 | VAPB | 9217 |
| ENSG00000124222 | 1480.760087 | 0.174420278 | 0.0833099 | 0.0122673 | 0.0734222 | STX16 | 8675 |
| ENSG00000254995 | 12.6411512 | -0.027647458 | 0.1385702 | 0.254186 | NA | STX16-NPEI | 100534593 |
| ENSG00000215440 | 190.1436594 | -0.007181928 | 0.1118382 | 0.9103042 | 0.9647135 | NPEPL1 | 79716 |
| ENSG00000087460 | 16051.36915 | -0.119906838 | 0.0515408 | 0.0122886 | 0.0734985 | GNAS | 2778 |
| ENSG00000101158 | 4095.817396 | -0.024593247 | 0.0571514 | 0.6350763 | 0.8266184 | NELFCD | 51497 |
| ENSG00000101160 | 1865.368639 | -0.010547922 | 0.0684475 | 0.8562255 | 0.9418544 | CTSZ | 1522 |
| ENSG00000124172 | 3397.264208 | -0.208656743 | 0.0781558 | 0.0020284 | 0.0190462 | ATP5F1E | 514 |
| ENSG00000101166 | 3514.397338 | -0.014662853 | 0.0598166 | 0.7860576 | 0.9108874 | PRELID3B | 51012 |
| ENSG00000196074 | 24.86173401 | -0.001600953 | 0.1321865 | 0.9625014 | NA | SYCP2 | 10388 |
| ENSG00000196227 | 890.110413 | -0.216742226 | 0.1020633 | 0.0075346 | 0.0516198 | FAM217B | 63939 |
| ENSG00000132825 | 161.9098972 | -0.042076898 | 0.1213884 | 0.5052876 | 0.7382367 | PPP1R3D | 5509 |
| ENSG00000228340 | 12.4008206 | -0.003904968 | 0.1345205 | 0.8692733 | NA | MIR646HG | 284757 |
| ENSG00000130699 | 1131.824096 | -0.088507704 | 0.0865997 | 0.1976988 | 0.4511271 | TAF4 | 6874 |

| | | | | | | | |
|---|---|---|---|---|---|---|---|
| ENSG00000149657 | 1742.45657 | 0.001024296 | 0.0706293 | 0.9873917 | 0.9948868 | LSM14B | 149986 |
| ENSG00000101182 | 8796.685431 | -0.165446837 | 0.058719 | 0.0017456 | 0.0171346 | PSMA7 | 5688 |
| ENSG00000184402 | 897.7114423 | -0.09322105 | 0.0877443 | 0.1780329 | 0.4271872 | SS18L1 | 26039 |
| ENSG00000101181 | 1748.968715 | 0.293742537 | 0.0815009 | 4.96E-05 | 0.0009763 | MTG2 | 26164 |
| ENSG00000130703 | 1246.458419 | 0.113348223 | 0.0825847 | 0.0941426 | 0.2909429 | OSBPL2 | 9885 |
| ENSG00000130706 | 5873.778846 | -0.03601067 | 0.0555237 | 0.4709457 | 0.7117502 | ADRM1 | 11047 |
| ENSG00000226332 | 80.85511077 | -0.069242092 | 0.1445515 | 0.2246565 | 0.4852777 | NA | NA |
| ENSG00000130702 | 9175.99078 | -0.408547045 | 0.0628298 | 8.46E-12 | 8.82E-10 | LAMA5 | 3911 |
| ENSG00000228812 | 8.521209871 | 0.01215275 | 0.1359617 | 0.5442104 | NA | LAMA5-AS1 | 101928158 |
| ENSG00000171858 | 8894.92446 | -0.059090271 | 0.0973228 | 0.4082259 | 0.6640358 | RPS21 | 6227 |
| ENSG00000149679 | 281.7808507 | 0.062612257 | 0.1155824 | 0.3774263 | 0.6392414 | CABLES2 | 81928 |
| ENSG00000174403 | 16.61727311 | 0.073033265 | 0.1641226 | 0.0130418 | NA | NA | NA |
| ENSG00000167046 | 19.13823323 | 0.045957917 | 0.1447013 | 0.1262964 | NA | NA | NA |
| ENSG00000101187 | 27615.68179 | -0.19534439 | 0.0672098 | 0.0010268 | 0.0113473 | SLCO4A1 | 28231 |
| ENSG00000232803 | 43.09402997 | -0.024403005 | 0.1327115 | 0.5506489 | 0.7717069 | SLCO4A1-A | 100127888 |
| ENSG00000101188 | 32.85262803 | 0.048963035 | 0.1438485 | 0.1897371 | NA | NTSR1 | 4923 |
| ENSG00000101189 | 1810.334514 | 0.074785574 | 0.0742591 | 0.2330098 | 0.4953562 | MRGBP | 55257 |
| ENSG00000060491 | 2961.786368 | -0.069726139 | 0.0629378 | 0.2123224 | 0.4691639 | OGFR | 11054 |
| ENSG00000101190 | 301.8294736 | 0.014235133 | 0.1061069 | 0.8339077 | 0.9305701 | TCFL5 | 10732 |
| ENSG00000101191 | 3638.708092 | -0.058486653 | 0.0638847 | 0.2996549 | 0.5694273 | DIDO1 | 11083 |
| ENSG00000101193 | 898.7915614 | -0.0590514 | 0.0897597 | 0.3904419 | 0.6505443 | GID8 | 54994 |
| ENSG00000149658 | 2616.464502 | 0.116817934 | 0.0661966 | 0.0444464 | 0.1791506 | YTHDF1 | 54915 |
| ENSG00000101199 | 3875.890838 | 0.055551298 | 0.0595327 | 0.2997794 | 0.5695711 | ARFGAP1 | 55738 |
| ENSG00000101210 | 5490.510911 | 0.163836507 | 0.0611369 | 0.002998 | 0.0256558 | EEF1A2 | 1917 |
| ENSG00000125534 | 1274.770054 | 0.139821946 | 0.0861922 | 0.0460668 | 0.1837167 | PPDPF | 79144 |
| ENSG00000125531 | 17.03143127 | 0.00652112 | 0.1338358 | 0.8186259 | NA | FNDC11 | 79025 |
| ENSG00000130589 | 2517.062649 | -0.058167622 | 0.0642243 | 0.3034293 | 0.5727723 | HELZ2 | 85441 |
| ENSG00000101216 | 765.7208012 | 0.377445541 | 0.1135554 | 8.94E-05 | 0.0015889 | GMEB2 | 26205 |
| ENSG00000232442 | 146.7781185 | 0.201657485 | 0.2279651 | 0.0399543 | 0.1675182 | MHENCR | 100505771 |
| ENSG00000197457 | 5962.089678 | -0.168017972 | 0.1242402 | 0.0531163 | 0.2006633 | STMN3 | 50861 |
| ENSG00000258366 | 79.49846133 | -0.138565438 | 0.211125 | 0.0529728 | 0.2002507 | RTEL1 | 51750 |
| ENSG00000026036 | 244.7054193 | -0.451872961 | 0.193826 | 0.0012494 | 0.0133766 | RTEL1-TNFI | 100533107 |
| ENSG00000101246 | 1915.530285 | -0.007002095 | 0.0684332 | 0.9042904 | 0.9622177 | ARFRP1 | 10139 |
| ENSG00000197114 | 453.130991 | -0.085919536 | 0.1082189 | 0.2470724 | 0.5129849 | ZGPAT | 84619 |
| ENSG00000203896 | 114.4037448 | 0.020237574 | 0.1211654 | 0.7297317 | 0.8795785 | LIME1 | 54923 |
| ENSG00000125520 | 2203.72482 | -0.088768641 | 0.0675382 | 0.130974 | 0.3557096 | SLC2A4RG | 56731 |
| ENSG00000130584 | 148.0182267 | -0.001926779 | 0.1181206 | 0.9727008 | 0.9893868 | ZBTB46 | 140685 |
| ENSG00000229299 | 11.03245102 | -0.01776341 | 0.1363163 | 0.4426941 | NA | NA | NA |
| ENSG00000268858 | 52.79317392 | 0.076307815 | 0.1563638 | 0.1282342 | 0.3519509 | LOC112268 | 112268269 |
| ENSG00000101150 | 5773.061608 | 0.211974677 | 0.0589473 | 9.16E-05 | 0.0016239 | TPD52L2 | 7165 |
| ENSG00000101152 | 5424.36469 | 0.007196723 | 0.054337 | 0.8814977 | 0.9522588 | DNAJC5 | 80331 |
| ENSG00000198276 | 837.8899044 | -0.100641887 | 0.0926444 | 0.1588896 | 0.3993092 | UCKL1 | 54963 |
| ENSG00000207554 | 17.55647555 | 0.017135175 | 0.1349508 | 0.5567394 | NA | MIR647 | 693232 |
| ENSG00000196700 | 2306.802812 | -0.118782684 | 0.0733011 | 0.0576288 | 0.2124208 | ZNF512B | 57473 |
| ENSG00000130590 | 161.1701188 | 0.069817747 | 0.1311981 | 0.3016405 | 0.5710237 | SAMD10 | 140700 |
| ENSG00000101161 | 3922.079639 | -0.065076699 | 0.0623687 | 0.2426028 | 0.508472 | PRPF6 | 24148 |
| ENSG00000196421 | 36.88456932 | 0.034398841 | 0.1363969 | 0.3848124 | NA | C20orf204 | 284739 |
| ENSG00000203883 | 17.63171683 | 0.010773089 | 0.1341681 | 0.7053519 | NA | SOX18 | 54345 |
| ENSG00000171703 | 475.031461 | 0.105506182 | 0.1097634 | 0.1734124 | 0.4206475 | TCEA2 | 6919 |
| ENSG00000171700 | 587.5812575 | 0.123822639 | 0.1084877 | 0.1110123 | 0.3231336 | RGS19 | 10287 |
| ENSG00000125510 | 20.07344099 | 0.058377101 | 0.151449 | 0.0696223 | NA | OPRL1 | 4987 |
| ENSG00000196132 | 12.67306909 | 0.004349726 | 0.1346606 | 0.8556723 | NA | MYT1 | 4661 |
| ENSG00000203880 | 1280.642942 | 0.072239343 | 0.0794349 | 0.2650524 | 0.5327957 | PCMTD2 | 55251 |
| ENSG00000225373 | 155.1812733 | -0.024086424 | 0.1181216 | 0.6950703 | 0.8606228 | NA | NA |
| ENSG00000141934 | 1160.369834 | -0.080443 | 0.0825115 | 0.228017 | 0.4890685 | PLPP2 | 8612 |
| ENSG00000105556 | 651.2635205 | 0.281896154 | 0.1213238 | 0.0028365 | 0.0247128 | MIER2 | 54531 |
| ENSG00000105549 | 48.0567932 | 0.062796374 | 0.1475906 | 0.1833985 | 0.4331335 | SPMAP2 | 51298 |
| ENSG00000183186 | 113.3002179 | -0.001437701 | 0.1203699 | 0.9797486 | 0.9924075 | C2CD4C | 126567 |
| ENSG00000099866 | 15.38779064 | 0.052693394 | 0.1491895 | 0.0557473 | NA | MADCAM1 | 8174 |
| ENSG00000141933 | 291.7767109 | 0.187412001 | 0.1593603 | 0.0462213 | 0.1839973 | TPGS1 | 91978 |
| ENSG00000099804 | 2262.134928 | 0.263339563 | 0.0743843 | 7.65E-05 | 0.0014096 | CDC34 | 997 |
| ENSG00000267751 | 122.9649016 | -0.128059678 | 0.1827819 | 0.0856734 | 0.2740282 | BSG-AS1 | 105372233 |
| ENSG00000172270 | 15083.15927 | 0.069939684 | 0.049286 | 0.1273133 | 0.3504943 | BSG | 682 |
| ENSG00000099822 | 244.150711 | -0.048898022 | 0.114785 | 0.4749672 | 0.714776 | HCN2 | 610 |
| ENSG00000099821 | 2563.786493 | 0.085044008 | 0.0665027 | 0.1413564 | 0.3715536 | POLRMT | 5442 |
| ENSG00000070423 | 1770.586432 | 0.005776751 | 0.0688185 | 0.9245969 | 0.9709924 | RNF126 | 55658 |
| ENSG00000070404 | 279.8009417 | 0.899821255 | 0.1745585 | 1.37E-08 | 7.89E-07 | FSTL3 | 10272 |
| ENSG00000099864 | 42.75982078 | 0.089892884 | 0.1736008 | 0.0468237 | 0.1850922 | PALM | 5064 |
| ENSG00000099812 | 36.09855118 | 0.144490698 | 0.2624685 | 0.0103001 | NA | MISP | 126353 |
| ENSG00000272473 | 8.932440453 | -0.003500375 | 0.1351832 | 0.8607466 | NA | NA | NA |

| | | | | | | | |
|---|---|---|---|---|---|---|---|
| ENSG00000267530 | 91.60196481 | -0.016786236 | 0.1235096 | 0.7578367 | 0.8961623 | NA | NA |
| ENSG00000266927 | 14.05383229 | -0.031355529 | 0.1394425 | 0.2253885 | NA | NA | NA |
| ENSG00000011304 | 11090.29648 | -0.137711652 | 0.0523681 | 0.0038107 | 0.0306773 | PTBP1 | 5725 |
| ENSG00000129951 | 32.76002513 | 0.030606238 | 0.1355516 | 0.4314776 | NA | PLPPR3 | 79948 |
| ENSG00000197766 | 171.2234183 | 0.021436389 | 0.1154052 | 0.7367341 | 0.8845504 | CFD | 1675 |
| ENSG00000175221 | 1318.233885 | -0.15430762 | 0.0852612 | 0.0274656 | 0.1295313 | MED16 | 10025 |
| ENSG00000198858 | 817.1906603 | 0.035579176 | 0.0867275 | 0.599305 | 0.80621 | R3HDM4 | 91300 |
| ENSG00000116014 | 11.66769427 | -0.005406316 | 0.1346812 | 0.8172412 | NA | KISS1R | 84634 |
| ENSG00000116017 | 929.4385235 | -0.162459841 | 0.0997919 | 0.035111 | 0.1531493 | ARID3A | 1820 |
| ENSG00000065268 | 1440.996445 | -0.14205147 | 0.0859535 | 0.0425198 | 0.1743325 | WDR18 | 57418 |
| ENSG00000116032 | 23.05144838 | -0.016492169 | 0.1338803 | 0.6112355 | NA | GRIN3B | 116444 |
| ENSG00000182087 | 4729.420301 | 0.17598875 | 0.0597839 | 0.0012034 | 0.0129676 | TMEM259 | 91304 |
| ENSG00000064666 | 6071.493293 | -0.235520074 | 0.0753049 | 0.0003921 | 0.0053503 | CNN2 | 1265 |
| ENSG00000267020 | 9.830197218 | -0.006696377 | 0.1352166 | 0.7495592 | NA | NA | NA |
| ENSG00000064687 | 369.7793738 | 0.03019532 | 0.1051895 | 0.6556954 | 0.8412864 | ABCA7 | 10347 |
| ENSG00000180448 | 886.2364905 | -0.046247663 | 0.0840876 | 0.4889546 | 0.7262901 | ARHGAP45 | 23526 |
| ENSG00000099817 | 5504.875469 | -0.168918117 | 0.0587774 | 0.0015792 | 0.0159435 | POLR2E | 5434 |
| ENSG00000167468 | 7559.576211 | -0.152114648 | 0.0727788 | 0.014407 | 0.0819203 | GPX4 | 2879 |
| ENSG00000064932 | 3848.228145 | -0.098467332 | 0.0601126 | 0.0678644 | 0.2357896 | SBNO2 | 22904 |
| ENSG00000118046 | 2573.293713 | -0.065510618 | 0.0687737 | 0.2699562 | 0.5379322 | STK11 | 6794 |
| ENSG00000267736 | 20.64036398 | -0.053291861 | 0.1486798 | 0.0798051 | NA | NA | NA |
| ENSG00000099625 | 370.8272849 | 0.154743302 | 0.1368737 | 0.0730103 | 0.2469116 | CBARP | 255057 |
| ENSG00000099624 | 1793.335501 | -0.011086351 | 0.0702689 | 0.8528195 | 0.9399684 | ATP5F1D | 513 |
| ENSG00000167470 | 1364.757293 | -0.166816247 | 0.0872591 | 0.0195519 | 0.1019682 | MIDN | 90007 |
| ENSG00000099622 | 1708.522783 | 0.241504311 | 0.0915368 | 0.001678 | 0.016696 | CIRBP | 1153 |
| ENSG00000267493 | 24.05473951 | 0.011177167 | 0.1329248 | 0.7385988 | NA | CIRBP-AS1 | 148046 |
| ENSG00000228300 | 939.5139241 | 0.129258173 | 0.0935533 | 0.0772682 | 0.256412 | FAM174C | 55009 |
| ENSG00000160953 | 1370.014355 | -0.120410197 | 0.0856778 | 0.0816152 | 0.2656303 | PWWP3A | 84939 |
| ENSG00000115286 | 1378.472561 | 0.064439131 | 0.0771387 | 0.3151263 | 0.5839695 | NDUFS7 | 374291 |
| ENSG00000248015 | 48.7424177 | -0.028943338 | 0.1335653 | 0.489094 | 0.7262901 | NA | NA |
| ENSG00000130005 | 436.6681371 | 0.01647007 | 0.0995778 | 0.810854 | 0.9219906 | GAMT | 2593 |
| ENSG00000071626 | 4551.64007 | -0.061641238 | 0.0567456 | 0.2320825 | 0.4945196 | DAZAP1 | 26528 |
| ENSG00000267759 | 28.5976648 | -0.018463484 | 0.1333036 | 0.6029469 | NA | NA | NA |
| ENSG00000115268 | 14375.41378 | -0.077939636 | 0.1016954 | 0.2874941 | 0.5573807 | RPS15 | 6209 |
| ENSG00000268798 | 32.88223657 | -0.067016563 | 0.1540565 | 0.1022324 | NA | NA | NA |
| ENSG00000115266 | 675.5401427 | -0.179638044 | 0.1115269 | 0.0298419 | 0.1378343 | APC2 | 10297 |
| ENSG00000267317 | 72.97270576 | 0.036112834 | 0.131316 | 0.4784008 | 0.7175397 | NA | NA |
| ENSG00000119559 | 1492.078846 | 0.297574992 | 0.0943202 | 0.0002367 | 0.0035311 | C19orf25 | 148223 |
| ENSG00000115257 | 43.11108077 | 0.003253795 | 0.1295138 | 0.9394606 | 0.9764939 | PCSK4 | 54760 |
| ENSG00000115255 | 151.3102997 | 0.10107399 | 0.1488413 | 0.1671899 | 0.4122331 | REEP6 | 92840 |
| ENSG00000185761 | 326.8716404 | 0.011910239 | 0.1016865 | 0.8609525 | 0.9439447 | ADAMTSL5 | 339366 |
| ENSG00000181588 | 1550.862871 | -0.013618802 | 0.0702767 | 0.8205053 | 0.9260506 | MEX3D | 399664 |
| ENSG00000267161 | 86.04663763 | -0.022644192 | 0.1251301 | 0.6746789 | 0.8505206 | NA | NA |
| ENSG00000071655 | 2210.8346 | -0.024326053 | 0.0648788 | 0.6703684 | 0.8489346 | MBD3 | 53615 |
| ENSG00000266926 | 10.70195774 | -0.023497208 | 0.1376673 | 0.304192 | NA | NA | NA |
| ENSG00000127540 | 972.1028235 | 0.015113314 | 0.0818409 | 0.8185787 | 0.9254567 | UQCR11 | 10975 |
| ENSG00000071564 | 4489.325719 | -0.166420656 | 0.0641483 | 0.0036556 | 0.0297778 | TCF3 | 6929 |
| ENSG00000130270 | 359.5048847 | -0.10553608 | 0.1214394 | 0.1742501 | 0.4220227 | ATP8B3 | 148229 |
| ENSG00000079313 | 1824.743149 | 0.05049729 | 0.0716032 | 0.4092797 | 0.6643076 | REXO1 | 57455 |
| ENSG00000267125 | 17.33260358 | -0.017905518 | 0.1351541 | 0.531747 | NA | NA | NA |
| ENSG00000129911 | 2412.440651 | 0.159001922 | 0.0700826 | 0.0094667 | 0.0615017 | KLF16 | 83855 |
| ENSG00000261526 | 108.9029527 | 0.009774049 | 0.1207753 | 0.8650852 | 0.9457239 | NA | NA |
| ENSG00000129968 | 3419.419127 | 0.086428275 | 0.0618372 | 0.1144017 | 0.3289015 | ABHD17A | 81926 |
| ENSG00000227500 | 1637.021513 | 0.064878601 | 0.0738584 | 0.2985813 | 0.5686822 | SCAMP4 | 113178 |
| ENSG00000213638 | 236.8904388 | 0.019118185 | 0.1088917 | 0.7753457 | 0.9053192 | ADAT3 | 113179 |
| ENSG00000133275 | 3737.085669 | -0.093492974 | 0.0602647 | 0.0833294 | 0.2698513 | CSNK1G2 | 1455 |
| ENSG00000133243 | 740.0022532 | 0.045246434 | 0.0866335 | 0.5040971 | 0.7381071 | BTBD2 | 55643 |
| ENSG00000267283 | 28.87285811 | -0.014568722 | 0.1325556 | 0.6833892 | NA | NA | NA |
| ENSG00000099875 | 2697.424692 | -0.084505809 | 0.0709309 | 0.1650566 | 0.4090699 | MKNK2 | 2872 |
| ENSG00000172081 | 912.0303198 | -0.028345414 | 0.0856123 | 0.6710742 | 0.8493681 | MOB3A | 126308 |
| ENSG00000099840 | 64.23028257 | -0.075214382 | 0.1516429 | 0.1670115 | 0.4122331 | IZUMO4 | 113177 |
| ENSG00000065000 | 6070.420408 | -0.046104917 | 0.0540412 | 0.3520927 | 0.6179079 | AP3D1 | 8943 |
| ENSG00000104885 | 1403.87236 | 0.066677189 | 0.0743664 | 0.2879087 | 0.5577216 | DOT1L | 84444 |
| ENSG00000104886 | 1834.649909 | 0.031684252 | 0.0737731 | 0.6110237 | 0.8135902 | PLEKHJ1 | 55111 |
| ENSG00000104897 | 1528.79428 | 0.149070915 | 0.0804297 | 0.0267523 | 0.1271074 | SF3A2 | 8175 |
| ENSG00000104899 | 189.4778478 | -0.143428651 | 0.1759621 | 0.0839676 | 0.2707908 | AMH | 268 |
| ENSG00000167476 | 26.24926274 | -0.043484254 | 0.1418534 | 0.2141146 | NA | JSRP1 | 126306 |
| ENSG00000104904 | 7687.775498 | -0.036444292 | 0.0545965 | 0.4121013 | 0.6662022 | OAZ1 | 4946 |
| ENSG00000130332 | 1777.395473 | -0.107960131 | 0.0735223 | 0.0843215 | 0.271264 | LSM7 | 51690 |
| ENSG00000005206 | 1045.8533 | 0.336664055 | 0.0966708 | 6.14E-05 | 0.0011757 | SPPL2B | 56928 |

| | | | | | | | | |
|---|---|---|---|---|---|---|---|---|
| ENSG00000099800 | 1453.064288 | -0.149278055 | 0.0832921 | 0.0301594 | 0.1388732 | TIMM13 | | 26517 |
| ENSG00000176619 | 11509.80687 | -0.210881301 | 0.0595553 | 0.0001132 | 0.0019268 | LMNB2 | | 84823 |
| ENSG00000267201 | 17.86268499 | -0.009010265 | 0.1339952 | 0.7478702 | NA | LINC01775 | | 101928602 |
| ENSG00000099860 | 693.8032319 | 0.645538114 | 0.1236581 | 1.16E-08 | 6.87E-07 | GADD45B | | 4616 |
| ENSG00000261342 | 17.77539676 | 0.011983389 | 0.1342492 | 0.6777403 | NA | NA | NA | |
| ENSG00000141873 | 639.5813644 | -0.058668516 | 0.0919115 | 0.3986462 | 0.6577099 | SLC39A3 | | 29985 |
| ENSG00000104969 | 2967.095118 | 0.044520109 | 0.0612259 | 0.4148842 | 0.6691885 | SGTA | | 6449 |
| ENSG00000172009 | 2671.16358 | 0.107007822 | 0.0666568 | 0.0666318 | 0.2333811 | THOP1 | | 7064 |
| ENSG00000172006 | 56.73676111 | 0.169060196 | 0.2928018 | 0.0205479 | 0.1055962 | ZNF554 | | 115196 |
| ENSG00000186300 | 171.8417057 | 0.071661368 | 0.1294608 | 0.2993386 | 0.5693326 | ZNF555 | | 148254 |
| ENSG00000171970 | 167.1369405 | 0.785672544 | 0.222117 | 2.04E-05 | 0.000463 | ZNF57 | | 126295 |
| ENSG00000253392 | 10.73498843 | 0.011183609 | 0.135334 | 0.6281713 | NA | NA | NA | |
| ENSG00000175691 | 139.7095415 | 0.04675401 | 0.1251626 | 0.4556159 | 0.7007253 | ZNF77 | | 58492 |
| ENSG00000104953 | 29.24585676 | 0.04009981 | 0.139572 | 0.2817218 | NA | TLE6 | | 79816 |
| ENSG00000065717 | 101.0902226 | 0.085141902 | 0.1500643 | 0.1829054 | 0.4326625 | TLE2 | | 7089 |
| ENSG00000267469 | 85.30552197 | -0.013484049 | 0.1240052 | 0.7990898 | 0.9162772 | NA | NA | |
| ENSG00000104964 | 6088.266494 | -0.146542296 | 0.0565626 | 0.0045724 | 0.0354411 | TLE5 | | 166 |
| ENSG00000088256 | 2662.85629 | 0.019357766 | 0.068217 | 0.743931 | 0.8886411 | GNA11 | | 2767 |
| ENSG00000267139 | 15.63105744 | 0.001711221 | 0.1337848 | 0.9523855 | NA | NA | NA | |
| ENSG00000125912 | 3130.696407 | 0.026203297 | 0.0616287 | 0.6298663 | 0.8238124 | NCLN | | 56926 |
| ENSG00000141905 | 2192.210973 | -0.134504762 | 0.0715335 | 0.0293391 | 0.1363205 | NFIC | | 4782 |
| ENSG00000129932 | 1088.585474 | 0.236626247 | 0.0957536 | 0.0027431 | 0.0240373 | DOHH | | 83475 |
| ENSG00000105325 | 1488.648274 | -0.127920703 | 0.0789081 | 0.0525517 | 0.1990455 | FZR1 | | 51343 |
| ENSG00000161091 | 1998.101832 | -0.003524588 | 0.0671742 | 0.9521457 | 0.9806656 | MFSD12 | | 126321 |
| ENSG00000267436 | 14.34471191 | 0.016568845 | 0.1354981 | 0.529036 | NA | NA | NA | |
| ENSG00000064961 | 1980.004482 | -0.319771266 | 0.0853012 | 2.43E-05 | 0.0005331 | HMG20B | | 10362 |
| ENSG00000006638 | 63.60284854 | -0.010985592 | 0.1267463 | 0.8192431 | 0.925668 | TBXA2R | | 6915 |
| ENSG00000226800 | 9.212472293 | 0.01979233 | 0.137145 | 0.3457138 | NA | CACTIN-AS1 | | 404665 |
| ENSG00000105298 | 1215.937679 | 0.07800716 | 0.0784733 | 0.2310068 | 0.4928567 | CACTIN | | 58509 |
| ENSG00000186111 | 969.2833067 | -0.110032285 | 0.088764 | 0.117211 | 0.3339557 | PIP5K1C | | 23396 |
| ENSG00000011132 | 495.6854509 | 0.199528274 | 0.1290203 | 0.026574 | 0.1266464 | APBA3 | | 9546 |
| ENSG00000183617 | 368.4207163 | -0.189865394 | 0.1473735 | 0.0405457 | 0.169099 | MRPL54 | | 116541 |
| ENSG00000167657 | 2557.445232 | 0.181860317 | 0.0716259 | 0.0037501 | 0.030371 | DAPK3 | | 1613 |
| ENSG00000167658 | 44820.50582 | -0.04759832 | 0.0459321 | 0.2835066 | 0.5529538 | EEF2 | | 1938 |
| ENSG00000105229 | 836.3892883 | 0.068718902 | 0.0941046 | 0.3315487 | 0.5992295 | PIAS4 | | 51588 |
| ENSG00000178951 | 1261.01489 | -0.000735103 | 0.0757392 | 0.9898914 | 0.9955163 | ZBTB7A | | 51341 |
| ENSG00000268670 | 10.02414001 | -0.002497738 | 0.1348133 | 0.9083669 | NA | NA | NA | |
| ENSG00000126934 | 2591.848326 | -0.007126346 | 0.0626783 | 0.8988506 | 0.9600836 | MAP2K2 | | 5605 |
| ENSG00000077463 | 371.2156617 | 0.08757721 | 0.1141683 | 0.2440372 | 0.5098718 | SIRT6 | | 51548 |
| ENSG00000105246 | 351.843951 | 0.12816171 | 0.134481 | 0.1185757 | 0.3359216 | EBI3 | | 10148 |
| ENSG00000105248 | 433.5351899 | 0.00604932 | 0.0985117 | 0.9306198 | 0.972984 | YJU2 | | 55702 |
| ENSG00000105255 | 770.5310173 | -0.001286496 | 0.0836765 | 0.9836075 | 0.9938171 | FSD1 | | 79187 |
| ENSG00000178078 | 231.4518912 | 0.014570725 | 0.1107632 | 0.8251108 | 0.9280808 | STAP2 | | 55620 |
| ENSG00000008382 | 260.1709371 | -0.325195545 | 0.18614 | 0.006879 | 0.0480881 | MPND | | 84954 |
| ENSG00000141985 | 2233.011197 | -0.159507685 | 0.072132 | 0.0108022 | 0.0669394 | SH3GL1 | | 6455 |
| ENSG00000267980 | 27.75790854 | -0.011371113 | 0.1323648 | 0.7443058 | NA | NA | NA | |
| ENSG00000167670 | 2568.081954 | 0.003229868 | 0.0619597 | 0.9519954 | 0.9806656 | CHAF1A | | 10036 |
| ENSG00000267255 | 9.086500651 | 0.015841509 | 0.136327 | 0.4572253 | NA | NA | NA | |
| ENSG00000167671 | 1030.238572 | -0.101120375 | 0.0864513 | 0.1425099 | 0.373493 | UBXN6 | | 80700 |
| ENSG00000267030 | 16.02631675 | 0.004406454 | 0.1338576 | 0.8764835 | NA | NA | NA | |
| ENSG00000267769 | 9.227116159 | 0.00697545 | 0.1352094 | 0.7454841 | NA | NA | NA | |
| ENSG00000167674 | 1885.224389 | -0.204517528 | 0.0814456 | 0.0032534 | 0.0272224 | HDGFL2 | | 84717 |
| ENSG00000167680 | 24.61065923 | -0.002201441 | 0.1323719 | 0.9438165 | NA | SEMA6B | | 10501 |
| ENSG00000185361 | 560.2331539 | -0.476106046 | 0.1254705 | 1.15E-05 | 0.0002846 | TNFAIP8L1 | | 126282 |
| ENSG00000074842 | 2093.295186 | -0.099755039 | 0.0693168 | 0.095984 | 0.2944213 | MYDGF | | 56005 |
| ENSG00000268565 | 12.32817624 | 0.005039546 | 0.1344984 | 0.8396424 | NA | NA | NA | |
| ENSG00000142002 | 3130.300928 | -0.004767582 | 0.0604732 | 0.9302801 | 0.972984 | DPP9 | | 91039 |
| ENSG00000205790 | 8.812270842 | -0.001811822 | 0.1350439 | 0.9284605 | NA | DPP9-AS1 | | 100131094 |
| ENSG00000141965 | 941.526048 | 0.072153609 | 0.0859826 | 0.2936651 | 0.5635456 | FEM1A | | 55527 |
| ENSG00000269604 | 18.07621378 | 0.000926141 | 0.1336935 | 0.9756279 | NA | NA | NA | |
| ENSG00000127666 | 652.754114 | 0.274174361 | 0.1171438 | 0.0028666 | 0.0248832 | TICAM1 | | 148022 |
| ENSG00000267484 | 17.67082589 | -0.032849828 | 0.1391064 | 0.2623663 | NA | NA | NA | |
| ENSG00000105355 | 4568.552913 | -0.102215109 | 0.0601201 | 0.0579632 | 0.213317 | PLIN3 | | 10226 |
| ENSG00000034063 | 6685.334453 | 0.081182102 | 0.0542628 | 0.1020066 | 0.3058365 | NA | NA | |
| ENSG00000127663 | 468.3331069 | -0.157614877 | 0.1272713 | 0.0636208 | 0.2267788 | KDM4B | | 23030 |
| ENSG00000105426 | 1660.172049 | -0.143067306 | 0.0781863 | 0.029742 | 0.1375363 | PTPRS | | 5802 |
| ENSG00000223573 | 9.924197853 | 2.240716285 | 1.281733 | 0.0028425 | NA | TINCR | | 257000 |
| ENSG00000130254 | 2779.951137 | 0.08537772 | 0.0668709 | 0.144083 | 0.3763992 | SAFB2 | | 9667 |
| ENSG00000160633 | 4059.703648 | -0.024423832 | 0.0631578 | 0.6599415 | 0.8426737 | SAFB | | 6294 |
| ENSG00000130255 | 5211.375838 | -0.087084183 | 0.0670134 | 0.1363833 | 0.363637 | RPL36 | | 25873 |

| | | | | | | | |
|---|---|---|---|---|---|---|---|
| ENSG00000174917 | 689.8692595 | -0.029833309 | 0.0866335 | 0.6623379 | 0.8437964 | MICOS13 | 125988 |
| ENSG00000167733 | 44.49284071 | 0.009034696 | 0.1295229 | 0.8328722 | 0.9305386 | HSD11B1L | 374875 |
| ENSG00000196365 | 1557.948796 | -0.004121242 | 0.071255 | 0.9451632 | 0.9784014 | LONP1 | 9361 |
| ENSG00000212123 | 80.8627671 | 0.025718994 | 0.1281639 | 0.6128816 | 0.8148077 | PRR22 | 163154 |
| ENSG00000141994 | 678.784182 | -0.009889351 | 0.0880825 | 0.8824309 | 0.9525622 | DUS3L | 56931 |
| ENSG00000174886 | 411.4602901 | -0.062945484 | 0.1067531 | 0.3680505 | 0.6308952 | NDUFA11 | 126328 |
| ENSG00000187650 | 16.01140396 | -0.010942146 | 0.1343515 | 0.6897786 | NA | VMAC | 400673 |
| ENSG00000105519 | 28.29649249 | 0.005133354 | 0.1317262 | 0.8870865 | NA | CAPS | 828 |
| ENSG00000031823 | 1526.433634 | -0.123342256 | 0.0777695 | 0.0586784 | 0.2149333 | RANBP3 | 8498 |
| ENSG00000266983 | 14.69419161 | 0.050352991 | 0.1479456 | 0.06136 | NA | RANBP3-DT | 100128568 |
| ENSG00000087903 | 306.3456687 | 0.060601293 | 0.1110646 | 0.3974116 | 0.6567942 | RFX2 | 5990 |
| ENSG00000267427 | 1192.589795 | -0.013583955 | 0.0770801 | 0.8302924 | 0.9302425 | NA | NA |
| ENSG00000130382 | 1101.748045 | 0.007582479 | 0.076824 | 0.9058012 | 0.9628103 | MLLT1 | 4298 |
| ENSG00000125656 | 1399.197653 | -0.05260575 | 0.0743587 | 0.4003297 | 0.6583091 | CLPP | 8192 |
| ENSG00000125652 | 404.5834587 | -0.378179597 | 0.1477006 | 0.0009108 | 0.0102698 | ALKBH7 | 84266 |
| ENSG00000125650 | 83.98823064 | 0.064788459 | 0.1427952 | 0.2436491 | 0.5095683 | PSPN | 5623 |
| ENSG00000125651 | 3255.564057 | 0.039534008 | 0.0619509 | 0.4761775 | 0.7156114 | GTF2F1 | 2962 |
| ENSG00000088247 | 8052.012415 | -0.152694138 | 0.0542791 | 0.0022673 | 0.0208214 | KHSRP | 8570 |
| ENSG00000125648 | 1221.309734 | -0.137860819 | 0.0840297 | 0.0452539 | 0.1812792 | SLC25A23 | 79085 |
| ENSG00000104833 | 73.85339692 | 0.158606833 | 0.2455684 | 0.0369176 | 0.1587321 | TUBB4A | 10382 |
| ENSG00000125657 | 294.1383592 | 0.039160936 | 0.1122177 | 0.5688655 | 0.7847255 | TNFSF9 | 8744 |
| ENSG00000125726 | 1505.248306 | -0.22490955 | 0.084324 | 0.001769 | 0.017289 | CD70 | 970 |
| ENSG00000125730 | 619.4434353 | -0.412635638 | 0.1240876 | 7.71E-05 | 0.0014108 | C3 | 718 |
| ENSG00000125734 | 636.4880937 | 0.02432411 | 0.0881071 | 0.7201934 | 0.8733776 | GPR108 | 56927 |
| ENSG00000125733 | 2680.44745 | -0.22206727 | 0.074103 | 0.0006741 | 0.0081512 | TRIP10 | 9322 |
| ENSG00000125731 | 257.6220895 | -0.111298645 | 0.1372377 | 0.1556749 | 0.3943696 | SH2D3A | 10045 |
| ENSG00000174837 | 16.19574001 | 0.004084456 | 0.1342683 | 0.8749049 | NA | ADGRE1 | 2015 |
| ENSG00000130544 | 192.7725447 | 0.09966133 | 0.1418217 | 0.1800793 | 0.42955 | ZNF557 | 79230 |
| ENSG00000171105 | 363.5187869 | 0.041893842 | 0.104622 | 0.5489504 | 0.7704064 | INSR | 3643 |
| ENSG00000104880 | 692.7116955 | 0.232478357 | 0.1159304 | 0.0084771 | 0.056519 | ARHGEF18 | 23370 |
| ENSG00000104883 | 26.27405981 | 0.00813939 | 0.1322831 | 0.8150156 | NA | PEX11G | 92960 |
| ENSG00000198816 | 165.3065066 | 0.015892811 | 0.1165199 | 0.7981464 | 0.9162772 | ZNF358 | 140467 |
| ENSG00000090674 | 741.004631 | 0.096086391 | 0.0976775 | 0.1880655 | 0.4389252 | MCOLN1 | 57192 |
| ENSG00000032444 | 3652.044507 | -0.335531327 | 0.1485617 | 0.0023418 | 0.0213484 | PNPLA6 | 10908 |
| ENSG00000076826 | 341.7759028 | 0.033628945 | 0.1032831 | 0.6278023 | 0.8225865 | CAMSAP3 | 57662 |
| ENSG00000076924 | 1393.984484 | 0.031227944 | 0.0725608 | 0.6129514 | 0.8148077 | XAB2 | 56949 |
| ENSG00000229833 | 37.68832545 | -0.033039645 | 0.1353027 | 0.4180141 | NA | PET100 | 100131801 |
| ENSG00000076944 | 555.7428021 | -0.091088299 | 0.104574 | 0.2194126 | 0.4793377 | STXBP2 | 6813 |
| ENSG00000181029 | 12.63144948 | 0.003516161 | 0.1343977 | 0.8897032 | NA | TRAPPC5 | 126003 |
| ENSG00000142459 | 263.0822968 | 0.579543001 | 0.2029672 | 0.0002344 | 0.0035054 | EVI5L | 115704 |
| ENSG00000171017 | 404.8907743 | 0.04361565 | 0.1013692 | 0.5329506 | 0.7581776 | LRRC8E | 80131 |
| ENSG00000214248 | 13.83247948 | -0.001378309 | 0.1341422 | 0.9548565 | NA | NA | NA |
| ENSG00000076984 | 1521.049915 | -0.058839086 | 0.0732339 | 0.3427687 | 0.6092767 | MAP2K7 | 5609 |
| ENSG00000260001 | 37.02607058 | 0.084027228 | 0.1669141 | 0.065114 | NA | TGFBR3L | 100507588 |
| ENSG00000104976 | 415.663753 | -0.074873117 | 0.1064998 | 0.3069654 | 0.5762568 | SNAPC2 | 6618 |
| ENSG00000178531 | 361.7250365 | 0.081272789 | 0.1143974 | 0.2732426 | 0.5414329 | CTXN1 | 404217 |
| ENSG00000104980 | 1839.4324 | 0.055281886 | 0.0696035 | 0.3577115 | 0.6209582 | TIMM44 | 10469 |
| ENSG00000066044 | 2458.101187 | -0.076752285 | 0.0654778 | 0.1811405 | 0.4306741 | ELAVL1 | 1994 |
| ENSG00000267939 | 8.598291773 | 0.021454157 | 0.1376686 | 0.2850862 | NA | NA | NA |
| ENSG00000090661 | 153.8812451 | 0.038402321 | 0.1208357 | 0.5439974 | 0.7661909 | CERS4 | 79603 |
| ENSG00000167775 | 2150.274741 | 0.047075648 | 0.0671468 | 0.4268072 | 0.6787658 | CD320 | 51293 |
| ENSG00000267855 | 12.35452777 | 0.004128936 | 0.1344746 | 0.8681152 | NA | NDUFA7 | 4701 |
| ENSG00000233927 | 6586.620456 | 0.001989337 | 0.0609141 | 0.981085 | 0.9930723 | RPS28 | 6234 |
| ENSG00000167772 | 217.9238713 | 1.295050877 | 0.1938837 | 1.30E-12 | 1.65E-10 | ANGPTL4 | 51129 |
| ENSG00000269386 | 18.65234372 | 0.008343511 | 0.1336129 | 0.7800375 | NA | RAB11B-AS | 100507567 |
| ENSG00000185236 | 1471.962849 | -0.133503369 | 0.0806609 | 0.046497 | 0.1842999 | RAB11B | 9230 |
| ENSG00000099785 | 254.2487637 | -0.066464786 | 0.1192874 | 0.3490466 | 0.6148358 | MARCHF2 | 51257 |
| ENSG00000099783 | 8713.512103 | -0.407961274 | 0.0603183 | 1.55E-12 | 1.93E-10 | HNRNPM | 4670 |
| ENSG00000133250 | 137.54362 | 0.152595776 | 0.1967404 | 0.0665428 | 0.2333139 | ZNF414 | 84330 |
| ENSG00000142303 | 13.77136885 | -0.028882751 | 0.1387085 | 0.2558861 | NA | ADAMTS10 | 81794 |
| ENSG00000167785 | 397.7140957 | -0.025452787 | 0.0984228 | 0.7109562 | 0.869096 | ZNF558 | 148156 |
| ENSG00000130803 | 1289.756022 | 0.521083369 | 0.0993021 | 1.20E-08 | 7.09E-07 | ZNF317 | 57693 |
| ENSG00000196110 | 164.6806232 | 0.072985312 | 0.1311433 | 0.2892431 | 0.5592864 | ZNF699 | 374879 |
| ENSG00000188321 | 130.3442785 | 0.024015026 | 0.1210728 | 0.6861765 | 0.8567854 | ZNF559 | 84527 |
| ENSG00000174652 | 1092.595669 | -0.036074293 | 0.077308 | 0.5716504 | 0.7864627 | ZNF266 | 10781 |
| ENSG00000130818 | 310.0539796 | 0.141668337 | 0.1386626 | 0.0938367 | 0.2905903 | ZNF426 | 79088 |
| ENSG00000197961 | 1268.197828 | -0.072633437 | 0.0823722 | 0.265129 | 0.5327957 | ZNF121 | 7675 |
| ENSG00000171469 | 696.1587737 | 0.210594816 | 0.1105532 | 0.0126749 | 0.0752314 | ZNF561 | 93134 |
| ENSG00000267106 | 10.83190802 | 0.011191545 | 0.1357522 | 0.5863791 | NA | ZNF561-AS1 | 284385 |
| ENSG00000171466 | 895.460727 | 0.050243922 | 0.082471 | 0.4468758 | 0.6944817 | ZNF562 | 54811 |

| | | | | | | | |
|---|---|---|---|---|---|---|---|
| ENSG00000196605 | 67.91111318 | 0.012737512 | 0.1262122 | 0.7998643 | 0.9169004 | ZNF846 | 162993 |
| ENSG00000127452 | 458.9791839 | -0.017275345 | 0.0960242 | 0.8001502 | 0.9169162 | FBXL12 | 54850 |
| ENSG00000198258 | 1941.173912 | -0.206247257 | 0.0759203 | 0.0018051 | 0.0175424 | UBL5 | 59286 |
| ENSG00000127445 | 2328.398842 | -0.075535992 | 0.0707684 | 0.2151825 | 0.4733322 | PIN1 | 5300 |
| ENSG00000130813 | 564.4305553 | 0.01131511 | 0.0954711 | 0.8805335 | 0.9518649 | SHFL | 55337 |
| ENSG00000130812 | 21.08911712 | 0.012969223 | 0.133755 | 0.6785323 | NA | ANGPTL6 | 83854 |
| ENSG00000130810 | 251.390473 | 0.04975713 | 0.1130075 | 0.4737966 | 0.7139512 | PPAN | 56342 |
| ENSG00000244165 | 368.3600777 | 0.126626711 | 0.1278785 | 0.1197277 | 0.3378071 | P2RY11 | 5032 |
| ENSG00000130811 | 2937.435766 | -0.116035202 | 0.0704207 | 0.0562659 | 0.2088473 | EIF3G | 8666 |
| ENSG00000130816 | 9264.930042 | -0.015923631 | 0.0516563 | 0.7387565 | 0.8857662 | DNMT1 | 1786 |
| ENSG00000175898 | 36.09962567 | 0.074477742 | 0.1611036 | 0.0606559 | NA | NA | NA |
| ENSG00000267534 | 15.3105348 | -0.00740314 | 0.1341367 | 0.7822124 | NA | S1PR2 | 9294 |
| ENSG00000105364 | 1961.39901 | -0.009846372 | 0.070381 | 0.8705746 | 0.9478556 | MRPL4 | 51073 |
| ENSG00000266978 | 22.20466628 | -0.00957745 | 0.1333324 | 0.7567082 | NA | NA | NA |
| ENSG00000090339 | 8784.17883 | 0.035234323 | 0.0508429 | 0.4543063 | 0.6994533 | ICAM1 | 3383 |
| ENSG00000267607 | 74.92007114 | -0.007306358 | 0.1267284 | 0.8772611 | 0.950252 | NA | NA |
| ENSG00000105371 | 14.547124 | -0.02512297 | 0.1374877 | 0.3304463 | NA | ICAM4 | 3386 |
| ENSG00000105376 | 125.1713457 | 0.021300788 | 0.1203859 | 0.7200759 | 0.8733776 | ICAM5 | 7087 |
| ENSG00000220201 | 25.94849142 | -0.018205666 | 0.1338195 | 0.5891707 | NA | ZGLP1 | 100125288 |
| ENSG00000161847 | 1185.493482 | -0.05045231 | 0.0765121 | 0.4271724 | 0.6791209 | RAVER1 | 125950 |
| ENSG00000076662 | 219.9625187 | -0.017458633 | 0.110029 | 0.7891121 | 0.9116361 | ICAM3 | 3385 |
| ENSG00000105397 | 2981.284936 | 0.00135298 | 0.0603597 | 0.9784297 | 0.9920178 | TYK2 | 7297 |
| ENSG00000105401 | 5767.802082 | 0.060586538 | 0.0572231 | 0.2427773 | 0.5085703 | CDC37 | 11140 |
| ENSG00000065989 | 27.60143398 | 0.035385184 | 0.1380236 | 0.3273219 | NA | PDE4A | 5141 |
| ENSG00000079999 | 2692.915333 | -0.065992348 | 0.0645595 | 0.2454728 | 0.5110917 | KEAP1 | 9817 |
| ENSG00000267197 | 13.33601974 | -0.012634199 | 0.1357199 | 0.5591743 | NA | NA | NA |
| ENSG00000180739 | 98.9988502 | 0.028760159 | 0.1268326 | 0.5953409 | 0.803301 | S1PR5 | 53637 |
| ENSG00000130734 | 408.9282413 | 0.027079738 | 0.0984528 | 0.6946213 | 0.8605415 | ATG4D | 84971 |
| ENSG00000129347 | 1674.81423 | -0.092957233 | 0.0740072 | 0.1379349 | 0.3663342 | KRI1 | 65095 |
| ENSG00000129355 | 247.846517 | 0.275283273 | 0.2291971 | 0.0188742 | 0.0997599 | CDKN2D | 1032 |
| ENSG00000129354 | 637.8118408 | -0.089270791 | 0.0975969 | 0.2184977 | 0.4783119 | AP1M2 | 10053 |
| ENSG00000129353 | 661.0062512 | -0.08055969 | 0.0947677 | 0.258588 | 0.5253712 | SLC44A2 | 57153 |
| ENSG00000267100 | 361.1503006 | 0.005396394 | 0.1021811 | 0.9384909 | 0.9761997 | ILF3-DT | 147727 |
| ENSG00000129351 | 12240.41653 | -0.223299168 | 0.0528433 | 6.33E-06 | 0.0001701 | ILF3 | 3609 |
| ENSG00000213339 | 949.1028384 | -0.011288761 | 0.0787817 | 0.8600309 | 0.9435431 | QTRT1 | 81890 |
| ENSG00000079805 | 3925.627485 | -0.127569068 | 0.0615306 | 0.0206255 | 0.1059482 | DNM2 | 1785 |
| ENSG00000099203 | 649.2888838 | -0.062428917 | 0.0947364 | 0.3750562 | 0.6367779 | TMED1 | 11018 |
| ENSG00000142453 | 1432.020579 | -0.080228824 | 0.0779064 | 0.2138328 | 0.4714574 | CARM1 | 10498 |
| ENSG00000130733 | 698.0916509 | -0.004803102 | 0.0854136 | 0.9417062 | 0.977176 | YIPF2 | 78992 |
| ENSG00000142444 | 404.8494892 | -0.008443649 | 0.0973933 | 0.9006294 | 0.9610169 | TIMM29 | 90580 |
| ENSG00000127616 | 462.7894281 | -0.029361087 | 0.0971233 | 0.6699144 | 0.8486358 | SMARCA4 | 6597 |
| ENSG00000130164 | 1017.839188 | -0.577727109 | 0.1148241 | 3.35E-08 | 1.77E-06 | LDLR | 3949 |
| ENSG00000161888 | 286.9201102 | -0.148009033 | 0.1463385 | 0.0849485 | 0.2726802 | SPC24 | 147841 |
| ENSG00000105514 | 291.63417 | -0.391377024 | 0.1922834 | 0.0029444 | 0.0253775 | RAB3D | 9545 |
| ENSG00000105518 | 203.9187638 | 0.0933144 | 0.135732 | 0.2057694 | 0.4608115 | TMEM205 | 374882 |
| ENSG00000183401 | 34.91987683 | 0.035563875 | 0.1367915 | 0.3701289 | NA | CCDC159 | 126075 |
| ENSG00000105520 | 273.5110562 | 0.057055591 | 0.1127471 | 0.4179893 | 0.6710348 | PLPPR2 | 64748 |
| ENSG00000173928 | 17.39971484 | -0.004996538 | 0.133615 | 0.8658059 | NA | SWSAP1 | 126074 |
| ENSG00000187266 | 50.82573781 | -0.023512236 | 0.1306046 | 0.6005656 | 0.8069949 | EPOR | 2057 |
| ENSG00000205517 | 84.27247683 | 0.000266245 | 0.1248678 | 0.9975016 | 0.998947 | RGL3 | 57139 |
| ENSG00000198003 | 9.068887345 | -0.002486166 | 0.1350058 | 0.903937 | NA | ODAD3 | 115948 |
| ENSG00000130175 | 3100.80704 | -0.0971783 | 0.0677097 | 0.0992646 | 0.3005468 | PRKCSH | 5589 |
| ENSG00000161914 | 41.4266261 | -0.000484311 | 0.1296887 | 0.9886685 | 0.9955163 | ZNF653 | 115950 |
| ENSG00000130159 | 233.6881239 | -0.00798175 | 0.1091319 | 0.9031298 | 0.9618565 | ECSIT | 51295 |
| ENSG00000130165 | 361.4579203 | -0.05993734 | 0.1082364 | 0.4008967 | 0.6587248 | ELOF1 | 84337 |
| ENSG00000198551 | 56.77611448 | 0.070753107 | 0.1505743 | 0.1719572 | 0.4182906 | ZNF627 | 199692 |
| ENSG00000267500 | 9.513140002 | -0.005294172 | 0.1350722 | 0.802722 | NA | NA | NA |
| ENSG00000214223 | 200.837782 | -0.354480292 | 0.2296168 | 0.0079713 | 0.0540412 | NA | NA |
| ENSG00000197933 | 75.05799351 | -0.021154368 | 0.1273893 | 0.6719218 | 0.8499086 | ZNF823 | 55552 |
| ENSG00000197044 | 48.91439722 | 0.018699215 | 0.1307384 | 0.6628163 | 0.8438537 | ZNF441 | 126068 |
| ENSG00000171295 | 136.847302 | -0.016225986 | 0.118556 | 0.786119 | 0.9108874 | ZNF440 | 126070 |
| ENSG00000198429 | 49.55961111 | 0.027139356 | 0.1319907 | 0.5413495 | 0.7645391 | ZNF69 | 7620 |
| ENSG00000196757 | 115.613418 | 0.017951079 | 0.1209463 | 0.7585164 | 0.8963508 | ZNF700 | 90592 |
| ENSG00000219665 | 38.64253687 | 0.038419856 | 0.1373204 | 0.3493105 | NA | ZNF433-AS1 | 101928464 |
| ENSG00000197647 | 17.9067362 | -0.028179488 | 0.1382154 | 0.2906046 | NA | ZNF433 | 163059 |
| ENSG00000257446 | 19.08612113 | 0.00797888 | 0.1337531 | 0.7875924 | NA | ZNF878 | 729747 |
| ENSG00000223547 | 97.41128308 | 0.107099248 | 0.1700592 | 0.108777 | 0.319172 | ZNF844 | 284391 |
| ENSG00000214189 | 9.835399662 | 0.018969975 | 0.1369198 | 0.372377 | NA | NA | NA |
| ENSG00000196646 | 114.2427748 | 0.052387249 | 0.1317781 | 0.3767085 | 0.6384862 | ZNF136 | 7695 |
| ENSG00000197857 | 83.2361684 | 0.0310819 | 0.1292056 | 0.5474127 | 0.7690111 | ZNF44 | 51710 |

| | | | | | | | | |
|---|---|---|---|---|---|---|---|---|
| ENSG00000188868 | 20.37567178 | -0.002954714 | 0.133125 | 0.9194726 | NA | ZNF563 | | 147837 |
| ENSG00000198342 | 14.79267429 | 0.017456619 | 0.1356081 | 0.5102443 | NA | ZNF442 | | 79973 |
| ENSG00000196466 | 77.82003434 | 0.047482401 | 0.1335389 | 0.3841376 | 0.6447633 | ZNF799 | | 90576 |
| ENSG00000180855 | 82.33253447 | 0.010743903 | 0.1239733 | 0.8406021 | 0.9344745 | ZNF443 | | 10224 |
| ENSG00000188033 | 9.653155923 | 0.005972403 | 0.1352639 | 0.7731845 | NA | ZNF490 | | 57474 |
| ENSG00000173875 | 221.2906491 | -0.010058124 | 0.1096229 | 0.9019427 | 0.9616276 | ZNF791 | | 163049 |
| ENSG00000178464 | 1266.820335 | 0.000750307 | 0.0746141 | 0.9912302 | 0.9962622 | RPL10P16 | | 284393 |
| ENSG00000104774 | 240.6539129 | -0.012371012 | 0.1110009 | 0.8490964 | 0.9377876 | MAN2B1 | | 4125 |
| ENSG00000123154 | 132.6913149 | -0.046846456 | 0.1252 | 0.4529082 | 0.6987417 | WDR83 | | 84292 |
| ENSG00000105583 | 308.0247976 | -0.062918628 | 0.1118971 | 0.3780423 | 0.6395077 | WDR83OS | | 51398 |
| ENSG00000095059 | 298.382745 | -0.104017531 | 0.1269491 | 0.1791204 | 0.4282247 | DHPS | | 1725 |
| ENSG00000132004 | 80.62181583 | 0.088715429 | 0.1602142 | 0.131517 | 0.3569354 | FBXW9 | | 84261 |
| ENSG00000105576 | 978.0619469 | 0.080792396 | 0.0848055 | 0.2364849 | 0.5007816 | TNPO2 | | 30000 |
| ENSG00000123144 | 1244.859486 | 0.018545308 | 0.0737085 | 0.766024 | 0.8994294 | TRIR | | 79002 |
| ENSG00000198356 | 879.8209633 | 0.06541227 | 0.0861783 | 0.3409358 | 0.6081036 | GET3 | | 439 |
| ENSG00000095066 | 100.486062 | 0.045706288 | 0.1301609 | 0.4277891 | 0.6791494 | HOOK2 | | 29911 |
| ENSG00000171223 | 286.2725641 | 0.685078882 | 0.1816221 | 9.19E-06 | 0.0002355 | JUNB | | 3726 |
| ENSG00000167815 | 524.9473374 | -0.04764071 | 0.098115 | 0.4957509 | 0.7310915 | PRDX2 | | 7001 |
| ENSG00000104889 | 363.3005074 | 0.066920496 | 0.1106174 | 0.3548089 | 0.618955 | RNASEH2A | | 10535 |
| ENSG00000132026 | 19.93575306 | 0.018462389 | 0.1348053 | 0.547592 | NA | RTBDN | | 83546 |
| ENSG00000105613 | 58.6101461 | 0.056662753 | 0.1421381 | 0.252538 | 0.5191652 | MAST1 | | 22983 |
| ENSG00000105612 | 425.8025824 | 0.113855896 | 0.1179242 | 0.1491392 | 0.3839752 | DNASE2 | | 1777 |
| ENSG00000105607 | 160.7758058 | 0.026057332 | 0.1177778 | 0.6779638 | 0.8532068 | GCDH | | 2639 |
| ENSG00000179115 | 938.0153207 | 0.059858087 | 0.0869443 | 0.3687762 | 0.6315515 | FARSA | | 2193 |
| ENSG00000179218 | 7734.439406 | -0.067615738 | 0.056655 | 0.1883969 | 0.4390789 | CALR | | 811 |
| ENSG00000267458 | 10.44584584 | -0.022240404 | 0.1373973 | 0.3258731 | NA | NA | NA | |
| ENSG00000179262 | 545.7953404 | 0.032212452 | 0.0932916 | 0.6341247 | 0.8257759 | RAD23A | | 5886 |
| ENSG00000179271 | 545.8487505 | -6.60E-05 | 0.0925838 | 0.9982349 | 0.9989595 | GADD45GIF | | 90480 |
| ENSG00000008441 | 394.9546716 | -0.090263924 | 0.1131679 | 0.229918 | 0.490982 | NFIX | | 4784 |
| ENSG00000104903 | 14.59872288 | -0.007646143 | 0.1342991 | 0.769453 | NA | LYL1 | | 4066 |
| ENSG00000104907 | 653.7742781 | 0.318355776 | 0.1193511 | 0.0009243 | 0.010381 | TRMT1 | | 55621 |
| ENSG00000160877 | 1490.263241 | -0.116607726 | 0.0775389 | 0.0728826 | 0.2468371 | NACC1 | | 112939 |
| ENSG00000104915 | 222.3376561 | -0.102090086 | 0.1390555 | 0.1774164 | 0.4268322 | STX10 | | 8677 |
| ENSG00000160888 | 732.2193881 | 0.187683238 | 0.1202452 | 0.0294671 | 0.1366977 | IER2 | | 9592 |
| ENSG00000104957 | 114.6083946 | 0.046043787 | 0.1292094 | 0.4336641 | 0.684513 | YJU2B | | 81576 |
| ENSG00000037757 | 267.4503487 | -0.344681688 | 0.1829202 | 0.004871 | 0.0373101 | MRI1 | | 84245 |
| ENSG00000104979 | 505.8176979 | -0.102867239 | 0.1065483 | 0.1735281 | 0.4207976 | C19orf53 | | 28974 |
| ENSG00000132003 | 80.19911205 | -0.04915686 | 0.133832 | 0.3713482 | 0.6334284 | ZSWIM4 | | 65249 |
| ENSG00000267519 | 97.16825075 | 0.003653449 | 0.1223158 | 0.9484922 | 0.9796055 | NA | NA | |
| ENSG00000132016 | 55.92835164 | 0.048025641 | 0.1377092 | 0.3261555 | 0.5942836 | BRME1 | | 79173 |
| ENSG00000132024 | 422.4335042 | 0.061980237 | 0.1026928 | 0.3873996 | 0.6478474 | CC2D1A | | 54862 |
| ENSG00000132017 | 350.2493384 | -0.05632697 | 0.107773 | 0.4266279 | 0.6787658 | DCAF15 | | 90379 |
| ENSG00000132005 | 229.8794057 | 0.061705522 | 0.1182784 | 0.3801873 | 0.641295 | RFX1 | | 5989 |
| ENSG00000104998 | 255.1869076 | -0.076283391 | 0.1239836 | 0.2894854 | 0.5595695 | IL27RA | | 9466 |
| ENSG00000187867 | 22.9161143 | -0.014443366 | 0.1342622 | 0.6281789 | NA | PALM3 | | 342979 |
| ENSG00000141854 | 20.38600634 | 0.000499172 | 0.1334141 | 0.9885515 | NA | MISP3 | | 113230 |
| ENSG00000141858 | 390.1831777 | -0.095192402 | 0.1142099 | 0.2097025 | 0.4663117 | SAMD1 | | 90378 |
| ENSG00000072062 | 459.9823185 | -0.071631964 | 0.1041201 | 0.3228121 | 0.5915158 | PRKACA | | 5566 |
| ENSG00000105011 | 597.6463779 | 0.348235402 | 0.122796 | 0.0004876 | 0.0062934 | ASF1B | | 55723 |
| ENSG00000267169 | 17.06715387 | 0.021354921 | 0.1361373 | 0.4428639 | NA | ADGRL1-AS | | 100507373 |
| ENSG00000072071 | 344.986233 | -0.111719338 | 0.1230838 | 0.1560418 | 0.3948708 | ADGRL1 | | 22859 |
| ENSG00000123146 | 398.4263198 | -0.120204592 | 0.1228578 | 0.1321732 | 0.3581236 | ADGRE5 | | 976 |
| ENSG00000123136 | 1149.80907 | -0.259817835 | 0.091969 | 0.0008454 | 0.0097006 | DDX39A | | 10212 |
| ENSG00000123143 | 805.4455694 | 0.056127409 | 0.0862279 | 0.4041417 | 0.6612662 | PKN1 | | 5585 |
| ENSG00000160951 | 14.58111819 | -0.004813074 | 0.1342602 | 0.8486919 | NA | PTGER1 | | 5731 |
| ENSG00000123159 | 942.1089439 | -0.17065256 | 0.0955505 | 0.0242932 | 0.1183359 | GIPC1 | | 10755 |
| ENSG00000132002 | 817.5105168 | 0.076565917 | 0.0914206 | 0.2756106 | 0.5439328 | DNAJB1 | | 3337 |
| ENSG00000099797 | 503.6523478 | -0.05621793 | 0.1017125 | 0.4277505 | 0.6791494 | TECR | | 9524 |
| ENSG00000099795 | 324.893787 | -0.048736765 | 0.1077903 | 0.4817093 | 0.720836 | NDUFB7 | | 4713 |
| ENSG00000269378 | 10.87102571 | -0.00936742 | 0.135135 | 0.6812211 | NA | NA | NA | |
| ENSG00000160961 | 32.35235496 | 0.036883775 | 0.1377245 | 0.3386197 | NA | ZNF333 | | 84449 |
| ENSG00000127507 | 21.85355437 | 0.08149882 | 0.1700511 | 0.0211964 | NA | ADGRE2 | | 30817 |
| ENSG00000105137 | 992.7885057 | -0.035098522 | 0.0806555 | 0.5903886 | 0.8000382 | SYDE1 | | 85360 |
| ENSG00000105135 | 790.4942987 | -0.009390021 | 0.0829455 | 0.8859085 | 0.9542113 | ILVBL | | 10994 |
| ENSG00000270143 | 20.30315949 | -0.020296124 | 0.1352912 | 0.4985871 | NA | NA | NA | |
| ENSG00000141867 | 1540.839052 | -0.122023849 | 0.0776826 | 0.0611451 | 0.2205795 | BRD4 | | 23476 |
| ENSG00000105127 | 965.4260406 | -0.196332312 | 0.0966078 | 0.011219 | 0.069046 | AKAP8 | | 10270 |
| ENSG00000011243 | 1053.228666 | -0.031765817 | 0.0816387 | 0.627796 | 0.8225865 | AKAP8L | | 26993 |
| ENSG00000011451 | 1411.340096 | 0.054735103 | 0.0767632 | 0.391971 | 0.6522832 | WIZ | | 58525 |
| ENSG00000167460 | 6076.957605 | -0.041637095 | 0.0636522 | 0.4569937 | 0.7016487 | TPM4 | | 7171 |

| | | | | | | | | |
|---|---|---|---|---|---|---|---|---|
| ENSG00000167461 | 1282.376102 | -0.071060055 | 0.0799873 | 0.2774807 | 0.546125 | RAB8A | | 4218 |
| ENSG00000269243 | 18.20895957 | 0.01830277 | 0.135238 | 0.5256809 | NA | NA | NA | |
| ENSG00000105058 | 931.0283361 | -0.026691297 | 0.0829821 | 0.6853791 | 0.8560726 | FAM32A | | 26017 |
| ENSG00000072958 | 1596.456928 | 0.026561902 | 0.0704059 | 0.660604 | 0.8426903 | AP1M1 | | 8907 |
| ENSG00000127528 | 102.2925554 | 0.038240109 | 0.1272192 | 0.5050016 | 0.7381959 | KLF2 | | 10365 |
| ENSG00000127527 | 557.5290305 | 0.07261414 | 0.0976958 | 0.3117385 | 0.5816031 | EPS15L1 | | 58513 |
| ENSG00000105072 | 108.8309263 | 0.306332384 | 0.3380831 | 0.0168937 | 0.091656 | C19orf44 | | 84167 |
| ENSG00000085872 | 1304.330345 | 0.0217707 | 0.0735041 | 0.7264408 | 0.8776165 | CHERP | | 10523 |
| ENSG00000127526 | 898.7905703 | 0.027249141 | 0.0818709 | 0.6791603 | 0.8535466 | SLC35E1 | | 79939 |
| ENSG00000105085 | 118.330396 | -0.022405679 | 0.1220311 | 0.6968674 | 0.8616368 | MED26 | | 9441 |
| ENSG00000214046 | 375.9026729 | -0.137955852 | 0.1285707 | 0.0959176 | 0.2943036 | SMIM7 | | 79086 |
| ENSG00000269044 | 35.7132687 | -0.032880386 | 0.1363445 | 0.3882138 | NA | NA | NA | |
| ENSG00000267904 | 25.05984541 | -0.006535648 | 0.1324113 | 0.8442153 | NA | NA | NA | |
| ENSG00000072954 | 134.7698603 | 0.10160449 | 0.1584246 | 0.1424545 | 0.3734315 | TMEM38A | | 79041 |
| ENSG00000127511 | 1157.154219 | -0.027847573 | 0.0766467 | 0.6604466 | 0.8426737 | SIN3B | | 23309 |
| ENSG00000131351 | 430.8709285 | -0.086639995 | 0.1088961 | 0.2380457 | 0.5026414 | HAUS8 | | 93323 |
| ENSG00000099331 | 2515.152775 | -0.175520006 | 0.0737195 | 0.0060353 | 0.0435839 | MYO9B | | 4650 |
| ENSG00000053501 | 130.8878477 | 0.223795896 | 0.2562668 | 0.0316898 | 0.1430325 | USE1 | | 55850 |
| ENSG00000099330 | 85.76373254 | 0.024733026 | 0.1256175 | 0.6481631 | 0.836587 | OCEL1 | | 79629 |
| ENSG00000160113 | 924.6901327 | -0.050569012 | 0.0826879 | 0.4432837 | 0.6918756 | NR2F6 | | 2063 |
| ENSG00000105393 | 290.4763695 | -0.10265503 | 0.1292639 | 0.182602 | 0.4321888 | BABAM1 | | 29086 |
| ENSG00000127220 | 294.0788287 | 0.424379617 | 0.1799242 | 0.0012866 | 0.0136374 | ABHD8 | | 79575 |
| ENSG00000130312 | 487.6759283 | 0.084438045 | 0.1044029 | 0.2528833 | 0.5196921 | MRPL34 | | 64981 |
| ENSG00000130311 | 1082.881408 | 0.086898356 | 0.0826558 | 0.1971411 | 0.4503823 | DDA1 | | 79016 |
| ENSG00000074855 | 193.0331732 | 0.085465755 | 0.1326536 | 0.2373546 | 0.5019079 | ANO8 | | 57719 |
| ENSG00000130299 | 568.9905695 | 0.267132997 | 0.1252734 | 0.0048908 | 0.0374374 | GTPBP3 | | 84705 |
| ENSG00000141971 | 358.6356941 | -0.013063467 | 0.1025521 | 0.8465446 | 0.9372292 | MVB12A | | 93343 |
| ENSG00000130304 | 79.29518072 | -0.083786146 | 0.1537609 | 0.1620497 | 0.4039797 | SLC27A1 | | 376497 |
| ENSG00000269439 | 17.57962197 | 0.013016596 | 0.1342973 | 0.6553297 | NA | PGLS-DT | | 100507551 |
| ENSG00000130313 | 216.2108781 | 0.102098189 | 0.1402004 | 0.1760403 | 0.4249487 | PGLS | | 25796 |
| ENSG00000130309 | 2934.731723 | -0.010527639 | 0.0601837 | 0.8311566 | 0.9304016 | COLGALT1 | | 79709 |
| ENSG00000130477 | 17.81866703 | 0.070736751 | 0.1620533 | 0.016288 | NA | UNC13A | | 23025 |
| ENSG00000130479 | 472.0099503 | -0.015789453 | 0.0970348 | 0.8168634 | 0.9252377 | MAP1S | | 55201 |
| ENSG00000130475 | 24.62346247 | -0.029393281 | 0.1368028 | 0.3778368 | NA | FCHO1 | | 23149 |
| ENSG00000105640 | 8598.205124 | 0.067978741 | 0.0607258 | 0.210582 | 0.4672888 | RPL18A | | 6142 |
| ENSG00000007080 | 1542.763993 | 0.029560118 | 0.0708393 | 0.6263913 | 0.8214749 | CCDC124 | | 115098 |
| ENSG00000105642 | 23.18159726 | -0.001793243 | 0.1325367 | 0.9540126 | NA | KCNN1 | | 3780 |
| ENSG00000105643 | 369.3458936 | 0.204766538 | 0.1531672 | 0.0328246 | 0.1463432 | ARRDC2 | | 27106 |
| ENSG00000099308 | 221.7750459 | 0.001984237 | 0.1088791 | 0.9771511 | 0.9913391 | MAST3 | | 23031 |
| ENSG00000254858 | 313.4201946 | 0.04427816 | 0.1064354 | 0.5204225 | 0.7488365 | MPV17L2 | | 84769 |
| ENSG00000105649 | 18.27071045 | -0.025110037 | 0.1370936 | 0.3637866 | NA | RAB3A | | 5864 |
| ENSG00000105650 | 9.198072727 | 0.024890992 | 0.13848 | 0.2233895 | NA | PDE4C | | 5143 |
| ENSG00000130518 | 21.61842621 | 2.488700641 | 0.7283277 | 3.12E-05 | NA | IQCN | | 80726 |
| ENSG00000130522 | 921.6479955 | 0.269524645 | 0.104692 | 0.0016211 | 0.0162407 | JUND | | 3727 |
| ENSG00000130520 | 1795.963317 | 0.122993475 | 0.0829044 | 0.0702843 | 0.2411841 | LSM4 | | 25804 |
| ENSG00000130517 | 436.1233364 | 0.014509167 | 0.0954589 | 0.8326912 | 0.9305286 | PGPEP1 | | 54858 |
| ENSG00000130513 | 2228.115275 | 0.425961325 | 0.1724699 | 0.0009735 | 0.0108193 | GDF15 | | 9518 |
| ENSG00000264175 | 9.844737477 | -0.017370683 | 0.1367692 | 0.3875702 | NA | MIR3189 | | 100422943 |
| ENSG00000130511 | 962.1687987 | -0.117557405 | 0.0955467 | 0.1082673 | 0.318502 | SSBP4 | | 170463 |
| ENSG00000105655 | 2930.786107 | -0.753295332 | 0.0732025 | 5.05E-26 | 2.68E-23 | ISYNA1 | | 51477 |
| ENSG00000105656 | 485.7915252 | 0.007728479 | 0.0963037 | 0.9107765 | 0.9648392 | ELL | | 8178 |
| ENSG00000105701 | 2727.043867 | -0.074658516 | 0.064952 | 0.1911825 | 0.4421326 | FKBP8 | | 23770 |
| ENSG00000105700 | 957.9256583 | 0.20397231 | 0.0986861 | 0.0096783 | 0.062425 | KXD1 | | 79036 |
| ENSG00000221983 | 4748.614752 | -0.014124726 | 0.0593094 | 0.7893668 | 0.9116361 | UBA52 | | 7311 |
| ENSG00000006016 | 14.85077839 | 0.020459114 | 0.1362276 | 0.4410274 | NA | CRLF1 | | 9244 |
| ENSG00000006015 | 244.715802 | -0.04917188 | 0.1150743 | 0.4720046 | 0.7123777 | REX1BD | | 55049 |
| ENSG00000105696 | 11.97737775 | -0.000529926 | 0.1345826 | 0.9795203 | NA | TMEM59L | | 25789 |
| ENSG00000167487 | 210.5052165 | 0.022205356 | 0.1115536 | 0.7378706 | 0.8854328 | KLHL26 | | 55295 |
| ENSG00000105662 | 238.4285907 | -0.018069859 | 0.1084082 | 0.7861062 | 0.9108874 | CRTC1 | | 23373 |
| ENSG00000005007 | 3605.152564 | 0.036126273 | 0.0598601 | 0.5051686 | 0.7382367 | UPF1 | | 5976 |
| ENSG00000223802 | 33.51335306 | 0.032083758 | 0.1359087 | 0.4074392 | NA | CERS1 | | 10715 |
| ENSG00000105669 | 795.692986 | 0.013001635 | 0.0837363 | 0.8451699 | 0.9366632 | COPE | | 11316 |
| ENSG00000105671 | 612.2114508 | 0.193529111 | 0.1151892 | 0.0229685 | 0.1142625 | DDX49 | | 54555 |
| ENSG00000051128 | 706.7895455 | -0.009320409 | 0.0850684 | 0.8878604 | 0.9545517 | HOMER3 | | 9454 |
| ENSG00000269019 | 9.531857498 | -0.007080699 | 0.1352254 | 0.7367658 | NA | NA | NA | |
| ENSG00000064607 | 2277.405661 | -0.083661196 | 0.0691668 | 0.1614975 | 0.4035192 | SUGP2 | | 10147 |
| ENSG00000269131 | 23.00119415 | 0.0002245 | 0.13313 | 0.9963297 | NA | NA | NA | |
| ENSG00000105676 | 993.6824714 | 0.018421265 | 0.0785082 | 0.7752807 | 0.9053192 | ARMC6 | | 93436 |
| ENSG00000181035 | 86.88590654 | 0.004611223 | 0.1235396 | 0.9319481 | 0.9735366 | SLC25A42 | | 284439 |
| ENSG00000064545 | 447.0579486 | 0.134990734 | 0.1193597 | 0.0961415 | 0.2946036 | TMEM161A | | 54929 |

| | | | | | | | | |
|---|---|---|---|---|---|---|---|---|
| ENSG00000254901 | 182.2855494 | -0.019734337 | 0.1150178 | 0.7535233 | 0.8933224 | BORCS8 | | 729991 |
| ENSG00000064490 | 357.7354526 | 0.04242888 | 0.1055163 | 0.493266 | 0.7288856 | RFXANK | | 8625 |
| ENSG00000184162 | 505.9831002 | 0.273669511 | 0.1368632 | 0.0061419 | 0.0442045 | NR2C2AP | | 126382 |
| ENSG00000105705 | 487.0261018 | -0.00769917 | 0.0930127 | 0.9089305 | 0.9640923 | SUGP1 | | 57794 |
| ENSG00000129933 | 589.0535417 | 0.131370043 | 0.1085533 | 0.0933031 | 0.2894347 | MAU2 | | 23383 |
| ENSG00000167491 | 2403.884865 | 0.004510431 | 0.0634865 | 0.9171154 | 0.96756 | GATAD2A | | 54815 |
| ENSG00000178093 | 37.93465788 | 0.05932021 | 0.1474624 | 0.1692349 | NA | TSSK6 | | 83983 |
| ENSG00000186010 | 33.66393922 | 0.020211704 | 0.1327767 | 0.5990238 | NA | NDUFA13 | | 51079 |
| ENSG00000250067 | 67.43938018 | 0.002174337 | 0.126222 | 0.9657423 | 0.9863288 | YJEFN3 | | 374887 |
| ENSG00000160161 | 39.75844132 | 0.003773726 | 0.1299782 | 0.9297585 | NA | CILP2 | | 148113 |
| ENSG00000105717 | 75.84346021 | 0.094032583 | 0.1644956 | 0.1159645 | 0.3320889 | PBX4 | | 80714 |
| ENSG00000064547 | 154.8153029 | 0.049982082 | 0.123295 | 0.4423838 | 0.6915799 | LPAR2 | | 9170 |
| ENSG00000089639 | 462.4945217 | 0.007176076 | 0.0945128 | 0.9172565 | 0.9676216 | GMIP | | 51291 |
| ENSG00000105726 | 983.0722554 | 0.027402915 | 0.0797247 | 0.6734236 | 0.8501877 | ATP13A1 | | 57130 |
| ENSG00000181896 | 442.3298054 | 0.071625398 | 0.1066434 | 0.3150235 | 0.58392 | ZNF101 | | 94039 |
| ENSG00000105708 | 120.7502519 | 0.032787404 | 0.12494 | 0.569007 | 0.7847255 | ZNF14 | | 7561 |
| ENSG00000266904 | 13.66786629 | 0.042499529 | 0.1442388 | 0.0909981 | NA | NA | NA | |
| ENSG00000267419 | 19.74537068 | 0.003739688 | 0.1331749 | 0.9036027 | NA | NA | NA | |
| ENSG00000081665 | 144.0996414 | 0.241561625 | 0.2546687 | 0.0273378 | 0.1291801 | ZNF506 | | 440515 |
| ENSG00000267481 | 15.15723951 | -0.017844409 | 0.1356584 | 0.4989876 | NA | NA | NA | |
| ENSG00000256771 | 176.9984847 | -0.03566435 | 0.1188218 | 0.5746489 | 0.7887497 | ZNF253 | | 56242 |
| ENSG00000184635 | 314.7898287 | 0.327478926 | 0.1829688 | 0.0063498 | 0.0454486 | ZNF93 | | 81931 |
| ENSG00000197124 | 66.62425443 | 0.006926619 | 0.1265068 | 0.8851278 | 0.9535774 | ZNF682 | | 91120 |
| ENSG00000213988 | 223.6089405 | 0.349284738 | 0.2052513 | 0.0065541 | 0.0465398 | ZNF90 | | 7643 |
| ENSG00000267383 | 70.67456655 | 0.017457859 | 0.1275144 | 0.7220399 | 0.8743675 | NA | NA | |
| ENSG00000231205 | 49.2863905 | 0.012458005 | 0.1295454 | 0.7743046 | 0.9046394 | NA | NA | |
| ENSG00000237440 | 118.220867 | -0.098575596 | 0.1551776 | 0.1537472 | 0.3916949 | ZNF737 | | 100129842 |
| ENSG00000188171 | 88.94063348 | 0.001263646 | 0.1228418 | 0.9826587 | 0.9934198 | ZNF626 | | 199777 |
| ENSG00000160229 | 76.53107465 | 0.020852956 | 0.1261168 | 0.6903793 | 0.8589854 | ZNF66 | | 7617 |
| ENSG00000105750 | 154.8301911 | 0.126819981 | 0.1719615 | 0.1005034 | 0.3028063 | ZNF85 | | 7639 |
| ENSG00000118620 | 202.5553524 | 0.029389653 | 0.1131194 | 0.6569507 | 0.8419 | ZNF430 | | 80264 |
| ENSG00000160352 | 565.1820849 | -0.116920038 | 0.1057793 | 0.1260786 | 0.3483265 | ZNF714 | | 148206 |
| ENSG00000268357 | 27.3213849 | -0.02841771 | 0.1358275 | 0.4224972 | NA | NA | NA | |
| ENSG00000196705 | 251.451253 | 0.108287816 | 0.1345024 | 0.1652172 | 0.4093113 | ZNF431 | | 170959 |
| ENSG00000268995 | 16.8628679 | -0.006182003 | 0.1338072 | 0.8252958 | NA | NA | NA | |
| ENSG00000182141 | 163.4094522 | -0.217910612 | 0.2240741 | 0.0342155 | 0.1504221 | ZNF708 | | 7562 |
| ENSG00000172687 | 151.6831071 | 0.065652081 | 0.1296883 | 0.3255392 | 0.5941598 | ZNF738 | | 148203 |
| ENSG00000213976 | 70.04029926 | -0.037606731 | 0.1321056 | 0.4544159 | 0.6995298 | NA | NA | |
| ENSG00000196268 | 151.7232156 | -0.0837599 | 0.1400329 | 0.2231954 | 0.4834315 | ZNF493 | | 284443 |
| ENSG00000197013 | 67.3717557 | -0.026988333 | 0.1289384 | 0.5872631 | 0.7976592 | ZNF429 | | 353088 |
| ENSG00000197020 | 409.4922135 | -0.072479876 | 0.1099203 | 0.3198525 | 0.5886556 | ZNF100 | | 163227 |
| ENSG00000198521 | 323.4078608 | 0.079807312 | 0.117253 | 0.2814211 | 0.550541 | ZNF43 | | 7594 |
| ENSG00000197360 | 104.5436282 | 0.038521644 | 0.1273869 | 0.5054087 | 0.7382367 | ZNF98 | | 148198 |
| ENSG00000229676 | 204.8112403 | 0.129131097 | 0.1540539 | 0.1127923 | 0.325741 | ZNF492 | | 57615 |
| ENSG00000180081 | 11.38055762 | 0.005962122 | 0.134791 | 0.80073 | NA | NA | NA | |
| ENSG00000213973 | 33.18519639 | 0.022412438 | 0.1332454 | 0.5603128 | NA | ZNF99 | | 7652 |
| ENSG00000267886 | 29.50135183 | 0.016253517 | 0.1333223 | 0.6380891 | NA | NA | NA | |
| ENSG00000183850 | 58.53303834 | -0.006714956 | 0.1270699 | 0.8851673 | 0.9535774 | ZNF730 | | 100129543 |
| ENSG00000196081 | 182.2264668 | 0.076074526 | 0.1314754 | 0.2753197 | 0.543704 | ZNF724 | | 440519 |
| ENSG00000269837 | 67.43642674 | -0.011325169 | 0.1261596 | 0.817208 | 0.9254296 | NA | NA | |
| ENSG00000213971 | 55.50004439 | -0.024056293 | 0.1298736 | 0.6083153 | 0.8121763 | NA | NA | |
| ENSG00000167232 | 351.287244 | 0.061102318 | 0.1101719 | 0.3933794 | 0.6540086 | ZNF91 | | 7644 |
| ENSG00000269416 | 108.1241503 | 0.03094103 | 0.1243849 | 0.593371 | 0.802414 | LINC01224 | | 104472717 |
| ENSG00000197372 | 265.9593814 | 0.155567863 | 0.1605555 | 0.0762644 | 0.2542365 | ZNF675 | | 171392 |
| ENSG00000196172 | 182.0661483 | 0.006480606 | 0.1127891 | 0.9294151 | 0.9726302 | ZNF681 | | 148213 |
| ENSG00000233836 | 45.46611509 | -0.081322448 | 0.162633 | 0.0884868 | 0.2801122 | NA | NA | |
| ENSG00000205246 | 219.8735552 | -0.063933549 | 0.1210571 | 0.3575764 | 0.6209582 | NA | NA | |
| ENSG00000213967 | 127.5904811 | -0.014715446 | 0.1188516 | 0.8039746 | 0.9185472 | ZNF726 | | 730087 |
| ENSG00000269397 | 21.78295183 | -0.011406067 | 0.1333903 | 0.7168244 | NA | NA | NA | |
| ENSG00000268362 | 24.61350044 | -0.005784183 | 0.1327999 | 0.8543983 | NA | NA | NA | |
| ENSG00000213096 | 269.7169251 | -0.0001762 | 0.1051544 | 0.9967513 | 0.998947 | ZNF254 | | 9534 |
| ENSG00000261824 | 230.9636081 | 0.107843205 | 0.1369577 | 0.1654394 | 0.409688 | LINC00662 | | 148189 |
| ENSG00000261770 | 44.68667251 | 0.017937916 | 0.1309842 | 0.6691464 | 0.848177 | NA | NA | |
| ENSG00000267575 | 259.867238 | 0.069501022 | 0.1179394 | 0.3347112 | 0.6022573 | LINC02987 | | 101927151 |
| ENSG00000169021 | 2847.039992 | -0.055054339 | 0.0629773 | 0.3279801 | 0.5961001 | UQCRFS1 | | 7386 |
| ENSG00000105171 | 1065.987064 | -0.150347391 | 0.0901666 | 0.0375379 | 0.1604979 | POP4 | | 10775 |
| ENSG00000166289 | 179.2712938 | 0.031043566 | 0.1159756 | 0.6321585 | 0.8251856 | PLEKHF1 | | 79156 |
| ENSG00000131943 | 523.2983364 | -0.109590846 | 0.1075734 | 0.1507918 | 0.3871206 | C19orf12 | | 83636 |
| ENSG00000105173 | 1226.893505 | 0.050590331 | 0.0793165 | 0.4363813 | 0.6867931 | CCNE1 | | 898 |
| ENSG00000105176 | 1814.462836 | -0.130414696 | 0.0752464 | 0.0411182 | 0.1703803 | URI1 | | 8725 |

| | | | | | | | | |
|---|---|---|---|---|---|---|---|---|
| ENSG00000168813 | 1031.668508 | 0.213677328 | 0.1139671 | 0.0130954 | 0.076855 | ZNF507 | | 22847 |
| ENSG00000178904 | 515.6693081 | 0.056963912 | 0.0995 | 0.4222675 | 0.6742808 | DPY19L3 | | 147991 |
| ENSG00000105185 | 1973.131099 | -0.141607659 | 0.0733986 | 0.0242468 | 0.1182672 | PDCD5 | | 9141 |
| ENSG00000105186 | 1191.565376 | -0.11567188 | 0.0859972 | 0.0940582 | 0.2908137 | ANKRD27 | | 84079 |
| ENSG00000186326 | 22.82613417 | 0.021603821 | 0.1350147 | 0.505062 | NA | RGS9BP | | 388531 |
| ENSG00000267475 | 21.51920589 | -0.010292435 | 0.1336683 | 0.7306491 | NA | NA | NA | |
| ENSG00000213965 | 674.2986589 | 0.053589251 | 0.0911519 | 0.4405804 | 0.6895939 | NUDT19 | | 390916 |
| ENSG00000266930 | 33.9197822 | -0.007872963 | 0.1309547 | 0.835391 | NA | NA | NA | |
| ENSG00000121289 | 897.6773875 | 0.140478321 | 0.0965741 | 0.0602941 | 0.2187251 | CEP89 | | 84902 |
| ENSG00000131944 | 256.5770603 | 0.013474755 | 0.1099493 | 0.8388056 | 0.9335959 | FAAP24 | | 91442 |
| ENSG00000131941 | 701.2772939 | -0.041339683 | 0.0876965 | 0.5425735 | 0.7657123 | RHPN2 | | 85415 |
| ENSG00000076650 | 262.7459819 | 0.026756194 | 0.108443 | 0.6928738 | 0.8596319 | GPATCH1 | | 55094 |
| ENSG00000130881 | 20.16785866 | 0.034315926 | 0.1391371 | 0.2733935 | NA | LRP3 | | 4037 |
| ENSG00000153879 | 1111.182168 | 0.409720199 | 0.111415 | 2.20E-05 | 0.0004896 | CEBPG | | 1054 |
| ENSG00000124299 | 1718.801181 | -0.097664361 | 0.0726929 | 0.1148669 | 0.329914 | PEPD | | 5184 |
| ENSG00000153885 | 395.8914815 | 0.190015473 | 0.1438831 | 0.0395108 | 0.1664864 | KCTD15 | | 79047 |
| ENSG00000257103 | 3153.30619 | -0.191160003 | 0.0674489 | 0.001441 | 0.014909 | LSM14A | | 26065 |
| ENSG00000166398 | 505.0035972 | -0.025837185 | 0.0953129 | 0.7060944 | 0.8671822 | GARRE1 | | 9710 |
| ENSG00000105220 | 5385.19771 | -0.012947818 | 0.0609305 | 0.8103545 | 0.9218496 | GPI | | 2821 |
| ENSG00000126249 | 176.0699264 | -0.018237453 | 0.1146808 | 0.7722747 | 0.9034493 | PDCD2L | | 84306 |
| ENSG00000126261 | 4198.491335 | -0.160901814 | 0.0618963 | 0.0037878 | 0.0305349 | UBA2 | | 10054 |
| ENSG00000142279 | 617.1412377 | 0.024854693 | 0.0936106 | 0.718205 | 0.8722654 | WTIP | | 126374 |
| ENSG00000256383 | 19.11470567 | 0.003450137 | 0.1333485 | 0.9087865 | NA | NA | NA | |
| ENSG00000089335 | 589.7899654 | 0.056101574 | 0.0989538 | 0.4567784 | 0.7015026 | ZNF302 | | 55900 |
| ENSG00000197841 | 180.5294125 | 0.168335335 | 0.1869465 | 0.061974 | 0.2223574 | ZNF181 | | 339318 |
| ENSG00000153896 | 82.47063943 | -0.033708377 | 0.1284487 | 0.5274584 | 0.7544502 | ZNF599 | | 148103 |
| ENSG00000168661 | 149.2490342 | 0.063076887 | 0.1293017 | 0.339242 | 0.6066573 | ZNF30 | | 90075 |
| ENSG00000180884 | 65.40730627 | -0.062902521 | 0.1433119 | 0.2435764 | 0.5095683 | ZNF792 | | 126375 |
| ENSG00000089351 | 558.4279802 | 0.117808319 | 0.1070544 | 0.1256277 | 0.3479035 | GRAMD1A | | 57655 |
| ENSG00000105711 | 13.3841532 | -0.009551886 | 0.1347185 | 0.7022545 | NA | SCN1B | | 6324 |
| ENSG00000089327 | 800.0646656 | 0.05608584 | 0.0860277 | 0.4039318 | 0.6611079 | FXYD5 | | 53827 |
| ENSG00000105699 | 1475.151635 | -0.06248952 | 0.0759502 | 0.3248301 | 0.5936077 | LSR | | 51599 |
| ENSG00000105698 | 1413.406592 | -0.125888719 | 0.0797715 | 0.0579934 | 0.2133418 | USF2 | | 7392 |
| ENSG00000161249 | 1795.570408 | -0.107495487 | 0.0784725 | 0.0996313 | 0.3012668 | DMKN | | 93099 |
| ENSG00000236144 | 239.2480837 | 0.023697832 | 0.1090257 | 0.7246574 | 0.8764468 | TMEM147-A | | 100506469 |
| ENSG00000105677 | 1201.676448 | 0.030105012 | 0.077507 | 0.6174159 | 0.8166725 | TMEM147 | | 10430 |
| ENSG00000249115 | 657.2735256 | 0.046310825 | 0.0918209 | 0.5032426 | 0.7374652 | HAUS5 | | 23354 |
| ENSG00000126254 | 1998.633161 | 0.014020081 | 0.0657395 | 0.8050218 | 0.9186624 | RBM42 | | 79171 |
| ENSG00000126267 | 1963.862686 | -0.16171213 | 0.0791002 | 0.0155147 | 0.0862988 | COX6B1 | | 1340 |
| ENSG00000105663 | 21.26014918 | -0.005206513 | 0.1331484 | 0.8622791 | NA | NA | NA | |
| ENSG00000161265 | 43.96744114 | 0.011817253 | 0.12979 | 0.782314 | 0.9095461 | U2AF1L4 | | 199746 |
| ENSG00000205155 | 194.2363275 | 0.02526929 | 0.113502 | 0.6993675 | 0.8632898 | PSENEN | | 55851 |
| ENSG00000267796 | 68.684982 | -0.009426585 | 0.1258955 | 0.8484087 | 0.9375782 | LIN37 | | 55957 |
| ENSG00000167595 | 338.3812504 | -0.029798549 | 0.1048446 | 0.6630423 | 0.8438599 | PROSER3 | | 148137 |
| ENSG00000004777 | 263.408318 | -0.038247261 | 0.1116741 | 0.5714954 | 0.7864627 | ARHGAP33 | | 115703 |
| ENSG00000161270 | 43.04021409 | -0.022277861 | 0.1325452 | 0.5780569 | 0.7912213 | NPHS1 | | 4868 |
| ENSG00000126259 | 266.5582382 | 0.104298915 | 0.1297928 | 0.1787839 | 0.4275952 | KIRREL2 | | 84063 |
| ENSG00000105290 | 493.6857921 | 0.17949839 | 0.1255834 | 0.0392483 | 0.1656881 | APLP1 | | 333 |
| ENSG00000167604 | 514.9625344 | -0.272556741 | 0.1549012 | 0.009844 | 0.063028 | NFKBID | | 84807 |
| ENSG00000126243 | 263.4375034 | -0.039460535 | 0.1108309 | 0.5619828 | 0.7800541 | LRFN3 | | 79414 |
| ENSG00000205138 | 303.7284761 | 0.127932533 | 0.1341569 | 0.1191508 | 0.3370792 | SDHAF1 | | 644096 |
| ENSG00000239382 | 65.14027747 | -0.00532868 | 0.1262658 | 0.9109846 | 0.9648392 | ALKBH6 | | 84964 |
| ENSG00000267698 | 33.46716384 | 0.037456084 | 0.1381338 | 0.3260932 | NA | NA | NA | |
| ENSG00000105270 | 17.26628309 | 0.037809045 | 0.1413571 | 0.1793121 | NA | CLIP3 | | 25999 |
| ENSG00000161277 | 44.78875746 | 0.053014199 | 0.1427428 | 0.2364939 | 0.5007816 | THAP8 | | 199745 |
| ENSG00000075702 | 1874.02272 | -0.008288497 | 0.0667368 | 0.8861376 | 0.9543156 | WDR62 | | 284403 |
| ENSG00000105258 | 691.9021045 | 0.06430304 | 0.0922398 | 0.3579019 | 0.621171 | POLR2I | | 5438 |
| ENSG00000105254 | 2268.102897 | -0.090096243 | 0.0710945 | 0.1397447 | 0.3691041 | TBCB | | 1155 |
| ENSG00000126247 | 6595.806022 | -0.116520943 | 0.0585296 | 0.0274676 | 0.1295313 | CAPNS1 | | 826 |
| ENSG00000196357 | 67.01610264 | 0.030827484 | 0.1298415 | 0.5408189 | 0.7642142 | ZNF565 | | 147929 |
| ENSG00000167635 | 3209.521191 | -0.018088641 | 0.0591414 | 0.7335771 | 0.8823017 | ZNF146 | | 7705 |
| ENSG00000232677 | 691.5746805 | 0.099333162 | 0.0963777 | 0.1687325 | 0.4145035 | LINC00665 | | 100506930 |
| ENSG00000142065 | 88.54617111 | -0.060443566 | 0.1377123 | 0.2960983 | 0.566076 | ZFP14 | | 57677 |
| ENSG00000181007 | 333.3323716 | 0.594611487 | 0.1741789 | 3.84E-05 | 0.0007928 | ZFP82 | | 284406 |
| ENSG00000186017 | 409.390577 | 0.589099894 | 0.1487658 | 4.83E-06 | 0.0001359 | ZNF566 | | 84924 |
| ENSG00000267309 | 37.7005291 | -0.038882294 | 0.1378214 | 0.3320519 | NA | ZNF566-AS1 | | 728752 |
| ENSG00000254004 | 882.204387 | 0.293227586 | 0.1051804 | 0.0007587 | 0.0089705 | ZNF260 | | 339324 |
| ENSG00000186020 | 437.3036302 | 0.327494742 | 0.155616 | 0.0034931 | 0.0286538 | ZNF529 | | 57711 |
| ENSG00000233527 | 72.50350054 | 0.141197232 | 0.2185992 | 0.0473563 | 0.1859327 | ZNF529-AS1 | | 101927599 |
| ENSG00000161298 | 9.549470804 | 0.010786886 | 0.135522 | 0.6204173 | NA | ZNF382 | | 84911 |

| | | | | | | | |
|---|---|---|---|---|---|---|---|
| ENSG00000197808 | 157.8684207 | 0.132785785 | 0.1700852 | 0.0979797 | 0.298358 | ZNF461 | 92283 |
| ENSG00000225975 | 23.48391563 | 0.030328377 | 0.1371093 | 0.3649684 | NA | ZNF567-DT | 101927621 |
| ENSG00000189042 | 290.871087 | 0.125877131 | 0.1360769 | 0.1230958 | 0.3439664 | ZNF567 | 163081 |
| ENSG00000267041 | 492.256077 | 0.978737349 | 0.1357658 | 3.21E-14 | 5.17E-12 | ZNF850 | 342892 |
| ENSG00000197863 | 175.4522113 | 0.129308271 | 0.1614697 | 0.1079419 | 0.3179999 | ZNF790 | 388536 |
| ENSG00000251247 | 60.20569514 | 0.079148708 | 0.1575296 | 0.1263905 | 0.3490229 | ZNF345 | 25850 |
| ENSG00000185869 | 387.1578605 | 0.649338151 | 0.1467084 | 5.93E-07 | 2.18E-05 | ZNF829 | 374899 |
| ENSG00000198453 | 222.7417211 | 0.647238095 | 0.1969479 | 5.53E-05 | 0.0010751 | ZNF568 | 374900 |
| ENSG00000197050 | 214.4948951 | 0.88427501 | 0.2028534 | 6.84E-07 | 2.49E-05 | ZNF420 | 147923 |
| ENSG00000196967 | 193.6967356 | 0.116183256 | 0.1513554 | 0.1355452 | 0.3626084 | ZNF585A | 199704 |
| ENSG00000245680 | 391.4542377 | 0.053554347 | 0.1039312 | 0.4506674 | 0.6974359 | ZNF585B | 92285 |
| ENSG00000188283 | 163.1558247 | 0.125115719 | 0.1624911 | 0.1111141 | 0.3233493 | ZNF383 | 163087 |
| ENSG00000226686 | 29.61559118 | -0.022740544 | 0.1342364 | 0.5220717 | NA | LINC01535 | 101927667 |
| ENSG00000181666 | 503.3349892 | 0.19925817 | 0.1294812 | 0.0268624 | 0.1273454 | ZNF875 | 284459 |
| ENSG00000267682 | 9.405381254 | -0.010156758 | 0.1357771 | 0.6037015 | NA | NA | NA |
| ENSG00000189164 | 227.0195854 | 0.079820919 | 0.1305754 | 0.2628701 | 0.5303256 | ZNF527 | 84503 |
| ENSG00000196437 | 397.4669907 | 0.194396583 | 0.1383641 | 0.0339944 | 0.1497884 | ZNF569 | 148266 |
| ENSG00000171827 | 176.6002076 | 0.192902977 | 0.2113111 | 0.0450902 | 0.1808717 | ZNF570 | 148268 |
| ENSG00000267381 | 40.93680105 | 0.024363695 | 0.1334616 | 0.5349784 | 0.7594484 | NA | NA |
| ENSG00000266916 | 10.25354508 | 0.010500538 | 0.135375 | 0.6391639 | NA | ZNF793-AS1 | 101927720 |
| ENSG00000188227 | 176.0993288 | 0.305961556 | 0.2364258 | 0.0140747 | 0.0809085 | ZNF793 | 390927 |
| ENSG00000171817 | 14.86851515 | -0.007816177 | 0.1345818 | 0.7521414 | NA | ZNF540 | 163255 |
| ENSG00000180479 | 115.9734419 | 0.956891989 | 0.2874154 | 3.79E-05 | 0.0007866 | ZNF571 | 51276 |
| ENSG00000120784 | 678.3848117 | 0.22921849 | 0.1155281 | 0.0091225 | 0.059899 | ZFP30 | 22835 |
| ENSG00000198182 | 183.6730928 | 0.1397388 | 0.1650213 | 0.0937835 | 0.2905267 | ZNF607 | 84775 |
| ENSG00000267152 | 23.7015958 | -0.009492507 | 0.1329693 | 0.767639 | NA | NA | NA |
| ENSG00000189144 | 61.30964284 | -0.032594324 | 0.1312028 | 0.503919 | 0.7380913 | ZNF573 | 126231 |
| ENSG00000267422 | 13.11377858 | 0.045431877 | 0.146203 | 0.0480683 | NA | NA | NA |
| ENSG00000105738 | 1785.006485 | -0.019101325 | 0.0675005 | 0.7427552 | 0.8879463 | SIPA1L3 | 23094 |
| ENSG00000011332 | 328.2177922 | 0.698580491 | 0.1597911 | 7.27E-07 | 2.61E-05 | DPF1 | 8193 |
| ENSG00000167642 | 4275.797016 | -0.099553041 | 0.0599012 | 0.0638113 | 0.2270144 | SPINT2 | 10653 |
| ENSG00000167644 | 12.73400578 | -0.016810608 | 0.1357838 | 0.500016 | NA | C19orf33 | 64073 |
| ENSG00000167645 | 766.2995092 | -0.118555274 | 0.1030956 | 0.1173867 | 0.3339561 | YIF1B | 90522 |
| ENSG00000099337 | 176.1472663 | 0.039674913 | 0.1212208 | 0.5309867 | 0.756397 | KCNK6 | 9424 |
| ENSG00000268913 | 20.62404604 | -0.00474655 | 0.1330971 | 0.8746719 | NA | NA | NA |
| ENSG00000099338 | 11.93871896 | 0.008835631 | 0.1348428 | 0.7173333 | NA | CATSPERG | 57828 |
| ENSG00000099341 | 3999.196983 | -0.072436337 | 0.0605199 | 0.178683 | 0.4275952 | PSMD8 | 5714 |
| ENSG00000188766 | 25.00262706 | 0.038917985 | 0.139911 | 0.2622957 | NA | SPRED3 | 399473 |
| ENSG00000267473 | 16.584842 | 0.014077887 | 0.1346927 | 0.6167216 | NA | NA | NA |
| ENSG00000130244 | 318.8003472 | -0.034339293 | 0.1059953 | 0.6169843 | 0.8164715 | FAM98C | 147965 |
| ENSG00000178982 | 2138.324869 | -0.008884122 | 0.0655853 | 0.8765534 | 0.9498164 | EIF3K | 27335 |
| ENSG00000130402 | 8338.339162 | 0.012419016 | 0.0512639 | 0.7536926 | 0.8933549 | ACTN4 | 81 |
| ENSG00000182472 | 16.52423598 | -0.013142364 | 0.1345685 | 0.6361479 | NA | CAPN12 | 147968 |
| ENSG00000104823 | 453.1856912 | 0.086916092 | 0.1078841 | 0.2433494 | 0.5092377 | ECH1 | 1891 |
| ENSG00000104824 | 4452.426648 | -0.277722521 | 0.067079 | 6.39E-06 | 0.0001712 | HNRNPL | 3191 |
| ENSG00000269688 | 56.07824202 | -0.047824235 | 0.1378471 | 0.3223003 | 0.5910188 | NA | NA |
| ENSG00000068903 | 748.6522497 | 0.292878532 | 0.1117928 | 0.0012245 | 0.0131584 | SIRT2 | 22933 |
| ENSG00000104825 | 1823.551336 | 0.72115789 | 0.0858864 | 3.00E-18 | 7.01E-16 | NFKBIB | 4793 |
| ENSG00000104835 | 94.27327801 | -0.107186537 | 0.1712077 | 0.1040761 | 0.3098146 | SARS2 | 54938 |
| ENSG00000128626 | 1130.026052 | 0.13424797 | 0.0904915 | 0.061192 | 0.2206803 | MRPS12 | 6183 |
| ENSG00000269486 | 10.5337669 | -0.007953816 | 0.1350815 | 0.7220991 | NA | NA | NA |
| ENSG00000269190 | 605.6649089 | 0.165153794 | 0.1173901 | 0.0481177 | 0.1877925 | FBXO17 | 115290 |
| ENSG00000161243 | 39.10770294 | 0.036053026 | 0.1362102 | 0.3853831 | NA | FBXO27 | 126433 |
| ENSG00000183760 | 37.45068795 | 0.10451334 | 0.1928632 | 0.0206359 | NA | ACP7 | 390928 |
| ENSG00000130669 | 1195.545345 | -0.081042727 | 0.0790163 | 0.2132933 | 0.4704455 | PAK4 | 10298 |
| ENSG00000128011 | 66.85711755 | 0.032360109 | 0.1305951 | 0.5174473 | 0.7467632 | LRFN1 | 57622 |
| ENSG00000179134 | 1849.201767 | 0.0259434 | 0.0691431 | 0.6660391 | 0.8452861 | SAMD4B | 55095 |
| ENSG00000006712 | 2034.644612 | -0.081316061 | 0.0696801 | 0.1756603 | 0.4242944 | PAF1 | 54623 |
| ENSG00000063322 | 1146.670754 | 0.035960921 | 0.0760814 | 0.558481 | 0.7775341 | MED29 | 55588 |
| ENSG00000128016 | 139.0482673 | 0.304445702 | 0.2884719 | 0.0166349 | 0.0905462 | ZFP36 | 7538 |
| ENSG00000090924 | 1142.966116 | -0.200881273 | 0.0944471 | 0.0087446 | 0.0580043 | PLEKHG2 | 64857 |
| ENSG00000105193 | 12262.44253 | -0.061885858 | 0.0510882 | 0.1892256 | 0.4402444 | RPS16 | 6217 |
| ENSG00000196235 | 3876.338017 | 0.029958515 | 0.0577136 | 0.5676046 | 0.7836298 | SUPT5H | 6829 |
| ENSG00000105197 | 2226.976185 | 0.071093995 | 0.0709118 | 0.2423167 | 0.5081168 | TIMM50 | 92609 |
| ENSG00000090932 | 70.80999685 | 0.023281173 | 0.1276825 | 0.64623 | 0.8354857 | DLL3 | 10683 |
| ENSG00000176401 | 105.1788702 | 0.028350158 | 0.1238229 | 0.6213938 | 0.818389 | EID2B | 126272 |
| ENSG00000176396 | 548.4344677 | 0.027828634 | 0.0939618 | 0.6793943 | 0.8536092 | EID2 | 163126 |
| ENSG00000105204 | 72.62715282 | 0.581504109 | 0.4178425 | 0.0049479 | 0.0376802 | DYRK1B | 9149 |
| ENSG00000105202 | 3625.792319 | -0.173773964 | 0.0657381 | 0.0029883 | 0.025591 | FBL | 2091 |
| ENSG00000187534 | 81.36594666 | 0.023827498 | 0.1261128 | 0.655198 | 0.8408446 | NA | NA |

| | | | | | | | | |
|---|---|---|---|---|---|---|---|---|
| ENSG00000013275 | 2597.795911 | -0.027507678 | 0.0621718 | 0.6185499 | 0.817617 | PSMC4 | | 5704 |
| ENSG00000187187 | 31.98283897 | 0.03266974 | 0.1364305 | 0.3876743 | NA | ZNF546 | | 339327 |
| ENSG00000269069 | 31.81853058 | -0.028790475 | 0.1352612 | 0.4408385 | NA | NA | NA | |
| ENSG00000128000 | 368.2683991 | 0.010359573 | 0.1005866 | 0.8788811 | 0.9512796 | ZNF780B | | 163131 |
| ENSG00000197782 | 295.1840732 | 0.014843663 | 0.1060508 | 0.826478 | 0.9286016 | ZNF780A | | 284323 |
| ENSG00000130758 | 242.3263426 | 0.196459142 | 0.1764124 | 0.0430536 | 0.1755967 | MAP3K10 | | 4294 |
| ENSG00000105221 | 2197.499436 | -0.065100957 | 0.0669433 | 0.2667278 | 0.5346062 | AKT2 | | 208 |
| ENSG00000205041 | 16.80711765 | 0.023603321 | 0.1369404 | 0.3751133 | NA | NA | NA | |
| ENSG00000160392 | 400.725558 | 0.025778101 | 0.1006369 | 0.709268 | 0.8682712 | C19orf47 | | 126526 |
| ENSG00000105223 | 1425.763508 | 0.14743955 | 0.0849225 | 0.0343309 | 0.150759 | PLD3 | | 23646 |
| ENSG00000105227 | 178.1481118 | 0.066378491 | 0.1272538 | 0.330607 | 0.5988497 | PRX | | 57716 |
| ENSG00000197019 | 550.6195714 | 0.478007973 | 0.1258098 | 1.14E-05 | 0.0002833 | SERTAD1 | | 29950 |
| ENSG00000167565 | 253.8497243 | -0.115777606 | 0.1370584 | 0.144804 | 0.3772945 | SERTAD3 | | 29946 |
| ENSG00000090013 | 559.4168606 | 0.253546883 | 0.1238914 | 0.0065283 | 0.0463852 | BLVRB | | 645 |
| ENSG00000160410 | 1107.709076 | 0.127933876 | 0.0858053 | 0.0660743 | 0.2322697 | SHKBP1 | | 92799 |
| ENSG00000090006 | 1120.530957 | -0.023453754 | 0.0811536 | 0.7189291 | 0.8729533 | LTBP4 | | 8425 |
| ENSG00000105245 | 407.607988 | -0.042305691 | 0.1029947 | 0.5463047 | 0.7682016 | NUMBL | | 9253 |
| ENSG00000123815 | 258.0842428 | 0.027642927 | 0.1082185 | 0.6840257 | 0.8552884 | COQ8B | | 79934 |
| ENSG00000086544 | 199.1320523 | 0.494515938 | 0.2193304 | 0.0013355 | 0.0140851 | ITPKC | | 80271 |
| ENSG00000188493 | 132.238557 | 0.106255485 | 0.1602564 | 0.1343049 | 0.3609792 | ACTMAP | | 284325 |
| ENSG00000077312 | 1356.771895 | 0.094777105 | 0.0845228 | 0.1612561 | 0.4033013 | SNRPA | | 6626 |
| ENSG00000167578 | 25.06226959 | 0.081270288 | 0.1694263 | 0.0243704 | NA | RAB4B | | 53916 |
| ENSG00000269858 | 296.0753642 | -0.088909664 | 0.1210167 | 0.2370512 | 0.5013571 | EGLN2 | | 112398 |
| ENSG00000167600 | 49.91326805 | 0.074713945 | 0.1548829 | 0.1358623 | 0.3628767 | CYP2S1 | | 29785 |
| ENSG00000167601 | 3387.294531 | -0.01926537 | 0.0654489 | 0.7367208 | 0.8845504 | AXL | | 558 |
| ENSG00000105323 | 7261.381427 | -0.10267171 | 0.0550643 | 0.0409881 | 0.1702639 | HNRNPUL1 | | 11100 |
| ENSG00000105329 | 2544.70328 | -0.001132131 | 0.0623241 | 0.9828869 | 0.9934365 | TGFB1 | | 7040 |
| ENSG00000142039 | 556.6401862 | 0.033766949 | 0.0954192 | 0.6213734 | 0.818389 | CCDC97 | | 90324 |
| ENSG00000142046 | 29.44433207 | 0.037452857 | 0.1391337 | 0.2894855 | NA | TMEM91 | | 641649 |
| ENSG00000123810 | 92.3338501 | -0.046138726 | 0.1308856 | 0.4161728 | 0.669787 | B9D2 | | 80776 |
| ENSG00000077348 | 718.5288765 | -0.010118605 | 0.0873892 | 0.8794382 | 0.9513156 | EXOSC5 | | 56915 |
| ENSG00000105341 | 1081.065634 | -0.119397301 | 0.0866793 | 0.0859232 | 0.2746016 | DMAC2 | | 55101 |
| ENSG00000105372 | 13291.92201 | -0.001546178 | 0.0523489 | 0.8503053 | 0.9386264 | RPS19 | | 6223 |
| ENSG00000076928 | 2540.887058 | -0.044243341 | 0.0670235 | 0.448124 | 0.6957325 | ARHGEF1 | | 9138 |
| ENSG00000268041 | 11.06766902 | -0.001536038 | 0.1349844 | 0.9396349 | NA | ERFL | | 390937 |
| ENSG00000105404 | 569.1815913 | 0.058475313 | 0.101137 | 0.4119143 | 0.6661689 | RABAC1 | | 10567 |
| ENSG00000105409 | 114.4323121 | -0.014483827 | 0.1212524 | 0.7983161 | 0.9162772 | ATP1A3 | | 478 |
| ENSG00000105732 | 494.178841 | 0.257864508 | 0.156631 | 0.0130158 | 0.0765231 | ZNF574 | | 64763 |
| ENSG00000028277 | 100.1304448 | -0.011493926 | 0.1218867 | 0.835725 | 0.9315873 | POU2F2 | | 5452 |
| ENSG00000254887 | 8.397339159 | 0.029060549 | 0.1399619 | 0.1301233 | NA | NA | NA | |
| ENSG00000160570 | 543.016674 | 0.358302333 | 0.1295236 | 0.0005708 | 0.0071217 | DEDD2 | | 162989 |
| ENSG00000167625 | 383.8808377 | 0.099266776 | 0.1162831 | 0.1958165 | 0.4484967 | ZNF526 | | 116115 |
| ENSG00000105723 | 1145.260157 | 0.068513719 | 0.0809167 | 0.3029376 | 0.5723556 | GSK3A | | 2931 |
| ENSG00000105722 | 1670.716277 | -0.046607192 | 0.0714032 | 0.4436796 | 0.6921236 | ERF | | 2077 |
| ENSG00000079432 | 1860.064206 | -0.110917251 | 0.0762021 | 0.0837521 | 0.2703169 | CIC | | 23152 |
| ENSG00000079462 | 1117.680597 | 0.039006731 | 0.0779481 | 0.5331222 | 0.758237 | PAFAH1B3 | | 5050 |
| ENSG00000188368 | 72.14269692 | 0.027710943 | 0.1290397 | 0.5814731 | 0.7933122 | PRR19 | | 284338 |
| ENSG00000167619 | 83.75000717 | -0.110691323 | 0.1795485 | 0.0836391 | 0.2700934 | TMEM145 | | 284339 |
| ENSG00000105429 | 615.8008595 | 0.011789146 | 0.0898915 | 0.8653461 | 0.9457908 | MEGF8 | | 1954 |
| ENSG00000105427 | 29.82983917 | -0.018931563 | 0.1332015 | 0.5999213 | NA | CNFN | | 84518 |
| ENSG00000213904 | 25.88400294 | 0.032157489 | 0.1372885 | 0.3559075 | NA | LIPE-AS1 | | 100996307 |
| ENSG00000079435 | 122.008161 | -0.067353845 | 0.1349223 | 0.2922552 | 0.5617739 | LIPE | | 3991 |
| ENSG00000231924 | 22.93390154 | 0.052318553 | 0.1468523 | 0.1273062 | NA | PSG1 | | 5669 |
| ENSG00000204941 | 10.36497647 | 0.024760383 | 0.1383139 | 0.246457 | NA | PSG5 | | 5673 |
| ENSG00000243137 | 355.72651 | 0.86813772 | 0.1736222 | 3.02E-08 | 1.66E-06 | PSG4 | | 5672 |
| ENSG00000183668 | 178.9765604 | -0.50137981 | 0.2299491 | 0.0015307 | 0.0155888 | PSG9 | | 5678 |
| ENSG00000204936 | 11.73333742 | 0.020372866 | 0.1367205 | 0.3970895 | NA | CD177 | | 57126 |
| ENSG00000176531 | 283.1005749 | 0.072969606 | 0.1164868 | 0.316067 | 0.5849906 | PHLDB3 | | 653583 |
| ENSG00000105755 | 309.9093041 | 0.242744047 | 0.1684847 | 0.0197744 | 0.1028623 | ETHE1 | | 23474 |
| ENSG00000176472 | 25.98692315 | 0.019132819 | 0.1338989 | 0.5779657 | NA | ZNF575 | | 284346 |
| ENSG00000073050 | 489.7478637 | 0.122058892 | 0.1163138 | 0.1235344 | 0.344475 | XRCC1 | | 7515 |
| ENSG00000167378 | 768.3248301 | 0.131924047 | 0.1108892 | 0.0986953 | 0.2997557 | IRGQ | | 126298 |
| ENSG00000124444 | 211.9904716 | 0.048409897 | 0.118981 | 0.4698348 | 0.7112787 | ZNF576 | | 79177 |
| ENSG00000226763 | 17.75866059 | 0.023123285 | 0.1361857 | 0.4323986 | NA | SRRM5 | | 100170229 |
| ENSG00000131116 | 679.7035491 | -0.014387992 | 0.0860978 | 0.8289771 | 0.9295652 | ZNF428 | | 126299 |
| ENSG00000105767 | 246.1127288 | -0.013117936 | 0.1083411 | 0.8423029 | 0.9350382 | CADM4 | | 199731 |
| ENSG00000011422 | 458.3631478 | 0.351934942 | 0.1439052 | 0.0013882 | 0.0144905 | PLAUR | | 5329 |
| ENSG00000105771 | 1817.046804 | 0.438784523 | 0.0893615 | 8.52E-08 | 4.15E-06 | SMG9 | | 56006 |
| ENSG00000104783 | 978.7520891 | 0.296348685 | 0.1046807 | 0.0006577 | 0.007986 | KCNN4 | | 3783 |
| ENSG00000159871 | 17.54737342 | 0.029140234 | 0.1378821 | 0.3209606 | NA | LYPD5 | | 284348 |

| | | | | | | | |
|---|---|---|---|---|---|---|---|
| ENSG00000167637 | 244.755413 | -0.02265877 | 0.1086068 | 0.7343867 | 0.8828208 | ZNF283 | 284349 |
| ENSG00000176222 | 71.18348286 | 0.038059544 | 0.1312005 | 0.4681148 | 0.7103207 | ZNF404 | 342908 |
| ENSG00000267058 | 25.08619695 | -0.007475882 | 0.132402 | 0.823354 | NA | LOC100505715 | 100505715 |
| ENSG00000124459 | 588.8050587 | 0.212858928 | 0.1273855 | 0.0190383 | 0.1002193 | ZNF45 | 7596 |
| ENSG00000159905 | 54.97900462 | 0.053596292 | 0.1406681 | 0.2737727 | 0.5422073 | ZNF221 | 7638 |
| ENSG00000204920 | 224.1610851 | 0.034525988 | 0.1151802 | 0.6010712 | 0.8073028 | ZNF155 | 7711 |
| ENSG00000266921 | 12.90577427 | -0.000236715 | 0.1344043 | 0.9899203 | NA | ZNF230-DT | 101928063 |
| ENSG00000159882 | 160.2175346 | 0.182897989 | 0.203385 | 0.0507891 | 0.194132 | ZNF230 | 7773 |
| ENSG00000159885 | 105.3887078 | 0.568539908 | 0.3190756 | 0.0029119 | 0.0251765 | ZNF222 | 7673 |
| ENSG00000178386 | 22.22799526 | 0.058680251 | 0.1509789 | 0.0843914 | NA | ZNF223 | 7766 |
| ENSG00000186026 | 49.25867448 | 0.025077345 | 0.1312097 | 0.5762161 | 0.7897843 | ZNF284 | 342909 |
| ENSG00000267680 | 481.8484019 | 0.059905941 | 0.0997479 | 0.4005505 | 0.6584337 | ZNF224 | 7767 |
| ENSG00000186019 | 27.09487152 | 0.046773042 | 0.1436318 | 0.1787304 | NA | ZNF225-AS1 | 100379224 |
| ENSG00000256294 | 162.0874616 | 0.074338411 | 0.1320525 | 0.2802358 | 0.5489576 | ZNF225 | 7768 |
| ENSG00000263002 | 261.7450754 | -0.025996878 | 0.1086672 | 0.6979215 | 0.8623251 | ZNF234 | 10780 |
| ENSG00000167380 | 370.2906737 | 0.133841334 | 0.1283022 | 0.1044861 | 0.3104023 | ZNF226 | 7769 |
| ENSG00000131115 | 516.6418073 | 0.451134337 | 0.1381207 | 8.48E-05 | 0.0015361 | ZNF227 | 7770 |
| ENSG00000159917 | 242.3130991 | 0.056756658 | 0.1156238 | 0.4175101 | 0.6708302 | ZNF235 | 9310 |
| ENSG00000159915 | 25.7442227 | -0.006243003 | 0.1326917 | 0.8454559 | NA | ZNF233 | 353355 |
| ENSG00000062370 | 83.97629855 | -0.070800012 | 0.1448261 | 0.2210659 | 0.4805935 | ZNF112 | 7771 |
| ENSG00000267508 | 44.32662257 | 0.011689091 | 0.1297868 | 0.7844162 | 0.9098848 | ZNF285 | 26974 |
| ENSG00000267242 | 30.19285855 | 0.017812521 | 0.1330126 | 0.6244886 | NA | NA | NA |
| ENSG00000167384 | 374.506675 | 0.095158934 | 0.1212118 | 0.2115622 | 0.4681851 | ZNF180 | 7733 |
| ENSG00000073008 | 2630.153063 | 0.125637587 | 0.0672572 | 0.033038 | 0.1466249 | PVR | 5817 |
| ENSG00000186567 | 25.76442429 | -0.005990516 | 0.1324469 | 0.8549484 | NA | CEACAM19 | 56971 |
| ENSG00000069399 | 333.5992273 | -0.741555131 | 0.1633595 | 3.16E-07 | 1.26E-05 | BCL3 | 602 |
| ENSG00000187244 | 127.7455371 | -0.067620843 | 0.1402905 | 0.258477 | 0.5253712 | BCAM | 4059 |
| ENSG00000130202 | 1201.026974 | -0.048443795 | 0.0764537 | 0.445523 | 0.6937435 | NECTIN2 | 5819 |
| ENSG00000130204 | 4122.223948 | 0.034239078 | 0.0591066 | 0.5437085 | 0.7661909 | TOMM40 | 10452 |
| ENSG00000130203 | 21.19273312 | 1.276122199 | 0.7950004 | 0.0026314 | NA | APOE | 348 |
| ENSG00000130208 | 33.21397089 | -0.022416647 | 0.1333329 | 0.5547512 | NA | APOC1 | 341 |
| ENSG00000104853 | 2184.841266 | -0.014921219 | 0.0642561 | 0.7918021 | 0.9129646 | CLPTM1 | 1209 |
| ENSG00000104856 | 1089.457703 | 0.059329989 | 0.0794573 | 0.3605421 | 0.6238323 | RELB | 5971 |
| ENSG00000104859 | 870.6452367 | -0.072496274 | 0.0882095 | 0.2922609 | 0.5617739 | CLASRP | 11129 |
| ENSG00000170684 | 136.8348651 | 0.201113977 | 0.2362438 | 0.0392797 | 0.1657608 | ZNF296 | 162979 |
| ENSG00000142252 | 422.6997695 | -0.077072447 | 0.1059689 | 0.2985103 | 0.5686822 | GEMIN7 | 79760 |
| ENSG00000007047 | 499.4419954 | -0.018790887 | 0.0952843 | 0.7827736 | 0.9096561 | MARK4 | 57787 |
| ENSG00000104866 | 699.2055197 | -0.030481842 | 0.0880961 | 0.6521374 | 0.8389068 | PPP1R37 | 284352 |
| ENSG00000007255 | 75.15242224 | 0.150926554 | 0.2364247 | 0.0387208 | 0.164233 | TRAPPC6A | 79090 |
| ENSG00000189114 | 269.6291148 | 0.340204044 | 0.1880733 | 0.0057748 | 0.0423185 | BLOC1S3 | 388552 |
| ENSG00000104892 | 209.7972476 | 0.021938046 | 0.1133615 | 0.7355908 | 0.8837227 | KLC3 | 147700 |
| ENSG00000104884 | 2067.686858 | -0.167633775 | 0.0767201 | 0.0105864 | 0.0658978 | ERCC2 | 2068 |
| ENSG00000104881 | 948.392371 | 0.064319365 | 0.0848732 | 0.3467939 | 0.6133079 | PPP1R13L | 10848 |
| ENSG00000117877 | 1338.365785 | 0.184897209 | 0.0859276 | 0.009687 | 0.062441 | POLR1G | 10849 |
| ENSG00000012061 | 4014.377347 | -0.063946141 | 0.0616572 | 0.2401569 | 0.5054546 | ERCC1 | 2067 |
| ENSG00000125740 | 129.2943106 | 1.14981176 | 0.2554097 | 3.31E-07 | 1.31E-05 | FOSB | 2354 |
| ENSG00000125744 | 197.1596271 | 0.037273058 | 0.1153121 | 0.5744433 | 0.7887248 | RTN2 | 6253 |
| ENSG00000213889 | 57.91652983 | 0.017232853 | 0.1283828 | 0.7161039 | 0.8714352 | PPM1N | 147699 |
| ENSG00000125753 | 4145.569052 | -0.081617198 | 0.0609833 | 0.1340026 | 0.3605814 | VASP | 7408 |
| ENSG00000125741 | 962.4011589 | 0.065974282 | 0.0840191 | 0.3307595 | 0.5988522 | OPA3 | 80207 |
| ENSG00000125746 | 281.5218878 | -0.08375745 | 0.1230293 | 0.2569902 | 0.5240334 | EML2 | 24139 |
| ENSG00000267757 | 26.25905203 | 0.0135692 | 0.132952 | 0.6932913 | NA | EML2-AS1 | 100287177 |
| ENSG00000010310 | 40.91079739 | 0.867940303 | 0.5588623 | 0.003086 | NA | GIPR | 2696 |
| ENSG00000125743 | 3458.387155 | -0.208764142 | 0.0662757 | 0.0004568 | 0.005969 | SNRPD2 | 6633 |
| ENSG00000011478 | 324.7372127 | -0.058716451 | 0.1091479 | 0.4080226 | 0.6640358 | QPCTL | 54814 |
| ENSG00000177051 | 429.5637187 | 0.019010599 | 0.0969193 | 0.7823063 | 0.9095461 | FBXO46 | 23403 |
| ENSG00000259605 | 15.88767394 | 0.001030019 | 0.133863 | 0.9717845 | NA | NA | NA |
| ENSG00000177045 | 431.8104396 | -0.255772396 | 0.1504854 | 0.0120876 | 0.072706 | SIX5 | 147912 |
| ENSG00000104936 | 391.8225715 | 0.098292767 | 0.1234525 | 0.2003835 | 0.4540225 | DMPK | 1760 |
| ENSG00000185800 | 786.7326872 | 0.252189021 | 0.1076355 | 0.0033558 | 0.0278477 | DMWD | 1762 |
| ENSG00000125755 | 2265.327136 | -0.007762339 | 0.063857 | 0.8984418 | 0.9598224 | SYMPK | 8189 |
| ENSG00000170604 | 375.1808023 | 0.406707558 | 0.1564627 | 0.000744 | 0.008841 | IRF2BP1 | 26145 |
| ENSG00000176182 | 137.7242526 | -0.054636097 | 0.1291329 | 0.3820639 | 0.6430282 | MYPOP | 339344 |
| ENSG00000104967 | 27.99883873 | -0.059416606 | 0.1499212 | 0.1173361 | NA | NOVA2 | 4858 |
| ENSG00000104983 | 118.0323006 | -0.0286999 | 0.1228214 | 0.6232189 | 0.8197072 | CCDC61 | 729440 |
| ENSG00000268621 | 53.91666313 | 0.080146244 | 0.1584215 | 0.1221198 | 0.3423225 | IGFL2-AS1 | 645553 |
| ENSG00000268460 | 14.12872689 | -0.003378386 | 0.1345357 | 0.885343 | NA | LOC93429 | 93429 |
| ENSG00000011485 | 2536.167649 | -0.030934489 | 0.0630947 | 0.5789751 | 0.7916677 | PPP5C | 5536 |
| ENSG00000160014 | 5697.894821 | -0.24462829 | 0.0676888 | 6.57E-05 | 0.0012371 | CALM3 | 808 |
| ENSG00000269292 | 105.9735489 | -0.070083497 | 0.1434576 | 0.2328231 | 0.4951943 | NA | NA |

| | | | | | | | | |
|---|---|---|---|---|---|---|---|---|
| ENSG00000197380 | 9.845465295 | -0.004942845 | 0.1350167 | 0.8174661 | NA | DACT3 | | 147906 |
| ENSG00000105287 | 765.3687346 | 0.022415347 | 0.0873037 | 0.7403463 | 0.8863406 | PRKD2 | | 25865 |
| ENSG00000090372 | 2706.990135 | 0.054625844 | 0.0655044 | 0.3427931 | 0.6092767 | STRN4 | | 29888 |
| ENSG00000269487 | 17.61283401 | -0.01449736 | 0.1347676 | 0.6022627 | NA | NA | NA | |
| ENSG00000181027 | 383.6901394 | 0.051844442 | 0.1032504 | 0.4643543 | 0.7082689 | FKRP | | 79147 |
| ENSG00000105281 | 4903.464849 | 0.409091564 | 0.0683689 | 2.33E-10 | 1.85E-08 | SLC1A5 | | 6510 |
| ENSG00000042753 | 2069.373331 | -0.10562061 | 0.074724 | 0.0946456 | 0.2918558 | AP2S1 | | 1175 |
| ENSG00000160007 | 2753.350931 | -0.200120098 | 0.0802332 | 0.0035047 | 0.0287286 | ARHGAP35 | | 2909 |
| ENSG00000130751 | 15.95368963 | 0.05335298 | 0.1496015 | 0.0534455 | NA | NPAS1 | | 4861 |
| ENSG00000130748 | 499.3259217 | 0.03142697 | 0.093907 | 0.6510769 | 0.8381342 | TMEM160 | | 54958 |
| ENSG00000130749 | 1447.567781 | -0.073323553 | 0.0760258 | 0.248389 | 0.5148194 | ZC3H4 | | 23211 |
| ENSG00000142230 | 5070.839267 | -0.010889775 | 0.0548942 | 0.8275835 | 0.9288449 | SAE1 | | 10055 |
| ENSG00000105327 | 130.4641014 | 0.050539892 | 0.1272981 | 0.4223017 | 0.6742808 | BBC3 | | 27113 |
| ENSG00000105321 | 823.2712731 | 0.198066846 | 0.1048224 | 0.0148923 | 0.083754 | CCDC9 | | 26093 |
| ENSG00000257704 | 131.9787577 | 0.468746776 | 0.2991391 | 0.0050016 | 0.0380117 | INAFM1 | | 255783 |
| ENSG00000134815 | 879.2310222 | 0.209578429 | 0.1019611 | 0.0094285 | 0.0614244 | DHX34 | | 9704 |
| ENSG00000105419 | 134.7742055 | -0.017625924 | 0.1186495 | 0.7688899 | 0.9009387 | MEIS3 | | 56917 |
| ENSG00000118162 | 575.4967553 | 0.000663083 | 0.0893846 | 0.9931095 | 0.9973787 | KPTN | | 11133 |
| ENSG00000268061 | 17.43864255 | 0.014235185 | 0.1345482 | 0.6212668 | NA | NA | NA | |
| ENSG00000105402 | 1357.296706 | 0.045318158 | 0.0738817 | 0.4677492 | 0.7102273 | NAPA | | 8775 |
| ENSG00000268746 | 8.835053347 | -0.012420588 | 0.135995 | 0.532654 | NA | NA | NA | |
| ENSG00000063169 | 379.1650313 | -0.077471137 | 0.1096807 | 0.2921149 | 0.5617705 | BICRA | | 29998 |
| ENSG00000024422 | 2105.533354 | -0.020344505 | 0.0689662 | 0.7318871 | 0.8810855 | EHD2 | | 30846 |
| ENSG00000105373 | 2294.661646 | 0.103810868 | 0.0689282 | 0.0828559 | 0.268546 | NOP53 | | 29997 |
| ENSG00000178980 | 1328.168596 | 0.121521189 | 0.0856307 | 0.079312 | 0.260603 | SELENOW | | 6415 |
| ENSG00000105499 | 33.23991402 | 0.102990762 | 0.1892103 | 0.0281301 | NA | PLA2G4C | | 8605 |
| ENSG00000105486 | 1693.937181 | -0.128870259 | 0.0772044 | 0.0473248 | 0.1859327 | LIG1 | | 3978 |
| ENSG00000269534 | 10.24453793 | 0.00083111 | 0.1348424 | 0.9722355 | NA | NA | NA | |
| ENSG00000185453 | 311.140832 | 0.107127453 | 0.1256228 | 0.1708025 | 0.4168708 | ZSWIM9 | | 374920 |
| ENSG00000178150 | 334.3541794 | 0.060288101 | 0.115334 | 0.394224 | 0.6548537 | ZNF114 | | 163071 |
| ENSG00000105483 | 304.8340462 | -0.12785495 | 0.1337242 | 0.1178736 | 0.3345134 | CARD8 | | 22900 |
| ENSG00000268001 | 36.5425868 | -0.046784352 | 0.1412321 | 0.2498284 | NA | CARD8-AS1 | | 100505812 |
| ENSG00000142227 | 1141.385713 | 0.014793912 | 0.080811 | 0.8213051 | 0.9266556 | EMP3 | | 2014 |
| ENSG00000161558 | 59.74839017 | 0.039748012 | 0.1338416 | 0.418073 | 0.6710348 | TMEM143 | | 55260 |
| ENSG00000105438 | 2182.155738 | -0.035212815 | 0.0652286 | 0.5387368 | 0.762324 | KDELR1 | | 10945 |
| ENSG00000105464 | 19.13525517 | -0.003533787 | 0.133285 | 0.9031427 | NA | GRIN2D | | 2906 |
| ENSG00000105447 | 2899.159398 | -0.00365991 | 0.0604505 | 0.9322592 | 0.9737744 | GRWD1 | | 83743 |
| ENSG00000182324 | 96.68313458 | 1.341585544 | 0.2962095 | 2.87E-07 | 1.16E-05 | KCNJ14 | | 3770 |
| ENSG00000269423 | 12.42831819 | 0.055631913 | 0.1517034 | 0.0265962 | NA | NA | NA | |
| ENSG00000105443 | 796.5854191 | -0.071682149 | 0.0939098 | 0.3100591 | 0.5796784 | CYTH2 | | 9266 |
| ENSG00000142235 | 20.87618792 | -0.016756896 | 0.1344697 | 0.5834426 | NA | LMTK3 | | 114783 |
| ENSG00000063177 | 9612.614671 | 0.001418319 | 0.0572159 | 0.9804099 | 0.9927719 | RPL18 | | 6141 |
| ENSG00000063176 | 399.6755262 | -0.056632675 | 0.1127047 | 0.4209671 | 0.6735758 | SPHK2 | | 56848 |
| ENSG00000105516 | 45.7067072 | 0.027307417 | 0.1333319 | 0.5104421 | 0.7416935 | DBP | | 1628 |
| ENSG00000063180 | 61.90139739 | -0.011683839 | 0.1276564 | 0.8018541 | 0.9172909 | CA11 | | 770 |
| ENSG00000176909 | 22.56594864 | 0.011724706 | 0.1334788 | 0.7119695 | NA | MAMSTR | | 284358 |
| ENSG00000105538 | 16.32405689 | 0.023225913 | 0.1367544 | 0.3913965 | NA | RASIP1 | | 54922 |
| ENSG00000174951 | 12.71676501 | -0.006322398 | 0.1345944 | 0.7940637 | NA | FUT1 | | 2523 |
| ENSG00000105552 | 582.8524199 | -0.084706888 | 0.0992968 | 0.2419689 | 0.5077131 | BCAT2 | | 587 |
| ENSG00000087076 | 85.37835751 | 0.056084308 | 0.1364169 | 0.3214395 | 0.590367 | HSD17B14 | | 51171 |
| ENSG00000105559 | 16.73223166 | 0.01938122 | 0.1355547 | 0.497015 | NA | PLEKHA4 | | 57664 |
| ENSG00000087074 | 1886.416067 | 1.214865045 | 0.2178966 | 1.21E-09 | 8.69E-08 | PPP1R15A | | 23645 |
| ENSG00000104805 | 1885.388566 | 0.008751614 | 0.067146 | 0.8822976 | 0.9525622 | NUCB1 | | 4924 |
| ENSG00000235191 | 19.56460366 | -0.00121879 | 0.133322 | 0.9647082 | NA | NUCB1-AS1 | | 100874085 |
| ENSG00000087088 | 1770.237865 | -0.077344709 | 0.0715225 | 0.2055552 | 0.4607272 | BAX | | 581 |
| ENSG00000087086 | 10082.23857 | 0.171973544 | 0.0661816 | 0.0035583 | 0.0291067 | FTL | | 2512 |
| ENSG00000104812 | 884.4038294 | 0.01303655 | 0.0805959 | 0.842128 | 0.9350382 | GYS1 | | 2997 |
| ENSG00000183207 | 3104.505665 | -0.060271359 | 0.0644823 | 0.288134 | 0.5578243 | RUVBL2 | | 10856 |
| ENSG00000104852 | 6393.397635 | 0.026539597 | 0.0527165 | 0.5866061 | 0.7971319 | SNRNP70 | | 6625 |
| ENSG00000104863 | 64.26766444 | 0.014425726 | 0.1271004 | 0.7683491 | 0.9006609 | LIN7B | | 64130 |
| ENSG00000221916 | 10.82583707 | -0.020514817 | 0.1370415 | 0.3587518 | NA | C19orf73 | | 55150 |
| ENSG00000177380 | 550.5686835 | 0.007397954 | 0.0905536 | 0.9140099 | 0.9662036 | PPFIA3 | | 8541 |
| ENSG00000130529 | 360.3101928 | 0.049271924 | 0.1095482 | 0.4824187 | 0.7213706 | TRPM4 | | 54795 |
| ENSG00000063127 | 52.80926864 | 0.011756047 | 0.1285685 | 0.7962872 | 0.9152729 | SLC6A16 | | 28968 |
| ENSG00000197813 | 44.17523703 | -0.066358577 | 0.1504073 | 0.1544939 | 0.3927351 | NA | NA | |
| ENSG00000104894 | 11.14819172 | -0.015987025 | 0.1361567 | 0.4729397 | NA | CD37 | | 951 |
| ENSG00000074219 | 495.0722849 | -0.298101455 | 0.1336413 | 0.0031091 | 0.026241 | TEAD2 | | 8463 |
| ENSG00000104901 | 15.51263347 | 0.041738961 | 0.1431643 | 0.1353699 | NA | DKKL1 | | 27120 |
| ENSG00000104888 | 60.60368223 | -0.060654758 | 0.1435825 | 0.2334418 | 0.4958342 | SLC17A7 | | 57030 |
| ENSG00000104872 | 1071.54833 | -0.09679523 | 0.0874735 | 0.1616743 | 0.4037019 | PIH1D1 | | 55011 |

| | | | | | | | |
|---|---|---|---|---|---|---|---|
| ENSG00000161618 | 265.118991 | 0.110178506 | 0.1346255 | 0.1602389 | 0.4013186 | ALDH16A1 | 126133 |
| ENSG00000142541 | 25614.53953 | 0.006491503 | 0.0526848 | 0.8286975 | 0.929341 | RPL13A | 23521 |
| ENSG00000142534 | 12332.71572 | -0.128976897 | 0.0558255 | 0.0116935 | 0.0710673 | RPS11 | 6205 |
| ENSG00000104870 | 52.08357775 | 0.030296027 | 0.1325588 | 0.50217 | 0.7365601 | FCGRT | 2217 |
| ENSG00000142546 | 2053.103408 | 0.08979558 | 0.0739439 | 0.1556462 | 0.3943696 | NOSIP | 51070 |
| ENSG00000126460 | 15.86810518 | 0.036824074 | 0.1410037 | 0.1889133 | NA | PRRG2 | 5639 |
| ENSG00000126464 | 755.9761642 | -0.099007928 | 0.0950279 | 0.1703698 | 0.4162267 | PRR12 | 57479 |
| ENSG00000126458 | 474.3891898 | -0.027719762 | 0.0991509 | 0.686874 | 0.857187 | RRAS | 6237 |
| ENSG00000126461 | 2483.013311 | 0.087567401 | 0.0670789 | 0.137332 | 0.3655822 | SCAF1 | 58506 |
| ENSG00000126456 | 1734.562577 | -0.059092419 | 0.0711336 | 0.3310702 | 0.5989207 | IRF3 | 3661 |
| ENSG00000126453 | 1337.787414 | -0.062194087 | 0.0758314 | 0.3261666 | 0.5942836 | BCL2L12 | 83596 |
| ENSG00000126457 | 6770.981986 | -0.205368455 | 0.0592791 | 0.0001568 | 0.0025129 | PRMT1 | 3276 |
| ENSG00000224420 | 70.95822054 | -0.035586954 | 0.1308889 | 0.4853166 | 0.7234177 | ADM5 | 199800 |
| ENSG00000196961 | 1888.931961 | -0.022054554 | 0.0672693 | 0.7058237 | 0.8670449 | AP2A1 | 160 |
| ENSG00000010361 | 117.5255494 | 0.032904605 | 0.1245051 | 0.5710972 | 0.786302 | FUZ | 80199 |
| ENSG00000104973 | 904.8136693 | -0.092731037 | 0.0925746 | 0.192141 | 0.4433917 | MED25 | 81857 |
| ENSG00000268006 | 17.91957268 | -0.005622468 | 0.1337467 | 0.8352261 | NA | PTOV1-AS1 | 100506033 |
| ENSG00000104960 | 1576.758078 | -0.307474801 | 0.0873218 | 6.15E-05 | 0.0011757 | PTOV1 | 53635 |
| ENSG00000269352 | 208.8770253 | 0.011763572 | 0.1115526 | 0.8569542 | 0.9422695 | NA | NA |
| ENSG00000267896 | 119.2408783 | -0.000466094 | 0.1204288 | 0.9940188 | 0.9977151 | NA | NA |
| ENSG00000039650 | 784.8409418 | -0.075088002 | 0.0888072 | 0.2772935 | 0.5460273 | PNKP | 11284 |
| ENSG00000204673 | 2486.506462 | 0.214754736 | 0.0779821 | 0.0015189 | 0.0155358 | AKT1S1 | 84335 |
| ENSG00000104946 | 555.982074 | -0.082871529 | 0.1001347 | 0.2536128 | 0.5207332 | TBC1D17 | 79735 |
| ENSG00000213024 | 2217.906475 | 0.150578373 | 0.070782 | 0.0145168 | 0.0823156 | NUP62 | 23636 |
| ENSG00000169136 | 1222.861536 | -0.310966915 | 0.0926918 | 0.0001089 | 0.001873 | ATF5 | 22809 |
| ENSG00000105053 | 537.1589944 | -0.151637623 | 0.1157126 | 0.0637758 | 0.226985 | VRK3 | 51231 |
| ENSG00000142528 | 922.6869433 | 0.569250833 | 0.1027292 | 2.22E-09 | 1.55E-07 | ZNF473 | 25888 |
| ENSG00000131408 | 1013.22392 | -0.013234926 | 0.0777758 | 0.8353258 | 0.931409 | NR1H2 | 7376 |
| ENSG00000062822 | 2081.177226 | -0.126261554 | 0.0756421 | 0.0486853 | 0.1890888 | POLD1 | 5424 |
| ENSG00000161671 | 1340.1001 | 0.014169319 | 0.0733276 | 0.8199731 | 0.9259577 | EMC10 | 284361 |
| ENSG00000161677 | 376.4523909 | 0.188266375 | 0.158416 | 0.0446394 | 0.1798046 | JOSD2 | 126119 |
| ENSG00000161681 | 670.9558577 | 0.11119998 | 0.1028171 | 0.1454624 | 0.3780081 | SHANK1 | 50944 |
| ENSG00000105472 | 33.69792479 | 0.020243764 | 0.1328265 | 0.5973642 | NA | CLEC11A | 6320 |
| ENSG00000167747 | 2502.070514 | 0.332511791 | 0.0744269 | 1.08E-06 | 3.60E-05 | C19orf48P | 84798 |
| ENSG00000142544 | 385.1580019 | 0.496571679 | 0.150071 | 6.55E-05 | 0.001236 | CTU1 | 90353 |
| ENSG00000186806 | 44.39816275 | 0.021765265 | 0.1313078 | 0.6129718 | 0.8148077 | VSIG10L | 147645 |
| ENSG00000267984 | 8.206592871 | 0.008652293 | 0.1356199 | 0.6623364 | NA | NA | NA |
| ENSG00000105379 | 329.5822934 | 0.067649363 | 0.1131628 | 0.348883 | 0.6148358 | ETFB | 2109 |
| ENSG00000160318 | 9.028500153 | 0.011573306 | 0.1357125 | 0.5853292 | NA | CLDND2 | 125875 |
| ENSG00000255441 | 9.017566009 | 0.028011076 | 0.1394175 | 0.1676997 | NA | NA | NA |
| ENSG00000105497 | 200.5006625 | 0.028380308 | 0.1130729 | 0.6677347 | 0.8470687 | ZNF175 | 7728 |
| ENSG00000182310 | 63.99952162 | -0.049997392 | 0.1378186 | 0.3189577 | 0.5875643 | SPACA6 | 147650 |
| ENSG00000161551 | 51.52100473 | 0.077168292 | 0.1563174 | 0.1303057 | 0.3547836 | ZNF577 | 84765 |
| ENSG00000198093 | 62.10452988 | 0.067287398 | 0.1468503 | 0.2029345 | 0.4573563 | ZNF649 | 65251 |
| ENSG00000176024 | 135.0102639 | -0.028486811 | 0.1201023 | 0.6411632 | 0.8315061 | ZNF613 | 79898 |
| ENSG00000269235 | 14.08278935 | -0.008976078 | 0.1344869 | 0.7285008 | NA | ZNF350-AS1 | 101669766 |
| ENSG00000256683 | 176.3570456 | 0.704101264 | 0.2388695 | 0.0001552 | 0.0024943 | ZNF350 | 59348 |
| ENSG00000197619 | 295.7198267 | 0.320124311 | 0.1827251 | 0.0071262 | 0.0494307 | ZNF615 | 284370 |
| ENSG00000142556 | 585.4254538 | 0.080377561 | 0.107612 | 0.2760435 | 0.5443052 | ZNF614 | 80110 |
| ENSG00000256087 | 373.5246721 | 0.058248021 | 0.1061537 | 0.4156436 | 0.6697358 | ZNF432 | 9668 |
| ENSG00000260160 | 18.11549679 | 0.006829922 | 0.1336854 | 0.8147127 | NA | NA | NA |
| ENSG00000197608 | 378.8573189 | 0.433918288 | 0.1537736 | 0.0003632 | 0.0050383 | ZNF841 | 284371 |
| ENSG00000204611 | 283.0137283 | 0.050198753 | 0.1113446 | 0.4727661 | 0.7131174 | ZNF616 | 90317 |
| ENSG00000196267 | 99.02699309 | 0.081057539 | 0.1474398 | 0.1995007 | 0.4530837 | ZNF836 | 162962 |
| ENSG00000105568 | 5045.146336 | -0.103997968 | 0.0573312 | 0.045221 | 0.1812453 | PPP2R1A | 5518 |
| ENSG00000268015 | 10.4335472 | 0.006898194 | 0.1350495 | 0.7589355 | NA | NA | NA |
| ENSG00000196214 | 392.7091905 | 0.53363254 | 0.1526629 | 3.15E-05 | 0.0006684 | ZNF766 | 90321 |
| ENSG00000198464 | 787.6059973 | 0.080770175 | 0.0907766 | 0.2496497 | 0.515769 | ZNF480 | 147657 |
| ENSG00000167554 | 33.17453116 | 0.068188427 | 0.1552495 | 0.0922014 | NA | ZNF610 | 162963 |
| ENSG00000221923 | 69.39636089 | 0.059183774 | 0.1406972 | 0.2709116 | 0.5386338 | ZNF880 | 400713 |
| ENSG00000269834 | 77.20553178 | 0.157948165 | 0.239326 | 0.0408253 | 0.1698292 | ZNF528-AS1 | 102724105 |
| ENSG00000167555 | 273.7596487 | 0.416440413 | 0.182996 | 0.0016031 | 0.0161017 | ZNF528 | 84436 |
| ENSG00000198482 | 278.5008272 | 0.074528515 | 0.1193077 | 0.2998508 | 0.569614 | ZNF808 | 388558 |
| ENSG00000167562 | 440.0684891 | 0.16596469 | 0.1323234 | 0.0566882 | 0.2101474 | ZNF701 | 55762 |
| ENSG00000123870 | 118.9920956 | 0.096631937 | 0.1549578 | 0.1547619 | 0.3931652 | NA | NA |
| ENSG00000167766 | 724.421382 | -0.000802504 | 0.0851308 | 0.9894941 | 0.9955163 | ZNF83 | 55769 |
| ENSG00000269825 | 502.5975399 | 0.084290031 | 0.1054595 | 0.2543595 | 0.5211459 | LOC122539 | 122539214 |
| ENSG00000213020 | 567.8136434 | 0.18403256 | 0.1186922 | 0.0313125 | 0.1420431 | ZNF611 | 81856 |
| ENSG00000189190 | 469.7562951 | 0.232635379 | 0.1408989 | 0.0161952 | 0.0888425 | ZNF600 | 162966 |
| ENSG00000198538 | 670.4258935 | 0.151150689 | 0.1061429 | 0.0537029 | 0.2023558 | ZNF28 | 7576 |

| | | | | | | | | |
|---|---|---|---|---|---|---|---|---|
| ENSG00000204604 | 766.3559076 | 0.176633355 | 0.1059165 | 0.0281053 | 0.1320054 | ZNF468 | | 90333 |
| ENSG00000268225 | 118.6770924 | -0.006236139 | 0.1214107 | 0.9099793 | 0.9646491 | NA | NA | |
| ENSG00000182986 | 3417.502082 | -0.062915409 | 0.1139081 | 0.3765877 | 0.6384862 | ZNF320 | | 162967 |
| ENSG00000213793 | 99.42092255 | -0.043051239 | 0.1309533 | 0.4337299 | 0.684513 | ZNF888 | | 388559 |
| ENSG00000180257 | 246.5745637 | 0.326829469 | 0.2205784 | 0.0101205 | 0.0641442 | ZNF816 | | 125893 |
| ENSG00000242779 | 1176.998765 | -0.282225056 | 0.102865 | 0.0009214 | 0.0103584 | ZNF702P | | 79986 |
| ENSG00000170949 | 574.3880418 | 0.198500459 | 0.1236658 | 0.0245574 | 0.1193242 | ZNF160 | | 90338 |
| ENSG00000197937 | 669.5616735 | 0.071211584 | 0.0926411 | 0.3128072 | 0.5819816 | ZNF347 | | 84671 |
| ENSG00000268613 | 13.12744171 | 0.01175583 | 0.13508 | 0.6349473 | NA | NA | NA | |
| ENSG00000197497 | 123.1897677 | 0.017903294 | 0.1205452 | 0.7606066 | 0.8972577 | ZNF665 | | 79788 |
| ENSG00000269051 | 47.48396337 | 0.034032351 | 0.1347907 | 0.4303561 | 0.6816257 | NA | NA | |
| ENSG00000269001 | 42.62117674 | 0.07366461 | 0.1561271 | 0.1167886 | 0.3335472 | NA | NA | |
| ENSG00000267809 | 12.82366387 | 0.013004198 | 0.1351992 | 0.6038439 | NA | NA | NA | |
| ENSG00000197928 | 220.3783412 | 0.034473005 | 0.1141602 | 0.6037291 | 0.8090122 | ZNF677 | | 342926 |
| ENSG00000213799 | 564.0534494 | 0.415927957 | 0.1372004 | 0.0002057 | 0.003132 | ZNF845 | | 91664 |
| ENSG00000203326 | 634.6237719 | 0.657219724 | 0.1240101 | 7.49E-09 | 4.66E-07 | ZNF525 | | 170958 |
| ENSG00000196417 | 346.3408196 | 0.221108233 | 0.1540185 | 0.0245682 | 0.119327 | ZNF765 | | 91661 |
| ENSG00000239912 | 9.676397323 | -0.018749298 | 0.1367989 | 0.3852085 | NA | NA | NA | |
| ENSG00000160336 | 661.5544439 | 0.378540683 | 0.1162235 | 0.0001124 | 0.001923 | ZNF761 | | 388561 |
| ENSG00000241015 | 347.4288626 | 0.122932938 | 0.1266363 | 0.1279504 | 0.3515852 | TPM3P9 | | 147804 |
| ENSG00000198346 | 423.6023423 | 0.045595678 | 0.1010603 | 0.5163077 | 0.7456659 | ZNF813 | | 126017 |
| ENSG00000250731 | 9.851884155 | 0.020649422 | 0.1372854 | 0.3331679 | NA | NA | NA | |
| ENSG00000130844 | 632.7811543 | 0.18871981 | 0.1167956 | 0.0269479 | 0.1276472 | ZNF331 | | 55422 |
| ENSG00000179820 | 2697.317949 | 0.072764652 | 0.068995 | 0.2223611 | 0.4821607 | MYADM | | 91663 |
| ENSG00000232220 | 31.32341549 | -0.009527946 | 0.1318239 | 0.790851 | NA | NA | NA | |
| ENSG00000126583 | 9.272904386 | -0.001868938 | 0.1349834 | 0.9275249 | NA | PRKCG | | 5582 |
| ENSG00000105605 | 205.9812764 | -0.025223281 | 0.1128438 | 0.6988822 | 0.8629314 | CACNG7 | | 59284 |
| ENSG00000142408 | 88.20078228 | -0.039256175 | 0.129824 | 0.4716509 | 0.712171 | CACNG8 | | 59283 |
| ENSG00000189068 | 256.3663935 | 0.001320489 | 0.106149 | 0.9855165 | 0.9940394 | VSTM1 | | 284415 |
| ENSG00000170909 | 75.23080569 | 0.061375545 | 0.1418752 | 0.2561179 | 0.5231846 | OSCAR | | 126014 |
| ENSG00000170906 | 561.7950343 | -0.082695511 | 0.0989637 | 0.2541948 | 0.5211409 | NDUFA3 | | 4696 |
| ENSG00000105619 | 566.8144359 | 0.024953352 | 0.0905126 | 0.7149966 | 0.8711099 | TFPT | | 29844 |
| ENSG00000105618 | 2387.496707 | -0.280486831 | 0.0781939 | 5.70E-05 | 0.0011054 | PRPF31 | | 26121 |
| ENSG00000237017 | 80.00059965 | -0.04363202 | 0.1321796 | 0.4173701 | 0.6707898 | NA | NA | |
| ENSG00000088038 | 1477.191736 | -0.086948377 | 0.0795249 | 0.1841345 | 0.4343447 | CNOT3 | | 4849 |
| ENSG00000105617 | 241.2815838 | 0.076827011 | 0.1253045 | 0.286246 | 0.5561508 | LENG1 | | 79165 |
| ENSG00000125505 | 1978.679239 | 0.012233931 | 0.0659451 | 0.8283387 | 0.9292062 | MBOAT7 | | 79143 |
| ENSG00000170892 | 1702.79988 | -0.057610476 | 0.0717443 | 0.3352267 | 0.602892 | TSEN34 | | 79042 |
| ENSG00000268816 | 16.4539196 | -0.029553239 | 0.1383141 | 0.2912191 | NA | NA | NA | |
| ENSG00000170889 | 9313.913556 | -0.087371196 | 0.0561379 | 0.0869155 | 0.2767899 | RPS9 | | 6203 |
| ENSG00000226696 | 43.29492946 | 0.058255001 | 0.1457502 | 0.1966285 | 0.4494065 | LENG8-AS1 | | 104355426 |
| ENSG00000167615 | 2621.400621 | -0.041101244 | 0.0668567 | 0.4800441 | 0.7191734 | LENG8 | | 114823 |
| ENSG00000182909 | 19.10707163 | 0.002445097 | 0.13329 | 0.9358685 | NA | NA | NA | |
| ENSG00000160439 | 479.6810148 | 0.233296199 | 0.131703 | 0.0131911 | 0.0772001 | RDH13 | | 112724 |
| ENSG00000131037 | 21.57248747 | -0.009871849 | 0.1339307 | 0.7312512 | NA | EPS8L1 | | 54869 |
| ENSG00000125503 | 1906.416797 | 0.149568198 | 0.0753111 | 0.0201682 | 0.104472 | PPP1R12C | | 54776 |
| ENSG00000105048 | 794.9504491 | -0.038223603 | 0.0841409 | 0.5650836 | 0.7820725 | TNNT1 | | 7138 |
| ENSG00000167646 | 71.813193 | 0.072819266 | 0.1481183 | 0.1961648 | 0.4489421 | DNAAF3 | | 352909 |
| ENSG00000080031 | 238.3968279 | 0.762648401 | 0.1846009 | 1.97E-06 | 6.13E-05 | PTPRH | | 5794 |
| ENSG00000180089 | 87.3373171 | -0.018940559 | 0.1245242 | 0.7237491 | 0.875711 | TMEM86B | | 255043 |
| ENSG00000269808 | 15.06170165 | 0.007429282 | 0.134388 | 0.7762697 | NA | NA | NA | |
| ENSG00000105063 | 3066.478582 | -0.056401256 | 0.0625567 | 0.309581 | 0.5790036 | PPP6R1 | | 22870 |
| ENSG00000133265 | 1409.244629 | 0.048006226 | 0.0735436 | 0.4405821 | 0.6895939 | HSPBP1 | | 23640 |
| ENSG00000160469 | 101.6736892 | 0.084436776 | 0.1514765 | 0.1761874 | 0.425128 | BRSK1 | | 84446 |
| ENSG00000133247 | 139.5310692 | -0.016305956 | 0.1179048 | 0.7871985 | 0.9115956 | KMT5C | | 84787 |
| ENSG00000095752 | 10.234228 | 0.026180909 | 0.1390364 | 0.1773298 | NA | IL11 | | 3589 |
| ENSG00000233493 | 51.14188201 | 0.087337731 | 0.1649848 | 0.0961151 | 0.2946 | TMEM238 | | 388564 |
| ENSG00000108107 | 6193.67325 | 0.079460925 | 0.0591008 | 0.1349025 | 0.3616363 | RPL28 | | 6158 |
| ENSG00000108106 | 5880.439759 | -0.078623449 | 0.0572058 | 0.1252833 | 0.347221 | UBE2S | | 27338 |
| ENSG00000063241 | 576.1616109 | -0.06588628 | 0.0960236 | 0.3513295 | 0.6171375 | ISOC2 | | 79763 |
| ENSG00000197483 | 207.4656767 | 0.055202466 | 0.1203609 | 0.4137718 | 0.6680399 | ZNF628 | | 89887 |
| ENSG00000090971 | 581.0091318 | 0.022385007 | 0.0918798 | 0.743883 | 0.8886411 | NAT14 | | 57106 |
| ENSG00000179954 | 16.8514464 | 0.054244586 | 0.1495626 | 0.0670771 | NA | SSC5D | | 284297 |
| ENSG00000231274 | 22.22391301 | 0.023403962 | 0.1355081 | 0.4662314 | NA | SBK3 | | 100130827 |
| ENSG00000218891 | 374.6808649 | 0.072509587 | 0.1124821 | 0.3203934 | 0.5890941 | ZNF579 | | 163033 |
| ENSG00000179943 | 372.7515558 | 0.040872326 | 0.1041893 | 0.5589071 | 0.7776292 | FIZ1 | | 84922 |
| ENSG00000171443 | 162.2664509 | 0.121875314 | 0.1609214 | 0.1171553 | 0.3339413 | ZNF524 | | 147807 |
| ENSG00000261221 | 535.8670625 | 0.02173663 | 0.0944812 | 0.7588228 | 0.8963893 | ZNF865 | | 100507290 |
| ENSG00000179922 | 137.8821804 | -0.01384488 | 0.1178571 | 0.8181147 | 0.9254567 | ZNF784 | | 147808 |
| ENSG00000213015 | 336.8552205 | 0.063481563 | 0.1090579 | 0.3790378 | 0.6403732 | ZNF580 | | 51157 |

| | | | | | | | | |
|---|---|---|---|---|---|---|---|---|
| ENSG00000171425 | 252.535417 | 0.129206226 | 0.151628 | 0.1138329 | 0.3279927 | ZNF581 | | 51545 |
| ENSG00000173581 | 456.7890147 | -0.210495133 | 0.1357902 | 0.0232817 | 0.1150885 | CCDC106 | | 29903 |
| ENSG00000063244 | 8425.841901 | 0.072524714 | 0.0556485 | 0.1531825 | 0.3908224 | U2AF2 | | 11338 |
| ENSG00000267096 | 16.35099939 | -0.016903842 | 0.1351856 | 0.5417459 | NA | NA | NA | |
| ENSG00000063245 | 2277.463131 | -0.015799924 | 0.0663241 | 0.7845513 | 0.9098848 | EPN1 | | 29924 |
| ENSG00000267183 | 13.13284274 | 0.016032922 | 0.1357951 | 0.51226 | NA | NA | NA | |
| ENSG00000142409 | 991.0872148 | 0.070383364 | 0.0829588 | 0.2939424 | 0.5639652 | ZNF787 | | 126208 |
| ENSG00000167685 | 513.9191718 | 0.16840226 | 0.12135 | 0.0471553 | 0.1857119 | ZNF444 | | 55311 |
| ENSG00000131848 | 151.6064238 | -0.056376353 | 0.1280306 | 0.3775298 | 0.6392414 | ZSCAN5A | | 79149 |
| ENSG00000197951 | 85.23064461 | -0.002606839 | 0.1236192 | 0.9589169 | 0.9831259 | ZNF71 | | 58491 |
| ENSG00000083844 | 231.9923107 | 0.059547895 | 0.1183958 | 0.3953219 | 0.6555586 | ZNF264 | | 9422 |
| ENSG00000105146 | 15.69459967 | 0.003964358 | 0.1340181 | 0.8838238 | NA | AURKC | | 6795 |
| ENSG00000204524 | 96.87377869 | 0.034374456 | 0.1266818 | 0.5428438 | 0.7657126 | ZNF805 | | 390980 |
| ENSG00000268713 | 31.483633 | 0.001780882 | 0.1313368 | 0.9639202 | NA | NA | NA | |
| ENSG00000197714 | 206.8912244 | -0.005252445 | 0.1132077 | 0.9332229 | 0.9743454 | ZNF460 | | 10794 |
| ENSG00000268205 | 286.0106987 | 0.086400883 | 0.1214757 | 0.2564904 | 0.5233301 | NA | NA | |
| ENSG00000178229 | 152.9554416 | 0.099653428 | 0.1471835 | 0.1715392 | 0.4177334 | ZNF543 | | 125919 |
| ENSG00000131845 | 430.7501714 | 0.355356918 | 0.1526624 | 0.0018189 | 0.017632 | ZNF304 | | 57343 |
| ENSG00000152433 | 50.7316063 | 0.063694077 | 0.147295 | 0.190216 | 0.4411132 | ZNF547 | | 284306 |
| ENSG00000256060 | 118.0671805 | 0.024059061 | 0.1213597 | 0.6837049 | 0.8549799 | TRAPPC2B | | 10597 |
| ENSG00000188785 | 192.581596 | 0.063619071 | 0.1235035 | 0.3568914 | 0.6201207 | ZNF548 | | 147694 |
| ENSG00000186272 | 91.87819194 | 0.016302675 | 0.1236208 | 0.7663589 | 0.8994294 | ZNF17 | | 7565 |
| ENSG00000186230 | 234.9275079 | 0.050823731 | 0.115598 | 0.4625773 | 0.7068767 | ZNF749 | | 388567 |
| ENSG00000197128 | 229.9064099 | 0.049771849 | 0.1171061 | 0.4656267 | 0.7090416 | ZNF772 | | 400720 |
| ENSG00000105136 | 185.7723052 | 0.175878195 | 0.1918386 | 0.0560509 | 0.208314 | ZNF419 | | 79744 |
| ENSG00000152439 | 201.2686932 | 0.884759186 | 0.2100052 | 1.31E-06 | 4.24E-05 | ZNF773 | | 374928 |
| ENSG00000121406 | 363.4538606 | 0.142212932 | 0.1315537 | 0.0900652 | 0.2833427 | ZNF549 | | 256051 |
| ENSG00000251369 | 281.6431724 | 0.717237995 | 0.1792303 | 3.48E-06 | 0.0001015 | ZNF550 | | 162972 |
| ENSG00000083817 | 155.2998737 | 0.115863458 | 0.1570086 | 0.1290355 | 0.3534858 | ZNF416 | | 55659 |
| ENSG00000171649 | 179.5231679 | 0.109662662 | 0.1479533 | 0.1514353 | 0.3880053 | ZIK1 | | 284307 |
| ENSG00000183647 | 257.2139257 | 0.074047515 | 0.1212343 | 0.3052421 | 0.5743305 | ZNF530 | | 348327 |
| ENSG00000213762 | 532.4411671 | 0.079458765 | 0.1079026 | 0.2810675 | 0.5500335 | ZNF134 | | 7693 |
| ENSG00000121417 | 275.7097628 | 0.010722248 | 0.1076631 | 0.8732057 | 0.9487142 | ZNF211 | | 10520 |
| ENSG00000204519 | 459.5879522 | 0.056476782 | 0.0994321 | 0.4267632 | 0.6787658 | ZNF551 | | 90233 |
| ENSG00000152443 | 369.8505676 | -0.003065166 | 0.1011797 | 0.9617008 | 0.9843697 | ZNF776 | | 284309 |
| ENSG00000083828 | 162.3878716 | -0.015612243 | 0.1159167 | 0.8008842 | 0.9169162 | ZNF586 | | 54807 |
| ENSG00000178935 | 105.5444951 | 0.08677029 | 0.1504027 | 0.180026 | 0.429519 | ZNF552 | | 79818 |
| ENSG00000269343 | 339.7675381 | -0.070955997 | 0.1149752 | 0.3274633 | 0.5955314 | ZNF587B | | 100293516 |
| ENSG00000204514 | 64.56369341 | 0.0793291 | 0.1551158 | 0.148037 | 0.3820469 | ZNF814 | | 730051 |
| ENSG00000198466 | 976.13218 | 0.03757572 | 0.0828069 | 0.5710505 | 0.786302 | ZNF587 | | 84914 |
| ENSG00000270804 | 73.94745068 | 0.091079871 | 0.1628613 | 0.1204121 | 0.3390055 | NA | NA | |
| ENSG00000173480 | 291.0302077 | 0.062483425 | 0.1144937 | 0.3803484 | 0.6413815 | ZNF417 | | 147687 |
| ENSG00000196724 | 11.37023168 | 0.022393758 | 0.1373685 | 0.3318768 | NA | ZNF418 | | 147686 |
| ENSG00000152454 | 198.6034969 | 0.410841598 | 0.2196617 | 0.0037774 | 0.0304936 | ZNF256 | | 10172 |
| ENSG00000166704 | 115.2257557 | 0.021262452 | 0.1215504 | 0.7158347 | 0.8713698 | ZNF606 | | 80095 |
| ENSG00000176593 | 48.50285201 | -0.003346177 | 0.1290995 | 0.9345007 | 0.9750297 | ZNF606-AS1 | | 100128398 |
| ENSG00000121413 | 228.6881823 | 0.167470747 | 0.1668968 | 0.0642049 | 0.2279566 | ZSCAN18 | | 65982 |
| ENSG00000181894 | 247.7971278 | 0.272776192 | 0.1895231 | 0.0153919 | 0.0856972 | ZNF329 | | 79673 |
| ENSG00000171606 | 487.7272667 | 0.358179981 | 0.1350469 | 0.0007865 | 0.0092273 | ZNF274 | | 10782 |
| ENSG00000198131 | 739.3047003 | -0.069708278 | 0.0896713 | 0.3140617 | 0.5827728 | ZNF544 | | 27300 |
| ENSG00000268516 | 58.30195798 | -0.109222978 | 0.1894831 | 0.0488779 | 0.1897448 | NA | NA | |
| ENSG00000268201 | 10.00776774 | 0.015321999 | 0.1360923 | 0.4898364 | NA | NA | NA | |
| ENSG00000267216 | 47.14337859 | -0.001864482 | 0.1285988 | 0.9640457 | 0.9854745 | ZNF8-ERVK | | 108903150 |
| ENSG00000142396 | 479.1895024 | -0.044776644 | 0.0997241 | 0.5218675 | 0.7494387 | ERVK3-1 | | 105372481 |
| ENSG00000182318 | 130.2672311 | 0.091251089 | 0.1471091 | 0.186621 | 0.4373905 | ZSCAN22 | | 342945 |
| ENSG00000174586 | 22.31417067 | 0.033124992 | 0.13826 | 0.3128505 | NA | ZNF497 | | 162968 |
| ENSG00000152475 | 46.73504832 | 0.605354576 | 0.6515944 | 0.0067019 | 0.0472732 | ZNF837 | | 116412 |
| ENSG00000083845 | 10460.66857 | 0.034993401 | 0.0515588 | 0.4638052 | 0.7079196 | RPS5 | | 6193 |
| ENSG00000171574 | 332.6104239 | 0.124540726 | 0.1330341 | 0.1260767 | 0.3483265 | ZNF584 | | 201514 |
| ENSG00000232098 | 206.4884038 | 0.173505534 | 0.1783375 | 0.059205 | 0.2161842 | NA | NA | |
| ENSG00000268543 | 27.5366421 | -0.021155027 | 0.1342957 | 0.5356767 | NA | NA | NA | |
| ENSG00000269473 | 29.33705206 | -0.024426188 | 0.1344147 | 0.500996 | NA | NA | NA | |
| ENSG00000249471 | 137.8857468 | 0.193436041 | 0.2481417 | 0.0395229 | 0.1664864 | ZNF324B | | 388569 |
| ENSG00000083812 | 248.7179965 | 0.132471571 | 0.1474952 | 0.1095538 | 0.3204051 | ZNF324 | | 25799 |
| ENSG00000083838 | 187.2198478 | 0.057647616 | 0.1217948 | 0.3960758 | 0.6562498 | ZNF446 | | 55663 |
| ENSG00000083807 | 52.67646852 | -0.031199721 | 0.1335129 | 0.473775 | 0.7139512 | SLC27A5 | | 10998 |
| ENSG00000119574 | 237.572704 | -0.031836675 | 0.1161694 | 0.6213716 | 0.818389 | ZBTB45 | | 84878 |
| ENSG00000130726 | 8882.235716 | 0.046080869 | 0.0513674 | 0.3317043 | 0.5992396 | TRIM28 | | 10155 |
| ENSG00000130724 | 760.4905854 | -0.041933213 | 0.0871513 | 0.5404561 | 0.7640164 | CHMP2A | | 27243 |
| ENSG00000130725 | 2315.126722 | 0.37347549 | 0.0750555 | 7.56E-08 | 3.76E-06 | UBE2M | | 9040 |

| | | | | | | | |
|---|---|---|---|---|---|---|---|
| ENSG00000267858 | 92.83957466 | 0.248628186 | 0.4157105 | 0.0193144 | 0.101171 | MZF1-AS1 | 100131691 |
| ENSG00000099326 | 375.7533843 | 0.055790709 | 0.109844 | 0.4319867 | 0.6832356 | MZF1 | 7593 |
| ENSG00000213753 | 438.7591024 | -0.29876824 | 0.1554235 | 0.0059577 | 0.0432399 | CENPBD2P | 65996 |
| ENSG00000206195 | 644.9105558 | -0.028397185 | 0.0919085 | 0.6769204 | 0.8525183 | DUXAP8 | 503637 |
| ENSG00000272872 | 85.26908496 | 0.043401223 | 0.1325067 | 0.4174687 | 0.6708302 | NA | NA |
| ENSG00000271127 | 27.16047281 | 0.000144069 | 0.1319301 | 0.998659 | NA | NA | NA |
| ENSG00000237438 | 173.8693753 | 0.223516329 | 0.2220669 | 0.0326404 | 0.1458578 | NA | NA |
| ENSG00000273203 | 30.37362557 | 0.02056376 | 0.1340742 | 0.5535264 | NA | NA | NA |
| ENSG00000177663 | 714.6768309 | -0.017512991 | 0.0854877 | 0.7923743 | 0.9130225 | IL17RA | 23765 |
| ENSG00000069998 | 872.5798447 | -0.125846755 | 0.0938627 | 0.0844197 | 0.2713742 | HDHD5 | 27440 |
| ENSG00000131100 | 1968.740672 | 0.203976655 | 0.0751665 | 0.001871 | 0.0180321 | ATP6V1E1 | 529 |
| ENSG00000099968 | 1269.113433 | 0.123680897 | 0.0820444 | 0.0671661 | 0.2346895 | BCL2L13 | 23786 |
| ENSG00000015475 | 1154.899813 | 0.020563895 | 0.075326 | 0.74444 | 0.8889216 | BID | 637 |
| ENSG00000243156 | 1940.028182 | -0.154326587 | 0.0750624 | 0.0163575 | 0.0895788 | MICAL3 | 57553 |
| ENSG00000225335 | 19.13552409 | 0.024732708 | 0.1363598 | 0.4139655 | NA | NA | NA |
| ENSG00000215193 | 1376.904966 | 0.084958901 | 0.0771173 | 0.186629 | 0.4373905 | PEX26 | 55670 |
| ENSG00000184979 | 103.9897257 | 0.027351075 | 0.1263462 | 0.6134864 | 0.8151672 | USP18 | 11274 |
| ENSG00000183628 | 104.1690505 | 0.102104521 | 0.1657531 | 0.1193078 | 0.3372785 | DGCR6 | 8214 |
| ENSG00000237517 | 55.34003406 | 0.060600109 | 0.1451182 | 0.2141086 | 0.4718872 | NA | NA |
| ENSG00000070413 | 1713.701562 | 0.008291598 | 0.0686393 | 0.8897338 | 0.9550616 | DGCR2 | 9993 |
| ENSG00000273311 | 64.17201315 | -0.129647292 | 0.2076997 | 0.0485973 | 0.1890328 | DGCR11 | 25786 |
| ENSG00000100056 | 692.0180954 | 0.019307233 | 0.0871466 | 0.7749719 | 0.9050637 | ESS2 | 8220 |
| ENSG00000260924 | 43.11985225 | 0.03648034 | 0.1361573 | 0.3867599 | 0.647427 | NA | NA |
| ENSG00000100075 | 2154.442476 | -0.038267046 | 0.0675174 | 0.5137261 | 0.7440588 | SLC25A1 | 6576 |
| ENSG00000070371 | 451.2982385 | -0.001207494 | 0.0951532 | 0.9845089 | 0.9940394 | CLTCL1 | 8218 |
| ENSG00000100084 | 268.9005915 | 0.014482915 | 0.1077607 | 0.8321988 | 0.9305286 | HIRA | 7290 |
| ENSG00000242259 | 404.1779125 | 0.072716042 | 0.1068466 | 0.3187505 | 0.5874843 | C22orf39 | 128977 |
| ENSG00000185608 | 683.7146473 | -0.0398546 | 0.0877316 | 0.5552909 | 0.775323 | MRPL40 | 64976 |
| ENSG00000185065 | 19.55081968 | -0.000850738 | 0.1332785 | 0.9749416 | NA | NA | NA |
| ENSG00000070010 | 1892.513036 | 0.004766799 | 0.0662416 | 0.9351831 | 0.9752526 | UFD1 | 7353 |
| ENSG00000273212 | 11.31675509 | -0.003759189 | 0.134881 | 0.8631761 | NA | NA | NA |
| ENSG00000093009 | 1340.234958 | 0.012086277 | 0.0742052 | 0.8472827 | 0.9374926 | CDC45 | 8318 |
| ENSG00000184702 | 293.736533 | -0.025156692 | 0.105136 | 0.71125 | 0.8691254 | SEPTIN5 | 5413 |
| ENSG00000185838 | 132.8139247 | -0.052428776 | 0.1273151 | 0.4050938 | 0.6614808 | GNB1L | 54584 |
| ENSG00000215012 | 784.2927548 | -0.017625381 | 0.0842615 | 0.7899206 | 0.9118019 | RTL10 | 79680 |
| ENSG00000184470 | 689.2209481 | -0.159161899 | 0.1079043 | 0.046343 | 0.1841023 | TXNRD2 | 10587 |
| ENSG00000093010 | 1013.350233 | 0.08029184 | 0.0856015 | 0.2428128 | 0.5085703 | COMT | 1312 |
| ENSG00000099889 | 286.5943198 | -0.067972346 | 0.1179682 | 0.34155 | 0.6087837 | ARVCF | 421 |
| ENSG00000183597 | 602.810036 | 0.058945259 | 0.094697 | 0.4021367 | 0.6596487 | TANGO2 | 128989 |
| ENSG00000128191 | 1422.211264 | -0.130576665 | 0.0816161 | 0.0530788 | 0.2005868 | DGCR8 | 54487 |
| ENSG00000099899 | 1015.065769 | 0.103971428 | 0.087035 | 0.1351377 | 0.3618023 | TRMT2A | 27037 |
| ENSG00000099901 | 6715.161121 | -0.024175967 | 0.0535997 | 0.6243027 | 0.8202204 | RANBP1 | 5902 |
| ENSG00000099904 | 1385.605674 | 0.010683645 | 0.0723442 | 0.8628942 | 0.9451863 | ZDHHC8 | 29801 |
| ENSG00000040608 | 137.989637 | -0.07162716 | 0.1370537 | 0.2681315 | 0.5361059 | RTN4R | 65078 |
| ENSG00000128185 | 715.8570634 | 0.129518419 | 0.1021889 | 0.089407 | 0.2821711 | DGCR6L | 85359 |
| ENSG00000273343 | 21.77751371 | -0.009763312 | 0.1334469 | 0.7495573 | NA | NA | NA |
| ENSG00000206176 | 16.66571137 | -0.035841903 | 0.140426 | 0.2113297 | NA | NA | NA |
| ENSG00000215513 | 775.2347668 | -0.010193933 | 0.0846273 | 0.8772808 | 0.950252 | NA | NA |
| ENSG00000185252 | 404.7385633 | 0.050748569 | 0.1037514 | 0.4729382 | 0.7132849 | ZNF74 | 7625 |
| ENSG00000244486 | 9.336706915 | 0.008027946 | 0.1354057 | 0.6990552 | NA | SCARF2 | 91179 |
| ENSG00000099910 | 305.713765 | 0.156129973 | 0.1466863 | 0.0742904 | 0.250052 | KLHL22 | 84861 |
| ENSG00000099917 | 1532.489794 | 0.151711308 | 0.0808563 | 0.0248408 | 0.1202483 | MED15 | 51586 |
| ENSG00000226287 | 78.73661097 | 0.017958333 | 0.1257836 | 0.7302077 | 0.87988 | NA | NA |
| ENSG00000241973 | 2377.437827 | -0.145089396 | 0.0697567 | 0.0169627 | 0.0919833 | PI4KA | 5297 |
| ENSG00000099940 | 1190.319736 | 0.273644393 | 0.0940696 | 0.0006007 | 0.0074014 | SNAP29 | 9342 |
| ENSG00000099942 | 2746.592338 | -0.06437712 | 0.0670051 | 0.2711558 | 0.5388519 | CRKL | 1399 |
| ENSG00000183773 | 10.4930158 | -0.001340251 | 0.1348638 | 0.9487661 | NA | AIFM3 | 150209 |
| ENSG00000099949 | 1307.886645 | 0.025250495 | 0.0749993 | 0.6884539 | 0.8581614 | LZTR1 | 8216 |
| ENSG00000184436 | 355.8534452 | 0.078085571 | 0.1126254 | 0.2903876 | 0.5601086 | THAP7 | 80764 |
| ENSG00000230513 | 23.04650624 | -0.036201773 | 0.1392245 | 0.2766917 | NA | THAP7-AS1 | 439931 |
| ENSG00000161149 | 18.53044571 | 0.014365553 | 0.1344963 | 0.6219303 | NA | NA | NA |
| ENSG00000169635 | 388.3437434 | 0.022783961 | 0.1046145 | 0.7390548 | 0.8857761 | HIC2 | 23119 |
| ENSG00000206140 | 14.37485953 | -0.021564597 | 0.1365848 | 0.4037834 | NA | TMEM191C | 645426 |
| ENSG00000183506 | 34.13871202 | 0.00716881 | 0.130897 | 0.8536872 | NA | PI4KAP2 | 375133 |
| ENSG00000185651 | 2773.597187 | -0.192097204 | 0.068124 | 0.0014943 | 0.0153656 | UBE2L3 | 7332 |
| ENSG00000161179 | 955.735074 | 0.326414246 | 0.1006511 | 0.0001495 | 0.0024094 | YDJC | 150223 |
| ENSG00000161180 | 15.71672826 | -0.020507187 | 0.1361378 | 0.444005 | NA | CCDC116 | 164592 |
| ENSG00000128228 | 1231.111973 | -0.03582616 | 0.0771069 | 0.5737553 | 0.7880113 | SDF2L1 | 23753 |
| ENSG00000100023 | 1018.958427 | -0.10674563 | 0.0866245 | 0.1229505 | 0.3437492 | PPIL2 | 23759 |
| ENSG00000100027 | 48.76308324 | 0.016985326 | 0.130364 | 0.6929453 | 0.8596319 | YPEL1 | 29799 |

| | | | | | | | | |
|---|---|---|---|---|---|---|---|---|
| ENSG00000100030 | 3177.646102 | -0.082498216 | 0.0618172 | 0.1339437 | 0.3605062 | MAPK1 | | 5594 |
| ENSG00000100034 | 2137.932725 | -0.017904688 | 0.0695069 | 0.7641363 | 0.8983744 | PPM1F | | 9647 |
| ENSG00000224086 | 25.93975319 | -0.000289015 | 0.1322658 | 0.9909586 | NA | NA | NA | |
| ENSG00000100038 | 648.9913273 | 0.048714484 | 0.0904257 | 0.4760771 | 0.7156114 | TOP3B | | 8940 |
| ENSG00000272216 | 44.37977592 | -0.020000493 | 0.1314007 | 0.6302296 | 0.8239333 | NA | NA | |
| ENSG00000272779 | 69.13017473 | 0.064680139 | 0.1439681 | 0.2318709 | 0.4941635 | NA | NA | |
| ENSG00000128266 | 117.5881304 | -0.010182792 | 0.1209423 | 0.856397 | 0.9418673 | GNAZ | | 2781 |
| ENSG00000100228 | 21.79786462 | 0.00360385 | 0.1328338 | 0.9108643 | NA | RAB36 | | 9609 |
| ENSG00000186716 | 1315.899201 | 0.11055651 | 0.0825608 | 0.1016326 | 0.3051852 | BCR | | 613 |
| ENSG00000230701 | 9.410228408 | 0.028289281 | 0.1393576 | 0.1815758 | NA | FBXW4P1 | | 26226 |
| ENSG00000133519 | 16.25224779 | -0.014195312 | 0.1347581 | 0.6070274 | NA | NA | NA | |
| ENSG00000234353 | 9.080603634 | 0.03172609 | 0.1405141 | 0.133953 | NA | NA | NA | |
| ENSG00000244723 | 12.25206645 | 0.002586728 | 0.134717 | 0.9125677 | NA | NA | NA | |
| ENSG00000272578 | 86.79259306 | 0.006065193 | 0.1234288 | 0.9108971 | 0.9648392 | NA | NA | |
| ENSG00000159496 | 11.397902 | -0.004049847 | 0.1347774 | 0.8569712 | NA | RGL4 | | 266747 |
| ENSG00000270041 | 10.93309984 | 0.005756054 | 0.1348098 | 0.8056013 | NA | NA | NA | |
| ENSG00000187792 | 93.58443716 | 0.044261207 | 0.1299366 | 0.4385421 | 0.6882367 | ZNF70 | | 7621 |
| ENSG00000250479 | 620.7655215 | 0.03992331 | 0.0925482 | 0.5639815 | 0.7815717 | CHCHD10 | | 400916 |
| ENSG00000099956 | 1998.810346 | -0.236774052 | 0.0755796 | 0.0003846 | 0.0052865 | SMARCB1 | | 6598 |
| ENSG00000133460 | 351.8790852 | -0.008058597 | 0.1023355 | 0.9044012 | 0.9622177 | SLC2A11 | | 66035 |
| ENSG00000240972 | 2066.078176 | -0.013035278 | 0.0665297 | 0.8216758 | 0.9268052 | MIF | | 4282 |
| ENSG00000218537 | 67.85979064 | -0.076088598 | 0.1532968 | 0.1551038 | 0.3935203 | NA | NA | |
| ENSG00000099974 | 79.79218693 | 0.005010413 | 0.124236 | 0.9247669 | 0.9709924 | DDTL | | 100037417 |
| ENSG00000272787 | 19.55576182 | 0.017228253 | 0.1347813 | 0.5656805 | NA | NA | NA | |
| ENSG00000099977 | 875.4334786 | 0.325800013 | 0.1064511 | 0.0002727 | 0.0039825 | DDT | | 1652 |
| ENSG00000099991 | 1709.361061 | -0.081859128 | 0.0728159 | 0.1857178 | 0.4358915 | CABIN1 | | 23523 |
| ENSG00000232545 | 24.3884293 | -0.000372745 | 0.1323774 | 0.9888488 | NA | NA | NA | |
| ENSG00000100014 | 840.0608283 | -0.043425975 | 0.0846475 | 0.5167688 | 0.7459684 | SPECC1L | | 23384 |
| ENSG00000128271 | 23.407284 | -0.012253276 | 0.1334205 | 0.699845 | NA | ADORA2A | | 135 |
| ENSG00000138867 | 1683.744707 | 0.096008565 | 0.074847 | 0.1288808 | 0.3532231 | GUCD1 | | 83606 |
| ENSG00000100028 | 2237.754201 | -0.122223917 | 0.0694537 | 0.04265 | 0.1746212 | SNRPD3 | | 6634 |
| ENSG00000100031 | 9.32083925 | 0.026357097 | 0.1388624 | 0.2016027 | NA | GGT1 | | 2678 |
| ENSG00000178026 | 13.17904921 | 0.054801363 | 0.1508936 | 0.036167 | NA | LRRC75B | | 388886 |
| ENSG00000240906 | 8.942915702 | -0.006729099 | 0.135285 | 0.7434239 | NA | NA | NA | |
| ENSG00000197077 | 1088.615174 | -0.110803891 | 0.0852854 | 0.1064886 | 0.3147502 | KIAA1671 | | 85379 |
| ENSG00000231466 | 17.52189039 | 0.037268953 | 0.1409109 | 0.2001229 | NA | NA | NA | |
| ENSG00000100068 | 174.2041818 | 0.092566064 | 0.1431042 | 0.1946733 | 0.4467541 | NA | NA | |
| ENSG00000100058 | 311.4187012 | 0.1796953 | 0.1531951 | 0.0507847 | 0.194132 | NA | NA | |
| ENSG00000272977 | 71.49405209 | 0.099231785 | 0.1693104 | 0.1031766 | 0.3079809 | NA | NA | |
| ENSG00000100077 | 795.7237367 | -0.129447098 | 0.1038482 | 0.0913214 | 0.2859853 | GRK3 | | 157 |
| ENSG00000128203 | 132.9261197 | -0.043002832 | 0.1281212 | 0.4598077 | 0.7041733 | ASPHD2 | | 57168 |
| ENSG00000100099 | 772.1624234 | 0.051297136 | 0.088188 | 0.4532565 | 0.6987417 | HPS4 | | 89781 |
| ENSG00000100104 | 673.0906525 | -0.040854885 | 0.0882354 | 0.5448309 | 0.7670446 | SRRD | | 402055 |
| ENSG00000100109 | 1021.081139 | -0.014190255 | 0.0781009 | 0.8240328 | 0.9274924 | TFIP11 | | 24144 |
| ENSG00000261188 | 47.09781358 | 0.017576599 | 0.1306617 | 0.6801888 | 0.853781 | TFIP11-DT | | 100507599 |
| ENSG00000260065 | 24.27842893 | -0.020841281 | 0.1349435 | 0.5125098 | NA | NA | NA | |
| ENSG00000128294 | 279.7785864 | -0.025153148 | 0.1066031 | 0.6922919 | 0.8594028 | TPST2 | | 8459 |
| ENSG00000244625 | 23.13840595 | 0.00950181 | 0.1331035 | 0.7681198 | NA | NA | NA | |
| ENSG00000169184 | 473.8054315 | 0.065421439 | 0.1014529 | 0.362747 | 0.6258859 | MN1 | | 4330 |
| ENSG00000180957 | 1511.87583 | -0.093555974 | 0.0838404 | 0.1667438 | 0.4119574 | PITPNB | | 23760 |
| ENSG00000235954 | 357.6439216 | 0.075920405 | 0.1110659 | 0.3013925 | 0.5710237 | TTC28-AS1 | | 284900 |
| ENSG00000100154 | 810.7939928 | -0.018964322 | 0.0825253 | 0.7725585 | 0.9036003 | TTC28 | | 23331 |
| ENSG00000183765 | 463.3335131 | -0.044138209 | 0.1000311 | 0.5279083 | 0.7546809 | CHEK2 | | 11200 |
| ENSG00000100209 | 256.1116794 | 0.027966148 | 0.1082202 | 0.6805107 | 0.8538239 | HSCB | | 150274 |
| ENSG00000159873 | 814.2412779 | 0.003433902 | 0.0833421 | 0.9594158 | 0.983237 | CCDC117 | | 150275 |
| ENSG00000100219 | 1157.073656 | 0.370850241 | 0.095283 | 1.09E-05 | 0.0002733 | XBP1 | | 7494 |
| ENSG00000183579 | 463.3310863 | 0.046112313 | 0.1002005 | 0.5124571 | 0.7428656 | ZNRF3 | | 84133 |
| ENSG00000183762 | 492.7117419 | -0.041962701 | 0.0960419 | 0.5457051 | 0.7679526 | KREMEN1 | | 83999 |
| ENSG00000100263 | 467.2376903 | 0.059440364 | 0.1005375 | 0.4051232 | 0.6614808 | RHBDD3 | | 25807 |
| ENSG00000182944 | 7651.470633 | -0.269956734 | 0.0601592 | 1.38E-06 | 4.43E-05 | EWSR1 | | 2130 |
| ENSG00000185340 | 898.4849853 | -0.090399523 | 0.0904633 | 0.1982628 | 0.4513782 | GAS2L1 | | 10634 |
| ENSG00000273216 | 11.23725021 | 0.032835127 | 0.1405207 | 0.1589232 | NA | NA | NA | |
| ENSG00000100280 | 2769.839697 | -0.040836666 | 0.0624195 | 0.4616048 | 0.7059447 | AP1B1 | | 162 |
| ENSG00000239127 | 48.01574732 | -0.016262891 | 0.1296499 | 0.7109079 | 0.869096 | SNORD125 | | 100113380 |
| ENSG00000100296 | 1391.341749 | -0.152735634 | 0.0827065 | 0.0260377 | 0.1247013 | THOC5 | | 8563 |
| ENSG00000184117 | 1040.077685 | -0.071727736 | 0.0832505 | 0.2836958 | 0.5531382 | NIPSNAP1 | | 8508 |
| ENSG00000186575 | 3556.010648 | -0.081035253 | 0.0608576 | 0.1357279 | 0.3628243 | NF2 | | 4771 |
| ENSG00000100319 | 290.0236708 | 0.016130323 | 0.1046409 | 0.812005 | 0.9224888 | ZMAT5 | | 55954 |
| ENSG00000184076 | 1261.555036 | -0.159087415 | 0.0861578 | 0.0242589 | 0.1182672 | UQCR10 | | 29796 |
| ENSG00000100325 | 1071.26528 | 0.120015105 | 0.0873801 | 0.0871169 | 0.277052 | ASCC2 | | 84164 |

| | | | | | | | |
|---|---|---|---|---|---|---|---|
| ENSG00000100330 | 1274.483953 | 0.002917643 | 0.0794483 | 0.9648228 | 0.9859238 | MTMR3 | 8897 |
| ENSG00000232530 | 21.17756003 | 0.075212235 | 0.1641923 | 0.0290533 | NA | LIF-AS1 | 110806283 |
| ENSG00000128342 | 3712.807905 | 0.07934108 | 0.0675389 | 0.1787409 | 0.4275952 | LIF | 3976 |
| ENSG00000268812 | 33.94146913 | 0.019882168 | 0.1325648 | 0.608885 | NA | LIF-AS2 | 91370 |
| ENSG00000099992 | 222.3639889 | 0.094076078 | 0.1324311 | 0.2079385 | 0.4641563 | TBC1D10A | 83874 |
| ENSG00000099995 | 3534.749589 | 0.026716066 | 0.0584561 | 0.6154161 | 0.8158539 | SF3A1 | 10291 |
| ENSG00000187860 | 91.97928877 | 0.027324396 | 0.1254115 | 0.6221303 | 0.8187116 | CCDC157 | 550631 |
| ENSG00000241528 | 62.20710343 | 0.095467522 | 0.1688727 | 0.0975265 | 0.2972103 | NA | NA |
| ENSG00000099999 | 390.0875348 | 0.087655091 | 0.11189 | 0.2420305 | 0.5077511 | RNF215 | 200312 |
| ENSG00000100003 | 458.1317049 | 0.030584683 | 0.1023359 | 0.6569067 | 0.8419 | SEC14L2 | 23541 |
| ENSG00000242114 | 92.38347628 | -0.020206476 | 0.123893 | 0.7123197 | 0.8695378 | MTFP1 | 51537 |
| ENSG00000100029 | 3451.16166 | 0.176297229 | 0.0696522 | 0.0040352 | 0.0320644 | PES1 | 23481 |
| ENSG00000185339 | 10.12203171 | 0.007686939 | 0.1350788 | 0.7337478 | NA | TCN2 | 6948 |
| ENSG00000100036 | 378.7821874 | 0.275089372 | 0.1564387 | 0.0095385 | 0.0617281 | SLC35E4 | 339665 |
| ENSG00000167065 | 163.449474 | 0.004849676 | 0.1152111 | 0.9390988 | 0.9763786 | DUSP18 | 150290 |
| ENSG00000250318 | 11.01658336 | 0.000969569 | 0.134612 | 0.969019 | NA | RPL13AP26 | 729212 |
| ENSG00000184792 | 110.5487399 | 0.004232113 | 0.1201464 | 0.9415243 | 0.9771609 | OSBP2 | 23762 |
| ENSG00000133422 | 1042.103021 | 0.126882342 | 0.0974613 | 0.0892052 | 0.2816232 | MORC2 | 22880 |
| ENSG00000253352 | 2502.211446 | 0.083982087 | 0.065753 | 0.1447213 | 0.3772573 | TUG1 | 55000 |
| ENSG00000269987 | 14.45454696 | 0.010907419 | 0.1346683 | 0.6783531 | NA | NA | NA |
| ENSG00000183963 | 4179.861394 | 0.254637138 | 0.0628881 | 1.07E-05 | 0.0002694 | SMTN | 6525 |
| ENSG00000273387 | 14.52929226 | 0.025628761 | 0.1374549 | 0.3387835 | NA | NA | NA |
| ENSG00000198832 | 386.7391614 | 0.297987933 | 0.1548108 | 0.0059887 | 0.0433955 | SELENOM | 140606 |
| ENSG00000185133 | 68.51698233 | 0.097145936 | 0.1683505 | 0.1032981 | 0.3081614 | INPP5J | 27124 |
| ENSG00000138942 | 965.3304851 | 0.04383531 | 0.0816211 | 0.499053 | 0.7341862 | RNF185 | 91445 |
| ENSG00000182541 | 519.2539556 | 0.069170174 | 0.0993658 | 0.3534453 | 0.6187516 | LIMK2 | 3985 |
| ENSG00000100100 | 9.085537154 | 0.027672275 | 0.1393519 | 0.1678648 | NA | PIK3IP1 | 113791 |
| ENSG00000100105 | 831.3019035 | 0.007152967 | 0.0827942 | 0.9143838 | 0.9662935 | PATZ1 | 23598 |
| ENSG00000213888 | 25.32053555 | 0.003531114 | 0.1322453 | 0.9183584 | NA | NA | NA |
| ENSG00000185721 | 1568.916132 | -0.012245473 | 0.0700552 | 0.8379211 | 0.9331898 | DRG1 | 4733 |
| ENSG00000184708 | 641.4152149 | -0.203753659 | 0.1156512 | 0.0177029 | 0.0950747 | EIF4ENIF1 | 56478 |
| ENSG00000198089 | 712.5464805 | 0.002402055 | 0.0865341 | 0.9723041 | 0.9893759 | SFI1 | 9814 |
| ENSG00000241878 | 1829.02305 | 0.088934812 | 0.0733929 | 0.1538524 | 0.3917987 | PISD | 23761 |
| ENSG00000183530 | 1374.104507 | -0.015212076 | 0.071775 | 0.8027216 | 0.9177708 | PRR14L | 253143 |
| ENSG00000100150 | 358.3968515 | -0.027592712 | 0.107432 | 0.6830411 | 0.8545146 | DEPDC5 | 9681 |
| ENSG00000128245 | 3742.551481 | -0.042066717 | 0.0583058 | 0.4239932 | 0.6759631 | YWHAH | 7533 |
| ENSG00000242082 | 15.09976085 | 0.017751364 | 0.1358042 | 0.492675 | NA | SLC5A4-AS1 | 110806273 |
| ENSG00000214076 | 108.5463979 | 0.011560808 | 0.1211917 | 0.8397159 | 0.9343419 | CPSF1P1 | 129099 |
| ENSG00000224050 | 15.83730749 | -0.00186044 | 0.1338009 | 0.9433895 | NA | NA | NA |
| ENSG00000100220 | 2621.815612 | -0.150318699 | 0.0715024 | 0.0153101 | 0.0854455 | RTCB | 51493 |
| ENSG00000100225 | 1523.971433 | -0.086500763 | 0.075504 | 0.1728206 | 0.4199537 | FBXO7 | 25793 |
| ENSG00000133424 | 82.00594949 | -0.008094948 | 0.1247604 | 0.8720263 | 0.9484107 | LARGE1 | 9215 |
| ENSG00000100281 | 1067.10052 | -0.058673171 | 0.0837935 | 0.3920065 | 0.6522832 | HMGXB4 | 10042 |
| ENSG00000100284 | 521.7646785 | 0.346368876 | 0.1484375 | 0.0018754 | 0.0180445 | TOM1 | 10043 |
| ENSG00000100292 | 3626.086489 | 0.69574905 | 0.0778243 | 2.66E-20 | 8.19E-18 | HMOX1 | 3162 |
| ENSG00000100297 | 4644.683235 | 0.215165935 | 0.0686335 | 0.0004577 | 0.0059734 | MCM5 | 4174 |
| ENSG00000100302 | 11.69935185 | 0.019965169 | 0.1366609 | 0.4037949 | NA | RASD2 | 23551 |
| ENSG00000198125 | 11.02481699 | -0.018875295 | 0.1365335 | 0.4157301 | NA | MB | 4151 |
| ENSG00000221963 | 575.5463236 | 0.109659446 | 0.1105954 | 0.1545253 | 0.3927351 | APOL6 | 80830 |
| ENSG00000100320 | 2020.919828 | -0.144276015 | 0.0753249 | 0.0241978 | 0.1182036 | RBFOX2 | 23543 |
| ENSG00000128335 | 702.0312064 | 0.167912058 | 0.1096286 | 0.0386532 | 0.1640656 | APOL2 | 23780 |
| ENSG00000100342 | 84.57702436 | 0.087766789 | 0.1553513 | 0.156899 | 0.3960106 | APOL1 | 8542 |
| ENSG00000100345 | 41633.37606 | -0.133364559 | 0.05372 | 0.0096379 | 0.062268 | MYH9 | 4627 |
| ENSG00000100348 | 886.1851076 | -0.088692478 | 0.0888905 | 0.2024017 | 0.4565961 | TXN2 | 25828 |
| ENSG00000100350 | 995.2049195 | 0.277025014 | 0.1048211 | 0.0012775 | 0.013591 | FOXRED2 | 80020 |
| ENSG00000100353 | 3999.839582 | 0.416967524 | 0.0675163 | 6.92E-11 | 5.90E-09 | EIF3D | 8664 |
| ENSG00000100360 | 137.7869067 | -0.031356695 | 0.123055 | 0.5948626 | 0.8031885 | IFT27 | 11020 |
| ENSG00000128311 | 157.1010399 | 0.097338951 | 0.145216 | 0.1802084 | 0.4297604 | TST | 7263 |
| ENSG00000128309 | 954.9802934 | -0.061588542 | 0.0827852 | 0.3510955 | 0.6170849 | MPST | 4357 |
| ENSG00000100379 | 628.3801008 | 0.00390955 | 0.0906556 | 0.9567233 | 0.982546 | KCTD17 | 79734 |
| ENSG00000133466 | 28.21118558 | 0.035684099 | 0.1380092 | 0.3286144 | NA | C1QTNF6 | 114904 |
| ENSG00000100055 | 9.193990477 | -0.00581228 | 0.1352191 | 0.7766195 | NA | CYTH4 | 27128 |
| ENSG00000243902 | 35.68231551 | 0.046848607 | 0.1413384 | 0.2505871 | NA | ELFN2 | 114794 |
| ENSG00000237862 | 13.04222979 | 0.022060864 | 0.1368324 | 0.3852225 | NA | LOC100506: | 100506271 |
| ENSG00000166897 | 2730.542488 | -0.173193428 | 0.0769503 | 0.0084616 | 0.0564475 | ELFN2 | 114794 |
| ENSG00000272694 | 14.23547644 | 0.001732269 | 0.1341752 | 0.9483798 | NA | NA | NA |
| ENSG00000100060 | 10.8534124 | -0.028468757 | 0.139044 | 0.2176125 | NA | MFNG | 4242 |
| ENSG00000100065 | 2756.401425 | -0.165029649 | 0.0720823 | 0.0084271 | 0.0562815 | CARD10 | 29775 |
| ENSG00000128283 | 2393.666106 | 0.471572343 | 0.0839223 | 1.71E-09 | 1.21E-07 | CDC42EP1 | 11135 |
| ENSG00000100083 | 1059.957396 | 0.197548309 | 0.0944379 | 0.0097834 | 0.0628603 | GGA1 | 26088 |

| | | | | | | | | |
|---|---|---|---|---|---|---|---|---|
| ENSG00000100092 | 618.7547705 | 0.075844254 | 0.0977556 | 0.2916648 | 0.5613671 | SH3BP1 | | 23616 |
| ENSG00000233360 | 10.09714828 | -0.004214474 | 0.1348705 | 0.8480395 | NA | PDXP-DT | | 101927051 |
| ENSG00000241360 | 169.5422377 | 0.536629364 | 0.232074 | 0.0010788 | 0.0118321 | PDXP | | 57026 |
| ENSG00000100097 | 6853.634011 | -0.120313135 | 0.1160679 | 0.1273059 | 0.3504943 | LGALS1 | | 3956 |
| ENSG00000100101 | 460.4094549 | 0.083437973 | 0.1059905 | 0.2708296 | 0.5386338 | NA | NA | |
| ENSG00000100106 | 351.1959416 | 0.067751833 | 0.1108095 | 0.3506186 | 0.6164323 | TRIOBP | | 11078 |
| ENSG00000189060 | 5001.259571 | -0.495842301 | 0.0664759 | 7.48E-15 | 1.34E-12 | H1-0 | | 3005 |
| ENSG00000100116 | 372.204008 | 0.074459598 | 0.111769 | 0.3108757 | 0.5809213 | GCAT | | 23464 |
| ENSG00000100124 | 914.1921322 | 0.168056346 | 0.1027154 | 0.032763 | 0.1462378 | ANKRD54 | | 129138 |
| ENSG00000100129 | 4842.350882 | -0.099495869 | 0.0583545 | 0.0588327 | 0.2153635 | EIF3L | | 51386 |
| ENSG00000100139 | 1767.101556 | 0.238926232 | 0.1986775 | 0.0261039 | 0.124916 | MICALL1 | | 85377 |
| ENSG00000128346 | 8.898359894 | 0.017762922 | 0.1367022 | 0.4007615 | NA | C22orf23 | | 84645 |
| ENSG00000100142 | 1485.576375 | -0.039449563 | 0.071786 | 0.5186531 | 0.7475798 | POLR2F | | 5435 |
| ENSG00000100151 | 271.5335776 | 0.240010987 | 0.1763183 | 0.0222369 | 0.1115282 | PICK1 | | 9463 |
| ENSG00000184381 | 41.58770474 | 0.023310716 | 0.1321312 | 0.5778282 | 0.7911317 | PLA2G6 | | 8398 |
| ENSG00000185022 | 1158.977996 | 0.626685392 | 0.0922676 | 7.82E-13 | 1.04E-10 | MAFF | | 23764 |
| ENSG00000198792 | 1171.286675 | -0.011302207 | 0.0746773 | 0.8558844 | 0.9418544 | TMEM184B | | 25829 |
| ENSG00000213923 | 2463.626127 | 0.037289241 | 0.0636925 | 0.4974447 | 0.7324734 | CSNK1E | | 1454 |
| ENSG00000244627 | 49.98764081 | 0.05738437 | 0.1437106 | 0.2281325 | 0.4892263 | NA | NA | |
| ENSG00000100196 | 521.5813579 | 0.100264992 | 0.1064436 | 0.1912385 | 0.4421728 | KDELR3 | | 11015 |
| ENSG00000100201 | 11157.97717 | -0.298943552 | 0.0611674 | 1.66E-07 | 7.29E-06 | DDX17 | | 10521 |
| ENSG00000184949 | 49.9561127 | 0.093926864 | 0.1735997 | 0.0665707 | 0.2333139 | FAM227A | | 646851 |
| ENSG00000100211 | 367.4914101 | 0.128255361 | 0.1272061 | 0.115809 | 0.331804 | CBY1 | | 25776 |
| ENSG00000228274 | 11.77322863 | 0.024287818 | 0.1378592 | 0.2942236 | NA | NA | NA | |
| ENSG00000100216 | 1892.431358 | -0.192188998 | 0.0788387 | 0.0044246 | 0.0344561 | TOMM22 | | 56993 |
| ENSG00000100221 | 1462.268805 | 0.210071059 | 0.0831372 | 0.0029846 | 0.025591 | JOSD1 | | 9929 |
| ENSG00000100226 | 866.0444847 | 0.049893815 | 0.084482 | 0.4542624 | 0.6994533 | GTPBP1 | | 9567 |
| ENSG00000100242 | 1791.694767 | -0.148470769 | 0.0751374 | 0.0206715 | 0.1060912 | SUN2 | | 25777 |
| ENSG00000230149 | 14.93425329 | -0.004209283 | 0.1340124 | 0.8724802 | NA | NA | NA | |
| ENSG00000225450 | 12.97484961 | -0.018803358 | 0.1363084 | 0.4385893 | NA | NA | NA | |
| ENSG00000272669 | 24.95916682 | 0.020314546 | 0.1346161 | 0.5346807 | NA | NA | NA | |
| ENSG00000100246 | 217.2705215 | 0.338112293 | 0.2116973 | 0.0081079 | 0.0546185 | DNAL4 | | 10126 |
| ENSG00000221890 | 169.8336305 | 0.733379356 | 0.2214191 | 4.70E-05 | 0.0009327 | NPTXR | | 23467 |
| ENSG00000183741 | 1739.478472 | 0.001158412 | 0.0697662 | 0.9854906 | 0.9940394 | CBX6 | | 23466 |
| ENSG00000179750 | 1096.445719 | 0.078473327 | 0.0857986 | 0.2500409 | 0.5162193 | APOBEC3B | | 9582 |
| ENSG00000244509 | 942.921337 | -0.001387573 | 0.0857641 | 0.9823921 | 0.9934198 | APOBEC3C | | 27350 |
| ENSG00000243811 | 48.50125568 | -0.024569835 | 0.1311707 | 0.5801801 | 0.792456 | APOBEC3D | | 140564 |
| ENSG00000128394 | 30.43952165 | 0.066346918 | 0.1540796 | 0.0968802 | NA | APOBEC3F | | 200316 |
| ENSG00000239713 | 62.56828218 | 0.140181577 | 0.2258582 | 0.0378554 | 0.1614418 | APOBEC3G | | 60489 |
| ENSG00000100307 | 246.3435908 | 0.104234933 | 0.1327494 | 0.1777269 | 0.4270449 | CBX7 | | 23492 |
| ENSG00000100311 | 1353.621591 | -0.048098169 | 0.1203173 | 0.4651387 | 0.7086768 | PDGFB | | 5155 |
| ENSG00000100316 | 16431.49978 | -0.222744864 | 0.059745 | 5.00E-05 | 0.0009826 | RPL3 | | 6122 |
| ENSG00000209482 | 25.40424613 | -0.043463508 | 0.1426519 | 0.1807165 | NA | SNORD83A | | 116937 |
| ENSG00000100321 | 269.5399553 | 0.052546445 | 0.1121289 | 0.4534754 | 0.6987417 | SYNGR1 | | 9145 |
| ENSG00000100324 | 752.6037426 | -0.039199179 | 0.0860388 | 0.5624989 | 0.7803504 | TAB1 | | 10454 |
| ENSG00000128268 | 10.73558802 | -0.009205722 | 0.1352161 | 0.6789028 | NA | MGAT3 | | 4248 |
| ENSG00000100335 | 1820.928137 | 0.143997062 | 0.0759468 | 0.0258926 | 0.124108 | MIEF1 | | 54471 |
| ENSG00000128272 | 7452.457894 | 0.191887234 | 0.0640153 | 0.0008787 | 0.0100119 | ATF4 | | 468 |
| ENSG00000187051 | 1705.703176 | 0.001622249 | 0.0689863 | 0.9791123 | 0.992236 | RPS19BP1 | | 91582 |
| ENSG00000226085 | 8.737525549 | -0.01627748 | 0.136474 | 0.4323005 | NA | NA | NA | |
| ENSG00000100354 | 1089.064888 | -0.038642221 | 0.0798607 | 0.551761 | 0.7725183 | TNRC6B | | 23112 |
| ENSG00000239900 | 1510.262861 | -0.284929229 | 0.0946357 | 0.0004015 | 0.0054472 | ADSL | | 158 |
| ENSG00000100359 | 892.4155238 | -0.267811843 | 0.1043732 | 0.0016701 | 0.0166551 | SGSM3 | | 27352 |
| ENSG00000196588 | 718.9585895 | 0.113507922 | 0.1098067 | 0.1419176 | 0.3722268 | MRTFA | | 57591 |
| ENSG00000100372 | 770.7224629 | 0.051824651 | 0.08671 | 0.4427748 | 0.6917073 | SLC25A17 | | 10478 |
| ENSG00000100380 | 4435.840519 | -0.111005461 | 0.0635248 | 0.0486927 | 0.1890888 | ST13 | | 6767 |
| ENSG00000196236 | 433.0980454 | 0.157789551 | 0.1264649 | 0.0635696 | 0.2266657 | XPNPEP3 | | 63929 |
| ENSG00000100387 | 343.2216851 | -0.115415646 | 0.1291952 | 0.1462932 | 0.379239 | RBX1 | | 9978 |
| ENSG00000100393 | 3146.095262 | -0.084880423 | 0.0626858 | 0.1269227 | 0.3499531 | EP300 | | 2033 |
| ENSG00000231993 | 21.78236949 | 0.051646102 | 0.1468119 | 0.121058 | NA | NA | NA | |
| ENSG00000100395 | 1167.534789 | 0.024056573 | 0.0782537 | 0.7088089 | 0.8682463 | L3MBTL2 | | 83746 |
| ENSG00000100401 | 11759.42838 | -0.035818543 | 0.0495164 | 0.4417789 | 0.6908823 | RANGAP1 | | 5905 |
| ENSG00000100403 | 8282.525521 | -0.199670842 | 0.059389 | 0.0002384 | 0.0035523 | ZC3H7B | | 23264 |
| ENSG00000167074 | 269.4640616 | -0.08843241 | 0.1234364 | 0.2368177 | 0.5010625 | TEF | | 7008 |
| ENSG00000183864 | 1609.997899 | -0.107350692 | 0.0755324 | 0.0930421 | 0.2888186 | TOB2 | | 10766 |
| ENSG00000100410 | 984.3957079 | -0.015017071 | 0.07861 | 0.8146189 | 0.9241103 | PHF5A | | 84844 |
| ENSG00000100412 | 1603.840976 | 0.025490818 | 0.0702024 | 0.6728882 | 0.8500061 | ACO2 | | 50 |
| ENSG00000100413 | 2156.213686 | -0.041198583 | 0.0704885 | 0.4947883 | 0.7302112 | POLR3H | | 171568 |
| ENSG00000100417 | 762.9141295 | 0.223130532 | 0.1078375 | 0.0080606 | 0.0543939 | PMM1 | | 5372 |
| ENSG00000100418 | 979.4246799 | 0.165686832 | 0.0983424 | 0.0309436 | 0.1411627 | DESI1 | | 27351 |

| | | | | | | | | |
|---|---|---|---|---|---|---|---|---|
| ENSG00000196419 | 11603.92758 | -0.319238413 | 0.0531786 | 2.93E-10 | 2.25E-08 | XRCC6 | | 2547 |
| ENSG00000100138 | 2333.356589 | 0.139060324 | 0.0712892 | 0.0243585 | 0.1185548 | SNU13 | | 4809 |
| ENSG00000184208 | 200.2393295 | 0.178961569 | 0.1856298 | 0.0549456 | 0.2055108 | C22orf46P | | 79640 |
| ENSG00000100147 | 181.3836375 | 0.060209141 | 0.1237417 | 0.3756659 | 0.6373499 | CCDC134 | | 79879 |
| ENSG00000184068 | 8.482282158 | -0.006683455 | 0.1353592 | 0.7391678 | NA | NA | NA | |
| ENSG00000198911 | 2847.389788 | -0.3115807 | 0.0691295 | 9.87E-07 | 3.36E-05 | SREBF2 | | 6721 |
| ENSG00000100162 | 1027.073407 | 0.12886043 | 0.1609684 | 0.1055389 | 0.3128139 | CENPM | | 79019 |
| ENSG00000205704 | 18.19199634 | 0.937546613 | 1.6062055 | 0.0060715 | NA | SMIM45 | | 339674 |
| ENSG00000100167 | 16.201204 | 0.074405266 | 0.1650735 | 0.0141105 | NA | SEPTIN3 | | 55964 |
| ENSG00000198951 | 1016.965506 | -0.100533233 | 0.0923592 | 0.1587852 | 0.3993045 | NAGA | | 4668 |
| ENSG00000183172 | 298.033845 | -0.035623133 | 0.1084326 | 0.6017779 | 0.8077875 | SMDT1 | | 91689 |
| ENSG00000184983 | 1961.640075 | -0.051703106 | 0.0691799 | 0.3849829 | 0.6453768 | NDUFA6 | | 4700 |
| ENSG00000270083 | 20.72696625 | -0.017309129 | 0.1344544 | 0.5764141 | NA | NA | NA | |
| ENSG00000237037 | 39.63688135 | -0.042043514 | 0.138343 | 0.3176783 | NA | NDUFA6-DT | | 100132273 |
| ENSG00000205702 | 23.86623485 | 0.005165883 | 0.1328105 | 0.8730657 | NA | NA | NA | |
| ENSG00000226450 | 12.85824903 | -0.007268046 | 0.1345786 | 0.7681055 | NA | CYP2D8P | | 1568 |
| ENSG00000100207 | 2180.665417 | 0.040725355 | 0.0732449 | 0.5114984 | 0.7421105 | TCF20 | | 6942 |
| ENSG00000182057 | 59.93833707 | -0.019725794 | 0.1294106 | 0.66604 | 0.8452861 | NA | NA | |
| ENSG00000233903 | 36.19875364 | 0.048214072 | 0.1420467 | 0.2354641 | NA | NA | NA | |
| ENSG00000273366 | 14.6237013 | -0.017055495 | 0.1355096 | 0.519067 | NA | NA | NA | |
| ENSG00000172250 | 8.1711392 | 0.020453675 | 0.1375502 | 0.2932533 | NA | SERHL | | 94009 |
| ENSG00000189306 | 2116.609786 | 0.117439968 | 0.0722015 | 0.0580251 | 0.2133433 | RRP7A | | 27341 |
| ENSG00000183569 | 10.35289627 | -0.012864837 | 0.1356454 | 0.5643072 | NA | SERHL2 | | 253190 |
| ENSG00000182841 | 465.7460262 | 0.046394243 | 0.0996902 | 0.5096604 | 0.7412953 | NA | NA | |
| ENSG00000100227 | 2096.908969 | 0.111078232 | 0.0714122 | 0.0704773 | 0.2417045 | POLDIP3 | | 84271 |
| ENSG00000100243 | 2906.178962 | -0.023535875 | 0.0610882 | 0.6582813 | 0.8423953 | CYB5R3 | | 1727 |
| ENSG00000128274 | 208.7839092 | -0.09036447 | 0.1350889 | 0.2173086 | 0.476602 | A4GALT | | 53947 |
| ENSG00000242247 | 927.480979 | -0.196642407 | 0.0983006 | 0.0120128 | 0.0724273 | ARFGAP3 | | 26286 |
| ENSG00000100266 | 2395.814512 | -0.067862283 | 0.0665281 | 0.2436909 | 0.5095683 | PACSIN2 | | 11252 |
| ENSG00000100271 | 42.89045579 | 0.045582001 | 0.1394898 | 0.2929118 | 0.5623098 | TTLL1 | | 25809 |
| ENSG00000100290 | 24.49161844 | 0.014010581 | 0.1335651 | 0.667019 | NA | BIK | | 638 |
| ENSG00000100294 | 504.573469 | 0.115456918 | 0.1117683 | 0.1378372 | 0.3661762 | MCAT | | 27349 |
| ENSG00000100300 | 1867.587895 | 0.041653987 | 0.0713911 | 0.4951525 | 0.7306326 | TSPO | | 706 |
| ENSG00000100304 | 1928.382976 | 0.108858789 | 0.0740374 | 0.083657 | 0.2700934 | TTLL12 | | 23170 |
| ENSG00000100344 | 18.06107394 | 0.033983117 | 0.1394952 | 0.2516306 | NA | PNPLA3 | | 80339 |
| ENSG00000100347 | 817.5016464 | -0.010808211 | 0.0823134 | 0.8685758 | 0.9471139 | SAMM50 | | 25813 |
| ENSG00000188677 | 715.398115 | 0.14683996 | 0.1026665 | 0.0580065 | 0.2133418 | PARVB | | 29780 |
| ENSG00000188636 | 539.1296022 | 0.078322347 | 0.1001487 | 0.2848868 | 0.5542576 | RTL6 | | 84247 |
| ENSG00000186654 | 122.3719008 | 0.064775097 | 0.1355296 | 0.3008807 | 0.5706425 | PRR5 | | 55615 |
| ENSG00000248405 | 10.05579759 | 0.023530808 | 0.1378026 | 0.2919449 | NA | PRR5-ARHC | | 553158 |
| ENSG00000241484 | 55.52088006 | 0.030833575 | 0.1321071 | 0.5070047 | 0.7394584 | ARHGAP8 | | 23779 |
| ENSG00000056487 | 254.1296401 | -0.060474964 | 0.1167771 | 0.3890473 | 0.6493015 | PHF21B | | 112885 |
| ENSG00000230922 | 10.64300236 | 0.006907123 | 0.1349202 | 0.7655785 | NA | LOC1019275 | | 101927551 |
| ENSG00000226328 | 124.4436326 | 0.968163345 | 0.2573026 | 7.95E-06 | 0.0002071 | NUP50-DT | | 100506714 |
| ENSG00000093000 | 4522.294668 | -0.148054668 | 0.060574 | 0.0066325 | 0.0469826 | NUP50 | | 10762 |
| ENSG00000100364 | 1798.745573 | -0.232309571 | 0.0809828 | 0.0009267 | 0.0103887 | KIAA0930 | | 23313 |
| ENSG00000100376 | 588.7272048 | -0.005392831 | 0.0891516 | 0.9353038 | 0.9752526 | FAM118A | | 55007 |
| ENSG00000128408 | 102.3374232 | 0.83283201 | 0.3005538 | 0.000235 | 0.0035098 | RIBC2 | | 26150 |
| ENSG00000077942 | 2640.2939 | -0.015534406 | 0.062458 | 0.7790801 | 0.907593 | FBLN1 | | 2192 |
| ENSG00000130638 | 1227.973656 | -0.120688504 | 0.083332 | 0.0760677 | 0.2537974 | ATXN10 | | 25814 |
| ENSG00000188064 | 228.9315789 | -0.541157565 | 0.1950489 | 0.0003157 | 0.0045025 | WNT7B | | 7477 |
| ENSG00000273145 | 21.47735922 | 0.033717243 | 0.1389011 | 0.2828566 | NA | LOC124905 | | 124905134 |
| ENSG00000231711 | 47.11703568 | 0.017820376 | 0.1300186 | 0.686697 | 0.857187 | NA | NA | |
| ENSG00000241990 | 73.63068103 | -0.045008584 | 0.1339123 | 0.3893783 | 0.6497612 | NA | NA | |
| ENSG00000197182 | 241.910656 | 0.031984063 | 0.110026 | 0.6382741 | 0.8296072 | MIRLET7BH | | 400931 |
| ENSG00000186951 | 609.9269144 | -0.08299196 | 0.1007448 | 0.253844 | 0.5210248 | PPARA | | 5465 |
| ENSG00000205643 | 203.3362358 | 0.054349925 | 0.1221036 | 0.4177872 | 0.6710348 | CDPF1 | | 150383 |
| ENSG00000075234 | 651.7494575 | -0.103300671 | 0.0985193 | 0.1599446 | 0.4009618 | TTC38 | | 55020 |
| ENSG00000075218 | 2823.235593 | -0.09217714 | 0.0663618 | 0.1123621 | 0.3254406 | GTSE1 | | 51512 |
| ENSG00000100416 | 1522.667174 | -0.045288096 | 0.0742401 | 0.4684996 | 0.7105867 | TRMU | | 55687 |
| ENSG00000075275 | 1657.247819 | -0.210100931 | 0.0805429 | 0.0022716 | 0.0208437 | CELSR1 | | 9620 |
| ENSG00000075240 | 504.7250521 | -0.075606067 | 0.1006292 | 0.2956266 | 0.5657298 | GRAMD4 | | 23151 |
| ENSG00000100422 | 1264.134059 | -0.116305968 | 0.0812336 | 0.0820033 | 0.266521 | CERK | | 64781 |
| ENSG00000260708 | 49.17222152 | 0.001248765 | 0.128308 | 0.9808961 | 0.9930139 | NA | NA | |
| ENSG00000054611 | 390.078302 | -0.057256157 | 0.1044975 | 0.4210065 | 0.6735758 | TBC1D22A | | 25771 |
| ENSG00000224271 | 33.09865706 | 0.037267351 | 0.1378233 | 0.3351683 | NA | EPIC1 | | 284930 |
| ENSG00000100425 | 860.7841085 | -0.084502026 | 0.0875507 | 0.2198402 | 0.479502 | BRD1 | | 23774 |
| ENSG00000100426 | 1082.037618 | 0.044623686 | 0.0821069 | 0.5004591 | 0.7347747 | ZBED4 | | 9889 |
| ENSG00000182858 | 533.4032482 | -0.032252568 | 0.0932634 | 0.6385252 | 0.8297489 | ALG12 | | 79087 |
| ENSG00000273192 | 8.765109502 | -0.001514545 | 0.1350825 | 0.938948 | NA | NA | NA | |

| | | | | | | | | | |
|---|---|---|---|---|---|---|---|---|---|
| ENSG00000184164 | 1180.89656 | 0.088569697 | 0.0842266 | 0.191413 | 0.4424011 | CRELD2 | | 79174 |
| ENSG00000198355 | 1295.531511 | 0.075604529 | 0.082651 | 0.2563251 | 0.5231846 | PIM3 | | 415116 |
| ENSG00000073150 | 17.42286125 | -0.008719316 | 0.1339052 | 0.7579292 | NA | PANX2 | | 56666 |
| ENSG00000170638 | 1489.896859 | 0.013045794 | 0.0709678 | 0.8310609 | 0.9304016 | TRABD | | 80305 |
| ENSG00000273253 | 14.67900128 | -0.015484802 | 0.1352797 | 0.5538511 | NA | NA | NA | |
| ENSG00000073169 | 650.1755098 | 0.096803742 | 0.1028411 | 0.1937399 | 0.4456981 | SELENOO | | 83642 |
| ENSG00000273137 | 12.50721486 | 0.014798768 | 0.1355406 | 0.5479098 | NA | NA | NA | |
| ENSG00000128159 | 1090.577726 | -0.062021863 | 0.0793088 | 0.3417923 | 0.6087837 | TUBGCP6 | | 85378 |
| ENSG00000100429 | 45.71023437 | -0.030123872 | 0.1333093 | 0.4849066 | 0.7233041 | HDAC10 | | 83933 |
| ENSG00000188130 | 801.7681981 | 0.074902406 | 0.0897953 | 0.2819869 | 0.5510941 | MAPK12 | | 6300 |
| ENSG00000185386 | 965.1407149 | 0.086259763 | 0.0883032 | 0.2102057 | 0.4667198 | MAPK11 | | 5600 |
| ENSG00000196576 | 6042.030441 | -0.335612266 | 0.06054 | 4.19E-09 | 2.74E-07 | PLXNB2 | | 23654 |
| ENSG00000205593 | 20.14607673 | 0.024649937 | 0.1363966 | 0.4113611 | NA | DENND6B | | 414918 |
| ENSG00000100239 | 1401.874226 | 0.025145779 | 0.0727793 | 0.6844186 | 0.8554818 | PPP6R2 | | 9701 |
| ENSG00000100241 | 2978.903289 | -0.033256686 | 0.0643113 | 0.5564317 | 0.7758245 | SBF1 | | 6305 |
| ENSG00000128165 | 152.455114 | 0.965416398 | 0.2527632 | 6.43E-06 | 0.0001716 | ADM2 | | 79924 |
| ENSG00000100258 | 2233.592536 | 0.154344826 | 0.0728587 | 0.0142278 | 0.0813883 | LMF2 | | 91289 |
| ENSG00000025770 | 1514.256873 | 0.027262588 | 0.0707319 | 0.6531467 | 0.8396881 | NCAPH2 | | 29781 |
| ENSG00000130489 | 409.5530933 | 0.049740044 | 0.1024408 | 0.4802228 | 0.7192566 | NA | NA | |
| ENSG00000272821 | 80.65573491 | -0.009021228 | 0.1249258 | 0.857419 | 0.942459 | NA | NA | |
| ENSG00000025708 | 215.485577 | 0.09357475 | 0.1326754 | 0.2092416 | 0.4657317 | TYMP | | 1890 |
| ENSG00000177989 | 13.09405572 | -0.008111155 | 0.1347499 | 0.7367261 | NA | CIMAP1B | | 440836 |
| ENSG00000205560 | 25.22224284 | -0.006761244 | 0.1326209 | 0.8352192 | NA | CPT1B | | 1375 |
| ENSG00000100288 | 11.90386487 | 0.000592104 | 0.1344455 | 0.9826418 | NA | CHKB | | 1120 |
| ENSG00000205559 | 27.64292536 | 0.001521994 | 0.1323165 | 0.9655034 | NA | CHKB-DT | | 100144603 |
| ENSG00000008735 | 1214.005263 | 0.345277044 | 0.0956398 | 3.67E-05 | 0.0007638 | MAPK8IP2 | | 23542 |
| ENSG00000100299 | 404.1454456 | 0.058036828 | 0.1057631 | 0.4168567 | 0.6704259 | ARSA | | 410 |
| ENSG00000251322 | 863.7796161 | 0.304211643 | 0.1136142 | 0.0009753 | 0.0108291 | SHANK3 | | 85358 |
| ENSG00000184319 | 330.2907878 | 0.280549658 | 0.1726828 | 0.0112928 | 0.069315 | NA | NA | |
| ENSG00000079974 | 380.861276 | 0.138107419 | 0.1277184 | 0.0957407 | 0.2939152 | RABL2B | | 11158 |
| ENSG00000264462 | 100.1515962 | -0.086571235 | 0.1570315 | 0.1469106 | 0.3801492 | MIR3648-2 | | 103504731 |
| ENSG00000264063 | 17.84112628 | -0.002899018 | 0.1338988 | 0.9122538 | NA | NA | NA | |
| ENSG00000188681 | 25.6920661 | 0.078386527 | 0.1655985 | 0.039089 | NA | NA | NA | |
| ENSG00000271308 | 58.11672493 | -0.003100858 | 0.1273005 | 0.9449913 | 0.9784014 | NA | NA | |
| ENSG00000228314 | 95.31260376 | 0.051963185 | 0.1324562 | 0.3737416 | 0.6352333 | NA | NA | |
| ENSG00000230965 | 13.56319182 | 0.000300149 | 0.1341743 | 0.9928797 | NA | NA | NA | |
| ENSG00000215559 | 65.62696392 | -0.027343151 | 0.130152 | 0.5612882 | 0.7795039 | NA | NA | |
| ENSG00000203616 | 11.78689176 | -0.014175928 | 0.1356991 | 0.5383776 | NA | NA | NA | |
| ENSG00000224905 | 16.64379738 | -0.014275599 | 0.134892 | 0.5979982 | NA | NA | NA | |
| ENSG00000185272 | 29.96579008 | 0.001342681 | 0.1314102 | 0.9724938 | NA | RBM11 | | 54033 |
| ENSG00000155304 | 1947.646092 | -0.025116676 | 0.0689071 | 0.6722671 | 0.8499086 | HSPA13 | | 6782 |
| ENSG00000235609 | 37.48088485 | 0.026699415 | 0.1336844 | 0.4770182 | NA | NA | NA | |
| ENSG00000180530 | 580.0919949 | 0.064354377 | 0.0984169 | 0.3726757 | 0.6345995 | NRIP1 | | 8204 |
| ENSG00000155313 | 1857.615869 | -0.114260175 | 0.0843823 | 0.0944335 | 0.2915109 | USP25 | | 29761 |
| ENSG00000154639 | 499.3978383 | 0.021252754 | 0.0930963 | 0.7572051 | 0.8957781 | CXADR | | 1525 |
| ENSG00000154640 | 1569.18094 | 0.203427328 | 0.0842959 | 0.0042723 | 0.0335383 | BTG3 | | 10950 |
| ENSG00000154642 | 605.7612887 | -0.072807177 | 0.0953068 | 0.3057525 | 0.5749349 | C21orf91 | | 54149 |
| ENSG00000198618 | 454.9522107 | -0.347717308 | 0.154232 | 0.0022442 | 0.0206413 | NA | NA | |
| ENSG00000233215 | 8.658115658 | -1.45E-05 | 0.1350186 | 0.9971037 | NA | LINC01687 | | 101927843 |
| ENSG00000234883 | 22.56620895 | 0.020032676 | 0.1346607 | 0.5365104 | NA | MIR155HG | | 114614 |
| ENSG00000154719 | 803.0426738 | -0.348618935 | 0.1151325 | 0.0002668 | 0.0039107 | MRPL39 | | 54148 |
| ENSG00000154721 | 16.00367494 | 0.008329151 | 0.1343535 | 0.7539651 | NA | JAM2 | | 58494 |
| ENSG00000154723 | 1367.209345 | -0.119079084 | 0.083584 | 0.0807094 | 0.2636371 | ATP5PF | | 522 |
| ENSG00000154727 | 758.117575 | -0.092810073 | 0.0933173 | 0.1931995 | 0.445121 | GABPA | | 2551 |
| ENSG00000142192 | 27479.67328 | -0.048673579 | 0.0551482 | 0.3378385 | 0.6054931 | APP | | 351 |
| ENSG00000166265 | 193.5575463 | 0.049103056 | 0.1185792 | 0.4667299 | 0.7094916 | CYYR1 | | 116159 |
| ENSG00000154734 | 2649.39663 | 0.116304288 | 0.0759058 | 0.0696664 | 0.2400304 | ADAMTS1 | | 9510 |
| ENSG00000154736 | 142.0503674 | 0.0541949 | 0.1274004 | 0.3952548 | 0.6555404 | ADAMTS5 | | 11096 |
| ENSG00000156239 | 217.1521841 | -0.047456379 | 0.1160131 | 0.4811934 | 0.7202486 | N6AMT1 | | 29104 |
| ENSG00000198862 | 1267.458582 | 0.196495548 | 0.0902102 | 0.0081228 | 0.054647 | LTN1 | | 26046 |
| ENSG00000156253 | 475.8311124 | 0.278072655 | 0.1465633 | 0.0072656 | 0.0501296 | RWDD2B | | 10069 |
| ENSG00000156256 | 788.3647109 | -0.168217289 | 0.1028978 | 0.0324381 | 0.1452349 | USP16 | | 10600 |
| ENSG00000156261 | 7036.146487 | -0.118674598 | 0.0546579 | 0.0176174 | 0.0948571 | CCT8 | | 10694 |
| ENSG00000231125 | 12.74389007 | 0.021637413 | 0.1368589 | 0.3819162 | NA | NA | NA | |
| ENSG00000156273 | 444.8817789 | 0.257892673 | 0.1450053 | 0.0105039 | 0.0656833 | BACH1 | | 571 |
| ENSG00000248476 | 11.36381282 | -0.003908303 | 0.1347122 | 0.8632592 | NA | NA | NA | |
| ENSG00000142168 | 3801.344887 | -0.117540622 | 0.0617909 | 0.0331355 | 0.1468901 | SOD1 | | 6647 |
| ENSG00000273271 | 8.630027103 | -0.01512662 | 0.1363075 | 0.4614456 | NA | NA | NA | |
| ENSG00000156304 | 1086.047769 | -0.074646935 | 0.0853273 | 0.2712723 | 0.5389637 | SCAF4 | | 57466 |
| ENSG00000159055 | 624.100322 | -0.111595451 | 0.1048613 | 0.1407439 | 0.3702607 | MIS18A | | 54069 |

| | | | | | | | |
|---|---|---|---|---|---|---|---|
| ENSG00000142207 | 2451.436863 | 0.442401805 | 0.0754653 | 4.45E-10 | 3.35E-08 | URB1 | 9875 |
| ENSG00000256073 | 42.80913494 | -0.000712343 | 0.1293021 | 0.9845663 | 0.9940394 | URB1-AS1 | 84996 |
| ENSG00000159079 | 369.8995954 | 0.094909828 | 0.1156526 | 0.2130916 | 0.4702669 | CFAP298 | 56683 |
| ENSG00000159082 | 670.736569 | 0.134978577 | 0.1032399 | 0.0783939 | 0.2585037 | SYNJ1 | 8867 |
| ENSG00000238197 | 24.38038565 | -0.021658732 | 0.1348405 | 0.5094412 | NA | PAXBP1-AS | 100506215 |
| ENSG00000159086 | 1809.142472 | 0.026427856 | 0.0690373 | 0.6594191 | 0.8426512 | PAXBP1 | 94104 |
| ENSG00000159110 | 777.2200919 | -0.046415161 | 0.0878595 | 0.4943351 | 0.729819 | IFNAR2 | 3455 |
| ENSG00000223799 | 19.66794211 | 0.026863253 | 0.136999 | 0.3691048 | NA | NA | NA |
| ENSG00000243646 | 257.7926639 | -0.023032258 | 0.1101043 | 0.7277599 | 0.8782138 | IL10RB | 3588 |
| ENSG00000142166 | 1410.591367 | -0.036949702 | 0.0722259 | 0.5467867 | 0.7687789 | IFNAR1 | 3454 |
| ENSG00000159128 | 1359.596793 | -0.059992282 | 0.0780354 | 0.3516188 | 0.6173544 | IFNGR2 | 3460 |
| ENSG00000142188 | 899.7908297 | -0.094493849 | 0.094032 | 0.1869253 | 0.4377456 | TMEM50B | 757 |
| ENSG00000177692 | 15.16584567 | -0.009180642 | 0.1344884 | 0.723713 | NA | DNAJC28 | 54943 |
| ENSG00000159131 | 4488.333679 | -0.008156135 | 0.0606798 | 0.880129 | 0.9518649 | GART | 2618 |
| ENSG00000159140 | 9542.823238 | -0.174074723 | 0.0535983 | 0.0004548 | 0.0059537 | SON | 6651 |
| ENSG00000159147 | 1113.185121 | -0.045371055 | 0.0794597 | 0.4839452 | 0.7223311 | DONSON | 29980 |
| ENSG00000205758 | 313.5128927 | -0.1949469 | 0.1560466 | 0.0395095 | 0.1664864 | CRYZL1 | 9946 |
| ENSG00000205726 | 1270.431263 | -0.262394477 | 0.0923619 | 0.0007922 | 0.0092624 | ITSN1 | 6453 |
| ENSG00000241837 | 1183.489354 | -0.133253035 | 0.0913533 | 0.0647367 | 0.2293569 | ATP5PO | 539 |
| ENSG00000237945 | 575.4713659 | -0.149077497 | 0.1276622 | 0.076458 | 0.2545912 | LINC00649 | 100506334 |
| ENSG00000243927 | 904.2861263 | 0.067375663 | 0.0925971 | 0.3375717 | 0.6053386 | MRPS6 | 64968 |
| ENSG00000227456 | 9.079735123 | 0.023060267 | 0.1378755 | 0.2768434 | NA | LINC00310 | 114036 |
| ENSG00000205670 | 261.4262331 | 0.000406116 | 0.1062345 | 0.9962198 | 0.9986992 | SMIM11 | 54065 |
| ENSG00000159200 | 587.5739403 | 0.253067333 | 0.1280509 | 0.0075813 | 0.051818 | RCAN1 | 1827 |
| ENSG00000159216 | 854.2457 | 0.132426784 | 0.0943457 | 0.071652 | 0.2440137 | RUNX1 | 861 |
| ENSG00000185917 | 325.1875658 | 0.190193321 | 0.1569429 | 0.0433286 | 0.1762261 | SETD4 | 54093 |
| ENSG00000159228 | 921.5122673 | -0.049733199 | 0.0838102 | 0.4558385 | 0.7007253 | CBR1 | 873 |
| ENSG00000159231 | 16.42539801 | 0.029511074 | 0.1384467 | 0.2838925 | NA | CBR3 | 874 |
| ENSG00000142197 | 372.7256337 | 0.030050203 | 0.1007349 | 0.6645346 | 0.8443847 | DOP1B | 9980 |
| ENSG00000273199 | 17.28164616 | 0.019653652 | 0.1356153 | 0.4907567 | NA | NA | NA |
| ENSG00000159256 | 891.9503485 | 0.083768072 | 0.0872338 | 0.2254626 | 0.4864477 | MORC3 | 23515 |
| ENSG00000159259 | 1411.79595 | 0.047735885 | 0.075079 | 0.4489794 | 0.6960224 | CHAF1B | 8208 |
| ENSG00000159263 | 128.6484308 | -0.034425838 | 0.1227133 | 0.5680706 | 0.783983 | SIM2 | 6493 |
| ENSG00000159267 | 397.0740337 | -0.065461258 | 0.1051091 | 0.3631251 | 0.6262607 | HLCS | 3141 |
| ENSG00000185808 | 167.57105 | 0.089535925 | 0.1388664 | 0.2111564 | 0.4679412 | PIGP | 51227 |
| ENSG00000182670 | 2721.104634 | -0.048486593 | 0.0640139 | 0.3904019 | 0.6505443 | TTC3 | 7267 |
| ENSG00000228677 | 10.27531839 | -0.002120885 | 0.1348004 | 0.9220352 | NA | TTC3-AS1 | 100874006 |
| ENSG00000157538 | 1009.005349 | -0.020850047 | 0.0780381 | 0.7442622 | 0.8888382 | VPS26C | 10311 |
| ENSG00000157540 | 1298.694061 | -0.010389612 | 0.0728563 | 0.8654828 | 0.9457908 | DYRK1A | 1859 |
| ENSG00000157557 | 532.869938 | 0.170451393 | 0.1222962 | 0.0455656 | 0.1822781 | ETS2 | 2114 |
| ENSG00000183527 | 1519.415897 | -0.049535526 | 0.072464 | 0.4209544 | 0.6735758 | PSMG1 | 8624 |
| ENSG00000185658 | 1380.823215 | -0.009891892 | 0.0729877 | 0.8719026 | 0.9484107 | BRWD1 | 54014 |
| ENSG00000205581 | 5758.413966 | -0.144830116 | 0.0601076 | 0.0074217 | 0.0509661 | HMGN1 | 3150 |
| ENSG00000182093 | 589.1715464 | -0.056164802 | 0.0931435 | 0.4206044 | 0.6733937 | GET1 | 7485 |
| ENSG00000157578 | 23.65054338 | 0.045726699 | 0.1438209 | 0.1588072 | NA | LCA5L | 150082 |
| ENSG00000185437 | 27.83500768 | 0.036386455 | 0.1386264 | 0.306225 | NA | SH3BGR | 6450 |
| ENSG00000182240 | 287.0651743 | 0.045114859 | 0.1101383 | 0.5158197 | 0.7452598 | BACE2 | 25825 |
| ENSG00000183421 | 729.5677925 | -0.064334257 | 0.1349924 | 0.3028302 | 0.5722957 | RIPK4 | 54101 |
| ENSG00000141956 | 505.0926483 | 0.037821158 | 0.0946639 | 0.5695047 | 0.7849421 | PRDM15 | 63977 |
| ENSG00000157617 | 299.0138763 | -0.244737224 | 0.169564 | 0.0192752 | 0.1010111 | C2CD2 | 25966 |
| ENSG00000173276 | 957.4789645 | 0.794473896 | 0.1172452 | 7.46E-13 | 1.00E-10 | ZBTB21 | 49854 |
| ENSG00000160190 | 47.94479811 | -0.067659305 | 0.1508882 | 0.1536092 | 0.3914288 | SLC37A1 | 54020 |
| ENSG00000225218 | 24.56696332 | 0.008483832 | 0.1326859 | 0.7996247 | NA | NA | NA |
| ENSG00000160193 | 971.4965818 | -0.140080789 | 0.0960983 | 0.0599595 | 0.2178498 | WDR4 | 10785 |
| ENSG00000160194 | 680.7440297 | -0.012136728 | 0.0869716 | 0.8556878 | 0.9418544 | NDUFV3 | 4731 |
| ENSG00000160199 | 440.5139262 | -0.174472703 | 0.1303141 | 0.0461967 | 0.1839834 | PKNOX1 | 5316 |
| ENSG00000160200 | 5032.572734 | 0.002992339 | 0.0593254 | 0.9569346 | 0.9825908 | CBS | 875 |
| ENSG00000160201 | 4594.858117 | -0.130951681 | 0.0591152 | 0.014117 | 0.0810877 | U2AF1 | 7307 |
| ENSG00000228120 | 44.17240444 | -0.036006024 | 0.1357006 | 0.3962296 | 0.6563185 | NA | NA |
| ENSG00000142178 | 853.6297897 | 0.3255522 | 0.111136 | 0.0004127 | 0.0055347 | SIK1 | 150094 |
| ENSG00000185186 | 19.01325355 | 0.003244271 | 0.1335816 | 0.9110991 | NA | LINC00313 | 114038 |
| ENSG00000160207 | 29.77679806 | 0.035135318 | 0.1375917 | 0.3432742 | NA | HSF2BP | 11077 |
| ENSG00000160208 | 2705.434691 | 0.15700464 | 0.0724357 | 0.0123585 | 0.0738414 | RRP1B | 23076 |
| ENSG00000160209 | 2698.461038 | 0.004192465 | 0.0621421 | 0.9403502 | 0.9766571 | PDXK | 8566 |
| ENSG00000160213 | 1316.658387 | -0.028926549 | 0.0736935 | 0.640903 | 0.8315061 | CSTB | 1476 |
| ENSG00000160214 | 2117.67211 | 0.147379455 | 0.0738687 | 0.0202025 | 0.1045381 | RRP1 | 8568 |
| ENSG00000160216 | 1782.663245 | -0.211308337 | 0.0778435 | 0.0017384 | 0.0170979 | AGPAT3 | 56894 |
| ENSG00000160218 | 1986.931038 | -0.046376066 | 0.0710855 | 0.4449858 | 0.693143 | TRAPPC10 | 7109 |
| ENSG00000241945 | 1667.379322 | -0.050212408 | 0.0715123 | 0.4097416 | 0.6648411 | PWP2 | 5822 |
| ENSG00000160221 | 733.2406429 | 0.133199309 | 0.1001523 | 0.0789161 | 0.2598869 | GATD3 | 8209 |

| | | | | | | | | |
|---|---|---|---|---|---|---|---|---|
| ENSG00000237604 | 9.74080806 | -0.008454958 | 0.1352085 | 0.6988769 | NA | NA | NA | |
| ENSG00000232698 | 12.41115516 | -0.000331665 | 0.134478 | 0.9866185 | NA | NA | NA | |
| ENSG00000160223 | 1442.217282 | -0.179671303 | 0.0857337 | 0.0115371 | 0.0703734 | ICOSLG | | 23308 |
| ENSG00000141959 | 2640.566628 | -0.05037183 | 0.0658187 | 0.3809346 | 0.641901 | PFKL | | 5211 |
| ENSG00000160226 | 158.3978966 | -0.025899825 | 0.1174175 | 0.678839 | 0.8535466 | CFAP410 | | 755 |
| ENSG00000184441 | 20.76242855 | -0.009052572 | 0.1333791 | 0.7668131 | NA | NA | NA | |
| ENSG00000142185 | 8.764146005 | 0.01191344 | 0.1358096 | 0.5689476 | NA | TRPM2 | | 7226 |
| ENSG00000160233 | 55.55980777 | 0.050150086 | 0.1391241 | 0.3006773 | 0.5703493 | LRRC3 | | 81543 |
| ENSG00000269430 | 30.34711734 | 0.014988209 | 0.1326722 | 0.6771119 | NA | NA | NA | |
| ENSG00000228709 | 47.97767949 | -0.047717039 | 0.1390709 | 0.3000619 | 0.5697989 | NA | NA | |
| ENSG00000184787 | 3134.08881 | 0.162819449 | 0.0656631 | 0.0052951 | 0.0396998 | UBE2G2 | | 7327 |
| ENSG00000184900 | 3282.304429 | -0.129568252 | 0.0647968 | 0.0238007 | 0.1168136 | SUMO3 | | 6612 |
| ENSG00000183255 | 6126.227606 | -0.066556703 | 0.0555554 | 0.1893972 | 0.4403437 | PTTG1IP | | 754 |
| ENSG00000160255 | 46.22481202 | 0.000785149 | 0.1286938 | 0.9895368 | 0.9955163 | ITGB2 | | 3689 |
| ENSG00000227039 | 22.31106916 | 0.026339664 | 0.1365404 | 0.4004806 | NA | ITGB2-AS1 | | 100505746 |
| ENSG00000273027 | 8.351637297 | -0.001747841 | 0.1351099 | 0.9295376 | NA | NA | NA | |
| ENSG00000183250 | 70.48457778 | 0.011717459 | 0.12591 | 0.8167299 | 0.9252377 | LINC01547 | | 84536 |
| ENSG00000160256 | 1096.823219 | 0.090808714 | 0.0826598 | 0.1781175 | 0.4271872 | SLX9 | | 85395 |
| ENSG00000235374 | 12.01259574 | 0.01486624 | 0.1356267 | 0.5407361 | NA | SSR4P1 | | 728039 |
| ENSG00000197381 | 490.26568 | 0.002278056 | 0.0957533 | 0.974953 | 0.9905401 | ADARB1 | | 104 |
| ENSG00000186866 | 1059.581568 | 0.157789198 | 0.090263 | 0.029992 | 0.1383635 | POFUT2 | | 23275 |
| ENSG00000215447 | 162.8791794 | 0.044899909 | 0.1206893 | 0.4908814 | 0.7273003 | NA | NA | |
| ENSG00000223768 | 151.0272168 | 0.221850047 | 0.2427142 | 0.0330249 | 0.1466249 | LINC00205 | | 642852 |
| ENSG00000182871 | 8907.300631 | -0.429317904 | 0.0637036 | 1.60E-12 | 1.95E-10 | COL18A1 | | 80781 |
| ENSG00000173638 | 1350.579281 | -0.023319022 | 0.0760589 | 0.7119056 | 0.8693997 | SLC19A1 | | 6573 |
| ENSG00000142156 | 183.4139571 | 0.195546497 | 0.2099493 | 0.0439533 | 0.177715 | COL6A1 | | 1291 |
| ENSG00000142173 | 1025.628907 | 0.128391714 | 0.0894728 | 0.0717404 | 0.2442435 | COL6A2 | | 1292 |
| ENSG00000160284 | 88.61167742 | -0.09691134 | 0.1615628 | 0.1308948 | 0.3556358 | SPATC1L | | 84221 |
| ENSG00000160285 | 2234.431397 | -0.330658646 | 0.0815045 | 6.58E-06 | 0.0001746 | LSS | | 4047 |
| ENSG00000215424 | 206.5858083 | -0.073325456 | 0.1259623 | 0.2993333 | 0.5693326 | MCM3AP-AS | | 114044 |
| ENSG00000160294 | 2888.804462 | -0.070846449 | 0.067161 | 0.2258159 | 0.4868367 | MCM3AP | | 8888 |
| ENSG00000228137 | 13.11029592 | 0.000828088 | 0.1343919 | 0.9753554 | NA | NA | NA | |
| ENSG00000239415 | 9.204924621 | -0.023570703 | 0.1379773 | 0.268006 | NA | NA | NA | |
| ENSG00000182362 | 122.1608899 | 0.004346255 | 0.1195233 | 0.9416263 | 0.977176 | YBEY | | 54059 |
| ENSG00000160298 | 333.7669685 | 0.098812983 | 0.1213239 | 0.1993498 | 0.4528599 | C21orf58 | | 54058 |
| ENSG00000160299 | 3424.974397 | -0.110290185 | 0.0717218 | 0.0735455 | 0.2482906 | PCNT | | 5116 |
| ENSG00000160305 | 1062.314898 | 0.075319189 | 0.0814641 | 0.2574141 | 0.52429 | DIP2A | | 23181 |
| ENSG00000160310 | 1496.901203 | -0.135480628 | 0.0792605 | 0.0410991 | 0.1703803 | PRMT2 | | 3275 |
| ENSG00000215750 | 36.69012193 | 0.027133628 | 0.1347027 | 0.4739644 | NA | NA | NA | |
| ENSG00000212857 | 163.1238786 | -0.072565374 | 0.1350845 | 0.2738339 | 0.5422366 | NA | NA | |
| ENSG00000215781 | 717.0755303 | 0.018285182 | 0.085 | 0.7846703 | 0.9099309 | NA | NA | |
| ENSG00000218497 | 32.96568421 | 0.074230118 | 0.1593399 | 0.0805894 | NA | NA | NA | |
| ENSG00000236575 | 30.78778373 | -0.034969635 | 0.1376469 | 0.3385271 | NA | NA | NA | |
| ENSG00000229631 | 16.4532004 | -0.02122306 | 0.1363882 | 0.4209185 | NA | NA | NA | |
| ENSG00000220023 | 155.8885207 | 0.031052926 | 0.1182584 | 0.6251497 | 0.8202285 | NA | NA | |
| ENSG00000244180 | 10.40215854 | 0.009961686 | 0.135318 | 0.6569971 | NA | NA | NA | |
| ENSG00000272060 | 651.3896021 | -0.018322429 | 0.1375237 | 0.2647235 | 0.5325931 | NA | NA | |
| ENSG00000242716 | 22.08209712 | -0.051041902 | 0.1473307 | 0.0942884 | NA | NA | NA | |
| ENSG00000266658 | 17063.08851 | -0.019188278 | 0.1373081 | 0.3134455 | 0.5822768 | NA | NA | |
| ENSG00000265830 | 43.33019429 | -0.010951911 | 0.1366245 | 0.4263014 | 0.678557 | NA | NA | |
| ENSG00000215615 | 118.11136 | 0.031695324 | 0.1232916 | 0.5915502 | 0.800756 | NA | NA | |
| ENSG00000215700 | 343.9900022 | -0.073587689 | 0.1116295 | 0.313616 | 0.5824083 | NA | NA | |
| ENSG00000215699 | 286.4247767 | -0.009676326 | 0.1049862 | 0.8846488 | 0.9535046 | NA | NA | |
| ENSG00000211459 | 11589.27149 | -0.046736748 | 0.0590804 | 0.3827633 | 0.6435041 | NA | NA | |
| ENSG00000210077 | 13.35867879 | 0.020108516 | 0.136325 | 0.4339107 | NA | NA | NA | |
| ENSG00000210082 | 42491.4992 | 0.050351763 | 0.0598 | 0.3493304 | 0.615186 | NA | NA | |
| ENSG00000209082 | 135.3537736 | 0.135864892 | 0.1861422 | 0.0809825 | 0.2642336 | NA | NA | |
| ENSG00000198888 | 17005.34998 | 0.042242738 | 0.0526232 | 0.3829331 | 0.6435917 | ND1 | | 4535 |
| ENSG00000210107 | 65.0641763 | 0.009579442 | 0.1272817 | 0.8402083 | 0.9344745 | NA | NA | |
| ENSG00000210112 | 19.06755295 | 0.024558755 | 0.1362905 | 0.4187105 | NA | NA | NA | |
| ENSG00000198763 | 17263.29543 | 0.134742115 | 0.0540662 | 0.0067145 | 0.0473337 | ND2 | | 4536 |
| ENSG00000210117 | 12.68771295 | -0.008380546 | 0.134672 | 0.7340859 | NA | NA | NA | |
| ENSG00000210127 | 12.36865842 | -0.029431475 | 0.139161 | 0.2222959 | NA | NA | NA | |
| ENSG00000210135 | 19.72778323 | 0.042685004 | 0.143228 | 0.1477375 | NA | NA | NA | |
| ENSG00000210140 | 20.38505146 | 0.031704785 | 0.1384887 | 0.2962455 | NA | NA | NA | |
| ENSG00000210144 | 15.97925902 | 0.003661189 | 0.1340758 | 0.8919075 | NA | NA | NA | |
| ENSG00000198804 | 85819.69169 | -0.038040494 | 0.0478559 | 0.3971181 | 0.6567942 | COX1 | | 4512 |
| ENSG00000210151 | 93.27486591 | 0.053191247 | 0.1350641 | 0.3457119 | 0.6118577 | MIR12136 | | 113219467 |
| ENSG00000210154 | 18.14108342 | -0.002039453 | 0.1337732 | 0.9385316 | NA | NA | NA | |
| ENSG00000198712 | 41111.80304 | -0.160073278 | 0.0545179 | 0.0014673 | 0.0151278 | COX2 | | 4513 |

| | | | | | | | | |
|---|---|---|---|---|---|---|---|---|
| ENSG00000228253 | 2059.874901 | -0.031623555 | 0.0746536 | 0.605504 | 0.8105052 | ATP8 | | 4509 |
| ENSG00000198899 | 25230.13454 | -0.063398583 | 0.0510625 | 0.182761 | 0.4324147 | ATP6 | | 4508 |
| ENSG00000198938 | 38414.48159 | -0.198095059 | 0.0676544 | 0.0010525 | 0.0116093 | COX3 | | 4514 |
| ENSG00000198840 | 9010.266048 | 0.025683343 | 0.0994299 | 0.7076408 | 0.8677254 | ND3 | | 4537 |
| ENSG00000210174 | 18.67463025 | 0.015892663 | 0.1347612 | 0.585487 | NA | NA | NA | |
| ENSG00000212907 | 10809.90893 | 0.009380855 | 0.0561508 | 0.863026 | 0.9452421 | ND4L | | 4539 |
| ENSG00000198886 | 43664.15764 | 0.04227164 | 0.0500235 | 0.3306849 | 0.5988522 | ND4 | | 4538 |
| ENSG00000198786 | 18649.96593 | -0.338642726 | 0.0551711 | 1.18E-10 | 9.94E-09 | ND5 | | 4540 |
| ENSG00000198695 | 11931.36708 | 0.091131867 | 0.1255653 | 0.2106394 | 0.4673275 | ND6 | | 4541 |
| ENSG00000210194 | 114.7397107 | 0.104765516 | 0.1603192 | 0.1349957 | 0.3616424 | NA | NA | |
| ENSG00000198727 | 17641.32461 | 0.023113199 | 0.0506999 | 0.6039417 | 0.8092043 | CYTB | | 4519 |
| ENSG00000210195 | 50.63485467 | -0.099154591 | 0.1804144 | 0.0502505 | 0.1929572 | NA | NA | |
| ENSG00000210196 | 96.04106429 | -0.031098634 | 0.1266119 | 0.5728658 | 0.7872645 | NA | NA | |
